
JOURNAL INFORMATION
==============================
NLM Title Abbreviation: Mol Oncol
Journal ID: Mol Oncol
NLM Journal ID: 101308230
Journal ID: MOL2

Molecular Oncology

ISSN: 1574-7891
EISSN: 1878-0261
Publisher: Wiley

ARTICLE INFORMATION
==============================
PMCID: PMC11156946
PMID: 38845177
DOI: 10.1002/1878-0261.13683
Article ID: MOL213683
Article version: 1
Subjects: Abstracts; EACR 2024: Innovative Cancer Science, 10‐13 June 2024, Rotterdam, Netherlands; Abstracts

Abstracts

Electronic publication date: 2024 Jun 6
Publication date: 2024 Jun
Volume: 18
Issue: Suppl 1
Issue title: Supplement: EACR 2024: Innovative Cancer Science, 10‐13 June 2024, Rotterdam, Netherlands
First page: 1
Last page: 495
Copyright: © 2024 The Authors. Molecular Oncology is published by John Wiley & Sons Ltd on behalf of Federation of European Biochemical Societies
License: This is an open access article under the terms of the http://creativecommons.org/licenses/by/4.0/ License, which permits use, distribution and reproduction in any medium, provided the original work is properly cited.
License URL: https://creativecommons.org/licenses/by/4.0/

Figure count: 0
Table count: 0
Page count: 2

source-schema-version-number: 2.0
cover-date: June 2024
details-of-publishers-convertor: Converter:WILEY_ML3GV2_TO_JATSPMC version:6.4.4 mode:remove_FC converted:07.06.2024

==============================

Molecular Oncology

Volume 18, Supplement 1, June 2024

### EACR 2024 Congress Abstracts

### Contents

**PROFFERED PAPER PRESENTATIONS ............... 2**

Top Abstracts ........ 2

Symposium: Modulating the Tumour Microenvironment . 3

Symposium: Drug Resistance ........ 5

Symposium: Mechanism-based Clinical Trials ........ 6

Symposium: Precision Cancer Medicine in the Post-genomic Era ........ 7

Joint EACR-ICSA Symposium: Senescence, Dormancy, and Related Cell States ........ 8

Symposium: Metabolism and Cancer ........ 9

Symposium: Cell-based Therapies ........ 10

Joint EACR-AACR Symposium: Spatial Analysis of Tumours/Spatial Transcriptomics ........ 12

Symposium: Mechanical Forces of Cancer ........ 13

Symposium: Mechanisms of Tumour Metastases ........ 14

Symposium: Targeting Mitochondria in Cancer ........ 15

Symposium: Liquid Biopsies ........ 16

Symposium: Drugging Tumour Suppressor Genes ........ 17

Joint EACR-ESR Symposium: Imaging the Tumour Microenvironment ........ 18

Symposium: Making Cold Tumours Hot ........ 19

Symposium: Drugging the RAS Pathway ........ 20

Joint EACR-EMBO Symposium: Modelling Cancer ........ 21

Symposium: Ageing, Gender, and Cancer ........ 22

**POSTER PRESENTATIONS (Tuesday/Wednesday)........ 24**

Bioinformatics and Computational Biology ........ 24

POSTER IN THE SPOTLIGHT ........ 42

Biomarkers in Tissue and Blood ........ 43

Cancer Cell Biology ........ 78

Cancer Genomics ........ 164

POSTER IN THE SPOTLIGHT ........ 178

Carcinogenesis ........ 192

Drug Resistance ........ 199

POSTER IN THE SPOTLIGHT ........ 224

POSTER IN THE SPOTLIGHT ........ 231

POSTER IN THE SPOTLIGHT ........ 231

Epigenetics ........ 236

POSTER IN THE SPOTLIGHT ........ 243

Experimental/Molecular Therapeutics, Pharmacogenomics ........ 245

Immunotherapy ........ 291

POSTER IN THE SPOTLIGHT ........ 298

POSTER IN THE SPOTLIGHT ........ 304

POSTER IN THE SPOTLIGHT ........ 307

Molecular and Genetic Epidemiology ........ 314

Prevention and Early Detection ........ 318

Radiobiology/Radiation Oncology ........ 330

Signalling Pathways ........ 337

Translational Research ........ 351

Tumour Biology ........ 383

Tumour Evolution and Heterogeneity ........ 439

Tumour Immunology ........ 445

POSTER IN THE SPOTLIGHT ........ 463

**LATE-BREAKING ABSTRACTS........ 481**

### Proffered Papers

10-minute talks awarded for the highest scored abstracts, embedded in the scientific symposia sessions. These presentations are not accompanied by a poster.

### Posters in the Spotlight

Tuesday 11 June, 18:15 - 18:40, Poster and Exhibition Hall
Wednesday 12 June, 18:15 - 18:40, Poster and Exhibition Hall

Dedicated sessions taking place in the spotlight area within the Poster and Exhibition Hall. Poster presenters with high scoring abstracts will give short presentations of up to 5 minutes each. Their posters will also be available to view during the Poster Discussion Sessions.

### Late-breaking Abstracts

Late-breaking abstracts are those for which full data were not available at the time of the regular abstract deadline.

Molecular Oncology 18 (Suppl. 1) (2024) 1–495
© 2024 The Authors. Molecular Oncology is published by John Wiley & Sons Ltd on behalf of Federation of European Biochemical Societies
This is an open access article under the terms of the Creative Commons Attribution License, which permits use, distribution and reproduction in any medium, provided the original work is properly cited

### PROFFERED PAPER PRESENTATIONS

### Top Abstracts

### EACR12024-0093
### Proffered Paper: Minor intron splicing is critical for survival of lethal prostate cancer

*A. Augspach[1], M. Rubin[1], R. Kanadia[2], M. Jaquet[1], K. Drake[3], J. Triscott[1], S. Piscuoglio[4], M. Gerstein[5], L. Roma[6], D. Clarke[7]*
*[1]University of Bern, Department of Biomedical Research, Bern, Switzerland*
*[2]University of Connecticut, Institute for Systems Genomics, Storrs, United States*
*[3]University of Connecticut, Physiology and Neuro-biology Department, Storrs, United States*
*[4]Institute of Pathology and Medical Genetics, University Hospital Basel, Basel, Switzerland*
*[5]Yale University, Department of Computer Science, New Haven, United States*
*[6]University Hospital Basel, Institute of Pathology and Medical Genetics, Basel, Switzerland*
*[7]Yale University, Department of Molecular Biophysics and Biochemistry, New Haven, United States*

**Introduction**
While the role of major intron splicing in prostate cancer (PCa) is established, the roles of the minor spliceosome (MiS) and minor intron splicing have remained under-explored. The evolutionarily conserved minor spliceosome (MiS) is required for protein expression of ~714 minor intron-containing genes (MIGs) crucial for cell cycle regulation, DNA repair, and MAP-kinase signaling. We explored the role of MIGs and MiS in cancer, taking prostate cancer (PCa) as an exemplar.

**Material and Methods**
This study utilized next-generation sequencing (NGS), gene expression analysis, splicing analysis, RNA interference (RNAi), and in situ RNAish techniques to explore the role of the minor spliceosome in prostate cancer.

**Results and Discussions**
Here, we show that minor intron-containing genes (MIGs) are interactors of PCa-causing genes and that their expression levels correlate with PCa progression. We show that the expression of U6atac, a small nuclear RNA and key regulator of the MiS, enable cell growth and correlate with PCa progression. Consequently, highly proliferative PCa cells are vulnerable to siU6atac-mediated MiS inhibition. In models of castration resistant prostate cancer (CRPC) and neuroendocrine PCa (NEPC), siU6atac decreases the tumor burden significantly; this is also true for enzalutamide resistant PCa models. Here siRNA knocking down U6atac was ~50% more efficient in lowering tumor burden compared to standard antiandrogen therapy. Mechanistically, MiS inhibition through siU6atac causes minor/major intron mis-splicing and aberrant expression of MIG/non-MIG encoded proteins, which enriches for MAPK activity, DNA repair, and cell cycle regulation. Single cell-RNAseq confirms cell cycle defects and lineage dependency on the MiS from primary to CRPC and NEPC. Our data provides strong evidence that androgen receptor (AR) signaling functions as a regulator of MiS activity throughout AR dependent PCa disease progression. Finally, we discovered that MiS inhibition reduces expression of full length (FL) AR while simultaneously upregulating the truncated AR isoform, AR45, believed to inhibit the function of AR FL. We have also found that siU6atac increases canonical REST in NEPC and decreases REST4, a dominant negative inhibitor of REST.

**Conclusion**
Taken together, these finding support MiS as an important disruptor of critical cancer pathways with potential roles in lineage plasticity and drug resistance.

### EACR2024-0519
### Proffered Paper: Convergent evolution within and across patients with advanced breast cancer reveals mechanisms of drug resistance

*I. Leshchiner[1,2], E. Martin[2], E. Leshchiner[2], C. Chen[3,4], K. Rhrissorrakrai[5], F. Utro[5], B. Danysh[2], L. Parida[5], D. Juric[4,6], G. Getz[2,4,6,7]*
*[1]Boston University, Computational Biomedicine, Boston, United States*
*[2]Broad Institute of MIT and Harvard, Cancer Program, Cambridge, United States*
*[3]Stanford University, School of Medicine, Stanford, United States*
*[4]Massachusetts General Hospital, Cancer Center, Boston, United States*
*[5]IBM Research, Thomas Watson Research Center, Yorktown Heights, United States*
*[6]Harvard University, Harvard Medical School, Boston, United States*
*[7]Massachusetts General Hospital, Department of Pathology, Boston, United States*

**Introduction**
Determining patterns and mechanisms of drug resistance is key for improving clinical outcome of cancer treatments. The ability to study multiple samples from different metastatic sites of the same patient is a clinically challenging task, which has become possible with the advent of "rapid" autopsy procedures (<10 hours from death) conducted on the patients deceased from cancer.

**Material and Methods**
We studied resistance mechanisms to hormone therapy and CDK4/6 cell cycle inhibitors in ER+ breast cancer by analyzing whole-exome, whole-genome, single-cell, and bulk transcriptomes in 120 autopsy samples from patients obtained by the Massachusetts General Hospital Rapid Autopsy program. For each patient, we inferred the clonal structure of the samples and tracked the metastatic spread of different clones throughout the body. We have integrated genomic and transcriptomic data through advanced clonal reconstruction methods (PhylogicNDT) to investigate how the patient developed resistance to multiple lines of anti-cancer therapy. Additionally, we have used previously collected cell-free DNA samples to establish both a spatial and temporal picture of cancer drug resistance and progression.

Molecular Oncology 18 (Suppl. 1) (2024) 1–495
© 2024 The Authors. Molecular Oncology is published by John Wiley & Sons Ltd on behalf of Federation of European Biochemical Societies

**Results and Discussions**
We discovered that in most patients' multiple metastatic sites share similar clonal structure, though often several related 'families' of resistant cancer cell populations spread throughout the body. These distinct populations often have developed independent resistance mechanisms to the many lines of treatment that the patients have received. Patients showed multiple known and potentially novel resistance mechanisms in separate phylogenetic branches of the phylogenetic tree, often converging on distinct mutations in the same resistance genes. Notably we have identified novel recurrent chromatin modifier convergent alterations across the cohort. Temporal analysis using cfDNA taken over the course of treatment allows us to identify clones that were selected for in response to a given treatment. Using RNA expression and pathway analysis, we identify unique transcriptional programs and differentially expressed genes between distinct clones within a patient as well as compare genetically similar clones across patients.
**Conclusion**
Overall, we developed a framework for investigating convergent mechanisms of resistance that leverages multi-site sampling from rapid autopsies to systematically identify significantly recurrent events in resistant clones across patients.

### EACR2024-0792
### Proffered Paper: Tertiary lymphoid structures contain stem-like T cell reservoirs and act as amplifiers of immunotherapy response

*N. Slingerland[1,2], L. Roelofsen[1,2], P. Kaptein[1,2], K. Moore[2,3], O. Isaeva[2,4], K. Harteminks[5], K. Monkhorst[6], L. Wessels[2,3], T. Schumacher[1,2], D. Thommen[1,2]*
*[1]Netherlands Cancer Institute, Molecular Oncology and Immunology, Amsterdam, The Netherlands*
*[2]Oncode Institute, Oncode Institute, Utrecht, The Netherlands*
*[3]Netherlands Cancer Institute, Division of Molecular Carcinogenesis, Amsterdam, The Netherlands*
*[4]Netherlands Cancer Institute, Division of Tumor Biology & Immunology, Amsterdam, The Netherlands*
*[5]Netherlands Cancer Institute, Department of Thoracic Surgery- Department of Gastrointestinal Surgery and Surgical Oncology, Amsterdam, The Netherlands*
*[6]Netherlands Cancer Institute, Department of Pathology- Division of Diagnostic Oncology, Amsterdam, The Netherlands*
**Introduction**
The presence of tertiary lymphoid structures (TLS) in tumors has been associated with better clinical outcome upon PD-1 blockade. However, it remains unclear whether TLS immunologically contribute to response and how their presence affects the T cell landscape.
**Material and Methods**
By combining ex vivo technologies with high-dimensional transcriptional and functional profiling of cancer tissues, we here investigated how TLS impact T cell specificity, state, and capacity for reinvigoration upon PD-1 blockade in lung cancer.
**Results and Discussions**
To understand whether TLS harbor a distinct T cell repertoire compared to the tumor parenchyma, we performed TCR-beta sequencing of microdissected TLS and tumor bed regions from non-small cell lung cancer samples. A majority of expanded TCRs was shared between TLS and tumor regions, with many TCRs occurring in multiple regions, suggesting that TLS likely do not expand a distinct T cell repertoire as compared to the tumor parenchyma. The vast majority of shared expanded TCRs were CD8+ and displayed multiple dysfunctional phenotypes, indicative of tumor reactivity. To assess whether the distribution of these dysfunctional states could be driven by the spatial context of the T cells, we employed patient-derived tumor fragment (PDTF) ex vivo technology and explored spatial transcriptomic datasets. Strikingly, we found that the expanded, likely tumor-specific T cell clones, were biased towards early dysfunctional, precursor-like (TCF7+) and transitional (GZMK+) states in TLS regions. Vice versa, in tumor bed regions, we observed an enrichment of cells with resident memory (ZNF683+) phenotype. Late dysfunctional (CXCL13+) cells appeared to be spread across both regions. To determine whether TLS and tumor-dwelling T cells differ in their capacity for reinvigoration, we treated PDTFs with PD-1 blockade ex vivo. This revealed that, while T cells in both tumor parenchyma and TLS could undergo immune reactivation, the intensity and diversity of cytokine and chemokine secretion was higher in TLS compared to tumor parenchyma, implicating TLS as a region where immune responses can be magnified and diversified.
**Conclusion**
Collectively, these findings suggest that TLS may contribute to therapy response via different functions, specifically by forming reservoirs of precursors-like cells that may replenish the antitumor T cell pool and by presenting niches where T cell responses can be amplified upon PD-1 blockade.

### Symposium: Modulating the Tumour Microenvironment

### EACR2024-0486
### Proffered Paper: Early reinvigoration and replenishment dynamics of the CD8 T cell landscape upon ICB in sequential cutaneous squamous cell carcinoma biopsies

*M. Machuca Ostos[1,2,3], J. Traets[1], P. Kaptein[2,3], S. Breukers[1,4], X. Qiao[1], J.B.A.G. Haanen[3,5], T.N. Schumacher[2,3,6], C.L. Zuur[1,4,7], D. Thommen[2,3]*
*[1]Netherlands Cancer Institute NKI-AvL, Tumor Biology and Immunology, Amsterdam, The Netherlands*
*[2]Oncode Institute, Oncode, Utrecht, The Netherlands*
*[3]Netherlands Cancer Institute NKI-AvL, Molecular Oncology and Immunology, Amsterdam, The Netherlands*
*[4]Netherlands Cancer Institute NKI-AvL, Head and Neck*

© 2024 The Authors. Molecular Oncology is published by John Wiley & Sons Ltd on behalf of Federation of European Biochemical Societies

*Surgery and Oncology, Amsterdam, The Netherlands*
*[5]Netherlands Cancer Institute NKI-AvL, Medical Oncology, Amsterdam, The Netherlands*
*[6]Leiden University Medical Center, Hematology, Leiden, The Netherlands*
*[7]Leiden University Medical Center, Otorhino-laryngology Head and Neck Surgery, Leiden, The Netherlands*

**Introduction**

Tumor-reactive T cells mediate antitumor immunity upon immune checkpoint blockade (ICB), but the contribution of distinct T cell states to ICB response to remains unclear. Pre- and on-treatment biopsies from clinical trials are often taken a few weeks to months after treatment initiation, making it impossible to understand which T cell subsets are directly reinvigorated by the treatment. We hereby exploit the unique setting of the MATISSE trial to dissect the early T cell dynamics upon neoadjuvant ICB.

**Material and Methods**

In the MATISSE trial (NCT04620200), 46 stage I-IVa cutaneous squamous cell carcinoma patients received either Nivolumab® or Nivolumab® with Ipilimumab®. Matched blood samples and biopsies were collected at baseline and in weeks 1, 2 and 4 on treatment. To understand the remodeling of the TME, single-cell RNA-&TCRseq was performed on CD45+ sorted cells from tumor and blood samples at the given timepoints for 19 patients. Moreover, to relate these changes to functional reinvigoration ex vivo, baseline biopsies were treated with ICB according to the patient-derived tumor fragment (PDTF) platform.

**Results and Discussions**

We could successfully map 16 CD8 states which showed distinct temporal dynamics, indicating deep on-treatment remodeling. Responding patients harboring a dysfunctional compartment at baseline showed an early induction of proliferation and activation of the pre-existing pool of dysfunctional T cell clones within the first days after ICB. In contrast, a second group of patients harbored a predominant effector-like compartment which was clonally expanded and activated early upon treatment. In both groups, clones were replenished throughout treatment course, and gave rise to (resident) memory-like states at the time of tumor clearance. Supporting this clonal replenishment, these samples showed functional immune reinvigoration upon ex vivo ICB treatment, with profound production of cytokines and chemoattractants. This early immune reactivation at the tumor site may mediate the later clonal replenishment and remodeling.

**Conclusion**

We here describe distinct responsive TME subtypes composed of dysfunctional or effector-like states. Both show early reinvigoration of pre-existing clones few days after ICB treatment, which are maintained through treatment and acquire in memory phenotypes upon antigen clearance. Altogether, these observations underline that assessment of early timepoints are key to understand the temporal remodeling of the T cell landscape upon ICB treatment.

### EACR2024-1083
### Proffered Paper: Characterization of the Epigenomic Profile Shaping Human CD4+FOXP3+ Regulatory T cell Identity in the Tumor Microenvironment

*C. Dossena[1], R. Bason[2], M. Fakiola[2], A. Beneggi[3], F. Simoncello[3], G. Veronesi[4], S. Siena[5], G. Rossetti[2], Y. Murakawa[6], M. Pagani[2]*
*[1]IFOM ETS - The AIRC Institute of Molecular Oncology, Molecular Oncology and Immunology, Milan, Italy*
*[2]IFOM ETS – The AIRC Institute of Molecular Oncology, Molecular Oncology and Immunology, Milan, Italy*
*[3]IFOM ETS – The AIRC Institute of Molecular Oncology, Molecular Oncology and Immunology, Milano, Italy*
*[4]IRCCS San Raffaele Scientific Institute, Division of Thoracic Surgery, Milano, Italy*
*[5]Niguarda Cancer Center, Dipartimento di Oncologia ed Emato-Oncologia, Milano, Italy*
*[6]RIKEN Center for Integrative Medical Sciences, Human Genomics, Yokohama, Japan*

**Introduction**

Within the tumor microenvironment (TME), the suppressive role of tumor-infiltrating CD4+ FOXP3+ regulatory T cells (TI-Tregs) supports tumor growth and progression. A promising direction in cancer immuno-therapy is the selective depletion or reprogramming of TI-Tregs, in order to enhance anti-tumor immunity without disrupting Treg-dependent homeostasis in healthy tissues. Our previous work revealed a distinct transcriptome in TI-Tregs compared to normal adjacent tissue Tregs (NAT-Tregs). Here, we focus on epigenomic rewiring as a key process that orchestrates Treg adaptation to TME cues and shapes their unique properties.

**Material and Methods**

$CD4^+$ $CD25^+$ $CD127^-$ Tregs were isolated from blood, tumor, and non-tumor adjacent tissues of CRC and NSCLC patients. ATAC-seq data on chromatin accessibility and different histone modification profiles were used to reconstruct the genome-wide active and repressed chromatin states and to precisely map the location of active enhancers. Next, single cell RNA-seq data were leveraged to discover bidirectionally transcribed noncoding RNAs (eRNAs) and to refine the functional core of enhancers. In silico transcription factor (TF) footprinting followed by experimental assays were employed to identify key trans regulators shaping the TI-Treg phenotype.

**Results and Discussions**

Through multi-omics integration, we drew a comprehensive map of the cis-acting regulatory landscape of human Tregs from normal and tumor tissues. We focused not only on chromatin accessibility and promoter regulation, but also on regulatory elements with bona fide active enhancer features, defined through de novo chromatin states discovery and identification of eRNA transcription. Integration of epigenomic and transcriptomic data revealed that the chromatin rewiring in TI-Treg cells involves active enhancers at a much greater extent than promoters. We identified active enhancers specific for TI-Treg signature genes and showed that the epigenomic changes between

Molecular Oncology 18 (Suppl. 1) (2024) 1–495
© 2024 The Authors. Molecular Oncology is published by John Wiley & Sons Ltd on behalf of Federation of European Biochemical Societies

NAT and TI-Tregs reflect their differences in gene expression. We further determined the TFs with the highest shift in binding activity across Treg cell states and highlighted key *trans* regulators that preferentially act upon the TI-Treg enhancerome.

**Conclusion**

Our findings reveal the enhancer-mediated regulatory circuits that distinguish human Tregs within tumors, pointing towards specificities and actionable vulnerabilities with potential clinical relevance for TI-Treg-targeted therapies.

### Symposium: Drug Resistance

### EACR2024-0389
### Proffered Paper: Neutrophil extracellular traps formed during chemotherapy confers treatment resistance

*A. Mousset[1], E. Lecorgne[1], I. Bourget[1], P. Lopez[1], J. Cherfils-Vicini[1], F. Mechta-Grigoriou[2], M. Green Terp[3], M. Egeblad[4], C. Gaggioli[1], J. Albrengues[1]*
*[1]Institute for Research on Cancer and Aging, Ircan, Nice, France*
*[2]Institut Curie, Stress and Cancer Laboratory, Paris, France*
*[3]Institute of Molecular Medicine- University of Southern Denmark, Department of Cancer and Inflammation Research, Odense, Denmark*
*[4]Johns Hopkins University School of Medicine, Departments of Cell Biology and Oncology, Baltimore, United States*

**Introduction**

Breast cancer is the most common cancer among women worldwide and metastasis is the major cause of patient death. Most of them are treated with chemotherapy but development of chemoresistance is common resulting in treatment failure. The tumor microenvironment (TME) can confer this chemoresistance in cancer cells. Recently, the role of immune cells in the TME, especially the role of neutrophils, has been further investigated. Neutrophils pro-tumoral activities were linked to their ability to form Neutrophil Extracellular Traps (NETs). NETs are DNA scaffold decorated with cytotoxic enzymes and proteases that are release in the extracellular space by neutrophils in response to an inflammatory context. However, their role in anti-cancer treatment response remains unclear.

**Material and Methods**

To study NET implication in breast cancer metastasis chemoresistance, we used a syngeneic mouse model of breast cancer lung metastatic subjected to chemotherapy. Neutrophils, NETs, and their subsequent interactions with microenvironment compounds were targeted using antibodies, inhibitors, or DNAse I. Three cell lines and two distinct chemotherapies used in clinic were tested in mice: cisplatin or a combination of adriamycin and cyclophosphamide (AC).

**Results and Discussions**

We show that chemotherapy treatment induces strong recruitment of neutrophils which then form NETs around metastatic cancer cells in mice lungs. We found that chemotherapy-treated cancer cells released adenosine triphosphate (ATP) causing other cancer cells to secrete IL-1β, which in turn triggered neutrophils to form NETs. Two NET-associated proteins where required for NETs' ability to induce chemoresistance: first, integrin-αvβ1 in NETs trapped latent TGFβ secreted by cancer cells; then, matrix metalloproteinase 9 (MMP9) cleaved and activated the trapped latent TGFβ. The NET-mediated TGFβ activation caused cancer cell to undergo epithelial to mesenchymal transition and correlate with chemo-resistance. Pharmacological inhibition of IL-1β-NET-integrinαvβ1-MMP9-TGFβ axis resulted to a significant decrease of cancer cells chemoresistance and thus significantly reduced the metastatic burden in mice lungs.

**Conclusion**

Our work highlights a new mechanism by which NETs regulate activities of neighboring cells treated by chemotherapy and thus, provide us some potential new therapeutic targets to treat chemoresistant patients. The final aim of our work is to increase the efficacy of chemotherapies and thus increase the chances of remission and survival of patients.

### EACR2024-1196
### Proffered Paper: Epigenetic, transcriptomic and genetic analyses of HR+/HER2- primary and metastatic breast cancer reveals heterogeneous mechanisms of endocrine therapy resistance

*J. Sangalang[1,2], D. Tran[1], N. Ibrahimi[3], T. Sourisseau[4], V. Scott[1], A. Tran-Dien[1], N. Servant[5], F. André[1], S. Nikolaev[1]*
*[1]Institut Gustave Roussy, Molecular Predictors and New Targets in Oncology, Villejuif, France*
*[2]Université Paris-Saclay, Faculty of Medicine, Le Kremlin-Bicêtre, France*
*[3]Institut Gustave Roussy, Bureau of Biostatistics and Epidemiology, Villejuif, France*
*[4]Institut Bergonié, Equipe Sarcome, Bordeaux, France*
*[5]Institut Curie, CurieCoreTech Bioinformatics, Paris, France*

**Introduction**

Endocrine therapy (ET) is typically the initial treatment method for hormone receptor-positive, human epidermal growth receptor 2-negative (HR+/HER2-) breast cancer patients; however, almost half of the patients exhibit ET resistance after initial response. Mechanisms of ET resistance are not fully understood, and requires an integrated epigenetic, transcriptomic and genetic approach.

**Material and Methods**

We conducted ATAC, RNA, and whole-exome sequencing on primary and metastatic breast cancer lesions from 464 HR+/HER2- patients, with 338 patients containing all three datasets. Primary breast cancers (pBC) were derived from patients who experienced either a relapse or sustained remission over a five-year period after tumour resection and adjuvant endocrine therapy. Metastatic lesions originated from patients with ET-resistant metastatic breast cancers (mBC). Analysis of

© 2024The Authors. Molecular Oncology is published by John Wiley & Sons Ltd on behalf of Federation of European Biochemical Societies chromatin accessibility, gene expression, and mutational data aimed to understand relapse mechanisms in pBC and resistance mechanisms in mBC. Cohort clustering utilized PAM50 probability scoring.

**Results and Discussions**

Two PAM50 scoring-derived pBC groups exhibited different relapse risks, with high-risk cases showing increased chromatin accessibility and expression of E2F targets, MYC targets, and the JAK-STAT pathway. Low-risk cases were enriched in estrogen response (ER) pathways. ESR1 and TP53 driver alterations were enriched in mBC vs pBC, while ER pathways and GATA3/FOXA1 transcription factors were more active in pBC. PAM50 probability scoring revealed subgroups of mBC based on perceived transdifferentiation to other intrinsic subtypes of breast cancer. ESR1 mutations characterized "Luminal B" tumours and resembled pBC, "Her2" subtype exhibited ERBB2 alterations. "Basal" subtype was associated with BRCA1/UBR5 alterations and poor overall survival.

**Conclusion**

This comprehensive analysis of HR+/HER2- breast cancer unveils key insights into ET resistance mechanisms. PAM50 probability scoring may further help dissect the heterogeneity within HR+/HER2- breast cancer tumours. These findings contribute to a deeper understanding of the mechanisms of relapse and molecular landscape of ET resistance in breast cancer.

### Symposium: Mechanism-based Clinical Trials

### EACR2024-0569
### Proffered Paper: FKBPL is differentially expressed across the Barrett's oesophagus to oesophageal adenocarcinoma disease progression pathway

*A. Heeran[1], C. Duggan[2], C. Gargan[1], M. Zaki[1], M. Menon[1], N. O'Connor[1], D. O'Toole[3], J. Reynolds[1], T. Robson[2], J. O'Sullivan[1]*

*[1]Trinity St. James's Cancer Institute- Trinity Translational Medicine Institute, Surgery, Dublin, Ireland*

*[2]RCSI University of Medicine and Health Sciences, School of Pharmacy and Biomolecular Sciences, Dublin, Ireland*

*[3]Trinity College Dublin, Clinical Medicine, Dublin, Ireland*

**Introduction**

Oesophageal adenocarcinoma (OAC) is the predominant subtype of oesophageal cancer in high-income regions. Barrett's oesophagus (BO) is a preneoplastic condition that increases OAC risk. FKBPL is a novel angiogenesis-related protein with known anti-tumour functions. High FKBPL has been identified as a good prognostic marker in breast and ovarian cancer. FKBPL peptide mimetics are promising anti-cancer therapeutics, with one such peptide having completed a phase I clinical trial, thus highlighting the therapeutic value of elucidating the role of FKBPL across the BO-OAC disease progression. We hypothesised that FKBPL would be differentially expressed across the BO-OAC disease progression.

**Material and Methods**

We utilised ex vivo human explants and serum from BO and upper GI cancer patients to screen for secreted FKBPL. We cultured matched normal oesophagus and BO explants, and cancer explants for 24h to generate conditioned media (CM). We assessed the levels of FKBPL in the CM and serum by ELISA and correlated this with clinical and pathological data. We performed immunohistochemistry (IHC) on TMAs from OAC patients with matched areas of normal, BO and tumour. FKBPL mRNA levels were assessed in normal, BO and OAC explant tissue.

**Results and Discussions**

Results from our human *ex vivo* explant work demonstrate that FKBPL secretion was significantly higher from BO (n=29, $p$<0.0001) and upper GI cancer tissue (n=24, $p$<0.01) compared to normal oesophageal tissue (n=21). FKBPL mRNA was significantly higher in OAC tissue (n=10) compared to BO (n=12, $p$<0.01) and normal tissue (n=12, $p$<0.01). IHC analysis revealed higher epithelial FKBPL expression in BO and OAC tissue compared to normal tissue and higher FKBPL stromal expression in BO compared to normal adjacent tissue (n=39). Correlation of FKBPL with clinical data identified an association between FKBPL and smoking status at the gene, secreted protein and circulating serum levels. Furthermore, lower secreted and circulating levels of FKBPL were associated with more advanced cancer disease, including lymph node involvement ($p$<0.05), presence of metastasis ($p$=0.05) and higher pathological N stage ($p$<0.01).

**Conclusion**

Increased FKBPL levels may play a role in the pathogenesis of BO-OAC, suggesting a novel oncogenic role in this disease setting. Smoking status is associated with differential expression of FKBPL. Further investigation into the functional role of FKBPL in the BO-OAC disease progression requires elucidation and may identify potential novel therapeutic targets.

### EACR2024-0960
### Proffered Paper: Oncolytic adenovirus ORCA-010 activates the tumor micro-environment and induces systemic tumor-specific T cell responses in patients with newly-diagnosed prostate cancer

*V.W. van Beusechem[1,2], T. Brachtlova[1,2], R. Nadafi[2], J. Giddens[3], K. Jansz[4], P. Incze[5], A. Abramovitch[6], B. Shayegan[7], R. Casey[5], W. Dong[2]*

*[1]Amsterdam UMC, Medical Oncology, Amsterdam, The Netherlands*

*[2]ORCA Therapeutics, Research & Development, 's-Hertogenbosch, The Netherlands*

*[3]Jonathan Giddens Medicine Professional Corp, Clinic, Brampton, Canada*

*[4]Kenneth Jansz Medicine Professional Corp, Clinic, Burlington, Canada*

Molecular Oncology 18 (Suppl. 1) (2024) 1–495

© 2024 The Authors. Molecular Oncology is published by John Wiley & Sons Ltd on behalf of Federation of European Biochemical Societies

[5]The Fe/Male Health Centres, Clinic, Oakville, Canada
[6]Urology and Male Infertility Clinic, Clinic, Scarborough, Canada
[7]St. Josephs Healthcare Hamilton, Surgery, Hamilton, Canada

**Introduction**

Oncolytic viruses are considered as new treatment for cancer. Virus-induced anti-tumor immune responses play a pivotal role in achieving treatment efficacy. We present findings from a phase I/IIA trial investigating oncolytic adenovirus ORCA-010 in early-stage prostate cancer patients without prior treatment (NCT04097002; sponsor ORCA Therapeutics). Primary objectives include assessment of safety and induction of anti-tumor immunity.

**Material and Methods**

In the Phase I dose-escalation part, nine patients with localized prostate cancer received a single intra-prostatic administration of ORCA-010 (n=3 at 1E^11, 5E^11, or 1.5E^12vp), followed by a one-year follow-up period. The Phase IIA component included 3 patients who underwent two administrations of 1.5E^12vp ORCA-010 and were followed for one year; and 9 patients receiving two administrations of 1.5E^12vp ORCA-010 before radical prostatectomy six-eight weeks later.

**Results and Discussions**

ORCA-010 administered once or twice was not associated with dose-limiting toxicities or severe adverse events. After ORCA-010 injection at the highest dose, prostate specific antigen levels dropped below pretreatment levels in several patients, suggesting a clinical response. Biopsies taken one year post-treatment revealed significant increases in intratumoral cytotoxic CD8+ T cell density, while regulatory T cell density did not change significantly. In prostatectomy specimens, increases in CD8+ and CD8+PD-1+ cells were seen, in particular in immune cell-infiltrated regions. Phenotypic analyses of PBMCs showed systemic CD8+ T cell responses, with significant increases in activated (HLA-DR+) and proliferating (Ki67+) CD8+ cells over the first few weeks after ORCA-010 injection. *Ex vivo* peptide restimulation assays showed that the CD8+ T cell repertoire of ORCA-010 treated patients included cognate CD8+ T cells expressing IFNg and TNF in response to adenovirus capsid protein and, importantly, also to prostate associated antigens.

**Conclusion**

Intra-prostatic administration of ORCA-010 demonstrated an excellent safety profile. Our findings strongly suggest that ORCA-010 has the potential to transform an immunologically "cold" tumor microenvironment into "hot" regions in prostate cancer. The composition of immune cells in treated tumors suggest that ORCA-010 activates the tumor microenvironment. Crucially, ORCA-010 treatment induced a systemic increase in tumor responsive CD8 T cells. Together, this offers promise for use of ORCA-010 in prostate cancer immunotherapy strategies.

### Symposium: Precision Cancer Medicine in the Post-genomic Era

### EACR2024-0664
### Proffered Paper: Oncogenic function of WTAP-mediated m6A methylation in glioma growth

M. Adiamah[1], M. Dieterle[1], R. Toth[2], A. Oudin[1], A. Lesur[3], E. Van Dyck[4], F. Fuks[5], S. Niclou[1], A. Golebiewska[1], S. Fritah[1,6]
*[1]Luxembourg Institute of Health, NORLUX Neuro-oncology Laboratory- Department of Cancer Research, Luxembourg, Luxembourg*
*[2]Luxembourg Institute of Health, Bioinformatics Platform, Department of Cancer Research, Luxembourg, Luxembourg*
*[3]Luxembourg Institute of Health, Quantitative Biology Unit- Department of Cancer Research, Luxembourg, Luxembourg*
*[4]Luxembourg Institute of Health, DNA Repair and Chemoresistance- Department of Cancer Research, Luxembourg, Luxembourg*
*[5]Université Libre de Bruxelles, Laboratory of Cancer Epigenetics- Faculty of Medicine, Brussels, Belgium*
*[6]Luxembourg Institute of Health, Cancer RNAs and Epigenetic Group- Department of Cancer Research, Luxembourg, Luxembourg*

**Introduction**

N6-methyladenosine (m6A) is the most abundant modification on mRNA and is intricately linked to post-transcriptional processing and RNA fate. Dysregulated m6A methylation is implicated in tumourigenesis, however understanding of its role in diffuse glioma is limited. Following the WHO 2021 classification, gain of function mutations in isocitrate dehydrogenase (IDH) enzymes stratify lower grade gliomas (LGG, grade 2/3) from glioblastoma (GBM, grade 4). The established impact of oncometabolite 2-hydroxyglutarate (2HG) on the DNA and histone methylation landscape of IDH mutant gliomas provides a rationale for similar effects on m6A RNA methylation in these tumours. In IDH1 wildtype GBM, disease-specific programmes of m6A deregulation in tumour-promoting/suppressive pathways remains unclear.

**Material and Methods**

To identify candidate m6A regulators in IDH1 wildtype and mutant gliomas, we used patient RNA expression datasets, patient-derived glioma stem-like cells (GSC) and engineered IDH1 mutant CRISPR knock-in and overexpression models. We generated CRISPR/Cas9-mediated genetic knockout (KO) of candidate m6A regulators to identify their functional significance in glioma biology in vitro and in vivo. We performed nanopore direct RNA sequencing for global m6A detection and untargeted proteomics for pathway-specific interrogation. We additionally profiled global m6A methylation changes associated with mutant IDH1 expression.

**Results and Discussions**

At the patient level, differential expression of m6A-modifiers WTAP and FTO correlated with IDH status and tumour type suggesting interconnectivity of

© 2024 The Authors. Molecular Oncology is published by John Wiley & Sons Ltd on behalf of Federation of European Biochemical Societies histological types and molecular features. Further validation in GSCs and genetically engineered models revealed consistent elevated expression of WTAP and FTO protein IDH1 wildtype cells. Loss of function studies in IDH1 wildtype GSCs revealed WTAP and not FTO as an important regulator of GBM proliferation, self-renewal and tumour progression. WTAP loss resulted in more striking alterations in global m6A methylation and protein expression compared to FTO loss suggesting a more significant functional role. Intriguingly, induced expression of mutant IDH1 resulted in m6A hypermethylation resembling FTO loss.

**Conclusion**

We reveal mutant IDH1-specific hypermethylation in m6A RNA similar to observed DNA and histone methylation changes. Furthermore, our findings indicate WTAP plays an important role in glioma progression and may be an important distinction between IDH1 wildtype and mutant gliomas determining oncogenic phenotypes.

### EACR2024-1242
### Proffered Paper: Mammalian SWI/SNF Chromatin Remodeling Complex Orchestrates Sequential Binding of Transcription Factors and Restricts Malignant Transformation

*D. Barisic[1], C. Chin[1], C. Meydan[1], M. Teater[1], C. Mlynarczyk[1], A. Weng[2], D.W. Scott[3], C. Mason[1], A. Melnick[1]*

*[1]Weill Cornell Medicine, Department of Medicine, New York, United States*

*[2]Terry Fox Laboratory, BC Cancer Agency, Vancouver, Canada*

*[3]British Columbia Cancer, Centre for Lymphoid Cancer, Vancouver, Canada*

**Introduction**

Mutations in the SWI/SNF-family chromatin remodeling complex BAF are a recurring feature in many cancers, with a notably high prevalence in lymphoma (>35%). Despite this, the exact process that connects the mutation of *ARID1A*, most frequently mutated SWI/SNF subunit, to malignant transformation is still not understood.

**Material and Methods**

We focused on elucidating the function of ARID1A in malignant B cells by developing novel lymphoma mouse models that allowed for the conditional deletion of *Arid1a*. This was combined with comprehensive genomic analysis using both bulk and single-cell techniques, alongside in vitro assays and detailed immune cell profiling.

**Results and Discussions**

We show that ARID1A disruption induces a profound alteration in the progression of B-cell transcriptional program, leading to genome-wide chromatin repression and reduction in nucleosome turnover. We showed that this chromatin repression is largely due to loss of PU.1 and NF-kB binding to chromatin. To investigate co-dependency of these two transcription factors, we used a synthetic reporter containing binding sites for both NF-kB and PU.1. We showed that NF-kB and PU.1-mediated gene activation requires ARID1A activity. Deleting only the PU.1 motif within the reporter we observed loss of reporter activation. This suggests that PU.1 directs ARID1A-containing BAF complex to remodel nucleosomes, facilitating subsequent NF-kB factor binding. Next, we leveraged a parallel single-cell RNA and ATAC assay to scrutinize subtle shifts within different populations of murine B cells in the course of an immune response. Strikingly, we found PU.1 and NF-kB factors are chronologically dependent on ARID1A with PU.1 accessibility initially compromised, followed by a decrease at NF-kB sites. Finally, the chromatin compaction upon *Arid1a* deletion prompted B cells to transition prematurely to the memory B-cell compartment by impairing specific immune signals. Linking this critical role of ARID1A in chromatin regulation to malignant transformation, we observed a reduction in overall survival for mice with *Arid1a* mutations in our Bcl2 lymphoma mouse model. Moreover, we show that lymphoma patients with *ARID1A*-inactivating mutations preferentially display an immature memory B-cell-like state with increased transformation risk to an aggressive lymphoma.

**Conclusion**

These observations offer mechanistic understanding into the emergence of both indolent and aggressive lymphomas in ARID1A-mutant patients through formation of immature memory-like clonal precursors.

### Joint EACR-ICSA Symposium: Senescence, Dormancy, and Related Cell States

### EACR2024-0525
### Proffered Paper: HMGB2 in PDAC – a stepping stone to replicative immortality?

*A. Mackay[1], I. Papadionysiou[1], T. Xie[1], Y. Zhu[1], A. Papantonis[1]*

*[1]University Medical Center Göttingen, Pathology, Göttingen, Germany*

**Introduction**

A key prerequisite of malignant transformation is the evasion of cellular senescence, which allow cancer cells to proliferate indefinitely. In non-transformed cells, commitment to senescence is marked by the nuclear loss of High Mobility Group-Box 2 (HMGB2) proteins. Conversely, HMGB2 is overexpressed across solid cancers, including Pancreatic Ductal Adenocarcinomas (PDAC). This led us to hypothesize that the proliferative capacity of PDAC cells relies on HMGB2, which renders it an attractive therapeutic target. However, until now, we lacked the means and understanding for exploiting this option.

**Material and Methods**

We combined immunofluorescence staining of histological PDAC patient samples with matching patient-derived organoids (PDOs) studied via single-cell genomics (scRNA-seq + scATAC-seq) to classify them in HMGB2$^{High}$ or HMGB2$^{Low}$ groups. HMGB2$^{High/Low}$ PDOs were then characterized as regards their 3D genome organization and distribution of structural (SV) and copy number variants (CNVs) using Hi-C in the

Molecular Oncology 18 (Suppl. 1) (2024) 1–495
© 2024 The Authors. Molecular Oncology is published by John Wiley & Sons Ltd on behalf of Federation of European Biochemical Societies presence or absence of treatment with a small molecule inhibitor targeting HMGB2. The response of stratified PDOs to HMGB2 inhibition was evaluated by longitudinal live-cell imaging and validated by knockdowns. Finally, the therapeutic potential of HMGB2 inhibition was confirmed in vivo in an orthotopic mouse model.

**Results and Discussions**

First, our analysis of publicly available patient data and patient slides showed that tumor cells express more HMGB2 compared to surrounding tissue. This was confirmed in both PDOs and data from mouse models. Next, we integrated data from scATAC-seq, scRNA-seq, and Hi-C to gauge PDAC regulatory heterogeneity and identify neoloops involved in the induction of several key PDAC genes. Moreover, scRNA-seq from control and HMGB2-inhibited PDOs implicated HMGB2 in cell cycle regulation, whereby HMGB2$^{High}$ cells displayed shorter G1- and prolonged S-phase, but converged to the slower HMGB2$^{Low}$ proliferative profiles upon HMGB2 inhibition. This was not merely the result of differential gene expression, but also of HMGB2-guided alternative splicing, where increased usage of alternative transcription start sites and exon skipping in HMGB2$^{High}$ PDOs affected key cell cycle-related genes. Finally, HMGB2 inhibition in orthotopic mouse models replicated these effects to constrain tumor growth better than conventional chemotherapy.

**Conclusion**

Our findings implicate HMGB2 as a vital player in PDAC proliferation and highlight senogenic treatment as a new therapeutic option in PDAC.

### EACR2024-1033

### Proffered Paper: Potential of testosterone-mediated DNA damage and senescence induction as a novel therapeutic approach in adrenocortical carcinoma

*D. Garcia-Garcia[1], J. Olabe[1], J. Wilmouth[1], L. Brugiere[1], A. Voisin[1], R. Guiton[1], A. Martinez[1], P. Val[1]*

*[1]institute of Genetics Reproduction & Development- iGReD, Reproduction- Physiopathology and Cancer, Clermont-Ferrand, France*

**Introduction**

The role of sex hormones in progression of cancers besides reproductive tissues is highly debated, although most cancers are more frequent in males. Adrenocortical carcinoma (ACC), a highly aggressive cancer which arises from steroidogenic cells of the adrenal cortex, is one of the rare exceptions to this rule with, a higher incidence in women than men. 20% percent of ACC patients present somatic inactivation of the WNT signaling inhibitor *ZNRF3*. We have recently shown that consistent with ACC patients, adrenal-specific inactivation of *Znrf3* in mice results in sexually dimorphic development of ACC: whereas most females progress to develop metastatic tumors, the initial hyperplasia regresses in males. We showed that this sexually dimorphic tumor development resulted from testosterone-mediated induction of senescence in male mice. This triggered recruitment and activation of highly phagocytic macrophages which cleared out senescent cells, preventing tumor progression in male mice. Analysis of human data suggested that these macrophages were more frequent in male ACC patients where they were associated with better prognosis. Together, these findings suggested that testosterone by inducing senescence, could promote antitumor innate immune response in the context of ACC.

**Material and Methods**

We have treated *Znrf3 KO* mice bearing aggressive ACC with testosterone pellets (protocol approved by the regional ethics committee with number Apafis#4559). We have then conducted an in-depth analysis of tumor and immune response to testosterone treatment, using a combination of bulk RNAseq, single cell sequencing and spectral flow cytometry.

**Results and Discussions**

Testosterone potently inhibits tumor progression, induces re-differentiation of the adrenal cortex and blocks metastatic dissemination. This is associated with recruitment of phagocytic macrophages and mobilization of the adaptive antitumor immune response. Mechanistically, we show that senescence is triggered by induction of DNA double-strand breaks, after testosterone treatment. We further show that this is the result of inhibition of DNA repair mechanisms, following testosterone-mediated inhibition of MYC expression and RB1 phosphorylation, which ultimately inhibits BRCA1 and BRCA2 expression, creating a BRCA-like state.

**Conclusion**

Our findings suggest that short-term testosterone treatment may be used in combination with PARP inhibitors as a novel therapeutic approach in ACC patients.

### Symposium: Metabolism and Cancer

### EACR2024-0210

### Proffered Paper: Neutrophil pyrimidine metabolism drives lung metastasis in mammary cancer

*C. Clarke[1], D. Whyte[1], J. Vande Voorde[2], J. Mackey[1], D. Sumpton[1], L. Carlin[1], S. Coffelt[2], K. Blyth[1], O. Sansom[1], J. Norman[1]*

*[1]CRUK Scotland Institute, CRUK Scotland Institute, Glasgow, United Kingdom*

*[2]University of Glasgow, Institute of Cancer Sciences, Glasgow, United Kingdom*

**Introduction**

Neutrophils play a role in formation of the pre-metastatic niche. Here we show that altered pyrimidine metabolism can influence neutrophil behaviour to facilitate metastasis.

**Material and Methods**

Mice: Mice expressing the MMTV-PyMT transgene were used to model metastatic breast cancer. Wild-type mice treated with intraperitoneal injection of 1mg/kg lipopolysaccharide (LPS) for 24h were used to model acute inflammation. Liquid Chromatography-Mass Spectrometry (LC-MS): Metabolites were extracted using

© 2024The Authors. Molecular Oncology is published by John Wiley & Sons Ltd on behalf of Federation of European Biochemical Societies a polar solvent & separated using a pHILIC column. Live Cell Imaging of Precision Cut Lung Slices (PCLS): Lungs were inflated with agarose, sliced into 300μm sections, stained with directly conjugated antibodies & imaged by confocal microscopy. Flow Cytometry: Lungs were excised, minced, agitated & filtered. Cells were stained for Live/Dead, Fc-Receptor blocked, stained with directly-conjugated fluorescent antibodies, fixed & events acquired by flow cytometry.

**Results and Discussions**

Using LC-MS uracil was identified as a serum metabolite that correlated with metastasis in mammary cancer. Serum uracil levels also increased following LPS challenge, suggesting uracil could originate from an immune cell source. Uridine Phosphorylase 1 (UPP1) is responsible for uracil generation. We observed elevated *Upp1* expression by qPCR in neutrophils from the blood, spleen & bone of mice post-LPS challenge. Increased *Upp1* was also detected in neutrophils from MMTV-PyMT tumour-bearing mice. Pharmacological inhibition of UPP1 (using benzylacyclouridine, BAU) reversed tumour-mediated alterations in neutrophil behaviour in the lung, including restoration of neutrophil cell surface adhesion molecule expression, and motility, to non-tumour-bearing levels. BAU-treated tumour-bearing mice also exhibited increased $CD4^+$ & $CD8^+$ T cells in the lungs, suggesting UPP1 modulation of neutrophil immunosuppressive function. T cells co-cultured with neutrophils from tumour-bearing MMTV-PyMT *Upp1*$^{-/-}$ mice displayed elevated surface CD69 & increased surface CD44 was also observed on T cells from tumour-bearing MMTV-PyMT *Upp1*$^{-/-}$ lungs. Furthermore, the proportion of MMTV-PyMT mice with lung metastasis at clinical endpoint was significantly decreased in *Upp1*$^{-/-}$ animals.

**Conclusion**

UPP1 can influence metastasis by modulating the immunosuppressive behaviour of neutrophils. This could have significant therapeutic implications, including use of UPP1 inhibitors to enhance immunotherapy efficacy & UPP1 inhibition to de-prime niches that facilitate metastatic disease.

### EACR2024-0397

### Proffered Paper: Compromised transcription elongation renders prostate cancer cells dependent on the OGT-dependent rewiring of the spliceosome machinery

*S. Pallasaho[1], A. Gondane[1], J. Kutz[2], J. Liang[1], S. Yalala[1], D.Y. Duveau[3], H. Pospiech[2], C.J. Thomas[3], M. Loda[4], H.M. Itkonen[1]*

*[1]University of Helsinki, Department of Biochemistry and Developmental Biology, Helsinki, Finland*

*[2]Leibniz Institute on Aging - Fritz Lipmann Institute, Project group Biochemistry, Jena, Germany*

*[3]National Center for Advancing Translational Sciences-NIH, Division of Preclinical Innovation, Rockville, United States*

*[4]Weill Cornell Medicine- New York- Presbyterian Hospital, Department of Pathology and Laboratory Medicine, New York, United States*

**Introduction**

Prostate cancer (PC) is the most common cancer in men. Inactivating mutations of cyclin-dependent kinase 12 (CDK12), a major transcription elongation kinase, define an aggressive subtype of PC. O-GlcNAc transferase (OGT) catalyses all nucleocytoplasmic O-GlcNAcylation. OGT and its activity are increased in aggressive PC and previously, we found that inhibition of OGT sensitizes PC cells to compounds targeting transcriptional kinases CDK7 and CDK9. We hypothesized that compromised CDK12 activity leads to significant OGT-mediated rewiring of the PC cells, and that this rewiring results in actionable synthetic lethal interactions.

**Material and Methods**

To identify how compromised CDK12 activity alters OGT function, we performed mass spectrometry-based profiling of the glycoproteome and RNA-seq based analysis of the global alternative splicing. In addition, we integrated combinatorial lethal screening with gene essentiality- and clinical data from *CDK12* mutant PC tumors to identify candidate synthetic lethal targets with *CDK12* inactivation.

**Results and Discussions**

We show that combined targeting of CDK12 and OGT is selectively lethal to PC cells and not to the normal prostate cells. However, OGT is not an ideal target for cancer therapeutics as it is essential in higher eukaryotes. Therefore, we used mass spectrometry profiling to identify how reduced CDK12 activity alters OGT target repertoire and found that inhibition of CDK12 induces O-GlcNAcylation of splicing machinery. We integrated small-scale drug screen results with gene essentiality data and found that compromised CDK12 activity renders PC cells dependent on a non-essential spliceosome regulator, serine-arginine protein kinase 1 (SRPK1). Clinical data revealed that *CDK12* mutant tumors overexpress SRPK1.

**Conclusion**

We show that the compromised CDK12 activity renders PC cells dependent on OGT-mediated rewiring of spliceosome machinery. We propose that inactivation of the *CDK12*-gene is a biomarker for sensitivity against inhibitors of the otherwise non-essential spliceosome components.

### Symposium: Cell-based Therapies

### EACR2024-0804

### Proffered Paper: LGR5-targeting CAR-T cells are a promising clinical treatment for metastatic colorectal cancer

*D. McPeake[1,2], J. Foeng[1,2], T. Tyllis[1,2], E. Thompson[2,3], V. Bandara[2,4], C. Bonder[2,3], I. Comerford[1], T. Sadlon[2,4,5], S. Barry[2,4,5], S. McColl[1,2]*

*[1]The University of Adelaide, Chemokine Biology Laboratory- Department of Molecular and Biomedical Science, Adelaide, Australia*

*[2]Carina Biotech, University of South Australia, Adelaide, Australia*

Molecular Oncology 18 (Suppl. 1) (2024) 1–495

© 2024 The Authors. Molecular Oncology is published by John Wiley & Sons Ltd on behalf of Federation of European Biochemical Societies

[3]Centre for Cancer Biology, Vascular Biology and Cell Trafficking, Adelaide, Australia
[4]The University of Adelaide, Robinson Research Institute, Molecular Immunology, Adelaide, Australia
[5]Women's and Children's Health Network, Department of Paediatrics, Adelaide, Australia

**Introduction**

Colorectal cancer (CRC) is the second most common cause of cancer-related deaths. CAR-T cell therapy presents a promising, novel treatment for CRC. However, translating the efficacy observed in haematological malignancies to solid cancers still remains a major hurdle for CAR-T cell therapies. Solid tumors present many challenges for CAR-T cell therapies, including a scarcity of recognized effective target antigens and limited tumor infiltration. Leucine-rich repeat-containing G-protein coupled receptor 5 (LGR5) is a cancer stem cell marker, which mediates important roles in tumor initiation and metastasis, positioning the receptor as a promising target antigen.

**Material and Methods**

Immunohistochemistry, qPCR, and flow cytometry were utilised to measure LGR5 expression by human cancer cell lines and tissue microarrays. LGR5-targeting CAR-T cells were manufactured using an optimized 9-day protocol and were tested in in vitro cytotoxicity assays and two subcutaneous human CRC xenograft mouse models. Flow cytometric analysis was performed to comprehensively phenotype LGR5-targeting CAR-T cells in vitro and ex vivo.

**Results and Discussions**

Analysis of human cancer cell lines and tissue microarrays revealed LGR5 is expressed by multiple cancer types, notably CRC. LGR5-targeting CAR-T cells generated possessed a highly cytotoxic and minimally differentiated phenotype at manufacturing endpoint. LGR5-targeting CAR-T cells displayed significant cytotoxicity towards LGR5-expressing cancer cell lines in vitro. Additionally, in LoVo and LIM1215 subcutaneous human CRC xenograft mouse models, LGR5-targeting CAR-T cells drove robust inhibition of tumor growth, when compared to untransduced control T cells. Flow cytometric analysis at experimental endpoint revealed tumor-infiltrating CAR-T cells persisted 4 weeks after transfer. Furthermore, LGR5-targeting CAR-T cells were shown to provide substantial inhibition of tumor growth when administered at 1, 2 or 3 weeks post-tumor inoculation.

**Conclusion**

Together, these data position LGR5-targeting CAR-T cells as a viable clinical treatment. Indeed, these results formed part of the preclinical investigation that lead Carina Biotech to initiate a Phase 1/2a clinical trial of LGR5-targeting CAR-T cells in metastatic colorectal cancer (NCT05759728).

### EACR2024-1164

### Proffered Paper: Personalized identification of neoantigen-specific TCRs using synthetic TCR library screens

Z. Moravec[1], Y. Zhao[2], R. Voogd[1], D. Cook[3], S. Kinrot[3], J. Haanen[1,4,5], T. Schumacher[1,4,6], X. Chen[2], E. Porter[3], W. Scheper[1]
[1]Netherlands Cancer Institute, Molecular Oncology and Immunology, Amsterdam, The Netherlands
[2]Rootpath- Inc., na, Guangzhou, China
[3]Rootpath- Inc., na, Watertown- MA, United States
[4]Leiden University Medical Center, Hematology, Leiden, The Netherlands
[5]Netherlands Cancer Institute, Medical Oncology, Amsterdam, The Netherlands
[6]Oncode Institute, na, Utrecht, The Netherlands

**Introduction**

T cell receptor (TCR) gene therapy is a promising form of cellular immunotherapy where peripheral blood T cells of cancer patients are genetically engineered with tumor-specific TCRs in vitro before being reinfused into the patient. Contrary to immune checkpoint blockade and tumor-infiltrating lymphocytes (TIL) therapy, TCR gene therapy does not rely on a pre-existing intratumoral T cell response, and holds promise for the treatment of less immunogenic cancers that respond poorly to current immunotherapies. Evidence suggests that T cell activity towards neoantigens – which arise as a result of tumor mutations – is an important driver of anti-tumor immunity, and there are strong efforts that aim to exploit neoantigen-specific TCRs for TCR gene therapy. However, the identification of such TCRs is complicated by both the patient-specific nature of tumor mutations, and the fact that these TCRs may be exceedingly rare.

**Material and Methods**

We developed a personalized TCR discovery pipeline that combines large-scale assembly of synthetic TCR libraries with functional genetic screening, enabling the profiling of 1,000s of TCRs in a single experiment. T cells engineered with patient-derived TCR libraries are cocultured with matched antigen-presenting cells (APC) that are modified to express the patient's tumor mutanome, and responding TCRs are identified by next-generation sequencing. Since the patient APCs are fully MHC class I- and class II-proficient, this enables the unbiased functional screening of both MHC-I- and MHC-II-restricted patient T cell repertoires.

**Results and Discussions**

We leveraged the method to screen ~3700 TIL-derived TCRs from three patients and identified dozens of neoantigen-specific TCRs. Notably, these included multiple neoantigen-specific TCRs identified in an ovarian cancer patient with a low mutational burden. In TCR transfer experiments, identified TCRs mediated cytotoxicity towards neoantigen-positive APCs and patient tumor cells. Correlating the TCR specificity back to the phenotype of their parental T cells indicated that a number of neoantigen-specific clonotypes scored relatively low on recently identified tumor reactivity-predicting gene signatures, underscoring the importance of identification of therapeutic TCRs using truly functional screening assays.

**Conclusion**

Collectively, these data demonstrate the feasibility of personalized and high throughput discovery of neoantigen-specific TCRs, and the presented technology should catalyze the development of neoantigen-targeted TCR therapy.

© 2024 The Authors. Molecular Oncology is published by John Wiley & Sons Ltd on behalf of Federation of European Biochemical Societies

### Joint EACR-AACR Symposium: Spatial Analysis of Tumours/Spatial Transcriptomics

### EACR2024-0278
### Proffered Paper: Pre-clinical development of CVGBM: A therapeutic mRNA-based multiepitope vaccine for glioblastoma

*J. Lutz[1], R.K. Feist[1], K. Wolter[1], R. Mülfarth[1], J. Krüger[1], J. Bauer[2], J.S. Walz[2], R. Heidenreich[1]*
*[1]CureVac SE, Oncology, Tübingen, Germany*
*[2]University and University Hospital Tübingen, Department of Peptide-based Immunotherapy, Tübingen, Germany*

**Introduction**
Peptide vaccination against peptides of tumor-associated antigens (TAAs) over-presented on HLA class I and II molecules of glioblastomas (GBM) has shown some promise in clinical trials. To leverage the immunogenic potential of messenger ribonucleic acid (mRNA) vaccines, we developed an mRNA-based multiepitope vaccine candidate for GBM, CVGBM. CVGBM consists of an mRNA with unmodified nucleotides which is formulated in lipid nanoparticles (LNPs). It encodes a fusion protein comprising 8 TAA-derived epitopes that have demonstrated immunogenicity as peptide vaccines. These peptides can be presented either on HLA-A*02:01 (HLA class I) to $CD8^+$ T cells or on various HLA-DR (HLA class II) molecules to $CD4^+$ T cells.

**Material and Methods**
Presentation of CVGBM-encoded peptides was measured by immunopeptidomic analysis using two human cell lines endogenously expressing HLA-A*02:01 (HEK293T and THP-1). Immunogenicity of CVGBM was assessed in naïve C57BL/6 x BALB/c F1 (CB6F1) mice vaccinated intramuscularly three times in weekly intervals. For a murine surrogate mRNA, the anti-tumoral efficacy was assessed in the B16.F10 tumor model by vaccinating tumor-bearing C57BL/6 mice.

**Results and Discussions**
The immunopeptidomic analysis of CVGBM mRNA-transfected HEK293T and THP-1 cells confirmed presentation of HLA-A*02:01-presented peptides encoded by CVGBM. Notably, no additional peptides derived from other parts of the fusion protein, such as the epitope junctions, were detected. Vaccination of naïve CB6F1 mice with CVGBM induced $CD8^+$ and $CD4^+$ T-cell responses against the encoded epitopes. This demonstrated correct translation and processing of the CVGBM-encoded fusion protein and presentation of the encoded peptides. To test potential anti-tumor efficacy of such a vaccine, a murine surrogate multiepitope mRNA encoding 10 epitopes derived from the murine B16.F10 tumor model and an additional synthetic T-helper epitope was tested in B16.F10 tumor-bearing mice. The surrogate mRNA vaccine demonstrated anti-tumoral immune responses by significantly extending the median survival time of mice.

**Conclusion**
These data supported the initiation of a first-in-human, phase 1 trial (CV-GBLM-001; NCT05938387) assessing CVGBM in HLA-A*02:01-positive patients with newly diagnosed and surgically resected MGMT-unmethylated glioblastoma (CNS WHO Grade 4).

### EACR2024-1177
### Proffered Paper: Epithelial-mesenchymal plasticity at scale: AI-powered insights from single cell and spatial transcriptomics

*E. Withnell[1], S. Pan[1], M. Secrier[1]*
*[1]University College London, Genetics- Evolution and Environment, London, United Kingdom*

**Introduction**
The epithelial to mesenchymal transition (EMT) is a key cellular process underlying cancer progression, with multiple intermediate states whose molecular hallmarks remain poorly characterized. Here, we introduce cutting-edge AI methods to provide a multi-scale view of the epithelial-mesenchymal plasticity in cancer from single cell and spatial transcriptomics data.

**Material and Methods**
First, we employed a large language model similar to the one underlying chatGPT but tailored for biological data (inspired by scBERT methodology), to predict individual stable states within the EMT continuum in single cell data and dissect the regulatory processes governing these states. Secondly, we leveraged spatial transcriptomics of breast cancer tissue to delineate the spatial relationships between cancer cells occupying distinct states within the EMT continuum and various hallmarks of the tumour microenvironment. We introduce a new tool, SpottedPy, that identifies tumour hotspots within spatial transcriptomics slides displaying enrichment in processes of interest, including EMT, and explores the distance between these hotspots and immune/stromal-rich regions within the broader environment at flexible scales. Finally, we employ graph neural networks (GNNs) to explore to what extent cancer cells occupying different EMT states are dependent on other cells in the microenvironment.

**Results and Discussions**
Using a newly developed large language model, we demonstrate that epithelial, mesenchymal and three hybrid E/M states can be predicted in single cell data with an average accuracy of 90% across breast, lung, ovarian and prostate cancers. We further show that the hybrid states identified are linked with distinct survival and therapy response outcomes. In spatial transcriptomics data, our tool SpottedPy helps us identify strong associations between tumour cells in a late hybrid, quasi-mesenchymal state and regions with potential for immunotherapy response. We further show that using GNNs we can predict this quasi-mesenchymal, immune exhausted state based on cell-cell interactions established within the entire tissue slide, achieving an accuracy of 87%, while the epithelial state was captured with 90% accuracy.

**Conclusion**
Here, we introduce a suite of statistical and AI-based tools that allow the exploration of EMT plasticity in single cell and spatial data. Our insights may inform strategies to counter immune evasion enabled by EMT and offer new opportunities to target vulnerabilities in hybrid E/M states for therapeutic benefit.

Molecular Oncology 18 (Suppl. 1) (2024) 1–495
© 2024 The Authors. Molecular Oncology is published by John Wiley & Sons Ltd on behalf of Federation of European Biochemical Societies

### Symposium: Mechanical Forces of Cancer

### EACR2024-0511
### Proffered Paper: PIK3CA H1047R mutation reprogrammes multicellular communication to drive collective cell functions and therapy tolerance in mammary epithelium

*A. Frismantiene[1], P.A. Gagliardi[1], M. Dobrzynski[1], S. Jägers[1], L. Hinderling[1], P. Ender[2], A. Cohen[3], O. Pertz[1]*
*[1]University of Bern, Institute of Cell Biology, Bern, Switzerland*
*[2]AMOLF, Department of Autonomous Matter, Amsterdam, The Netherlands*
*[3]Drexel University, Department of Electrical and Computer Engineering, Philadelphia, United States*

**Introduction**

Hotspot mutations in PI3K3CA gene (H1047R/L and E545/542K) are frequently reported in breast cancer and associated with poor response to therapy. How PIK3CA mutations alter signalling networks and how this leads to aberrant functional phenotypes that drive oncogenesis and therapy tolerance is not well understood. Even less is known how oncogenic alterations at the molecular level reprogram tissue-scale signalling and collective functional properties.

**Material and Methods**

To model oncogenic PI3K signalling in mammary epithelium we use isogenic knock-in derivative of the parental MCF10A cell line harbouring PIK3CA H1047R mutation. This we combined with genetically encoded AKT, ERK and cell cycle biosensors and employ automated imaging and computer-vision image processing to follow spatial and temporal AKT and ERK signalling dynamics together with cell functional outputs – cell motility and proliferation at the single-cell and high temporal resolution but in the context of multicellular tissue-scale systems – living epithelial monolayers (2D) and spheroid cultures (3D). For automatic detection and quantification of intercellular signalling we use computational tools, and to explore signalling dynamics landscapes we use convolutional neural network techniques.

**Results and Discussions**

We find that hyperactivation of PI3K/AKT pathway by oncogenic PIK3CA H1047R mutation activates PI3K/AKT signalling crosstalk with MAPK/ERK pathway. This crosstalk is intercellular communication dependent and propagates as AKT and ERK activity wave in the epithelial monolayers and 3D acini. The propagation of AKT and ERK signalling waves is EGFR- and MMP- dependent, which suggest paracrine signalling that is driven by spatially coordinated EGFR ligand cleavage. Importantly, intercellular PI3K/MAPK pathway crosstalk is coupled with tissue deformation, which points to mechanochemical nature of the signalling waves. Blocking PI3K and MAPK crosstalk by PI3K, MAPK, or PI3K and MAPK inhibitors blocks excessive proliferation and re-sensitizes PIK3CA H1047R oncogene-reprogrammed epithelium to therapy.

**Conclusion**

PIK3CA H1047R mutation activates multicellular, tissue-scale PI3K/AKT – MAPK/ERK signalling crosstalk. This crosstalk depends on spatially and temporarily coordinated paracrine signalling and mechanocontrol and translates to excessive proliferation and increased drug tolerance. Detailed understanding of this signalling crosstalk at the tissue-scale might open new possibilities for targeting oncogenic signalling in PIK3CA mutated cancers.

### EACR2024-0570
### Proffered Paper: Elimination of Hyaluronic acid in Pancreatic Ductal Adenocarcinoma (PDAC) as a potential Therapeutic Strategy

*P. Sun[1], J. Li[2], B. Mariano[3], C. Guerra[1]*
*[1]Fundación Sector Público Estatal Centro Nacional Investigaciones Oncológicas Carlos III CNIO, Molecular Oncology Program, Madrid, Spain*
*[2]Fundación Sector Público Estatal Centro Nacional Investigaciones Oncológicas Carlos III CNIO, Molecular Oncology Program, Madrid, Spain*
*[3]Fundación Sector Público Estatal Centro Nacional Investigaciones Oncológicas Carlos III, Molecular Oncology Program, madrid, Spain*

**Introduction**

Pancreatic ductal adenocarcinoma (PDAC) is the fourth leading cause of cancer-related deaths, with a five-year survival rate around 5–7%. In PDAC, stromal cancer-associated fibroblasts (CAFs) play a vital role in promoting the desmoplastic and immunosuppressive tumor microenvironment (TME), as well as tumour growth and malignancy, and have emerged as cancer targets (1). Hyaluronic synthases (Has) proteins are responsible for the production of hyaluronic acid (HA), which is overexpressed in 90% of PDACs (2). Has overexpression results in accumulation of HA which leads to high pressure on neighbouring structures as well as high interstitial fluid-pressure (IFP) within PDAC, which can interfere with drug delivery (3) and has also been linked with tumor escape from immune surveillance. Previous results from our lab showed that Has1 and Has2 was differentially expressed between PDGFRa+ CAFs and PDGFRα+ normal pancreatic fibroblasts (NPFs) (4).

**Material and Methods**

To study the role of HA in PDAC development, progression and therapeutic efficacy, we have developed a genetically modified PDAC mouse model (GEMM) that includes germline Has1/3 KOs and Has2 conditional KO alleles (5). This triple Has knock-out model (TKO) was essential to obtain a good elimination of HA in tumour bearing mice. The elimination of the Has2 floxed allele was achieved by the exposure of the mice to a Tamoxifen diet to activate the CreERT2 recombinase that was ubiquitously expressed.

**Results and Discussions**

© 2024The Authors. Molecular Oncology is published by John Wiley & Sons Ltd on behalf of Federation of European Biochemical Societies

Interestingly, elimination of HA resulted in slower tumour progression due to increased apoptosis and lower proliferative rate of tumor cells. Besides, tumour microenvironment was characterized by less collagen and fibrosis, higher cytokeratin19 and more epithelial cell adhesion molecule (EpCAM) positive tumor cells. Prominently, it exhibits a dramatic increase infiltration of macrophages and CD8+ cytotoxic T cells, stimulating expression of the PD-1 ligand (PD-L1) in pancreatic cancer cells. Notably, HA clearance enhanced the chemotherapy and immunotherapy efficiency in GEMMs and in allograft studies of PDAC.

**Conclusion**

Targeting HA disrupts the desmoplastic and immunosuppressive TME, increases PD-L1 expression on pancreatic cancer cells. Deletion of HA render aggressive PDAC eradicable by synergizing with combination therapy. These studies may help to design future therapeutic strategies with targeting HA in PDAC.

*(1) Sahai E et al. 2020*
*(2) Khare V et al. 2014*
*(3) Z Luo et al. 2019*
*(4) M Djurec et al. 2018*
*(5) Matsumoto K et al. 2019*

### Symposium: Mechanisms of Tumour Metastases

### EACR2024-0257
### Proffered Paper: IKKα acts as a metastasis suppressor through regulation of tight-junction proteins ZO-1 and CLDN2

*D. Alvarez[1], A. Villanueva[2], L. Espinosa[1], A. Bigas[1]*
*[1]Hospital del Mar Research Institute, Cancer, Barcelona, Spain*
*[2]ICO, ProCure, Hospitalet de Llobregat, Spain*

**Introduction**

Colorectal cancer (CRC) remains the second leading cause of cancer death worldwide. Acquisition of therapeutic resistance, tumor relapse and metastasis are the leading cause of cancer-related death. In epithelial tumors, metastasis has been associated with epithelial-to-mesenchymal transition; however, it was recently shown that collective migratory of tumor cells can provide some metastatic advantages over single-cell migration. The mechanisms controlling the different metastatic modalities remain poorly understood, but cell-cell junction elements might play a critical role. Recently our group has demonstrated that IKKα regulates the levels and distribution of the tight-junction proteins ZO1 and CLDN2, which impact in the migratory and metastatic activity of CRC cells.

**Material and Methods**

We performed mass spectrometry analysis to identify new IKKα targets. The in vitro migratory capacity of CRC cells was determined by wound-healing and transwell assay. WB, IF and IHC were used for the analysis of protein levels and distribution. Metastatic activity of patient-derived organoids (PDO) was tested by intra-splenic transplantation in nude mice.

**Results and Discussions**

IKKα-depleted CRC cells showed reduced migratory capacity in vitro but higher tendency to migrate in clusters. This phenotype is associated with increased ZO1 and CLDN2 levels at the leading edge of the cell colonies, suggestive of tight-junction stabilization. To test whether the effects of IKKα activity on migration and cell-to-cell contacts impact on CRC metastasis, we performed intra-splenic transplantation of IKKα depleted cells. IKKα depletion imposed a massive increase in the metastatic activity of PDO cells. IHC analysis of mice liver metastases demonstrated a significant stabilization of ZO1 and CLDN2 in these metastases, consistent with in vitro data. Published scRNA-seq data (Batlle, 2023) from a metastasis murine model unveiled accumulation of CRC cells carrying high ZO1 and CLND2 levels together with the stem cell marker Lgr5 in the small metastasis. Our results suggest that IKKα, which exert different pro-tumorigenic activities, also acts as metastasis suppressor by preventing collective cell migration. Further investigation of the mechanisms of IKKα are required before using IKKα-pathway elements for cancer treatment.

**Conclusion**

IKKα functions as a metastasis regulator in CRC through the regulation of cell-cell contacts, which should be further investigated for its clinical relevance.

### EACR2024-0433
### Proffered Paper: Tumor-draining lymph node fibroblasts support pre-metastatic niche formation by promoting immunosuppressive cellular networks

*G. Mattavelli[1], M. Helal[1], A. Frank[1], E. Riemer[1], S.L. Herbert[2], T. Schlaiß[2], K. Mielert[3], A. Rosenwald[3], A. Wöckel[2], A. Riedel[1]*
*[1]University Hospital Würzburg, Mildred Scheel Early Career Centre MSNZ for Cancer Research, Würzburg, Germany*
*[2]University Hospital Würzburg, Department of Gynecology and Obstetrics, Würzburg, Germany*
*[3]University of Würzburg, Institute of Pathology, Würzburg, Germany*

**Introduction**

Immune checkpoint blockade (ICB) targeting PD/L-1 has recently been approved for triple-negative breast cancer (TNBC), but response rates are variable and independent of primary tumor PD-L1 expression. Emerging evidence suggests that tumor-draining lymph nodes (TDLNs) play a critical role in the response to ICB therapy. Furthermore, TDLN involvement is a major determinant of the occurrence of distant metastasis. In the steady state, however, LNs are responsible for effective immune responses, which are initiated upon well-organized cellular networks. How these cellular networks are reorganized to support cancer metastasis and TDLN-mediated immune evasion, via the PD/L-1 axis, remains unclear.

**Material and Methods**

We combined patient samples and several TNBC mouse models with state-of-the-art technologies, such as single-

© 2024 The Authors. Molecular Oncology is published by John Wiley & Sons Ltd on behalf of Federation of European Biochemical Societies cell and spatial transcriptomics, multi-color flowcytometry, proteomics and in vitro models.

**Results and Discussions**

We show critical data, on how myeloid cells, in particularly monocytes, increase in TNBC-draining lymph nodes and have immunosuppressive capacity via PD-L1. We further show how this immunosuppressive niche is established using our TNBC mouse models and tumors with varying metastatic potential. Single cell- and spatial transcriptomic and functional data indicate that TDLN fibroblasts recruit monocytes that preferentially home to and colocalize with fibroblast-rich niches in a CCL2/ CCL7 - CCR2 axis-dependent manner. These monocytes accumulate and exhibit suppressive capacity via the T cell inhibitory molecules PD-L1 and iNOS. Proteomic analysis of tumors and TDLN interstitial fluids and in vitro studies suggested that the factor inducing Ccl2/ Ccl7 expression in TDLN fibroblasts is a TLR4 ligand. Using a signature of just three of these TLR4 ligands in patient primary tumor samples, including TNC, S100A9 and LMNA, a subset of TNBC patients with significantly worse survival was identified. Most strikingly, local inhibition – targeted at the TDLN site - of the monocyte recruitment axis, either via Ccr2 or Tlr4 antagonism, in combination with anti-PD-1 inhibition resulted in reduced LN and distant metastasis in mouse models.

**Conclusion**

Our results suggest a novel mechanism by which metastatic TNBC tumors reprogram the TDLN niche to support immune evasion via PD-L1, which precedes and promotes metastasis. By identifying this mechanism, we open opportunities for improving the efficacy of ICB in TNBC patients.

### Symposium: Targeting Mitochondria in Cancer

### EACR2024-1059
### Proffered Paper: PI3Kβ acts with mTORC1 to control DRP1 phosphorylation and mitochondrial dynamics in pancreatic cancer

*B. Thibault[1], S. Arcucci[1], N. Therville[1], C. Cayron[1], D. Ferreira Da Mota[2], M. Dufresne[1], S. Najib[3], C. Basset[4], J. Guillermet-Guibert[1]*
*[1]Inserm- CRCT, Team SigDyn, Toulouse, France*
*[2]Inserm- I2MC, Histologie, Toulouse, France*
*[3]Inserm- I2MC, Team Laurent Martinez, Toulouse, France*
*[4]Inserm- CRCT, Team SigDyn - IUCT Oncopole, Toulouse, France*

**Introduction**

Molecular pathways linking PI3Ks to genetic changes account for some isoform sensitivity profiles. However, these alterations do not appear to be robust predictive factors for all cancers. The question of a link between cancer-related isoform specificity and precise genetic landscape remains unanswered. In this line of research, we recently investigated the role of PI3Kβ in pancreatic cancer (PDAC, pancreatic ductal adenocarcinoma), where oncogenic KRAS is the most frequent genetic alteration.

**Material and Methods**

We based our work on innovative genetically engineered mouse models, PI3K gene-targeted mice that mimic pharmacological inactivation. Isoform-selective conditional mice in which the catalytic activity of PI3Kβ is lost upon Cre recombinase expression was crossed with spontaneous mouse models of PDAC. Those models are based on the oncogenic KRAS mutation in the pancreas (Pdx1-KrasG12D/+ called the KC model) combined or with loss of function of p53 (Pdx1-KrasG12D/+; Trp53R172H/+ called the KPC model) or with induction of inflammation by an analog of CCK, Caerulein. To complete those genetic tools, isoform-selective PI3K inhibitors in vitro and in vivo was used as an alternative strategy to modulate PI3K function, in murine cell lines derived from the model described above as well as in the Human pancreatic cancer cell lines.

**Results and Discussions**

Mice with genetically inactive PI3Kβ in the pancreas showed increased survival upon oncogenic KRAS-induced carcinogenesis. Genetic or pharmacological inactivation of PI3Kβ in pancreatic cells cell-autonomously blocked spontaneous 3D outgrowth. PI3Kb inhibition promoted mitochondrial fission, a pathway driven by DRP1 and prevented the regulation by mTORC1 of mitochondrial dynamics. Monotherapy using the clinically-tested PI3Kβ inhibitor AZD8186 to treat in situ pancreatic tumours significantly prolonged mice survival upon cancer diagnosis. PI3Kβ inactivation altered tumour immune cell infiltration.

**Conclusion**

In conclusion, PI3Kβ activity contributes to cancer progression by preventing cytotoxic mitochondrial fission and controlling infiltration of immune cells in the pancreatic tumours.

### EACR2024-1089
### Proffered Paper: Tumour mitochondrial DNA mutations enhance checkpoint blockade in melanoma

*M. Mahmood[1], E. Minwei Liu[2], D. Sumpton[1], K. Blyth[1,3], E. Roberts[1,3], E. Reznik[2], P. Gammage[1,3]*
*[1]CRUK Scotland Institute, Glasgow, United Kingdom*
*[2]Memorial Sloan Kettering Cancer Center, Computational Oncology Service, New York, USA*
*[3]School of Cancer Sciences, University of Glasgow, United Kingdom*

**Introduction**

Mitochondria are essential metabolic organelles possessing mitochondrial DNA (mtDNA), a multi-copy genome where the relative ratio of mutant to wild-type mtDNA is described as heteroplasmy. Mutations in mtDNA are highly prevalent in tumours, with sequencing studies reporting >50% of tumours bear somatic mtDNA mutations. However, functional interrogation of mtDNA mutant tumour biology has been lacking due to historically limited capacity to manipulate the genome. To address this, we adapted novel mitochondrial base editing tools to model highly recurrent truncating

© 2024The Authors. Molecular Oncology is published by John Wiley & Sons Ltd on behalf of Federation of European Biochemical Societies mutations in complex I of the mitochondrial respiratory chain.

**Material and Methods**

B78-D14, Hcmel12 and 4434 murine melanoma cells were transfected with base editors targeting complex I subunit Mt-Nd5 at m.12,436. Hcmel12 cells were additionally transduced with cytoLbNOX, a cytoplasmic NADH oxidase. Animal experiments were done in accordance with the UK Animals Act 1986.

**Results and Discussions**

In vitro, mtDNA mutation-dependent loss of NADH oxidation by complex I promoted a Warburg-like metabolic shift, with cells exhibiting increased glycolytic flux with high lactate output, confirming that mtDNA mutations can influence metabolic phenotypes in cancer cells. We next studied their tumour biology in vivo, discovering that metabolic changes seen in mutant tumours led to an up-regulation of pro-inflammatory pathways and a significant decrease of tumour-resident neutrophils, with no impacts on tumour growth. This altered composition of the tumour microenvironment sensitised mutant tumours to immune checkpoint blockade (ICB) in a mutation dose-dependent manner. Human clinical trial data reinforced these observations as patients with >50% mutant heteroplasmy cancers demonstrated a >2.5 fold enhanced response to ICB. Curiously, tumours expressing cytoLbNOX showed the strongest response to treatment. In contrast with complex I mutant cells, cytoLbNOX cells exhibit an increased rather than decreased NAD+:NADH ratio, suggesting that alteration of redox state is sufficient to sensitise tumours to ICB. Furthermore, this sensitisation could be manipulated by increasing tumour-resident neutrophils in tumours, decreasing sensitisation to ICB and suggesting a role for neutrophils in mediating response to treatment.

**Conclusion**

Together, these data provide mechanistic understanding of the effects highly recurrent mtDNA mutations exert in tumour biology, demonstrating potential to stratify patients for ICB response and generate novel therapeutic approaches in melanoma.

### Symposium: Liquid Biopsies

### EACR2024-0396
### Proffered Paper: Study of Epigenetic Tumor Plasticity through the carcino-genesis and progression of Non Small Cell Lung Cancer (NSCLC) in liquid biopsy

*M. Burdiel*[1]
[1]*IdiPAZ- HULP, Biomarkers and Experimental Therapeutics in Cancer, Madrid, Spain*

**Introduction**

The prognosis of patients with Non-Small Cell Lung Cancer (NSCLC) has improved in recent years thanks to the approval of new drugs directed at therapeutic targets. However, the molecular alterations responsible for 85% of NSCLC remain unidentified. In this sense, epigenetics and liquid biopsy provide potential biomarkers to carry out long-term studies on the molecular changes along the carcinogenesis, tumor progression and treatments in a single patient. Nonetheless, the fact that molecules of tumor and non-tumor origin are mixed in the blood of cancer patients and the lack of specific normalizers has led to heterogeneous results and worsened the identification of specific tumor-origin markers.

**Material and Methods**

We performed an innovative approach encompassing comprehensive analysis of the transcritome (mRNA and miRNAs) and DNA methylation profiles (via EPIC methylarrays) across adjacent-normal and tumor tissue explants together with their extracellular vesicle contents secreted to the culture medium, totalling 60 samples, to isolate tumor-specific characteristics. Subsequently, we tracked the identified profiles in blood samples collected longitudinally from the same patients throughout their disease trajectory, spanning various treatments until relapse or decease. Additionally, validation will extend to a separate cohort of over 400 NSCLC patients and healthy controls.

**Results and Discussions**

We are validating the miRNome identified in liquid biopsy, consisting of 28 miRNAs across 480 patient plasma samples using Taqman OpenArray technology. Simultaneously, bioinformatics analysis on EPIC arrays will decipher DNA methylation profiles in both tissue and liquid biopsy samples. Additionally, we have integrated omics data obtained from the smallRNAseq and RNAseq analyses conducted on paired tumor-adjacent lung tissue samples. This comprehensive approach has enabled us to uncover 158 miRNA-mRNA pairs involving 77 miRNAs and 99 distinct genes, followed by subsequent functional enrichment analyses.

**Conclusion**

In conclusion, this endeavour represents a pioneering cross-sectional studies aimed at establishing a longitudinal epigenetic signature for NSCLC carcino-genesis and progression, offering crucial insights for personalized therapeutic interventions.

### EACR2024-0835
### Proffered Paper: Early detection of prostate cancer by liquid biopsy exploiting DNA damage sensitivity

*E. Dillier*[1], *A. Faisst*[1], *J. Godau*[1], *D. Engeler*[2]
[1]*4D Lifetec AG, Research and Development, Cham, Switzerland*
[2]*Kantonsspital St. Gallen, Department of Urology, St. Gallen, Switzerland*

**Introduction**

Prostate cancer is estimated to cause 1.4 million new cancer cases and 375,000 cancer deaths annually worldwide. It is one of the most frequent cancers in men. The current diagnosis based on serum prostate-specific antigen levels and digital rectal examination followed by tissue biopsy has a high false positive detection rate and causes unnecessary expensive interventions. This gives rise to the need of more specific early prostate cancer detection methods. Recently, the use of circulating biomarkers such as circulating tumor DNA and cells, driven from minimally invasive liquid biopsy has received tremendous attention. Despite the progress being made in this field, there are still considerable

Molecular Oncology 18 (Suppl. 1) (2024) 1–495
© 2024 The Authors. Molecular Oncology is published by John Wiley & Sons Ltd on behalf of Federation of European Biochemical Societies technical limitations in terms of sensitivity especially in the early cancer detection due to the low abundance of the mentioned biomarkers.

**Material and Methods**

Therefore, we propose to exploit the influence tumor cells exert already in early disease on the highly abundant peripheral blood mononuclear cells (PBMCs). In an observational study, 'Prospective Evaluation of 4D Lifetest™ Parameters to Develop a Universal Early Cancer Diagnosis Test', blood samples from 46 participants were collected (50% patients with newly diagnosed, untreated prostate cancer and 50% non-cancer) and PBMCs isolated. They were exposed to UV-B radiation and DNA damage sensitivity (DDS) quantified with high-performing single-cell gel electrophoresis.

**Results and Discussions**

Evaluation of DDS comparing non-cancer with cancer samples resulted in more than 95% sensitivity at 95% specificity (95% CI: 79.0% to 99.8%) across all stages. The mean DDS for early-stage prostate cancer I and II did not differ significantly from late-stage III and IV, suggesting high performance for early detection. Disease-specific gene regulatory landscapes inferred from DNAse I hypersensitive sites confirm that gene expression changes affect DNA structure. Additionally, specific differences detected in PBMCs transcriptomes of breast cancer patients have been shown to be promising early diagnostic biomarkers. Combined these findings support our hypothesis that DDS of PBMCs can be used for early prostate cancer detection.

**Conclusion**

We demonstrate the DDS biomarker assay's potential in detecting prostate cancer at an early stage with high accuracy in a simple and non-invasive way. These data suggest that this assay is expected to become a practical method to support clinical diagnostics.

*Authors listed under the institution "4D Lifetec AG, Cham, Switzerland" have a financial interest in products or processes described in the abstract, namely the 4D Lifetest™.*

### Symposium: Drugging Tumour Suppressor Genes

### EACR2024-0567
### Proffered Paper: Novel CRISPR-Cas9 mediated efficient demethylation and reactivation of transcription of tumor suppressor genes

*P. Castellanos Silva[1], L.L. Stemmler[1], A.M. Richter[2]*
*[1]Justus-Liebig-University, Institute for Genetics, Giessen, Germany*
*[2]Justus-Liebig-Universtity, Institute for Genetics, Giessen, Germany*

**Introduction**

One of the hallmarks of cancer, the non-mutational epigenetic reprogramming, appears as a promising approach to correct the expression of relevant genes in carcinogenesis. Tumor suppressor genes, normally expressed in healthy cells, often show an increase in methylation of their CpG islands in promoter regions (hypermethylation) in many cancer cells. This characteristic of many tumors leads to a non-detectable or lowered expression of those genes. During DNA methylation in humans the DNA methyltransferases (DNMT´s) convert cytosine from CG-regions into 5´-methylcytosine. As a result, the chromatin condenses, which closes the promotor region, so that transcription factors can not bind and stimulate transcription of these genes. Given the fact that DNA methylation is a reversible process, our research focusses on the precise target gene demethylation for cancer suppression as a therapeutic tool.

**Material and Methods**

Therefore, we have developed an improved demethylation strategy based on the targeted approach by CRISPR-dCas9. To open the chromatin again and re-express the gene we make use of a modified and nuclease inactivated Cas9 enzyme (dCas9), originally discovered in bacteria as part of the CRISPR-Cas system.

**Results and Discussions**

By coupling the dCas9 with novel and efficient effectors and giving it directional guides to localize the effect in the promotor region of the gene of interest, we succeeded in significant demethylation and reactivation of transcription of tumor suppressor genes in our cell culture models. This is accompanied by the reestablishment of tumor suppressor function.

**Conclusion**

The identification of tumor-associated genes and their targeted reactivation in cancer offers us extensive research opportunities, which may lead to patient-specific therapy in the future.

### EACR2024-0890
### Proffered Paper: Thyroid hormone receptor beta is a novel tumor suppressor in prostate cancer

*A. Fesiuk[1,2], D. Pölöske[1,3,4], O. Aksoy[1,5], O. Merkel[1,6], B. Hantusch[1], L. Kenner[1,2,7,8]*
*[1]Medical University of Vienna, Department of Pathology, Vienna, Austria*
*[2]Medical University Vienna, Christian Doppler Labor for Applied Metabolomics CDL-AM, Vienna, Austria*
*[3]University of Veterinary Medicine, Unit of Functional Cancer Genomics- Institute of Animal Breeding and Genetics, Vienna, Austria*
*[4]Medical University Vienna, Christian Doppler Labor for Applied Metabolomics CDL-AM-, Vienna, Austria*
*[5]Karl Landsteiner University of Health Sciences, Department for Basic and Translational Oncology and Hematology- Division Molecular Oncology and Hematology, Krems, Austria*
*[6]European Research Initiative, on ALK-Related Malignancies ERIA, Cambridge, United Kingdom*
*[7]University of Veterinary Medicine, Unit of Laboratory Animal Pathology, Vienna, Austria*
*[8]Center for Biomarker Research, in Medicine CBmed, Graz, Austria*

**Introduction**

Prostate cancer (PCa), the second most common malignancy in men, relies heavily on androgen receptor (AR) signaling for tumor development. Resistance to

© 2024 The Authors. Molecular Oncology is published by John Wiley & Sons Ltd on behalf of Federation of European Biochemical Societies

AR-targeted therapies in 20% of patients highlights the necessity for innovative treatment strategies. In addition, hyperthyroidism is recognized as a PCa risk factor, and a growing body of literature suggests that thyroid hormone receptors (THRs) contribute to cancer development and progression. PCa samples sequenced in our lab showed enrichment of mutated thyroid hormone (TH) signaling genes. Our goal was to investigate the role of TH signaling in PCa.

**Material and Methods**

Analysis of *THRB* mRNA levels was performed with publicly available RNAseq datasets. TRβ expression was assessed by IHC on PCa biopsies and correlated with time to biochemical recurrence (BCR). PCa cell lines were treated with T3 and a TRβ selective inhibitor and cell proliferation was assessed by resazurin assays and FACS. Gene expression changes were monitored by qRT-PCR and Western blotting. RNA-seq of PCa cells undergone treatment with the TRβ-selective inhibitor was performed. For in vivo analyses, 22Rv1 cells were subcutaneously injected into NSG mice and mice were treated intraperitoneally (i.p.) or orally with the TRβ selective inhibitor.

**Results and Discussions**

Publicly available dataset analyses showed elevated levels of *THRB* mRNA in PCa compared to healthy controls. Moreover, significantly higher TRβ expression was observed in IHC stainings of PCa tissues, which correlated with faster time to BCR. In a range of PCa cell lines, TRβ expression was assessed, and enhanced cell proliferation was observed upon T3 stimulation. The significance of TH signaling on PCa cell viability was investigated by pharmacological blockade using the TRβ-selective inhibitor. Treatment resulted in dose-dependent inhibition of cell proliferation in LNCaP and 22Rv1, which was reflected by gene expression changes associated with cell cycle regulation, such as CDKN1A and downregulation of AR and AR-targeted genes. Further, administration of the TRβ inhibitor in 22Rv1 xenografted mice. i.p. and orally resulted in significantly smaller tumor volumes and masses.

**Conclusion**

Our results show the significance of TH signaling in PCa development and progression, as demonstrated by human data, in vitro and in vivo. Further studies will focus on investigating the molecular mechanism of TRβ-driven gene expression changes in PCa.

### Joint EACR-ESR Symposium: Imaging the Tumour Microenvironment

### EACR2024-0353

### Proffered Paper: From breast cancer cell homing to the onset of early bone metastasis: The role of bone (re)modeling in early lesion formation

*S.A.E. Young[1], A.D. Heller[1], D.S. Garske[1], M. Rummler[1], V. Qian[1], A. Ellinghaus[2], G.N. Duda[2], B.M. Willie[3], A. Grüneboom[4], A. Cipitria[5]*

*[1]Max Planck Institute of Colloids and Interfaces, Biomaterials, Potsdam, Germany*

*[2]Charité – Universitätsmedizin Berlin, Julius Wolff Institute, Berlin, Germany*

*[3]McGill University, Shriners Hospital for Children - Canada, Shriners Hospital for Children - Canada, Canada*

*[4]Leibniz-Institute for Advancing Analytics – ISAS, Biospectroscopy, Biospectroscopy, Germany*

*[5]Biogipuzkoa Health Research Institute, Bioengineering, San Sebastián, Spain*

**Introduction**

Breast cancer cells frequently home to bone leading to osteolytic lesions and lowering the prognosis of survival. Much work has been reported on advanced overt metastasis, but less is known about the early phases of metastasis. Further, structural and biophysical changes are rarely studied, yet are hypothesized to influence metastatic progression.

**Material and Methods**

We developed a mouse model of early bone metastasis and multimodal imaging to quantify cancer cell homing, dynamic bone (re)modeling and onset of metastasis. Bone-tropic human metastatic breast cancer cells (MDA-MB-231 BoM 1833) were injected intracardially into BALB/c nude mice. Animals were monitored with in vivo longitudinal microcomputed tomography (microCT) to quantify dynamic bone (re)modeling. Using tissue clearing and 3D light sheet fluorescence microscopy (LSFM), we located eGFP$^+$ breast cancer cells and small clusters in 3D (intact) bones and quantified their size and spatial distribution. We developed a new microCT image analysis tool to detect and track the growth of early lesions over time. Finally, we characterized the structural microenvironment around early bone lesions with multiscale imaging.

**Results and Discussions**

With LSFM, we showed evidence of heterogenous spatial distribution of single cancer cells, small and larger clusters in 3D, in the absence of detectable bone lesions. We showed that cancer cells home to all bone compartments and are in close proximity to bone surfaces. With the new microCT image analysis tool, we could detect and track early bone lesions in cortical bone, quantifying their onset, specific location and growth. Lesions were only detected in the metaphysis, a region of high (re)modeling. We characterized the changes in the structural microenvironment using multiscale tissue characterization with backscattered electron microscopy, confocal and second-harmonic generation imaging, and histology. Immunofluorescence depicted breast cancer cells and small clusters based on eGFP$^+$ fluorescence, and their proliferative status with Ki67 marker. In vivo microCT revealed altered bone remodeling in the absence of detectable bone lesions, suggesting an early systemic effect of cancer cells.

**Conclusion**

We showed that cancer cells home in all bone compartments, while osteolytic lesions are only detected in the metaphysis, a region of high (re)modeling. Our study [1] suggests that higher rates of (re)modeling act as a driver of lesion formation during early metastasis.

*[1] Young et al., Sci. Adv. 10, eadj0975 (2024)*

© 2024 The Authors. Molecular Oncology is published by John Wiley & Sons Ltd on behalf of Federation of European Biochemical Societies

### EACR2024-0426

### Proffered Paper: Patient-derived 3D-printed tumours as a relevant tool for pre-clinical research and drug development

K. Pawlicka[1], M. Raab[1], L. Watt[1], E. Smith[1], B. Kennedy[1]
[1]Carcinotech, Scientific Department, Edinburgh, United Kingdom

**Introduction**

Several advances in tissue engineering, including 3D bioprinting, pushed the development of more physiologically relevant 3D in vitro cancer models, allowing not only for more closer representation of the tumour microenvironment (TME), but also creation of a high-throughput drug screening platform. To accelerate drug development and pre-clinical testing, we propose a novel, patient cell-derived, 3D-printed lung cancer model. We incorporated cells isolated from the patient's own cancer biopsy, including cells from the TME like cancer stem cells, CAFs, tumour-infiltrating immune cells, and extracellular matrix components, known to play critical roles in tumorigenesis and metastasis and contribute towards drug resistance. As such, these advanced 3D constructs allowed to replicate TME with high accuracy.

**Material and Methods**

Patient tumour biopsies were sectioned, processed into FFPE blocks and TME was assessed using immunofluorescence (IF) technique. Patient-derived cells were cultured as a heterogeneous population and 3D-bioprinted with custom TME bioink onto 96-well. After 14 days in culture, characterisation of 3D-model composition was performed. Comparison of original tumour and bioprinted tumour with relevant downstream markers was carried out to assure high representation of the original sample. Additionally, immunotherapeutic and standard of care drug treatment was performed to assess the response and changes in cellular composition of bioprints using viability, cytotoxicity, apoptosis assays as well as visualisation through IF microscopy.

**Results and Discussions**

Characterisation of tumour tissues was performed to assure the development of 3D-printed models mimicking the original cancer. The analysis involved determination of the immune cell composition and TME carried out via immunofluorescence and flow cytometry. To characterise our engineered lung cancer models and ensure the presence of the initial cell types, we have developed a staining protocol for the whole 3D-prints. We have successfully shown that the immune and tumour cell types identified in the initial tissue characterisation are still present in our 3D lung tumour models 14 days post-printing, indicating the accuracy of the manufactured models. Lastly, we proved that the 3D-printed tumours respond to standard of care and immunotherapeutic treatment.

**Conclusion**

We demonstrated that our 3D-printed tumours offer a platform for in vitro high-throughput, accurate and rapid drug discovery and screening for novel, combinatorial and repurposed drugs.

### Symposium: Making Cold Tumours Hot

### EACR2024-0543

### Proffered Paper: The C5a/C5aR1 axis has an immunosuppressive function in cancer-associated dendritic cells: implications for cancer immunotherapy

Y. Senent Valero[1,2,3], D. Repáraz[2,4,5], D. Llopiz[2,4,5], R. Entrialgo-Cadierno[1,2,6], L. Suarez[3], A. Rouzaut[2,3], B. Tavira[1], P. Sarobe[2,4,5], D. Ajona[1,2,3,6], R. Pio[1,2,3,6]
[1]Cima-Universidad de Navarra, Department of Solid Tumors- Cancer Division- Cancer Center Clinica Universidad de Navarra CCUN, Pamplona, Spain
[2]Navarra's Health Research Institute IDISNA, Oncology, Pamplona, Spain
[3]Universidad de Navarra, Department of Biochemistry and Genetics- School of Sciences, Pamplona, Spain
[4]Cima-Universidad de Navarra, Department of Immunology and Immunotherapy- Cancer Division- Cancer Center Clinica Universidad de Navarra CCUN, Pamplona, Spain
[5]Centro de Investigación Biomédica en Red Enfermedades Hepáticas y Digestivas CIBEREHD, Enfermedades Hepáticas y Digestivas, Madrid, Spain
[6]Centro de Investigación Biomédica en Red Cáncer CIBERONC, Lung cancer, Madrid, Spain

**Introduction**

The complement system, a central component of the innate immune response, co-opts the tumor microenvironment (TME) to facilitate tumor progression. Nevertheless, the role of complement in cancer-associated DCs, a myeloid population with unique functions in tumor immunity, remains unknown. In this study, we explored the impact of complement C5a, in its receptor C5a receptor 1 (C5aR1), on the biology of cancer-associated DCs and the implications for cancer immunotherapy.

**Material and Methods**

C5aR1 expression was evaluated in cancer-associated DCs in human and mouse lung adenocarcinomas using scRNAseq and flow cytometry data. Ex vivo studies (OT-I/OT-II presentation assays, FITC-dextran uptake assays) were performed to assess the effect of the C5a/C5aR1 axis on the biology of cancer-associated DCs. In vivo functional studies with syngeneic tumor models and transgenic models (Vert-X (B6(Cg)-Il10tm1.1Karp/J) mice, Kikume Green-Red (KikGR) mice) were conducted to evaluate the role of C5a/C5aR1 signaling in the antitumor responses. Synergistic responses were characterized by flow cytometry, multiplexed immunofluorescence and RNAseq analysis.

**Results and Discussions**

C5aR1 expression in both human and mouse tumor-infiltrating DCs was associated with a tolerogenic phenotype, and was restricted to type 2 conventional DCs (cDC2) and monocyte-derived DCs (moDCs). Functionally, C5aR1-expressing DCs were able to inhibit C5aR1-negative DC priming of T cells. Moreover, the engagement of C5aR1 drove the migration of tolerogenic DCs from tumors to tumor-draining lymph nodes and the

© 2024 The Authors. Molecular Oncology is published by John Wiley & Sons Ltd on behalf of Federation of European Biochemical Societies production of IL-10 in the TME. We leveraged this knowledge to optimize an anticancer therapy aimed at boosting DC activity. C5aR1 inhibition significantly enhanced the effectiveness of poly I:C, a toll-like receptor 3 (TLR3) agonist, combined with Programmed cell death protein 1 (PD-1) blockade. The therapeutic effect was associated with high numbers of tumor-specific CD8 T cells, an increase in interferon gamma (IFN-γ) expression and signaling, antigen processing and presentation and cytotoxic response.

**Conclusion**

This study reveals the importance of C5aR1 in the biology of cancer-associated DCs, emerging as a tolerogenic-associated marker with the capacity to modulate DC migration and cytokine production. Our study provides compelling evidence that supports the therapeutic potential of modulating the complement system to enhance DC-mediated immune responses against solid tumors.

### EACR2024-0641

### Proffered Paper: Patient-specific co-cultures of lung cancer organoid libraries and T-cells reveal immune evasion at the level of individual cancer subclones

*K. Dijkstra*[1], *R. Vendramin*[2], *D. Karagianni*[3], *F. Gálvez-Cancino*[3], *M. Hill*[4], *K. Foster*[5], *B. Chain*[6], *E. Voest*[1], *S. Quezada*[3], *C. Swanton*[4]

[1]*Antoni van Leeuwenhoek - Netherlands Cancer Institute, Molecular Oncology and Immunology, Amsterdam, The Netherlands*

[2]*University College London Cancer Institute, The Tumour Immunogenomics and Immunosurveillance TIGI Lab, London, United Kingdom*

[3]*University College London Cancer Institute, Immune Regulation and Tumour Immunotherapy Group, London, United Kingdom*

[4]*The Francis Crick Institute, Cancer Evolution and Genome Instability Laboratory, London, United Kingdom*

[5]*University College London Cancer Institute, Cancer Research UK Lung Cancer Centre of Excellence, London, United Kingdom*

[6]*University College London, Division of Infection and Immunity, London, United Kingdom*

**Introduction**

Subclonal immune evasion is a major barrier to achieving complete response to immune checkpoint blockade in non-small cell lung cancer (NSCLC). While tumours consist of multiple genetically distinct clones, the extent to which separate tumour subclones differ in their capacity for immune evasion, the tumour-intrinsic mechanisms underlying any such heterogeneity, and its impact on cancer immunosurveillance remain largely unexplored. So far, this has been challenging to study due to an inability to isolate, propagate and functionally interrogate individual tumour subclones from human cancers.

**Material and Methods**

We previously developed personalised co-culture systems of organoids and autologous T-cells. Here, we leverage the multi-region TRACERx lung cancer evolution study to generate a patient-derived platform that allows the evaluation of T-cell responses to individual cancer subclones. We generated libraries of >20 separate non-small cell lung cancer (NSCLC) organoid lines per patient, based on isolating individual (clonal) organoids established from multiple spatially separated tumour regions. Each organoid subline was co-cultured with autologous tumour infiltrating lymphocytes (TIL) to evaluate their capacity to elicit a T-cell response. We combined functional assays with DNA, RNA and TCR sequencing for an in-depth characterization of 44 individual organoid lines from 6 tumour regions of two patients.

**Results and Discussions**

Organoid lines derived from separate tumour regions, or from single clones within individual regions, differ vastly in their capacity to elicit a T-cell response. TIL reactivity ranged from 1-42% between organoid lines, showing the coexistence of intrinsically ‘hot’ and ‘cold’ clones in the same primary tumour. Organoid lines escaping T cell recognition represent genetically and transcriptionally unique subclones with a distinct evolutionary history. Based on the ability to recover T-cell activation upon expression of a model antigen, we show that subclonal immune escape can be driven by both antigen-dependent and -independent mechanisms.

**Conclusion**

These data show (i) that tumour evolution can give rise to distinct cancer clones with intrinsic differences in immune evasion capacity and (ii) provide an approach to prospectively identify, isolate and characterise immune evading subclones from cancer patients. This patient-derived study platform allows moving beyond descriptive analyses alone to functionally interrogate how intra-tumour heterogeneity affects cancer immunosurveillance.

### Symposium: Drugging the RAS Pathway

### EACR2024-0487

### Proffered Paper: Evaluation of a Multi-Selective RAS Inhibitor as a Therapeutic Strategy for KRAS-Mutant Cholangiocarcinoma

*R. Entrialgo*[1], *I. Feliu*[1], *I. Lopez*[1], *I. Macaya*[1], *Y. Senent*[1], *S. Vicent*[1]

[1]*CIMA, Solid Tumours, Pamplona, Spain*

**Introduction**

Cholangiocarcinoma (CCA), comprises approximately 3% of all gastrointestinal tumors, and is characterized by a highly dismal prognosis. Therapeutic strategies predominantly rely on chemotherapy, and treatment advancements have been stagnant over the past decades, underscoring the critical need for innovative therapies. Mutant KRAS is among the most prevalent oncogenes in CCA, highlighting a potential role for novel KRAS inhibitors as therapeutic avenues. Here, we present preclinical data on RMC-7977, a first-in class RAS(ON) inhibitor, as a potential therapeutic strategy for cholangiocarcinoma with KRAS mutations.

**Material and Methods**

© 2024 The Authors. Molecular Oncology is published by John Wiley & Sons Ltd on behalf of Federation of European Biochemical Societies

Human and murine KRAS mutated cell lines were utilized to determine the effect of RMC-7977 both in vitro and in vivo. RNA-seq and Western blot assays were conducted to analyse the molecular changes and potential resistance mechanisms. The tumor immune micro-environment was assessed through multiplex flow cytometry. Combinatorial pharmacological treatments were conducted in vitro and in vivo to study resistance mechanisms overcome. Both human and murine cell lines resistant to the RAS(ON) inhibitor were generated. Subsequently RNA seq and whole exome sequencing were performed to identify possible transcriptomic changes and acquired mutations in the resistant phenotype.

**Results and Discussions**

RMC-7977 elicited antiproliferative effects in mouse and human cholangial cell lines with different KRAS mutations (G12D/G12V/G13C) in vitro. In vivo experiments with human xenografts or mouse allografts revealed heterogenous antitumor responses ranging from tumor regressions to growth delay. Tumor immune phenotyping of immunocompetent mice showed a notable increase in $NK^+$, $CD8^+$, and $CD4^+$ infiltration, and a reduction of PMN-MDSCs, resulting in a less immunosuppressive microenvironment. RNA-seq and Western blot analysis revealed increased activity of the SHP2, FGFR, and JAK/STAT pathways. Combinatorial treatments showed synergistic effects with SHP2 (both in naïve and resistant models) and JAK2 inhibitors but not FGFR inhibitors. In vitro results nominate RAS(ON) plus SHP2 inhibitor combinations for in vivo testing. Identification of transcriptomic signatures and potential de novo mutations in resistant models is currently ongoing.

**Conclusion**

In summary, our preclinical findings support the clinical testing of a RAS(ON) multi-selective inhibitor, either alone or in combination with other in-pathway or out-of-pathway partners, as a potential therapeutic strategy for cholangiocarcinoma treatment.

### EACR2024-0834
### Proffered Paper: KRAS G12V neoantigen-specific therapy modalities development and assessment

*S. Sagie[1], T. Babu[2], S. Chani[3], T. Golan[4], Y. Samuels[1]*
*[1]Weizmann Institute of Science, Molecular Cell Biology, Rehovot, Israel*
*[2]Weizmann Institute, Molecular Cell Biology, Rehovot, Israel*
*[3]Sheba Medical Center, Cancer Research Center, Ramat Gan, Israel*
*[4]Sheba Medical Center, Oncology, Ramat Gan, Israel*

**Introduction**

Cancer immunotherapy revolutionized cancer treatment, presenting superior clinical outcomes relative to previous standard treatments. However, only a minority of solid cancer patients respond to immunotherapy, necessitating an innovative alternative approach. HLA-based approaches for discovering novel immunotherapy targets hold great potential to be clinically relevant to a wider range of patients. A specific subgroup of antigens, neo-antigens, have emerged as promising targets in cancer immunotherapy since they are tumor-unique and can mediate an effective tumor-directed T-cell response. Recurrent neoantigens, derived from hotspot mutations, are becoming increasingly attractive targets for immuno-therapy as they are shared among large groups of patients and may pave the way toward "off-the-shelf" cancer therapies. KRAS is one of the top driver genes across all solid cancers, and the G12V mutation is frequent in 30% of pancreatic cancer patients, 10% of colon cancer patients and 6% of Lung adenocarcinoma patients.

**Material and Methods**

We combined bioinformatic prediction tools, with HLA peptidomics and immunological essays, to identify and validate a neoantigen from the KRAS G12V:HLA-A*03:01 combination and its cognate TCR.

**Results and Discussions**

We have identified a neoantigen derived from KRAS G12V mutation and the HLA-A*03:01 common allotype-VVVGAVGVGK. We validated the presentation of this neoantigen in monoallelic B-cells introduced with a minigene including the mutation and in endogenous expressing cell lines from both pancreatic cancer and lung cancer, with heavy peptides. We found a robust, and specific immune response of donors' T-cells against this peptide and not against the corresponding WT peptide. We isolated the reactive T-cells, by a peptide-specific dextramer, for single-cell TCR and RNA sequencing and identified a cognate TCR. We retrovirally introduced the TCR to naïve donor PBMCs and found that the TCR is highly reactive against the mutated peptide in nM concentrations and not against the WT peptide. The TCR also induces the killing of endogenous KRAS G12V-expressing cancer cells.

**Conclusion**

We have identified a potent and specific TCR for a frequent driver mutation. This TCR could be clinically relevant for thousands of patients every year.

### Joint EACR-EMBO Symposium: Modelling Cancer

### EACR2024-0633
### Proffered Paper: Identifying Ewing sarcoma developmental mechanisms using zebrafish genetic models

*E. Vasileva[1], C. Arata[2], Y. Luo[1], G. Crump[2], J. Amatruda[1]*
*[1]Children's Hospital Los Angeles, Cancer and Blood Disease Institute, Los Angeles, United States*
*[2]University of Southern California, Keck School of Medicine, Los Angeles, United States*

**Introduction**

Ewing sarcoma (ES) is a pediatric cancer of bones or soft tissues with an extremely poor prognosis for patients with metastatic or relapsed disease. ES is associated with the appearance of one of several chimeric fusion oncogenes, most frequently EWSR1-FLI1 (EF1), which drives tumor development. Intriguingly, due to the high toxicity of the driver oncofusion, there is no genetic mouse model. In the absence of a representative in vivo model, the factors facilitating tumor initiation and progression remain a

© 2024 The Authors. Molecular Oncology is published by John Wiley & Sons Ltd on behalf of Federation of European Biochemical Societies subject of constant debate. Here, using our zebrafish model of ES, we track the behavior of pre-cancer cells using high-resolution imaging approaches and show that EF1 hijacks developmental pathways during cell transformation and tumor development.

**Material and Methods**

We developed a new in vivo genetic model of ES by integrating the human EF1 fusion oncogene into the zebrafish genome (Vasileva et al., 2022, eLife). Our system allows broad and tissue-specific expression of human EF1 in zebrafish, which causes rapid onset of ES at high penetrance. Here, we use innovative genetic, epigenetic, and transcriptomic approaches in combination with multiplex RNA in situ hybridization to better understand early events of EF1-induced tumorigenesis.

**Results and Discussions**

Our novel zebrafish model of ES allows us to study the behavior of GFP-labeled cancer cells throughout tumor initiation and progression, which is not currently possible in mammals. Using this model, we tracked the behavior of EF1-expressing cells from the early stages of zebrafish development. We observed that expression of the onco-fusion begins at the bud and early segmentation stages, being selectively tolerated by normal cells. Additionally, we demonstrated that EF1 expression is associated with the activation of early developmental regulators, including the mesodermal regulator tbxta (human T or Brachyury). Strikingly, our findings indicate that EF1 expression triggers an abnormal program of fin development, involving the activation of HOX genes and FGFR signaling, thereby engaging surrounding tissue in the formation of outgrowths and tumor propagation.

**Conclusion**

Taken together, using our zebrafish genetic model of ES, we tracked the behavior of pre-cancer cells throughout organism development and characterized early events of EF1-induced cell transformation. In this study, we demonstrate that EF1 can induce tumorigenesis by aberrantly activating developmental pathways, ultimately leading to tumor development in zebrafish.

### EACR2024-1176
### Proffered Paper: Dissecting the mechanistic basis of chromothripsis, one step at a time

*G. Philippos[1,2], W. Zaatra[3], P. Smirnov[1,4], J. Otoničar[1,2], O. Lazareva[4,5], G. Sigismondo[6], J. Korbel[4,7], O. Stegle[4,5,8], B. Kerem[3], A. Ernst[1]*

*[1]German Cancer Research Center, Group Genome Instability in Tumors, Heidelberg, Germany*
*[2]Heidelberg University, Faculty of Biosciences, Heidelberg, Germany*
*[3]The Hebrew University, Department of Genetics, The Life Sciences Institute, Jerusalem, Israel*
*[4]European Molecular Biology Laboratory, Genome Biology Unit, Heidelberg, Germany*
*[5]German Cancer Research Center, Division of Computational Genomics and Systems Genetics, Heidelberg, Germany*
*[6]German Cancer Research Center, Section Translational Cancer Epigenomics - Division of Translational Medical Oncology, Heidelberg, Germany*
*[7]German Cancer Research Center, Bridging Research Division on Mechanisms of Genomic Variation and Data Science, Heidelberg, Germany*
*[8]Wellcome Sanger Institute, Wellcome Trust Genome Campus, Cambridge, United Kingdom*

**Introduction**

Chromothripsis (CT) is a form of genome instability detected in 30 to 50% of all cancer cases. Even though CT plays a major role in tumor development, direct evidence of what predisposes cells to CT is missing. Although several mechanisms have been proposed, the mechanistic basis of CT and the precise sequence of events are still poorly understood. The timing of telomere stabilization, which is essential for chromothriptic cells to proliferate and to avoid further catastrophic events is still largely unclear. Early events in cancer evolution are challenging to capture in humans, as fully developed tumors are analyzed in the vast majority of cancer studies.

**Material and Methods**

To answer these questions, we perform longitudinal analyses starting from cells without CT and analyze the clonal evolution up to the appearance of a dominant clone with CT. We use a unique model of spontaneous CT in primary fibroblasts from Li-Fraumeni syndrome patients (germline *TP53* variant) to study CT under physiological conditions and without artificial induction. We apply a multi-disciplinary approach combining single-cell genomics (strand-seq, PTA, HIPSD&R-seq), transcriptomics, proteomics, metabolomics and functional studies.

**Results and Discussions**

Phenotypic profiling identifies chromosome bridges multipolar spindles and micronuclei already at early passages. DNA sequencing revealed a high diversity of CT events at early passages and later selection of dominant clones. Furthermore, telomere stabilization happens shortly after the crisis and leads to dominant clones. "Winning subclones" show extrachromosomal circular DNAs and gene fusions. RNA sequencing and mass-spectrometry-based proteomics identifies critical pathways that play a role in CT initiation and in clonal dominance after CT occurrence. We also unveil a potential novel mechanism of replication stress following wild type p53 loss due to nucleotide mis-regulation, which leads to DNA damage and micronuclei driving CT initiation.

**Conclusion**

Altogether, this research will lead to a better understanding of the underlying biological processes leading to CT. This will be a prerequisite for laying the basis for the development of novel intervention strategies.

### Symposium: Ageing, Gender, and Cancer

### EACR2024-0621
### Proffered Paper: Evolutionary Subtypes of Prostate Cancer

*D. Wedge[1], D.J. Woodcock[2], A. Sahli[1], R. Teslo[3],*

© 2024 The Authors. Molecular Oncology is published by John Wiley & Sons Ltd on behalf of Federation of European Biochemical Societies

D.S. Brewer[4], R. Eeles[5], C. Cooper[4]
[1]University of Manchester, Manchester Cancer Research Centre, Manchester, United Kingdom
[2]University of Oxford, Duffield department of Surgery, Oxford, United Kingdom
[3]Wellcome Sanger Institute, Canger Ageing and Somatic Mutation, Hinxton, United Kingdom
[4]University of East Anglia, Norwich Medical School, Norwich, United Kingdom
[5]Institute of Cancer Research, Oncogenetics, London, United Kingdom

**Introduction**

The development of cancer is an evolutionary process, involving the acquisition of complementary genetic alterations that confer carcinogenic functionality. In this study, we aimed to discover whether subsets of prostate cancers undergo differing evolutionary trajectories in a cohort of 159 Whole Genome Sequenced prostate cancers.

**Material and Methods**

We investigated the genomic evolution of prostate cancer through the application of three separate classification methods that each identified characteristic imprints in the tumour genome that arise from different evolutionary behaviour. Integrating the results revealed the existence of two distinct subtypes of prostate cancer that arise from divergent evolutionary trajectories. These are designated the Canonical and Alternative evolutionary subtypes, or ‘evotypes’.

**Results and Discussions**

While no single genetic aberration is sufficient to define either trajectory, we are able to identify a number of common characteristics of each evotype. Canonical tumours are more likely to be ETS-positive; to have copy number loss affecting *TP53*; and to be enriched for structural variations (SVs) close to Androgen Receptor (*AR*) binding sites (ARBS). Alternative evotype tumours, on the other hand, are more likely to have losses of a number of chromosomal regions including those covering *CHD1*, *MAP3K7* and *IL6ST*; catastrophic complex genomic events including kataegis and chromothripsis; mutations in *SPOP*; and depletion for SVs close to ARBS, suggesting non-canonical androgen receptor DNA binding in this evotype. *CHD1* protein is involved in *AR* binding, which causes DNA loops that can precipitate double strand breaks, and we observe further reduction in SVs close to ARBS in tumours with loss of *CHD1*, supporting a role for *CHD1* inactivation in AR dysregulation.

**Conclusion**

The Alternative evotype is more aggressive than the Canonical evotype, with an associated hazard ratio for biochemical recurrence of 2.3. The evotype classification is independent of ETS status and Gleason grade, yet this model unifies many previous molecular observations in prostate cancer. The evotypes concept provides a powerful paradigm for cancer stratification, which we anticipate yielding fruitful results when applied to other tumour types.

### EACR2024-0657
### Proffered Paper: Multiomic Landscape of Chemo-Refractory Ovarian Cancer

D. Afenteva[1], T.A. Muranen[1], A. Rajavuori[2], K. Lavikka[1], K. Zhang[1], I.M. Launonen[1], A. Vähärautio[1], A. Färkkilä[1], J. Hynninen[2], S. Hautaniemi[1]
[1]University of Helsinki, Faculty of Medicine, Helsinki, Finland
[2]University of Turku and Turku University Hospital, Department of Obstetrics and Gynecology, Turku, Finland

**Introduction**

Chemotherapy resistance in ovarian high grade serous carcinoma (HGSC) represents a critical challenge, with a subset of patients not responding to standard treatment, highlighting the urgent need for novel therapeutic strategies. Our research focuses on these primarily chemo-refractory HGSC patients treated with neoadjuvant chemotherapy (NACT), employing multi-omic analyses to uncover molecular drivers of resistance.

**Material and Methods**

We established a subcohort of 31 NACT patients with chemo-refractory HGSC from the prospective, longitudinal, observational DECIDER trial (NCT 04846933). Chemo-refractory status was determined based on RECIST 1.1 criteria, indicating stable or progressive disease after primary therapy. For comparative analysis, we selected 62 chemo-sensitive patients with the same baseline treatment but showing at least a partial response to primary therapy and a platinum-free interval over six months. To discover the molecular drivers of the primarily chemo-refractory disease, we integrated bulk RNA-sequencing (bulk RNA-seq), single-cell RNA-sequencing (scRNA-seq), whole-genome sequencing (WGS), and single-cell proteomic (t-CyCIF) data from treatment-naive HGSC tumors.

**Results and Discussions**

Analysis of pathway activity using PROGENy, supported by Gene Set Enrichment Analysis on bulk RNA-seq data, revealed a suppression of the interferon alpha response pathway ($P < 0.001$) in cancer compartment of chemo-refractory compared to chemo-sensitive patients. Moreover, Cox proportional hazards model analysis indicated that higher pathway activity was associated with prolonged overall survival (HR = 0.73, 95% CI = 0.58-0.92, $P = 0.007$) in the whole DECIDER cohort ($n = 162$). Analyzing the pathway heterogeneity with scRNA-seq identified a fraction of cancer cells with highly active interferon alpha response in chemo-sensitive tumors. This observation was supported by t-CyCIF, highlighting distinct clusters of pSTAT1-positive cells, underscoring the pathway's variability. Genomic analysis revealed no differences in *TP53* mutations, homologous recombination repair genes, or oncogenic drivers between chemo-refractory and chemo-sensitive HGSC patients.

**Conclusion**

In conclusion, our in-depth study presents the molecular landscape of HGSC with intrinsic resistance to platinum and reveals the pivotal role of basal interferon alpha response pathway activity in mediating chemotherapy response in HGSC.

© 2024The Authors. Molecular Oncology is published by John Wiley & Sons Ltd on behalf of Federation of European Biochemical Societies

### POSTER PRESENTATIONS (Tuesday/Wednesday)

### Bioinformatics and Computational Biology

### EACR2024-0036
### Pan-cancer analyses verified the ubiquitination-related gene TRIM54 as a prognostic biomarker and immunotherapy predictor

*X.D. Yu[1], R.J. Liu[1], W.F. Gao[1], S.S. Yan[1], X.B. Zao[1], Y.S. Zhang[1]*
*[1]Beijing University of Chinese Medicine, Dongzhimen Hospital, Beijing, China*

**Introduction**
Ubiquitination modification significantly impacts biological processes and regulates tumor micro-environment (TME). Tripartite Motif Containing 54 (TRIM54) is an E3 ubiquitin ligase, which, in recent years, has been shown to control how certain malignancies develop.
**Material and Methods**
Data from the public databases were utilized to explore the expression, prognostic value, clinical features, enrichment analysis, and genetic alterations of TRIM54 in tumors. Associations between TRIM54 expression and immune cell infiltration (ICI), immunotherapy response, and targeted drugs were performed by bioinformatics analysis.
**Results and Discussions**
TRIM54 was expressed abnormally in a diverse range of malignancies compared to normal tissues. Moreover, its expression could predict diagnosis and prognosis in most cancers. The enrichment analysis showed that TRIM54 and its related proteins were closely associated with ubiquitination modification and tumor-associated pathways. In addition, TRIM54 was tightly associated with ICI and immunotherapy. Finally, we screened out several targeted drugs that could regulate TRIM54 expression during immunotherapeutic responses.
**Conclusion**
TRIM54 plays a crucial part in pan-cancer development, and its aberrant expression in tumors may be associated with ICI in TME. At the same time, TRIM54 has an excellent prognostic, predictive ability and is also a promising predictive biomarker for immunotherapy.

### EACR2024-0037
### TTC21A: a novel prognostic biomarker associated with immune cell infiltration in hepatocellular carcinoma

*X.D. Yu[1], R.J. Liu[1], K. Wu[2], S.S. Yan[1], X.B. Zao[1], Y.S. Zhang[1]*
*[1]Beijing University of Chinese Medicine, Dongzhimen Hospital, Beijing, China*
*[2]University of Iowa, Neurology, Iowa State, United States*

**Introduction**
Hepatocellular carcinoma (HCC) is one of the most malignant tumors in the world. TTC21A gene is rarely reported in cancer. The aim of this study was to investigate the role of TTC21A in HCC.
**Material and Methods**
In this study, we conducted a comprehensive bio-informatics analysis of the correlation between TTC21A and HCC based on TCGA and HPA databases. The Kaplan-Meier platform was used to assess the impact of TTC21A expression on the survival of HCC patients. In addition, we used the TIMER and TISIDB platforms to analyze the correlation between TTC21A expression and HCC immune infiltrates.
**Results and Discussions**
The expression of TTC21A mRNA in HCC tissues was significantly higher than in paired normal tissues (P<0.0001). The expression of TTC21A protein was consistent with its mRNA expression. In addition, TTC21A expression had an excellent diagnostic efficacy for HCC (AUC=0.778). High expression of TTC21A predicted poor prognosis (P=0.041). In immune cell infiltration (ICI), TTC21A expression was significantly positively correlated with the degree of infiltration of multiple immune cells in HCC. In addition, we also found that TTC21A expression was positively correlated with most immune stimulators and immunosuppressants in HCC, including well-known T cell checkpoints such as PD-1, PD-L1, and CTLA-4.
**Conclusion**
This study demonstrated that TTC21A was upregulated in HCC liver tissues. It also indicates a poor prognosis for HCC patients. Significantly, TTC21A may partly affect prognosis by modulating ICI levels in HCC patients. TTC21A may be a potential biomarker and therapeutic target for evaluating prognostic value and regulating ICI levels.

### EACR2024-0041
### Upregulation of CHAC1 promotes bladder inflammation and predicts poor prognosis in Bladder Urothelial Carcinoma

*X.D. Yu[1], R.J. Liu[1], W.F. Gao[1], S.S. Yan[1], Y.S. Zhang[1]*
*[1]Beijing University of Chinese Medicine, Dongzhimen Hospital, Beijing, China*

**Introduction**
CHAC1 is a newly discovered ER inducible gene aberrantly expressed in various cancers. However, the underlying mechanisms of CHAC1 in bladder urothelial carcinoma (BLCA) remain largely unknown.
**Material and Methods**
All original data of CHAC1 was collected from the public databases, including The Cancer Genome Atlas Human Protein Atlas, and further analyzed by R (version 3.6.3). The correlations between CHAC1 and tumour immune characteristics were analyzed via the TIMER and TISIDB databases.
**Results and Discussions**
The results showed that CHAC1 mRNA and protein expression levels in BLCA tissues were higher than in normal bladder tissues. CHAC1 is up-regulated in BLCA tissues, which has clinical diagnostic value and is associated with poor prognosis. CHAC1 mRNA expression level was negatively correlated with CHAC1

© 2024 The Authors. Molecular Oncology is published by John Wiley & Sons Ltd on behalf of Federation of European Biochemical Societies methylation level. This study showed that CHAC1 could affect BLCA development by regulating glutathione metabolism and ferroptosis signalling pathways. Further analysis revealed that CHAC1 expression positively correlated with the infiltration of multiple immune cells. In addition, CHAC1 expression in BLCA is also closely related to immunomodulators and methylated immunomodulators.

**Conclusion**

The high sensitivity of CHAC1 makes it possible to be used as a diagnostic biomarker for BLCA. In addition, high expression of CHAC1 predicts poor prognosis in BLCA patients. In addition, the upregulation of CHAC1 was able to promote the infiltration of T cells, B cells, macrophages, and DC cells in BLCA. CHAC1 is an essential target gene in the malignant progression of BLCA and is associated with immune checkpoints. Therefore, CHAC1 may be a potential biomarker and a new immunotherapy target for BLCA.

### EACR2024-0150
### scMitoMut: an R Package for calling single-cell lineage informative mitochondrial mutation

*W. Sun*[1], *D. Ginniken*[1], *L. Perie*[1]
[1]*Institut Curie, Physics of cells and cancer UMR168, Paris, France*

**Introduction**

The acquisition of lineage tracing markers is pivotal for the exploration of development, tumors, and stem cell biology. Within scATACSeq or single cell multi-omics sequencing, mitochondria DNA is enriched due to their histone-free nature with somatic mutations in mitochondria acting as innate lineage tracers. Nonetheless, the precise identification of these markers is impeded with computational hurdles, as the existing mutation calling tools are ill-equipped for the polyploid nature of the mitochondrial genome or lack a robust statistical framework.

**Material and Methods**

To deal with these obstacles, we introduce scMitoMut, an innovative R package that leverages statistical methodologies, including the beta-binomial model, to accurately identify mitochondrial mutations at the single-cell level. scMitoMut assigns a p-value to mutations at each locus within individual cells, ensuring unparalleled sensitivity and precision in comparison to current methodologies. We tested scMitoMut using colorectal cancer, Erythroleukemic cell line (TF1 cell line), and peripheral blood mononuclear cells (PBMCs) datasets.

**Results and Discussions**

The results show that scMitoMut detected more mitochondrial mutations in the colorectal cancer dataset. Cells with these mutations are concentrated in a specific lineage. In the TF1 cell line results, the clusters of cells with detected mitochondrial mutations are consistent with clusters obtained from open chromatin characteristics. Importantly, scMitoMut detected lineage informative mutations both in PBMC datasets of libraries with or without mitochondrial enrichment. Using these mutations for clustering can distinguish the differentiation sequence of B, T, and Mono during development.

**Conclusion**

Consequently, scMitoMut significantly advances the application of scATACSeq or single cell multi-omics sequencing, facilitating the precise delineation of mitochondrial mutations for lineage tracing purposes.

### EACR2024-0179
### Smoking related gene expression in former smokers with lung adenocarcinoma discerns biological features and clinical outcomes

*P. Wang*[1], *R. Ng*[2], *S. Lam*[1], *W. Lockwood*[3]
[1]*British Columbia Cancer Research Institute, Integrative Oncology, Vancouver, Canada*
[2]*University of British Columbia, Computer Science, Vancouver, Canada*
[3]*British Columbia Cancer Research Institute, Pathology and Laboratory Medicine, Vancouver, Canada*

**Introduction**

An increasing proportion of lung cancers are found in patients who have stopped smoking. Here, we aimed to determine whether diversity in smoking induced gene expression across formerly smoking patients with lung adenocarcinoma (LUAD) corresponds to different biological and clinical outcomes to potentially influence early detection and personalized treatment for patients.

**Material and Methods**

LUAD expression profiles from patients who had never smoked and those who were currently smoking at the time of surgery in TCGA and British Columbia Cancer Agency cohorts were used to derive a gene signature associated with active tobacco smoking. This was utilized to create a random forest model (RF) as a classifier for smoking status. The RF was subsequently applied to 299 formerly smoking patients from the TCGA cohort, who were classified as either having a currently smoking (CS-like) or a never smoking (NS-like) gene expression profile and then correlated with patient demographics, biological traits, and clinical outcomes.

**Results and Discussions**

The derived 123 gene signature robustly classified those who had never smoked and those who currently smoke in a TCGA holdout test set (AUC=0.85). The RF classified 213 formerly smoking patients as CS-like and 86 as NS-like from the TCGA cohort. CS-like and NS-like formerly smoking patients were poorly correlated with patient demographics but had significantly different biological features including tumor mutational burden (TMB), mutational signatures, and immune cell populations. Overall survival outcome could also be clearly distinguished through the RF categorization of formerly smoking patients; NS-like patients had 17.5 months longer overall survival than CS-like patients.

**Conclusion**

LUAD in patients who have stopped smoking are a diverse population that can be stratified through smoking related gene expression. This classification was able to separate several variables in a meaningful manner, including clinical outcome and TMB, which can inform treatment selection and prognosis.

### EACR2024-0254
### Oncoexporter: Conversion of NCI CRDC

© 2024The Authors. Molecular Oncology is published by John Wiley & Sons Ltd on behalf of Federation of European Biochemical Societies

### Data to the GA4GH Phenopacket Schema

D. Meerzaman[1], M. Sierk[2], R. Reese[3], P. Robinson[4], Q. Chen[5], C. Yan[1], D. Danis[6], R. Mondal[7], N. Fernandes[8], A. Jha[9]
[1]NIH/NCI, CBIIT, Rockville, United States
[2]NCI/NIH, Cbiit, Rockville, United States
[3]Lawrence Berkeley National Laboratory, Environmental Genomics and Systems Biology, Berkeley, United States
[4]Berlin Institute of Health at Charité, computational Biology, Berlin, Germany
[5]NIH/NCI, cbitt, Rockville, United States
[6]Jackson Lab, Genomics and Computational Biology, Farmington, United States
[7]Elucidata, Data Science, Kolkata, India
[8]Elucidata, Data science, Bengaluru, India
[9]Elucidata, Data science, San Francisco, United States

**Introduction**

Cancer data is represented using heterogeneous terminologies and data schemas, which makes it difficult to exchange data and often requires each individual dataset to be prepared for analysis using bespoke scripts or tools. The Global Alliance for Genomics and Health (GA4GH) Phenopacket schema was developed in the rare disease field as a common framework for capturing phenotypic and genotypic data in a consistent but flexible manner amenable to downstream machine learning applications. A Phenopacket characterizes an individual person or biosample, linking that individual to detailed phenotypic descriptions, genetic information, diagnoses, and treatments. The Cancer Research Data Commons (CRDC) is a cloud-based infrastructure providing public and controlled-access to multiple large-scale cancer data sets that can be analyzed without download using the NCI Cloud Resources. Converting data commons data into the Phenopackets schema will facilitate data integration efforts both within the CRDC and between the CRDC and external data sources. Here we illustrate the application of the Phenopacket standard to CRDC and demonstrating this conversion and the subsequent application to a simple clinical analysis.

**Material and Methods**

We used the Cancer Data Aggregator python library, which uses data commons APIs to aggregate data from bespoke queries into dataframes, to extract cohorts from CRDC data. We then used the Phenopackets python library to produce a phenopacket for each subject. We created python classes that take the CDA records for individuals, biosamples, diseases, medical actions, and mutations and map them to the proper Phenopacket data elements.

**Results and Discussions**

The code is freely available on Github under an MIT license (github.com/monarch-initiative/oncoexporter). The modular code can be extended for use with other data sources provided the appropriate ETL (extract, transform, load) code. Additionally, we developed python code for ingesting phenopackets and performing basic QC/QA analysis and visualization, as well as Cox regression and survival analysis.

**Conclusion**

The CRDC data can be converted into the Phenopacket schema and fed into downstream analysis tasks facilitating integration and analysis of both data within the CRDC and external data. This integration has the potential to unlock critical insights into cancer development, progression, and treatment hidden in the massive reservoirs of heterogeneous cancer datasets worldwide.

### EACR2024-0288
### Integrative transcriptomic and DNA methylation analysis of small cell lung cancer patient-derived models reveals a subgroup defined by high RET kinase with potential for targeted therapy.

Y. Chen[1,2,3], K. Brennan[1,3], M. Revel[1,2], S. Pearce[1,2,3], M. Galvin[1,2], M. Carter[1,4], L. Burks[1,2,5], F. Blackhall[4], K. Simpson[1,2,5], C. Dive[1,2,5]
[1]Cancer Research UK, National Biomarker Centre, Manchester, United Kingdom
[2]Cancer Research UK, Lung Cancer Centre of Excellence, Manchester, United Kingdom
[3]Bioinformatics and Biostatistic team, CRUK National Biomarker Centre, Manchester, United Kingdom
[4]The Christie NHS Foundation Trust, Medical Oncology, Manchester, United Kingdom
[5]SCLC Biology Group, CRUK National Biomarker Centre, Manchester, United Kingdom

**Introduction**

Small cell lung cancer (SCLC) is an aggressive neuroendocrine tumour. Platinum-based chemotherapy is standard-of-care but rapid chemoresistance follows initial response. Immunotherapy brings some durable responses in an unselected minority of patients but overall, patient outcomes remain poor. The SCLC genomic landscape includes almost universal inactivation of TP53 and RB1 tumour suppressors, commonly elevated MYC family members and mis-regulation of NOTCH signalling, challenging territory for drug development. Characterisation of transcriptional (ASCL1, NEUROD1, ATOH1, POU2F3) and inflamed subtypes heralds potential for stratified therapy based on subtype vulnerabilities in preclinical models, but this is as-yet untested in the clinic. We developed a biobank of SCLC CDX models derived from patients' circulating tumour cells that recapitulate the donors' disease and clinical inter- and intra-tumoural heterogeneity. Here we undertook a multi-omic analysis of 56 CDX models searching for druggable targets.

**Material and Methods**

Whole exome sequencing (WES), RNA-sequencing (RNA-Seq) and methyl-CpG-binding protein sequencing (MBD-seq) was performed. Gene expression and DNA methylation data were integrated using similarity network fusion (SNF) and multi-omics factor analysis (MOFA). Differential gene expression and methylation analyses were examined between distinct SCLC subgroups identified via unsupervised clustering.

**Results and Discussions**

Integrative transcriptomic analysis and DNA methylation analysis revealed a distinct CDX subgroup exhibiting high expression and abnormal methylation of RET, that encodes a druggable kinase, independent of molecular subtype in 66% (37/56) of the CDX models. While activating RET mutation and rearrangements are known

Molecular Oncology 18 (Suppl. 1) (2024) 1–495
© 2024 The Authors. Molecular Oncology is published by John Wiley & Sons Ltd on behalf of Federation of European Biochemical Societies oncogenic drivers in other cancers, WES analysis revealed that this CDX subgroup expresses wild-type RET. Correlated gene and protein expression was confirmed by immunohistochemistry. In silico analysis of prior bulk and/or single cell data from SCLC cell lines and primary tumours confirmed subgroups with elevated RET expression compared to normal lung.

**Conclusion**

Wild-type RET is highly expressed in a distinct subset of SCLC CDX models. Ongoing studies are evaluating RET kinase signalling activity and response to small molecule RET inhibitors as a potential therapeutic strategy for patients with high expression of RET.

### EACR2024-0319

### An integrative multi-omics approach for the comprehensive characterization of liver cancer

*M. Villa*[1], *F. Malighetti*[1], *V. Crippa*[1], *L. Mologni*[1], *D. Ramazzotti*[1]

[1]*University of Milano-Bicocca, School of Medicine and Surgery, Monza, Italy*

**Introduction**

Liver cancer represents an ongoing global health issue, projected to surpass one million cases by 2025. Hepato-cellular carcinoma (HCC) stands as the predominant subtype. Despite significant advancements, there remains a need for additional biomarkers to improve precision medicine and enhance overall survival.

**Material and Methods**

This study exploits extensive omics datasets to profile HCC. We conducted an in-depth analysis of multi-omics data from The Cancer Genome Atlas to identify molecular subtypes showing significant prognostic differences. Specifically, we performed integrative clustering on 179 HCC samples with comprehensive multi-omics data. Furthermore, we associated mutations with differential expression by regularized regression.

**Results and Discussions**

The analysis revealed three distinct clusters (C1 to C3) based on multi-omics features. Comparing these clusters uncovered molecular differences linked to survival outcomes. To further explore multi-omics features specifically linked to prognosis, we conducted both univariate and multivariate regularized analyses, and enrichment using the tools DAVID, GSEA, and miRPath. Finally, we reviewed the literature to determine if the obtained results correlated with the genes function, specifically examining whether increased expression was associated with an oncogenic function. Employing the selected features from these analyses, we were able to stratify patients within the dataset into survival-differentiating groups. The analysis identified "GO BP_Stem cell differentiation" (GO:0048863) as the most interesting annotation associated with cancer prognosis, encapsulating the most robust features. Within this ontology, seven features consistently emerged as prognostically significant, including PITX2, hsa-miR-105-5p, hsa-miR-18a-3p, hsa-miR-550a-3p (positively associated with risk), and ESR1|ERα (negatively associated with risk). At the genomic level, mutations were the most prominent alterations across clusters. We confirmed the correlation between mutated genes, notably TP53 and ARID1A, and the differential expression of the features selected by our analysis. These two genes were also associated with dismal disease outcomes, confirming the selected multi-omics features as prognostic biomarkers.

**Conclusion**

This study highlights the potential of translating comprehensive multi-omics computational analyses into translational approaches for understanding the molecular mechanisms of tumors evolution and identifying prognostic markers.

### EACR2024-0328

### A new method for cell-line and sample authentication using ultra low-pass whole genome sequencing

*A. Ferrarini*[1], *C. Forcato*[1], *V. del Monaco*[1], *M. Garonzi*[1], *M. Pellicano*[1], *G. Buson*[1], *F. Fontana*[1], *P. Tononi*[1], *N. Manaresi*[1]

[1]*Menarini Silicon Biosystems S.p.a., Research and Development, Castel Maggiore BO, Italy*

**Introduction**

Cell line authentication helps guarantee reproducibility of scientific results and is required for publication. The gold standard method, Short Tandem Repeat (STR), suffers from several drawbacks in the framework of oncology research, such as: impairment by microsatellite instability, inherent in certain cell lines used in immune-checkpoint inhibitors studies; the lack of information on genomic drifts due to cell passage; requirement of capillary electrophoresis equipment. Here we present a method which enables cell line authentication with minimal amounts of input material, resilient to DNA degradation, providing information on both cell line identity and its genomic profile using NGS.

**Material and Methods**

We sequenced NGS libraries prepared using whole genome amplification with ligation-mediated-PCR (LM-PCR; Ampli1™ WGA kit) and library preparation using Ampli1™ LowPass from 12 DNA samples from 6 cancer cell lines, and 40 single-cells isolated from Formalin Fixed Paraffin Embedded (FFPE) samples from 4 patients. We compared the allelic content among sample pairs over a large number of highly heterozygous SNP loci (>200.000) producing a measure of similarity. The same data provides information on the DNA/cell-line copy-number alteration profile.

**Results and Discussions**

Reduced-representation of the genome from LM-PCR, enhanced by the fragmentation-free library preparation with Ampli1 LowPass, boosts the number of overlapping genomic regions available for the comparison of allelic content among the paired samples under analysis. Thus, even for an ultra-low coverage < 0.02x , the similarity score obtained by the comparison of matching samples was on average 0.64, with no overlap with that of unrelated samples (similarity < 0.18). FFPE single-cells from the same or different patients were distinguished using the same method, demonstrating applicability down to single-cells amounts of DNA. Copy-number alteration profiles were obtained from the same sequencing data, providing a control on genomic drift.

**Conclusion**

© 2024 The Authors. Molecular Oncology is published by John Wiley & Sons Ltd on behalf of Federation of European Biochemical Societies

This new method enables the authentication of cell lines and samples for oncology research and biobanking, using widely available NGS sequencers and simple bio-informatic pipelines without the need of special-purpose equipment. The method is robust to DNA degradation and low inputs, providing convenience of use and cost reductions compared to STR analysis.

*AF, CF, VdM, MG, MP, GB, FF, PT, NM are employees of Menarini Silicon Biosystems Spa.. AF, CF, NM are inventors on a patent based on the method described.*

### EACR2024-0360
### Chromosomal instability signatures in stage I epithelial ovarian cancer highlight different genomic instability patterns

*L. Beltrame[1], L. Mannarino[1,2], A. Velle[3], R. Zadro[2], R. Fruscio[4], D. Lo Russo[2,5], F. Borella[6], M. D'Incalci[1,2], C. Romualdi[3], S. Marchini[1]*

*[1]IRCCS Humanitas Research Hospital, Laboratory of Cancer Pharmacology, Rozzano, Italy*

*[2]Humanitas University, Department of Biomedical Sciences, Pieve Emanuele, Italy*

*[3]University of Padova, Department of Biology, Padova, Italy*

*[4]Università degli Studi Milano-Bicocca- Fondazione IRCCS San Gerardo dei Tintori, Department of Obstetrics and Gynaecology, Monza, Italy*

*[5]Humanitas San Pio X, Gynecologic Oncology Unit, Milano, Italy*

*[6]San Anna Hospital- University of Torino, Gynecology and Obstetrics 1- Department of Surgical Sciences, Torino, Italy*

**Introduction**

The prognosis of stage I epithelial ovarian cancer (EOC), that is the tumor confined to one or both ovaries, is more favorable than that of stage III-IV. Treatment involves staging procedure, and platinum-based chemotherapy can be withheld according to ICON1 parameters. Unfortunately, 20-30% of patients relapse and ultimately die. The identification of biomarkers able to predict the risk of relapse better than current classifiers is a currently unmet clinical need. We have previously identified three copy number alteration patterns (stable, S; unstable, U; highly unstable, HU), with prognostic value in stage I EOC (Pesenti et al., 2022). However, these patterns recapitulate multiple alterations leading to the same phenotype, thus the underlying molecular causes are unclear. Taking advantage of the development of a compendium of pan-cancer chromosomal instability signatures (Drews et al., 2022), we have built upon our past results to elucidate the potential mechanisms behind the three patterns and their relationship to clinical parameters.

**Material and Methods**

Copy number features were extracted from shallow whole sequencing data from a cohort of 385 Stage I EOC biopsies. Linear combination decomposition was used to associate each sample to one of the previously published 17 signatures (Drews et al., 2022).

**Results and Discussions**

Hierarchical clustering identified six clusters of samples with differing patterns of signature activity. Cluster 1 was enriched in low grade tumors, included only U samples and was characterized by high activity of CX1, a signature related to chromosomal segregation defects due to defective mitosis and / or telomere dysfunction; Cluster 2 included samples with no detectable signatures (S samples and U samples with less than 20 CNAs); the other four clusters (3-6) were enriched in high grade tumors and HU samples, with different patterns of activity of signatures CX1, CX3 (Impairment of homologous recombination, IHR, replication stress and DNA damage sensing defects), CX2 (IHR), and CX5 (IHR and replication stress).

**Conclusion**

These results suggest that Stage I EOC tumors with the U phenotype are for the vast majority characterized by a single etiopathogenetic mechanism, shown by the activity of signature CX1. Samples with the HU phenotype exhibited instead a number of different mechanisms underlying the same phenotype. These differences could be exploited to identify better predictors of the risk of relapse.

### EACR2024-0384
### Chemotherapy induces myeloid-driven spatial T-cell exhaustion in high-grade serous ovarian cancer

*A. Farkkila[1], I.M. Launonen[2], E. Pekcan Erkan[3], I. Niemiec[2], M. Hincapie-Otero[2], A. Virtanen[4], S. Hautaniemi[2], P. Sorger[5], A. Vähärautio[2]*

*[1]University of Helsinki, Research Programs Unit- Oncology and Gynecology, Helsinki, Finland*

*[2]University of Helsinki, Research Program in Systems Oncology, Helsinki, Finland*

*[3]University of Helsinki, Resarch Programs Unit, Helsinki, Finland*

*[4]University of Helsinki- Helsinki University Hospital, Research Program in Systems Oncology- Department of Pathology, Helsinki, Finland*

*[5]Harvard Medical School, Laboratory of Systems Pharmacology, Boston, United States*

**Introduction**

To enhance the efficacy of immunotherapeutic treatment strategies for ovarian cancer, it is essential to attain a comprehensive understanding of the intricate spatio-temporal dynamics within the tumor-immune-stromal microenvironment.

**Material and Methods**

We performed integrative molecular and single-cell spatial characterization of the tumor-immune microenvironment of a total of 97 HGSC samples collected before and after neoadjuvant chemotherapy (NACT) using genomics, transcriptomics, single-cell RNA sequencing (scRNA-seq), tissue cyclic immuno-fluorescence highly-multiplexed imaging (t-CycIF) and spatial transcriptomics. The multimodal features were interrogated in patient-matched samples.

**Results and Discussions**

Using single-cell spatial analyses, we identified unique cellular microcommunities, associated with functionally relevant cell states and spatiotemporal changes in the immunoarchitecture induced by chemotherapy. Importantly, chemotherapy-induced distinct peritumoral patterns of communication between CD8+ T-cells and macrophages, specifically in the cellular micro-

Molecular Oncology 18 (Suppl. 1) (2024) 1–495
© 2024 The Authors. Molecular Oncology is published by John Wiley & Sons Ltd on behalf of Federation of European Biochemical Societies communities localizing to the tumor-stromal niches. Myeloid cells formed functionally important networks termed "Myelonets", contributing to T-cell state dynamics. Spatial transcriptomics of CD8+T-cell Myeloid interactions revealed distinct transcriptional programs and inherently spatial multicellular dynamics in response to chemotherapy. Further dissection of the underlying programs of the cell-cell interactions identified prominent TIGIT-NECTIN2 ligand-receptor pair interaction induced by chemotherapy. Using a patient-derived functional immuno-oncology platform, we show enhanced CD8+T cell activity by combining immune checkpoint blockade with chemotherapy.

**Conclusion**

Our study exploits multiomics data incorporation at an unprecedented single-cell and spatial scale uncovering the dynamic changes in the TME cell subpopulations, cell states, spatial patterns and underlying molecular programs occurring in response to platinum-based chemotherapy. We demonstrate myeloid-driven spatial T-cell exhaustion that can be functionally targeted thus opening new immunotherapeutic opportunities and rationale for patient stratification.

### EACR2024-0421

### Introducing CancerHubs: A Systematic Data Mining and Elaboration Approach for Identifying Novel Cancer-Related Protein Interaction Hubs

*N. Manfrini[1], I. Ferrari[1], G. Lai[1], F. De Grossi[1], S. Oliveto[1], S. Biffo[1]*

*[1]Fondazione Istituto Nazionale di Genetica Molecolare - INGM, Molecular Histology and Cell Growth Unit, Milan, Italy*

**Introduction**

Discovering the players and understanding the molecular mechanisms involved in cancer initiation and progression is fundamental for developing effective diagnostic tools and targeted therapies. Traditional approaches for predicting protein involvement in cancer have mostly relied on genomics or transcriptomics, often overlooking the complex interplay between genetic mutations, gene expression, and clinical outcomes.

**Material and Methods**

Here, by crossing publicly available mutational datasets with clinical outcome prediction and interactomic data, we constructed an R pipeline capable to define novel protein hubs predicted to play important roles in cancer. By adopting this comprehensive strategy, which we defined ‘CancerHubs’, we were able to rank genes according to a newly introduced metric termed the 'network score'. The network score predicts the level of involvement of a certain gene in a particular cancer by defining the number of mutated interactors that its encoded protein has.

**Results and Discussions**

Taking advantage of the CancerHub*s* approach, we identified several novel broad-cancer and cancer-specific genes. Among these we validated TGOLN2, a protein with broad tumor suppressor functions, and EFTUD2, a protein with oncogenic properties in Multiple Myeloma. Our findings underline the importance of considering diverse molecular data types and network-level interactions in order to fully unravel the complexity of cancer biology and pinpoint novel potential therapeutic targets.

**Conclusion**

CancerHubs introduces a pioneering method for forecasting gene involvement in cancer. By ranking cancer-associated genes based on the number of mutant interactors their encoded proteins have, this approach identifies protein hubs potentially involved in cancer. This methodology globally improves the overall detection of cancer-associated genes, as demonstrated by its ability to accurately predict protein hubs previously never found related to cancer.

### EACR2024-0472

### Development of a molecular classification of neuroendocrine tumors of different origins through multi-omic integration models. the new neuroendocrine subtypes and their clinical relevance

*C. Carretero[1,2], B. Anton[1,3], A. Lens-Pardo[1,2], M. Benavent[4], A. Teijo[5], Y. Rodriguez[5], G. Gómez-Lopez[6], F. Al-Shahour[6], B. Soldevilla[1,2], R. García-Carbonero[1,2,3]*

*[1]Instituto de Investigación Sanitaria Hospital 12 de Octubre imas12, Grupo de Investigación en Tumores Gastrointestinales y Neuroendocrinos, Madrid, Spain*

*[2]CNIO, Grupo de Investigación en Tumores Gastrointestinales y Neuroendocrinos, Madrid, Spain*

*[3]Hospital Universitario Doce de Octubre, Medical Oncology Department, Madrid, Spain*

*[4]Hospital Universitario Virgen del Rocío- IBIS, Medical Oncology Department, Sevilla, Spain*

*[5]Hospital Universitario Doce de Octubre, Pathology Department, Madrid, Spain*

*[6]CNIO, Bioinformatic Unit, Madrid, Spain*

**Introduction**

Neuroendocrine tumors (NETs) are a group of tumors with a wide anatomical distribution. Nevertheless, NETs share common clinical and molecular features. They have a low mutational burden and few recurrent mutations, with epigenetics playing a notable role. However, the molecular bases of the disease are not deeply understood, and published studies are based on tumors with a specific primary location.

**Material and Methods**

The main goal is to develop a molecular classification of NETs independent of their origin, aiming to facilitate the development of new diagnostic, prognostic, and predictive biomarkers. Transcriptomic and methylomic profiles were generated from paraffin-embedded tumor samples of 194 patients diagnosed NETs from various primary locations. A Multi-Omics Factor Analysis (MOFA) model consisting of 8 factors was constructed using both omics datasets to apply unsupervised clustering algorithms. Differences in discrete variables among subtypes were evaluated by Fisher's test, and overall survival was analyzed using Kaplan-Meier and Cox regression models. Immune cell populations were deconvolution techniques. Differential expressed genes were studied and Gene Set Enrichment Analysis (GSEA) was performed.

© 2024 The Authors. Molecular Oncology is published by John Wiley & Sons Ltd on behalf of Federation of European Biochemical Societies

**Results and Discussions**
An 8-factor MOFA model was generated to applay a molecular classification that identified three neuro-endocrine subtypes (NSs) significantly associated with patient prognosis. NS1 demonstrated the best prognosis characterized by enrichment in diverse immune populations and heightened expression of genes implicated in metabolism and nutrient digestion. Conversely, NS3, with the worst prognosis, showed elevated levels of methylation and copy number variations (CNVs), along with associations with clinically more aggressive features (G2-3, stage IV at diagnosis, higher 5-HIAA levels). Notably, NS3 also exhibited higher expression of genes associated with neuroendocrine activity, including members of the chromogranin family and others also linked to cell proliferation such as PTPRN, BEX1, and NOVA1. NETs with intermediate prognosis, NS2, presented a mixed molecular profile.
**Conclusion**
This molecular classification reveals common sources of biological variability across distinct primary tumor origins that classify samples into three prognostic neuroendocrine subtypes associated with differential clinical and molecular characteristics. Additionally, this study may facilitate the identification of universal prognostic biomarkers and therapeutic targets for NETs of various tissue origins.

### EACR2024-0518
### High resolution phylogenetic analysis and timing in cancer through whole genome methylation

*I. Leshchiner[1,2], N. Guteneva[1], E. Lichter[1]*
*[1]Boston University, Computational Biomedicine, Boston, United States*
*[2]Broad Institute of MIT and Harvard, Cancer Program, Cambridge, United States*
**Introduction**
Learning the genetic evolutionary paths tumors take to become cancer through sequential acquisition of somatic alterations from the normal to malignant state is essential for understanding tumorigenesis biology, identifying targets for early cancer detection and prevention studies. Presently, the determination of cancer progression histories is done by two approaches: the direct sequencing of premalignant lesions, which are rarely available for most tumor types, and computational methods that perform "timing" - infer the sequence of events based on primary tumor somatic mutational clocks. Timing methods had allowed to explore pre-malignant progression of multiple cancer types for the first time, but still offer limited resolution on a single patient level due to the comparatively low burden of somatic mutations.
**Material and Methods**
We focused on increasing timing resolution through the use of much more prevalent genome methylation changes. Over 28 million methylated CpG sites exist in the genome with several percent of them differentially methylated between tissue types and tumor vs adjacent normal tissue. Much higher density of distinctly methylated sites provides unprecedented resolution, needs fewer tumors per cancer type, can be merged with driver mutation data and also allows to co-analyze "methylation clock" information that allows to establish early development, phylogeny and progression at resolution not available previously.
**Results and Discussions**
We developed a pipeline and methods based on our earlier tool, Phylogic, that perform high resolution timing and phylogeny analysis with the use of whole genome bisulfite sequencing (WGBS), reduced representation bisulfite sequencing (RRBS) and Oxford Nanopore Technologies native methylation sequencing data. Somatic methylation sites have distinct properties from somatic point mutations, can appear and disappear in the process of tumor evolution and development and require distinct modeling algorithms compared to mutational timing.
**Conclusion**
Our tools for the first time perform single sample and cohort level methylation "timing", multisample phylogeny and subclonal populations reconstruction by integrating methylation and mutational data from the same sample. Our methods will allow researchers to define progression histories of tumors at a resolution not available previously.

### EACR2024-0535
### Integrative circRNA profiling from RNA-sequencing of colorectal cancer and adenoma tissues shows a down-regulation in early stages of the disease

*A. Camandona[1], A. Gagliardi[2,3], N. Licheri[1], D. Festa[2], S. Tarallo[2,3], S. Cutrupi[1], M. De Bortoli[1], B. Pardini[2,3], A. Naccarati[2,3], G. Ferrero[1]*
*[1]University of Torino, Department of Clinical and Biological Sciences, Turin, Italy*
*[2]IRCCS - Candiolo, Italian Institute for Genomic Medicine, Candiolo- Turin, Italy*
*[3]FPO-IRCCS, Candiolo Cancer Institute, Candiolo-Turin, Italy*
**Introduction**
Detection of Colorectal Cancer (CRC) at an early stage significantly improves its management, response to therapy, and overall patient survival. Circular RNAs (circRNAs) are peculiar covalently closed transcripts involved in the modulation of gene expression whose dysregulation has been extensively reported in cancer cells. However, little is known on the circRNA alterations occurring in the early stages of colorectal carcinogenesis. A concomitant deep circRNA characterization in tumor tissue samples and precancerous lesions may provide novel evidence on the alterations of these molecules in the CRC onset.
**Material and Methods**
Total RNA-Seq data of lesions and matched adjacent mucosa of 96 patients with CRC and 29 with colorectal adenomas were analyzed to explore the circRNA expression profiles. CIRI2 and DESeq2 tools were applied respectively for circRNA detection and paired differential expression analysis. Integration with the expression of host genes and interacting RNA binding proteins (RBPs) was performed to further characterize

© 2024 The Authors. Molecular Oncology is published by John Wiley & Sons Ltd on behalf of Federation of European Biochemical Societies the identified circRNAs. Altered circRNA levels were explored with respect to patients' clinical data and other omics information, including miRNome from small RNA-Seq and mutational profiles from target DNA sequencing.

**Results and Discussions**

A widespread circRNA downregulation was observed in advanced adenomas and early CRC stages. Specifically, out of 34 identified dysregulated circRNAs, 33 were downregulated in tumor tissue. Profiling of advanced adenoma tissue reflected this trend. Observed down-regulation was coherent with the circRNA host-gene repression and the downregulation of RBPs involved in the circRNA biogenesis, such as *NOVA1*, *RBMS3*, and *CIRBP*. Functional analysis predicted several interactions between circRNAs, miRNAs, and RBPs supported by significant correlations between their expression levels. Moreover, analysis of cancer hallmark-related pathways showed significant associations with circRNA levels. According with a circRNA downregulation at early CRC stage, the predicted activity of cell proliferation-related pathways, DNA repair and c-Myc signaling was higher in samples with low DE circRNA expression.

**Conclusion**

These data support that multiple molecular alterations occur in concomitance with a widespread circRNA downregulation, which can represent an early molecular perturbation in colorectal carcinogenesis. Investigating specific circRNA levels could provide a candidate signature for an early CRC diagnosis.

### EACR2024-0550
### Harnessing Integrative Bioinformatic Analysis to Unveil Crucial Target Genes Associated with Breast Cancer

F.B. Berber[1,2], G. Turan[2,3], G. Korkmaz[2,4]
[1]*Koç University, Biomedical Sciences and Engineering, İstanbul, Turkiye*
[2]*KUTTAM, Research Center for Translational Medicine, İstanbul, Turkiye*
[3]*Koç University, Graduate School of Health Sciences, İstanbul, Turkiye*
[4]*Koç University, School of Medicine, İstanbul, Turkiye*

**Introduction**

Breast cancer, a leading cause of cancer-related mortality in women, presents substantial therapeutic challenges due to its heterogeneity. Luminal breast cancer, characterized by estrogen receptor alpha (ERα) expression, represents a significant proportion of cases. Crucial transcription factors, including ERα, FOXA1, and GATA3, play pivotal roles in gene regulation and influence tumor progression. Copy number variations (CNVs) emerge as key drivers in breast cancer, impacting gene expression. Our study integrates CNV data on ERα and GATA3 binding sites and gene expression in luminal breast cancer. Leveraging public data sources, we aim to elucidate the molecular mechanisms underlying the disease.

**Material and Methods**

We analyzed three RNA-seq datasets for MCF10A, MCF12A, MCF7, and T47D cell lines and seven ChIP-seq datasets for ERα and GATA3 in MCF7 and T47D cell lines sourced from GEO. Additionally, we used a CNV dataset from CCLE. Differentially expressed genes are identified using DESeq2, MACS2, and HOMER to determine the transcription factors' peaks and binding motifs. Integration of RNA-seq, ChIP-seq, and CNV data identified shared genes. Functional enrichment analysis identified enriched pathways and hub genes were selected for constructing protein-protein interaction networks. Finally, clinical relevance was assessed through expression analysis using the TCGA dataset.

**Results and Discussions**

Our study revealed 2391 differentially expressed genes, with 54.7% showing copy number variations. Correlations between transcription factor binding and copy number alterations were established. Dataset integration identified 195 genes genome-wide. Through six protein-protein interaction analyses, 11 shared hub genes were identified. Analysis of hub gene expression in patient data using the TCGA dataset highlighted the clinical relevance of TFF3, KRT8, KRT19, KDR, and AGR2 genes, some already linked to breast cancer. To delve deeper into their function, we plan CRISPR-Cas9 knockout experiments, focusing on cellular proliferation and migration.

**Conclusion**

Our study investigates the intricate molecular landscape of luminal breast cancer, focusing on how CNVs and transcription factors influence gene activity. By analyzing RNA-seq, ChIP-seq, and CNV data, we uncovered effective genes and pathways linked to tumor progression. Notably, well-established hub genes emerged as clinically relevant. Future CRISPR-Cas9 experiments may pinpoint new target genes, potentially improving breast cancer treatments.

### EACR2024-0576
### Dynamic Analysis of Pancreatic Cancer Cells' Drug Resistance through Lentiviral Barcoding and Single-Cell RNA Sequencing

D. lucarelli[1], D. Anggraini Ngandiri[1], S. Baerthel[1], F. Theis[2], D. Saur[1]
[1]*Technical University of Munich, Institute of Translational Cancer Research and Experimental Cancer Therapy, Munich, Germany*
[2]*Helmholtz, Institute for Computational Biology, Munich, Germany*

**Introduction**

Understanding the heterogeneity of cancer cells' response to treatment is pivotal for developing more effective therapies. Many studies overlook the diverse response of different clones, limiting our understanding of treatment effectiveness. In this study, we barcoded Pancreatic cancer cell lines with the Lineage And RNA RecoverY (LARRY) lentiviral library. We then treated the cells with Trametinib and Nintedamib to follow the resistant cells over multiple time points, individuate their resistance drivers, and assess the interplay between clonality and plasticity.

**Material and Methods**

Cancer cells were transduced with the LARRY library to ensure each cell received a unique identifier. A subset of cells was picked post-transduction and separately

© 2024 The Authors. Molecular Oncology is published by John Wiley & Sons Ltd on behalf of Federation of European Biochemical Societies expanded in culture. DNA sequencing was performed to assess the barcodes each clone integrated. After the expansion and sequencing, cells from individual clones were pooled together at equal concentrations. Single cell RNA sequencing was performed on day 0, to split the cells into control and treated branches, and on days 3, 14, and 25 in both treated and control cells.

**Results and Discussions**

The analysis revealed two distinct subpopulations of cancer cells present before treatment initiation. One population associated with dormancy markers, while the other expressed markers associated with proliferation. Over time, the proliferating cell population outgrew the dormant one in the control branch, whereas in the treated branch, the opposite occurred. Using LARRY's barcodes, we identified specific clones that were associated with either the dormant or proliferating status. Interestingly, some clones displayed an overlap of both statuses, highlighting the interplay between plasticity and clonality in tumor cells.

**Conclusion**

Our experimental setup allowed us to select specific clones and monitor their dynamic evolution across different conditions. The prospect of implanting these barcoded clones into mouse models and performing single-cell and spatial transcriptomics in vivo provides the opportunity to gain deeper insights into how these clones behave within the host, how they differently influence and are influenced by their surrounding microenvironment, how they differently respond to treatment in vivo, and how their characteristics and interactions evolve. This approach can significantly advance our understanding of pancreatic cancer and the drivers of its immunosuppressive microenvironment contributing to the development of more effective therapeutic strategies.

### EACR2024-0606
### Dehydrogenase/Reductase 2 (DHRS2) is a new game changer in breast cancer cells

*B. Salman*[1,2], *H. Karaca*[1,2], *Ş. Punar*[1,2], *S. Sırma Ekmekci*[1], *N. Abacı*[1]
[1]*İstanbul University Aziz Sancar Institute of Experimental Medicine, Genetics, İstanbul, Turkiye*
[2]*İstanbul University Institute of Graduate Studies in Health Sciences, Genetics, İstanbul, Turkiye*

**Introduction**

While DHRS2 is found at basal levels in breast epithelium under normal conditions, it has been shown that amplification or deletions are found in cancerous cells. This study examined the possible effects of DHRS2 gene by transcriptomic approach, whose intracellular function has not been fully defined and which we think has a crucial role in cell homeostasis in breast cancer cells.

**Material and Methods**

After RNA isolation of DHRS2 gene expression altered cells (MCF10A, MCF7, T47D, and MDA MB 231) RNA quality was performed by Qubit3 and Bioanalyzer 2100, and samples with RIN score above 7 were studied. After this quality control step, Illumina Stranded mRNA Prep kit was used for library preparation. Samples indexed and barcoded according to the kit protocol were sequenced using the Illumina NovaSeq 6000 next-generation sequencing platform. Sequence data were analyzed on the Illumina DRAGEN Bio-IT and Galaxy platforms. The workflow was similar on both platforms, and the reference genome was chosen as the genome (Homo Sapiens GRCh38.p13). After gene expression values were obtained, gene ontology and gene set enrichment analyses were performed with the genes that emerged in comparisons between groups.

**Results and Discussions**

As a result of enrichment and ontology analysis, when DHRS2 gene expression increased in the cell, a decrease in the expression of genes involved in the cytoskeleton (GO:0005200) and signal transduction (GO:0005102) was observed. Again, cytokine and chemokine activity (GO:0006955) increased in luminal A subgroup breast cancer cells when DHRS2 gene expression was increased. In triple-negative cell lines, increased expression of genes related to transcription and TP53 regulation was observed. In the healthy cell line, catalytic activation was observed in parallel with the enzymatic activity of the DHRS2 gene.When DHRS2 gene expression was suppressed, receptor-ligand binding (GO:0048018), receptor-signal transduction (GO:0007165), kinase activity (GO:0016301), $Ca^{+2}$, and other ion binding processes (GO:0005509, GO:0043167) were the most affected.

**Conclusion**

DHRS2 gene expression was altered in MCF10A, MCF7, T47D, and MDA MB 231 cells. DHRS2, which has been shown to play a role in invasion, metastasis, cell division, ion balance, signal and transcription regulation, is thought to play an essential role in cancer development, we believe that this study can be a pioneer for the DHRS2 gene in cancer research.

### EACR2024-0654
### Predicting and Investigating Response to Immunotherapy Using Machine Learning on Immune Single-cell RNA-seq Data

*A. Pinhasi*[1], *K. Yizhak*[1,2]
[1]*Technion – Israel Institute of Technology, The Ruth and Bruce Rappaport Faculty of Medicine, Haifa, Israel*
[2]*Technion – Israel Institute of Technology, The Taub Faculty of Computer Science, Haifa, Israel*

**Introduction**

Understanding patient response to checkpoint blockade (CPB) therapy and building prognostic models are key challenges in cancer research. Single-cell RNA sequencing (scRNA-seq) provides rich insights into patients' immune profiles during treatment. However, the inherent complexity of this data and of the tumor micro-environment (TME), requires the development of more advanced analytical models. To this end, our study focuses on developing a machine learning model to predict therapy response and uncover contributing factors.

**Material and Methods**

We developed a machine learning predictive model using XGBoost, trained on scRNA-seq data from a cohort of melanoma patients treated with CPB. Focusing on different cell types, the model was trained on the cell-level gene expression, and then aggregated to get patient-

Molecular Oncology 18 (Suppl. 1) (2024) 1–495
© 2024 The Authors. Molecular Oncology is published by John Wiley & Sons Ltd on behalf of Federation of European Biochemical Societies level predictions. We utilized feature selection techniques, notably Boruta, and feature importance scores to get the most contributing genes to response. We further analyzed these genes with SHAP (SHapley Additive exPlanations) values, allowing to find complex gene behaviors and interactions. Further, to investigate the cell-level contribution to the immune response, we built a novel reinforcement-learning (RL) framework that labels the contribution of each cell in the dataset. Validation was conducted on several independent datasets spanning over multiple cancer types.

**Results and Discussions**

Our model achieves high AUC scores, ranging to over 0.9, effectively distinguishing responders from non-responders to CPB therapy. The different accuracies of the model in the different cell-types suggest the differential participation of cell-types in the immune response. We identify an 11-gene signature, predictive across various cancer types. We also show the co-dependency between the predictive power of gene pairs. Finally, we classify cells as either predictive or non-predictive in order to boost models' accuracy.

**Conclusion**

Our study underscores the necessity and potential of employing more sophisticated models for analyzing immune responses and single-cell data. We identified key genes and cell-level contributions to the immune response, provided a predictive gene signature, and got insights into gene interactions. This study laid some groundwork for further development of more advanced computational approaches and manifested the strength of such models in deep exploration of cancer immunity and treatment response.

### EACR2024-0656
### Biomarkers in sight? Multi-omics analysis of vulvar squamous cell carcinoma

*F. Lombardo[1], T. Zwimpfer[2], N. Rimmer[1], S. Götze[3], F. Singer[4], A. Bertolini[4], M. Prummer[4], F. Jacob[1], V. Heinzelmann-Schwarz[2]*
*[1]University Hospital Basel and University Basel, Department of biomedicine DBM, Basel, Switzerland*
*[2]University Hospital Basel, Department of Gynecological Oncology, Basel, Switzerland*
*[3]ETH Zurich, Institute of Translational Medicine ITM-Dep. of Health Sciences and Technology D-HEST, Zurich, Switzerland*
*[4]ETH Zürich, NEXUS Personalized Health Technologies, Zürich, Switzerland*

**Introduction**

Vulvar cancer exhibits poor response to traditional systemic therapies, and targeted treatment options remain limited compared to other gynecological cancers. However, breakthroughs in molecular oncology may soon enable precision analysis of the genomic landscape of vulvar cancer genomic landscape. This would pave the way for personalized treatment strategies integrating targeted therapies, immunotherapy, and established treatment modalities in other cancers types. Here, we characterized the proteogenome of vulvar squamous cell carcinoma (vSCC).

**Material and Methods**

We integrated clinicopathological, whole-exome sequencing, bulk RNA-sequencing (RNA-seq), and proteomics data derived from a cohort of 23 patients with vSCC. We computationally identified the HPV status of each patient's sample. To provide context for our findings, we also incorporated RNA-seq data from 5543 female The Cancer Genome Atlas (TCGA) samples, with analysis conducted using R/Bioconductor.

**Results and Discussions**

Our cohort consists of 34.8% (8/23) human papillomavirus (HPV)+ and 65.2% (15/23) HPV- vSCC patients. Early stage (12% (1/8) vs. 86% (12/15), $p<0.001$) was significantly associated with HPV-status. The most prevalent pathological mutated genes were FAT1 52% and KMT2D 48%. Mutations of TP53 and CDKN2 were exclusively present in HPV- patients with a frequency of 53% and 13.3%. HPV+ patients showed a higher frequency in PIK3CA mutations with 37.5% vs. 6.7% in HPV-. Proteomics showed an overexpression of POF1B and LGALSL in HPV+, which are both implicated in cancer progression. Integration of TCGA data identified tongue SCC (tSCC) as a valid comparison cohort. vSCC was associated with disadvantageous immune cell types such as elevated activated mast cells ($p=0.0031$), monocytes ($p=0.0027$), and M2 macrophages ($p<0.001$) compared to tSCC independent of the HPV-status.

**Conclusion**

We identified with tSCC a molecularly and clinico-pathologically similar cancer tissue to vSCC. We have identified several prognostic biomarkers for vSCC that are currently being tested in tissue microarrays of vSCC and tSCC for further confirmation and validation.

### EACR2024-0658
### Tribus: Semi-automated panel-informed discovery of cell identities and phenotypes from multiplexed imaging and proteomic data

*Z. Kang[1], A. Szabo[1], T. Farago[1], I.M. Launonen[1], F. Perez[1], E. Anttila[1], K. Elias[2], J. Casado[1], P. Sorger[3], A. Färkkilä[1,4,5,6]*
*[1]University of Helsinki, Research Program in Systems Oncology, Helsinki, Finland*
*[2]Brigham and Women's Hospital- Harvard Medical School, Division of Gynecologic Oncology, Boston, United States*
*[3]Harvard Medical School, Laboratory of Systems Pharmacology, Boston, United States*
*[4]University of Helsinki, iCAN Digital Precision Cancer Medicine Flagship, Helsinki, Finland*
*[5]Helsinki University Hospital, Department of Obstetrics and Gynecology, Helsinki, Finland*
*[6]University of Helsinki, Institute for Molecular Medicine Finland, Helsinki Institute of Life Sciences, Helsinki, Finland*

**Introduction**

Multiplexed imaging at single-cell resolution is becoming widely used to decipher the role of the cellular micro-environment in cancer and other complex diseases. To identify spatial patterns of single cells on a tissue, accurate cell-type phenotyping is a crucial step. We

© 2024 The Authors. Molecular Oncology is published by John Wiley & Sons Ltd on behalf of Federation of European Biochemical Societies present Tribus, an interactive, knowledge-based classifier that avoids hard-set thresholds and manual labeling, is robust to noise, and takes fewer iterations from the user than current methods of labeling.

**Material and Methods**

We developed Tribus, an open-source Python 3 package utilizing self-organizing maps for data clustering with an integrated Napari plug-in for fast visualization. It assigns the cell phenotypes based on user-defined label description tables and median marker expressions. Tribus has built-in visualization functions to gain insight into the input data and to evaluate the results. The Napari plug-in provides a user-friendly way to visualize the results and perform quality control. To explore the performance of Tribus, we used 1) manually expert annotated datasets from different tissue origins 2) datasets with cluster-based annotation 3) previously unpublished datasets. We benchmarked Tribus with two other similar prior knowledge-based tools: ACDC and Astir.

**Results and Discussions**

Tribus recovers fine-grained cell types as accurately as human experts on a MIBI-TOF Ductal carcinoma in situ (DCIS) dataset with a 37-plex antibody staining panel and 23 cell types. Tribus can also target ambiguous populations and discover phenotypically new subtypes in the DCIS dataset, such as CD36+CD31+ fibroblast and HER2-tumor luminal subtypes. Tribus outperforms other methods comparing efficiency and accuracy. Benchmarking on four public mass cytometry and multiplexed imaging datasets all with ground truth labels, Tribus reaches high accuracy compared with ACDC and Astir but with a shorter running time in one order of magnitude. Tribus yields rapid and accurate cell type calling in a large whole slide imaging dataset, consisting of over a million cells from five matched HGSC samples collected before and after neoadjuvant chemotherapy (NACT).

**Conclusion**

Overall, Tribus is a novel cell-type classification tool to analyze different sizes of datasets across different technologies, resulting in accelerated analysis of the spatial tumor microenvironment (TME). Tribus shows advantages in running times, accuracy, user-friendly and reproducibility.

### EACR2024-0686

### Dissecting melanoma intratumor heterogeneity through the lens of cellular morphology

H. De Blander[1], A. Jovic[2], V. Lu[2], J. Lamote[3], T. Swings[4], K. Miller[2], M. Ray[2], J. Aerts[2], P. Karras[1], J.C. Marine[1]
[1]*Laboratory for Molecular Cancer Biology- Center for Cancer Biology- VIB, Department of Oncology- KU Leuven, Leuven, Belgium*
[2]*Deepcell Inc, 4025 Bohannon Dr, Menlo Park- CA, United States*
[3]*VIB Flow Core, Leuven, Leuven, Belgium*
[4]*VIB Tech Watch Core, Leuven, Leuven, Belgium*

**Introduction**

Melanoma is renowned for its pronounced intratumor heterogeneity (ITH) and plasticity, crucial drivers of tumor evolution and therapy resistance. The conventional method for dissecting ITH, single-cell RNAseq, is time- and resource-intensive and involves cell lysis, leading to cell destruction. In response to this challenge, Deepcell has introduced an innovative platform enabling real-time exploration of the heterogeneity within mixed cell populations.

**Material and Methods**

The DeepCell platform utilizes high-dimensional morphological analysis of single-cell suspensions on a microfluidic chip, capturing bright-field images analyzed by a deep-learning method based on the Deepcell Human Foundation Model combining self-supervised learning and morphometrics. To evaluate whether cellular morphological features can capture cell state diversity defined through transcriptomics, we employed the Deepcell platform to profile well-characterized homogeneous human melanoma cell lines with distinct transcriptomic states – Melanocytic, Mesenchymal-like, Neural Crest-like (NCL), and transitory (Wouters et al., Nat Cell Biol 2020). Profiling encompassed both treatment-naive cells and those exposed to standard-of-care targeted therapeutics.

**Results and Discussions**

Unsupervised analysis of 19 different human cell cultures identified 8 distinct morpho-clusters. Importantly, cells representing the 4 different transcriptomic cell states were classified into 4 separate morpho-clusters. Even cells from the NCL and transitory transcriptomic cell states, which are generally considered as intermediate states lying in between the melanocytic and mesenchymal root states, could be distinguished. Cells subjected to various therapeutic modalities occupied distinct morpho-clusters, underscoring substantial morphological changes during therapy. These findings demonstrate that melanoma cell states defined by transcriptome analysis can be discerned through morphological features.

**Conclusion**

This emphasizes the immense potential of platforms like Deepcell, offering a rapid, non-destructive, and label-free approach to monitoring the plasticity of cancer cell states in real time. The morphodynamic trajectory embedding introduced by such platforms holds broad applicability for quantitatively analyzing cell responses through live-cell imaging across diverse biological and biomedical applications.

### EACR2024-0699

### Intrinsic and extrinsic regulation of cell cycle arrest in breast cancer

C. Celik[1], E. Withnell[1], M. Secrier[1]
[1]*University College London, Genetics Evolution and Environment, London, United Kingdom*

**Introduction**

Cell cycle dysregulation stands as a pivotal aspect of cancer progression, demanding a comprehensive understanding of the mechanisms underlying tumorigenesis and therapy resistance. In this study, we delve into the immune context of cell cycle arrest in breast cancer to elucidate its intricate molecular mechanisms.

**Material and Methods**

We conducted a thorough analysis of 43 primary breast cancer samples, encompassing diverse subtypes, using

Molecular Oncology 18 (Suppl. 1) (2024) 1–495
© 2024 The Authors. Molecular Oncology is published by John Wiley & Sons Ltd on behalf of Federation of European Biochemical Societies published single-cell RNA sequencing datasets. Our analysis revealed a complex transcriptional landscape of breast cancer cells, enabling us to identify distinct subsets of highly proliferating and G0 arrested tumour cells by leveraging a robust G0 arrest expression signature developed in our group previously.

**Results and Discussions**

G0 arrest was observed as a pervasive state across all major breast cancer subtypes, with increased prevalence in ER+ tumours and reduced in triple negative breast cancer, as expected. Furthermore, we found that interactions established between tumour cells and their microenvironment were considerably rewired when cells were arrested in G0, compared to their proliferative counterparts. Specifically, we observed TGFB1-mediated cell cycle arrest actively induced by tumour-associated macrophages, CAFs, B cells and Tregs within the tumour microenvironment, alongside a significant depletion of interactions with T cells. We also observed immune cell-enriched semaphorin 4D ligand and plexin B1 receptor interactions, hindering T cell infiltration. In addition, exploration of gene regulatory networks in G0 arrested cells revealed a rewiring of modules associated with growth inhibition, unfolded protein response-induced stress and stemness. The G0 arrested cancer cells also presented increased EMT hallmarks, with the majority residing in a hybrid E/M state, which has previously been linked with metastatic potential. These findings were confirmed in 12 spatial transcriptomics slides across breast cancer subtypes, where we identified G0 arrest hotspots enriched in EMT hallmarks that appeared to be shielded from cytotoxic T cell recognition by myCAFs and macrophages.

**Conclusion**

To summarise, our study defines a G0 arrested, hybrid E/M state that is actively induced within developing primary tumours as a response to proteostatis-linked stress and appears more successful at evading immune recognition compared to fast cycling cells. These insights hold promise for developing tailored therapeutic strategies to overcome immune resistance mechanisms in breast cancer.

### EACR2024-0736

### Assessment of pathogenicity and dynamics of clinically reported mutations in Ribosomal s6 Kinase1 (RSK1): an in-silico study

*V. Chikhale*[1,2], *A. Varma*[1,2]

[1]*Advanced Centre for Treatment, Structural and molecular biology, Navi Mumbai, India*

[2]*Homi Bhabha National Institute, Life science, Navi Mumbai, India*

**Introduction**

RSK1 is a highly specific serine-threonine protein kinase working downstream of the RAS-MAPK pathway. It is composed of two distinct functional kinase domains, an N-terminus kinase domain (NTKD) and a C-terminus kinase domain (CTKD). RSK1 regulates crucial cellular processes such as growth, division, proliferation, and differentiation and is also observed to be abnormally active in different cancer conditions leading to adverse disease outcomes. This implies that RSK1 may have a role in the regulation of key cellular functions in cancer which makes it a potential therapeutic target. The structural, and functional correlation of RSK1 missense mutations has not been explored. Therefore it is important to understand the pathogenicity and structural characteristics of RSK1 mutations which can then provide better insights into functional consequences and designing inhibitors. The in-silico pathogenicity prediction tools aid the screening and selection of deleterious mutants from larger mutational datasets. This study aimed to evaluate the effect of cancer-associated missense mutations on the RSK1-CTKD structure.

**Material and Methods**

Missense mutations reported in different cancer studies for RSK1 were retrieved from cBioPortal. These mutations were further screened for pathogenicity using SIFFT, PolyPhen2, PMUT, PhD-SNP, and PROVEAN in-silico prediction tools. Molecular dynamic simulations were performed to assess the alteration in the dynamics of the mutant RSK1-CTKD.

**Results and Discussions**

Based on the outcome of in-silico pathogenicity tools mutations R434P, H533N, P613L, S720C, T701M, A704T, R725Q, R725W, R726Q, and S732F were categorized as deleterious. Comparative molecular dynamic simulation and principal component analysis of these mutants and WT indicated that R434P, T701M, A704T, R725W, and R726Q perturb the conformational stability, flexibility, and integrity of RSK1-CTKD.

**Conclusion**

Overall in-silico and molecular dynamics simulation analysis implicated that R434P, T701M, A704T, R725W, and R726Q may have a substantial effect on the structure of RSK1-CTKD. Considering the importance of RSK1 in the regulation of cellular activity, these mutations may have diverse effects on the function and regulation of RSK1. This study provides significant insights into the mutation-induced alteration in the dynamics of RSK1-CTKD.

### EACR2024-0768

### An ovarian cancer scRNA-seq atlas to dissect tumor-host interactions underlying metastatization and chemoresistance

*P. Lo Riso*[1], *M.R. Sallese*[1], *C.E. Villa*[2], *G. Testa*[1]

[1]*European Institute of Oncology, Experimental Oncology, Milan, Italy*

[2]*Human Technopole, Neurogenomics, Milan, Italy*

**Introduction**

The high intra-tumoral and inter-patient heterogeneity of high grade serous ovarian cancer (HGSOC) is one of the main factors hampering the identification of novel therapeutic treatment to improve survival of HGSOC patients. Single cell technologies are affording new inroads for the deep molecular characterization of such tumor heterogeneity, in both primary and metastatic samples.

**Material and Methods**

In this context, we built a manually curated HGSOC transcriptomics atlas (~1,5 million cells, 79 patients)

© 2024 The Authors. Molecular Oncology is published by John Wiley & Sons Ltd on behalf of Federation of European Biochemical Societies based on publicly available datasets and developed a data integration strategy specifically applicable to very heterogenous single cell data. Indeed, to obtain a more faithful representation of the data, we performed identification of cell populations through a new cell labeling strategy and metacells derivation separately for every sample, to identify the distinct subpopulations characterizing each patient. In addition, to robustly represent the space describing the four main cell populations of the dataset (i.e. tumoral, immune, stromal and endothelial) for each of the latter, we selected the space identified by the union of highly variable genes, computed separately on every dataset, to preserve the variability of the system. Next, we fed a variational autoencoder with metacells data to integrate and characterize the different subpopulations that may be similar across samples.

**Results and Discussions**

After integration, we were able to phenotype tumor subpopulations involved in therapy response by characterizing the chemotherapy-induced transcriptional features. The robust deep learning-based framework allows to easily and iteratively expand the atlas with newly generated datasets, facilitating the interpretation of disease-associated features of HGSOC in external datasets.

**Conclusion**

This atlas represents a transformative resource for the community by enabling the investigation of the interaction between tissue microenvironment (TME) cells and tumoral cells in shaping the metastatic process.

### EACR2024-0795
### Enhancing Omics Data Integration with Correlation-Based Feature Extraction in Single-Cell RNA-Sequencing

*A. Kollotzek*[1], *V. Franke*[1]

[1]*Max Delbrück Center - Berlin Institute for Medical Systems Biology, Bioinformatics and Omics Data Science, Berlin, Germany*

**Introduction**

Recent advancements in omics technologies have highlighted the need for robust data integration methods. Traditional integration methods often rely on an empirically selected set of genes, such as highly variable genes, which are believed to carry the most biological information. However, this approach can overlook crucial aspects of biological variability.

**Material and Methods**

Here, we introduce AngleMania, a first principles derived feature extraction algorithm designed to optimize the preprocessing phase for omic data integration, particularly in single-cell RNA sequencing (scRNA-seq). Unlike traditional methods that depend on empirical gene selection, AngleMania identifies genes or gene pairs that exhibit conserved correlation patterns across multiple datasets. This approach ensures the capture of stably correlated gene pairs, which represent conserved biological variability, while filtering out variations predominantly arising from technical sources.

**Results and Discussions**

When integrated into existing pipelines, AngleMania demonstrates significant improvements in the accuracy and biological relevance of the integrated omics data. We have used AngleMania to integrate tumour cells originating from four different cohorts of neuroblastoma patients. The integration revealed a common transcriptional manifold, and enabled definition of multiple novel cellular states.

**Conclusion**

AngleMania represents a significant step forward in omic data integration, especially for scRNA-seq. By moving beyond empirical gene selection and focusing on conserved correlation patterns, it provides a more robust and biologically relevant method of integrating complex datasets. This approach has the potential to uncover new biological insights and facilitate a deeper understanding of cellular states in cancer.

### EACR2024-0891
### UMIvar: Unveiling Insights in UMI Deduplication Software Benchmarking through UMI-aware Variant Simulation

*D. G. Camblor*[1], *J. Martín-Arana*[1], *F. Gimeno-Valiente*[2], *B. García-Micó*[1], *B. Martínez-Castedo*[1], *J.F. Català-Senent*[3], *F. Robledo-Yagüe*[3], *S. Zúñiga-Trejos*[3], *A. Cervantes*[1], *N. Tarazona*[1]

[1]*INCLIVA Biomedical Research Institute, Department of Medical Oncology, Valencia, Spain*

[2]*University College London Cancer Institute, Cancer Evolution and Genome Instability Laboratory, London, United Kingdom*

[3]*INCLIVA Biomedical Research Institute, Bioinformatics Unit, Valencia, Spain*

**Introduction**

The use of Unique Molecular Identifiers (UMIs) has proven to hold great value for NGS analysis, especially in liquid biopsy samples and for low-depth settings such as whole-exome sequencing (WES), where it aids in the detection of low frequency variants. However, the comparative benefit of using different UMI deduplication software in variant calling has not been thoroughly investigated, partially due to a lack of accessible ground truth resources with UMI information. To address this, we developed UMIvar, a bioinformatics tool that simulates UMI-aware variants in alignment data, specifically designed with convenience and flexibility in mind for the purpose of variant calling benchmarking analyses.

**Material and Methods**

UMIvar was developed in Python using the pysam library to handle BAM files. The source code and documentation are available at: https://github.com/dgcamblor/UMIvar. UMIvar (v1.1) was firstly tested by generating a set of 1283 variants with allele frequencies (VAFs) ranging from 1% to 100% in 5 different BAM files and performing sensitive variant calling with VarDict (v1.8.3). The tool was later used to benchmark the main UMI deduplication software available: UMI-tools (v1.1.4), fgbio (v2.1.0), UMICollapse (v1.0.0), gencore (v0.17.2), and MarkDuplicates (Picard v3.0.0). A set of 621 variants (VAFs: 1% - 10%) were simulated in 32 WES-derived BAM files ranging from 51X to 141X median coverage. Variant calling was subsequently performed on each deduplicated BAM using Lofreq (v2.1.5). For comparative purposes, the same

Molecular Oncology 18 (Suppl. 1) (2024) 1–495

© 2024 The Authors. Molecular Oncology is published by John Wiley & Sons Ltd on behalf of Federation of European Biochemical Societies benchmarking approach was performed in the BRP dataset (Sample Ef) from the SEQC2 Liquid Biopsy study, which contains 230 true variants (median VAF = 0.2%; median coverage range: 5231X - 5942X).
**Results and Discussions**
UMIvar proved to accurately simulate the initial testing of 1283 variants, with a global root mean square error of 0.106 for VAF differences (which are mostly explained by the UMI availability and discarding of unfit reads). In the benchmarking analysis, fgbio provided the best performance of mean recall with Lofreq: 0.784 ± 0.039. Results were consistent with those obtained in the BRP dataset, where fgbio again surpassed the other UMI deduplication software with a mean recall of 0.809 ± 0.035.
**Conclusion**
UMIvar emerges as a valuable tool for benchmarking UMI-related software and to help select optimal bioinformatics approaches to improve the detection of low frequency variants in cancer data.

### EACR2024-0918
### Single cell RNA sequencing reveals tumor-supporting features in primary CNS lymphoma

*K. Pickard[1], J. Thompson[2], A. Joshi[2], E. Stephenson[1], R. Botting[1], T. Ness[3], A. Rose[1], C. Bacon[4], M. Haniffa[1], L. Jardine[1]*
*[1]Newcastle University, Biosciences Institute, Newcastle upon Tyne, United Kingdom*
*[2]Newcastle upon Tyne Hospitals NHS Foundation Trust, Neuropathology, Newcastle upon Tyne, United Kingdom*
*[3]Newcastle upon Tyne Hospitals NHS Foundation Trust, Cellular Pathology, Newcastle upon Tyne, United Kingdom*
*[4]Newcastle University, Northern Institute for Cancer Research, Newcastle upon Tyne, United Kingdom*
**Introduction**
Primary central nervous system lymphoma (PCNSL) is an aggressive B cell lymphoma with poor outcomes and a need for novel therapies. We used single cell RNA sequencing to provide an overview of the cells within the PCNSL tumour and microenvironment, and to profile the interactions between these cells to identify potential therapeutic targets.
**Material and Methods**
We obtained fresh tissue specimens of PCNSL from four individuals who underwent neurosurgical biopsy, and one control sample from a patient with glioblastoma multiforme (GBM). We processed the samples using the 10x Genomics Chromium Single Cell 5' protocol. Downstream computational analysis was performed using Cell Ranger software and the Python Scanpy package. CellphoneDB was used to explore receptor-ligand interactions
**Results and Discussions**
Following preprocessing and quality control, 69,125 cells were available for analysis. Principal component analysis and embedding followed by data integration and Leiden graph-clustering revealed 20 discrete clusters. We then identified highly differential genes in each cluster to find marker genes which we used for manual annotation. We confirmed these annotations with automated cell type annotation using a CellTypist model. PCNSL tumour cells represented the most abundant cell population. Immune cells included CD8+ cytotoxic T cells, CD4+ T cells, NK cells and myeloid cells, as well as non-malignant B cells. Malignant B cells are heterogenous and sub-clustering reveals differing phenotypes including those closely resembling germinal center B cells and plasma cells. We used CellphoneDB v5 to explore receptor-ligand interactions and found significant interactions of the tumor necrosis factor superfamily (TNFSF) system between immune and malignant B cells (e.g. BAFF and APRIL ligand/receptor interactions on myeloid and malignant B cells) with downstream signaling of the NF-kB pathway. NF-kB activation could contribute to PCNSL tumour survival and proliferation.
**Conclusion**
PCNSL samples comprise malignant cells in an immune milieu, of which T cells are the most abundant cell type. Ligand-receptor interaction analysis reveals upregulated immune and PCNSL tumour cell interactions of the TNFSF system which activates the NF-kB pathway and contributes to tumour survival and proliferation.

### EACR2024-0961
### Context-dependent effects of CDKN2A and other 9p21 gene losses during the evolution of oesophageal cancer

*C. Colomina Basanta[1,2], P. Ganguli[1,2], A. Mendez[1,2], M. Armero[1,2], A.S. Amelia[1,2], A. Zahra[1,2], H. Misetic[1,2], R.C. Fitzgerald[3], F. Ciccarelli[1,2]*
*[1]Barts Cancer Institute, Centre for Cancer Genomics & Computational Biology, London, United Kingdom*
*[2]Francis Crick Institute, Cancer Systems Biology Laboratory, London, United Kingdom*
*[3]Early Cancer Institute, Hutchison Research Centre, Cambridge, United Kingdom*
**Introduction**
Oesophageal Adenocarcinoma (OAC) develops from Barrett's Oesophagus (BO) through the progressive acquisition of genetic alterations. Loss of function (LoF) of *CDKN2A* is frequent in BO and OAC patients, it is predictive of bad prognosis, and occurs mostly through the homozygous deletion of chromosome 9p21 locus very early in BO pathogenesis. How *CDKN2A* and other 9p21 gene co-deletions affect OAC evolution remains understudied.
**Material and Methods**
We explored the effects of 9p21 loss in 1032 OACs, 257 cancer progressors and 99 non-progressor BOs with matched genomic, bulk transcriptomic, and clinical data. We applied high-dimensional tissue profiling coupled with RNAscope to study the impact of 9p21 loss in the tumour microenvironment of OACs.
**Results and Discussions**
Using logistic regression models of OAC initiation based on LoF of TP53 and CDKN2A we observed that despite its cancer driver role, *CDKN2A* loss in BO prevents OAC initiation by counter-selecting the acquisition of *TP53* alterations. Our analysis revealed that 9p21 gene co-deletions predict poor patient survival in OAC but not BO through context-dependent effects on cell cycle, oxidative phosphorylation, and interferon response. Immune quantifications using bulk transcriptome,

© 2024 The Authors. Molecular Oncology is published by John Wiley & Sons Ltd on behalf of Federation of European Biochemical Societies

RNAscope and high-dimensional tissue imaging showed that *IFNE* loss reduces immune infiltration in BO but not OAC. Mechanistically, *CDKN2A* loss suppresses the maintenance of squamous epithelium, contributing to a more aggressive phenotype.

**Conclusion**

Our study demonstrates context-dependent roles of cancer genes during disease evolution, with consequences for cancer detection and patient management.

### EACR2024-1006
### CancerHallmarks.com: a gene set enrichment analysis of the cancer hallmarks

*W.J. Kothalawala[1], M. Otília[1], B. Győrffy[1]*
*[1]Semmelweis University, Department of Bioinformatics, Budapest, Hungary*

**Introduction**

The "Hallmarks of Cancer" framework serves as a crucial model for understanding fundamental principles shared among various cancer types. However, the absence of a consensus gene set for these hallmarks leads to diverse interpretations across studies. In this study, we aimed to establish a consensus gene set for cancer hallmarks by integrating data from multiple mapping resources.

**Material and Methods**

Data from seven projects were consolidated to identify genes associated with ten cancer hallmarks. Subsequently, a cancer hallmarks enrichment analysis was conducted for prognostic genes related to overall survival across twelve types of solid tumors.

**Results and Discussions**

Our analysis revealed that "Tissue invasion and metastasis" was notably prominent in stomach, pancreatic, bladder, and ovarian cancers. "Sustained angiogenesis" predominated in lung squamous carcinomas, while "genome instability" showed significant enrichment in lung adenocarcinomas, liver, pancreatic, and kidney cancers. Pancreatic cancers exhibited the highest enrichment of hallmarks, indicating their complex nature. Conversely, in melanomas, liver, prostate, and kidney cancers, a single hallmark was enriched among prognostic markers of survival. Additionally, we developed an online tool (www.cancerhallmarks.com) for identifying cancer-associated hallmarks from new gene sets.

**Conclusion**

We established a consensus list of cancer hallmark genes, providing insight into the unique patterns of hallmark enrichment associated with survival across different tumor types. This project enhances the utility of the hallmark concept as an organizational tool by linking genes to specific biological functions, with potential pharmacological implications.

### EACR2024-1008
### MILO: Microsatellite Instability detection in LOw-quality samples reveals tumour evolutionary dynamics

*Q. Guo[1,2,3], E. Lakatos[1,4], J. Househam[1], S. Nowinski[1], L. Zapata[1], I. Al-Bakir[3], A.M. Baker[1], V. Mustonen[2], T.A. Graham[1,3]*
*[1]The Institute of Cancer Research, Centre for Evolution and Cancer, London, United Kingdom*
*[2]University of Helsinki, Department of Computer Science, Helsinki, Finland*
*[3]Queen Mary University of London, Barts Cancer Institute, London, United Kingdom*
*[4]Chalmers University of Technology, Department of Mathematical Sciences, Gothenburg, Sweden*

**Introduction**

Deficient mismatch repair (MMRd) machinery can lead to varying levels of microsatellite instability (MSI) in cancers. Assessing MSI levels provides insights into progressive biomarkers for disease advancement and determines eligibility for immunotherapy. However, current MSI detection methods demonstrate diminished efficacy when applied to biopsies with low tumor content and low coverage. Here, we aim to develop a bio-informatic tool that facilitate accurate, time-efficient, and cost-effective MSI detection in these challenging samples.

**Material and Methods**

We explored mutational spectra in a large cohort of n=835 shallow whole genome sequenced (~0.1X) samples encompassing different organs. Our goal was to identify signature features that could be reliably detected despite multiple sources of technical noise in these datasets. We then validated these features in a set of deep-sequenced MMRd samples (n=544). Using these features, we developed a bioinformatic tool for detecting Microsatellite Instability levels in LOw-quality samples (MILO), which is further applied to study MSI evolution over time from precursor lesions to metastasis.

**Results and Discussions**

We discovered that MMRd causes a novel indel signature characterised by longer deletions at microsatellites, which is distinct from any source of technical noise. Our tool MILO demonstrates 100% accuracy in detecting MMRd within samples with 2%-15% tumour purity using shallow sequencing data, outperforming MSIsensor2, which achieves a recall rate of only 28% in comparison. We also applied MILO to longitudinal biopsies from the inflammatory bowel disease colon and detected the emergence of MMRd clones before the development of cancer. Our analysis revealed that the longer-deletion signature was notably more prevalent in metastatic MMRd cancers compared to primary ones. We observed an accumulation of these mutations in a step-wise manner, indicating that the MSI level within an MMRd cancer evolved progressively over time.

**Conclusion**

Our novel bioinformatics tool MILO, utilising a novel longer-deletion signature, offers robust MSI detection even in samples with low tumour purity and low sequencing coverage. This indel signature provides valuable insights into the evolving genome of MMRd cancers. Our tool presents an opportunity to monitor MSI evolution over time where sequencing quality cannot be guaranteed.

### EACR2024-1010
### Decoding Transcriptional Heterogeneity and Metastasis in High-Grade Serous

© 2024 The Authors. Molecular Oncology is published by John Wiley & Sons Ltd on behalf of Federation of European Biochemical Societies

### Carcinoma through Inferred Transcription Factor Activity

K. Zhang[1], E. Kahelin[2], A. Häkkinen[3], O. Lehtonen[1], K. Lavikka[1], S. Hietanen[4], J. Hynninen[4], A. Virtanen[2], S. Hautaniemi[1]
[1]University of Helsinki, Research Program in Systems Oncology, Research Programs Unit, Faculty of Medicine Helsinki, Finland
[2]Helsinki University Hospital, Department of Pathology- University of Helsinki and HUS Diagnostic Center, Helsinki, Finland
[3]Boston Children's Hospital- Harvard Medical School, Computational Health Informatics Program, Boston, United States
[4]University of Turku and Turku University Hospital, Department of Obstetrics and Gynecology, Turku, Finland

**Introduction**

High-grade serous carcinoma (HGSC) is the most lethal gynecologic malignancy, characterized by extensive genomic instability and widespread metastasis. Transcription factors (TFs) are crucial in regulating gene expression and play key roles in cancer progression and metastasis. By inferring TF activities, we aim to uncover the transcriptional and phenotypic heterogeneity of HGSC, providing insights into HGSC metastasis.

**Material and Methods**

We performed RNA-sequencing and whole-genome sequencing for 350 HGSC samples from 160 patients. We modeled TF and copy number regulations on gene expression using linear conditional Gaussian networks and inferred TF activities using an Expectation-Maximization (EM) procedure. To estimate morphological changes, we utilized a panoptic segmentation model to simultaneously segment both tissue regions and nuclei in scanned hematoxylin-eosin stained slides.

**Results and Discussions**

Our findings reveal significant inter-site heterogeneity in cancer-cell transcriptional profiles at the time of diagnosis and varying chemotherapy responses across different anatomical sites. Notably, solid tumor sites exhibited changes in TF activities post-treatment, in contrast to the consistent profiles in ascites samples, indicating a site-specific adaptive response to therapy. The activities of the epithelial-mesenchymal transition (EMT) and chemokine TF modules were identified as key prognostic indicators, with high activity levels associated with shorter platinum-free intervals and reduced overall survival, independent of established prognostic factors. Trajectory analysis revealed two distinct metastatic lineages linked to the EMT and chemokine modules, highlighting their cooperative yet distinct roles in HGSC metastasis. We found a significant increase of stromal cell proportions along the metastasis trajectory and morphological transformations of cancer cells at the cancer-stroma interface, indicating active cancer-stroma interactions.

**Conclusion**

This study provides a comprehensive view of HGSC inter-site heterogeneity and molecular dynamics of metastasis, enhancing the understanding of HGSC metastasis and offering potential avenues for targeted therapies.

### EACR2024-1067
### Mutation-Attention 2: joint tumour type and subtype classification using deep learning on somatic variants of 14,537 tumour whole genomes

P. Sanjaya[1], G.E.R. Consortium[2], E. Pitkänen[1]
[1]Institute for Molecular Medicine Finland FIMM, Helsinki Institute of Life Science- University of Helsinki, Helsinki, Finland
[2]Genomics England, Genomics England, London, United Kingdom

**Introduction**

Deep learning has enabled accurate tumour type classification in cancer whole genome datasets. However, tumour subtyping on somatic mutations is still difficult due to cancer heterogeneity. Moreover, how to best utilise deep representation learning in tumour subtype discovery remains unexplored.

**Material and Methods**

Here we introduce Mutation-Attention 2 (MuAt2), a deep neural network designed to learn representations of tumours based on simple and complex somatic alterations. Building upon the previous model (MuAt1) [1], MuAt2 classifies the type and subtype of a tumour in a single forward pass. To train MuAt2 models, we transferred pretrained parameters from MuAt1 to MuAt2, and retrained models on 14,527 whole tumour genomes across 15 tumour types, spanning 68 subtypes in Genomics England (GEL) [2].

**Results and Discussions**

MuAt2 achieved classification accuracy of 88% for tumour types and 58% for tumour subtypes, with top-5 accuracies of 96% and 91%, respectively. We demonstrate that MuAt2 learns representations pertaining to clinically and biologically significant tumour entities, including microsatellite unstable cancers, metastases, and cancers of unknown primary.

**Conclusion**

In conclusion, MuAt2 representations offer insights into tumour type and subtype, with potential applications in clinical settings.

*This research was made possible through access to data and findings in the National Genomic Research Library via the Genomics England Research Environment.*

*References: [1] Sanjaya, Prima, et al. "Mutation-Attention (MuAt): deep representation learning of somatic mutations for tumour typing and subtyping." Genome medicine 15.1 (2023): 47. [2] Caulfield, Mark, et al. "The National Genomics Research and Healthcare Knowledgebase v5". Genomics England (2020)*

### EACR2024-1078
### Prediction of novel genes involved in Prostate Cancer using Integrated Bioinformatics Methods

E.E. Yıldırım[1], B. Afifi[1], E. Bertan[1], S. Vural Korkut[1]
[1]Yıldız Technical University- Institute of Science, Molecular Biology and Genetics, Istanbul, Turkiye

**Introduction**

Prostate cancer is a complex disease with a range of molecular mechanisms. Understanding the underlying

© 2024The Authors. Molecular Oncology is published by John Wiley & Sons Ltd on behalf of Federation of European Biochemical Societies biological pathways and genetic factors that contribute to the development and progression of the disease is crucial for developing effective diagnostic and treatment approaches. In this study, we aimed to identify potential novel genes that may play a role in the molecular mechanisms of prostate cancer.

**Material and Methods**

The researchers analyzed six prostate cancer datasets from the Gene Expression Omnibus (GEO) repository. They identified overlapping upregulated and down-regulated genes in at least two out of the six datasets separately as two lists of genes. For the identified DEGs, various types of analyses were conducted, including GO and KEGG pathway enrichment analysis, construction of PPI network using STRING, and PPI network analysis in Cytoscape. After selecting the previously unreported genes associated with prostate cancer, their expressions were validated using GEPIA, and disease-free survival plots were generated using the Kaplan-Meier Plotter.

**Results and Discussions**

In our study, we identified 264 upregulated genes and 469 downregulated genes across multiple datasets, with functional analysis revealing their involvement in various biological processes and pathways. Using Cytoscape, we identified 86 upregulated and 82 downregulated genes as hub genes via different methods. Furthermore, a PubMed search unveiled 19 candidate novel genes potentially linked to prostate cancer. Validation through GEPIA and UCSC Xena platforms confirmed differential expression of these genes in cancer tissues, with SLC27A2, APOF, CPLX3, SBSPON, and LDB3 showing significant associations with prostate cancer survival times.

**Conclusion**

Our findings discovered that potential novel genes include RAD51AP1, APOF, SAMD13, B3GAT1, DUS1L, HIST3H2A, SLC27A2, VSTM2L, FXYD1, AHNAK2, BEX1, COL16A1, COL17A1, CPLX3, LDB3, NTF4, PPP1R3C, RCAN2, and SBSPON may play a important role in the molecular mechanisms of prostate cancer and they have not been connected to prostate cancer in any publication in PubMed. Importantly, the study found that the high and low expression of the SLC27A2, APOF, CPLX3, SBSPON, and LDB3 genes were significantly related to long disease-free survival in prostate cancer patients. These findings suggest that these genes may be important prognostic biomarkers for prostate cancer.

### EACR2024-1118
### Country-specific differences in the colorectal cancer microbiome

*A. Stafford[1], A. Basgaran[1], J.M. Lam[1], M. Hobbs[1], D. Delanoue[1], J. Arney[1], D. Korovilas[1], S. Moore[1], M. Alomari[1], N. Sharma[1]*

*[1]BioCorteX Inc, BioCorteX Inc, London, United Kingdom*

**Introduction**

There is increasing interest in the role of the gut microbiome in the aetiology of colorectal cancer (CRC) (Sun et al, 2020) and an altered gut microbiome has been evidenced in CRC (Marchesi et al., 2011). China and the USA are predicted to have the highest number of new CRC cases in the next 20 years (Xi & Xu, 2021). Country-specific differences in microbiome profiles have been noted, and are attributed to changes in host genetics, lifestyle, and diet (Parizadeh & Arrieta, 2023). Given the growing interest in the causative role of the microbiome in CRC, and the increasing prevalence of CRC in China and the USA, a deeper understanding the CRC-associated, country-specific microbiome is needed.

**Material and Methods**

We leveraged BioCorteX's industry-leading knowledge graph, Carbon Knowledge v20240301_102231, to identify 2,964 stool samples across CRC patients from four countries: USA (n=1,504), China (n=791), Germany (n=357), and Austria (n=312). Analysis included alpha and beta diversity, compositional abundance, and PERMANOVA.

**Results and Discussions**

CRC patients from the USA harboured significantly lower microbial diversity (Shannon Index, SI=2.88) than patients from China (SI=4.18, $p<0.0001$), Austria (SI=4.65, $p<0.0001$), and Germany (SI=4.80, $p<0.0001$). T-SNE showed clear separation of the USA CRC-associated microbiome. Microbiomes from China, Germany, and Austria formed a separate cluster, within which, China occupied a distinct space. It is noteworthy that the European countries harboured greater similarity to China than the USA. PERMANOVA confirmed a significant difference between patients from the USA and China (p=0). Bacteroides fragilis, Escherichia coli, and Bacteroides vulgatis were associated with the Chinese CRC-associated microbiome than the USA; these species produce genotoxins and have been proposed as non-invasive CRC biomarkers (Dramsi et al, 2022, Jia et al, 2022, Bonnet et al, 2013). Streptococcus parasuis, Staphylococcus aureus, and Klebsiella pneumoniae were associated with USA patients.

**Conclusion**

The BioCorteX Carbon Mirror platform demonstrates country-species differences in CRC-associated stool microbiomes among patients, and is suggestive of country-specific microbiome-based drivers of CRC. It will also be particularly important to account for these differences in the pursuit of microbiome-based biomarkers for CRC.

*(Declaration: A. Stafford is an employee of Biocortex and has options)*

### EACR2024-1137
### Survival analysis based on Circulating Tumor Cells identified by Deep Learning

*F. Coumans[1], N.H. Stoecklein[2], L.W. Terstappen[2], C.J. Punt[3], J.Y. Pierga[4], F.C. Bidard[4], G. Signorini[5], L. Biasiolli[5], G. Medoro[5]*

*[1]Decisive Science, Data Science, Amsterdam, The Netherlands*

*[2]Heinrich-Heine University- University Hospital Düsseldorf, Department of General- Visceral and Pediatric Surgery, Düsseldorf, Germany*

*[3]Julius Center for Health Sciences and Primary Care-University Medical Center Utrecht- Utrecht University, Dept. of Epidemiology, Utrecht, The Netherlands*

*[4]Institut Curie, Department of Medical Oncology, Paris and Saint Cloud, France*

*[5]Menarini Silicon Biosystems, Innovation, Bologna, Italy*

**Introduction**

© 2024 The Authors. Molecular Oncology is published by John Wiley & Sons Ltd on behalf of Federation of European Biochemical Societies

The presence of Circulating Tumor Cells (CTCs) assessed with the CELLSEARCH® system is strongly associated with poor overall survival in metastatic cancer [1]. An operator classifies immunofluorescent cell images as CTCs based on cell-like morphology and presence of DNA and cytokeratin, and abscence of CD45. This procedure is time-consuming and potentially subjective. Therefore, we have developed a deep learning (DL) algorithm (CellFind®, Menarini Silicon Biosystems) for automated CTC identification based on previous work [2], and compared the prognostic performance on clinical studies of metastatic carcinoma.

**Material and Methods**

CELLSEARCH image sets from studies in metastatic colorectal [3] and breast [4] cancer were used to evaluate the prognostic performance of operator CTC (OP-CTC) and CellFind CTC (CF-CTC) counts. In total, 446 image sets from 442 patients before initiation of a new line of therapy and 440 image sets from 440 patients 1-6 weeks after initiation of treatment were evaluated. The evaluation was performed by computing the Cox hazard ratio for overall survival (HR-OS) after dichotomizing the results into unfavorable and favorable using a threshold of 3 for colorectal cancer (i.e. ≥3 and < 3), and a threshold of 5 for breast cancer.

**Results and Discussions**

In all 886 image sets, OP-CTC counts ranged from 0 to 14274 (median 0, σ 495.5) and CF-CTC counts from 0 to 15397 (median 0, σ 538.9). The correlation between OP-CTC and CF-CTC of $R^2 = 0.996$ (slope 1.08 intercept 2.73). The concordance rate of favorable vs unfavorable CTC groups by OP-CTC and CF-CTC was 96%. For colorectal cancer, the HR-OS of 352 patients at baseline was 2.3 (95% confidence interval 1.7-3.0) for OP-CTC and 2.3 (1.7-3.0) for CF-CTC. 440 patients at first follow-up had an HR-OS of 3.2 (2.2-4.8) for OP-CTC and 4.0 (2.7-5.8) for CF-CTC. For 90 breast cancer patients at baseline, the HR-OS was 2.3 (0.9-6.4) for OP-CTC and 2.9 (1.0-8.0) for CF-CTC.

**Conclusion**

We introduced CellFind, a DL algorithm for research use that eliminates operators' bias and review time in CELLSEARCH CTC identification. HR results suggest that CellFind can perform similarly to or better than human reviewers in predicting OS, thus enabling fast, reproducible, and bias-free enumeration of clinically relevant CTCs.

1. *Full intended use: documents.cellsearchctc.com*
2. *Zeune LL et al. Nature Machine Intelligence 2, 124–133, 2020.*
3. *Cohen SJ et al. Journal of Clinical Oncology. 2008; 26(19): 3213–3221.*
4. *Pierga, J.Y. et al. Ann Oncol, 2012. 23(3): p. 618-24.*

### EACR2024-1144
### Phylogeny of Systemic Cancer via Single Cell Lineage Reconstruction

*A. Onn[1], Z. Czyz[1], M. Werner-Klein[1], M.S. Ghosh[1], O. Raz[2], Y. Neumeier[2], T. Marx[2], E. Shapiro[2], C.A. Klein[1]*
*[1]University of Regensburg, Experimental Medicine and Therapy Research, Regensburg, Germany*
*[2]Weizmann Institute of Science, Faculty of Mathematics and Computer Science, Rehovot, Israel*

**Introduction**

The evolution of solid cancers from local to systemic disease is only circumstantially understood. Conventional approaches rely on the comparative analysis of primary tumours and matched metastasis, however, the analysis of only two snapshots may fall short of capturing disease dynamics. Moreover, most phylogenies assume that each driver mutation is generated maximally once, which is not justified in cancer. To address these points, we included the analysis of disseminated cancer cells (DCCs) isolated from bone marrow or lymph nodes long before metastatic manifestation and based our phylogenies not on selected aberrations that may change cellular fitness levels, but on unselected short tandem repeats (STRs) that are unaffected by convergent evolution.

**Material and Methods**

We included 10 patients suffering from either melanoma, non-small cell lung cancer, or breast cancer that were followed from localized disease to systemic cancer over several years. Of these we prepared 1695 samples (mostly single-cell) from primary tumours, metastatic biopsies, lymph node, bone marrow, oral mucosa and blood. We isolated single healthy cells, stained for example by the markers CD3 (T-cells) or CD31 (endothelial cells), and cancer cells. All samples were analysed using a novel single-cell genomics protocol, based on sequencing of approximately 14,000 STRs that were then used to reconstruct the phylogenetic structure of each individual disease. Following sequencing, the samples were filtered according to various criteria, and out of the remaining 1026 samples, lineage trees were reconstructed. Additionally, most samples underwent analysis for copy number variation (CNV).

**Results and Discussions**

Eight out of ten trees displayed clusterings that reflected the histogenetic origin of the isolated cells. Primary tumour-derived cells clustered apart from metastases and DCCs. Interestingly, several DCCs or metastatic cells clustered together with healthy control cells, despite harboring CNV. Lymph node-derived DCCs often appeared to reflect an evolutionary advanced stage compared to bone marrow-derived DCCs.

**Conclusion**

Primary tumours often belong to a developmental branch separate from some DCCs and later arising metastases. The occasional clustering of DCCs or metastatic cells with normal cells may reflect periods of cellular dormancy, whereas clustering of DCCs with years later arising advanced metastatic cells may identify metastasis founder cells already detectable at the time point of curative surgery.

### EACR2024-1238
### A Circulating miRNA Signature for Pancreatic Cancer Prediction and The Comprehensive Interaction Network

*C. Wu[1]*
*[1]National Chengchi University, Interdisciplinary Artificial Intelligence Center, Taipei, Taiwan*

**Introduction**

Pancreatic cancer, known for its high aggressiveness, shows improved patient prognosis with early detection. Liquid biopsies offer advantages over traditional tissue

© 2024The Authors. Molecular Oncology is published by John Wiley & Sons Ltd on behalf of Federation of European Biochemical Societies biopsies. Circulating miRNAs, being non-invasive and stable, can detect early cancer alterations before distinct biopsy or imaging evidence. There is an urgent need for a non-invasive and efficient early detection system for pancreatic cancer. Our study focuses on establishing a unique circulating miRNA signature to improve early pancreatic cancer detection via liquid biopsies and constructing a comprehensive interaction network for biological mechanism discovery and drug development.

**Material and Methods**

In this study, we analyzed miRNA expression data of pancreatic cancer and normal samples from several public databases. Differential expression analysis of miRNAs in blood and pancreatic tumor tissue was conducted, the robust and the top-ranking differentially expressed circulating miRNAs between blood from pancreatic cancer and normal blood were identified. These key miRNAs were utilized in developing a pancreatic cancer prediction system and conducting survival analysis. Additionally, we constructed comprehensive interaction networks (miRNA-target gene/ miRNA-circRNA/ miRNA-lncRNA/ miRNA-sncRNA/ miRNA-pseudogene/ miRNA-drug) and conducted functional enrichment analysis.

**Results and Discussions**

We collected and integrated miRNA expression data from pancreatic cancer and normal samples, followed by differential expression analysis of circulating miRNAs. The robust and the top-ranking differentially expressed circulating miRNAs between blood from pancreatic cancer and normal blood were identified, and some of these key miRNAs also were top-ranking differentially expressed miRNAs of pancreatic tumor tissue. These key circulating miRNAs demonstrated predictive and prognostic potential in pancreatic cancer across multiple datasets. These miRNAs improve early diagnostic efficiency and facilitate the development of a non-invasive tool. Moreover, comprehensive interaction networks (target gene/ circRNA/ lncRNA/ sncRNA/ pseudogene/ drug) for these key miRNAs were established to uncover biological mechanisms and potential therapeutic strategies.

**Conclusion**

The study identified top-ranking differential circulating miRNAs that can act as diagnostic and prognostic biomarkers. Comprehensive interaction networks of these key miRNAs were established, aiding in the development of a non-invasive tool for early pancreatic cancer diagnosis and therapeutic approaches.

### EACR2024-1244
### EFNA4-BMP4 as tumor-stroma vicious circle in osteosarcoma preclinical models

*G. Giordano*[1,2], *C. Tucciarello*[1,3], *N. Russo*[1,2], *J. Wesche*[4], *O. Myklebost*[4], *L.A. Meza-Zepeda*[4], *A. Merlini*[2], *S. Cutrupi*[3], *C. Isella*[1,2], *Y. Pignochino*[1,3]
[1]*Candiolo Cancer Institute, FPO-IRCCS, Candiolo, Italy*
[2]*Dept. of Oncology, University of Turin, Candiolo, Turin, Italy*
[3]*Dept. of Clinical and Biological Sciences, University of Turin, Orbassano, Turin, Italy*
[4]*Oslo University Hospital, Oslo, Norway*
[5]*Dept- Medical Sciences University of Turin, Turin, Italy*

**Introduction**

Advanced osteosarcoma (OS) has a poor prognosis and limited therapeutic options. OS progression depends on both intrinsic cancer traits and extrinsic stromal assets. Understanding the paracrine interactions between cancer and stroma might give rise to novel therapeutic targets.

**Material and Methods**

Human and murine sequences from RNA-Seq data of 20 OS xenografts (OXs) were analyzed to identify gene expression signatures of neoplastic and stromal origin, respectively. Stromal assets were defined as average expression of genes (metagene) typically expressed by the three main classes of stromal cells (cancer-associated fibroblasts, leucocytes, and endothelial cells) and were related to tumor growth rate (GR). Matched human-murine ligands-receptors and CAF score were correlated by Spearman rank. Candidate validation was done by in situ hybridization (ISH) and immunohistochemistry (IHC). Kaplan Meier curves showing overall survival in high-expressing and low-expressing biomarkers were generated starting from a publicly available dataset obtained from OS samples with annotated clinical features (GSE39055). Cell viability, migration, and CAF marker expression (FAP, ACTA2, and COL1A1) were studied in both normal fibroblasts (LL86, NF) and CAFs treated with tumor-conditioned medium (CM) or directly with tumor ligands, while cell viability, migration, and cancer-stem cell (CSC) marker (OCT4, NANOG, SOX2) modulation were evaluated in OS cells treated with fibroblast-CM or fibroblast-produced ligands.

**Results and Discussions**

In the total OXs, the murine stromal content ranged from 0.05- 47.34% (average 14.89%; median 12.23%). High-CAF (HC) and low-CAF (LC) content, as defined by metagene, was confirmed also at the protein level by αSMA IHC. CAF score was inversely related to GR. However, HC OS-CM activated CAF proliferation and migration, and turned NF into CAFs, increasing CAF marker expression both at mRNA and protein levels. Vice versa, fibroblast-CM induced OS cell growth and migration, and increased CSC marker expression. EFNA4, VEGFB, PDGFA-C were the tumor ligands more related to CAF content (Spearman coefficient>0.5). EFNA4 was variably expressed in an independent cohort of OS cells, but not in normal osteoblasts and normal mesenchymal cells. EFNA4 induced CAF (but not NF) proliferation, and the differentiation of NF to CAF (increased mRNA and protein CAF markers). The most CAF-score related murine receptor was Epha2 and the murine ligands were kitl, bmp4, and cxcl9. BMP4 was related to worse prognosis in GSE39055 dataset. BMP4 increased cell proliferation and migration and upregulated CSC markers in OS cells.

**Conclusion**

EFNA4-BMP4 is a crucial determinant of tumor-stroma interplay and a promising therapeutic target in OS.

### EACR2024-1262
### POSTER IN THE SPOTLIGHT
### SpottedPy quantifies relationships between spatial transcriptomic hotspots

© 2024 The Authors. Molecular Oncology is published by John Wiley & Sons Ltd on behalf of Federation of European Biochemical Societies

### and uncovers new environmental cues of epithelial-mesenchymal plasticity in cancer

E. Withnell[1], M. Secrier[1]
[1]UCL, UCL Genetics Institute- Department of Genetics-Evolution and Environment- University College London-London- WC1E 6BT- UK, London, United Kingdom

**Introduction**

The epithelial-to-mesenchymal transition (EMT) is a process in which polarised epithelial cells undergo multiple transformations to acquire a mesenchymal phenotype. The EMT is not a binary process; multiple hybrid EMT states drive the transition. How EMT arises spatially within the tumour and how this shapes the tumour microenvironment (TME) is poorly understood, but could have great therapeutic relevance.

**Material and Methods**

While a growing range of tools exist for the exploration of spatial transcriptomics data, there is a demand for specialised tools designed to visualise and quantify the cellular organisation of the tissue within biologically relevant hotspots. Analysing the tissue at different scales and within different areas of interest can provide a more comprehensive understanding of the tumour micro-environment. To address this, we have developed SpottedPy, a package designed to quantify the relationship between hotspots of user-defined cell types and signatures using spatial transcriptomics data, and compare the relationships at varying scale. We utilised SpottedPy to delineate the spatial relationships between cancer cells occupying distinct states within the EMT and hallmarks of the TME.

**Results and Discussions**

We uncovered a significant association between tumour cells undergoing EMT and the angiogenic and hypoxic regions of the tumour, along with myofibroblastic cancer-associated fibroblasts (myCAFs), macrophages and perivascular cells. Notably, EMT hotspots, regions with heightened EMT activity, showed a marked enrichment of immunosuppression and exhaustion markers compared to areas with lesser EMT activity (EMT coldspots), building upon evidence that EMT may offer crucial insights for existing strategies in immunotherapy. We observed that hybrid states demonstrate more hetero-geneous and weaker associations with the TME compared to mesenchymal states. This suggests a greater plasticity, posing challenges in defining clear relationships. Our use of graph neural networks enabled us to quantify the extent to which different states are influenced by their microenvironment.

**Conclusion**

By analysing the relationships between gene signatures and cells across a spectrum of spatial scales—from neighbourhoods to tumour hotspots— we demonstrate the usefulness of SpottedPy in unveiling the underlying biology of the TME.

### Biomarkers in Tissue and Blood

### EACR2024-0088
### Liquid Biopsy and miRNAs: an Integrated and Systematic Approach to Esophageal Tumor Biology and Monitoring

E. Chin[1], H. Liu[1,2], B.C.M. Tan[1,3]
[1]Chang Gung University, Graduate Institute of Biomedical Sciences, Taoyuan, Taiwan
[2]Chang Gung University, Department of Cell and Molecular Biology, Taoyuan, Taiwan
[3]Chang Gung University, Department of Biomedical Sciences, Taoyuan, Taiwan

**Introduction**

Esophageal squamous cell carcinoma (ESCC) ranks among the most malignant of cancers, characterized by a poor prognosis and a high mortality rate. The late diagnosis of ESCC patients, primarily due to the lack of noticeable symptoms in the early stages, exacerbates the prognosis. Liquid biopsy emerges as a particularly viable method for cancer detection, with the investigation of exosomes—small extracellular vesicles (EV) containing cell-free proteins, mRNAs, and notably non-coding RNAs like miRNAs—showing great promise as potential biomarkers for monitoring cancer progression and therapeutic outcomes.

**Material and Methods**

We conducted small RNA sequencing on plasma samples to identify miRNA biomarkers and investigated the role of candidate miRNAs through in vitro assays.

**Results and Discussions**

Our study identified one EV-miRNA candidate as a significant biomarker, showing considerable down-regulation in the plasma of ESCC patients, with this decrease becoming more pronounced as the disease progressed from early to late stages. A lower expression of this EV-miRNA was associated with poorer survival rates and increased likelihood of recurrence. To further explore this miRNA's role in ESCC, we manipulated its expression in ESCC cell lines, finding that higher levels reduced cell migration and invasion, and made cells that were resistant to cisplatin more susceptible to the drug. Additionally, we isolated EVs from the conditioned medium of cells overexpressing our EV-miRNA candidate and found that these EVs could transfer miRNA molecules to recipient cells, reducing their migratory and invasive abilities, albeit modestly. This highlights the potential of EVs to effectively deliver miRNAs to target cells, influencing their behavior.

**Conclusion**

Our research has unveiled one candidate EV-miRNA as a promising biomarker that is downregulated in ESCC, contributing to increased metastatic potential and resistance to therapy. This places EV-miRNA candidate as a tumor suppressor in the context of ESCC, offering new avenues for diagnostic and therapeutic strategies.

### EACR2024-0098
### Unravelling the Theranostics potential of microRNA in Prostate Cancer

M.K. Ahmad[1], A. Manoj[1], M. kumar[2], M.L.B. Bhatt[3]
[1]King George's Medical University, Biochemistry, Lucknow, India
[2]King Georg's Medical University, Urology, Lucknow,

© 2024The Authors. Molecular Oncology is published by John Wiley & Sons Ltd on behalf of Federation of European Biochemical Societies

India
[3]King George's Medical University, Radiotherapy, Lucknow, India

**Introduction**

Prostate cancer (PCa) is the most common malignant tumor in men and the second leading cause of cancer deaths globally. Understanding the mechanism of metastasis progression is crucial for developing anti-metastatic therapies. MicroRNA are highly conserved small non-coding RNA that affect the central dogma at the translational level. Our previously published micro-array data indicate the involvement of miRNA 145, miRNA 221, and miRNA 125b in PCa progression. (Waseem et al, 2017) Keeping this view in mind, the present study is designed to explore the biomarker as well as the therapeutic potential of miRNA 145, miRNA 221, and miRNA 125b by mimicking them in PC3 cell lines to determine their role in PCa progression and development.

**Material and Methods**

The expression pattern of miRNA in both tissue and serum samples of PCa(n=55), BPH(n=60) and was compared with healthy controls(n=30) by qPCR. ROC curve analysis was carried out to demonstrate their biomarker potential. With In-Silico analysis, we determined the target genes of each miRNA. Later, explore the therapeutic potential of each miRNA by in vitro experiments by proliferation, migration assays, and FACS.

**Results and Discussions**

The miRNA 145, miRNA 125b, and miRNA 221 are tumor suppressor miRNAs with significant down-regulation in PCa tissue and serum. The ROC of all the miRNAs indicates greater AUC with significant sensitivity and specificity with non-invasive diagnostic biomarker potential for differentiating PCa from healthy controls. The in-vitro experimental indicates the miRNA expression was enhanced up to 4 folds in the PC3 cell line which led to a delay in metastasis and proliferation of the PC3 cell line. The downregulated expression of mmp9 and mmp3 indicates the same. Further, the cell cycle arrest at the G1 phase and decreased DNA content in the S-phase along with altered gene expression of ccdn1, foxo1, and e2f3 indicate that mimic miRNAs reduce the PCa progression.

**Conclusion**

Our study suggests that with the gain of function of miRNA 145, miRNA 125b, and miRNA 221, the cell cycle could be arrested at cell-check point G1/S point which could cross-talk with an increase in apoptosis. Thus, allowing the miRNAs and their target genes to develop novel therapeutic strategies as well as act as non-invasive diagnostic biomarkers in early screening of PCa.

### EACR2024-0109

### The PALM score: a new prognostic tool for metastatic colorectal cancer patients treated with anti-angiogenic agents - a single institution experience

*E. Lai[1], E. Palmas[1], G. Papalexis[1], S. Mariani[1], P. Ziranu[1], D. Spanu[1], M. Puzzoni[1], V. Pusceddu[1], A. Pretta[1], M. Scartozzi[1]*
[1]University Hospital and University of Cagliari, Medical Oncology Unit, Cagliari, Italy

**Introduction**

Anti-angiogenic agents are a milestone for metastatic colorectal cancer (mCRC) patients (pts) treatment; however, to date, no validated prognostic biomarkers are available. We performed at our Institution a retrospective research to find a prognostic tool to be applied in clinical routine in this population.

**Material and Methods**

We retrospectively collected laboratory, radiological and clinical data of mCRC pts receiving anti-angiogenic drugs at the Medical Oncology Unit of Cagliari University Hospital (2018- 02/2024) in order to identify a potential prognostic tool. Statistical analysis was performed with MedCalc (survival distribution: Kaplan-Meier; survival comparison: log-rank test; cut-off: ROC curves; differences among variables: Chi-square test).

**Results and Discussions**

Globally, 33 mCRC pts were included in our research (19 male, 14 female; 13 RAS wild-type, 22 left-sided primary). 10 received anti-angiogenic agents in the 1st-line, 13 in the 2nd-line (bevacizumab and aflibercept) and 10 in 1st-2nd line. Median OS was 39.5 months (m) (95%CI:28.2-41.3). Pts with lower platelet to lymphocyte ratio (P; ≤253; p<0.0001, HR= 0,00000048; 95%CI 24.7-39.5 versus [vs] 95% CI 8.2-10.5), alkaline phosphatase (A; <136 U/l; p=0.0001, HR=0.0001; 95%CI 12-39.5 vs 95% CI 8.2-10.5) and lactate dehydrogenasis (L; <365 U/l; p<0.0001, HR=0.0000004; 95%CI 24.7-39.5 vs 95% CI 8.2-10.5) and higher monocyte count (M; >0.3x10**3**/µL; p=0.0093, HR 0.01; 95%CI 26.5-39.5 vs 95%CI 8.2-24.7), had a statistically improved OS. We identified the "PALM score" and separated pts in two prognostic groups: good prognostic group (0 unfavorable features: PALM=0) and poor prognostic group (≥1 unfavorable features, PALM=1). OS was significantly improved in the good prognostic group (PALM=0): 31.5 m (95%CI:24.7-39.5) vs 10.5 m (PALM=1) (p=0.0031, HR=0.00006). Chi-square test revealed also a statistically significant correlation of upfront resection of primary tumour with the PALM score (73.9% of patients belonging to the PALM=0 group had undergone surgery for primary tumour and all patients with resected primary belonged to the PALM=0 group (p=0.0352).

**Conclusion**

Our research demonstrated a promising prognostic role of the easy-to-use PALM score tool in mCRC patients receiving anti-angiogenic agents in a limited population at our center. Further prospective studies with larger sample size are needed to confirm our findings and to validate the role of the PALM score.

### EACR2024-0113

### Rapid and effective isolation of human platelets from whole blood: Maximizing purity for evaluating proteomics methods in clinical studies for cancer patients

*V. Markhus[1], D. Goplen[2], F. Selheim[3]*
[1]University of Bergen- Haukeland Hospital, Department

Molecular Oncology 18 (Suppl. 1) (2024) 1–495
© 2024 The Authors. Molecular Oncology is published by John Wiley & Sons Ltd on behalf of Federation of European Biochemical Societies of Biomedicine- Department of Oncology, Bergen, Norway
[2]Helse-Bergen- Haukeland Hospital, Department of Oncology, Bergen, Norway
[3]University of Bergen, Department of Biomedicine, Bergen, Norway

**Introduction**

Platelets are tiny, disc-shaped anucleate cells found in the blood that play a pivotal role in hemostasis and have emerged as key players in various physiological and pathological processes. To understand platelets function in cancer progression and their potential as biomarkers in cancer research and clinical studies, it is vital to gain insights into their proteomic profile. Isolating high-purity platelets from whole blood is crucial for accurate proteomic analysis. In this study, we present a rapid and effective protocol for the isolation of human platelets, maximizing purity. We evaluate this protocol using various proteomic methods, aiming to determine the most suitable proteomics pipeline for analyzing platelets in cancer patients.

**Material and Methods**

Blood samples were collected in ACD anticoagulant solution tubes to prevent platelet activation. The samples were then treated according to protocol and stored in -20 C for subsequent analysis. The platelets, red blood cells and white blood cells were counted in an automated cell counter at the laboratory clinic at Haukeland Hospital, Bergen. To validate the efficiency of the isolation protocol, we conducted proteomics analysis using Data Dependent Acquisition (DDA), Data Independent Acquisition (DIA), and Tandem Mass Tag (TMT) labeling.

**Results and Discussions**

The protocol with our optimized centrifugation time exhibited minimal contamination from other blood components. The scatter plot controls showed positive correlation with no activation of platelets. The results from DDA, DIA and TMT demonstrated a notable and novel identification of platelet-specific proteins, facilitating a more accurate and detailed characterization of the platelet proteome.

**Conclusion**

Our rapid and effective platelet isolation protocol enhances the purity of isolated platelets and demonstrates its applicability for robust downstream proteomic analyses. DIA demonstrated slightly better coverage and sensitivity in identifying platelet proteins and would be the preferable choice.

### EACR2024-0139
### LEDGF/p75 – a possible candidate for colorectal cancer classification?

*V. Liedtke[1], T. Wartmann[2], S. Wenjie[2], U. Kahlert[2]*
[1]BTU Cottbus-Senftenberg, Faculty of Environment and Natural Sciences, Senftenberg, Germany
[2]University of Magdeburg, Faculty of Medicine and University Hospital Magdeburg-Molecular and Experimental Surgery, Magdeburg, Germany

**Introduction**

Colorectal cancer ranks third in men and second in women, with 153,020 new cases and 52,550 deaths in 2023. While predominantly affecting those over 65, 13% occur in individuals under 50 years. Biomarker analysis, including APC, BRAF, KRAS, and TP53, aids early detection, but rising incidence due to lifestyle changes projects 2.2 million new cases by 2030. Early screening is crucial for treatment, highlighting the need for prognostic biomarkers.

**Material and Methods**

In this study, we analyzed the expression of the stress oncoprotein LEDGF/p75 in 15 cancer tissue sample lysates, corresponding lysates from adjacent non-tumor tissue and verified our results by LEDGF/p75 RNA expression in a cohort of > 500 patients. Additionally, we compared the obtained results to CRISPR/Cas9 generated cell lines were we established the role of LEDGF/p75 in DNA damage repair as well as the effect of complete LEDGF knockout in HEp-2, LoVo and U2OS cells.

**Results and Discussions**

LEDGF/p75 expression was significantly elevated in nearly all tumor tissue samples compared to adjacent tissue (11 / 15, 73.3 %). Additionally, the expression of the ubiquitin E2 conjugating enzyme UBC13, a key regulator in the degradation of signaling molecules, was also increased in most tumor tissue samples (8 / 15, 53.3 %). Co-overexpression of LEDGF/p75 and UBC13 was evident in 6 / 6 patients. Cell lines lacking LEDGF/p75 demonstrated reduced survival and increased chemo-sensitivity which can be reversed and even improved with LEDGF/p75 overexpression. Therefore, LEDGF/p75 seems to plays a crucial role in genomic stability, influencing DNA damage response and repair. Dys-regulation in colorectal cancer suggests its potential as a diagnostic biomarker.

**Conclusion**

Our study reveals a remarkable variation in LEDGF/p75 expression across 15 case studies, substantiated by big data transcriptome analysis of LEDGF/p75 RNA expression in over 500 patients, highlighting the role of LEDGF/p75 in maintaining genomic stability through active involvement in the DNA damage response, modulation of homology-directed repair and interaction with UBC13. Dysregulation of the DNA damage response in colorectal cancer underlines the potential of LEDGF/p75 as a diagnostic biomarker. Therefore, identification and analysis of elevated LEDGF/p75 expression represents a possibility for early detection of colorectal cancer and tailoring of personalized therapies.

### EACR2024-0156
### MeD-seq, a method for genome-wide dna methylation detection, can be used to characterize tumors, detect hpv subtypes and is compatible with liquid biopsies

*R. Boers[1,2]*
[1]Erasmus MC, Developmental Biology, Rotterdam, The Netherlands
[2]Methylomics, Methylomics, Rijswijk, The Netherlands

**Introduction**

DNA methylation serves as an important marker for mis-regulation of gene expression in cancer. DNA methylation can be utilized for cancer diagnosis, for classification and origin determination of tumors. Promising applications such as personalized medicine

© 2024 The Authors. Molecular Oncology is published by John Wiley & Sons Ltd on behalf of Federation of European Biochemical Societies include treatment outcome prediction and disease monitoring.

**Material and Methods**

We created a new, low-cost method to facilitate the genome-wide methylation profiling and the identification of new methylation markers for pre-cancer and cancer. The technique involves isolation and purification of DNA from formalin-fixed paraffin-embedded (FFPE), fresh biopsies or liquid biopsies (only 5-50ng DNA is needed). This Methylated DNA sequencing (MeD-seq) assay is very robust, allowing detection of DNA methylation at more than 50% of the 30 million CpGs present in our genome as well as detection of HPV integrated DNA. With respect to costs and sequencing depth MeD-seq is superior to all available technologies and requires no disruptive DNA bisulfite treatment.

**Results and Discussions**

We compared MeD-seq profiles of different types of cancers from vulva, cervix, endometrium, fallopian tube, and ovary between cancers vs controls and cancers vs other cancers. Identification of Differentially Methylated Regions (DMR) was achieved by comparing MeD-seq profiles with genome wide statistical testing using a sliding window approach. Data was visualized through the Integrative Genomics Viewer (IGV). In addition to DNA methylation data, MeD-seq generates sequencing data which enables the detection of Human Papilloma Virus (HPV) DNA that is incorporated in the genome of HPV-infected cells. Around half of the gynecological cancer types in our study are HPV-associated and we were able to detect HPV genomic integration and call HPV subtypes.

**Conclusion**

MeD-seq is a reliable low-cost technology to establish genome-wide DNA methylation profiles able to interrogate >50% of all the CpGs in the human genome. Its exceptional feature of requiring only a minimal amount of DNA sets it apart from other methods and makes MeD-seq seamlessly compatible with cell-free DNA/liquid biopsy samples, tumor enrichment in FFPE material by laser capture microdissection (LCM) and small populations of FACS-sorted cell types. MeD-seq can be applied to both FFPE and fresh tumor samples for tumor classification. We found that MeD-seq is also able to detect and genotype integrated HPV subtypes.

### EACR2024-0157
### The proof-of-principle of Med-seq, a method for genome-wide DNA methylation profiling for marker discovery to detect different gynecological cancers

J. Boers[1,2]

[1]*Erasmus MC, Developmental Biology, Rotterdam, The Netherlands*

[2]*Methylomics, Methylomics, Rijswijk, The Netherlands*

**Introduction**

DNA methylation acts as a crucial indicator for the mis-regulation of gene expression in cancer and can be used as a diagnostic tool. DNA-methylation markers can be utilized to diagnose cancer, categorize tumors, and monitor disease progression and therapy responses.

**Material and Methods**

We developed a novel method that that enables the genome-wide discovery of methylation markers, effectively identifying methylation changes associated with pre-cancer and cancer at very low cost. The assay involves isolation and purification of DNA from formalin-fixed paraffine-embedded (FFPE) or fresh biopsies (only 10-50ng DNA is needed). This Methylated DNA sequencing (MeD-seq) assay is very robust, allowing detection of DNA methylation at more than 50% of the 30 million CpGs present in our genome. With respect to costs and sequencing depth MeD-seq is superior to all available technologies and requires no DNA bisulphite treatment. MeD-seq is compatible with low amounts of DNA derived from solid tumor tissue enriched by laser capture microdissection (LCM) and liquid biopsies.

**Results and Discussions**

We compared MeD-seq profiles of different types of cancers from vulva, cervix, endometrium, fallopian tube and ovary between cancers vs controls and cancers vs other cancers. Identification of Differentially Methylated regions (DMR) was achieved by comparing MeD-seq profiles using genome wide statistical testing using a sliding window approach, visualized through the Integrative GenomicsViewer (IGV) and subsequent identification of primer and probe regions for quantitative Methylation-specific PCRs (qMSP) to detect tumor-specific or general-tumor markers. Our proof of principle led to four novel marker regions able to detect all gynecological cancers.

**Conclusion**

MeD-seq is a reliable low-cost technology to establish genome-wide DNA methylation differences between cancer and controls and can be used to call DMRs for the rapid development of PCR-based assays.

### EACR2024-0174
### COX-2-associated inflammation as a relapse biomarker for early-stage lung cancer

V. Fife[1], M. Roberts[2], A. Chaturvedi[3], D. Millrine[1], C. Swanton and The TRACERx Consortium[4], E. Kilgour[1], L.M. Coussens[5], P.A.J. Crosbie[6], C. Dive[7], S. Zelenay[8]

[1]*Cancer Research UK National Biomarker Centre, Translational Immunology, Manchester, United Kingdom*

[2]*Cancer Research UK National Biomarker Centre, Bioinformatics and Biostatistics, Manchester, United Kingdom*

[3]*The Christie Hospital, Histopathology, Manchester, United Kingdom*

[4]*University College London Cancer Institute, Cancer Evolution and Genome Instability Laboratory, London, United Kingdom*

[5]*Oregon Health and Science University Knight Cancer Institute, Department of Cell- Developmental & Cancer Biology, Portland- Oregon, United States*

[6]*University of Manchester, Division of Infection-Immunity and Respiratory Medicine, Manchester, United Kingdom*

[7]*Cancer Research UK National Biomarker Centre, Director, Manchester, United Kingdom*

[8]*Cancer Research UK Manchester Institute, Cancer*

© 2024 The Authors. Molecular Oncology is published by John Wiley & Sons Ltd on behalf of Federation of European Biochemical Societies

*Inflammation and Immunity, Manchester, United Kingdom*

**Introduction**

Approximately 30% of patients with early-stage (stages I-IIIA) non-small cell lung cancer (NSCLC) relapse after surgery with curative intent. Biomarkers to predict relapse risk are thus of great interest. Our research identified the COX-2/$PGE_2$ inflammatory axis as a critical determinant of malignant tumour progression and immune escape. We hypothesise that a COX-2 associated pro-tumorigenic inflammatory signature (PTI) may provide a relapse-prediction biomarker for early-stage lung adeno- (LUAD) and lung squamous carcinoma (LUSC) patients. We asked whether the PTI signature, composed of extracellular mediators selectively upregulated in pan-cancer COX-$2^{high}$ tumours, predicts disease relapse and overall survival (OS) following surgery.

**Material and Methods**

Gene expression analysis was performed on two early-stage NSCLC RNA-seq datasets: The Cancer Genome Atlas (TCGA, LUAD n=327, LUSC n=299) and TRACERx (LUAD n=155, LUSC n=98). An in-house cohort (LUAD n=51, LUSC n=50) was analysed with a method compatible with formalin-fixed-paraffin embedded (FFPE) tissue (the routine clinical sample format). RNA was extracted from up to 10 tissue sections marked up by a pathologist. The PTI was benchmarked against a published T cell-inflamed signature (TIS) measuring IFNγ activity and correlations sought with patient outcome data.

**Results and Discussions**

Survival analysis revealed marked association between intra-tumour PTI level and OS in TCGA (LUAD HR=1.8, p=0.02, LUSC HR=1.6, p=0.03) and TRACERx (LUAD HR=1.9, p=0.02, LUSC HR=2.2, p=0.02) datasets. In multivariate analysis, PTI predicted disease free survival independent of age, stage, sex, performance status or adjuvant treatment (LUAD HR=1.5, p=0.04, LUSC HR=1.9, p=0.004). In contrast, TIS, generally considered a feature of immune "hot" tumours, did not correlate significantly with clinical outcomes. Patients with stage I $PTI^{high}$ tumours had a poorer outcome that was comparable to stage IIIA and in stage I disease PTI was particularly prognostic within a year after surgery (LUAD AUC=0.87, LUSC AUC=0.81). Molecular profiling of in-house FFPE surgical specimens confirmed PTI prognostic utility in early-stage NSCLC (LUAD HR=1.8, p=0.04 and LUSC HR=1.6, p=0.02).

**Conclusion**

We uncovered a critical link between intra-tumour inflammation and outcomes of patients with early-stage LUAD and LUSC. PTI scores were powerful in predicting outcomes within the first year after surgery implying that, with further validation, PTI holds potential to inform patient management.

### EACR2024-0186
### Extracellular vesicle-encapsulated non-coding RNAs as functional and clinical biomarkers in non-smoker Asian females with early-stage lung adenocarcinoma

*Y.Y. Lai*[1], *H. Liu*[1,2]
[1]*Chang Gung University, Graduate Institute of Biomedical Sciences, Taoyuan, Taiwan*
[2]*Chang Gung University, Department of Cell and Molecular Biology, Taoyuan, Taiwan*

**Introduction**

Early detection and treatment of lung cancer can significantly improve the five-year survival rate, potentially reaching up to 80%. In Taiwan, a notable proportion of female lung adenocarcinoma patients are non-smokers. Liquid biopsies, particularly through the analysis of extracellular vesicles (EVs), have emerged as a promising tool for early cancer detection, monitoring treatment efficacy, and identifying recurrence, representing a forefront area in precision medicine. This study focuses on the utilization of EVs from liquid biopsies to detect early-stage lung adenocarcinoma in non-smoking females.

**Material and Methods**

We established EV-miRome profiles for both non-smoking females with early-stage lung adenocarcinoma and healthy non-smoking females. Our approach involved a systematic search for effective biomarker combinations for detection and prognosis. We also investigated the mechanistic roles of these biomarkers in carcinogenesis through in vitro assays.

**Results and Discussions**

Our research identified 63 differentially expressed extracellular microRNAs (EV-miRs), among which 9 demonstrated promising potential for early detection. Bioinformatics analyses revealed that these EV-miRs target pathways involved in epithelial-mesenchymal transition (EMT) and the dynamics of the tumor micro-environment. EVs secreted during EMT were found to inhibit cancer cell proliferation while enhancing mobility, invasiveness, and drug resistance. Further analysis of two selected EV-miR candidates showed their ability to promote EMT and increase drug resistance in lung cancer cells. Additionally, RNA sequencing was employed to map the target gene networks of these EV-miRs, revealing intricate regulatory networks between EV-miRs and mRNAs.

**Conclusion**

The lung cancer liquid biopsy platform developed in this study offers a comprehensive overview of the circulating EV-miRome and its functional roles in carcinogenesis. This contributes significantly to the identification of clinically relevant and highly applicable biomarkers for early-stage lung adenocarcinoma in non-smoking females.

### EACR2024-0238
### Identification of novel exosomal miRNAs and their prognostic significance in Triple Negative Breast Cancer

*A. Choudhary*[1], *B. Das*[1]
[1]*Amity University, Amity Institute of Molecular Medicine and Stem Cell Research, Gautam Buddh Nagar, India*

**Introduction**

Triple-negative breast cancer (TNBC) is a highly aggressive subtype of breast cancer and shows the worst prognosis among all breast cancer types. TNBCs are also characterized by the presence of treatment-resistant

© 2024 The Authors. Molecular Oncology is published by John Wiley & Sons Ltd on behalf of Federation of European Biochemical Societies breast cancer stem cells (CSCs) that allow disease to relapse. It is crucial to identify specific therapeutic targets of TNBC to develop more effective treatment strategies. Currently, there is no specific universal biomarker which may be used to diagnose TNBC. Through our study, we have investigated the clinical utility of exosomal miRNAs as non-invasive prognostic indicators of TNBC.

**Material and Methods**

After a comprehensive literature survey of global databases (Web of Science, Cochrane Library, Pubmed) and subsequent meta analysis on GEO datasets, we shortlisted a set of five miRNAs expressed in breast cancers. Overall survival analysis indicated these miRNAs were associated with poor prognosis of disease. Target prediction studies, GO and KEGG enrichment analyses outlined the role of these oncomiRs in several cancer signaling pathways such as Wnt, Notch etc involved in tumor progression. All miRNAs were consistently expressed in TNBC and CSCs and their secretory nature was revealed when we detected their presence in TNBC derived exosomes by RT-qPCR. Upon clinical correlation in patient samples (n=15), hsa-miR-1180 and hsa-miR-4728 were found significantly upregulated. We used miRNA inhibitors for these oncomiRs and subsequent functional assays were conducted to determine their role in TNBC progression.

**Results and Discussions**

We identified a set of five novel secretory oncomiRs (hsa-miR 6803, hsa-miR 1180, hsa-miR 4728, hsa-miR 1915 and hsa-miR 940) in TNBC and TNBC stem cells (TNBCSC) which demonstrate great prognostic potential. Significant overexpression of two miRNAs namely: hsa-miR 1180 and hsa-miR 4728 was found in clinical samples. High expression of these miRNAs was correlated with poor disease prognosis and OS. Post miRNA knockdown, in-vitro functional assays highlighted the role of these miRNAs in proliferation, invasion and migration of TNBC.

**Conclusion**

The identification of novel secretory miRNAs in TNBC and TNBCSCs is promising with potential application in diagnosis and treatment of highly metastatic breast cancers. Our study highlights their utility as therapeutic targets for TNBC. These miRNAs could serve as a liquid biopsy tool for early detection and diagnosis and routinely monitoring TNBC prognosis.

### EACR2024-0246
### Fecal snoRNAs as novel non-invasive biomarkers for colorectal cancer detection

*J. Gómez-Matas[1], S. Duran-Sanchon[1], J.J. Lozano[2], C. Di Battista[3], G. Ferrero[4], S. Tarallo[5], B. Pardini[5], A. Naccarati[5], A. Castells[1], M. Gironella[1]*
*[1]Hospital Clinic of Barcelona. IDIBAPS. Centro Investig ación Biomédica en Red CIBER, Gastrointestinal & Pancreatic Oncology Group, Barcelona, Spain*
*[2]Centro de Investigación Biomédica en Red de Enfermedades Hepáticas y Digestivas CIBERehd, Bioinformatics platform, Barcelona, Spain*
*[3]Hospital Clinic of Barcelona. IDIBAPS. Centro Investigación Biomédica en Red CIBER, Gastro-intestinal & Pancreatic Oncology, Barcelona, Spain*
*[4]University of Turin, Department of Clinical and Biological Sciences, Turin, Italy*
*[5]Italian Institute for Genomic Medicine- Italian Institute for Genomic Medicine, FPO Istituto di Ricovero e Cura a Carattere Scientifico, Candiolo, Italy*

**Introduction**

Small nucleolar RNAs (snoRNAs) are a class of small noncoding RNAs which are in charge of post-transcriptional modifications of rRNA, but other roles have been recently described: regulation of alternative splicing of genes or regulation of mRNA abundance. SnoRNAs have been identified dysregulated in several pathologies, and these alterations can be detected in tissues and in circulation. The main aim of this study was to analyze the whole snoRNome in advanced colorectal neoplasms (including advanced adenomas [AA] and colorectal cancer [CRC]) and to identify new potential non-invasive snoRNA-based biomarkers in fecal samples by different analytical approaches.

**Material and Methods**

In total, 521 samples were analyzed in this study. 88 paired advanced colorectal neoplasia and healthy adjacent tissues obtained from 44 patients (23 CRC, 21 AA) from a Spanish cohort were analyzed by snoRNA-sequencing. Significantly altered snoRNA profiles in CRC and AA were validated in an independent Italian set of 262 matched colonic neoplastic tissues from CRC (105) and AA (26) patients. These results were validated in a subset of colorectal tissues (60) by RT-qPCR. SnoRNA candidates were analyzed in a set of fecal samples from positive fecal immunochemical test individuals (FIT+) from the Barcelona CRC screening program (171) by droplet digital PCR.

**Results and Discussions**

SNORA51, SNORD15B, SNORA54, SNORD12B, SNORD12C, SNORD72, SNORD89, and several members of SNORD115 and SNORD116 clusters were consistently deregulated in both tissue sets. After technical validation, SNORA51 and SNORD15B were detected in FIT+ leftover samples by ddPCR. SNORA51 was significantly upregulated in FIT+ samples from CRC patients compared to healthy controls. This upregulation, together with fecal hemoglobin concentration was sufficient to discriminate, among FIT+ individuals, patients with CRC (AUC = 0.86) or AA (AUC = 0.68) between healthy individuals.

**Conclusion**

These findings portray snoRNAs as an alternative source of biomarker candidates for further studies and fecal SNORA51 appears as a promising non-invasive biomarker for CRC detection.

### EACR2024-0290
### Mass spectrometry-based proteomics uncover novel plasma biomarkers for colorectal cancer associated with inflammation

*V. Urbiola-Salvador[1], A. Jabłońska[1], D. Miroszewska[1], R. Baber[2,3], I. Gockel[4], P. Czaplewska[5], N. Filipowicz[6],*

Molecular Oncology 18 (Suppl. 1) (2024) 1–495
© 2024 The Authors. Molecular Oncology is published by John Wiley & Sons Ltd on behalf of Federation of European Biochemical Societies

*A. Piotrowski[6], J.P. Dumanski[7,8,9], Z. Chen[1,10]*
*[1]University of Gdańsk, Intercollegiate Faculty of Biotechnology of University of Gdańsk and Medical University of Gdańsk, Gdańsk, Poland*
*[2]Universitätsklinikum Leipzig, Institute of Laboratory Medicine, Clinical Chemistry and Molecular Diagnostics Leipzig, Germany*
*[3]Universitätsklinikum Leipzig, Leipzig Medical Biobank, Leipzig, Germany*
*[4]University Hospital Leipzig, Department of Visceral-Transplant- Thoracic and Vascular Surgery, Leipzig, Germany*
*[5]Intercollegiate Faculty of Biotechnology University of Gdańsk and Medical University of Gdańsk- University of Gdańsk, Laboratory of Mass Spectrometry-Core Facility Laboratories, Gdańsk, Poland*
*[6]Medical University of Gdańsk, 3P-Medicine Laboratory, Gdańsk, Poland*
*[7]3P-Medicine Laboratory, Medical University of Gdańsk, Gdańsk, Poland*
*[8]Medical University of Gdańsk, Department of Biology and Pharmaceutical Botany, Gdańsk, Poland*
*[9]Uppsala University, Department of Immunology-Genetics and Pathology and Science for Life Laboratory, Uppsala, Sweden*
*[10]University of Oulu, Faculty of Biochemistry and Molecular Medicine, Oulu, Finland*

**Introduction**

Colorectal cancer (CRC) is the third most incident malignancy and the second most lethal cancer, worldwide. CRC prognosis depends on the tumor stage as well as other factors such as location, genetic factors, and inflammation among others. Currently, CRC prevention screening uses colonoscopy with high cost, invasiveness risks, and poor patient compliance. Therefore, alternative non-invasive, cost-effective, accurate, and easily measurable CRC screening strategies are urgently required.

**Material and Methods**

We applied proteomics analysis by using tandem mass spectrometry coupled with liquid chromatography (LC-MS/MS) to plasma samples from 36 CRC patients and 26 healthy subjects to determine the plasma protein changes involved in CRC development, progression, and cancerassociated inflammation.

**Results and Discussions**

LC-MS/MS analysis identified 322 proteins associated with multiple biological processes, from which, 139 proteins were quantified across the samples. Statistical analysis revealed 37 differentially expressed proteins (DEPs) between CRC patients and healthy subjects. These DEPs were involved in the complement cascade, cholesterol metabolism and included several members from the SERPIN family. Moreover, several protein plasma levels were linked to cancer-associated inflammation. We also found several novel potential biomarkers to differentiate early and late CRC stages.

**Conclusion**

Our study unveils novel potential biomarkers of CRC development such as members from the complement cascade and proteins related to pro-inflammatory conditions. Additionally, we identified novel proteins related to cancer-associated inflammation and a signature that may be associated with CRC progression. The application of these novel biomarkers in clinics could improve patient care.

### EACR2024-0291
### Colorectal cancer development-associated changes of plasma proteins levels

*D. Miroszewska[1], V. Urbiola-Salvador[1], A. Jabłońska[1], Q. Huang[2], N. Filipowicz[3], A. Piotrowski[3], J.P. Dumański[3,4], B. Li[2], Z. Chen[1,5], J. Ryś[6]*
*[1]University of Gdańsk, Intercollegiate Faculty of Biotechnology of University of Gdańsk and Medical University of Gdańsk, Gdańsk, Poland*
*[2]Center for Immune-Related Diseases at Shanghai Institute of Immunology, Department of Respiratory and Critical Care Medicine of Ruijin Hospital- Department of Immunology and Microbiology- Shanghai Jiao Tong University School of Medicine, Shanghai, China*
*[3]Medical University of Gdańsk, 3P-Medicine Laboratory, Gdańsk, Poland*
*[4]Medical University of Gdańsk, Department of Biology and Pharmaceutical Botany, Gdańsk, Poland*
*[5]University of Oulu, Faculty of Biochemistry and Molecular Medicine, Oulu, Finland*
*[6]Maria Skłodowska-Curie National Research Institute of Oncology, Department of Tumor Pathology, Kraków, Poland*

**Introduction**

Colorectal cancer (CRC) is the second leading cause of cancer-related death and third mostly common malignancy worldwide. Usually, the diagnosis of the patient occurs at more advance stage of the disease as early symptoms are non-specific and are often assigned to common diseases. Therefore there is an urgent need for the discovery of blood biomarkers that would facilitate early diagnostics of the disease. One of the most resent proteomics technologies Proximity Extension Assay (PEA) combines DNA-labelled antibodies and next generation sequencing for quantification of thousands of proteins from a low amount of sample.

**Material and Methods**

For the identification of novel biomarkers of CRC, 690 plasma proteins from 38 CRC patients and 38 healthy controls were quantified using Proximity Extension Assay (PEA)

**Results and Discussions**

Levels of 202 plasma proteins were significantly altered between CRC patients and healthy subjects. Several novel proteins changes linked to the Th17 activity, cancer-related inflammation, and oncogenic pathways were found with potential application in CRC diagnosis. Interferon γ (IFNG), interleukin (IL) 32, and IL17C proteins were found to be associated with the early stage of the disease whilst lysophosphatidic acid phosphatase type 6 (ACP6), Fms-related tyrosine kinase 4 (FLT4), and MANSC domain-containing protein 1 (MANSC1) correlated with the late-stage.

**Conclusion**

The identified altered plasma proteins might serve as potential novel biomarkers to distinguish the state of the cancer progression in patients, however further studies in larger cohort is needed.

© 2024 The Authors. Molecular Oncology is published by John Wiley & Sons Ltd on behalf of Federation of European Biochemical Societies

### EACR2024-0326

### Utilizing Deep Learning for the Early Detection of Multiple Cancers through Minimal cfDNA Quantities

*A. Danilevsky*[1], *I. Margolin*[2], *B. Braun*[3], *S. Ben Asher*[2], *M. Grad*[3], *H. Volkov*[3], *N. Moskovits*[4], *S.M. Stemmer*[4], *N. Shomron*[1]

[1]*Tel Aviv University, Faculty of Medical and Health Sciences and Edmond J. Safra Center for Bioinformatics, Tel Aviv, Israel*

[2]*Tel Aviv University, School of Electrical Engineering, Tel Aviv, Israel*

[3]*Tel aviv university, Faculty of Medical and Health Sciences, Tel Aviv, Israel*

[4]*Rabin Medical Center- Beilinson Campus, Felsenstein Medical Research Center and Davidoff Center, Petah Tikva, Israel*

**Introduction**

Cell-free DNA (cfDNA) holds promise for early cancer detection, but distinguishing tumor-derived cfDNA in a normal background is challenging without complex sequencing or reference samples. Our hypothesis posits that advanced deep neural networks can surmount this obstacle by directly learning intricate cancer-specific patterns from raw sequencing data.

**Material and Methods**

We developed a deep learning platform and applied it to multiple clinical datasets containing raw genomic sequencing data from cancer patients and healthy individuals, including sequencing data generated by our team. We assessed the performance of our deep learning models in classifying multiple types of cancer simultaneously and discretely. The models were evaluated through leave-one-out cross-validation method and were further tested on down-sampled sequencing samples, with the lowest sampling of 75,000 reads per sample (coverage < 0.01x). We compared our cancer detection performance to traditional cfDNA cancer detection methodologies.

**Results and Discussions**

Our deep learning model achieved 80% sensitivity at 85% specificity in multi-cancer classification, which improved from sensitivity of 68% with multi-cancer model to 86% for cancer-specific model (breast cancer). Interestingly, our classification predictions per-sample were significantly correlated with the prediction scores of traditional cancer detection methods (Pearson correlation, r=0.53; $P<1.1\times10^{-8}$). Notably, while traditional cancer detection methods show a dramatic drop in performance with lower sequencing coverage, our method shows similar cancer detection ability across all tested depths (50Mil, 5Mil, 100K).

**Conclusion**

Deep learning shows potential for direct analysis of raw genomic data. With sufficient training data, optimized models may approach accuracies needed for clinical use. Our results demonstrate the feasibility of early cancer detection in cfDNA, specifically with an extremely low amount of sequencing data which enables employing this technique for a wide-scale screening. Ongoing work includes expanding the training data to nanopore sequencing, adding biologically relevant annotations and applying advanced natural language processing techniques (such as transformers and BERT architectures).

### EACR2024-0332

### miR-30e-3p/CXCL3 axis predicts early tumor escape in sorafenib-treated HCC patients

*C. Vianello*[1,2], *I. Leoni*[1,2], *G. Galvani*[1,2], *E. Monti*[1,2], *G. Marisi*[3], *A. Casadei Gardini*[4], *G.F. Foschi*[5], *C. Stefanelli*[1], *L. Gramantieri*[6], *F. Fornari*[1,2]

[1]*University of Bologna, Department for Life Quality Studies, Rimini, Italy*

[2]*University of Bologna, Centre for Applied Biomedical Research - CRBA, Bologna, Italy*

[3]*Istituto di Ricovero e Cura a Carattere Scientifico di Natura Pubblica- Istituto Romagnolo per lo Studio dei Tumori "Dino Amadori", Biosciences Laboratory, Meldola, Italy*

[4]*Vita-Salute San Raffaele University- IRCCS San Raffaele Scientific Institute Hospital, Department of Oncology, Milano, Italy*

[5]*Degli Infermi Hospital-AUSL Romagna, Department of Internal Medicine, Faenza, Italy*

[6]*IRCCS Azienda Ospedaliero-Universitaria di Bologna, Division of Internal Medicine- Hepatobiliary and Immunoallergic Diseases, Bologna, Italy*

**Introduction**

Curative treatment options for hepatocellular carcinoma (HCC) remain largely limited to a minority of patients identified at an early stage of the pathology. Immuno-therapy and Tyrosine Kinase Inhibitors (sorafenib and lenvatinib) represent the first-line treatments in advanced cases. Despite immunotherapy has revolutionized HCC treatment, only a minority of patients show a prolonged response to systemic treatments. The identification of biomarkers predictive of drug response or early tumor escape remains an unsolved clinical need. MicroRNAs and chemokines are pivotal players in the progression and the development of drug resistance of HCC. We previously reported higher miR-30e-3p levels in non-responder patients undergoing sorafenib treatment.

**Material and Methods**

Serum and tissue miR-30e-3p and CXCL3 levels were analyzed by microarray and qPCR analysis in HCC patients and DEN-HCC rats. To assess CXCL3 targeting by miR-30e-3p in HCC cell lines we performed functional analysis and luciferase reporter assay. The contribution of CXLC3 to sorafenib response was evaluated in the DEN-HCC rat model by analyzing the expression of apoptotic markers. ELISA assay evaluated serum CXCL3 levels in sorafenib-treated HCC patients. Statistical analysis was performed to evaluate clinicopathological associations.

**Results and Discussions**

CXCL3 is upregulated in human and rat HCCs and show a direct correlation with its receptor CXCR2 and a negative one with miR-30e-3p. Functional analyses demonstrated CXCL3 targeting by miR-30e-3p in HCC cell lines, as confirmed by a luciferase reporter assay. In the HCC rat model, higher CXCL3 tissue levels correlated with sorafenib resistance. In line, a negative correlation was detected between CXCL3 and apoptotic markers and a positive one with tumor size. Higher

© 2024 The Authors. Molecular Oncology is published by John Wiley & Sons Ltd on behalf of Federation of European Biochemical Societies

CXCL3 and miR-30e-3p circulating levels were observed in blood samples of non-responder patients collected at the two-month of follow-up. Higher CXCL3 basal levels were detected in patients with microvascular whereas circulating miR-30e-3p levels correlated with alfa-fetoprotein. CXCL3 and miR-30e-3p are promising predictive candidates as suggested by a preliminary ROC curve analysis showing an AUC of 0.777 and 0.712 (p=0.026), respectively.

**Conclusion**

CXCL3 is a novel target of miR-30e-3p in HCC and is involved in sorafenib resistance. CXCL3 and miR-30e-3p are promising circulating biomarkers of early tumor escape in sorafenib-treated HCCs.

### EACR2024-0350

### Genome wide copy number alteration in plasma of patients with high-grade serous ovarian cancer is an independent prognostic marker of relapse: the MITO-16a/MANGO-OV2a/ clinical trial experience

*S. Marchini[1], L. Paracchini[2], L. Arenare[3], L. Mannarino[2], A. Ferrero[4], P. Chiodini[5], D. Califano[6], T. Elisa[2], S. Pignata[7], M. D'Incalci[8]*

*[1]Humanitas Mirasole S.p.A., Cancer Pharmacology, Pieve Emanuele, Italy*

*[2]Humanitas Research Hospital, Cancer Pharmacology, Pieve Emanuele, Italy*

*[3]Istituto Nazionale Tumori- IRCCS- Fondazione G. Pascale, Clinical Trial Unit, Naples, Italy*

*[4]Mauriziano Hospital, University of Turin, Turin, Italy*

*[5]University of Campania "Luigi Vanvitelli", Medical Statistic Unit, Naples, Italy*

*[6]Istituto Nazionale Tumori IRCCS e Fondazione G. Pascale, Functional Genomic, Naples, Italy*

*[7]Istituto Nazionale Tumori- IRCCS- Fondazione G. Pascale-, Medical Oncology Division/Department of uro-gynaecology, Naples, Italy*

*[8]Humanitas Research Hospita, Cancer Pharmacology, Pieve Emanuele, Italy*

**Introduction**

Although 80% of high grade serous ovarian cancer (HGSOC) patients are initially responsive to platinum (Pt)-based chemotherapy, the majority of them experience relapse with a progressive Pt-resistant disease. One of the most challenging issue that hampers the possibility to effectively treat relapsed disease is the lack of biological information about tumor recurrence. To date, the Pt-free interval (PFI), an empirical measure of the time lagging between the end of front-line chemo-therapy and relapse, is the only and widely accepted parameter to predict patient outcome and sensitivity to Pt second-line chemotherapy. We have previously developed an "agnostic-tumor platform" for circulating tumor DNA (ctDNA) analysis which resulted a suitable tool to predict patient outcome and to intercept molecular relapsed (Paracchini et al., PMID:33323403). In the present study, we quantified the fraction of tumor DNA (TF) in plasma of patients enrolled within the frame of a phase III clinical trial (MITO-16A/MaNGO-OV2A-EUDRACT number: NCT01706120) to demonstrate, at baseline, the prognostic value of untargeted ctDNA analysis.

**Material and Methods**

172 eligible patients enrolled with the frame of the MITO-16A/MaNGO-OV2A clinical trial, for whom plasma sample was collected at surgery, before chemo-therapy, were selected for the study. For each plasma sample, circulating-free DNA (cfDNA) was purified and whole genome libraries (HyperPlus, Roche) sequenced (mean coverage 0.5X). ichorCN algorithm was used to infer the percentage of TF, as previously published (Paracchini et al . PMID:33323403).

**Results and Discussions**

In 168 out of 172 plasma samples analyzed, the TF was detectable and it ranged from 2.4% to 47%. Cox-model univariate analysis with patients' overall survival (OS) and Progression-Free Survival (PFS), indicates association of TF levels with both OS and PFS ($p<0.001$). The prognostic relevance of TF was also confirmed in multivariate analysis, where, considering CA-125, residual tumor, stage of disease, histology and age as co-variables, TF maintained its statistical significance in PFS (HR = 2.1, CI 95%, p-value < 0.003).

**Conclusion**

Retrospective analysis of baseline plasma samples collected within the frame of the MITO-16A/MaNGO-OV2A clinical trial, demonstrated that the percentage of TF is an independent prognostic marker of relapse. This finding confirms the importance of liquid biopsy analysis based on untargeted sWGS tool as an innovative approach to predict response to front line therapy and patient's outcome.

### EACR2024-0431

### A metastasis-stemness associated prognostic signature for improving clinical management of prostate cancer

*A. Sabater[1], P. Sanchis[1], G. Pascual[1], R. Seniuk[1], E. Vazquez[1], J. Cotignola[1], A. Toro[1], G. Gueron[1], J. Bizzotto[1]*

*[1]University of Buenos Aires, Biological Chemistry, Buenos Aires, Argentina*

**Introduction**

Prostate cancer (PCa) represents a predominant malignancy among males worldwide, with survival rates plummeting to 30% upon aggressive progression. Cancer stem cells are implicated in metastasis and recurrence, making them potential targets for prognostic biomarkers. This study aims to refine the clinical management of prostate cancer through a novel prognostic signature derived from metastasis and stemness-associated genes that are dysregulated upon aggressive disease, enabling personalized treatment strategies.

**Material and Methods**

We performed bioinformatic analyses on 11 transcriptomics datasets (n=1259) from PCa patients, assessing 144 genes linked to metastasis, stemness, and the STAT3 pathway. These datasets include comparisons between tumors, metastases, and normal/adjacent tissues. We integrated these findings with clinical data from public repositories, involving 9 datasets (n=1311), to analyze the genes' associations with PCa progression events, including mortality, recurrence and metastasis, by

© 2024 The Authors. Molecular Oncology is published by John Wiley & Sons Ltd on behalf of Federation of European Biochemical Societies uni and multivariable analyses. The Random Forest algorithm guided the selection of critical genes according to their relative relevance in predicting the events, while the Lasso algorithm helped develop a prognostic signature, which was subsequently validated, also including 4 additional independent datasets (n=415).

**Results and Discussions**

The analysis identified 14 genes with increased expression and 23 with decreased expression across multiple datasets (at least in 6 of the analyzed comparisons). Survival analyses pinpointed genes significantly associated with PCa progression, independently of clinicopathological factors, like the Gleason Score and PSA levels. The Random Forest machine learning algorithm highlighted 15 key genes, from which the Lasso algorithm employed extracted a prognostic signature comprising 4 stemness-associated genes (*TYMS*, *DPP4*, *KMT5C*, and *TAF5L*). This signature was validated and compared with existing commercial prognostic genesets, underscoring the prognostic potential of stemness-associated genes in clinical decision-making.

**Conclusion**

Our comprehensive transcriptomics investigation has unveiled a robust prognostic signature tied to PCa progression. By correlating transcriptomic profiles with clinical outcomes, we propose a foundation for novel therapeutic interventions and molecular predictive tools centered on the tumor's stemness characteristics.

### EACR2024-0493
### Kinase activity: a diagnostic tool for prostate cancer?

*E. Lishman-Walker[1], J. Dobson-Smith[1], H.X. Choo[1], L. Wilson[1], B. Hunter[2], G. Merces[3], C. Robson[1], S. McCracken[1], A. Filby[2], K. Coffey[1]*

*[1]Newcastle University, Newcastle University Centre for Cancer, Newcastle, United Kingdom*

*[2]Newcastle University, Flow Cytometry Core Facility, Newcastle, United Kingdom*

*[3]Newcastle University, Image Analysis Unit, Newcastle, United Kingdom*

**Introduction**

Normal prostate development and function relies on androgen receptor (AR) signalling, this is de-regulated in prostate cancer (PC), therefore current treatments target this pathway. Androgen deprivation therapy may initially be effective, but resistance commonly arises, highlighting a need for alternative therapies. Post-translational modifications such as phosphorylation by protein kinases can regulate AR activity. We hypothesise that inhibiting such kinases will enable indirect regulation of AR signalling, offering a new therapeutic option.

**Material and Methods**

A kinome-wide siRNA screen was performed to prioritise 7 kinases that modulate AR activity. To investigate kinase activity and AR signalling in PC an imaging mass cytometry (IMC) panel was developed. Our IMC analysis pipeline allows us to quantify each marker at the single cell level. Furthermore, we developed an ex vivo tissue slice model to culture patient tissue in the presence of kinase inhibitors. Using IMC, we can compare patient tissue pre- and post-treatment to understand the impact upon kinase activity. Finally, we have generated cell line (CMA) and prostate tumour tissue microarrays (TMA) to evaluate kinase signalling in PC.

**Results and Discussions**

We used our CMA (369 cores) which included siRNA knockdowns of 7 kinases in 6 PC cell lines, kinase inhibitors and anti-androgens, to validate IMC antibody specificity and characterise kinase activity biomarkers and AR-kinase crosstalk. Applying IMC to our TMA, 100% of cores showed expression of >2 kinases, and 94% showed >1 active kinase. We observed significant differences in intensity for 60% of markers between benign prostate and PC samples. In a pre-treatment *ex vivo* slice 16/25 markers showed changes in intensity between cancerous and adjacent benign tissue with 3 kinases selectively active in the tumour. Finally, using IMC spatial information we characterised cell neighbourhoods and identified cell-state interactions differ between benign and cancerous prostate tissue.

**Conclusion**

We present a novel approach to study kinase signalling in PC. Our data shows kinase expression is heterogenous in localised PC, and may not correlate with kinase activity confirming the need for biomarkers to evaluate suitability of kinase inhibitor treatment. We have developed resources to further investigate this in local and advanced PC to evaluate where within the treatment pathway kinase inhibitors will provide most benefit to patients. This will help us develop a diagnostic tool to deliver personalised medicine.

### EACR2024-0561
### Characterization of PARP-inhibitor therapy resistance hallmarks in blood cell-free DNA from ovarian cancer patients

*T. Senturk Kirmizitas[1,2], C. Van Den Berg[3], R. Broers[4], W. Van Eijcken[5], J. Helmijr[1], T. Gunel[2], S. Wilting[1], J. Martens[1], M. Jansen[1], I. Broere[1]*

*[1]Erasmus Medical Center, Medical Oncology and Cancer Genomics, Rotterdam, The Netherlands*

*[2]Istanbul University, Molecular Biology & Genetics, Istanbul, Turkiye*

*[3]Erasmus Medical Center, Gynecological Oncology, Rotterdam, The Netherlands*

*[4]Erasmus Medical Center, Developmental Biology, Rotterdam, The Netherlands*

*[5]Erasmus Medical Center, Cell Biology, Rotterdam, The Netherlands*

**Introduction**

Patients with epithelial ovarian cancer (EOC) receive PARP-inhibitor (PARPi) maintenance therapy after adjuvant chemotherapy, yet half of these patients acquire therapy resistance. This study aims to characterize epigenetic and genomic changes in cell-free DNA (cfDNA) associated with PARPi resistance.

**Material and Methods**

Blood was drawn from 31 EOC patients receiving PARPi therapy at two timepoints: At baseline before treatment and at disease progression after treatment. The cohort included 14 patients resistant within 6 months PARPi

Molecular Oncology 18 (Suppl. 1) (2024) 1–495

© 2024 The Authors. Molecular Oncology is published by John Wiley & Sons Ltd on behalf of Federation of European Biochemical Societies treatment and 17 patients responding for 6.1 to 44 months. Eight healthy blood donors (HBDs) and 10 EOC patients with tumor tissue and treatment-naïve blood were used as references to distinguish disease (blood) and tumor-specific (tissue) cfDNA alterations. The cfDNA and DNA from all blood and tumor tissue samples were evaluated by MeDseq to identify differentially methylated regions (DMRs). All cfDNAs were also analyzed by mFastSeqS for aneuploidy, and shWGS/ exome NGS to define ploidy, tumor fraction, somatic variants and mutational signatures.

**Results and Discussions**

Sequencing data from the various assay for pre- and post-treatment cfDNA samples were compared between PARPi resistant and responding patients. Aneuploidy detected by mFastSeq in cfDNA indicating high tumor load was undetectable pre-treatment but scored positive post-treatment in six patients: four resistant and two responding patients with trended towards significance (p=0.06). IchorCNA analyses of shWGS from post-treatment cfDNA confirmed these latter findings (p=0.07). In contrast, MeDseq analyses demonstrated more DMRs (P adjusted <0.1) in pre-treatment (24 DMRs) than in post-treatment (2 DMRs) when comparing cfDNA methylation profiles from resistant and responding patients. All DMRs were hyper-methylated in resistant patients at both timepoints and 4 DMRs were identified as tumor specific.

**Conclusion**

Our genome-wide NGS analyses identified epigenetic and genomic cfDNA hallmarks for PARPi resistance when comparing cfDNA from resistant with that from responding patients. Interestingly, epigenetic differences were especially seen at baseline before treatment, whereas genomic alterations were more frequently observed in resistant patients after progression on treatment. The epigenetic differences at baseline are especially interesting to be further explored as putative predictive biomarkers for PARPi resistance.

### EACR2024-0581
### ctDNA shedding in early colorectal cancer

*S. Andaloro[1,2], F. Aigner[3], S. Uranitsch[3], S. Lax[4], K. Sallinger[5], A. El- Heliebi[6], S. Kühberger[1,2], E. Heitzer[1,2]*

*[1]Medical University of Graz, Institute of Human Genetics- Diagnostic and Research Center for Molecular Biomedicine, Graz, Austria*

*[2]Medical University of Graz, Christian Doppler Laboratory for Liquid Biopsies for Early Detection of Cancer, Graz, Austria*

*[3]St. John of God's Hospital, Department of Surgery, Graz, Austria*

*[4]Hospital Graz II, Institute of Pathology-, Graz, Austria*

*[5]Medical University of Graz-, Department of Cell Biology- Histology and Embryology, Graz, Austria*

*[6]Medical University of Graz, Department of Cell Biology- Histology and Embryology, Graz, Austria*

**Introduction**

The analysis of circulating tumor DNA (ctDNA) from plasma holds great promise for early cancer detection. However, detecting minute amounts of ctDNA in early-stage tumors remains challenging, and the biological factors affecting ctDNA release are not yet understood. Therefore, we aim to shed light onto ctDNA release kinetics and its association with tumor biology in treatment-naïve, nonmetastatic colorectal cancer patients (CRC).

**Material and Methods**

A cohort of 59 patients (stage I, n=17; stage II, n=22; stage III, n=20) underwent matched targeted sequencing of resected tumor tissue and plasma samples collected prior surgery using the TSO500 platform (Illumina), a panel enriching for 523 cancer associated genes targeting multiple variant types, including microsatellite instability (MSI) and tumor mutational burden (TMB). Blood was collected prior surgery and recovery rates of variants detected in the tumor in plasma were calculated.

**Results and Discussions**

MSI high tumors (n=12) had a significantly higher mutational burden compared to MSS tumors. At least one tumor-specific variants could be detected 5/17 (29.4%), 18/22 (81.8%), and 18/20 (90.0%) of stage I, II, and III patients, respectively with mean recovery rates of 8.9% (range 0.2 - 33.3), 31.0 (range 4.8 - 81.8), 40.0% (range 2.9 - 70.8). The average VAF in plasma were 0.5% (stage I), 0.6% (stage II), and 1.1% (stage III). Surprisingly, neither MSI or a high TMB nor the clonality of the variants were significantly associated with recovery rates in plasma. Although in 36% of patients clonal tissue variants were predominately picked up in plasma, in 74% of patients subclonal variants (defined as subclonal if the purity corrected VAF was less than 25% of the highest VAF in the sample) could be identified in plasma, sometimes even when clonal variants were not detected.

**Conclusion**

Our data confirm that mutation-based detection ctDNA rates in plasma are rather low in stage I tumors, but significantly higher in stage II/III tumors. The fact that in the majority of our samples, both clonal and subconal mutation could be identified in plasma hints to additional factors that may contribute to the ctDNA sheeding. Analyses focusing on correlating ctDNA detection with tissue phenotype and mutation localization are ongoing.

### EACR2024-0588
### Can the pattern of NEUROD1, POU2F3, and YAP1 expression distinguish the tumor profile in high-grade neuroendocrine lung carcinomas?

*T. Gutierrez Prieto[1], C. Machado Baldavira[2], C. Silvério Faria[2], A. Ab'Saber[2], T. Takagaki[3], V. Luiza Capelozzi[1]*

*[1]Faculty of Medicine of University of Sao Paulo, Pathology, Sao Paulo, Brazil*

*[2]Faculty of Medicine of University of Sao Paulo, Pathology, São Paulo, Brazil*

*[3]Instituto do Coração Incor- University of São Paulo, Pneumology Division, São Paulo, Brazil*

**Introduction**

Small cell lung cancer (SCLC) is an aggressive malignancy that accounts for 20% of all primary lung tumors. Both SCLC and large cell neuroendocrine carcinoma (LCNEC) are high-grade neuroendocrine tumors with poor patient outcomes. Recent studies have

© 2024The Authors. Molecular Oncology is published by John Wiley & Sons Ltd on behalf of Federation of European Biochemical Societies shown inconsistent expression of NE markers in SCLC, including transcription factors NEUROD1, POU2F3, and YAP1. Understanding how these proteins impact tumor growth and progression can lead to better treatments. Our aim is to clarify the relationship between these NE markers' expression and their clinicopathological implications for SCLC and LCNEC patients.

**Material and Methods**

We enrolled 26 surgically resected samples that were obtained by archival formalin-fixed paraffin-embedded of SCLC (N=21) and LCNEC (N=5). NEUROD1, POU2F3 and YAP1 protein expression was detected by immunohistochemistry staining and quantified by QuPath software.

**Results and Discussions**

YAP1 expression was positive in 20 (76.9%) cases, 16 SCLC and 4 LCNEC. NEUROD1 and POU2F3 were positive in both SCLC and LCNEC (mean expression of 34.76% of positive cells vs 31.64% of positive cells, respectively). In our cohort, YAP1 was expressed above the mean in 48.0% of patients. No correlation was found between all markers and the patient´s clinicopathological features, however, we noticed a strong positive correlation between YAP1 and NEUROD1 expression ($\rho = 0.830$, $p = 0.00$), and also between POU2F3 and lymph node metastasis ($\rho = 0.471$, $p = 0.04$). Notably, we observed a mixed YAP1 expression pattern in 4 SCLC cases suggesting that cells with NE features may exhibit YAP1 loss, while non-NE-type cells (in the tumor core) may display YAP1 expression. Previous studies already reported a correlation between YAP1 expression and the chemo-resistance in high-grade neuroendocrine tumors. Although was recently proposed a new molecular subtype classification for SCLC comprising the expression of (NEUROD1, ASCL1, POU2F3, and YAP1), the validation in primary tumors remains unclear. In this ongoing study we found patterns of YAP1, NEUROD1, and PO2F3 that match previous research. We confirmed the "classic" NE profile and identified tumor heterogeneity.

**Conclusion**

Faced with this unfolding scenario, we found differences in NEUROD1, POU2F3, and YAP1 expression, highlighting the challenge of molecular subtyping high-grade neuroendocrine tumors. Moreover, patients with the YAP1 mixed phenotype may benefit from effective treatment options, as YAP1 expression has been linked to chemo-resistance.

### EACR2024-0652
### AI-Driven Exploration of Cancer Stem Cells in Pancreatic Cancer: Unravelling SALL4-A Expression and Clinical Significance

*A. Babajani[1], M. Moradzadeh[2], M. Abolhasani[1], M.V. Sanian[3], S. Abdous[2], E. Kalantari[1], S. Kasaei[2], M.H. Rohban[2], Z. Madjd[1]*

*[1]Iran University of Medical Sciences, Oncopathology Research Center, Tehran, Iran*

*[2]Sharif University of Technology, Department of Computer Engineering, Tehran, Iran*

*[3]University of Helsinki, Institute for Molecular Medicine Finland, Helsinki, Finland*

**Introduction**

Pancreatic cancer poses a formidable challenge due to its high mortality rate, limited treatment options, and lack of effective early detection methods, resulting in low five-year survival rates. Emerging evidence suggests a pivotal role of cancer stem cells (CSCs) in tumor progression, treatment resistance, and recurrence, highlighting the importance of targeting these cells for improved outcomes. This study focuses on exploring the expression of the Sal-like (SALL)4-A transcription factor, a known biomarker of CSCs, in pancreatic tumor tissues. Leveraging artificial intelligence (AI), we analyze immunohistochemistry (IHC) images to interpret SALL4-A expression and investigate its correlation with clinicopathological characteristics and survival outcomes.

**Material and Methods**

A study involving 195 formalin-fixed paraffin-embedded tissue specimens from pancreatic cancer patients was conducted, with a specific focus on SALL4-A expression through IHC analysis on tissue microarrays (TMA). AI-driven prediction was employed using neural networks trained on preprocessed IHC images. The preprocessing steps involved acquisition, standardization, region of interest (ROI) selection, annotation, and segmentation. Expert pathologists conducted h-scoring for system training. The malignant region underwent processing by convolutional neural networks, including ResNet, culminating in H-score prediction. Subsequently, the correlation between SALL4-A expression and clinicopathologic features was assessed.

**Results and Discussions**

AI evaluation demonstrated an impressive 91% accuracy in discriminating malignant and nonmalignant areas in IHC cores. Test results indicated mean squared error (MSE) values of 18 for segments and 28 for the entire core, underscoring the efficacy of the AI-driven approach in predicting SALL4-A expression levels. Analysis of SALL4-A expression and clinicopathologic features revealed that cytoplasmic SALL4-A overexpression correlated with smaller tumor size, well-differentiated histological grade, and reduced lymph node involvement. However, survival analysis did not demonstrate a correlation between SALL4-A expression and improved survival.

**Conclusion**

Our AI-driven approach demonstrates the capability to predict the H-Score with acceptable accuracy. The observed cytoplasmic overexpression of the SALL4-A isoform correlates with more favorable pancreatic tumor behavior, highlighting its potential as a prognostic marker in pancreatic cancer.

### EACR2024-0663
### Quantitative Approaches to Capture tRNA Fragmentation Patterns from small RNA sequencing, as a Biomarker for Colorectal Cancer Detection in Liquid Biopsy

*A.J. Jeon[1], C. He[1], F. Zhu[2], K.Y. Wong[3], R. Liang[4], K.K. Tan[2], J. Lee[5], K.G. Yeoh[2]*

© 2024 The Authors. Molecular Oncology is published by John Wiley & Sons Ltd on behalf of Federation of European Biochemical Societies

[1]Mirxes Lab, Research and Development, Singapore, Singapore
[2]National University of Singapore, Yong Loo Lin School of Medicine, Singapore, Singapore
[3]Tan Tock Seng Hospital, Department of Surgery, Singapore, Singapore
[4]Tan Tock Seng Hospital, Department of Gastro-enterology and Hepatology, Singapore, Singapore
[5]National University Hospital, Department of Gastro-enterology and Hepatology, Singapore, Singapore

**Introduction**

Transfer RNA fragments (tRFs) have emerged as a noteworthy small RNA-based diagnostic biomarker in liquid biopsy. However, quantifying these fragments poses challenges due to their closely related sequences and the scarcity of up-to-date databases. We developed two small RNA sequencing based bioinformatics methods that are independent of tRF annotations, aimed at quantitatively characterizing tRNA fragmentation patterns. We evaluated the diagnostic efficacy of the derived tRF-based features using plasma samples from colorectal cancer (CRC) patients.

**Material and Methods**

Small RNA-sequencing was conducted on 129 CRC tissues (67 tumors; 62 adjacent normal), along with plasma samples from 57 CRC patients and 100 healthy individuals. Frequent tRNA fragmentation boundaries were first identified in CRC tissue data. These coordinates were used to obtain the tRF expression count matrix in plasma samples. Second method measures the similarity of each sample to a collection of CRC tumor tissues using tumor similarity scores (tScores). Scoring matrices were constructed based on tRNA mapped read profiles in tumor tissues, and were used to generate scores to each plasma sample. Linear support vector classifier model explored different feature combinations (tRF counts, and tScores). Through 4x5 cross-validation on plasma data, we assessed the significance of tRF-based features in detecting CRC in plasma samples.

**Results and Discussions**

Our approach successfully uncovered novel tRFs within CRC samples. Among the 1254 tRFs incorporated in the feature matrix, 270 tRFs were annotated sequences in MINTbase v2.0. Including the non-annotated tRFs broadened the feature space and markedly improved model performance (p-value: 0.0126), as evidenced by an improved AUC of 0.856 compared to the model comprising solely of annotated tRFs (AUC: 0.781). Notably, we observed substantial disparities in tRNA read patterns between tissue and plasma samples, as well as across different datasets. This discrepancy was underscored by the presence of numerous sample-specific tRFs and the low tScores observed, indicative of the poor retention of tumor tissue tRNA read profiles in plasma samples. Furthermore, our analysis revealed a weaker model performance by tScores (AUC of 0.745) compared to tRF counts (AUC of 0.856).

**Conclusion**

Our results underscore the ability of our method to identify novel unannotated tRFs, thereby expanding the range of features available for use in cancer detection models.

### EACR2024-0665

### A tumor-agnostic approach to monitor disease recurrence in plasma of patients affected with pleural mesothelioma: a proof-of-principle study

L. Mannarino[1], M. Piemontese[2], L. Paracchini[1], M. Aliprandi[1], L. Cecchi[1], M. Perrino[3], N. Cordua[3], M. D'Incalci[1], P.A. Zucali[1], S. Marchini[2]
[1]Humanitas University, Department of Biomedical Sciences, Pieve Emanuele, Italy
[2]IRCCS Humanitas Research Hospital, Laboratory of Cancer Pharmacology, Rozzano, Italy
[3]IRCCS Humanitas Research Hospital, Department of Oncology, Rozzano, Italy

**Introduction**

Pleural mesothelioma (PM) has a latency period spanning from 20 to 50 years. It manifests as a malignant disease at diagnosis with a very poor prognosis. Evaluating therapy response through computed tomography (CT) poses significant challenges, largely attributed to the circumferential and axial growth patterns characteristic of PM. Therefore, there is a pressing need for complementary approaches to aid clinicians in the assessment and monitoring of treatment response.

**Material and Methods**

A retrospective cohort of 79 plasma samples was withdrawn from 17 patients with a diagnosis of PM enrolled in a clinical trial (ONC/OSS-02/2020 trial). Patients were divided into two groups: group A (n=7) including patients who received neoadjuvant chemo-therapy followed by surgery, and group B (n=10) composed of patients treated with first-line chemotherapy alone. In both groups, a blood sample was available at diagnosis naïve to treatment, and at least one matched blood sample was available after surgery or treatment. Clinical data and CT scan images were available. Plasma was processed and sequenced on Illumina Nextseq550 for shallow whole genome sequencing (sWGS). Data were analyzed with an ad-hoc developed bioinformatic pipeline to extract information on the tumor fraction (TF) and to identify regions of copy number alterations (CNAs). When possible, formalin-fixed, paraffin-embedded samples from matched tumor tissue collected at the diagnosis were retrieved and analyzed.

**Results and Discussions**

A tumor-agnostic approach based on sWGS allows to measure the plasma TF as a surrogate marker of PM. Indeed, CNAs in plasma closely mirrored those present in matched tumor biopsies, thus confirming their pathological origin. By monitoring the longitudinal evolution of the TF, we observed that the dynamic of TF parallels the conventional clinical assessment of the tumor response. Specifically, an increased TF corresponded to disease progression, while lower TF values reflected a tumor reduction. Moreover, in two specific cases with stable disease, we found an increased TF months before the radiological assessment of progressive disease.

**Conclusion**

In this proof-of-principle study, we demonstrated the feasibility of our tumor-agnostic approach in assessing the TF in the plasma of PM patients. Results from the

© 2024 The Authors. Molecular Oncology is published by John Wiley & Sons Ltd on behalf of Federation of European Biochemical Societies longitudinal analysis suggest that TF monitoring could help clinicians in the evaluation of therapy response and the timing of cancer recurrence.

### EACR2024-0675
### Characterization of Early Development Murine Embryos using Spatio-temporal Phenotyping with Ultrahigh-plex Antibody Panels

*T. Tam[1], B. Cheikh[1], N. Nikulina[1], D. Klymyshyn[1], N. Ma[1], R. Stad[1], S. Seidel[1]*
*[1]Akoya Biosciences, Applications, Marlborough, United States*

**Introduction**
The mouse embryo model stands as one of the predominant animal models utilized in the exploration of developmental biology. The process of embryonic development is highly regulated and requires precise spatial and temporal expression of proteins crucial for regional specialization and tissue-specific functions. Employing spatial proteomics techniques in the study of developing embryos allows more comprehensive understanding of cellular organization, intercellular interactions within tissues, and regulatory mechanisms at the single-cell level.
**Material and Methods**
We designed a 36-plex panel tailored specifically for analyzing mouse embryos at various developmental stages using the PhenoCycler®-Fusion (PCF) 2.0 Platform. This panel was curated by selecting biomarkers representing immune cell lineages, immune activation, proliferation, structural elements, and neuronal components. Purified antibodies were labeled with PhenoCycler barcodes and rigorously assessed for specificity and reproducibility. Following successful validation, the panel was applied to formalin-fixed paraffin-embedded (FFPE) mouse embryos, and ultrahigh-plex whole-slide imaging was conducted using the PCF 2.0 platform.
**Results and Discussions**
Unsupervised clustering analysis and deep phenotyping revealed a high-resolution spatial atlas of mouse embryos with regional specialization in the cellular landscape of different organs at distinct stages of development.
**Conclusion**
The tools devised within this study offer the capability for comprehensive exploration into multiple facets of organogenesis. They enable the investigation of spatio-temporal dynamics concerning cellular and molecular expression in embryonic development.

### EACR2024-0749
### Determination of Blood-Based Biomarker for Discriminating Chromophobe Renal Cell Carcinoma from Renal Oncocytoma

*J.W. Song[1], B. Lee[2], K. Um[1], S.K. Kang[2], J.T. Cha[1], J.W. Choi[3]*
*[1]Kyunghee University, Pharmacology- Institute of Regulatory Innovation Through Science, Seoul, South Korea*
*[2]Kyunghee university, Biomedical and Pharmaceutical Sciences, Seoul, South Korea*
*[3]Kyunghee University, Pharmacy, Seoul, South Korea*

**Introduction**
Chromophobe RCC is the second prevalent type in non-ccRCC. ChRCC is originated from intercalated cell of the collecting duct which makes it distinct from ccRCC. Renal oncocytoma (RO) is also originated from the intercalated cell. Recent findings of hybrid oncocytic/chromophobe tumor (HOCT) show direct lineage between ChRCC and RO. Although RO is unnecessary for immediate surgical removal, the first line for renal mass intervention is surgical excision since it is impossible to distinguish ChRCC and RO via imaging. So, a non-invasive method for distinguishing both is needed. In this research, in silico analysis of several GEO datasets distinguished differentially expressed genes (DEGs) between ChRCC and RO. Our analysis was able to find a significant blood marker to distinguish ChRCC and RO. Also, a possible mechanism of malignant transformation from RO to chRCC is addressed.
**Material and Methods**
Secretome database was enriched from the human protein atlas(HPA) and Genotype-Tissue Expression Project (GTEx) and matched with in silico analysis of GSE dataset. Tissue and blood samples of 45 human chRCC and 13 RO samples was acquired from Seoul National University Hospital. Tissue samples were harvested to run qPCR and tissue microarry on FAT1. ELISA for FAT1 was performed on patient blood. ChRCC cell line UOK276 was used for in vitro validation. ARACNE was used to find activated transcription factors responsible for FAT1 overexpression.
**Results and Discussions**
Using the secretome database and GSE dataset, we found FAT1 upregulation in ChRCC. qPCR data of human tissues showed 5-fold upregulation of FAT1 in ChRCC (p=0.000194). Also, IHC microarray of the tissue with FAT1 showed distinct staining strength between two groups. The intensity of the signal was scored accordingly (2; strong 1; intermediate, 0; weak. Normalized by number of patients). The score was higher in ChRCC by 1.7-fold (ChRCC= 1.27, RO=0.75). ELISA of patient blood sample showed discrete level of FAT1 secretion in serum (ChRCC=3.719ng/ml, RO=1.635ng/ml, p=0.0058). ARACNE and FAT1 promoter sequence analysis found activated transcription factors responsible for FAT1 upregulation in ChRCC. Functional analysis of FAT1 with shRNA and FAT1 overexpression vector found oncogenic role of FAT1 in ChRCC.
**Conclusion**
Secreted protein FAT1 can be used as a serum marker to distinguish ChRCC and RO. Validation was performed with patient tissue samples and blood samples. Functional in vitro study disclosed mechanism of FAT1 upregulation in ChRCC.

### EACR2024-0753
### Collagen Triple Helix Repeat Containing 1 (CTHRC1) is a prostate cancer prognosis biomarker regulated by androgen receptor (AR) activity

*A. Gonzalo Paulano[1], A. Macchia[1,2], U. Armendariz[1],*

© 2024 The Authors. Molecular Oncology is published by John Wiley & Sons Ltd on behalf of Federation of European Biochemical Societies

J.I. Lopez[3], A. Carracedo[2,4,5], V. Torrano[1,5]
[1]University of the Basque Country UPV/EHU, Biochemistry and Molecular Biology, Leioa, Spain
[2]CICbioGUNE, Bizkaia Technology Park- 801 building, Derio, Spain
[3]University Hospital Cruces, Pathology Department, Bilbao, Spain
[4]IKERBASQUE- Basque foundation for science, Basque foundation for science, Bilbao, Spain
[5]Centro de Investigación Biomédica en Red de Cáncer CIBERONC,
Centro de Investigación Biomédica en Red de Cáncer CIBERONC, Madrid, Spain

**Introduction**

Prostate cancer (PCa) ranks second in incidence among male cancers and sixth in global cancer mortality. Androgen deprivation therapy (ADT) targets androgen receptor (AR) activity, being the current standard treatment for PCa. Despite its efficacy, 15% of patients experience relapse and develop metastasis. Identifying molecular markers of relapse and metastasis is crucial for prognosis. Transcriptomic data from cancer specimens have become vital for classifying, stratifying, and identifying clinically relevant genes in tumors. This has shown the gene expression deregulation is crucial for cancer pathogenesis and progression. Cancer cells communicate with each other and the tumor micro-environment mainly through secreted molecules to sustain their characteristics, and the transcriptional deregulation of these factors may affect cancer progression.

**Material and Methods**

We started exploring secretome-related genes in PCa for prognosis, progression, and therapy improvement. Through computational screenings of patient cohorts, we identified Collagen Triple Helix Repeat Containing 1 (CTHRC1) as the top gene for PCa prognosis.

**Results and Discussions**

CTHRC1 expression is strongly increased in PCa lesions compared to benign ones, both at mRNA and protein level. We have approached to study molecular mechanisms that may drive this increased expression at genomic and transcriptomic level. The interrogation of PCa patients 'data showed that, although 5-30% frequencies of different CTHRC1 genomic alterations can be detected in PCa patients, none of these events could fully explain the increase. On the other hand we observed that CTHRC1 mRNA expression was consistently and inversely correlated with three independent AR transcriptional signatures. Furthermore, we aimed at studying if CTHRC1 expression directly respond to the modulation of AR expression and activity by activating and/or inhibiting the AR activity. Hence, CTHRC1 mRNA expression is negatively regulated by AR activity, possibly through the TGF-β signaling cascade disruption.

**Conclusion**

Our results show that CTHRC1 expression is increased and has a strong prognostic potential in PCa. This increase is robustly associated with a reduced AR transcriptional activity. We propose implementing PCa precision medicine strategies based on monitoring CTHRC1 expression to provide prognostic information and guide therapeutic responses to ADT. We believe AR-TGFβ crosstalk lays groundwork for novel therapeutic strategies targeting both pathways in CTHRC1-high aggressive PCa tumors.

### EACR2024-0764
### A comparative study of four cell-free DNA assays for detecting circulating tumor DNA in early breast cancer

C. Mulder[1], E. Jongbloed[2], M. Liefaard[1], S. Makrodimitris[2], D. Hazelaar[2], L. Mulder[1], S. Wilting[2], M. John[2], M. Jansen[2], E. Lips[1]
[1]The Netherlands Cancer Institute, Division of Molecular Pathology, Amsterdam, The Netherlands
[2]Erasmus MC Cancer Institute, Department of Medical Oncology, Rotterdam, The Netherlands

**Introduction**

Early prediction of the response to neoadjuvant chemo-therapy (NAC) enables tailoring treatment strategies to the specific needs of individual breast cancer patients. Circulating tumor DNA (ctDNA) has shown to be able to predict response on NAC during treatment. However, at this point in time mostly tumor-informed ctDNA detection methods are used which are costly, have relatively long turnaround times and due to scarcity of biopsy material, are potentially less feasible for wide clinical application.

**Material and Methods**

In this study, we investigated four tumor-agnostic methods to determine their ability to detect circulating tumor DNA (ctDNA) at baseline. These methods were the Oncomine Breast cell free DNA (cfDNA) NGS panel, the LINE-1 sequencing assay mFAST-SeqS, low pass shallow whole genome sequencing (WGS) and the genome-wide methylation profiling assay MeD-Seq.

**Results and Discussions**

In total 40 patients with triple negative or luminal B breast cancer were included and cell free DNA (cfDNA) from plasma before the start of NAC was analyzed with the four assays. We detected ctDNA in 3/24 (12.5%) patients with Oncomine, 5/40 (12.5%) with mFast-SeqS, 3/40 (7.7%) with low pass shallow WGS and 23/40 with MeD-Seq (57.5%).

**Conclusion**

In conclusion, we demonstrated that tumor agnostic methods, in particular MeD-Seq, can detect ctDNA in part of the patients with early breast cancer, but further optimisation is needed to reach the potential currently demonstrated by tumor-informed.

### EACR2024-0781
### Carcinoembryonic antigen (CEA) is linked to poor patient prognosis in prostate cancers lacking TMPRSS2:ERG gene fusions

N. Heckmann[1], M. Lennartz[1], R. Simon[1], H. Heinzer[2], A. Haese[2], S. Minner[1], T. Schlomm[3], G. Sauter[1], M. Graefen[2], H. Plage[3]
[1]University Medical Center Hamburg-Eppendorf, Institute of Pathology, Hamburg, Germany
[2]University Medical Center Hamburg-Eppendorf, Martini-Clinic- Prostate Cancer Center, Hamburg,

© 2024 The Authors. Molecular Oncology is published by John Wiley & Sons Ltd on behalf of Federation of European Biochemical Societies

Germany
[3]Charité – Universitätsmedizin Berlin- Corporate Member of Freie Universität Berlin- Humboldt-Universität zu Berlin and Berlin Institute of Health, Department of Urology, Berlin, Germany

**Introduction**

Prostate cancer is the most frequent tumor type in males. About 10% of tumors develop into a life-threatening disease and must be identified and treated as early as possible. The established clinical-pathological prognostic markers are not sufficient to reliably identify these patients. It is hoped that molecular markers could further improve assessment of patient prognosis. Carcino-embryonic antigen (CEA) is a cell surface glycoprotein which has been associated with poor prognosis in several tumor types, but data on its role in prostate cancer are discrepant.

**Material and Methods**

To better understand the prognostic value of CEA, a tissue microarray containing 17,747 prostate cancer spots with associated follow-up and molecular data was analyzed by immunohistochemistry.

**Results and Discussions**

CEA immunostaining was observed in 1,225 (8.5%) of 14,344 interpretable tumors, including 7.7% with weak and 0.8% with moderate to strong (high) staining intensity. High CEA staining was significantly linked to high tumor stage ($p<0.0001$) but was unrelated to Gleason grade. CEA positivity was more frequent in tumors harboring the prostate cancer specific TMPRSS2:ERG fusion (11.7% of 4,894 tumors) than in ERG-fusion negative tumors (6.5% of 6,155 tumors, $p<0.0001$). Subset analyses revealed that high CEA expression was linked to early PSA recurrence in ERG-negative cancers, which was independent from other prognostic parameters available from presurgical biopsies in a multivariate analysis. Comparison with other molecular markers showed that high CEA expression was linked to genomic deletions of the PTEN tumor suppressor gene ($p<0.0001$), but unrelated to androgen receptor expression or tumor cell proliferation.

**Conclusion**

Elevated CEA expression occurs in a small subset of prostate cancers and is linked to high pathological stage. In tumors lacking TMPRSS2:ERG fusions, CEA immunohistochemistry may be suitable to complement other prognostic markers to identify patients with an adverse clinical course.

### EACR2024-0802

### Molecular profiling of cell-free DNA from cerebrospinal fluid is a promising tool to diagnose leptomeningeal metastasis

B.M.H. van Linder[1], D. Brandsma[2], E. Verhoeven[1], L. Jongejan[1], K. Monkhorst[1], A.J. de Langen[3], D. van den broek[4], T. van Wezel[1], M.C. Boelens[1]
[1]Netherlands cancer institute - Antoni van Leeuwenhoek NKI-AvL, Department of Pathology, Amsterdam, The Netherlands
[2]Netherlands cancer institute - Antoni van Leeuwenhoek NKI-AvL, Department of Neuro-Oncology, Amsterdam, The Netherlands
[3]Netherlands cancer institute - Antoni van Leeuwenhoek NKI-AvL, Department of Thoracic Oncology, Amsterdam, The Netherlands
[4]Netherlands cancer institute - Antoni van Leeuwenhoek NKI-AvL, Department of Laboratory Medicine, Amsterdam, The Netherlands

**Introduction**

Leptomeningeal metastasis (LM) is associated with poor prognosis and limited treatment options. Confirmation of LM is based on cytological examination of the cerebro-spinal fluid (CSF). However, its sensitivity is limited, leading to delayed or missed diagnoses of LM. Previous work showed an improved sensitivity through circulating tumour cell (CTC) analysis by flow cytometry. This study explores the diagnostic potential of next generation sequencing (NGS) by analysing tumour driver mutations in CSF.

**Material and Methods**

CSF cell-derived DNA, CSF-derived cell-free DNA (cfDNA) and plasma-derived cfDNA were isolated from a group of ten patients with confirmed LM from a tumour with known driver mutation. NGS was performed to examine the presence of these driver mutations in each sample type. Next, CSF from 89 patients with a suspicion on LM (based on clinical symptoms and/or radiological lesions) were used for cfDNA-NGS, CTC analysis and cytology to compare diagnostic sensitivities.

**Results and Discussions**

Sequencing CSF-cell-DNA showed variant allele frequencies (VAF) dependent on the estimated tumour cell percentage in the CSF. In contrast, sequencing CSF-cfDNA did not depend on TCP. The average VAF of oncogenic driver mutations was approximately 50%, suggesting the majority of cfDNA was tumour cell-derived. VAFs of driver mutations in plasma-cfDNA were modest, indicating a negligible contribution to cfDNA in CSF. Analysing CSF from 89 patients resulted in a sensitivity of 100%, 82%, 93% and a specificity of 96%, 100%, and 100% for cfDNA-NGS, cytology and CTC-analysis respectively. Driver mutations were found in the CSF from one out of 28 patients without LM, potentially caused by an active brain metastasis (BM) bordering the CSF. However, six other negative LM patients with an active BM bordering the CSF, showed no driver mutations in the CSF.

**Conclusion**

This study demonstrates that molecular profiling of CSF-cfDNA is a promising tool to diagnose LM from tumours harbouring a driver mutation, exceeding the sensitivity of CTC analysis and cytology. Caution is warranted regarding patients diagnosed with active BM bordering the CSF. Although cytology is still the golden standard applicable for all tumour types, we incorporated CTC-analysis and cfDNA-NGS to our diagnostic routine for LM. Alongside diagnosing LM, knowledge of driver mutations and the ability to detect potential resistance mechanisms using NGS of CSF-cfDNA can be of additional value for treatment.

### EACR2024-0803

### Patterns of glucose transporter 1 (GLUT1) expression in 95 different human cancer types

S. Büyücek[1], K. Möller[1], F. Lutz[1], C. Bernreuther[1],

Molecular Oncology 18 (Suppl. 1) (2024) 1–495
© 2024 The Authors. Molecular Oncology is published by John Wiley & Sons Ltd on behalf of Federation of European Biochemical Societies

*G. Sauter[1], R. Simon[1], N. Gorbokon[1], M. Lennartz[1], E. Burandt[1], V. Chirico[1]*
*[1]University Medical Center Hamburg-Eppendorf, Institute of Pathology, Hamburg, Germany*

**Introduction**

Glucose transporter 1 (GLUT1) is a key protein for trans membranous glucose uptake of cells. GLUT1 is often overexpressed in cancer to improve glucose uptake in a nutrient deprived environment or to further accelerate the metabolism even in the presence of physiological oxygen concentrations. High levels of GLUT1 have been linked to adverse tumor features and poor prognosis in some cancer types, and several GLUT1 inhibitors have been developed that may hold promise for novel anti-cancer therapies. However, literature data on GLUT1 expression in human cancers are highly discordant.

**Material and Methods**

To comprehensively determine the prevalence of GLUT1 expression across a broad range of human cancers, a tissue microarray containing 5,206 samples from 95 different tumor types and subtypes as well as 608 samples of 76 different normal tissue types was analyzed by immunohistochemistry.

**Results and Discussions**

GLUT1 immunostaining was observed in 2,606 (68%) of 3,832 analyzable tumors, including 16.6% with weak, 10.7% with moderate, and 40.6% with strong staining intensity. GLUT1 positivity was found in 88 of 95 tumor categories, and 71 tumor categories contained at least one strongly positive case. Highest rates of GLUT1 positivity were found in squamous cell carcinomas of various organs (98.6-100%), endometrial carcinomas (95.1-100%), carcinomas of the ovaries (70-100%), Brenner tumors (100%), embryonal carcinomas (100%) and yolk sac tumors of the testis (97.5%), urothelial cancers (96%), and clear cell renal cell carcinomas (91.1%). Tumor types with markedly lower rates of GLUT1 positivity included breast cancers of no special type (57.6%), lobular breast cancers (7.8%), papillary renal cell carcinomas (46.3%), and prostate cancers (3.2-40.7%). GLUT1 was only occasionally (<10%) positive in 11 tumor types including gastrointestinal stroma tumors (4.9%) and hepatocellular carcinomas (4%). In 512 squamous cell carcinomas, high level GLUT1 expression was linked to high grade cancer phenotype ($p=0.0072$).

**Conclusion**

High level GLUT1 expression is often found in many different tumor entities and particularly in squamous cell carcinomas. However, many tumors are also GLUT1 negative. Immunohistochemical GLUT1 expression analysis may be useful to select patients for anti-cancer therapies targeting GLUT1.

### EACR2024-0813
### Identifying and characterizing Circulating Tumor Cell subpopulations to enhance tumor progression understanding

*B. Martínez Castedo[1,2], B. García-Micó[1,2], F. Gimeno-Valiente[3], J. Martín-Arana[1,2], D.G. Camblor[1], P. Martín-Martorell[1], A. Insa[1], D. Roda[1,2], A. Cervantes[1,2], N. Tarazona[1,2]*
*[1]INCLIVA Biomedical Research Institute, Medical Oncology, Valencia, Spain*
*[2]Instituto de Salud Carlos III, Ciberonc, Madrid, Spain*
*[3]UCL Cancer Institute, Cancer Evolution and Genome Instability Laboratory, London, United Kingdom*

**Introduction**

The presence of circulating tumor cells (CTCs) plays a pivotal role in liquid biopsy, offering insights into tumor dissemination and helping to our comprehension of metastasis and relapse mechanisms. Originating from the primary tumor, CTCs propagate into the peripheral blood (PB), providing a real-time depiction of intratumor heterogeneity through a minimally invasive approach. Despite their potential, the inherent challenge lies into the low abundance of CTCs, hindering effective isolation, analysis and the generation of preclinical models. Hence, our aim is to detect, characterize and establish CTC cultures, aiming to enhance our understanding of the metastatic disease

**Material and Methods**

A cohort of PB samples was collected from 7 metastatic colon and lung cancer patients prior to any treatment. Subsequently, 20mL of PB were processed using a negative selection system involving the depletion of $CD45^+$ cells and density gradients to enrich viable CTCs. CTCs were expanded in vitro and individually separated using the DEPArray$^{TM}$ technology. Their epithelial-mesenchymal features were examined by imaging flow cytometry using Cytokeratin (CK)-555, Vimentin-488 and CD45-APC

**Results and Discussions**

Through the CTC separation procedure, enrichment experiments using a test cohort demonstrated a recovery rate of 75% in studied samples. The average detection rate of CTCs was 50%, allowing the identification of at least 2 epithelial CTCs per sample. In our main cohort of metastatic patients, potential CTCs were detected in 5 out of 7 individuals, showing a higher success rate in lung cancer patients compared to those with colon cancer. Among the 5 patients subjected to in vitro cultivation, at least 4 CTCs could be initially observed on day 1 after establishment, displaying specific morphological characteristics such as size, granularity, circularity and the formation of protrusions. Using imaging cytometry with the described marker panel, CTCs were detected in 4 out of 5 patients, highlighting that in 3 patients the number of CTCs with an epithelial-like phenotype, expressing CK, exceeded the count of CTCs with a mesenchymal-like phenotype. One patient showed an opposite observation

**Conclusion**

In our study, we successfully isolated and classified CTCs into subpopulations, enhancing our understanding of tumor progression and the behavior of these cells. Additionally, the presented CTC cultivation model allows for their characterization, demonstrating potential for discovering biomarkers and testing targeted drug therapies

### EACR2024-0818
### High level expression of glucocorticoid receptor (GR) expression is linked to aggressive tumor features, early

© 2024 The Authors. Molecular Oncology is published by John Wiley & Sons Ltd on behalf of Federation of European Biochemical Societies

### biochemical recurrence, and genetic instability in prostate cancer

*N. Heckmann[1], M.C. Tsourlakis[1], R. Simon[1], H. Heinzer[2], A. Haese[2], S. Minner[1], G. Sauter[1], T. Schlomm[3], M. Graefen[2], H. Plage[3]*
*[1]University Medical Center Hamburg-Eppendorf, Institute of Pathology, Hamburg, Germany*
*[2]University Medical Center Hamburg-Eppendorf, Martini-Clinic- Prostate Cancer Center, Hamburg, Germany*
*[3]Charité – Universitätsmedizin Berlin- Corporate Member of Freie Universität Berlin- Humboldt-Universität zu Berlin and Berlin Institute of Health, Department of Urology, Berlin, Germany*

**Introduction**

The glucocorticoid receptor (GR) is a nuclear receptor protein for cortisol and other glucocorticoids and regulates the transcription of thousands of genes involved in metabolism, development, stress and inflammatory response. In prostate cancer, GR may confer resistance to anti-androgen receptor therapies by bypassing AR blockade. However, only few data are available on the prognostic role of GR expression in prostate cancer.

**Material and Methods**

To estimate the prognostic value of GR in a large set of prostate cancers, a tissue microarray containing 17,747 prostate cancers with associated follow-and molecular data was analyzed by immunohistochemistry. All patients had undergone radical prostatectomy.

**Results and Discussions**

GR immunostaining was found in 10,832 (89.1%) of 12,125 interpretable tumors, including 48.5% with weak, 29.8% with moderate and 11% with strong staining intensity. Increased GR staining was strongly linked to adverse feature of the disease, including high tumor stage (pT), high classical and quantitative Gleason grade, presence of nodal metastases (pN+), a positive surgical margin (R) status, and early biochemical recurrence ($p<0.0001$ each). A multivariate analysis showed that the prognostic value of strong GR staining was independent from pT, Gleason grade, pN and R status. Comparison with available molecular data revealed associations between strong GR staining and presence of the prostate cancer specific TMPRSS2:ERG fusion ($p<0.0001$), genomic deletions of PTEN ($p<0.0001$) and 6 additional genomic loci (3p14, 8p21, 12p13, 16q24, 17p13 and 18q21, and a positive androgen receptor status ($p<0.0001$ each).

**Conclusion**

High level expression of GR is strongly linked to prostate cancer aggressiveness and genomic instability in uni- and multivariate analysis. GR immunohistochemistry – alone or in combination with other markers – holds great potential to identify patients with a high risk for tumor progression.

### EACR2024-0819
### Glutathione peroxidase 2 (GPX2) expression in human tumors: A tissue microarray study involving 18,555 cancers from 148 tumor entities

*V. Chirico[1], G. Sauter[1], D. Dum[1], A.H. Marx[2], R. Simon[1], T.S. Clauditz[1], P. Lebok[1], C. Fraune[1], M. Lennartz[1], S. Steurer[1]*
*[1]University Medical Center Hamburg-Eppendorf, Institute of Pathology, Hamburg, Germany*
*[2]Academic Hospital Fuerth, Department of Pathology, Hamburg, Germany*

**Introduction**

Glutathione peroxidase 2 (GPX2) is a selenium-dependent glutathione peroxidase with a pivotal role in removing reactive oxygen species (ROS) from cells. Altered GPX2 expression has been reported from several tumor types, and up- or downregulation may have prognostic value in some of them. However, literature data on GPX2 expression in different cancer types are controversial.

**Material and Methods**

To better comprehend the role of GPX2 expression in cancer, GPX2 was analyzed by immunohistochemistry (IHC) on tissue microarrays (TMAs) containing 18.555 samples from 148 different tumor types.

**Results and Discussions**

GPX2 immunostaining was always cytoplasmic and/or nuclear. Detectable GPX2 staining occurred in 5,919 (37.8%) of the 15,654 analyzable tumors, including 1,177 (7.5%) with weak, 1,171 (7.5%) with moderate, and 3,571 (22.8%) with strong positivity. A total of 95 of 148 tumor categories showed GPX2 expression in at least one case, 39 tumor categories showed GPX2 staining in ≥50% of cases, and 61 tumor categories included at least one case with strong GPX2 positivity. The frequency of GPX2 positivity was highest in non-invasive urothelial carcinomas (88.9-100%) and tumors of the gastro-intestinal tract, including adenomas (100%) and carcinomas of the colorectum (97.9-100%), gallbladder tumors (84.4-94.7%), pancreatic adenocarcinomas (83.4-89%), gastric cancers (83.1-87.3%), and hepatocellular carcinomas (80.4%). Clinico-pathological data were available form carcinomas of the urinary bladder, colon, and ovarian cancers. GPX2 positive decreased significantly during progression of these cancers. In bladder cancer, reduced GPX2 positivity was significantly linked muscle-invasive disease ($p<0.0001$ for pTa vs pT2-4). In colorectal cancers, reduced GPX2 positivity was linked to high tumor stage ($p=0.0044$), lymph node metastasis ($p=0.0093$), and microsatellite instability ($p<0.0001$). In ovarian cancers, reduced GPX2 expression was linked to high tumor stage ($p<0.0044$) and lymph node metastasis ($p=0.0002$).

**Conclusion**

GPX2 expression is particularly frequent in tumors of the urinary and gastrointestinal tract but can also occur at lower frequencies in many other tumor types. Since a loss of GPX2 expression was linked to adverse clinical features in cancers of the urinary bladder, colorectum and the ovaries, our data may suggest a general role of low GPX2 expression as a feature of cancer aggressiveness across different cancer types.

### EACR2024-0822
### Comprehensive tumor profiling identifies predictive biomarkers expanding clinical options for male breast cancer patients

*V. Valentini[1], A. Bucalo[1], G. Conti[1], V. Porzio[1], I. Zanna[2],*

Molecular Oncology 18 (Suppl. 1) (2024) 1–495
© 2024 The Authors. Molecular Oncology is published by John Wiley & Sons Ltd on behalf of Federation of European Biochemical Societies

D. Calistri[3], L. Cortesi[4], G. D'amati[5], V. Silvestri[1], L. Ottini[1]
[1]Sapienza University of Rome, Department of Molecular Medicine, Rome, Italy
[2]Institute for Cancer Research- Prevention and Clinical Network ISPRO, Cancer Risk Factors and Lifestyle Epidemiology Unit, Florence, Italy
[3]Istituto Scientifico Romagnolo per lo Studio e la Cura dei Tumori IRST IRCCS, Biosciences Laboratory, Meldola, Italy
[4]University of Modena and Reggio Emilia, Department of Oncology and Haematology, Modena, Italy
[5]Sapienza University of Rome, Department of Radiological- Oncological and Anatomo-Pathological Science, Rome, Italy

**Introduction**

Male Breast Cancer (MBC) is a rare disease, much less investigated and with a higher mortality compared with female breast cancer (BC). Increasing evidence suggest gender-specific differences in BC, in terms of biological and clinical behavior as well as response to treatment. Comprehensive tumor profiling provided advancements in treating cancer patients according to their molecular profiles. Data on specific tumor profiles in MBC are lacking, hindering the development of tailored treatments for the patients. In this study, we performed a comprehensive molecular profiling of MBCs by multigene panel sequencing, to identify molecular biomarkers that may inform novel treatment strategies for MBC patients.

**Material and Methods**

A series of 106 MBC samples, including cases with *BRCA1/2* germline pathogenic variants (PVs), was analyzed by gene panel sequencing with TruSight Oncology 500® panel (Illumina) targeting 523 cancer-relevant genes. Somatic variants were classified into Tier I and Tier II, according to their strong or potential clinical significance, respectively and as specified by Catalogue of Somatic Mutations in Cancer database. MBCs were classified as having high-TMB with ≥10 mutations/Megabase (mut/Mb) and high-MSI with ≥10% of unstable sites. The associations between these biomarkers and pathology data were evaluated.

**Results and Discussions**

According to their clinical significance, 29 Tier I and seven Tier II somatic variants were identified in about 30% of MBCs. In line with previous evidence, *PIK3CA* was the most frequently mutated gene, with clinically actionable alterations identified in about 23% of MBCs. Mutual exclusivity between somatic variants and germline PVs in *BRCA1/2* was observed (p=0.03). High-TMB and high-MSI values were observed in about 10% and 12% of MBCs, respectively. One MBC was classified as both high-TMB and high-MSI. High-TMB MBCs showed a higher percentage of tumor infiltrating lymphocytes (p=0.04), as reported in other type of tumors. Overall, these results show that specific subsets of MBC may be characterized by clinically actionable somatic variants, and high levels of TMB and MSI.

**Conclusion**

Our findings suggest that a comprehensive tumor profiling may provide data on predictive biomarkers useful for the selection of MBCs eligible for innovative therapeutic approaches, including therapy targeting *PIK3CA* and immunotherapy.

*Study supported by AIRC (IG21389) to LO.*

### EACR2024-0824
### Deep Amplicon Sequencing of PIK3CA gene Using fastGEN Technology

J. Stranska[1,2], R. Slavkovsky[1], G. Korinkova[3], B. Liznerova[1], J. Drabek[1,2], M. Onderkova[4], V. Chladova[4], K. Bezdekova[4], P. Broz[5,6], M. Hajduch[1,2,7]
[1]Faculty of Medicine and Dentistry Palacky University Olomouc, Institute of Molecular and Translational Medicine, Olomouc, Czech Republic
[2]Olomouc University Hospital, Laboratory of Experimental Medicine, Olomouc, Czech Republic
[3]Olomouc University Hospital and Palacky University Olomouc, Department of Clinical and Molecular Pathology, Olomouc, Czech Republic
[4]BioVendor Group, Research & Diagnostic Products, Brno, Czech Republic
[5]BIOXSYS s.r.o., BIOXSYS s.r.o., Usti nad Labem, Czech Republic
[6]Charles University Prague and Faculty Hospital Motol Prague, Department of Biology and Medical Genetics, 2nd Faculty of Medicine, Prague, Czech Republic
[7]Palacky University Olomouc, Czech Advanced Technology and Research Institute, Olomouc, Czech Republic

**Introduction**

The *PIK3CA* gene encodes Phosphatidylinositol-4,5-bisphosphate 3-kinase catalytic subunit *alpha*, also called p110α protein. PI3K enzymes are part of the PI3K/AKT/mTOR pathway regulating cell growth and survival. *PIK3CA* is the most recurrently mutated gene in breast cancer (up to 36 %) and is also detected in other cancer types. It acts as a molecular target for treatment with PIK3CA inhibitor alpelisib (or alpelisib/fulvestrant), which has a benefit for hormone receptor-positive and HER2-negative metastatic breast cancer patients. Testing most *PIK3CA* mutations, not only two main hotspots, 542/545 and 1047, is still challenging for laboratories.

**Material and Methods**

Deep amplicon sequencing (DAS) has a high potential to be a suitable method for the simultaneous detection of somatic mutations within hotspot regions with a defined detection limit down to 1 % minor allelic frequency (MAF). We have developed and validated a unique fast, sensitive, and robust method called fastGEN using Illumina platforms. Formalin-fixed paraffin-embedded breast tumors were genotyped for more than 25 clinically relevant mutations of the *PIK3CA* exonuclease domain and sequenced on MiSeq (Illumina).

**Results and Discussions**

We have prepared *PIK3CA* test including 25+ mutations in exons 2, 3, 5, 7, 8, 10, 14, and 21. Larger somatic NGS panels (Qiagen QIAseq TMB Panel or QIAseq Custom Panel) were used for validation of fastGEN results confirming mutations in all exons. Using samples (n = 10), where results of both methods were available, we observed a concordance with 100 % specificity and sensitivity. The test was highly reproducible (n=6, *PIK3CA* p.H1047R, MAF = 47,2% ± 1,5%; n=4, *PIK3CA* p.V344G, MAF = 6% ± 0,4%) and sensitive (n = 4, input = 1 ng DNA). A minimum turn-around time

© 2024 The Authors. Molecular Oncology is published by John Wiley & Sons Ltd on behalf of Federation of European Biochemical Societies

(sample to final report) was less than 24 hours. fastGEN technology is routinely performed for tumor testing of *RAS*, *BRAF*, *EGFR*, *IDH1/2*, *POLE*, *TERT* and *TP53* in our lab; other genes are under development. The technology was licensed by the partner BioVendor Group.

**Conclusion**

Detection of *PIK3CA* somatic mutations by fastGEN technology is really fast and easy to perform with a high success rate, including samples with low amount and low quality DNA. With other often requested predictive biomarkers, laboratories with Illumina sequencers can easily implement fastGEN kits. A user-friendly and robust bioinformatics pipeline is based on Genovesa fastGEN platform is used. We have shown that using the fastGEN kit could be a suitable method for routine diagnostics.

### EACR2024-0846
### Polyribonucleotide Phosphorylase is overexpressed in Hepatocellular Cancer and associates with epithelial phenotype maintenance and tumor progression

*F. Revert*[1], *F. Revert-Ros*[1], *I. Ventura*[1], *J.Á. Prieto-Ruiz*[1], *E. Giner-Moreno*[2], *Á. Pérez-Cervera*[1], *J. Pérez-Rojas*[2], *J.M. Hernández-Andreu*[1]

[1]*Universidad Católica de Valencia San Vicente Mártir-Facultad de Medicina y Ciencias de la Salud, Research group in Mitochondrial and Molecular Medicine, Valencia, Spain*

[2]*Hospital Universitario y Politécnico La Fe, Department of Pathology, Valencia, Spain*

**Introduction**

Hepatocellular carcinoma (HCC) presents a significant global health concern, driving research into the molecular factors influencing its advancement. Among these factors, the mitochondrial enzyme Polyribonucleotide Phosphorylase (PNPase), encoded by the PNPT1 gene, emerges as a candidate for its potential role in cancer. This study delves into the impact of PNPT1 expression on HCC, particularly its involvement in preserving an epithelial phenotype and in the progression induced by oxidative stress.

**Material and Methods**

Immunohistochemical methods were employed to analyze PNPase expression in HCC samples. RNA-seq data of HCC from The Cancer Genome Atlas program were acquired via the OncoDB website, and the expression of particular genes was assessed. Gene silencing, RT-qPCR, Western blotting, mitochondrial activity assays, and immunofluorescence microscopy investigations were conducted using HepG2 liver cancer cells.

**Results and Discussions**

Here we show that the gene encoding PNPase (*PNPT1*) is overexpressed in HCC tumors compared to matched normal tissues, and that a high *PNPT1* expression is mainly found in HCC tumors with epithelial phenotype expressing E-cadherin. In HepG2 cells, an HCC cellular model, *PNPT1* downregulation induced EMT-like changes, including increased expression of fibrotic and inflammatory markers. Besides, *PNPT1* expression correlated with epithelial and oxidative stress biomarkers in HCC tumors and predicted poor outcomes in non-viral HCC tumors.

**Conclusion**

Our results suggest that an increased PNPase expression appears linked to tumor transformation, whereas decreased PNPase levels in previously transformed hepatocytes may facilitate the development of the mesenchymal phenotype crucial for HCC advancement. Finally, an excessive PNPase expression in epithelial and differentiated HCC tumors could induce heightened oxidative stress, potentially accelerating genetic alterations and worsening prognosis.

### EACR2024-0858
### Recommendations for Molecular Diagnostics of Pleural Effusion

*E. Verhoeven*[1], *B.M.H. van Linder*[1], *E.M.P. Steeghs*[2], *C. Timmer*[2], *F.J. Borm*[3], *K. Monkhorst*[1], *A.J. de Langen*[4], *T. van Wezel*[1], *D. Cohen*[2], *M.C. Boelens*[1]

[1]*Netherlands Cancer Institute, Department of Pathology, Amsterdam, The Netherlands*

[2]*Leiden University Medical Center, Department of Pathology, Leiden, The Netherlands*

[3]*Leiden University Medical Center, Department of Pulmonology, Leiden, The Netherlands*

[4]*Netherlands Cancer Institute, Department of Thoracic Oncology, Amsterdam, The Netherlands*

**Introduction**

Malignant pleuritis is frequently observed in patients with non-small cell lung cancer (NSCLC), which can be drained for both symptom relief and diagnostic purposes. Molecular diagnostics can be requested for diagnosis, choice of therapy, and/or resistance mechanism. Low tumor cell percentage (TCP) and low amount of cells are challenging for reliable results. This study aims to evaluate and compare the diagnostic yield of cell-free DNA (cfDNA) versus cellular DNA, and cfRNA versus cellular RNA isolated from pleural fluid.

**Material and Methods**

From 33 pleural effusion samples of patients with advanced cancer, cfDNA and cellular DNA was isolated from supernatant and cells, respectively. An amplicon-based NGS method was used for variant detection in these samples. Variant calling and respective variant allele frequencies (VAFs) were compared between cfDNA and cellular DNA. In addition, cfRNA and cellular RNA was isolated from 20 samples, which was used for fusion gene detection using the Archer Lung Fusionplex.

**Results and Discussions**

In pleural effusion samples with an estimated TCP of ≥ 10%, cfDNA and cellular DNA yielded comparable results as mutations were consistently detected (n=12). The mutations in these cfDNA and cellular DNA samples showed a comparable median VAF (45% versus 41%, p=ns). In samples with a TCP of < 10%, mutations were more often detected in cfDNA (n=13 versus n=5). The mutations showed a significantly higher median VAF in cfDNA as compared to cellular DNA (6.4% versus 1.0%, p<0,01). For RNA analyses our observations were contrary: in Archer analyses cellular RNA provided qualitatively and quantitatively superior

© 2024 The Authors. Molecular Oncology is published by John Wiley & Sons Ltd on behalf of Federation of European Biochemical Societies results compared to cfRNA. In three out of six primary fusion-positive cases, fusions were detected in cellular RNA but not in cfRNA, independent of TCP. Although this hints towards preferred use of cellular RNA, it requires further investigation whether these results are caused by the cfRNA isolation method or the inherent instability of cfRNA in pleural fluid.

**Conclusion**

Cell-free DNA from pleura effusion is preferred for reliable mutation detection, especially if the TCP is < 10%. In contrast, cellular RNA is, independent of TCP, recommended for fusion detection, though this requires further investigation.

### EACR2024-0868

### Testing for molecular driver alterations (MDA) in non-small cell lung cancer (NSCLC): a real world experience from a tertiary cancer centre in Belarus

M. Puchinskaya[1]

[1]*Minsk City Clinical Oncologic Dispensary, Out-patient, Minsk, Belarus*

**Introduction**

Testing of NSCLC for MDA and administration of appropriate targeted agents has become widely accessible in clinical practice in recent years. However, the percentage of tested cases remains inadequate in many countries, thus preventing patients (pts) from getting effective therapies. We aimed to assess the results of MDA testing in NSCLC in real world practice in LMIC.

**Material and Methods**

Testing results for pts treated at Minsk City Clinical Oncology Centre (MCCOC) – a high volume tertiary cancer centre – in 2020-2023 were analyzed. Data on the numbers of NSCLC pts potentially eligible for testing were obtained from Belarusian cancer registry. Data on MDA testing results as well as some features of tested cases were derived from molecular pathology reports. *EGFR*, *BRAF* and *KRAS* mutations were assessed by PCR and Sanger sequencing, while *ALK* and *ROS1* rearrangements by FISH (IHC for *ALK* from 2022).

**Results and Discussions**

Markers that were tested varied at different time periods due to availability of reagents. Only MDAs for which the corresponding targeted therapy was available were tested (also *KRAS* in some cases to exclude other tests if mutated). Besides, sequential testing was applied, so in most pts not all druggable MDA were tested. The number and percentage (of stage IV and progressive lung adenocarcinomas) of tested pts rose during 4 years (see table).

| | **2020** | **2021** | **2022** | **2023** | **Total** |
|---|---|---|---|---|---|
| Potentially eligible | 113 | 120 | 113 | 131 | 477 |
| Tested (% of eligible) | 60 (53.1) | 84 (70.0) | 97 (85.8) | 119 (90.8) | 360 (75.5) |
| With druggable MDA (% of tested), of them: | 15 (25.0) | 16 (19.0) | 25 (25.8) | 30 (25.2) | 86 (23.9) |
| - *EGFR* exon 19 deletion | 9 (15.0) | 6 (7.1) | 7 (7.2) | 11 (9.2) | 33 (9.2) |
| - *EGFR* L858R | 4 (6.7) | 4 (4.8) | 7 (7.2) | 9 (7.6) | 24 (6.7) |
| - *ALK* | 1 (1.7) | 5 (6.0) | 6 (6.2) | 8 (6.7) | 20 (5.6) |
| - *ROS1* | 1 (1.7) | 1 (1.2) | 1 (1.0) | 1 (0.8) | 4 (1.1) |
| - *BRAF* | Not tested | 0 | 4 (4.1) | 1 (0.8) | 5 (1.4) |

2 patients had mutations in *EGFR* and *ALK* simultaneously. 2 more patients had *EGFR* L861Q mutation. Among tested pts 36.9% were women, but among those with druggable MDA – 61,6%, corresponding to higher prevalence of MDA in women. Mean percentage of tumor cells in submitted samples (assessed semiquantitatively) was 47.8% (5-95%). 82,6% of MDA-positive pts got matched targeted therapies.

**Conclusion**

Access to targeted therapies caused definite growth of MDA testing in NSCLC in our country, with better quality of histologic samples being sent for testing. Percentages of cases with MDA are in line with the numbers in other European countries. Further implementation of genetic testing in real world clinical practice is ongoing.

### EACR2024-0874

### ctDNA Whole-Exome sequencing unveils prognostic insights, actionable alterations, and organ tropisms in pancreatic ductal adenocarcinoma

J. Martin-Arana[1,2], M. Huerta[1,2], F. Gimeno-Valiente[3], B. García-Micó[1,2], B. Martínez-Castedo[1,2], D. G. Camblor[1], M. García-Bartolomé[1], D. Roda[1,2], A. Cervantes[1,2], N. Tarazona[1,2]

[1]*INCLIVA Biomedical Research Institute- University of Valencia, Department of Medical Oncology, Valencia, Spain*

[2]*CIBERONC- Instituto de Salud Carlos III, CIBERONC group, Madrid, Spain*

[3]*University College London Cancer Institute, Cancer Evolution and Genome Instability Laboratory, London, United Kingdom*

**Introduction**

The unfavorable prognosis inherent to pancreatic ductal adenocarcinoma (PDAC) underscores the need for a deeper understanding of progression mechanisms and the implementation of personalized treatments. While the prognostic value of ctDNA has already been established, this study proposes to apply plasma whole-exome sequencing (WES) to identify molecular alterations beyond *KRAS*, predicting survival, uncovering progression mechanisms, and identifying therapeutic targets

**Material and Methods**

Eighty metastatic PDAC patients were prospectively recruited and divided into two consecutive cohorts: discovery (n=37) and validation (n=43). Tumor and plasma from the discovery cohort obtained at diagnosis

© 2024 The Authors. Molecular Oncology is published by John Wiley & Sons Ltd on behalf of Federation of European Biochemical Societies were analyzed using WES. The variant allele frequency (VAF) of *KRAS* mutations was quantified by ddPCR in plasma at baseline and at response from all patients (n=80)

**Results and Discussions**

Plasma WES identified at least one pathogenic variant across all individuals, with *TP53* (79%), *KRAS* (55%), and *BRCA2* (28%) emerging as the most prevalent mutated genes. Mutations were categorized according to their association within 4 specific cancer pathways: oncogenic mechanisms, DNA repair, microsatellite instability and TGFb pathway alterations. Plasma sequencing unveiled a higher number of actionable mutations compared to those identified in their paired tissues (11 vs. 7, including *PTEN*, *BRCA2*, and *TSC2* as plasma-exclusive genes). In patients with a survival less than 11 months, an enrichment in the regulatory pathway of cellular organization components was observed. Conversely, in those with a longer survival, exclusive mutations were detected in genes associated with DNA regulation and repair. Notably, patients with liver metastasis exhibited distinctive mutations, encompassing not only *KRAS* but also genes within the adaptive immune response pathway. Baseline plasma *KRAS* mutations detected by ddPCR correlated with worse progression-free survival (HR=2.315, CI 95%= 1.027-5.217, p = 0.038; and HR= 3.022, CI 95% = 1.299 - 7.030, p= 0.0075 in the discovery and validation cohort, respectively). A significant risk of progression was observed if *KRAS* VAF at response assessment did not decrease, at least, 84.75% in both cohorts

**Conclusion**

ctDNA WES reveals molecular signatures indicative of rapid progression, potential actionable alterations, and mutations specific to liver metastasis in the adaptive immune response pathway in PDAC. The detection of *KRAS* at baseline and its dynamic changes during treatment emerge as a prognostic biomarker

### EACR2024-0884

### Pyrimidine-dependent UV-mediated crosslinking magnifies minor genetic or epigenetic changes in clinical samples

*S. Smilkou[1], F. Yu[2], F. Ahmed[3], S. Yasmin-Karim[2], F. Darberheshti[2], M. Bullock[4], V. Adalsteinsson[5], I. Boukovinas[6], E. Lianidou[7], G.M. Makrigiorgos[2]*

*[1]Analysis of Circulating Tumor Cells Lab, Department of Chemistry- University of Athens, Athens, Greece*

*[2]Dana-Farber Cancer Institute and Brigham and Women's Hospital, Department of Radiation Oncology- Harvard Medical School, Boston MA, United States*

*[3]Dana Farber Cancer Institute and Brigham and Women's Hospital, Department of Radiation Oncology- Harvard Medical School, Boston MA, United States*

*[4]Hormones and Cancer Group- Cancer Genetics Laboratory, Kolling Institute of Medical Research- Royal North Shore Hospital, Sydney, Australia*

*[5]Gerstner Center for Cancer Diagnostics, Broad Institute of MIT and Harvard, Cambridge MA, United States*

*[6]Bioclinic Thessaloniki, Medical Oncology Unit, Thessaloniki, Greece*

*[7]Analysis of Circulating Tumor Cells Lab, Departement of Chemistry- University of Athens, Athens, Greece*

**Introduction**

Detection of minor DNA allele alterations is becoming increasingly important for cancer early detection, monitoring, and treatment selection. As applications increase, so does the need for methodologies that provide improved sensitivity combined with robust performance and specificity. We describe a new method that uses ultraviolet light to eliminate wild-type DNA alleles and enables improved detection of minority genetic or epigenetic changes in tissues and liquid biopsies.

**Material and Methods**

Pyrimidine-Dependent UV-based Minor-allele Enrichment (PD-UVME) employs oligonucleotide-probes that hybridize to WT sequences and incorporate a UVA-sensitive molecule (CNVK) placed directly opposite interrogated pyrimidines, such as thymidine (T) in targeted DNA. Upon UVA illumination CNVK crosslinks with T, preventing subsequent amplification of WT DNA strands. Mutations that remove the T escape crosslinking and are readily amplified and detected. Similarly, when CNVK is placed opposite cytosines in CpG dinucleotides it discriminates between methylated and unmethylated cytosine, enabling direct enrichment of unmethylated DNA targets.

**Results and Discussions**

PD-UVME was applied for detecting *BRAF* V600E mutations in model systems, thyroid patient cancer samples and cfDNA from melanoma patients. The data indicates a substantial increase in V600E mutation detection sensitivity when PD-UVME is performed prior to digital droplet PCR, while normal volunteer samples remain negative. Furthermore, enrichment of unmethylated alleles within excess of methylated alleles in MAGE promoters is demonstrated.

**Conclusion**

PD-UVME mutation/methylation enrichment performed prior to ddPCR magnifies signals from low-level mutations or epigenetic changes and can increase confidence in the results. It can assist with clinical decisions that hinge on the presence of traces of alterations like *BRAF* V600E.

### EACR2024-0886

### Profiling of small non-coding RNAs in tissue and fecal samples of colorectal cancer patients shows alterations related to tumor progression

*A. Gagliardi[1,2], G. Ferrero[1,3], G. Francescato[1,3], V. Alberini[1,3], S. Tarallo[1,2], L. Follia[3], C. Di Battista[4], A. Camandona[3], B. Pardini[1,2], A. Naccarati[1,2]*

*[1]Italian Institute for Genomic Medicine, Genetic and molecular epidemiology, Candiolo- Torino, Italy*

*[2]Candiolo Cancer Institute, FPO-irccs, Candiolo-Torino, Italy*

*[3]Università di Torino, Department of Clinical and Biological Sciences, Torino, Italy*

*[4]Centro de Investigación Biomédica en Red de Enfermedades Hepáticas y Digestivas, Gastrointestinal & Pancreatic Oncology Group, Barcelona, Spain*

**Introduction**

© 2024 The Authors. Molecular Oncology is published by John Wiley & Sons Ltd on behalf of Federation of European Biochemical Societies

Small non-coding RNAs (sncRNAs) have recently emerged for their crucial regulatory role in cellular processes, tumor development, and progression. Due to their extracellular stability, they can be detected in tumor and surrogate tissues, representing promising candidate biomarkers for early cancer diagnosis. We provide an overview of the non-miRNA sncRNA landscape in relation to cancer progression by analysing small RNA sequencing (sRNA-seq) data from advanced adenomas (AA) to late-stage of colorectal cancers (CRC). Next, we explored how the identified patterns of expression in tissues mirrored in stool samples of the same patients.

**Material and Methods**

sRNA-seq was performed on tumor (n=96) or adenoma (n=26) tissues and their paired adjacent mucosa collected in a cross-sectional study. sRNA-Seq was performed on stool samples of the same patients and those from colonoscopy-negative controls (n=87). Human miRNome-unmapped reads were aligned against an in-house sncRNAs reference, and differential expression (DE) analysis was performed with DESeq2 and integrative analyses with miXOmics.

**Results and Discussions**

A total of 15,509 sncRNAs were identified in tissue, with piRNAs (n=13,274) and tRNAs (n=1,095) as the most represented biotypes. Comparing tumor tissue to adjacent mucosa, 417 DE sncRNAs were observed, with tRNAs being mainly over-expressed (97.4%) and snoRNAs down-regulated (83.3%). Advanced cancer stages showed a higher number of DE sncRNAs (n=401) compared to early stages (n=269). With respect to adjacent mucosa, the expression of tRNAs progressively increase from AA to late-stage CRC. In stool samples, comparing CRC patients with healthy controls, a prevalent decrease was observed (n=280), with snoRNAs, piRNAs and tRNAs showing a marked dysregulation. As in tissue samples, the fecal levels of most snoRNAs (95%) decreased in CRC patients and showed a decreasing expression from AA to late-stage CRC. Sparse PCA based on 82 DE sncRNAs detected in both tissue and stool analyses showed a distinct clustering of AA and CRC samples, mainly driven by altered snoRNA levels. This clustering was observed in both biospecimens.

**Conclusion**

We show that sncRNAs expression profiles change with cancer progression. Notably, an increase in tRNAs levels and a decrease in snoRNAs levels from AA to stage IV was observed in tissue. Decreased snoRNA levels are also reflected in stool samples, and they contribute to discrimination between CRC and AA samples, suggesting a possible use as biomarkers in early diagnosis of CRC.

### EACR2024-0887
### Genetic Diversity of Breast Cancer in Nigerian and Senegalese Patients

*A. Udoakang*[1,2], *F. Mbaye*[3], *A. Ademola*[4], *G. Arinola*[5], *T. Ogundiran*[4], *M. Sembene*[3]
[1]*University of Medical Sciences- Ondo City- Nigeria, Department of Biosciences and Biotechnology UNIMED, Ondo City-, Nigeria*
[2]*University of Ghana- West African Centre for Cell Biology of Infectious Pathogens, Department of Biochemistry- Cell and Molecular Biology, Legon-Accra, Ghana*
[3]*Universite Cheikh Anta Diop, Department of Animal Biology, Dakar, Senegal*
[4]*University College Hospital- Division of Oncology, Department of Surgery, Ibadan, Nigeria*
[5]*University of Ibadan- College of Medicine, Department of Chemical Pathology and Immunology, Ibadan, Nigeria*

**Introduction**

African women have more aggressive breast cancer with higher mortality rates than women of European descent. Although several therapeutic interventions exist, challenges of resistance, relapse, and poor survival outcomes persist, including methods of defining patients for different treatments. Microsatellite instability is a biomarker of clinical importance and plays a pivotal role in treatment response and selecting patients for treatment. Therefore, this study aimed to investigate the genetic diversity of breast cancer patients in Nigeria and Senegal by utilizing the microsatellite instability phenotype.

**Material and Methods**

Microsatellite analysis (*NR-21* and *NR-24*) of 100 histologically confirmed breast tumours, fifty each from Nigerian and Senegalese women, were phenotype using Sanger sequencing by capillary electrophoresis. The BioEdit software was used for the sequence alignment while statistical parameters were calculated using Arlequin and the significance level was assessed after 10,000 coalescence simulations. For each marker, the number of polymorphic sites, haplotype (h) and nucleotide ($\pi$) diversities were estimated and the haplotype and nucleotide differences calculated independently for each population.

**Results and Discussions**

The microsatellite analysis (*NR-21* and *NR-24*) showed high instability phenotypes in 52.2% and 67.4% of tumours from Nigeria and Senegal, respectively. There was more genetic variability for each marker within (*NR-21*: 91.52 and *NR-24*: 88.59) than between (*NR-21*: 8.48 and *NR-24*: 11.40) the two countries. High haplotype (*NR-21*: 0.7353 ± 0.0277, *NR-24*: 0.8981 ± 0.0268) and low nucleotide (*NR-21*: 0.0069 ± 0.0121; *NR-24*: 0.0026 ± 0.0072) diversities were observed for the two markers, suggesting rapid tumour growth and expansion. The evolution of multiple tumour clones at a higher rate has been reported as a natural outcome of genomic instability phenotype in cancers. These results show a promising outcome in the utilization of *NR-21* and *NR-24* for breast cancer diagnosis and prognosis in Nigeria and Senegal.

**Conclusion**

This study underscores the need for large-scale genetic studies in the African population. In conclusion, our study demonstrates the importance of characterizing molecular markers, including microsatellite markers, that could aid in stratifying breast cancer patients in Nigeria and Senegal for different therapeutic regimens such as immunotherapy.

### EACR2024-0901
### Gender-Specific Insights into HNSCC: Examining Sex Hormone Receptors Expression and Their Clinical Significance

© 2024 The Authors. Molecular Oncology is published by John Wiley & Sons Ltd on behalf of Federation of European Biochemical Societies

J. Čonkaš[1], J. Josić[1], J.K. Kranjčević[1], N. Milutin Gašperov[1], O. Vugrinec[2], I. Mumlek[3], A. Kvolik Pavić[3], M. Sabol[1], D. Leović[2], P. Ozretić[1]
[1]Ruđer Bošković Institute, Division of Molecular Medicine, Zagreb, Croatia
[2]Zagreb University Hospital Centre, Department of Otorhinolaryngology and Head and Neck Surgery, Zagreb, Croatia
[3]University Hospital Osijek, Department of Maxillofacial and Oral Surgery, Osijek, Croatia

**Introduction**

Head and neck squamous cell carcinoma (HNSCC) encompasses a heterogeneous spectrum of malignancies originating from the squamous cells lining the mucosal surfaces in the head and neck region. Beyond the established risk factors such as tobacco and alcohol consumption, along with infections by high-risk HPV strains, emerging research highlights the endocrine microenvironment as another risk factor contributing to the development of HNSCC. Notably, males exhibit a considerably higher relative risk for HNSCC, up to six times greater than females. This gender-specific susceptibility suggests the presence of either male-specific risk factors or protective hormonal and metabolic mechanisms in females. Consequently, we have initiated a comprehensive investigation into the involvement of both nuclear and membrane sex hormone receptors (SHRs) in HNSCC.

**Material and Methods**

Using quantitative real-time PCR (qPCR), we analyzed the mRNA expression of nuclear and membrane androgen (*AR*, *OXER1*, *CACNA1C*, *SLC39A9*, *GPRC6A* and *TRPM8*), estrogen (*ESR1*, *ESR2*, *GPER1* and *SCN2A*), and progesterone receptors (*PGR*, *PAQR5*, *PAQR6*, *PAQR7*, *PAQR8*, *PAQR9*, *PGRMC1* and *PGRM C2*) in 93 primary HNSCC tumors, 26 positive lymph nodes, and 42 healthy oral mucosa fresh tissue samples. The difference in relative gene expression levels was compared with the patient's age, sex, stage and grade of cancer, HPV status, and primary tumor site.

**Results and Discussions**

Our findings have revealed that the median age at the time of diagnosis was 64 years. Patients above this median age exhibited elevated expression levels of the *CACNA1C*, *SLC39A9*, *GPER1*, *SCN2A*, *PGR*, *PAQR 5* and *PAQR6* genes. Remarkably, among the patient cohort, 77.3% were men, while 22.7% were women. In women, there was a notable increase in expression levels of the *CACNA1C*, *OXER1*, *GPER1*, *SCN2A*, *PGR*, *PAQR6*, *PAQR7*, *PAQR8,* and *PAQR9* genes. Additionally, HPV DNA was detected in 18 (15.1%) out of 119 samples. The majority of tissue samples were categorized as stage IV, exhibiting a significant decline in the expression level of the *PAQR8* gene compared to stage II. Notably, there was a significant decrease in the expression levels of *AR* and *PGR* genes in grade 3 tumors compared to predominant grade 2 tumors.

**Conclusion**

This study represents the first comprehensive investigation into all three types of sex hormone receptors identified in HNSCC, including nuclear and membrane forms. Our findings underscore the notable contribution of all sex hormone receptor forms to the development of HNSCC tumors.

### EACR2024-0913
### Assessment of fecal microRNAs and microbiome profiles in longitudinal samples from colorectal cancer patients: prospects for non-invasive diagnosis and prognostic monitoring

D. Festa[1], B. Pardini[1,2], N.A. Keles[3], G. Ferrero[1,4], S. Tarallo[1,2], G. Francescato[1,4], V. Alberini[1,4], B.G. Tuna[5], O. Duzgun[6], A. Naccarati[1,2]
[1]Italian Institute for Genomic Medicine, Genetic and Molecular Epidemiology, Candiolo- Turin, Italy
[2]Candiolo Cancer Institute, FPO - i.r.c.c.s., Candiolo, Turin, Italy
[3]Yeditepe University, Department of Medical Biology, School of Medicine, Istanbul, Turkiye
[4]University of Turin, Department of Clinical and Biological Sciences, Turin, Italy
[5]Yeditepe University, Department of Biophysics- School of Medicine, Istanbul, Turkiye
[6]University of Health Sciences- Umraniye Training and Research Hospital, Department of Surgical Oncology, Istanbul, Turkiye

**Introduction**

Colorectal cancer (CRC), a leading cause of cancer mortality, requires the identification of new molecular markers for a more accurate diagnostic evaluation and patients monitoring. To enhance novel non-invasive diagnostic methods, our group identified a fecal microRNA (miRNA) signature able to accurately distinguish sporadic CRC from healthy controls. This study investigated the fecal miRNome and gut microbiome in CRC patients pre- and post-surgical tumor resection, examining the presence of molecular alterations before tumor resection (in comparison with controls) and their persistence after tumor removal. In parallel, the reliability and stability of fecal omics as biomarkers for follow-up study was assessed in healthy subjects who provided stool samples repeatedly over 6 months.

**Material and Methods**

We performed small RNA and shotgun metagenomic sequencing in stool samples of 34 CRC patients collected before surgery and after 6 months and 40 matched healthy controls. A similar multi-omics profiling was performed on stool from 6 volunteers who collected samples once a month for 6 times.

**Results and Discussions**

In pre-surgery samples of CRC patients, 494 miRNAs resulted differentially expressed (DE) respect to controls. Among them were included the 5 miRNAs (miR-149-3p, miR-607-5p, miR-1246, miR-4488, and miR-6777-5p) of the machine-learning-identified miRNA signature previously identified by us. Interestingly, in CRC the levels of the 5-miRNA signature returned similar to those of controls 6 months after surgery (for miR-1246, $p<0.001$ in comparisons between pre- and post-surgery). The analyses of longitudinal fecal metagenome data are currently ongoing to compare microbial species and metabolic pathways that characterize patients at diagnosis and at follow up. For the 6 volunteers repeated sampling, Principal Component Analysis based on 259 miRNAs

Molecular Oncology 18 (Suppl. 1) (2024) 1–495
© 2024 The Authors. Molecular Oncology is published by John Wiley & Sons Ltd on behalf of Federation of European Biochemical Societies detected with at least 15 reads in 4 timepoints, allowed a clear identification of each subject. 18 miRNAs were commonly detected in all subjects at all time points and could be used as references for stool miRNA analyses: notably, their levels were stable and comparable among the different subjects.

**Conclusion**

The longitudinal design of this study strengthens the hypothesis that miRNAs could be accurate biomarkers. The assessment of their temporal changes over time shows that their expression levels remain relatively stable in physiological conditions and revert to a "non-disease" status after tumor resection, similar to those of healthy subjects.

### EACR2024-0926
### Detection of DNA damage in circulating tumor cells harvested from blood samples of ovarian and prostate cancer patients

*A. Mezni[1], J. Kimber[1], R. Bourne[1], L. Vlietinck[1], M. Ciccioli[1], A.S. Pailhes-Jimenez[2]*
*[1]ANGLE plc, Cell Biology and Imaging, Guildford, UK*
*[2]ANGLE plc, Research and Development, Guildford, UK*

**Introduction**

Gamma H2AX (ɣ-H2AX) and Phospho KAP1 (pKAP1) are biomarkers used to detect activation of the DNA damage response (DDR) pathway. Monitoring of the DDR in human tissues and cells is valuable in establishing effectiveness of cancer therapies, with tumor biopsies being the standard method for assessing DDR marker expression. Obtaining tissue biopsies is challenging due to invasiveness, preventing repeated sampling to monitor response to therapy. Circulating Tumor Cells (CTCs), enriched from a liquid biopsy, allow for minimally invasive, real-time assessments of treatment response. This study used ANGLE's Parsortix® microfluidic technology, to harvest CTCs based on size and compressibility, combined with ANGLE's DDR immunofluorescence (IF) assays, to assess ɣ-H2AX and pKAP1 co-expression in CTCs isolated from blood of ovarian and prostate cancer patients.

**Material and Methods**

Analytical linearity, specificity and sensitivity of the assays were assessed by spiking cancer cells into healthy volunteers' blood drawn in Streck Cell-Free DNA tubes. Samples were processed on Parsortix® PR1 systems within 144 hours from draw and harvested onto ANGLE's CellKeep® slides for staining with ANGLE's DDR IF assays for detection of epithelial and/or mesenchymal CTCs, combined with either pKAP1 or ɣ-H2AX detection. Slides were imaged with a BioView imaging system. The same workflow (unspiked) was applied to 15 prostate and 13 ovarian cancer patients. Up to 6 draws, 2 blood tubes per draw (1 per assay), were taken per patient with 5-19 weeks between draws. At each draw, an updated clinical status was provided.

**Results and Discussions**

Analytical data demonstrated linearity over the range of 0 to ~500 cells, sensitivity >80% and specificity >99%. CTCs (≥1) were identified in at least 1 draw in 92% of ovarian and 87% of prostate cancer patients, with ≥1 DDR+ CTC identified in 58% of ovarian and 62% of prostate CTC+ patients. Among the CTC+ samples, concordance between ɣ-H2AX and pKAP1 was 75%. In the prostate cancer cohort, the presence of DDR+ CTCs was associated with response to treatment in 50% of the cases as reported at the time of blood draw, compared to 22% for DDR- CTCs (p<0.0001). A similar correlation was not observed in the ovarian cohort (p=0.69).

**Conclusion**

This research study demonstrates the ability to determine DDR status of CTCs isolated from a liquid biopsy using the Parsortix® system, highlighting it as a non-invasive tool to provide valuable insight into the efficacy of cancer therapies.

### EACR2024-0934
### Reduced occludin expression is related to unfavorable tumor phenotype and poor prognosis in many different tumor types: A tissue microarray study on 16,870 tumors

*S. Büyücek[1], N. Leege[1], F. Viehweger[1], C. Bernreuther[1], G. Sauter[1], R. Simon[1], E. Burandt[1], S. Steurer[1], C. Fraune[2], M. Lennartz[1]*
*[1]University Clinic Hamburg-Eppendorf, Department of Pathology, Hamburg, Germany*
*[2]Clinical Center Osnabrueck, Department of Pathology, Osnabrueck, Germany*

**Introduction**

Occludin is a tight junction component with a role in the regulation of formation, maintenance, stability, and barrier function of tight junctions. Reduced occludin expression in cancer cells has been linked to cancer progression.

**Material and Methods**

To comprehensively determine the prevalence of occludin expression in cancer, a tissue microarray containing 16,870 samples from 148 different tumor types and subtypes as well as 608 samples of 76 different normal tissue types was analyzed by immunohistochemistry.

**Results and Discussions**

Occludin immunostaining was observed in 10,746 (76.6%) of 14,017 analyzable tumors, including 18.9% with weak, 16.2% with moderate, and 41.6% with strong staining intensity. Occludin positivity was found in 134 of 148 tumor categories, and 108 tumor categories contained at least one strongly positive case. Occludin positivity was most frequently seen in adenocarcinomas (37.5-100%) and in neuroendocrine neoplasms (67.9-100%), slightly less common in squamous cell carcinomas (23.8-93%) and in malignant mesotheliomas (up to 48.1%) and only rarely in Non-Hodgkin's lymphomas (1-2%) and in mesenchymal tumors (3.4-41.7%). Reduced occludin staining was associated with advanced pT stage (p<0.0001), L1 status (p=0.0384), and absence of microsatellite instability (p<0.0001) in colorectal adenocarcinoma, advanced pT stage in pancreatic adenocarcinoma (p=0.005), poor ISUP grade (p<0.0001), advanced pT stage (p<0.0001), high UICC stage (p<0.0001), distant metastasis (p=0.0422), and

© 2024 The Authors. Molecular Oncology is published by John Wiley & Sons Ltd on behalf of Federation of European Biochemical Societies shortened overall (p=0.0045) or recurrence-free survival (p=0.0116) in clear cell renal cell carcinoma (RCC), high pT stage (p<0.0001) and UICC stage (p=0.0228), distant metastasis (p=0.0338), and recurrence-free survival (p=0.006) in papillary RCC, and advanced pT stage (p=0.0133) in serous high-grade ovarian cancer. Occludin staining was unrelated to parameters of tumor aggressiveness in breast, gastric, endometrial and thyroidal cancer.

**Conclusion**

Our data demonstrate significant levels of occludin expression in many different tumor entities and identify reduced occludin expression as a potentially useful prognostic marker in several tumor entities.

### EACR2024-0937

### Expression of TYMS in cancer: A tissue microarray study involving 9,015 cancers from 127 tumor entities

*F. Lutz[1], K. Möller[1], A.M. Luebke[1], G. Sauter[1], D. Dum[1], A.H. Marx[2], R. Simon[1], N. Gorbokon[1], M. Lennartz[1]*

*[1]University Clinic Hamburg-Eppendorf, Department of Pathology, Hamburg, Germany*

*[2]Academic Hospital Fuerth, Department of Pathology, Fuerth, Germany*

**Introduction**

Thymidylate synthase (TYMS) is essential for DNA synthesis and represents an important target for chemo-therapeutic drugs including 5-fluorouracil (5-FU) and methotrexate. Some studies suggest that tumors high levels of TYMS confer a certain degree of resistance against 5-FU. Although several authors have studied TYMS expression in different cancer types, the available literature data are highly discrepant.

**Material and Methods**

To better comprehend the role of TYMS expression in cancer, TYMS was analyzed by immunohistochemistry (IHC) on tissue microarrays (TMAs) containing 17,371 samples from 136 different tumor types as well as 608 samples of 76 different normal tissue types.

**Results and Discussions**

TYMS positivity found in few normal cell types, particularly in germinal centers and in the thymus as well as in hematopoetic cells of the bone marrow. TYMS staining was seen in 6,590 (42.9%) of the 15,361 analyzable tumors, and included 5,433 (35.4%) tumors with weak, 874 (5.7%) with moderate, and 283 (1.8%) with strong positivity. Of 136 tumor categories, 127 showed TYMS expression in at least one case, 71 tumor categories showed TYMS staining at least 50% of cases, and 56 tumor categories included at least one case with strong TYMS positivity. Highest rates of TYMS positivity were seen in B- and T-cell lymphomas (81-96.5%), sarcomas, carcinosarcomas and sarcomatoid carcinomas (33.3-92.5%), primary and metastatic malignant melanoma (70.5-90.7%), adenocarcinoma of the cervix (78.3%), squamous cell carcinomas of various sites of origin (57.1-77.9%), and testicular germ cell tumors (19.4-67.4%). High TYMS expression was linked to advanced stage (p=0.0097), high grade (p<0.0001) and triple negative receptor status (p<0.0001) in breast cancers of no special type, high grade (ISUP, Fuhrman, Thoenes, p≤0.003), high UICC stage (p=0.006), and lymph node metastasis (p=0.0288) in clear cell renal cell carcinoma, high grade (p≤0.0422), and lymph node metastasis (p=0.0045) in papillary renal cell carcinoma, high grade (p<0.0001) in endometrioid endometrium carcinoma, advanced stage (p=0.0061) in high-grade serous ovarian carcinoma, and advanced stage (p<0.0001) and microsatellite instability (p<0.0001) in colon cancers.

**Conclusion**

TYMS is often overexpressed as compared to normal tissues in a broad range of different cancer entities. Our data suggest that high-level of TYMS expression is a feature of cancer aggressiveness in many different cancer types.

### EACR2024-0957

### Stimulator of Interferon Genes (STING) is frequently expressed in tumor cells of a wide range of human cancer types: A tissue microarray study on 18,000 tumors

*A. Menz[1], J. Zerneke[1], S. Büyücek[1], M. Kluth[1], C. Hube-Magg[1], G. Sauter[1], M. Lennartz[1], R. Simon[1], S. Minner[1], K. Till[2]*

*[1]University Clinic Hamburg-Eppendorf, Department of Pathology, Hamburg, Germany*

*[2]Clinical Center Osnabrueck, Department of Pathology, Osnabrueck, Germany*

**Introduction**

Stimulator of Interferon Genes (STING) plays a crucial role in the innate immune response to DNA derived from pathogens such as viruses or bacteria. Altered STING expression has been linked to adverse tumor features in some tumor types. Targeting the STING pathway has emerged as a promising approach for cancer therapy. A systematic analysis of STING expression in human cancers is currently lacking.

**Material and Methods**

To study STING expression in cancer, a tissue micro-array containing 18,001 samples from 139 different tumor types and subtypes as well as 608 samples of 76 different normal tissue types was analyzed by immunohistochemistry.

**Results and Discussions**

STING immunostaining was observed in 8,908 (58.1%) of 15,341 analyzable tumors, including 27.2% with weak, 13.1% with moderate, and 17.8% with strong staining intensity. STING positivity was found in 130 of 139 tumor categories, and 96 tumor categories contained at least one strongly positive case. Particularly high rates of STING positivity occurred in squamous cell carcinomas of different sites of origin (up to 96%), malignant mesothelioma (88.5%-95.7%), ductal adenocarcinoma of the pancreas (94.9%), pulmonary adenocarcinoma (90.3%), cervical adenocarcinoma (90.0%), serous high-grade ovarian cancer (86.0%), anaplastic thyroid carcinoma (82.9%), colorectal adenocarcinoma (75.2%), and adenocarcinoma of the gallbladder (68.8%). Strong STING staining was linked to a positive hormone receptor status (p<0.0001) and shortened overall survival (p=0.0196) in breast cancer of no special type, early tumor recurrence (0.0352), advanced pT (p<0.0001) and UICC stage (0.006), and high ISUP grade (p=0.0002) in

Molecular Oncology 18 (Suppl. 1) (2024) 1–495

© 2024 The Authors. Molecular Oncology is published by John Wiley & Sons Ltd on behalf of Federation of European Biochemical Societies clear cell renal cell carcinoma (RCC), presence of lymph node metastases in papillary RCC (p=0.0353), thyroid cancer (p=0.0074), and hepatocellular cancer (p=0.0435), high CD8+ T-cell density in endometrioid ovarian cancer, pancreatic adenocarcinoma (p=0.0489), and low CD8+ density in gastric adenocarcinomas (p=0.0013). Across all tumor types, strong STING positivity was linked to a high fraction of PD-L1 positive tumor and tumor-associated immune cells (p<0.0001 each).

**Conclusion**

STING expression can be detected immuno-histochemically in many tumor types and is associated to tumor progression and aggressiveness particularly in clear cell renal cell carcinomas. The role of STING expression in the immune environment of tumors may be complex, however, and variable in different tumor types.

### EACR2024-0985
### Mucin 6 (MUC6) is expressed in a subset of prostate cancers but unrelated to tumor aggressiveness or patient prognosis

*H. Plage[1], S. Dwertmann Rico[2], R. Simon[2], H. Heinzer[3], A. Haese[3], S. Minner[2], G. Sauter[2], N. Heckmann[2], T. Schlomm[3], M. Graefen[3]*
*[1]Charité - Universitätsmedizin Berlin- Corporate Member of Freie Universität Berlin, Department of Urology, Berlin, Germany*
*[2]University Clinic Hamburg-Eppendorf, Department of Pathology, Hamburg, Germany*
*[3]Martini-Clinic- Prostate Cancer Center- University Medical Center Hamburg-Eppendorf, Department of Urology, Hamburg, Germany*

**Introduction**

Mucin 6 (MUC6) is a secreted gel-forming mucin covering the surfaces of gastrointestinal tissues, seminal vesicles, and other organs. The role of MUC6 in prostate cancer is not well-established. Some studies have reported variable expression levels of MUC6 in prostate cancer tissues compared to normal prostate tissue, and others suggested potential correlations between MUC6 expression and clinicopathological parameters of prostate cancer, such as tumor grade, stage, and patient prognosis. However, these findings are inconsistent across studies.

**Material and Methods**

To learn more about the role of MUC6 in prostate cancer, a tissue microarray containing 17,747 prostate cancer spots with associated follow-up and molecular data was analyzed by immunohistochemistry.

**Results and Discussions**

Positive MUC6 immunostaining was infrequent. Detectable staining was found in 1,283 (10.3%) of 12,512 interpretable tumors, including 3.6% with weak, 2.5% with moderate and 4% with strong staining intensity. Strong MUC6 staining was weakly associated with high tumor stage (p=0.0229), high classical (p=0.0421) and quantitative (p=0.0442) Gleason grade, and high preoperative PSA levels (p=0.026). MUC6 was unrelated to prostate cancer patient prognosis. The molecular database attached to the TMA allowed to search for associations between MUC6 and the genomic phenotype. MUC6 staining was less frequent in prostate cancers harboring certain genomic deletions, including deletions of 16q24 (p=0.02), 17p13 (p=0.0281), 5q21 (p=0.0162), and 6q15 (p=0.0215). No differences in MUC6 expression were found in the subsets of prostate cancers with presence or absence of the TMPRSS:ERG gene fusion. MUC6 staining was also unrelated to androgen receptor expression and cell proliferation measured by the Ki67 labeling index.

**Conclusion**

MUC6 expression is found in about 10% of prostate cancers, but largely unrelated to histo-pathological parameters of tumor aggressiveness or patient prognosis.

### EACR2024-1016
### Development of a peripheral blood culture assay to discover and clinically measure low-baseline DNA-Damage Response (DDR) pharmacodynamic biomarkers

*A. Karapetsas[1], U. Polanska[1], L. Oplustil O'Connor[1], L. Wang[2], M. Glover[2], S. Hess[2], E. Harrington[1], P. Marco Casanova[1]*
*[1]AstraZeneca plc, Early Oncology Translational Medicine, Cambridge, United Kingdom*
*[2]AstraZeneca plc, Discovery Sciences Dynamic Omics, Gaithersburg, United States*

**Introduction**

Patients' blood can serve as surrogate tissue that enables longitudinal analysis of drug target engagement in clinical studies. Some key pharmacodynamic (PD) biomarkers, such as phosphorylated targets of the DNA Damage Response (DDR) are expressed at very low levels in Peripheral Blood Mononuclear Cells (PBMCs) and this can limit the use of this surrogate tissue, but ex vivo PBMC stimulation can overcome this challenge. Thus, our aim was to develop a clinically amenable, peripheral blood-based assay for ex vivo DDR activation resulting in measurable PD biomarkers in patients' PBMCs.

**Material and Methods**

We implemented a blood tube culture system containing PBMC mitogenic stimulants and various DNA damaging agents that resulted in a robust activation of the DDR in a cohort of healthy volunteers' blood. DDR effects were monitored by standard immunoblot and orthogonally confirmed and complemented by mass spectrometry-based phosphoproteomics.

**Results and Discussions**

Using healthy volunteer's blood samples, we established a blood culture system that results in the potent induction of classic DDR phospho-biomarkers (e.g., pATM, pDNAPKcs, pRAD50, γH2AX; >10-fold increase) and their robust decrease when the blood is supplemented with DDR inhibitors (>8-fold decrease). Moreover, we integrated mass spectrometry-based phospho-proteomics to further investigate and unveil novel DDR biomarkers with the highest dynamic range in PBMCs. We identified 21,378 phosphosites from 3,645 phosphoproteins out of 6,170 total proteins. 42% of the 185 phosphosites upregulated upon DDR induction decreased their levels with AZ DDR inhibitors. Finally, we identified some bona fide DDR biomarkers in PBMCs that are

© 2024 The Authors. Molecular Oncology is published by John Wiley & Sons Ltd on behalf of Federation of European Biochemical Societies being implemented as PD biomarkers in current DDR-focused clinical studies.

**Conclusion**

This is a technically feasible clinical assay to be applied on pre/post- treatment blood samples to assess longitudinally the activity of DDR inhibitors and guide dose selection in Phase I clinical studies. This novel approach could be broadly adapted to other disease settings where low-baseline PD biomarkers are key to inform drug effects.

### EACR2024-1026
### Comprehensive liquid biopsy analysis for monitoring NSCLC patients under second-line osimertinib treatment

*A. Ntzifa[1], T. Marras[1], G. Kallergi[2], A. Kotsakis[3], V. Georgoulias[4], E. Lianidou[1]*
*[1]National and Kapodistrian University of Athens, Analysis of Circulating Tumor Cells Lab, Lab of Analytical Chemistry, Department of Chemistry, Athens, Greece*
*[2]University of Patras, Laboratory of Biochemistry/ Metastatic Signaling- Section of Genetics- Cell Biology and Development- Department of Biology, Patra, Greece*
*[3]General University Hospital of Larissa, Department of Medical Oncology, Larissa, Greece*
*[4]Metropolitan General Hospital of Athens, First Department of Medical Oncology, Athens, Greece*

**Introduction**

The aim of this study was to perform a comprehensive LB analysis for monitoring NSCLC patients under second-line osimertinib treatment, by combining plasma-cfDNA and CTC analysis to identify molecular alterations at resistance and potential targets for subsequent treatments.

**Material and Methods**

Peripheral blood from 30 NSCLC patients was collected before treatment (baseline) and at disease progression (PD). Plasma-cfDNA was analyzed for DNA mutations (*EGFR*, *PIK3CA*, *KRAS*-G12C, *BRAF*-V600E) using digital PCR and for DNA methylation (*RASSF1A*, *BRMS1*, *SLFN1*, *RASSF10*, *APC*, *RARβ*, *FOXA1*, *WIF-1*, *SHISA3)* using methylation specific PCR. CTCs were enriched from identical blood draws using Parsortix (Angle, UK). CTC-derived gDNA was analyzed for the same DNA mutations and methylation markers. CTCs were analyzed for *HER2* and *MET* amplification with FISH. RT-qPCR was performed in CTCs-derived mRNA for *CK-8*, *CK-18*, *CK-19*, *VIM*, *TWIST-1*, *AXL*, *ALDH-1*, *PD-L1*, *PIM-1*, *B2M* genes. PD-L1 was detected in CTCs enriched using ISET (Rare cells, France) using immunofluorescence (IF).

**Results and Discussions**

*EGFR* mutation analysis in plasma-cfDNA and CTCs have shown complementary information; T790M was detected only in CTC from three patients at PD, but not in paired plasma-cfDNA. *PIK3CA* mutations were detected only in plasma-cfDNA but not in CTCs. *KRAS*-G12C and *BRAF*-V600E were not detected in any sample. *MET* amplification was detected in CTCs of two patients at baseline whereas *HER2* amplification was detected in CTCs of three patients at baseline and in one patient at PD. DNA methylation between CTCs and cfDNA revealed low concordance. Data from IF and RT-qPCR for the presence of PD-L1 positive CTCs in matched samples revealed high detection rates suggesting a theoretical background for immunotherapy in EGFRm NSCLC patients. *PD-L1*, *PIM-1* and *AXL* expression in CTCs indicate a potential benefit of targeted therapies for NSCLC patients who relapse following osimertinib treatment.

**Conclusion**

Our results indicate the importance of complementary information obtained through parallel analysis of CTC and ctDNA. Comprehensive LB analysis efficiently represents the heterogeneous molecular landscape and provides prominent information on subsequent treatments for NSCLC patients progressing with osimertinib based on different druggable molecular alterations.

### EACR2024-1041
### CCNE1 amplifications and overexpression display intra-patient heterogeneity and are not mutually exclusive with HR deficiency in ovarian high-grade serous carcinoma

*E. Kahelin[1,2], J. Oikkonen[1], K. Lavikka[1], G. Micoli[1], Y. Li[1], S. Hautaniemi[1], L. Kauppi[1], J. Hynninen[3], A. Virtanen[1,2]*
*[1]University of Helsinki, Research Program in Systems Oncology, Research Programs Unit, Faculty of Medicine, Helsinki, Finland*
*[2]Helsinki University Hospital, Department of Pathology, University of Helsinki and HUS Diagnostic Center, Helsinki, Finland*
*[3]University of Turku and Turku University Hospital, Department of Obstetrics and Gynaecology, Turku, Finland*

**Introduction**

Ovarian cancer is the eighth most common cancer diagnosis and cause of cancer deaths in women. High-grade serous carcinoma (HGSC) is the most common subtype of epithelial tubo-ovarian cancer. Currently the only implemented biomarker in HGSC is genomic homologous recombination (HR) deficiency (HRD) that associates to better chemotherapy and poly (ADP-ribose) polymerase (PARP) inhibitor responses. *Cyclin E1 (CCNE1)* amplification is found in about 20 % of HGSC cases and high-level amplification and overexpression have been linked to shorter overall survival. Moreover, *CCNE1*-amplified cases are often assumed to be HR proficient and CCNE1 has been suggested to be a promising biomarker to identify the largest subgroup of HR proficient HGSC (Kang et al. Cancer 2023).

**Material and Methods**

We used data from an on-going, observational clinical trial DECIDER that provides longitudinal and multi-site data from HGSC patients (www.deciderproject.eu). Whole-genome sequencing was performed for 1116 samples from 233 patients. HRD was estimated using ovaHRDScar and single base substitution signature 3 (Sig3, Koskela et al. Gynecol Oncol. 2024). CCNE1 amplification was defined by a copy number $\geq 8$ and $\geq 2$ times ploidy. For Cyclin E1 protein expression analysis by immunohistochemistry (IHC) tumors from two

© 2024 The Authors. Molecular Oncology is published by John Wiley & Sons Ltd on behalf of Federation of European Biochemical Societies different sites (e.g., adnexal and omental) were selected to explore intra-patient heterogeneity. As previously suggested, overexpression was defined by > 60 % of cells and ≥ 5 % with strong intensity.

**Results and Discussions**

Among the 233 patients, we identified 48 patients (21%) with at least one CCNE1-amplified tumor sample using the selected threshold. Nine (19%) of the 48 patients had both amplified and non-amplified samples. Further, 32 % of CCNE1-amplified samples (31/96 samples, 19/48 patients) were also estimated as HRD (ovaHRDscar). No *BRCA1/2*-mutations were detected among the patients with CCNE1-amplified samples. Cyclin E1 protein expression was detected in 30 samples (77 %) among 39 currently evaluated samples of 26 patients with CCNE1 amplification.

**Conclusion**

Our results show that genomic HRD estimates, CCNE1 amplifications and overexpression display intra-patient heterogeneity. On a patient level, CCNE1 amplification and overexpression are mutually exclusive with BRCA1/2 mutations but not with HR deficiency. As HRD is already implemented as a biomarker and CCNE1 amplification is emerging as treatment target, the heterogeneity should be further explored to find optimal combinations of these biomarkers.

### EACR2024-1047

### lncRNAs: a promising new class biomarkers for pediatric T-cell acute lymphoblastic leukemia

F.M. Orlandella[1]

[1]Università degli studi di Napoli "Parthenope"- Naples-Italy, Dipartimento delle Scienze Mediche- Motorie e del Benessere, Naples, Italy

**Introduction**

B-cell and T-cell acute lymphoblastic leukemias (ALL) are characterized by different initiating genetic alterations, different prognoses and different therapeutic approach. For both of them, the diagnosis and the stratification of recurrence risk are a crucial step during the management of these patients occurring on the base of genetic aberrations and on the evaluation of the minimal residual disease. However, the knowledge of the aberrant expression of long non-coding RNAs (lncRNAs) could provide further information regarding this malignant transformation and the clinical outcomes. Thus, this study aims to unveil the lncRNAs differentially expressed in childhood B-ALL and T-ALL delving deeper their potential application as biomarkers in these patients.

**Material and Methods**

NGS technology was performed on 9 B-ALL and 6 T-ALL pediatric patients and on naive B and T lymphocytes from cord blood of healthy donors to detect lncRNAs differentially expressed. Public datasets and q-RT-PCR were carried out to confirm the results obtained. Functional experiments in T-ALL cell line were performed to investigate the role of the selected lncRNA in the transformation of T-cells.

**Results and Discussions**

The transcriptomic results unveil a set of lncRNAs (with an adjusted p-value < 0.05) able to discriminate B-ALL, T-ALL, B- and T naïve lymphocytes in four distinct clusters. From the lncRNA signature associated with childhood T-ALL patients, we found 187 lncRNAs upregulated and 164 downregulated compared to naïve T cells. Focusing on up-regulated lncRNAs, q-RT-PCR showed that HHIP-AS1, an inhibiting factor of Hedgehog signaling, was also upregulated in T-ALL compared to healthy subjects. Moreover, we found that the higher expression of this lncRNA occurs in younger patients. Preliminary data suggested that the silencing of HHIP-AS1 induced a reduction in cell proliferation rate in T-ALL cell line (RPMI-8402) compared to control cells.

**Conclusion**

We identified a lncRNAs profile of T-ALL patients suggesting their potential role as biomarkers and novel therapeutic strategy for this malignancy.

*This work was funded by the Italian Ministry of Health (Ricerca Finalizzata- Giovani Ricercatori) with a Project Code: GR-2021-12372945.*

### EACR2024-1052

### Towards liquid biopsy for glioblastoma: relying on plasma extracellular vesicles for tumor diagnosis and monitoring

E. Zaccheroni[1], E. Costanzi[1], D. Osti[1], S. Cavallo[1], M. Del Bene[2], F. Di Meco[2], G. Pelicci[1]

[1]IEO, Molecular Oncology, Milan, Italy

[2]Istituto Neurologico Besta, Neuro-oncology, Milan, Italy

**Introduction**

Glioblastoma (GBM) stands out as the most aggressive brain tumor in adults. Its diagnosis and follow-up rely on neuroimaging and tissue sampling, with limited specificity and sensitivity. The risks associated to surgical resection, and the evolution of GBM pose significant challenges to monitoring the disease by solid biopsy. Addressing these challenges requires the identification of circulating biomarkers for non-invasive GBM detection and monitoring. Extracellular Vesicles (EVs) cross the blood brain barrier and reflect GBM burden. This makes them a promising target for the assessment of GBM presence and status.

**Material and Methods**

We isolate EVs by Size Exclusion Chromatography (SEC) from 2mL of plasma, and characterize them by immunoblot, flow cytometry and transmission electron microscopy. We measure EVs concentration and size by Tunable Resistive Pulse Sensing (TRPS) in 50 GBM patients before surgery (baseline) and in non-GBM controls, including 100 healthy subjects and 50 patients with brain malignancies that mimic GBM at imaging. We monitor changes in EV concentration over time in 44 GBM plasma samples to capture GBM evolution. We profile EVs surface proteins by bead-based multiplex flow cytometry, and the total EV proteome by mass spectrometry in healthy individuals, and in matched pre- and 72h post-operative GBM to identify GBM-specific biomarkers.

**Results and Discussions**

The concentration of plasma EVs is higher in baseline GBM with respect to controls. The size distribution indicates that larger EVs are enriched in GBM.EV concentration and size significantly drop in matched 72h post-operative GBM, serving as potential indicators of

© 2024 The Authors. Molecular Oncology is published by John Wiley & Sons Ltd on behalf of Federation of European Biochemical Societies

GBM burden. Results from multiplex flow cytometry demonstrates overlapping expression profiles in EVs from GBM and controls. This is likely due to the presence of non-tumor EVs in circulation that can dilute GBM-derived EVs, making it challenging to distinguish tumor from non-tumor EVs based solely on surface markers expression. Among the few differentially expressed surface proteins, the EV markers CD63 and CD81, the T-cell markers CD8 and HLA-DRDPDQ are all enriched in GBM. Profiling of EV proteome identified 117 proteins specifically upregulated in GBM samples and enriched in pathways of the complement cascade.

**Conclusion**

The assessment of plasma EVs parameters is a reliable, non-invasive approach which may complement traditional imaging to enhance diagnostic accuracy and guide personalized treatment strategies, supporting the implementation of liquid biopsy for GBM care.

### EACR2024-1069
### Observation and characterization of the cell free DNA released by organoids from colorectal cancer patients

*A. Mirandola[1], J. Cartry[2], S. Bedja[2], F. Jaulin[2], A.R. Thierry[1]*
*[1]INSERM, U1194-Institut de Recherche en Cancérologie de Montpellier, Montpellier, France*
*[2]INSERM, U1279-Institut Gustave Roussy- Université Paris-Saclay, Villejuif, France*

**Introduction**

Patient-derived organoids (PDO) are three-dimensional self-organized and differentiated structures derived from tumor tissues and have emerged as promising tools in oncology. Investigating DNA in the supernatant of PDOs offers a novel approach to select personalized treatments based on the patient's mutational profile, and to explore mitochondrial dysfunction. Our team already detected cell-free DNA (cfDNA) in cell cultures (Otandault et al. 2020). Knowledge on cfDNA is less characterized as compared to circulating DNA (cirDNA). CirDNA is of significant interest, enabling non-invasive monitoring of the disease, identification of specific genetic mutations associated with tumors, and customization of treatment strategies for cancer patients. This collaborative work aims at evaluating cfDNA from PDO culture as a tool to determine in a “ex vivo” setting the appropriate treatment to patients.

**Material and Methods**

PDO were derived from a clinical study on metastatic colorectal cancer patients. We examined 21 supernatants from 13 patients. Cell-free nuclear and mitochondrial DNA were specifically quantified by qPCR. Mutations were previously detected in cirDNA samples from matched patients. We identified actionable mutations in genes of interest (KRAS, TP53, EGFR and PIK3CA). Mutation status and mutation allele frequency were tested using Inplex® test (EP2011 / 065333) and NGS in the supernatant of PDO.

**Results and Discussions**

Level of cell-free nuclear DNA in the supernatant of PDOs showed significant variations upon patient and the number of PDO culture passages. Concentrations of cfDNA ranged from less than 1 ng/mL to over 290 ng/mL. We could detect in PDO culture supernatant at least one mutation in 72% of patients. All the mutations detected with Inplex® were confirmed by NGS analysis of the supernatant. Additionally, we discovered the presence of cell-free mitochondria released by PDO cells in the culture medium in over 75% of the supernatant and in 85% of the patients.

**Conclusion**

Except to one report, this is the first observation of cfDNA from PDO culture. We quantify cell-free nuclear and mutant DNA. Inplex® facilitates rapid and highly sensitive tracking of a selected set of mutations over time. Lastly, the discovery of free mitochondria in these supernatants opens avenues for personalized mitochondrial dysfunction studies.

*-Otandault et al., British journal of cancer vol. 122,5 (2020) PMC7054557*

### EACR2024-1082
### Targeted sequencing of Circulating Tumor Cells captured by Parsortix® System enables low frequency variant analysis with NuProbe VarMap™ Pan-Cancer NGS Panel

*F. Mahbubinejad[1], M. Coates[1], A. Utting[1], M.C. Geraldizo[1], M. Vasiliadis[1], K. Nguyen[2], J. Tran[2], W. Li[2], A. Papadimitropoulos[1], A.S. Pailhes-Jimenez[1]*
*[1]ANGLE Europe Limited, Research and Development, Guildford, United Kingdom*
*[2]NuProbe USA, R&D Services, Houston, United States*

**Introduction**

ANGLE's Parsortix® system is an FDA-cleared system for capturing and harvesting viable circulating tumor cells (CTCs) from blood samples of metastatic breast cancer patients for user-validated downstream analysis. This research study evaluated the analytical sensitivity of NuProbe VarMap™ Pan-Cancer NGS Panel on CTC samples, isolated with Parsortix system, and cell-free DNA (cfDNA) separated from the same blood sample.

**Material and Methods**

Two cell lines, SKMEL28 and H1975, were spiked simultaneously into healthy donor blood at varying concentrations (2 to 50 input cells). In parallel, blood samples from 10 breast, 10 lung, 10 prostate and 10 ovarian cancer patients were also collected. Blood samples were processed using the Parsortix system to capture CTCs. Enriched CTCs (or cell lines) and matched cfDNA were analysed by multiplex PCR-based target enrichment using the NuProbe 61-gene VarMap™ Pan-Cancer NGS Panel, followed by next-generation sequencing using Illumina sequencing system. Variants with allele frequencies equal to or greater than 0.1% were reported as positive calls.

**Results and Discussions**

Positive mutation calls were identified in expected genes such as BRAF, EGFR, TP53, PTEN and PIK3CA when a minimum of 5 cancer cells were spiked into blood samples prior to processing with Parsortix system. No somatic variants with allele frequencies ≥0.1% were identified in unspiked blood samples for the afore-mentioned targets, demonstrating the analytical specificity of the assay. In metastatic breast, lung and ovarian cancer patient samples, mutations were identified

Molecular Oncology 18 (Suppl. 1) (2024) 1–495
© 2024 The Authors. Molecular Oncology is published by John Wiley & Sons Ltd on behalf of Federation of European Biochemical Societies in CTCs and cfDNA fractions. Mutations observed in CTC-fraction only were identified in 60%, 70% and 70% of the cohort respectively. A low number of shared mutations were identified between CTC and cfDNA fractions, 9%, 11% and 26% respectively, in matched patient samples. Some targets identified in the CTC-fraction are known to be cancer-related, such as ESR1, PIK3CA or EGFR, showing potentially actionable targets identifiable in CTCs enriched using the Parsortix system but not in cfDNA extracted from the same blood sample.

**Conclusion**

The analysis highlights the complementary value of profiling both CTC-DNA and cfDNA in cancer patients. This study identified potentially actionable biomarkers in CTCs enriched using the Parsortix system, from cancer patient samples. Analysing the genetic profile of both CTCs and cfDNA, concomitantly, has the potential to better guide personalised treatment selection and monitoring of therapeutic response outcomes.

### EACR2024-1092

### Spatial Localization of Specific Nucleic Acid Sequences in FFPE Tissues with AMPIVIEW™ RNA Probes, Powered by LoopRNA™ ISH Technology

*N. Chandel[1], F. Mazzoni[1], H. Pohl[2], K. Tian[1], J. Coleman[1]*

*[1]Enzo Life Sciences- Inc., Research and Development, Farmingdale, United States*

*[2]Enzo Life Sciences- Inc., Application Scientist, Lausen, Switzerland*

**Introduction**

The analysis of nucleic acid biomarkers in clinical specimens enables the study of the progression and etiology of many cancers, which depend largely upon mutation, dysregulation, overexpression and deletion of these key biomarkers, which can consequently become important therapeutic targets. in situ hybridization (ISH) is a method used for the detection of the temporal and spatial localization of specific nucleic acid targets within their morphological context in formalin-fixed, paraffin-embedded (FFPE) cell and tissue samples.

AMPIVIEW™ ISH RNA probes are uniquely designed with the precision of targeted, sequence-specific RNA, powered by Enzo's LoopRNA™ ISH technology to deliver superior sensitivity for the detection of not only viral infections such as human papilloma virus (HPV), but also the expression of endogenous genes such as EGFR and HER-2. To validate the specific biomarker detection, we chose to study the expression of PD-1, PD-L1, GATA3, EGFR and HER-2 in tissue samples.

**Material and Methods**

Manual and automated protocols were performed starting from baking and deparaffinization with xylene or dewaxing agent, before rehydrating the cells or tissue samples, followed by antigen retrieval and proteinase K treatment. Hybridization was performed with AMPIVIEW™ probes according to product specifications. Since the AMPIVIEW™ RNA probes are conjugated with biotin, digoxigenin, or dinitrophenol (DNP) labels, amplification and detection can be performed with nanopolymer-based detection reagents widely used in immunohistochemistry (IHC) and compatible chromogens.

**Results and Discussions**

Aforementioned methods of staining were used to resolve mRNA expression of EGFR, PD-1 and PD-L1 in mantle lymphoma, lung carcinoma, ovarian, and prostate cancers; and HER2 and GATA3 in graded breast cancers.

**Conclusion**

AMPIVIEW™ probes enable robust, specific detection of target mRNAs in cancerous cells across multiple human tissues. Detection via chromogenic or fluorescent means, singly or in multiplex combination, can impart crucial information regarding expression levels, localization, and correlative interplay between biomarkers in malignancies on a cellular and whole-tissue basis. In the current study, these relationships are represented by the specific detection of EGFR, PD-1, PD-L1, HER2 and GATA3 mRNAs across several different organs and representative malignancies.

### EACR2024-1106

### Refining liquid biopsy with the 6-base genome: Generating more information from cell free DNA

*T. Charlesworth[1], F. Puddu[2], R. Crawford[3], N. Harding[2], A. Johansson[2], E. Lleshi[3], A. Modat[3], J. Scotcher[2], J. Teyssandier[2], P. Creed[2]*

*[1]biomodal Limited, RnD, Cambridge, United Kingdom*

*[2]biomodal, Ifx, Cambridge, United Kingdom*

*[3]biomodal, RnD, Cambridge, United Kingdom*

**Introduction**

Liquid biopsy for profiling cell free DNA (cfDNA) holds huge promise to transform how we detect and identify cancer, and track residual disease. However, a standard blood draw yields only ~10 ng of cfDNA, of which circulating tumour DNA is a minority. Obtaining maximum information from limited samples is, therefore, crucial. Standard genetic sequencing can only provide actionable mutation information at limited loci. Including epigenetic variation at single-base resolution, by measuring 5-methylcytosine (5mC) and 5-hydroxy-methylcytosine (5hmC), provides the ability to probe more loci and could enable more sensitive liquid biopsy assays from cfDNA.

**Material and Methods**

Current methods for sequencing the epigenome sacrifice genetic information or are unable to measure 5mC and 5hmC simultaneously. Using a new technology which provides the 6-base genome (full genetics and 5mC distinguished from 5hmC) at single base resolution, we obtained a complete, integrated dataset derived from 87 individuals (healthy volunteers or colorectal cancer (CRC) patients) from 10ng of cfDNA

**Results and Discussions**

Using synthetic controls and reference DNA samples, we showcase accurate measurement of 5-mC and 5-hmC as well as high accuracy for SNV calling and demonstrate the derivation of cfDNA fragment characteristics. In addition, the 6-base genome reveals novel biomarkers that would be invisible using existing approaches including variant associated methylation and enhancer state. We highlight examples of novel biomarkers that

© 2024The Authors. Molecular Oncology is published by John Wiley & Sons Ltd on behalf of Federation of European Biochemical Societies may have utility in detecting early-stage cancer or predicting disease progression.

**Conclusion**

With the 6-base genome it is possible to obtain the most comprehensive multi-modal information, including SNPs, 5mC, 5hmC, fragmentomics, copy number variation and novel two-dimensional biomarkers, from a single low-input sample of cfDNA. The simultaneous identification of multiple modalities enhances the signal available for earlier detection of cancer.

### EACR2024-1115
### Analytical performance of TruSight™ Oncology 500 ctDNA v2: Improved sensitivity for small nucleotide variants with reduced DNA input requirements and reduced hands-on time

*N. Sedlyarova[1], M. Cuadras[2], M. Wang[2], A. Mai[2], M. Kellogg[2], J. Beytebiere[2], R. Garcia[2], T. Pawlowski[2], L. Watson[2], J.S. LoCoco[2]*
*[1]Illumina, Research Segment Marketing, Vienna, Austria*
*[2]Illumina, Product Development, San Diego, USA*

**Introduction**

TruSight™ Oncology 500 ctDNA version 2 (TSO 500 ctDNA v2, Illumina, San Diego, CA; Research Use Only) is an NGS-based research assay that utilizes the same gene panel as TSO 500 ctDNA (targeting 523 cancer-related genes), with similar analytical sensitivity, but using lower cell free DNA (cfDNA) input. The TSO 500 ctDNA v2 assay incorporates unique molecular identifiers (UMIs) for error suppression, and the workflow utilizes a recommended input of 20ng of cfDNA into a single hybrid capture workflow resulting in a decreased overall workflow hands-on time.

**Material and Methods**

Libraries generated from patient cfDNA, commercially available cfDNA-like reference materials, and nucleosome (npDNA) prepared from cell lines harboring variants of interest were sequenced on the NovaSeq™ 6000 instrument (Illumina) and analyzed with the DRAGEN™ TSO500 ctDNA Analysis Software v2.1. The software detects small nucleotide variants, gene amplifications and deletions, gene rearrangements and determines a Tumor Mutational Burden (TMB) score and Microsatellite Instability (MSI) status. Assay analytical sensitivity was evaluated using titrations at specific variant allele frequencies (AF), or specific tumor fractions. Analytical specificity was evaluated testing cfDNA replicates from healthy donors. Assay precision was evaluated testing a panel of samples across multiple operators, reagent lots and sequencing instruments.

**Results and Discussions**

TSO 500 ctDNA v2 consistently detected key pathogenic small variants at AFs ≤ 0.2% using a 20ng input. Detection of indels and gene fusions with > 95% sensitivity was observed at AFs ≤ 0.4%. Testing of reference samples demonstrated sensitive detection of 1.25-fold change gene amplifications and 0.8-fold change gene deletions. Testing of cfDNA from samples from healthy donors showed that TSO 500 ctDNA v2 can achieve a > 99.999% assay specificity. Precision evaluation showed >99% precision for targeted small DNA variant calls.

**Conclusion**

Together these results demonstrate > 99.999% analytical specificity with high analytical sensitivity to AFs ranging from 0.2% to 0.4% depending on variant class. This performance was achieved using a lower recommend input of 20ng of cfDNA and utilizes a workflow that reduces total turn-around time and hands-on time while still delivering all key biomarker data available from the TSO 500 gene panel.

### EACR2024-1150
### Correlation between serum fascin concentration and its expression in tumor tissue

*M.D. López Abellán[1], F. Postigo-Corrales[2], A. Albaladejo-González[2], D. Torres-Moreno[2], M.A. Beltrán-Videla[2], M. Losada-Echeberría[3], A. Sánchez-Espinosa[2], E. Rodríguez-Braun[4], A.M. Hurtado-López[2], P. Conesa-Zamora[1]*
*[1]Hospital General Universitario Santa Lucía, Análisis Clínicos, Cartagena, Spain*
*[2]Hospital General Universitario Santa Lucía, Anatomía Patológica, Cartagena, Spain*
*[3]Instituto de Investigación- Desarrollo e Innovación en Biotecnología Sanitaria de elche, Diseño y Desarrollo de Moléculas Bioactivas, Cartagena, Spain*
*[4]Hospital General Universitario Santa Lucía, Oncología Médica, Cartagena, Spain*

**Introduction**

Fascin is an actin bundling protein that plays an important role in the cytoskeleton rearrangement. It is poorly expressed in normal epithelia but upregulated in many human carcinomas, with a crucial role in tumor progression, invasion and metastasis, making it a potential marker for tumor progression. The correlation between tissue and serum fascin levels could be relevant in the study of new serum biomarkers for tumor progression, since if serum fascin levels reflect changes in those tissues where this protein is expressed, it could facilitate the detection and monitoring of tumorigenesis in a less invasive way, using blood samples instead of tissue biopsy.

**Material and Methods**

We analyzed blood and tumor tissue samples extracted during surgery of the primary tumor from 60 patients diagnosed with colorectal cancer stage I to III. Fascin was quantified in blood simples with a double antibody-sandwich ELISA method and the positivity of paraffin-embedded tissue samples for fascin was determined by immunohistochemistry. Staining scores for fascin were calculated by multiplying the staining intensity score (0 = no staining, 1 = weak, 2 = moderate, 3 = strong) in each tumoral area by the stained area score (0 = <10%, 1 = 10-50%, 2 = 50-75%, 3 = >75%). The expression of fascin in the tissue was expressed as categorical, considering it positive when the score was ≥1. Statistical analyses were performed with IBM SPSS version 21.

**Results and Discussions**

There were no differences between the concentration of serum fascin in fascin-positive tumor tissue samples compared to tumor fascin-negative tissue samples. Tissue

Molecular Oncology 18 (Suppl. 1) (2024) 1–495
© 2024 The Authors. Molecular Oncology is published by John Wiley & Sons Ltd on behalf of Federation of European Biochemical Societies expression of fascin is associated with prognosis in patients with colorectal cancer, so it has been proposed as a potential anticancer target and novel fascin inhibitors are being identified. Although several fascin quantification methods, such as ELISA or LC-MS/MS, have been developed, the mechanism of fascin release into serum remains unknown. This mechanism and its correlation with fascin pathogenesis will need to be understood so that it can be used as a non-invasive surrogate marker to identify patients who might benefit from antifascin therapy.

**Conclusion**

The distribution of serum fascin concentration was equivalent between tissue fascin positive and negative categories. Given these results, the mechanism of fascin release into serum may be independent of its expression in tissue. Hence, the concentration of serum fascin may not be serve as a predictive biomarker of fascin expression in tumor tissue.

### EACR2024-1157
### Identification of immune profile of advanced cutaneous cell carcinoma predicting immunotherapy response

*L. Amato[1], C. De Rosa[1], C. Tuccillo[1], A. Ariano[1], A. Di Liello[1], S. Napolitano[1], C.M. Della Corte[1], F. Ciardiello[2], T. Troiani[1], A. Esposito[1]*

*[1]University of Campania Luigi Vanvitelli, Precision Medicine, Napoli, Italy*

*[2]University of Campania Luigi Vanvitelli, Precision Medicine, Napoli, Italy*

**Introduction**

Cutaneous squamous cell carcinoma (cSCC) is the second most common form of skin cancer, characterized by malignant proliferation of epidermal keratinocytes. If treated at an early stage with surgical excision, the 5-year cure rate is over 90%. However, in a minority of cases, they are diagnosed at locally advanced(lacSCC) or metastatic (mcSCC) stages, not amenable to surgery, radiotherapy, or a combination of the two. Cemiplimab, a monoclonal antibody anti-PD-1, is the first drug approved for the treatment of mcSCC and lacSCC. However, 1/3 of patients show primary resistance to Cemiplimab. Here we showed that early dynamics in PBMCs subsets reflect changes in the tumour micro-environment (TME) and predict antitumor immune responses, ultimately reflecting clinical outcomes with Cemiplimab.

**Material and Methods**

With the intent to identify potential parameters that specifically respond to Cemiplimab treatment, we focused on cytokine profiling. To this end, PBMCs from the blood of lacSCC patients or healthy donors were collected. The expression levels of genes encoding for immune checkpoint (i.e., PD-L1, CTLA4) and of several proinflammatory cytokines, such as IL-6, IL-1β, IL12, TNFα, IFNγ and anti-inflammatory ones (i.e., IL4, IL-10) were analysed by RT-qPCR. The changes in immune subsets populations at baseline and during treatment were characterized by FACS.

**Results and Discussions**

Profile gene expression with Nanostring Technologies of 20 patients of which 9 R and 11 NR to cemiplimab, and 3 H biopsies-derived RNA identified 85 common de-regulated genes and CCL-20 and CXCL-8 as the top up-regulates cytokines in NR-patients Accordingly, we found a significant increase in mRNA expression levels of CXCL8 and TNFα in NR patients under treatment. In addition, IL4 mRNA expression was further associated with response to treatment. FACS analysis showed an increase of CD8+ (%) in both R and NR patients under treatment, whereas CTLA4 expression (%) remained stable in NR while it was reduced in R patients. Interestingly, we found an increase of tissue-resident (tr) Treg (CD45RA-, CD127lo, CTLA4hi, ICOShi) in NR patients while treatment reduced the % of tr-Treg in R patients.

**Conclusion**

Our study identifies Tregs markers (CXCL8) and pro-inflammatory cytokines (TNFα) as putative biomarkers involved in primary resistance to Cemiplimab in cSCC patients. Further investigations may identify biomarkers to select and treat tumours resistant to anti-PD-1 therapies.

### EACR2024-1225
### Tumor-Infiltrating Lymphocytes Accurately Predict the Risk of Progression in Sentinel Node Negative Stage II Melanoma

*G. Adigbli[1]*

*[1]University of Oxford, Nuffield Department of Surgical Sciences, Oxford, United Kingdom*

**Introduction**

Adjuvant therapies are increasingly being used to prevent progression of resected early stage melanoma. As a significant proportion of these patients are cured with surgery alone, and these treatments cause acute and long-lasting toxicities, there is an urgent need for accurate biomarker-based risk stratification to balance risks and benefits and guide appropriate patient selection. This study demonstrates the potential of tumor-infiltrating lymphocyte (TIL) scoring to effectively distinguish high and low risk sentinel lymph node-negative SLN(-) patients, offering an accurate and cost-effective method of risk stratification.

**Material and Methods**

A retrospective cohort study of 573 stage II melanoma patients who underwent sentinel lymph node biopsies at four UK hospitals (2004-2017) was conducted. The primary outcome was progression-free survival (PFS). Clinicopathologic factors including age, sex, Breslow thickness, tumor location, histotype, ulceration, and tumor-infiltrating lymphocytes (TILs) were analysed in univariate and multivariate analyses.

**Results and Discussions**

Median follow-up was 8.6 years. Melanoma progression occurred in 23% of patients, with a median PFS of 9.2 years. Sex and TIL score independently predicted progression and PFS, with male sex and non-brisk TILs associated with significantly shorter PFS.To evaluate the prognostic value of TIL scoring, we compared PFS stratified by TIL score (absent, non-brisk, brisk) and AJCC8 substage (IIA, IIB/IIC). PFS of stage IIA melanomas was similar to PFS of melanomas with absent

© 2024The Authors. Molecular Oncology is published by John Wiley & Sons Ltd on behalf of Federation of European Biochemical Societies and brisk TILs. Conversely, PFS of melanomas with non-brisk TILs was similar to stage IIB/IIC melanomas, and less favourable. Notably, non-brisk TILs were more prevalent than stage IIB/IIC tumours (63% vs 44.5%). This finding suggests that current staging may under-estimate progression risk for a significant proportion of stage IIA patients with non-brisk TILs.

**Conclusion**

TIL scoring effectively distinguishes high and low risk SLN(-) stage II melanoma patients, providing an accurate and cost-effective method for refined risk stratification beyond current AJCC sub-staging. Incorporating TIL scoring allows more refined risk stratification and outcome prediction than afforded by current AJCC sub-staging alone, identifying clinically important differences in PFS not captured by current prognostic tools. Even within the lower risk stage IIA designation, TIL scoring identifies patients at higher risk of progression who may benefit from adjuvant immunotherapy.

### EACR2024-1237
### Tumour-Infiltrating Lymphocyte Scoring Refines Risk Stratification in Patients with Sentinel Node Negative Stage II Melanoma

G. Adigbli[1]

[1]*University of Oxford, Nuffield Department of Surgical Sciences, Oxford, United Kingdom*

**Introduction**

The use of adjuvant immunotherapies to prevent disease progression in patients with resected early stage melanoma is becoming more common. However, as many of these patients will have already been cured by surgery, and given the risk of severe and long-term side effects associated with these treatments, there is a pressing need for precise biomarker-based risk stratification to inform more balanced assessment of treatment benefit and risk, and guide optimal patient selection. This study highlights the potential of tumor-infiltrating lymphocyte (TIL) scoring as an effective tool to discriminate between sentinel lymph node-negative stage II patients with high vs low risk of progression.

**Material and Methods**

We conducted a retrospective cohort study of 573 stage II melanoma patients who had undergone SLNB at four UK hospitals. Clinicopathologic factors—including age, sex, Breslow thickness, location, histotype, ulceration, and tumor-infiltrating lymphocyte score (TILs)—were analysed in univariate and multivariate analyses, with progression-free survival (PFS) as the primary outcome.

**Results and Discussions**

Median follow-up was 8.6 years. Progression occurred in 23% of SLN(-) patients. Median PFS was 9.2 years. Sex and TIL score independently predicted progression and PFS, with male sex and non-brisk TILs associated with significantly worse PFS. To compare the predictive capabilities of TIL scoring and AJCC8 substaging, PFS was evaluated following stratification of the cohort using each method. Stage IIA melanomas demonstrated similar PFS to melanomas with absent and brisk TILs, whereas stage IIB/IIC melanomas had PFS similar to melanomas with non-brisk TILs. In stage IIA and IIB/IIC melanomas, absent and brisk TILs were associated with significantly longer PFS than non-brisk TILs. Interestingly, 20% of melanomas expressing non-brisk TILs were stage IIA tumours, and 10 year PFS in these patients was almost 10% lower than stage IIB/IIC melanomas with brisk/absent TILs.

**Conclusion**

TIL scoring effectively distinguishes high and low risk SLN(-) stage II melanoma patients, providing an accurate and cost-effective method for refined risk stratification beyond current AJCC sub-staging. Incorporating TIL scoring allows more refined risk stratification and outcome prediction than afforded by current AJCC sub-staging alone, identifying clinically important differences in PFS not captured by current tools. Even within the lower risk stage IIA designation, TIL scoring identifies patients at higher risk of progression.

### EACR2024-1241
### Chronic Off-Target Cardiotoxicity of Doxorubicin is Mediated by Pathological Changes in Paracrine Signaling and miRNA Prevalence

G. Ronan[1], F. Ketchum[1], N. Kowalczyk[1], N. Behnam[2], L. Çelebi[1], A. Lowery[3], M. Kerin[3], P. Zorlutuna[1,2,4]

[1]*University of Notre Dame, Aerospace and Mechanical Engineering, Notre Dame, United States*

[2]*University of Notre Dame, Chemical and Biomolecular Engineering, Notre Dame, United States*

[3]*University of Galway, Lambe Institute for Translational Research, Galway, Ireland*

[4]*University of Notre Dame, Harper Cancer Research Institute, Notre Dame, United States*

**Introduction**

Doxorubicin (DOX) is a chemotherapeutic used to treat many cancers, including breast cancer. Breast cancer is the most common cancer globally, comprising nearly 30% of yearly diagnoses. DOX is a highly successful chemotherapeutic but has a high propensity for off-target toxicity. Over 10% of women develop acute cardio-toxicity and approximately 2% of women develop toxicity up to 10 years post-treatment. This is despite known clearance of DOX within 48 h, and the precise mechanisms for these effects remain elusive. We suggest that DOX exposure alters the paracrine signaling of cardiac cells to propagate cardiotoxicity independent of DOX.

**Material and Methods**

We first assess the effects of DOX treatment, sEV treatment, and pre-treatment and co-culture in 2D and 3D models. Stem cell derived cardiomyocytes (iCMs) and cardiac fibroblasts (iCFs) were treated (10 nM DOX, 25 µg/mL sEVs, or PBS blank) for 48 h. Beating behavior and $Ca^{2+}$ handling of iCMs, epithelial-to-mesenchymal transdifferentiation (EMT) of iCFs, and metabolic activity and reactive oxygen species (ROS) production were assessed. sEVs from cell cultures were assayed for DOX transport, and miRNA cargo was profiled with downstream pathway analysis. Clinical plasma samples were obtained from our collaborators, and the miRNA cargo of sEVs from these profiled and analyzed. Target miRNAs were validated in a heart-on-a-chip model.

Molecular Oncology 18 (Suppl. 1) (2024) 1–495

© 2024 The Authors. Molecular Oncology is published by John Wiley & Sons Ltd on behalf of Federation of European Biochemical Societies

**Results and Discussions**
Cardiotoxic effects can be induced independent of direct DOX exposure, including increased ROS production, dysregulation of beating behavior, but not rate, and increased EMT in both 2D and 3D models using either sEV treatment or pre-treated cells in co-culture. This indicates that direct DOX exposure is not essential for the onset of chronic cardiotoxicity. These effects coincide with changes to sEV profile, which show both morphological and surface protein shifts we have previously associated with cardiac oxidative stress. Additionally, these sEVs do not transport DOX and instead drive changes via shifts in miRNA cargo profile. Analysis of miRNA cargo reveals 14 miRNAs acting synergistically across 4 pathways, the disruption of which are known to be causal for arrhythmias and heart failure, as likely actors in the propagation of cardiotoxic effects.
**Conclusion**
DOX-induced cardiotoxicity can occur independently of direct DOX exposure through changes in paracrine signaling. The effect of these changes are driven by a shift in miRNA population, which can propagate cardiac dysfunction chronically post-DOX treatment.

### EACR2024-1255
### TCR and BCR immune repertoire profiling and immunophenotyping of single cells with a 96-well plate sorted-cell approach

*A. Chenchik[1], T. Liu[1], M. Makhanov[1], D. Hu[1], L. Kobzik[1], K. Ghias[1], P. Diehl[1]*
*[1]Cellecta Inc., Mountain View, California USA*
**Introduction**
Single-cell immune receptor profiling represents a groundbreaking method enabling the detailed analysis of immune cell clonotypes, their paired-chain configurations, and cellular phenotypes, such as cell subtypes. Traditional medium-throughput methods like microwell arrays and droplet microfluidics, capable of processing 1,000-5,000 cells, offer insights but come with higher complexity, costs, and limitations when compared to bulk profiling techniques.
**Material and Methods**
We introduce a low-throughput, cost-effective single-cell immune profiling approach utilizing sorted cells in 96-well plates, pre-aliquoted with T-cell receptor (TCR) α/β or γ/δ primers and 30 essential T-cell markers. This method involves multiplex RT-PCR amplification and sequencing of the CDR3 regions, allowing for comprehensive analysis of clonotype abundance, chain pairing, and T-cell subtype determination based on gene expression profiling.
**Results and Discussions**
Our approach yields detailed data on clonotype counts, chain pairing for α/β and γ/δ chains, and T-cell subtype identification, enhancing our understanding of T-cell development, proliferation, and clonality. Such insights are pivotal for researching diseases like cancer, immunodeficiency, and autoimmunity. Additionally, when data are paired with RNA sequencing datasets, our method aids in identifying γδ T cells, offering a standardized tool for exploring γδ T cell-based cancer immunotherapies. The affordability and integrated analysis of clonotypes and immunophenotypes position this as a valuable technique in deciphering immune system dynamics in various pathological conditions.
**Conclusion**
This single-cell immune receptor profiling strategy presents a streamlined, cost-efficient solution for the detailed examination of T-cell clonotypes and phenotypes. Its ability to provide important immune insights in a single workflow makes it an indispensable tool for advancing our understanding of immune responses in health and disease, with significant implications for identifying novel immunotherapy targets.

### EACR2024-1257
### Enabling Fast and Convenient Immune Profiling of Fresh and Long-Term Stabilized Human Whole Blood Samples With CyTOF ->

*F. Spada[1], H. Koppejan[1], M. Cohen[1], S. Hasan[1], S. Li[1], C. Loh[1]*
*[1]Standard BioTools Canada Inc., Markham, ON, Canada*
**Introduction**
Accurate phenotyping of immune cells in whole blood (WB) from patients with cancer is critical for making disease prognoses and monitoring clinical efficacy of immunotherapies. Fresh WB must be analyzed within 24 hours of collection to minimize changes in cellular composition. However, WB collection and cytometric analysis are often performed at different sites, which can result in sample processing delays. Several WB preservation reagents have been developed to address this challenge, including PROT1 (Smart Tube Inc.) and Cytodelics Whole Blood Cell Stabilizer (Cytodelics). However, not all antibody panels are compatible with these reagents. A CyTOF® panel was developed to be compatible with these commercial WB stabilizers and for use in pharmaceutical and clinical research.
**Material and Methods**
CyTOF flow cytometry uses metal-tagged antibodies to identify cellular and functional phenotypes. Advantages and features of CyTOF technology enable rapid design and application of 50-plus-marker panels and convenient workflows in which samples can be stained and acquired in a single tube. Compensation is not required since CyTOF flow cytometry has low signal spillover and no autofluorescence. Moreover, antibody cocktails and stained samples can be frozen for later use and acquisition. Thus, CyTOF technology overcomes major hurdles of fluorescence-based cytometry and provides a streamlined and flexible workflow in clinical research. The CyTOF panel contains 20 antibodies to identify over 30 immune cell populations. For easy customization, there are more than 30 additional open channels to analyze markers of interest. The panel works with fresh and stabilized WB samples and is amenable to different staining and acquisition workflows. To show the flexibility of sample staining and stabilization, WB from three healthy donors was assessed using two stabilization workflows. First, fresh WB samples were stained with the antibody panel, followed by PROT1 or Cytodelics stabilization and storage at -80 °C. The second workflow involved immediate stabilization/fixation of WB with

© 2024 The Authors. Molecular Oncology is published by John Wiley & Sons Ltd on behalf of Federation of European Biochemical Societies

PROT1 or Cytodelics and storage at -80 °C. Subsequently, the samples for the second workflow were thawed, surface stained, and acquired. To reduce technical variability from staining, the antibodies were pooled together and frozen at -80 °C as single-use aliquots. Furthermore, all samples were barcoded and acquired as a single tube to reduce variability from sample acquisition.

**Conclusion**

The CyTOF panel developed for broad immune profiling is compatible with WB stabilizers, which overcomes traditional and logistical challenges with WB processing and acquisition. Furthermore, freezing antibody cocktails is a unique feature of CyTOF flow cytometry, ensuring batch-to-batch consistency in clinical research. Thus, CyTOF workflows enable swift and convenient analysis of WB samples.

### Cancer Cell Biology

### EACR2024-0031
### The role of Autophagy dysregulation in Low and High-Grade Non-Muscle Invasive Bladder Cancer: A Survival Analysis and Clinicopathological Association

A. Kumar[1], V. Singh[1], M.K. Singh[1]
*[1]King George's Medical University, Urology, Lucknow, India*

**Introduction**

Bladder cancer disproportionately affects men and often presents as non-muscle-invasive bladder cancer (NMIBC). Despite initial treatments, the recurrence and progression of NMIBC are linked to autophagy. This study investigates the expression of autophagy genes (mTOR, ULK1, Beclin1, and LC3) in low and high-grade NMIBC, providing insights into potential prognostic markers and therapeutic targets.

**Material and Methods**

A total of 115 tissue samples (n =85 NMIBC (pTa, pT1, and CIS) and n = 30 control from BPH patients) were collected. The expression level of autophagy genes (mTOR, ULK1, Beclin-1, and LC3) was assessed in low-grade and high-grade NMIBC, along with control tissue samples, using a quantitative real-time polymerase chain reaction. Association with clinicopathological characteristics and autophagy gene expression was analysed by multivariate and univariate survival analysis using SPSS.

**Results and Discussions**

In high-grade NMIBC, ULK1, $p = 0.0150$, Beclin1, $p = 0.0041$, and LC3, $p = 0.0014$, were substantially down-regulated, whereas mTOR, $p = 0.0006$, was significantly upregulated. The KM plots show significant survival outcome with autophagy genes. The clinicopathological characters, high grade ($p = 0.019$), tumor stage (CIS $p = 0.039$, pT1 $p = 0.018$, $p = 0.045$), male ($p = 0.010$), lymphovascular invasion ($p = 0.028$), and autophagy genes (ULK1 $p = 0.002$, beclin1 $p = 0.010$, $p = 0.022$) were associated as risk factors for survival outcome in NMIBC patients.

**Conclusion**

The upregulated mTOR, downregulated ULK1, and beclin1 expressions are linked to a high-grade CIS and pT1 stage, resulting in poor recurrence-free and progression-free survival and progression free survival and highlights the prognostic significance of the autophagy gene in non-muscle-invasive bladder cancer.

### EACR2024-0063
### Melanoma patient-derived organoids for development of combination therapies

B. Shannan[1], S. Scharfenberg[1], A. Sucker[1], D. Schadendorf[1], A. Roesch[1]
*[1]University Hospital Essen, Dermatology, Essen, Germany*

**Introduction**

Cutaneous melanoma is the most aggressive form of skin cancer, and survival rates dramatically drop following distant organ metastases. Therapy of advanced melanoma is hampered by tumor heterogeneity and plasticity between different metastatic sites (especially brain vs. other organs). Patient-derived organoids (PDOs) have a high potential as translational tool for drug responses and ex vivo investigations. Although PDOs are well-studied for many cancer entities, melanoma PDOs are not fully established.

**Material and Methods**

We have tested different matrices and different medium composition to study melanoma patient-derived organoids (n= 30). We have used well known matrices, e.g., matrigel and other site-specific matrices, e.g., skin-based hydrogels.

**Results and Discussions**

We have found that melanoma heterogeneity affects the growth of PDO. Medium composition is an important factor in determining how well PDOs grow. Moreover, the size of the tissue is usually a limiting factor for generating PDOs. Generating PDOs from clinically annotated melanomas will allow navigation from laboratory models to co-clinical applications as well as the development of novel combination therapies.

**Conclusion**

We aim to mechanistically characterize melanoma PDOs and study microenvironmental interactions to target therapy resistance and apply this biological knowledge to improve patient survival.

### EACR2024-0064
### Downregulation of microRNA-30c-5p in the Remnant Liver Contributes to Hepatocellular Carcinoma Recurrence after Surgical Resection

C.F. Teng[1], M.C. Lee[1], F.Y. Shih[2]
*[1]China Medical University, Graduate Institute of Biomedical Sciences, Taichung, Taiwan*
*[2]China Medical University, Ph.D. Program for Biotech Pharmaceutical Industry, Taichung, Taiwan*

**Introduction**

Hepatocellular carcinoma (HCC) recurrence remains a serious problem responsible for poor patient survival after surgical resection. Besides primary tumor, molecular pathogenesis in the remnant liver after

© 2024 The Authors. Molecular Oncology is published by John Wiley & Sons Ltd on behalf of Federation of European Biochemical Societies resection surgery is implicated in HCC recurrence but largely unexplored.

**Material and Methods**

This study analyzed the expression profiles of a variety of microRNAs (miRNAs) in the remnant liver and recurrent HCC tumor and non-tumor liver tissues in a hepatitis B virus X protein (HBx)-transgenic partial hepatectomy (PH) mouse model, followed by elucidation of their in vitro tumorigenic functions.

**Results and Discussions**

The results showed that the expression of miRNA-30c-5p (miR-30c-5p) was downregulated in the recurrent HCC tumortissues of HBx-transgenic mice after PH, suggesting that dysregulation of miR-30c-5p in post-resection remnant liver was closely associated with HCC recurrence. Moreover, overexpression of miR-30c-5p was shown to exhibit inhibitory effects on cell proliferation, migration, and invasion in human HCC cell lines, suggesting that miR-30c-5p acted as a tumor suppressor in HCC and its downregulation in the remnant liver positively contributed to tumorigenesis.

**Conclusion**

This study demonstrated that downregulation of miR-30c-5p in the remnant liver played an important role in promoting HCC recurrence after surgical resection. Targeting miR-30c-5p held promise as a novel therapeutic strategy for the treatment of HCC recurrence.

### EACR2024-0065
### p53 amyloid plays oncogenic role and promotes colorectal cancer progression via the interleukin axis

*S. Dasgupta*[1], *S. Jaiswal*[2], *S.M. Ghufran*[1], *A. Khan*[1], *S. Biswas*[1]
[1]*Amity University Uttar Pradesh, Amity Institute of Molecular Medicine and Stem Cell Research, NOIDA, India*
[2]*Amity University Uttar Pradesh, Amity Institute of Molecular Medicine and Stem Cell Resaerch, Noida, India*

**Introduction**

The prevalence of mutations in the p53 gene exceeds 50% across various cancer types, making it a critical focus in cancer research. Notably, wild-type and mutant p53 proteins have been observed to form stable amyloids, a phenomenon occurring in the nucleus or cytoplasm. To unravel the intricacies of the p53 signaling network, this study investigates distinct signaling pathways associated with specific uncharacterized p53 mutations that may or may not form amyloid.

**Material and Methods**

Cell-based assays are utilized to assess the ability of these mutations to form amyloid structures. Transcriptomic analyses, including the identification of altered transcription factors and chemokine pathways, are conducted to elucidate the impact of these mutations on cellular processes. The differentially upregulated genes mainly of the interleukin family were knockdown using siRNAs.

**Results and Discussions**

Our findings reveal a distinctive propensity of some of the hotspot p53 mutations, in contrast to others, to form amyloid structures within cells and the associated loss-of-functions. Transcriptomic analyses demonstrate the activation of over 1000 genes in various p53 mutants/amyloids, with approximately 900 genes downregulated compared to null or wild type p53. Moreover, we observe significant downregulation of amyloid several tumor suppressors in both mutant and wild-type p53 amyloids. The altered transcriptional profile suggests that gain-of tumorigenic properties lead to the activation of oncogenic signaling genes and pathways, predicting a more unfavorable prognosis. Notably, p53 amyloids exhibit a selective enhancement of proliferation, migration, invasion, and metastasis compared to non-amyloid mutant forms. We further demonstrated the potential of histone deacetylase inhibitors (HDACi) to induce apoptosis that showed more promising results in non-amyloid mutants. Modulating the p53 amyloid/mutant based interleukin expression could depict tumor-suppressive and anti-proliferative effects.

**Conclusion**

In conclusion, our comprehensive investigation provides crucial insights into the diverse contributions of wild-type/mutant amyloid to cancer progression. The distinct transcriptional profiles and functional outcomes underscore the complexity of p53-related signaling networks. Furthermore, our study highlights the potential of histone deacetylase inhibitors (HDACi) and modulating the interleukin expressions to induce apoptosis, offering promising avenues for precision-based personalized medicine in cancer treatment.

### EACR2024-0078
### Red blood cell-derived vesicles for safe and efficient siRNA delivery

*N. Kostevšek*[1], *G. Della Pelle*[1], *B. Markelc*[2], *T. Božič*[2], *J. Šribar*[3], *M. čemažar*[2]
[1]*Jožef Stefan Institute, Department for nanostructured materials, Ljubljana, Slovenia*
[2]*Institute of oncology, Department of Experimental Oncology, Ljubljana, Slovenia*
[3]*Jožef stefan Institute, Department of Molecular and Biomedical Science, Ljubljana, Slovenia*

**Introduction**

Among different circulatory cells, erythrocytes are the most abundant and thus can be isolated in sufficiently large quantities to decrease complexity and cost of the treatment compared to other cell-based vaccines. In the present work, we used fresh red blood cells as starting material for a neutral therapeutic nucleic acid delivery system. Our therapeutic nucleic acid of choice is siRNA, for its compactness and already well-established clinical applications. As proof-of-concept, we loaded an anti-TdTomato siRNA in erythrocytes membrane vesicles (EMVs). EMVs were proven to be a successful, cheap and safe delivery system for siRNA on tumor-induced mice.

**Material and Methods**

We optimized purification strategy and long-term storage techniques of EMVs. siRNA-loaded EMVs were valuated for nuclease resistance. Vesicles were analysed via TEM and cryo-TEM. siRNA release and RNA interference ability was evaluated in vitro. Finally, siRNA-EMVs, containing Cy5-labeled anti-tdtomato siRNA, were injected via caudal vein in tumor-induced

© 2024The Authors. Molecular Oncology is published by John Wiley & Sons Ltd on behalf of Federation of European Biochemical Societies mice. Blood was collected ten minutes after EMVs injection and right before sacrifice (2h, 24h and 48 h after injection). Spleen, lungs, brain, kidneys, tumor, heart and liver were imaged using confocal microscopy and processed for RNA extraction (real-time PCR).

**Results and Discussions**

We confirmed the occurred loading with siRNA in our EMVs via freeze-fracture TEM and STED-laser equipped confocal microscope, effectively visualizing siRNA within the lumen of vesicles. siRNA-EMVs showed a high level of protection against RNAse A. In vitro, silencing ability of siRNA-EMVs was found to be around 80%, at a final well concentration of 0.3 nM, a remarkable lower dose when compared to HEK293-derived EV and Neuro2a-EV loaded with lipidic siRNA. In vivo, on melanoma-bearing mice, at 2.5 mg/kg of body weight, siRNA-EMVs were capable, upon systemic administration, to elicit a 60% of Tdtomato gene silencing, while intact EMVs, containing anti TdTomato siRNA lead strand, were still present in the blood flux after 48 hours from administration.

**Conclusion**

We were therefore able to develop a simple, cost-effective, therapeutic nucleic acid carrier, that is also prone to easy scaling-up. Future developments for these carriers will include active targeting to tumor sites and the simultaneous loading to a reporter agent, in order to obtain an all-in-one theranostic agent.

### EACR2024-0087
### Persistent Properties of Cancer Cells Overexpressing the Hedgehog Receptor Ptch1

*I. Mus-Veteau[1], Á. Javier Feliz Morel[1], C. Sarah[1], M. Aurelie[2], F. Judith[2], P. Claude[3]*
*[1]Institut of Molecular and Cellular Pharmacology, CNRS - University Côte d'Azur, Valbonne, France*
*[2]PARCC, Université de Paris - INSERM, Paris, France*
*[3]Laboratoire d'Informatique- Signaux et Systèmes de Sophia Antipolis, CNRS - Université Côte d'Azur, Valbonne, France*

**Introduction**

Despite the development of new therapeutic strategies, cancer remains one of the leading causes of mortality worldwide. One of the current major challenges is the resistance of cancers to chemotherapy treatments inducing metastases and relapse of the tumor. The Hedgehog receptor Ptch1 is overexpressed in many types of cancers. We showed that Ptch1 contributes to the efflux of doxorubicin and plays an important role in the resistance to chemotherapy in adrenocortical carcinoma (ACC), a rare cancer which presents strong resistance to the standard of care chemotherapy treatment.

**Material and Methods**

ACC cell culture, Flow Cytometry, Immunofluorescence, Western-Blot, Cytotoxicity Assay, Doxorubicin Accumulation in Cells, Clonogenic Assay, Transwell Invasion Assay, Wound-Healing Assay, RNA-Seq, xenografts in chick eggs and in Mice

**Results and Discussions**

In the present study, we isolated and characterized a subpopulation of the ACC cell line H295R in which Ptch1 is overexpressed endogenously and more present at the cell surface. We showed that this cell subpopulation is more resistant to doxorubicin, grows as spheroids, and has a greater capability of clonogenicity, migration, and invasion than the parental cells. Xenograft experiments performed in mice and in ovo showed that this cell subpopulation is more tumorigenic and metastatic than the parental cells. These results suggest that this cell subpopulation has cancer stem-like or persistent cell properties which were strengthened by RNA-seq.

**Conclusion**

Altogether, our results reveal the presence of a subpopulation of cells overexpressing Ptch1 with persistent and metastatic properties in the human ACC cell line H295R. If present in tumors from ACC patients, these cells overexpressing Ptch1 could be responsible for treatment resistance, relapse, and metastases.

### EACR2024-0094
### Naïve and primed embryonic stem cells can promote distinct immunotherapeutic responses against breast cancer

*M. Correia[1], M. Casanova[2], R. Vitorino[1], S. Nóbrega-Pereira[1], B. Bernardes de Jesus[1]*
*[1]Institute of Biomedicine, Department of Medical Sciences- University of Aveiro, Aveiro, Portugal*
*[2]Instituto de Medicina Molecular João Lobo Antunes, Faculdade de Medicina- Universidade de Lisboa, Lisboa, Portugal*

**Introduction**

Despite notable advancements in cancer treatment, metastatic solid tumors remain remarkably challenging. Immunotherapy has emerged as a leading-edge approach, especially for advanced tumors that resist the existing therapies. A rising level of interest in cancer immuno-therapy is emerging, holding great promise for the future of personalized medicine. Nonetheless, accurate antigen loading, and precise tumor targeting is limiting the effectiveness of current immunotherapeutic strategies.

**Material and Methods**

Patient-derived induced pluripotent stem cells (iPSCs) and Embryonic Stem Cells (ESC) exhibit significant similarities with cancer cells. The process of artificial cellular reprogramming has been extensively compared to the tumor biogenesis. iPSCs and ESC share known and unknown tumor-associated antigens and tumor-specific antigens (TAAs/TSAs), suggesting a potential to stimulate the immune response against cancer. In fact, it was previously successfully tested an iPSC related cancer vaccine in distinct mouse models of cancer. Exploring ESCs/iPSCs characteristics raises intriguing possibilities for their utilization in the development of cancer vaccines.

**Results and Discussions**

Here, we established that TNG-A cells (mouse embryonic stem cells line derived from E14, with $GFP^{+}$ coupled to the Nanog gene) have transcriptomic and proteomic similarities with the E0771 (mouse breast cancer cell line). Additionally, we demonstrated that TNG-A can trigger an anti-tumor immunologic response against breast cancer in C57BL/6J mice.

**Conclusion**

Our preliminary findings indicate that immunization with TNG-A, across different pluripotency states, effectively

Molecular Oncology 18 (Suppl. 1) (2024) 1–495
© 2024 The Authors. Molecular Oncology is published by John Wiley & Sons Ltd on behalf of Federation of European Biochemical Societies primed the immune system to recognize and combat tumor growth under specific conditions, in an orthotopic breast cancer mouse model.

### EACR2024-0110
### Iroquois homeobox 4 (IRX4) Derived Micropeptide Promotes Prostate Cancer Progression and Chemoresistance Through Wnt Signaling Dysregulation

*J. Batra[1], A. Fernando[2]*
*[1]Queensland University of Technology, Center for Genomics and Personalised Health, Brisbane, Australia*
*[2]Queensland University of Technology, Centre for Genomics and Personalized Health, Brisbane, Australia*

**Introduction**
Genome-wide association studies have implicated Iroquois (IRX) gene clusters in cancer susceptibility, yet their functional roles remain unclear. Micropeptides (miPEPs), short open reading frames (sORFs) encoding small peptides, have emerged as regulators of diverse biological processes. However, the significance of cancer-related miPEPs in tumorigenesis and therapy response remains unexplored.

**Material and Methods**
Here, we utilized a Sequential Window Acquisition of all Theoretical Mass Spectra (SWATH-MS/MS)-based proteomic approach to discover miPEPs generated by IRX clusters in prostate, breast, endometrial, and ovarian cancers. The differential expression of identified miPEPs was measured across different cancer subtypes, grades, and stages, and identified IRX4_PEP1, a miPEP derived from *IRX4* as a promising candidate. The role of IRX4_PEP1 was evaluated in prostate cancer (PCa) in vitro via functional assays and comprehensive pathway analysis. Additionally, the expression of IRX4_PEP1 was evaluated in PCa patient samples for it's potential diagnostic and prognostic significance.

**Results and Discussions**
We identified 17 miPEPs from IRX clusters in prostate, breast, endometrial, and ovarian cancers. Among these, IRX4_PEP1 exhibited significant roles in PCa progression. IRX4_PEP1 promoted PCa cell proliferation, migration, and invasion by interacting with heterogeneous nuclear ribonucleoprotein K (hnRNPK). Notably, IRX4_PEP1 disrupted Wnt signaling by interacting with Catenin beta 1 (β catenin; CTNB1), elevating PCa stemness markers, and fostering docetaxel resistance. Clinically, IRX4_PEP1 expression was elevated in PCa tissues, correlating positively with disease aggressiveness. CTNB1, hnRNPK levels and ssGSEA enrichment score of WNT/CTNB1 signaling were correlated positively with IRX4_PEP1 in PCa tissues.

**Conclusion**
These findings highlight IRX4_PEP1's role in PCa stemness and chemoresistance, suggesting it as a therapeutic target and potential diagnostic marker. This study unveils the intricate links between miPEPs, gene clusters, and cancer progression, offering insights for innovative interventions.

### EACR2024-0111
### Identification and Characterization of Senescence Phenotype in Lung Adenocarcinoma with High Drug Sensitivity

*A. Chatterjee[1], S. Shukla[2], S. Khadirnaikar[3]*
*[1]Indian Institute of Technology- Dharwad, Department of Biosciences and Bioengineering, Dharwad, India*
*[2]Indian Institute of Technology- Dharwad- India, Department of Biosciences and Bioengineering, Dharwad, India*
*[3]Indian Institute of Technology Dharwad- Dharwad- India, Department of Biosciences and Bioengineering-Department of Electrical Engineering, Dharwad, India*

**Introduction**
Lung adenocarcinoma (LUAD) represents a formidable health concern characterized by a poor prognosis. The intricacies of heterogeneity play a pivotal role in determining treatment outcomes, underscoring the need for discerning new subclasses within LUAD. Notably, senescence has recently gained prominence as a pivotal regulator of both metastasis and drug response.

**Material and Methods**
Ionizing radiation and doxorubicin-induced senescence-associated genes were employed in K-means clustering to delineate high- and low-senescence (HS and LS) classes within The Cancer Genome Atlas- Lung Adeno-carcinoma (TCGA-LUAD) patients. Utilizing a machine learning–based recursive feature elimination technique, we identified a 20-gene senescence signature within the TCGA-LUAD samples. The presence of a senescence phenotype associated with poor survival was subsequently validated in an independent patient cohort and a cell-line cohort using unsupervised clustering of samples based on the identified 20-gene signature.

**Results and Discussions**
The LS (low-senescence) group exhibited markedly diminished survival ($P = 0.01$) along with heightened activation of proliferative signaling pathways, increased cellular proliferation, augmented wound healing capabilities, and a higher frequency of genetic aberrations ($P < 0.05$). The HS (high-senescence) group displayed a significantly elevated TP53 mutation rate ($P < 0.0001$), providing a mechanistic explanation for the observed phenotype. Furthermore, genome-wide hypomethylation was notably more pronounced in the LS group compared to the HS group. Intriguingly, pathway analysis revealed the suppression of Wnt signaling in the HS group. Upon further investigation, HS cells demonstrated enhanced resistance to various drugs, particularly histone deacetylase inhibitors.

**Conclusion**
In conclusion, our study has unveiled a previously unidentified subtype within lung adenocarcinoma (LUAD), characterized by suppressed Wnt signaling and heightened resistance to pharmacological interventions. These results emphasize the pivotal role of senescence as a key determinant contributing to the intricate heterogeneity of LUAD, thus presenting novel prospects for precision-oriented therapeutic strategies.

© 2024 The Authors. Molecular Oncology is published by John Wiley & Sons Ltd on behalf of Federation of European Biochemical Societies

### EACR2024-0112

### Interaction of CD24 with SIGLEC10 promotes Breg cell formation and metastasis in urothelial carcinoma of the bladder

D. Raja[1], A. Singh[1], A. Seth[2], A. Sharma[1]
[1]All India Institute of Medical Sciences, Biochemistry, New Delhi, India
[2]All India Institute of Medical Sciences, Urology, New Delhi, India

**Introduction**

The expression of CD24 is associated with invasiveness and poor survival outcomes in urothelial carcinoma of the bladder (UCB). Amid its role in the migration of cancer cells and inhibition of immune cells, it also promotes anti-inflammatory function (M2-like phenotype) in macrophages. Interaction of CD24 with its receptor Siglec10 on B cells inhibits activation and development of B cells although there are no reports of its involvement in $B_{reg}$ cell formation. In this study, we investigated the distribution of $B_{reg}$ cells in bladder cancer tissue and their interaction with cancer.

**Material and Methods**

We evaluated regulatory B cells (IL10+ and TGFb+) in peripheral blood and bladder tissue (both normal and tumor). Expression of CD24 was assessed in the tumor tissue. Isolated B cell was co-cultured with T24 cell line transfected with CD24 with and without the anti-CD24 antibodies; and cell proliferation, migration, and cytokine secretions were evaluated.

**Results and Discussions**

In UCB patients we observed the percentage of TGFβ+ and IL10+ $B_{reg}$ cells to be higher in the periphery and tumour tissue(n=40) in comparison to controls. Also, the expressions of CD24 and Siglec10 were significantly high in high-grade tumours (n=30) as compared to the low-grade tumours (n=10) and adjacent normal tissues (n=40). Upon co-culturing with isolated B cells, the migration and invasion of T24 cells (bladder cancer cell line) transfected with CD24 was significantly increased while the proliferation was reduced. TGFβ not IL10 augmented the migration and invasion of T24 cells. The co-culture also caused a spike in the frequency of TGFβ + and IL10+ $B_{reg}$ cells with a reduction in TNFα+ B cells. The addition of anti-CD24 reduced the frequency of TGFβ + and IL10+ $B_{reg}$ cells and the migration and invasion of T24 cells; but caused an increase in the levels of TNFα and HLA-DR in B cells.

**Conclusion**

Our study demonstrates the interaction of B cells with Bladder cancer cells via Siglec10-CD24 inhibits inflammatory B cells and promotes $B_{reg}$ cell formation, which in turn augments metastasis in bladder cancer.

### EACR2024-0118

### Evaluating the expression and regulation of human endogenous retrovirus K (HERV-K) in ovarian cancer

J. Florek Carlson[1], J. Yang[1], J. McAuliffe[2], S. Glynn[2], M.S. Stack[1]
[1]University of Notre Dame Harper Cancer Research Institute, Chemistry and Biochemistry, South Bend, USA
[2]University of Galway, Lambe Institute, Galway, Ireland

**Introduction**

HERV-K is a member of the human endogenous retroviruses, a group of ancient viruses that are integrated into the human genome. HERV-K has been extensively studied in breast and prostate cancer, and upregulation of its mRNA and proteins has been observed in several tumor types. HERV-K is thought to contribute to tumor growth and metastasis, although its functional mechanism has not been thoroughly investigated. Associations have been observed between HERV-K and inducible nitric oxide synthase (iNOS) in breast and prostate cancer, suggesting a link between iNOS signaling and HERV-K expression. Transcriptional activity of HERV-K has also been reported in ovarian cancer, but knowledge concerning its role in ovarian cancer is limited. Ovarian cancer presents a clinical challenge due to a lack of reliable biomarkers and few obvious symptoms early in the disease. Considering the diagnostic potential of HERV-K apparent from its study in breast and prostate cancer, it warrants further study in ovarian cancer as well. Here, we evaluate the expression of HERV-K across multiple ovarian cancer cell lines and its relationship to iNOS.

**Material and Methods**

We cultured human Ovcar5, Ovcar8, and Caov3 cell lines and tested expression levels of HERV-K capsid (Gag) and envelope (Env) proteins via Western Blot. We used qRT-PCR to measure expression of *HERV-K gag, env1*, *env2*, and *HERV-W* genes. To recapitulate the effect of nitric oxide release by iNOS, we treated cells with DETA NONOate (DETA/NO), a nitric oxide donor that mimics intracellular iNOS activity. Cells were incubated in media containing 300 or 500µM DETA/NO for 0.5 to 24 hours, after which lysates were harvested for analysis by Western Blot and qRT-PCR.

**Results and Discussions**

In all three cell lines, we observed expression of the HERV-K Env protein. qRT-PCR also revealed expression of HERV-K *env1* and *env2* genes, while *gag* and *HERV-W* expression was weak. DETA/NO appeared to differentially affect HERV-K Env expression in different cell lines. Ovcar5 saw a decrease in Env expression with increased duration of DETA/NO exposure, while Ovcar8 and Caov3 both saw an increase in expression.

**Conclusion**

Our data provides evidence for an implication of HERV-K in ovarian cancer and relation to the iNOS signaling pathway. Further study is needed to gain further directional and mechanistic insights into the interaction between HERV-K and iNOS, as well as elucidating the actual role that HERV-K plays in ovarian cancer.

### EACR2024-0124

### Metabolic interplay unleashed: CB-839 and FK-866 to target MYC overexpression multiple myeloma

L. Hasan Bou Issa[1], L. Fléchon[1], W. Laine[1], R. Sklavenitis Pistofidis[2], B. Quesnel[1,3], I. M. Ghobrial[2], J. Kluza[1], S. Manier[1,3]
[1]ONCOLille institute, Canther-INSERM UMR-S1277

© 2024 The Authors. Molecular Oncology is published by John Wiley & Sons Ltd on behalf of Federation of European Biochemical Societies

*and CNRS UMR9020- Lille University, Lille, France*
*[2]Dana-Farber Cancer Institute, Harvard Medical School, Boston- MA 02215, United States*
*[3]CHU Lille, Department of Hematology, Lille, France*

**Introduction**

Multiple myeloma (MM) is an incurable hematological malignancy, characterized by the uncontrolled proliferation of malignant plasma cells in the bone marrow. MM progresses from precursor stages, known as monoclonal gammopathy of undetermined significance and smoldering multiple myeloma, to the symptomatic form, MM. MYC alterations play an essential role in this progression. However, MYC remains a long-pursued target. To overcome this, we hypothesized that the proliferative advantage induced by MYC overexpression creates genomic dependencies on other signalling pathways that become essential for cell survival.

**Material and Methods**

To test this hypothesis, we applied large-scale loss of function screen (Achilles) and 1869 small molecules screen to identify vulnerabilities in MYC overexpressing MM cells. We generated an isogenic model of MYC overexpression in U266 cell line using EF1A-C-MYC lentiviral vector. Then, we performed RNA-seq and quantitative proteomics by Tandem Mass Tag mass spectrometry. For validation, we performed pharmacological inhibition of glutamine catabolism and NAD synthesis as well as shRNA-mediated GLS1 knockdown. To determine the functional mechanisms, we used capillary electrophoresis-mass spectrometry (CE-MS) and Agilent Seahorse XF analyzer. To confirm the potential therapeutic relevance of these dependencies observed in vitro, we have used xenograft (MM1S luc-GFP, SCID mice).

**Results and Discussions**

Our analyses demonstrate the dependence of MYC overexpressing cells on glutamine metabolism, in particular on the GLS1. We validated and functionally delineated the differential modulation of glutamine to maintain mitochondrial function and cellular biosynthesis in *MYC* overexpressing cells. Furthermore, our small molecule screen highlighted that NAD synthesis inhibitors had a preferential effect on the proliferation of MYC overexpressing cells.Considering the interlinked metabolic network of glutamine and NAD, we investigated the possibility of a potential synergistic effect between GLS1 and NAMPT inhibitors. Herein, we have exacerbated the differential metabolic vulnerability on glutaminolysis and NAD synthesis in MYC over-expressing by combining CB-839 and FK-866. We demonstrated the effectiveness of this new synergistic combination to target MYC-driven MM cells in vitro and in vivo.

**Conclusion**

These results establish a solid methodological basis that can be used to develop new therapeutic approaches to address unmet therapeutic needs to target MYC in MM.

### EACR2024-0132
### COH29 treatment induces alterations in the cell cycle and suppresses the DNA damage response in ATRT through the ATM-Rb-E2F1 pathway

<u>L.H. Giang</u>[1], T.T. Wong[2], C.C. Chang[1,3]
*[1]Taipei Medical University, International Ph.D. Program for Translational Science- College of Medical Science and Technology, Taipei, Taiwan*
*[2]Taipei Medical University, Graduate Institute of Clinical Medicine- College of Medicine, Taipei, Taiwan*
*[3]Taipei Medical University, The Ph.D. Program for Translational Medicine- College of Medical Science and Technology, Taipei, Taiwan*

**Introduction**

Atypical teratoid rhabdoid tumor (ATRT) is an aggressive tumor of the central nervous system that mainly occurs in children, lacking a specific treatment protocol and often presenting with poor prognosis. Our recent investigation has unveiled promising avenues for therapeutic intervention by targeting RRM2, which induces DNA damage, impedes homologous recombination repair, and triggers apoptosis in ATRT. Furthermore, in vivo experiments employing the RRM2 inhibitor COH29 demonstrated notable suppression of tumor growth and prolonged survival in ATRT xenograft mice. Despite these encouraging results, the underlying molecular mechanisms controlling these observed changes remain elusive. Elucidating these mechanisms is crucial to enhance the potential clinical applicability of COH29 in ATRT treatment and advance clinical trials.

**Material and Methods**

The study analyzed the molecular profiling of ATRT patients and treated cells to identify the specific signaling pathways affected by the COH29 treatment. In-silico results were confirmed using a shRNA knockdown system, flow cytometry, q-PCR, and western blot.

**Results and Discussions**

Our study reveals the importance of the E2F1 signaling pathway in ATRT and suggests that elevated E2F1 levels may be associated with poor patient prognosis. Notably, inhibition of RRM2 was found to induce DNA damage and activate ATM. This cascade subsequently leads to reduced phosphorylation of Rb, promoting its association with the E2F1 transcription factor and thereby inhibiting its function. Suppression of E2F1 activity resulted in decreased expression of E2F1-dependent transcriptional targets. Consequently, cells become arrested in the G1 phase of the cell cycle, with a concomitant reduction in the proportion of S phase. Furthermore, this suppression blocks DNA damage repair mechanisms, potentially prompting cells to exit the cell cycle and undergo apoptosis.

**Conclusion**

Our findings highlight the critical role of the ATM-Rb-E2F1 signaling pathway in orchestrating DNA damage-induced cell cycle arrest and subsequent activation of apoptosis during COH29 treatment. This insight sheds light on the molecular mechanisms underlying the therapeutic effects of COH29 and highlights the potential of targeting this pathway for therapeutic intervention in ATRT.

### EACR2024-0134
### SOS1 inhibitor BI-3406 demonstrates anti-tumor activity akin to SOS1 genetic

© 2024The Authors. Molecular Oncology is published by John Wiley & Sons Ltd on behalf of Federation of European Biochemical Societies

### ablation in KRASG12D mutant tumors

F. Calvo Baltanas[1], M. Kramer-Drauberg[2], R. Garcia-Navas[3], E. Patrucco[2], A. Olarte-San Juan[3], B. Mair[4], K. Kostyrko[4], M.H. Hofmann[4], C. Ambrogio[2], E. Santos[3]
[1]Instituto de Biomedicina de Sevilla, Universidad de Sevilla, Lab 103, Sevilla, Spain
[2]University of Torino-Molecular Biotechnology Center, Molecular Biotechnology and Health Sciences, Torino, Italy
[3]Instituto de Biología Molecular y Celular del Cáncer, Lab 1, Salamanca, Spain
[4]Boehringer Ingelheim RCV GmbH & Co KG, Oncology research, Vienna, Austria

**Introduction**

Therapeutic responses to currently approved KRAS inhibitors are not always durable and resistance frequently occurs. To enhance durability of response the quest for new anti-RAS cancer therapies is continuing with a focus on KRAS inhibitor combinations with drugs targeting downstream or upstream components of RAS pathway. In this regard, recent studies support the consideration of SOS1, a guanine nucleotide exchange factor (GEF) for RAS as a relevant therapeutic target in RAS-dependent tumors.

**Material and Methods**

Here, we evaluated both the in vitro and in vivo therapeutic efficacy and tolerability of BI-3406-mediated pharmacological inhibition of SOS1 in comparison to its genetic ablation in various KRAS-dependent experimental tumor settings.

**Results and Discussions**

In contrast to the rapid lethality of SOS1 conditional knock-out in a wild-type or SOS2-deficient mice, pharmacological inhibition of SOS1 using a SOS1-selective inhibitor BI-3406, did not significantly affect animal weight or viability, nor cause noteworthy systemic toxicity. In allograft assays in immuno-compromised mice, treatment of different $KRAS^{mut}$ MEFs with BI-3406 resulted in reduced RAS-GTP levels. Reduced RAS downstream signaling led to significantly decreased tumor burden and slower disease progression as a result of both tumor-intrinsic and extrinsic antitumor immune effects of the drug. Moreover, in $KRAS^{G12D}$ allografts genetic and pharmacological inhibition of SOS1 displayed anti-tumor efficacy comparable to treatment with a $KRAS^{G12D}$ inhibitor MRTX1133 and resulted in synergistic antitumor effect when both inhibitors were combined. Furthermore, consistent with previous genetic evidence from our laboratory, in vivo administration of BI-3406 in an immunocompetent mouse model of $KRAS^{G12D}$-driven lung cancer resulted in a strong reduction of lung tumor burden and significant downmodulation of the pro-tumorigenic tumor micro-environment (TME).

**Conclusion**

Our data confirm the SOS GEFs as bona fide therapeutic targets in RAS-dependent cancers and identify a therapeutic window for SOS1 pharmacological inhibition that may translate to clinical benefits through reduction of intrinsic tumor burden and impairment of extrinsic pro-tumorigenic contributions of the surrounding TME.

### EACR2024-0135
### Vitamin-C-dependent downregulation of the citrate metabolism pathway potentiates pancreatic ductal adenocarcinoma growth arrest

A. Cenigaonandia Campillo[1], A. Garcia Bautista[1], A. Rio Vilariño[1], Ó. Aguilera[1], J.M. Garcia-Foncillas[1]
[1]IIS-FJD, Translational Oncology, Madrid, Spain

**Introduction**

In pancreatic ductal adenocarcinoma (PDAC), metabolic rewiring and resistance to standard therapy are closely associated. PDAC cells show enormous requirements for glucose derived citrate, the first rate-limiting metabolite in the synthesis of new lipids. Both the expression and activity of citrate synthase (CS) are extraordinarily upregulated in PDAC. However, no previous relationship between gemcitabine response and citrate metabolism has been documented in pancreatic cancer.

**Material and Methods**

Using 13C6 glucose tracer metabolomics, we unveiled metabolic alterations induced by vitamin C upon exposure to ascorbate in KRAS mutant PDAC cell lines. Validation of these findings was performed through enzyme activity assays conducted both in vitro and in vivo, utilizing a PDAC patient-derived xenograft (PDX) model. Lentiviral knockdown of CS and FASN highlighted the involvement of citrate metabolism in gemcitabine response, with the efficacy of combinatory treatment underscored by results obtained from the PDX model

**Results and Discussions**

Here, we report for the first time that pharmacological doses of vitamin C are capable of exerting an inhibitory action on the activity of CS, reducing glucose-derived citrate levels. Moreover, ascorbate targets citrate metabolism towards the de novo lipogenesis pathway, impairing fatty acid synthase (FASN) and ATP citrate lyase (ACLY) expression. Lowered citrate availability was found to be directly associated with diminished proliferation and, remarkably, enhanced gemcitabine response. Moreover, the deregulated citrate-derived lipogenic pathway correlated with a remarkable decrease in extracellular pH through inhibition of lactate dehydrogenase (LDH) and overall reduced glycolytic metabolism.

**Conclusion**

Modulation of citric acid metabolism in highly chemo-resistant pancreatic adenocarcinoma, through molecules such as vitamin C, could be considered as a future clinical option to improve patient response to standard chemotherapy regimens

### EACR2024-0137
### Temporal regulation of PARP1 DNA damage and metabolic response by HDAC5 promotes colon tumorigenesis

W. Tyagi[1], S. Das[1]
[1]National Institute of Immunology, Molecular Oncology Laboratory, New Delhi, India

**Introduction**

© 2024 The Authors. Molecular Oncology is published by John Wiley & Sons Ltd on behalf of Federation of European Biochemical Societies

PARP1, a rapid responder to cellular stress, facilitates DNA repair via poly ADP-ribosylation (PARylation), recruiting repair proteins. Beyond DNA repair, PARP1 influences gene transcription through diverse mechanisms, implicated in cancers such as colorectal cancer. HDAC5 modulates gene expression by deacetylating histones and interacting with proteins to regulate oncogenic pathways. Exploring its substrates can illuminate its contribution to tumorigenesis. We identify PARP1 as key substrate of HDAC5 impacting tumorigenesis.

**Material and Methods**

An acetylome screening identified potential HDAC5 substrates, followed by confirmation of the interaction using a proximity ligation assay. PARP1 was validated as a substrate through a deacetylation assay. Stable knockdown cell lines were generated using the psiRNA-DUO plasmid, and protein expression levels were assessed by Western blot. DNA damage response was evaluated using an alkaline comet assay. Changes in gene transcription were analyzed using transcriptomics and RT-PCR, while metabolomics analysis identified alterations in cellular metabolism. In vivo tumorigenic studies were conducted in nude mice.

**Results and Discussions**

Mechanistically, we demonstrate that the deacetylation of PARP1 at Lys498 regulates its poly ADP-ribosylation (PARylation) in response to genotoxic stress. This modification prevents PARP1 from being trapped on chromatin, allowing for the effective recruitment of DNA repair proteins to damaged sites and promoting cell cycle progression. Additionally, deacetylation at Lys521 activates ATF4 target genes, resulting in metabolic homeostasis changes. Consequently, Pharmacological inhibition of PARP1 in mouse invivo models does not robustly halt tumor progression, as its transactivation function remains unperturbed. Elevated HDAC5 levels in advanced colon adenocarcinoma grades correlate with reduced acetylated PARP1 levels. Hence, our study provides valuable insights for effectively inhibiting tumorigenesis through targeted interventions.

**Conclusion**

In this study, we investigated how PARP1 acetylation affects its DNA repair and transcriptional functions. Our proteomics approach revealed HDAC5 as the key deacetylase regulating PARP1 function which is crucial for active DNA damage response and metabolic reprogramming under genotoxic stress. These findings offer new insights into how PARP1 is regulated and further elucidate its role in tumor development.

### EACR2024-0141
### Effects of Ailanthone in inflammatory breast cancer cell lines

*B. Crespo Cortés*[1], *S. Caceres*[1], *G. Silvan*[1], *M.J. Illera*[1], *J.C. Illera*[1]

[1]*Veterinary medicine School. University Complutense of Madrid, Animal Physiology, Madrid, Spain*

**Introduction**

Inflammatory breast cancer (IBC) is considered the most aggressive type of breast cancer in both human and canine species. Canine IBC has been shown to be a good research model for human disease due to their broad similarities. This type of cancer can be presented as triple negative breast cancer (TNBC) due to the negative expression of estrogen and progesterone receptors, and HER-2. However, the expression of the androgen receptor (AR) is not contemplated. Therefore, this preliminary study aims to determine the role of AR expression in IBC-tiple negative cell lines and the effect of different anti-androgenic therapies.

**Material and Methods**

To this purpose, the cell lines of canine IBC (IPC-366), human IBC (SUM149) and luminal androgen receptor-TNBC (MDA-453) were used. These cell lines were maintained in culture flasks in an incubator at 5% CO2 and 37ºC. To determine the AR expression, immuno-fluorescence techniques were performed. Also, cells were treated with different anti-androgenic treatments: Nilutamide and Bicalutamide (that bind to AR inhibiting its function); VPC-13566 and Ailanthone (that block AR at transcriptional level); and Abiraterone (that inhibit androgen synthesis). Sensitivity assays were performed in order to obtain the half-maximal effective concentration of each compound. According to sensitivity results, proliferation and migration assays were performed using a concentration 1 µM of each compound.

**Results and Discussions**

Results revealed that more than 80% of MDA-453 and IPC-366 express AR, however SUM149 resulted in a weak AR expression. Sensitivity assays showed that all cell lines studied (MDA-453, IPC-366 and SUM149) were sensitive to all treatments, being the cells more sensible to Ailanthone treatment. Regarding in vitro assays, MDA-453 showed a significant reduction in cell viability, specially with Abiraterone and Ailanthone, and significantly reduced cell migration specially with VPC-13566 and Ailanthone treatments. However, SUM149 only showed significant cell viability reduction with Ailanthone treatment, but all treatments reduced cell migration. Interestingly, on IPC-366 only Ailanthone showed a significant reduction in cell viability and cell migration.

**Conclusion**

These preliminary results revealed that blocking AR expression can be an effective therapy for triple negative-IBC, being Ailanthone a potential therapy for its use. However, more research is needed in order to determine the mechanism of action of Ailanthone and its effects on in vivo studies.

### EACR2024-0147
### Effect of KRAS gene dosage and molecular subtype on radiosensitivity in PDAC

*J. Asche*[1], *C. Münch*[2], *M. Trajkovic-Arsic*[2], *J. Matschke*[1], *V. Jendrossek*[1], *J. Siveke*[2]

[1]*University hospital Essen, Institute for Cell Biology-Tumor Research, Essen, Germany*

[2]*University hospital Essen, Brückeninstitut für Experimentelle Tumortherapie BIT, Essen, Germany*

**Introduction**

Late diagnosis, early metastasis as well as a lack of therapeutic targets of pancreatic ductal adenocarcinoma (PDAC) lead to a five-year survival rate below 10 %.

© 2024 The Authors. Molecular Oncology is published by John Wiley & Sons Ltd on behalf of Federation of European Biochemical Societies

Oncogenic mutations in KRAS are harbored by 90 % of PDAC cells  and an additional classification into the classical and the quasi-mesenchymal (QM) subtypes can be performed by analysis of transcriptome and metabolome. The proto-oncogene KRAS encodes for a small GTPase transductor protein, which is involved cell differentiation, cell growth, chemotaxis and apoptosis. Beside surgical resection and chemotherapy, radiotherapy (RT) might also be a useful part of a multimodal therapy concept of PDAC.

**Material and Methods**

This project aims to investigate, how transcriptome, metabolome as well as KRAS gene dosage of PDAC subtypes influence their radiosensitivity. Established and patient-derived PDAC cell lines are analyzed for their KRAS gene dosage, KRAS protein activity and whole transcriptome. In addition, sensitivity to radiation is investigated and KRAS- as well as MEK-inhibitors are applied to reveal alterations in cell proliferation with and without irradiation. Computational modelling  will be performed to correlate KRAS related pathways to the cellular responses after irradiation. Next, the pathways, which in silico revealed an impact on radiosensitivity, will be tested in advanced in vitro models to confirm further relevance. Finally, a restriction of the validated target pathways will be attempted by siRNA application and small molecule inhibitor trials aiming to prevent radioresistance in PDAC.

**Results and Discussions**

KRAS copy numbers in the cell lines were determined via ddPCR. Expression levels were analyzed with RNA Seq data and Ras activation assay. KRAS copy numbers seem to influence but not fully determine active Ras occurrence. Radiosensitivity of the cell lines was determined with limiting dilution assay and reveal a high range of radiosensitivies, which could only be correlated to KRAS gene dosage in QM subtypes. Inhibitor application reveals sensitivity of KRAS G12D- and G12C- inhibitors to the corresponding established cell lines, while MEK-inhibitor showed an overall strong effect on proliferation of all cell lines.

**Conclusion**

All in all, this project will identify individual transcriptomic and metabolomic traits of PDAC subtypes and their dynamic responses to irradiation. Obtained data will help to reveal targetable signaling pathways and predictive biomarkers, which are critical for the enhancement of PDAC therapy.

### EACR2024-0152
### The RNA-binding protein KSRP aggravates malignant progression of renal cell carcinoma through transcriptional inhibition and post-transcriptional destabilization of the NEDD4L ubiquitin ligase

*M.H. Chien[1,2], Y.C. Yang[1], Y.C. Wen[3,4], Y.W. Lin[4,5]*
*[1]Taipei Medical University, Graduate Institute of Clinical Medicine- College of Medicine, Taipei, Taiwan*
*[2]Taipei Medical University, TMU Research Center of Cancer Translational Medicine, Taipei, Taiwan*
*[3]Taipei Medical University, Department of Urology- School of Medicine, Taipei, Taiwan*
*[4]Wan Fang Hospital- Taipei Medical University, Department of Urology, Taipei, Taiwan*
*[5]Taipei Medical University, International Master/PhD Program in Medicine- College of Medicine, Taipei, Taiwan*

**Introduction**

KH-type splicing regulatory protein (KHSRP, also called KSRP), a versatile RNA-binding protein, plays a critical role in various physiological and pathological conditions through modulating gene expressions at multiple levels. However, the role of KSRP in clear cell renal cell carcinoma (ccRCC) remains poorly understood.

**Material and Methods**

KSRP expression was detected by a ccRCC tissue microarray and evaluated by an in silico analysis. Cell loss-of-function and gain-of-function, colony-formation, anoikis, and transwell assays, and an orthotopic bioluminescent xenograft model were conducted to determine the functional role of KRSP in ccRCC progression. Micro (mi)RNA and complementary (c)DNA microarrays were used to identify downstream targets of KSRP. Western blotting, quantitative real-time polymerase chain reaction, and promoter- and 3-untranslated region (3'UTR)-luciferase reporter assays were employed to validate the underlying mechanisms of KSRP which aggravate progression of ccRCC.

**Results and Discussions**

Our results showed that dysregulated high levels of KSRP were correlated with advanced clinical stages, larger tumor sizes, recurrence, and poor prognoses of ccRCC. Neural precursor cell-expressed developmentally downregulated 4 like (NEDD4L) was identified as a novel target of KSRP, which can reverse the pro-tumorigenic and prometastatic characteristics as well as epithelial-mesenchymal transition (EMT) promotion by KSRP in vitro and in vivo. Molecular studies revealed that KSRP can decrease NEDD4L messenger (m)RNA stability via inducing mir-629-5p upregulation and directly targeting the AU-rich elements (AREs) of the 3'UTR. Moreover, KSRP was shown to transcriptionally suppress NEDD4L via inducing the transcriptional repressor, Wilm's tumor 1 (WT1). In the clinic, ccRCC samples revealed a positive correlation between KSRP and mesenchymal-related genes, and patients expressing high KSRP and low NEDD4L had the worst prognoses.

**Conclusion**

The current findings unveil novel mechanisms of KSRP which promote malignant progression of ccRCC through transcriptional inhibition and post-transcriptional destabilization of NEDD4L transcripts. Targeting KSRP and its pathways may be a novel pharmaceutical intervention for ccRCC.

### EACR2024-0153
### Loss of LECT2 contributes to the advancement of ovarian cancer by promoting cancer invasiveness and creating an immunosuppressive environment

*C.J. Wu[1], K.F. Pan[2], K.T. Hua[2]*
*[1]National Taiwan University Hospital- College of*

© 2024 The Authors. Molecular Oncology is published by John Wiley & Sons Ltd on behalf of Federation of European Biochemical Societies

*Medicine- National Taiwan University, Department of Obstetrics and Gynecology, Taipei, Taiwan*
*[2]College of Medicine- National Taiwan University, Graduate Institute of Toxicology, Taipei, Taiwan*

**Introduction**

Epithelial ovarian cancer (EOC), the deadliest gynecologic malignancy, presents clinical challenges. Despite treatments like chemotherapy and targeted therapy, recurrence rates remain high. Immune checkpoint blockade therapy shows limited efficacy due to intrinsic tumor features and an immunosuppressive microenvironment. Human leukocyte cell-derived chemotaxin 2 (LECT2), initially identified as a neutrophil chemotaxin, exhibits immunomodulatory effects, with emerging evidence suggesting its tumor-suppressive role in hepatocellular carcinoma. However, its involvement in EOC progression and its potential therapeutic implications remain largely unexplored.

**Material and Methods**

We evaluated serum levels of Lect2 in human specimens and a syngeneic EOC mouse model. EOC allograft models were also utilized to assess the tumor-inhibiting effects of Lect2 and c-Met targeting. Surveillance of the tumor immune microenvironment, including cytokine levels and immune cell characterization, was also conducted. Additionally, we examined the therapeutic potential of programmed cell death-1 (PD-1)/PD-L1 pathway blockade in mouse models with different Lect2 genotypes.

**Results and Discussions**

We have demonstrated reduced serum levels of LECT2 in patients with epithelial ovarian cancer (EOC), with circulating Lect2 down-regulated as the disease progresses in a syngeneic mouse ID8 EOC model. Using the murine EOC model, we discovered that loss of Lect2 promotes EOC progression by modulating both tumor cells and the tumor microenvironment. Lect2 inhibited the invasive phenotype of EOC cells and suppressed transcoelomic metastasis by targeting c-Met signaling. Additionally, Lect2 downregulation induced the accumulation and activation of myeloid-derived suppressor cells (MDSCs), fostering an immuno-suppressive microenvironment in EOC. Consequently, the therapeutic efficacy of PD-1/PD-L1 pathway blockade for the ID8 model was significantly hindered.

**Conclusion**

Overall, our data highlight multiple functions of Lect2 during EOC progression and reveal a rationale for synergistic immunotherapeutic strategies targeting Lect2.

### EACR2024-0162

### Antimetastatic properties of a low-anticoagulant heparin related to the impairment of tumor cell-platelet and tumor cell-endothelium interactions

*J.M. Motta[1], C. Roberto-Fernandes[1], K.V.A. Micheli[1], A.G.F. Lima[1], V. Morandi[2], P.A.S. Mourão[1], M.S. Pereira[1]*
*[1]Federal University of Rio de Janeiro, Medical Biochemistry Institute, Rio de Janeiro, Brazil*
*[2]State University of Rio de Janeiro, Biology Institute, Rio de Janeiro, Brazil*

**Introduction**

Metastasis is the leading cause of death in cancer patients. During metastatic dissemination, circulating tumor cells need to survive in the bloodstream and their interaction with platelets through P-selectin/ligand seems to be crucial. Subsequently, tumor cells should interact with endothelial cells to extravasate the vessel, and this contact is primarily mediated by VCAM-1/VLA-4. It was shown that heparin (porcine UFH), a glycosaminoglycan composed by repetitive disaccharides of uronic acid and glucosamine, may interfere both with P-selectin and VCAM-1 interactions. Recently our group purified by ion exchange chromatography a fraction from bovine heparin that displays low anticoagulant potential, named LABH (~15% porcine UFH). The advantage of LABH is its reduced risk of bleeding side effects. Our aim here was to investigate the LABH potential as an antimetastatic agent in vivo and evaluate in vitro its ability to interfere with tumor cell-platelets and tumor cell-endothelium interactions.

**Material and Methods**

For this, we challenged 8-12 weeks C57Bl/6 wt or P-selectin KO mice with 4 mg/kg of porcine UFH or LABH followed by intravenous injection of B16F10 cells (murine melanoma). After 21 days, lungs were collected and the metastatic foci were counted. In vitro, MV3 cells (human melanoma) were incubated with platelets for 30 min or with HUVEC cells for 1h in the presence of porcine UFH or LABH. Additionally, MV3 cells were added to plates where we previously immobilized P-selectin or VCAM-1 chimeras.

**Results and Discussions**

The number of metastatic foci was strongly reduced when animals were pretreated with heparins (~60 foci in control vs ~15 in treated mice). Even in P-selectin KO mice, which present lower rates of metastases, LABH was still able to reduce metastasis burden (~25 foci in control vs ~10 in treated mice). In vitro, both heparins inhibited platelet adhesion to tumor cells in a dose-response manner, and similarly blocked tumor cell interaction with HUVEC. Moreover, it was observed that the porcine UFH and LABH could significantly suppress the direct binding of tumor cells to P-selectin or VCAM-1.

**Conclusion**

In summary, despite presenting negligible anticoagulant activity, LABH maintained the antimetastatic potential previously described for the porcine standard heparin and demonstrated a quite interesting multitarget effect to suppress metastasis.

### EACR2024-0170

### Mast cell heparanase increases stem-related features in breast cancer cells

*R. Bongiorno[1], M.T. Majorini[1], D. Lecis[1], M.P. Colombo[1]*
*[1]Fondazione IRCCS Istituto Nazionale dei Tumori, Department of Experimental Oncology, Milan, Italy*

**Introduction**

Mast cells (MCs) constitute a very plastic immune population able to release several classes of mediators and displaying even opposite roles depending on the cancer type. This heterogeneous behavior depends on the

© 2024 The Authors. Molecular Oncology is published by John Wiley & Sons Ltd on behalf of Federation of European Biochemical Societies crosstalk that MCs establish in the microenvironment, and it can be observed also in the different breast cancer subtypes. Indeed, MCs infiltrate more luminal and HER2+ breast cancers where they are also associated with worse prognosis. We hypothesized that this detrimental effect of MCs can be due to the induction of stem-like traits in tumor cells.

**Material and Methods**

Stem-like cells are able to propagate as spheroid bodies, so, we used both human commercial (MCF7) and mouse-derived (PyMT41c) mammary cancer cell lines to assess the effect of MC conditioned medium on mammosphere formation and to investigate the mechanisms that underlie this phenotype. In vivo experiments performed with mice that lack MCs confirmed the role of this immune population in promoting cancer aggressiveness, since the co-injection of breast cancer cells in limiting dilutions with MCs increased the engraftment rate.

**Results and Discussions**

Since we have previously demonstrated that MCs can induce the expression of estrogen receptor (ER) in adjacent cells, we hypothesized that MCs affect ER levels and promote stemness through the induction of an inflammatory microenvironment. In agreement with our hypothesis, the activity of the enzyme heparanase (HPSE), expressed by MCs, causes the upregulation of Muc1-ER axis in cancer cells. HPSE promotes the release of several classes of cytokines and induces the activation of TLR4. Indeed, in our models, we observed that the inhibition of HPSE and the activation of TLR4 on MCs both affect the capacity of cancer cells to form spheroids, by decreasing and increasing it, respectively. Regarding Muc1, we found that the inflammatory microenvironment caused by HPSE stimulated the expression of this mucin in cancer cells. Furthermore, Muc1 silencing impaired the upregulation of ER in cancer cells and also their capacity to form spheroids. In the attempt to understand which factor released by MCs induces stem-related features, we tested inhibitors of a few cytokines released upon TLR4 activation, finding that TNF inhibition reduced the effect of MC conditioned medium in the formation of spheroids.

**Conclusion**

Our findings support the hypothesis that MCs promote the formation of stem-like cancer cells in an ER-dependent manner, opening to the possibility to target MCs for breast cancer treatment.

### EACR2024-0173

### P-cadherin expression is a biomarker of a hybrid EMT phenotype

*C. Esquível[1], L. Faria[2], C. Tomaz[1], T.T. Jesus[3], M.M. Castro[1], R. Canário[1], F. Janody[2], B. Sousa[1], M.J. Oliveira[4], J. Paredes[1]*

*[1]Instituto de Investigação e Inovação em Saúde, Cancer Metastasis Group, Porto, Portugal*

*[2]Instituto de Investigação e Inovação em Saúde, Cytoskeletal Regulation & Cancer, Porto, Portugal*

*[3]Instituto de Investigação e Inovação em Saúde, Cancer Signalling & Metabolism, Porto, Portugal*

*[4]Instituto de Investigação e Inovação em Saúde, Tumour and Microenvironment Interactions, Porto, Portugal*

**Introduction**

Epithelial-to-mesenchymal transition (EMT) is the process by which epithelial (E) cells acquire a mesenchymal (M) phenotype. While traditionally seen as a binary switch, recent evidence highlights the presence of hybrid phenotypes, where cells exhibit traits of both epithelial and mesenchymal phenotypes. Moreover, cancer cells with hybrid phenotypes play a key role in cancer progression by promoting stemness, collective invasion, and immune evasion. P-cadherin (P-cad) is frequently overexpressed in tumors and correlates with poor patient prognosis. Our group has demonstrated that P-cad induces collective cell invasion, stem cell features and *anoikis*-resistance. However, its role as a biomarker for hybrid phenotypes and its involvement in immune evasion remain unexplored. Thus, we aim to evaluate P-cad expression along a dynamic Src-induced EMT model stimulated by Tamoxifen (TAM) treatment.

**Material and Methods**

MCF10A-ER-Src cells were treated with TAM for 6h, 12h, 24h and 36h. Flow cytometry was used to study the expression of E (E-cadherin), M (CD44 and CD61), and hybrid markers (CD104, CD49f, and P-cad). PDL1 expression was evaluated as an immune evasion marker and a Live/Dead kit was used to determine the live cell population. FlowJo10.5.3. and GraphPad Prism.V8 were used for data and statistical analysis, respectively ($p <$ 0.05 considered significant).

**Results and Discussions**

TAM treatment induced E cells to acquire a clear M phenotype after 36h. This was evidenced by a significant decrease in E-cadherin expression and CD49f median fluorescence intensity (MFI), along with an increase in CD61 expression and CD44 MFI over time. Additionally, CD104 MFI was enriched in the early timepoints, with a significant decrease at 36h. Furthermore, both P-cad and PDL1 expression exhibited significant increases over time following TAM treatment.

**Conclusion**

Our observations indicate that P-cad is a biomarker of a hybrid phenotype. Further, we will study if there is a functional relation between P-cad enrichment and the increased expression of PDL1 and a possible role on immune evasion.

### EACR2024-0184

### The investigation of histone methyltransferase G9a-regulated metastasis in head and neck cancer

*H.Y. Ho[1]*

*[1]Changhua Christian Hospital, Oral Cancer Research Center, Changhua, Taiwan*

**Introduction**

Head and neck squamous cell carcinoma (HNSCC) has the characteristics of metastasis and recurrence in the primary location or metastasize to the lymph nodes, leading to advanced stages. Disorders of epigenetic modifications are often found in cancers, and they are also considered to be key markers of cancers. Lysine (K) at specific positions on histone 3, such as H3K4, H3K9, H3K18, and H3K27, is frequently observed to undergo methylation or acetylation. However, few studies have explored the impact of histone methyltransferase G9a/ EHMT2 in oral cancer. This project aims to analyze the

© 2024 The Authors. Molecular Oncology is published by John Wiley & Sons Ltd on behalf of Federation of European Biochemical Societies role of G9a in the invasion and metastasis of head and neck cancer.

**Material and Methods**

The differences of gene expression data and phenotypic data were obtained from The Cancer Genome Atlas database. G9a expression in HNSCC cell lines SCC-1, HSC-3, Ca9-22, and FaDu was assessed using Western blot assays. The real-time PCR, Western blot assay, cell viability assay, cell cycle distribution, wound healing assay, and transwell assay were conducted to investigate the function of G9a in EHMT2 siRNA and EHMT2 overexpression system. The differences of protein expression data of Ca9-22 siEHMT2/siNC and HSC-3 EHMT2 overexpression/pcDNA were analyzed using mass spectrometry.

**Results and Discussions**

In this study, we identified the expression of EHMT2 and found that EHMT2 was higher in various tumor types compared to control tissues, especially in HNSCC, within the TCGA database. Notably, high EHMT2 expression correlated with lymph node metastasis in HNSCC patients. To investigate the function of EHMT2, HNSCC cell lines (SCC-1 and HSC-3) expressing low G9a were transfected with pcDNA or an EHMT2-overexpressed plasmid, while cell lines with high G9a expression (Ca9-22 and FaDu) were transfected with scramble siRNA or EHMT2 siRNA. Cell viability and cell cycle distribution remained unchanged in EHMT2 knockdown and over-expression cells. Wound healing assay and tanswell assays revealed that EHMT2 overexpression activated cell migration and invasion in HNSCC cell lines. In addition, NAP1L1 was upregulated in EHMT2-over-expressed HSC-3 cell line and downregulated in EHMT2 knockdown Ca9-22 cell line. Data from TCGA also provided evidence that high NAP1L1 correlated with high EHMT2 expression and lymph node metastasis.

**Conclusion**

Our finding suggested that EHMT2 induces the migration and invasion of HNSCC cell lines. Moreover, NAP1L1 was identified as a positive correlation target of EHMT2 in the EHMT2 siRNA and EHMT2 overexpression system.

### EACR2024-0191
### Induction G2/M cell cycle arrest was through ROS-mediated HDAC6/p53/p21Waf1/Cip1-dependent signalling pathway by shikonin in pancreatic cancer cells

*M.S. Weng[1], H.P. Huang[1], J.T. Pai[2]*
*[1]Fu Jen Catholic University, Nutritional Science, New Taipei City, Taiwan*
*[2]Tao-Yuan General Hospital- Ministry of Health and Welfare, Division of Hematology and Oncology, Taoyuan City, Taiwan*

**Introduction**

Histone deacetylase 6 (HDAC6) is a pivotal enzyme to increase target protein stability. Ectopic expression of HDAC6 protein and activity has been discovered in numerous cancers. Therefore, searching the bioactive components to target HDAC6-mediated protein stability might be a strategy for cancer therapy and/or prevention. Shikonin, an active component of *Lithospermum erythrorhizon*, has anti-inflammatory, anti-cancer, and anti-metastasis activities. However, it is still a mystery for shikonin-regulated HDAC6-mediated cancer cell proliferation.

**Material and Methods**

We evaluated the cell viability and cell cycle distribution in shikonin-treated cells. Real-time PCR and Western blotting technologies analyzed the mRNA and protein expression of cell cycle regulators, respectively. Immuno-precipitation Western blotting assay was used to verify the association ability of HDAC6 and p53. Flow cytometry detected ROS production in shikonin-treated cells. We also used NAC, a well-known antioxidant, to verify the role of ROS in HDAC6/p53-regulated cell cycle arrest.

**Results and Discussions**

The results revealed that a dose-dependent and time-dependent G2/M cell cycle arrest was induced by shikonin in pancreatic cancer cells. The expression of p53 was suppressed and $p21^{Waf1/Cip1}$ expression was up-regulation in shikonin-treated cells. Silencing $p21^{Waf1/Cip1}$ expression by siRNA reversed shiknonin-inhibited cell viability. Furthermore, the expression of HDAC6 was also suppressed by shikonin. Ectopic expression of HDAC6 protein rescued shikonin-repressed p53 expression and shikonin-suppressed cell viability in pancreatic cancer cells. Meanwhile, the association of p53 and HDAC6 was disrupted by shikonin and resulted in the accumulation of acetylation of p53. The results also indicated that shikonin-inhibited cell viability was through a ROS-dependent pathway. Down-regulation of HDAC6 expression by shikonin was reversed by NAC, a well-known anti-oxidant. Interestingly, shiknonin-inhibited p53 expression and induced $p21^{Waf1/Cip1}$ expression were reversed by NAC treatment. Shikonin-disrupted HDAC6/p53 association was reversed by NAC treatment.

**Conclusion**

Accordingly, the results implicated that shikonin-induced G2/M cell cycle arrest might be through ROS-mediated HDAC6/p53/$p21^{Waf1Cip1}$-dependent signaling pathway in pancreatic cancer cells.

### EACR2024-0196
### Evaluation of the combinatory anticancer effect of chemotherapeutic compounds and Prodigiosin against HCT116, LoVo, and A549 Cell lines

*F. Elghali[1], D. Msalbi[2], M. Alonazi[3], E. Sahli[4], B. Hakim[2], S. Mnif[2], A. Ben bacha[3], M.S. Aifa[5]*
*[1]Center of Biotechnology of Sfax, LPCMC, Safx, Tunisia*
*[2]Centre of Biotechnology of Sfax, Lpcmc, Sfax, Tunisia*
*[3]Science College- King Saud University, Biochemistry Department, Riadh, Saudi Arabia*
*[4]Centre of Biotechnology of Sfax, Unity of Analysis, Sfax, Tunisia*
*[5]Centre of Biotechnology of Sfax, Lpcmc, Safx, Tunisia*

**Introduction**

Chemotherapy is still among the most adopted treatment of cancer such 5-FU, Paclitaxel, oxaliplatin and others. Prodigiosin (PG), an example of a secondary metabolite

© 2024 The Authors. Molecular Oncology is published by John Wiley & Sons Ltd on behalf of Federation of European Biochemical Societies from various bacteria, exerts different biological activities including cancer-preventive and anticancer effects. Despite their wide usage in reducing tumors and improving patients' survival, chemotherapeutic drugs, and other natural compounds are facing the development of cancer resistance. Many experimental data and clinical trials are showing that combinatorial treatment could be an efficient solution for some resistance problems

**Material and Methods**

the combinatorial treatment of chemotherapeutic drugs showed variable effects on the used cell lines, ranging from a toxicity improvement to a neutral effect or a decrease in toxicity.

**Results and Discussions**

PG effectively inhibited cell proliferation and induced apoptosis. The combination of 20 µM of PG + IC50 of paclitaxel and oxaliplatin increases cell viability by more than 50%, while the combinatorial treatment of 20 µM of PG + IC50 of 5-FU shows a significant decrease in cell viability in the three cancer cell lines; LoVo: 48% HCT-116: 42% and A549: 36%. Based on PG and 5-FU, the percentage of cells in the G0/G1 phase was higher and the percentage of cells in the S phase was lower compared to treated cells separately by PG and 5-FU in all the used cell lines. Our docking studies supported targeting Akt1 by PG which could explain its proapoptotic effect separately or in combination with 5-FU. PG inhibited tumor growth and enhanced the 5-FU therapeutic efficacy in HCT116, LoVo, and A549 cells. Taken together, our findings highlight that PG effectively inhibited the growth of tumors and enhanced the sensitivity to thermotherapy, indicating PG is an inhibitor of Akt1.

**Conclusion**

It was concluded that PG has the ability to interact with Akt1, and in silico results confirmed the previous studies which showed that PG interacts with Akt1. The co-treatment of PG with 5-Fu promotes programmed cell death (apoptosis) in HCT116, LoVo and A549 cells and reduces the toxicity of 5-FU (used low doses of 5-FU).

### EACR2024-0208

### Deciphering the interplay of tumor cells, immune cells and fibroblasts of peritoneal metastases of colorectal cancer using a 3D co-cultivation system

*M. Sachet[1], N. Walterskirchen[1], C. Ramos[1], C. Müller[1], H.D. Helmut Dolznig[2], T. Bachleitner-Hofmann[1], R. Oehler[1]*

*[1]Medical University of Vienna, Department of General Surgery, Vienna, Austria*

*[2]Medical University of Vienna, Institute of Medical Genetics, Vienna, Austria*

**Introduction**

Tumour cell growth and spreading can be influenced by stromal cells, especially by fibroblasts and immune cells. However, the knowledge about this interplay in the setting of peritoneal metastasis is still limited. 3D cultivation of human primary tumor cells has become recently a standard tool to investigate tumor cells ex vivo. Here we developed a method to investigate the interaction of tumor cells with fibroblasts, macrophages and T-cells in a co-culture model.

**Material and Methods**

Cancer tissue and peripheral blood was prepared from patients with primary colorectal cancer (pCRC) or peritoneal metastasis (pmCRC). Tumour cells, fibroblasts and autologous monocytes/macrophages and T cells were isolated and seeded in a 3D collagen gel. In triple- and quadruple culture experiments, using fluorescence microscopy and flow cytometry, we investigated which cell type or which specific heterotypic cell interaction is responsible for tumor cell growth and invasion, macrophage differentiation, and T cell activation.

**Results and Discussions**

Co-cultivated peritoneal fibroblasts induced a significantly higher invasiveness of cancer cells as compared to fibroblasts from pCRC. Furthermore, peritoneal fibroblasts reduced the expression of CD86, CD163, and HLA-DR on macrophages pointing to an anti-inflammatory polarisation of these cells. Finally, we found that peritoneal fibroblasts reduced the activation and proliferation of T-cells.

**Conclusion**

Taken together, these results indicate that metastatic peritoneal fibroblasts boost tumor cells invasion and at the same time contribute to an immunosuppressive tumor microenvironment in pmCRC. This suggests that targeting these cells might be an interesting anti-tumor approach in peritoneal metastasis.

### EACR2024-0212

### Contactin 4-mediated Tumor Suppressor Functions through Coupling with Protein Tyrosine Phosphatase Receptor Type G in Colorectal Cancer

*M.H. Tsai[1], Y.H. Tang[2], H.T. Lee[2], I.C. Lin[2], P.L. Chen[2], J.X. Lee[2], S.Y. Chiang[2], T.M. Jao[3], Y.C. Yang[2]*

*[1]Cardinal Tien Hospital, Department of Surgery, New Taipei City, Taiwan*

*[2]National Taiwan University College of Medicine, Department of Clinical Laboratory Sciences and Medical Biotechnology, Taipei, Taiwan*

*[3]National Taiwan University College of Medicine, Global Innovation Joint-Degree Program International Joint Degree Master's Program in Agro-Biomedical Science in Food and Health, Taipe, Taiwan*

**Introduction**

The incidence of colorectal cancer (CRC) has been rising around the world. In our previous study, a minimal deletion region (MDR) at 3p26.2-p26.3 was delineated in CRC tumors via loss of heterozygosity study (LOH), followed by clinical relevance assessment of CRC patients.

**Material and Methods**

Using LOH analyses, we further performed a fine deletion mapping with 179 colorectal carcinomas to disclose the putative tumor suppressor gene (TSG). The expression of Contactin 4 (CNTN4) was analyzed in CRC cell lines, paired primary tissues and public datasets from TCGA and GEO databases. Tumor suppressor activity of CNTN4 was identified by in vitro cell models and tumor xenograft models. CNTN4-

Molecular Oncology 18 (Suppl. 1) (2024) 1–495

© 2024 The Authors. Molecular Oncology is published by John Wiley & Sons Ltd on behalf of Federation of European Biochemical Societies modulated molecular mechanisms in CRC cells were explored by using gene set enrichment analysis (GSEA) of RNA-sequencing data, followed by further experimental verification. The study was conducted in accordance with the Declaration of Helsinki and all patients provided informed consent as approved by the Institutional Review Board of Cardinal Tien Hospital.

**Results and Discussions**

Within the MDR, we proposed CNTN4, a cell adhesion molecule, as a candidate TSG associated with CRC. CNTN4-expressing single stable clones established in CRC cell lines exhibited attenuated malignant phenotypes, including cell proliferation, anchorage-dependent and -independent colony formation, as well as reduced xenograft tumorigenicity in nude mice. Based on literature review, we expected that CNTN4 may interact with PTPRG (Protein Tyrosine Phosphatase Receptor Type G), and thereupon mediate tumor suppression via decreasing the phosphorylation of different components of EGFR signaling pathways. Therefore, we first verified the interaction between CNTN4 and PTPRG by proximity ligation assay. Subsequently, we demonstrated CNTN4 expression decreased EGFR phosphorylation after EGF treatment. GSEA revealed that CNTN4 expression is negatively correlated to PI3K signaling pathway related genes. Correspondingly, the expression of PI3K, AKT and mTOR was down-regulated in CNTN4-expressing CRC cells, indicating that CNTN4 could inhibit the PI3K/AKT/mTOR signaling pathway, resulting in the modulation of the 4EBP1-eIF4E axis to block mRNA translation and cell proliferation.

**Conclusion**

CNTN4 is a novel tumor suppressor through coupling with PTPRG to reduce phosphorylation of the molecules involved in EGFR-associated signaling pathways, and further to decrease the expression of tumor-promoting factors in CRC.

### EACR2024-0220

### Mechanisms underlying the enhanced aggressiveness of apoptosis reversed cancer cells

*Y.W. Chong*[1], *L. Yiu*[1], *S.Y. Tsang*[1,2]
[1]*The Chinese University of Hong Kong, School of Life Sciences, Hong Kong SAR, Hong Kong-China*
[2]*The Chinese University of Hong Kong, State Key Laboratory of Agrobiotechnology, Hong Kong SAR, Hong Kong-China*

**Introduction**

Cancer relapse remains a great challenge to be solved regardless of the variety of treatments available. It was reported that cancer cells could escape and survive chemotherapy by reversal of apoptosis, the programmed cell death, resulting in more aggressive cells that promote cancer recurrence, but the exact mechanism remains to be elucidated. Mitophagy is the selective macroautophagy of mitochondria, which participates in mitochondria quality control to support cancer cell physiology and contributes to the aggressive properties like chemoresistance.

**Material and Methods**

Reversal of apoptosis was performed on human breast cancer cells (BCC) to derive reversed BCCs (BCC-R). The level of mitophagy was measured by mt-Keima. The effect of apoptotic inducer and several mitophagy inhibitors on BCC and BCC-R were investigated by MTT assay. BCC lines with mitophagy-related protein knockdown were established by the lentiviral vector to study the role of mitophagy in modulating cancer cell aggressiveness.

**Results and Discussions**

Mitophagy level was found to increase upon apoptosis reversal. No significant difference was found between the effect of apoptotic inducer on BCC and BCC-R. Interestingly, one among the three tested mitophagy inhibitors showed a more significant inhibitory effect on BCC-R than on BCC. In addition, knockdown of mitophagy proteins increased the sensitivity of BCCs to the apoptotic inducer.

**Conclusion**

Findings from this study have suggested the possible relationship between mitophagy and the enhanced aggressiveness of apoptosis reversed BCCs. The findings imply the potential of targeting mitophagy-related molecules in cancer treatment and in prevention of cancer recurrence.

### EACR2024-0226

### Reprogramming of lipid metabolism in apoptosis reversed breast cancer cells

*L. Yiu*[1], *Y.W. Chan*[1], *S.Y. Tsang*[1,2]
[1]*The Chinese University of Hong Kong, School of Life Sciences, Hong Kong, Hong Kong-China*
[2]*The Chinese University of Hong Kong, State Key Laboratory of Agrobiotechnology, Hong Kong, Hong Kong-China*

**Introduction**

Previous studies have shown that, when chemo-therapeutic drugs are applied to cancer cells, if the drugs are withdrawn at an inappropriate time, some of the cells can survive from apoptosis in a process known as apoptosis reversal. The reversed cancer cells become more aggressive in terms of proliferation and metastasis, and have an elevated populations of cancer stem cells (CSCs). To date, the underlying mechanism of the apoptosis reversal remains to be studied. Peroxisome is one of the organelles for lipid metabolism. One of the crucial metabolic activities in peroxisome is fatty acid oxidation (FAO). We previously reported that the aggressiveness of the apoptosis reversed cells were increased. Here, we hypothesized that the fatty acid metabolism through peroxisome would be altered to provide more energy for the survival and growth of the apoptosis reversed cells.

**Material and Methods**

SWATH-MS was used to quantify the amount of different proteins in the apoptosis reversed and control breast cancer cells. Data from SWATH-MS was imported to Gene Set Enrichment Analysis (GSEA). 41 proteins in peroxisome and FAO by peroxisome were quantified. Quantitative reverse transcription PCR (RT-qPCR) was performed to assess the expressions of peroxisomal- and FAO-related genes. Immunostaining and live cell staining of peroxisome followed by confocal microscopy were performed to assess the size and number of peroxisomes.

**Results and Discussions**

© 2024 The Authors. Molecular Oncology is published by John Wiley & Sons Ltd on behalf of Federation of European Biochemical Societies

Based on the related hallmark proteins, fatty acid metabolism and peroxisome were found to be upregulated in the apoptosis reversed cells. We found that some of the FAO-related genes were upregulated in apoptosis reversed cells. Also, the size and number of peroxisome were increased significantly in apoptosis reversed cells. The amount of acetyl-CoA was increased in trend, which might indicate the increase in FAO of long-chain fatty acid in apoptosis reversed cells.

**Conclusion**

This work demonstrated that the lipid metabolism through peroxisome may have been reprogrammed in apoptosis reversed breast cancer cells. In the near future, we will elucidate how FAO contributes to the apoptosis reversal of breast cancer.

### EACR2024-0231
### CDK9 inhibitor-induced transcriptional stress actives innate immune response in cancer cells

*S. Yalala[1], A. Gondane[1], N. Poulose[2], J. Liang[1], I. Mills[2], H. Itkonen[1]*

*[1]University of Helsinki, Department of Biochemistry and Developmental Biology, Helsinki, Finland*

*[2]University of Oxford, Nuffield Department of Surgical Sciences, Oxford, United Kingdom*

**Introduction**

Cancer cells frequently exhibit hyper-activation of transcription, which can lead to increased sensitivity to compounds targeting the transcriptional kinases, in particular cyclin-dependent kinase 9 (CDK9). However, the mechanism-of-action of CDK9 inhibitors on cancer cells is largely unknown.

**Material and Methods**

We use time-resolved transcriptional profiling (SLAM-seq), ChIP-seq, alternative splicing analysis, targeted proteomics and secretome-profiling to define how prostate cancer cells respond to CDK9 inhibition. To confirm the significance of our results, we apply the appropriate statistical tests including Kruskall-Wallis and two tailed Student's t tests.

**Results and Discussions**

In MYC over-expressing prostate cancer cells, CDK9 inhibition leads to excessive accumulation of mis-spliced RNAs, which are prone to form double-stranded RNA (dsRNA). PKR (dsRNA-activated kinase) can recognize these mis-spliced RNAs, and we show that the anti-proliferative effects of CDK9 inhibition can be reversed by co-treating the cells with a PKR inhibitor. Using SLAM-seq, targeted proteomics and ChIP-seq, we show that, similar to viral infection, CDK9 inhibition significantly suppresses transcription of most genes but allows selective transcription and translation of the cytokine genes including *TNFα*, *CXCL10* and *CXCL8*. In particular, CDK9 inhibition activates NFkB-driven cytokine-signaling at the transcript- and the secretome-levels. A transcriptional signature induced by CDK9 inhibition is associated with prostate cancers with a high level of genome instability.

**Conclusion**

We show that CDK9 inhibition activates innate immune response through viral mimicry in prostate cancer cells. In the future, it is important to establish if CDK9 inhibitors can potentiate the effects of immunotherapy against the late-stage prostate cancer, a currently lethal disease.

### EACR2024-0235
### Unveiling Senescent Cell Heterogeneity: Characterizing Origin and Traits through CRISPaint-Engineered LNCaP Clones

*M. Saponaro[1], N. Mönig[2], M. Effern[3], A. Winkler[1], D. Schmidt[1], K. Wörsdörfer[1], M. Ritter[1], M. Hölzel[3], A. Alajati[1]*

*[1]University Hospital Bonn, Department of Urology and Pediatric Urology, Bonn, Germany*

*[2]Max Planck Institute for Biology of Ageing, AG Jachimowicz, Cologne, Germany*

*[3]University Hospital Bonn, Institute of Experimental Oncology, Bonn, Germany*

**Introduction**

Cellular senescence consists of the blockade of the cell cycle after DNA damage and frequently occurs following chemotherapy in prostate cancer. Despite its anti-proliferative effects on tumors, senescence-associated secretory phenotype (SASP) paradoxically facilitates tumor cell proliferation. Therefore, eliminating senescent cells that develop after chemotherapy appears crucial to improve patient prognosis. However, targeting senescence remains challenging due to senescent cells' heterogeneity and their proteomic similarity to proliferating cells. Therefore, the establishment of a solid method to study cell populations post-chemotherapy is essential to identify new targets for senescent cell clearance.

**Material and Methods**

Senescence markers (p16, p21, ß-Gal) were engineered with the CRISPaint method, creating C-terminal mNeon-tagged fusion genes in LNCaP. To mitigate protein tagging interference, our CRISPaint constructs encode a self-cleaving peptide (P2A) preceding mNeon. Thus, mNeon is cleaved off during translation, and tagged proteins can exert their function. Using this method, 3 engineered LNCaP cell lines (p16-mNeon, p21-mNeon, ß-Gal-mNeon) were generated. LNCaP sublines were treated with docetaxel to induce senescence, and cells were sorted by flow-activated cell sorting (FACS) based on their mNeon expression intensity. mRNA was extracted from sorted cell populations and mRNA sequencing was performed.

**Results and Discussions**

FACS analysis on engineered LNCaP before and after treatment with docetaxel revealed that they cluster into 3 populations based on mNeon intensity (mNeon-1+, mNeon-2+, and mNeon-3+). Remarkably, mNeon-3+ is exclusive to docetaxel-treated condition, suggesting that they might be the chemotherapy-induced senescent population. The presence of mNeon-2+ cells in the untreated and treated groups suggests that different senescence stages might be present. Indeed, mRNA sequencing revealed a cluster of genes, some of which were not previously reported in senescence, showing a progressive upregulation along populations. Thus, single-cell RNA sequencing is planned to uncover the heterogeneity of senescent populations and their origin.

**Conclusion**

© 2024 The Authors. Molecular Oncology is published by John Wiley & Sons Ltd on behalf of Federation of European Biochemical Societies

CRISPaint mutants facilitated the successful visualization of senescent cells and the identification of potentially diverse senescent stages or populations. The current focus is on characterizing these populations and their origins, as comprehending senescence heterogeneity in tumors may unveil new therapeutic avenues.

### EACR2024-0241
### The secreted protein ANGPTL4 drives proinflammatory secretome of senescent cells and promotes tumorigenesis

*G. Makulyte[1], D. Goerhig[1], A. Huna[1], A. Palazzo[1], N.S. Tan[2], P. Dubus[3], C. Mann[4], P. Bertolino[1], J.M. Flaman[1], D. Bernard[1]*
*[1]Cancer Research Center of Lyon, Cancer Initiation and Tumor cell Identity, Lyon, France*
*[2]Nanyang Technological University Singapore, Lee Kong Chian School of Medicine and School of Biological Sciences, Singapore, Singapore*
*[3]Bordeaux Institute of Oncology, Université de Bordeaux, Bordeaux, France*
*[4]Institute for Integrative Biology of the Cell, Genome Biology Department, Paris, France*

**Introduction**
Oncogene-induced senescence (OIS) acts as a fail-safe program that inhibits tumorigenesis. However, emerging evidence suggests that the accumulation of senescent cells due to decreased elimination and/or increased exposure to pro-senescent stressors can promote cancer development. The senescence-associated phenotype (SASP), which encompasses growth factors, proteases, pro-inflammatory cytokines and/or pro-fibrotic factors, is considered particularly important in mediating the pro-tumoral effects of senescent cells. Nevertheless, the regulation, composition and role of the SASP are far from being fully understood, especially during OIS and beyond the well-studied proinflammatory factors.
**Material and Methods**
In this study, we used in silico transcriptomic dataset analysis to identify conserved SASP factors. In order to analyze ANGPTL4 function, we mainly used normal human fibroblasts transduced with vector coding B-RAF:ER as a model of OIS. We used retroviral and lentiviral transductions and siRNA technologies to overexpress proteins or knock down genes of interest. In order to analyze mRNA expression, we used RT-qPCR, for protein–western blot or Elisa. Genome-wide localization of HIF2A was performed by Cut & Tag technology. To investigate ANGPTL4 role in lung tumorigenesis, we infected KrasG12D,R26RTomato mice with Lenti-CRE virus (to activate Kras and Tomato) and treated them with ANGPTL4 blocking antibody. Immunohistochemistry was used to analyze protein expression in the lung of mice. GSEA was performed using GSEA website on TCGA lung cancer datasets.
**Results and Discussions**
We demonstrated that ANGPTL4 is one of the rare secreted factors induced in many different types of senescent cells. During OIS, a hypoxia-like response is activated and the HIF2A transcription factor is involved in ANGPTL4 upregulation. Importantly, ANGPTL4 knockdown or its constitutive expression, respectively, inhibits or induces classical proinflammatory SASP factors during OIS. The latter effect is mediated upstream of the IL1A/NF-kB pathway. Furthermore, ANGPTL4 levels are high in human lung tumors and are correlated with poor prognosis. Blocking ANGPTL4 antibody attenuates KRAS-dependent lung tumorigenesis in mice, which was associated with downregulation of IL1A. Moreover, high ANGPTL4 expression in human lung tumors is associated with hypoxia, SASP and proinflammatory signatures.
**Conclusion**
These findings shed light on ANGPTL4 as a critical SASP factor promoting senescence-associated chronic inflammation and tumor development.

### EACR2024-0242
### Metformin Treatment Reduces CRC Aggressiveness in a Glucose-Independent Manner

*M. Boutaud[1], N. Christou[2], M. Verdier[1]*
*[1]OmegaHealth, Inserm UMR1308- Faculté de Médecine, Limoges, France*
*[2]Centre Hospitalier Universitaire de Limoges, Service de Chirurgie Digestive, Limoges, France*

**Introduction**
According to the World Health Organisation report (Globoccan, 2020), colorectal cancer (CRC) is the second most deadly cancer. Furthermore, CRC frequently reoccurs even when detected at its initial stages. Epithelial–mesenchymal transition (EMT) is a pivotal process promoting the dissemination of cancerous cells. It is chiefly characterized by the loss of epithelial markers like E-cadherin, promoting cell movements, partly attributed to extracellular matrix modification by metalloproteinases such as MMP2 and MMP9. Metformin, primarily employed in the management of type 2 diabetes due to its actions on mitochondrial metabolism and AMPK, has been investigated for its inhibitory effects on EMT in various cancer types, although its impact on colorectal cancer remains under explored.
**Material and Methods**
This study aims to investigate the influence of Metformin on the suppression of EMT-related genes, migration, and invasion in 2 stages colorectal cancer cell lines (HCT-116 and SW-620). Special attention is given to its effect on E-cadherin and MMPs. In addition, we investigated the part of AMPK in the observed metformin effects using AMPK siRNA. To assess the effect of glucose on metformin-induced EMT inhibition in vitro, all experiments were conducted under two glucose conditions, normoglycemia (7.8 mM) and hyperglycemic conditions (17.5 mM).
**Results and Discussions**
The results indicate that metformin lead to favourable eeffects especially in early stage of colorectal cancer in vitro. Indeed, metformin appears to reduce the cleavage of E-cadherin during EMT, which is coupled with a decrease of the metalloproteases MMP2 and MMP9. This effect is mediated by activation of AMPK. In addition, the migratory and invasive capacities of the cells were drastically reduced after metformin treatment. This action is similar in both glucose conditions, it could suggest a beneficial effect of metformin on non-diabetic patients

© 2024The Authors. Molecular Oncology is published by John Wiley & Sons Ltd on behalf of Federation of European Biochemical Societies with normal blood sugar levels. Such obvious results seem not to be so clear in late CRC stage. Further experiments are still going on to validate these results.

**Conclusion**

In summary, our study showed the beneficial effects of metformin in reducing colon cancer progression. This noteworthy result relied on the independence of glucose concentration and emphasized the effects mainly in the early stages. Although such observations need to be confirmed, this bodes well for adjuvant treatment with metformin in the early stages of cancer in both diabetic and non-diabetic patients.

### EACR2024-0243

### Long pentraxin-3 as a tumor promoter in SHH-Medulloblastoma

*S. Filiberti*[1], *C. Tavani*[1], *D. Capoferri*[1], *R. Bortolozzi*[2], *E. Rampazzo*[2], *G. Viola*[2], *T. Annese*[3], *A. d'Amati*[3], *A. Turtoi*[4], *R. Ronca*[1]

[1]*University of Brescia, Department of Molecular and Translational Medicine, Brescia, Italy*

[2]*University of Padova, Department of Woman's and Child's Health, Padova, Italy*

[3]*University of Bari "Aldo Moro", Department of Translational Biomedicine and Neuroscience DiBrainN, Bari, Italy*

[4]*University of Montpellier- IRCM, Tumor Micro-environment and Resistance to Treatment Lab, Montpellier, France*

**Introduction**

Medulloblastoma (MB) is an aggressive neuroectodermal tumor of the cerebellum and represents the most common brain tumor of the childhood. MB comprises four distinct subgroups: WNT, SHH, group 3, and group 4. With an annual incidence of 5 cases per 1 million individuals, it is noteworthy that 30%-40% of MB patients encounter tumor recurrence. In the treatment of MB, the balance between effective therapeutic intervention and avoiding severe neurocognitive deficits is crucial. To date,despite efforts to understand MB biology and identify treatment targets, a lot remains to be investigated. Long Pentraxin 3 (PTX3), an innate immunity component implicated in tumorigenesis, has unclear roles in cancer, acting as either an oncosuppressor or a pro-tumoral factor. Nowadays, no data is available regarding the impact of PTX3 in MB.

**Material and Methods**

PTX3 expression was analysed in MB cell lines and subgroups, by Western blot (WB), immuno-histochemistry (IHC) and R2 database analysis. Then, PTX3 was knocked-down, using short hairpin RNA (shRNA) and knocked-out (KO), using CRISP/Cas9 technology, in the prototypic DAOY cell line (representing the SHH subgroup). Silenced cells and KO clones were used to elucidate the role of PTX3 in SHH-MB performing both in vitro and in vivo assays.

**Results and Discussions**

Our results show that PTX3 is expressed at low levels across the MB-subgroups, while is overexpressed in the SHH subgroup. This was further confirmed by additional analyses on patient-derived samples using IHC and RNAscope techniques. In vitro assays revealed that both the silencing and the KO of PTX3 in DAOY cells significantly impaired key tumor features, including proliferation, migration and clonogenic potential. Also, metabolomic analysis revealed decreased glycolysis and TCA cycle activity in both PTX3 silenced and KO cells. In vivo, PTX3-KO clones exhibited a reduced tumor growth when implanted subcutaneously in immune-deficient mice, and IHC showed lower proliferation (Ki67$^+$ cell percentage) and diminished vascularization (CD31$^+$ cells) in the absence of PTX3. Accordingly, a chicken embryo chorioallantoic membrane (CAM) assay further supported these findings, demonstrating a reduction in the angiogenic potential of MB cells in the absence of PTX3.

**Conclusion**

These data support the hypothesis of a pro-oncogenic effect of PTX3 in SHH-MB and provide the basis to better study the molecular pathways driven by PTX3 in this tumor subtype.

### EACR2024-0244

### Impact of cathepsin B and X inhibition on signaling pathways in breast cancer stem cells

*A. Mitrović*[1,2], *D. Knez*[2], *I. Sosič*[2], *S. Gobec*[2], *J. Kos*[1,2]

[1]*Jožef Stefan Institute, Department of Biotehnology, Ljubljana, Slovenia*

[2]*University of Ljubljana, Faculty of Pharmacy, Ljubljana, Slovenia*

**Introduction**

In treatment of breast cancer many patients still experience drug resistance and tumor relapse, whereas especially recurrent form of breast cancer remains incurable. Tumor relapse emerges mainly due to the presence of small population of cancer stem cells (CSCs). Efficient targeting of CSCs could lead to a longer period without disease or a full recovery for breast cancer patients and is therefore the major challenge in the development of new therapeutics for cancer treatment. Lysosomal cysteine peptidases, cathepsins B and X that have important role in different processes of development and progression of cancer, could serve as promising molecular targets. Cathepsins B and X are unique among cysteine peptidases due to their carboxypeptidase activity. Increased activity of both cathepsins can be selectively regulated at multiple levels, including specific small molecular inhibitors. In this study we evaluated the impact of cathepsin B and X inhibition on signaling pathways activated in CSCs.

**Material and Methods**

CSCs were isolated from breast cell lines based on their ability to form tumorspheres. Isolated CSCs after tumorsphere formation were treated with selective, reversible small molecular inhibitors of cathepsins B and X. We determined the effect of cathepsin B and X inhibition on phenotype of CSCs using Western blot and qPCR. The effect of cathepsin B and X inhibition on signaling pathways in CSCs was determined by following expression of selected proteins, involved in key signaling pathways by Western blot. Additionally, we determined the effect of inhibition of signal kinases on cathepsins B and X protein levels and activity in CSCs.

**Results and Discussions**

© 2024 The Authors. Molecular Oncology is published by John Wiley & Sons Ltd on behalf of Federation of European Biochemical Societies

Our results show that cathepsin B and X inhibitors effected CSC phenotype by decreasing the expression of stemness markers and markers of mesenchymal cell phenotype. Next, we demonstrated that cathepsin B and X inhibition effects signaling pathways in CSCs important for tumor progression. Moreover, following inhibition of signaling kinases we demonstrated the involvement of cathepsins B and X in signaling pathways of CSCs.

**Conclusion**

In conclusion the results of this study give mechanistic insight on how cathepsin B and X inhibition effects CSCs and confirm the use of cathepsins B and X inhibitors as promising approach to improve existing antitumor therapy.

### EACR2024-0249
### Drp-1 mediated mitochondrial fragmentation and its spatial re-localization facilitate TGF-β induced EMT in glioblastoma cells

*M. Choudhary*[1], *R. Chowdhury*[1], *S. Mukherjee*[1]
[1]*Birla Institute of technology and sciences, Biological sciences, Pilani- Jhunjhunu, India*

**Introduction**

Glioblastoma multiforme (GBM) is an aggressive tumor with poor prognosis. Despite advancements in combination treatments using radiation and chemo-therapy, the median patient survival after initial diagnosis remains as low as 14.6 months. Herein, induction of epithelial to mesenchymal transition (EMT) in GBM cells is known to impart invasive features to this cancer type. During EMT cells lose apical-basal polarity, cell-cell connections leading to the spread of cancer, acquisition of additional stem cell-like characteristics and/or chemo-resistance. In this regard, how organelle dynamics shapes EMT is poorly understood. This study investigates the contribution of mitochondria in regulating EMT in GBM cells.

**Material and Methods**

U-87 MG and LN-229 cell lines were obtained from National Centre for Cell Science (NCCS), Pune, India. Cells were further grown in appropriate medium supplemented with 10% FBS. Mitochondrial morphology was analyzed by MitoTracker Deep Red FM staining through confocal microscopy. Cytoskeletal alterations were analyzed by phalloidin staining and scanning electron microscopy (SEM). Further, western blotting, qRT-PCR and immunofluorescence were used to determine the expression of various proteins and genes involved in the study. Cell cycle, mitochondrial ROS and mitochondrial membrane potential were determined using flow-cytometer.

**Results and Discussions**

We observed that a proliferative arrest was associated with TGF-β-induced EMT in GBM cells. Interestingly, EMT undergoing cells showed a fragmented mitochondrial morphology, high expression of fission markers such as DRP1, MFF, FIS1 and importantly a transportation of mitochondria to the cell boundaries. This was associated with a high mitochondrial ROS but an increased oxygen consumption rate. Importantly, si-RNA mediated knockdown of DRP-1 or pharmaceutical suppression of fission by Mdivi-1 resulted in the reversal of EMT phenotype alongside redistribution of cytoskeleton as analyzed through phalloidin staining. Alternatively, cytoskeleton disrupting drugs reoriented the spatial distribution of mitochondria to perinuclear region negatively impacting EMT.

**Conclusion**

Taken together, our study provides critical insights into the existing crosstalk between mitochondrial spatial and functional dynamics with cytoskeletal elements regulating EMT which can have a deep therapeutic implication. Keywords: Mitochondrial dynamics, cytoskeletal remodeling, Epithelial-mesenchymal transition, Mitochondrial ROS

### EACR2024-0258
### Differential effects of coverslip-induced hypoxia and cobalt chloride mimetic hypoxia on cellular stress, metabolism, and nuclear structure in CAL-27 oral cancer cells

*G.M. Arocena*[1]
[1]*Facultad de Odontología- Universidad de la República, Departamento de Biología Odontológica, Montevideo, Uruguay*

**Introduction**

Hypoxic conditions have profound effects on cell physiology and organization, and hypoxic micro-environments are key determinants of cell behavior in cancer. We have begun characterizing a variant of coverslip-induced hypoxia that recapitulates the gradients of intracellular hypoxia observed in the tumor micro-environment, and we asked whether coverslip-induced hypoxia would have similar effects on HIF-1α induction, cellular stress levels, mitochondrial metabolism and cellular organization compared to the well established mimetic model of hypoxia induced by cobalt chloride

**Material and Methods**

We studied CAL-27 oral squamous carcinoma cells either in coverslip-induced hypoxia or treated with cobalt chloride. We assessed nuclear HIF-1α by immuno-cytochemistry and stress levels by reactive oxygen species production using a fluorescent probe, as well as by lipid droplet accumulation. We assessed mitochondrial ATP production using a specific fluorescent probe, and we evaluated changes in nuclear organization by measuring nuclear dry mass density distribution using quantitative phase microscopy.

**Results and Discussions**

Similar levels of nuclear HIF-1α were observed after 24h of coverslip-induced hypoxia or cobalt chloride treatment. Cellular stress levels were markedly increased in coverslip-induced hypoxia, whereas mitochondrial ATP production sharply decreased in this condition, but not with cobalt chloride. Coverslip-induced hypoxia also had profound effects in nuclear organization, which were much less marked after cobalt chloride treatment. Taken together, our results show that coverslip-induced hypoxia effects on cell physiology and structure are more pronounced than mimetic hypoxia induced by cobalt chloride treatment.

© 2024 The Authors. Molecular Oncology is published by John Wiley & Sons Ltd on behalf of Federation of European Biochemical Societies

**Conclusion**
Considering also the simplicity of coverslip-induced hypoxia, our results therefore underscore the usefulness of this method to recapitulate in vitro the effects of hypoxic microenvironments encountered by cancer cells in vivo.

### EACR2024-0266
### Characterizing the roles of post-translational modifications on tumor suppressive functions of LACTB

C.W. Phang[1], Z. Kečkéšová[2]
[1]*Institute of Organic Chemistry and Biochemistry of the Czech Academy of Sciences IOCB Prague, Biochemisry and Molecular Biology, Prague, Czech Republic*
[2]*Institute of Organic Chemistry and Biochemistry of the Czech Academy of Sciences IOCB Prague, Biochemistry and Molecular Biology, Prague, Czech Republic*

**Introduction**
The eukaryotic serine β-lactamase-like protein (LACTB) is a recently discovered mitochondrial tumor suppressor that was shown to exert its tumor suppressive activities in many types of cancers. Its mechanism of action involves modulation of cancer cell differentiation, apoptosis, epithelial-mesenchymal transition (EMT) and autophagy. Given its widely spread important roles in tumor suppression, it will be of great significance to identify the role and regulatory potential of post-translational modifications (PTMs) on LACTB thus gaining the ability to modulate LACTB expression in cancer cells. However, the mechanisms behind the occurrence of PTMs on LACTB and their biological significance currently represents an understudied in cancer.
**Material and Methods**
In order to identify the PTMs on LACTB, peptide immunoaffinity enrichment coupled with MS-based proteomics was carried out to identify the exact PTM sites on LACTB. Upon identifying the PTM sites, site-directed mutagenesis has been carried out to investigate the regulatory functions of the PTM on LACTB's tumor suppressive function, enzymatic activity, and polymerization. To further investigate the potential proteins that regulate the PTMs, bioinformatic analysis together with molecular and biochemical approaches, including gene knockdown, dot blot and proximity ligation assay have been integrated in this study.
**Results and Discussions**
Our alignment analysis showed that there is a short sequence in the middle region in PBP-βLs domain of LACTB which is highly conserved across eukaryotes. Interestingly, this middle region contains a long lysine-rich motif that has a high chance to undergo post-translational modifications. Here, the deletion of the middle region or mutation of all the lysine residues in this region of LACTB was found to decrease its tumor suppressive activity against breast cancers. Through immunoprecipitation and western blot analysis, we identified that the middle region is a major site of lysine acetylation in LACTB. This indicates that there is an association between acetylation and tumor suppressive function of LACTB. Our results also show that the lysine mutation in this region impaired the enzymatic activity and polymerization of LACTB. Our bioinformatic analysis showed several potential proteins that might be involved in the regulation of acetylation of LACTB have been identified.
**Conclusion**
Overall, these studies uncover significant aspects in our understanding of the roles of PTMs in LACTB for its regulation of tumor suppression.

### EACR2024-0279
### Repurposing of FDA-approved CNS-accumulating drugs as autophagy modulators for potential treatment against glioblastoma

S. Dey[1], R. Chowdhury[1], S. Chowdhury[1]
[1]*Birla Institute of Technology and Sciences- Pilani, Biological Sciences, Jhunjhunu, India*

**Introduction**
The standard treatment for glioblastoma multiforme involves surgery followed by temozolomide (TMZ) treatment. However, many patients develop TMZ resistance either due to autophagic clearance of unwanted materials or reversal of TMZ-induced DNA alterations by methyl guanine o-methyl transferase. Additionally, other drugs are often ineffective because they cannot cross blood-brain barrier (BBB). Hence, our objective is to repurpose FDA-approved drugs capable of crossing BBB and regulating autophagic pathways to improve quality of life for GBM patients.
**Material and Methods**
Herein, we identified differentially expressed genes regulating cellular processes in GBM. We used Connectivity-MAP (C-Map) database to identify potential drugs, capable of reversing differentially expressed autophagy-associated genes. Following in-silico screening, we conducted cytotoxicity assays with selected drugs to evaluate IC50 values of drugs. We also evaluated the effects of drugs on 3-D spheroids. Additionally, immunoblotting and immunofluorescence were performed to examine protein expression. Furthermore, we employed MDC staining to identify autophagic vacuoles, Annexin-PI to quantify apoptotic cells, and flow cytometric and colorimetric assays to analyse the amount of ROS generation post-treatment.
**Results and Discussions**
Analysing GEO dataset, we identified top 10 dysregulated genes. Most downregulated genes were linked to calcium signalling, while upregulated ones were tied to PI3-Akt pathway. After identifying key genes and associated pathways, we used C-Map to identify the top 5 candidate drugs, e.g., Vorinostat, Valproic Acid, Doxylamine, Gemfibrozil, and Diprophylline. These drugs, with high connectivity scores and BBB penetrance, were assessed in-vitro for their potential against GBM. Among them, Doxylamine and Gemfibrozil exhibited the best cytotoxicity, surpassing the standard drug TMZ. These drugs also hindered autophagic flux, verified by elevated levels of p62 and LC3B II proteins. We also checked if inhibition of clearance mechanism and increased stress conditions induce UPR effect. Overexpression of PERK and CHOP confirmed ER stress-induced apoptosis. AnnexinV-PI assay demonstrated that increased ER stress and

© 2024 The Authors. Molecular Oncology is published by John Wiley & Sons Ltd on behalf of Federation of European Biochemical Societies subsequent restriction of autophagic flux led to cell death, further supporting correlation between these pathways.

**Conclusion**

Our research highlighted that Doxylamine and Gemfibrozil could serve as potential alternatives to TMZ for inducing apoptosis, by inhibiting autophagy, inducing ER stress, and generating ROS.

### EACR2024-0285

### Multiparameter cell-cycle measurement enables a better assessment of cancer aggressiveness in urothelial bladder cancer than Ki67 LI alone

*E. Bady[1], H. Plage[2], S. Elezkurtaj[2], A.H. Marx[3], M. Fisch[4], M. Slojewski[5], S. Hallmann[6], H. Zecha[7], T. Schlomm[2], J.H. Müller[1]*

*[1]University Medical Center Hamburg-Eppendorf, Institute of Pathology, Hamburg, Germany*

*[2]Charité Berlin, Department of Urology, Berlin, Germany*

*[3]Academic Hospital Fuerth, Department of Pathology, Fuerth, Germany*

*[4]University Medical Center Hamburg-Eppendorf, Department of Urology, Hamburg, Germany*

*[5]University Hospital Stettin, Department of Urology, Stettin, Poland*

*[6]Helios Hospital Bad Saarow, Department of Urology, Bad Saarow, Germany*

*[7]Albertinen Hospital, Department of Urology, Hamburg, Germany*

**Introduction**

Ki67 is expressed in the G1, S, G2 and M phase of the cell cycle. Immunohistochemical determination of the Ki67 labeling index (LI) is a clinically well-established tool for assessing the proliferative activity of tumors and critical clinical decisions are based on this parameter. Other cell cycle associated proteins have been evaluated for clinical utility much less intensively. This for example includes minichromosome maintenance-3 (MCM3) which is expressed earlier in the cell cycle than Ki67.

**Material and Methods**

To evaluate the difference between Ki67 and MCM3 based proliferation indices, both proteins were analyzed by multiplex fluorescent immunohistochemistry in more than 1,994 urothelial bladder cancers that were available in a tissue microarray format and results were compared with clinico-pathological parameters (pT, pN, grade, CK20, p16 and p63). A deep-learning based algorithm for automated cell and marker detection was used for image analysis.

**Results and Discussions**

In our 1,994 evaluable urothelial bladder. cancers, the average Ki67 LI was 16% and the average MCM3 LI was 39%. There was a significant overlap between Ki67 and MCM3 staining: Of all panCK positive tumor cells, 14% were positive for both Ki67 and MCM3, 20% were only positive for MCM3, 0.002% showed positivity only for Ki67, and 67% were negative for both markers. According to the expression of Ki67 and MCM3, the following compartments of proliferating cells were defined: early ($MCM3^+/Ki67^-$), intermediate ($MCM3^+/Ki67^+$), late phase of the cell cycle ($MCM3^-/Ki67^+$) as well as full proliferation ($MCM3^+$ or $Ki67^+$). Comparison with clinico-pathological parameters revealed the best F-ratio and p-values for the MCM3 LI in the comparison with pT (F=93.34, p<0.0001), pN (F=1.25, p<0.0001), grade (F=460.38, p<0.0001) as well as p16 expression (F=107.54, p<0.0001) and a high p63 expression (F=56.76, p<0.0001) of a previous brightfield study on urothelial bladder cancer. While the "full proliferation" LI was most tightly linked to a high CK20 expression (F=28.42, p<0.0001).

**Conclusion**

The combined analysis of MCM3 and Ki67 enables the distinction of cells in early, intermediate, and late phase of the cell cycle. In urothelial bladder cancer MCM3 quantification alone or in combination with Ki67 often resulted in stronger relationships with clinicopathological parameters than the Ki67 LI alone. These results suggest that a more subtle analysis of cell cycle proteins might enable a better evaluation of cancer aggressiveness than Ki67 measurement alone.

### EACR2024-0292

### Proline dehydrogenase affects lung adenocarcinoma cell survival, proliferation, and 3D growth

*E. Berno[1], C. Priscilla[1], R. Cinquetti[1], P. Campomenosi[1]*

*[1]University of Insubria, Department of Biotechnology and Life Sciences, Varese, Italy*

**Introduction**

Proline dehydrogenase (PRODH) is a mitochondrial flavoenzyme that catalyzes the oxidation of proline, generating two electrons that can be used to produce ATP or ROS. The latter can promote apoptosis, cellular senescence, and autophagy. We previously showed that PRODH is expressed in most lung adenocarcinomas (LUAD) at stages I-II, but not at higher stages, or in the other subtypes. We demonstrated that PRODH promotes cellular senescence in LUAD cell lines via ROS production, causing a reduction in proliferation and survival. This work aims to further analyze the role of PRODH in lung cancer development by investigating its effects on 3D growth and migration. We also aim to assess whether the growth retardation and the impaired cell survival we observed are due to the catalytic activity of PRODH.

**Material and Methods**

We used NCI-H1299 LUAD cell clones stably transfected with a PRODH expression construct or with empty vector (as control). To assess how PRODH expression affects 3D growth, we compared the size, morphology, and number of cells of spheroids obtained from PRODH-expressing or control clones. On the same clones, we compared the migration ability by wound healing assay. We also carried out survival and proliferation experiments with NCI-H1299 cell clones expressing a PRODH variant with reduced catalytic activity (p.L441P).

**Results and Discussions**

The spheroids of PRODH-expressing clones are smaller, contain a lower number of cells than those of controls and appear visibly less compact and flatter.

© 2024 The Authors. Molecular Oncology is published by John Wiley & Sons Ltd on behalf of Federation of European Biochemical Societies

Wound healing assay did not show significant differences in the migration of cells from PRODH-expressing and control clones. We will confirm these preliminary results by transwell assays. In clonogenic assay, we observed that the reduced catalytic activity of PRODH-L441P is sufficient to decrease cell survival comparably to the wild-type enzyme. By growth curve analysis, we observed that clones expressing the mutated protein have an intermediate proliferation between controls and clones expressing the wild-type protein.

**Conclusion**

This work has helped to better clarify the role of PRODH in LUAD. We have shown that catalytic activity of the enzyme affects proliferation more than cell survival in the tested conditions. We have also observed that PRODH expression affects the 3D growth of cells at both size and morphological levels. Our next studies will better define PRODH role in cell migration by transwell assays, analysis of epithelial-to-mesenchymal transition and stemness markers.

### EACR2024-0308
### Peritoneal mesothelial cells and fibroblasts display premature senescence and procancerogenic phenotype upon exposure to carboplatin and paclitaxel

*S. Rutecki[1], A. Leśniewska-Bocianowska[1], J. Matuszewska[1], J. Mikuła-Pietrasik[1], K. Książek[1]*
*[1]Poznan University of Medical Sciences, Department of Pathopysiology of Ageing and Civilization Diseases, Poznan, Poland*

**Introduction**

Carboplatin (CPT) and paclitaxel (PCT) are the optimal non-surgical treatment of epithelial ovarian cancer (EOC). Although their impact on EOC cells is well known, their influence on normal peritoneal cells, including mesothelium (PMCs) and fibroblasts (PFBs), is elusive. Here, we verified if CPT and PCT may induce senescence in PMCs and PFBs, and whether this effect may translate to the development of their pro-cancerogenic phenotype.

**Material and Methods**

Experiments were made on primary, omental PMCs and PFBs obtained from non-oncologic patients, established (A2780, SKOV-3, OVCAR-3), and primary ovarian cancer cells. Senescence of PMCs and PFBs was induced by their exposure to 50 μM CPT and 25 nM PCT (PMCs) and 25 μM CPT and 10 nM PCT (PFBs). Several markers associated with proliferation (BrdU, cell-cycle distribution), senescence (SA-β-Gal, γ-H2A.X, 53BP1, telomeres, p16, p21, p53, SASP), oxidative stress (ROS, 8-OH-dG, CAT, SOD, carbonylated proteins), and signaling pathways (AKT, ERK1/2, p38 MAPK, JNK, AP-1, NF-κB, and STAT3) were tested. Moreover, cancer cell progression (adhesion, proliferation, migration, invasion) and their transcriptome were analyzed upon exposure to senescent PMCs and PFBs secretome.

**Results and Discussions**

Results showed that CPT and PCT induce senescence of normal peritoneal cells and that the most potent effect occurs when the drugs are used together. PMCs senesce in a telomere-independent fashion along with the induction of p16. In drug-treated PFBs, telomeres shorten, which coincides with p21 and p53 induction. Oxidative stress participates in the CPT+PCT-dependent senescence of PMCs and PFBs, and its inhibition prevents this effect, especially in PFBs. Drug-induced senescence of PMCs proceeds through AKT and STAT3 signaling, whereas PFBs senesce via ERK1/2. PMCs and PFBs subjected to PCT+PCT support the proliferation, migration, and invasion of all EOC cell lines tested and modify their transcriptome (VEGF and TGF-β1 over-expression). This activity is linked with the formation of SASP, controlled by p38 MAPK, NF-kB, STAT3, Notch1, and JAK3.

**Conclusion**

Our study shows that CPT combined with PCT promote senescence of normal peritoneal cells, causing the possibility of the development of a pro-cancerogenic activity of these cells.

*The study was supported by a grant from the National Science Centre, Poland (2020/37/B/NZ5/00100).*

### EACR2024-0310
### Exploiting metabolic adaptations in mitochondrial reprogramming of prostate cancer

*V. Baumgartner[1], S. Salemi[1], D. Eberli[1]*
*[1]University Hospital Zurich, Urology, Schlieren, Switzerland*

**Introduction**

Drug treatment options in prostate cancer (PCa) are largely limited by the appearance of resistance due to the quick metabolic flexibility of malignant cells to adapt to stressors. We hypothesize that altered mitochondrial dynamics is a survival mechanism that ensures sustained energy production and tumour growth in PCa. Here we examined how targeting the mitochondria with a potent complex I inhibitor (IACS-010759) in combination with the androgen receptor blocker apalutamide (ARN-509) affects mitochondrial morphology and cytotoxicity in PCa cells.

**Material and Methods**

Human PCa cells (PNT1A, LNCaP, C4-2, PC-3) were treated for 3 days with ARN-509, IACS-010759 and a combination. We investigated alterations in mitochondrial fission and fusion upon treatment by Western blot and transmission electron microscopy (TEM). Mitochondrial respiration was measured by Seahorse assay. Cell death was assessed by Annexin V and mitochondrial ROS production was quantified by MitoSOX staining.

**Results and Discussions**

Androgen-sensitive LNCaP and C4-2 exhibited strong reductions in fission protein p-DRP1 (Ser616) and increased fusion protein OPA1 after treatment with ARN-509 and/or IACS-010759. TEM morphological analysis revealed significantly increased mitochondrial area and length in treated LNCaP samples compared to control. Conversely, androgen supplementation led to increased mitochondrial respiration and fission in LNCaP cells, suggesting that androgen dependency is linked to mitochondrial respiration. Moreover, treatment increased cell death exclusively in androgen-sensitive cell lines but

© 2024 The Authors. Molecular Oncology is published by John Wiley & Sons Ltd on behalf of Federation of European Biochemical Societies not in benign PNT1A and metastatic PC-3 cells. Similarly, mitochondrial ROS was strongly upregulated in androgen-sensitive cell lines.

**Conclusion**

Our study reveals that targeting mitochondrial dynamics through a combination of IACS-010759 and apalutamide induces mitochondrial elongation, enhances oxidative stress and cell death specifically in androgen-sensitive prostate cancer cells. This approach shows promise in overcoming drug resistance and improving treatment outcomes in prostate cancer.

### EACR2024-0313

### Targeting senescence through autophagy inhibition: in vitro evidence for a novel one-two punch approach for the treatment of anaplastic thyroid cancer

*B. Mazzoleni[1], M. Mazzoni[1], A. Greco[1]*

*[1]Fondazione IRCCS Istituto Nazionale dei Tumori, Experimental Oncology Department, Milan, Italy*

**Introduction**

Anaplastic thyroid cancer (ATC) is among the deadliest solid tumors, often inoperable and completely resistant to conventional treatments. A potential new therapeutic approach is CDK4/6 inhibition, a strategy already used in other aggressive cancers that relies on the induction of senescence. Unfortunately, in the context of anti-cancer therapy, senescence acts as a double-edged sword. In facts, while therapy-induced senescent (TIS) cells are unable to replicate, they occasionaly escape the arrested status, and cause tumor progression. Moreover, TIS cells secrete several factors, through which they can drive the growth and migration of other cancer cells, and shift the surrounding microenvironment toward a pro-tumoral direction. For these reasons, the selective targeting of TIS cells is a valid strategy to avoid their detrimental effects. While it is known that CDK4/6 inhibition increases autophagy levels, whether targeting this process in TIS cells could modify or eliminate them is still uncertain.

**Material and Methods**

To study the effects of autophagy inhibition on CDK4/6 inhibitor-treated cells, we used different human ATC cell lines, with diverse genetic backgrounds. Dose-response curves were performed to identify the most suitable concentration of CDK4/6 inhibitor Palbociclib. Senescence and autophagy were analyzed through β-gal assay, western blot (p21, p53, HMGA2, LC3B, p62), immunofluorescence (γH2AX, p62), and lysosomal staining (LysoTracker). Autophagy inhibition was performed using either Chloroquine or LY294002. The effects of this perturbation were analyzed by cell viability and colony forming assays.

**Results and Discussions**

The analysis showed that Palbociclib induced both senescence and autophagy in ATC cell lines, but the functionality of the autophagic flux was cell-line specific (as indicated by p62 protein levels). The colony forming assay showed that a fraction of Palbociclib-treated cells was capable of escaping senescence upon drug removal, and this ability was significantly reduced by LY294002 treatment. More interestingly, Chloroquine completely abolished TIS cells' ability to form colonies. When cell viability was assessed, TIS cells treated with either inhibitor showed only a small viability reduction, suggesting that the impairment in colony formation wasn't mainly caused by senolysis.

**Conclusion**

Our results suggest that the use of Palbociclib followed by autophagy inhibition may represent a valid approach to prolong and improve the effects of CDK4/6 inhibition in ATC cell lines.

### EACR2024-0318

### An easy-to-use and reliable method of ovarian cancer cell dormancy induction and termination in vitro using serum starvation

*A. Krawiec[1], A. Leśniewska-Bocianowska[1], K. Chmielewska[1], J. Matuszewska[1], S. Rutecki[1], J. Mikuła-Pietrasik[1], K. Książek[1]*

*[1]Poznań University of Medical Sciences, Department of Pathophysiology of Ageing and Civilization Diseases, Poznań, Poland*

**Introduction**

Epithelial ovarian cancer (EOC) is one of the most common and lethal gynecological cancers. The greatest challenge in treating patients with EOC is the high rate of disease relapse. The causes of the relapse are poorly understood, and two hypotheses of its development are considered: i) the presence and activity of cancer stem cells and ii) the awakening of so-called dormant cells. The existence of dormant cancer cells and their involvement in EOC relapse is highly probable; however, research in this area is significantly hampered by the limited number of convincing and reproducible models of this phenomenon. Therefore, this study aimed to establish an easy-to-use and reliable model of EOC dormancy induction and termination.

**Material and Methods**

The study was performed on primary EOC cells and three established EOC cell lines (A2780, OVCAR-3, SKOV-3). To induce dormancy, cells were incubated with a serum-free medium (SFM) for 72 h. Termination of dormancy was achieved by 72-h exposure to a growth medium with 5% exosome-free fetal bovine serum (FBS). Reversible cell growth inhibition was detected by assessing Ki67 protein, PKH26 fluorescence, and cell cycle distribution (flow cytometry). Cells were also monitored for ERK1/2/p38 MAPK activity ratio, apoptosis (JC-1 fluorescence, subG1 fraction), and senescence (SA-β-Gal expression).

**Results and Discussions**

Experiments showed that 72-hour incubation in SFM caused cell cycle arrest at the G0/G1 stage in a significant fraction of cells, with no signs of apoptosis or senescence. Simultaneously, Ki67 expression and ERK1/2/p38 MAPK activity ratio decreased, while PKH26 fluorescence remained unchanged, confirming the lack of cell replication. The dormancy state was effectively terminated by SFM withdrawal and its replacement with 5% FBS. In this case, cells regained Ki67 expression which was accompanied by an increased fraction of DNA replicating cells in the S phase of the cell cycle, increased ERK1/2/p38 MAPK activity ratio,

© 2024 The Authors. Molecular Oncology is published by John Wiley & Sons Ltd on behalf of Federation of European Biochemical Societies and declined PKH26 fluorescence. These results were highly reproducible, as they were obtained uniformly for all cancer cell lines tested.

**Conclusion**

To sum up, serum starvation appeared to be a convenient and effective way of modeling EOC cells' dormancy induction and termination, which can be successfully used in experimental research of the disease recurrence.

*The study was supported by a grant from the National Science Centre, Poland (2020/37/B/NZ5/00100).*

### EACR2024-0329
### Glucocorticoid receptor activity influences breast cancer progression through altered migration dynamics

*S. Pósa[1], É. Saskői[1], L. Bársony[1], L. Pongor[2], F. Fekete[3], E. Szőcs[3], A. Patócs[1], H. Butz[1]*

*[1]National Institute of Oncology, Department of Molecular Genetics and the National Tumor Biology Laboratory, Budapest, Hungary*

*[2]Hungarian Centre of Excellence for Molecular Medicine HCEMM, Cancer Genomics and Epigenetics Core Group, Szeged, Hungary*

*[3]National Institute of Oncology, Department of Oncology Biobank, Budapest, Hungary*

**Introduction**

Glucocorticoid receptor (GR) has a strong context-dependent role in tumorigenesis. In our previous work, we assessed the role of GRα and β isoforms in breast cancer due to their opposite function. As GR agonist dexamethasone (dex) is routinely administered as adjuvant therapy along with chemotherapy novel functions may be revealed. Therefore, we investigated the effect of GR transactivation on breast cancer cells.

**Material and Methods**

We treated 4 oestrogen receptor-positive (ER+) and triple negative (TN) breast cancer cell lines with dex and mifepristone (mif, GR antagonist) in monolayer (2D) and spheroid (3D) cultures. We investigated cell viability and proliferation using Alamar and Trypan Blue assays. Migration was assessed by single-cell tracking and classic wound healing test. Transcriptome sequencing was done to analyze the dex effect on ER+ and TN breast cancer cells. We used an independent in silico dataset of 1085 breast cancer and 291 normal tissue samples to cross-validate our findings and to identify the potential clinical impact.

**Results and Discussions**

GR activation increased cell migration in TN but not in ER+ tumour cells. Additionally, time-lapse cell tracking indicated an altered time-dependent migration dynamics upon dex effect in TN breast cancer cells. Cell proliferation was increased in TN and decreased in ER+ cells upon GR activation in both 2D and 3D cultures. Dex effect was eliminated by mif treatment on cell proliferation, but not on cell migration. RNA sequencing also showed that dex had a significant impact on cell migration in TN cells while it inhibited cell proliferation in ER+ breast cancer cells. Expression of members of GR pathway significantly correlated with cell migratory signature ($R=0.79$; $p<0.01$) in human breast cancer specimens. In addition, this pattern differentiated cancer samples from normal tissues in principal component analysis and GR-regulated genes (*RASA1, TFAP2A, TJP2, OCLN, CCND3, COL17A1*) had a prognostic role regarding overall survival.

**Conclusion**

GR activity was strongly dependent on the presence of ER. Dex treatment altered migration dynamics and increased cell motility that was mediated by GR trans-activation. This was in line with the survival data on human samples indicating the role of GR activation in TN breast cancer progression. This effect may have a direct implication for adjuvant therapy of patients with TN breast cancer.

### EACR2024-0334
### In vitro study of the chemosensitizing activity of manuka honey in colonsphere enriched with cancer stem-like cells

*Y. Armas Diaz[1], D. Cianciosi[1], T. Forbes-Hernandez[2], F. Giampieri[1]*

*[1]Polytechnic University of Marche, Clinical Science, Ancona, Italy*

*[2]University of Granada, Physiology, Granada, Spain*

**Introduction**

Colon cancer is the 3rd most common cancer in the world. The aim of the present study was to evaluate the effect of Manuka honey and its combination with 5-fluoracil (5-Fu) on cancer stem-like cells derived from a colon adenocarcinoma cell line and cultured in a 3D model.

**Material and Methods**

The effects of honey alone and in combination with 5-Fu on stem cell-enriched colonspheres were evaluated by measuring apoptosis, ROS production, chemoresistance, chemosensitivity, migration capacity, self-renewal, stemness traits and telomere length.

**Results and Discussions**

Manuka honey alone and to a greater extent with 5-Fu was able to: decrease some morphological and physical parameters of the colonospheres, increase the levels of ROS and apoptosis, reduce chemoresistance, the ability to migrate and self-renewal, telomere length and increase chemosensitivity, modulating several genes involved in these processes, such as IGFs, HSPs, ABCG2, Wnt/β-catenin, Hedgehog, Notch, Snail and Twist.

**Conclusion**

These results indicate that Manuka honey has an in vitro chemosensitizing effect towards 5-Fu on colon stem-like cells and could be useful in furthering studies on natural compounds to be used in combination with conventional medical therapies.

### EACR2024-0336
### Investigating the role of PTX3 in the biology of glioblastoma

*C. Tavani[1], E. Somenza[1], S. Filiberti[1], F. Pagani[2], P.L. Poliani[3], C. Boccaccio[4], F. Orzan[4], F. De Bacco[4], C. Romani[5], R. Ronca[1]*

*[1]University of Brescia, Molecular and Translational Medicine, Brescia, Italy*

*[2]University Medical Center Hamburg-Eppendorf, Laboratory for Translational Research, Hamburg, Germany*

*[3]Vita-Salute San Raffaele University, Pathology Unit,*

© 2024 The Authors. Molecular Oncology is published by John Wiley & Sons Ltd on behalf of Federation of European Biochemical Societies

*Milano, Italy*
[4]*Candiolo Cancer Institute, Cancer Stem Cell Research, Candiolo, Italy*
[5]*ASST Spedali Civili di Brescia, Angelo Nocivelli Institute of Molecular Medicine, Brescia, Italy*

**Introduction**

Glioblastoma Multiforme (GBM) is the most common and aggressive brain tumor in adults, classified as grade IV tumor by WHO. Common therapies include surgical removal, chemotherapy and radiotherapy; however, relapses are inevitable. In addition, it is hypothesized that the relapses are mainly due to a subpopulation of stem cells with self-renewal properties, called glioblastoma stem cells (GSC) localized in specialized niches. These cells are resistant to conventional treatments thanks to their ability to escape apoptosis and activate DNA repair mechanisms. Pentraxin 3 (PTX3) is a soluble pattern recognition receptor belonging to the humoral arm of the innate immunity that is also involved in several aspects of tumor growth, angiogenesis, metastasis and cancer immune-regulation. To date, a correlation between PTX3 and tumor aggressiveness in GBM has been described, but studies regarding its possible implication in GSC stemness are still missing.

**Material and Methods**

We used human GSC BT302 cells, derived from glioblastoma specimens diagnosed according to WHO criteria, to obtain a PTX3 silenced cells. PTX3 presence and production was assessed by Western blot, qPCR, ELISA and immunostainings. Proliferation, invasion and angiogenic assays were performed to analyse the effects of PTX3 silencing.

**Results and Discussions**

Preliminary observations revealed a wide expression of PTX3 in GSC, and specific silencing in a GSC line revealed a significant reduction of cell growth, invasiveness and angiogenic capacity in GSC after PTX3 knock-down that results in a decreased tumorigenic capacity in vivo.

**Conclusion**

Our data suggest that PTX3 is expressed and may play a relevant role in GSC cells. In this context, PTX3 silencing may impair tumor features in vitro and in vivo. This set the basis for further characterization of the pro-tumoral and pro-stemness role of PTX3 in glioblastoma.

### EACR2024-0340

### Botanical extract BEP1 suppresses stemness and angiogenesis of triple negative breast cancer cells

Y.C. Huang[1], S.J. Huang[2], L.H. Wang[3]
[1]*Graduate Institute of Biomedical Sciences, China Medical University, Taichung, Taiwan*
[2]*Chinese Medicine Research Center, China Medical University, Taichung, Taiwan*
[3]*Institute of Integrated Medicine, China Medical University, Taichung, Taiwan*

**Introduction**

Breast cancer is the most common cancer affecting women, with triple-negative breast cancer (TNBC) accounting for approximately 15% of cases, characterized by high rates of distant metastasis and relatively poor survival. Cancer stem cells (CSCs) and angiogenesis are believed to play crucial roles in cancer recurrence, metastasis and drug resistance. Therefore, targeting CSCs and angiogenesis may hold promise as a strategy to inhibit cancer recurrence.

**Material and Methods**

In collaboration with a biotechnology company, this study aims to develop a long-term dietary supplement as a remedy to prevent post-treatment recurrence and metastasis in cancer patients using a non-toxic botanical extract, which we termed BEP1. Initially, we enriched cancer stem cells (CSCs) in human /mouse TNBC Hs578T and 4T1 cells using a serum-free 3-D spheroid culture method. Then, we investigated BEP1's inhibitory effects on CSCs and angiogenesis through MTS, sphere formation assays, Western blot assays, and HUVECs tube formation assays. Additionally, we utilized orthotopic breast cancer mouse models and post-surgery tumor recurrence models to study BEP1's impact on TNBC tumor progression and metastasis.

**Results and Discussions**

Firstly, we demonstrated increased expression of CSC markers and enhanced sphere and colony-forming capabilities in Hs578T and 4T1 spheres. Subsequently, we found that the IC50's of BEP1 for inhibiting sphere formation (stemness) was 37-150 times lower than those for inhibiting monolayer cell growth (cytotoxicity) in three TNBC cell lines. Therefore, BEP1 possesses the ability to inhibit CSC growth, with much lower IC50 values reflecting their lack of significant general toxicity as reflected in the monolayer growth. BEP1 could suppress the expression of CSC markers, including CD133, ALDH1A1, CD44, Oct-4, and EpCAM in Hs578T and Bt549 sphere cells. Furthermore, we observed that BEP1 inhibited the VEGFR/PI3K/AKT/mTOR and Wnt/β-catenin signaling pathways, as well as strongly suppressed angiogenesis as shown in HUVECs tube formation assays. In a 4T1 TNBC mouse model, BEP1 significantly inhibited tumor metastasis and recurrence. BEP1 could modulate serum cytokines in mice and markedly reduce the expression of cytokines promoting angiogenesis and tumor metastasis.

**Conclusion**

Our study demonstrates that BEP1 has the ability to inhibit cancer stem cell growth and angiogenesis without general cytotoxicity likely through inhibiting the Wnt / β-catenin and VEGFR/PI3K/AKT/mTOR pathways.

### EACR2024-0345

### A neosis-like de-differentiation of spontaneously senescent ovarian cancer cells as a plausible cause of the disease recurrence

J. Matuszewska[1], J. Mikuła-Pietrasik[1], K. Książek[1]
[1]*Poznan University of Medical Sciences, Department of Pathophysiology of Ageing and Civilization Diseases, Poznan, Poland*

**Introduction**

Cancer cells undergo senescence upon radiation or chemotherapy. The knowledge about the senescence of epithelial ovarian cancer (EOC) cells is minimal. Here, we analyzed mechanisms of primary EOC senescence, with particular attention to its role in cancer progression.

© 2024 The Authors. Molecular Oncology is published by John Wiley & Sons Ltd on behalf of Federation of European Biochemical Societies

**Material and Methods**
Experiments were made using primary EOC cells, established from chemotherapy-naïve patients. The senescence was induced by serial passaging. The identification of senescent cells was based on the activity of senescence-associated β-galactosidase (SA-β-Gal), the presence of DNA damage response elements (γ-H2A.X, 53BP1), telomere length, expression of cell-cycle inhibitors (p16, p21, p53), cell-cycle distribution, and proliferation markers (Ki67, BrdU).
**Results and Discussions**
Tumors obtained from EOC patients display a significant fraction of senescent cells, which display limited capacity to divide in vitro. After just a few divisions, they become enlarged and display all classic signs of senescence, like G0/G1 growth arrest, SA-β-Gal activity, the presence of DNA damage foci, and up-regulated p16, p21, and p53 proteins. Telomeres in senescent cancer cells were shortened, which was accompanied by decreased telomerase activity. Significantly, we observed that in about 10% of cultures investigated, the senescent populations contained a sub-population of polyploid giant (PGCCs) whose surface area and nuclei number were remarkably higher than remaining senescent cells. Nonetheless, they displayed SA-β-Gal(+)/γ-H2A.X(+)/Ki67(-) phenotype. When cultures with PGCCs were left for several weeks, those cells appeared to generate very small, rapidly proliferating progeny. Those daughter cells were released from the mother PGCCs by a neosis-like mechanism and had no molecular markers of senescence. Instead, they were Ki67-positive and capable of forming spheroids.
**Conclusion**
Our results indicate that EOC cells undergo senescence, similarly to normal somatic cells. However, some senescent cancer cells permanently ceased their proliferation, whereas some (PGCCs) appear to be the source of a population of vigorously proliferating cancer cells. Our observation may indicate that senescent ovarian cancer cells may plausibly contribute to cancer expansion and disease renewal. Further tests are required to understand the molecular nature of PGCCs and their progeny.
*The study was supported by a grant from the National Science Centre, Poland (2020/37/B/NZ5/00100).*

### EACR2024-0348
### Autophagy plays a suppressive role in hyperglycemia-related colorectal cancer tumorigenesis and drug resistance
H.S. Liu[1]
[1]*Kaohsiung Medical University, M. Sc. Program in Tropical Medicine- College of Medicine, Kaohsiung, Taiwan*
**Introduction**
Hyperglycemia-related recurrence of colorectal cancer (CRC), low survival rate and chemoresistance remain unsolved problems, which are affected by epigenetic regulation. Deficient autophagy of β cells leads to obesity and hyperglycemia. The mice with β cell specific Atg7 knockout showed abnormal insulin secretion accompanied by hypoinsulinemia and hyperglycemia. We have reported that autophagy promotes insulin secretion of β cells and alleviates hyperglycemia symptoms both in vitro and in vivo (Autophagy, 2022). We also reported that degradative autophagy inhibits CRC tumorigenesis. Therefore, we hypothesize that degradative autophagy suppresses local CRC tumorigenesis both in vitro and in vivo.
**Material and Methods**
HCT116 and SW480 CRC cells were used for immunoblotting, immunofluorescent staining, lentiviral shRNA, CRISP-Cas9 system, MTT, focus formation, wound healing, and Transwell$^{TM}$ assays. Clinical CRC specimens were analyzed by tissue microarray (IR) and TCGA database. Nude mice were used for xenograft CRC cell tumor formation after feeding a high-fat diet (HFD).
**Results and Discussions**
We found that high glucose (25 mM) decreases autophagy activity and increases proliferation, colony formation, migration and drug resistance of CRC cells. Induction of autophagy activity by inducers suppresses proliferation, colony formation, and decreases drug resistance of CRC cells under high glucose conditions. Knockdown or knockout of autophagy-related gene *Atg5* reverses the effects of autophagy on the tumorigenesis and drug resistance of CRC cells under high glucose conditions. Autophagy marker protein LC3B was detected at low level in the tumor parts of CRC patient specimens Consistently, LC3B mRNA was expressed at low level as well in CRC tumors by TCGA big database analysis. Our high fat diet xenograft mouse model confirmed that HFD increased tumor formation, and autophagy inducer "rapamycin" increased autophagy activity accompanied by promotion of the level of insulin in the blood and reduction of tumor formation of CRC cells under hyperglycemic conditions.
**Conclusion**
Our findings reveal that autophagy activity of CRC cells was suppressed under high glucose conditions. Notably, further induction of autophagy by autophagy inducers confirms that autophagy plays a suppressive role in CRC tumorigenesis and drug sensitivity. Our findings shed lights on the development novel autophagy inducers to treat high glucose-related metabolic syndromes and cancers.

### EACR2024-0356
### TXNIP inhibits glucose uptake and migration in high-grade serous ovarian cancer cells through GLUT1
Z. Yang[1], A. Nasimian[2], J.U. Kazi[2], I. Hedenfalk[1]
[1]*Lund University, Department of Clinical Sciences Lund, Lund, Sweden*
[2]*Lund University, Department of Laboratory Medicine, Lund, Sweden*
**Introduction**
Deregulated glucose uptake is a hallmark of cancer metabolism. It is well known that thioredoxin-interacting protein (TXNIP) regulates glucose metabolism in various tissues and cells. TXNIP can induce glucose transporter 1 (GLUT1) internalization, decline enriched GLUT1 on the cell membrane, and inhibit glucose uptake in mouse embryonic fibroblasts. High-grade serous ovarian cancer (HGSOC) is the most common and lethal form of

© 2024 The Authors. Molecular Oncology is published by John Wiley & Sons Ltd on behalf of Federation of European Biochemical Societies epithelial ovarian cancer. Whether TXNIP affects HGSOC cell proliferation, migration, and glucose uptake by GLUT1 is unclear.

**Material and Methods**

HGSOC cell line was obtained from ATCC. Cell proliferation and migration were evaluated by CellTiter Glo 2.0 and scratch assay. Immunofluorescence was used to detect the location of GLUT1. Glucose uptake assay was performed using the Glucose Uptake-Glo™ Assay kit. Interaction between TXNIP and GLUT1 was explored using Western blotting, co-immunoprecipitation (co-IP), AlphaFold2, and the web tool PDBePISA.

**Results and Discussions**

Our results showed that inducing TXNIP expression significantly inhibited OVCAR3 cell proliferation and migration. Moreover, the upregulation of TXNIP inhibited GLUT1 expression. Immunofluorescence results showed that GLUT1 was almost depleted from the cell membrane after overexpression of TXNIP in OVCAR3 cells. Overexpression of TXNIP significantly inhibited glucose uptake and co-IP and reverse co-IP results suggested that TXNIP interacts with GLUT1. AlphaFold2 predicted that the C-domain of TXNIP binds to GLUT1. PDBePISA results showed the protein interface of the TXNIP-GLUT1 binding site for future design peptides. Taken together, our results suggested that TXNIP inhibits cell migration and glucose uptake through GLUT1 in HGSOC. We propose designing specific peptides based on the TXNIP-GLUT1 interaction site to explore the potential of regulating TXNIP levels in HGSOC for therapeutic utility.

**Conclusion**

These findings indicate that TXNIP overexpression can inhibit glucose uptake via downregulating GLUT1 in the cell membrane, thereby preventing the proliferation and migration of HGSOC cells.

### EACR2024-0362
### MiR-151a is involved in DNA damage response and exerts an oncogenic role in pancreatic cancer

*A. Muñoz Villanova[1], E. Fernandez-Castañer[1], E. Vila-Navarro[1], N. Navarro[1], C. Parra[1], A. Castells[1], M. Gironella[1]*

*[1]CIBERehd/Fundació Recerca Clinic Barcelona-IDIBAPS/Hospital Clínic de Barcelona, Gastrointestinal and Pancreatic Oncology, Barcelona, Spain*

**Introduction**

Pancreatic cancer is currently considered a huge health challenge and new therapeutic strategies are urgently needed. Previous studies from our group have shown that microRNAs (miRNA) are highly deregulated in this disease and that some of them could be good circulating biomarkers for early diagnosis. Understanding the specific consequences of miRNA deregulation in this type of cancer would help to elucidate new therapeutic targets. We have previously shown that miR-151a is up-regulated in pancreatic cancer. The main aim of this study is to decipher the role and the mechanisms of action of miR-151a in the pancreatic cancer context.

**Material and Methods**

MiR-151a expression was knocked-out (KO) in pancreatic cancer PANC-1 cells using CRISPR/Cas9 editing technique and stably overexpressed in normal pancreatic HPDE cells by retroviral transduction. Cell viability, colony formation, migration and invasion were assessed. Proteomic analysis was also performed using LC-MS in the different cell models to obtain a list of deregulated proteins and prioritize possible direct targets. The most relevant proteomic results were validated by Western Blot. Finally, DNA damage assessment by comet assay, flow cytometry and apoptosis analysis were performed in order to validate results from functional enrichment analysis.

**Results and Discussions**

PANC-1 KO-miR-151a cells showed significantly reduced viability, colony-forming ability, migration and invasion relative to control cells. HPDE cells over-expressing miR-151a showed a significant increase in viability, colony formation, migration and invasion, compared to corresponding control cells. Proteomic analysis of the above-mentioned cell models and functional enrichment analysis of significant altered genes revealed a consistent alteration in DNA damage repair mechanisms. Comet assay and apoptosis determinations showed a functional alteration in oxidative stress-induced DNA damage repair mechanisms. Cell cycle analysis demonstrated arrest at G2/M in PANC-1 KO-miR-151a. Validation of the proteomics results using Western Blot demonstrated a significant alteration in proteins involved in DNA damage response.

**Conclusion**

MiR-151a acts as an oncomiR in pancreatic cancer, at least in part by modulating DNA damage response. It could form the basis to develop new therapeutic strategies to treat pancreatic cancer.

### EACR2024-0365
### MED12 and CDK8/19 modulate the androgen receptor activity and enzalutamide resistance in prostate cancer

*C. Andolfi[1], C. Bartolini[1], E. Morales[1], M. Puhr[1], S. Wach[2], H. Taubert[2], A. Aigner[3], E. Iris E.[1], F. Handle[4], Z. Culig[1]*

*[1]Medical University of Innsbruck, Dept. of Urology-Division of Experimental Urology, Innsbruck, Austria*
*[2]Universitätsklinikum Erlangen, Dept. of Urology and Pediatric Urology, Erlangen, Germany*
*[3]University of Leipzig, Rudolf-Boehm-Institute for Pharmacology and Toxicology- Clinical Pharmacology, Leipzig, Germany*
*[4]Medical University of Innsbruck, Institute of Pathology-Neuropathology & Molecular Pathology, Innsbruck, Austria*

**Introduction**

The Mediator complex is a multi-subunit protein that regulates gene expression in a genome-wide manner. Its subunits MED12 and CDK8 (or the paralogue CDK19) compose its kinase module and have been related to prostate cancer (PCa). PCa is strongly driven by the transcriptional activity of the androgen receptor (AR), which is the target of the therapy drug enzalutamide. In this project, we aimed at exploring the role of MED12

© 2024 The Authors. Molecular Oncology is published by John Wiley & Sons Ltd on behalf of Federation of European Biochemical Societies and CDK8/19 in the modulation of the AR activity and resistance to enzalutamide.

**Material and Methods**

LNCaP, 22Rv1, and PC3 cells were chosen as our PCa in vitro models. Cells were transfected with MED12-targeting siRNA or treated with a CDK8/19 inhibitor (SEL 120-34A). To evaluate the inhibitory effects on enzalutamide resistance, 22Rv1 cells were co-treated with 10µM enzalutamide. RNA-sequencing and pathway analysis were performed in cells upon MED12 knockdown. Cell proliferation was quantified through a fluorescent-based counting of nuclei in both 2D and 3D (spheroid) cell models. AR activity was quantified by measuring the prostate-specific antigen (PSA) concentration in cell medium through an immunoassay. Gene and protein expression were quantified, respectively, through quantitative RT-PCR and Western Blot.

**Results and Discussions**

MED12 knockdown significantly decreased cell proliferation in all our 2D and 3D cell models. Concordantly, MED12 knockdown inhibited c-Myc pathway in all three cell lines and the androgen response in 22Rv1 cells. c-Myc protein expression was also reduced in 22Rv1 and PC3 cells. Both MED12 downregulation and CDK8/19 inhibition decreased PSA secretion in 22Rv1 cells, while only CDK8/19 inhibition significantly downregulated PSA secretion in LNCaP cells. In 22Rv1 cells, MED12 downregulation significantly decreased AR-V7 protein expression, a ligand-independent form of AR that promotes enzalutamide resistance. Co-treatment of 22Rv1 cells with MED12 knockdown and enzalutamide additively reduced PSA protein secretion and its mRNA expression in 22Rv1 and LNCaP cells. The dual treatment of enzalutamide and CDK8/19 inhibition significantly decreased 22Rv1 cell proliferation in an additive way.

**Conclusion**

MED12 and CDK8/19 downregulation decreased AR activity in our PCa models. The co-treatment with enzalutamide shows an additive effect on AR activity and cell proliferation. Inhibiting MED12 or CDK8/19 may therefore be a therapeutic approach to restore enzalutamide sensitivity.

### EACR2024-0371

### Role of lipoproteins in the modulation of prostate cancer cell cholesterol and proliferation

*F. Cetti[1], A. Ossoli[1], C. Garavaglia[1], M. Gomaraschi[1]*
*[1]University of Milano, Dept. of Pharmacological and Biomolecular Sciences- Center E. Grossi Paoletti, Milano, Italy*

**Introduction**

Cancer cells need cholesterol to proliferate, since it is a structural component of cell membranes and is involved in several cellular functions. Within lipid rafts it regulates the activation of transmembrane receptors, including those for growth factors. Moreover, cholesterol can be converted into biologically active metabolites, as oxysterols, isoprenoids, or hormones. Cholesterol can be directly synthesized by cancer cells, or it can be taken up by lipoproteins in the extracellular fluids. Aim of the study was to investigate cholesterol homeostasis in androgen receptor (AR)-positive and AR-null prostate cancer (PCa) cell lines and the modulation by lipoproteins.

**Material and Methods**

AR-positive LnCaP and AR-null PC3 cells were compared to the non-tumorigenic human prostatic epithelial cell line PNT2.

**Results and Discussions**

The rate-limiting enzyme of cholesterol synthesis, HMG-CoA reductase, was not increased in PCa cell lines and its inhibition by lovastatin did not reduce cell cholesterol content. On the contrary, receptors involved in lipoprotein endocytosis were significantly overexpressed in cancer cells. Consistently, the exposure to low-density lipoproteins (LDL) increased cholesterol content and proliferation of PCa cells. ATP-binding cassette transporters A1 and G1, which promote cholesterol efflux towards high-density lipoproteins (HDL), were dysregulated in cancer cells: ABCA1 expression was blunted while ABCG1 expression was upregulated in LnCaP and PC3 cells compared to PNT2. ABCA1 down-regulation was key to impair cholesterol efflux from cancer cells to HDL, since restoring its expression by proteasome inhibition promoted cholesterol removal and reduced the proliferation of PCa cells.

**Conclusion**

For their cholesterol needs PCa cell lines mainly rely on lipoprotein uptake rather than on synthesis, likely because the mevalonate pathway is a time- and energy-consuming process. Concomitantly, cholesterol efflux towards extracellular acceptors as HDL is impaired due to the accelerated catabolism of the ABCA1 transporter.

### EACR2024-0374

### The role of Toll-like receptor 3 and Damage-associated molecular patterns in Head and neck cancer stem cells

*T. Vasiljevic[1], M. Tarle[2], K. Vukovic Djerfi[1], E. Zapletal[1], D. Müller[3], T. Matijevic Glavan[1]*
*[1]Rudjer Boskovic Institute, Division of Molecular Medicine, Zagreb, Croatia*
*[2]Dubrava University Hospital, Department of Maxillofacial Surgery, Zagreb, Croatia*
*[3]Dubrava University Hospital, Department of Pathology and Cytology, Zagreb, Croatia*

**Introduction**

Toll-like receptor 3 (TLR3) belongs to the Toll-like receptor family, a group of pattern recognition receptors that identify pathogen-derived motifs. TLR3 responds to viral double-stranded RNA (dsRNA) or synthetic analogs like poly(I:C) and poly(A:U). It can also be activated by endogenous ligands known as damage-associated molecular patterns (DAMPs). While primarily involved in innate immunity, TLR3 also has implications in tumorigenesis. Its activation triggers apoptosis, yet it can also promote tumor progression. In this study, we try to establish the role of TLR3 in cancer stem cells (CSC), a subpopulation of tumor cells capable of initiating tumor growth. As a model, we are using head and neck squamous cell carcinomas (HNSCC).

**Material and Methods**

© 2024 The Authors. Molecular Oncology is published by John Wiley & Sons Ltd on behalf of Federation of European Biochemical Societies

Pharyngeal cancer cell line Detroit 562 was treated with poly(I:C) or poly(A:U) to stimulate TLR3. Tumor spheres were quantified based on number and size. Immunocytochemistry was performed on tumor spheres to detect CSC markers. CSC genes were analyzed using qPCR. HMGB1 secretion was analyzed by ELISA. DAMPs expression was analyzed by western blot. Immunohistochemistry was used to determine the expression of TLR3, DAMPs, and CSC markers in patients on different types of HNSCC tissue.

**Results and Discussions**

The quantity/size of tumor spheres were increased after poly(A:U) treatment. TLR3 activation increased CD133 and DAMPs expression in tumor spheres, including the increased S100A9 expression, a protein associated with poor prognosis and immunosuppression. HMGB1 and its receptor RAGE were increased in tumor spheres after the treatment with poly(A:U). HSP70, which can suppress apoptosis and support metastasis, showed increased expression in CSC after poly(I:C) treatment. CSC marker ALDH1A1 showed increased expression with the advancement of tumor sphere generations. Expression of *CD133* and *SOX* was significantly higher in tumor spheres after poly(A:U) treatment while poly(I:C) induced the expression of *OCT4* and *SNAIL*. The secretion of HMGB1 was upregulated after TLR3 activation. TLR3 was strongly expressed in HNSCC tissue, and its expression strongly colocalized with ALDH1, CD133, RAGE, and HSP70. Our results suggest TLR3 activation in CSC prompts DAMPs secretion into the microenvironment promoting tumor survival.

**Conclusion**

This study confirms that stimulation of TLR3 has an important role in promoting stemness and maintenance of CSCs in HNSCC. TLR3 activation on cancer cells and CSCs results in DAMPs production which contributes to stemness and cancer progression.

### EACR2024-0382

### New experimental approaches to deepen into the role of TGF-β in liver cancer

*<u>A. Cantos Cortés</u>[1], R. Espinosa-Sotelo[1], J. Amengual[1], E. Bertran[1], M. Ruiz de Galarreta[2], P. Molina-Sanchez[2], A. Lujambio[2], E. Gonzalez-Sanchez[3], I. Fabregat[1]*
*[1]Bellvitge Biomedical Research Institute IDIBELL - CIBERehd ISCIII, TGF-beta and Cancer Group – Oncobell Program, Barcelona, Spain*
*[2]Icahn School of Medicine at Mount Sinai, Department of Oncological Sciences, New York, United States*
*[3]University of Salamanca, Department of Physiology and Pharmacology, Salamanca, Spain*

**Introduction**

Hepatocellular carcinoma (HCC) has increased in incidence in recent years, showing a high mortality rate and moderate response to treatment. Transforming Growth Factor-beta (TGF-β) is emerging as a potential therapeutic target in cancer, particularly combined with immune checkpoint inhibitor therapies. However, it is a pleiotropic cytokine that could be exerting different functions, behaving as tumor suppressor or promoting factor, depending on the tumor cell and micro-environment. Molecular gene signatures reflecting the TGF-β oncogenic arm are being identified in HCC, but no efficient biomarkers of utility in the clinics have been proposed, except the circulating level of TGF-β1. Moreover, there are no evidence reflecting the dependency of TGF-β in stroma cells during cancer associated fibroblasts activation, inflammatory processes or immunosuppression. The aim of this work is developing new in vitro and in vivo models to better analyze the role of TGF-β in tumor cells and stroma, and the possibility that TGF-β inhibitors could be a therapeutic option in liver cancer.

**Material and Methods**

Characterization of new mouse model liver cell lines (AL1099 and AL1184), with different TGF-β gene signatures, isolated from tumors induced by hydrodynamic tail vein injection of two different transposon vector constructions. Analysis of the adequacy of these cell lines for in vitro and in vivo studies.

**Results and Discussions**

AL1099 and AL1184 showed different phenotype and response to TGF-β in terms of growth inhibition, apoptosis and Epithelial-Mesenchymal Transition. We have studied the effect of TGF-β when these cells are co-cultured in 3D together with other stromal cells, macro-phages and fibroblasts. AL1099 cells maintained the response to TGF-β in terms of tumor suppression both in absence and in presence of mouse fibroblast and macrophages. AL1184 cells increased cell proliferation in response to TGF-β when they are co-cultured in presence of mouse macrophages but not with mouse fibroblasts. Finally, we have set up a syngeneic model of liver tumorigenesis through orthotopic injection of AL1099 or AL1184 cells for testing pharmacological treatments. Both tumors types showed differences in the inflammatory tumor microenvironment as well as in the fibroblast arrangement.

**Conclusion**

These cells represent a valuable model to analyze whether TGF-β inhibitors, alone or in combination with kinase inhibitors or immunotherapy, could be a therapeutic option in HCC.

### EACR2024-0388

### Oncogene competition shapes molecular landscape and phenotype of cells with mutant p53, mutant KRAS, and overexpressed CMYC

*L. Dolinchuk[1], M. Grochowski[1], W. Kaźmierczak[2], T. Olesiński[2], <u>D. Walerych</u>[1]*
*[1]Mossakowski Medical Research Institute PAS, Laboratory of human diesease multiomics, Warsaw, Poland*
*[2]Maria Sklodowska-Curie National Research Institute of Oncology, Department of gastroenterology, Warsaw, Poland*

**Introduction**

The major driver oncogenes *CMYC*, mutant *KRAS*, and mutant TP53 often coexist and cooperate to promote human neoplasia. However, little research has been conducted on whether redundancy and competition among oncogenes affect their programs and ability to drive neoplasia.

© 2024 The Authors. Molecular Oncology is published by John Wiley & Sons Ltd on behalf of Federation of European Biochemical Societies

**Material and Methods**
Using viral vectors we introduced two distinct *TP53* missense hotspot mutants (R175H and R273H), mutant KRAS (G12V), and overexpressed CMYC to human fibroblasts immortalized by telomerase, with silenced WT p53. The oncogenes were introduced either solo or in various combinations – each combination in two possible orders of introduction – within a 30-day time-course. This model allowed us to determine how the studied oncogenes, and their order of introduction, affect global transcriptional programs and phenotypes driven by the oncogenes.
**Results and Discussions**
We determined which transcripts and molecular pathways are co-activated by oncogene cooperation, and which are redundant or downregulated by oncogene competition. The results demonstrate that p53 mutants are under competitive pressure from mutant KRAS and CMYC, which diminishes the gain-of-function influence of p53 mutants and shifts the molecular landscape of the cells towards specific pathways. However, both p53 mutants exert their own competitive pressure towards mutant KRAS and CMYC, significantly downregulating hundreds of transcripts driven by these oncoproteins.
**Conclusion**
Our results suggest that the resulting molecular "signatures" of cells with specific configurations of activated oncogenes are shaped just as much by competition as by cooperation among the oncogenic drivers. We are continuing to determine how the signatures of oncogene cooperation vs. competition are represented by phenotypes, cellular processes, and how they affect therapeutic protocols against cancer cells - using the fibroblast model, cancer cell lines, and tumor-derived organoids.

### EACR2024-0393
### Novel key genes involved in cancer malignancy

*M. Otaegi Ugartemendia*[1], *J. Elizazu*[1], *A. Artetxe-Zurutuza*[1], *A. Matheu*[1,2], *E. Carrasco-Garcia*[1,2]
[1]*Biogipuzkoa Health Research Institute, Cellular Oncology group, San Sebastian, Spain*
[2]*IKERBASQUE, Basque Foundation for Science, Bilbao, Spain*
**Introduction**
There are key drivers in cancer progression and malignancy. In this context, our group and others have demonstrated that SOX9, a developmental transcription factor that is a master regulator controlling super-enhancer dynamics in stem cell plasticity, exerts a relevant pro-oncogenic role in multiple tumour types by regulating cancer stem cell (CSC) activity. Thus, different genes highly correlated with *SOX9* in tumor samples represent outstanding candidates to play a relevant role in CSC activity, tumor malignancy, prognosis, and cancer therapy.
**Material and Methods**
We performed computational analyses to identify the top 80 genes most positively correlated with *SOX9* expression in gastric cancer (GC) samples. Among them, we selected a subset of 10 candidates and studied their clinical relevance in multiple cancer types. We completed loss-of-function and pharmacological inhibition studies in vitro and in vivo, as well as molecular studies (transcriptomics and proteomics) to elucidate the role and mechanism of the most promising candidates.
**Results and Discussions**
The expression of the candidates displayed a prognostic impact in multiple cancer types where they correlated with cancer progression and malignancy. Among them, bioinformatic studies revealed a 4-gene signature (*ECT2, TPX2, KIF11* and *DIAPH3)* associated with poor prognosis, therapy resistance and progenitor cell population. We selected *KIF11* and *DIAPH3,* and deciphered their role in tumor cell activity finding that both are required for tumor cell survival and CSC activity in different types of cancer. Of note, the pharmacological inhibition of KIF11 and DIAPH3 severely reduces GC cell viability and self-renewal in vitro, and tumor incidence and growth in vivo.
**Conclusion**
We used a novel and unbiased computational approach to identify novel drivers of cancer progression and malignancy. In particular, we identified a novel 4-gene signature related to cancer prognosis and revealed the role of *KIF11* and *DIAPH3* for tumor stem cell maintenance as well as the potential of their inhibition as a promising therapeutic strategy in cancer.

### EACR2024-0400
### Extracellular vesicles derived from pro-inflammatory macrophages induce inflammatory and invasive phenotype of melanoma cells

*K. Mäki-Mantila*[1], *K. Kainulainen*[1], *A. Rönkä*[2], *N. Aaltonen*[1], *K. Rilla*[1], *S. Pasonen-Seppänen*[1]
[1]*University of Eastern Finland, Institute of Biomedicine, Kuopio, Finland*
[2]*Kuopio University Hospital, Cancer Center, Kuopio, Finland*
**Introduction**
Melanoma progression and metastasis depend on intercellular communication in the tumor micro-environment (TME), in which extracellular vesicles (EVs) have emerged as essential mediators via their molecular cargo. In melanoma, different immune cells, especially macrophages, are enriched in the TME and their abundance and infiltration to the tumor area have been associated with poor prognosis. Tumor associated macrophages are roughly divided into proinflammatory M1 macrophages and immunosuppressive M2 macrophages, which both secrete EVs to modify the inter-cellular communication in the TME. As the macrophages play crucial role in the melanoma progression, we have investigated the effects of M1 and M2 macrophage derived EVs on melanoma cells to gain insight on EV-mediated macrophage-tumor cell interactions that drive melanoma progression.
**Material and Methods**
THP-1 cells were cultured in the presence of PMA to differentiate monocytes into M0 macrophages, which were further polarized to M1 and M2 macrophages with LPS and IFN$^{\gamma}$ or with IL-4 and IL-13, respectively. EVs were isolated from the conditioned media with

Molecular Oncology 18 (Suppl. 1) (2024) 1–495
© 2024 The Authors. Molecular Oncology is published by John Wiley & Sons Ltd on behalf of Federation of European Biochemical Societies differential ultracentrifugation. Thereafter, EVs were characterized with Western blot, transmission electron microscopy and nanoparticle tracking analysis, and the effects of M0-, M1- and M2-EVs on MV3 melanoma cells were studied with invasion assays, qPCR and confocal microscopy.

**Results and Discussions**

Our results revealed that M1-EVs induce invasion of MV3 melanoma cells in 3D invasion model. Furthermore, they upregulated the expression of inflammation (CXCL8, IL-6) and invasion (MMP-9) related genes on melanoma cells. With Western blot and MMP-zymography, we discovered that M1-EVs themselves contain both pro and active forms of MMP-9 on their cargo. Since MMP-9 has matrix degrading functions, we assume that it might be responsible for the enhanced invasion of melanoma cells. Moreover, inhibitor experiments have shown that NF-kB and TLR4 signaling pathways drive M1-EV mediated effects as their inhibitors suppressed M1-EV induced expression of pro-inflammatory genes and MMP-9 in melanoma cells.

**Conclusion**

Our results show that M1-EVs promote the malignancy of melanoma cells by inducing their invasive and inflammatory phenotype by activating TLR4 and NF-kB signaling pathways. These results emphasize the importance of EV-mediated interactions between immune and tumor cells on cancer progression.

### EACR2024-0419
### Carbonic anhydrase IX induces human osteosarcoma cell metastasis by activating heat shock protein family A member 6 expression via the adenosine monophosphate-activated protein kinase pathway

*J.S. Yang[1], S.F. Yang[2]*
*[1]Chung Shan Medical University Hospital, Department of Medical Research, Taichung, Taiwan*
*[2]Chung Shan Medical University, Institute of Medicine, Taichung, Taiwan*

**Introduction**

Osteosarcoma, the most common primary bone cancer, has a high metastatic potential and contributes to high mortality rates. Carbonic anhydrase IX (CAIX), a hypoxia-induced transmembrane protein, shows high expression in myriad cancers. Over the past decades, scientists have made extensive efforts to find the role of CAIX in various tumor progressions. However, the effects of CAIX on the cancer metastasis of osteo-sarcoma cell lines remain unclear. Therefore, we examined the effectiveness of CAIX in cellular invasion and migration of human osteosarcoma and the underlying molecular mechanisms.

**Material and Methods**

We conducted reverse transcription-polymerase chain reaction (RT-PCR), real-time PCR, western blot, Boyden chamber assay, flow cytometry, microculture tetrazolium colorimetric (MTT) assay and RNA sequencing technology to investigate human osteosarcoma cell lines.

**Results and Discussions**

In this study, we established the CAIX-overexpressing vector and found that the migratory and invasive abilities were dramatically increased in HOS and U2OS cell lines. In addition, CAIX overexpression increased the mRNA and protein expression of heat shock protein family A (Hsp70) member 6 (HSPA6) and the phosphorylation of adenosine monophosphate-activated protein kinase (AMPK) signaling proteins. After the knockdown of HSPA6, U2OS cells' biological behaviors of cellular invasion and migration were significantly reduced. Moreover, treatment with an AMPK inhibitor (Dorsomorphin) inhibited CAIX-induced HSPA6 expression and cell motility in U2OS cells.

**Conclusion**

According to the results, it can be inferred that CAIX overexpression induces HSPA6 expression via the AMPK signaling pathway, which consequently induces the metastasis of osteosarcoma cells.

### EACR2024-0423
### ADGRD1 and ADGRL4 as novel therapeutic approaches for glioblastoma treatment

*E. Lloret López[1], C. Pastor Moya[2], J.L. Neira Faleiro[3], L.M. Valor Becerra[4], M. Saceda Sánchez[1], C. De Juan Romero[1]*
*[1]Fundación para el Fomento de la Investigación Sanitaria y Biomédica de la Comunitat Valenciana FISA BIO, Hospital General Universitario Elche - Molecular Oncology, Elche, Spain*
*[2]Instituto de Investigación- Desarrollo e Innovación en Biotecnología Sanitaria IDiBE - Universidad Miguel Hernández, Molecular Oncology, Elche, Spain*
*[3]Instituto de Investigación- Desarrollo e innovación en Biotecnología Sanitaria IDIBE - Universidad Miguel Hernández, Agroquímica y Medio Ambiente, Elche, Spain*
*[4]Unidad de Investigación- Hospital General Universitario Dr. Balmis, Instituto de Investigación Sanitaria y Biomédica de Alicante ISABIAL, Alicante, Spain*

**Introduction**

Glioblastoma (GBM) is a highly aggressive grade IV glioma. This brain tumor has a poor prognosis mainly due to the lack of effective therapies for its treatment. This work focuses on the identification of new biomarkers, therapeutic targets, and effective methods of drug administration to treat GBM, based on the study of adhesion G Protein-coupled Receptors (aGPRs). The aGPR family is conformed by 33 membrane proteins named as ADGR.

**Material and Methods**

RNA-Seq analysis was performed to better understand the expression of aGPRs in human samples derived from patients that went under surgery for astrocytoma, oligodendroglioma, ependymoma, and GBM. Parallelly, we performed an exploratory bioinformatic analysis comparing GBM cells and GBM stem cells (GSC). In vitro analyses were performed in GBM cell lines, as well as primary cultures, obtained from patients from the Hospital General Universitario de Elche. Molecular biology techniques and immunocytochemistry protocols were used to confirm the expression of ADGRL4 and

© 2024 The Authors. Molecular Oncology is published by John Wiley & Sons Ltd on behalf of Federation of European Biochemical Societies

ADGRD1 on GBM cells and visualize them in a cellular context. To better understand their role of these receptors, we performed gene silencing protocols followed by molecular biology techniques.

**Results and Discussions**

We observed altered expression of certain aGPRs in GBM compared with other kind of brain tumors, suggesting their implication on GBM onset, development, and progression. To narrow down the selection, we search them in the literature. We found an overexpression in GSC of ADGRD1, that may underlie an important role regarding treatment resistance. ADGRL4 was found widely related with angiogenic processes in GBM, crucial for tumor survival and progression. Therefore, we focused on determining the expression and role of them on GBM patient derived tumor cell lines. RNA levels were found to be very different depending on GBM cell line. Immuno-fluorescence techniques showed a great percentage of GSC cells that expressed those receptors. The relevance of targeting these channels in tumors is supported by their expression in several GBM cells, where they may represent a link between the tumor microenvironment and the specific metabolic and adaptive properties of tumor cells to the tumor niche.

**Conclusion**

We propose that a novel therapeutic strategy based on the pharmacological inactivation of ADGRL4 and ADGRD1, could induce significant GBM and GSCs damage while being non-toxic for neurons. This approach might offer a promising and appealing new translational pathway for the treatment of GBM.

### EACR2024-0424
### N-acetyl-L-cysteine (NAC) impairs osteosarcoma aggressiveness: finding new therapeutic opportunities

*A. Ucci[1], L. Giacchi[1], C. Puri[1], K. Ferrante[2], S. Falone[2], N. Rucci[1]*
*[1]University of L'Aquila, Department of Biotechnological and Applied Clinical Sciences, L'Aquila, Italy*
*[2]University of L'Aquila, Department of Life- Health and Environmental Sciences, L'Aquila, Italy*

**Introduction**

Osteosarcoma (OS) is the most common primary bone tumour, predominantly affecting children and adolescents, and is prone to develop drug resistance and lung metastases. Currently, chemotherapy with high-dose methotrexate (MTX), often used combined with Cisplatin and Doxorubicin, is the gold standard treatment. The over-the-counter product N-acetyl-L-cysteine (NAC), a reactive oxygen species scavenger, has been demonstrated to exert controversial effects on cancer, however its effect on OS has been poorly elucidated. We aimed to investigate this aspect, to develop alternative therapeutic approaches for counteracting OS growth.

**Material and Methods**

We treated the human OS cell line MNNG/HOS with 5mM NAC for 48 hours(h) and we examined cell viability, proliferation, aggressiveness, and death. To exploit the effect of NAC on OS cells in combination with chemotherapy, we treated MNNG/HOS with NAC and MTX, assessing cell metabolic activity.

**Results and Discussions**

After treatment of MNNG/HOS with NAC for 48h, we found a concentration-dependent reduction of cell number and metabolic activity, evaluated by crystal violet and MTT assay, compared to untreated cells. Due to the mesenchymal/osteoblastic origin of OS, we also treated primary mouse osteoblasts and the human fetal osteoblastic cell line FOB with NAC, finding a reduction of metabolic activity only at the highest concentration tested (10 mM), thus suggesting that tumour cells are more sensitive to NAC than normal cells. We next evaluated any effect of NAC on MNNG/HOS proliferation, finding no effect after 24h of treatment with 5mM NAC, while a reduction was observed at 48h compared to untreated cells. However, apoptosis and cell viability, observed by flow cytometry and trypan blue, were not affected by NAC treatment. Interestingly, MNNG/HOS pre-treated for 48h with 5mM NAC also showed a significant lower ability to invade through Matrigel. Consistently, zymography performed on conditioned medium collected from MNNG/HOS treated with 5mM NAC showed a significant reduction of MMP-2 activity, compared to untreated cells. Finally, we assessed the effect of NAC in combination with 0.5µM MTX for 48h, finding that it was more effective in reducing metabolic activity than single treatments.

**Conclusion**

Our data indicate that treatment of OS cells with NAC significantly impairs their aggressiveness. Further experiments will allow to elucidate the mechanisms involved, with the proposal to understand whether NAC could be a valid antitumoral molecule to be used in OS therapy.

### EACR2024-0428
### Antiproliferative effects of HDL on prostate cancer cell lines: role of oxysterols

*F. Cetti[1], C. Garavaglia[1], M. Pedrelli[2], P. Parini[2], M. Gomaraschi[1]*
*[1]University of Milan, Department of Pharmacological and Biomolecular Sciences, Milan, Italy*
*[2]Karolinska Institute, Department of Medicine and Department of Laboratory Medicine, Huddinge, Sweden*

**Introduction**

As one of the key lipoproteins involved in cholesterol transport, high density lipoproteins (HDL) plays an important role in maintaining and regulating the cholesterol homeostasis in tumor cells. In this context, HDL were shown to reduce prostate cancer (PCa) cell content of cholesterol, thus inhibiting cell proliferation. Nevertheless, little is known about the overall effect on cholesterol homeostasis. Besides its important role as regulator of several cellular functions, cholesterol can also be converted in oxysterols, bioactive lipids usually known for their antiproliferative effects. However, tumor cells have an aberrant production of oxysterols, and some of them can contribute to tumor growth, as demonstrated for breast cancer. Aim of the study was to assess the impact of oxysterols on PCa cell proliferation and to investigate whether HDL can affect their intracellular levels.

**Material and Methods**

Molecular Oncology 18 (Suppl. 1) (2024) 1–495
© 2024 The Authors. Molecular Oncology is published by John Wiley & Sons Ltd on behalf of Federation of European Biochemical Societies

Experiments were performed in androgen-sensitive (LnCap) and androgen-independent (PC3) PCa cell lines, compared to non-tumor PNT2. HDL were isolated by ultracentrifugation from human plasma. Intracellular levels of oxysterols were assessed by GC/MS. Cell proliferation was evaluated by cell count and MTS assay on cells exposed to 27-hydroxycholesterol (27-HC) and/or HDL.

**Results and Discussions**

PCa cell lines showed a different distribution of oxysterols compared to non-tumor PNT2. In particular, 27-HC levels were up to 5 times higher in PC3 cells than in PNT2. 27-HC significantly increased cell proliferation up to 30% in LnCap and to 15% in PC3. The exposure to HDL drastically reduced the content of all oxysterols, including 27-HC, in the three cell lines. Consequently, cell proliferation induced by 27-HC was completely prevented when cells were exposed to HDL.

**Conclusion**

Our results showed that 27-HC increased the proliferation of PCa cell lines, which is prevented by HDL. Therefore, the reduction of intracellular levels of oxysterols could contribute to the antiproliferative effects of HDL on tumor cells. Further studies are needed to deeply investigate the underlying mechanisms, which could be related to the activation of hormone receptors by 27-HC, as observed in breast cancer, and to the overexpression of the ABCG1 transporter in PCa cells.

### EACR2024-0432
### The tumour suppressor FAM46C Is an Interferon-Stimulated Gene That Inhibits Lentiviral Particle Production by Modulating Autophagy

*G. Lai[1], M. Mancino[1], F. De Grossi[1], A. Cuomo[2], L. Manganaro[3], G.M. Butta[3], I. Ferrari[1], E. Vincenzi[4], S. Biffo[1], N. Manfrini[1]*
*[1]INGM, Molecular Histology Cell Growth, Milan, Italy*
*[2]IEO, Milan, Milan, Italy*
*[3]INGM, Virology, Milan, Italy*
*[4]San Raffaele Scientific Institute, Viral Pathogenesis and Biosafety Unit, Milan, Italy*

**Introduction**

FAM46C is a well-established tumor suppressor gene associated with Multiple Myeloma (MM). Recent evidence, however, suggested a role for FAM46C way beyond the MM scenario, depicting it as a pan-cancer tumor suppressor. Despite the increasing number of studies regarding FAM46C, its mode of action still remains debated. Preliminary data in the lab showed an involvement of FAM46C with viral particle production. Given that viral infection/replication is known to affect cancer risk through different mechanisms, we wanted to test if FAM46C could regulate the viral life cycle.

**Material and Methods**

To test FAM46C involvement in viral replication, at first we produced GFP-expressing lentiviral particles in HEK-293T in the presence or absence of FAM46C and assessed viral particle production. We did so by western blot, through detection of p24 viral capsid abundance in lentiviral producing cells and by flow cytometry, quantifying the number of $GFP^+$ cells upon reinfection with equal volumes of lentiviral-containing supernatants. To understand if FAM46C was affecting lentiviral particle production at the transcriptional or translational level, viral mRNA abundance was monitored by RT-qPCR on whole cell lysates or on polysomal profiles. Given that FAM46C was shown to affect the autophagic flux in MM, autophagy involvement was investigated by monitoring p62 and LC3B protein levels. Finally, we investigated the interactome of FAM46C upon lentiviral production through mass spectrometry and tested if FAM46C could be stimulated by Interferon (IFN) administration.

**Results and Discussions**

We found that FAM46C is a type I and type II interferon-stimulated gene and that the expression of its wild-type form, but not of its most frequently found mutant variant, the D90G variant, inhibits the production of both HIV-1-derived and HIV-1 lentiviruses in HEK-293T cells. Moreover, we showed that the inhibitory effect of FAM46C on lentiviral production is not due to transcriptional or translational regulation, but rather to FAM46C capability to inhibit autophagy, as previously seen in the MM context. Finally, by mass spectrometry we found that upon lentiviral particle production the interactome of FAM46C is enriched in proteins involved in organelle homeostasis and intracellular trafficking.

**Conclusion**

In conclusion we found that FAM46C inhibit lentiviral production in HEK-293T cells by inhibiting autophagy and possibly altering intracellular trafficking dynamics is a manner similar to that describing its tumor suppressor function in MM.

### EACR2024-0434
### Unveiling SYNPO2L: A Novel Tumor Suppressor in Breast Cancer

*S. Machado[1], J. Forner[1], A. Cantat[1], T.P. Páníková[1], J. Morena[1], S. Lopes[1], Z. Kečkéšová[1]*
*[1]Institute of Organic Chemistry and Biochemistry of the CAS IOCB, Molecular Biology and Biochemistry, Praha, Czech Republic*

**Introduction**

Cancer represents one of the main health problems worldwide, being the second leading cause of death globally. Tumor suppressors constitute the frontline defense against cancer, acting as vigilant gatekeepers of cellular integrity and genomic stability. Their pivotal role lies in orchestrating intricate signaling pathways that regulate fundamental cellular processes, including cell cycle progression, DNA repair, and cell death. Our screening of tissues resistant to cancer formation led to the discovery of SYNPO2L as a potential novel tumor suppressor. SYNPO2L is a paralog of SYNPO2 and is involved with structural development and function of the cardiac myocyte. We implemented molecular biology approaches to elucidate the tumor suppressor role of SYNPO2L and its mechanism of action.

**Material and Methods**

We generated breast cancer cell lines with inducible expression of SYNPO2L, and performed cell proliferation, migration, and cell death assays to assess whether SYNPO2L promoted cancer cell death and/or

© 2024 The Authors. Molecular Oncology is published by John Wiley & Sons Ltd on behalf of Federation of European Biochemical Societies senescence. SYNPO2L expression levels were analyzed by immunohistochemistry in a panel of human breast cancer tissues of different stages of tumor progression. Xenograft assays on mice were performed by injecting cancer cells expressing SYNPO2L, to evaluate its impact on tumor growth. We identified by RNA-seq the SYNPO2L downstream effectors and characterized its molecular mechanisms to devise ways of modulating SYNPO2L tumor suppressor activity, as well as bioinformatic analyses to correlate SYNPO2L expression with clinical data.

**Results and Discussions**

SYNPO2L was shown to act as a tumor suppressor by decreasing cancer cell proliferation and migration, promoting cell death, and preventing tumor growth in mice, with no detectable impact in non-tumorigenic samples. SYNPO2L expression in cancer tissues decreased with tumor stage, and cancer patients' survival rate is increased in patients with higher SYNPO2L expression. SYNPO2L significantly altered the transcriptional signature of several breast cancer cell types, and key downstream genes and pathways were identified.

**Conclusion**

The investigation of SYNPO2L opened avenues for research with exciting questions we want to address, such as pharmacologically targeting SYNPO2L or its downstream effectors to modulate tumor suppressor activity. Ultimately, the objective of this study is to identify and characterize novel tumor suppressive circuitries operating in human cancer that will uncover therapeutic sensitivities of cancer cells.

### EACR2024-0435

### Human amniotic membrane extract as an approach for liver cancer: decoding therapeutic insights through a proteomic analysis

*R. Teixo[1,2,3], S. Aveiro[4,5], B. Serambeque[1,2,3], C. Almeida-Ferreira[1,2,3], M. Laranjo[1,2,3], M.J. Carvalho[1,2,3,6,7], A.M. Abrantes[1,2,3], M.F. Botelho[1,2,3], P. Domingues[4]*

*[1]Univ Coimbra, Coimbra Institute for Clinical and Biomedical Research iCBR area of Environment Genetics and Oncobiology CIMAGO- Institute of Biophysics- Faculty of Medicine, Coimbra, Portugal*

*[2]Univ Coimbra, Center for Innovative Biomedicine and Biotechnology CIBB, Coimbra, Portugal*

*[3]Clinical Academic Center, Coimbra, Coimbra, Portugal*

*[4]University of Aveiro, Mass Spectrometry Centre- Department of Chemistry & QOPNA, Aveiro, Portugal*

*[5]University of Algarve, GreenCoLab - Green Ocean Association, Faro, Portugal*

*[6]Univ Coimbra, Universitary Clinic of Gynecology- Faculty of Medicine, Coimbra, Portugal*

*[7]Unidade Local de Saúde de Coimbra, Gynecology Service- Department of Gynecology- Obstetrics- Reproduction and Neonatology, Coimbra, Portugal*

**Introduction**

Human amniotic membrane (hAM) presents anti-tumor properties, including anti-angiogenic and pro-apoptotic activity. We previously showed that total hAM extract (hAME) leads to anti-tumor effects on liver cancer cells. Our goal was to characterize the changes in the proteomic profiles of liver cancer cell lines induced by hAME and identify potential key players responsible for its anti-tumor effects of hAME.

**Material and Methods**

hAM was collected by elective cesarean section after informed consent. hAME was obtained by tissue fragmentation and homogenization and the supernatant was collected after centrifugation (14000 G, 15min, 4°C). Human liver cancer cells HepG2, Hep3B and HuH7 were incubated for 72h with a protein concentration of 1µg/µL. Total protein extracts were obtained, and then SDS-PAGE electrophoresis was performed with 30µg of protein from each sample (non-treated cells and cells incubated with hAME) in a 12.5% acrylamide gel, resolved at 150V. The gel was stained with 0.12% commassie blue G-250 solution. In gel digestion was performed and identification of proteomic profile was determined by mass spectrometry (MS). Subsequent bioinformatics analyses were used to explore the results.

**Results and Discussions**

We successfully identified a total of 2900 proteins in all samples. In HepG2 cells, 41 proteins were found significantly more abundant after incubation with hAME, while 57 were significantly less abundant compared to untreated cells. In Hep3B cells, hAME incubation led to 30 proteins significantly more abundant and 20 significantly less abundant than control cells. Lastly, HuH7 cells incubated with hAME showed 52 proteins significantly more abundant and 39 proteins less abundant compared to control cells. Identification of these significantly altered proteins allowed us to confirm their involvement in several cell pathways.

**Conclusion**

Our findings demonstrate that incubation with hAME leads to significant alterations in the proteomic profiles of the human liver cancer cells. Identification of altered proteins involved in different cell pathways suggests that hAME could induce a complex set of mechanisms that ultimately lead to anti-tumor effects.

*Funding: CIBB strategic projects UIDB/04539/2020 (DOI: 10.54499/UIDB/04539/2020) and UIDP/04539/2020 (DOI: 10.54499/UIDP/04539/2020) and Associated Laboratory funding LA/P/0058/2020 (DOI: 10.54499/LA/P/0058/2020) from FCT. Scholarship grants from FCT and European Social Funding (SFRH/BD/116794/2016, 2020.07672.BD).*

### EACR2024-0443

### Blunting Axitinib-related side effects with antioxidants

*A. Formato[1], M. Salbini[1], E. Orecchini[1], Q.G. D'Alessandris[2], R. Pallini[3], M.L. Falchetti[1], M.P. Mongiardi[1]*

*[1]Institute of Biochemistry and Cellular Biology, Consiglio Nazionale delle Ricerche, Monterotondo RM, Italy*

*[2]Department of Neurosurgery, Fondazione Policlinico Universitario A. Gemelli IRCCS, Rome, Italy*

*[3]Neurosurgery Section, Department of Neuroscience- Università Cattolica del Sacro Cuore, Rome, Italy*

**Introduction**

Axitinib (Axi) is a small and selective inhibitor of Vascular Endothelial Growth Factor Receptors, approved for advanced renal cell carcinoma therapy and under

Molecular Oncology 18 (Suppl. 1) (2024) 1–495

© 2024 The Authors. Molecular Oncology is published by John Wiley & Sons Ltd on behalf of Federation of European Biochemical Societies investigation for glioblastoma treatment. Others and we demonstrated that Axi triggers cellular senescence in vitro through ROS increase and ATM kinase activation, both in tumor and in normal cells (Morelli; Mongiardi). We observed that the establishment of senescence in normal cells is strongly dependent on ROS accumulation and that the use of antioxidant drugs effectively prevents Axi-dependent senescence. Conversely, in glioblastoma cells, Axi maintains its pro-senescence activity also in co-treatment with antioxidants.

**Material and Methods**

These results represent the basis of the present study in which we deeply characterize, in vitro and ex vivo, normal endothelial and glioblastoma cells' response to Axi plus antioxidants (N-acetylcysteine, NAC). The characterization of tumor counterpart has been performed on patients-derived glioma stem cells (GSC), considered the gold standard for preclinical models.

**Results and Discussions**

In vitro, co-treatment maintains the antitumor effect exerted by Axi on GSCs and does not prevent the establishment of the senescent phenotype, characterized by 2D and 3D approaches. In vivo, in xenograft models of brain tumors, we confirmed that NAC does not limit the anti-tumor effectiveness of Axi. Strikingly, we observed that NAC co-treatment reduces Axi-dependent toxicity in filter organs, as revealed by analyzing their vasculature.

**Conclusion**

Our results demonstrate that the use of antioxidants in combination with Axi reduces Axi-related toxicity, with no impairment of Axi antitumor efficacy.

### EACR2024-0446

### ETS-mediated regulation of SLPI, an inflammation related gene, may affect prostate cancer development

*I. Cosi*[1,2], *A. Moccia*[2], *D. Rosini*[2,3], *C. Nannelli*[2], *M. Sica*[2], *R. Notaro*[2], *M. De Angioletti*[2,4]

[1]*University of Study of Florence, Neurofarba, Firenze, Italy*

[2]*Istituto per lo Studio- la Prevenzione e la Rete Oncologica ISPRO, Core Research Laboratory, Florence, Italy*

[3]*University of Siena, Doctorate in Genetics- Oncology and Clinical Medicine GenOMeC, Siena, Italy*

[4]*CNR, Istituto di Chimica dei Composti Organo Metallici, Florence, Italy*

**Introduction**

ETS transcription factors (ERG, ETV1, ETV4) have a direct pathogenic role in prostate cancer (PC). We have shown that ETV4 increases migration, invasion, proliferation, cell cycle and anchorage-independent growth in vitro and induces prostatic intraepithelial lesion in a transgenic mouse model (ETV4 mice). Secretory Leukocyte Peptidase Inhibitor (SLPI) is a protease inhibitor that protects epithelial tissues from protease at sites of inflammation. SLPI seems to play a role in several cancers including PC. Low levels of SLPI have been reported in patients with early stage PC and high levels in patients with advanced PC.

**Material and Methods**

We investigated SLPI expression in ETV4 mice and human prostate cells by RT-qPCR and Western blot. The effect of ETV4 and ETV1 on SLPI expression has been investigated in human prostate cells by overexpression (RWPE1) and silencing (ETV4 in PC3; ETV1 in LNCap). ETV4 and ETV1 regulation of SLPI has been investigated by ChIP and Luciferase assays. The effects of SLPI in prostate cells have been tested on aptoptosis (Annexin V), migration (scratch test), invasion (matrigel).

**Results and Discussions**

Our ETV4 mice, a model of early PC, express low SLPI levels in prostate: this is intriguingly similar to the finding in early stage PC patients. We found that ETV4 and ETV1 silencing upregulates SLPI in, respectively, PC3 and LNCap cell lines whether their overexpression downregulates SLPI in normal prostate cell line RWPE. In addition, we found that ETV4 and ETV1 reduce luciferase expression driven by the SLPI promoter but ChIP experiments have not shown a direct binding to SLPI promoter. SLPI silencing in RWPE cells increases apoptosis mainly through caspase 8, reduces cell migration and invasion by the inhibition of matrix metalloproteinases and of Epithelial-Mesenchymal Transition through the inhibition of SLUG, TWIST and ZEB1. Thus the low levels of SLPI seem to counteract and reduce the neoplastic features induced by ETV4.

**Conclusion**

ETV4 and ETV1 downregulate SLPI in mice and in human prostate cell lines. These low levels of SLPI increasing apoptosis, reducing EMT and favoring the increase protease activity could affect negatively the growth of early prostate cancer cells. Thus, ETS-mediated downregulation of SLPI, that parallel the low SLPI levels in both ETV4 mice and in patients with early PC, may play a role in determining the indolent features of early PC.

*Funded by Regione Toscana-bando Ricerca Salute 2018*

### EACR2024-0454

### Enhancing cytotoxicity of docetaxel using plant-derived hexadecanoic acid

*S.G. Wright*[1], *W. Aiken*[2], *H. Asemota*[3], *K. Foster*[4], *R. Delgoda*[5]

[1]*University of the West Indies, Biomedical Engineering, St. Andrew, Jamaica*

[2]*University of the West Indies Mona, Department of Surgery, St. Andrew, Jamaica*

[3]*University of the West Indies Mona, Department of Basic Medical Sciences, St. Andrew, Jamaica*

[4]*University of the West Indies, Natural Product Institute, St. Andrew, Jamaica*

[5]*University of The West Indies Mona, Natural Product Institute, St. Andrew, Jamaica*

**Introduction**

Combining multiple drugs to enhance the efficacy of cancer treatment while mitigating adverse effects is a critical area of research in oncology. More specifically, plant-derived medicines have emerged as a compelling area of focus, offering a diverse and rich resource of bioactive compounds with the potential to support or enhance conventional medical therapies

**Material and Methods**

© 2024 The Authors. Molecular Oncology is published by John Wiley & Sons Ltd on behalf of Federation of European Biochemical Societies

In this study, we investigated the potential of plant-derived hexadecanoic acid as a complementary agent in conjunction with the commonly used chemotherapeutic drug, docetaxel, to assess its impact on cytotoxicity and cell migratory properties. We conducted cytotoxicity screens using PC3 cell lines and evaluated the inhibitory effects of the hexadecanoic acid on cell migration in PC3-emt cells.

**Results and Discussions**

Our results revealed that hexadecanoic acid exhibits significant cytotoxicity against PC3 cell lines, accompanied by a statistically significant reduction in cell migration in the PC3-emt cells. Furthermore, when combined with docetaxel, hexadecanoic acid demonstrated a synergistic effect by reducing the IC-50 of docetaxel from 0.005 µg/ml to 0.004 µg/ml. This decrease in the IC-50 underscores the potential of hexadecanoic acid to enhance the cytotoxicity of docetaxel, offering a promising strategy for improved cancer treatment outcomes. The use of plant-derived hexadecanoic acid in combination with docetaxel may possibly reduce the adverse side effects typically associated with docetaxel monotherapy. This observation emphasizes the need for further investigation to assess the safety and efficacy of this combination therapy in prostate cancer patients.

**Conclusion**

In conclusion, our study suggests that the incorporation of plant-derived hexadecanoic acid as an adjunct to docetaxel represents a significant advancement in cancer therapy. This approach may not only enhance the effectiveness of current chemotherapeutic regimens but also help alleviate the side effects associated with these treatments, offering new hope for cancer patients. Future studies are warranted to fully explore the potential of this novel drug combination.

### EACR2024-0459
### Role of Plexin A3 in Hepatocellular Carcinoma Progression

*J.Y. Tan*[1], *S.K.Y. Ma*[1], *T.L. Wong*[1]
[1]*The University of Hong Kong, School of Biomedical Sciences, Hong Kong, Hong Kong-China*

**Introduction**

Plexin/semaphorins are important regulators for cell migration and recent studies have implicated their involvement of cancer growth and metastasis. This study aims to investigate the potential involvement of PLXNA3 during hepatocellular carcinoma (HCC) progression.

**Material and Methods**

Gene expression profiles from two publicly available datasets, The Cancer Genome Atlas - Liver Hepato-cellular Carcinoma (TCGA-LIHC) and GSE14520, were compared. Statistical analysis using the Fisher exact test or chi-square test was performed to evaluate the association between PLXNA3 expression and clinical parameters. HCC cell lines, Huh1 and HepG2, were used for CRISPR activation and CRISPR knockout of PLXNA3, respectively. Validation of PLXNA3 protein expression was done by western blotting. Functional assays such as migration, invasion assay, foci formation, and limiting dilution assay (LDA) were then performed. In vivo experiments were conducted to validate the functional role of PLXNA3. Furthermore, regulatory mechanisms of PLXNA3 were investigated through bioinformatics analysis and luciferase reporter assay.

**Results and Discussions**

PLXNA3 expression was associated with worse clinical outcomes, suggesting its potential as a prognostic marker in HCC. Functional assays demonstrated a significant association between PLXNA3 expression and enhanced migratory, invasive, cell proliferative, and tumor-initiating abilities in HCC cells. Furthermore, luciferase reporter assays revealed higher luciferase activity under hypoxic conditions, indicating increased transcriptional activity of PLXNA3 in response to low oxygen levels. Additionally, gene and protein expression of PLXNA3 were found to be upregulated, further supporting the potential link between PLXNA3 expression and the hypoxic tumor microenvironment.

**Conclusion**

In summary, current findings demonstrated the role of PLXNA3 expression in the development of HCC. Future work will focus on elucidating the downstream mechanism of PLXNA3 in HCC progression.

### EACR2024-0460
### Interferon-induced protein IFIT5 interacts with focal adhesion protein TRIP6 and regulates the proliferation and migration of glioblastoma and its cancer stem cells

*Y.J. Lai*[1], *Y.H. Hung*[1], *T.C. Chen*[1]
[1]*National Taiwan Normal University, Department of Life Science, Taipei, Taiwan*

**Introduction**

Glioblastoma multiforme (GBM) is currently the most malignant primary brain cancer with low survival rates and a poor prognosis. Studies have found that cancer stem cells (CSCs) of GBM are associated with poor treatment effects, as they increase cancer cell proliferation and migration. Understanding how cancer stem cells are regulated in glioblastoma multiforme is crucial for the development of novel therapeutics. TRIP6 is a focal adhesion molecule that regulates cell adhesion, migration, and stem cell maintenance. Our previous studies have demonstrated that TRIP6 promotes GBM progression and cell migration. Interferon-induced protein with tetratricopeptide repeats 5 (IFIT5), which belongs to the IFIT protein family, plays an important role in the regulation of cell proliferation, migration, signal transduction, and virus replication. IFIT5 is one of the interferon-inducing proteins induced upon infection. It interacts with exogenous 5'-PPP-RNAs and inhibits cellular protein synthesis.

**Material and Methods**

To explore the regulatory role of IFIT5 in GBM, we analyzed the IFIT5 expression pattern from the TCGA database. We also generated IFIT5-overexpressing GBM cell lines and spheroids to examine their proliferation and migration abilities compared to control cells. Furthermore, we investigated the interaction between TRIP6 and IFIT5 and its effects on the migration of GBM cells.

**Results and Discussions**

© 2024 The Authors. Molecular Oncology is published by John Wiley & Sons Ltd on behalf of Federation of European Biochemical Societies

According to the analysis of TCGA clinical data, the higher expression level of IFIT5 correlates with better patient survival. We demonstrated that overexpression of IFIT5 inhibited cell proliferation and migration in GBM cell lines and their spheroid cells. It also caused cell cycle G1 arrest. Moreover, we found that IFIT5 interacts with TRIP6 and co-localizes with TRIP6 at actin filaments. IFIT5 binds to actin through interaction with TRIP6 and interferes with TRIP6-promoted cell migration.

**Conclusion**

IFIT5 may inhibit GBM progression and migration through interaction with TRIP6 and may have the potential to be a novel therapeutic for GBM.

### EACR2024-0467

### Drug resistance and stemness is promoted by GALNT14 and GDF-15 via β-catenin signaling pathway in breast cancer

*A. Gadwal*[1], *P. Purohit*[1], *J.R. Vishnoi*[2], *P. Pareek*[3], *R. Choudhary*[4], *P. Elhence*[5], *M. Banerjee*[1], *P. Sharma*[1]

[1]*All India Institute of Medical Sciences Jodhpur, Biochemistry, Jodhpur, India*

[2]*All India Institute of Medical Sciences Jodhpur, Surgical Oncology, Jodhpur, India*

[3]*All India Institute of Medical Sciences Jodhpur, Radiation Oncology, Jodhpur, India*

[4]*All India Institute of Medical Sciences Jodhpur, General Medicine, Jodhpur, India*

[5]*All India Institute of Medical Sciences Jodhpur, Pathology, Jodhpur, India*

**Introduction**

Altered glycosylation, particularly by GALNT14, a member of the N-acetylgalactosaminyltransferase family, is implicated in breast cancer (BC) progression. This enzyme modify proteins in a way that could enhance cancer cell survival and proliferation. Growth Differentiation Factor 15 (GDF-15), involved in inflammation, cell growth and apoptosis, promotes tumor progression and metastasis in BC. Elevated GDF-15 levels correlate with advanced BC stages and poorer prognosis. The precise molecular mechanisms of GALNT14 and GDF-15 in BC stemness(OCT4,SOX2) and drug resistance(ABCC5) remain unclear. This study evaluates GALNT14 and GDF-15 expression and their interactions with these markers in BC.

**Material and Methods**

Tumor and adjacent non-tumor tissue samples were collected from 30 BC pretherapeutic and post therapeutic patients, along with serum samples from patients and healthy controls. Bioinformatic analysis, including protein-protein interaction (PPI) studies, was conducted. Serum GALNT14 expression was measured and the expression levels of GALNT14,GDF-15,OCT4, SOX2,ABCC5, β-catenin were analyzed in BC tissue and MCF-7 cells using RTPCR. Knockdown of GALNT14 and GDF-15 in MCF-7 cells was achieved with siRNA and β-catenin protein expression were assessed using western blotting.

**Results and Discussions**

The PPI network revealed interactions among GALNT14, GDF-15, OCT4, SOX2, ABCC5, and β-catenin. BC tumor tissues exhibited significantly higher expression of these genes compared to adjacent non-tumor tissues. Serum GALNT14 levels were significantly elevated in BC patients (80.7±65.3 pg/ml) compared to healthy controls (12.2±9.12 pg/ml)($p<0.000$), suggesting a role in BC progression. Knockdown of GALNT14 and GDF-15 in MCF-7 cells reduced OCT4, SOX2, ABCC5, and β-catenin expression; co-knockdown further decreased their expression. Our previous study has shown that GDF-15 promotes aggressiveness of BC via AKT pathway and in this study we have shown β-catenin is stabilized by glycosylation through GALNT14 therefore, GALNT14 and GDF-15 together regulate the expression of β-catenin which ultimately regulates the stemness and drug resistance in BC.

**Conclusion**

Taken together, we showed evidence that GALNT14 and GDF-15 have the potential to promote stemness and intrinsic drug resistance in BC, possibly through Wnt/β-catenin axis, which leads to the aggressiveness of BC. Therefore, GALNT14 and GDF-15 presents as an exciting target of BC therapy which would help in the management of BC patients efficiently in the future

### EACR2024-0482

### NDUFS1 promotes breast cancer progression through mitochondrial activation and c-Myc signaling

*H.H. Yuan*[1]

[1]*Kaohsiung Medical Univ, Graduate Institute of Medicine, Kaohsiung, Taiwan*

**Introduction**

Adipose-derived stem cells (ADSCs) can promote breast cancer development through interactions in the tumor microenvironment. In the current study, we observed that ADSCs co-cultured with the adipocytokine resistin, enhanced breast cancer cell proliferation and colony formation in comparison to untreated ADSCs.

**Material and Methods**

For in vitro assays, overexpression and knockdown of NDUFS1 followed by cell proliferation, migration/ invasion and tumorsphere formation assays, seahorse and CLARIOstar for mitochondrial activities and RNA-seq analysis were applied. For in vivo study, syngeneic orthotopic mouse model was applied.

**Results and Discussions**

Breast cancer cells co-cultured with resistin-treated ADSCs showed elevated expression of NDUFS1, the largest subunit of mitochondrial complex I. Knockdown of NDUFS1 decreased, while overexpression increased, breast cancer cell proliferation, migration/invasion and tumorsphere formation through mitochondrial metabolism-mediated c-Myc/PROX1 pathway. Inhibition of NDUFS1-activated mitochondrial metabolism by metformin decreased Myc and PROX1 expression and breast cancer cell proliferation and migration. In vivo study using syngeneic orthotopic mouse model showed that NDUFS1 downregulation decreased mammary tumor growth with decreased expression of Ki67, Myc and PROX1.

**Conclusion**

Together, these data demonstrated that resistin may be involved in metabolic reprogramming in the tumor

© 2024 The Authors. Molecular Oncology is published by John Wiley & Sons Ltd on behalf of Federation of European Biochemical Societies microenvironment via NDUFS1 signaling, promoting breast cancer progression through activation of mitochondrial metabolism and c-Myc/PROX1 pathway. These pathways provide avenues for repurposing of metabolic inhibitors such as metformin.

### EACR2024-0491
### The cAMP elevating agent Forskolin inhibits proliferation and migration and enhances Paclitaxel-induced cytotoxicity in non-small-cell lung cancer (NSCLC) cells

S. Kafeel[1], A. Salzillo[1], A. Ragone[1], G. Palmiero[1], L. Sapio[1], S. Naviglio[1]
[1]*University of Campania "Luigi Vanvitelli", Department of Precision Medicine- School of Medicine, Naples, Italy*

**Introduction**

Non-Small-Cell Lung Carcer (NSCLC) is recognized as one of the deadliest neoplastic tumor and foremost cause of cancer associated mortality globally. Besides conventional treatment strategies, targeted and immune therapies suited only for particular subsets of NSCLC. Thus, more advanced therapeutics are needed to overcome the constraints of reoccurrence and invasive behavior of NSCLC. A naturally occurring compound Forskolin, extracted from the roots of Coleus forskohlii, frequently used in ayurvedic medicine and even as dietary supplement to lose weight, has shown anticancer potential in previous studies. However, the impact on NSCLC in response to Forskolin is still unclear and not completely understood. Aim of this study is to explore the in vitro response of Forskolin alone or in combination with Paclitaxel in NSCLC H1299 and A549 cell lines.

**Material and Methods**

H1299 and A549 cells were maintained in culture and treated with different concentrations of Forskolin and Paclitaxel supplemented alone or in combination. Cell proliferation and cytotoxicity was evaluated by cell growth assessment and viability assays. Cell cycle profile was evaluated by flow cytometry and migration ability of cells by Wound Healing Assay. Combination Index of various doses of Forskolin with Paclitaxel was estimated by Chou-Talalay method. Difference in the expression of related proteins was analyzed by Western Blotting.

**Results and Discussions**

Forskolin inhibits the growth and migration of both H1299 and A549 cell lines. Changes in cell cycle progression and epithelial-mesenchymal markers were noted in response to Forskolin treatment. Interestingly, IBMX, a cAMP phosphodiesterase inhibitor, also showed similar effects. The use of SQ22536, an adenylyl cyclase inhibitor, counteracted the Forskolin-induced effects. Furthermore, Forskolin was found to enhance the cytotoxic effects of Paclitaxel, primarily through the induction of apoptosis. Notably, inhibition of protein kinase A (PKA) by H89 compound exacerbated the combined effects of Forskolin and Paclitaxel.

**Conclusion**

Our data support the role of Forskolin as a possible anticancer therapeutic molecule in NSCLC treatment, identifying the adenylyl cyclase/cAMP axis as one of the relevant signaling pathways involved in. The findings of this preclinical study encourage further investigations on Forskolin-based approaches for NSCLC therapy.

### EACR2024-0492
### Doxorubicin-induced apoptosis of colon cancer cells is modulated by protease-activated receptor 2

H. Shah[1], T. Hill[1], J. Lim[1], D. Fairlie[2]
[1]*Institute for Molecular Bioscience- University of Queensland, Centre for Chemistry and Drug Discovery, Brisbane, Australia*
[2]*Institute for Molecular Bioscience- University of Queensland, Australian Research Council Centre of Excellence for Innovations in Peptide and Protein Science, Brisbane, Australia*

**Introduction**

A major problem with colon cancer treatments is that almost 50–60% of patients at early stages of cancer and 90% of patients with metastatic cancer develop resistance to chemotherapeutic drugs. New information is needed about colorectal cancer cell survival mechanisms and effective strategies to combat them. Doxorubicin (Adriamycin®) is an injectable DNA-intercalating chemotherapy drug that reduces cancer cell growth, but its long-term effectiveness is compromised by onset of drug resistance. Here, we investigated the association of protease-activated receptor 2 (PAR2), a G-protein coupled receptor, with doxorubicin-induced cell death in human colon cancer cells.

**Material and Methods**

Normalised gene expression data for human patients, recorded in the cancer genome atlas and genotype-tissue expression databases, was assessed using the UCSC Xena platform. In vitro experiments were performed to measure cancer cell viability, reactive oxygen species and caspase output, ERK1/2 phosphorylation levels and CRISPR-Cas9 gene knockout.

**Results and Discussions**

PAR2 gene expression in human colon adenocarcinoma tissues was highest among 32 different cancer types ($n = 10,989$), and higher in colon adenocarcinoma tissues ($n = 331$) than normal colon tissues ($n = 308$), revealing an association between PAR2 expression and human colon cancer. To investigate this, we selected human colon adenocarcinoma cell lines that are sensitive to doxorubicin and express high levels of PAR2. We found that PAR2 activation in cancer cells, either by endogenous protease or exogenous agonist, significantly reduced doxorubicin-induced cell death, reactive oxygen species production, caspase 3/7 activity and cleavage of caspase-8 and caspase-3 apoptotic markers. Moreover, PAR2-mediated MEK1/2-ERK1/2 signalling led to upregulated MCL-1 and Bcl-xL anti-apoptotic proteins that promote cell survival. These findings suggest that PAR2 activation compromises the efficacy of doxorubicin in colon cancer. Support for this conclusion came from cell experiments, either with PAR2 gene deleted or presence of a PAR2 inhibitor, where full restoration of all doxorubicin-induced effects was observed.

**Conclusion**

Collectively, our functional studies indicate that PAR2 signalling pathways interact with the mode of action of

© 2024 The Authors. Molecular Oncology is published by John Wiley & Sons Ltd on behalf of Federation of European Biochemical Societies doxorubicin. Thus, an optimised PAR2 inhibitor might be beneficial in combination therapy that minimises doxorubicin resistance, improves colon cancer treatment options, and potentially allows use of lower doses of doxorubicin for treating cancer.

### EACR2024-0495
### Aurora Kinase B inhibitors promote a hyper-polyploid state and continued endomitotic cycles in RB and p53 defective cells

*S. Vora[1], A. Andrew[1], R. Prassana Kumar[1], D. Nazareth[1], M. Fernando[1], M.J. Jones[2], Y. He[1], J.D. Hooper[1], J. Urosevic[3], B. Gabrielli[1]*
*[1]Mater Research Institute- The University of Queensland, Faculty of medicine, Brisbane, Australia*
*[2]Frazer Institute- The University of Queensland, Faculty of Medicine, Brisbane, Australia*
*[3]Research and Early Development, Oncology R&D- AstraZeneca, Cambridge, United Kingdom*

**Introduction**
Polyploid giant cancer cells have been reported to have increased chemotherapy resistance and to be a source of aggressive disease. Aurora kinase B inhibitors (AURKBi) are potent and efficient inducers of polyploidy in vitro and in vivo, but the fate of these AURKBi-induced polyploid cells has not been adequately investigated. Interestingly, although many AURKBi have been assessed in in vivo models and clinically trialed in patients for a range of cancers, there have been no reports of these drugs promoting more aggressive disease.

**Material and Methods**
We evaluated cellular proliferation and colony forming potential using a selection of RB and p53 wild type and RB+p53 defective cell lines and a range of AURKBi. Multiple markers of proliferation were assessed using high content and live cell timelapse imaging, and colony forming assays. Induction of polyploidy was quantified using DNA staining and timelapse microscopy. Centrosome numbers in cells undergoing mitosis were determined through immunofluorescent staining of γ-tubulin. In vivo tumour forming potential of polyploid cells was evaluated in nude mice.

**Results and Discussions**
We demonstrate AURKBi treatment of cells that have defective RB+p53 results in cells that become hyper-polyploid and undergo continuous rounds of replication and failed mitosis, whereas RB and p53 functional cells will eventually exit the cell cycle. These hyper-polyploid cells are viable and undergo continuous endomitotic cycles but lose the ability proliferate in vitro or form tumours in vivo. Investigation of mitosis in these cells revealed that centrosome duplication remained coupled to DNA replication, with hyper-polyploid cells containing high numbers of centrosomes that were each capable of supporting functional spindle poles. Time lapse imaging revealed occasional small colonies of polyploid cells, but these failed to form viable colonies with long term proliferative potential. However, when AURKBi was removed after 1 day and cells had failed a single cytokinesis and become tetraploid, they retained long term colony forming ability.

**Conclusion**
This work demonstrates that AURKB inhibition is a potent driver of polyploidy and the fate of these cells is dependent on the RB and p53 status of the cell. Hyper-polyploid cells do not form colonies in vitro and lack the ability to form tumours in vivo. Tetraploid cells can proliferate long term, indicating that the tetraploid state is well tolerated by cells, but higher ploidy states are incompatible with long term proliferative potential.

### EACR2024-0498
### Esculetin-Mediated Differentiation in Leukemic and Solid Tumor Cancer Stem Cells: A Promising Frontier in Differentiation Therapy Strategies

*A. Mathur[1]*
*[1]Institution of Eminence- University of Delhi, Delhi School of Public Health, Delhi, India*

**Introduction**
Acute Myeloid Leukemia (AML) is a heterogeneous condition with a verity of distinct genetic alterations resulting in a block of differentiation (maturation arrest). In extension to the revolutionising therapeutic outcomes of All Trans Retinoic Acid to induce terminal differentiation of Acute Promyelocytic Leukemic blast cells, we decipher the potential effect of a natural compound “Esculetin (Es)” to serve as a differentiating agent in AML. Underlaying role of Wnt signaling pathways in Es mediated blast cell differentiation was also evaluated.

**Material and Methods**
Human acute myeloid leukemic cells (Kasumi-1) with t(8;21/AML-ETO) translocation were used as a model system. Growth inhibitory and cytotoxic activity of Es were analysed using growth kinetics and MTT assay. Morphological alterations, cell scatter characteristics, NBT reduction assay and cell surface marker expression patterns were analysed to detect terminally differentiated phenotypes. We employed RT2profiler PCR array system for the analysis of transcriptome profile of Wnt signaling components. Calcium inhibitors and Transforming growth factor beta (TGF-β) were used to modulate the Wnt signaling axes. To investigate whether Es exerts the similar effects on the invasive solid tumour cancer stem cells subset, we used a cellular model system of colon carcinoma HCT116 cells reflecting EMT phenotype.

**Results and Discussions**
We illustrate cytotoxic as well as blast cell differentiation potential of Es on Kasumi-1 cells. Morphological alterations akin to neutrophilic differentiation as well as the corresponding acquisition of myeloid lineage markers indicate terminal differentiation potential of Esin leukemic blast cells. Es also suppressed canonical Wnt axis while upto ~ 21 fold upregulated non-canonical axis associated genes. Es was also found to attenuate the aggressive mesenchymal features and migration of colon carcinoma cells by reversing EMT phenotypes. Es also showed potential to revert the CSC marker expressions consistent with reduced functional CSC properties in colon cancer cells.

© 2024 The Authors. Molecular Oncology is published by John Wiley & Sons Ltd on behalf of Federation of European Biochemical Societies

**Conclusion**
Our study highlights the importance of selective use of calcium pools as well as "axis shift" of the canonical to non-canonical Wnt signaling upon esculetin treatment which might abrogate the inherent proliferation to release maturation arrest and induce the differentiation in leukemic blast cells. The current findings provide further therapeutic interventions to consider esculetin as a potent differentiating agent to counteract relapses.

### EACR2024-0505
### LaNt α31 Overexpression Inhibits Pancreatic Adenocarcinoma Formation

B. Sari[1], T. Zech[2], K. Hamill[3]
[1]*Institute of life course and medical sciences, Clinical Science, Liverpool, UK*
[2]*Institute of systems- molecular and integrative biology, Molecular & clinical cancer medicine, Liverpool, UK*
[3]*Institute of life course and medical sciences, Eye and Vision Science, Liverpool, UK*

**Introduction**
Pancreatic adenocarcinoma (PAAD) is an aggressive tumour type, therefore, identifying molecular mechanisms of tumour progression is essential to developing new treatments. Recent studies have demonstrated that high expression of laminin α3 (*LAMA3*), one of the 12 laminins, predicts patient survival outcome in PAAD. However, the *LAMA3* gene produces three structural and functionally distinct isoforms; *LAMA3A, LAMA3B* and *LAMA3LN1* (LaNt α31 protein), of particular note, LaNt α31 has never been investigated in PAAD.
**Material and Methods**
Transcript abundance of isoforms were detected in four PAAD cell lines by RT-qPCR. Stable Mia-PACA-2 cells expressing LaNt α31 (MiaLaNt) were generated using lentivirus; size and shape of WT and Mia-LaNt cells were compared. To detect the impact of matrix environments, WT and MiaLaNt cells were seeded onto LM332, collagen I, or the extracellular matrix (ECM) derived from WT or LaNt α31 overexpressing cells. To examine the effects of LaNt α31 on cancer progression, proliferation and migration assays were performed in 2D and in 3D using chorioallantoic membrane (CAM) chicken egg models. CAM tumours were embedded into wax, and stained with antibodies against proliferation and apoptosis related proteins.
**Results and Discussions**
Mia-PACA-2 cells displayed the lowest transcript levels of all three isoforms. This line was selected as a situation where LaNt α31 effects could be studied largely independent of isoforms. LaNt α31 overexpression in Mia-PACA-2, increased LAMA3B and decreased LAMA3A transcripts. It also increased cell spreading, decreased cell-cell contact with the cells exhibiting a wider and elongated cell shape. Moreover, it slowed cell proliferation and migration in 2D assays. WT and MiaLaNt appearance did not change on collagen I matrix or the ECM derived from the WT compared with uncoated dishes, but WT morphology changed on LM332 or the ECM derived from the MiaLaNt, and it looked like MiaLant cells. These suggested a matrix-driven response. In in vivo CAM model, WT formed large tumours surrounded by vessels, whereas MiaLaNt tumours were smaller or did not form at all. MiaLaNt differed in terms of expression of proliferation or apoptosis related proteins. Altogether, results showed that LaNt a31 overexpression inhibited cancer growth.
**Conclusion**
As LaNt α31 affects pancreatic cell behaviour, understanding the role of this new protein LaNt α31, the gene switch mechanism controlling it, and investigating the effects on cancer progression may help to lead to new treatments.

### EACR2024-0510
### Loss of STAT3 directs leukemic cell infiltration in acute myeloid leukemia

B. Zdársky[1], S. Edtmayer[1], A. Witalisz-Siepracka[1], S. Boigenzahn[1], S. Weiss[1], K. Heindl[1], S. Zahma[2], B. Győrffy[3], R. Moriggl[2], D. Stoiber[1]
[1]*Karl Landsteiner University of Health Sciences, Pharmacology, Krems an der Donau, Austria*
[2]*Paris London University of Salzburg, Department of Biosciences & Medical Biology, Salzburg, Austria*
[3]*Semmelweis University, Department of Bioinformatics, Budapest, Hungary*

**Introduction**
Acute myeloid leukemia (AML) is a highly heterogeneous disease resulting from abnormal proliferation of hematopoietic stem and progenitor cells. AML development is often accompanied by aberrant signal transducer and activator of transcription 3 (STAT3) signaling, which controls various cancer hallmarks and was found to act as both a tumor promoter or suppressor in various cancers. STAT3 inhibitors reached clinical trials for AML but have failed to provide therapeutic benefits. These diverse treatment outcomes are probably due to the heterogenicity underlying AML development and the capability of leukemic blasts to migrate to the hematopoietic tissues or extramedullary sites. A better understanding of the complex mechanisms involved in STAT3 signaling in AML development and the identification of predictive biomarkers is of significant necessity.
**Material and Methods**
To better determine the detailed role of STAT3 in AML development, we generated different CRISPR/Cas9-mediated STAT3 knockout (KO) human AML cell lines and characterized the cells via flow cytometry, Western blot and qPCR. In order to understand the impact of STAT3 loss on AML disease development, these cells were used for transplantation via tail vein into immuno-compromised NSG mice. In addition, we analyzed RNA-sequencing data sets and correlated gene expression to the overall survival of AML patients.
**Results and Discussions**
We show here that mice receiving STAT3-deficient MLL-AF9-driven AML cells exhibited decreased overall survival rates and increased infiltration of leukemic cells into the liver. Furthermore, STAT3 knockout led to the downregulation of common epithelial-mesenchymal transition (EMT) related genes but increased C-X-C chemokine receptor type 4 (CXCR4) and type 2 (CXCR2) expression. CXCR2 as well as CXCR4

© 2024 The Authors. Molecular Oncology is published by John Wiley & Sons Ltd on behalf of Federation of European Biochemical Societies expression correlated with worse overall survival in AML patients and negatively with STAT3 expression.

**Conclusion**

This study provides first data on an unexpected pro-tumorigenic effect after loss of STAT3 and describe a STAT3-dependent regulation of CXCR2 and CXCR4 in AML We revealed a novel function of STAT3 in regulating invasive migration in AML independent of EMT-gene expression and suggest STAT3 together with CXCR2/CXCR4 as prognostic markers to improve stratification of patients with AML.

### EACR2024-0516

### Hypoxia-dependent BCR/Abl loss in chronic myeloid leukemia cells is driven by extracellular vesicles release

*A. Biagioni[1], G. Versienti[1], N. Di Menna[1], W. Bieniek[2], G. Menegazzi[1], S. Peppicelli[1], P. Nardini[3], P. Dello Sbarba[1]*

*[1]University of Florence, Department of Experimental and Clinical Biomedical Sciences "Mario Serio", Florence, Italy*

*[2]Polish Academy of Sciences, Hirszfeld Institute of Immunology and Experimental Therapy- Department of Experimental Therapy, Wroclaw, Poland*

*[3]University of Florence, Department of Experimental and Clinical Medicine, Florence, Italy*

**Introduction**

Chronic Myeloid Leukemia (CML) is a myelo-proliferative disease driven by a singular oncogene, BCR/Abl, which encodes a constitutively active tyrosine kinase. Despite the efficiency of the tyrosine kinase inhibitors (TKi), such therapy does not cure CML. Indeed, the persistence of a subpopulation of TKi-resistant leukemia stem cells (LSCs) sustains the so-called minimal residual disease. The LSCs, along with normal hematopoietic stem cells, are believed to persist in bone marrow stem cell niches, which are sites characterized by severe reduction of oxygen and nutrients, and are likely genomically *BCR/Abl*-positive but oncoprotein-negative.

**Material and Methods**

CML cells were subjected to severe hypoxia (0.1% $O_2$) for 96 hours in order to downregulate the oncoprotein BCR/Abl expression. Extracellular Vesicles (EVs) were isolated from conditioned media via the ultra-centrifugation method and quantified through the Nanosight NS300, which also allowed us to analyze the size distribution of the particles. The isolated EVs morphology was visualized throughout the Transmission Electron Microscope (TEM) and their cargo was analyzed via Western Blot and droplet digital PCR, while the biological effect was evaluated by viability assay and quantitative PCR. The inhibition of EVs biogenesis and secretion was achieved by treating CML cells with Sulfisoxazole.

**Results and Discussions**

During the incubation in low oxygen conditions, mimicking in this way the stem cell niche micro-environment, we observed increased secretion of EVs compared to normoxia. Moreover, the EVs isolated in hypoxic conditions resulted loaded with high levels of BCR/Abl and were capable of transferring their cargo to *BCR/Abl*-negative cells. Upon uptake of hypoxia-induced EVs, these cells demonstrated to become sensitive to TKi, phosphorylate CrkL, and increase proliferation rate. By inhibiting EVs biogenesis and secretion with Sulfisoxazole, an endothelin receptor antagonist, we were able to maintain high levels of BCR/Abl oncoprotein in CML cells subjected to hypoxia, resulting thereby susceptible to TKi.

**Conclusion**

EVs are typically secreted by all cells but their biogenesis is commonly enhanced by stress signals such as metabolic limitations. We speculate that such a mechanism is exploited by LSCs to rapidly “get rid” of BCR/Abl expression facilitating entry into a quiescent status. Therefore, the inhibition of EVs biogenesis and release might prevent BCR/Abl loss thereby re-sensitizing to TKi.

### EACR2024-0522

### Cytotoxic and apoptotic effect of Tomatine against human oral cancer cell line(KB) by altering notch signaling pathways

*S. Kathiresan[1], N. Ahamed[2], T. Azhamuthu[2], P. Ravichandran[2], R. Vasu[1]*

*[1]Annamalai university, biochemistry and biotechnology, Chidambaram, India*

*[2]Annamalai university, Department of biochemistry and biotechnology, Chidambaram, India*

**Introduction**

Notch signaling is essential for cell proliferation, differentiation, development and homeostasis. Several cancers are associated with dysregulated notch signaling. Oral squamous cell carcinoma (OSCC) of the oral cavity is most prevalent among other cancers in which tumour growth and metastasis are brought on by abnormal Notch signaling pathway. Oral cancer's fatality and morbidity contributes to it being a global health burden. The intention of this research is to investigate the anticancer effects of α-tomatine on the cell growth and apoptosis in KB cells via Notch signaling pathway.

**Material and Methods**

we analyzed cytotoxic effect of Tomatine (MTT-Assay) by the generation of reactive oxygen species (ROS), the level of mitochondrial membrane potential (DYm), DNA damage and apoptotic morphological changes (AO/EtBr, and Hoechst staining). Further, apoptotic and notch signaling( Notch-1 Notch-2, Hes-1 and Hey-1) protein expressions were analyzed by western blotting techniques.

**Results and Discussions**

Tomatine has demonstrated encouraging outcomes in preclinical research as a cytotoxic agent towards several cancer cell lines in oncology, indicating its potential as a therapeutic tool in the treatment of cancer. Our results indicated that tomatine induces apoptosis as evidenced by loss of cell viability, enhanced ROS, and reduction of cellular glutathione levels resulting in depolarization of mitochondrial membrane potential, increased DNA damage in KB cells. Further more , tomatine enhanced the down regulation of notch signaling pathway proteins (Notch-1, Notch-2, Hes-1, Hay-1 and notch receptor

© 2024The Authors. Molecular Oncology is published by John Wiley & Sons Ltd on behalf of Federation of European Biochemical Societies binding proteins) by the effective dose of Tomatine treated with KB cell line

**Conclusion**

The overall results confirmed that Tomatine exhibit cell toxic effect, by inhibiting cell proliferation and modulating notch signaling pathways protein expression. Therefore, α-tomatine might be used as an effective therapeutic agent for the treatment of oral cancer .

### EACR2024-0524

### A new genetically engineered mouse model to study CRIPTO, a multifaceted orchestrator of lethal prostate cancer hallmarks

*E. Rodrigues Sousa[1], S.D. De Brot[2], A. Brunello[1], M. Scarpa[3], S. Zeinali[4], B. Kruithof[5], D. Freeman[6], B. Spike[6], P. Chouvardas[1], M. Kruithof-de Julio[7]*

*[1]Urology Research Laboratory- University of Bern, Department for Biomedical Research, Bern, Switzerland*

*[2]Institute of Animal Pathology, Department of Infectious Diseases and Pathobiology, Bern, Switzerland*

*[3]Translational Organoid Research- University of Bern, Department for BioMedical Research, Bern, Switzerland*

*[4]ARTORG Center- University of Bern, Organs-on-Chip Technologies Laboratory, Bern, Switzerland*

*[5]Leiden University Medical Center, Department of Cardiology, Leiden, The Netherlands*

*[6]Huntsman Cancer Institute, Department of Oncological Sciences, Salt Lake City, United States*

*[7]Urology Research Laboratory- University of Bern-University Hospital-Inselspital, Department for BioMedical Research, Bern, Switzerland*

**Introduction**

In early-stage prostate cancer (PCa), treatment involves surgery or androgen deprivation therapy. However, tumor can become castration-resistant, possibly due to pre-existing stem cell-like cells promoting tumorigenesis. High CRIPTO levels correlate with tumorigenesis and progression. To examine its role in PCa, we established new GEMMs with a prostate-specific, inducible CRIPTO knockout in Castration-Resistant Nkx3.1 expressing cells (CARNs), a subset of luminal cells prone to oncogenic transformation in PCa.

**Material and Methods**

We deleted CRIPTO (CRIPTO$^{flox/flox}$) in N (Nkx3.1$^{CreERT2}$, R26 $^{LSL-YFP/LSL-YFP}$), NP (Nkx3.1$^{CreERT2}$; Pten$^{flox/flox}$, R26 $^{LSL-YFP/LSL-YFP}$) and NPK (Nkx3.1$^{CreERT2}$; Pten$^{flox/flox}$; Kras$^{LSL-G12D/+}$, R26 $^{LSL-YFP/ LSL-YFP}$) resulting in NC, NPC and NPKC, respectively. N mice exhibit normal epithelium, while NP displays high-grade prostatic intraepithelial neoplasia/carcinoma lesions with localized invasive epithelium. NPK mice develop invasive prostate adenocarcinoma. In vivo experiments presented the following workflow: 8-week-old mice were castrated and induced with tamoxifen. Mice were treated weekly with testosterone. Single cells were isolated from tissue and the $^{YFP+/-}$ population was recovered by FACS sorting and cultured as organoids. Organoids$^{OECRIPTO}$ were obtained via lentiviral transduction.

**Results and Discussions**

CRIPTO knockout reduces invasive phenotype in NPK. Organoids$^{YFP+}$ recapitulate molecular features of matched tissue, forming multilayered structures. Morphological characterization showed distinct physical features: N organoids appear cystic with low densities, indicative of low-grade PIN phenotypes, NP and NPK organoids are small and dense, resembling an oncogenic transformation. Organoids$^{OECRIPTO}$ exhibits solid and dense characteristics, presenting a higher efficiency formation and a different extravasation potential. Transcriptomic analyses from mouse tissue and organoids revealed a unique CRIPTO/MYC co-activation signature. This signature was associated with PSA progression in human PCa TMA. Notably, CRIPTO IHC staining on human PCa TMA showed that high CRIPTO levels correlates with clinical and PSA progression.

**Conclusion**

We confirmed that selective CRIPTO depletion in CARNs in vivo decreases the invasiveness potential. In vitro CRIPTO manipulation revealed organoid structural changes that correlate with tumor progression. Our findings were further confirmed by examining PCa TMA, consistently indicating CRIPTO's role in both the onset and advancement of PCa.

### EACR2024-0557

### Multiomics analysis of inhibition of CIP2A-PP2A interaction in triple negative breast cancer cells

*P. Doan[1], N. Gupta[1], J. Westermarck[1,2]*

*[1]Turku Bioscience Centre, University of Turku, Turku, Finland*

*[2]Institute of Biomedicine, University of Turku, Turku, Finland*

**Introduction**

CIP2A (PP2A inhibitor) is associated with clinical aggressivity and promotes the malignant growth of triple negative breast cancer (TNBC). Recently, the inhibitory effect of CIP2A on PP2A-B56α has been identified as a crucial mechanism for TNBC tumorigenesis. Notably, a single point mutation in CIP2A (lysine 21, K21A mutation) disrupts B56α-CIP2A binding, leading to the abrogation of tumorigenicity in TNBC cells. However, the exact mechanism underlying tumorigenic abrogation in PP2A-reactivated cells remains unexplored. This study aims to identify effectors of PP2A reactivation in TNBC and elucidate the mechanism of tumorigenicity abrogation by the B56α binding-deficient CIP2A, offering potential insights for TNBC therapy development.

**Material and Methods**

To identify the key effectors of CIP2A K21A mutation that are critical for TNBC tumor growth, we employed a multiomics approach by combining RNA sequencing, proteome analysis, and phosphoproteome analysis.

**Results and Discussions**

Our multiomics data reveal significant transcriptional modulation in TNBC attributed to the K21A mutation, with 1202 Differentially Expressed Genes (DEGs) identified. Proteome data highlight 265 enriched proteins, and phosphoproteome data reveal 151 enriched phosphoproteins. Notably, the K21A mutation affected proteins related to NFE2L2 regulating tumorigenic genes which could explain tumorigenic abrogation in PP2A-reactivated cells.

Molecular Oncology 18 (Suppl. 1) (2024) 1–495

© 2024 The Authors. Molecular Oncology is published by John Wiley & Sons Ltd on behalf of Federation of European Biochemical Societies

**Conclusion**
The data underscores the substantial impact of the CIP2A K21A mutation in TNBC, affecting numerous genes, proteins, and phosphoproteins, resulting in profound alterations in multiple signaling pathways. In the future, utilizing CRISPR-Cas9 screens and functional analysis of identified targets from omics data could uncover critical effectors of CIP2A K21A mutation in TNBC tumor growth.

### EACR2024-0558
### Epithelial-mesenchymal transition (EMT) and cancer stem cells (CSC) markers coexpression in prostate cancer (PCa)

*M. Puchinskaya*[1]
[1]*Minsk City Clinical Oncologic Dispensary, Out-patient, Minsk, Belarus*

**Introduction**
EMT and CSC both were shown to be important for cancer metastasis and treatment resistance. The data on their interrelations are controversial and obtained mostly in experimental models. Relations of those phenotypes in clinical cancer samples are not enough studied. In the present work correlations between EMT and CSC markers expression and coexpression in clinical specimens of PCa were analyzed.

**Material and Methods**
56 PCa cases and 5 lymph node metastases were analyzed. E-cadherin (cad) and N-cad as EMT markers, CD44 and CD133 as CSC markers were stained using double immunofluorescence on two consecutive slides. Staining was analyzed semiquantitatively ad oculus, assessing staining intensity for both widely expressed E-cad and CD44, presence of CD133 or weighed staining index (WSI) for N-cad. N-cad and CD133 with very patchy expression were analyzed in the whole slide, while E-cad and CD44 – in N-cad+ and CD133+ high power fields (HPF, x400) and additionally 3-15 random non-crossing HPF per case. As no direct matching of HPFs evaluated for EMT and CSC markers was possible, mean numbers of staining characteristics were calculated for the whole case for further comparisons.

**Results and Discussions**
56 PCa cases and 5 lymph node metastases were analyzed. E-cadherin (cad) and N-cad as EMT markers, CD44 and CD133 as CSC markers were stained using double immunofluorescence on two consecutive slides. Staining was analyzed semiquantitatively ad oculus, assessing staining intensity for both widely expressed E-cad and CD44, presence of CD133 or weighed staining index (WSI) for N-cad. N-cad and CD133 with very patchy expression were analyzed in the whole slide, while E-cad and CD44 – in N-cad+ and CD133+ high power fields (HPF, x400) and additionally 3-15 random non-crossing HPF per case. As no direct matching of HPFs evaluated for EMT and CSC markers was possible, mean numbers of staining characteristics were calculated for the whole case for further comparisons.

**Conclusion**
No significant correlations were found between EMT and CSC markers expression in this clinical primary PCa cohort. This may point out that those processes are regulated separately and may play independent roles in cancer progression.

### EACR2024-0562
### Genetically-defined, syngeneic mouse organoid platform for understanding heterogeneity and response to therapy in liver cancer

*H. Wu*[1], *T.L. Wong*[1,2], *H. Yu*[1], *I.B. Huang*[1], *L. Zhou*[3], *K.H. Lam*[1], *M. Tong*[4], *A. Cheng*[4], *J. Wong*[1], *S. Ma*[1,2,5,6]
[1]*The University of Hong Kong, School of Biomedical Sciences- Li Ka Shing Faculty of Medicine, Hong Kong, Hong Kong-China*
[2]*The University of Hong Kong, State Key Laboratory of Liver Research, Hong Kong, Hong Kong-China*
[3]*The First Affiliated Hospital- Sun Yat-Sen University, Precision Medicine Institute, Guangdong, China*
[4]*The Chinese University of Hong Kong, School of Biomedical Sciences, Hong Kong, Hong Kong-China*
[5]*Health@InnoHK, Centre for Translational and Stem Cell Biology, Hong Kong, Hong Kong-China*
[6]*The University of Hong Kong - Shenzhen Hospital, Department of Clinical Oncology- Shenzhen Key Laboratory for Cancer Metastasis and Personalized Therapy, Shenzhen, China*

**Introduction**
Hepatocellular Carcinoma (HCC) is a disease with diverse cells and distinct molecular signatures, resulting in a complex and heterogeneous nature. The traditional 'one-size-for-all' approach to treatment has been inefficient or ineffective for many individuals. A major challenge in analyzing clinical samples is distinguishing driver from passenger mutations, leading to an incomplete understanding of the pathway dependency of specific driver mutations.

**Material and Methods**
This study utilized hydrodynamic tail vein injection to establish driver-specific HCC mouse models by delivering customized combinations of proto-oncogenes (sgPten/MYC, Δ90CTNNB1/MYC, sgTp53/MYC, sgAxin1/MYC, and Mcl1/MYC). Morphological and OMIC characterization was performed on the resulting mouse HCC organoids (mHCCOs), which were also used for high-throughput drug screening.

**Results and Discussions**
Whole-exome sequencing revealed clean mutation profiles in tumor tissues and mHCCOs. Immunostaining and transcriptome analysis demonstrated that both mouse HCC tissues and mHCCOs expressed hepatic-lineage markers HNF4α and AFP, but not the cholangiocyte-lineage marker CK19. Additionally, these models recapitulated human HCC subclasses, with distinct profiles present in each. Specifically, the Δ90CTNNB1, sgAxin1, and sgPten models displayed enriched cholesterol-related and fatty acid-related signatures compared to the sgTp53 models. High-throughput screening of mHCCOs with an FDA-approved anticancer library showed that the Δ90CTNNB1 model displayed higher sensitivity to lovastatin than sgTp53, with a higher potency for lovastatin in CTNNB1 mutant human HCC patient-derived organoids (PDOs) than in CTNNB1 wild-type PDOs. Lovastatin treatment specifically reduced the

© 2024 The Authors. Molecular Oncology is published by John Wiley & Sons Ltd on behalf of Federation of European Biochemical Societies tumor burden in the Δ90CTNNB1/MYC, but not in the sgTP53/MYC in vivo mouse model.

**Conclusion**

Our study not only involved the development and characterization of mouse HCC models and their corresponding organoids, but also utilized the mHCCOs platform to evaluate drug response heterogeneity. This scalable platform offers insights into the characteristics of cancer arising from specific driver mutations and has the potential to incorporate other functional aspects, such as therapeutic response, tumor heterogeneity, and micro-environment interaction.

### EACR2024-0565
### Investigating the relationship between KDEL Receptor 1 and Multiple Myeloma Pathogenesis

*Ş. Punar[1,2], B. Salman Yaylaz[1,2], H. Karaca[1,2], S. Sırma Ekmekci[1], N. Abacı[1]*

*[1]Istanbul University- Aziz Sancar Institute of Experimental Medicine, Genetics, Istanbul, Turkiye*

*[2]Istanbul University- Institute of Graduate Studies in Health Sciences, Genetics, Istanbul, Turkiye*

**Introduction**

Multiple myeloma (MM) typically produces excessive levels of paraproteins. The continuous paraprotein production exposes MM cells to constant endoplasmic reticulum (ER) stress. In our previous studies, we have shown that KDELR1 gene expression is increased in MM patients compared to control. KDELR1 encodes an integral membrane protein with seven transmembrane domains and acts as a receptor. It is involved in the packaging of chaperones with KDEL motifs into COPI vesicles. In this study, we aimed to examine the effect of suppressing KDELR1 gene expression on cell proliferation, apoptosis and ER stress genes in U266 and RPMI8226 cell lines.

**Material and Methods**

In our study, to understand the effect of KDELR1 in MM, gene expression was suppressed by siRNA in U266 and RPMI8226 cells. To investigate the effect of KDELR1 suppression on ER stress genes, expression changes of BiP, ATF-6, PERK and XBP-1 genes were determined by qRT-PCR. Protein level changes of KDELR1 and BiP were analyzed by western blot analysis. For apoptosis determination, control and siRNA-treated groups were visualized by confocal microscopy using fluorescent dye. Caspase3/7 activity in apoptotic cells was then determined by fluorescence imaging. The ratios of apoptotic and viable cells were calculated using ImageJ program. MTT assay was performed to determine the effect on cell proliferation.

**Results and Discussions**

KDELR1 gene was suppressed by 80% at 24 h and 74% at 48 h in U266 cells. In RPMI8226 cells, it was suppressed by 85% at 24 hours and 75% at 48 hours. Western blot analysis showed that BiP level decreased in KDELR1 suppressed groups. When the expression level of ER stress genes was analyzed in cells in which KDELR1 gene was suppressed, a decrease was detected especially in XBP-1 expression. In both cell lines, it was shown that the rate of apoptotic cells increased in the siRNA-treated cell group compared to the control group. Caspase 3/7 activity was detected in apoptotic cells. MTT assay showed that cell proliferation decreased by 62% in U266 cells and 37% in RPMI8226 cells, particularly at 24 hours.

**Conclusion**

In this study, we found that suppression of KDELR1 gene expression decreased the viability of MM cells and triggered apoptosis. Similar studies have shown that KDELR1 gene regulates cell homeostasis under cell stress conditions and has different activities in different cells. Our results reveal that KDELR1 has an important function in maintaining cell homeostasis in MM exposed to continuous ER stress.

### EACR2024-0575
### Expression of CD133 and CD44 as cancer stem cells (CSC) markers in clinical prostate cancer (PCa) samples

*M. Puchinskaya[1]*

*[1]Minsk City Clinical Oncologic Dispensary, Out-patient, Minsk, Belarus*

**Introduction**

CSC are thought to be a source of cancer recurrence, metastases and treatment resistance. Due to their quiescence, targeting them is challenging, as well as defining the cells with CSC features in tissues. CSC markers and their patterns of expression are varying in different cancers. In PCa, CD133 and CD44 were shown to be CSC markers in experimental models. In the current work patterns of CD133 and CD44 expression in clinical PCa samples were studied.

**Material and Methods**

Staining was performed using double immuno-fluorescence on radical prostatectomy specimens. 1 representative slide per case was selected for evaluation. Primary mouse anti-CD133 and rabbit anti-CD44 antibodies (Abs) were used, secondary goat Abs were labeled with AlexaFluor 488 and 555 dyes, respectively. Staining was assessed ad oculus semiquantitatively. CD133 expression was studied with x400 magnification in the whole slide with CD44 evaluation in CD133+ high power fields (HPF) and also in 3-15 random CD133- HPFs.

**Results and Discussions**

A total of 673 HPFs from 56 cases and 5 lymph node metastases were analyzed. CD133 was seen in only rare cells or glands per case on apical or lateral membranes, while CD44 was expressed rather diffusely, mostly on basolateral membranes, but with varying intensity, including high intratumoral heterogeneity in some cases. If both markers were present in the same cell/gland, their direct coexpression was seen. CD133 was present in 198 (29.4%) HPFs, corresponding to 76.7% of cases. Apical staining of the whole PCa gland was slightly less prevalent than lateral membrane staining in separate cells (41% vs. 59%). Mean CD44 staining intensity for the case was calculated. CD44 staining intensity appeared to be significantly higher in CD133+ HPFs (pMann-Whitney <0.0001), but not if CD133 positivity was assessed at the case level. However, as even within the same HPF CD133 was present in only some cells or glands, CD44 expression didn't differ significantly in those cells and adjacent CD133- cells.

© 2024 The Authors. Molecular Oncology is published by John Wiley & Sons Ltd on behalf of Federation of European Biochemical Societies

**Conclusion**
Significant correlation was found between both CSC markers expression in PCa at the level of HPF, but not the whole case. However, no special patterns of their coexpression in the cells/glands or HPFs could be derived from studied cases. Higher numbers of CD133+ cells or higher CD44 expression in primary PCa may be a predisposing factor for disease progression, but this needs further investigations.

### EACR2024-0583
### MAFG is a novel oncogene that rewires the tumor microenvironment to promote melanoma

*S. Ruiz[1], M. Martinez[2], F.A. Karreth[2], I. Ibanez de Caceres[1], O. Vera[3]*
*[1]IdiPAZ, Experimental Therapies and Biomarkers in Cancer, Madrid, Spain*
*[2]Moffitt Cancer Center, Molecular Oncology, Tampa, US*
*[3]Instituto de Investigación Sanitaria del Hospital la Paz- IdiPAZº, Experimental Therapies and Biomarkers in Cancer- INGEMM, Madrid, Spain*

**Introduction**
The small MAF family of transcription factors, consisting of MAFG, MAFF, and MAFK, dimerize with CNC/BACH proteins to regulate diverse transcriptional programs. Previously, we have shown that miRNA-29 suppresses melanoma development, at least in part, by repressing MAFG expression. Moreover, analysis of the TCGA revealed frequent amplification and/or over-expression of MAFG in melanoma. However, while MAFG has been proposed to have oncogenic potential in different tumor types, its role in melanoma is uncharacterized. Here, we investigated the role of MAFG in melanomagenesis.

**Material and Methods**
We used human immortalized melanocytes and melanoma cell lines to modulate MAFG expression by overexpression vectors. We performed cell biological assays to determine the role of MAFG in melanoma and its oncogenic potential. Additionally, we used RNA-sequencing and bioinformatic analysis to identify down-stream effectors of MAFG responsible for the observed phenotypes. Epigenetic reactivation treatment and qRT-PCR was used to characterize the regulation of the identified effectors.

**Results and Discussions**
We found that MAFG overexpression significantly enhanced proliferation and focus formation in both human melanocytes and melanoma cell lines. RNA-sequencing revealed that MAFG regulates transcriptional programs associated with the extracellular matrix (ECM) and immune responses. MAFG-overexpressing cells exhibited increased expression and secretion of cytokines. Furthermore, we discovered that MAFG epigenetically modulates several genes involved in extracellular matrix reorganization.

**Conclusion**
Collectively, our study demonstrates that MAFG plays a pivotal role in melanomagenesis. By regulating chemokine and ECM expression genes, MAFG potentially reshapes the tumor immune micro-environment. Our results are promising, and future studies will address if MAFG can be used as a therapeutic target.

### EACR2024-0590
### Expression of ITGB1 and other epithelial-mesenchymal transition factors modulate the biological and clinical behavior of Non-Small Cell Lung Carcinoma

*C. Machado Baldavira[1], T. Gutierrez Prieto[1], C. Silvério Faria[1], A. Ab'Saber[1], T. Takagaki[2], V. Luiza Capelozzi[1]*
*[1]Faculty of Medicine of University of Sao Paulo, Pathology, Sao Paulo, Brazil*
*[2]Instituto do Coração Incor- University of Sao Paulo, Pneumology Division, Sao Paulo, Brazil*

**Introduction**
Non-small cell lung carcinoma (NSCLC) is a leading cause of morbidity and mortality globally. The most common histological types are adenocarcinomas (ADC) and squamous cell carcinomas (SCC). The spread of NSCLC, both through local advancement and distant metastasis, has been linked to various mechanisms, especially to the process known as epithelial-mesenchymal transition (EMT). In this study, we explored whether the expression of factors involved in EMT, such as desmoplakin (DSP), integrin β1 (ITGB1), osteopontin (SPP1), and vimentin (VIM), influences the biological behavior of NSCLC.

**Material and Methods**
The expression of DSP, SPP1, ITGB1, and VIM proteins was detected in tumors from a cohort of 62 patients diagnosed with NSCLC, including 54 patients with ADC and 8 patients with SCC, all at early clinical stages (I to IIIA). This was achieved using immunohistochemical staining, with quantification analyzed through QuPath software. High and low expression were defined based on the median.

**Results and Discussions**
In our cohort, comprising both ADC and SCC cases, we observed high stromal expression of VIM (median: 21.74% of positive cells), contrasted with low expression levels of DSP (median: 0.22% of positive cells), SPP1 (median: 2.11% of positive cells), and ITGB1 (median: 2.73% of positive cells). Notably, a strong correlation was found between SPP1 and ITGB1 expression ($\rho=0.792$, $P<0.001$). There was also a moderate correlation between SPP1 and VIM ($\rho=0.344$, $P=0.006$), and a moderate inverse correlation between DSP and ITGB1 ($\rho=-0.357$, $P=0.004$). Additionally, high DSP expression was significantly associated with SqCC cases ($P=0.001$). High SPP1 expression correlated with early tumor stages T1 and T2 ($P=0.012$) and tumors smaller than or equal to 3.4 cm in diameter ($P=0.019$). In terms of survival, Cox multivariate analysis revealed that lower expression levels of ITGB1 were associated with an increased risk of death (HR: 3.96, $P=0.03$), with tumor size and stage included as covariates to refine the model. Our findings suggest that in the emergent scenario, exploring the ETM factors as biomarkers during the diagnosis can be helpful for the selection of patients for personalized treatments.

**Conclusion**

© 2024 The Authors. Molecular Oncology is published by John Wiley & Sons Ltd on behalf of Federation of European Biochemical Societies

In conclusion, the expression of EMT factors, DSP, SPP1, ITGB1, and VIM, significantly influences the biological and clinical outcomes of NSCLC, positioning them as promising targets for personalized therapy.

### EACR2024-0591
### Key role of Galectin-3 in activating dissemination and dormancy program of highly aggressive papillary thyroid cancer cells

C. Modica[1], V.D. pantina[2], C. D' accardo[3], N. Roozafzay[3], S. Di Franco[4], M. Gaggianesi[4], A. Turdo[3], F. Verona[3], M. Todaro[3], G. Stassi[4]
[1]university of palermo, Dichirons, Palermo, Italy
[2]university of palermo, Dichirons, MePreCC, Italy
[3]university of palermo, Promise, palermo, Italy
[4]University of Palermo, MePreCC, Palermo, Italy

**Introduction**
Galectin-3 (Gal-3) expression is associated with the malignant phenotype of papillary thyroid carcinoma (PTCs) whereas neither healthy thyroid tissue nor benign thyroid adenomas express a detectable level of the protein. The distribution of Gal-3 between the cytoplasm and nucleus is notably different in rapidly proliferating human PTC cells, suggesting its potential role in the regulation of the cell cycle. These interactions underscore the multifaceted role of Gal-3 in cancer biology, influencing processes such as cell adhesion, proliferation, apoptosis, and metastasis. Gal-3 expression in TC cells contributes to disease progression, marked by the acquisition of mesenchymal features (EMT). Recently EMT was associated with the acquisition of plasticity and dormancy in metastatic cells, a phenomenon referred to as EMT-Associated Dormancy.

**Material and Methods**
Thyroid progenitor cells (TPCs) obtained from human embryonic stem cells were engineered using a CRISPR/Cas9 technology in BRAF or TP53 genes. Single mutated TPCs or double mutated generate tumors that recapitulate respectively human PTC or anaplatistic thyroid cancer (ATC). Knockdown or overexpressed Gal-3 cells underwent to a biochemical and biological evaluation for dormancy genes, proliferation rate, invasion ability. In vitro data were validated in vivo in orthotopic models rigorously analysing primary tumor and lung metastatis derived tissues.

**Results and Discussions**
Immunofluorescence analysis of primary and secondary PTC tumor xenografts shows an abundance of Gal-3 positive cells in metastatic sites. Suppression of Gal-3 results in a decrement expression of dormancy-related genes, whereas overexpression enhances these genes along with SOX9 accumulation in the nuclei and improved invasive properties. Knockdown of Gal-3 activates ERK, reduces p27 levels, and prompts cell cycle exit, while overexpression promotes p38 phosphorylation and G0-G1 arrest. Consistently, Gal-3 knockdown leads to the development of large tumors and distant metastases, while overexpression results in smaller tumors and the presence of isolated disseminated cells or low-proliferating clusters in the lungs.

**Conclusion**
Our findings suggest that TC cells, under the influence of Gal-3, activate a dormant dissemination program. Gal-3 acts as a critical link between the dissemination machinery and dormancy state in PTC, highlighting its potential role as a therapeutic target to counteract TC progression and metastasis formation.

### EACR2024-0594
### Deciphering Thyroid Metastasis Initiating Cells via Barcode Clonal Tracking

V.D. pantina[1], C. Modica[2], S. Di Franco[2], F. Verona[3], T. Porcelli[3], N. Roozafzay[3], K. Shams[3], M. Todaro[3], G. Stassi[2]
[1]University of Palermo, Dichirons, Palermo, Italy
[2]university of palermo, MePreCC, palermo, Italy
[3]university of palermo, Promise, palermo, Italy

**Introduction**
Thyroid carcinoma (TC) is the most common malignancy of endocrine organs. Especially anaplastic thyroid cancer (ATC) has an invasive and highly metastatic phenotype, representing a major subject of oncology research. Despite significant advances in the comprehension TC's hallmarks and biological behavior, the cell subpopulation hierarchy responsible for tumorigenesis, promotion and progression of the disease is still unclear.

**Material and Methods**
The data were obtained using BRAF/TP53 or NRAS/TP53 mutated thyroid progenitor cells (TPCs). Barcoded double mutated TPCs generate tumors that recapitulate human ATC. For qualitative and quantitative assessment of clonal composition, primary tumors obtained at several in vivo passages underwent to DNA extraction and targeted-NGS analysis to identify the profile of each enriched barcode. RNAseq analyses were conducted on samples derived from the in vivo passages and Gene Set Enrichment Analysis (GSEA) allowed to detect the enriched pathways. Cells derived from the P1-P3 in vivo passages underwent to in vitro assays to determine the proliferation rate, invasion ability, cell plasticity and metabolic assessment of tumor cells.

**Results and Discussions**
We generated an orthotopic model by the injection of double-mutated TPCs permanently transduced with a barcode library. Barcoded engrafted tumor has been consecutively transplanted to establish serial passages. P3-derived cells retain high invasion capacity compared to P1 together with high mesenchymal markers expression. Qualitative and quantitative analysis of clonal distribution revealed, after the first passage in vivo, a drastic reduction in the number of observed barcodes. Tumors derived from PDX 1 underwent a drammatic reduction in barcode composition and a further decrease across serial passages. Notably, variable clonal dynamics were observed across in vivo passages: the majority of clones were either initially exhausted, while others were initially undetected or minimally represented but became enriched across passages. RNAseq analysis revealed a metabolic switch along the in vivo passages that could be associated with the enriched cell clones.

**Conclusion**
The generation of barcoded TPCs in vitro and the establishment of progressive PDX passages will allow us to identify the cellular subclones mainly involved in

© 2024 The Authors. Molecular Oncology is published by John Wiley & Sons Ltd on behalf of Federation of European Biochemical Societies thyroid tumor initiation, promotion, and progression. Our data identified a subpopulation of cells undetectable or rarely represented in primary tumor play a critical role in tumor promotion and progression.

### EACR2024-0610
### SPINT2 reduces melanoma aggressiveness and improves therapeutic response

*S. Pires Celeiro[1,2], R. Dias Oliveira[1,2], V. de Lima Vazquez[3,4], F. Baltazar[1,2], R. Manuel Reis[1,2,3], M. Viana Pereira[1,2,5]*
*[1]Life and Health Sciences Research Institute ICVS, School of Medicine- University of Minho- Campus of Gualtar, Braga, Portugal*
*[2]ICVS/3B's-PT, Government Associate Laboratory, Guimarães- Braga, Portugal*
*[3]Molecular Oncology Research Center, Barretos Cancer Hospital, Barretos, Brazil*
*[4]Melanoma Sarcoma and Mesenchymal Tumors Surgery Department, Barretos Cancer Hospital, Barretos, Brazil*
*[5]Department of Oncology, Hospital de Braga, Braga, Portugal*

**Introduction**
Melanoma is the most fatal and aggressive form of skin cancer, and its incidence has been continuously raising. Identifying suitable biomarkers, to improve diagnosis, prognosis and an efficient selection of personalized therapies for melanoma patients is crucial. Approximately 50% of melanoma cases present *BRAF* mutations leading to a higher aggressiveness, brain metastatic capacity and shorter survival. BRAF inhibitors improved melanoma survival, however resistance mechanisms lead to therapeutic failure. Hepatocyte growth factor (HGF) secreted levels seem to be related to BRAF inhibitors' resistance. Serine protease inhibitor Kunitz-type-2 (SPINT2) inactivates serine proteases responsible for pro-HGF conversion into its active form. SPINT2 has been identified as a tumor suppressor in various solid tumors. However, little is known about SPINT2 in melanoma. Therefore, our work aim is to understand the role and therapeutic influence of SPINT2 in melanoma.

**Material and Methods**
733 melanoma samples were used to evaluate SPINT2 expression and 53 to evaluate methylation status and perform the clinicopathological correlations. SPINT2 overexpressed transfectants in melanoma cell lines (A375; WM9) were obtained and SPINT2 functional role evaluated through 2D and 3D melanoma cell culture models (CCM). SPINT2 impact on ex vivo tumor growth and angiogenesis was assessed by Chick Chorioallantoic Membrane (CAM) assay. Nanostring (PanCancer progression panel) and proteome profiler (Human XL oncology) were used to study SPINT2 biological role in melanoma. SPINT2 influence on melanoma cells sensitivity against the standard BRAF and MEK targeted therapies and the respective cumulative effect on melanoma aggressiveness (tumor spheroid growth and migration) were evaluated.

**Results and Discussions**
SPINT2 seems to be downregulated in melanoma by promoter hypermethylation. Overexpression of SPINT2 decreased cell viability, migration, and proliferation capacity in 2D and 3D CCM, as well as decreased tumor growth and angiogenesis capacity on CAM assay. Moreover, SPINT2 influenced the expression of genes and proteins related with extracellular matrix degradation, angiogenesis, and cell migration capacity. Finally, SPINT2 overexpression increased melanoma sensitivity to BRAF and MEK inhibitors, showing a cumulative effect on 3D spheroid growth and migratory capacity.

**Conclusion**
SPINT2 absence is related to melanoma aggressiveness and shows a great potential as a therapeutic biomarker for *BRAF*-mutated melanoma patients.

### EACR2024-0614
### Investigating the potential of BACH1 as an anti-metastatic target in lung cancer

*D. Klenja[1], M. Higgins[1], D. Walker[1], L. de la Vega[1]*
*[1]University of Dundee, School of Medicine- Jacqui Wood Cancer Centre, Dundee, United Kingdom*

**Introduction**
Lung cancer is the leading cause of cancer-related deaths worldwide. Metastasis, the spread of cancer cells to secondary tissues and organs, accounts for 90% of these cancer deaths. Nevertheless, we still lack effective therapies targeting metastasis. Therefore, a better understanding of the mechanisms underlying the spread of cancer is crucial. The transcription factor BACH1 is considered an important pro-metastatic factor in lung cancer, however, it is not clear how BACH1 drives metastasis and the specific target genes involved. Additionally, there are no approved BACH1 inhibitors in the clinic.

**Material and Methods**
To identify direct BACH1 target genes in lung cancer cells, we performed RNA and ChIP sequencing in the lung adenocarcinoma cell line A549. We then validated these target genes in other cell lines and assessed their potential role in BACH1-mediated pro-metastatic effect by studying their function in lung cancer cell migration. Furthermore, given the therapeutic promise of BACH1 inhibition in treating metastasis, we performed a high-throughput drug screen in lung cancer cells to identify potential BACH1 inhibitors.

**Results and Discussions**
We validated seven genes from the RNA and ChIP sequencing results as highly regulated direct BACH1 target genes. We also demonstrated that modulation of two of these target genes significantly impacts on lung cancer cell migration. Moreover, four compounds from the high-throughput drug screen were validated as BACH1 inhibitors and two of these compounds significantly reduced cell migration in various lung cancer cell lines in vitro.

**Conclusion**
Overall, our study suggests a possible mechanism of BACH1-driven metastasis. Furthermore, we identify novel BACH1 inhibitors and confirm the potential of BACH1 inhibition to reduce lung cancer metastasis.

© 2024 The Authors. Molecular Oncology is published by John Wiley & Sons Ltd on behalf of Federation of European Biochemical Societies

### EACR2024-0616
### Exploring the metabolic profiling of Multiple Myeloma: implications for targeted therapies

L. Di Martino[1], S. Gilmore[2], Y. Wang[2], S. Glavey[3], T. Ni Chonghaile[2]
[1]Royal College of Surgeons of Ireland, Physiology & Medical Physics, Dublin, Ireland
[2]Royal College of Surgeons of Ireland, Physiology and Medical Physics, Dublin, Ireland
[3]Royal College of Surgeons of Ireland, Department of Haematology- Beaumont Hospital, Dublin, Ireland

**Introduction**

Multiple myeloma (MM) is a haematological cancer marked by abnormal plasma cell growth in the bone marrow (BM). Despite therapeutic advancements, managing MM remains challenging due to its heterogeneity, recurrence, and treatment resistance. Metabolic processes and cell death mechanisms are pivotal in MM progression, sustaining MM cell survival in the BM milieu. Our primary goal is to understand the interplay between these factors, aiming to develop innovative therapies. To achieve this, we have delineated the following objectives: employ cutting-edge technology capable of dissecting metabolic pathways at the single-cell level in primary MM samples; implementing a co-culture model involving bone marrow fibroblasts to manipulate specific parameters and evaluate their influence on MM cell metabolism and apoptosis.

**Material and Methods**

SCENITH profiling was performed to asses at single cell level the metabolic profile of MM primary samples. This innovative approach uses protein translation as a functional read-out of metabolism following 2-deoxy-D-glucose (2DG) treatment to inhibit glycolysis or oligomycin to inhibit OXPHOS. We investigated the impact of BM microenvironment on the metabolic profile of MM by co-culturing MM cell lines (SK-MM-2, MM1S, H929) with bone marrow fibroblasts and we measured the oxygen consumption rate with the Seahorse XFe96 assay.

**Results and Discussions**

Utilising the SCENITH technology on plasma cells from MM patients, we were able to optimise a rapid metabolic profiling ex vivo. The results showed that primary samples reliant on BCL-2 for survival are more sensitive to oligomycin inhibition leading us to hypothesize a possible link between oxidative phosphorylation (OXPHOS) and BCL-2 dependency within MM cells. Furthermore, our in vitro preliminary observations demonstrate diverse metabolic characteristics among different types of MM cell lines (SK-MM-2, MM1S and H929). Specifically, within the bone marrow environment, we observe that cells sensitive to BCL-2 inhibition display heightened glycolytic ability and sustain mitochondrial function to a greater extent compared to their resistant counterparts. Moving forward, we plan to delve deeper into the anti-apoptotic needs of these cells and clarify the mechanistic pathways linking increased metabolic demands to BCL-2 dependencies.

**Conclusion**

Conclusively, we aim to enrich our comprehension of the molecular mechanisms underlying venetoclax sensitivity and potentially reveal new therapeutic targets for multiple myeloma patients.

### EACR2024-0626
### Meta-Analysis of 62 Cancer Expression Studies Including Potassium Channel (KCN) Genes Among Genes of Interest Reveals 4 KCN Groups in 85% of Studies and Highlights KCNMA1 and KCNN4 Genes

M. Beckner[1]
[1]Kent State University, Brain Health Research Institute, Willoughby Hills, United States

**Introduction**

The potassium channel (KCN) family of proteins is large (≥80), highly interactive, and influenced by unknown proteins which hinders analysis. Importantly, as a readily available, small, monovalent cation, K+ is the likely candidate (versus Na+ when both are hydrated) to displace protons from fixed anions of cell membranes to free H+ for diffusion in cytosolic water for exiting cells with lactate during pH reversal. Earlier, published expression studies with no bias to include KCN genes, E1 - E23, yielded results in various malignancies that showed KCN multiplicity, interactivity, and pH sensitivity in gene landscapes (Beckner, Proc AACR, A6034, 2023).

**Material and Methods**

Continued meta-analysis of studies with no initiating KCN bias, but with results that yielded KCN genes among altered genes of interest (GOI) in malignant expression landscapes, was performed to detect KCNs that potentially aid K+ flux in cancer and their relevant non-KCN GOI. Heatmaps, Venn diagrams, tables, etc. were analyzed. Count data and correlation were used.

**Results and Discussions**

In E1 - E62 studies of 26 types of malignancies, the average numbers of KCNs and all GOI in 62 studies were 1.56 (± 1.34 SD) and 43.24 (± 28.27 SD), respectively. Groups of (1) pH sensitive (*KCNK* subfamily, *KCNJ16*, etc.). (2) calcium sensitive (*KCNM* and *KCNN* subfamilies), (3) regulatory, and (4) *KCNQ* subfamily members were identified in 26 (41.9%), 22 (35.5%), 20 (32.3%), and 10 (16.1%) of studies, respectively, with at least 1 KCN from any of the 4 groups in 53 (85.5%) of studies. Among 97 instances of altered 48 KCN gene loci transcripts, there were 67 (69.1%) instances of repeats, with 24, 18, 12, 6, and 7 occurring due to 2X, 3X, 4X, 6X, and 7X repetitions, respectively. *KCNJ16*, *KCNK1*, and *KCNN3* were in 4 studies each. *KCNMA1* and *KCNN4* were each in 6 and 7 studies, respectively. There was no correlation between the number of KCN gene transcripts found as significantly altered and the size of the GOI landscape, $p = 0.421$. Among the 2,628 instances of non-KCN companion GOI, the instances of repetition (2X - 5X) were 793 (30.18%). *FGFR3*, *ITGA2*, and *PLAUR* were each found in 5 studies. *ATP1A2* (Na+/K+ ATPase) and *AQP3* (aquaporin 3) were each found in 4 studies.

**Conclusion**

Malignant gene expression landscapes suggest a role for altered (increased/decreased, diffuse/localized) plasma

Molecular Oncology 18 (Suppl. 1) (2024) 1–495
© 2024 The Authors. Molecular Oncology is published by John Wiley & Sons Ltd on behalf of Federation of European Biochemical Societies membrane K+ channeling in K+ replacement of H+ on membrane fixed anions to enhance H+ efflux in malignant pH reversal with an emerging gene signature of participants.

### EACR2024-0627
### Targeting MSI1-S347 Phosphorylation: Decoy Peptide Suppression of GBM Invasion and Tumor Formation

X. Liu[1], L.T. Lin[1]
[1]*Hong Kong Polytechnic University, health technology and informatics, Hongkong, Hong Kong-China*

**Introduction**
Glioblastoma (GBM) is one of the most aggressive brain cancers, known for its high recurrence and the paucity of effective treatments. The RNA-binding protein Musashi-1 (MSI1) is implicated in the aggressive nature of GBM. However, direct suppression of MSI1 is problematic due to its critical role in normal physiology. Therefore, discovering a strategy to therapeutically target MSI1 is essential for advancing GBM treatment and enhancing patient prognosis.
**Material and Methods**
This study incorporated three GBM cell lines—U87-MG, 05MG, and S1R1—for in vitro analysis and an orthotopic GBM model using immunodeficient BALB/c nude mice for in vivo experiments. We utilized Phos-Tag and Western blot analysis with a specially designed antibody to measure MSI1 phosphorylation levels. The study applied wound healing and transwell assays to examine cellular migration and invasion, respectively. We employed colony formation and three-dimensional cell culture techniques to assess cellular tumorigenic potential. Cells were exposed to peptide treatment through plasmid transfection.
**Results and Discussions**
We discovered a new phosphorylation site at serine-347 (S347) on MSI1. Our data indicate that phosphorylation at S347 leads to a marked rise in GBM migratory and invasive potential, accompanied by distinct cytoskeletal formations. Furthermore, MSI-S347 phosphorylation significantly boosts the cells' capacity to initiate tumors both in cells and animal models, in conjunction with an upsurge in CD133 (Prominin-1), a marker of tumor-initiating cells. Thus, MSI1-S347 phosphorylation is critical for enhancing the invasive and tumor-initiating properties of GBM. After screening, we discovered that a peptide designed to mimic the MSI1-S347 site was able to decrease the natural phosphorylation of S347 by acting as a competitive inhibitor. Treatment with this decoy peptide led to a decrease in cellular invasion and alterations to the cytoskeletal integrity. Additionally, applying the decoy peptide to both GBM cells and a mouse model resulted in a reduction of their tumor-forming potential. In conclusion, mitigating MSI1's cancer-promoting activities by targeting the S347 phosphorylation site reduces the metastatic propensities of GBM cells.
**Conclusion**
This research elucidates the significant connection between GBM metastasis and newly cancer-promoting phosphorylation at MSI1-S347. The use of an MSI1-S347 mimicking peptide was found to attenuate the protein's tumor-enhancing activity, leading to a decrease in GBM spread.

### EACR2024-0635
### Involvement of ASAP3 in Platinum Drug Resistance in High-Grade Serous Type Ovarian Cancer

J. Sheu[1], Y.Y. Zheng[1], T. Hwang[1], C. Chang[2]
[1]*National Sun Yatsen University, Institute of Biomedical Sciences, Kaohsiung, Taiwan*
[2]*National Sun Yatsen University, Department of Applied Mathematics, Kaohsiung, Taiwan*

**Introduction**
We have previously established a platform HDMAC (high-dimensional analysis of molecular alterations in cancer) to discover novel cancer-associated genes. Based on HDMAC, we identified the correlation of ASAP3 levels with poor responses to platinum treatments in high-grade serous ovarian cancer (HGSOC). This study aims to define molecular mechanisms mediated by ASAP3 in the development of drug resistance.
**Material and Methods**
Gene knockdown against ASAP3 was performed in OVCAR3 cells, a cell line known to be resistant toward platinum treatment. Gene segment of ASAP3 was cloned and introduced to CAOV3 and IOSE cells, two cell lines with low/undetectable ASAP3 levels. Drug sensitivity of those treated cells were tested and compared with the ones from vector control cells. Drug resistant cell clones were generated in CAOV3 cells by treating them with gradually increased concentrations of cisplatin and carboplatin. The key functional networks governed by ASAP3 were further verified by RNA-seq transcriptome analysis and cell-based studies.
**Results and Discussions**
ASAP3 knockdown triggered significant cell death in OVCAR3 cells. Once overexpressed, ASAP3 can reduce drug sensitivity toward cisplatin and carboplatin in CAOV3 and IOSE cells. On the other hand, cisplatin and carboplatin resistant clones generated in CAOV3 cells expressed higher ASAP3 levels. Interestingly, cells expressing ASAP3 represented the phenotype of multinucleation, commonly found in platinum drug resistant cells. The resultant cells also showed increased migration and invasion abilities as compared to vector controls. Downstream pathway analyses revealed active ECM remodeling, cell adhesion/migration, and cytoskeleton remodleing mediated by over-expressing collagen proteins. More studies will be conducted to investigate the functional impacts of such alterations on anti-apoptosis processes and cancer aggressiveness.
**Conclusion**
ASAP3 elevation boosts drug resistance against platinum-based chemotherapy in HGSOC, suggesting it as a crucial therapeutic target for clinical research and new drug/treatment development.

### EACR2024-0640
### Fatty liver-derived macrophage migration inhibitory factor facilitates liver metastasis of pancreatic ductal

© 2024 The Authors. Molecular Oncology is published by John Wiley & Sons Ltd on behalf of Federation of European Biochemical Societies

### adenocarcinoma

Q. Yu[1], H. Song[1], X.Y. Shi[1], Y. Liang[1], H.Z. Wang[1]
[1]The Affiliated Hospital of Qingdao University, Tumor Immunology and Cytotherapy, Qingdao, China

**Introduction**

How pathological livers shape tumors, thereby driving pancreatic ductal adenocarcinoma (PDAC) metastasis to the liver, is poorly understood. The globally rising rates of non-alcoholic fatty liver disease (NAFLD) is likely to contribute to an increase in the incidence of liver metastasis. The mechanisms by which a fatty liver mediates and enhances PDAC liver metastasis are poorly known. In the present study, we focus on examining key molecules implicated in this process and assessing their translational significance.

**Material and Methods**

Non-contrast and contrast enhanced computed tomography (CT) were used to assess fatty liver of PDAC patients of The Affiliated Hospital of Qingdao University. Diet-induced NAFLD model was generated by feeding C57/B6 mice with a choline-deficient L-amino acid-defined, high-fat diet (CDAA-HFD) for 4 weeks, followed by implantation of LSL-KrasG12D/+; LSL-Trp53R172H/+; Pdx-1-Cre (KPC) cells into the spleen to construct a preclinical, murine pancreatic tumor model of hepatic metastases model. Multiplex immuno-histochemistry (mIHC) was performed on murine and human liver metastasis tissues. Hepatic macrophage migration inhibitory factor (MIF) knockdown and overexpression were achieved through in vivo AAV vector treatment. Mouse liver metastases organoid culture followed by sphere formation assay was applied in this study to examine the tumor cell stemness. Transmigration of KPC cells toward MIF gradient was assessed in vitro.

**Results and Discussions**

We show that in pancreatic patients and mouse models, NAFLD enhanced pancreatic cancer cell stemness within the liver, and then we screen MIF and its receptor CD44 as important factors using single-cell sequencing analysis. After we confirm the expression and distribution of MIF and CD44 in PDAC metastatic samples from patients and mouse models, we find that NAFLD-induced MIF mediated the progression of PDAC liver metastasis by attracting CD44 positive pancreatic cells, and thereby enhancing cancer stemness and focal adhesion. Targeting the MIF-CD44 axis by either a MIF tautomerase inhibitor, IPG1576, or by CD44 knockdown in tumor cells significantly attenuate liver metastasis of PDAC within the NAFLD context, which was associated with decreased cancer cell stemness and focal adhesion

**Conclusion**

Collectively, our study highlights a pivotal role for MIF-CD44 in cancer stemness and offer novel avenues for tailoring therapeutic strategies to individual patients with NAFLD as an underlying condition.

### EACR2024-0643
### IL-17A modulates metabolic reprogramming in Cutaneous T-cell lymphoma (CTCL) via Mitochondrial complex I

D. Attrish[1], B. Dhamija[1], D. Mukherjee[1], V. Sawant[1], S. Marathe[1], N. Sharma[2], T. Shet[2], H. Jain[2], R. Purwar[1]
[1]Indian Institute of Technology Bombay, Biosciences and Bioengineering, Mumbai, India
[2]Tata Memorial Hospital, Medical oncology, Mumbai, India

**Introduction**

CTCL is a malignancy of skin resident T cells. As the disease advances, there's a noticeable increase in Th2 cytokines, Th17 (IL-17), and regulatory T cells. Several studies have observed an elevated expression of IL-17A, and correlated increased levels with an aggressive form of the disease. Despite knowing that IL-17A is abundant in the CTCL microenvironment, we still lack understanding about how exactly it contributes to the tumor cell biology, particularly tumor metabolism.

**Material and Methods**

As IL-17A is known to regulate mitochondrial metabolism in many disorders so, we examined IL-17RA and MTCO1 (subunit of mitochondrial complex IV involved in oxidative phosphorylation (OxPhos) bioenergetics) expression levels in ctcl skin biopsy using immunofluorescence. Further, to understand how IL-17A signaling modulates mitochondrial metabolism we used a comprehensive multi-omics study and studied dysregulated proteins using flow cytometry. Biochemical assays elucidated the mechanistic role of IL-17A signaling. Pharmacological inhibition studies revealed the dependency of lymphoma cells on OxPhos.

**Results and Discussions**

Elevated IL-17RA (1.9-fold, p=0.0367) and MTCO-1(2.2-fold, p=0.0008) expression were observed in skin tumor biopsy associating IL-17A signaling with mitochondrial dysregulation. Further, multi-omics study identified tricarboxylic acid (TCA) cycle anaplerosis and OxPhos biogenesis as the primary metabolic changes due to upregulated mitochondrial complex I activity induced by IL-17A. Flow cytometry analysis confirmed the upregulated levels of complex I (1.8-fold, p= 0.0127) in response to IL-17A treatment. Next, this finding was corroborated clinically as high expression of complex I protein (1.44-fold, p=0.0175) was observed in blood of CTCL patients as compared to the healthy individuals. Mechanistically, IL-17A induced metabolic rewiring by enhanced complex I activity, increased mitochondrial mass, high mitochondrial reactive oxygen species (mROS). These metabolic shifts led to the potent growth stimulation of lymphoma cells. Finally, disrupting OxPhos by pharmacological inhibition of complex I reduced cell viability significantly via mitochondrial metabolic shut-down in IL-17A-treated cells.

**Conclusion**

Our findings identify IL-17-mediated mitochondrial metabolic deregulation as a critical adaptation in the pathogenesis of CTCL and present mitochondrial complex I as a rational therapeutic target to deal with the metabolic adaptations and enhance therapeutic responsiveness in T-cell lymphoma.

### EACR2024-0647
### Identifying genetic and epigenetic mediators of tumour cell-intrinsic immune evasion in nasopharyngeal

© 2024 The Authors. Molecular Oncology is published by John Wiley & Sons Ltd on behalf of Federation of European Biochemical Societies

### carcinoma

F. Chung[1], E.P. Peter[1], L.P. Tan[2], C.O. Leong[3], L.W. Hii[4], A.S.B. Khoo[5], T.P. Lau[6]
[1]*Sunway University, Department of Medical Sciences, Subang Jaya, Malaysia*
[2]*Institute for Medical Research, Molecular Pathology Unit- Cancer Research Center, Shah Alam, Malaysia*
[3]*AGTC Genomics Sdn Bhd, AGTC Genomics, Kuala Lumpur, Malaysia*
[4]*Institute for Research- Development and Innovation IRDI, Center for Cancer and Stem Cell Research, Kuala Lumpur, Malaysia*
[5]*Sidney Kimmel Medical College- Thomas Jefferson University, Department of Medical Oncology, Pennsylvania, United States*
[6]*Sunway University, Department of Biological Sciences, Subang Jaya, Malaysia*

**Introduction**

Nasopharyngeal cancer (NPC) is a deadly malignancy that exhibits relatively high incidence rates in parts of Asia. While the precise mechanisms underlying the pathogenesis of NPC have yet to be fully elucidated, a key finding has been the observation that immuno-suppression often occurs in NPCs through multiple distinct pathways, including the exhaustion of cytotoxic T cells, defects in type I interferon genes and overexpression of viral proteins that inhibit innate immune response. In this study, we aim to identify genetic and epigenetic alterations that may be responsible for acquired resistance towards cytotoxic T lymphocytes (CTLs) in NPCs.

**Material and Methods**

Immune evasion was modelled in vitro by exposing C666-1 and NPC43 NPC cell lines to activated CD8+ CTLs until the cells acquired significant resistance towards CTL-mediated cytotoxicity. The epigenome and genome of CTL-resistant sublines were profiled using the Illumina Infinium MethylationEPIC methylation array and whole genome sequencing respectively.

**Results and Discussions**

Extensive differential methylation was observed in CTL-resistant sublines of NPC43 (6095 DMRs) and C666-1 (8454 DMRs) relative to their parental lines. The majority of the detected DMRs (>80%) exhibited hypermethylation. Pathway enrichment analysis revealed that the common DMRs were enriched in cellular pathways associated with neuronal systems, O-linked glycosylation, and netrin-1 signalling. Whole genome sequencing revealed variants in 12,772 genes in CTL-resistant sublines in both C666-1 and NPC43 relative to their parental sublines. These include genes encoding for human leukocyte antigens (HLA-A, HLA-DRB5, HLA-DQA1, HLA-DRB1), cell adhesion molecule CNTNAP2, and membrane-associated signalling protein DLGAP2. Selected targets will be validated by gene reconstitution studies, and the utility of these targets as prognostic or cancer biomarkers will be evaluated by mining publicly available datasets.

**Conclusion**

This study has revealed several candidate mechanisms by which NPC cells may acquire resistance towards CTLs. We expect that this study will delineate the cellular events that lead to or accompany immune evasion in NPCs, providing novel insights into the aetiology of NPC and potential avenues for NPC treatment and prevention.

### EACR2024-0679
### Exploiting of subtype-specific metabolic vulnerabilities in KRAS-driven NSCLC

S. Etemadi Afshar[1], J. Matschke[1,2], V. Jendrossek[1,2]
[1]*University Hospital Essen AöR, Institute of Cell Biology Cancer Research, Essen, Germany*
[2]*German Cancer Consortium DKTK, partner site Essen a partnership between DKFZ and University Hospital Essen, Essen, Germany*

**Introduction**

*KRAS*-driven Lung cancers display molecular heterogeneity and can be characterized by 3 major subsets defined by the co-occurring somatic mutations (*SKT11/LKB1*- combined with KEAP1 or *ATM, TP53, CDKN2A/B*)[1]. These mutations are predictive of therapy outcomes and limit the success of Radiotherapy (RT). However, the exact contribution of clinically relevant co-occurring somatic mutations in *KRAS*-driven lung cancer cells to the dynamics of cellular metabolism, damage response and RT resistance needs to be defined. We speculate, that the molecular heterogeneity of *KRAS*-driven NSCLC may converge on functional phenotypes predictive for RT response. *1 Skoulidis et al., Cancer Discov. 2015; PMID: 26069186*

**Material and Methods**

We are implementing a panel of KRAS-driven NSCLC isogenic cell lines harboring clinically relevant co-mutations (provided by the Prof. Heymach group at MD Anderson) e.g. LKB1 proficiency and deficiency, to explore the dynamic radiation-induced alterations in metabolic profile (Seahorse Bioanalyser), DNA damage response, proliferation and clonogenic ability. We aim to use functional data and mathematical modeling to identify and validate key metabolic and signaling targets in lung cancer subtypes, aiming to overcome radioresistance. Moreover, we plan to expand this concept by obtaining and implementing isogenic KRAS-driven NSCLC cell lines harboring further clinically relevant co-occurring mutations (*TP53, CDKN2A/B, ATM,* and *KEAP1*) created using CRISPR-Cas9 technology

**Results and Discussions**

Using isogenic A549, NCI-H460 and Calu6 cell lines (± LKB1) revealed increased sensitivity to ionizing radiation (IR) of LKB1-proficient isogenic cell lines compared to its LKB1- deficient counterpart. This might be, at least partially, due to higher dependency of LKB1-deficient cells on glutamine metabolism and reliance on KEAP1 anchor function to ensure protection against ROS (under further investigation). This is supported by the fact that treatment with glutaminase inhibitor (CB-839) has shown re-sensitizing effect on LKB1-deficient cells to IR

**Conclusion**

First results reinforce the hypothesis that co-occurring mutations in *KRAS*-driven NSCLC cell lines influence the resistance to IR, this in return opens an avenue for possible metabolic dependencies and vulnerabilities for each subsets of NSCLC that can be targeted and therapeutically exploited

© 2024 The Authors. Molecular Oncology is published by John Wiley & Sons Ltd on behalf of Federation of European Biochemical Societies

### EACR2024-0684
### Schwann Cells IGF-1 receptor intracellular signaling promotes an endothelial nitric oxide synthase-mediated Transient receptor potential ankyrin 1 (TRPA1) activation

*M. Marini*[1], *L. Landini*[1], *D. Souza Monteiro de Araujo*[1], *M. Montini*[2], *P. Geppetti*[1], *R. Nassini*[1], *F. De Logu*[1]
[1]*University of Florence, Department of Health Sciences-Clinical Pharmacology and Oncology Section, Florence, Italy*
[2]*University of Florence, Department of Experimental and Clinical Biomedical Sciences "Mario Serio"-Medical Genetics Unit, Florence, Italy*

**Introduction**
Chronic pain affects around 70% of cancer patients and becomes more frequent and devasting in the presence of bone metastases. Transient receptor potential ankyrin 1 (TRPA1) is a proalgesic ion channel and an oxidative stress biosensor highly expressed in nociceptive sensory fibers and also in Schwann cells (SCs). SC TRPA1 in mechanical hypersensitivity has been highlighted in different mouse models of pain. Here we investigated the role of SC TRPA1 and oxidative stress in a in vitro model of metastatic bone cancer pain (MBCP) focusing on the contribution of insulin growth factor-1 (IGF-1).
**Material and Methods**
Human primary SCs (HSCs), primary mouse SCs and dorsal root ganglion neurons, human and mouse breast cancer (MDA-MB-231 and E0771 respectively) and osteclast-like induced (U937 and RAW 264.7 respectively) cell lines were used. Calcium ($Ca^{2+}$), nitric oxide (NO) and hydrogen peroxide ($H_2O_2$) imaging experiments were carried out. The IGF-1 effect was measured also in the presence of antagonists of IGF-1 receptor (IGF1-R) (PPP) and TRPA1 (A967079) receptors and inhibitors of: the IRS-1 (NT157), the Akt (Akti-1/2), eNOS (L-NAME) and a NO scavenger (cPTIO). Triple co-culture chambers were established to mimic the in vivo mechanism. Immunofluorescence, western immunoblot assay and RNA scope were also performed.
**Results and Discussions**
SCs generated an oxidative stress burst. The treatment with the IGF-1R antagonist, PPP, prevented the release of $H_2O_2$, but not IGF-1, thus suggesting that osteoclast-derived IGF-1 increases oxidative stress by targeting SC IGF- 1R. IGF-1 stimulation of HSCs induced IGF-1R trans-phosphorylation and the activation of IRS-1 and Akt. IGF-1R activation in HSCs caused eNOS phosphorylation and the release of NO and $H_2O_2$. Both releases were attenuated by PPP, NT157, Akti-1/2 and L-NAME. Stimulation of HSCs with IGF-1 produced an increase in $H_2O_2$, which was prevented by A967079. In the triple chamber, A967079 attenuated the release of $H_2O_2$, but not that of IGF-1 or NO, suggesting that TRPA1 activation and oxidative stress generation are downstream of IGF-1R.
**Conclusion**
Breast carcinoma cells stimulates osteoclasts to liberate IGF-1. The ensuing IGF-1R activation in SCs promotes an eNOS-mediated TRPA1 activation and release of reactive oxygen species that targets SC TRPA1. Glial cell-selective targeting of the various mediators that sustain cascade signals initiated by SC IGF-1R activation could be a promising area for future treatments against MBCP.

### EACR2024-0695
### Trop-2 induces pro-survival autophagy in colorectal cancer cells

*M. Ceci*[1], *M. Ronci*[1], *S. Rome*[2], *M. Trerotola*[1]
[1]*Center for Advanced Studies and Technology CAST- G. d'Annunzio University of Chieti-Pescara, Department of Medical- Oral and Biotechnological Sciences, Chieti, Italy*
[2]*CarMeN Laboratory- INSERM 1060-INRA 1397- Lyon-Sud Hospital- University of Lyon, Department of Human Nutrition, Lyon, France*

**Introduction**
The transmembrane $Ca^{2+}$ signal transducer, Trop-2, is a key driver of tumour growth and malignancy and its up-regulation is associated to poor prognosis of several carcinomas. Currently, how Trop-2 modulates cancer cell survival has been partially elucidated. Autophagy is a key process involved in the regulation of cell death and survival, in normal and pathological conditions, including several malignancies. We hypothesized that Trop-2 could promote long-term survival of colorectal cancer (CRC) cells through regulation of autophagy.
**Material and Methods**
We monitored the growth of KM12SM cells for 20 days through an impedence-based real time analysis to investigate the impact of Trop-2 on cancer cell resistance to nutrient deprivation. Transmission Electron Microscopy and immunogold labelling were performed to evaluate the dynamics of the autophagic process. Biochemistry, proteomics/mass spectrometry and super-resolution confocal microscopy were carried out in cells with or without Trop-2 expression to define the molecular determinants of the Trop-2-driven pro-survival autophagy, both in conditions promoting (serum starvation) or inhibiting (treatment with Bafilomycin A1) the autophagic process. High-content imaging assays were then performed under specific stress conditions to assess the impact of Trop-2 on survival or death.
**Results and Discussions**
We demonstrated that Trop-2 is able to protect CRC cells from cell death during extended periods of culture in exhausted medium. Ultrastructural analysis revealed that Trop-$2^{high}$ cells accumulate significantly larger autophagolysosomes than Trop-$2^{low}$ cells. Trop-2-driven promotion of cancer survival through engagement of the autophagic machinery was confirmed by biochemistry assays showing activation of key autophagic effectors. Consistently, when autophagic flux is blocked by Bafilomycin A1, significantly increased rate of apoptotic cell death was observed.
**Conclusion**
This study demonstrates for the first time that Trop-2 can stimulate the resistance of CRC cells to environmental perturbations through modulation of autophagy. Our results reveal that the inhibition of autophagy is able to convert Trop-2 from a stimulator of cancer cell growth to

Molecular Oncology 18 (Suppl. 1) (2024) 1–495
© 2024 The Authors. Molecular Oncology is published by John Wiley & Sons Ltd on behalf of Federation of European Biochemical Societies an inducer of cancer cell death. The underlying mechanisms require further studies, but this discovery can allow to optimize novel strategies of CRC patient stratification and treatment.

### EACR2024-0696
### MET and NMDAR interaction in colorectal cancer pathogenesis

G. Francescato[1,2], A. Vitacolonna[3,4], C.B. Folco[3,4], S. Gallo[3,4], G. Ferrero[2,5], S. Tarallo[1,4], V. Alberini[1,2], B. Pardini[1,4], A.G. Naccarati[1,4], T. Crepaldi[3,4]
[1]Italian Institute for Genomic Medicine IIGM, Genetic and Molecular Epidemiology, Candiolo TO, Italy
[2]University of Turin, Department of Clinical and Biological Sciences, Orbassano TO, Italy
[3]University of Turin, Department of Oncology, Orbassano TO, Italy
[4]Candiolo Cancer Institute, FPO-irccs, Candiolo TO, Italy
[5]University of Turin, Department of Computer Science, Turin, Italy

**Introduction**
Recent studies have shown that MET interacts with the N-methyl-D-aspartate receptor (NMDAR) in the nervous system. *MET* is a proto-oncogene encoding the tyrosine kinase receptor that, upon binding the hepatocyte growth factor (HGF) ligand, transduces oncogenic signals involved in the invasive program of cancer cells. The calcium ion channel NMDAR, codified by the *GRIN* gene family and composed by four subunits including NMDAR2B, contains an agonist binding site for glutamate. Outside the nervous system, NMDAR is expressed in various tissues, and previous works documented its involvement in cancer progression. Thus, the interest in exploring the crosstalk between NMDAR and the HGF-–MET pathway out of the context of neuron cells, is increased. For instance, in triple-negative breast cancer cell lines, high levels of MET and NMDAR2B co-expression were found. In this study, we investigated the existence of this crosstalk in colorectal cancer (CRC).
**Material and Methods**
Based on previous RNA sequencing data on 90 CRC patients, the expression levels of *MET* and GRIN genes were analysed in matched CRC tissues pairs. *MET*/GRIN gene expression was confirmed in selected CRC cell lines (e.g., HT115, LS411N), and the potential molecular crosstalk between MET and NMDAR2B was further investigated by several immunoassays (e.g., co-immuno-precipitation, proximity ligation assays, immuno-fluorescence). The role of NMDAR in MET-induced migration and invasion was also assessed through wound healing and Matrigel invasion assays and two NMDAR inhibitors (MK-801 and ifenprodil).
**Results and Discussions**
Tumor tissues showed significant upregulated expression levels of *MET*, *GRIN2B*, and *GRIN2D* genes in comparison to adjacent normal mucosa, as also seen in by TCGA data analysis. The comparison between tumor and adjacent normal tissue highlighted altered expression in CRC tissues of genes enriched in the nervous system and metabolic processes in addition to expected cancer-related signalling pathways. In vitro analysis showed a physical interaction between MET and NMDAR2B. In addition, the increase of cancer cell migration and invasion induced by HGF was inhibited by the MK-801 and ifenprodil treatment.
**Conclusion**
With the present results we have demonstrated the existence of a MET-NMDAR interplay driving the CRC invasive program, paving the way for a new combinatorial therapy.

### EACR2024-0703
### The BASP1 signaling protein interferes with the oncogenicity of MYC

L. Weber[1], A.S. Egger[1], M. Kwiatkowski[1], J. Ramos-Pittol[1], M. Hartl[1]
[1]Center for Chemistry and Biomedicine, Institute of Biochemistry, Innsbruck, Austria

**Introduction**
The MYC oncoprotein represents a transcription factor that regulates crucial cellular processes like proliferation, differentiation, or apoptosis. While its activity is essential and highly regulated in normal cells, MYC is found to be deregulated in ~70 % of all human tumors where it represents a major cancer driver. One of the multiple transcriptional MYC targets is the brain acid-soluble protein 1 (*BASP1*), which is downregulated in a variety of MYC-dependent cancer cells. We found previously that ectopic BASP1 expression interferes with MYC-induced cell transformation.
**Material and Methods**
Using the human colon cancer cell line SW480 featured by high MYC expression and a silenced *BASP1* gene, we further investigated the putative tumor-suppressive property of BASP1. Three different cell types were established in which BASP1 is re-expressed. Whereas two cell lines are characterized by ectopic BASP1 expression, the third one was generated by CRISPR-mediated *BASP1* gene activation. Relevant cell lines were subjected to qPCR and Northern analysis, RNA sequencing, immunoblotting, agar colony assay, and liquid chromatography coupled to mass spectrometry (LC-MS).
**Results and Discussions**
Expression of BASP1 in SW480 leads to a decrease of MYC protein and mRNA levels, and a significantly reduced transformed phenotype. Proteome comparison of SW480 cells with those ectopically expressing BASP1 showed that 278 proteins were found to be specifically activated in BASP1-expressing cells including the tumor suppressor TP53. Among the 252 downregulated proteins are the MYC-associated factor X (MAX) and the metastasis-associated protein 1 (MTA1). Metabolome analyses from the same cell types revealed a potential effect of BASP1 on the glucose metabolism manifested by decreased lactate levels. Further insights will be obtained from an immunological BASP1 protein pulldown followed by MS analysis. Currently, *MYC* promoter analyses are performed and preliminary results suggest a downregulation by the transcriptional co-suppressor BASP1. Furthermore, we also test BASP1-mimetic peptides to develop strategies for the treatment of tumor cells featured by high MYC expression.
**Conclusion**

© 2024 The Authors. Molecular Oncology is published by John Wiley & Sons Ltd on behalf of Federation of European Biochemical Societies

Re-expression of BASP1 in SW480 cells leads to a significantly changed cell morphology with a drastically reduced transformed phenotype supporting a tumor-suppressive function of BASP1.

### EACR2024-0711
### Novel insights into TAZ oncogene regulation by CRISPR-based approach: a focus on FERMT2

*A. Chiesa[1], V. Poli[1], O. Croci[2], P. Fuentes[3], F. Nicassio[1], S. Campaner[1]*
*[1]Italian Institute of Technology IIT, Center for Genomic Science, MIlan, Italy*
*[2]Human Technopole, Computational Biology, Milan, Italy*
*[3]Human Technopole, National Facility for Genome Engineering & Disease Modelling, Milan, Italy*

**Introduction**
YAP/TAZ are activated in a broad range of carcinomas and responsible for endowing cells with many oncogenic features. In particular, TAZ overexpression has been correlated with increasing aggressiveness of mammary gland tumors. Yet, their mutation or the genetic alterations of their known upstream regulators are relatively rare, raising the question on how YAP/TAZ are upregulated in cancer cells.
**Material and Methods**
To uncover novel TAZ regulators within the context of breast cancer, we conducted loss-of-function CRISPR/Cas9 screens on a selected subset of genes, previously identified in our laboratory as modulators of TAZ transcriptional activity. We assessed these genes in a set of phenotypic screens, both in vitro and in vivo. Specifically, we evaluated their role in (i) cell proliferation and survival (by screening in adherent culture conditions), (ii) anchorage-independent growth and self-renewal (by culturing cells in sphere-forming conditions), and (iii) in the regulation of tumor initiation and maintenance in mammary gland (by orthotopic transplantation, in vivo).
**Results and Discussions**
Among the identified hits, FERMT2 emerged as a top candidate across all the screening conditions. FERMT2 exhibits tumour-promoting functionsand it has been linked to YAP/TAZ activation. However, genetic data on the mechanism of interaction with the components of the Hippo pathway and a mechanistic analysis of FERMT2-TAZ regulation are missing. Here, we have characterized FERMT2 role both in vitro and in vivo, confirming that its loss impairs fitness and aggressiveness of breast cancer cells, with a more pronounced phenotype observed in vivo. Moreover, genetic analyses revealed that depletion of FERMT2 leads to the downregulation of YAP/TAZ transcriptional activity through a Hippo-independent mechanism of action, that can be rescued by the overexpression of an active mutant form of TAZ ($TAZ^{S89A}$) both in vitro and, with enhanced efficacy, in vivo. Currently, our investigations are focused on evaluating a Hippo-independent mechanism of YAP/TAZ regulation.
**Conclusion**
In this study, we linked genetic depletion of candidate TAZ regulators to the impairment or acceleration of breast cancer cell growth, paving the way for future investigations on their mechanism of action, their functional relationship with the TAZ oncogene and their potential exploitation to design novel strategies to decommission TAZ activity selectively in cancer cells. Among others, we identified FERMT2 as a prominent upstream activator of YAP/TAZ.

### EACR2024-0715
### Significant spare signaling capacity of oncogene-driven MAPK pathway in melanoma

*S. Uldrijan[1,2], N. Vadovicova[1], K. Smolkova[1], A. Novakova[1], B. Valcikova[1]*
*[1]Masaryk University- Faculty of Medicine, Department of Biology, Brno, Czech Republic*
*[2]St. Anne's University Hospital, International Clinical Research Center, Brno, Czech Republic*

**Introduction**
Oncogenic *BRAF* and *NRAS* mutations activating the RAS/RAF/MEK/ERK mitogen-activated protein kinase (MAPK) pathway are well-known drivers of malignant melanoma. However, due to the high activating potential of the mutations, the intensity of ERK MAPK signaling must be limited by various negative feedback mechanisms to allow for optimal tumor growth. The extent of ERK signaling downregulation by the feedback and spare signaling capacity of the MAPK pathway has yet to be quantitatively analyzed.
**Material and Methods**
We recently identified several novel inhibitors of the phosphatase-mediated negative feedback in the MAPK pathway. Here, we used Western blotting and luciferase reporter cell lines to analyze ERK activity changes in *BRAF*- and *NRAS*-mutant human melanoma cells in response to the feedback disruption.
**Results and Discussions**
Our results indicate that the steady-state ERK MAPK signaling flux in melanoma cells bearing oncogenic *BRAF* and *NRAS* mutations represents only a tiny fraction of the total signaling capacity of the ERK pathway. Small-molecule compounds disrupting the phosphatase-mediated negative feedback can potently disturb the control of the MAPK optimum in melanoma cells.
**Conclusion**
We quantitatively analyzed the MAPK optimum in melanoma cells and identified significant spare signaling capacity that can be mobilized by drugs disrupting the negative feedback control.
*Acknowledgments: This work was the Czech Science Foundation (GA22-30397S), the European Union – Next Generation EU - the project National Institute for Cancer Research (Programme EXCELES, Project No. LX22NPO5102), and Masaryk University (MUNI/A/1603/2023).*

### EACR2024-0752
### Exploiting MASTL inhibition as a new therapeutic target for Acute Myeloid Leukaemia

*S.M. Martin Guerrero[1], T. Shields[1], R. Zach[2], P. Casado Izquierdo[1], H. Gerdes[1], V. Rajeeve[1], H. Hochegger[2], P.R. Cutillas[1]*
*[1]Barts Cancer Institute. Queen Mary University of*

© 2024 The Authors. Molecular Oncology is published by John Wiley & Sons Ltd on behalf of Federation of European Biochemical Societies

*London,Centre for Cancer Genomics and Computational Biology, London, UK*
[2]*School of Life Sciences. University of Sussex, Genome Damage and Stability Centre, Brighton, UK*

**Introduction**
MASTL (Microtubule Associated Serine Threonine Like kinase) is a key regulator of cell cycle progression that has been associated with poor prognosis in solid tumours. Acute Myeloid Leukaemia (AML) is a haematological malignancy with low survival rates and no cure for most patients. Although MASTL is a promising target in solid tumours, its role in leukaemia is less well understood.
**Material and Methods**
MASTL dependency data was obtained from the CCLE database (DeepMap). Viability of 12 AML cell lines was assessed after 72h treatment with a novel MASTL inhibitor (MASTLi) provided by Prof. Helfrid (Un. of Sussex). Cell cycle was evaluated after 24h of MASTLi treatment. Correlation analysis was calculated by the Spearman method. Quantitative phosphoproteomics was carried out using label free LC-MS/MS after 2h of MASTLi treatment (2 μM).
**Results and Discussions**
AML cell lines showed high genetic dependency on MASTL, and this dependency correlated with the phosphorylation of mitotic proteins. Similarly, MASTLi treatment reduced proliferation and viability in most AML cells tested. The FLT3-ITD mutant-positive cells MOLM13 and MV4-11 were the most sensitive, while HL60, HEL, NB4 and KG1 cells were relatively resistant to the cytotoxic effect of MASTLi. Additionally, the reduction in cell viability after MASTLi treatment correlated with PPP2CA (Phosphatase PP2A catalytic subunit) levels, but not with MASTL expression or the phosphorylation of its substrate ENSA/ARPP19. Thus, most of the AML cell lines tested are dependent on MASTL, but unexpectedly, MASTL levels or its activity do not predict response to MASTLi. MASTLi induced G2/M arrest and polyploidization in P31 but not in MV4-11 cells. Phosphoproteomics analysis after MASTLi treatment of unsynchronized cells showed heterogeneous changes in the phosphorylation pattern across cell lines. MASTLi treatment decreased the phosphorylation of STAT5 signalling proteins in MV4-11 cells, while it increased the phosphorylation of mitotic proteins in P31 cells. In HL60 cells, almost no changes were found. In mitotic P31 cells, MASTLi reduced the phosphorylation of proteins linked to chromosome segregation and kinetochore stability. Thus, MASTLi induced mitotic defects in sensitive cells negative for FLT3-ITD, while impaired FLT3 signalling in positive cells.
**Conclusion**
AML cells are highly dependent on MASTL, and in MASTLi sensitive cells, MASTLi interferes with the mitosis of cells negative for FLT3-ITD and with the FLT3 signalling in FLT3-ITD mutant-positive cells.

### EACR2024-0758
### Unravelling the role of EPH receptors in endometrial cancer

*<u>L. Incampo</u>[1], S. Gatius[2,3], D. Arango[1], X. Matias-Guiu[2,3,4], Á. Martínez-Barriocanal[1]*
[1]*Institut de Recerca Biomèdica de Lleida IRBLleida, Molecular Oncology, Lleida, Spain*
[2]*Institut de Recerca Biomèdica de Lleida IRBLleida, Pathological Oncology, Lleida, Spain*
[3]*Hospital Universitari Arnau de Vilanova HUAV, Department of Pathology, Lleida, Spain*
[4]*Hospital Universitari Bellvitge HUB, Department of Pathology, Hospitalet de Llobregat, Spain*

**Introduction**
Endometrial cancer is the sixth most diagnosed cancer in women worldwide, and the first gynaecological malignancy in Europe. The histological type and grade of tumours have been integrated with molecular alterations by The Cancer Genome Atlas (TCGA) into a 4-subtype classification with prognostic value. However, the treatment for these subtypes remains unvaried and without any targeted therapy approved. Thus, further investigation for improving patients' stratification and treatment is required. We have identified a subset of EPH receptors with mutually exclusive overexpression in the copy-number high serous-like molecular subtype. EPH receptors are the largest receptor tyrosine kinase family, and their signalling pathways comprise a wide range of downstream molecules and cross-talks. EPH receptors have been described to be involved in cancer progression, and might constitute key genetic drivers in endometrial tumours useful in the screening of tumor burden, identify patients at high risk and personalize cancer treatments with targeted-therapies.
**Material and Methods**
We performed association studies with EPH expression levels in tumor samples (RNAseq, microarray and shot-gun proteomics) and patients' clinicopathological features. We built a tissue microarray with patient samples enriched in serous subtype tumours and evaluated the expression of EPH receptors by immuno-histochemistry. Additionally, we modulated the expression of EPH receptors in endometrioid and serous cancer cell lines using short-hairpin RNAs (shRNAs) and evaluated the tumorigenic properties of the engineered cell lines through functional assays, such as wound-healing, proliferation and clonogenic assays.
**Results and Discussions**
We observed that high levels of specific EPH receptors associate with aggressive tumorigenic features, such as increased tumor size, stage, lymphovascular infiltration, myometrial invasion, and poor patient survival. To confirm these data at a functional level, we reduced the expression of one of these receptors in endometrial cancer cell lines using shRNA sequences, both constitutively and upon doxycycline induction. We observed a detrimental effect in cell proliferation and colony formation in the engineered cell line models with EPH downregulation.
**Conclusion**
Despite further investigations are needed, a subset of EPH receptors seems to have an oncogenic role in endometrial cancer, which might be exploited at the prognostic and therapeutic level.

### EACR2024-0759
### Characterization of the lactate sensing receptor HCAR1's role in cancer cell growth, including initial investigation of

© 2024 The Authors. Molecular Oncology is published by John Wiley & Sons Ltd on behalf of Federation of European Biochemical Societies

### potential synthetic lethal targets

S. Liljewall[1], E. Goncalves Alves[1], S. Falsig Pedersen[2], T.W. Schwartz[1], M. Trauelsen[3]
[1]University of Copenhagen, Novo Nordisk Foundation Center for Basic Metabolic Research, Copenhagen, Denmark
[2]University of Copenhagen, Department of Biology, Copenhagen, Denmark
[3]SOLID Therapeutics, SOLID Therapeutics, Copenhagen, Denmark

**Introduction**

The Warburg effect is by now a well-known concept; cancer cells metabolise glucose to lactate and the tumor microenvironment (TME) contains high levels of extracellular lactate. Lactate is however not just a metabolite contributing to the acidity of the TME but is also an important signalling molecule that signals via the lactate receptor, HCAR1. HCAR1 is known to be involved in cancer cell growth, proliferation, metastasis, and immune evasion. HCAR1 is highly expressed tumors compared to healthy tissues and high expression of HCAR1 correlates with poor patient survival in several different cancers, this makes HCAR1 a good novel target for anti-cancer therapy.

**Material and Methods**

HCAR1-KO MDA-MB-231 cells were generated using a dual-guide-RNA Cas9n mediated gene editing. HCAR1-KO was validated by Sanger sequencing and qPCR. HCAR1-KO phenotype was characterized in vitro by 2D and 3D growth, wound healing assay, as well as in vivo, in a nod-scid-gamma (NSG) xenograft model. Investigations of potential combination treatment targets and patient stratification was initially evaluated by various database analyses and then further validated by siRNA mediated knock-down (KD) of genes of interest. Evaluation of potential synthetic lethal (SL) targets was done by tracking cell proliferation by live-cell imaging, as well as by dead versus live cell quantification.

**Results and Discussions**

Both cell growth in 2D and 3D cultures is severely impaired by HCAR1-KO. In the wound healing assay, it's concluded that HCAR1 is involved in cancer cell migration, as the HCAR1-KO cells migration properties are negatively affected compared to WT cells. When implanted as tumors in immunocompromised mice (NSG), we observe a major reduction in tumor growth for the HCAR1-KO tumors compared to the WT, strengthening the claim that HCAR1 is essential for tumor growth.

**Conclusion**

HCAR1 is crucial for MDA-MB-231 cancer cell growth and proliferation in vitro as well as in vivo, this makes it a good novel target for treatment of different kinds of solid tumors. However, to best know which patients that will benefit from receiving such a treatment investigating potential combination treatments and biomarkers for patient stratification is imperative.

### EACR2024-0760
### Integrated Analysis of Targeted Alterations and Whole Transcriptomics in Blood Cancer at the Single-Cell Level: A Comprehensive Solution

P. Leka[1], N. Fang[2], M. Rivandi[1]
[1]Singleron Biotechnologies GmbH, Research and Development, Cologne, Germany
[2]Singleron Biotechnologies GmbH, CEO and Founder, Cologne, Germany

**Introduction**

Despite advances in blood cancer treatment, between 40% and 50% of adults diagnosed with Acute Lymphoblastic Leukemia (ALL) experience a relapse, underscoring the need for more sensitive, single-cell resolution approaches to detect subclonal mutations which may be overlooked by conventional bulk analysis methods. Most current high-throughput single-cell sequencing methods primarily focus on detecting gene expression levels, potentially missing crucial markers for risk assessment and treatment selection. To overcome this limitation and enhance our understanding of tumor responses to systemic therapy, we introduce FocuSCOPE®. This high-throughput multi-omics sequencing solution utilizing specially designed barcoding beads and the innovative SCOPE-chip®, a portable microfluidic chip, efficiently captures comprehensive genetic information, including common leukemia-associated genetic variants and transcriptomes from a very same single cell.

**Material and Methods**

We assessed the technology's performance by applying it to three different cell lines (NB4, CCRF, and K562), which harbor KRAS (A18D) and TP53 (R248Q) mutations, as well as PML-RARA and BCR-ABL1 fusion genes. To compare the mutation and gene fusion detection rates using probe-based targeted beads versus traditional poly-T beads. The sensitivity of the FocuSCOPE® kit was evaluated through a serial dilution of mixed cell lines, distinguishing those harboring BCR-ABL1 fusion genes from those that do not.

**Results and Discussions**

FocuSCOPE® successfully analyzed gene expression profiles and identified point mutations and fusion genes in cell lines harboring these critical markers at a single-cell level. The capture rate significantly exceeded that of conventional poly-T beads in detecting SNVs and fusion genes of interest. Remarkably, FocuSCOPE® achieved 100% sensitivity by detecting nearly all K562 cell lines containing the BCR-ABL1 fusion gene in a 1:1000 ratio with 3T3 cell lines lacking the fusion gene.

**Conclusion**

FocuSCOPE® represents a significant advancement in single-cell sequencing, providing a powerful multi-omics solution for the simultaneous analysis of genetic variants and transcriptomes in leukemia cells. This technology promises to advance personalized medicine by providing a more detailed and accurate understanding of individual patient biology.

### EACR2024-0761
### Comparison of membrane potential changes in different cell cycle phases of MCF-7 human breast cancer cells using flow cytometry

S. Langthaler[1], W. Schreibmayer[2], H. Strohmaier[3],

Molecular Oncology 18 (Suppl. 1) (2024) 1–495
© 2024 The Authors. Molecular Oncology is published by John Wiley & Sons Ltd on behalf of Federation of European Biochemical Societies

*B. Pelzmann[2], T. Rienmüller[1], S. Schrübel[2], Z.P. Klaus[2], N. Shrestha[2], A. Gorischek[2], C. Baumgartner[1]*
*[1]Graz University of Technology, Institute of Health Care Engineering with European Testing Center of Medical Devices, Graz, Austria*
*[2]Medical University of Graz, Gottfried Schatz Research Center for Cell Signaling- Metabolism and Aging, Graz, Austria*
*[3]Center for Medical Research, Core Facility Imaging, Core Facility Imaging, Austria*

**Introduction**

Membrane potential changes are considered to trigger cell cycle transitions and recognized as essential bioelectric signals in cellular processes linked to tumor development and progression. Traditional methods for measurement of the membrane potential like patch-clamp are time-consuming and limit the number of cells studied. To overcome the technical limits and screen multiple cells simultaneously, voltage indicators combined with e.g. flow cytometry might offer an alternative technique. However, such GEVIs are limited to short time intervals, i.e. action potentials, and varying expression levels hinder comprehensive comparisons between individual cells. Ratiometric GEVIs like ASAP3-mCyRFP2 might open the door for quantitative comparison of the membrane potential in large pools of cells and different subpopulations, e.g. stages of the cell cycle. This pilot study explores ASAP3-mCyRFP2's potential for membrane potential assessment with flow cytometry, specifically focusing on changes during the cell cycle in MCF-7 breast cancer cells.

**Material and Methods**

MCF-7 cells were cultured and transfected following standard procedures. The subcellular localization of the probe was obtained via confocal laser scan microscopy (Leica Microsystems, Heidelberg, Germany). Flow cytometry was performed on MCF-7 cells, harvested by trypsination 48h after transfection and stained with 20µg/ml Hoechst33342, on a CytoFLEX LX flow cytometer (Beckman Coulter). Cells were identified by fluorescence in the red channel and classified into G1, S and G2/M phase by Hoechst intensity.

**Results and Discussions**

This method reveals a minimum mEGFP/mCyRFP2 fluorescence ratio in G1-phase, indicating depolarization, while the S-phase exhibits hyperpolarization compared to G1-phase. G2/M-phase cells display significant hyper-polarization compared to G1- and S-phase in G2/M-phase, which is consistent with previous findings of Wonderlin et al. (1995). Hyperpolarization is generally associated with cancer cell cycle progression, driving cells toward DNA replication and division.

**Conclusion**

ASAP3-mCyRFP2 proved effective in reporting membrane potential over prolonged intervals and enables comparisons between distinct cellular subpopulations. This approach facilitates the investigation of membrane potential changes in large cell populations throughout the cell cycle, addressing the challenge of cellular hetero-geneity in single-cell analysis and thus contribute to a clearer understanding of bioelectric signals in tumorigenesis and cancer progression.

### EACR2024-0772
### Targeting the cysteine-glutathione pathway in pancreatic cancer

*Z. Nwosu[1]*
*[1]Cornell University, Molecular Biology and Genetics, Ithaca, United States*

**Introduction**

Pancreatic ductal adenocarcinoma (PDAC) is a lethal cancer with limited treatment options. PDAC rely on amino acids for growth and altered amino acid metabolism is a feature of cancer cells.

**Material and Methods**

To identify the most essential amino acid for PDAC, we cultured PDAC cells in a media lacking one amino acid at a time, followed by viability assay, metabolomics, and gene expression profiling analysis. Additional analyses were performed based in mice upon dietary cysteine manipulation.

**Results and Discussions**

The results identified cysteine as the most essential amino acids for a PDAC subset. Further assays revealed three subsets based on cysteine dependency, namely independent-, moderately- and highly-dependent subsets. Cysteine highly-dependent PDAC cells showed a significantly reduced viability, sensitivity to oxidative stress, and several molecular alterations. Metabolomics profiling revealed that while cysteine-independent cells ramp up intracellular glutathione in the absence of cysteine, the cysteine-dependent cells fail to utilize such compensatory response. We further found that the cysteine-glutathione pathway is consistently altered in human PDAC tumors.

**Conclusion**

Collectively, our data reveal the prospects of selectively targeting the cysteine-glutathione pathway for therapy in a subset of PDAC patients.

### EACR2024-0777
### Anti-cell proliferative and cytotoxic effect of ethanolic extract of Caesalpinia bonducella and Cresentia cujute in human oral epidermal cancer cell line (Hep-2)

*A. Chinnaiyan[1], P. Deivasigamani[1], N. Ahamed[2], S. Kathiresan[2]*
*[1]Thanthai Periyar Government Arts and Science College Autonomous-Affiliated to Bharathidasan University, Post Graduate and Research Department of Biochemistry, Tiruchirappalli, India*
*[2]Annamalai University, Department of Biochemistry and Biotechnology, Annamalainagar, India*

**Introduction**

Oral cancer remains a significant global health concern, necessitating the exploration of therapeutic agents. *Caesalpinia bonducella* and *Cresentia cujute* are traditionally renowned medicinal plants with documented physiological and pharmacological properties. This study aimed to investigate the anticancer potential of ethanolic extracts from *Caesalpinia bonducella* and *Cresentia cujute* against Hep-2 cells, a well-established model for oral cancer research

© 2024 The Authors. Molecular Oncology is published by John Wiley & Sons Ltd on behalf of Federation of European Biochemical Societies

**Material and Methods**
Ethanolic extracts of *Caesalpinia bonducella* and *Crescentia cujete* were prepared, and their cytotoxic effects were evaluated against Hep-2 cells. Hep-2 cell lines were obtained from National Centre for Cell Science (NCCS), Pune, India. The cell viability was analysed by MTT assay
**Results and Discussions**
The effective doses of ethanolic extract of *Caesalpinia bonducella* and *Cresentia cujute* were calculated by MTT assay with different concentrations (10-100 µM/ml) treated with the cell line showed significant cytotoxic activity against Hep-2 cells with an $IC_{50}$ value of 60µM/ml and 43 µM/ml respectively. Our results revealed that both *Caesalpinia bonducella* and *Cresentia cujute* extracts exhibited dose-dependent cytotoxicity against Hep-2 cells, with $IC_{50}$ values indicating significant anti-proliferative effects. These extracts also significantly increased intracellular ROS levels after treatment, suggesting that they may cause oxidative stress-mediated cytotoxicity. Moreover, modifications in MMP were observed, suggesting that mitochondrial dysfunction may be a factor in the anticancer effect of these extracts.
**Conclusion**
The overall results confirmed that *Caesalpinia bonducella* and *Crescentia cujute* ethanol extracts show significant anticancer effects against Hep-2 cells, possibly mediated through ROS generation and disruption of mitochondrial membrane potential.This necessitates further studies on the active components for proper assessment for a possible development as promising anticancer drug.

### EACR2024-0787
### Activating p110β mutations induce changes in cell proliferation and migration

*Ö.A. Işık*[1], *B. Daloglu*[1], *I. Badshah*[2], *V. Rajeeve*[2], *P. Cutillas*[2], *O. Çizmecioğlu*[1]
[1]*Bilkent University, Molecular Biology and Genetics, Ankara, Turkiye*
[2]*Queen Mary University of London, Barts Cancer Institute, London, United Kingdom*
**Introduction**
PI3 kinases (PI3K) phosphorylate $PIP_2$ to generate a potent second messenger, $PIP_3$. By the growth factor activity, the induction of the PI3K signal and the generation of $PIP_3$ activate signaling pathways of cell proliferation and survival. Out of four isoforms of PI3Ks, p110α and β are expressed ubiquitously, while p110δ and γ are specific to immune cells. p110α and p110β are redundant for many functions, although different physiological contexts can result in the emergence of distinct dominant PI3K isoforms. Activating p110α mutations (E545K and H1047R) have been described for their oncogenic activity, yielding divergent signaling from wild-type (wt) p110α. Although p110β amplification is more abundant in cancer, equivalent mutations of p110β (N553S and E1051K) have also been documented. However, little is known about the physiological or pathophysiological effects of p110β mutations. Identifying mutant-specific effectors of PI3K is crucial to intervene in isoform and mutant-specific PI3K activity in cancer. This study aimed to uncover and characterize phosphorylations downstream of p110β-wt and p110β-mutant activities.
**Material and Methods**
We engineered $PIK3CA^{flox/flox}$, $PIK3CB^{flox/flox}$ MEFs to generate p110β-wt-inducible and p110β-mutant-inducible (p110β-N553S and p110β-E1051K) lines via viral transduction. Upon generation of p110β-inducible cells, we abolished the expression of the endogenous p110α and β by Cre recombinase delivery. By doing so, we controlled PI3K isoform specificity by our inducible system. Then, we determined the phosphoproteomic profiles of p110β-wt- and p110β-mutant-specific MEFs by enriching phosphopeptides with titanium dioxide followed by LC-MS/MS proteomics.
**Results and Discussions**
We determined p110β-wt- and p110β-mutant specific phosphoproteome with this constructed molecular system. In silico analyses of phosphoproteomes showed that the p110β-E1051K mutant was involved in the regulation of cytoskeleton and cell migration, and the p110-β-N553S mutant is related to RNA metabolism, nuclear import, as well as the cytoskeletal organization. In agreement with our in silico findings, cellular proliferation and migration assays revealed that both p110β mutants exhibited better proliferative and migration capabilities compared to p110βwt.
**Conclusion**
In summary, we found that p110β mutants N553S and E1051K impact cellular proliferation and migration by modulating effector phosphorylations directly or indirectly and exhibit further oncogenic gain of function activity than p110β amplification.

### EACR2024-0796
### Establishment of a 3D in vitro model to investigate early metastatic colonization of prostate cancer cells to the bone

*S.M. Lissek*[1,2], *M. Gelinsky*[3], *R. Mayr*[2], *R. Offner*[4], *C.A. Klein*[1,5], *M. Gužvić*[2]
[1]*University of Regensburg, Experimental Medicine and Therapy Research, Regensburg, Germany*
[2]*St. Josef Medical Center- University of Regensburg, Department of Urology, Regensburg, Germany*
[3]*Faculty of Medicine University Hospital Carl Gustav Carus-Technische Universität Dresden, Centre for Translational Bone- Joint- and Soft Tissue Research, Dresden, Germany*
[4]*Institute for Clinical Chemistry and Laboratory Medicine, Transfusion Medicine- University Hospital Regensburg, Regensburg, Germany*
[5]*Fraunhofer Institute for Toxicology and Experimental Medicine, Division of Personalized Tumour Therapy, Regensburg, Germany*
**Introduction**
Metastasis is the leading cause of cancer-related deaths, with bone being the most frequent site for colonization in prostate cancer. However, the cellular interactions between disseminated cancer cells (DCC) and metastatic niche cells are largely unknown. To study such interactions, models reflecting this crucial step in cancer

© 2024 The Authors. Molecular Oncology is published by John Wiley & Sons Ltd on behalf of Federation of European Biochemical Societies progression are required to develop effective adjuvant therapies.

**Material and Methods**

We aimed to establish a humanized 3D in vitro BM-model, incorporating both vascular and endosteal niches. The vascular niche comprised blood-derived endothelial precursor cells and patient's BM mesenchymal stromal cells (pBM-MSCs), while the osteoblastic niche involved coating calcium phosphate cement scaffolds with pBM-MSCs and their differentiation into osteoblasts. Further, human platelet lysate (hPL) was investigated as a substitute for fetal bovine serum (FBS). To model early metastatic colonization, we seeded <50 cells of the bone metastasis-derived prostate cancer cell line PC3 into niches comprising either ECM alone, vascular cells or vascular and osteoblastic cells. As DCC-associated variable, we investigated the impact of the epithelial-cell-adhesion molecule (EpCAM) expression that had previously been linked to the stemness potential of prostate cancer cells. Confocal imaging was used to assess cancer cell growth after 10 days.

**Results and Discussions**

We successfully isolated pBM-MSCs from various donors and noted better expansion by hPL compared to FBS (n=13, p=0.0002). Next, we assessed the impact of BM-niches on cancer cell colony outgrowth, revealing significant variations among niche conditions. The vascular niche supported growth of cancer cells up to 20-fold compared to extracellular matrix (ECM). However, adding osteoblasts to the vascular niche resulted in an up to 3.5 times reduction of cell volume, indicating an inhibitory effect on cell proliferation. EpCAM expression increased proliferation in the ECM niche by about 3.6.-fold but not within BM niches.

**Conclusion**

PC3 cell proliferation varied between the three niches, with the vascular niche showing the highest rates and the osteoblastic niche displaying an inhibitory effect. The impact of EpCAM expression was found to be niche-dependent. Thus, the presented model enables to dissect DCC-intrinsic and DCC-extrinsic mechanisms of early bone metastasis formation.

### EACR2024-0798
### Neutrophil Extracellular Traps modulate Cancer Cell Adhesion through α5β1 integrin and CCDC25 Receptor

*J. Dimitrov[1], M. Maddalena[1], C. Terlizzi[1], G.G. Altobelli[1], V. De Rosa[2], F. Iomelli[2], S. Del Vecchio[1]*
*[1]University of Naples Federico II, Advanced Biomedical Sciences, Naples, Italy*
*[2]National Research Council, Institute of Biostructures and Bioimaging, Naples, Italy*

**Introduction**

The interplay between cancer cells and neutrophils has been widely investigated and, depending on the context, neutrophils can either promote or inhibit cancer growth and progression. Here we investigated the role of neutrophil extracellular traps released by activated neutrophils in modulating the adhesion properties of cancer cells. Previous studies reported that α5β1 integrin can bind to fibronectin included in NET structure and CCDC25 receptor can bind to DNA component of NETs. Therefore, we determined the expression of β1 chain and CCDC25 receptor in a panel of cancer cell lines including H1975, HCC827, A549, H1993 lung cancer cells, MCF-7, MDA-MB-231 breast cancer cells, fibrosarcoma HT-1080 cells and glioblastoma U87-MG cells.

**Material and Methods**

Cells were subjected to a solid-phase adhesion assay using NET-coated plates. PBS and conditioned medium (CM) of neutrophil-like cells were used as negative controls along with pre-treatment of NET-coated wells with DNase I. Then 3x105 cells were seeded in each well and allowed to adhere for 1, 2 or 4 h in a humidified incubator at 37°C and 5% CO2. Adherent cells were expressed as percentage of total cell number. Furthermore, we tested whether an excess of antibodies against α5β1 and CCDC25 could prevent cell adhesion to NETs. In parallel experiments, H1975 and HCCC827 cells were allowed to adhere to uncoated plates and then NET suspension was added to culture medium. After 48h adherent and non-adherent cells were counted.

**Results and Discussions**

Cell adhesion to NET-coated plates varied between 30 % and 92.7% depending on the cell line. In all cell lines, adhesion was significantly higher than that obtained in uncoated plates. With the exception of H1993 cell line, cell adhesion to NETs decreased significantly when NET-coated wells were subjected to pre-treatment with DNase I. The addition of antibodies against α5β1 or CCD25 caused a strong reduction of cell adhesion to NETs indicating that both molecules can be involved in cell binding to NETs. When adherent cells were incubated with NET suspension for 48h, 87.7% of H1975 cells and 43.8 % of HCC827 cells lost their adhesion to uncoated plates whereas only 7.8 % of untreated H1975 cells and 9.3% of untreated HCC827 cells were detached at the same time point.

**Conclusion**

In conclusion, when NETs are used as an adhesion substrate in a solid-phase adhesion assay, they promote cell attachment. On the contrary, when NETs are used in suspension on adherent cells for a prolonged time, they promote cell detachment

### EACR2024-0799
### Neutrophil Extracellular Traps (NETs) induce epithelial-mesenchymal transition in EGFR- driven lung cancer cells and breast cancer cells

*M. Maddalena[1], J. Dimitrov[1], C. Terlizzi[1], G.G. Altobelli[1], V. De Rosa[2], F. Iommelli[2], S. Del Vecchio[1]*
*[1]University of Naples Federico II, Advanced Biomedical Sciences, Naples, Italy*
*[2]National Research Council, Institute of Biostructures and Bioimaging, Naples, Italy*

**Introduction**

Neutrophil extracellular traps (NETs) are complex filamentous structures released by activated neutrophils and composed of DNA, histones and granular proteins. Previous studies reported that NETs can promote metastatic dissemination of cancer cells by entrapment of

© 2024The Authors. Molecular Oncology is published by John Wiley & Sons Ltd on behalf of Federation of European Biochemical Societies circulating tumor cells at distant organs. More recent evidences indicate that NETs can modulate different steps of the metastatic cascade through not completely elucidated mechanisms.The aim of our study was to test whether cell exposure to NETs can activate the epithelial-to-mesenchymal transition (EMT) program thus enhancing the migratory and invasive properties of tumor cells.

**Material and Methods**

To this end we selected a panel of cancer cell lines including EGFR-driven H1975 and HCC827 lung cancer cells and estrogen-dependent MCF-7 breast cancer cells. Briefly, cells were seeded in 6-well flat-bottomed plates and allowed to adhere overnight at 37°C and 5% CO2. After washing with cold PBS, adherent cells were incubated with 0.5 µg/ml of NET suspension in serum-free medium for 4h, 24h and 48h in a humidified incubator and then lysed for subsequent analysis. The levels of E-cadherin and vimentin, well-known markers of EMT activation, were determined by western blotting along with the expression of transcription factors that are effectors of EMT including SLUG, SNAIL, and ZEB1. In parallel experiment the loss of epithelial phenotype was tested by determining the levels of mediators of EGFR signaling cascade.

**Results and Discussions**

We found that after 24-48 h of exposure to NETs, levels of E-cadherin were decreased whereas expression of vimentin was increased in NET-treated H1975, HCC827 and MCF-7 cells as compared to untreated controls. At the same time points, NETs also induced the down-regulation of the whole EGFR signaling cascade by reducing the levels of EGFR, pEGFR, AKT, pAKT , ERK 1/2, pERK 1/2 and cyclin D1 in EGFR-driven H1975 and HCC827 cell lines thus confirming the loss of the epithelial phenotype. In agreement with these findings, levels of SLUG, SNAIL and ZEB1 were increased in NET-treated cells as compared to untreated controls.

**Conclusion**

In conclusion, the exposure of EGFR-driven H1975, HCC827 lung cancer cells and MCF-7 breast cancer cells to NETs for 24-48 h can activate the EMT program and promote the loss of the epithelial phenotype. The acquisition of the mesenchymal phenotype may enhance the migratory and invasive properties of these cells that in addition become resistant to EGFR targeted therapy.

### EACR2024-0805
### Targeting Tumor Metabolism with Arginine Deprivation: A Novel Approach to Anticancer Therapy

*H.J. Wang[1,2], S.C. Hsu[3], H.J. Kung[4]*
*[1]Institute of Medical Sciences- Tzu Chi University, NA, Hualien, Taiwan*
*[2]Doctoral Degree Program in Translational Medicine, Tzu Chi University and Academia Sinica, Hualien, Taiwan*
*[3]Institute of Biotechnology. National Tsing Hua University, Dept of Life Sciences, Hsinchu, Taiwan*
*[4]Ph.D. Program for Cancer Biology and Drug Discovery, College of Medical Science and Technology- Taipei Medical University, Taipei, Taiwan*

**Introduction**

Arginine deprivation has emerged as a promising strategy for cancer therapy by targeting tumor metabolism. Many types of cancer become dependent on external sources of arginine due to the selective silencing of the ASS1 gene. ADI-PEG20, a PEGylated arginine deiminase, effectively metabolizes arginine into citrulline and ammonia, and has demonstrated impressive results in clinical trials for treating a variety of cancers.

**Material and Methods**

IIn this study, we combined transcriptome and metabolic profiling to identify the cellular pathways involved in arginine depletion. In addition, chromatin immuno-precipitation sequencing (ChIP-Seq) was used to determine the occupancy of repressive histone methylation markers. Several prostate cancer cell lines were used in this study. LNCaP and CWR22Rv1 cells show low ASS1 expression, while the prostate cell line RWPE1 exhibits high ASS1 expression.

**Results and Discussions**

Our research has shown that ADI-PEG20 induces a unique mechanism of tumor cell death by disrupting mitochondrial function and inducing chromatin autophagy. Additionally, we have discovered that KDM8, a lysine demethylase that regulates PKM2 activity, has a significant impact on tumor metabolism. We also noticed a significant decrease in the levels of metabolites of glycolysis and glutaminolysis, including α-ketoglutrate, which is essential for KDMs. Additionally, the genes responsible for metabolic and nuclear processes in TCA cycles are simultaneously reduced but can be restored by introducing arginine. To investigate whether this coordinated gene suppression occurs through an epigenetic mechanism or as a result of KDM inhibition, we examined histone methylation at the promoter sites of metabolic genes.

**Conclusion**

Our findings revealed consistent accumulation of histone suppressive marks associated with transcriptional repression in cells treated with arginine deprivation. Therefore, our results indicate that epigenetic reprogramming plays a crucial role in coordinating the suppression of metabolic gene expression in arginine-deprived cells.

### EACR2024-0825
### Modulating Stat1 activity as therapeutic option in Anaplastic Large Cell Lymphoma

*N. Mora[1], C. Giordano[1], M. Bećirović[1], J. Miao[2], S.D. Turner[3], L. Kenner[4], Z.Y. Zhang[2,5,6,7], O. Merkel[1]*
*[1]Medical University of Vienna, Department of Pathology, Vienna, Austria*
*[2]Purdue University, Department of Medicinal Chemistry and Molecular Pharmacology, West Lafayette, USA*
*[3]University of Cambridge, Division of Cellular and Molecular Pathology- Department of Pathology, Cambridge, United Kingdom*
*[4]University of Veterinary Medicine Vienna, Unit of Laboratory Animal Pathology, Vienna, Austria*
*[5]Purdue University, Institute for Drug Discovery, West*

© 2024 The Authors. Molecular Oncology is published by John Wiley & Sons Ltd on behalf of Federation of European Biochemical Societies

Lafayette, United States
[6]Purdue University, Center for Cancer Research, West Lafayette, United States
[7]Purdue University, Department of Chemistry, West Lafayette, United States

**Introduction**

Anaplastic large cell lymphoma (ALCL) is an aggressive, $CD30^+$ T-cell lymphoma with 50% of patients bearing the typical NPM-ALK fusion. This causes the constitutive activation of members of the JAK/STAT pathway including JAK2, JAK3, STAT3 and STAT5, which are known to drive tumor formation. However, the role of highly expressed transcription factor STAT1 in ALCL tumorigenesis and immune activation is still widely unexplored.

**Material and Methods**

Immunohistochemistry (IHC) for STAT1, phosphorylated STAT1 (Tyr701, Ser727), STAT3, phosphorylated STAT3 (Tyr705), CD4-NPM-ALK transgenic mouse models with T-cell specific STAT1 knock-out, retroviral transduction of green fluorescent protein (GFP) marked mutated forms of STAT1 in cell lines, inhibitors and molecular degraders of PTPN1/PTPN2 phosphatases.

**Results and Discussions**

It is known that pTyrSTAT3 is a highly expressed tumor driver in ALCL. We show here that STAT1, which has been demonstrated to heterodimerize with STAT3, is also highly expressed in ALCL. Mutation of the STAT1 Tyr701 site into phenylalanine creates a constitutively inactive STAT1 form. Interestingly, overexpression of this STAT1 version led to cell death of ALCL cells suggesting a dominant negative effect resulting in deactivated STAT1/STAT1 and/or STAT1/STAT3 dimers. Moreover, in the established CD4 NPM-ALK mouse model we knocked-out STAT1 in a T-cell specific manner with the goal to study effects of STAT1 depletion on overall-survival but also on anti-tumor immunity. Phosphatases are known to deactivate the JAK/STAT pathway therefore we tested the effect of PTPN1/PTPN2 phosphatase inhibition on STAT1 phosphorylation. Hyperphosphorylation of STAT1 and STAT3 was observed together with cell death induction.

**Conclusion**

STAT1 and its tyrosine 701 phosphorylated form is highly expressed in ALCL as assessed by IHC. In contrast to STAT3, STAT1 can be found also in the cytoplasmic compartment. Inhibition of STAT1 701 phosphorylation by peptidomimetics or activation of STAT1 by phosphatase abrogation are both interesting new avenues for ALCL treatment. T-cell specific knock-out of STAT1 in an ALCL mouse model will enable us to study the interaction of STAT1 and new inhibitors in a systemic setting.

### EACR2024-0831

### Exploring lipid diversity in breast cancer cell lines: implications for cell proliferation and migration

*H. Leegwater[1], Z. Zhang[2], X. Zhang[1], X. Wang[1], T. Hankemeier[2], A. Kindt[2], A. Zweemer[1], A. Harms[2], S. Le Dévédec[1]*
[1]Leiden University, Division of Drug Discovery and Safety- Leiden Academic Centre for Drug Research, Leiden, The Netherlands
[2]Leiden University, Metabolomics and Analytics Centre- Leiden Academic Centre for Drug Research, Leiden, The Netherlands

**Introduction**

Breast cancers show an increase in tumor growth and depending on the subtype have metastatic potential. These events require changes in cellular metabolism. While some metabolic aspects, such as increased glucose uptake, are shared across breast cancer subtypes, there is a wide metabolic diversity on how cancerous cells achieve cell growth at the subtype and even at the individual patient level. We previously demonstrated that breast cancer cell lines have clear heterogeneity in the expression of metabolic genes. Shared gene expression profiles were related to molecular breast cancer subtypes and cell line morphology, but some cancer cell lines contained unique metabolic profiles. This study aims to understand how this heterogeneity in gene expression influences cell phenotypes. Studying the lipidome can elucidate how the building blocks of membranes and energy storage in lipid droplets contribute to more aggressive cancer cell characteristics.

**Material and Methods**

LC-MS/MS was used to identify and quantify 870 lipid species belonging to 18 lipid classes from a panel of 50 breast cancer cell lines. These lipids are components of lipid droplets, structural lipids and ceramide-related signaling processes. We studied whether cell lines with similar morphologies shared lipid profiles, which lipids were linked to cells with faster proliferation rates, and if cell lines with a higher metastatic potential contained unique lipid profiles.

**Results and Discussions**

Mesenchymal cell lines have lower overall abundances of triglycerides, phosphatidylinositols, and phosphatidyl-ethanolamines, and higher abundances of phosphatidyl-serines and cholesterol esters than epithelial cell lines. Since triglycerides in cells are the main components of lipid droplets and are used as an energy resource, this could mean that these cell lines have different mechanisms of energy storage and energy use. In addition, cell lines with higher proliferation rates exhibit distinct lipid profiles. These profiles consist of a combination of individual lipids spanning multiple classes, characterized by variations in fatty acid chain lengths and double bonds.

**Conclusion**

The lipidome of breast cancer cell lines is heterogeneous, with shared trends related to morphology and proliferation. This detailed insight into lipid composition expands our comprehension of cancer cell membrane makeup and paves the way for uncovering novel metabolic vulnerabilities in cell lines.

### EACR2024-0839

### Paracrine interactions in bone drive breast cancer proliferation and altered osteoblast phenotypes in D mimetic cultures

*S. Nano[1,2], V. Kumar[2,3], S.M. Naqvi[3], L. Littlepage[2,4],*

© 2024 The Authors. Molecular Oncology is published by John Wiley & Sons Ltd on behalf of Federation of European Biochemical Societies

L. McNamara[2,3], G. Niebur[1,2]
[1]University of Notre Dame, Aerospace and Mechanical Engineering, Notre Dame, United States
[2]Lambe Institute for Translational Research and Harper Cancer Research Institute, Biseach Initiative, Notre Dame, United States
[3]University of Galway, Biomedical Engineering, Galway, Ireland
[4]University of Notre Dame, Department of Chemistry and Biochemistry, Notre Dame, United States

**Introduction**

Bone is the most common site of breast cancer metastasis. Cancer-secreted factors acting on bone cells increase bone resorption and secretion of cytokines that enhance cancer cell proliferation in a vicious feedback cycle. Bone and cancer cells are mechanosensitive, and mechanical stimulation can inhibit tumor progression and prevent bone destruction. The cytokine CXCL5 promotes breast cancer cell proliferation in bone. Whether bone cell secreted CXCL5 regulates tumor spheroid growth and how tumor cells influence bone cell phenotypes is not understood. We created a multicellular model to investigate whether (1) CXCL5 production by osteoblasts and/or osteocytes mediates tumor spheroid growth and (2) if osteoblast phenotypes are altered by cancer cells under mechanical stimulation.

**Material and Methods**

(1) Murine OCY454 osteocyte, MC3T3E1 osteoblast, and 4T1 breast cancer cells were embedded in trans-glutaminase crosslinked gelatin in 0.58 or 0.88 kPa matrices. Triculture, osteoblast+4T1, osteocyte+4T1, and 4T1 gels were cultured for 7 days under standard conditions or with the CXCR2 inhibitor SB225002. Proliferation and bone-tumor cell signaling were studied by immunostaining (DAPI, Ki67, PKH26) and qRT-PCR (SOST, RANKL, OPG, OPN, PTHrP, CXCL5). (2) Osteoblast hydrogels were cultured with 4T1 separate gels, or without, for 21 days with osteogenic supplements and then mechanically stimulated for 7 days. Cell proliferation and osteogenic differentiation were analyzed on days 7, 14, 21, and 28 by immunostaining (LIVE/DEAD, DAPI, Actin, DMP1), histology (Von Kassa), biochemical assays (DNA, calcium, ALP), and micro-CT.

**Results and Discussions**

(1) Co-culture of osteoblasts with cancer cells increased breast cancer spheroid growth, and this growth was mitigated by the CXCR2 inhibitor. Osteocytes were insufficient alone to promote spheroid growth but in tricultures decreased osteoblast-induced spheroid growth. However, the effect of osteocytes in combination with CXCR2 inhibition was inconsistent and depended on matrix stiffness. (2) In separated cultures, osteoblast viability and cell spreading decreased in the presence of cancer cells. Osteoblast mineral deposition and calcium content was lower in the presence of cancer cells, while extracellular ALP activity was higher.

**Conclusion**

We developed a novel 3D model of the breast cancer metastatic niche in bone and show that osteoblasts increase metastatic progression and breast cancer cells alter the morphology and differentiation of osteoblasts.

### EACR2024-0847
### The Simulated Cell identifies LIG4 as a potential novel target in PARPi resistance via the modulation of NHEJ deficiency

C. Laczka[1], N. Sándor[1], I. Fekete[2], D. Németh[3], O. Papp[3], B. Elek[4], D. Veres[1]
[1]Turbine Simulated Cell Technologies Ltd., BioLabs, Budapest, Hungary
[2]Turbine Simulated Cell Technologies Ltd., Screening, Budapest, Hungary
[3]Turbine Simulated Cell Technologies Ltd., IRT team, Budapest, Hungary
[4]Turbine Simulated Cell Technologies Ltd., Discovery, Budapest, Hungary

**Introduction**

*Poly (ADP-ribose) polymerase* (*PARP*) is a key enzyme in DNA repair and an approved target in various homologous recombination (HR)-deficient tumors, including breast, ovarian, pancreatic and lung cancer. However, after initial responsiveness, tumors often develop resistance toward PARP inhibitors (PARPi). One of the major mechanisms of PARPi resistance is the suppression of the non-homologous DNA end joining (NHEJ) pathway. The major aim of our study was to apply Turbine's Simulated Cell™ technology, an AI-driven model of cancer cell signaling, to find novel, synthetic lethal targets to overcome PARPi resistance.

**Material and Methods**

We performed in silico experiments on hundreds of simulated biosamples to identify targets that can modulate cancer cells' response toward the PARPi inhibitor, Olaparib. Next, in silico identified targets were prioritized based on effect, druggability, clinical relevance and mechanistic connection with PARPi.
The in silico identified target, DNA Ligase 4 (LIG4) was then validated in in vitr*o* experiments using pharmacological and genetic perturbations, and it's effect on DNA damage repair (DDR) was also assessed. In these experiments, sensitization against Olaparib was investigated in the background of LIG4 downregulation achieved by CRISPR/Cas9 technology. DNA damage was followed by gH2AX labeling. Finally, rescue experiments in engineered cell lines were performed.

**Results and Discussions**

LIG4 is an ATP-dependent DNA ligase that joins double-stranded DNA breaks and promotes DNA repair in the NHEJ pathway. Based on our in silico experiments, HR and NHEJ deficient cells are more vulnerable to LIG4 inhibition, resulting in previously not described synthetical lethality in a selected population of PARPi resistant, HR and NHEJ deficient cells. Guided by this in silico observed responsiveness, we selected 7 intrinsically PARPi resistant cell lines for in vitro validation. By using an in vitro competition assay setup accounting for the necessary duration of treatment for efficacy via DNA damage accumulation, we could validate the novel dependencies in 6 out of the 7 cell lines in a dose-dependent manner. Moreover, in selected experiments PARPi synergy was also observed.

**Conclusion**

Based on our in silico and in vitro experiments LIG4 is an overlooked target with the potential to re-sensitize PARPi resistant HR-deficient tumors.

© 2024 The Authors. Molecular Oncology is published by John Wiley & Sons Ltd on behalf of Federation of European Biochemical Societies extracellular biomechanical signals that affect tumor cell plasticity and therapeutic adaptation.

### EACR2024-0862
### Role of collagen receptors DDR1/2 in metabolic adaptation of dedifferentiated melanoma cells to extracellular mechanical signals

*O. Bouvet*[1], *M. Lecacheur*[1], *A. Soares Hirata*[1], *A. Carminati*[1], *N. Mazure*[1], *C. Boscagli*[2], *I. Nemazanyy*[3], *C. Girard*[1], *S. Tartare Deckert*[1], *M. Deckert*[1]
[1]*Centre Méditerranéen de Médecine Moléculaire, U1065, NICE, France*
[2]*Centre Commun de Microscopie Appliquée CCMA, UniCA, Nice, France*
[3]*Plateforme d'étude du métabolisme, SFR Necker, Paris, France*

**Introduction**

Cutaneous melanoma is a highly malignant and invasive skin cancer. Despite successful therapies targeting the BRAFV600E oncogenic pathway or immune checkpoints, resistances and metastatic relapse occur (Centeno et al. Nat Rev Cancer. 2023). Upon micro-environment and therapeutic pressures, melanoma cells can switch from a melanocytic state to dedifferentiated mesenchymal states. Such adaptive plasticity was described as a driver of therapy resistance (Rambow et al. Genes Dev. 2019). Our team has demonstrated a process of biomechanical adaptation of melanoma to targeted therapies that favors the deposition of extracellular matrix (ECM) components such as collagen, tumor stiffening and therapeutic escape, and identified DDR1/2 collagen receptors in the protection conferred by stromal cell-derived ECM (Girard et al. Cancer Res 2020; Diazzi et al. EMBO Mol Med 2022; Berestjuk et al. EMBO Mol Med 2022).

**Material and Methods**

Here, we have investigating DDR's role in melanoma mechanical plasticity using cell culture models on collagen hydrogel of controlled rigidity combined with 'omics' approaches and using electron and fluorescent microscopy.

**Results and Discussions**

We found that DDR promote proliferation, invasion and drug resistance of dedifferentiated melanoma cells induced by collagen stiffening via an actomyosin/YAP/NFkB pathway. Further experiments revealed that DDR also mediate metabolic adaptation of dedifferentiated cells in response to mechanical signals. We showed that collagen stiffness affects mitochondrial dynamics of melanoma cells and their lipid storage capacities, depending on the expression of DDR in dedifferentiated cells. Metabolomic analyses revealed the activation of the mitochondrial β-oxidation and carnitine biosynthesis pathways in dedifferentiated cells cultivated on stiff collagen matrices. Finally, we found that the fatty acid degradation pathway induced by mechanical signals is enabled by the loss of perilipin-2, a lipid droplet surface protein.

**Conclusion**

Together, these findings provide an original link between ECM signaling, collagen receptors DDRs and melanoma cell metabolism and improves our understanding on the

### EACR2024-0866
### The effects of piR-823, piR-36712, and piR-020326 on ovarian cancer stem-like cells

*E. Gumusoglu-Acar*[1], *G. Ergul*[1], *S.S. Ozdemir*[1], *T. Gunel*[1]
[1]*Istanbul University, Molecular Biology and Genetics, Istanbul, Turkiye*

**Introduction**

Ovarian cancer (OC), also known as the "silent killer", is the type of cancer with the highest mortality among gynecological cancers. Studies have found that cell groups called cancer stem cells (CSCs) found in the tumor mass are important factors in the development of metastasis, chemotherapy resistance and recurrence in OC. CSCs are groups of stem cells that are tumor initiators and have multipotent differentiation ability. Piwi-interacting RNAs (piRNAs), which are effective in regulating gene expression in CSCs, are a class of non-coding RNAs responsible for epigenetic regulations. piRNAs have been used as targets due to their effects on the initiation and progression of cancer and the formation of CSCs.

**Material and Methods**

Analysis of the effects of silencing and mimicking the expression of 3 previously detected piRNAs (piR-823, piR-36712, and piR-020326), whose expression was significantly increased in ovarian CSC (OCSC) using OVSAHO, SKOV3 and CaOV3 cell lines, was examined through qRT-PCR. The effects of piR-823, piR-36712, and piR-020326 on CSC were evaluated by comparing the gene expression of 2D and 3D cultures after siRNA transfection for 48h. Analysis of the effects of these piRNAs on stem cells through transfection was performed by comparing the expressions of stem cell-specific genes (ALDH1A1, ALDH1A2, ALDH1A3, SOX2, CD133, and NANOG).

**Results and Discussions**

According to results, piR-36712 and piR-020326 increased significantly in the 3D spheroids, and piR823 increased significantly in all three cell lines in the CSC markers (CD133 / ALDH (+/-)). Alteration of the expression of these 3 piRNAs caused changes in spheroid formation in ovarian cancer cell lines. Alterations in spheroid formation, which is suggested as a biological feature of CSC, prove the effects of these piRNAs on CSC. The alterations are demonstrated both by changes in the 3D structure of the spheroids and by changes in the expression of CSC-specific genes.

**Conclusion**

According to the results obtained, it was found that there were significant changes in piRNA expression in OCSCs. These findings indicate that piR-823, piR-36712 and piR-020326 affect stem cell potential in OC. As a next step, this suggests that piRNAs could be investigated as a possible therapeutic agent in CSC-targeted treatments.

### EACR2024-0869
### WNT inhibition reduces tumor-mediated osteolytic response in 3D multicellular metastatic models

© 2024 The Authors. Molecular Oncology is published by John Wiley & Sons Ltd on behalf of Federation of European Biochemical Societies

V. Kumar[1,2], S. Nano[2,3], M. Machireddy[3], L. McNamara[1,2], G. Niebur[2,3]
[1]University of Galway, Biomedical Engineering, Galway, Ireland
[2]Lambe Institute for Translational Research and Harper Cancer Research Institute, Biseach Initiative, Notre Dame, United States
[3]University of Notre Dame, Aerospace and Mechanical Engineering, Notre Dame, United States

**Introduction**

In bone metastasis, breast cancer cells and bone cells interact biochemically perpetuating tumor growth and bone destruction. Mechanical loading activates Wnt signalling in bone cells and protects against tumor-induced osteolysis. Bone marrow-derived cytokine IL1β activates Wnt signalling in cancer cells, promoting their colonisation of the bone. However, tumor cells and osteocytes secrete Sclerostin, a Wnt antagonist, which may enhance osteolysis. Thus, how Wnt signalling governs the osteolytic-metastatic response in the presence of mechanical signals is not clear. This study aims to provide an advanced mechanistic understanding of the role of the Wnt signalling pathway for regulating bone cells and tumor cells.

**Material and Methods**

Murine OCY454 osteocytic cells and MC3T3-E1 osteoblastic cells were encapsulated in separate gelatin hydrogel solutions and were layered on top of each other and cultured in osteogenic media for 14 days. A layer of hydrogel encapsulating either 4T1 tumor cells or RAW264.7 osteoclast precursors, or both, was deposited onto these mineralized hydrogels. The media was changed to standard growth media, supplemented with Wnt inhibitor or Sclerostin neutralizing antibody or a combination of the two, and constructs were cultured for a further 7 days. Hydrogels with 4T1 cells alone served as control. Through qRT-PCR, osteogenic (RUNX2) and metastatic activities (PTHrP), osteoclastogenesis (RANKL:OPG), and SOST gene expressions were quantified. Tumour spheroid size and proliferation (KI67) were also assessed. Samples are being analysed by RNA-sequencing to identify differentially expressed genes.

**Results and Discussions**

Osteolysis: Gene expressions of RUNX2 and SOST were significantly downregulated while RANKL:OPG was significantly upregulated in the presence of tumor cells when compared to the mineralized construct with no tumour or osteoclast precursor cells. Wnt-Inhibition: Mean tumor spheroid diameter was reduced in the presence of the Wnt inhibitor. Gene expressions of PTHrP and RANKL:OPG were significantly reduced in multicellular metastatic constructs and mineralized constructs with tumor cells and no osteoclast precursors, respectively, in the presence of Wnt Inhibitor when compared to their untreated control.

**Conclusion**

In our novel 3D multicellular model of bone metastasis, we demonstrate that tumor cells and osteoclast precursor cells reduce osteogenic potential and increase osteolytic behaviour. However, administration of Wnt Inhibitor reduces tumor spheroid growth and osteolysis.

### EACR2024-0876
### CDK6 ablation prevents therapeutic resistance and disease development in EGFR-mutant NSCLC

M. Trkulja[1], E. Casanova[1,2], I. Uras Jodl[1]
[1]Medical University of Vienna, Institute of Pharmacology, Vienna, Austria
[2]Medical University of Vienna, Ludwig Boltzmann Institute for Hematology and Oncology, Vienna, Austria

**Introduction**

Non-small cell lung cancer (NSCLC) is the most prevalent lethal cancer worldwide. One out of five patients harbors EGFR-activating mutations. Osimertinib, an EGFR tyrosine kinase inhibitor (TKI), is the standard care; yet its use is limited by the rapid development of resistance, illustrating the need for additional therapeutic strategies. Sequencing efforts in patients linked elevated expression levels of CDK6 (cyclin-dependent kinase 6) with poor prognosis. The present work aims to study the role of CDK6 in the EGFR-mutant NSCLC tumorigenesis and TKI resistance development.

**Material and Methods**

We analysed a panel of TKI-sensitive and -resistant human EGFR-mutant NSCLC cell lines that express wild-type or loss of CDK6 by performing in vitro proliferation, clonogenicity and apoptosis assays, as well as by using xenograft mouse models. CDK6´s role was further validated by CDK6 degradation and its kinase inhibition. A high-throughput drug screen was performed to identify compounds that could enhance CDK6 inactivation-induced cell death.

**Results and Discussions**

We describe a critical role for CDK6 in NSCLC evolution. The absence of CDK6 not only reduced survival and clonal potential in human EGFR-mutant cells, but also prevented resistance development to osimertinib in naïve-setting. CDK6-specific degraders and kinase inhibitors phenocopied CDK6 deletion. CDK6 inhibition/degradation could also re-sensitize resistant cells to osimertinib. These inhibitory effects were specific for EGFR mutations as KRAS-mutant NSCLC failed to respond to CDK6 depletion. Increased dependence on CDK6 rendered EGFR-mutant cells particularly vulnerable to selective BET (bromodomain and extra-terminal motif) inhibitors. Co-administration of CDK6 and BET drugs effectively targeted TKI-sensitive and -resistant cells. Consistent with our in vitro findings, CDK6 deficiency completely blocked in vivo tumor formation. Our findings show that CDK6 is required for the development and drug resistance of EGFR-mutant NSCLC, and thus provide a rational for targeting CDK6 in lung tumors.

**Conclusion**

Our study defines CDK6 as a predictive marker for TKI response and paves the way for improved clinical outcome for patients with EGFR-mutant NSCLC.

### EACR2024-0880
### Study of the role of RhoA mutations in diffuse gastric cancer oncogenesis

N. Vivancos[1], Á. Martínez-Barriocanal[1], D. Arango[1]
[1]Institut de Recerca Biomèdica de Lleida - IRBLleida,

© 2024 The Authors. Molecular Oncology is published by John Wiley & Sons Ltd on behalf of Federation of European Biochemical Societies

*Group of Molecular Oncology, Lleida, Spain*
**Introduction**
RhoA is a member of Rho-like GTPases with GTP-binding activity. In the active state it allows the regulation of cellular functions such as the rearrangement of actin cytoskeleton and cell proliferation, differentiation and migration. These outputs are dependent on RhoA interaction with downstream protein effectors. In cancer, RhoA is mainly overexpressed and/or activated, contributing to tumour development. However, our group demonstrated that RhoA loss in colorectal tumours contributes to growth, dedifferentiation and metastasis. In gastric cancer, hotspot RhoA mutations (Y42C, R5Q, L57V, G17E) are found in 14.3% of gastric tumours with diffuse histology. These mutations are clustered in the effector and GAP/GEF binding regions, and presumably could alter RhoA activity. Currently, gastric cancer is the sixth most frequent cancer type worldwide and diffuse gastric cancer (DGC) has poor prognosis and limited therapeutic options. Due to the importance of RhoA in the development of gastrointestinal tumours, and the presence of recurrent mutations in diffuse gastric tumours, in this study we aimed to investigate the role of RhoA hotspot mutations in RhoA signalling and DGC oncogenesis.

**Material and Methods**
Predominant RhoA mutant forms were overexpressed in DGC cell lines to interrogate its role in DGC oncogenesis through functional assays. The RhoA interactome was analysed following a high-throughput and unbiased approach. Specifically, glutathione/GST-wild-type or mutated RhoA beads were produced and used to pull-down interactors from a pooled protein lysate from 9 DGC cell lines. Eluted proteins were trypsinized and analysed through HPLC-MS/MS. DGC cell lines were engineered to modulate the levels of expression of the identified RhoA effector proteins, and evaluated in functional assays.

**Results and Discussions**
Specific RhoA mutations exhibited a strong impact on cell migration capacity. A novel interactome with protein gains and losses was identified for each RhoA mutant in comparison to the wild-type RhoA protein. The modulation of mutant RhoA interactors at the protein level in DGC cell lines showed an impact on diffuse gastric tumorigenesis. All together elucidates a RhoA signalling pathway modulation due to hotspot mutations.

**Conclusion**
Wild-type and mutant RhoA display relevant differences in the interactome contributing to DGC oncogenesis.

### EACR2024-0896
### Unveiling diverse ALDH1 isoforms in distinct breast tumor subpopulations: An opportunity to Novel Multitargeting Therapeutic Strategies

*<u>A. Constantinescu</u>[1], R. Pequerul Pavon[2], R. Rebollodo-Rios[3], X. Pares[2], G. Fournet[4], G. Martin[5], I. Ceylan[5], J. Farres[2], M. Perez-Alea[5]*
*[1]Advanced Biodesign, Research and Development, Saint-Priest, France*
*[2]Faculty of Biosciences- Universitat Autonoma de Barcelona, Biochemistry and Molecular Biology, Barcelona, Spain*
*[3]Faculty of Medicine and Univerity Hospital of Cologne, Internal Medicine, Cologne, Germany*
*[4]Université de Claude Bernard-Lyon, Institut de Chimie et Biochimie Moléculaire et Supramoléculaire, Lyon, France*
*[5]Advanced BioDesign, Research and Development, Saint Priest, France*
**Introduction**
Breast cancer is the leading cancer affecting women globally, with 2.3 million new cases and 685,000 fatalities in 2020. This disease exhibits significant molecular and clinical heterogeneity, influencing prognosis and treatment choices. The ALDH superfamily, comprising 19 isoforms, plays essential roles in cellular defense and signaling. Notably, the ALDH1 family is recognized as a marker for breast cancer stem cells, especially in the aggressive triple-negative breast cancer (TNBC) subtype, where it is linked to chemotherapy resistance and a higher metastasis rate. Despite this, the specific contributions of individual ALDH1 isoforms to cancer progression remain elusive, with ALDH1A1 and ALDH1A3 frequently cited as key contributors. Understanding these distinctions is vital for the development and clinical application of ALDH inhibitors.

**Material and Methods**
We conducted a comprehensive bioinformatics analysis using a large bulk RNA-seq dataset of 1103 primary human breast cancers from three independent TCGA datasets and 50 breast cancer cell lines from CCLE 2019. This analysis was complemented by scRNA-seq data from 26 breast cancer patients, sourced from the Single Cell Portal. Functional in vitro studies, including cell signaling, drug-drug interactions, and evaluations of metastatic potential, along with preclinical in vivo efficacy tests in various cell lines and two orthotopic TNBC models, were also performed.

**Results and Discussions**
Our findings revealed distinct expression patterns for ALDH1A1, ALDH1A2, and ALDH1A3 across specific cell subpopulations within breast tumors. Notably, we identified significant differential expression profiles across breast cancer molecular subtypes, particularly distinguishing TNBC basal-like and claudin-low tumor phenotypes. Co-targeting relevant ALDH1 isoforms, in conjunction with pathways associated with drug resistance, suggested a promising synergistic approach for the treatment of TNBC.

**Conclusion**
This study provides a comprehensive overview of the intricate roles played by ALDH1 isoforms across different cell populations in the breast tumor microenvironment. It highlights the limitations of breast cancer cell lines in capturing this complexity and proposes a novel therapeutic strategy that combines specific ALDH1 inhibitors with targeted treatments for TNBC, addressing a critical unmet need in breast cancer therapy.

© 2024 The Authors. Molecular Oncology is published by John Wiley & Sons Ltd on behalf of Federation of European Biochemical Societies

### EACR2024-0924

### Deciphering the role of desmoglein 2 in maintaining the epithelial phenotype of breast cancer cells

*O. Vacek*[1,2,3], *L. Knapeková*[1,2], *J. Ondřejová*[1,2], *P. Jirgalová*[1,2], *R. Fedr*[1], *P. Beneš*[2,3], *K. Souček*[1,2,3]
[1]*Institute of Biophysics of the CAS - v. v. i., Department of Cytokinetics, Brno, Czech Republic*
[2]*Masaryk University, Department of Experimental Biology - Faculty of Science, Brno, Czech Republic*
[3]*St. Anne's University Hospital, International Clinical Research Center, Brno, Czech Republic*

**Introduction**

Desmoglein 2 (DSG2) is a surface protein with a physiological role in desmosomal cell-cell adhesion and maintaining epithelial tissue integrity. In cancer, the role of DSG2 is controversial, as it has been reported to have both pro-tumorigenic and tumor-suppressive effects. Since the connection of DSG2 with the metastatic cascade in cancer is understudied, we focused on its association with epithelial-mesenchymal transition (EMT) traits in breast cancer cells.

**Material and Methods**

CRISPR-Cas9 knockout (KO) cells were validated using Western blot, immunofluorescence, and sequencing of gRNA targets to confirm the loss of DSG2 in the T-47D cell line. DSG2 KO cells were tested for attachment to ECM matrix components with the ECM Select® Array Kit Ultra-36 (Advanced Biomatrix). Multicolor spectral flow cytometry was employed to detect the epithelial-mesenchymal transition (EMT) surface profile of DSG2 KO cells, assessing markers such as EpCAM, Trop2, CD9, CD29, CD49c, GD2, and ITGB5.

**Results and Discussions**

To assess the effect of DSG2 loss in vitro, we established a T-47D DSG2 CRISPR KO model and successfully confirmed DSG2 deletion. We examined the ability of DSG2 KO cells to adhere to various extracellular matrix (ECM) proteins and observed an overall decrease in cell adhesion to most of the individual ECM components. Measurement of the in-house developed and validated EMT surface panel revealed the upregulation of multiple mesenchymal markers in DSG2 KO cells and the downregulation of epithelial markers.

**Conclusion**

Our study demonstrates that in the T-47D breast cancer cell model, the loss of DSG2 leads to a decreased ability to adhere to various ECM components. These deregulations are also reflected in the overall mesenchymal-like cell surface fingerprint of DSG2 KO cells. We conclude that DSG2 in the tested model associates with EMT and thus might have a role in the repression of the metastatic process.

*Acknowledgment: This work was supported by Czech Science Foundation, grant nr. 24-11793S*

### EACR2024-0946

### The proteasome activator complex, PA28ab, regulates stemness in glioblastoma

*K. Heemskerk*[1], *R. Bahia*[1], *O. Cseh*[1], *X. Hao*[1], *H.A. Luchman*[1], *S. Weiss*[1]
[1]*University of Calgary, Arnie Charbonneau Cancer Institute, Calgary, Canada*

**Introduction**

Glioblastoma (GBM) is the most common adult malignant primary brain tumour with inevitable recurrence and poor survival. Brain tumour stem cells (BTSCs), a population of slowly dividing, treatment-resistant cells, are hypothesized to initiate disease recurrence. There is limited understanding of signaling changes that occur in BTSCs upon tumour recurrence. We have identified an up-regulation of the proteasome activator complex, PA28ab, in recurrent BTSCs. PA28ab binds to and activates the proteasome, contributing to the production of specific peptides for antigen presentation during immune responses. Investigating how PA28ab changes cell signaling, and proteasome function will help resolve its role in both fundamental cell biology and in GBM pathogenesis, providing new therapeutic opportunities.

**Material and Methods**

Human and murine GBM BTSCs were used for in vitro and in vivo investigation. CRISPR/Cas9 and shRNA were used for genetic perturbation of PA28ab and Flag-tagged lentiviral overexpression was used to assess protein-protein interaction. In vitro growth was assessed with Alamar blue™. Sphere formation was examined using limiting dilution assays. In vivo growth was assessed with orthotopic xenografts in SCID mice. Transcriptomics and proteomics were performed using total mRNA and label free quantitative LC-MS/MS respectively.

**Results and Discussions**

Transcriptomic and genomic data from our cohort of over 50 BTSC lines, tumours, and xenografts revealed an upregulation of PA28ab expression in recurrent samples. Genetic perturbation of PA28ab did not alter in vitro growth while significantly reducing sphere forming frequency of primary BTSCs. These data suggest a role of PA28ab in self-renewal. Furthermore, PA28ab knockout led to improved survival in vivo. Transcriptomic profiling of PA28ab knockout BTSCs showed enrichment of inflammatory response signatures and downregulation of neuronal like signatures, with conserved downregulation of NCAM1. Proteomic analysis of PA28ab knockout BTSCs revealed upregulation of NFIX, which is known to bind the promoter of NCAM1 and regulate its expression. Co-immunoprecipitation showed direct binding of PA28ab to NFIX, which could in turn lead to its degradation by the proteasome.

**Conclusion**

PA28ab is a potential novel target that could promote GBM stemness via its interaction with NFIX and subsequent regulation of NCAM1.

### EACR2024-0949

### A reporter platform to study damage-induced senescence

*B. Haspels*[1,2], *J. van de Grint*[1], *R. Sangers*[1], *H. Odijk*[1], *T. Reuvers*[1], *R. Kanaar*[1,2], *M. Kuijten*[1,2]
[1]*Erasmus Medical Center, Molecular Genetics, Rotterdam, The Netherlands*

© 2024 The Authors. Molecular Oncology is published by John Wiley & Sons Ltd on behalf of Federation of European Biochemical Societies

[2]Erasmus MC Cancer Institute, Oncode Institute, Rotterdam, The Netherlands

**Introduction**

Cellular senescence is a highly regulated cell-cycle arrest, important for embryonic development and aging, that can be induced prematurely for instance by DNA damage. Therapy-induced senescence (TIS) is a promising anti-cancer strategy, especially when combined with senolytic compounds, which clear senescent cells by targeting anti-apoptotic factors expressed by these cells. To improve the efficacy of senolytics, it is important to better understand the heterogeneous nature of TIS.

**Material and Methods**

To be able to study TIS in cancer, we made reporter constructs based on transgenic lamin A and B1 expression. We used these reporters to screen and sort for senescent and non-senescent cells. We validated our FACS method in breast cancer cells and characterized sorted populations using western blot, immuno-fluorescence staining, and RNA sequencing. We also extended our lamin reporter system with additional senescence-related features such as a p21 promoter-element and a cell cycle marker.

**Results and Discussions**

We developed an imaging- and data-analysis program which distinguishes senescent (S) from non-senescent (NS) cells based on changes in fluorescent intensities of the transgenic lamins upon senescence-induction. We validated this method in immortalized fibroblasts and breast (MCF7), lung (A549) and prostate (DU145) cancer cell lines. Analysis of FACS populations confirmed the senescent state: lower endogenous lamin B1 protein expression, higher amount of Sa-β-gal+ cells (96% vs. 4%), lower amount of cells in G2/M cycle and higher amounts of SAHFs formed in S compared to NS cells. Further, we observed an increased sensitivity to ABT-263 in S compared to NS cells. When live-monitoring S cells with our reporter, we observed that a fraction of these cells was able to re-enter the cell cycle.

**Conclusion**

We show that our reporter platform can be used to distinguish between and sort for senescent and non-senescent cells. Moreover, we show that the extension of our reporters with additional features is useful to monitor senescence-induction and -escape, which may help to find the optimal therapeutic window for senolytics.

### EACR2024-0954
### CITED2 Expression Level Impacts the Self-renewal Properties of Glioblastoma Stem Cells

*M. Fernandes[1], A. Matos[2], S.M. Calado[2], C.D. Correia[2], A.L. De Sousa-Coelho[1], F. Esteves[3], J. Bragança[2]*
*[1]Algarve Biomedical Center Research Institute- University of Algarve, School of Health- University of Algarve, Faro, Portugal*
*[2]Algarve Biomedical Center Research Institute- University of Algarve, Faculty of Medicine and Biomedical Sciences- University of Algarve, Faro, Portugal*
*[3]Algarve Biomedical Center Research Institute- University of Algarve, Algarve Biomedical Center, Faro, Portugal*

**Introduction**

Glioblastoma (GBM) is a type 4 glioma and the most lethal primary brain tumor. The few treatment options currently available and the resistance to therapies largely contribute to its dismal prognosis. Accumulating evidence shows that these tumors are composed of a heterogeneous population of cells that seem to originate from a smaller subset known as Glioblastoma Stem Cells (GSCs). GSCs were shown to present relative quiescence and to be resistant to standard chemotherapy and radiation, which target mainly bulk cells. Therefore, therapeutic approaches targeting these cells may prevent relapse but the molecular mechanisms regulating their maintenance are poorly understood. CITED2 is a co-transcriptional regulator and a key pluripotency factor in embryonic and adult stem cells. This protein was implicated in several types of cancer either with pro-tumorigenic or anti-tumorigenic roles. Nevertheless, a role for CITED2 in GBM was not previously reported.

**Material and Methods**

In primary gliomas, CITED2 expression was analyzed in the lower grade glioma (LGG) and GBM cohorts from The Cancer Genome Atlas (TCGA). CITED2 mRNA and protein expression in GBM cell lines was determined by RT-qPCR and Western Blot, respectively. To determine the impact of CITED2 levels on GBM biology, cell lines with CITED2 overexpression and knockdown were prepared, and self-renewal, proliferation, migration, viability, and tumorigenesis were evaluated on these cells vs the respective controls.

**Results and Discussions**

By analyzing TCGA LGG and GBM cohorts, we found that CITED2 is significantly more expressed in GBM than in LGG. Moreover, CITED2 higher expression was associated with an adverse prognosis. High levels of CITED2 were also detected in several GBM cell lines. By performing functional assays, we found that CITED2 overexpression promoted cancer stem cell properties such as clonogenic potential and sphere formation, an indirect measure of tumorigenicity assessed in vitro.

**Conclusion**

Altogether, our results suggest that CITED2 may contribute the self-renewal and tumorigenic properties of GBM. Therefore, modulating CITED2 expression in this context may be an effective approach to interfere with the stemness properties of GSCs and potentially lead to a better prognosis.

### EACR2024-0962
### Gasdermin-B Isoforms in Cancer: Pro-tumoral roles to Pyroptotic Cell Death

*S. Colomo del Pino[1], S. S Oltra[2], D. Ros-Pardo[3], P. Gómez-Puertas[3], D. Sarrió[4], G. Moreno-Bueno[2]*
*[1]Universidad Autónoma de Madrid, Translational Cancer Research Lab, Madrid, Spain*
*[2]Fundación MD Anderson & Universidad Autónoma de Madrid & CIBERONC,*
*Translational Cancer Research Lab, Madrid, Spain*
*[3]Centro de Biología Molecular Severo Ochoa- CBMSO CSIC-UAM, Molecular Modeling Group, Madrid, Spain*
*[4]Universidad Autónoma de Madrid & CIBERONC,*

© 2024The Authors. Molecular Oncology is published by John Wiley & Sons Ltd on behalf of Federation of European Biochemical Societies

*Translational Cancer Research Lab, Madrid, Spain*

**Introduction**

Pyroptosis, a highly inflammatory form of programmed cell death, is under intense study for its role in tumor immunogenicity. Gasdermin B (GSDMB), as a Gasdermin family member, is an executor of pyroptosis. Its N-terminal domain (NTD) oligomerizes forming pores in plasma membrane, leading to inflammatory cytokine release, influx of ions and water, and ultimately, cell death. Recent studies suggest lymphocyte-derived Granzyme A (GZMA) activates GSDMB pore-forming activity, potentially hindering tumour progression. However, conflicting reports suggest GSDMB overexpression promotes tumorigenesis and associates to poor prognosis, underscoring the need for further investigation. Here, we unveil a key aspect of this paradox: the existence of four GSDMB splicing variants, which may play distinct roles in cancer cell death and tumor progression.

**Material and Methods**

To elucidate the role of GSDMB variants in cancer, we performed mutagenesis, Western blotting, confocal microscopy, and flow cytometry to assess protein function, visualize pore formation/mitochondrial targeting, and quantify cell death in cancer cell lines. MD simulations explored GSDMB-GZMA interactions and predicted pore interface residues in exon 6. TCGA data analysis investigated clinical associations.

**Results and Discussions**

Our findings reveal a critical role for exon 6 in GSDMB-mediated pyroptosis. Among various GSDMB NTD constructs, only those containing exon 6 trigger cell death when overexpressed in cancer cells. MD analysis further identified critical residues (R225, K227, and K229) within exon 6, potentially involved in pore formation. Notably, the triple mutant R225A/K227A/K229A completely abolishes pyroptosis, with K227A single mutation significantly reducing cell death capacity. These point mutations, along with others under investigation, mediate GSDMB-NTD mitochondrial targeting preceding cell death. Additionally, MD simulations suggest that GZMA preferentially interacts with GSDMB exon 6-containing isoforms, potentially influencing their activation. Clinical relevance is emphasized by the observed correlation between the overexpression of GSDMB isoforms lacking exon 6 and poor prognosis in breast tumors.

**Conclusion**

Our study shows that cancer cell death via the GZMA-GSDMB pyroptotic axis is specific to exon 6-expressing GSDMB isoforms. These isoforms may serve as biomarkers to forecast therapeutic response. Further investigations into the regulation of GSDMB isoform expression is needed to clarify their potential as therapeutic targets.

### EACR2024-0975

### Esophageal Cells Undergoing Dedifferentiation: A Reservoir for Pre-Cancerous Metaplasia

A. Vercauteren Drubbel[1], B. Beck[2]

[1]*Université libre de Bruxelles - ULB, IRIBHM, Brussels, Belgium*

[2]*Université Libre de Bruxelles, Institut de Recherche Interdisciplinaire en Biologie Humaine et moléculaire, Bruxelles, Belgium*

**Introduction**

Esophageal adenocarcinoma (eAC) and its precursor, Barrett's esophagus (BE), have surged in Western populations. The etiology of eAC involves a multistep process triggered by chronic gastroesophageal reflux disease, leading to esophagitis, BE, dysplasia, and ultimately eAC. Despite this, the precise cell of origin for BE and eAC remains contentious. BE is defined by the replacement of the squamous epithelium by a columnar epithelium. Several studies indicate that different cell types could be at the origin of BE-like metaplasia, including keratinocytes. However, it would be crucial to recognize that eAC originating from esophageal cells may exhibit less responses to treatment compared to those originating from gastric cells. This emphasizes the significance of studying the cell of origin for eAC. Nonetheless, mechanisms driving transdifferentiation of esophageal progenitors remain elusive. Our recent findings reveal that activation of the hedgehog (HH) pathway in esophageal cells due to chronic acid reflux, is not sufficient to trigger the development of specialized metaplasia but induces the dedifferentiation of esophageal cells into embryonic-like progenitors in vivo. Interestingly, multi-omics data suggest that human metaplasia and eAC may originate from a pool of undifferentiated cells. We thus hypothesized that HH-dedifferentiated cells could be a reservoir for columnar metaplasia and/or eAC initiation.

**Material and Methods**

Our project analyzed copy number variations in human eAC and developed new transgenic mouse models mirroring eAC-associated gene amplification in esophageal epithelium. Through lineage tracing, histology, and RNA sequencing, we characterized esophageal cells.

**Results and Discussions**

GATA4 is a transcription factor crucial for columnar epithelial morphogenesis in the stomach. While it is absent from normal esophagus, we found that it emerges as one of the most frequently amplified genes in eAC and is overexpressed in human BE samples. Therefore, GATA4 looks like a good candidate to drive columnar metaplasia and/or eAC. Our results reveal that while ectopic GATA4 expression in esophageal cells had minimal effects, its expression in HH-dedifferentiated cells induced tissue reorganization and a transcriptomic profile resembling gastric columnar metaplasia.

**Conclusion**

In conclusion, our data suggest that embryonic-like esophageal progenitors constitute a cellular reservoir that has the competence to transdifferentiate into metaplasia that may progress toward eAC by accumulating mutations.

### EACR2024-0976

### Biological impact of pharmacological inhibition of Choline kinase enzyme in ovarian cancer models

E. Citeroni[1], A. Rizzo[1], E. Iorio[2], F. Quartieri[3],

Molecular Oncology 18 (Suppl. 1) (2024) 1–495

© 2024 The Authors. Molecular Oncology is published by John Wiley & Sons Ltd on behalf of Federation of European Biochemical Societies

P. Gnocchi[3], P. Corsetto[4], D. Mezzanzanica[1], M. Bagnoli[1], A. Tomassetti[1]
[1]Fondazione IRCCS Istituto Nazionale dei Tumori di Milano, Experimental Oncology, Milan, Italy
[2]Istituto Superiore di Sanità, Core Facilities, Rome, Italy
[3]Nerviano Medical Sciences Srl, Discovery Pharmacology Department, Nerviano, Italy
[4]Università degli Studi di Milano, Department of Pharmacological and Biomolecular Sciences, Milan, Italy

**Introduction**

Choline-kinase alpha (Chok-α) over-expression supports aberrant choline metabolism in epithelial ovarian cancer (OC), characterized by increased phosphocholine (PCho) and total Choline containing compounds. Our group previously showed that ChoK-α silencing affects PCho intracellular content, cell proliferation, cell migration and invasion in vitro and in vivo, suggesting ChoK-α as a novel putative molecular target. Here, the biological effect of ChoK-α perturbation on OC cells has been evaluating using the first in class ATP-mimetic ChoK-α inhibitor (NMS-P830) highly selective for ChoK-α enzymatic activity, thus able to avoid the toxicity observed with other choline-mimetic compounds.

**Material and Methods**

The mechanism/s by which ChoK-α participates in OC cells dissemination has been assessed in advanced in-vitro models both in static and dynamic conditions, to mimic as close as possible the in vivo growth of OC cells. The efficacy and the effects of the inhibition of ChoK-α catalytic activity were assessed 72 hr after NMS-P830 treatment. PCho intracellular content was assessed by magnetic resonance spectroscopy. Cell adhesion and proliferation were evaluated in real time by xCELLigence.

**Results and Discussions**

Experiments performed on OC cells from different hystotypes showed that 10 μM NMS-P830 is able to:
i) efficiently inhibit the catalytic activity of Chok-α enzyme (assessed as PCho content reduction);
ii) decrease colony formation and cell proliferation due to alterations of cell cycle in G2-M and S phases;
iii) significantly decrease OC spheroids' dimension.
Cells grown in 3D were more susceptible to NMS-P830 compared to cells grown in 2D, suggesting that the inhibition of the catalytic activity of Chok-α by drug treatment affects in 2D cultures both cell-cell and cell-extracellular matrix (ECM) adhesion while in 3D cultures only cell-cell adhesion proteins. Accordingly, Chok-α perturbation affected cell-ECM adhesion in a cell-dependent manner and was sufficient to decrease cell proliferation on different substrates.

**Conclusion**

These data showed a cytostatic role of NMS-P830 in OC cells from different hystotypes and it is effective in inhibiting spheroids formation. The microfluidic-based system is now helping to confirm these data in a dynamic 3D model.

*Acknowledgements: Partially supported by Associazione Italiana Ricerca sul Cancro and by Ministero della Salute Ricerca Corrente L2.2.1/45*

### EACR2024-0979
### Twist1 forms a trimeric complex with p53 and MDM2 and attenuates the efficacy of MDM2 inhibitors

S. Piccinin[1], F. Pivetta[1], C. Rosano[2], R. Maestro[1]
[1]CRO Aviano- National Cancer Institute, Oncogenetics and functional oncogenomics, Aviano, Italy
[2]IRCCS Ospedale Policlinico San Martino, Proteomics and Mass Spectrometry Unit, Genova, Italy

**Introduction**

Soft tissue sarcomas (STS) are rare and aggressive mesenchymal tumors. A significant fraction of STS retains wild-type TP53. In these tumors p53 is inactivated through alternative mechanisms, including MDM2 amplification. Alleviation of this inhibitory activity is expected to trigger a tumor suppressive p53 response. Based on these evidence, inhibitors of the p53:MDM2 interaction (MDM2i) have been developed and tested in clinical trials. Unfortunately, the results obtained so far, although supportive of some clinical activity, have not met the initial expectations due to still uncharacterized mechanisms of primary resistance. Here we present evidence for a role for Twist1 in this context.

**Material and Methods**

Co-immunoprecipitation, GST-pull down, TurboID proximity-dependent biotinylation, and docking simulation analysis were used to investigate and map the interaction between Twist1, p53 and MDM2. Twist1 loss-of-function (shRNA, CRISPR/Cas9) and gain-of-function (ectopic expression) sarcoma cell models were generated. MDM2i sensitivity of the generated cell models was evaluated in vitro and in vivo in mouse xenografts.

**Results and Discussions**

Co-immunoprecipitations experiments, both using ectopically expressed genes as well es endogenous proteins, show that Twist1 binds both p53 and MDM2. Twist:MDM2 binding occurs also in p53 null cells, indicating that this interaction is independent of p53. The interaction is direct, as demonstrated by GST pull downs, and occurs under para-physiological conditions, as shown by TurboID proximity-dependent biotinylation experiments. Modulation of Twist1 expression in p53 wild type/MDM2 overexpressing sarcoma cell models affects p53 and the response to MDM2i (Nutlin-3a, Milademetan/DS-3032, Idasanutlin/RG7388, SAR405838). Specifically, Twist knock-down/knock-out associates with increased p53 levels and enhanced sensitivity to MDM2i in different sarcoma cell models, both in vitro and in vivo. Conversely, ectopic Twist1 expression attenuates MDM2i efficacy. This effect depends on Twist1 binding to p53 and MDM2, as a Twist1 mutant defective for this binding loses the inhibitory capacity.

**Conclusion**

Twist forms a p53:Twist:MDM2 trimeric complex and, by enhancing MDM2-mediated degradation of p53, attenuates the efficacy of MDM2i

### EACR2024-0981
### Non-permissive basal progenitor cell population requires SOX2 activation to unlock tumour growth

P. Centeno[1,2], C. Chester[2], C.A. Ford[1], K. Gilroy[1],

© 2024 The Authors. Molecular Oncology is published by John Wiley & Sons Ltd on behalf of Federation of European Biochemical Societies

T. Jamieson[1], P. Cammareri[1,3], G.J. Inman[4], R. Marais[2,5], A.D. Campbell[1], O.J. Sansom[1]
[1]CRUK Scotland Institute, Colorectal Cancer and Wnt Signalling Group, Glasgow, UK
[2]CRUK Manchester Institute, Molecular Oncology, Manchester, UK
[3]CRUK Scotland Centre, Institute of Genetics and Cancer- University of Edinburgh, Edinburgh, UK
[4]CRUK Scotland Institute, Growth Factor Signalling and Squamous Cancers, Glasgow, UK
[5]Oncodrug Ltd, Alderley Park, Manchester, UK

**Introduction**

Cutaneous squamous cell carcinoma (cSCC) is an escalating global health challenge, with an unparalleled surge of over 310% from 1990 to 2017. However, this figure underestimates the real prevalence due to the underreporting in cancer registries grappling with the multitude of cases. cSCC has a diverse origin, and the precise identity and features of the cancer cell of origin remain elusive and open for debate.

**Material and Methods**

We have developed mouse models expressing the potent oncogene BRAFV600E in two different keratinocyte populations (Ivl+ and K14+/K5+) to study cell-specific susceptibility to oncogene transformation. We measured tumour onset and progression followed by tumours immunophenotyping and bulk transcriptomic profiling. We also employed a human cSCC dataset to validate findings from the mouse models.

**Results and Discussions**

Ivl+ and K14+/K5+ progenitor cell types coexist in the basal layer and give rise to differentiated cells that migrate to the suprabasal layers. Despite these shared characterises, they show profound differences in susceptibility to transformation. Krt14/Krt5+ cells displayed permissibility to tumour development, with rapid growth onset (10±1 days), contrasted with Ivl+ cells (135 days) and a slower growth rate. However, once the tumours developed and lost hierarchical epidermal organisation, their histopathological features were indistinguishable. Similarly, both transcriptomic profiles show activation of wound healing, skin embryonic development, and tumour-specific keratinocyte signatures. Notably, SOX2 emerged as a key transcriptional difference being highly expressed in Ivl+ tumours, where it appears to rewire cells for tumour initiation and growth. Meanwhile, SOX2 expression was negligible in normal skin during homeostasis or in tumours of Krt5/Krt14+ origin. Human cSCC dataset analysis classified 25% of samples as high SOX2 expression, underscoring diverse cell origins and a link between development and oncogenic signalling.

**Conclusion**

Our mouse models recapitulated cSCC-associated pathways and transcriptional profiles observed in human cSCC. SOX2 emerged as a critical epigenetic regulator required to unlock tumour growth from basal Ivl+ cells, and it serves as a marker to identify and classify cSCC according to cell of origin. Further characterisation of the cSCC cell of origin and its vulnerabilities will allow the development of targeted therapeutic strategies to contain tumours at its source.

### EACR2024-0982
### Distrupting Hypoxia-Induced Metabolic Adaptation in Oral Cavity Carcinoma Cells: Identifying the Potential of Rivaroxaban as an Anti-Coagulant Agent

M.E. Kasap[1], O. Bayrak[2], F.A. Bayrak[3], Y. Basbinar[2], S. Bayrak[2]
[1]Institute of Health and Sciences, Department of Translational Oncology, İzmir, Turkiye
[2]Institute of Health Sciences, Department of Translational Oncology, Izmir, Turkiye
[3]Faculty of Medicine, Department of Otolaryngology, Izmir, Turkiye

**Introduction**

Cancer cells are known to exhibit metabolic adaptations in hypoxic environments. It is believed that transcription factors that are members of the HIF family are the key to this adaptability. Although metabolic adaptability keeps cancer cells alive, it also gives them an aggressive phenotype and reduces the effectiveness of treatments. In this work, we used a oral cavity cancer cell-line that is known to be resistant to a hypoxic microenvironment in an effort to contribute to new treatment approaches.

**Material and Methods**

In order to investigate the effects of rivaroxaban on the UPCI-SCC-131 Oral Cavity cancer cell line under normoxic and hypoxic conditions, this study used viability analysis, wound healing experiments, and immunofluorescence to measure the expression levels of HIF-1 alpha, GLUT-1, and LDHA. A method of chemical hypoxia using sodium sulfite, which had been optimised in our previous studies, was used to create the hypoxic conditions.

**Results and Discussions**

Cell viability decreased in the hypoxic condition compared to the normoxic condition as the rivaroxaban concentration increased. In parallel, when analysing wound healing results, it was observed that wound opening did not change in the hypoxic group, whereas wound closure occurred in the normoxic group. Furthermore, immunofluorescence staining results showed that E-cadherin, LDH-A, HIF-1 alpha and GLUT-1 expression levels, which were observed to increase in hypoxia compared to normoxia, decreased after rivaroxaban treatment in hypoxia.

**Conclusion**

The suppression of HIF-1 alpha, which is essential for enhanced metabolic adaptability in hypoxic environments, was found to produce positive feedback loops in cell behaviour, as per our data. The augmentation of pro-coagulant activity in the tumor microenvironment was assumed to be caused by rivaroxaban, an inhibitor of FXa, which inhibits HIF-1 alpha. In the hypoxic tumor microenvironment, rivaroxaban might be a novel target in this situation.

*O.B. was supported by TUBITAK 2211C Domestic Priority Doctoral Scholarship Program, and Council of Higher Education 100/2000 scholarship in priority field program. The study is funded by Dokuz Eylul University Scientific Research Projects Coordination Unit with project number TSG20222576.*

### EACR2024-0987
### Identification of therapy-induced

© 2024 The Authors. Molecular Oncology is published by John Wiley & Sons Ltd on behalf of Federation of European Biochemical Societies

### senescent targets in non-Hodgkin lymphoma

J. Balibrea Rull[1], C. Guardia[1], D. Olivares[1], V. Rodilla[1]
[1]Josep Carreras Leukaemia Research Institute, Cancer Heterogeneity and Hierarchies, Badalona, Spain

**Introduction**

Non-Hodgkin lymphoma (NHL) constitutes 90% of all lymphomas, and despite its heterogeneity, the standard treatment remains being R-CHOP, a combination of chemotherapy; cyclophosphamide, doxorubicin and vincristine (CHO); with rituximab. However, relapse affects 30-40% of patients, underscoring the need to understand treatment resistance mechanisms. Senescence, recognized as a cancer hallmark, is induced by chemotherapy, termed therapy-induced senescence (TIS). Although it halts cell cycle progression, senescent cells (SCs) may promote tumorigenesis through traits like resistance to apoptosis and the senescence-associated secretory phenotype (SASP). Identifying senescence targets is crucial due to its role in cancer and the lack of reliable biomarkers. Recent preclinical studies demonstrate promising outcomes in mice treated with chemotherapy and senolytics, targeting SCs in both tumor and non-tumor tissues. Our study aims to discover biomarkers and senescence targets in NHL, developing methods to selectively eliminate SCs, thereby restraining tumor progression and preventing relapse.

**Material and Methods**

We characterized TIS in NHL cells using three cell lines representative of different NHL subtypes, treated with the chemotherapeutic regimen CHO. We assessed senescence induction via RT-qPCR for classical senescence markers and SA-βgal staining. Through comprehensive transcriptomic and proteomic analyses, we aimed to pinpoint common differentially expressed candidates as potential senescence targets. Validation of these candidates was primarily conducted using immunocytochemistry, Western blot, and flow cytometry. In vivo studies are anticipated to use PDX models of NHL.

**Results and Discussions**

Our initial findings from TIS characterization in NHL cells showed distinct senescence induction across the cell lines, evidenced by SA-βgal staining and upregulation of senescence markers. Subsequent analyses revealed a set of differentially expressed potential targets, which we further validated in vitro. Moreover, we have evaluated different strategies to inhibit these senescence markers and their consequences. With the idea of stratifying patients that could benefit from these novel regimens, we have correlated the expression of certain genes in peripheral blood in response to CHO.

**Conclusion**

Integrating transcriptomic data and validation studies, we strive to identify and evaluate senescence targets, potentially offering new therapeutic avenues to disrupt pro-tumorigenic pathways and enhance treatment responses in NHL.

### EACR2024-0994
### Impact of Stem Cell Yield on Outcomes and Toxicity in Autologous Stem Cell Transplantation - an exploratory study from a regional cancer care centre in South India

S.C. Saldanha[1]
[1]Kidwai Memorial Institute Of Oncology, Medical Oncology & Bonemarrow Transplantation, Bengaluru, India

**Introduction**

Autologous Stem Cell Transplantation (ASCT) is part of standard of care for many hematologic diseases. Patients undergo multiple chemotherapy before ASCT, impacting their stem cell yield. This study looks at the relationship between stem cell yield and associated toxicity and outcomes in ASCT patients.

**Material and Methods**

A retrospective case record analysis was done of 45 ASCT patients from June 2022 to January 2024, at Kidwai Memorial Institute of Oncology, Bengaluru. Granulocyte-Colony Stimulating Factor (GCSF) for 5 days, starting at 10 mcg/kg daily, with 0.24 mg/kg of Inj. Plerixafor for stem cell mobilization before apheresis and additional apheresis if CD34+ cell count was ≤2 million/kg were given. Multiple myeloma patients received either the MEL200 or MEL140 regimen, and lymphoma patients received BEAM protocol. Neutrophil engraftment was considered a neutrophil count of >500 cells/uL for 3 consecutive days, and platelet engraftment was a platelet count >20,000 cells/uL for 3 consecutive days without needing transfusion. Infections & complications were managed with institutional guidelines.

**Results and Discussions**

Patient median age was 45 yrs (range 17-72 yrs) and 68% (31/45) were males. Transplant reasons included multiple myeloma (23), Hodgkin's lymphoma (10), diffuse large B-cell lymphoma (3), peripheral T-cell lymphoma (3), and other indications (6). Most got ≥2 lines of chemotherapy before transplant. Average stem cell yield post apheresis was 11 million/mm^3 (range 2.2 to 21.9 million/mm^3). The median duration of GCSF use after ASCT was 9 days and average hospital stay was 20 days. 41 patients achieved complete remission or very good partial remission. The mean neutrophil and platelet engraftment time was 10 days. 82% (32/45) developed diarrhea during hospital stay, with 28.9% (13/45) having grade III/IV diarrhea. Patients with higher stem cell yield had earlier neutrophil (r=-0.301, p=0.007) and platelet (r=-0.463, p=<0.001) engraftment time. Patients with lower stem cell yields had longer hospital stays (r=-0.144, p=0.030) and received GCSF injections (r=-0.212, p=0.048) for a longer duration. A higher yield of stem cells had a lower incidence of grade III/IV diarrhea (p=<0.001) and lower incidence of febrile neutropenia (p=0.017).

**Conclusion**

Optimizing stem cell mobilization strategies is crucial since higher stem cell yield is associated with favorable outcomes including earlier engraftment, reduced hospital stay and decreased incidence of severe toxicity like diarrhea and febrile neutropenia.

© 2024The Authors. Molecular Oncology is published by John Wiley & Sons Ltd on behalf of Federation of European Biochemical Societies

### EACR2024-0998

### New therapeutic strategies for soft tissue sarcomas using a CDK4/6 inhibitor

R. López-Alemany[1], H. Jaara[2], M. Rashidi[2], B. Willis[2], O.M. Tirado[3], J. García del Muro[4]
[1]IDIBELL- Bellvitge Research Institute, ONCOBELL- Sarcoma Research Group, L'Hospitalet de Llobregat, Spain
[2]IDIBELL, Oncobell- Sarcoma research Group, L'Hospitalet de Llobregat, Spain
[3]IDIBELL, Oncobell- Sarcoma Research Group, L'Hospitalet de Llobregat- Barcelona, Spain
[4]Catalan Oncology Institute- ICO, Unidad Multi-disciplinar de Sarcomas, L'Hospitalet de Llobregat- Barcelona, Spain

**Introduction**

Soft Tissue Sarcomas (STS) are a group of heterogenous malignancies from mesenchymal origin with bad prognosis and few effective treatments. Cell cycle is a complex biological process controlled by cyclins and cyclin-dependent kinases (CDKs), valuable targets for cancer treatment. Recent studies have shown that Rb1, CDK4 and Cyclin E appears among the most prevalent somatic mutaions in STS patients. Our objective is to evaluate the effect of pharmacological inhibition of CDK4 in sarcoma models.

**Material and Methods**

In the present study we have used Palbocibclib, a CDK4/6 inhibitor, in several STS cell lines: 402.91 (myxoid liposarcoma), SKL-MS-1 (leiomyosarcoma), SW982 (synovial sarcoma) and HT-1080 (fibrosarcoma) to evaluate its effect on the neoplastic phenotype in those cell lines.

**Results and Discussions**

Our results indicated that the 4 cell lines tested expressed CDK4, as shown by western blotting. Palbociclib treatment inhibited the ability to form colonies in all cell lines and also cell proliferation, being SW982 and HT-1080 cell lines more sensible to CDK4/6 inhibition than SK-LMS-1 and 408.91 cells. Palbociclib effect on cell viability was not due to an increase in cell death or in apoptosis induction, as it did not result in Caspase-3 activation. Flow citomery analysis confirmed that Palbociclib treatment induce a cell cycle arrest in G1 phase. Accordingly, Palbociclib treatment resulted in a reduction of Rb1 phosphorylation and an inhibition of CDK2 expression. Several drugs were tested searching for a synergistic effect with Palbociclib. The combination of Palbociclib and Docetaxel affected the cell viability significantly more than each drug alone, indicating a synergistic effect of both drugs. Docetaxel is a taxane whose primary target is β-tubulin of polymerized microtubules and suppress microtubule dynamics impairing mitosis and is considered as second-line therapy for metastatic STSs.

**Conclusion**

Our results indicate that Palbociclib inhibits cell growth and induce cell cycle arrest without affecting cell viability in several STS cell lines. Palbociclib could be used as a treatment for STS, in monotherapy or in combination with other drugs. Palbociclib is an FDA-approved CDK4 inhibitor for the treatment of other cancers as breast cancer. The advantage of using approved drugs is that after validation in animal models, it is possible to move reach to Phase II clinical trial. As such, this treatment can reach the clinical practice sooner and increase the overall survival of advanced STS patients.

### EACR2024-1000

### LncRNA AFAP-1 is overexpressed in metastatic breast tumors and associated to vasculogenic mimicry

C. Lopez-Camarillo[1], A.P. García-Hernández[1], M. Sierra-Martínez[2], D. Núñez-Corona[1], E. Ibarra-Sierra[3]
[1]Universidad Autonoma de la Ciudad de Mexico, Genomics Sciences program, Mexico, Mexico
[2]Hospital Regional de Alta Especialidad de Ixtapaluca- Estado de Mexico- México, Unidad de Investigacion en Salud, Estado de Mexico, Mexico
[3]Instituto Estatal de Cancerologia Dr. Arturo Beltran Ortega, Departamento de investigacion, Mexico, Mexico

**Introduction**

In vivo, tumor cells may organize in three-dimensional (3D) channels-like structures denoted as vasculogenic mimicry (VM), which provides a novel route for nutrients and oxygen acquisition. VM is activated by hypoxia and associated with metastasis and poor prognosis. LncRNAs may regulate genes involved in metastasis, however, if they also control VM in breast cancer remains elusive. The aim of this study was to evaluate the expression of VM-associated lncRNAs in breast tumors that developed metastasis. Moreover, we performed the functional analysis of AFAP-1 an lncRNA overexpressed in metastatic breast tumors and associated with VM.

**Material and Methods**

Tumors were collected from breast cancer patients after signed agreement. The study was approved by the Ethics Committee of the Hospital Regional de Alta Especialidad Mexico (number NR-16-2020). The presence of VM and blood vessels in tumors was quantified by immuno-histochemistry using CD31/periodic acid Schiff staining. RT-PCR assays were performed to quantify the expression of lncRNAs associated with VM. VM experiments in vitro were performed using 3D cultures under hypoxia conditions. The analysis of signaling pathways was performed using bioinformatic software's. Knockdown of AFAP-1 lncRNA was performed using specific siRNAs. One-way analysis of variance followed by Tukey's test were used. A $p < 0.05$ was considered as statistically significant.

**Results and Discussions**

Our results showed that NEAT1, XIST, MALAT1, HOTAIR, AFAP-1 and CDC5 lncRNAs were overexpressed in metastatic breast tumors and showed interactions with microRNAs regulating mRNAs involved in cell invasion and VM. Our data showed that VM was increased in tumors from patients with metastasis in comparison with no-metastatic group. Gene expression analysis indicated that AFAP-1 and CDC5 lncRNAs were upregulated in primary tumors from patients with metastasis and positive for VM. Moreover, AFAP-1 showed a predictive clinical value in overall survival. The role of AFAP-1 in formation of hypoxia-

Molecular Oncology 18 (Suppl. 1) (2024) 1–495
© 2024 The Authors. Molecular Oncology is published by John Wiley & Sons Ltd on behalf of Federation of European Biochemical Societies induced 3D channels-like was evaluated using an in vitro model that recapitulates the early stages of VM. Data showed that knockdown of AFAP-1 was able to abolish the VM development in both metastatic Hs578t and MDA-MB-231 breast cancer cells.

**Conclusion**

In conclusion, manipulation of AFAP-1 levels may represent a therapeutic approach in metastatic breast cancer patients that developed VM.

*This research was funded by CONAHCYT, Mexico, Grant A3-S-33674. The authors declare that they have no competing interests.*

### EACR2024-1004
### Transient reprogramming in cancer cells drives senescence and apoptosis inhibiting lung tumorigenesis

*D. Muñoz-espin[1], P. Pedrosa[2], Z. Zhang[3], D. Macias[3], J. Ge[3], M. Denholm[3], A. Dyas[3], J.E. Martin[4], S. Da Silva-Alvarez[2], M. Collado[2]*
*[1]University of Cambridge, Oncology, Cambridge, UK*
*[2]Health Research Institute of Santiago de Compostela ID IS, University of Santiago de Compostela, Santiago de Compostela, Spain*
*[3]Early Cancer Institute- University of Cambridge, Oncology, Cambridge, UK*
*[4]Centro of Molecular Diagnostics Laboratories CMDL-University of Cambridge, Oncology, Cambridge, UK*

**Introduction**

Oncogenic transformation and OSKM-mediated induction of pluripotency are two independent and incompatible cellular fates. While continuous expression of OSKM can convert normal somatic cells into teratogenic pluripotent cells, it remains speculative what is the impact of transient OSKM expression in cancer cells.

**Material and Methods**

We have further explored this relationship between oncogenic transformation and cell reprogramming by analyzing the effect of transient expression of the 4 Yamanaka reprogramming factors (Oct4, Sox2, Klf4 and cMyc) on human A549 and mouse L1475 KP lung cancer cells. Our experimental settings incorporate a variety of functional analyses, molecular characterisation by high throughput transcriptomic and proteomic profiles, histopathology analyses, and validation/proof-of-concept with a number of models of lung carcinogenesis. These include allografts and orthotopic transplantation of murine L1475 KP cells, and a new genetically-engineered reprogrammable mouse model (OSKM; KrasG12V mice) expressing OSKM in a doxycycline-dependent manner and activating oncogenic KrasG12V in the lung by intratracheal AdFLP administration.

**Results and Discussions**

Here, we find that transient OSKM expression limits the growth of transformed lung cells by inducing apoptosis and senescence. We identify Oct4 and Klf4, but not Sox2 and cMyc, as the main individual reprogramming factors responsible for this effect. Mechanistically, the induction of cell cycle inhibitor p21 downstream of the Oct4 and Klf4 reprogramming factors acts as mediator of cell death and senescence. Using a variety of in vivo systems, including allografts, orthotopic transplantation and KRAS-driven lung cancer mouse models, we demonstrate that transient reprogramming by OSKM expression in cancer cells impairs tumour growth and reduces tumour burden.

**Conclusion**

Altogether, these results show that the induction of transient reprogramming in cancer cells does not lead to pluripotency but is antitumorigenic. Our identification of key regulatory elements of cancer cell identity can be instrumental for the discovery of novel vulnerabilities and open novel potential therapeutic avenues in oncology.

### EACR2024-1009
### Lactobacillus salivarius as an Adjuvant to Antitumor Effect of Paclitaxel on Oral Squamous Cell Carcinoma

*D. Mitic[1], M. Lazarevic[1], M. Jaksic Karisik[1], J. Milasin[1]*
*[1]School of Dental Medicine- University of Belgrade, Implant-research Center, Belgrade, Serbia*

**Introduction**

Oral squamous cell carcinoma (OSCC) remains a significant global health concern, necessitating continuous exploration of adjunctive therapies to improve treatment outcomes. Paclitaxel, or taxol, is an anti-microtubule agent that binds to microtubules during cell division and induces apoptosis in various tumor cell types. However, its efficacy can be limited by factors such as drug resistance and adverse effects. Probiotics, particularly *Lactobacillus salivarius* (*L.s.*), have shown potential in enhancing anticancer treatment efficacy through modulation of the tumor microenvironment and immune response. This study investigated the synergistic effects of *L. s.* in combination with paclitaxel treatment on OSCC cell line.

**Material and Methods**

SCC-25 cell line was used, and as addition its' CD44 positive subpopulation. Cells were seeded in 96-well plates in concentration 10.000 cells/well. The next day complete medium was discarded and $1\times10^6$/well of *L. salivarius* were added in corresponding wells. After 4 hours of incubation medium was discarded form all wells, and washed with PBS. Paclitaxel (0.1 μg/mL) was added in corresponding wells. Mitochondrial activity assay was performed after 1, 3 and 7 days. As a control, cells grown in complete medium with addition of DMSO in concentration used to dissolve paclitaxel was used. All experiments were done in triplicate, in two separated experiments.

**Results and Discussions**

On line SCC-25, after one day there were no difference in mitochondrial activity of the groups. On day 3 and day 7, mitochondrial activity was significantly reduced in group Paclitaxel+*L.s.* , $p<0.05$ and $p<0.01$, respectively, in comparison to paclitaxel alone. On separated CD44+ SCC-25 cell line, significant reduction was observed on day 7, $p<0.001$, in group Paclitaxel+*L.s.*, in comparison to drug alone. Synergistic effect of paclitaxel and probiotic lead to a significantly enhanced antitumor effect compared to drug alone. Further, on CD44+ subpopulation, more resistant cancer stem cells, adjuvant effect of probiotic become relevant on day 7.

**Conclusion**

Our study underscores the importance of exploring complementary approaches to traditional cancer

© 2024The Authors. Molecular Oncology is published by John Wiley & Sons Ltd on behalf of Federation of European Biochemical Societies treatments and highlights the promising role of probiotics in improving therapeutic outcomes for oral cancer patients. Further research should provide insights into mechanism of antitumor action of probiotics.

### EACR2024-1021
### Catalysing Synergistic Strategies: Molecular Docking Insight and Multitargeted Tocotrienol Integration in Optimising Colorectal Cancer Treatment

*S.H.S. Loh[1], Z.H. Yap[1], A.S.Y. Kong[1], S. Maran[2], J.Y. Fu[3], K.S. Lai[4], S.M. Then[5]*
*[1]University of Nottingham Malaysia, School of Biosciences, Semenyih- Selangor, Malaysia*
*[2]Monash University Malaysia, School of Pharmacy, Bandar Sunway- Selangor, Malaysia*
*[3]Malaysian Palm Oil Board, Nutrition Unit, Kajang-Selangor, Malaysia*
*[4]Abu Dhabi Women's College- Higher Colleges of Technology, Faculty of Health Sciences, Abu Dhabi, United Arab Emirates*
*[5]University of Nottingham Malaysia, Division of Biomedical Sciences, Semenyih- Selangor, Malaysia*

**Introduction**
Colorectal cancer (CRC) presents a significant global health challenge, with notably high mortality rates among Chinese Malaysians. This study investigates the molecular intricacies of CRC, with a specific focus on the pivotal role of BCL-2 family proteins in disease progression. Employing a molecular docking simulation approach, the study aims to elucidate the interaction dynamics of BCL-2 proteins with potential therapeutic agents. Additionally, in vitro studies integrate Tocotrienol (T3) isomers with Fluorouracil (5-FU) and Doxorubicin (Dox), seeking to uncover synergistic therapeutic benefits for CRC.
**Material and Methods**
The structural data for the preliminary molecular docking simulation were sourced from established databases, and protein-ligand interaction analyses were conducted using AutoDock Tools. In vitro studies assessed the therapeutic potential of T3 in combination with 5-FU and Dox on Caco-2 and SW48 cell lines. These studies investigated various anticancer effects, including morphological changes, DNA damage, cell cycle alterations, and protein expression profiles related to apoptosis and autophagy.
**Results and Discussions**
Strong binding interactions were observed between the ligands and target proteins, with T3 showing promising affinity, especially with mutated BCL-2 family proteins. Mutations influenced protein-ligand interactions, indicating potential alteration in therapeutic efficacy. The combination treatment exhibited pharmacological synergism, inducing DNA breaks, apoptosis, and cellular stress, with mitochondria emerging as a critical target. Activation of caspase-3 and downregulation of Inhibitor of Apoptosis (IAP) proteins, especially Livin and XIAP underscore a chemosensitisation effect, while autophagy induction presents a novel therapeutic avenue.
**Conclusion**
Molecular docking simulation provides valuable insights into potential mechanisms controlling CRC invasiveness, while in vitro studies demonstrate the efficacy of T3 in synergistic CRC treatment strategies. Ongoing investigations into key proteins of apoptotic and autophagic pathways offer valuable implications for CRC management. These findings, derived from multitargeted approaches, hold promise for developing innovative therapeutic interventions to combat CRC and enhance patient outcomes.

### EACR2024-1032
### Therapeutic combination with Gossypol to overcome adaptation of Glioblastoma Multiforme to Temozolomide

*P. Alfonso Triguero[1], N. Gispets[2], M. Alfonso-Triguero[3], M. Mulero-Acevedo[2], P. Melón[2], A. Arranz[3], A.P. Candiota[4], F. Novio[5], J. Lorenzo[2], D. Ruiz-Molina[1]*
*[1]Catalan Institute of Nanoscience and Nanotechnology ICN2- CSIC and BIST, Nanostructured functional Materials group, Barcelona, Spain*
*[2]Instituto de Biotecnología y Biomedicina IBB, Bioquímica y Biología Molecular, Barcelona, Spain*
*[3]Achucarro Basque Center for Neuroscience, Laboratory of Humanized Models of Disease, Bilbao, Spain*
*[4]Facultad de Biociencias. Universidad Autónoma de Barcelona UAB, Bioquímica y Biología Molecular, Barcelona, Spain*
*[5]Facultad de Química. Universidad Autónoma de Barcelona UAB, Química, Barcelona, Spain*

**Introduction**
Glioblastoma Multiforme (GBM) stands out as the most prevalent and aggressive primary brain tumor. Despite receiving standard care treatment, which includes Temozolomide (TMZ) chemotherapy, the prognosis for GBM patients remains discouraging. In a previous study, an immune-enhanced metronomic schedule (IMS) protocol was implemented, involving TMZ administration every 6 days, designed to be compatible with immune system function. Results revealed substantial improvement in the survival of mouse models with GBM, surpassing the efficacy of standard TMZ treatment. Another study demonstrated that Gossypol (GSP) is one of the few pharmacological agents capable of activating the DFF40/CAD nuclear protein in GBM-affected cells, triggering apoptosis appropriately. Based on the presented data, it is suggested that the combination of TMZ and GSP administered according to the IMS protocol could have a positive impact on the survival of GBM patients.
**Material and Methods**
Two commercial human GBM cell lines and one murine GBM cell line (GL261) were used. Additionally, two non-commercial GBM cell lines and a non-cancerous human astrocytic cell line (hiPSC-astrocytes) were included. Cell death assays were conducted to assess the effects of TMZ and GSP combination. Immunoblotting assays were also performed to understand the underlying molecular mechanisms of TMZ and GSP combination effect. These in vitro results were validated *in vivo* in an orthotopic C57BL/6j mouse model, implanted with GL261 cells. Tumor mass volume analysis was conducted using magnetic resonance imaging.
**Results and Discussions**

© 2024 The Authors. Molecular Oncology is published by John Wiley & Sons Ltd on behalf of Federation of European Biochemical Societies

Cell death analyses indicated that TMZ and GSP synergistically interacted to increase cell death in all evaluated GBM cell lines. However, this synergy was not observed in hiPSC astrocytes. Immunoblotting assay results revealed that GSP could synergistically interact with TMZ by inhibiting anti-apoptotic proteins**.** Finally, in vivo experiments demonstrated that intragastric joint administration of TMZ and GSP, via the IMS protocol, significantly prolonged survival in the C57BL/6j mouse model implanted with GL261 cells compared to monotherapy treatments with TMZ and GSP.

**Conclusion**

GSP exhibits considerable potential as a pharmacological agent against GBM due to its synergistic capacity when combined with TMZ and its selective properties, as it does not seem to manifest a synergistic effect with TMZ in astrocytes**.** Joint intragastric administration of TMZ and GSP, according to the IMS, could constitute an improvement in GBM treatment.

### EACR2024-1036

### Limiting radiation-induced lung injury by modulating the caveolin-senescence-axis

*M. Buchholzki[1], L.M. Stasch[1], Z. Sevendik[1], V. Jendrossek[1], D. Klein[1]*

*[1]University Hospital Essen, Institute of Cell Biology Cancer Research, Essen, Germany*

**Introduction**

For effective and tolerable tumor therapies, the simultaneous consideration of the radiation response in tumor and normal tissue is essential. On one hand radiation-induced senescence of lung epithelial cells and the associated development of a senescence-associated secretory phenotype determine the normal tissue toxicity after thoracic irradiation. On the other hand, it is known that the membrane protein caveolin-1 (CAV1) is a crucial regulator of lung damage and acts as a main regulator of cellular senescence. However, radiotherapy (RT)-induced CAV1 alterations as well as the role of CAV1 for RT-induced senescence remain to be clarified.

**Material and Methods**

The impact of CAV1 expression levels on RT-induced normal tissue damage was investigated in a preclinical mouse model of RT-induced pneumopathy using wild-type (WT) and CAV1-deficient littermates. The impact of RT on cellular features was further investigated in primary bronchial epithelial cells, lung endothelial cells and fibroblasts in a CAV1-dependent fashion to complementary complete and further specify a potential CAV1-senescence-axis using short-term (cell-cycle, cell viability, apoptosis) and long-term (survival, senescence, colony formation) assays. Beside single cell responses the focus was made on the cellular cross talks by using co-culture systems of different normal lung cells.

**Results and Discussions**

Whole thorax radiation causes a decline of CAV1 in lungs of WT mice, an effect that came along with RT-induced senescence in bronchial epithelium. While senescence could not be detected in endothelial cells the impact of senescent fibroblasts, particularly in fibrosis-prone CAV1-deficient animals, remains to be clarified in current investigations. In vitro, RT induced senescence of lung epithelial cells could not be linked to declining CAV1 levels, even so not in endothelial cells, while CAV1 levels decline in fibroblasts. CAV1-deficient fibroblasts however showed less senescence induction following RT, although these cells are considered to be more radioresistant. Modulation of RT-induced senescence either by senolytic or/and Cav1-targeting agents is currently investigated in more complex epithelial-fibroblasts co-cultures to specify radioprotective treatment strategies.

**Conclusion**

The connection between CAV1 regulations and senescence remains contractionary and maybe tissue and/or cell-type-dependent. Herein the spatiotemporal regulation of CAV1 could be decisive for the onset of (RT-induced) cellular senescence.

### EACR2024-1037

### The effect of genetic attenuation of ALDH1A1 overexpression in a novel patient-derived colorectal cell line; its traits in vitro and in vivo; and genomic characterisation

*M. Poturnajova[1], Z. Kozovska[1], S. Gulati[1], P. Makovicky[1], O. Pos[2], M. Hrnciar[2], T. Szemes[2], M. Tomas[3], P. Dubovan[4], M. Matuskova[1]*

*[1]Cancer Research Institute- Biomedical Research Center of Slovak Academy of Sciences, Department of Molecular Oncology, Bratislava, Slovakia*

*[2]Comenius University, Science Park, Bratislava, Slovakia*

*[3]National Cancer Institute, Clinic of surgical oncology, Bratislava, Slovakia*

*[4]National Cancer Institute, Clinic of chirurgical oncology, Bratislava, Slovakia*

**Introduction**

Colorectal cancer (CRC) stands for the death of 930,000 patients annually. New models are needed to study genotype/phenotype correlations. Aldehyde dehydrogenase 1A1 is a stem cell marker with a broad spectrum of biological processes essential for cell survival and protection. It has antioxidant and osmoregulatory functions and detoxicates potentially hazardous aldehydes. It converts vitamin A to retinoic acid (RA). ALDH1A1 overexpression is correlated with a poor prognosis in patients and a rise in proliferation, resistance to treatment, and tumorigenicity.

**Material and Methods**

We established a patient-derived cell line and examined its surface markers, proliferation capacity, response to chemotherapy and migration potential. Genome and transcriptome were characterised by karyotype analysis, copy number variant (CNV) analysis and RNAseq. The ALDH1A1 gene editing was done by CRISPR/Cas9. The ALDH1A1 knockouts were confirmed by sequencing, RT-qPCR expression and ALDEFLUOR™ assay. By transwell invasive and scratch assays, we compared the invasive potential of ALDH1A1 knockouts vs. parental cells. Tumorigenicity and metastatic potential were examined in vivo.

**Results and Discussions**

We confirmed the high proliferative activity and tumorigenic potential of a novel epithelial cell line from moderately differentiated rectal adenocarcinoma. The

© 2024 The Authors. Molecular Oncology is published by John Wiley & Sons Ltd on behalf of Federation of European Biochemical Societies

CNV analysis revealed a broad spectrum of chromosomal aberrations between the original tumour and the cell line. The overexpression of aldehyde dehydrogenase 1A1 isoform (ALDH1A1) was detected. Genetic silencing of ALDH1A1 expression led to changes in biological and molecular properties. The most significant was an increase in invasive and metastatic potential and a decrease in the proliferation capacity of subcutaneous xenografts. RNA sequencing revealed the changes in the expression of genes associated with the inhibition of proliferation and promotion of invasion and metastases.

**Conclusion**

In a novel colorectal cell line, we obtained a valuable model for studying the role of ALDH1A1 in tumour biology and the metastatic process. Gene editing of ALDH1A1 using CRISPR-Cas9 led to functional, cellular and molecular changes confirming the role of ALDH1A1 in carcinogenesis and metastatic potential.

*Financial support: This work was supported by Slovak Research and Development Agency under contract APVV-21-0296; by VEGA 2/0178/21 and 2/0170/22; by Alliance4Life_ACTIONS 012722, and by Slovak Cancer Research Foundation.*

### EACR2024-1049
### Derivation and long-term culture of patient-derived tumoroid lines in a serum- and conditioned medium-free system

*C. Yankaskas[1], B. Balhouse[1], C. Paul[1], S. Salen[1], P. Shahi Thakuri[1], S. Beam[1], M. Kennedy[1], M. Dallas[1], D. Kuninger[1]*

*[1]Thermo Fisher Scientific, Cell Biology, Frederick, United States*

**Introduction**

Traditional 2D cancer cell lines often fail to accurately model primary cancer cells, hindering the translation of in vitro research to the clinic. An emerging solution is the use of patient tissue-derived cells expanded in 3D, known as cancer organoids or tumoroids. To facilitate the adoption of these more relevant models, we developed Gibco™ OncoPro™ Tumoroid Culture Medium, the first serum- and conditioned medium-free system for both deriving tumoroid models from a variety of cancers and supporting the culture of tumoroids established in other media.

**Material and Methods**

Tumoroid lines were derived from colorectal, lung, breast, and endometrial cancer tissue by supplementing OncoPro medium with indication-specific growth factors. Tumoroids were cultured for up to 50 passages, with microscopy, cell counts, and DNA/RNA sequencing used to assess morphology, growth rate, genomic mutations, and gene expression patterns. Simultaneously, colorectal, lung, pancreas, breast, and head and neck tumoroid models from the National Cancer Institute Patient-Derived Models Repository (PDMR), which were established in medium containing Wnt-3A, R-spondin 3, and/or Noggin, were cultured in OncoPro and PDMR-recommended media. PDMR tumoroid cultures were then monitored via microscopy, cell counts, and DNA/RNA sequencing.

**Results and Discussions**

Newly derived tumoroid cultures adopted donor-specific morphologies that were maintained during long-term culture. Population doubling times stabilized within the first few passages, were donor-dependent, and averaged 65-100 hours. Critically, tumoroid lines maintained their gene expression patterns across 20,000 human genes during long-term culture, with correlations between tumor and late-passage samples of >0.8. The allelic frequency of single nucleotide variations in 161 cancer-related genes was also tightly correlated (R>0.9 for nearly all samples) between tumor and late-passage tumoroids. Culture of PDMR models in OncoPro medium was similarly successful, with high correlations in gene expression and mutational profiles across media systems.

**Conclusion**

Altogether, tumoroid derivation and culture in this novel medium enables the long-term preservation of patient-specific cellular genotype and phenotype, which should allow for expansion, biobanking, and performance of experimental repeats within the same patient tissue-derived cultures across labs and over time.

### EACR2024-1051
### Loss of RIMKLA sensitises cancer cells to hypoxia

*A. Patsalias[1], C. Zhou[1], E. Hammond[1]*

*[1]University of Oxford, Department of Oncology, Oxford, United Kingdom*

**Introduction**

*RIMKLA* encodes the enzyme N-acetyl-aspartyl-glutamate synthetase II (NAAGS II) which together with its paralogue, *RIMKLB*, control the synthesis of N-acetyl-aspartyl-glutamate (NAAG), an acetylated dipeptide, previously known to be one of the most abundant neuro-transmitters in the mammalian central nervous system. However, recent reports, suggest that is also NAAG an important metabolite which promotes cancer cell survival and tumour aggressiveness. Here, we investigated both the expression and function of *RIMKLA* and *RIMKLB* in hypoxia.

**Material and Methods**

Gene expression analyses in a number of cancer cell lines was determined using RT-qPCR assay. Protein levels of RIMKLA in normoxia and hypoxia, were analysed by western blot and immunofluorescence assays. *RIMKLA* levels were depleted using siRNA and the impact on cell viability was determined, by colony survival assays. Fluorescent molecular probes were used to quantify changes in glutathione (GSH) and reactive oxygen species (ROS), in normoxic and hypoxic cells, with or without *RIMKLA*. Immunofluorescence assays were carried out to assess the status of DNA damage and replication stress in hypoxic cancer cells, plus/minus *RIMKLA*. Finally, publicly available transcriptomics data from patient tumour samples were used for differential survival and gene correlation analysis.

**Results and Discussions**

*RIMKLA*, but not *RIMKLB* was shown to be upregulated in hypoxia, in a HIF-1α-dependent manner. Loss of *RIMKLA* in hypoxia, caused a decrease in GSH levels and consequently, an increase in ROS levels, in the absence of detectable DNA damage. Colony survival

© 2024 The Authors. Molecular Oncology is published by John Wiley & Sons Ltd on behalf of Federation of European Biochemical Societies analyses of hypoxic cells after siRNA-mediated loss of RIMKLA, showed a decreased viability of those cells, compared to their normoxic counterparts. Finally, analysis of publicly available transcriptomics data from patient tumour samples associates *RIMKLA* mRNA levels with worse patient prognosis, in several different types of cancer.

**Conclusion**

*RIMKLA* was upregulated in hypoxic cancer cells and loss of *RIMKLA* caused an increase in ROS levels of hypoxic cells, a decrease of GSH, as well as reduced viability of cancer cells in hypoxia.

### EACR2024-1060
### Decoding the role of BCAS1 in brain tumors

R. Morales Gallel[1], M.J. Ulloa-Navas[2], R. Bueno[3], J.M. García-Verdugo[1], V. Capilla-González[3]
[1]Institute Cavanilles of Biodiversity and Evolutionary Biology- University of Valencia-CIBERNED, Laboratory of Comparative Neurobiology, Paterna, Spain
[2]Mayo Clinic, Laboratory of Neuroscience, Jacksonville-Florida, United States
[3]Andalusian Molecular Biology and Regenerative Medicine Center CABIMER-CSIC-US-UPO, Integrative Pathophysiology and Therapies, Seville, Spain

**Introduction**

Brain Enriched Myelin Associated Protein 1 (BCAS1) is a marker of pre-myelinating oligodendrocytes in the human brain. In addition, the expression of BCAS1 has been shown to be frequently altered in some cancers, including breast, pancreas, colorectal and brain tumors. However, the function of this protein and its involvement in the generation or progression of tumors remain unknown. Recently, our group identified a BCAS1+ cell population in diffuse gliomas with proliferative capacity, suggesting a possible role of BCAS1 in tumor aggressiveness. Here, we investigate how the modification of BCAS1 expression in brain tumor cells influences cell behavior.

**Material and Methods**

To elucidate the role of BCAS1 in brain tumor cells, we have transfected two human glioma cell lines (HOG and U-87) with a plasmid that induces overexpression of BCAS1 (pBCAS1-EGFP). An empty vector was used as control of transfection (pEGFP). Immunofluorescence and western blot techniques were used to study the expression of BCAS1 (anti-BCAS1 marker) and the proliferation (anti-Ki67 marker). Electron microscopy and different cellular assays (scratch, radiation, MTT, colony formation) were carried out to identify how BCAS1 overexpression affects the morphology and behavior of brain tumor cells. Publicly available databases (e.g. TCGA) were used to associate the expression of BCAS1 with brain tumor prognosis.

**Results and Discussions**

The results showed that overexpression of BCAS1 does not modify the proliferation, migration, and survival of brain tumor cells, compared to empty vector transfected cells. However, TCGA database (GBMLGG) evidenced that changes in BCAS1 expression can correlate with patient prognosis, suggesting a possible role of BCAS1 in tumor aggressiveness.

**Conclusion**

After these observations, we conclude that the overexpression of BCAS1 does not significantly affect the behavior of brain tumor cells in vitro. We consider that BCAS1 expression could be saturated in the studied cancer cell lines, thereby unmasking its effects upon overexpression. Further experiments should be performed to elucidate the role of BCAS1 in tumorigenesis.

### EACR2024-1086
### Iron metabolism affects the survival of ovarian cancer cells in anchorage-independent culture conditions

E. Giorgio[1], A.M. Battaglia[1], L. Petriaggi[1], A. Sacco[1], E. Vecchio[1], S. Scicchitano[1], C. Faniello[1], G. Natali[1], C. Galeano[1], F. Biamonte[1]
[1]Magna Graecia University of Catanzaro, Department of Experimental and Clinical Medicine, Catanzaro, Italy

**Introduction**

Successful metastasis requires the ability of cancer cells to detach from the extracellular matrix (ECM). This phenomenon is governed by the crosstalk between cancer cells and their tumor microenvironment (TME). Iron is a key element within the TME, but its role during ECM detachment has not been fully elucidated. Here, we investigate the capacity of iron to influence the survival of ovarian cancer (OVCA) cells in an anchorage-independent culture condition.

**Material and Methods**

3D spheroids derived from HEY and PEO1 OVCA cells were generated using a culture media supplemented or not with an iron compound (Ferlixit®), to mimic an iron rich TME. Cell mortality was measured by propidium iodide (PI) cytofluorimetric assay. Spheroids size and number were obtained by Leica Thunder microscope. 3D invasion capability was assessed with the Matrigel™ assay. Western blot analyses of CD71 and FtH1, and the quantification of labile iron pool (LIP) (Calcein-AM assay), were performed to investigate iron metabolism. Ferroptosis was assessed by quantifying mitochondrial reactive oxygen species (ROS) and lipid peroxides through MitoSOX™ and BODIPY-C11™ assays. Ferroptosis markers GPX4 and VDAC2 were measured by Western blot. The role of iron-related proteins was performed by loss- or gain- of function assays.

**Results and Discussions**

During 2D to 3D transition, HEY and PEO1 cells reduce LIP content; this effect is more evident in PEO1 cells with a reduction of about 9-fold. This is accompanied by the upregulation of FtH1 and the decrease of CD71 in 3D HEY, while 3D PEO1 enhance both, and significantly reduce ROS. This leads to an enhanced mortality only in HEY cells. Ferlixit® administration reduces both number and size of 3D HEY, while PEO1 show the opposite effect. Excess iron further upregulates FtH1 in 3D PEO1, thus preventing LIP accumulation and ROS. HEY, instead, accumulate LIP, lose their invasion ability, and undergo ferroptosis, as shown by the downregulation of GPX4 and VDAC2 and the increase of mitochondrial ROS and lipid peroxides. *FtH1* knockdown impairs the formation of 3D PEO1, without sensitizing them to ferroptosis.

**Conclusion**

© 2024 The Authors. Molecular Oncology is published by John Wiley & Sons Ltd on behalf of Federation of European Biochemical Societies

Iron metabolism reprogramming is essential to allow HEY and PEO1 cells to grow in detached culture conditions. Iron supplementation impairs sphere-forming ability and causes ferroptosis only in HEY sensitive cells. Understanding the iron addiction is mandatory to develop new strategies to kill ECM-detached OVCA cells.

### EACR2024-1091
### SdFFF and UHF-DEP as a cancer stem cell isolation technology for the treatment and personalised medicine of colorectal cancer

*C. Jemfer[1], N. Blasco[2], B. Alies[3], A. Pothier[2], C. Dalmay[2], P. Barthelemy[3], F. Lalloué[1], G. Begaud[1], M. Mathonnet[1], S. Battu[1]*
*[1]Contrôle de l'Activation cellulaire- Progression Tumorale et Résistance thérapeutique CAPTuR UMR1308, University of Limoges, 2 rue du docteur Raymond marcland- LIMOGES, France*
*[2]Xlim institute, umr cnrs 7252, 123 Av. Albert Thomas- 87000 Limoges Cedex, France*
*[3]ARNA, Inserm U1212 UMR CNRS 5320, Université de Bordeaux- 146 rue Léo Saignat- 33076 Bordeaux, France*

**Introduction**
Over the past decade, sedimentation field flow fractionation (SdFFF) has shown potential in cell separation in oncology, specifically in isolating cancer stem cells from colorectal cancer and glioblastoma. These cells hold significant interest due to their role in recurrence of cancers. In this project, we aimed to develop a pairing between SdFFF and Ultra-High-frequency-dieletrophoresis (UHF-DEP) to identify CSCs, to automate their separations and characterisations. Finally, to evaluate the properties of these cells and select the most relevant chemotherapy treatments for patients, we are developing 3D models of spheroids from CSCs isolated by SdFFF in synthetic matrix. The aim is to perform chemotherapy tests and potentially apply this process as a personalized medicine method for future colorectal cancer patients.
**Material and Methods**
For the SdFFF and UHF-DEP pairing needs, the initially used PBS mobile phase was replaced by DEP (dielectrophoresis medium). Consequently, we identified new elution conditions for this mobile phase and conducted a new biological characterization of sorted cells (proliferation, cycle analysis, RTqPCR and Dot blot proteomic). In addition, thanks to RNA sequencing, SdFFF sorting method will enable us to acquire a deeper level of detail on the genomic characteristics of the sorted fractions. This approach will enable us to explore genomic data, which will notably strengthen our understanding between the signatures realized by the UHF-DEP on the fractions and their biological states.
**Results and Discussions**
Biological characterization of CSCs showed enrichment of CSCs in F1 fraction for stem cells RNA expression and in the F3 fraction for the functional properties of these cells. Indeed the replacement of the mobile phases SdFFF can still isolate cells with CSCs properties. We observed that cells recovered in F3 post-SdFFF had a significantly lower signature at UFH-DEP, than in other fractions and were comparable with cells signature cultivated in defined medium, indicating an enrichment in CSCs. This highlights the interest of coupling these technologies to potentially avoid future biological characterization tests.
**Conclusion**
The sensitivity of SdFFF makes it a cell sorting method capable of isolating CSCs. The results obtained demonstrate the potential association of SdFFF with UFH-DEP to automate the identification and isolation of colorectal cancer CSCs. This coupling could facilitate the creation of 3D models from patients for chemotherapy sensitivity testing in the future.

### EACR2024-1104
### Deciphering the upstream molecular mechanisms governing Choline Kinase alpha aberrant transcription in Ovarian Cancer

*A. Rizzo[1], E. Citeroni[1], B. Frigerio[2], E. Luison[2], A. Tomassetti[1], D. Mezzanzanica[1], M. Bagnoli[1]*
*[1]Fondazione IRCCS Istituto Nazionale dei Tumori, Experimental Oncology, Milan, Italy*
*[2]Fondazione IRCCS Istituto Nazionale dei Tumori, Applied Research and Technical Development, Milan, Italy*

**Introduction**
The aberrant overexpression of Choline Kinase alpha (*CHKA*) in Ovarian cancer (OC) drives to a metabolic alteration that plays a critical role for OC aggressiveness. We previously showed that *CHKA* silencing hampers OC behavior by reducing cell proliferation/migration ability and sensitizing cells to chemotherapeutics and to TRAIL-mediated apoptosis. Although *CHKA* impairment and resulting functional effects were extensively investigated, the molecular events contributing to *CHKA* over-expression remain poorly understood. We are now focusing on deciphering the upstream molecular mechanisms sustaining the aberrant *CHKA* expression both at transcriptional and post-transcriptional levels.
**Material and Methods**
For in vitro studies human cell lines representative of different OC subtypes were used. In silico analysis performed from ChIP-seq public databases were employed to predict transcription factors binding sites on *CHKA* promoter. ChIP assay was adopted to check the in vitro HIF1A binding. Bioinformatic algorithms predicted the miRNAs targeting of the 1119bp *CHKA* 3'UTR. Luciferase and GFP-based reporter vector systems were used for cloning and expression strategies.
**Results and Discussions**
The transcriptional activity of the putative full-length *CHKA* promoter region (2500bps upstream transcription start site) cloned in the Luc/GFP reporter systems showed an efficient but variable expression rate among the different OC models. Since several Hypoxia-Response Elements were found to be present in *CHKA* promoter, we evaluated in vitro the direct HIF1A involvement and investigated about miRNAs predicted to target *CHKA* 3'UTR and to be induced by hypoxia. ChIP

© 2024 The Authors. Molecular Oncology is published by John Wiley & Sons Ltd on behalf of Federation of European Biochemical Societies assay showed that modulation of *CHKA* transcript in hypoxia did not depend by the direct binding of HIF1A, suggesting the involvement of other transcriptional regulators. Regarding the post-transcription set we observed a marginal and histotype-related reduction of *CHKA* mRNA induced by the expression of miR-30, miR-200 family and miR-199/miR214 cluster, all involved in the EMT and in hypoxia response.

**Conclusion**

*CHKA* transcription in OC appears to be regulated at multiple levels and in a histotype-dependent manner. In order to better understand the network governing *CHKA* regulation, our current focus is to identify the CHKA Minimal Core Promoter. This will define the specific DNA sequences crucial for the transcription machinery recruiting and will help to identify the specific Transcription Factors engaged.

*Supported by AIRC and Min. Salute Ricerca Corrente L.2.2.1/45*

### EACR2024-1110
### Alterations of the TGFb-sequestration complex member ADAMTSL1 levels are associated with muscle defects and fusion-positive rhabdomyosarcoma aggressiveness

*C. Berthelot[1], A. Bertrand[1], P. Castets[2], R. Mounier[3], M. Castets[1]*
*[1]Cancer Research Center of Lyon, Citi, Lyon, France*
*[2]UNIGE, Cell Physiology and Metabolism, Lyon, France*
*[3]PGNM, Metabolic regulation of adult muscle stem cell fate, Lyon, France*

**Introduction**

Rhabdomyosarcoma (RMS) is the most frequent form of pediatric soft-tissue sarcoma and remains a medical challenge. RMS shares histological features with cells of the muscle lineage and this cancer is thought to arise from malignant transformation of myogenic precursors. It has been proposed that some early steps of myogenic differentiation are blocked in RMS, and that understanding how the normal process has gone awry could help to decipher the biological underpinnings of tumorigenesis and tumor escape.

**Material and Methods**

Here, we combine the use of murine transgenic and xenograft models, in vitro tools including organoids, and integrative multi-omic bulk and single-cell data analyses, to define the function of ADAMTSL1 in muscle pathophysiology.

**Results and Discussions**

Our results show that a matrix protein with hitherto unknown function, ADAMTSL1, is involved in skeletal muscle regeneration, via modulation of TGF-β-pathway activity. We demonstrate that ADAMTSL1 is a good prognosis factor in Fusion Positive-RMS (FP-RMS). In FP-RMS, ADAMTLS1 drives a phenotypic switch that modifies invasion potential of tumor cells, suggesting that it may constitute a new therapeutic target.

**Conclusion**

Our results identify ADAMTSL1 as a new prognostic marker in FP-RMS and underlies the need to characterize in depth the crosstalk between matrix and tumor cells, as a new putative lever to impact tumor aggressive cell state.

### EACR2024-1119
### Use of organoid models to study invasive cell states in Diffuse Intrinsic Pontine Glioma

*C. Berthelot[1], P. Huchedé[1], M. Hamadou[1], E. Cosset[1], S. Meyer[1], M. Castets[1]*
*[1]Cancer Research Center of Lyon, Citi, Lyon, France*

**Introduction**

DIPG (Diffuse Intrinsic Pontine Glioma) represents one of the biggest challenges in pediatric oncology. Despite half a century of clinical trials, median overall survival remains stagnant at ~12 months post-diagnosis. One common hallmark of DIPG is the global alteration of epigenetic marks, notably resulting from H3.1 and H3.3K27M mutations, which reshape chromatin organization and lead to the establishment of an oncogenic transcription program. Crosstalk between epigenomic rewiring and activation of oncogenic transcriptional signalings allows to establish functional intra-tumoral hierarchy, ranging from oligodendrocytic precursors-like proliferative cells to more differentiated astrocytic- and oligodendrocytic-like cells. Understanding this tumor hierarchy is crucial to optimize therapeutic intervention and target the aggressive quiescent/invasive cell states.

**Material and Methods**

We combined an integrative multi-omics approach based on bulk, single-cell and spatial transcriptomic analyses, with functional characterization on cutting-edge DIPG and mini-brain organoid models to study the mechanisms involved in the acquisition of invasive properties.

**Results and Discussions**

We identify the existence of an oncogenic synergy between BMP and H3.3K27M mutations, which is sufficient to induce the transition to a quiescent but invasive cell state, which could likely be involved in resistance to treatments

**Conclusion**

Our data suggest that BMPs produced by the micro-environment may install an autocrine regulatory loop in H3.3-mutated DIPGs, leading to the onset of an aggressive cell state. Targeting this oncogenic node could provide a new therapeutic lever to improve DIPG care management.

### EACR2024-1122
### Potentiating ERK hyperactivation by targeting the proteostasis network

*D. Farnsworth[1], L.I.J. Ma[2], E. Macdougall[2], D. Lu[2], F. Naznin[2], R. Shi[2], R. Somwar[3], A.M. Unni[4], W.W. Lockwood[2]*
*[1]British Columbia Cancer Research Center, Integrative Oncology, Vancouver, Canada*
*[2]BC Cancer Research Center, Integrative Oncology, Vancouver, Canada*
*[3]Memorial Sloan Kettering Cancer Center, Department of Pathology, New York, United States*
*[4]Weill Cornell Medicine, Meyer Cancer Center, New*

© 2024 The Authors. Molecular Oncology is published by John Wiley & Sons Ltd on behalf of Federation of European Biochemical Societies

York, United States

**Introduction**

Recent findings have highlighted that cancer cells retain sensitivity to excessive signaling driven through the extracellular regulated kinase (ERK) pathway, referred to as ERK hyperactivation. In normal cells, excessive ERK signaling drives proliferative arrest through activation RB and p53, and induces senescence. The mechanism of proliferative arrest driven by ERK signalling in cancer cells, as well as the effect of different genetic backgrounds on this response, remain unclear. We aimed to characterize both in the context of targeted therapy resistant lung adenocarcinoma (LUAD).

**Material and Methods**

We performed a genome wide CRISPR-Cas9 knockout screen paired with transcriptome profiling in models of ERK hyperactivation to identify pathways that could potentiate cell death.

**Results and Discussions**

We uncovered components of the unfolded protein response and proteostasis network as key dependencies during states of ERK hyperactivation. We found that inhibiting these nodes further sensitized cells to ERK hyperactivation. We next sought to characterize potential for ERK hyperactivation across different genetic alterations detected in the context of targeted therapy resistance. Using dox inducible promoters, we overexpressed genes associated with epidermal growth factor receptor (EGFR) inhibitor resistance in EGFR mutant LUAD cells and found several that induced proliferative arrest when co-expressed with mutant EGFR. Finally, we developed models of resistance to EGFR, Kirsten rat sarcoma virus (KRAS) and hepatocyte growth factor (MET) inhibitors, respectively. In these models, we found that disrupting proteostasis sensitized cells to a drug holiday, suggesting clinical translation for this novel dependency.

**Conclusion**

Together, this work highlights the various positive mediators of ERK hyperactivation in LUAD as well as the mechanisms by which cancer cells tolerate it.

### EACR2024-1130
### Iron overload restores sensitivity of resistant ovarian cancer cells to erastin-mediated ferroptosis

*L. Petriaggi[1], A.M. Battaglia[1], E. Giorgio[1], A. Sacco[1], M.C. Faniello[1], G. Natali[1], C. Galeano[1], F. Biamonte[1]*
*[1]Magna Graecia University, Department of Experimental and Clinical Medicine- "Magna Graecia" University of Catanzaro- Catanzaro- Italy, Catanzaro, Italy*

**Introduction**

Targeting ferroptosis, a new form of regulated cell death driven by iron-dependent lipid peroxidation and/or mitochondrial dysfunction, is emerging as a new therapeutic approach to treat cancer cells, especially those showing resistance to chemotherapy. However, the effectiveness of ferroptosis inducers (FINs) can be limited by still poorly defined genetic or metabolic determinants of cancer cells. Here, we investigate the molecular mechanisms underlying sensitivity or resistance of ovarian cancer (OVCA) cells to the ferroptosis inducer erastin.

**Material and Methods**

A panel of OVCA cell lines (HEY, COV318, PEO4, A2780CP) were treated with 8 and 25µM of erastin until 24h. To cause intracellular iron overload, 100 and 250µM of $Fe^{3+}$ compound (Ferlixit®) has been administrated to all cell lines. Flow cytometry was used to assess ferroptotic cell death (PI assay), mitochondrial ROS (MitoSOX™ assay) and membrane potential (TMRM™ assay), lipid peroxidation (BODIPY-C11™ assay), and intracellular labile iron pool (LIP) (CA-AM assay). Western Blot was used to quantify biomarkers of mitochondrial dysfunction (VDAC2) and ferritinophagy (FtH1, NCOA4). The ferroptosis-specific "ballooning" phenotype and mitochondrial ultrastructural changes were detected by using optical microscopy and Transmission Electron Microscopy (TEM), respectively.

**Results and Discussions**

We found that erastin triggers ferroptosis only in HEY cells, while COV318 appear resistant. HEY sensitivity depends on their higher baseline LIP, which results further increased upon erastin treatment. This phenomenon is caused by NCOA4-mediated ferritinophagy, that leads to the iron-mediated accumulation of mitochondrial ROS, reduction of VDAC2, mitochondrial membrane depolarization and their ultrastructural alteration. When erastin administration was preceded by iron chelation (deferoxamine, 200µM for 24h) ferroptosis of HEY was totally prevented. The increase of intracellular iron by the use of Ferlixit® fully sensitizes COV318 to erastin (97% of mortality). Notably, the sole administration of Ferlixit® killed over 40% of COV318 and 95% of HEY cells.

**Conclusion**

Together, our data suggest that enough baseline or treatment-induced LIP is a strong determinant for OVCA cell sensitivity to erastin. Besides, we propose Ferlixit® as a new FIN in OVCA cells intrinsically characterized by a higher iron addiction; alternatively, we suggest using Ferlixit® to sensitize resistant OVCA cells to erastin.

### EACR2024-1131
### Exploring SNF2 Histone Linker PHD Ring Helicase (SHPRH)'s Role in Initiation and Development of Lung Adenocarcinoma

*Y.C.. Chuang[1], T. Sihota[2], A. Nagelberg[2], J. Chow[1], R. Shi[1], W. Lockwood[3]*
*[1]BC Cancer Research Institute, Integrative Oncology, Vancouver, Canada*
*[2]BC Cancer Research Institute, Pathology and Lab Medicine, Vancouver, Canada*
*[3]BC Cancer Research Institute, Pathology and Lab Medicine & Integrative Oncology, Vancouver, Canada*

**Introduction**

Late-stage diagnosis of lung cancer (LC) is associated with poor prognosis and survival, highlighting a need for increased understanding of risk factors to support early screening and treatment strategies. While environmental factors play a significant role, genetic factors can greatly enhance LC risk. Using whole exome sequencing of

© 2024 The Authors. Molecular Oncology is published by John Wiley & Sons Ltd on behalf of Federation of European Biochemical Societies never-smokers with lung adenocarcinoma (LUAD), our lab identified a candidate gene linked to LC susceptibility: SNF2 Histone Linker PHD Ring Helicase (SHPRH). Our lab has demonstrated that *SHPRH* acts as a tumour suppressor gene in the context of LUAD, but its mechanism has yet to be elucidated. This project aims to investigate the mechanism of SHPRH-mediated tumour suppression in LUAD cells and identify the key pathways involved with its phenotype.

**Material and Methods**

LUAD datasets were used to establish clinical relevance by evaluating the association between SHPRH expression levels and survival outcomes. The role of SHPRH in LUAD tumourgenesis was functionally characterized in vitro and in vivo. A doxycycline-inducible system was used to conditionally express SHPRH in LUAD cell lines, and in vitro colony formation assays and in vivo xenograft models were used to assess alterations in tumorigenic potential. RNA-sequencing (RNA-seq) and immunoprecipitation-mass spectrometry (IP-MS) were performed to identify transcriptomic and proteomic changes with SHPRH expression.

**Results and Discussions**

SHPRH was mutated or deleted in 7% of LUADs, and patients with reduced SHPRH expression have significantly worse survival outcomes. SHRPH re-expression in cell lines with existing inactivating alterations reduced colony growth in vitro. Implantation of these same cell lines into NRG mice show that SHPRH re-expression significantly reduces tumour burden in vivo. RNA-seq and IP-MS performed in SHPRH re-expressing cells were able to identify major biological processes and interactors associated with SHPRH, respectively. The pathways and interactors are under active investigation to determine their necessity for SHPRH's tumour suppressive function in vitro.

**Conclusion**

Our results show that SHPRH plays a tumour suppressive role in LUAD. Improving our understanding of SHPRH's tumour suppressive function and its contribution to LC initiation may help identify at-risk patients and increase opportunities for early screening and interventions.

### EACR2024-1151
### Mitochondrial metabolic plasticity is required for proliferation of glioblastoma multiforme cells

M. Tsujishita[1], H. Bae[1], S. Skobelkina[1], E. Lacasse[1], J.E. Kim[1], S. Zhang[1], P. Amin[1], P.J. Smith[2], K.N. Alavian[1]
[1]Imperial College London, Brain Sciences, London, UK
[2]University of Southampton, Institute for Life Sciences, Southampton, UK

**Introduction**

Gliomas comprise over 75% of all malignant brain tumours. Current therapeutic methods including surgery, radiation and chemotherapy fail to yield significant clinical outcomes in the malignant types, including glioblastoma multiforme (GBM). GBM has one of the worst 5-year survival rates among all cancers with a median survival time of less than 14 months. A characteristic feature of cancer cells is their reprogramming of energy metabolism. De novo ATP production through mitochondrial oxidative phosphorylation and production of the intermediates of the tricarboxylic acid (TCA) cycle, required for anabolic growth, are both dependent on generation of an electrochemical gradient across the mitochondrial inner membrane, which is dependent on ion leak currents. We have demonstrated that a proton leak channel, within the F1Fo ATP synthase, is modulated by Bcl-xL, an anti-apoptotic member of Bcl-2 family. In this work, we examined the role of Bcl-xl-mediated metabolic efficiency in proliferation of GBM cells.

**Material and Methods**

Using proximity ligation assay, we established a grade-dependent correlation between the mitochondrial inner membrane interaction of Bcl-xL and the rate of proliferation. We then used mitochondrial patch clamp electrophysiology and single cell oxygen flux measurements to characterise the biophysical properties of inner membrane ion channels in mitochondria isolated from cancer tissue and examine metabolic efficiency of the mitochondria. We have also performed live cell imaging of the mitochondrial membrane potential in primary glioma cells.

**Results and Discussions**

We observed a grade-dependent increase in interaction of Bcl-xL with the F1Fo ATP synthase within glioma cells. Our results demonstrate a correlation between mitochondrial metabolic efficiency and proliferation of glioma cells. They suggest that an enhanced activity and coupling of oxidative phosphorylation and anaplerotic flux, modulated by ion leak channels, are required for proliferation of GBM cells. The coupling of mitochondria in GBM cells is modulated by Bcl-xL-dependent differences in ion leak currents across the inner membrane. The less proliferative anaplastic astrocytoma cells demonstrated a different metabolic phenotype, i.e. less dependency on oxidative metabolism, mitochondria and this specific metabolic efficiency mechanism for proliferation.

**Conclusion**

Our results suggest that a Bcl-xL-dependent increase in mitochondrial metabolic efficiency is required for rapid proliferation of GBM cells.

### EACR2024-1159
### CD52-expressing immunosuppressive myeloid cells induce breast mesenchymal cancer stem cells by membrane-bound TGF-β1

T. Boyer[1], C. Blaye[2], A. Moisand[3], D. Chalopin[4], J. Boucher[5], M. Madéry[3], P. Roux[6], C. Varon[7], C. Domblides[8], N. Larmonier[3]
[1]ImmunoConcEpT UMR CNRS 5164, Université de Bordeaux, Bordeaux, France
[2]Bergonié Institute, Bergonié Institute, Bordeaux, France
[3]ImmunoConcEpT UMR CNRS 5164, University of Bordeaux, Bordeaux, France
[4]Centre de Bioinformatique de Bordeaux CBiB, University of Bordeaux, Bordeaux, France
[5]Institut de Recherche en Immunologie et en Cancérologie IRIC, Montréal- QC, Montréal, Canada
[6]Institut de Recherche en Immunologie et en

© 2024 The Authors. Molecular Oncology is published by John Wiley & Sons Ltd on behalf of Federation of European Biochemical Societies

*Cancérologie IRIC, University of Montréal- QC, Montréal, Canada*
*[7]Bordeaux Institute of Oncology BRIC INSERM UMR 1312, University of Bordeaux, Bordeaux, France*
*[8]University Hospital of Bordeaux, Department of Medical Oncology, Bordeaux, France*

**Introduction**

Suppressive myeloid cells play a central role in cancer escape from anti-tumor immunity. Beyond their immuno-suppressive function, these cells are capable of exerting a large array of pro-tumoral activities, including the promotion of cancer cell survival, invasion and metastasis. Some reports have suggested that suppressive myeloid cells may also be equipped with the capability to promote Epithelial-to-Mesenchymal Transition, which might convey stem-like properties to cancer cells. However, the impact of suppressive myeloid cells in the acquisition and maintenance of cancer stemness and the underlying mechanisms remain to be fully comprehended.

**Material and Methods**

To investigate suppressive myeloid cells induction of cancer stemness, we co-cultured MCF7 breast cancer cell line with HuMoSC (Human Monocyte-Derived Suppressive Cells), a strongly immunosuppressive in vitro generated model. We next performed transcriptomic (single cell RNA sequencing) and surface proteomic analysis to puzzle out interaction networks between HuMoSC and MCF7 and investigate interactions of interest involved in the stemness plasticity mediated by HuMoSC. Finally, we performed single cell RNA sequencing on myeloid cells of 10 breast cancer patients followed by functional experiments to confirm our hypothesis and findings previously made with the HuMoSC model.

**Results and Discussions**

We demonstrated that human suppressive myeloid cells from cancer patients or generated in vitro promoted cancer stemness and specifically the plasticity towards a mesenchymal, but not epithelial phenotype. This cancer-stemness-inducing function was restricted to only myeloid cells exerting immunosuppressive activities, and more specifically to subsets expressing the glycoprotein CD52. Transcriptomic and surface proteome-based interactome analysis led to the identification of membrane-bound TGF-β1 as the main mechanism of cancer stemness induction, while no secretion of TGF-β1 was observed. Functional inhibition of membrane-bound TGF-β1 and related pathway blocked the emergence of cancer stem cells induced by suppressive myeloid cells.

**Conclusion**

Altogether, our results identified a new, pro-tumoral function of CD52 expressing suppressive myeloid cells. These cells foster breast cancer cell stemness and favor their plasticity towards a mesenchymal, migratory phenotype. Interestingly, we uncovered a new mechanism of the poorly studied membrane-bound TGF-β1 isoform in fostering cancer stemness.

### EACR2024-1160
### Effect of BET inhibitors on oral squamous cell carcinoma cell line and its' CD44 positive subpopulation

*M. Lazarevic[1], M. Karisik Jaksic[2], D. Mitic[2], J. Milasin[2]*
*[1]School of Dental Medicine- University of Belgrade, Implant-research Center, Belgrade, Serbia*
*[2]School of Dental Medicine- University of Belgrade, Implant-research Center, Belgrade, Serbia*

**Introduction**

Oral squamous cell carcinoma (OSCC) is the predominant malignant tumor in the oral and maxillofacial region, comprising 80% of oral cavity tumors. Despite advancements in OSCC management, survival rates remain suboptimal, and the development of novel anti-neoplastic agents is needed.

**Material and Methods**

The study investigated the effects of three BET inhibitors (JQ1, iBET-151, iBET-762) on oral squamous cell carcinoma cell line (SCC-25) and its' CD44 positive subpopulation. Magnetic sorting was used to gain CD44 positive cells. Cells were seeded in 96 well plates, and 10 μM dose of drugs were added to the wells. After 24, 72 hours, and 7 days MTT was performed. As control served cells cultured in medium with DMSO in concentration needed for drugs dissolution. All experiments were performed in triplicate, in two separated experiments.

**Results and Discussions**

Real-time PCR analysis and flow cytometry confirmed adequate sorting of CD44+ positive cells. After 24h of treatment significant cytotoxicity of the drug was observed only for JQ1 on SCC-25 cell line ($p<0.05$), in comparison to untreated cells. On 72 h of treatment there was significant reduction of the cells in the presence of the drugs, but no difference was observed between reduction of SCC-25 cell line and CD44+ positive cells. In longer treatment period of 7 days, there were significant difference in cell survival between SCC-25 and its CD44+ cells, in presence of the drugs, for JQ1 ($p<0.0001$) and iBET-151 ($p<0.001$). Mentioned BET inhibitors had significantly higher cytotoxic effect on CD44+ cells.

**Conclusion**

Results of the study indicate better antitumor effect of BET inhibitors on CD44+ population of tumor cells during prolonged treatment of tumor cells. New investigations should provide more insight into mechanism of action of BET inhibitors on specific oral cancer cells subpopulations.

### EACR2024-1161
### Dietary sulfur restriction promotes antitumor immunity via T cell activation

*X. Gao[1], B. Chakraborty[2], H. Quang[1], B. Vilaysane[1], M. Aguilar[1], S. Artham[2], C.Y. Chang[2], P.K. Juras[2], D.P. McDonnell[2]*
*[1]Baylor College of Medicine, Pediatrics, Houston, USA*
*[2]Duke University, Pharmacology & Cancer Biology, Durham, USA*

**Introduction**

Methionine, an essential amino acid, is involved in a myriad of cellular functions including epigenetic stability, redox balance, biosynthesis, and many other metabolic processes. Dietary methionine restriction (MR) has shown antitumor effect in a wide variety of preclinical models, alone or as an adjuvant therapy with frontline chemotherapy and radiotherapy. Yet, whether

Molecular Oncology 18 (Suppl. 1) (2024) 1–495
© 2024 The Authors. Molecular Oncology is published by John Wiley & Sons Ltd on behalf of Federation of European Biochemical Societies

MR exerts antitumor effect in the presence of an intact immune system remains controversial. Here, we are to untangle the anti-tumor effect and immunity of MR.

**Material and Methods**

To evaluate the antitumor effect, we first conducted dietary intervention with MR in a series of syngeneic mouse models for breast cancer (A7C11 and MET1), melanoma (GEMM6 and Yumm5.2), colorectal cancer (MC38 and CT26), and lung cancer (LLC),, where the immune system remains intact, and in xenograft models established in immune deficient background. We then carried out flow cytometry analysis of intratumor immune cells. Furthermore, we evaluated the combinatory effect of MR with immune checkpoint blockade. Finally, to understand the mechanisms, we performed metabolomics and RNAseq analysis on T cells derived from non-tumor bearing mice fed a control or MR diet. Intratumor nutritional status was also evaluated by metabolomics.

**Results and Discussions**

In all examined mouse models , MR led to tumor inhibition to different extents depending on the model. Such tumor inhibition effect was replicated or completely reversed in tumor models established with the same cancer cells in immune deficient NSG background, suggesting the relevance of immune system is tumor type dependent. Flow cytometry analysis of intratumor immune cells revealed a consistent activation of CD8+T cell phenotype across different models. Anti-CD8 antibody treatment reversed, at least partially, the MR-mediated tumor inhibition. Furthermore, MR synergized with anti-PD1 immune checkpoint blockade. In healthy mice, MR significantly increased the splenic effector memory CD8+T cell population. Metabolomics and RNAseq analysis revealed an active metabolic state towards T cell activation. Lastly, intratumor methionine level remained higher than that in the circulation, arguing against the limitation of methionine for T cell function in tumor microenvironment.

**Conclusion**

Together, dietary MR promotes antitumor immunity via activation of CD8+T cells.

### EACR2024-1170
### Addressing the roles of KDM6A and KDM6B histone demethylases in isocitrate dehydrogenase (IDH) mutant glioma

*S. Sarac Yalcinkaya[1,2], E. Yilmaz[1,2], A. Kayabolen[2], T. Bagci-Onder[1,2]*

*[1]Koç University School of Medicine, Brain Cancer Research and Therapy Lab, Istanbul, Turkiye*

*[2]Koç University, Research Center for Translational Medicine, Istanbul, Turkiye*

**Introduction**

Glioma, representing 80% of malignant brain tumors, is highly aggressive. Most IDH-mutant gliomas harbor a heterozygous point mutation in IDH1, triggering the accumulation of 2-hydroxyglutarate, inhibiting DNA and histone demethylases, and causing a hypermethylated phenotype. Given their distinct epigenetic profile, we aimed to examine the epigenetic vulnerabilities of IDH-mutant gliomas. From a chemical screen targeting chromatin modifiers, we previously identified GSK-J4, an inhibitor of histone demethylases KDM6A and KDM6B, as an IDH1-mutant selective compound. In this study, we aimed to establish a genetic approach to phenocopy the effects of chemical inhibition of KDM6A/6B.

**Material and Methods**

We employed CRISPR/Cas9-based gene ablation in primary IDH-mutant glioma cells, MGG152 and TS603. Using sgRNA sequences cloned into backbones with different selection markers, we established double KDM6A/6B-depleted cells. Standard molecular cloning, qRT-PCR, western blot, Annexin V staining, oxidative stress assay, and cell titer glo assay were used for cell-based assays, and RNA sequencing was employed to interrogate transcriptomic changes upon KDM6A/6B loss.

**Results and Discussions**

We established IDH-mutant primary lines with reduced KDM6A/6B expression, confirmed via qRT-PCR and western blot. Double knockout displayed reduced growth rates, morphological differences, and increased H3K27me3 levels. KDM6A/6B-deficient cells were sensitized to irradiation but not to temozolomide. Apoptosis assays detected no significant increase in apoptosis-related phenotypes. RNA sequencing analysis revealed upregulation of transforming growth factor beta (TGF-β), its targets, and senescence-related genes. To test the link between KDM6A/6B regulated TGFß and reactive oxygen species (ROS), we compared ROS levels in KDM6A/6B-depleted cells and controls. To assess the relation between ROS and senescence, we performed ß-galactosidase staining. As a more in vivo compatible cell line, TS603 cells resulted in tumor formation with histological features of gliomas. We are currently testing the tumor formation ability of KDM6A/KDM6B-depleted TS603 cells in mice.

**Conclusion**

Despite KDM6A and KDM6B playing significant, sometimes conflicting roles in various tumor types, their functions in IDH-mutant glioma remain unexplored. These findings contribute to the ongoing exploration of the roles of KDM6A and KDM6B in IDH-mutant gliomas, offering insights into potential novel therapeutic strategies with epigenetic intervention points of the tumors.

### EACR2024-1182
### Effective elimination of glioblastoma cancer stem cells through the combination of temozolomide and disulfiram

*P. Melón Bajo[1], P. Alfonso Triguero[2], T. Fernández Cabada[1], A.P. Candiota[3], S. Villegas[3], J. Lorenzo Rivera[1]*

*[1]Instituto de Biotecnología y Biomedicina IBB, Bioquímica y Biología Molecular, Barcelona, Spain*

*[2]Catalan Institute of Nanoscience and Nanotechnology ICN2 - CSIC and BIST, Nanostructured functional Materials group, Barcelona, Spain*

*[3]Facultad de Biociencias UAB, Bioquímica y Biología*

© 2024 The Authors. Molecular Oncology is published by John Wiley & Sons Ltd on behalf of Federation of European Biochemical Societies

*Molecular, Barcelona, Spain*
**Introduction**
Glioblastoma multiforme (GBM) is the most common and aggressive type of brain tumor, known for its limited treatment success due to its high infiltrative potential and tumor heterogeneity. Standard therapy involves surgery, radiation, and chemotherapy with temozolomide (TMZ). However, tumor recurrence remains common. Cancer Stem Cells (CSCs), characterized by high ALDH expression, are crucial for tumor growth and recurrence. Our study investigates disulfiram (DSF), an ALDH inhibitor, combined with copper (Cu), which has shown promise in eliminating CSCs in GBM models when used alongside low-dose TMZ. We aim to understand the effectiveness of this combined therapy in targeting CSCs in GBM using in vitro models.
**Material and Methods**
2D and 3D models of immortalized human and murine GBM cell lines (U87-MG and GL261) were used in our studies. Cytotoxicity assays were performed to evaluate the effect of the drugs and minimum effective dose required. We assessed the efficacy of TMZ versus DSF in the presence of $CuSO_4$ and the combination of TMZ + DSF/ $CuSO_4$. Cell viability was measured using Presto Blue and results expressed as IC50 (half maximal inhibitory concentration) values. Cell death assays in order to establish if cells died by apoptosis or necrosis, were conducted using annexin and propidium iodide by flow cytometry, analysing on this way the potential synergistic effect of the combination of both drugs.
**Results and Discussions**
Cytotoxicity assays reveal a time-dependent decrease in the minimum effective dose across all cell lines treated with both drugs, suggesting significant synergistic effects between the compounds. Flow cytometry demonstrate these synergistic effects of the combined drug treatment across all cell lines. In GL261, lower doses of DSF/ $CuSO_4$ are required, particularly when combined with TMZ, indicating heightened sensitivity. This may be explained to the higher expression of ALDHs in GL261 compared to U87-MG, the latter characterized by its resistance and heterogeneity due to elevated CSCs presence. Cells cultured in 3D exhibit enhanced susceptibility to drug effects, as evidenced by lower IC50 values and the reduced effective dose requirement of both drugs.
**Conclusion**
A significative synergistic effect between TMZ and DSF/$CuSO_4$ is observed in GBM CSCs, decreasing notably the effective doses needed to eliminate GBM CSCs. Low doses of chemotherapeutic drugs would involve fewer side effects for GBM patients, therefore being a very promising therapeutic option for future clinical trials.

### EACR2024-1183
### The role of mtDNA in metabolism and tumour development in melanoma models

*C. Mendes Correia[1], M. Mahmood[1], L. Mitchell[1], E. Shokry[1], A. Mackintosh[1], G. Malviya[1], D. Sumpton[1], D. Lewis[1], K. Blyth[1], P. Gammage[1]*
*[1]CRUK Scotland Institute, University of Glasgow, Glasgow, United Kingdom*
**Introduction**
Mitochondrial DNA (mtDNA) encodes 13 proteins that are part of the respiratory complexes. Therefore, mtDNA is essential for the maintenance of normal mitochondrial metabolism, the impairment of which is a known hallmark of cancer. Cells depleted of mtDNA ($\rho^0$ cells) have been used in vitro as negative controls for mitochondrial processes dependent on a functional respiratory chain, but characterisation of their metabolism or the process by which they develop into tumours in vivo has been limited. Transfer of mtDNA from immune cells to cancer cells has recently been described as necessary for tumour establishment; however, the mechanism underlying mtDNA transfer has not yet been elucidated.
**Material and Methods**
We characterised the phenotype of $\rho^0$ cells in vitro and in vivo to assess how mtDNA affects tumour metabolism and development. We studied $\rho^0$ cells derived from melanoma lines B78 and Hcmel12 using high-resolution microscopy, metabolomics, lipidomics and proteomics. We subcutaneously injected these cells in immuno-competent C57BL/6J or immunodeficient NSG mice and sampled the tumours at endpoint. Tumours were analysed by Sanger-seq, metabolomics, histology and live PET imaging, and their mtDNA copy number status was characterised by ddPCR. Animal experiments were carried out according to the UK Animals Act 1986 and the ARRIVE guidelines.
**Results and Discussions**
We extensively characterised the metabolism of B78-derived $\rho^0$ cells and observed truncated TCA cycle and reductive carboxylation. The lipidome of $\rho^0$ cells was markedly altered, with lipid storage in lipid droplets confirmed by high-resolution microscopy, and alterations to membrane composition associated with abnormal mitochondrial and cellular morphology. Tumours derived from $\rho^0$ cells showed delayed tumour growth. We confirmed the metabolic phenotype observed in vitro, with higher reliance on glucose uptake and altered lipid metabolism. These tumours partially reacquired mtDNA from the host and exhibited distinct regions with contrasting mtDNA content, proliferation and expression of key metabolic genes. Subcut tumours derived from $\rho^0$ cells in NSG mice exhibited similar patterns of mtDNA reacquisition, suggesting an mtDNA origin other than immune cells.
**Conclusion**
Our results suggest that mtDNA is not essential for tumour development and is readily acquired from the tumour microenvironment when mtDNA status is compromised, implying that mitochondrial mutation burden in tumours might be altered by the surrounding stroma.

### EACR2024-1191
### Cellular and mitochondrial biology of colorectal cancer cells overexpressing gene for Mitofusin 2

*S. Tyciakova[1], V. Lišková[2], B. Svitková[3], K. Frimmel[4], J. Janko[5], M. Pastorek[5], O. Križanová[2], M. Matúšková[1]*

Molecular Oncology 18 (Suppl. 1) (2024) 1–495
© 2024 The Authors. Molecular Oncology is published by John Wiley & Sons Ltd on behalf of Federation of European Biochemical Societies

[1]Cancer Research Institute- Biomedical Research Center of Slovak Academy of Sciences, Department of Molecular Oncology, Bratislava, Slovakia
[2]Institute of Clinical and Translational Research, Biomedical Research Center- Slovak Academy of Sciences, Department of Molecular Physiology, Bratislava, Slovakia
[3]Cancer Research Institute- Biomedical Research Center of Slovak Academy of Sciences, Department of Nanobiology, Bratislava, Slovakia
[4]Institute for Heart Research- Centre of Experimental Medicine- Slovak Academy of Sciences, Department of Histochemistry and Electron microscopy, Bratislava, Slovakia
[5]Institute of Molecular Biomedicine- Faculty of Medicine - Comenius University, Institute of Molecular Biomedicine, Bratislava, Slovakia

**Introduction**

Colorectal cancer is the third most common type of cancer, representing 10% of all cancers diagnosed annually. One of the most important hallmarks of cancer cells is reprogrammed mitochondrial bioenergetics towards aerobic glycolysis (Warburg effect). Tumor cells usually display different mitochondrial morphology and shortened mitochondria. Cells maintain their energetics by adapting mitochondrial function, which is closely linked to continuously changing mitochondrial morphology as a result of fission and fusion. Fission is executed by the cytosolic protein Drp1, often activated in cancer cells. Mitofusins 1 and 2 (Mfn1, Mfn2) are responsible for mitochondrial outer membrane fusion and usually are downregulated in cancer cells. The Opa1 protein is responsible for inner mitochondrial membrane fusion.

**Material and Methods**

Colorectal cancer cell lines HCT116 and HT-29 were engineered to stable overexpress the human *MFN2* gene. Cell biology and mitochondrial functions of transduced cells were monitored using assays detecting ATP and pyruvate production, oxidative stress, glucose uptake, the onset of apoptosis, autophagy, and mitophagy, the mitochondrial status, and mitochondrial DNA content. The mitochondrial network was visualised by fluorescent microscopy.

**Results and Discussions**

We achieved *MFN2* gene overexpression up to 15- and 65 times for HCT116 and HT-29 cells respectively, but this was not followed by changes in the expression of genes *MFN1*, *OPA1,* and *DRP1*. Cell proliferation was not impaired, but the proportion of apoptotic HCT116 cells increased. Viability was slightly decreased in low-glucose media and in the presence of metformin. The mitochondrial mass and the amount of mitochondrial DNA were unchanged, but the percentage of cells with decreased mitochondrial membrane was decreased. These findings follow the increased production of ATP, pyruvate, and ROS. We did not detect a significant induction of autophagy and mitophagy in engineered cells, and similarly, any significant changes in sensitivity to 5-FU, oxaliplatin, and irinotecan/SN-38.

**Conclusion**

Our study demonstrated complex characteristics of colorectal cancer cells stable overexpressing Mitofusin 2 gene. We noticed differences among the two cell lines and found limitations in trying to promote significant fusion of mitochondria by *MFN2* gene overexpression, to promote the shift of tumor glycolytic metabolism to more oxidative, towards the status of the mitochondria of a healthy cell.

### EACR2024-1202
### Elucidating a novel role for the Bcl-2 family member BAD in breast cancer metastasis

L. Pirayeshfard[1], J. Maringa Githaka[2], S. Luo[2], A. Saini[2], N. Touret[2], O. Julien[2], I.S. Goping[2]
[1]University of Alberta, Biochemsitry, Edmonton, Canada
[2]University of Alberta, Biochemistry, Edmonton, Canada

**Introduction**

Breast cancer is the most diagnosed cancer type in women and metastasis is the major cause of death. Understanding cancer cell metastasis at a molecular level will undoubtedly provide tools for improved clinical outcome. Our lab identified the Bcl2 family protein BAD as a novel regulator of metastasis as well as a prognostic marker for breast cancer patient survival. BAD is regulated by coordinated phosphorylation of three key serine residues, and using a genetic mouse model wherein the three serines are mutated to alanine (BAD3SA), we demonstrated that BAD3SA inhibits mammary epithelial cell migration in both mammary gland development and breast cancer metastasis. BAD3SA represses localized mRNA translation specifically in subcellular protrusions. This blocks focal adhesion maturation, which drives cancer cell motility and invasion. Notably, anti-metastatic BAD3SA localizes to the mitochondria, whereas wild-type BAD is cytosolic. Thus, this study investigates (i) whether BAD subcellular localization regulates cell motility and, (ii) the molecular mechanisms by which BAD modulates cell motility.

**Material and Methods**

To investigate whether BAD localization to mitochondria inhibits cell motility, we generated BAD mutant constructs that either disrupt or enhance mitochondrial targeting. We examined the subcellar localization and its effect on cell motility in normal mammary epithelial cells, MCF10A. To gain molecular insight, we used a proximity-labeling system and identified BAD3SA-binding proteins in 2D and 3D culture by mass spectrometry.

**Results and Discussions**

Our results revealed targeting wild-type BAD to the mitochondria inhibits cell motility, indicating a significant role for mitochondria in this process. Furthermore, BAD3SA only inhibits migration of cells cultured in 3D but not in 2D. This suggests that BAD3SA regulates 3D-specific cell motility processes, possibly related to cell-extracellular matrix interactions. Pairwise comparison of experimental and control sample identified significant hits that were analyzed by unsupervised hierarchical clustering. BAD3SA 3D-unique candidate interactors were identified. Pathway analysis revealed their roles in RhoGTP pathways. Top candidates will be independently confirmed and functionally validated by pharmacological/genetic inhibition.

**Conclusion**

© 2024 The Authors. Molecular Oncology is published by John Wiley & Sons Ltd on behalf of Federation of European Biochemical Societies

Our data will identify molecular players that mediate the BAD effect on migration, which may uncover new cellular processes and druggable targets that inhibit breast cancer metastasis.

### EACR2024-1213
### Methylglyoxal: a potent oncometabolite involved in epigenetic regulation

*A. Overs[1], E. Hervouet[1], P. Peixoto[1], Z. Selmani[1], A. Bellahcene[2]*
*[1]University of Bourgogne Franche-Comté, EPIgenetics and GENe EXPression Technical Platform EPIGENExp, Besançon, France*
*[2]University of Liège, Metastasis Research Laboratory- GIGA Resarch Institute, Liège, Belgium*

**Introduction**

Triple-negative breast cancer (TNBC) represents 15% of breast cancers. This subtype has the worst prognosis as it is generally resistant to standard chemotherapy. Therefore, it is an urgent need to develop therapies based on novel strategies. TNBC metabolic profiling indicates that this subtype of breast tumors is generally glycolytic. Methylglyoxal (MG) is a very reactive molecule derived from glycolysis. MG interacts with DNA and proteins to form Advanced Glycation Products (AGEs). Our previous studies have demonstrated that MG glycating stress triggers enhanced tumor growth and metastasis in breast cancer. Glyoxalase 1 (GLO1) enzyme is the rate-limiting step for the detoxification of MG that is present in all mammalian cells.

**Material and Methods**

We generated breast cancer cell lines stably depleted for GLO1 to induce an endogenous MG stress. TNBC/glycolytic (MDA-MB-231) and estrogen receptor positive/non glycolytic (MCF-7) cell lines will be used to characterize the impact of MG stress. We performed RNA sequencing and 850K CpGs array on GLO1-depleted MDA-MB-231 cells to study gene expression and methylation profiles, respectively. Protein expression of genes of interest was validated using western-blot and RT-QPCR.

**Results and Discussions**

MDA-MB-231 transcriptomic analysis revealed a pro-metastatic MG signature notably comprising the regulation of invasion and metastasis-related genes. Interestingly, the expression of major epigenetic regulators, such as genes coding for de novo DNA methyltransferases and for demethylases was significantly modulated, indicating an impact of MG stress on epigenetic regulation. The integrative analysis of gene expression data with gene promoter methylation status notably revealed a significant down-regulation of several tumor suppressor genes under MG stress. The detailed analysis of these omics data, and their comparison with those to be generated from GLO1-depleted MCF-7 hormone-receptors positive cells, will help us determine how oncogenes are regulated under MG stress? What is the status of these genes in non metastatic and non glycolytic MCF-7 breast cancer cells under MG stress?

**Conclusion**

We observed that MG stress induces the hyper-methylation of the promoters of specific cancer-related genes in TNBC. The analysis of gene expression will help us characterize an MG signature to be further validated on large series of breast tumors. Finally, the search for a potential prognosis value of MG signature will be achieved on collections of early breast cancer.

### EACR2024-1248
### PI3K signaling controls metabolic adaptations in pancreatic cancer

*B. Thibault[1], C. Cayron[1], M. Di-Luoffo[1], N. Therville[1], C. Guyon[1], J.E. Sarry[2], J. Guillermet-Guibert[1]*
*[1]Inserm- CRCT, Team SigDyn, Toulouse, France*
*[2]Inserm- CRCT, Team MetAML, Toulouse, France*

**Introduction**

Patients with pancreatic cancer (PDAC) have a low survival rate and poorly benefit from chemotherapeutic treatments. KRAS mutations are at the origin of this cancer in 80% of cases. The PI3K pathway is a downstream target of KRAS that regulates glycolysis and insulin response. However, the importance of PI3K isoforms in the regulation of tumour metabolism in PDAC is unknown. This knowledge is crucial as modulation of tumour metabolism in PDAC is a promising therapeutic strategy.

**Material and Methods**

Using transcriptomic databases of human pancreatic tumours and transcriptomic data from human pancreatic organoids, we searched for specific metabolic signatures associated with the level of PI3K pathway activation. We created a novel murine model where PI3Ks are genetically inactivated in established tumours to study evolution of metabolic gene signatures at single cell level. We analysed metabolic flexibility after long-term treatment with PI3K inhibitors (vehicle, BYL-719 (PI3Kα selective inhibitor), IPI-549 (PI3Kγ selective inhibitor) or the combination of BYL-719 and IPI-549) in vitro (cell lines) or in vivo.

**Results and Discussions**

Patients with low PI3K activity exhibited characteristics of the classic pancreatic cancer subtype, known to present a better prognosis. These tumours (PI3Klow) showed distinct metabolic signatures such as oxidative phosphorylation, glycolysis, TCA cycle and fatty acid metabolism, whereas tumours with high PI3K activity were enriched for the epithelial-mesenchymal transition signature, an indication of aggressiveness. In human pancreatic adenocarcinoma samples, high levels of PI3Kα and PI3Kγ in the tumour compartment is associated with high levels of mitochondrial complexes and mitochondrial biogenesis proteins. Modulation of PI3K activity altered metabolic dependencies (redirection towards complete glucose oxidation metabolism) and sensitized cells to its targeting. Under gemcitabin chemo-therapeutic regimen, PI3Klow PDAC cells were less flexible than PI3Khigh PDAC cells. In vitro and in vivo, pancreatic tumour cells treated with the combination of BYL-719 and IPI-549 were more sensitive to the combination of gemcitabine + metabolic modulators (CPI-613, a lipoic acid analogue that blocks the activity of α-KG and PDH.

**Conclusion**

Inhibiting PI3Ks is a proposed way to force pancreatic cancer tumour cells into a metabolism state sensitive to

© 2024 The Authors. Molecular Oncology is published by John Wiley & Sons Ltd on behalf of Federation of European Biochemical Societies combinations of chemotherapy drugs and metabolic inhibitors.

### EACR2024-1251
### Patient-derived organoids platform to establish personalized treatment for colorectal cancer

*M. Andreu Carbó[1], G.M. Ramzy[1], J. Meyer[2], C. Toso[2], L. Rubbia Brandt[3], P. Meraldi[4], P. Nowak-Sliwinska[5]*
*[1]University of Geneva, Molecular Pharmacology Group- School of Pharmaceutical Sciences / Department of Cell Physiology and Metabolism, Geneva, Switzerland*
*[2]University Hospitals of Geneva, Department of Visceral Surgery, Geneva, Switzerland*
*[3]University Hospitals of Geneva, Division of Clinical Pathology- Diagnostic Department, Geneva, Switzerland*
*[4]University of Geneva, Department of Cell Physiology and Metabolism, Geneva, Switzerland*
*[5]University of Geneva, Molecular Pharmacology Group- School of Pharmaceutical Sciences, Geneva, Switzerland*

**Introduction**
Colorectal cancer (CRC) is one of the most diagnosed and deadly cancers worldwide. Despite the advancements in cancer treatment strategies, the main treatment modality for CRC still relies on conventional chemo-therapy which lacks selectivity, and often leads to the development of side effects and drug resistance. Given the heterogeneous nature of CRC, there exists a need to tailor treatments to individual patients based on their specific tumor characteristics.

**Material and Methods**
To address this challenge, we previously validated a pipeline for personalized CRC treatment by employing our phenotypic approach called Therapeutically Guided Multidrug Optimization (TGMO) on patient-derived organoids (PDOs). Here, we report the successful establishment and expansion of PDOs from freshly isolated colon samples, both healthy and tumor tissue. By growing single organoids per well at standardized size (300-450 µm), we evaluated the efficacy and selectivity of different drug combinations on tumor PDOs compared to normal PDOs.

**Results and Discussions**
Starting with a pool of 8 drugs targeting diverse signaling pathways, we aim to determine the optimal low-dose synergistic drug combination (ODC) for each patient. Employing our TGMO-based screen and modeling we identified personalized ODCs, which are patient-specific. For instance, in PDOs derived from patient 44, a combination of four drugs containing tyrosine kinase receptor inhibitors and protein kinase inhibitors exhibited high selectivity towards tumor cells. This ODC efficiently decreased the viability of tumor PDOs while leaving the viability of healthy PDOs unaffected. Moreover, given that each patient's tumor exhibits a unique molecular profile, we intend to study the molecular mechanism underlying the ODC efficacy. In addition, the increased number of patient samples will allow us to predict and give a faster personalized therapy based on ODC efficacy and molecular characterization.

**Conclusion**
Altogether, we propose a "bench to bedside and back" approach, focusing on the optimization of synergistic multidrug combination therapies patient-specific within a clinically relevant timeframe.

### EACR2024-1252
### Exploiting synthetic-lethal interactions for therapeutic development against aggressive MYC-driven tumors

*B. Amati[1]*
*[1]European Institute of Oncology, Dept. of Experimental Oncology, Milan, Italy*

**Introduction**
The oncogenic transcription factor MYC is induced by growth-promoting stimuli and drives the activation of biosynthetic and metabolic pathways inherent to proliferating cells. The same pathways are deregulated in MYC-driven tumors where they contribute to cell proliferation and survival, but concomitantly elicit multiple stresses, to which cancer cells must adapt during disease progression. Hence, MYC-overexpressing cells depend on a fragile equilibrium between conflicting signals, which creates opportunities to exploit synthetic lethality as a strategy for targeted therapeutic intervention.

**Material and Methods**
We use B-cell lines with conditional activation of MYC, as well as a series of MYC-driven lymphoma models to explore the therapeutic potential of targeted drug combinations against aggressive forms of B-cell lymphoma.

**Results and Discussions**
Work in our laboratory revealed that MYC activation sensitizes cells to drugs inhibiting distinct mitochondrial activities: tigecycline, an antibiotic that cross-reacts the mitochondrial ribosome [1], and IACS-010759, a selective inhibitor of the electron transport chain (ETC) complex I [2]. In preclinical models, tigecycline and IACS-010759 independently synergized with the BCL2 inhibitor venetoclax (ABT-199) against double-hit lymphoma (DHL), a high-grade form of B-cell lymphoma with concurrent activation of the *MYC* and *BCL2*oncogenes [2, 3]. Our mechanistic analysis unraveled important roles for (i.) oxidative stress, (ii.) the integrated stress response (ISR) signaling pathway and (iii.) the intrinsic apoptotic machinery in the drug-induced killing of MYC-overexpressing cells [2, 4].

**Conclusion**
In a number of tumor types, including MYC-driven lymphoma, the ISR was shown to have cytoprotective, pro-tumoral effects. Thus, our data and others reveal that the reciprocal is also true, with super-activation of the ISR contributing to the cytotoxic action of diverse drugs (reviewed in ref. *5*). Our current research aims to unravel the mechanistic basis of this dual effect and to understand how it might be further exploited toward therapeutic development.

*1) D'Andrea et al. Oncotarget 7, 72415-72430 (2016).*
*2) Donati et al. Mol Oncol 16, 1132-1152 (2022).*
*3) Ravà et al. Sci Transl Med 10, eaan8723 (2018).*
*4) Donati et al. EMBO Mol Med 15, e16910 (2023).*
*5) Donati et al.Mol Oncol 16, 3828-3854 (2022).*

© 2024 The Authors. Molecular Oncology is published by John Wiley & Sons Ltd on behalf of Federation of European Biochemical Societies

### Cancer Genomics

### EACR2024-0008
### The role of mir29a and mir143 in the control of anti-apoptotic MCL-1/cIAP-2 genes in non-small cell lung cancer patients

*Z. Abrehdari-Tafreshi*[1], *E. Arefian*[2], *M. Arab Najafia*[1]
[1]*University of Tehran, Cell and Molecular Biology, Tehran, Iran*
[2]*University of Tehran, Microbiology, Tehran, Iran*

**Introduction**
Lung cancer is a fatal disease worldwide with greater than 1 million deaths each year. Targeted therapy has been used to block cancer growth and proliferation by developing new drugs. Though, the survival rate of lung cancer is low because of the high frequency of drug resistance in patients with mutations in the driver genes. Overexpression of anti-apoptotic genes is one of the most significant features of tumor drug resistance. EGFR signaling make the expression of anti-apoptotic genes. In addition, microRNAs have a critical task in regulating biological functions such as apoptosis; a process mostly evaded in cancer progression.
**Material and Methods**
Bioinformatics analysis predicted that *miR-29a* and *miR-143* might regulate *MCL-1* and *cIAP-2* expression. We surveyed the expression of *MCL-1*, *cIAP-2*, *miR-29a*, and *miR-143* encoding genes in adenocarcinoma patients with or without *EGFR* mutations. The potential role of *miR-29a* and *miR-143* on gene expression was evaluated by overexpression and luciferase assays in HEK-293T cells.
**Results and Discussions**
The expression levels of *MCL-1* and *cIAP-2* genes in patients with mutated *EGFR* were higher than those of wild-type *EGFR*. Contrary, compared to those of patients with wild-type *EGFR*, the expression levels of *miR-29* and *miR-143* were lower in the patients carrying *EGFR* mutations. In cell culture, overexpression of *miR-29a* and *miR-143* considerably downregulated the expression of *MCL-1* and *cIAP-2*. Dual-luciferase reporter experiments confirmed that *miR-29a* and *miR-143* target *MCL-1* and *cIAP-2* mRNAs, respectively.
**Conclusion**
Our results suggest that upregulation of EGFR signaling in lung cancer cells may increase anti-apoptotic *MCL-1* and *cIAP-2* gene expression, possibly through downregulation of *miR-29a-3p* and *miR-143-3p*.

### EACR2024-0017
### Variant-Specific Landscape of Mutual Exclusivity Among BRAF, EGFR, and KRAS Oncogenes in Human Cancer: Roadmap for Targeted Therapies

*M. Noeparast*[1], *F. Vaeyens*[2], *J.P. Hetzel*[3], *M. Mernberger*[3], *C. Olsen*[2], *K. Maes*[2], *F. Hes*[2], *M. Pichler*[1], *P. Giron*[2], *O. Timofeev*[3]
[1]*Universitätsklinikum Augsburg, AG Pichler: Translational Oncology- University Medical Center Augsburg- Augsburg- Germany, Augsburg, Germany*
[2]*Vrije Universiteit Brussel, Centre for Medical Genetics- Research Group Reproduction and Genetics- Clinical Sciences- Laarbeeklaan 101- 1090 Brussels- Belgium, Brussels, Belgium*
[3]*Institute of Molecular Oncology- Member of the German Center for Lung Research DZL- Philipps University- 35043 Marburg- Germany, Institute of Molecular Oncology- Member of the German Center for Lung Research DZL- Philipps University- 35043 Marburg- Germany, Marburg, Germany*

**Introduction**
Mutual exclusivity (ME) and co-occurrence (CO) patterns of gene mutations can be associated with the redundancy or diversification of functions, synthetic lethality, or senescence. Understanding these patterns is crucial for mutation-specific targeting. Regarding BRAF, KRAS, and EGFR oncogenes—the three actionable targets in human cancer—ME patterns have been extensively studied in the past two decades. Today, the consensus, inspired by lung cancer studies, is that EGFR/KRAS and BRAF/KRAS gene mutations are typically mutually exclusive. However, a significant limitation of the previous studies is that they do not simultaneously consider different mutational classes and variants. Instead, at least on one side of the analysis, all mutations of the same gene are pooled together.
**Material and Methods**
In this cross-sectional study, we present the results of our investigation into the mutual exclusivity and co-occurrence patterns of BRAF, KRAS, and EGFR mutations in human cancer. Our analysis takes into account previously overlooked mutational subtypes with distinct clinical implications. Creating an automated R framework, we analyzed mutation data and performed co-occurrence analysis on 64,807 unique cBioPortal samples, 1,570 cell lines, and 2,714 unique Belgian cancer samples.
**Results and Discussions**
Consistently, across all three datasets, co-occurrence is less likely among class I BRAF, hydrolysis KRAS, and classical-like EGFR mutations. Bilateral variant-assigned co-occurrence matrices reveal several novel inter-class and inter-type co-occurrence and mutual exclusivity scenarios, encompassing both conventional and atypical mutations. In the publicly available database, only 9 samples harbored such exceptional pairs where extreme ME scenarios still co-occurred. Interestingly, 4/6 of samples with existing information on the allele frequency (AF) for each oncogene in a tumor had an AF for one of the co-occurring components (KRAS) smaller than 10 percent. Apart from Class I BRAF, various mutation classes demonstrate diverse co-occurrence patterns, underscoring the necessity to refine mutational classifications.
**Conclusion**
We provide a variant-specific database for precision oncology showcasing ME among three actionable oncogenes. While challenging it in specific scenarios, we add a significant layer of complexity to the actual consensus on ME among BRAF, KRAS, and EGFR oncogenes. Finally, we propose a conceptual framework for leveraging this unveiled ME landscape to discover novel targets against human cancer.

© 2024 The Authors. Molecular Oncology is published by John Wiley & Sons Ltd on behalf of Federation of European Biochemical Societies

### EACR2024-0077
### Investigation of the combined use of PARP inhibitors for targeted therapy in cholangiocarcinoma with different DNA damage response mutations

*T. Lerksuthirat*[1], *S. Prasopporn*[2], *R. Wikiniyadhanee*[3], *S. Chitphuk*[1], *W. Stitchantrakul*[1], *P. Own-eium*[3], *S. Jirawatnotai*[2], *D. Dejsuphong*[3]
[1]*Faculty of Medicine Ramathibodi Hospital Mahidol University, Research Center, Bangkok, Thailand*
[2]*Faculty of Medicine Siriraj Hospital Mahidol University, Department of Pharmacology, Bangkok, Thailand*
[3]*Chakri Naruebodindra Medical Institute Faculty of Medicine Ramathibodi Hospital Mahidol University, Program in Translational Medicine, Samutprakarn, Thailand*

**Introduction**
Cholangiocarcinoma (CCA) poses a significant challenge as a biliary tract carcinoma, characterized by its inherent heterogeneity and limited treatment options. Genetic alterations in DNA damage response (DDR) pathways are commonly observed in CCA, suggesting that the disease may be driven by a homologous recombination (HR) defect. This leads to increased interest in the use of ataxia-telangiectasia and Rad3-related protein (ATR) and poly(ADP-ribose) polymerase (PARP) inhibitors. This study aimed to investigate the impact of ATR and various PARP inhibitors, both individually and in combination, on CCA cell lines with different DDR mutation backgrounds.
**Material and Methods**
The genetic alterations of the DDR genes in CCA cell lines were analyzed. These cell lines were treated with PARP inhibitors (olaparib, talazoparib, veliparib) and an ATR inhibitor (AZD6738), both alone and in combination, to assess cellular viability, proliferation, and the combination index. Changes in DNA damage were evaluated using micronuclei and γ-H2AX focus formation assays.
**Results and Discussions**
Our results showed that a patient-derived cell line, harboring a pathogenic mutation in its DDR genes, was particularly sensitive to the PARP inhibitors. Interestingly, AZD6738 exhibited a more robust cytotoxic effect compared to the PARP inhibitors alone. The current investigation focuses on determining whether the combination of AZD6738 and various PARP inhibitors produces a synergistic effect against CCA cell lines.
**Conclusion**
This study advances knowledge on synthetic lethality's potential in personalized medicine for CCA, addressing the urgent need for effective treatments.

### EACR2024-0084
### Exploring notch signaling pathway mutations in primary breast cancer among the Indian population: a comprehensive genetic analysis

*H. Goel*[1], *R. Kumar*[2], *P. Tanwar*[2]
[1]*All India Institute of Medical Sciences, Laboratory Oncology, New Delhi, India*
[2]*AIIMS, Laboratory Oncology, New Delhi, India*

**Introduction**
Breast cancer (BC) is a prevalent global malignancy, accounting for 11.7% of cancer diagnoses worldwide and 13.5% in India. Notch signaling, a canonical oncogenic pathway pivotal for cell growth and apoptosis, exhibits frequent genetic alterations, however, its mutation prevelance remains unknown among Indian BC patients. This study aims to elucidate the mutation profile of Notch pathway-related genes through whole exome sequencing followed by Sanger sequencing.
**Material and Methods**
Tumor samples and adjacent normal tissues were collected postoperatively from 23 BC patients. Genomic DNA was extracted using salting out method, Library preparation was done as per manufacturer instruction and the Illumina platform was used for whole exome sequencing with an average depth coverage of 100X. Raw reads were analyzed using an in-house pipeline. MAML3 and NOTCH4 deletions were validated via Sanger sequencing. Additionally, mRNA expression of MAML3 and NOTCH4 was assessed using quantitative PCR.
**Results and Discussions**
Among the 10 canonical pathways, the NOTCH pathway exhibited the highest mutation frequency, with alterations identified in all 25 patients. A total of 47 mutated genes were identified, comprising 16 tumor suppressors, 3 oncogenes, and 28 passenger genes. MAML3 and NOTCH4 demonstrated somatic mutation rates of 17.5% and 20%, respectively. MAML3 mutations included missense and frameshift deletions, while NOTCH4 exhibited in-frame deletions and missense mutations. Sanger sequencing confirmed these mutations. Both MAML3 and NOTCH4 showed significantly decreased expression compared to normal tissues.
**Conclusion**
This study provides comprehensive insights into the frequently mutated genes within the NOTCH pathway, with MAML3 and NOTCH4 emerging as predominantly mutated genes in Indian BC cases. These findings hold promise for utilizing these genes in risk assessment, screening, diagnosis, and early prognosis of BC. The study contributes to an enhanced understanding of the genetic landscape of BC among the Indian.

### EACR2024-0149
### Genetic Concordance in Melanoma: Insights from early-stage Primary Tumors and their matched Distant Metastases

*T. Kerkour*[1], *L. M. Hollestein*[1], *T. Nijsten*[1], *A. Mooyaart*[2]
[1]*Erasmus MC, Dermatology, Rotterdam, Netherlands*
[2]*Erasmus MC, Pathology, Rotterdam, Netherlands*

**Introduction**
Revealing the conserved genetic patterns within primary tumors undergoing metastasis is critical for optimal treatment selection and prognosis. Here, we compared the genetic profile of primarily thin primary melanoma and matched metastasis to assess their genetic clonal relationship.

© 2024The Authors. Molecular Oncology is published by John Wiley & Sons Ltd on behalf of Federation of European Biochemical Societies

**Material and Methods**
Using a targeted sequencing panel encompassing 330 amplicons, we targeted hotspot regions in 41 cancer genes and 154 single nucleotide polymorphisms (SNPs). We compared the mutational status and the copy number variations profile in 17 patients with primarily thin melanomas and/or a long latency between the primary melanoma and the distant metastasis. We evaluated tumor-relatedness using hierarchical clustering.
**Results and Discussions**
*BRAF* V600, *TERT* promotor, and *NRAS* Q61 mutations were the most detected variants within primary and metastatic melanoma tumors, with a frequency of 68%, 56%, and 18%, respectively. Of the patients tested 15 (88%) had primary and metastatic melanomas with the exact clonal origin, and two exhibited unshared genetic patterns.
**Conclusion**
The mutational status concordance is high during melanoma progression. Further investigations are warranted to explore the predictive value of the clonal patterns in metastasis risk prediction.

### EACR2024-0193
### Immune metabolism predicts cancer patient response to checkpoint immunotherapy

O. Shorer[1], K. Yizhak[1,2]
[1]*Technion - Israel Institute of Technology, The Ruth and Bruce Rappaport Faculty of Medicine, Haifa, Israel*
[2]*Technion - Israel Institute of Technology, The Taub Faculty of Computer Science, Haifa, Israel*
**Introduction**
Over the past decade cancer therapy has been revolutionized by the usage of immune checkpoint inhibitors (ICI), resulting in unprecedented rates of long-lasting tumor responses in patients with a variety of cancers. Nevertheless, most patients do not respond to treatment or acquire resistance. To address this challenge, multiple biomarkers of response have been suggested; however, they are rarely predictive across datasets, and certainly across different cancer types. More recently, differences in the metabolic adaptation of tumor and immune cells and their competition for resources have been shown to contribute to patient response. Therefore, studying the metabolic transcriptomic changes that occur in immune cells found in the tumor microenvironment (TME) of patients treated with ICI, can shed light on mechanisms of response to therapy and highlight immune metabolic vulnerabilities.
**Material and Methods**
In this study we performed a semi-supervised analysis of ~1700 metabolic genes using single-cell RNA-seq data of >1 million immune cells from ~230 samples of cancer patients treated with ICI. We utilized a collection of datasets containing longitudinal tumor biopsies and blood samples from major cancer types such as melanoma, lung and breast cancers. We applied various computational methods in order to detect predictive metabolic signatures for patient response across multiple cancer types.
**Results and Discussions**
When clustering cells based on their metabolic gene expression, we found that different immune cell states share similar metabolic activity and vice versa, resulting in a novel classification to cellular states, mainly involving $CD8^+$ T cells. Furthermore, we detected metabolic clusters that are significantly associated with patient response. A non-discrete analysis of metabolic programs revealed a metabolic gene signature that is significantly associated with tumor progression or regression across various cancers. Finally, we show a metabolic involvement in the polarization of macrophages to a suppressive M2-like phenotype in an acquired resistance patient, together with the associated tumor metabolic changes.
**Conclusion**
In this study, we metabolically analyzed immune cells taken from cancer patients treated with ICI. Taken together, our results demonstrate the importance of metabolic processes in determining patient response to therapy across different cancer types.

### EACR2024-0199
### Matched fresh frozen and FFPE patient tissues reveal the enhanced sensitivity and data quality of a novel DNA library prep method

M. Petridis[1], M.R. Heider[2], J. Sun[2], A. Reich[2], B.S. Sexton[2], B.W. Langhorst[2], P. Liu[2]
[1]*New England Biolabs, Applications, Frankfurt am Main, Germany*
[2]*New England Biolabs Inc., Applications and Product Development, Ipswich - MA 01938, United States*
**Introduction**
In cancer genomics, a common source of DNA is formalin-fixed, paraffin-embedded (FFPE) tissue from patient surgical samples, where in most cases high quality fresh or frozen tissue samples are not available. FFPE DNA poses many notable challenges for preparing NGS libraries, including low input amounts and highly variable damage from fixation, storage, and extraction methods. It is difficult to obtain libraries with sufficient coverage and the sequencing artifacts arising from damaged DNA bases confound somatic variant detection.
**Material and Methods**
We developed a novel NGS library prep method compatible with both high quality and very low quality FFPE DNA samples, employing a novel enzymatic DNA repair mix, enzymatic fragmentation mix, and PCR master mix. To validate this workflow on real patient sample sets, we obtained DNA extracted from matched tumor and normal tissue of various tissue types preserved by both fresh frozen and FFPE methods. The FFPE DNA samples ranged in quality from DNA integrity number (DIN) 1.5 to 6.8. We prepared libraries using this method compared against other library prep workflows and sequenced them by WGS and target capture. We assessed the performance of these workflows by library yield, library quality metrics, depth and evenness of coverage, and somatic variant calling with fresh frozen-extracted DNA providing the gold standard for library quality and mutation content.
**Results and Discussions**

© 2024 The Authors. Molecular Oncology is published by John Wiley & Sons Ltd on behalf of Federation of European Biochemical Societies

This new enzymatic fragmentation-based library prep workflow not only reduced the false positive rate in somatic variant detection by repairing damage-derived mutations in FFPE DNA samples but also improved the library yield, library quality metrics, library complexity, coverage depth, and coverage uniformity. Comparing the variant calls from matched FFPE and frozen tissues revealed an improved sensitivity and accuracy of variant calling using this library prep method compared to mechanical shearing and other enzymatic fragmentation library prep approaches.

**Conclusion**

This new suite of enzyme mixes allows even highly damaged FFPE samples to achieve high quality libraries with a greater sensitivity for somatic variant identification. The workflow is robust and flexible, compatible with both FFPE DNA and matched high quality DNA samples as well as automation-friendly for convenience in sample processing.

### EACR2024-0312
### Targeting super-enhancer-driven oncogenic transcription in melanoma with a novel synthetic molecule

*J. Obid[1], M. Cigrang[1], M. Nogaret[1], F. Coin[1]*
*[1]I.G.B.M.C. - Institut de génétique et de biologie moléculaire et cellulaire, Functional Genomics and Cancer, Illkirch-Graffenstaden, France*

**Introduction**

Cutaneous malignant melanoma is one of the most aggressive forms of cancer, mainly due to the emergence of heterogenic invasive and drug-resistant melanoma cellular phenotypes. This underscores the urgent need for new therapeutic approaches uniformly targeting the different melanoma cellular states. Transcriptional addiction is a cancer hallmark defined as the high dependence of cancer cells on the robust transcriptional activity of specific oncogenes, promoting their proliferation and survival. One of the main mechanisms leading to this phenomenon consists in the acquisition of large clusters of enhancers known as super-enhancers (SEs), bound by extremely high levels of ubiquitous transcription factors allowing the robust expression of oncogenes. Hence, our laboratory has addressed this challenge by developing and testing Compound IA (Comp IA), a synthetic transcriptional inhibitor derived from Lurbinectedin, which is an established anti-cancer agent already used for metastatic small cell lung cancer treatment.

**Material and Methods**

To assess the efficiency of our molecule, IC50s of 6 melanoma cell lines towards Comp IA were measured with PrestoBlue. In vivo studies were conducted on Nude mice that were subcutaneously inoculated with LOX-IMVI melanoma cells, and their survival and tumor growth were then measured following treatment with Placebo or Comp IA. Finally, RNA-seq & Chem-map using biotinylated version of Comp IA were performed to determine its genomic and transcriptional targets in melanoma cells.

**Results and Discussions**

Through extensive in vitro and in vivo evaluations, we have demonstrated a potent anti-cancer activity of Comp IA against a large panel of melanoma cells, including those exhibiting resistance to conventional treatments. Moreover, our comprehensive genomic analyses have revealed that Comp IA heavily downregulates the expression of lineage-specific melanoma oncogenes through two-distinct mechanisms. Firstly, Comp AI binds to the promoters of these oncogenes, impeding the formation of pre-initiation complex necessary for transcription initiation. Secondly, it inhibits the expression of the ubiquitous transcription factors and co-activators known to be heavily enriched at the super-enhancers (SEs) of these oncogenes.

**Conclusion**

Collectively, our findings underscore the promising potential of targeting transcriptional addiction and provide a compelling rationale for investigating Comp IA in clinical trials for melanoma patients having developed resistance to conventional therapies.

### EACR2024-0317
### Automating cell-free DNA library preparation and hybridization capture for breast cancer panel

*N. Roseman[1], L. Tucker[1], L. Ren[1], D. Wang[2], K. Larkin[3], A. Dvorak[3], J. Topp[4], Z. Smith[5], I. Shoemaker[5]*
*[1]Integrated DNA Technologies, Research and Development, Coralville, United States*
*[2]Integrated DNA Technologies, Research and Development, Redwood City, United States*
*[3]Integrated DNA Technologies, Applications Development, Coralville, United States*
*[4]Integrated DNA Technologies, Scientific Applications Support, Coralville, United States*
*[5]Beckman Coulter Life Sciences, Applications Development, Indianapolis, United States*

**Introduction**

Automation is often counted on in laboratories to generate reproducible next generation sequencing (NGS) sample libraries. Automated workflows need to work well and consistently for cancer research utilizing low input cell-free DNA (cfDNA) from liquid extractions. The Biomek NGeniuS Next Generation Library Prep System provides a flexible sample batch size in a closed and controlled instrument while also allowing for technicians to walk away, reduce hands on time, errors, and re-work. Integrated DNA Technologies (IDT) xGen™ cfDNA & FFPE DNA Library Prep kit utilizes novel chemistry to maximize sample input conversion, suppress adapter-dimer formation, and facilitate consensus analysis. IDT's xGen Hybridization Capture products maintain high library diversity, obtain high on-target, and provide consistent and uniform sequencing coverage regardless of panel size.

**Material and Methods**

Apostle MiniMax™ High Efficiency cfDNA Isolation Kit was used to isolate cfDNA from plasma collected from blood collection tubes. Blood samples were drawn from donors who had breast cancer diagnoses. xGen™ cfDNA & FFPE DNA Library Prep kit was used to prepare cfDNA libraries from 10ng input of plasma extracted material on the Biomek NGeniuS Next Generation Library Prep system (n=6). Hybridization capture was then also run on the Biomek NGeniuS

© 2024 The Authors. Molecular Oncology is published by John Wiley & Sons Ltd on behalf of Federation of European Biochemical Societies system with an xGen™ breast cancer hyb panel and xGen™ Hybridization Capture Core Reagents (n=6). xGen™ breast cancer hyb panel targets approximately 80 mutated genes for target pull down.

**Results and Discussions**

Low depth WGS analysis to evaluate library quality demonstrated high mapping rates of ≥99.2%, almost zero dimer percentage of ≥0.2%, and a low percentage of chimera molecules at ≥2%. Hybridization capture samples with xGen Hyb panels yield high library diversity, high on-target, and provide consistent and uniform sequencing coverage. The combination of this workflow within a closed automation instrument, provides a reliable and consistent solution for analysis of low input cancer research samples and we present expected allele frequencies against the sample targeted space.

**Conclusion**

The combination of IDT's xGen library preparation and hybridization capture on the Biomek NGeniuS Next Generation Library Prep System has enabled variant identification across a range of variant allele frequencies from plasma extracted cfDNA, providing a walk away solution for researchers.

### EACR2024-0342
### Stereo-seq FFPE reveals the spatial profilings of long non-coding RNAs in lung cancer FFPE sample

*J. Yuan[1], Y. Zhao[1], Y. He[1], Z. Li[1], J. Zhang[1], D. Li[1], X. Zhang[1], S. Liao[1], A. Chen[1]*

*[1]BGI Research, BGI Research, Shenzhen, China*

**Introduction**

Stereo-seq is the state-of-art technology for investigation of spatially resolved high resolution multi-omics data. Here, we present the new stereo-seq FFPE which is compatible with FFPE samples promising the clinico-pathogenesis-associated discoveries.Long non-coding RNAs (lncRNAs) play crucial roles in regulating development and progression of cancer. The spatial information allows the investigation of cellular origin, intracellular molecular activities, intercellular communications of lncRNAs to support comprehensive understanding of the underlying mechanisms. In this study, we presented a novel spatial transcriptomics technology called stereo-seq FFPE, which utilizes random primers for total RNA capture including lncRNAs and demonstrated its ability in decoding the tumor immune microenvironment (TME).

**Material and Methods**

Stereo-seq FFPE uses random probes to capture total RNA with a spatial resolution of diameter 220nm. We applied stereo-seq FFPE on the FFPE lung cancer sample to obtain the spatially resolved transcriptomic data. We used Cell2location for cell annotation and Scanpy for clustering at resolution of bin50. SPARK was applied to identify spatially variable lncRNAs and mRNAs. InferCNV was applied to identify malignant cells. And StereoSiTE was applied to analyze cell communication activities.

**Results and Discussions**

We depicted TME landscape of lung cancer with spatial transcriptomics. We annotated seven major cell types including epithelial cells and different immune cells. The result of clustering was aligned with pathologist's annotation. We then analyzed the spatial profiling of lncRNAs and discovered spatially featured lncRNAs, such as MALAT1 and NEAT1. MALAT1 and NEAT1 have been reported to be associated with the development of lung cancer. We discovered that the expression of MALAT1 can be spatially associated with the expression of an mRNA, PTPN13, which was a novel finding underlying pathogenesis of lung cancer. Finally, we observed significantly higher expression of MALAT1 and NEAT1 in malignant cells, and found different cell communication activities between regions with high and low expression of MALAT1.

**Conclusion**

Stereo-seq FFPE enables high-resolution spatial profiling of both mRNAs and lncRNAs simultaneously. Therefore, we can analyze the regulatory effects of lncRNAs on mRNA and investigate its associations with the TME. Stereo-seq FFPE paves the ways for discoveries of in-depth mechanisms, drugable targets, and new treatment strategies.

### EACR2024-0343
### Single-cell RNA sequencing reveals actinic keratosis and squamous cell carcinoma pathogenesis

*S.P. Hong[1], K.H. Min[2], J.Y. Choi[2], D.S. Yu[2], C. Bukyoung[3], J.I. Kim[1,3], Y.B. Lee[2]*

*[1]Seoul National University College of Medicine, Department of Biomedical Sciences, Seoul, South Korea*

*[2]Catholic University College of Medicine, Department of Dermatology, Seoul, South Korea*

*[3]Seoul National University, Genomic Medicine Institute-Medical Research Center, Seoul, South Korea*

**Introduction**

Actinic keratosis (AK) is a common pre-cancerous keratinocyte lesions resulting from UV exposure. Although AK rarely progress to squamous cell carcinoma (SCC), two-third of SCC arise from AK. Even if many studies have attempted to elucidate the pathogenesis of AK and SCC, there is a lack of research at single cell level using samples from same patient. To understand pathogenesis AK and SCC, we performed single cell RNA sequencing (scRNA-seq) from normal-AK-SCC matched skin sample of 4 patient. In this study, we described between keratinocytes and Tumor micro-environment (TME) landscape and interaction, providing insight of AK and SCC pathogenesis.

**Material and Methods**

Adjacent normal-AK-SCC matched skin samples of four patients were collected from the department of dermatology, Uijeongbu St. Mary's Hospital. The fresh tissues enzymatically digested using Multi Tissue Dissociation Kit and GentleMACS dissociator (Miltenyi Biotec).scRNA-seq was performed with the 10X Chromium 3' v3.1 and sequenced on the NovaSeq6000 system (Illumina) in paired-end mode. Raw sequenced data were aligned to the GRCh38 human reference genome using Cell Ranger (v7.1.0) and were processed using Scanpy (v1.9.6) and Seurat (v4.3.0). To identify significant different pathways and genes between groups, we used pydeseq2 (v0.4.4), gseapy (v1.1.0) and

© 2024 The Authors. Molecular Oncology is published by John Wiley & Sons Ltd on behalf of Federation of European Biochemical Societies decoupler (v1.5.0). We used CellChat (v2.1.1) for analyzing cell-cell communication to identify between tumor cells and TME cells and used CellRank (v2.0.2) for trajectory analysis.

**Results and Discussions**

Using scRNA-seq analysis, We identified several keratinocyte lineages and characterized tumor specific keratinocytes (TSKs). TSKs were largely divided sub-clusters with differentiated features and undifferentiated features. Trajectory analysis showed clues of progression from AK to SCC. We also found that normal, AK and SCC group had significant different cellular composition. The existence of tumor associated cells could help to explain the pathogenesis of the tumor. There were many cellular interaction between TSKs and TME specifically through immune and collagen signaling pathway.

**Conclusion**

We comprehensively described AK and SCC features and pathogenesis. Our finding might provide better understand AK, SCC and cancer progression.

### EACR2024-0346
### Deciphering the role of tumor-secreted neuropeptides in mediating neuroimmune axis in head and neck cancer

_R. Kishan_[1], _W. Yang_[1], _R. Su_[1]
[1]*The University of Hong Kong, Faculty of Dentistry, Hong Kong, Hong Kong-China*

**Introduction**

Neuronal infiltration within tumors is a critical determinant of tumor progression, immune suppression and treatment resistance, especially in head and neck cancer (HNSC), where neural innervation is pronounced. Neuropeptides, secreted by solid tumors, are integral to mediating neuronal signals that impact immuno-modulation. Our study aims to scrutinize the expression of neuropeptides in HNSC tumors and their potential role in modulating the neuroimmune landscape.

**Material and Methods**

Utilizing neuropeptide databases and bioinformatics tools leveraging TCGA data, we conducted a comprehensive analysis of neuropeptide expression patterns in HNSC tumors. Through correlation map analysis, we have explored the relationships between neuropeptide expression, immune cell infiltration, immune checkpoint genes (IC), and neurotrophic factors gene expression. Further, datasets from ICBatlas have been analysed to investigate the impact of immunotherapy on neuropeptide expression followed Kaplan Meir analysis.

**Results and Discussions**

More than half of neuropeptide genes have expression in HNSC tumors, with a subset (16%) exhibiting differential expression patterns. Significantly, tumor-derived neuro-peptides demonstrate substantial correlations (>0.3) with immune cell infiltration, IC genes, and neurotrophic factors gene expression, highlighting their pivotal role in shaping the tumor microenvironment. Around 75% of differentially expressed neuropeptide genes are intricately linked to key neuroimmune mediators. Moreover, the extensive correlation observed between the neurotrophic gene NTRK1 and immune checkpoint genes underscores the pervasive nature of neuroimmune crosstalk in HNSC. Further, differential expression of neuropeptide genes has been observed in responder and non-responder patients undergone immunotherapy indicates the involvement of neuropeptides in this axis.

**Conclusion**

This study provides compelling evidence regarding the critical involvement of tumor-derived neuropeptides in neuroimmune regulation within HNSC. These findings not only deepen our understanding of the complex interplay between neural and immune systems but also offer promising avenues for the development of targeted therapeutic interventions. Our study lays the groundwork for future investigations aimed at harnessing the potential of neuroimmune modulation to improve clinical outcomes in HNSC patients.

### EACR2024-0366
### Circulating Tumor DNA as a valuable biomarker for Head and Neck Cancer: a proof of principle study

_L. Paracchini_[1], *L. Lorini*[2], *L. Mannarino*[1], *C. Resteghini*[2], *C. Gurizzan*[2], *F. Riva*[3], *G. Mercante*[4], *B. Fiamengo*[5], *S. Marchini*[6], *P. Bossi*[2]
[1]*IRCCS Humanitas Research Hospital and Humanitas University, Cancer Pharmacology, Rozzano MI, Italy*
[2]*IRCCS Humanitas Research Hospital, Oncology and Hematology, Rozzano MI, Italy*
[3]*Humanitas University, Cancer Pharmacology, Pieve Emanuele MI, Italy*
[4]*IRCCS Humanitas Research Hospital, Otorhino-laryngology, Rozzano MI, Italy*
[5]*IRCCS Humanitas Research Hospital, Pathology, Rozzano MI, Italy*
[6]*IRCCS Humanitas Research Hospital, Cancer Pharmacology, Rozzano MI, Italy*

**Introduction**

Tissue biopsy is the gold standard for diagnosing head and neck cancer (HNC). Given tumor heterogeneity, biopsies may miss important cancer-associated molecular signatures, and, after the tumor is excised or treated with radiation, there is the need to assess the presence of minimal residual disease. Circulating tumor DNA (ctDNA) may identify in vivo molecular genotypes and complement tumor tissue analysis in cancer assessment. However, data on its use in HNC with a tumor-uninformed approach is still lacking.

**Material and Methods**

Five patients with locally advanced or metastatic HNC for whom plasma sample was collected, were selected for this proof-of-principle study. From 1 mL of each plasma sample, circulating-free DNA (cfDNA) was purified and untargeted shallow whole genome sequencing libraries were prepared (HyperPlus, Roche). After barcoding procedure, libraries were pooled and sequenced (Illumina Sequencing System Platform; median coverage 0.5-1X) and ichorCNA was used to estimate the amount of ctDNA (Tumor Fraction, TF) in each sample.

**Results and Discussions**

Three patients had locally advanced disease and two had metastatic disease at the time of enrollment. In all the 5 collected plasma samples ctDNA was quantifiable and ranged from 4.4 ng to 47.2 ng, with mean fragment size

© 2024The Authors. Molecular Oncology is published by John Wiley & Sons Ltd on behalf of Federation of European Biochemical Societies of 185 base pair. Particularly, tumor fraction (TF) values, representing the percentage of ctDNA on the total amount of cfDNA, were detectable and ranged from 4.2% to 48.87%. To note, the patient from whom TF was 48,87% had the highest burden of disease (metastasis at lung, liver and brain).

**Conclusion**

ctDNA analysed with a tumor-uninformed approach represents a valuable and feasible biomarker for HNC, being detected in all samples analyzed, either in patients with locally advanced or metastatic disease. TF levels seems to being representative of tumor burden. Paired data with tissue biopsy and longitudinal evaluation of ctDNA will give more insight on the role of ctDNA evaluation on HNC management.

### EACR2024-0485

### Multi-omic and clinicopathological analysis of BRCA-altered tubo-ovarian high-grade serous carcinoma with exceptionally poor survival:identification of predictive biomarkers for personalized treatment

*T.A. Zwimpfer[1], A. Pandey[1], S. Fereday[1], L. Twomey[1], D. Ariyaratne[1], E.L. Goode[2], B.H. Nelson[3], A. DeFazio[4], D.D.L. Bowtell[1], D. Garsed[1]*

*[1]Peter MacCallum Cancer Centre, Cancer Research, Melbourne, Australia*

*[2]Mayo Clinic, Department of Quantitative Health Sciences, Rochester, United States*

*[3]The Deeley Research Centre, BC Cancer, Victoria, Canada*

*[4]The Westmead Institute for Medical Research, Department of Gynaecological Oncology, Sydney, Australia*

**Introduction**

Loss of *BRCA1* or *BRCA2* in tubo-ovarian high-grade serous carcinoma (HGSC) is associated with better responses to chemotherapy and long-term survival. However, patients often develop acquired resistance and there is currently no predictive markers to differentiate poor or good outcome *BRCA*-driven HGSC. Our study seeks to uncover factors associated with exceptionally poor clinical outcomes, and identify therapeutic vulnerabilities.

**Material and Methods**

Primary tumors from patients with advanced-stage HGSC (n=154) with and without *BRCA* alterations were analyzed using whole-genome sequencing, bulk RNA-sequencing, and DNA methylation profiling, comparing short (≤3-years) and >3-year survival. We also assessed clinicopathological features in 1405 patients with HGSC, including 293 with pathogenic germline *BRCA* variants (g*BRCA*var), in which we also analyzed tumor immune markers by multiplex immunofluorescence.

**Results and Discussions**

Suboptimal primary debulking surgery was strongly associated with poorer overall survival (OS) in non-carriers ($p$<0.001, HR 2.10) but was not significant in g*BRCA*var-carriers with or without residual disease ($p$=0.188 and 0.221, HR 1.17 and 0.8, respectively). While most patients with g*BRCA*var had a more favourable OS compared to non-carriers, this was not seen in those with variants involving the *BRCA1* RING or C-terminal domains or *BRCA2* DNA-binding domain. HGSC patients with a homologous recombination DNA repair deficiency (HRD) score ≥63 had significantly longer OS compared to those with HRD scores of 42-63 and <42, regardless of *BRCA* status. Mutational signature clustering identified a group of *BRCA*-altered HGSC, characterised by short survival, low HRD-scores, and C1/mesenchymal molecular subtype. *BRCA2*-altered HGSC with *NF1* structural variants were associated with exceptionally good outcomes (median OS 17 years). Patients with *CCNE1* and *AKT2* co-amplification (amp) had a significantly worse OS compared to unaltered HGSC ($p$=0.016, HR 2.50), and OS in those with *CCNE1*amp alone was not inferior to unaltered HGSC patients ($p$=0.698, HR 1.10).

**Conclusion**

Our findings suggest that the adverse effect of residual disease is diminished in g*BRCA*var-carriers compared to non-carriers. While the HRD score strength correlated with survival, the association was imperfect and most poor survival carriers exceeded established HRD classification thresholds. Specific gene alterations such as *NF1* loss and co-amp of *AKT2* and *CCNE1* appear to be associated with differential therapy response and survival.

### EACR2024-0515

### The Role of Nucleotide Excision Repair (NER) in the Mutagenicity of Tobacco and Alcohol

*F. Rajabi[1], Y. Andrey[1], W. Jixin[1], A. Said[2], N. Sergey[1]*

*[1]Institut Gustave Roussy, Molecular Medecine, Villejuif, France*

*[2]Institut Gustave Roussy, Department of Genome Integrity & Cancers, Villejuif, France*

**Introduction**

Xeroderma Pigmentosum (XP) is a genetic disorder caused by a constitutive deficiency of nucleotide excision repair (NER). XP patients are characterized by a 10’000-fold increased risk of skin cancer due to their inability to repair UV-induced DNA lesions. It is shown that XP patients have increased risks of internal cancer. In XP-C patients, we have recently shown an increased intensive damage-induced mutagenesis associated with the purine residues. The etiology of this mutational process is not well understood. However, it may be associated with genotoxins such as acetaldehyde (AC) or formaldehyde (FA), which are produced in the cell and can have endogenous but also exogenous origins such as benzo[a]pyrene. This project aims to identify the mutational consequences of chronic exposures to broadly distributed genotoxic substances.

**Material and Methods**

In vitro: Using CRISPR technology, we generated XPC-KO from the RPE-1 cell line. XPC-KO and NER proficient cell lines are treated with various genotoxins. In vivo: XPC-/- and WT mice were treated with the mentioned alcohol and tobacco-derived genotoxins for two months mutational signatures of hematopoietic stem cells were analyzed.

**Results and Discussions**

© 2024 The Authors. Molecular Oncology is published by John Wiley & Sons Ltd on behalf of Federation of European Biochemical Societies

The WGS results showed five times more mutational burden caused by BPDE in XPC-KO cells than XPC expressing cells. However, mutagenesis of AC and FA in XPC-KO cells showed a non-significant increased mutational rate in XPC-KO cells. Comparative analysis of the mutational profiles from in vitro and invivo experiments may reveal the nature of the mutational process-induced above-listed genotoxins. We further assess the impact of attenuation of NER (predominant pathway repairing DNA lesions that are induced by these genotoxins) on the mutagenesis. This project will provide clearer information concerning the increased risk of developing internal cancers in individuals with NER mutations.

**Conclusion**

The results of the WGS displayed different mutational signatures with internal cancers in XP patients. We conclude that tested endogenous or exogenous chemicals are not underlying the unique mutational signature associated with XP patient-internal cancers. Therefore, we added new drugs to the experiments to investigate the mutagenesis of H2O2 and KBrO3. Moreover, the etiology of the revealed signature in internal tumors of XP patients can be due to the different roles of XPC in DNA repair, which investigation is ongoing.

### EACR2024-0520

### A Coupled transcriptomic analysis of Zebrafish vs. Human gallbladder to identify differentially expressed genes associated with carcinogenesis in pre-existing inflammatory gallbladder lesion

*P. TANWAR[1], R. Kumar[2], H. Goel[3], S. Minocha[4], I. Gupta[5]*

*[1]All India Institute of Medical Sciences- New Delhi-India, Laboratory Oncology- Dr. B.R.A. Institute Rotary Cancer Hospital, New Delhi, India*

*[2]All India Institute Of Medical Science-New Delhi-India, Laboratory Oncology- Dr B. R. A. Institute Rotary Cancer Hospital, New Delhi, India*

*[3]All India Institute of Medical Sciences- New Delhi India, Laboratory Oncology- Dr B.R.A. Institute Rotary Cancer Hospital, New Delhi, India*

*[4]Indian Institute of Technology, Kusuma School of Biological Sciences, New Delhi, India*

*[5]Indian Institute of Technology, Department of Biochemical Engineering and Biotechnology, New Delhi, India*

**Introduction**

The Gallbladder (GB) cancer (GBC) though rare in western world but is commonly seen in northern India. The disease carries very poor prognosis having low survival due to its delayed presentation with limited treatment options. Despite such an aggressive cancer, it still lacks an authentic animal model till date. This study offers comparative transcriptomic analyses of normal GB of human and ZF with human GBC, emphasizing on differential gene expression (DEG) & dysregulated signalling pathways associated with development of GBC with special reference to pre-existing inflammatory lesions

**Material and Methods**

The ZF GB was dissected out from GB-Liver complex and human Inflammatory GB and GBC samples were collected operatively after requisite informed consents and ethical approvals. The RNA was extracted from Zebrafish (ZF):6 GB & human: 24GBC vs. 20 GB was done . After assessing the RNA quality cDNA libraries was done using mRNA fragments, followed by fragmentation, adapter ligation, and PCR amplification. Then RNA libraries were sequenced on Illumina platform followed by bioinformatics analysis done on in-house pipeline. The DEGs were analyzed between ZF vs human GB and human inflammatory GB vs. GBC. The DEGs having possible impact on cancer development between ZF GB and human GBC were identified.

**Results and Discussions**

An in-depth examination of the transcriptome revealed 1172 differentially expressed genes (DEGs) in GBC cases. Of these, 583 were downregulated (including PTX3, ACTG2, PENK, and IL6) and 589 were upregulated (MAGE3A, SPINK4, REG3A, SULT1E1). The parallel analysis of ZF transcriptomics identified 549 upregulated genes (SERPINB8, CORO2B) and 579 downregulated genes (MTO1, DRC7). Comparative investigation of common DEGs between human GBC vs. zebrafish data unveiled common shared patterns, with consistent down regulation of PAH, MYOC, and SCARA5 and upregulation of CHEK2, DHX32, and RBM34. Gene Ontology (GO) analysis highlighted significant enrichment of DEGs in various biological and cellular processes, emphasizing involvement in critical functions such as cell proliferation, cell adhesion, receptor ligand activity, and integrin binding.

**Conclusion**

These findings advances insight on differentially expressed genes associated with carcinogenesis in pre-existing inflammatory gallbladder lesion The study also explores ZF transcriptomics and potentiates further possibility of establishing an authentic ZF model to study GBC with primary goal to achieve personalized therapy in GBC.

### EACR2024-0531

### Molecular determinants to neoadjuvant chemotherapy resistance in four breast cancer molecular subtypes

*H.M. Guevara-Nieto[1], C.A. Orozco-Castano[2], R. Parra-Medina[3], J. Zabaleta[4], L. Lopez-Kleine[5], A.L. Combita[2]*

*[1]Instituto Nacional de Cancerologia, Translational Oncology, Bogotá, Colombia*

*[2]Instituto Nacional de Cancerologia, Translational Oncology, Bogota, Colombia*

*[3]Instituto Nacional de Cancerologia, pathology, Bogota, Colombia*

*[4]Louisiana State University Health Science Center, Inter-disciplinary Oncology, New Orleans, United States*

*[5]Universidad Nacional de Colombia, Statistics, Bogota, Colombia*

**Introduction**

Neoadjuvant chemotherapy (NAC) is essential for primary breast cancer (BC) treatment, but its varying success rates lack full understanding. This study aims to pinpoint transcriptomic changes linked to non-response to NAC, enhancing our grasp of treatment resistance,

© 2024 The Authors. Molecular Oncology is published by John Wiley & Sons Ltd on behalf of Federation of European Biochemical Societies

**Material and Methods**
This study undertook a transcriptomic analysis of 44 paired (pre- and post-NAC) BC samples from non-responders at the Colombian National Cancer Institute. These samples, spanned across four distinct breast cancer subtypes: Luminal A, Luminal B HER2-positive, Luminal B HER2-negative, and Triple-Negative Breast Cancer (TNBC). Differential gene expression analysis, enrichment analysis, and tumor microenvironment (TME) infiltration estimation (deconvolution algorithm) were conducted.
**Results and Discussions**
The transcriptomic analysis revealed distinct gene expression profiles differentiating pre-treatment from post-treatment samples across the various breast cancer subtypes, with the FOS gene emerging as a common denominator in all subtypes. Notably, significant pathways varied among the subtypes, encompassing negative regulation of signal transduction and estrogen biosynthetic process in Luminal A; extracellular matrix organization in Luminal B HER2-positive; myeloid dendritic cell activation in Luminal B HER2-negative; and regulation of B cell activation in TNBC. In terms of immune cell composition, post-treatment samples of Luminal A exhibited higher levels of CD4 memory T cells, regulatory T cells (Tregs), and neutrophils. For Luminal B HER2-positive subtypes, an increase in CD8 T cells and activated CD4 memory T cells was observed post-treatment. In the Luminal B HER2-negative group, elevated levels of CD4 memory resting B cells, M2 macrophages, and neutrophils were identified in post-treatment samples. Furthermore, TNBC samples showed a post-treatment increase in resting mast cells and resting CD4 memory T cells.
**Conclusion**
Our findings indicate that NAC modulate gene expression, as well as cellular interactions within the TME, that could contribute to treatment non-response. Comprehensive detection and evaluation of TME are crucial for predicting NAC efficacy and preventing disease recurrence. These results underscore the importance of investigating diverse patient populations to uncover novel insights and address treatment disparities. This study highlights the need for a deeper understanding of the TME's role in chemotherapy resistance, paving the way for the development of more effective, tailored therapeutic strategies.

### EACR2024-0538
### Investigating a Candidate Oncogene on Chromosome 1q in Breast Cancer

*E. Guzar*[1,2], *G. Turan*[1,2], *G. Korkmaz*[2,3]
[1]*Koç University, Graduate School of Health Sciences, Istanbul, Turkiye*
[2]*KUTTAM, Research Center for Translational Medicine, Istanbul, Turkiye*
[3]*Koç University, School of Medicine, Istanbul, Turkiye*
**Introduction**
Breast cancer, characterized by its molecular heterogeneity, is propelled by an array of genetic aberrations driving its complex pathogenesis. Chromosome 1q alterations play a pivotal role in this context, impacting tumor behavior, clinical outcomes, and therapeutic responses. This study aimed to unveil the functional significance of a candidate oncogene, identified for its concurrent upregulation in gene expression and copy number, in breast cancer. Through this investigation, novel molecular insights were sought to inform the development of targeted therapies.
**Material and Methods**
We performed subtype-specific gene expression analysis on 1q genes in breast cancer across six publicly available RNAseq datasets. These included normal-like, luminal, basal A, and basal B cell lines, revealing consistent significant expression changes (P<0.001) across all subtypes. Copy number alterations were explored using Cancer Cell Line Encyclopedia data. The identified candidate gene was targeted in MDAMB231 cells using CRISPR-Cas knockout, followed by 2D and 3D culture for further analysis. The functional impact was assessed through migration and spheroid assays, along with quantitative evaluation of the candidate oncogene's influence on epithelial-mesenchymal markers using qPCR.
**Results and Discussions**
Significant alterations (P<0.001) were observed across different breast cancer subtypes: 220, 146, and 173 genes in the luminal, basal A, and basal B subtypes, respectively. Further analysis identified 18 genes whose expression consistently increased across all subtypes. 15 genes increased in copy number and gene expression were identified, including C4BPB. Despite its unknown function in breast cancer, C4BPB presents a promising avenue for further investigation. Then, using the CRISPR knock-out system, C4BPB gene expression was significantly reduced by 83% in the MDAMB231 cells. According to migration assay results, C4BPB had a statistically significant decrease in migration. Moreover, silencing C4BPB significantly reduced the spheroid-forming ability. Finally, the absence of C4BPB in 2D conditions showed a mild effect in epithelial marker expression but a significant reduction in 3D conditions.
**Conclusion**
The oncogenic function of C4BPB in breast cancer highlights its significance as a potential biomarker for prognosis and a promising therapeutic target for the development of novel precision therapies. Further research is needed to elucidate the underlying molecular mechanisms and clinical implications of breast cancer.

### EACR2024-0568
### Deciphering Crosstalk: Investigating Candidate Orphan GPCR's Interaction with ERα in Breast Cancer

*E. Kansız*[1,2], *F.B. Berber*[1,2], *G. Korkmaz*[2,3]
[1]*Koç University, Graduate School of Health Sciences, Istanbul, Turkiye*
[2]*KUTTAM, Research Center for Translational Medicine, Istanbul, Turkiye*
[3]*Koç University, School of Medicine, Istanbul, Turkiye*
**Introduction**
Breast cancer is a heterogeneous disease characterized by diverse molecular alterations that influence its development and progression. Among these alterations, Estrogen Receptor alpha (ERα; encoded by the *ESR1* gene) has emerged as important player in breast cancer

© 2024 The Authors. Molecular Oncology is published by John Wiley & Sons Ltd on behalf of Federation of European Biochemical Societies biology, regulating gene expression and cellular processes. Additionally, an orphan GPCR candidate (GPR-X), has attracted attention for its potential role in breast cancer, but its exact role is still unclear. The complex molecular interaction between GPR-X and ERα indicates potential modulation of ERα signaling pathways by GPR-X. Furthermore, expression levels of GPR-X have been associated with different breast cancer subtypes and clinical outcomes. This demonstrates its importance as both a prognostic indicator and a therapeutic target. Understanding the GPR-X - ERα interaction holds promise for developing new therapeutic strategies tailored to breast cancer subtypes, thereby potentially improving patient outcomes and treatment efficacy.

**Material and Methods**

ChIP-Seq analysis using publicly available datasets were conducted to determine the transcription factors binding sites (TFBSs) of ERα. Among them GPR-X was selected as a target gene. sgRNAs targeting *GPR-X* and *ERa* genes were individually cloned in pLentiCRISPR v2. T47D and MCF7 cells were obtained from ATCC and cultured in RPMI and DMEM medium, respectively. After qPCR confirmation of the individual knock-out (KO) in T47D and MCF7 cells (GPR-X-KO and ERα-KO), cell viability, cell proliferation and invasion were investigated between WT and KO cells.

**Results and Discussions**

The ERα ChIP-Seq peaks were identified around the GPR-X gene in MCF7 and T47D cells. Expression of GPR-X was examined across various molecular types of breast cancer cells, revealing a significant increase in luminal types. In both T47D-GPR-X KO and MCF7-GPR-X KO cells, GPR-X gene and protein levels were notably reduced, accompanied by decreased ERα gene expression. Silencing GPR-X hindered colony formation, cell proliferation, and viability in cells cultured in both 2D and 3D environments.

**Conclusion**

The interaction between ERα and GPR-X highlights their significance in breast cancer. Understanding how GPR-X influences ERα signaling offers new avenues for therapeutic development, potentially improving outcomes for patients with ERα-positive breast cancer. Further research into this interaction is crucial for advancing our understanding of breast cancer biology and developing targeted treatments.

### EACR2024-0571
### Role of SWI/SNF chromatin-remodeling complexes in tumorigenesis : insights in dedifferentiated melanoma cells

A. Lallement[1], B. Vokshi[2], G. Davidson[1], A. Haller[1], I. Davidson[1]
[1]Institut de génétique et de biologie moléculaire et cellulaire, Génomique Fonctionnelle et Cancer, Illkirch, France
[2]John Hopkins, Medical International, Baltimore, USA

**Introduction**

Melanoma is responsible for the majority of skin cancer deaths. Although immunotherapy and targeted therapies provide clinical benefit, acquired resistance limits their efficacy. Cell heterogeneity is a key feature of melanoma tumours that contributes to resistance. Understanding the mechanisms involved in switching between cell states is therefore key to improving therapy. The melanocyte lineage identity factor Microphthalmia-associated transcription factor (MITF) regulates gene expression in more differentiated melanocytic-type melanoma cells using the SWI/SNF chromatin-remodeling complex as a cofactor. However, the role of SWI/SNF in undifferentiated/mesenchymal melanoma, involved in therapy resistance, has not been characterized.

**Material and Methods**

The SWI/SNF catalytic subunit BRG1 was silenced in 3 mesenchymal melanoma cell cultures in both 2D monolayer and 3D melanosphere conditions and the effects on proliferation and invasion were characterized. Genomic binding of BRG1 was profiled in mesenchymal cells and compared with melanocytic type cells. Interaction of SWI/SNF with the key AP1 and PRRX1 mesenchymal cell transcription factors and their genomic occupancy was analyzed.

**Results and Discussions**

BRG1 silencing regulated motility and invasion, but had minimal effects on proliferation of mesenchymal cells cultured in 2D conditions. In contrast, BRG1 was required for the formation of 3D melanospheres that involved transition towards a more neural-crest stem cell-like (NC) state. This NC/MES intermediary state, highly expressing PRXX1 and other key EMT transcription factors, more closely mimics the characteristics of metastatic melanoma than fully mesenchymal cells. BRG1 binds a set of mesenchymal-specific enhancers together with PRRX1. BRG1 and PRRX1 bound sites overlap with those of AP1 and we show that PRRX1 interacts with both AP1 and SWI/SNF.

**Conclusion**

Our data show that BRG1 binds to mesenchymal-specific enhancers and is required for transition from the MES to a MES/NC intermediary state that involves up-regulated expression of PRRX1 and its target genes. PRRX1 occupies and activates a subset of MES enhancers via interactions with AP1 and SWI/SNF leading to regulation of genes involved in melanoma metastasis. Our results highlight the role of AP1 as platform to integrate other transcription factors such as TEAD4, PRRX1, recruiting them to the genome and activating mesenchymal enhancers.

### EACR2024-0587
### Exploring molecular changes in Gastric Cancer for prospective targeted therapy: Perspectives from an Indian Cohort

P. Shenoy[1], A. Sakpal[1], M. Bal[2], A. Ramaswamy[3], V. Ostwal[4], S. Shrikhande[5], S. Gupta[4], P. Ray[6]
[1]Advanced Centre For Treatment Research and Education in Cancer- Homi Bhabha National Institute, Imaging Cell Signalling and Therapeutics Lab, Kharghar, India
[2]Tata Memorial Hospital- Homi Bhabha National Institute, Department of Pathology, Mumbai, India
[3]Tata Memorial Hospital- Homi Bhabha National Institute, Medical Oncology, Parel, India
[4]Tata Memorial Hospital- Homi Bhabha National

© 2024 The Authors. Molecular Oncology is published by John Wiley & Sons Ltd on behalf of Federation of European Biochemical Societies

Institute, Medical Oncology, Mumbai, India
[5]Tata Memorial Hospital- Homi Bhabha National Institute, Surgical Oncology, Mumbai, India
[6]Advanced Centre for Treatment- Research and Education in Cancer- Homi Bhabha National Institute, Imaging Cell Signalling and Therapeutics Lab, Kharghar, India

**Introduction**

Despite being a class of molecularly heterogeneous tumors, Gastric cancer (GC) treatment comprises cytotoxic chemotherapy (Epirubicin, Oxaliplatin, 5-FU), which eventually leads to relapse and resistance. HER2 targeted (Trastuzumab) & VEGFR2 targeted (Ramucirumab) are the only two approved targeted therapeutics & are given either to metastatic or resistant patients (second line therapy), respectively. Overall, the subset of patients that can benefit from HER2/VEGFR2 targeted therapy and the response rate to these drugs is relatively poor (10-40%). TCGA (2014) and ACRG (2015) suggest that 50% gastric tumors have p53 mutations. However, the association of mutant p53 with potentially targetable mutations is poorly understood. Herein, we aim to understand the genetic landscape of a small cohort of Indian GC patients to identify targetable mutations and their association with mutant p53.

**Material and Methods**

IHC & NGS were performed in FFPE patient-derived tumor blocks. Using site-directed mutagenesis, we created wtp53 & patient-derived p53 mutation (R175H/R273H) overexpressing cells in KATO-III$^{p53-/-}$ cell line. Mutants were assessed for their drug sensitivity, & relevant target gene expression.

**Results and Discussions**

TCGA (2014) data analysis showed alterations in TP53 (50%), ARID1A (33%), PIK3CA (24%), ERBB2 (17%), PTEN (11%) & DNA Damage Response (DDR) genes (10-15%). Using locally advanced GC samples (n=13), clinically significant pathogenic mutations were identified in DDR genes (BRCA1, BRCA2, CHEK2, RAD51, ATM), KRAS, PIK3CA, & PTEN. Additionally, 56% of patients showed >70% expression of p53 (indicative of mutp53), as observed by IHC (n=25) and was verified by NGS. Three p53 DNA binding domain hotspot mutations were identified (R175H, R248W & R273H), of which, R273H was most frequently observed (~23%). Interestingly, 12% of patients showed HER2 (Score 3+) by IHC & all harbored mutant p53 (L344N/S367N). Further, patient-derived mut-R273H when overexpressed in KATO-III cells was significantly sensitive to Oxaliplatin ($IC_{50}$ 4 μM) as compared to wtp53 ($IC_{50}$ 28 μM) & showed upregulated PIK3CA signaling. Investigations are currently ongoing to understand the potential of a few clinically approved therapeutics in the background of mutant p53 & their role in the pathogenesis of GC.

**Conclusion**

Exploring the genetic landscape & the influence of predominant p53 mutations in Indian GC patients may lay the foundation to stratify patients for targeted therapy.

### EACR2024-0598
### The Genomic Landscape Alterations in Primary Tumor and Matched Mediastinal Lymph Node in Non-Small Cell Lung Cancer

*C. Silvério Faria[1], C. Machado Baldavira[2], T. Gutierrez Prieto[2], E. Caroline Toledo do Nascimento[3], E. Sobroza de Mello[4], F. Regina Rotea Mangone[5], M. Aparecida Nagai[5], R. Mingarini Terra[6], V. Luiza Capelozzi[2], L. Antonangelo[1]*
[1]*Faculty of Medicine of University of Sao Paulo, Medical Research Laboratory 03- Department of Pathology, Sao Paulo, Brazil*
[2]*Faculty of Medicine of University of Sao Paulo, Pathology, Sao Paulo, Brazil*
[3]*Instituto do Coração Incor- University of Sao Paulo, Pathological Anatomy Laboratory, Sao Paulo, Brazil*
[4]*Instituto do Cancer do Estado de São Paulo ICESP, Pathology Laboratory, Sao Paulo, Brazil*
[5]*Instituto do Cancer do Estado de São Paulo ICESP, Center for Oncology Translational Research, Sao Paulo, Brazil*
[6]*Instituto do Cancer do Estado de São Paulo ICESP, Oncological Thoracic Surgery Service, Sao Paulo, Brazil*

**Introduction**

Histological analysis and tumor staging, guided by the Tumor, Node, Metastasis (TNM) classification, are currently the pillars in guiding treatment decisions for patients with non-small cell lung cancer (NSCLC). Echo-bronchoscopy-guided transbronchial needle aspiration (EBUS-TBNA) has proven effective in obtaining biopsies from hilar and mediastinal lymph nodes (MLN), making it the preferred initial method for tissue collection for cancer staging. Assessing genomic alterations in the surgically removed primary tumor (PT) and corresponding MLN can offer valuable insights into patients' recurrence risk, detect hidden metastases, and identify candidates for adjuvant therapies. We aimed to analyze somatic variants of significant clinical relevance in genomic DNA (gDNA) extracted from formalin-fixed paraffin-embedded PT tissues and MLN aspirates using Next-Generation Sequencing (NGS).

**Material and Methods**

gDNA was extracted from paired samples from 32 patients with lung adenocarcinoma, resulting in 64 samples. These patients were divided into two groups: A) 21 cases with histologically negative MLN for metastasis; and B) 11 cases with MLN positive for metastasis. The samples were sequenced using a custom genetic panel, the SureSelect XTHS2 kit (Agilent Technologies, Santa Clara, CA, USA).

**Results and Discussions**

In group A, variants were detected in 62% of the samples. In contrast, 95.4% of the samples in group B harbored variants of strong and potential clinical significance, as defined by the Association for Molecular Pathology (AMP). These variants were identified in several genes, including EGFR, TP53, KRAS, BRAF, ATM, PTEN, PIK3CA, CHEK2, RET, SMARCA4, SKT11, NRAS, KIT, and APC. Notably, while Group A samples did not show identical variants between PT and MLN pairs, 60% of cases in Group B exhibited the same variants in both the PT and MLN samples. This suggests that the identified variants may not directly correlate with recurrence or metastasis but may indicate the presence of different subclones or mutations in genes associated with

© 2024 The Authors. Molecular Oncology is published by John Wiley & Sons Ltd on behalf of Federation of European Biochemical Societies metastatic adenocarcinoma, potentially affecting patient outcomes.

**Conclusion**

In summary, we discovered distinct somatic variants between PT and MLN samples, especially in Group A, while Group B showed a more uniform genomic profile, with variants likely contributing to disease progression. Thus, incorporating molecular testing with cytological analysis of MLN could enhance the precision of the TNM staging system, offering a more comprehensive approach to patient management.

### EACR2024-0637
### Mutations of cis-regulatory elements in classic Hodgkin lymphoma

*A. Ustaszewski*[1], *G. Sawicz*[1], *M. Kostrzewska-Poczekaj*[1], *M. Rydzanicz*[2], *N. Russiñol*[3], *V. Chapaprieta*[4], *K. Dzieńdziura*[1], *J.I. Martín-Subero*[4], *R. Siebert*[5], *M. Giefing*[1]

[1]*Institute of Human Genetics, Polish Academy of Sciences, Department of Cancer Genetics, Poznan, Poland*

[2]*Medical University of Warsaw, Department of Medical Genetics, Warsaw, Poland*

[3]*Institut d'Investigacions Biomèdiques August Pi I Sunyer - IDIBAPS, Molecular pathology of lymphoid neoplasms, Barcelona, Spain*

[4]*Institut d'Investigacions Biomèdiques August Pi I Sunyer - IDIBAPS, Biomedical epigenomics, Barcelona, Spain*

[5]*Ulm University and Ulm University Medical Center, Institute of Human Genetics, Ulm, Germany*

**Introduction**

Despite improvement in understanding classic Hodgkin lymphoma (cHL) biology, there are no reports on the significance of variants in gene regulatory regions in this lymphoma. To close this gap we performed global sequencing of regulatory elements in cHL cell lines to investigate their association with chromatin state and potential impact on gene expression.

**Material and Methods**

Integrated experiments on cHL cell lines and Germinal Center B (GCB) cell controls:

- targeted NGS of the regulome: regions located up to 10 kbp from transcription starting sites of protein genes overlapping ENCODE cis-regulatory elements (CRE's),
- ATAC-seq and ChIP-seq,
- gene expression: Affymetrix U95 profiles and The Human Protein Atlas data.

Standard filtering of NGS data (DP > 5, QUAL > 30) was used to select variants returned by Freebayes, DeepVariant and HaplotypeCaller. We selected regulatory regions that (I) harboured variants in ≥ 2/7 cHL cell lines, (II) had closed chromatin in cHL cell lines with the variant and (III) open chromatin in GCB controls. Such variants were validated by downstream sequencing.

**Results and Discussions**

We identified 25 regions (CRE's) upstream of protein coding genes which contained 65 variants. Among these, 12 regions were associated with genes deregulated in cHL ($p < 0.05$) as compared to GCB controls. Using JASPAR we analysed if these variants alter binding motifs of transcription factors expressed in cHL. We hypothesised that this is a mechanism underlying altered chromatin accessibility and gene expression. This analysis indicated, among others, two candidates:

- *ST6GAL1* (involved in generation of cell-surface antigens CD75 and CD76) found 6.3 logFC downregulated and with closed chromatin in cHL; identified variants chr3:187022345 (T/A) and chr3:187022340 (T/C) (GRCh38) (SUP-HD1) abolish the *FOXI1* binding motif (TT[T/C]TCCC[T/A]ACACA);
- *CCR7* (C-C motif chemokine receptor 7, activates B and T lymphocytes) found 4.6 logFC upregulated and with open chromatin in cHL; identified variant chr17:40564491 (G/A) in L-1236; introduces the *ATF4* binding motif (TGCTGGTGA[G/A]ACAA).

Functional validation by luciferase reporter assays is being performed.

**Conclusion**

We provide evidence of mutations recurrently targeting non-coding regulatory sequences of genes deregulated in cHL. This is exemplified by variants in *ST6GAL1* and *CCR7* CRE's that alter TFB motifs and are a potential mechanism behind altered chromatin state and expression of these genes.

### EACR2024-0645
### A novel technology to extract RNA from FFPE tissues enhancing compatibility with transcriptome analysis

*N. Bahlmann*[1], *T. Schughart*[2], *I. Rose*[3], *T. Hess*[2], *L. Torres-Benito*[4]

[1]*BioEcho Life Sciences GmbH, Sales, Cologne, Germany*

[2]*BioEcho Life Sciences GmbH, Research and Development, Cologne, Germany*

[3]*Reference Medicine- Inc., Managing, Phoenix, USA*

[4]*BioEcho Life Science GmbH, Marketing, Cologne, Germany*

**Introduction**

In oncology, the possibility of capturing the whole tumor transcriptome from formalin-fixed paraffin-embedded (FFPE) tissues has improved diagnosis and facilitated more precise drug selection for personalized treatment strategies. However, the extraction of high-quality RNA from FFPE tissues poses significant challenges due to the chemical modifications and degradation caused by formalin fixation. In addition, conventional methods have been plagued by lengthy, labor-intensive protocols and the utilization of hazardous reagents. We developed a unique technology to extract RNA from FFPE tissues, which offers various benefits: notably a quicker protocol compared to alternative methods, and no need for xylene or other organic solvents, such as ethanol, minimizing the use of hazardous reagents and mitigating safety risks for operators. Moreover, this technology shows high efficiency and enhances compatibility with RNA-Seq.

**Material and Methods**

We validated the performance of our method across a diverse selection of standardized human FFPE samples, assessing RNA quantity and quality in comparison to two

© 2024 The Authors. Molecular Oncology is published by John Wiley & Sons Ltd on behalf of Federation of European Biochemical Societies silica-based extraction kits. To evaluate the compatibility with downstream applications and systematic differences with other common extraction procedures, we selected specific tumor-normal sample pairs for whole transcriptome RNA sequencing.

**Results and Discussions**

The processing time needed for the RNA extraction with our method is substantially shorter as compared to conventional extraction methods. Results demonstrated that our novel protocol consistently yielded high RNA quantities and integrity, surpassing the performance benchmarks set by the silica-based methods. The whole transcriptome RNA sequencing data suggested that rRNA depletion was more efficient for FFPE RNA samples extracted with our method. Conclusively, RNA sequencing analysis performed better regarding basic specifications, such as the number of unique mapping reads and detected genes. Those results also had beneficial effects on downstream differential expression analyses.

**Conclusion**

In conclusion, we establish that our unique nucleic acid extraction technology improves RNA-seq results and facilitates the processing of FFPE samples, hence enabling new avenues for molecular pathology and personalized medicine research.

### EACR2024-0649

### Case-only GWAS study highlights potential germline variants associated with prostate cancer progression

*Z. Kolodynska*[1], *B. Aboulmaouahib*[2], *Q. Deng*[3], *C. Geisenberger*[4], *F. Klauschen*[4], *I. Asangani*[3], *M. Müller-Nurasyid*[5], *A. Buchner*[6], *K. Strauch*[7], *T. Grünewald*[1]

[1]*Deutsches Krebsforschungszentrum- DKFZ, Division of Translational Pediatric Sarcoma Research B410- German Cancer Research Center DKFZ, Heidelberg, Germany*

[2]*Institute for Medical Biometry- Epidemiology and Informatics- University Medicine Mainz, Department of Genomic Statistics and Bioinformatics, Mainz, Germany*

[3]*Perelman School of Medicine- University of Pennsylvania- Philadelphia- US, Department of Cancer Biology, Philadelphia, United States*

[4]*Institute of Pathology- LMU Munich- Munich- Germany, Department of Pathology, Munich, Germany*

[5]*Institute for Medical Biometry- Epidemiology and Informatics- University Medicine Mainz- Mainz, Department of Statistical Genomics and Bioinformatics, Mainz, Germany*

[6]*University Hospital- LMU Munich- Munich- Germany, Department of Urology, Munich, Germany*

[7]*Institute for Medical Biometry- Epidemiology and Informatics- University Medicine Mainz- Mainz- Germany, Department of Statistical Genomics and Bioinformatics, Mainz, Germany*

**Introduction**

Accumulating evidence suggests that the incidence of prostate adenocarcinoma (PCa) depends on the germline genetic context across human populations. Whether germline variants contribute to PCa progression and outcome, however, is thus far has been largely unexplored.

**Material and Methods**

To investigate the potential association of germline variants and associated gene to outcome parameters in PCa, we collected peripheral blood from >2000 European PCa patients that were uniformly treated in the same high-volume center. Extracted DNA was analyzed with the Illumina Global Screening Array (GSA) covering SNPs that were enriched in clinically relevant genes. In addition, all relevant clinicopathological data, such as metastasis, Gleason-score and PSA-levels were collected. After stringent data quality controls, genetic outlier detection, and imputation of missing SNPs (Michigan imputation server) a comprehensive case-only genome-wide association study (GWAS) was carried out (Info score >0.3). Results were displayed on Manhattan plots and top hits manually inspected with LocusZoom.

**Results and Discussions**

By stratifying the cohort by pathological lymph node status as a surrogate marker for aggressive disease, the GWAS identified several significant loci, including one with genome-wide significance, and highlighted various candidate genes that may account for the phenotype. These genes are currently under investigation for their functional relevance for PCa aggressiveness in experimental assays using cell line models. Furthermore, transcriptome profiling of tumor-derived RNA from a subset of the GWAS-cohort is currently being carried out to enable expression quantitative trait-locus (eQTL) analyses.

**Conclusion**

Our preliminary results indicate that several germline loci and candidate genes may contribute to aggressiveness of PCa, which may have implications for future risk-stratification of patients and assignment to an optimized treatment regimen.

### EACR2024-0668

### An RNAseq risk classifier to differentiate low- from high-risk Ducal Carcinoma In Situ of the breast

*E. Lips*[1], *M. Roman-Escorza*[2], *T. Bismeijer*[3], *M. Sheinman*[3], *W. Harley*[1], *V. Shah*[2], *L. Wessels*[3], *J. Wesseling*[1], *E. Sawyer*[2], *C.G.C. PRECISION Consortium*[1]

[1]*The Netherlands Cancer Institute, Molecular Pathology, Amsterdam, The Netherlands*

[2]*King's College London, School of Cancer and Pharmaceutical Sciences- Faculty of Life Sciences and Medicine- Guy's Cancer Centre, London, UK*

[3]*The Netherlands Cancer Institute, Molecular Carcino-genesis, Amsterdam, The Netherlands*

**Introduction**

Ductal carcinoma in situ (DCIS) is considered a non-obligate precursor of invasive ductal carcinoma. With the aim of preventing a subsequent invasive cancer, all DCIS lesions are currently treated with surgical excision often supplemented with radiotherapy (RT). To prevent DCIS over- or under treatment, a reliable marker of DCIS progression risk is urgently needed.

**Material and Methods**

© 2024 The Authors. Molecular Oncology is published by John Wiley & Sons Ltd on behalf of Federation of European Biochemical Societies

We studied two large DCIS cohorts: a Dutch population-based cohort (median follow-up 13 years), and the Sloane cohort, a prospective breast screening cohort from the UK (median follow-up 12.5 years). FFPE tissue specimens from patients with pure primary DCIS after breast-conserving surgery that did develop a subsequent ipsilateral event (DCIS or invasive) were considered as cases, whereas patients that did not develop a recurrence were considered as controls. We performed RNAseq analysis on 229 cases (149 invasive and 80 in situ recurrences) and 344 controls, and build a gene expression classifier using a penalized Cox model.
**Results and Discussions**
When we compared gene expression between cases and controls, we did not identify any genes significantly associated to recurrence. However, by limiting the analysis to samples that had not had RT and focusing only on invasive recurrences, we developed a penalized Cox model from RNAseq data. The model was trained on weighted samples (to correct for the biased sampling of the case control cohorts) from the Dutch series with double loop cross validation. The genes were selected using the Elastic net framework of penalization. Using this predicted hazard ratio, the samples were split into high, medium and low risk quantiles, with a recurrence risk of 20%, 9% and 2.5%, respectively at 5 years (p = 10-10, Wald test). The Dutch-cohort-trained predictor was independently validated in the Sloane cohort (p = 0.02, Wald test). Our RNAseq predictor was more predictive of recurrence than PAM50, clinical features (Grade, Her2 and ER) and the 12-gene Solin DCIS score ($p < 0.001$, permutation test using the Wald statistic) in both the Dutch cohort and Sloane cohort.
**Conclusion**
Using large unbiased samples series, we successfully build and validated an RNAseq classifier to predict invasive breast cancer risk after DCIS. This RNAseq predictor could change clinical practice by allowing clinicians to identify a subgroup of low-risk DCIS suitable for treatment de-escalation and studies of active surveillance.

### EACR2024-0671
### Whole genome sequencing population studies

*<u>Z. Rozankova</u>[1], P. Zizkovicova[1], M. Skopova[1], B. Liznerova[1,2], H. Jurtikova[1,2], J. Drabek[1,2], M. Hajduch[1,2,3]*
*[1]Faculty of Medicine and Dentistry- Palacky University Olomouc, Institute of Molecular and Translational Medicine, Olomouc, Czech Republic*
*[2]University Hospital, Laboratory of Experimental Medicine, Olomouc, Czech Republic*
*[3]Palacky University Olomouc, Czech Advanced Technology and Research Institute, Institute of Molecular and Translational Medicine, Olomouc, Czech Republic*

**Introduction**
Massively parallel sequencing (MPS) delivers fast, cost-effective and high-throughput sequencing of DNA and RNA molecules, enabling faster progress in genomic research, diagnostics, personalized medicine and other areas of biotechnology. Whole genome sequencing (WGS), as one of the MPS options, provides a comprehensive view of an organism's entire genome, including coding and non-coding regions, enabling the detection of different types of genetic variants, including single nucleotide polymorphisms (SNPs), insertions, deletions, copy number variations (CNVs) and structural rearrangements. The aim of these studies was to map genetic variants in local populations and to create a genome-wide database of healthy the Czech population allowing comparison with other populations.
**Material and Methods**
We enrolled in total 1159 Czech healthy blood donors' gDNA samples into those studies. TruSeq DNA PCR Free Library input amount was 1 µg and all samples were mechanically fragmented before library preparation. Sequencing libraries were prepared using TruSeq DNA UD Indexes v0. After quality control of all libraries, sequencing at the Illumina NovaSeq 6000 was performed. Bioinformatic analysis was performed using an in-house pipeline and additional quality parameters control steps by FastQC, Qualimap bamqc, SAMtools stats, and MultiQC.
**Results and Discussions**
In order to create a genome-wide database that would capture the full spectrum of human genetic variation, we sequenced the genomes of participating individuals at high resolution using next-generation sequencing (NGS) technologies. There was an emphasis on data sharing and making our sequencing data freely available through public databases and repositories. The project has produced reference panels of population genetic variants that serve as valuable resources for genome-wide association studies (GWAS) and other genetic analyses, such as haplotype structures and linkage disequilibrium patterns within and between populations. Data generated by WGS can be used in population genetics studies, disease association studies, pharmacogenomic research, and other areas of genomic research.
**Conclusion**
WGS technology facilitates population studies to understand genetic diversity, population structure, migration patterns and evolutionary relationships between different species or human populations, particularly with respect to genetic adaptations, demographic history and the genetic basis of complex traits and diseases.

### EACR2024-0672
### Targeting upregulated Phosphodiesterase 4D to suppress GNAS-mutated colorectal cancers

*<u>S. Zafar</u>[1], P. Nummela[1], E. Veikkolainen[1], A. Ayo[2], M.Y. Asghar[3], K. Törnquist[4], P. Laakkonen[2], T. Salo[5], L. Aaltonen[1], A. Ristimäki[1]*
*[1]University of Helsinki, Applied Tumor Genomics Research Program- Research Programs Unit-, Helsinki, Finland*
*[2]University of Helsinki, Translational Cancer Medicine Research Program- Research Programs Unit-, Helsinki, Finland*
*[3]University of Helsinki, Cell and Tissue Dynamics Research Program- Institute of Biotechnology- HiLife-, Helsinki, Finland*
*[4]Minerva Foundation Institute, Minerva Foundation*

© 2024 The Authors. Molecular Oncology is published by John Wiley & Sons Ltd on behalf of Federation of European Biochemical Societies

*Institute for Medical Research, Helsinki, Finland*
*[5]University of Helsinki, Department of Oral and Maxillofacial Diseases, Helsinki, Finland*

**Introduction**

Colorectal cancer (CRC) ranks as the third most common malignancy worldwide and is the second leading cause of cancer-related deaths. Mutations (mt) in the *GNAS* codon 201 have been identified as activators of downstream adenylate cyclase, initiating constitutive cAMP signaling. GNASmt has been linked to colorectal carcinogenesis, particularly in mucinous carcinomas. In intestinal mucinous carcinomas, over 60% of cases exhibit activating GNASmt.

**Material and Methods**

This study aimed to explore the mechanism of action of GNASmt in carcinogenesis, which remains poorly understood. We utilized CRC cell lines with CRISPR/Cas9-induced activating GNASmt, followed by RNA sequencing and multiple functional in vitro and in vivo assays.

**Results and Discussions**

The study revealed that the cAMP hydrolyzing phosphodiesterase 4D (PDE4D) was notably upregulated in GNASmt cells. Knocking down *GNAS* expression using siRNAs not only suppressed this upregulation but also led to a decrease in cAMP levels. GNASmt cells displayed increased migration and invasion compared to parental cells. Intriguingly, inhibiting PDE4D activity using either a pan-PDE4 inhibitor or a PDE4D selective inhibitor elevated cAMP levels and significantly reduced the oncogenic properties of GNASmt cells, including proliferation, migration, and invasion, with no effect on parental cells. Similarly, in vivo studies demonstrated that the use of a pan-PDE4 inhibitor exhibited a significant antitumor effect on GNASmt tumors. These findings underscore the potential role of PDE4D in mediating the effects of GNASmt on cancer progression.

**Conclusion**

In conclusion, our study elucidates that oncogenic GNASmt induces constitutive cAMP elevation, resulting in increased *PDE4D* expression. Inhibition of PDE4D activity effectively suppresses the oncogenic properties of GNASmt cells. These findings suggest that targeting PDE4D could serve as a promising therapeutic strategy for controlling GNAS-mutated tumors.

### EACR2024-0682

### In situ detection and subcellular localization of 5,000 genes using Xenium Analyzer in cancer tissue samples

*M. Rouault[1], H. Sasaki[1], J. Sicherman[1], V. Kuman[1], F. Meshi[1], P. Marks[1]*
*[1]10x Genomics, Research and Development, Pleasanton, United States*

**Introduction**

Spatial transcriptomics has emerged as a powerful tool that allows researchers to explore the spatial organization of cell types, cell-cell interactions, and cell states by quantifying and localizing gene expression within intact tissues and maintaining the morphological context. The Xenium Analyzer provides an end-to-end solution to perform spatial analysis with highly sensitive and specific detection of RNA with fully automated multiplexed decoding and computational analysis that occurs on the instrument.

**Material and Methods**

Here, we show how the RNA multiplexing capabilities of Xenium can detect up to 5,000 genes simultaneously, providing comprehensive in situ gene expression analysis for any tissue, healthy and diseased. We developed a 5,000-plex gene panel that allows for in-depth pan-tissue cell typing in all major tissues, analysis of cell signaling pathways and identification of genes that are known to have aberrant expression in cancer and that are relevant to understanding the tumor microenvironment.

**Results and Discussions**

We combined 5,000 gene plex with advances in cell segmentation, using a cell membrane-based approach to define the cell boundaries. Using multiple human healthy and diseased formalin fixed & paraffin embedded (FFPE) tissues (kidney, liver, skin, pancreas, colon, lung, and brain), we show how the detection of 5,000 targets and the new segmentation capabilities improve the accuracy of cell typing, with a wider range of cell types being identified with confidence. We also demonstrate that we can identify and characterize unique biological micro-environments across a broad range of healthy and diseased tissue types.

**Conclusion**

The advances that we describe here highlight the flexibility of the Xenium platform, with the ability to use smaller plex panels for targeted, defined studies, and higher plex panels for discovery research. This flexibility provides researchers with multiple options for generating high-resolution spatial gene expression data that are key to furthering our understanding of cancer biology.

### EACR2024-0722

### POSTER IN THE SPOTLIGHT

### Structural Variation signatures reveal novel stratification of ovarian cancer patients

*G. Micoli[1], K. Lavikka[1], Y. Li[1], T. Muranen[1], G. Marchi[1], A. Virtanen[2], J. Hynninen[3], J. Oikkonen[1], S. Hautaniemi[1]*
*[1]University of Helsinki, Research Program in Systems Oncology- Faculty of Medicine, Helsinki, Finland*
*[2]HUS Diagnostic Center, Department of Pathology, Helsinki, Finland*
*[3]Turku University Hospital, Department of Obstetrics and Gynecology, Turku, Finland*

**Introduction**

High-grade serous carcinoma (HGSC) is characterized by pronounced chromosomal instability and genomic rearrangements rather than mutations. Despite the effective use of signatures for mutations, there is no consensus for copy number variation (CNV) and structural variation (SV) signatures. Our research aims to uncover the underlying processes that drive HGSC heterogeneity, by leveraging genomic signatures from SVs and CNVs. Stratification of patients and association of signatures with specific biological processes is fundamental for deepening our comprehension of HGSC's molecular basis.

**Material and Methods**

Molecular Oncology 18 (Suppl. 1) (2024) 1–495
© 2024 The Authors. Molecular Oncology is published by John Wiley & Sons Ltd on behalf of Federation of European Biochemical Societies

We conducted whole-genome sequencing (WGS) on 640 multiregional samples from HGSC patients in the DECIDER cohort, following informed consent. Our analysis encompassed calling CNVs and SVs, calculating novel SV and CNV derived features, extracting signatures, and statistically linking these signatures to biological processes and features.

**Results and Discussions**

Our analysis revealed 11 distinct and robust genomic signatures. Three signatures are linked to a favorable prognosis and differentiate between BRCA1 and BRCA2 mutations in homologous recombination deficiency (HRD), a key distinction given that half of HGSC patients show HRD and PARP inhibitors are more effective in these cases. Including SVs in our analysis underscores their significant role in identifying these distinctions. Another signature features the presence of tandem duplications and pyrgos (together with tyfonas, constitute novel classes of complex SVs) and is associated with CDK12 mutations, which has a critical role in pathways involved in DNA repair. Furthermore, three signatures connected to whole-genome duplication (WGD) present a notable contrast: while one exhibits a highly segmented profile, the other two, despite their simpler genomes, reveal breakage-fusion-bridges and tyfona patterns, suggesting distinct mechanisms.

**Conclusion**

This comprehensive analysis of genomic signatures in HGSC deepens our understanding of genomic instability. Indeed, by associating nine identified signatures with specific biological processes, we offer valuable insights into their genomic contexts, significantly advancing our understanding of HGSC. In general, mutational signatures have gained a critical role also in clinics but capture only part of variation in the genome. Our work underscores the importance of incorporating SVs in addition to CNVs to achieve robust signatures and offer a more complete picture of genomic alterations.

### EACR2024-0740

### An integrative approach to identify p73-regulated biomarkers in colorectal cancer and to unravel their mechanism in cancer progression

*C. BAREJA[1], K. Dwivedi[2], A. Uboveja[3], A. Mathur[4], D. Saluja[1]*
*[1]University of Delhi, DR. B.R. Ambedkar Center for Biomedical Research, Delhi, India*
*[2]University of Delhi, Department of Computer sciences-, Delhi, India*
*[3]University of Pittsburgh, Department of Pharmacology and Chemical Biology, Pittsburgh, United States*
*[4]University of Delhi, Delhi School of Public Health, Delhi, India*

**Introduction**

This work aims to decipher p73-regulated biomarkers for a prompt diagnosis of colorectal cancer (CRC) by employing a combination of integrative bioinformatics and expression profiling technologies.

**Material and Methods**

Transcriptome profile of HCT116 cell line p53$^{-/-}$ p73$^{+/+}$ and p53$^{-/-}$ p73 knockdown was performed to identify differentially expressed genes (DEGs) and long non-coding RNAs followed by cross-checking with CRC tissue expression datasets available in GEO and TCGA. KEGG and Gene ontology were performed on differentially expressed transcripts obtained via the transcriptome profile and intersected genes. The PPI network was constructed via Cytoscape to extract hub genes. Kaplan-Meier plots assisted in investigating the prognostic significance of the hub genes. The clinicopathological relevance was explored using GEPIA and UALCAN databases. qPCR was carried out for expression. Promoter analysis was employed for the identification of p73 binding sites in the selected upregulated or downregulated lncRNAs which was further confirmed by Luciferase reporter, and ChIP assay. Machine learning algorithms were employed to perform TNM-stage classification. We utilized the Stem Checker database to investigate any possible effect of p73 deletion on the stemness in cells displaying mesenchymal features.

**Results and Discussions**

ChIP showed promoter enrichment of the selected lncRNAs. Transcriptome profiling revealed 1,289 upregulated and 1,897 downregulated genes. When intersected with employed CRC datasets, 284 DEGs were obtained. The analysis of gene ontology and KEGG showed enrichment of the DEGs in metabolic process, fatty acid biosynthesis, etc. The PPI network constructed using these 284 genes assisted in identifying 20 hub genes. Kaplan-Meier, GEPIA, and UALCAN analyses uncovered the prognostic and diagnostic relevance of these hub genes. Conclusively, the deep learning model achieved TNM-stage classification accuracy of 0.78 and 0.75 using 284 DEGs and 20 hub genes, respectively. This is a novel study utilizing transcriptomics, publicly available tissue datasets, and machine learning to unveil key CRC-relevant genes. These genes are found relevant regarding the patients' prognosis and diagnosis. We observed embryonic and hematopoietic stem cell-related genes to be the most overlapped genes with our dataset.

**Conclusion**

This is a novel study utilizing transcriptomics, publicly available tissue datasets, and machine learning to unveil key CRC-relevant genes that may be important for patients' prognosis and diagnosis.

### EACR2024-0786

### Local mutation rate variations at Transcription Factor Binding Sites in liver cancer

*N. George[1], A.K. Singh[1], S. Radhakrishnan[1]*
*[1]National Centre for Biological Sciences NCBS- TIFR, Biophysics- Biochemistry and Bioinformatics, Bangalore, India*

**Introduction**

Distribution of somatic mutations across the cancer genome is highly heterogeneous, due to differential DNA damage and repair activity. For example, the DNA regions bound by transcription factors (TF) and histones have elevated somatic mutation rates in melanoma and lung cancers, likely due to the inaccessibility of Nucleotide Excision Repair (NER) to these protein-bound regions. However, the extent of this in other

© 2024 The Authors. Molecular Oncology is published by John Wiley & Sons Ltd on behalf of Federation of European Biochemical Societies tumour types is not fully understood. In particular, how multiple TFs co-binding influences the repair efficiency and whether that influences local mutation rate variations. In this study, we investigate this using more than 200 TFs ChIPseq data from HepG2 liver cancer cell line and study the mutation rate variations in liver cancers with respect to TFs co-binding.

**Material and Methods**

The ChIPseq data of 208 TF in HepG2 line was obtained from ENCODE. The somatic mutation data from whole-genome sequencing of liver cancers was obtained from PCAWG (n=314) and ICGC (n=112). The somatic mutations were mapped to the TF binding sites (TFBS) and the enrichment over the expected mutation rate (Foldchange - FC and P-value) was calculated. Further, the peak shape analysis was carried out to study the spread and magnitude of elevated mutation rates at TFBS. The influence of co-binding of TFs was analyzed using the Jaccard scores.

**Results and Discussions**

We identified 47 TFBS including CTCF, HNF4A, HNF4G and NFIA with significant FC at the core TF binding region (as compared to the expected mutation rate). Most of the TFBS exhibited a general trend of decreasing mutation rates from motif to core to flanks. Regions with high TF occupancy exhibit significantly higher per-base mutation rates compared to regions with medium and low occupancy. CTCF sites exhibit decreased mutations upon the removal of RAD21 and SMC3 sites, highlighting the influence of co-binding TFs on the mutation rates. However, no discernible decrease in FC at core regions was observed for unique TFBS from the other co-binding clusters. Based on the spread of peak shapes of mutation profiles, most broad peaks were equally present in both proximal and distal Transcription Start Sites (TSS) regions and sharper peaks coincided with distal TSS regions.

**Conclusion**

Our analysis reveals that TF binding significantly influences mutation rates at TFBS in liver cancer, which vary for different TFs and co-binding contexts. The spread of the mutation rate varies between different enhancers and promoters, likely due to the local chromatin structure.

### EACR2024-0823

### Analysis of somatic structural variation in malignant pleural mesothelioma using Oxford Nanopore long-read sequencing

*M. Alhazmi[1], E. Baitei[2], A. Dawson[2], C. Poile[2], K. Kutywayo[2,3], A. Bzura[2], A. Nakas[3], J. Dzialo[2], D. Fennell[2], E. Hollox[1]*

*[1]University of Leicester, Genetic and Genome Biology, Leicester, United Kingdom*

*[2]University of Leicester, Leicester Cancer Research Centre, Leicester, United Kingdom*

*[3]University Hospitals of Leicester NHS Trust, Department of Thoracic Surgery., Leicester, United Kingdom*

**Introduction**

Identifying somatic driver mutations is critical in understanding the development of tumours and developing new therapies. Malignant pleural mesothelioma (MPM) is known to show high levels of somatic genome structural variation yet much is currently missed using short-read sequencing technology. Long-read DNA sequencing using Oxford Nanopore Technology (ONT) can detect chromosomal rearrangements and shorter insertion/deletions, collectively known as structural variants (SVs). In this proof-of-principle study, we identify and validate genomic structural variants using long-read sequencing of six primary MPM cell lines, and compare with matched short-read DNA sequencing and RNASeq.

**Material and Methods**

Six primary MPM cell lines were sequenced using ONT, and SVs identified using minimap2, Sniffles and Severus. SVs were then compared to SVs found by whole exome sequencing (WES) using BWA and Manta. The segmentation copy number alteration was detected by cnvkit. The RNA-seq data was used to detect gene expression using Salmon tool.

**Results and Discussions**

The six primary MPM cell lines were sequenced to between 1.6x and 6.5x coverage, with a median read length between 28 kb and 45 kb, and a maximum read length between 254 kb and 467 kb. The most common SV type was an insertion (54%), followed by deletion (42%), translocation (3.3%), inversion (0.2%), and duplication (0.14%). We identified between 5434-15806 potential SVs not reported by WES, between 50 bp to 165 Mb in size. These included 9 SVs affecting known MPM driver genes such *CDKN2A/B* and they part of complex rearrangement. These SVs affecting driver genes were associated with copy number loss and loss of gene expression. ONT was able to identify both simple and complex structural variations. For example, an apparent large deletion of about 12.4Mb at chromosome 9p affecting *CDKN2A/B* was detected by cnvkit from WES but not detected by manta. However, Sniffles, using long read sequence, detects the correct breakpoints, revealing a 31.5 Mb deletion, and Severus analysis on the long-read sequence suggests this deletion is part of a complex rearrangement on chromosome 9, including a large duplicated inverted region on the q arm.

**Conclusion**

Long read sequencing using Oxford nanopore technology identifies structural variants in MPM not identified by short-read sequencing, including potential chromothripsis events. We are currently directly validating complex rearrangements, and are extending our analysis to long-read sequence, at high-coverage, MPM biopsies.

### EACR2024-0829

### Genome-wide CRISPR Screening Identifies RUVBL1/2 Complex as a Druggable Vulnerability in Bladder Cancer

*B. Akdemir[1,2], S. Eris[1,2], M. Kalyoncu[2], E. Cakıroglu[1,2], O.S. Coskun[1,2], G. Karakülah[1,3], S. Senturk[1,2]*

*[1]Izmir International Biomedicine and Genome Institute/ Dokuz Eylul University, Department of Genomics and Molecular Biotechnology, Izmir, Turkiye*

*[2]Izmir Biomedicine and Genome Center, Basic and Translational Research Program, Izmir, Turkiye*

*[3]Izmir Biomedicine and Genome Center, Technological*

© 2024 The Authors. Molecular Oncology is published by John Wiley & Sons Ltd on behalf of Federation of European Biochemical Societies

*Research Program, Izmir, Turkiye*

**Introduction**
Bladder cancer is a common urological cancer and is characterized by high recurrence and limited therapeutic options. Therefore, there is an ongoing need to explore potential new biomarkers and therapeutic targets. The RUVBL1/2 complex, formed by RUVBL1 and RUVBL2 and functioning as a AAA+ ATPase, plays critical roles in regulating various cellular processes. Dysregulated RUVBL1/2 activity has been associated with cancer, with studies demonstrating that genetic and pharmacological inhibition of RUVBL1/2 can mitigate cancer phenotypes. The significance of the RUVBL1/2 complex in bladder cancer remains to be elucidated.

**Material and Methods**
A genome-wide CRISPR/Cas9 screening using the Brunello library was conducted in J82 and RT4 cell lines. Bioinformatic analyses and primary experimental validations in a panel of cell lines identified RUVBL1 and RUVBL2 genes as druggable vulnerabilities in bladder cancer. Probing the impact of RUVBL1/2 complex on cell growth and aggressive cancer traits, extensive cellular and molecular studies were carried out in RUVBL1/2-depleted cells, achieved through CRISPR/Cas9-mediated genetic perturbation or pharmacological inhibition. In-depth functional studies involved in-silico analyses, competitive cell proliferation assay, BrdU incorporation, cell cycle and apoptosis analyses, 2D clonogenic and 3D soft agar anchorage-independent growth assays, migration and invasion, as well as pharmacological inhibition assays.

**Results and Discussions**
Genetic depletion or pharmacological inhibition of the RUVBL1/2 complex using a selective inhibitor, CB-6644, significantly attenuated fundamental cellular and functional hallmarks of cancer cells, including cell proliferation and survival, and 3D anchorage-independent growth. Consistent with these findings, cells subjected to RUVBL1/2 depletion or inhibition demonstrated cell cycle arrest and a notable increase in apoptosis. Furthermore, elevated expression of RUVBL1/2 was observed in bladder cancer tumors compared to normal tissues, with higher expression levels correlating with shorter overall survival.

**Conclusion**
Our novel findings highlight the significant dependency of bladder cancer on the RUVBL1/2 complex, suggesting its potential as a biomarker and a promising therapeutic target for the disease. Further studies focusing on cellular and molecular interactions will contribute to the understanding of the molecular role of RUVBL1/2 in bladder cancer.

### EACR2024-0842
### Functional characterization of ERα-interacting lncRNA PVT1 as a novel putative molecular target for breast cancer treatment

*L. Palo[1], V. Melone[1], D. Palumbo[1], A. Salvati[1], A. Giordano[1], G. Giurato[1], F. Rizzo[1], G. Nassa[1], A. Weisz[1], R. Tarallo[1]*
*[1]University of Salerno, Department of Medicine- Surgery and Dentistry "Scuola Medica Salernitana" DIPMED, Baronissi, Italy*

**Introduction**
In estrogen receptor alpha (ERα) positive breast cancers (BC) the recurrent development of anti-estrogen resistance outlines the need to find alternatives to endocrine therapies. Previous works from our group, aiming to dissect ERα-involving nuclear molecular networks, revealed the key role of RNAs in mediating ERα association with epigenetic modifiers, such as the functional enzymatic component of PRC2 complex EZH2 and the histone methyltransferase DOT1L. The known side effects related to DOT1L pharmaceutical inhibition represent an important limit for the clinical management, so the identification and molecular targeting of specific RNAs bridging the association of such factors with ERα may be crucial for estrogenic signaling disruption and to overcome the occurrence of anti-estrogen resistance. Recent evidences described the existence of a functional cooperation between ERα and multiple lncRNAs in therapy resistance appearance and BC progression. In this context, among ERα-DOT1L molecular partners, we selected lncRNAs for further investigation due to their role in transcriptional regulation and epigenetic chromatin modifications.

**Material and Methods**
Co-immunoprecipitation and native nuclear RNA immunoprecipitation coupled to next generation sequencing (RIP-Seq) were performed in ERα positive MCF7 BC cell model. This method allowed the identification of multiple ERα-interacting lncRNAs, many of them being also associated with DOT1L. Using ASO-mediated lncRNA gene silencing, we assessed the functional effects of these molecules on cell proliferation, migration, apoptosis and their impact on the estrogenic signaling.

**Results and Discussions**
Among lncRNAs identified, we focused our attention on PVT1, since it was found commonly associated with ERα and DOT1L and previously demonstrated to be a molecular partner of EZH2 in other cancer models. Functional assays showed significant decrease of cell proliferation and migration and apoptosis induction following ASO-mediated knockdown of PVT1. Furthermore, we evaluated the role of ERα-PVT1 functional complexes in the regulation of hypoxia and estrogen signaling at transcriptional level.

**Conclusion**
PVT1 was previously described to be essential for BC cell proliferation and, altogether, the obtained results suggest that this lncRNA could represent a key component of estrogen signaling machinery in luminal-like BC cells and a novel molecular target to interfere with ERα depending BC progression.

### EACR2024-0848
### Proffered Paper: Sequence your Cell and Keep it Alive: Cytoplasmic Live-Cell Biopsies for Temporal Single Cell Profiling

*T. Gerecsei[1], K. Keevend[2], T. Pekeč[2], L. Rizzo[2], R. Lafleur[2], M. Milla[2], D. Ossola[2]*

© 2024 The Authors. Molecular Oncology is published by John Wiley & Sons Ltd on behalf of Federation of European Biochemical Societies

[1]Cytosurge AG, SAM, Glattbrugg, Switzerland
[2]Cytosurge AG, RnD, Glattbrugg, Switzerland

**Introduction**

Advances in single-cell transcriptomics have greatly contributed to the understanding of cellular heterogeneity and dynamic gene expression patterns. However, conventional RNA-seq methods require cell lysis - which is terminal in nature, making the understanding of cell lineage decisions and fate determination, challenging, and relying on complex bioinformatic models.

**Material and Methods**

With FluidFM Nanosyringes, cytoplasmic biopsies from cultured single cells can be retrieved (Guillaume-Gentil, Cell, 2014), while preserving their viability. In combination with a highly sensitive, low-input RNA-seq protocol, it was demonstrated that transcriptome snapshots can be captured (Live-seq) and are faithful representations of lysed cell transcriptomes (Chen, Nature, 2022).

**Results and Discussions**

Preserving cell viability maintains temporal information, complements high-throughput datasets, and facilitates the unraveling of subtle cell trajectories. Here we show how the FluidFM OMNIUM® platform enables the serial collection of cytoplasmic biopsies, streamlining the workflow from the insertion of the FluidFM Nanosyringe into the cytoplasm, to the ejection of the biopsy into lysis buffer droplets for downstream analysis.

**Conclusion**

In conclusion, the integration of FluidFM Nanosyringes with sensitive RNA-seq protocols represents a significant advancement in single-cell transcriptomics, offering a non-terminal approach to capturing transcriptome snapshots while preserving cell viability. This innovative method provides a more nuanced understanding of cellular dynamics, enabling the exploration of cell lineage decisions and fate determination with greater accuracy and efficiency. The streamlined workflow offered by the FluidFM OMNIUM® platform demonstrates the potential for future research to delve deeper into the complexities of cellular heterogeneity and gene expression dynamics.

### EACR2024-0850

### Targeting the bromodomain-containing protein BRPF1 as a new therapeutic strategy against endocrine therapy-sensitive and -resistant breast cancers

A. Salvati[1], G. Giurato[1], J. Lamberti[1], I. Terenzi[1], V. Melone[1], L. Palo[1], F. Rizzo[1], R. Tarallo[1], A. Weisz[1], G. Nassa[1]
[1]University of Salerno, Department of Medicine- Surgery and Dentistry “Scuola Medica Salernitana”, Baronissi, Italy

**Introduction**

In hormone-responsive breast cancer (BC), estrogen receptor alpha (ERα) plays a central role in driving tumor growth and progression. Current endocrine-based therapeutic options include ERα-blockade, estrogen synthesis inhibition, and selective ERα degradation. Although Endocrine Therapy (ET) extends patients overall survival, nearly one third of ERα-positive tumors fail to respond to these therapies leading to disease recurrence and relapse. Aberrant activity and constitutive expression of ERα occurs in a substantial fraction of ET-resistance (ET-R) BCs. Given the evidence that ERα oncogenic activity depends upon its interaction with different epigenetic regulators, involved in chromatin remodelling events associated to altered gene expression patterns, our work pointed out how new molecules playing a crucial role in mediating not only ERα nuclear signalling but also the effectiveness to ET regimens.

**Material and Methods**

By combining bioinformatics analysis of genome-wide ‘drop-out’ screenings and ERα interactome profiling revealed a set of essential genes that includes, as the most effective, the bromodomain containing protein BRPF1, an epigenetic reader acting as chromatin remodellers to control gene transcription. To gather mechanistic insight into the role of this epienzyme in breast cancer, we applied chromatin and transcriptome profiling, gene ablation and specific pharmacological inhibition followed by cellular and functional assays. to understand the mechanistic role of BRPF1 in BC, particularly its association with ERα

**Results and Discussions**

Our results suggests that BRPF1 interacts with ERα at chromatin level and its inhibition determine a profound remodelling of chromatin structure and a transcriptional reprogramming in hormone-responsive BC. This is associated to suppression of ER-dependent hormonal signalling and altered cell cycle progression, reduced proliferation, and activation of apoptosis mechanisms, observed in antiestrogen-sensitive and -resistant BC cells and in Patient Derived Organoids (PDOs).

**Conclusion**

In summary, these results indicate that BRPF1 is a critical regulator of BC proliferation and survival, offering new actionable therapeutic targets for treatment of these aggressive tumors.

*Work was supported by Fondazione AIRC per la Ricerca sul Cancro (Grant IG- 23068) to AW. Ministry of Health (GR-202-12373937) and Ministry of University and Research (PRIN 202282CMEA and PRIN-PNRR P2022N28FJ) to GN.*

### EACR2024-0861

### Improving quality and controllability of cancer cell line development with direct intranuclear injection of CRISPR reagents

G. Santos[1], T. Gerecsei[1], A. Kerjouan[2], T. Pekec[3], A. Migenda Herranz[2], S. Mailand[2], M. Milla[3], T. Beyer[1]
[1]Cytosurge AG, SAM, Glattbrugg, Switzerland
[2]Cytosurge AG, CellEDIT RnD, Glattbrugg, Switzerland
[3]Cytosurge AG, RnD, Glattbrugg, Switzerland

**Introduction**

Single-cell analysis has greatly impacted our understanding of population heterogeneity and cellular mechanisms. Our FluidFM® technology addresses many hurdles in single cell research via direct nuclear CRISPR injection. Our objective was to use FluidFM to develop novel approaches for editing cancer cells in a highly controlled manner to improve quality and minimize off target editing.

**Material and Methods**

© 2024 The Authors. Molecular Oncology is published by John Wiley & Sons Ltd on behalf of Federation of European Biochemical Societies

The FluidFM® technology works with the world smallest syringe, enabling precise intranuclear injection of single cells for precision CRISPR mediated gene engineering. Here, we present CellEDIT, a novel approach for single cell-based cell line engineering. Through intranuclear injection of CRISPR ribonucleic protein complexes (RNPs) into single cells, we achieve high editing efficiencies and specificity even in sensitive and hard to transfect cell types. Further, by starting our cell line development process with single cells, we accomplish monoclonality without tedious selection processes.

**Results and Discussions**

Direct intranuclear injection of CRISPR RNPs into a single cell circumvents harsh transfection methods and permits gene editing in hard-to-transfect and sensitive cells. Fine-tuning of the injection parameters enables to minimize the amount of CRISPR RNPs and deliver optimized amounts of HDR-templates. This enhances homologous recombination.

**Conclusion**

To illustrate the power of FluidFM® mediated genome engineering we show how we can generate a triple knock-out in CHO cells in one editing cycle, starting from a few cells only to produce monoclonal lines.

### EACR2024-0888
### Investigating the Effect of Sleep on Cancer Progression

X. Li[1], K.F. Chen[1]
[1]*University of Leicester, Department of Genetics and Genome Biology, Leicester, United Kingdom*

**Introduction**

CFIm25 (*NUDT21/CPSF5*) is a crucial subunit of the Cleavage and Polyadenylation Complex, is a highly conserved cleavage factor I enzyme in humans and plays a vital role in recruiting other subunits of the cleavage complex in alternative polyadenylation (APA), a post-translational process that results in different mRNA isoforms from the same gene, thus APA has been associated with several types of cancer. In a recent study by Flores et al, unique APAs were identified in the rat forebrain, with time-of-day dependent oscillations in expression, and differential expression of APAs was also observed after sleep deprivation and subsequent recovery periods. These findings suggest a connection between circadian rhythmicity and APA. Sleep deprivation has been linked to major illnesses, including cardiovascular disease, depression, and cancer. Although it is well-known that cancer patients often experience insomnia and other sleep disorders, the exact prevalence and impact on cancer progression are still uncertain.

**Material and Methods**

In this study, we will use the *Drosophila melanogaster* animal model to investigate the relationship between sleep and cancer development. We will induce isolated tumorigenesis in the accessory gland with *CPSF5* as our target to interfere with APA processes and monitor the progression of tumour development using the MARCM (Mosaic Analysis with a Repressible Cell Marker) technique, allowing a realistic timeline of tumour development for analysis. These models will then be monitored for sleep analysis under different circadian factors, such as sleep deprivation, different light-dark cycles, and under blue and red-light exposure.

**Conclusion**

Our initial goal is to generate a novel cancer model using *Drosophila melanogaster* to study how tumours develop under various circadian factors, such as sleep quality and timing of sleep. Furthermore, the use of this novel cancer model will also provide insights into the impact of sleep deprivation on health and disease, providing valuable insights into the interplay between sleep, circadian rhythms, and cancer progression.

### EACR2024-0907
### Exploring Cancer Dependencies with Genome-Wide CRISPR Screens: Unveiling BUB1 Kinase as a Druggable Vulnerability in Malignant Pleural Mesothelioma

E. Çakıroğlu[1,2], S. Eris[1,2], O. Oz[1,3,4], G. Karakülah[1,5], S. Senturk[1,2]
[1]*Izmir International Biomedicine and Genome Institute-Dokuz Eylul University, Department of Genomics and Molecular Biotechnology, Izmir, Turkiye*
[2]*Izmir Biomedicine and Genome Center, Basic and Translational Research Program, Izmir, Turkiye*
[3]*Izmir Bozyaka Education and Research Hospital-University of Health Sciences, Department of Pathology, Izmir, Turkiye*
[4]*Izmir Biomedicine and Genome Center, Technological Research Program, Izmir, Turkiye*
[5]*Izmir Biomedicine and Genome Center, Bioinformatics Platform, Izmir, Turkiye*

**Introduction**

Malignant pleural mesothelioma (MPM) is a rare but highly lethal cancer with limited treatment options and low survival rates, creating an urgent need for new therapeutic targets and strategies for personalized treatment. BUB1 (Budding uninhibited by benzi-midazoles) is a mitotic checkpoint serine-threonine kinase that functions at spindle control and accurate chromosome segregation. High levels of BUB1 expression have been linked to poor prognosis in several cancers, and functional studies have shown that silencing and/or pharmacological inhibition of BUB1 attenuates cancer phenotypes. However, the significance of BUB1 in MPM remains unknown.

**Material and Methods**

A genome-wide CRISPR/Cas9 screen was conducted by cross-comparing three different MPM cell lines with a non-tumorigenic mesothelial cell line to explore druggable vulnerabilities for MPM. AURKA, BUB1, and VPS37A genes were identified and validated as high-confidence vulnerabilities. For in-depth analysis of BUB1 in MPM, CRISPR-depleted cells were created and pharmacological inhibition studies were conducted to examine the impact of BUB1 on cell growth and aggressive cancer phenotypes. Finally, single cell knock-out clones were obtained for characterization of molecular and cellular changes in BUB1-deficient MPM cells.

**Results and Discussions**

© 2024 The Authors. Molecular Oncology is published by John Wiley & Sons Ltd on behalf of Federation of European Biochemical Societies

BUB1 gene depletion or inhibition by BUB1 kinase inhibitor, BAY-1816032, attenuated proliferation and 3D anchorage-independent growth of MPM cells. In line with these results, BUB1 depleted/inhibited MPM cells displayed G2/M cell cycle arrest as well as a slight but significant increase in apoptosis. Furthermore, BUB1 depletion reduced the migration and invasion capacity of MPM cells and inhibited tumor growth in a mouse xenograft model. Conversely, ectopic overexpression of BUB1 enhanced cell proliferation and 3D anchorage-independent growth, as well as the migration and invasion capacity of MPM cells. Consistently, high BUB1 expression positively correlates with proliferation and EMT specific gene sets. Most critically, higher levels of BUB1 expression were associated with shorter MPM patient survival.

**Conclusion**

BUB1 is a potent cancer dependency gene and may potentially serve as a biomarker and therapeutic target for MPM treatment. Further studies focusing on molecular interactions and characterization on preclinical models, such as patient-derived tumoroids, will contribute to the understanding of the therapeutic potential of BUB1 in MPM.

### EACR2024-0942

### COMPReS study: Whole exome sequencing reveals organ-specific mutational patterns in metastases and identifies chemotherapy-resistant clones in relapsed localized colon cancer

*B. García Micó[1,2], F. Gimeno-Valiente[3], B. Martínez-Castedo[1,2], J. Martín-Arana[1,2], D. G. Camblor[1], M. García-Bartolomé[1], B. Palomar-de Lucas[4], D. Roda[1,2], A. Cervantes[1,2], N. Tarazona[1,2]*

*[1]Biomedical Research Institute INCLIVA, Medical Oncology, Valencia, Spain*

*[2]CIBERONC, Instituto de Salud Carlos III, Madrid, Spain*

*[3]UCL Cancer Institute, Cancer Evolution and Genome Instability Laboratory, London, United Kingdom*

*[4]Biomedical Research Institute INCLIVA, Pathology, Valencia, Spain*

**Introduction**

Nearly 40% of patients with localized colon cancer (LCC) do not clear circulating tumor DNA after adjuvant chemotherapy (ACT) and eventually relapse. Identifying recurrence risk signatures and selecting patients for more personalized therapies relies on comprehending cellular resistance mechanisms and the host tissue micro-environment. Our goal is to analyze the genomic profile of primary tumors (PTs) with a high risk of relapse and organ-specific metastatic lesions to enhance personalized therapeutic strategies.

**Material and Methods**

Whole exome sequencing (WES) was conducted on 100 FFPE tissue samples from 61 patients with LCC as part of the COMPReS study at the Hospital Clínico Universitario of Valencia, Spain. Of these, 35 samples were PTs from non-relapsed patients and 65 from 26 relapsed patients, which included both PT and their paired metastases (METs), and 9 of these patients exhibited more than one metastatic lesion in different locations. Functional enrichment analysis was done by GSEA, with a significance threshold set at FDR<0.05.

**Results and Discussions**

WES analysis unveiled evolutionary early alterations in *APC*, *KRAS* and *TP53*, being the most frequently mutated genes in both PTs and METs. Additionally, exclusive oncogenic late mutations were identified in METs, encompassing *AURKA*, *NOTCH3*, *FOXO1*, *STAT1,* and *SETD2*, among others. Moreover, in patients with 2 metachronous METs after 1$^{st}$ and 2$^{nd}$ line ACT, respectively, specific variants in *APC* and *TP53* were exclusively detected in the PT and in the last post-treatment MET, suggesting the existence of a selection of residual chemo-resistant clones. Besides that, distinctive genomic profiles were observed among different metastatic sites and a GSEA analysis showed a mutational enrichment in genes encoding cell cycle related targets of E2F transcription factors just in liver metastases. Furthermore, specific mutational signatures were observed in the comparison of PTs from relapsed and non-relapsed patients, with mutated genes in relapsed PT being associated with Wnt β-Catenin, PI3K-AKT-mTOR or TGF-β pathways.

**Conclusion**

WES revealed the emergence of late mutations as well as organ-specific mutational patterns in METs. Moreover, this analysis allowed to identify chemotherapy-resistant clones in relapsed LCC and distinctive mutational signatures that might be related with a higher risk of relapse.

### EACR2024-0983

### Molecular Insights into Colorectal Cancers with Mucinous Morphology

*V. Alberini[1,2], A. Gagliardi[1,3], G. Francescato[1,2], D. Festa[1], D. Ricupero[1], M.P. Dragomir[4], S. Tarallo[1,3], B. Pardini[1,3], G. Ferrero[1,2], A. Naccarati[1,3]*

*[1]Italian Institute for Genomic Medicine, Genetic and Molecular Epidemiology, Candiolo TO, Italy*

*[2]University of Torino, Clinical and Biological Sciences, Torino, Italy*

*[3]Candiolo Cancer Institute, FPO - irccs, Candiolo TO, Italy*

*[4]Universität zu Berlin, Institute of Pathology- Charité, Berlin, Germany*

**Introduction**

Colorectal cancer (CRC) with a mucinous morphology represents a distinct and most frequent (10-20%) histological tumor subtype. Mucinous CRC (mCRC) is conventionally defined by extracellular mucin comprising > 50% of the tumor area, while tumors with ≤ 50% mucin are designated as CRC with mucinous component (CRCmc). This study examined coding and non-coding transcriptome in mCRC and CRCmc in comparison to non-mucinous CRC (nmCRC) to explore differences and peculiarities in relation to patients' clinical features, molecular subtypes, and tumor microenvironment (TME).

**Material and Methods**

An integrated RNA-seq and small RNA-seq profiling followed by paired differential expression (DE) analysis was performed between primary tumor and matched

Molecular Oncology 18 (Suppl. 1) (2024) 1–495
© 2024 The Authors. Molecular Oncology is published by John Wiley & Sons Ltd on behalf of Federation of European Biochemical Societies adjacent tissue of 84 nmCRC patients, 28 CRCmc, and 7 mCRC. RNA-seq data from tissues also allowed consensus molecular subtyping (CMS) and TME cell population estimation to assess, respectively, molecular subtypes and tumor infiltrate populations that best describe mucinous morphology.

**Results and Discussions**

The investigated cohort reflects clinical characteristics of mucinous cancers as reported in literature: patients with mCRC were associated with a worst prognosis, while a higher proportion of invasive tumors characterized both CRCmc and mCRC. From transcriptome analysis, 742 differentially expressed genes (DEGs) were found only for CRC with mucinous morphology and 5,504 DEGs were in common with nmCRC. Among the DEGs unique to mCRC and CRCmc, six (*MCAM*, *ADAMTS9*, *POMT2*, *SEMA5A*, *MUC20*, *ST3GAL1*) are involved in the mucin metabolism pathway. Enrichment analysis of the upregulated mucinous-specific DEGs highlighted pathways linked to extracellular matrix adhesion and organization, development and differentiation. Besides 291 miRNAs commonly DE between tumor and adjacent tissue in CRC with a mucinous morphology and nmCRC, 33 were specific for CRCmc and mCRC. Functional enrichment performed on 130 target genes of the latter DE miRNAs identified the secretory granule organization as the most significant biological process. Ongoing analyses on CMS and TME identified some aspects that seem to characterize the mucinous morphology subtype.

**Conclusion**

Several molecular alterations in CRC with mucinous morphology have been observed at transcriptome level. However, research carried out on larger cohorts is necessary to strengthen the hypothesis that mCRC and CRCmc share a similar molecular landscape.

### EACR2024-1002
### Mitochondrial DNA mutations facilitate tumour initiation while limiting metastatic burden of colorectal cancer

*E. Tolla[1], C. Mendes Correia[1], J. Tait-Mulder[1], R. Ridgway[1], N. Vlahov[1], O. Sansom[1], P. Gammage[1]*
*[1]Cancer Research UK Scotland Institute, University of Glasgow, Glasgow, United Kingdom*

**Introduction**

Mutations in mitochondrial DNA (mtDNA) are among the most common somatic events in the cancer genome (Gorelick et al., 2021). The mitochondrial genome is multi-copy, with typically hundreds to thousands of copies per cell. Mutations in this genome often present as a mixture of mutant and wild-type copies, known as heteroplasmy. In colorectal cancer (CRC) specifically, it has been demonstrated that ~25% of all CRC presents at least one mtDNA truncating mutation, and that mtDNA mutations are predictive of prolonged survival (Gorelick et al., 2021). To date, the role of mitochondrial mutations in cancer initiation, progression and metastasis is unclear. Here, we interrogate the impact of mtDNA mutation on tumour initiation, progression and metastasis in mouse models of CRC.

**Material and Methods**

The mt-tRNA$^{Ala}$ model of mitochondrial dysfunction (Kauppila et al., 2016), a mouse strain which bears heteroplasmic mutation in all tissues, was crossed with well-characterised genetically engineered mouse models of CRC: VillinCre$^{ER}$; APC$^{fl/+}$(APC) and VillinCre$^{ER}$ ; Kras$^{G12D/+}$ ;Trp53$^{fl/fl}$ ;Rosa26$^{N1icd/+}$ (KPN), and the impact of the tRNA$^{Ala}$ mutation in tumourigenesis , cancer progression and metastasis was assessed. Animal experiments were carried out in accordance with the UK Animals (Scientific Procedures) Act 1986 and by adhering to the ARRIVE guidelines with approval from the local Animal Welfare and Ethical Review Board of the University of Glasgow.

**Results and Discussions**

In our studies, survival in tRNA$^{Ala}$/APC mice was inversely correlated to mutant heteroplasmy burden. However, no change in small intestine (SI) and colon tumour burden was apparent compared to controls, indicating a role for mtDNA mutation in cancer initiation. Conversely, tRNA$^{Ala}$/KPN mice exhibited prolonged survival, demonstrating a dramatic reduction in metastatic burden with no observable change in SI or colon tumour burden.

**Conclusion**

Taken together, these data support a role for mtDNA mutation in modulating tumour initiation and metastasis in CRC. Further analyses are ongoing to understand the impact of mtDNA mutation on tumour metabolism and potential genetic/epigenetic processes underlying these survival differences.

### EACR2024-1003
### Modeling castration resistance in GEMMs identify Nsd2 as a target of AR sensitivity

*R. El Bizri[1], K. Ruggero[1], P. Salamanca[1], A. Martinez Tebar[2], X. Wang[1], N. Garcia Lozano[1], A. Semiao[1], A. Aytes[1]*
*[1]IDIBELL, ProCure, Barcelona, Spain*
*[2]IDIBELL, PoCure, Barcelona, Spain*

**Introduction**

The significance of epigenetic deregulation in the emergence of castration resistant prostate cancer (CRPC) and resistance to AR pathway inhibitors (ARPi) is well established. New mutations have been identified in several epigenetic remodelers known for their interaction with the androgen receptor (AR), a principal driver and drug target in PC. Previously, we demonstrated that the chromatin remodeler Nsd2 drives CRPC and that targeting Nsd2 increases survival of PC models. Interestingly, Nsd2 had also been found to act downstream of Ezh2 and required for its oncogenic funcions. Herein, we aimed at exploring the potential benefits of targeting Nsd2 in CRPC. To achieve this goal we (i) generated new genetically engineered models to demonstrate that overexpression of the Nsd2 drives CRPC; (ii) investigated the changes in chromatin accessibility and AR cistrome remodeling mediated by Nsd2 loss and (iii) suggest the Ezh2 inhibition is a potent treatment for CRPC exibiting overexpression of Nsd2.

**Material and Methods**

The NP and NPp53 GEM models were crossed with a gain of function knock-in mutant allele of Nsd2 (Nsd2$^{EK}$) to generate NPN$^{EK}$ and NPp53N$^{EK}$ GEM models. Longitudinal histological analysis was performed in intact and castrated mice to assess tumor penetrance,

© 2024 The Authors. Molecular Oncology is published by John Wiley & Sons Ltd on behalf of Federation of European Biochemical Societies grade and latency. Gene expression and chromatin profiling was carried out in NPp53 and NPp53N$^{EK}$ models and the AR regulatory networks investigated to assess the extent of AR regulon remodeling. Moreover, preclinical anti-Ezh2 drugs (GSK126) treatment was tested on NPp53N$^{EK}$ GEM models.

**Results and Discussions**

Our data demonstrated that Nsd2 gain of function results in shorter latency and enhanced aggressiveness in the NPp53 CRPC model but not in the castration sensitive PC (CSPC) NP model. Moreover, Nsd2 gain in CRPC greatly enhances cell plasticity into heterogeneous mixed basal and luminal phenotypes with focal AR negative, neuroendocrine positive phenotypes. Preclinical targeting of Ezh2 restores AR sensitivity and reverts cell plasticity via a decrease in neuroendocrine and basal differentiation. Finally, gene expression and transcriptional regulation analysis demonstrated that Nsd2 overexpression shapes the AR cistrome in CRPC.

**Conclusion**

In short, Nsd2 overexpression cooperates with AR to drive CRPC and resistance to anti-androgen therapies, and mainly via favoring cell plasticity. Moreover, the data generated in this study suggest new therapeutic opportunities for combination treatments with epigenetic drugs to re-sensitize CRPC to anti-androgen.

### EACR2024-1031
### A restriction-enzyme independent PCR and sequencing-based protocol for rapid identification of virus integration sites in endocrine resistant breast cancer clones

*Z. Huang*[1], *M. Massimino*[1,2,3], *J. Helmijr*[1], *P. Vigneri*[2,4], *J. Martens*[1], *M. Jansen*[1]
[1]*Erasmus Medical Center, Medical Oncology, Rotterdam, The Netherlands*
[2]*A.O.U. Policlinico "G. Rodolico - S. Marco", Center of Experimental Oncology and Hematology, Catania, Italy*
[3]*University of Catania, Department of Surgery and Medical-Surgical Specialties, Catania, Italy*
[4]*University of Catania, Department of Clinical and Experimental Medicine, Catania, Italy*

**Introduction**

Retroviral transduction of cancer cells has been used to identify biologically relevant genes including those related to therapy resistance. Characterization of virus integration sites (VIS) pinpointing these genes, however, has been cumbersome. This study compared innovative protocols for rapid characterization of genomic regions having VIS in retrovirally transduced cancer cell lines.

**Material and Methods**

We developed a protocol to selectively amplify virus-host genome junctions using neomycin (NEO) as unique retroviral vector sequence present in a VIS. The method combines a genomic walking approach, with direct linker-mediated PCR with NEO as a start (LM-NEO-PCR) or a capture of VIS sites using a probe against NEO. Next, a PCR of long terminal repeats (LTR-PCR) was performed to further enrich VIS genomic target regions. LTR-genome junction regions were sequenced (NGS) and reads were mapped to the genome and quantified. Only target regions with at least 100 reads per million bases (RPMs) were evaluated. The protocol performance was assessed on DNA samples from 4 previously reported single clones holding 20 VIS in total. Pure DNA samples but also mixtures and dilution series down to 1% were compared as well as the added value capture versus PCR-based pre-enrichment methods.

**Results and Discussions**

Overall, our protocol detected significantly ($p<0.016$) more VIS-targets at high reads coverage (>1000 RPMs) in samples having VIS-clones compared to those without (controls). In pure samples, LM-NEO-PCR alone was able to find 89% (17 f 20) of the reported VIS-genes at high read coverage in the correct clones; also 7 novel VIS were detected. In mixed samples and dilution series, 15 reported and 2 novel VIS-genes were also detected >1000 RPMs. Three of the six VIS-genes evaluated for all dilutions were detectable at the lowest fraction tested (1%). LM-NEO-PCR alone or NEO-PCR-capture detected a greater number of VIS-genes in mixtures than capture-NEO-PCR, and NEO-PCR-capture resulted generally in higher RPM. The described protocol can conduct VIS target characterization in one month.

**Conclusion**

We developed a restriction enzyme-independent LM-NEO-PCR and NGS-based protocol for rapid characterization of target genes next to VIS. The protocol successfully detected nearly all reported VIS target genes in pure VIS clones and detected half of them at clone fractions of 1%. Therefore, our method will enable VIS target gene identification in future retroviral screens.

### EACR2024-1040
### Changes in Phospho-signalling and transcription in breast tumor Hypoxia and Hypoxia-Reoxygenation

*A. Aboalmaaly*[1], *A. Aboalmaaly*[2], *A. McIntyre*[1], *A. Benest*[1], *C. Carrol*[1]
[1]*School of Medicine, Translational Medical Sciences, Nottingham, United Kingdom*
[2]*Qatar Foundation, Qatar National Research Fund, Doha, Qatar*

**Introduction**

Hypoxia (Low oxygen) is found in 50% of breast cancers. Clinically it is associated with increased invasion, metastasis, therapy resistance, and worse patient survival. Hypoxia drives major hallmarks of cancer phenotypes, including invasion. Breast cancer adaptation to hypoxia is regulated by the HIF transcription factors and epigenetic modifications. In addition, hypoxia modulates signalling pathways. Hypoxic tumour cells can become reoxygenated as they invade away from hypoxic tumour regions or via hypoxia-induced vascularisation of the tumour. We investigated the impact of hypoxia-reoxygenation on signalling, gene expression and cell phenotypes.

**Material and Methods**

Breast tumour cells (MDA-MB-231, MCF7) were subjected to physiological-normoxia (72 hours 8.5% $O_2$), hypoxia (72 hours 1% $O_2$) or hypoxia-reoxygenation (72 hours 1% $O_2$-72 hours 8.5% $O_2$). We investigated the phosphorylation levels of receptor-tyrosine-kinases and intracellular kinases. Gene expression levels were assessed by RNA-Seq. We identified changes in

Molecular Oncology 18 (Suppl. 1) (2024) 1–495
© 2024 The Authors. Molecular Oncology is published by John Wiley & Sons Ltd on behalf of Federation of European Biochemical Societies phenotype including cell migration, and invasion through Matrigel.

**Results and Discussions**

Phosphorylation levels of RTKs were increased for 24% of RTKs in hypoxia and 70% in reoxygenation (n=3). Phosphorylation levels of intracellular kinases were increased for 31% in hypoxia and 67% in reoxygenation (n=3). RNAseq analysis of MDA-MB-231 and MCF7 revealed significant ($p<0.05$, n=3) changes in gene expression in hypoxia (1537 and 1965 genes respectively) and in reoxygenation (2091 and 737 genes respectively). A number of gene expression changes in hypoxia were maintained upon reoxygenation (38% in MDA-MB-231 and 11.5% in MCF7). These genes showed enrichment for metabolic process and signalling pathways including Wnt and TGFβ signalling ($p<0.05$, n=3). Furthermore hypoxia-reoxygenation resulted in increased invasion (58% in MDA-MB-231 and 56% in MCF7) compared to normoxia ($p<0.05$, n=3). Inhibitors of kinases found upregulated in hypoxia and hypoxia-reoxygenation supressed migration and invasion induced by hypoxia-reoxygenation (66% reduction by STAT3 inhibitors and 71% reduction by ALK inhibitors) ($p<0.05$, n=3).

**Conclusion**

Hypoxia and hypoxia- reoxygenation induce changes in kinase signalling, RNA levels, and the capacity of tumour cells to invade. Kinase inhibitors can supress migration induced by hypoxia-reoxygenation, this can lead to developing new inhibitors in the future that can overcome resistance in TNBC and Lumina A and improve patient's survival.

### EACR2024-1075
### Identifying Modifiers of EGFR Induced Tumourigenesis to Develop New Therapeutic Strategies for Lung Cancer

*J. Jajarmi*[1,2], *A. Nagelberg*[1,3], *W. Lockwood*[1,3]
[1]*BC Cancer Research Institute, Integrative Oncology, Vancouver, Canada*
[2]*University of British Columbia, Interdisciplinary Oncology, Vancouver, Canada*
[3]*University of British Columbia, Department of Pathology and Laboratory Medicine, Vancouver, Canada*

**Introduction**

Lung cancer is the deadliest form of cancer worldwide, and accounts for 24% of all cancer deaths in Canada. The most common subtype of lung cancer is lung adeno-carcinoma (LUAD) which is frequently drive by activating mutations in epidermal growth factor receptor (EGFR). While the development of targeted therapies such as tyrosine kinase inhibitors has significantly improved patient outcomes, acquired resistance continues to be a major challenge. It is therefore essential to identify other targetable genes that cooperate with mutant EGFR in driving LUAD tumourigenesis.

**Material and Methods**

Our lab previously utilized a sophisticated in vivo Sleeping Beauty (SB) screen to model EGFR driven LUAD in mice and identify functionally relevant genes that drive tumourigenesis alongside EGFR. A thorough computational analysis of the SB screen results along with TCGA data was performed to determine the genes that were frequently mutated in both SB mice and LUAD patients. This produced a list of 385 genes, several of which have never been implicated in LUAD. We have developed a customized CRISPR library targeting these genes to further investigate these targets in EGFR mutant NIH-3T3 cells.

**Results and Discussions**

Candidate genes identified after culture under numerous conditions are being further studied to determine their role(s) in essential cellular pathways, drug resistance, and LUAD progression.

**Conclusion**

The results of this study will greatly expand our understanding of LUAD progression and lay the groundwork for future therapies against this devastating disease.

### EACR2024-1081
### The local microenvironment induces a functional rewiring in colorectal cancer cells

*P. Andrei*[1,2], *A. Acha-Sagredo*[1,2], *K. Clayton*[1,2], *E. Connick*[3], *M. Green*[4], *F. Ciccarelli*[1,2]
[1]*Barts Cancer Institute, Queen Mary University of London, London, United Kingdom*
[2]*The Francis Crick Institute, Cancer Systems Biology Laboratory, London, United Kingdom*
[3]*The Francis Crick Institute, Advanced Sequencing Facility, London, United Kingdom*
[4]*The Francis Crick Institute, Experimental Histo-pathology, London, United Kingdom*

**Introduction**

Cancer patients showing clinical response to immune checkpoint inhibitors (ICIs) are usually enriched in tumour-infiltrating immune cells, particularly cytotoxic lymphocytes. However, it is not clear whether cytotoxic lymphocytes are responsible for any transcriptional modulation of their surrounding cell neighbourhood. In this study, we attempted to better characterise the transcriptional changes occurring in epithelial cells in contact with neighbouring lymphocytes.

**Material and Methods**

We used CosMx spatial technology on four colorectal cancer (CRC) samples to profile their local immune environment. We performed cell type annotation, and we used boundaries of segmented cells to identify cell-cell contacts between epithelial cells and lymphocytes. Finally, we conducted differential gene expression analysis between epithelial cells surrounded or not by lymphocytes. We validated results derived from differential expression in vitro using co-culture of human CRC cell lines with activated primary lymphocytes.

**Results and Discussions**

We observed a significantly higher expression of genes involved in antigen presentation and interferon response in cancer epithelial cells in touch with intra-epithelial lymphocytes (IEL) compared to epithelial cells distant from any type of IE infiltrates. We obtained the same result across samples, independently on their global level of immune cell infiltration. The induction of antigen presentation and interferon response programmes in cancer cells by IEL was then validated in vitro by co-culturing two CRC cell lines with activated CD8 T cells,

© 2024 The Authors. Molecular Oncology is published by John Wiley & Sons Ltd on behalf of Federation of European Biochemical Societies indicating that cancer epithelial cells can be instructed to become antigen-presenting cells by the neighbouring IEL.

**Conclusion**

Cancer epithelial cells are stimulated to up-regulate genes involved in antigen presentation and interferon response by the presence of neighbouring tumour infiltrating lymphocytes.

### EACR2024-1109

### Advancing Cancer Research: A Novel Digital PCR Tool for Parallel Detection of Multiple Hallmark Mutations in BRAF and EGFR

*C. Kappmeier[1], S. Edward[1], C. Hochstein[1], E. Bruske[1], F. Di Pasquale[1], R. Kellner[1]*

*[1]QIAGEN GmbH, Life Science, Hilden, Germany*

**Introduction**

In the dynamic landscape of precision oncology and translational research the identification of genetic mutations driving cancer becomes more and more important. Our study underscores the critical task of identifying genetic mutations in genes such as BRAF and EGFR, which are important in various cancer types. Our new method for digital PCR (dPCR) reflects a highly sensitive method to analyze key mutations in cancer-associated genes in more detail.

**Material and Methods**

Introducing the innovative dPCR PanCancer Kits (RUO), our research focuses on a pioneering digital PCR (dPCR) approach meticulously designed to concurrently detect hallmark mutations in BRAF and EGFR genes that are pivotal in diverse cancer types. The optimized assays specifically target multiple V600 hallmark mutations in BRAF and many critical exon 19 deletions in EGFR. Including a reference gene for PCR efficiency control and genome copy number quantification, our optimized dPCR setups demonstrate exceptional sensitivity, allowing for the detection of multiple mutations in a single channel, even at allelic frequencies below 1%.

**Results and Discussions**

Extending our study across various sample types, including blood, plasma and FFPE samples, we demonstrate the dPCR PanCancer Kits´ versatility in various research applications. Beyond their utility in pre-screening samples before next-generation sequencing, they prove effective in the ongoing monitoring of cancer cells. Simultaneous assessment of mutations streamlines processes, reducing time, costs, and preserving precious sample materials.

**Conclusion**

Notably, the adaptable nature of our technology suggests the potential development of analogous assays for other cancer-associated genes. In essence, our study highlights the dPCR PanCancer Kits (RUO) as a robust and efficient technological advancement, offering a nuanced understanding of critical mutations in BRAF and EGFR-driven cancers. The dPCR PanCancer Kit is for research use only. Not for the diagnosis, prevention, or treatment of a disease.

### EACR2024-1113

### Application of functional genomics to interrogate the FLCN-TFE axis in Birt-Hogg-Dubé renal cell carcinoma

*F. Johnson[1], I. Glykofridis[1], K. de Lint[1], J. Balk[1], A. Houweling[1], R. Wolthuis[1]*

*[1]Amsterdam UMC - Cancer Center Amsterdam, Human Genetics, Amsterdam, The Netherlands*

**Introduction**

Birt-Hogg-Dubé syndrome (BHD) is characterized by mutations in the FLCN gene, significantly elevating the risk of developing kidney cancer. Restoration of FLCN or knockdown of TFE/TFEB transcription factors is reported to mitigate tumorigenesis in vivo, in xenograft and genetically engineered mouse models (Hong et al., 2010; Di Malta et al., 2023). The current standard of care is primarily surgical, which underscores the need for novel therapeutic strategies. Our work explores the molecular underpinnings of BHD-renal cell carcinoma (BHD-RCC), focusing on functional genomics strategies to understand the dependencies that arise upon FLCN loss and subsequent activation of TFE3/TFEB transcription factors in BHD-RCC cell line models.

**Material and Methods**

Using renal proximal tubule epithelial cells (RPTECs) harbouring deletion of FLCN by CRISPR-Cas9, and the UOK257 BHD-RCC cell line and its FLCN-restored counterpart, we studied the effect of FLCN loss on cellular behavior and gene expression profiles. We applied RNA sequencing, global proteomics, and developed a TFE transcription factor reporter system for the real-time tracking of TFE activity. We are conducting genome-wide CRISPR screens to identify crucial regulators of cell proliferation and the BHD-RCC phenotype, best characterized by a TFE3/TFEB activation state.

**Results and Discussions**

The loss of FLCN and subsequent TFE3/TFEB activation was found to significantly affect gene transcription, enhancing the expression of genes associated with the BHD-RCC phenotype. CRISPR screens underway at the time of this writing are expected to reveal genetic factors essential for maintaining proliferation in FLCN-deficient RCC cell line models and the BHD phenotype, offering potential new targets for therapeutic intervention.

**Conclusion**

Our findings help to understand the central role of FLCN and TFE3/TFEB in the development of BHD-RCC, demonstrating the potential of targeting these pathways. The identification of genetic dependencies associated with the BHD-RCC phenotype may provide a foundation for the development of novel therapeutic strategies, potentially improving the management of BHD patients.

### EACR2024-1143

### Genotyping of unresectable soft tissue sarcomas

*K. Bobak[1], J. Piątkowski[2], P. Golik[2], K.A. Seliga[3], A. Tysarowski[3], M.J. Spałek[1], A. Szumera-Ciećkiewicz[4], H. Koseła-Paterczyk[1], P. Rutkowski[1], A.M. Czarnecka[1]*

*[1]Maria Sklodowska-Curie National Research Institute of Oncology, Department of Soft Tissue/Bone Sarcoma and*

© 2024 The Authors. Molecular Oncology is published by John Wiley & Sons Ltd on behalf of Federation of European Biochemical Societies

*Melanoma, Warsaw, Poland*
*[2]Institute of Genetics and Biotechnology, Warsaw, Poland*
*[3]Maria Sklodowska-Curie National Research Institute of Oncology, Cancer Molecular and Genetic Diagnostics Laboratory, Warsaw, Poland*
*[4]Maria Sklodowska-Curie National Research Institute of Oncology, Department of Pathology and Laboratory Diagnostics, Warsaw, Poland*

**Introduction**

Soft tissue sarcomas (STS) remain a diagnostic challenge due to diverse STS subtypes. The genetic profile of STS is still poorly understood. However, distinctive gene fusions are commonly detected in STS. This study aimed at identify useful diagnostic biomarkers in STS.

**Material and Methods**

We analyzed FFPE core biopsies of 38 patients divided into two groups. These two groups consisted of 32 sarcoma patients and 6 healthy patients as a control group. Enrolled patients included 15 patients with undifferentiated pleomorphic sarcoma (UPS), 8 patients with myxofibrosarcoma (MFS), 1 patient with leiomyosarcoma (LMS), 1 patient with pleomorphic liposarcoma (PLPS), 1 patient with dedifferentiated liposarcoma (DDLPS) 2 patients with myxoid liposarcoma (MLPS), and 4 patients with malignant peripheral nerve sheath tumor (MPNST). For Next-Generation Sequencing (NGS), TruSight$^{TM}$ Oncology 500 kit for Illumina NextSeq was used.

**Results and Discussions**

On average, each patient had at least one gene fusion. The *NTRK1/CHPF* gene fusion was found in one UPS patient. The *PI3KCA/HFM1* gene fusion was detected in one MLPS case. Alterations in the *PIK3CA* gene are one of the most frequent in MLPS. In one MPNST case, we reported the *TP53/RP11-846F4.11* gene fusion. The *TP53/ATP1B2* gene fusion was also found in the UPS patient. In the same UPS patient, the *PIK3CA/SLC25A3* gene fusion was also detected. Other interesting fusions identified included: *JAK2/TRPC6* gene fusion in the MFS patient and the *SDCCAG8/AKT3* gene fusion in the UPS patient.
In 75% of MPNST patients, different variants of the *NF1* gene were reported. In two cases (50%), there were two variants per patient. In one MPNST and two UPS cases, three variants of the *DICER1* gene were found. Mutations in genes encoding protein kinases involved in PI3K-AKT-mTOR or MAPK pathways (e.g. *PDGFRA, PIK3CA, PIK3CD, MAP3K1, MAP3K4, MAP3K13*) were found. Three variants of the *EP300* gene were detected in three different patients (one UPS and two MFS cases). In two MFS patients, two different variants of the *ATM* gene were found. The missense mutation of the *BCL2L2* suppressive (anti-apoptotic) gene was detected in one MFS case.

**Conclusion**

Proliferation, cell cycle, and apoptosis are deregulated in STS. Many sarcomas may be driven by gene fusions. However, further research is needed on a larger group of patients.

### EACR2024-1199
### Multi-omic spatial analysis with simultaneous detection of small RNAs, mRNAs and proteins using the novel RNA in situ hybridization technology

*S. Wrobel[1], A. Dikshit[2], S. Basak[2], S. Deshpande[3], M. Sambhi[3], L.C. Wang[3], M. Srinivasan[3]*
*[1]Bio-Techne, Spatial Biology, Abingdon, UK*
*[2]Advanced Cell Diagnostics, Applications, Newark, USA*
*[3]Advanced Cell Diagnostics, Research & Development, Newark, USA*

**Introduction**

Regulatory RNA molecules such as microRNAs (miRNA) and long non-coding RNAs are important in regulating translation of mRNA to protein. Several miRNAs have been implicated in disease initiation and progression, especially in cancer. The regulatory mechanisms of miRNAs, other small RNAs like antisense oligos (ASOs) and silencing RNAs (siRNA) have been exploited to develop oligonucleotide therapies for 1. undruggable targets, 2. achieving longer-term effects 3. rapid production of therapeutics and 4. lower drug development costs. RNA therapies are typically delivered as a part of nanoparticles or a viral vector. Regardless of the delivery mechanism, methods to understand the biodistribution of the vector, transgene expression and cell type identification in a spatial context is crucial to study the safety and efficacy of these therapies.

**Material and Methods**

Built on flagship RNAscope technology, the RNAscope Plus assay detects 1 small RNA and 3 mRNA targets using TSA-based fluorescent readouts. Fixed, fresh frozen samples and formalin fixed paraffin embedded (FFPE) tissues are supported by manual and automated workflows using the Leica Bond Rx system. This technology was used to investigate spatial expression profile of miRNA and associated RNA targets across different tissue types. We can also combine protein detection using a target antibody to visualize cell-type specific markers.

**Results and Discussions**

We demonstrated expression of miRNAs and target genes implicated in tumor initiation, progression, and angiogenesis. Expression of miR-205 , associated tumor target genes such as *PanCK*, *PTEN* and tumor suppressor *TP53* was visualized in head and neck cancer tumors. Downregulation of tumor-suppressor, *TP53* resulted in upregulation of miR-205 which downregulates *PTEN* expression. *Pan-CK* stained the tumor region in the tissue. Similarly, miR-155 expression was observed in niche areas within breast cancer, head and neck cancer and cervical cancer tumors. Expression of miR-155 demonstrated correlation with high *VEGF* expression suggesting its role in angiogenesis.

**Conclusion**

This novel platform will enable researchers to visualize regulatory RNA simultaneously with target RNAs, cell-type and morphology markers in intact cells/tissues with single cell resolution. It can provide meaningful insights into disease pathology driven by miRNAs as well as assess biodistribution and efficacy of oligonucleotide therapeutics.

© 2024 The Authors. Molecular Oncology is published by John Wiley & Sons Ltd on behalf of Federation of European Biochemical Societies

### EACR2024-1200
### Interrogating the tumor-immune landscape with a novel automated RNA in situ Hybridization assay for multiplexed detection of RNA and protein

*A. Dikshit[1], S. Basak[1], V. Murlidhar[2], G.A. Kim[2], S. Wrobel[3], J. Yu[2], S. Deshpande[2], L.C. Wang[2], M. Srinivasan[2]*
*[1]Advanced Cell Diagnostics, Applications, Newark, USA*
*[2]Advanced Cell Diagnostics, Research & Development, Newark, USA*
*[3]Bio-Techne, Spatial Biology, Abingdon, UK*

**Introduction**
Understanding tissue heterogeneity is critical for elucidating cell-cell interactions with important implications in immuno-oncology, inflammation, and neuroscience. Tissue heterogeneity poses immense challenges to understanding underlying molecular mechanisms using techniques such as qRT-PCR or bulk sequencing. While single-cell RNA sequencing can provide information about precise cellular composition of tissues, data analysis can be cumbersome and spatial context is lost.

**Material and Methods**
With single-cell spatial platforms such as RNAscope, target gene and protein expression can be visualized to characterize cell types and tissue neighborhoods. Here, we demonstrate a novel method for the simultaneous detection of RNA and protein using a modified co-detection assay. This novel co-detection assay enables visualization of a combination of up to 12 RNA and/or protein targets on the same sample. We used a set of antibodies targeting key immune and tumor cell markers- PD1, CD3, CD4, CD8, CD68, FOXP3 and KRT17, along with RNA biomarkers to interrogate the tumor micro-environment (TME) in human FFPE tumor samples.

**Results and Discussions**
Using a combination of RNA and protein targets, we characterized different cell types such as T cells, macrophages, and tumor cells in the TME. We also developed a novel method for visualizing intercellular interactions between PD1 and PD-L1, offering insights into popular checkpoint mechanisms that are targeted for therapeutic intervention in cancer treatment.

**Conclusion**
The assay offers a powerful technique for visualizing target RNA biomarkers in specific cell-types identified by cell-marker protein expression. This is a valuable tool for multiomic analysis and accurate interrogation of complex tissues to obtain insights into novel biomarkers and therapeutic targets.

### EACR2024-1204
### Investigating neuroinflammation in the human brain tumor microenvironment using a novel RNA-protein co-detection assay

*A. Dikshit[1], G.A. Kim[2], J. Yu[2], S. Wrobel[3], S. Deshpande[2], L.C. Wang[2], M. Srinivasan[2]*
*[1]Advanced Cell Diagnostics, Applications, Newark, USA*
*[2]Advanced Cell Diagnostics, Research & Development, Newark, USA*
*[3]Bio-Techne, Spatial Biology, Abingdon, UK*

**Introduction**
Glioblastomas are one of the most aggressive forms of brain tumors characterized by distinct genetic and molecular signatures. The progression of these tumors as well as their response to therapy is impacted by the complex tumor-immune interactions in the micro-environment. Compared to other solid tumors, role of immune cells in progression, invasion and prognosis is not well studies for CNS tumors. Assessing unique features of the brain tumor microenvironment requires a multiomic strategy to identify unique immune cells infiltrating the tumor and their dynamic interactions with other cells within the tumor. Using the flagship single-cell spatial RNAscope technology, target gene and protein expression can be visualized to characterize cell types and tissue neighborhoods.

**Material and Methods**
Here, we demonstrate a novel method for the simultaneous detection of RNA and protein using a modified co-detection assay. With this novel TSA amplification-based co-detection assay we visualized a few combinations of 3 RNA and 3 protein marker panels on human FFPE normal brain and brain tumor tissues. Antibodies targeting key immune cell markers such as CD8, IBA1 and CD68 were used. In addition neuronal and morphology marker antibodies for PanCK and NeuN were included in the panels. RNA probes targeting chemokines and cytokines such as *CXCL10, IFNG, TNFA, CXCL2. IL-6* were also used in the panels. Immune cells infiltrating the brain tumor tissues were characterized by studying co-expression of key RNA and protein markers.

**Results and Discussions**
The tumors demonstrated infiltration of immune cells such as T cells, microglia and macrophages represented by expression of CD8, IBA1 and CD68 protein markers. In addition, expression of cytokines was used to assess the activation status of these immune cells which is an important indicator for potential success of certain therapeutic interventions. Distinct differences in neuro-inflammation signatures were also observed between the normal and tumor brain tissues.

**Conclusion**
The assay offers a powerful technique for visualizing target RNA biomarkers in specific cell-types identified by cell-marker protein expression. This is a valuable tool for multiomic analysis and accurate interrogation of complex tissues such as the brain to obtain insights into novel prognostic and therapeutic biomarkers.

### EACR2024-1222
### Exploring synthetic lethal interactions in the sister chromatid cohesion pathway by multiple parallel genome-wide isogenic CRISPR screens

*K. de Lint[1]*
*[1]Cancer Center Amsterdam- Amsterdam UMC, CRISPR Center- Human Genetics, Amsterdam, The Netherlands*

**Introduction**

© 2024 The Authors. Molecular Oncology is published by John Wiley & Sons Ltd on behalf of Federation of European Biochemical Societies

The cohesin complex is essential for maintaining higher order chromosome architecture and genomic stability, and its impaired function is linked to several genetic syndromes and cancer. A better understanding of the establishment and maintenance of cohesion, and the genetic depedencies that arise when pathway components are mutated or lost, could help identify druggable targets for targeted cancer therapy. By performing genome-wide CRISPR knockout screens in multiple isogenic cell lines, we previously discovered that MMS22L-TONSL functions in the establishment pathway of sister chromatid cohesion, and found that PAXIP1-PAGR1 acts as regulator of chromatin association of cohesin parallel to DSCC1-RFC.To further explore the genetic dependencies in sister chromatid cohesion and identify novel genes involved in the establishment and maintenance of cohesion, we performed additional isogenic screens, and combined the data to identify high-confidence sythetic lethal interactions.

**Material and Methods**

We performed multiple isogenic screens at 400-fold library representation in independent triplicates in the retinal epithelial cell line RPE-1 using the TKOv3 library. The data was analyzed using IsogenicZ, a normalization and analysis method for isogenic screens that is an adaptation of DrugZ, a method for analysis of chemogenetic screens.

**Results and Discussions**

We have currently identified over 250 high-confidence synthetic lethal interactions, and are performing additional screens to further explore the genetic dependencies in sister chromatid cohesion.

**Conclusion**

Our full-genome CRISPR screens in a set of isogenic cell lines are an unbiased and powerful tool to discover genetic interactions.

### EACR2024-1231
### The role of snoRNAs in GBM cells, GSCs, neuronal precursor cells (NPCs), astrocytes, and normal brain tissues

*S. Chatterjee[1], S. Kuersten[2]*
*[1]Illumina, Cancer Research, San Diego, USA*
*[2]illumina, Research & Development, San Diego, USA*

**Introduction**

RNAs select which nucleotides in rRNAs get modified. Typically, C/D box snoRNAs are implicated in 2'-O-ribose methylation (2'Omet) while H/ACA box snoRNAs are responsible for pseudo-uridylations (Y). Alterations in snoRNA expression have been described in multiple tumor types while distinct snoRNAs have been linked to cancer-relevant phenotypes. Our initial study on snoRNA expression using a dedicated Ampliseq platform revealed a specific glioma stem cell (GSC) signature.

**Material and Methods**

We propose this snoRNA signature creates a unique rRNA modification pattern that shapes the ribosome, favoring the translation of genes in pathways critical for GSC growth and survival. We will expand our study and characterize the expression profile of snoRNAs and 2'Omethylation in a large panel of GBM cells, GSCs, neuronal precursor cells (NPCs), astrocytes, and normal brain tissues. Next, we will select snoRNAs displaying differential expression in GSCs and determine if changes in their levels affect proliferation, viability, response to temozolomide and radiation, and translation.

**Results and Discussions**

We and others have observed that the integrity of protein complexes that regulate ribosomal biogenesis depends on PolyADP-ribosylation (PARylation)and PAR-binding. In agreement, genomic screenings determined that multiple genes regulating ribosome biogenesis confer sensitivity to PARP inhibitors. We will investigate the functional impact of PARylation on rRNA modification by determining the effect of PARP inhibitors on snoRNP assembly and 2'Omethylation.

**Conclusion**

These findings offer a therapeutic opportunity. Finally, we will test the hypothesis that PARP and ribosome biogenesis inhibitor combinations would be more effective than PARP inhibition alone in treating GBM.

### EACR2024-1254
### Combinatorial loss-of-function and gain-of-function screening using CRISPR/saCas9 and CRISPR/spCas9 systems

*N. Isachenko[1], G. Aleksanyan[1], P. Diehl[1], D. Tesdesco[1]*
*[1]Cellecta Inc, Mountain View, California, USA*

**Introduction**

This investigation aims to explore the application of orthogonal CRISPR-based gene editing and modulation systems for comprehensive genetic screens. By integrating CRISPR knockout (CRISPR-KO), CRISPR activation (CRISPRa), and CRISPR interference (CRISPRi) functionalities, we seek to enable simultaneous gene silencing and activation within the same cellular environment. This work focuses on the use of Staphylococcus aureus (sa)Cas9 as a complement to the widely used Streptococcus pyogenes (sp)Cas9, particularly in contexts requiring the co-expression of multiple independent CRISPR systems within a single host cell.

**Material and Methods**

To assess the potential of employing various combinations of saCas9 and spCas9 CRISPR systems for concurrent gene inactivation (via CRISPR-KO or CRISPRi) and activation (via CRISPRa), we developed a comprehensive toolkit for CRISPR/saCas9 gene editing and modulation. We designed the toolkit to be compatible with CRISPR/spCas9 co-expression, ensuring versatility in experiments. We developed and validated optimized saCas9 and sg(sa)RNA lentiviral and adeno-associated virus (AAV) vectors, along with dual expression (sp)/(sa)sgRNA lentiviral library vectors. Additionally, we developed fluorescence-based activity kits for (sa)CRISPR-KO, (sa)CRISPRi, and (sa)CRISPRa, facilitating the functional validation of cell lines expressing saCas9.

**Results and Discussions**

The outcomes of this work underscore the practicality of executing orthogonal genetic screens. Our findings demonstrate the effective simultaneous inactivation and transactivation of target genes within the same host cell, leveraging the distinct capabilities of saCas9 and spCas9

© 2024The Authors. Molecular Oncology is published by John Wiley & Sons Ltd on behalf of Federation of European Biochemical Societies

CRISPR systems. The compatibility and functionality of the developed tools and vectors for CRISPR/saCas9 and CRISPR/spCas9 co-expression were successfully validated, showcasing the potential for diverse genetic perturbation strategies.

**Conclusion**

This work validates the feasibility of utilizing orthogonal CRISPR-based gene editing and modulation systems for dual gene inactivation and activation within single cells. The toolkit, encompassing saCas9 and spCas9 CRISPR systems, offers a promising avenue for sophisticated genetic screens and expands the potential for complex genetic studies. Our work lays the groundwork for future investigations into gene function and regulation, paving the way for innovative therapeutic strategies and advancements in drug target and biomarker discovery.

### Carcinogenesis

### EACR2024-0013

### Could oncogenesis be caused by disruption of the molecular structure of intracellular water and its restoration by irradiation?

*V. Kalantaryan[1], R. Martirosyan[1], Y. Babayan[2], V. Petrosyan[3]*

*[1]Yerevan State University, Radiophysics and Electronics, Yerevan, Armenia*

*[2]National University of Architecture and Construction., dept. Physics, Yerevan, Armenia*

*[3]Saratov State Medical University, dept. Physics, Saratov, Russia*

**Introduction**

Previously, we suggested the probability of the occurrence of carcinogenesis due to transformation of the molecular structure of intracellular water in healthy tissues from a conventional hexagonal modification to a cubic one under the influence of various external and internal damaging factors (radiation, chemical, viral, mechanical, microbiological ). The aim of the study was to test this assumption experimentally.

**Material and Methods**

Active microwave exposure was carried out for 30 minutes by streams of therapeutic electromagnetic waves of extremely low intensity of 1 $\mu W/cm^2$ at a resonant frequency of 1000 MHz or at a pathologic frequency of 990 MHz . To register the spectrum of resonant frequencies of water - containing objects, a modulation microwave radiometer with a fluctuation sensitivity of 0.5K was used.

**Results and Discussions**

Specific resonant anomalous spectra of water and malignant tumor tissues were detected and their connection with the cubic structure of water was shown. The resonant spectrum of cubic water completely coincides with the similar spectrum of cancerous tissue. It is shown that the influence of one of the carcinogenic factors on water, gamma radiation, really transforms the normal hexagonal molecular structure of water into an abnormal cubic modification. A local change of the hexagonal water matrix to a cubic one leads to a change in metabolic reactions, cells begin to reproduce abnormal cubic water, which leads to a change in the biochemical mechanisms of their activity up to genetic ones.

**Conclusion**

Preliminary experimental studies show that acting on the water of the cubic structure with a flow of therapeutic microwaves at 1000 MHz, it is possible to change its structure to a normal hexagonal one, which corresponds to a healthy tissue. The use of an external source of therapeutic wave flows gives real control over the process of development of malignant neoplasms. Therefore, it should be expected that the use of non-ionizing and non-thermal electromagnetic radiation at selective frequencies should lead to the recovery of the body and in the future this method may find wide application for the diagnosis, prevention, and therapy of cancer without damage to other tissues and harmful ionizing radiation therapy.

### EACR2024-0143

### Inflammasome-independent function of NLRC4 in promoting the DNA damage response and attenuating tumour development

*C. Shen[1], A. Pandey[1], D.E. Tuipulotu[1], A. Mathur[1], N. Adikari[1], C. Ngo[1], Y. Hao[1], M. Kirkby[1], N. Kaakoush[2], S.M. Man[1]*

*[1]The Australian National University, The John Curtin School of Medical Research, Canberra, Australia*

*[2]The University of New South Wales, School of Biomedical Sciences, Sydney, Australia*

**Introduction**

Intestinal cancer is a leading cause of cancer-related deaths. The inflammasomes, a vital part of innate immune responses, contribute to prevent intestinal cancer induced by chronic inflammation. However, whether the inflammasomes could mediate intestinal cancer development driven by genetic predispositions has remained poorly characterised.

**Material and Methods**

Mice with *adenomatous polyposis coli* mutations ($Apc^{min/+}$) were used to identify the role and mechanisms of inflammasome sensors in tumour development. Mouse intestinal organoids and other cell types, including macrophages, HEK293 and THP-1 cells, were used to further investigate the functions identified in vivo.

**Results and Discussions**

We screened the role of several inflammasome pathway sensors, including AIM2, NLRP3, NLRC4 and NLRP6, in tumour development driven by *Apc* mutations. Our study revealed that the inflammasome sensor NLRC4 attenuates tumour development in $Apc^{min/+}$ mice. In addition, we found that the inflammasomes are functional during tumour development, whereas NLRC4 functions independently of its canonical inflammasome signalling capability. Further, ontology analysis on the differential phosphorylated proteins in the absence of NLRC4 during tumour development revealed a DNA-damage-related role of NLRC4. Indeed, we found that NLRC4 reduces the accumulation of DNA damage both in vitro and in

© 2024 The Authors. Molecular Oncology is published by John Wiley & Sons Ltd on behalf of Federation of European Biochemical Societies vivo. Mechanistically, NLRC4 interacts with the DNA-damage sensing ATR-ATRIP complex to promote the recruitment of the checkpoint adaptor protein Claspin, licensing the activation of the kinase CHK1. Genotoxic-induced activation of NLRC4 drives the DNA damage response, diminishing DNA damage accumulation, thus preserving genomic integrity and shielding against cancer.

**Conclusion**

These findings demonstrate a non-canonical function of an inflammasome protein in promoting the DNA damage response and mediating protection against cancer.

### EACR2024-0148

### Hypermethylation responses to inorganic arsenic exposure may increase the risk of liver cancer occurrence

*P.J. Liao[1], K.H. Hsu[2]*

*[1]Chang Gung University, Master Degree Program in Health and Long-term Care Industry, Taoyuan, Taiwan*

*[2]Chang Gung University, Laboratory for Epidemiology-Department of Health Care Management, Taoyuan, Taiwan*

**Introduction**

Exposure to inorganic arsenic (InAs) has been known as a risk factor for liver cancer. However, documents have indicated that specific forms of arsenic may inhibit the activity of hepatitis viruses. This study investigated the association between InAs exposure and liver cancer occurrence while examining the effects of arsenic methylation capacity in an exposed cohort.

**Material and Methods**

This study followed 4,056 residents, an average of 19.2 years of follow-up, from an arseniasis area in Taiwan, and identified 90 liver cancer cases. The inorganic arsenic metabolites were determined by ICP-MS and methylation capacity was calculated from the combination of InAs, monomethylarsonic acid (MMA), and dimethylarsinic acid (DMA). The primary methylation index (MMA/InAs) and the secondary methylation index (DMA/MMA) and their median high/low combinations were used. Cox proportional hazards model was performed to calculate the hazard ratio (HR) of liver cancer occurrence.

**Results and Discussions**

The analyses revealed a significant association between liver cancer occurrence and arsenic methylation capacity, which demonstrated a lower risk of the participants with low secondary methylation index. The results showed that participants with lower primary and lower secondary methylation index than their respective median values were at a lower risk of liver cancer (HRs from 0.230 to 0.231) than those with high methylation capacity. The incidence density of liver cancer ranging from 48.2/100,000 ($year^{-1}$) to 100.4/100,000 ($year^{-1}$) for residents with low secondary methylation index and from 99.1/100,000 ($year^{-1}$) to 186.8/100,000 ($year^{-1}$) for residents with high secondary methylation index when the arsenic exposure dose was classified into quartiles.

**Conclusion**

Hypomethylation responses to InAs exposure may associate with lower liver cancer occurrence. Two mechanisms were conjectured. First, a specific forms of arsenic may inhibit the hepatitis viral activity. Second, hypermethylation activities among residents chronically exposed to inorganic arsenic may induce arsenic carcinogenesis, which warrants for further investigations.

### EACR2024-0158

### The R2TP complex is a novel host factor in the HPV-mediated molecular pathogenesis of cervical cancer

*M. Shadang[1], S. Mathur[2], R. Mir[3]*

*[1]All India Institute of Medical Sciences- Delhi, Biochemistry, Delhi, India*

*[2]All India Institute of Medical Sciences, Pathology, Delhi, India*

*[3]All India Institute of Medical Sciences, Biochemistry, Delhi, India*

**Introduction**

PIH1D1 (PIH1 domain containing 1/NOP17, nucleolar protein 17) is an evolutionarily conserved subunit of the R2TP complex (RUVBL1-RUVBL2-RPAP3-PIH1D1), an HSP90 co-chaperone. The complex stabilises and assembles multimeric molecular complexes. PIH1D1 recruit clients via adaptors/proteins phosphorylated by casein kinase II. The interactions and collaborative functions of the R2TP complex with its adaptors and clients remain poorly understood and unexplored. The present study investigates the role of the R2TP complex in human papillomavirus (HPV)-mediated cervical cancer.

**Material and Methods**

The expression of the R2TP complex and survival analyses were explored using open-access online tools, GEPIA2 and Kaplan-Meier plotter. Protein-protein interactions were analysed by GST pull-down assay and co-immunoprecipitation in cervical cancer cell lines, HeLa and Ca Ski. The key residues responsible for the interaction were identified and verified by site-directed mutagenesis. Co-localisation of proteins was via immunofluorescence. Functional assays like PI staining, wound healing and MTT assay were conducted after cell lines were transfected with siRNA targeting PIH1D1. Protein and transcript levels were measured via immunoblotting and quantitative real-time PCR. Expression of the R2TP complex in tissue sections of cervical carcinoma (n=30) was analysed by immuno-histochemistry.

**Results and Discussions**

TCGA data shows a high expression of the R2TP complex was correlated with increased survival. Silencing of PIH1D1 destabilized E7 levels, reducing cell proliferation, migration and invasion in vitro. There was a significant decrease in the levels of E2F1 targets - PCNA, CYCA2, CYCE1 and DHFR. PIH1D1 interacts with E7 proteins of high-risk HPV types 16 and 18 and the RB (retinoblastoma) protein. The mutation of HPV16 E7 at S31/S32 to A31/A32 abolishes the phosphorylation of E7 by casein kinase II, eliminating the interaction of PIH1D1 with E7. However, PIH1D1 still interacted with RB, irrespective of the phosphorylation status of E7. In cell lines, PIH1D1, RPAP3 and E7 predominantly co-localizes in the nucleus. Immunohistochemistry staining in carcinoma samples shows higher expression of the R2TP complex than in normal tissue.

© 2024 The Authors. Molecular Oncology is published by John Wiley & Sons Ltd on behalf of Federation of European Biochemical Societies

**Conclusion**
Our study has identified the R2TP complex as a novel interacting partner of E7 in HPV-mediated cervical cancer and a critical host factor in the disruption of the cell cycle. These results also shed light on E7-host interactions as potential therapeutic interventions.

### EACR2024-0263
### The effect of PDGFA on cell cycle transcription factors during the malignant transformation of neural progenitor cells

M. Meode[1], H.K. Omairi[1], S. Sait[1], S. Black[1], J.G. Cairncross[1]
[1]Arnie Charbonneau Cancer Institute- University of Calgary, Department of Clinical Neurosciences- University of Calgary, Calgary, Canada

**Introduction**
The lethal brain cancer, Glioblastoma (GBM), has a distinctive genomic architecture, but no known cause or curative treatment.
**Material and Methods**
Using a new in vitro murine model of GBM [Bohm et al., 2020 and Omairi et al., 2023] in which P53 null neural progenitors (NPCs) undergo defective mitosis, become growth factor independent, and acquire a GBM-like genome during exposure to Platelet-Derived Growth Factor-AA (PDGFA), we examined gene and protein expression to determine how this mitogen could cause continuous chromosome instability (CIN).
**Results and Discussions**
We found that PDGFA fails to induce the transcription of kinetochore and spindle assembly checkpoint genes, while simultaneously driving NPCs to enter mitosis. These dual effects caused poor metaphase plate development and abnormal chromosome segregation in continuously proliferating NPCs. Transcriptome analysis of NPCs cultured in PDGFA revealed significant under-expression of *Foxm1*, the major regulator of kinetochore transcription, and over-expression and phosphorylation of the immediate early response gene, FOS. In addition, analysis of cell signalling downstream of PDGFRα, the sole receptor for PDGFA, suggested that the Ras-MAPK pathway, especially ERK, was responsible for activation of FOS. [These phenomena were also seen in P53 WT NPCs exposed to PDGFA, but unlike null cells, WT cells did not survive defective mitosis.] The pool of surviving P53 null NPCs gradually expanded while accumulating random and clonal chromosomal rearrangements. Expansion and tumorigenicity were associated with re-expression of *Foxm1* and kinetochore and spindle assembly checkpoint proteins, and were accompanied by over-expression of *Egfr*, a receptor tyrosine kinase commonly activated in GBM.
**Conclusion**
By stimulating proliferation without setting the stage for proper mitosis, exposure to PDGFA transforms p53 null NPCs and generates *Egfr* amplified GBM-like cancer cells.

### EACR2024-0415
### Investigating the Immunomodulatory Potential of Pancreatic Cancer-Derived Extracellular Vesicles via Proteomic and Functional Profiling

A. PIRO[1], M.C. Cufaro[2], P. Lanuti[3], L. De Lellis[1], R. Florio[1], S. Pagotto[4], S. Pilato[1], D. Brocco[5], A. Cama[1], S. Veschi[1]
[1]G. d'Annunzio University of Chieti-Pescara, Department of Pharmacy, Chieti, Italy
[2]G. d'Annunzio University of Chieti-Pescara, Department of Pharmacy & Center for Advanced Studies and Technology, Chieti, Italy
[3]G. d'Annunzio University of Chieti-Pescara, Center for Advanced Studies and Technology & Department of Medicine and Aging Sciences, Chieti, Italy
[4]G. d'Annunzio University of Chieti-Pescara, Center for Advanced Studies and Technology, Chieti, Italy
[5]G. d'Annunzio University of Chieti-Pescara, Department of Medical- Oral and Biotechnological Sciences, Chieti, Italy

**Introduction**
Pancreatic cancer (PC) displays resistance to immunotherapy and exhibits a strongly immuno-suppressive tumor microenvironment. A better understanding of the effects of tumor-derived extra-cellular vesicles (EVs) on immune responses may contribute to improved immunotherapy.
**Material and Methods**
EVs derived from Capan-2 and BxPC-3 PC cells were isolated by ultracentrifugation and characterized by atomic force microscopy, western blot (WB) and nanoparticle tracking analysis. Fresh PBMCs from healthy donors were treated with EVs and CD3+ lymphocytes were isolated by fluorescence-activated cell sorter. The expression of CD69 and PD-1 in CD8+ and CD4+ lymphocytes was assessed by flow cytometry in EV-treated PBMCs. Proteomics of EVs and CD3+ lymphocytes from EV-treated, or -untreated PBMCs was performed and IFN-γ concentration in supernatants was measured by ELISA.
**Results and Discussions**
Capan-2 and BxPC-3 derived EVs had a globular shape, an average size of 241 nm and 137 nm respectively and expressed EV-specific markers by WB. Proteomic analysis of Capan-2 and BxPC-3 EVs identified proteins connected in a single functional network ($p=1x10^{-16}$) by STRING analysis. According to STRING, the majority of proteins identified in Capan-2 and BxPC-3 were involved in "Extracellular exosome" (FDR: 2.09x10-63 and 2.74x10-75, respectively), confirming the EV origin of the protein dataset. Notably, most of these proteins were involved in "Immune System" (FDR: 1.10x10-24 and 3.69x10-19, respectively). Interestingly, stimulation of healthy donor-derived PBMCs with Capan-2 derived EVs, but not BxPC-3 or control EVs increased the percentages of CD69 and PD-1 expression both in CD8+ and CD4+ lymphocytes. Since EVs from Capan-2 promoted the expression of lymphocyte activation markers, we analyzed the proteomics of lymphocytes treated with Capan-2 EVs or control EVs. Proteomics of CD3+ sorted from PBMCs treated with Capan-2 versus control EVs showed the activation of "Cell viability" (z-score 5.801) and "Cell survival" (z-score 5.811) associated with activation of IFN-γ, as an upstream regulator, after Capan-2 EV-treatment. This

Molecular Oncology 18 (Suppl. 1) (2024) 1–495
© 2024 The Authors. Molecular Oncology is published by John Wiley & Sons Ltd on behalf of Federation of European Biochemical Societies result was validated by ELISA measurements of IFN-γ in the supernatants of EV-treated CD3+.

**Conclusion**

Our proteomic and functional analyses indicate that pancreatic cancer-derived EVs have a pleiotropic role on immune regulation.

*This study was supported by PRIN 2017 EKMFTN_005 granted to Alessandro Cama.*

### EACR2024-0440
### The inducible KPC:APC mouse model for studying colon carcinogenesis in vivo and ex vivo

*M. Gomot[1], N. Essakhi[2], Q. Pascal[3], D. Klokov[2], G. Varès[2]*
*[1]Institut de Radioprotection et de Sureté Nucléaire IRSN, Pse-sante/Sesane/Lrtox, Fontanay-aux-roses, France*
*[2]Institut de Radioprotection et de Sureté Nucléaire IRSN, Pse-sante/Sesane/Lrtox, Fontenay-aux-roses, France*
*[3]Commissariat à l'énergie atomique et aux énergies alternatives CEA, Imva-Hb/Idmit, Fontenay-aux-roses, France*

**Introduction**

The B6.Cg-Krastm4Tyj Apctm1Tno Tg(CDX2-cre/ERT2)752Erf/MaraJ mouse model (referred to as KPC:APC) recapitulates colon carcinogenesis through the chromosomal instability pathway via inducible *Apc* and *Kras* mutations in *Cdx2*-expressing intestinal cells (Maitra et al., 2019). Following induction with tamoxifen, KPC:APC mice develop tumors resembling human colon cancer. However, further investigations are needed to better characterize the time course and characteristics of tumor development. Furthermore, we developed an ex vivo inducible colorectal cancer model derived from the KPC:APC mouse to study cellular and molecular pathways involved in Apc-Kras-driven colon carcinogenesis.

**Material and Methods**

*Apc* and/or *Kras* mutations were induced in adult KPC:APC mice via single or repeated 10 to 20 mg/kg intraperitoneal tamoxifen administration. Tumor development was histologically assessed between 4 and 12 weeks post induction. Using intestinal crypts extracted from KPC:APC mice, we developed 3D organoids to perform cellular and molecular characterizations.

**Results and Discussions**

Tumor development was highly dependent on tamoxifen administration regimen and mouse genotype (mutations in *Kras* and/or *Apc* genes*). Apc* mutation induction alone resulted in hyperplasia and dysplastic lesions, while the co-induction of *Apc* and *Kras* mutations accelerated adenoma development. Regardless of the genotype, lesions remained primarily restricted to the colon. KPC:APC mouse organoids were treated with tamoxifen to induce tumorigenesis.

**Conclusion**

Here we show that the KPC:APC mouse model is a relevant model for colon cancer research with its inducibility allowing for the coexistence of *Apc* and *Kras* mutation and its lesions mainly contained to the colon. Enhanced knowledge on tumor development will allow investigations on the effects of stressors (such as medical diagnostic exposures to low dose radiation) on colon carcinogenesis. The establishment of an ex vivo model combined with the fully characterized in vivo model provides the opportunity to conduct parallel histopathologic and mechanistic studies to advance colon cancer research.

### EACR2024-0448
### Polθ inhibitors show preclinical efficacy as monotherapy and strong synergy with PARP inhibitors in vivo

*P. Costales Vigil[1], J. Majithiya[1], M. Roy-Luzarraga[1], V. Grinkevich[1], E. Rajendra[1], M. Stockley[2], R.A. Heald[2], H. Robinson[1], G. Smith[1]*
*[1]Artios Pharma, Biology, Cambridge, United Kingdom*
*[2]Artios Pharma, Chemistry, Cambridge, United Kingdom*

**Introduction**

DNA polymerase theta (Polθ) is essential for microhomology-mediated DNA double strand break repair and has been proposed as an attractive target for the treatment of BRCA-deficient and other DNA damage response (DDR) defective cancers. Polθ has limited expression in normal tissues but is frequently over-expressed in cancer cells and, therefore, inhibition of this enzyme is expected to offer high therapeutic index**.** Artios has developed potent, highly selective, and oral inhibitors of the DNA polymerase activity of Polθ, specifically inhibiting MMEJ but not other DNA repair mechanisms.

**Material and Methods**

To explore the in vivo activity of these compounds we have performed several tumour xenograft models including those deficient for homologous recombination (HR) or the TP53BP1/Shieldin complex. Polθ inhibitors (Polθi) were administered orally as monotherapy or in combination with different PARP inhibitors. Tumour growth and body weight were monitored and micronuclei (MN) analysis in blood reticulocytes was evaluated for in vivo target engagement.

**Results and Discussions**

MN analysis revealed that Polθi significantly induced MN in blood reticulocytes, confirming in vivo target engagement. Notably, the combination of Polθi with PARP inhibitors in BRCA-deficient tumours significantly improved tumour growth inhibition, causing tumour regression at low doses. Furthermore, as monotherapy, Polθi inhibited tumour growth in BRCA1 null, SHLD2 deficient tumour xenografts. As loss of the TP53BP1/Shieldin complex has been described as a source of PARP inhibitor resistance, Polθ inhibitors have potential to provide clinical benefit to cancer patients that have relapsed on a PARP inhibitor.

**Conclusion**

Collectively, our results show that Artios Polθ inhibitors are well tolerated and elicit strong synergy with PARP inhibitors in vivo. These data support the clinical exploration of Polθi for the treatment of genetically defined subsets of cancer, alone and in combination with PARP inhibitors.

### EACR2024-0545
### Macrophage-specific knockout of MTDH

© 2024 The Authors. Molecular Oncology is published by John Wiley & Sons Ltd on behalf of Federation of European Biochemical Societies

### discordantly regulates tumor growth and metastasis by TSP-1

X. Liu[1], W. Zhang[1]
[1]*Central Hospital Affiliated to Shandong First Medical University, Research Center of Translational Medicine, jinan, China*

**Introduction**

Macrophages are critical mediators during the process of tumor progression such as angiogenesis and fibrosis. Here ,we elucidate function and mechanism of macrophage with MTDH knockout in tumor progession.

**Material and Methods**

RNA sequencing was employed on bone marrow derived macrophage (BMDMs) isolated from control and knockout mice. The mouse colorectal cancer cells and melanoma cells were subcutaneously and intravenouly injected into myeloid-specific MTDH-/- mice to assess the tumor growth and lung metastasis. Anti-TSP-1 antibody was used to assess the effect of MTDH-/- macrophage on angiogenesis and tumor progression.TSP-1/TGFβ specifc inhibitor was used for suppressing fibrosis in vivo and in vitro.

**Results and Discussions**

Loss of MTDH in BMDMs transcriptionly significantly upregulated the expression TSP-1, which is an angiogenesis inhibitor and an activator of transforming growth factor-β1 (TGF-β1). Myeloid-specific knockout of MTDH abrogated angiogenesis in vivo and in vitro, which further reduced lung metastases but not the subcutaneous tumot growth. Interestingly, we found increased fibrosis accompanied with reduced angiogenesis in subcutaneous tumors but not lung metastases. The use of specific anti-TSP-1 blocking antibodies led to a drastic inhibition of angiogenesis. Moreover, treatment with TGFβ1 induced MTDH-/- BMDMs to undergo macrophage-to-mesenchymal transition (MMT). TSP-1/TGF-β1 inhibitor attenuated the fibrosis and subcutenous tumor growth in vivo and in vitro. Further analysis indicated that MTDH knockout in macrophages led to a pronounced increase of TSP-1 production due to increased transcriptional activation of p53.

**Conclusion**

In conclusion, macrophages with MTDH knockout regulating angiogenesis and fibrosis via TSP-1, which played different roles in tumor growth and metastasis.

### EACR2024-0609
### Obesogenic action of tributyltin in periprostatic adipose tissue: from adipokine and chemokine imbalance to prostate carcinogenesis

M. Feijó[1], L.R.S. Fonseca[1], C.R. Casanova[1], E. Kiss-Toth[2], S. Correia[1], S. Socorro[1]
[1]*CICS-UBI - Health Sciences Research Centre, University of Beira Interior, Covilhã, Portugal*
[2]*Department of Infection- Immunity and Cardiovascular Disease, University of Sheffield, Sheffield, UK*

**Introduction**

The 'obese' periprostatic adipose tissue (PPAT) has been implicated in the aggressiveness of prostate cancer (PCa), with the dysregulation of secreted adipokines and chemokines significantly contributing to a tumour-promoting microenvironment. Evidence also links obesity with environmental influences, namely, by the action of the so-called obesogens, i.e. endocrine-disrupting chemicals capable of dysregulating adipose tissue and promoting fat accumulation. Herein, we hypothesize that obesogens alter the PPAT phenotype, stimulating prostate carcinogenesis.

**Material and Methods**

PPAT isolated from 4-month-old rats was exposed *ex vivo* to the first described obesogen tributyltin (TBT, 100 nM) for 48 h. Morphological features of PPAT and its secretome were evaluated by histological analysis and colorimetric assays. Conditioned media assays were performed by exposing non-neoplastic human prostate cells (PNT1A) to the secretome of TBT-treated PPAT (and control) for 24 h. PNT1A cell fate was evaluated by MTT assays, Ki-67 immunocytochemistry, caspase-3- and caspase-9-like activity, scratch assay, and Western blot analyses.

**Results and Discussions**

TBT promoted adipocyte enlargement, which was accompanied by alterations in its secretome. TBT increased leptin and C-C motif chemokine ligand 7 (CCL7) content in the PPAT culture media while decreasing adiponectin. Previous findings have indicated that augmented leptin/adiponectin ratio is associated with increased cell proliferation and cancer progression. In turn, increased CCL7 secretion has been shown to stimulate adipocyte-dependent migration of PCa cells that express the C-C motif chemokine receptor 3 (CCR3). Indeed, our results demonstrated that the PPAT alterations driven by TBT impact non-neoplastic prostate cell fate. TBT-deregulated PPAT secretome increased the viability, proliferation and migration of PNT1A cells, whereas suppressing apoptosis as indicated by the decreased caspase-3-like activity and caspase-8 expression. These alterations were also underpinned by the increased expression of tribbles pseudokinase 1, phosphorylated extracellular signal-regulated kinase 1/2, and CCR3.

**Conclusion**

This study first demonstrated that exposure to the obesogen TBT dysregulates PPAT in supporting a pro-tumorigenic microenvironment. It highlighted the role of the environment-obesity-PCa triad in fuelling PCa onset, bringing functional evidence about the capability of TBT as a driving force in prostate carcinogenesis by disrupting the cell fate balance of non-neoplastic cells.

### EACR2024-0660
### EBV Detection Among Solid Tumors by Next-generation Sequencing

J. Honecker[1], D. Paffenholz[1], J. Jungmann[1], T. Wieland[1], C.M. Vanderbilt[2], S.R. Yang[2], S. Lakis[3], B. Decker[4], D.A. Mata[4]
[1]*Foundation Medicine GmbH, Computational Biology, Penzberg, Germany*
[2]*Memorial Sloan Kettering Cancer Center, Department of Laboratory Medicine and Pathology, New York, USA*
[3]*Foundation Medicine GmbH, Pathology, Penzberg, Germany*
[4]*Foundation Medicine Inc., Pathology, Cambridge, USA*

**Introduction**

© 2024 The Authors. Molecular Oncology is published by John Wiley & Sons Ltd on behalf of Federation of European Biochemical Societies

Multigene panel-based comprehensive genomic profiling (CGP) assays are important diagnostic tools to assess genomic alterations in a tumor-agnostic manner. Including probes against nonhuman DNA during hybrid-capture allows for the detection of DNA attributable to viruses such as EBV in a single sequencing reaction. As viral read status is determined independent of the tumor type, CGP can help refine the diagnosis for cases where EBV encoded RNA (EBER) in situ hybridization (ISH) was not performed due to a lack of clinicopathologic suspicion. Here we report a comparison of EBV viral read detection using the FoundationOne®CDx (F1CDx®) and FoundationOne®Heme (F1H™) next-generation sequencing (NGS) assays against orthogonal EBER ISH testing.

**Material and Methods**

CGP with F1CDx or F1H was performed in an accredited laboratory and 423 samples consisting of squamous cell carcinomas, gastric carcinomas, and diffuse large B-cell lymphomas (DLBCLs) with orthogonal EBER ISH results were included in the study. EBER ISH results were extracted from pathology reports submitted by the ordering facilities. Area under the curve (AUC), accuracy, sensitivity, specificity, and precision were calculated. A dilution experiment of DNA derived from three EBV immortalized cell lines was performed to assess linearity and reproducibility.

**Results and Discussions**

Across all cancer types, the AUC was 97.4%, specificity was 99.1%, sensitivity was 94.0%, accuracy was 98.3%, and precision was 95.5%. A strong correlation between the median number of EBV viral reads per dilution step and the proportion of EBV-positive cell-line content was observed across all three dilution experiments ($R_{pearson} > 0.99$ and $P < 0.001$). A high reproducibility was demonstrated by a low coefficient of variation between repeats, ranging from 2.5 – 7.5%. The genomic landscape of the EBV-associated carcinomas differed from the non-EBV-related cancers in a predictable manner, with each group exhibiting expected characteristic mutational and cytogenetic profiles congruent with their EBV status, further confirming the validity of the method.

**Conclusion**

NGS-based EBV detection performed well when compared to the gold standard orthogonal testing strategy. Our data demonstrate that CGP is a promising tool for detecting EBV together with genomic alterations in tumor specimens. Our approach could help reduce the complexity of biomarker testing, providing comprehensive results as part of a single sequencing reaction.

### EACR2024-0814
### Estimation of the contribution of HRR panel testing for identification of the genetic predisposition in breast and ovarian cancer

*R. Dodova*[1], *P. Mitev*[1], *S. Usheva*[2], *A. Nachev*[3], *M. Koleva*[4], *V. Ivanova*[5], *T. Sedloev*[2], *R. Dimitrov*[3], *K. Timcheva*[6], *R. Kaneva*[1]

[1]*Medical University, Molecular Medicine Center, Sofia, Bulgaria*

[2]*UMHAT "Queen Joanna - ISUL", Clinic of Surgery, Sofia, Bulgaria*

[3]*University Obstetrics and Gynecology Hospital "Maichin dom", Second Gynecological Clinic, Sofia, Bulgaria*

[4]*MHAT "St. Sofia", Clinic of Oncology, Sofia, Bulgaria*

[5]*Medical University, Department of Clinical Pathology, Sofia, Bulgaria*

[6]*MHAT "St. Ivan Rilski', Clinic of Oncology, Sofia, Bulgaria*

**Introduction**

In Bulgaria, over 16,000 women develop cancer each year. The most frequent is breast cancer (BC) with 26.2% of all cases, while ovarian cancer (OC) is 5.3%. Previous genetic analyses of BRCA1 and BRCA2 genes in 890 BC and OC patients showed that 11 recurrent mutations in the two genes are found in 14,16% of the patients, while 9.32% of all patients carry the most frequent mutation c.5266dup (p.Gln1756fs) in BRCA1 gene. Loss of function mutations in other genes coding partners of BRCA1/2 in Homologous Recombination Repair (HRR) pathways are also contributing to breast and ovarian cancer. The advent of NGS allows the estimation of their frequencies among cancer patients and may have clinical implications for precision treatment.

**Material and Methods**

A total of 49 Bulgarian patients, 18 with ovarian cancer and 31 with breast cancer, that turned negative after preliminary screening for recurrent BRCA1/2 mutations were included in the study. Blood was collected from all patients for DNA isolation, following informed consent. Genetic analysis with a custom panel of 31 HRR related genes, was done by sequencing on PGM (IonTorrent) next generation sequencing platform (ThermoFisher) and analyzed with VarSeq Torrent Suite software. All identified genetic variants were evaluated for their clinical effect in ClinVar.

**Results and Discussions**

After the assessment of the clinical significance of the identified variants, in 28.57% (14/49) of the patients pathogenic and possibly pathogenic variants were identified in the genes ATM (c.9139C>T, p. Arg3047Ter), BRCA1 (c.139T>C, p.Cys47Arg; c.181T>G, p.Cys61Gly; c.2557C>T, p.Gln853Ter; c.3700_3700GTAAA, p.Val1234Glnfs*8), BRCA2 (c.3545_3546delTT, p.Phe1182fs), MUTYH (c.1187G>A, p.Gly396Asp), PALB2 (c.172_175delTTGT, p.Gln60Argfs*7), RAD51D (c.803G>A, p.Trp268Ter), and TP53 (c.725G>A, p.Cys242Phe; c.560-1G>A; c.794T>C, p.Leu265Pro). In addition, in 26.53% (13/49) of the patients, variants of unclear clinical effect were found.

**Conclusion**

The HRR panel testing helped in identification of the genetic predisposition to breast and ovarian cancer in about one third of the investigated cases. The results are of clinical relevance and can predict the risk of cancer in patients' families. The timely use of such a panel can be informative also for the identification of patients that might benefit from PARP inhibitor treatments and potentially minimizing exposure to unnecessary therapies.

*Acknowledgment. Funding: MES, and NRRP: Grants D01-302/17.12.2021, D01-165/28.07.2022, BG-RRP-2.004-0004-C01*

© 2024 The Authors. Molecular Oncology is published by John Wiley & Sons Ltd on behalf of Federation of European Biochemical Societies

### EACR2024-0841
### A changed perspective on oral carcinogenesis by longitudinal analysis of precancerous lesions

*L. Wils[1], J. Poell[1], A. Brink[1], L. Peferoen[2], I. Evren[3], E. Brouns[3], J. de Visscher[3], E. van der Meij[3], E. Bloemena[2], R. Brakenhoff[1]*
*[1]Amsterdam UMC, Otolaryngology / Head and Neck Surgery, Amsterdam, The Netherlands*
*[2]Amsterdam UMC, Pathology, Amsterdam, The Netherlands*
*[3]Amsterdam UMC, Oral and Maxillofacial Surgery and Oral Pathology, Amsterdam, The Netherlands*

**Introduction**
Progression models of cancer still define the carcinogenesis paradigm, but the order of genetic events is now commonly deduced from cross-sectional analyses of tumor and precancer biopsies, rather than empirically determined based on longitudinal analyses of progressing precancers. Here, we describe a unique longitudinal study of oral leukoplakia (OL) lesions that transformed into carcinoma, in which we analyze genetic and histomorphological changes over time to reconstruct the carcinogenic process.

**Material and Methods**
We included 21 OL lesions that transformed into an oral squamous cell carcinoma during follow-up, and had biopsy material available for minimally the OL lesion at first visit and the tumor. Biopsy material for 29 intermittent time points were also included. Median follow-up was 60 months (range: 12-183). Lesions were histopathologically assessed for epithelial dysplasia and CK13 and CK17 staining pattern. Genomic copy number alterations and mutations in oral cancer driver genes were detected by low-coverage whole genome sequencing and target-enriched deep sequencing respectively. The longitudinal genetic data was used in Pyclone-VI and CALDER to reconstruct the phylogenetic history of all 21 lesions.

**Results and Discussions**
We distinguished three progression routes for OL lesions. Ten lesions followed the canonical progression model with increasing morphological and genetic changes over time, in seven patients the morphologic and genetic changes remained constant during follow-up and malignant transformation and in four patients a non-linear trajectory with heterogeneous clone selection was followed. Treatment interventions impacted the clonal composition, underscoring the heterogeneity of the lesion and the associated genetically altered field.

**Conclusion**
This study provides a novel perspective on the temporal evolution of OL and the different routes to cancer and further showcases the key role of field cancerization in lesion recurrence, cancer development and cancer heterogeneity.

### EACR2024-0853
### Deficiencies in Homologous Recombination or the Fanconi Anemia Pathway Sensitize Tumors to the Novel Hypoxia-Activated Prodrug CP-506

*L. Schuitmaker[1], A. van der Wiel[1], N. Mutsters[1], V. Claudino Bastos[2], S. Langie[2], J. Setton[3], J. Theys[1], A. Yaromina[1], L. Dubois[1], P. Lambin[1]*
*[1]Research Institute for Oncology & Reproduction GROW Maastricht University, The M-Lab- Department of Precision Medicine, Maastricht, The Netherlands*
*[2]Research Institute of Nutrition and Translational Research in Metabolism NUTRIM- Maastricht University, Department of Pharmacology and Toxicology, Maastricht, The Netherlands*
*[3]Memorial Sloan-Kettering Cancer Center, Department of Radiation Oncology, New York, United States*

**Introduction**
The novel hypoxia-activated DNA crosslinking agent CP-506 selectively targets hypoxic tumor cells, which are associated with disease progression and treatment resistance. Here, we evaluated the role of DNA damage repair pathways in the antitumor response to CP-506.

**Material and Methods**
Isogenic cancer cell lines (HCT116, DLD-1, LNCaP AR) proficient or deficient for the homologous recombination (HR), non-homologous end joining (NHEJ) or Fanconi Anemia (FA) pathway were cultured as 2D monolayers and 3D spheroids. Cell viability, clonogenic cell survival, and spheroid growth inhibition were assessed following CP-506 exposure. DNA damage induction and repair were evaluated by γH2AX immunofluorescence and comet assay. Mice bearing subcutaneous isogenic xenografts received CP-506 (600 mg/kg) or vehicle (water for injection) for five consecutive days upon reaching a tumor starting volume (SV) of 200 $mm^3$. Tumor response was quantified as time to reach 4xSV (T4xSV) and respective enhancement ratios (ER) defined as the ratio of $T4xSV_{CP\text{-}506\text{-}treatment}$ to $T4xSV_{vehicle\text{-}treatment}$. DNA damage induction in isogenic xenografts was evaluated using γH2AX expression.

**Results and Discussions**
In vitro*, c*ell lines deficient for HR or FA, but not NHEJ, showed enhanced sensitivity to CP-506 as compared to parental cells in viability and clonogenic assays. This was confirmed in spheroid growth inhibition studies. CP-506 caused DNA damage exclusively under anoxic conditions showing the most pronounced induction in HR-deficient cells as assessed by immunofluorescence. Comet assay data are currently being analyzed. In vivo, antitumor response to CP-506 was more pronounced (P<0.0001) in HR-deficient $HCT116^{BRCA2-/-}$ (ER 4.0±0.6) xenografts compared to NHEJ-deficient $HCT116^{DNA\ PKcs-/-}$ (ER 1.4±0.3) and parental HCT116 (ER 1.7±0.5) xenografts. Similar results were obtained in $DLD\text{-}1^{BRCA2-/-}$ (ER 2.9±0.7) versus parental DLD-1 (ER 1.3±0.2; P<0.0001) xenografts. CP-506 treatment of FA-deficient LNCaP $AR^{FANCA-/-}$ (ER 4.0±1.0) and LNCaP $AR^{FANCD2-/-}$ (ER 3.9±0.7) xenografts resulted in significantly (P<0.0001) higher ER compared to parental LNCaP AR (ER 1.5±0.3) xenografts. CP-506-induced DNA damage in ex vivo tumors by γH2AX expression is currently being evaluated.

**Conclusion**
Deficiencies within HR and FA, but not NHEJ, enhanced the antitumor effects of CP-506. Therefore, DNA repair status in addition to tumor hypoxia is an important

© 2024 The Authors. Molecular Oncology is published by John Wiley & Sons Ltd on behalf of Federation of European Biochemical Societies patient stratification factor to maximize the treatment benefit in the upcoming clinical trial of CP-506 (NCT04954599).

### EACR2024-1142
### Elucidation of molecular features associated with progression of Monoclonal Gammopathies to Multiple Myeloma

*J. Zoidakis*[1,2], *J. Courraud*[3], *F. Paradeisi*[4], *G. Medard*[5]
[1]*Biomedical Research Foundation Academy of Athens, Biotechnology, Athens, Greece*
[2]*National and Kapodistrian University of Athens, Biology, Athens, Greece*
[3]*National and Kapodistrian University of Athens, Clinical Therapeutics, Athens, Greece*
[4]*Agricultural University of Athens, Biotechnology, Athens, Greece*
[5]*National and Kapodistrian University of Athens, Chemistry, Athens, Greece*

**Introduction**
Multiple Myeloma (MM) is a chronic malignancy characterized by slow progression and recurrences. Currently there is no effective cure since eventually the disease develops resistance to all the available therapeutic approaches. Although recent advances have expanded our understanding of the cellular functions associated with health to disease transition, recurrence and response to therapy, critical aspects of this complex pathology remain to be elucidated. MGUS (monoclonal gammopathy of undetermined significance ) is present in 3-5% of the ageing European population and every year, 1% progress to incurable MM that imposes a significant burden on EU societies and health systems. Thus, the best chances of curing MM may be in preventing its progression in the first place.
**Material and Methods**
The proteomic profiles of CD138+ cells isolated from bone marrow biopsies were analyzed by high resolution mass spectrometry (Bruker TIMS-TOF Flex). Statistical and bioinformatics analysis of the identified proteins in 60 samples collected from individuals with MGUS, smoldering MM (sMM) and MM was performed using R scripts and the Metascape platform in order to identify deregulated proteins and biological pathways associated with progression of MM.
**Results and Discussions**
Proteomics analysis yielded more than 4000 protein identifications per sample and offered a comprehensive molecular phenotype of the three stages characterising MM progression. A progressive up-regulation of protein synthesis and proteasomal activity was detected when comparing MGUS with sMM and MM. Moreover, significant changes in the metabolic profile of the three stages indicated that the onset of MM is associated with activation of the oxidative phosphorylation, oxidative stress and mitochondrial dysfunction.
**Conclusion**
Application of proteomics and bioinformatics approaches on samples obtained from all informative stages (MGUS, sMM, MM) allowed us to identify biological pathways and molecules responsible for the onset and progression of MM.

### Drug Resistance

### EACR2024-0052
### DEAD-box RNA Helicase DP103 Regulated SUMO/Acetylation Switch of p53 Determines Response to Docetaxel in ERα-positive Breast Cancer

*A.P. Kumar*[1]
[1]*National University of Singapore, Department of Pharmacology, Singapore, Singapore*

**Introduction**
Docetaxel is an essential drug for treatment of metastatic breast cancer, however, patients often develop resistance, precluding effective treatment. Given that the molecular determinants responsible for docetaxel resistance are not known, there is an urgent need to identify robust biomarkers that could predict drug sensitivity to prevent development of chemoresistance. Recently, DP103, a DEAD-box RNA helicase, was found elevated in breast cancer and has been associated with reduced p53 pro-apoptotic signaling. The present research aimed to investigate the mechanism underlying the DP103-mediated docetaxel resistance and DP103's potential to serve as chemo-responsive marker in metastatic breast cancer.
**Material and Methods**
We employed transcriptomic and immunohistochemical analyses of breast cancer biopsies from patients enrolled in a phase II randomized study to evaluate DP103 expression following alternating docetaxel and doxo-rubicin chemotherapy regimens. This was followed by gain- or loss-of-function experiments, luciferase reporter assays and chromatin immunoprecipitation assays to determine the binding occupancy of DP103 on ERα. Additionally, we carried out computational protein docking analysis and simulation dynamics to evaluate interaction surfaces and binding energy between DP103 and p53.
**Results and Discussions**
Clinical trial data demonstrated DP103 expression decreased progressively in response to chemotherapy in ERα-positive patients and associated significantly with docetaxel response. Similarly, in vitro studies revealed that docetaxel reduced DP103 and ERα expression in docetaxel-sensitive breast cancer cells but not in drug-resistant derivatives. Gain- or loss-of-function experiments indicated that DP103 modulated breast cancer cell sensitivity to docetaxel via p53. In silico docking predicted direct interactions for DP103 at p53's DNA-binding domain. The mechanism underlying docetaxel chemosensitivity involves DP103-mediated activation of ERα via suppression of p53 by promoting its sumoylation over acetylation, which, in turn, increased expression of DP103 via a feedback loop.
**Conclusion**
Together, our results indicate for the first time DP103 acts as a "master switch" that controls p53

© 2024 The Authors. Molecular Oncology is published by John Wiley & Sons Ltd on behalf of Federation of European Biochemical Societies transcriptional activation and serves as a surrogate marker for clinical docetaxel response. These findings suggest that targeting DP103 to relieve its interaction with p53 offers a potential therapeutic opportunity to enhance docetaxel sensitivity of ERα-positive breast cancer.

### EACR2024-0068
### Comprehensive treatment of lung cancer with EGFR-ALK double target mutation

S. Liu[1], H. Yu[1]
[1]Cancer Hospital Affiliated to Chongqing University, Department of Geriatric Oncology/Department of Palliative Care, Chongqing, China

**Introduction**

Female, 67 years old, was admitted to hospital due to "diagnosis of 'lung adenocarcinoma' for 2 years after comprehensive treatment" as the main complaint, to be evaluated. The patient was diagnosed with "malignant tumor of the lower lobe of the right lung (adeno-carcinoma, T3N3M0 stage IIIC)" in our hospital 2 years ago (October 2021) with 75.3% exon deletion mutation of EGFR 19. The disease progressed after 6 months of targeted therapy with ohicitinib, focal radiotherapy and palliative surgery. Exon 2 of EML4 and exon 20 of gene ALK fuse, and the targeted drug Latinib is replaced for targeted therapy. The patient currently has a high quality of life and stable condition assessment, and continues to receive targeted therapy with loratinib.

**Material and Methods**

Imaging, tissue and molecular pathology were used to diagnose the disease.

**Results and Discussions**

The patient's tumor condition was stable, quality of life was high, and continued to receive targeted therapy of loratinib.

**Conclusion**

The tumor was stable and continued to be treated with loratinib.

### EACR2024-0069
### Comprehensive analysis of on-target HER2 resistance mutations

I. Farahani[1,2], K.J. Kurppa[1,2], K. Elenius[1,2]
[1]University of Turku, Medicity Research Laboratories and Institute of Biomedicine, Turku, Finland
[2]University of Turku and Åbo Akademi University, Turku Bioscience Centre, Turku, Finland

**Introduction**

The HER2 receptor tyrosine kinase, also known as ERBB2, has been extensively studied as a cancer drug target. HER2-activating point mutations and small indels have been found in various human cancers. These mutations can drive malignancies similar to HER2 amplification and are responsive to HER2-targeted tyrosine kinase inhibitors (TKI). However, recent studies have revealed disease progression in cancer patients treated with TKIs due to the development of on-target resistance mutations. Moreover, different TKIs might retain activity in the presence of on-target resistance mutations for another specific TKI. Therefore, it is crucial to identify the resistance mutation spectra for different HER2 TKIs. This study addresses the development of on-target HER2 resistance mutations using the in vitro screen for activating mutations (iSCREAM), a functional genetics screen previously developed in our laboratory.

**Material and Methods**

To perform a modified version of iSCREAM, retroviral libraries of randomly mutated HER2 cDNAs containing one of the well-known HER2 activating mutations (S310F, L869R, V777L, or L755S) were generated and expressed in Ba/F3 cells. Ba/F3 cells rely on exogenous interleukin-3 (IL-3) for survival and gain IL-3-independence by ectopic expression of an active kinase, such as HER2. The Ba/F3 cells expressing the HER2 mutation libraries will be expanded under IL-3-depleted conditions and treated with HER2 TKIs. Under HER2 TKI treatment, clones containing on-target resistance mutations will survive. Next-generation long-read sequencing of the HER2 retroviral inserts from the surviving Ba/F3 cell pools will be carried out to identify functional HER2 mutations. Further in vitro cell models and in silico analyses will be used to validate the identified mutation hits.

**Results and Discussions**

The known activating HER2 mutations (S310F, L869R, V777L, or L755S) demonstrated varying proliferation rates and sensitivity to HER2 TKIs in IL-3-independent Ba/F3 cells, validating the modified iSCREAM pipeline.

**Conclusion**

Oncogene-targeted therapy using TKIs may lead to resistance due to on-target mutations. On the other hand, some other targeted TKIs may retain functionality in the presence of the on-target resistance mutations. We will identify and validate the HER2 on-target resistance mutation spectrum of different HER2 TKIs. This work can provide invaluable information in choosing suitable HER2 TKI therapy for tumors harboring HER2 resistance mutations.

### EACR2024-0085
### Extracellular vesicles in breast cancer-hepatic communication and their role in chemoresistance

C. Ramos[1], J. Rueff[1], M. Kranendonk[1], F. Esteves[1]
[1]NOVA Medical School/Faculty of Medical Sciences-Universidade NOVA de Lisboa, Center for Toxico-genomics & Human Health ToxOmics, Lisbon, Portugal

**Introduction**

Tumour-derived extracellular vesicles (TEV) play a pivotal role in cell-to-cell communication, both local and systemic, and have been suggested to contribute to chemoresistance by shuttling functional cargo affecting drug efflux/transport and metabolism. Systemic effects of TEV, particularly on the liver, the major site of drug metabolism, remain unclear. This study aimed to determine the effect of breast cancer (BC) TEV on the expression profiles of Phase I drug metabolism enzymes (DMEs) and other oxygenises and reductases in hepatic cells, and their role in the development of chemoresistance.

**Material and Methods**

Small TEV (< 200 nm) were isolated from two types of BC (MCF7) spheroids, namely parental/doxorubicin

Molecular Oncology 18 (Suppl. 1) (2024) 1–495
© 2024 The Authors. Molecular Oncology is published by John Wiley & Sons Ltd on behalf of Federation of European Biochemical Societies

(DOX)-sensitive - TEV$^S$ and resistant to 45 nM DOX (resembling the early development of DOX resistance stages in BC) - TEV$^R$. Subsequently, hepatic (HepG2) spheroids were exposed to both types of TEV, during 24 hrs. Expression of 92 genes of HepG2 spheroids, incubated with DOX alone, or DOX in combination with the two types of MCF7-derived TEV, was assessed using RT-qPCR.

**Results and Discussions**

TEV$^R$-titers were significantly higher ($p < 0.001$), when compared with those of TEV$^S$. Expression of *PGRMC1*, *CYP3A7* and *CYP2D6* was upregulated in HepG2 spheroids, in the presence of both types of TEV, while additional 15 genes (particularly *CYP1A1*, *2J2*, *4F11*) were upregulated only with TEV$^R$ and no DOX treatment. *POR,* which sustains multiple pathways in drug metabolism, showed upregulation in response to both types of TEV, following DOX stimulation, but exclusively in the case of TEV$^R$ in spheroids without DOX exposure. Upregulation of *SQLE*, involved in cholesterol metabolism and dependent on *POR* activity, was exclusive for TEV$^R$ and independent of DOX exposure.

**Conclusion**

Both types of TEV induced upregulation of DMEs involved in DOX metabolism, indicating that even chemo-sensitive BC cells secrete TEV that can potentially enhance DOX metabolism in hepatocytes. This also suggests a TEV-dependent basal communication between BC and the hepatic niche. Over-expression profiles induced by TEV$^R$ are associated with xenobiotic, cholesterol, and fatty acid metabolism, which has been implicated in chemoresistance, growth, and metastasis. TEV$^S$ and TEV$^R$ modulate the gene expression landscape differentially, altering drug response mechanisms of recipient hepatocytes, indicating their putative role in BC-liver communication axis.

### EACR2024-0086

### Inhibition of Ptch1 drug efflux activity : a promising strategy to overcome chemotherapy resistance of cancer cells

*I. Mus-Veteau[1], S. Kovachka[2], S. Cogoluegnes[1], E. Martin[3], S. Azoulay[3]*

*[1]Institut of Molecular and Cellular Pharmacology, CNRS – University Côte d'Azur, Valbonne, France*

*[2]Institut de Chimie de Nice ICN, CNRS - University Côte d'Azur, Nice, France*

*[3]Institut de Chimie de Nice, CNRS - University Côte d'Azur, Nice, France*

**Introduction**

90% of deaths from cancer are the result of recurrence or metastasis due to tumor cell resistance to anti-cancer treatments. The presence of proteins capable to efflux chemotherapeutic agents out of tumor cells is one of the major mechanisms of chemotherapy resistance and the main cause of therapeutic failures. Some multidrug transport proteins (known as multidrug resistance (MDR) proteins or drug efflux pumps) from the ABC transporters family have been identified as responsible for chemotherapy resistance. Numerous inhibitors of these ABC transporters have been developed but none of these molecules has obtained marketing authorization (MA). These molecules are either ineffective, or toxic given the importance of these transporters for homeostasis of healthy cells. The need for drugs capable of combating resistance to chemotherapies and targeted therapies via inhibition of the MDR transporters involved in treatment resistance is therefore as strong as ever.

**Material and Methods**

TCGA data analysis, cancer cell culture, Ptch1 silencing, doxorubicin efflux, cell viability, wound-healing, microscale thermophoresis, in silico docking, cancer cells xenografts in Chick Eggs and mice.

**Results and Discussions**

Hedgehog signaling is aberrantly activated, and the Hedgehog receptor Ptch1 is overexpressed in many recurrent and metastatic cancers. We showed that Ptch1 pumps chemotherapeutic agents such as doxorubicin out of cancer cells using the proton motive force and contributes to chemotherapy resistance of several cancer cell types, and that cells overexpressing Ptch1 at their plasma membrane have persistent (or cancer stem cell) properties. We identified a small molecule which inhibits the doxorubicin efflux activity of Ptch1 and enhances its cytotoxicity in different cancer cell lines endogenously overexpressing Ptch1, and thereby mitigates the resistance of these cancer cells to doxorubicin. We also showed that this Ptch1 drug efflux inhibitor enhances the efficacy of kinase inhibitors such as vemurafenib against melanoma cells resistant to the treatment in cellulo and in vivo on xenografts in mice.

**Conclusion**

Our data suggest that the use of an inhibitor of Ptch1 drug efflux in combination with chemotherapy or targeted therapy could be a promising therapeutic option to improve treatment efficacy against cancer cells expressing Ptch1, reduce relapse and increase patient survival.

### EACR2024-0095

### Selection of RT-qPCR housekeeping genes for investigations of hypoxia-driven breast cancer progression

*J. Malcolm[1], K. Bridge[1], A. Holding[1], W. Brackenbury[1]*

*[1]York Biomedical Research Institute- University of York, Department of Biology, York, United Kingdom*

**Introduction**

Breast cancer is the most common malignancy diagnosed worldwide. Prognosis is improving, however one third of patients develop therapy resistance. Thus, identification of novel therapeutic targets for effective breast cancer treatment is imperative. Oxygen deprivation (hypoxia) occurs in solid tumours and affects expression of genes regulating cell behaviour, metabolism, and resistance to therapy. RT-qPCR is routinely used to quantify mRNA levels, and is vital for identifying important genes involved in malignant progression. Housekeeper genes (HKGs) are endogenous controls essential for normalising measured mRNA levels, so discrete alterations in expression are accurately assessed. However, hypoxia affects expression of common HKGs (e.g. *GAPDH*, *ACTB*) rendering them unsuitable for important RT-qPCR studies. The goal of this work was to identify HKGs suitable for use in hypoxia studies of breast cancer progression.

© 2024 The Authors. Molecular Oncology is published by John Wiley & Sons Ltd on behalf of Federation of European Biochemical Societies

**Material and Methods**
RNA-seq data of MCF-7, T-47D, MDA-MB-231 and MDA-MB-468 breast cancer cells cultured in normoxia (20% O2) or hypoxia (1% O2) were analysed, and HKG candidates were selected based on read count stability between each condition. RT-qPCR was performed to validate HKGs in the four cell lines cultured in normoxia, or hypoxia for 8 or 48 hours, mimicking acute and chronic hypoxia. Computational HKG screening programmes were employed (RefFinder, comparative ΔCt method, BestKeeper, NormFinder or geNorm) to identify stably expressed HKGs.
**Results and Discussions**
*RPLP1*, *RPL27* and *OAZ1* were ranked the most stable HKGs in each cell line with differing O2 availability. Additionally, geNorm found using *RPLP1* and *RPL27* together to be optimal when investigating hypoxic-driven gene transcription in breast cancer cell lines. Conversely, *CCSER2* and *GUSB* were the worst ranked candidates by all algorithms.
**Conclusion**
Our findings suggest *RPLP1* and *RPL27* are the best HKGs to use in RT-qPCR for normalising expression of genes of interest in hypoxic breast cancer cell lines. Our work highlights the importance of fully evaluating HKG expression under experimental conditions prior to starting RT-qPCR studies. This is particularly important for studies attempting to recapitulate aspects of the tumour microenvironment (e.g. hypoxia). Findings that are relevant in 2D without these considerations may not translate clinically if the in vitro controls don't account for these factors.

### EACR2024-0105
### Mangifera indica L. kernel ethanol extract inhibits cell viability and proliferation with induction of cell cycle arrest and apoptosis in lung cancer cells

*H.A. Alshwyeh[1], W. Ibrahim[2]*
*[1]Imam Abdulrahman Bin Faisal University, Department of Biology- College of Science, Damam, Saudi Arabia*
*[2]Qatar University, Biomedical Sciences, Doha, Qatar*
**Introduction**
Lung cancer remains a significant public health concern, necessitating the exploration of novel therapeutic agents. In this study, we focused on investigating the effects of an ethanolic extract derived from Mangifera indica L. kernel on the viability and proliferation of human lung cancer cells. Understanding the potential of natural compounds like M. indica L. kernel extract could offer promising avenues for lung cancer treatment.
**Material and Methods**
To assess the impact of the M. indica L. kernel extract, we employed a range of assays including MTT and BrdU cell proliferation assays, morphological evaluations, cell cycle analyses, and apoptosis assays. These assays were conducted on both lung cancer cell lines (A549 and NCI-H292) as well as normal lung cells (MRC-5), allowing us to compare the extract's effects between cancerous and non-cancerous cell populations.
**Results and Discussions**
Our findings revealed that the M. indica L. kernel extract exhibited a notable toxicity towards lung cancer cells in comparison to normal lung cells. Furthermore, the extract demonstrated a dose-dependent anti-proliferative effect on lung cancer cells. Analysis of the cell cycle indicated that the extract induced G0/G1 arrest and increased the Sub-G1 population in both cancerous and normal lung cells. Additionally, the extract induced distinct morphological changes such as loss of membrane integrity, shrinkage, membrane blebbing, and apoptosis specifically in lung cancer cells, while normal cells exhibited only early apoptosis. Interestingly, the extract displayed higher toxicity towards the NCI-H292 lung cancer cell line, followed by A549 cells, with normal MRC-5 cells being the least affected.
**Conclusion**
Our study underscores the potential therapeutic significance of the ethanolic extract of M. indica L. kernel in targeting lung cancer cells. The extract's ability to induce apoptosis selectively in cancerous cells while causing minimal harm to normal cells suggests its promise as a novel therapeutic agent for lung cancer treatment. These findings warrant further exploration into the mechanisms underlying the extract's anti-cancer properties and its potential for clinical application in lung cancer therapy.

### EACR2024-0125
### A small molecule reverses chemotherapy resistance by targeting DNA polymerase η-mediated translesion DNA synthesis in ovarian cancer

*S. Bose[1], P. Saha[1], M.T. Alam[1], M. Sarkar[1], R.K. Pathak[2], P. Tripathi[3], A.K. Srivastava[1]*
*[1]CSIR-Indian Institute of Chemical Biology, Cancer Biology and Inflammatory Disorder Division, Kolkata, India*
*[2]Indian Institute of Science Education and Research Berhampur, Department of Chemistry, Berhampur, India*
*[3]CSIR-Indian Institute of Chemical Biology, Cell Biology and Physiology, Kolkata, India*
**Introduction**
Chemotherapy outcomes are often hindered by the formidable challenges posed by chemoresistance and tumour relapse**.** Within this complex landscape, a particularly promising avenue for enhancing chemotherapy efficacy involves the strategic targeting of mutagenic translesion DNA synthesis (TLS) facilitated by DNA polymerase η (pol η) in ovarian cancer. Nevertheless, the task of identifying small molecule inhibitors to target pol η-mediated TLS with in vivo efficacy, remains a challenge.
**Material and Methods**
Virtual high throughput in silico screening of small molecules was performed using NuBBe, Sellechem, and Zinc databases. The circoss model was utilized to elucidate the networking dynamics among the identified compounds. Stable small molecules were further analysed for pol η binding potential. Molecular dynamics was conducted to understand the interaction strength using Schrödinger suite 2019. Pol η inhibitory potential of the identified small molecule was validated by reporter

© 2024 The Authors. Molecular Oncology is published by John Wiley & Sons Ltd on behalf of Federation of European Biochemical Societies strand displacement assay. Real-time PCR and western blot were performed to check pol η expression. Further, CD44+/ CD117+ analysis and live dead assay were carried out for cancer stem-like cells (CSCs) using flow cytometry. CSC sensitizing potential was studied on the human ovarian cancer xenograft mice model.

**Results and Discussions**

After the refinement process, a collection of 284 potent small molecules was considered. The small molecule chrysin displayed the lowest ΔG and highest binding efficiency with pol η. Chrysin sensitized ovarian CSCs to platinum treatment by effectively impeding pol η-mediated TLS. This inhibitory action of chrysin on pol η expression culminated in a pronounced reduction of platinum-induced CSC enrichments and enhancement of platinum-induced cell death, both in in vitro and in vivo conditions. Beyond its impact on chemo-sensitization, chrysin treatment exhibited a noteworthy reduction in both spontaneous and platinum-induced mutagenesis. Furthermore, pre-treatment of chrysin exerted a suppressive effect on tumour progression in the ovarian cancer pre-clinical xenograft model.

**Conclusion**

These findings highlight chrysin as a novel category of TLS inhibitors, showcasing its substantial potential as an adjuvant to overcome chemoresistance and enhance treatment efficacy in ovarian cancer patients.

### EACR2024-0131
### The effect of Moringa leaf extract on Cisplatin-Induced Apoptosis in Gastric Cancer Cells

*S. Al Bahlani[1], N. Al Abri[2], H. Al Toubi[3], J. Al Sabahi[4], A. Al Hashami[5], S. Al Jaaidi[6]*

*[1]Sultan Qaboos University, Biomedical Science, Muscat, Oman*

*[2]Sultan Qaboos University, Pathology, Al khoud, Oman*

*[3]Sultan Qaboos University, Biomedical Science, Al khoud, Oman*

*[4]Sultan Qaboos University, College of Agriculture and Marine Sciences, Al khoud, Oman*

*[5]Samail Sewage Treatment Plant, Plant treatment, Samail, Oman*

*[6]Sultan Qaboos University, Biology, Al khoud, Oman*

**Introduction**

Cisplatin (CDDP) is an effective anticancer drug that is used for treating Gastric Cancer (GC). CDDP acts by altering protein (p53) and other anti-apoptotic proteins leading to cells death by apoptosis; however, chemo-resistance and nephrotoxicity remain a big vital obstacle. Previous studies have reported that Moringa leaf extract has anticancer activity against several human cancer cells. Moringa leaf extract has a promising anticancer activity to overcome chemotherapy resistance. However, its effect on CDDP- induced apoptosis in GC has not been fully studied. This study aims to examine the effect of Moringa leaf extract on Cisplatin-induced apoptosis in GC by figuring out the implicit mechanisms of it is anticancer activity.

**Material and Methods**

Two GC cell lines (AGS & NCI-N8) were treated with different concentrations of Omani and Indian Moringa leaf extract (0, 200, 400, 800 μM) for 24 hours. Cell proliferation and apoptosis were assessed through both Alamar blue, and MTT assays and Hoechst stain respectively.

**Results and Discussions**

The LD50 of both extract were calculated to be 408 μg/ml for the Omani and Indian 372 μg/ml Moringa. Both Omani and Indian MO leaves extract significantly increase apoptosis and inhibit cell proliferation in AGS-GC cells in a concentration-dependent manner.

**Conclusion**

Altogether, the results suggests that Indian Moringa extract was more effective in inducing apoptosis and inhibiting cell proliferation of the AGS-GC cells than Omani one.

### EACR2024-0160
### TanCAR design for PDAC enhances antitumor effects and reduces antigen escape

*D. Camille[1,2,3], K. Vanderliek[1], D. Schäfer[1]*

*[1]Miltenyi Biotec B.V. & Co. KG, R&D Reagents, Bergisch Gladbach, Germany*

*[2]Max Planck Institute for Experimental Medicine, Translational Molecular Imaging, Göttingen, Germany*

*[3]University Medical Center Göttingen, Translational Molecular Imaging- Institute for Diagnostic and Interventional Radiology & Clinic for Hematology and Medical Oncology, Göttingen, Germany*

**Introduction**

Genetically engineered cellular products have become a newly established pillar of cancer therapy. While cellular therapies have been unprecedentedly successful against leukemia, they still lack efficacy to solid tumors. A major obstacle is the limited spectrum of T cell specificity in the face of tumour heterogeneity and potentially dynamic antigen escape variants, which can lead to tumour recurrence after initial treatment with CAR T cells directed to a single tumor antigen.

**Material and Methods**

Given the heterogeneous expression of antigens on many solid malignancies, we hypothesised that a bispecific CAR molecule with an OR-gated system (tandem CAR, TanCAR) can mitigate antigen escape and improve the antitumor activity of CAR T cells. To this end, we generated TanCARs varying in spacer length (short vs. extra short), myc-tag position (N- vs. C-terminal), and scFv combinations for three previously identified PDAC targets. We assessed CAR functionality based on transduction potential, tag-detection, cytotoxic potential, marker upregulation and cytokine release. CAR T cell were also co-cultured with cell lines reflecting target escape variants.

**Results and Discussions**

We observed as a general pattern, that tagged CARs were less expressed and had lower efficiencies as compared to non-tagged CARs, leading to the hypothesis that tags can interfere with proper CAR folding. This inspired us to develop detection reagents specific to our TanCARs, to enable robust CAR detection without the need for additional tags. These reagents facilitated fast and efficient verification of CAR presence, while not impeding CAR folding. Although, several constructs exhibited satisfactory cytotoxicity, we could not deduce a

© 2024 The Authors. Molecular Oncology is published by John Wiley & Sons Ltd on behalf of Federation of European Biochemical Societies clear correlation between TanCAR design and functionality.

**Conclusion**

In conclusion, this study demonstrates that TanCAR T cells specific for PDAC can enhance immunotherapeutic effects by reducing chances for tumor antigen escape and increasing T cell functionality, which can be extended to other target combinations and other solid tumor entities. Future studies should investigate if our results generated in vitro can be replicated in murine models. In addition, using CARs specific for two antigens, TanCARs come along with increased risk for on-target/off-tumor toxicities, which should be addressed carefully. If successful, TanCARs could be a valid option to remedy some of the current issues CAR T cell therapy is facing in the context of solid malignancies.

### EACR2024-0169
### Underscoring Menin's Role as a Strategy to Overcome Drug Resistance in Castration Resistant Prostate Cancer

*I. bulut*[1], *B. Cevatemre*[2], *H. Syed*[2], *C. Acilan Ayhan*[3]
[1]*Koc University, Graduate School of Health Sciences, Istanbul, Turkiye*
[2]*Koc University, Kuttam, Istanbul, Turkiye*
[3]*Koc University, Medicine Faculty, Istanbul, Turkiye*

**Introduction**

Prostate cancer (PC) typically relies on hormones for growth, with androgen deprivation therapy (ADT) being the primary treatment for advanced cases [1]. However, patients often advance to a castration-resistant (CR) stage, where tumor growth becomes unresponsive to androgen deprivation [2]. Despite the common use of conventional chemotherapeutics like Docetaxel (DTX) and Cabazitaxel (CBZ), either alone or in conjunction with hormone therapies, certain tumors may experience recurrence [3].

**Material and Methods**

Resistant castration-resistant prostate cancer (CRPC) cell lines were generated for our study, where we employed global proteomics and RNA sequencing techniques to compare these resistant cell lines with sensitive counterparts. Through a library drug screen, we identified epigenetic targets, which were subsequently knocked out using the CRISPR-Cas9 system. Additionally, RNA-sequencing and chromatin immunoprecipitation (ChIP) experiments were conducted to elucidate the crucial effects of these factors on the genome of resistant cells.

**Results and Discussions**

Our epigenetic screen revealed, MLL complex inhibitors MI-2, MI-3, MI-136, MI-463 (iMLL-Menin) synergized with both taxanes and significantly induced apoptosis when combined with the Taxol's. To determine the roles for individual proteins in the MLL complex, we knocked out MLL, and its known partners using CRISPR-Cas9 technology. The absence of Menin (an MLL partner) had no effect on the parental cells; however, it significantly halted the growth of the resistant cells. Parental cells required Menin expression for the acquisition of the drug resistance. Menin knockouts were analyzed through RNA-sequencing, and Menin target genes were identified via ChIP-qPCR. The expression of Myc and mTOR were halted in Menin absence, and mTOR inhibition via Torin further sensitized Menin knockout cells. Our chromatin pulldown also showed that Menin is highly enriched in Myc promoter in the resistant cells.

**Conclusion**

Our study sheds light on the intricate mechanisms underlying the development of resistance in CRPC. Our data demonstrate the indispensable role of Menin, in conferring resistance, and further exploration revealed the downstream effects of Menin deficiency, including the suppression of Myc and mTOR expression, which sensitized cells to mTOR inhibition. These findings offer promising avenues for the development of novel therapeutic strategies targeting epigenetic regulators to overcome drug resistance and improve outcomes for patients with CRPC.

### EACR2024-0204
### Functional expression of ABCB1, ABCC1 and ABCG2 efflux transporters influences the accumulation and efficacy of cytotoxic drugs in ex vivo lung tumor explants: role in combating multidrug resistance

*J. Hofman*[1], *Y. Budagaga*[1], *Y. Zhang*[1], *Z. Sabet*[1], *I. Hanke*[2], *T. Rozkoš*[3]
[1]*Charles University- Faculty of Pharmacy in Hradec Králové, Department of Pharmacology and Toxicology, Hradec Králové, Czech Republic*
[2]*University Hospital Hradec Králové, Department of Cardiosurgery, Hradec Králové, Czech Republic*
[3]*University Hospital Hradec Králové, The Fingerland Department of Pathology, Hradec Králové, Czech Republic*

**Introduction**

Lung cancer is the mortality leader within oncological diseases in both genders. Unfortunately, the clinical success of anticancer pharmacotherapies is frequently hindered by multidrug resistance (MDR), which leads to disease relapse. Beside others, this phenomenon is mediated by pharmacokinetic mechanisms, specifically by drug efflux via ATP-binding cassette (ABC) transporters. Although some expression studies in lung tumor tissues have been reported in the past, the functional activity of MDR-associated ABC transporters and their possible value as MDR-combating targets have not yet been explored in detail.

**Material and Methods**

Primary ex vivo explants were isolated from non-small cell lung (NSCLC) patients´ biopsies based on our protocol including fibroblast removal using magnetic beads. Expression and activity of examined transporters were quantified using western blotting and flow-cytometric accumulation studies, respectively. MDR-combating ability of tazemetostat, a potential dual-activity modulator (inhibiting ABC transporters in addition to its own targeted anticancer effect), were subsequently investigated in drug combination studies in explants with varying expression of ABC transporters.

**Results and Discussions**

ABCB1, ABCC1 and ABCG2 transporters' expression levels were detected by western blotting in 16 primary NSCLC explants, showing substantial interindividual

© 2024 The Authors. Molecular Oncology is published by John Wiley & Sons Ltd on behalf of Federation of European Biochemical Societies variability. Importantly, we found an association between expression levels of ABC transporters and the results of subsequent accumulation studies. In explants with low expression, model inhibitors caused insignificant changes in the accumulation of probe cytostatic substrates and vice versa. Interestingly, also outcomes of drug combinations were in relationship with expression/ accumulation studies in the set of 4 selected explants. Synergy between tazemetostat and doxorubicin or mitoxantrone were recorded in samples with high transporters´ expression/activity, whereas additivity or even antagonism were observed in sample with an opposite pattern.

**Conclusion**

In conclusion, we demonstrate that ABCB1, ABCC1 and ABCG2 may play a significant role in MDR to conventional chemotherapeutics in NSCLC patients. At the same time, they could represent a promising target for MDR combating via the novel dual-activity modulation approach.

*The study was supported by New Technologies for Translational Research in Pharmaceutical Sciences/NETPHARM, project ID CZ.02.01.01/00/22_008/0004607, co-funded by the European Union.*

### EACR2024-0213
### Unveiling the Enigma: Understanding and Overcoming Resistance to Immune Checkpoint Blockade and Immune-Based Therapies in Cancer Treatment

O. Bolaji[1], G. Bello[2]
[1]*Federal College of Animal Health and Production Technology, Animal Production and Health, Ibadan, Nigeria*
[2]*Institute of Agricultural Research and Training, Research and Training, Ibadan, Nigeria*

**Introduction**

Resistance to immune checkpoint blockade (ICB) and other immune-based therapies presents a significant challenge in cancer treatment. Despite initial success, many patients develop resistance, limiting the long-term effectiveness of these therapies. Understanding the mechanisms underlying resistance is essential for improving patient outcomes. This study aims to investigate the mechanisms of resistance to ICB and other immune-based therapies and explore potential strategies to overcome this resistance.

**Material and Methods**

A comprehensive approach was employed to study resistance mechanisms to ICB and other immune-based therapies. Clinical data from patients receiving these treatments were analyzed to identify patterns of resistance. Preclinical models, including cell culture and animal studies, were utilized to validate these findings and explore potential therapeutic interventions. Molecular and immunological assays were performed to elucidate the mechanisms underlying resistance, focusing on tumor microenvironment dynamics, immune cell function, and tumor immune evasion strategies.

**Results and Discussions**

It reveals several mechanisms of resistance to ICB and other immune-based therapies. These included tumor-intrinsic factors such as loss of antigen presentation, upregulation of alternative immune checkpoint pathways, and alterations in tumor mutational burden. Additionally, tumor-extrinsic factors such as the immunosuppressive tumor microenvironment, including regulatory T cells and myeloid-derived suppressor cells, contributed to therapy resistance. Furthermore, dynamic changes in the tumor-immune interface, such as immune escape variants and clonal evolution, were observed. Strategies to overcome resistance included combination therapies targeting multiple immune checkpoints, modulation of the tumor microenvironment, and strategies to enhance antigen presentation and T cell function.

**Conclusion**

The resistance to immune checkpoint blockade and other immune-based therapies is a complex and multifactorial process. Understanding the underlying mechanisms of resistance is crucial for developing effective strategies to overcome it. Combination therapies targeting multiple aspects of the immune response, along with strategies to modulate the tumor microenvironment, hold promise for improving the efficacy of immune-based therapies and enhancing patient outcomes in cancer treatment. Continued research efforts are needed to further elucidate these mechanisms and translate them into clinically effective interventions.

### EACR2024-0284
### Response of urothelial cancer cell lines and their cisplatin-resistant sublines to BET inhibitor PLX51107 in combination with different PARP inhibitors

J. Schmitz[1], A.L. Bartkowiak[1], M.J. Hoffmann[1], G. Niegisch[1]
[1]*Heinrich-Heine-University, Department of Urology-Medical Faculty and University Hospital- Centre for Integrated Oncology CIO Düsseldorf, Duesseldorf, Germany*

**Introduction**

Treatment resistance is a frequent problem in urothelial carcinoma (UC). PARP inhibitors (PARPi) targeting DNA repair are a potent treatment option for cancers with BRCA1/2 mutations (BRCAness), which only rarely occur in UC. Earlier we demonstrated that treatment of UC cells with the bromodomain and extra-terminal motif inhibitor (BETi) PLX51107, results in a pharmaceutically induced BRCAness phenotype. In this study, we thus investigated potential synergism between BETi PLX51107 and PARPi in UC cell lines (UCCs) and their cisplatin-resistant sublines (LTTs).

**Material and Methods**

Dose response curve analyses were performed with BETi PLX51107 (PLX) and PARPi Olaparib (Ola) and Talazoparib (Tala) for RT112, T24 and J82 as well as their LTTs after 72 hours of treatment. Benign HBLAK cells served as control. Combination treatment was analysed for synergism using the Chou Talalay method. Combinations with reduced dosages were used to further analyse effects on clonogenicity, cell cycle and apoptosis.

**Results and Discussions**

LTTs were more sensitive to PLX than their parental cell lines. For the PARPi, in all UCCs lower $IC_{50}$ for Tala than for Ola were observed. While benign HBLAK cells have similar $IC_{50}$ for PARPi as UCCs (Tala $IC_{50}$ T24: 0.2

© 2024 The Authors. Molecular Oncology is published by John Wiley & Sons Ltd on behalf of Federation of European Biochemical Societies

μM; T24-LTT: 0.4 μM; HBLAK: 0.3 μM), the BETi proved to be more toxic (PLX $IC_{50}$ T24: 1.3 μM; T24-LTT: 0.36 μM; HBLAK: 0.14 μM). Combined treatment with BETi and PARPi resulted in a strong synergism on all UCCs, especially when using Tala as combination partner, allowing a dose reduction for further analyses (0.25x - 0.5x $IC_{50}$ PLX; 0.75x $IC_{50}$ Ola and 0.5x $IC_{50}$ Tala). Treatment with reduced PLX dose had minor effect on the clonogenicity and the cell cycle on UCCs. Combined treatment with PLX and PARPi strongly reduced long-term proliferation capacity of the cells. PARPi caused a strong G2/M arrest in UCCs, which was only moderately enhanced by combination therapy. While the combination of PLX and Tala achieved an increased induction of apoptosis in all UCCs compared to the mono-treatments, this effect could not be observed with Ola. Reduced UCC Tala doses were better tolerated by benign HBLAK cells than reduced Ola doses.

**Conclusion**

PLX induced BRCAness results in strong synergism when combined with PARPi in UCCs and LTTs. Talazoparib proved to be the better PARPi for combination with PLX51107. Thus, we suggest combined treatment with PLX51107 and Talazoparib as a new highly potent treatment option for UC.

### EACR2024-0286
### DNA damage response pathway activation boots the transcription of endothelin A receptor contributing to a lower response to PARPi therapy

C. Roman[1], P. Tocci[2], R. Sestito[2], V. Di Castro[2], A. Bagnato[2]

*[1]Regina Elena National Cancer Institute, Preclinical Models and New Therapeutic Agents Unit, Roma, Italy*
*[2]Regina Elena National Cancer Institute, Preclinical Models and New Therapeutic Agents Unit, Rome, Italy*

**Introduction**

The use of PARP inhibitors (PARPi) has changed the treatment paradigm of high-grade serous ovarian cancer (HG-SOC), however, resistance to PARPi commonly develops through diverse mechanisms, limiting its impact in a high percentage of patients. In ovarian cancer, endothelin-A receptor ($ET_AR$) overexpression is associated with poor prognosis. It has been documented that the ET-1/$ET_AR$ axis activation, driving mutp53/YAP transcriptional alliance, fuels the escape from platinum-based therapy and PARPi, as olaparib, in HG-SOC. This data highlights the need to develop combinatorial therapeutic strategies to improve responses to PARPi.

**Material and Methods**

Patient-derived (PD) HG-SOC cells were analysed for DNA damage response at RNA and protein level, and promoter activity assay, using a combination of pharmacological treatments and loss-of-function approaches. *In vivo* therapeutic efficacy of drug combinations was evaluated in PD xenografts models.

**Results and Discussions**

To better characterize the regulation of $ET_AR$ expression during PARPi treatment, we documented that olaparib induced significant increase in $ET_AR$ expression in PD HG-SOC models. This increase, observed at the transcriptional level with an enhanced $ET_AR$ promoter activity, was DNA damage response (DDR)-dependent, as shown by the upregulation of ATR and ATM pathways. Indeed, ATR and ATM inhibitors blocked the olaparib-induced $ET_AR$ expression, as well as $ET_AR$ promoter activity, envisaging that olaparib may amplify the $ET_AR$ feed-forward loop that sustains PARPi tolerance. Similarly, UVB- and cisplatin-induced DNA damage favoured the induction of the ATR pathway, associated with $ET_AR$ upregulation. In silico analysis revealed an enriched expression of transcription factors with a predicted $ET_AR$ promoter binding site whose up-regulation was associated to DDR-related pathways. Among these, STAT3 activation, elicited by the induction of the DDR pathway upon olaparib treatment, triggered $ET_AR$ upregulation. Interestingly, the combination of macitentan, a dual ET-1R antagonist, with olaparib, further enhanced the activation of ATR/ATM pathways. In HG-SOC PD xenografts, macitentan synergized with olaparib inhibiting metastatic growth and favouring apoptosis and DNA damage.

**Conclusion**

These results demonstrate that DDR activation induces a positive loop by stimulating $ET_AR$ transcription. This mechanism of PARPi escape reveals $ET_AR$ as novel druggable vulnerability, which may be targeted with ET-1R therapeutics to enhance the efficacy of PARPi treatment.

### EACR2024-0296
### Suppression of epithelial-mesenchymal transition of olaparib resistant- High Grade Serous Ovarian Cancer cells by upregulating miR-188-5p

E.M. Aydın[1], İ. Durmaz Sahin[2]

*[1]Koc University, Research Center for Translational Medicine KUTTAM, Istanbul, Turkiye*
*[2]Koc University, School of Medicine, İstanbul, Turkiye*

**Introduction**

Tumor resistance to chemotherapy is one of the main challenges in cancer treatment. Epithelial–mesenchymal transition (EMT) is known to play an important role in cancer metastasis and drug resistance. High-grade serous ovarian cancer (HGSOC) is one of the most aggressive forms of ovarian cancer contributing to poor prognosis due to several factors, including tumor heterogeneity and the rapid development of resistance towards standard and second line chemotherapy with PARP inhibitors (olaparib, niraparib, etc.). miRNAs have clinical significance in the prognosis and treatment of many cancer types and changes in the expression of certain miRNAs have been demonstrated in HGSOC. In this study we aimed to investigate the reversal role of miR*188-5p* on cell migration and EMT in olaparib resistant-HGSOC.

**Material and Methods**

Differential gene expression level of *188-5p* in parental OVCAR-3 and olaparib resistant OVCAR-3 (OVCAR-3-OR) cells were determined by qRT-PCR assay. Lipofectamine was used for transfection of the mimic molecule of *188-5p. S*ynergy analysis was performed by SRB assay and combination index was calculated. Cell

Molecular Oncology 18 (Suppl. 1) (2024) 1–495
© 2024 The Authors. Molecular Oncology is published by John Wiley & Sons Ltd on behalf of Federation of European Biochemical Societies cycle changes and apoptosis were investigated by PI staining, Annexin V, and caspase 3/7 Muse experiment and western blotting. Wound healing assay was used for determining the cell migration. EMT marker protein levels were checked by western blot experiments. mRNA targets of *188-5p* were explored through miRBase and TargetScan databases.

**Results and Discussions**

*188-5p* gene expression level was found significantly downregulated in OVCAR-3-OR cells. Synergistic treatment of olaparib and *188-5p* mimic has decreased the cell survival rate of the resistant cells. Depend on cell cycle experiments, subG1 arrest was observed in resistant cells upon *188-5p* mimic transfection. Downstream effects of synergistic treatment were exhibited an increase in caspase-8 mediated extrinsic apoptosis level in resistant. By upregulating *188-5p* in OVCAR-3-OR cells, N-cadherin and SNAIL protein levels were decreased and EMT capacity in resistant was suppressed. In silico analysis showed that CD2AP, IL6ST and, FOXN2 genes were possible targets of *188-5p.*

**Conclusion**

miR*188-5p* was found significantly downregulated in resistant HGSOC and upregulation of *188-5p* gene has a significant role in reversal of HGSOC resistant. It was concluded that upregulation of miR*188-5p* suppress the cell migration and invasion abilities by facilitating epithelial to mesenchymal transition mechanism which has an important effect on cancer metastasis.

### EACR2024-0306
### Identification of pre-sensitizing agents to FLT3 inhibitors in acute myeloid leukemia with lineage tracing

*J. Eriksson[1], S. Zheng[1], J. Bao[1], J. Dai[1], W. Wang[1], A. Vähärautio[1], J. Tang[1]*

*[1]University of Helsinki, Research Program in Systems Oncology, Helsinki, Finland*

**Introduction**

Acute myeloid leukemia (AML) is a highly aggressive hematological malignancy. A key contributor to the poor prognosis of AML patients is the presence of the internal tandem duplication (ITD) mutation within the FLT3 tyrosine kinase gene, affecting roughly 20% of newly diagnosed cases. Although the introduction of newly approved FLT3 inhibitors have led to significant improvement in the treatment of FLT3-ITD-mutated AML, primary therapeutic resistance and short-lived responses are still a major problem. The aim of our study was to unravel mechanisms behind primed resistance against FLT3 inhibitors driven by intrinsic transcriptional heterogeneity, and to identify novel pre-sensitizing agents to FLT3 inhibitors.

**Material and Methods**

To uncover pre-existing resistant states against FLT3 inhibitors midostaurin and quizartinib in an FLT3-ITD-positive MOLM-13 AML cell line, we employed our recently published single-cell lineage-tracing method ReSisTrace that combines genetic barcoding with single-cell RNA sequencing and utilizes the analysis of shared transcriptomic features of sister cells. We then performed differential gene expression analyses between the pre-resistant and pre-sensitive cells to obtain pre-resistance signatures. Synergistic drugs were predicted and validated by targeting individual pre-resistance signature genes with small molecule inhibitors, as well as searching the L1000 database for drugs that would induce gene expression changes opposite to the pre-resistance signatures.

**Results and Discussions**

Targeting the pre-resistance signature gene G1 to S phase transition 1 (GSPT1) with a selective cereblon E3 ligase modulating drug CC-90009 or enolase 1 (ENO1) with ENOblock showed synergy with the FLT3 inhibitors in MOLM-13 and MV4-11 FLT3-ITD-mutated cell lines. Furthermore, vistusertib (mTOR inhibitor), linsitinib (IGF1R and insulin receptor inhibitor), and meisoindigo (IGF1R and Src family kinase inhibitor), all inhibiting pathways parallel or downstream of oncogenic FLT3 signaling, were predicted and validated to pre-sensitize AML cells to the FLT3 inhibitors.

**Conclusion**

Primed resistance against FLT3 inhibitors may be mediated by activation of signaling pathways downstream or parallel to oncogenic FLT3 signaling, and inhibition of these pathways sensitizes cells to FLT3 inhibitors. Furthermore, CC-90009 and ENOblock could potentially be used to increase efficacy to FLT3 inhibitors in FLT3-ITD-positive AML.

### EACR2024-0316
### Sensitizing ovarian cancers to PARP inhibitors: efficacy of the HDAC inhibitor belinostat

*S. Guillemot[1], C. Thomine[1], L. Thorel[1,2], R. Florent[3], E. Abeilard[1,2], E. Brotin[4], E. Dolivet[1,2,5], L.B. Weiswald[1,2,3], L. Poulain[1,2,3,6,7], M. Villedieu[1]*

*[1]Université de Caen Normandie- INSERM- ANTICIPE U1086- Interdisciplinary Research Unit for Cancer Prevention and Treatment, Comprehensive Cancer Center F. Baclesse, Caen, France*

*[2]UNICANCER, Comprehensive Cancer Center F. Baclesse, Caen, France*

*[3]Université de Caen Normandie- Services Unit PLATON "support platforms for preclinical and translational research in oncology", ORGAPRED core facility, Caen, France*

*[4]Université de Caen Normandie- Services Unit PLATON "support platforms for preclinical and translational research in oncology", ImpedanCELL, Caen, France*

*[5]UNICANCER- Comprehensive Cancer Center F. Baclesse, Department of Surgery, Caen, France*

*[6]Université de Caen Normandie- Services Unit PLATON "support platforms for preclinical and translational research in oncology", Biological Ressources Center "OvaRessources", Caen, France*

*[7]UNICANCER- Comprehensive Cancer Center F. Baclesse, Biological Ressources Center "OvaRessources", Caen, France*

**Introduction**

The clinical management of ovarian cancer, a pathology with a poor prognosis, has recently been improved with the introduction of PARP inhibitors (PARPi), such as olaparib, into treatment protocols. Inhibition of PARP by these molecules leads to the accumulation of DNA double-strand breaks, which are normally repaired by the

© 2024 The Authors. Molecular Oncology is published by John Wiley & Sons Ltd on behalf of Federation of European Biochemical Societies homologous recombination (HR) pathway. When this HR is deficient, the cells are unable to repair the PARPi-induced damages and die by a mechanism of "synthetic lethality". PARPi are therefore indicated for HR-deficient tumours. However, in more than half of the patients, tumours have a functional HR pathway (HRP phenotype) and do not respond to this targeted therapy. Identifying PARPi sensitization strategies thus constitutes a major challenge for these patients. HDAC inhibitors (HDACi), which modulate gene expression, have been described as capable of disrupting the HR pathway by indirectly silencing some of its effectors. In this context, our objective was to evaluate the interest of belinostat, an FDA-approved pan-HDAC inhibitor, to sensitize HRP ovarian cancer cells to olaparib.

**Material and Methods**

The efficacy of the belinostat/olaparib combination was investigated using various techniques assessing cell viability, apoptosis and DNA damage in two types of ovarian cancer preclinical models: the SKOV3 cell line and different HRP tumoroid models, which are three-dimensional models derived from patients' tumours and representative of them.

**Results and Discussions**

We first showed that belinostat efficiently sensitized SKOV3 cells to olaparib. We then validated this result in around half of our tumoroid models, in which the belinostat/olaparib combination significantly reduced viability as compared to each molecule used alone. Our results also showed that this decreased viability was associated with apoptosis induction. This study is the first to highlight the efficacy of belinostat/olaparib treatment in ovarian tumoroids, suggesting the potential clinical relevance of this association. Interestingly, this combination induced more DNA damage than olaparib in the sensitive models, but not in the resistant ones. This suggests that the sensitizing effect of belinostat may be based on its ability to inhibit the HR pathway, which needs to be further investigated.

**Conclusion**

This study therefore allows us to propose the belinostat/olaparib combination as an alternative option to olaparib treatment for HRP ovarian cancer, with the predictive markers for response still to be identified.

### EACR2024-0323

### R-loop mediated epigenetic regulation and treatment resistance in Head and Neck Squamous Cell Carcinoma (HNSCC)

*H. Crane[1], K. Hunter[2], S. El-Khamisy[3]*

*[1]University of Sheffield, School of Clinical Dentistry, Sheffield, United Kingdom*

*[2]University of Liverpool, Liverpool Head and Neck Centre, Liverpool, United Kingdom*

*[3]University of Sheffield, School of Biosciences, Sheffield, United Kingdom*

**Introduction**

Head and Neck Cancer is the 8$^{th}$ most common cancer in the UK and the majority of these cancers are Head and Neck Squamous Cell Carcinoma (HNSCC). A site-defined subset of HNSCC occurs in the base of tongue, soft palate and tonsils, known as Oropharyngeal Squamous Cell Carcinoma (OPSCC). OPSCC has two distinct clinical subtypes, one associated with high-risk Human Papilloma Virus (HPV-associated/HPV+) and one associated with the traditional head and neck cancer risk factors, typically smoking and alcohol (HPV-independent/HPV-). HPV- OPSCC has a dismal 3-year survival and although HPV+ OPSCC typically has an improved response to therapy, a subset of these recur or present with distant metastases, including to bone. Genomic instability is one of the hallmarks of cancer, with many tumours possessing defects in DNA repair which lead to advantageous mutations and cellular survival. R-loops are epigenetic three-stranded DNA:RNA hybrids which occupy up to 10% of the genome and are known to modulate genome dynamics as well as being a potential source of genomic instability.

**Material and Methods**

R-loop levels were explored in tissue using S9.6 immunohistochemistry on a cohort of OPSCC with unfavourable clinical outcomes, including metastases to soft tissue and bone. In vitro assays were used to modulate R-loop levels in a cisplatin resistant HPV+ and HPV- cell line. siRNA was utilised to deplete USP11 and senataxin (R-loop resolving enzymes) and the effects on cell viability and DNA damage were assessed using MTS assays and immunofluorescence for Gamma-H2AX.

**Results and Discussions**

S9.6 immunohistochemistry revealed that HPV+ tumours had a higher burden of R-loops when compared to HPV- tumours. Additionally, bone metastases had a higher R-loop level when compared to primary tumours or soft tissue metastases. There was no difference in S9.6 expression by tumour stage, nodal stage, smoking status or alcohol consumption. In resistant cell lines, senataxin depletion reduced cell viability and increased DNA damage following cisplatin treatment through an R-loop mediated mechanism. In HPV- cells, USP11 depletion led to decreased senataxin expression, increased DNA damage and reduced cell viability in response to cisplatin.

**Conclusion**

In a cohort of OPSCC tumours, bone metastases had a higher R-loop burden, and in vitro assays highlighted the potential of USP11 to modulate sensitivity to cisplatin in HPV- tumours. R-loops may represent a potential therapeutic target and deserve further investigation.

### EACR2024-0335

### Crosslinking of Ly6a metabolically reprogram CD8 T cells for cancer immunotherapy

*A. maliach[1], Y. Carmi[2], C. levy[3]*

*[1]Tel Aviv university, Genetics, hadera, Israel*

*[2]Tel Aviv uneversity, Department of Pathology, Tel Aviv, Israel*

*[3]Tel Aviv university, Genetics, Tel Aviv, Israel*

**Introduction**

T-cell inhibitory mechanisms prevent autoimmune reactions. While cancer immunotherapy aims to remove these inhibitory signals, chronic UVB exposure attenuates autoimmunity through promotion of unknown immune suppression mechanisms. Although immune checkpoint inhibitors have shown remarkable efficacy as a therapeutic intervention in melanoma, 50% of patients

© 2024 The Authors. Molecular Oncology is published by John Wiley & Sons Ltd on behalf of Federation of European Biochemical Societies are treatment-resistant . We reasoned that targeting factors, such as UVB exposre, that inhibit autoimmunity as well as the immune response to cancer could serve as a platform for investigation the regulation of the immune response to cancer leading to the development of new treatments for cancer.

**Material and Methods**

We investigate the impact of chronic UVB exposure on tumor progression using in vivo models of tumor and metastatic growth. Through the application of mass cytometry, single-cell RNA sequencing, and flow cytometry, we identified Ly6a as a marker for UVB-immunosuppressed T cells. Proteomic analyses and additional molecular biology techniques were employed to explore the downstream effects of anti-Ly6a antibodies.

**Results and Discussions**

We here show that mice with subcutaneous melanomas were unresponsive to checkpoint blockade therapy following chronic UV irradiation. This no responsiveness was due to the suppression of skin-draining lymph node T-cells' killing ability. Using mass cytometry analysis and single cell RNA sequncing, we uncovered a skin-specific UV-induced suppression of T-cells marked by upregulation of Ly6a. Out of the UV context, we found enrichment of type-1 interferon exposed Ly6a$^{high}$ T-cells in the tumor microenvironment and demonstrate that Ly6a crosslinking enhances T-cell anti-tumoral cytotoxic activity and reprograms their mitochondrial metabolism. Remarkably, in vivo treatment with anti-Ly6a antibody significantly inhibited tumor growth in mice resistant to anti-PD1 therapy.

**Conclusion**

We discovered that UVB induced expression of Ly6a in T cells. Ly6a expression was also increased upon chronic exposure to type 1 interferon, which accelerates the exhaustion of tumor-infiltrating T cells. Moreover, antibodies against Ly6a enhanced the T cell response to tumor cells in vitro and in vivo. Taken together, our data indicate that targeting Ly6a should enhance the immune response to cancer.

### EACR2024-0341

### Sterol-like drugs potentiate statin-triggered prostate cancer cell death by inhibiting SREBP2 nuclear translocation

*D. Zipinotti dos Santos[1], M. Elbaz[1,2], E. Branchard[1], W. Schormann[3], C. Brown[4], A. Meek[4], R. Hamilton[5], M. Reed[4,6,7], D. Andrews[3,8,9], L. Penn[1,8]*

*[1]Princess Margaret Cancer Centre, University Health Network, Toronto, Canada*

*[2]Helwan University, Department of Pharmacology and Toxicology, Cairo, Egypt*

*[3]Sunnybrook Research Institute, Biological Sciences, Toronto, Canada*

*[4]Krembil Research Institute, University Health Network, Toronto, Canada*

*[5]Princess Margaret Cancer Centre, Department of Surgical Oncology, Toronto, Canada*

*[6]University of Toronto, Department of Pharmacology and Toxicology, Toronto, Canada*

*[7]University of Toronto, Department of Chemistry, Toronto, Canada*

*[8]University of Toronto, Department of Medical Biophysics, Toronto, Canada*

*[9]University of Toronto, Department of Biochemistry, Toronto, Canada*

**Introduction**

There is an urgent need to provide immediate and effective options for the treatment of prostate cancer (PCa) to prevent progression to lethal castration-resistant PCa (CRPC). The mevalonate (MVA) pathway is dysregulated in PCa, and statin drugs commonly prescribed for hypercholesterolemia, effectively target this pathway. Statins exhibit anti-PCa activity, however the resulting intracellular depletion of cholesterol triggers a feedback loop that restores MVA pathway activity, thus diminishing statin efficacy and contributing to resistance.

**Material and Methods**

To address the challenge of statin resistance, we conducted a high-content image-based screen of a 1508 drug library enriched for FDA-approved compounds. The screen aimed to identify drugs capable of blocking the feedback response induced by statin treatment and enhancing their pro-apoptotic activity. Cell-based assays were employed to validate hits and investigate their mechanisms of action. Molecular modeling techniques were utilized to assess the structural similarity of identified compounds to 25-hydroxy-cholesterol (25HC) and predict their binding to a known protein-binding site of 25HC.

**Results and Discussions**

Two compounds, Galeterone (GAL) and Quinestrol, emerged as validated hits from the drug screen. These compounds share a cholesterol-related tetracyclic structure reminiscent of the FDA-approved CRPC drug Abiraterone (ABI). Molecular modeling revealed that GAL, Quinestrol, and ABI exhibit structural similarity to 25HC and are predicted to bind similarly to a protein-binding site of 25HC. Moreover, cell-based assays demonstrated that these compounds inhibit nuclear translocation of sterol-regulatory element binding protein 2 (SREBP2) and transcription of MVA genes, thus blocking the statin-induced feedback response. Importantly, sensitivity to these compounds was independent of androgen status. Furthermore, the combination of Fluva and GAL significantly impeded CRPC tumor xenograft growth, indicating their potential as a potent therapeutic strategy against CRPC progression.

**Conclusion**

By identifying cholesterol-mimetic drugs that inhibit SREBP2 activation upon statin treatment, we provide a potent "one-two punch" against CRPC progression and pave the way for innovative therapeutic strategies to combat additional diseases whose etiology is associated with SREBP2 dysregulation.

### EACR2024-0361

### Acetylome analysis of aspirin treated AML cells reveals potentially cytostatic modifications in cytosolic and mitochondrial proteins

© 2024 The Authors. Molecular Oncology is published by John Wiley & Sons Ltd on behalf of Federation of European Biochemical Societies

L. Higgins[1], T. Shields[1], V. Rajeeve[1], P.R. Cutillas[1]
[1]Barts Cancer Institute- Queen Mary University of London, Genomics and Computational Biology, London, United Kingdom

**Introduction**

Cell signalling circuitries are key determinants of drug responses in Acute Myeloid Leukaemia (AML), a genetically diverse cancer where genetic stratification has proven insufficient to rationalise drug response mechanisms. Proteomic characterisation of drug response phenotypes has the potential to reveal opportunities for repurposing current medicines in AML. Aspirin, a widely used and well tolerated drug, has the potential to modify cell signalling, making it an interesting candidate for inducing therapeutic vulnerability. To test this notion, we characterized how aspirin impacts the proteomes, acetylomes and cell viability of Kasumi-1, P31-Fuj, HL-60 and NB-4 AML cell lines.

**Material and Methods**

Cells were treated with aspirin over 3-days at 8 different concentrations (for viability screens) or at 1mM or 3mM (for proteomics). To account for aspirin's influence on pH, we included a pH adjusted vehicle (DMSO + HCL) control. After treatment, cellular proteomes were profiled with label-free mass spectrometry. Differential abundance of proteins and acetylated peptides was determined using the limma R package, a Fishers Exact test for ontology enrichment analysis, and Benjamini-Hochberg method for p-value corrections.

**Results and Discussions**

Three-day viability testing revealed reasonable cell line tolerance to aspirin, with evidence of a cytostatic effect at 3 mM (20-30% across all cell lines). To assess aspirin's impact on cell signalling, proteomics was undertaken following 2 consecutive treatments (5 days total), revealing modest changes. Given that aspirin's biological effects are associated with acetylation, we specifically analysed acetylated peptide abundance. This revealed more insightful changes with 38, 84, 57 and 99 serine acetylations significantly more abundant in aspirin treated HL-60, Kasumi-1, NB-4 and P31-Fuj cell lines, respectively, relative to pH adjusted controls ($q < 0.05$). Overrepresentation analysis revealed enrichment ($q < 0.05$) of proteins involved in glycolysis and MTORC1. ANOVA analysis of aspirin dose across cell lines on peptides revealed increased acetylation of GAPDH ($F = 57.49$, $q = 0.002$), ENO1 ($F = 100.72$, $q = 0.0003$) and the mitochondrial channel SLC25A5 ($F = 38.44$, $q = 0.009$).

**Conclusion**

Our findings suggest aspirin's primary actions in AML cell lines manifest as increased cytosolic and mitochondrial protein acetylations with potentially metabolic consequences. This could explain the cytostatic impact observed at 3mM and could be exploited therapeutically.

### EACR2024-0381
### Gasdermin B (GSDMB) Amplification in HER2+ Tumors: Insights from Zebrafish Xenografts

M. Santos[1], C. Ramos[2], S. Morales[2], D. Sarrió[2], L. Sánchez[1], G. Moreno-Bueno[2]
[1]Universidade de Santiago de Compostela, Zoology-Genetics and Physical Anthropology, Lugo, Spain
[2]Instituto de Investigaciones Biomédicas "Alberto Sols" CSIC-UAM, Biochemistry, Madrid, Spain

**Introduction**

*HER2/ErbB2*, a receptor involved in cell growth, is a promising target for cancer therapy. Its potential is particularly encouraging in cancers with HER2 overexpression mainly due to gene amplification like breast and gastric tumors, among others. Despite the success of HER2-targeted therapies, some tumors develop resistance. *Gasdermin B* (*GSDMB*), chromosomally located close to *HER2*, has emerged as a key contributor to this resistance in HER2-positive (HER2+) tumors. Notably, *GSDMB* overexpression has been frequently observed in patients with HER2+ cancers associated with poor clinical outcome.

**Material and Methods**

To validate the GSDMB-conferred resistance, we utilized a zebrafish xenotransplantation model. Zebrafish embryos at 48 hours post-fertilization were injected in the duct of Cuvier with HCC1954 cells in which GSDMB is targeted with a short hairpin RNA (HCC1954 sh794) or non-targeting control cells (HCC1954 shNTC), both lines expressing GFP. Treatment with lapatinib (2 μM) was administered 24 hours post-injection, and tumor growth was assessed over 72 hours.

**Results and Discussions**

In HCC1954 shNTC-injected embryos, no significant difference in tumor growth was observed between treated and control fish ($p>0.05$). However, in HCC1954 sh794-injected embryos, a significant difference was noted ($p<0.001$), indicating that GSDMB confers resistance to therapy. These findings underscore the importance of GSDMB as a potential therapeutic target and highlight its role in mediating resistance to anti-HER2 therapies.

**Conclusion**

Our study demonstrates that *GSDMB* overexpression contributes to resistance against anti-HER2 therapy in HER2+ tumors. Using the zebrafish xenotransplantation model, we provide compelling evidence supporting GSDMB as a promising target for overcoming therapy resistance in HER2+ breast cancer. The unique ability of the zebrafish model enables the evaluation of different stages of the tumorigenic process in vivo, further strengthening the translational relevance of our findings. However, further research is needed to elucidate the underlying mechanisms driving GSDMB-mediated resistance and to explore novel treatment strategies aimed at enhancing therapeutic outcomes in HER2+ tumors.

### EACR2024-0383
### The Role of Homologous Recombination Repair in Cisplatin-Resistant Bladder Cancer Cell Lines

N.C.H. van Creij[1], F.J. Roa[1], F.R. Santer[1], P.S. Holm[2], Z. Culig[1], R. Pichler[1]
[1]Medical University of Innsbruck, Urology, Innsbruck, Austria
[2]Medical University of Innsbruck, Oral and Maxillofacial Surgery, Innsbruck, Austria

**Introduction**

© 2024 The Authors. Molecular Oncology is published by John Wiley & Sons Ltd on behalf of Federation of European Biochemical Societies

Neoadjuvant cisplatin-based chemotherapy (NAC) followed by radical cystectomy is still the golden standard for localized and locally advanced muscle invasive bladder cancer (MIBC). However, around 65% of the patients do not respond to the treatment, mainly caused by resistance to the platinum-based chemotherapy. Our group has found a link between HUS1, a key protein in homologous recombination repair (HRR), and the resistance to NAC. Other studies also highlight the importance of the HRR pathway, including RAD51 and CHK1, in platinum-based chemotherapy resistance, but the mechanisms in MIBC have not yet been explored in large detail. The aim of this study is to investigate the role of HRR in cisplatin-based chemotherapy resistant bladder cancer.

**Material and Methods**

The MIBC cell lines UMUC3 and HT1197 were used and cisplatin-resistant (CR) cell lines were generated by exposure to increasing concentrations of cisplatin. A DRGFP assay was developed to measure HRR in the cell lines. Protein expression levels were evaluated using western blot (WB). HUS1 and RAD51 were silenced or overexpressed using transient transfection. Cell proliferation was analysed by measuring confluence in an Incucyte® imager.

**Results and Discussions**

Our DRGFP assay showed increased HRR in the CR cell lines, compared to the parental cell lines. We also observed higher basal RAD51 and CHK1 protein levels in the CR cells, again indicating increased HRR. Furthermore, the elevated RAD51 and CHK1 protein levels represent potential new biomarkers that predict patient response to cisplatin. Proliferation was decreased in the parental cells but not in the CR cells, when silencing HUS1 in combination with cisplatin treatment. Interestingly, proliferation was decreased in both parental and CR cells when silencing RAD51 in combination with cisplatin treatment. Targeting RAD51 in combination with platinum-based chemotherapy might be a promising new therapy strategy in overcoming platinum-based chemotherapy resistance. Lastly, WB analysis showed decreased HUS1 levels when RAD51 was silenced, and the opposite when RAD51 was overexpressed, suggesting a regulatory mechanism for these two proteins.

**Conclusion**

Our study showed for the first time increased HRR in cisplatin-resistant bladder cancer cells, and takes the first steps towards unravelling the role of HRR in platinum-based chemotherapy resistance. Besides this, it opens the field towards new possible biomarkers predicting drug response and new treatment options for bladder cancer patients.

### EACR2024-0385

### Tumor-Associated Macrophages Trigger Synergy of SMAC Mimetics with Chemotherapy in Ovarian

*R. Coelho[1], B. Seashore-Ludlow[2], S. Schütz[1], C.F. Lombardo[1], A. Fedier[1], O. Kallioniemi[2], L. David[3], V. Heinzelmann-Schwarz[1], F. Jacob[1]*

*[1]University Hospital Basel and University of Basel, Department of Biomedicine, Basel, Switzerland*

*[2]Karolinska Institute, Department Oncology and Pathology, Solna, Sweden*

*[3]Institute of Molecular Pathology and Immunology of the University of Porto IPATIMUP, Institute of Molecular Pathology and Immunology of the University of Porto IPATIMUP, Porto, Portugal*

**Introduction**

Resistance to chemotherapy and PARP inhibitors presents a significant challenge in treating high-grade serous ovarian cancer (HGSOC), primarily attributable to impaired apoptotic pathways in malignant cells. To address this challenge, we investigate the potential of Second Mitochondria-derived Activator of Caspases (SMAC) mimetics to counter apoptosis resistance when combined with chemotherapy, focusing on identifying mechanisms of action and predictive biomarkers for drug response.

**Material and Methods**

We conducted drug screening assays with single treatments and combinations (n=6) to determine synergies in ovarian cancer cells. Promising combinations were validated using patient-derived ex vivo cultures and in vivo experiments. Mechanistic insights and predictive biomarkers for drug response were elucidated using multi-omic data from chemo naive and neoadjuvant treated HGSOC samples (n=104) across two independent cohorts.

**Results and Discussions**

Our dose-response matrix revealed a compelling synergistic effect of SMAC mimetics in combination with either carboplatin or paclitaxel (bimodal treatment). This synergy was further validated in ex vivo patient-derived cultures and zebrafish embryo tumor xenograft models. Mechanistically, the combination of SMAC mimetics and chemotherapy triggered the activation of a Caspase 8-dependent apoptotic program mediated by TNFa signaling. Multimodal analysis of chemo naive and neoadjuvant-treated samples revealed an association between M2-like macrophages and activation of TNFa-related pathways.

**Conclusion**

Our proposed bimodal treatment approach holds promise for enhancing the clinical treatment response in HGSOC patients by combining SMAC mimetics with conventional chemotherapy. Furthermore, our data suggest that M2-like macrophages may serve as potential predictors for treatment response.

### EACR2024-0391

### Adaptor protein CIN85 modulates drug resistance of human osteosarcoma cells

*I. Horak[1,2], L. Drobot[2], L. Knopfová[1]*

*[1]Masaryk University, Department of Experimental Biology, Brno, Czech Republic*

*[2]Palladin Institute of Biochemistry- National Academy of Sciences of Ukraine, Department of Cell Signaling, Kyiv, Ukraine*

**Introduction**

Adaptor protein CIN85 is involved in multiple signaling cascades, including EGFR, HGFR, and PI3K, and regulates endocytosis, cell growth, adhesiveness, and motility. Previously, CIN85 was identified as one of the

© 2024 The Authors. Molecular Oncology is published by John Wiley & Sons Ltd on behalf of Federation of European Biochemical Societies proteins overexpressed in doxorubicin (DOX)-resistant osteosarcoma (OS) cells. This study aimed to investigate the impact of CIN85 on the survival and drug resistance of OS cells.
**Material and Methods**
Parental HOS and SAOS-2 cell lines and DOX-resistant derivatives with CIN85 knockdown or overexpression were used. Cell survival under treatment with DOX or CDDP (cisplatin) was evaluated by MTT assay and Sytox green exclusion assay. PARP content was estimated by Western blotting. Gene expression data were obtained from the GSE154540 dataset. Statistical analysis was performed using Student's t-test or ANOVA.
**Results and Discussions**
CIN85 silencing in DOX-resistant HOS and SAOS-2 cells resulted in an increased percentage of Sytox green-positive (dead) cells upon DOX treatment thus leading to the elevated sensitivity to DOX. Parental HOS cells with CIN85 knockdown were characterized by increased sensitivity to DOX, and especially to CDDP treatment. Vice versa, CIN85-overexpressing HOS cells were less sensitive to both DOX and CDDP by 30 %. CIN85-silenced SAOS-2 cells demonstrated elevated sensitivity to both DOX and CDDP by 35 % and 55 %, respectively, and CIN85 overexpression resulted in a reduction of the percentage of Sytox green-positive cells after DOX and CDDP treatments by 30 %. In addition, increased content of cleaved PARP was observed in CIN85-silenced cells. RNA sequencing of cell variants with CIN85 over-expression/knockdown will help to unravel the molecular mechanism of CIN85-dependent chemoresistance. These findings were complemented with the analysis of CIN85 expression in OS samples of patients with good versus poor response to chemotherapy, which found that CIN85 expression is slightly higher in the cohort of poor-responders.
**Conclusion**
Taken together, the obtained results indicate the substantial role of adaptor protein CIN85 in the acquisition of chemoresistance by osteosarcoma cells.
*This project has received funding through the MSCA4Ukraine project, which is funded by the European Union. Views and opinions expressed are however those of the author(s) only and do not necessarily reflect those of the European Union. Neither the European Union nor the MSCA4Ukraine Consortium as a whole nor any individual member institutions of the MSCA4Ukraine Consortium can be held responsible for them.*

### EACR2024-0402
### CDK7 and MITF repress a transcription program involved in survival and drug tolerance in melanoma

*L. Seno[1], P. Berico[1], M. Cigrang[1], C. Capelli[1], F. Coin[1], I. Davidson[1]*
*[1]IGBMC, Functional Genomics and Cancer, Illkirch-Graffenstaden, France*
**Introduction**
Intratumoral heterogeneity and phenotype switching allow melanoma cells to acquire therapy-resisting abilities. While naive tumors are comprised in large part of melanocytic-type melanoma cells displaying high expression of the transcription factor MITF, MAP kinase inhibitor (MAPKi) and immune checkpoint (ICI) treatments lead to emergence of resistant cell states, notably the undifferentiated/mesenchymal state. In many cancers, CDK7 is overexpressed and its inhibition represses super-enhancer-associated oncogenes. In melanoma cells, we observed that acquired resistance to the CDK7-inhibitor (CDK7i) THZ1 correlated with melanoma cell epithelial–mesenchymal transition (EMT) and acquisition of MAPKi tolerance. One of the potential actors in this resistance is transcription factor GATA6 that drives a drug resistance gene expression program.
**Material and Methods**
Cell survival was assessed with IC50 assays. CDK7i-resistant and MAPKi-resistant cell lines were generated to compare their transcriptional signatures with mesenchymal-like melanoma cells by bulk RNA-seq. Results were confirmed by RT-qPCR and immunoblots.
**Results and Discussions**
RNA-seq analysis showed that CDK7i promotes melanoma cell EMT and revealed a GATA6-dependent transcription program in CDK7i-resistant melanoma cells. GATA6 up-regulates the ABCG2 transporter in mesenchymal-like melanoma cells, involved in tolerance to CDK7i and MAPKi. Reanalysis of scRNA-seq data from PDX tumors before and after MAPKi treatment further showed that GATA6 was upregulated in the "interferon (IFN)-active" phenotype cells during the drug-resistant and relapse phases. To mimic this cell state, we used intermediary state melanoma cells where IFNγ treatment stimulated GATA6 expression as well as the IFN response pathway and their EMT. In agreement with a role for GATA6 in drug resistance phenotype 'IFN-active cells', we show that IFNγ induced drug resistance required GATA6 expression.
**Conclusion**
This study revealed that CDK7i resistance induces EMT and GATA6 expression that in turn activates a drug resistance program. This is further consolidated in a model of IFN-induced EMT and drug resistance that reveals an essential role for GATA6 in melanoma multidrug resistance.

### EACR2024-0408
### circMETTL9 induces Apatinib resistance in gastric cancer via KRT16/NF-κB pathway

*L. Ma[1], H. Zhou[1], M. shou[1], Y. liu[1], Y. shu[1]*
*[1]The First Affiliated Hospital of Nanjing Medical University, Department of Oncology, NanJing, China*
**Introduction**
Gastric cancer is the fifth highest number of incidence and the fourth of deaths globally now. Anti-angiogenic therapy such as Apatinib is one of the most common treatments, but many patients are susceptible to drug resistance. circRNA has been reported to play significant roles in reversing drug resistance. However, the mechanism of Apatinib resistance in gastric cancer are still unclear.
**Material and Methods**
We used cell derived xenograft (CDX) to establish a drug resistance model and sequenced the tumours. We then identified circMETTL9, which was highly expressed in the resistance model. Cck-8, flow cytometry, transwell, clone formation assay and CDX were used to investigate the role of circMETTL9. Moreover, mass spectrometry,

© 2024 The Authors. Molecular Oncology is published by John Wiley & Sons Ltd on behalf of Federation of European Biochemical Societies

RNA pulldown, RIP and WB assays explored the potential molecular mechanisms.

**Results and Discussions**

We identified circMETTL9 as a cyclic structure with strong stability and noticed that circMETTL9 was highly expressed in gastric cancer Apatinib-resistant cells. CDX and cck-8 assay showed circMETTL9 enhanced the proliferation of cells. Clone formation assay showed that the clone ability of cells was enhanced after upregulation of circMETTL9. Meanwhile, flow cytometry detected the apoptosis ability and the results showed that circMETTL9 enhanced cell apoptosis. Transwell assay and cell migration xenograft showed circMETTL9 enhanced cell invasion and migration ability. Then we tested the effect of circMETTL9 on drug-resistant cells and found that circMETTL9 modulated the sensitivity of drug-resistant cells to anti-angiogenic therapy. Moreover, knockdown of circMETTL9 inhibited the clone formation ability of gastric cancer drug-resistant cells and enhanced the apoptosis rate. Next, we constructed overexpression plasmid of circMETTL9 containing MS2 tag for RNA pull-down assay, and then used mass spectrometry to find the binding protein KRT16 of circMETTL9. RIP assay was used to validate the binding of KRT16 and circMETTL9. GSEA revealed that KRT16 could regulate the NF-κB pathway. Then, WB was used to verify that circMETTL9 regulates cell proliferation, apoptosis, and invasion ability by targeting the KRT16/NF-κB pathway. We also found inhibition of the circMETTL9/KRT16/NF-κB pathway reversed the resistance to anti-angiogenic therapy in gastric cancer.

**Conclusion**

circMETTL9 overexpression induces Apatinib resistance in gastric cancer. Moreover, inhibition of circMETTL9/ KRT16/NF-κB pathway can reverse Apatinib resistance of gastric cancer.

### EACR2024-0411

### The transcription factors NFATc1 and NFATc2 control glucocorticoid resistance in pediatric T-cell acute lymphoblastic leukemia

*G. Veltri[1], A. Peloso[2], A. Cani[2], E. Mariotto[2], D. Corallo[3], E. Iorio[4], B. Buldini[2], P. Ntziachristos[5], S. Bresolin[6], V. Serafin[7]*

*[1]Pediatric Research Institute- Città Della Speranza, Phosphoproteomics for ALL diagnostics and research group, Padova, Italy*

*[2]University of Padova, Division of Pediatric Hematology- Oncology and Stem Cell Transplant- Maternal and Child Health Department, Padova, Italy*

*[3]Pediatric Research Institute Foundation Città della Speranza, Laboratory of Target Discovery and Biology of Neuroblastoma, Padova, Italy*

*[4]Istituto Superiore Di Sanità- Roma, High Resolution NMR Unit Core Facilities, Roma, Italy*

*[5]University of Ghent, Department of Biomolecular Medicine, Ghent, Belgium*

*[6]Pediatric Research Institute Foundation Città della Speranza, Gene expression profiling and next generation sequencing in JMML and B ALL, Padova, Italy*

*[7]University of Modena and Reggio Emilia, Cellular Signaling Unit- Department of Biomedical- Metabolic and Neural Sciences, Modena, Italy*

**Introduction**

Resistance to Glucocorticoids (GCs) is still a limitation in the treatment of pediatric T-cell Acute Lymphoblastic Leukemia (T-ALL) patients and a well-defined poor outcome predictor. Thus, the comprehension of GC resistance underlying mechanisms could improve T-ALL patients' overall survival. Interestingly, our research group has already unveiled the LCK kinase's pivotal role in T-ALL cells' GC resistance onset, although the downstream regulated biological processes remained to be elucidated. To this end, here we focused on the LCK downstream NFAT family transcription factors.

**Material and Methods**

To identify the NFAT family members modulating GC resistance we performed in vitro and in vivo proliferation assays in *NFATs* silenced or overexpressing T-ALL cells treated with GCs. Next, transcriptome analysis in NFATc1 or NFATc2 knock down cells allowed to infer the NFATc1 or NFATc2 driven biological processes responsible for GC resistance. Moreover, by Nuclear Magnetic Resonance and Chromatin Immune Precipitation we characterized the lipidomic landscape in *NFATc1* knock down cells and the *NFATc1* or *NFATc2* direct target genes.

**Results and Discussions**

We demonstrated that exclusively NFATc*1* or *NFATc2* specific gene silencing restores GC sensitivity in T-ALL GC resistant cells, whereas their overexpression in GC sensitive cells restores the resistance. Furthermore, we revealed that NFATc1 confer GC resistance by directly regulating the transcription of cholesterol biosynthesis' genes. In agreement, exogenous cholesterol addition to *NFATc1* knock down cells rebuild GC resistance, on the contrary the cholesterol biosynthesis inhibitor simvastatin sensibilizes T-ALL cells to GCs. Besides, we revealed that NFATc2 sustains GC resistance by directly controlling the transcription of *LRP6*, a Wnt/β-catenin pathway player. Interestingly, the Wnt/β-catenin signaling activation restores GC resistance in *NFATc2* knock down cells, whereas its inhibition increases GC sensitivity. Finally, we revealed that NFATc1 and NFATc2 promote GC resistance by hindering the Glucocorticoid Receptor (GR) transcriptional activity. In agreement, diagnosed pediatric GC resistant T-ALL patients display a high NFATc1-NFATc2 and a low GR transcriptional activity.

**Conclusion**

Overall, the identification of NFATc1 and NFATc2 as new regulator of GC resistance through the modulation of cholesterol biosynthesis, Wnt/β-catenin signaling and GR transcriptional activity, will provide the rationale for alternative therapeutic options to overcome T-ALL GC resistance.

### EACR2024-0447

### BIGH3: a novel target to treat immunotherapy-resistant cancer through stroma modulation

*S. Bachy[1], K. Aguera[1], S. Martins[1], M. Masmoudi[1,2], A. Deshiere[1], H. Hernandez[3], C. De la Fouchardiere[4], G. Rawadi[1], A. Hennino[1,2]*

© 2024 The Authors. Molecular Oncology is published by John Wiley & Sons Ltd on behalf of Federation of European Biochemical Societies

[1]StromaCare, RnD, Lyon, France
[2]Cancer Research Center of Lyon - UMR INSERM 1052 CNRS 5286, RnD, Lyon, France
[3]Genomics consulting, RnD, Lyon, France
[4]Centre Leon Berard, Clinical, Lyon, France

**Introduction**

Solid tumors are associated with a stromal reaction promoting immune escape and cell proliferation. We identified BIGH3 as a key regulator of this mechanism, harboring a dual pro-tumoral function, by (1) inhibiting CD8+ T-cell cytotoxic activity, and (2) limiting their infiltration through the induction of the tumor stroma stiffness.

**Material and Methods**

We assessed BIGH3 expression in human tissue samples using in silico data analysis by AI-driven data mining of RNA sequencing data sets combined with Tissue Microarray Analysis (TMA). In vivo experiments to test a specific anti-BIGH3 monoclonal antibody (STRO-501) were performed using genetically engineered mice models for pancreatic cancer. The safety of STRO-501 was evaluated using immunohistochemistry-based ex vivo Tissue Cross Reactivity (TCR) GLP study, performed from 37 human and cynomolgus monkey tissues. For in vivo toxicity studies, cynomolgus was selected based on the highest homology to human BIGH3 protein sequence (>98%) and a full conservation of the specific epitope recognized by STRO-501.

**Results and Discussions**

In silico data analysis revealed that BIGH3 is enriched in various cancer types, in particular pancreatic and colorectal adenocarcinoma. We observed a correlation of BIGH3 mRNA levels with the stiffness score associated with these tumors. BIGH3 enrichment in the tumor stroma was confirmed in pancreatic and colorectal cancer biopsies. Levels of BIGH3 were significantly higher in TMA sections when compared to samples from healthy patients ($p < 0.001$). The neutralization of BIGH3 in vivo using anti-BIGH3 STRO-501 limited tumor growth in mice by reducing the stiffness of the stroma and increasing the number of activated infiltrating CD8+ T cells. Moreover, targeting BIGH3 with STRO-501 in PD-1 resistant cancer models could potentiate the efficacy of Pembrolizumab and trigger T-cells reactivation. Finally, a preliminary in vivo toxicity study conducted in 9 cynomolgus monkeys demonstrated a good tolerability profile for STRO-501 without significant adverse events and until 200 mg/kg. TCR results highlighted a major BIGH3 on-target stromal signal for STRO-501.

**Conclusion**

Altogether, these data support the role of BIGH3 in the modulation of the tumor microenvironment and resistance against immunotherapy targeting T-cell activation. Anti-BIGH3 monoclonal antibody STRO-501 is well tolerated and represents a novel promising therapeutic molecule to treat patients with cancers associated with an important stromal reaction.

### EACR2024-0451
### Teneurin-4 induces doxorubicin resistance through endoplasmic reticulum stress attenuation in triple negative breast cancer

G. Peppino[1], G. Barutello[1], F. Atzori[1], E. Bolli[1], E. Quaglino[1]
[1]University of Torino, Molecular Biotechnology and Health sciences, Torino, Italy

**Introduction**

Endoplasmic reticulum (ER) stress, a mechanism resulting from the accumulation of misfolded proteins, has been implicated in different steps of tumorigenesis such as tumor progression, autophagy and chemo-resistance. When cells undergo ER stress, they activate an unfolded protein response to limit the stress. However, whether it persists, cells undergo cell death through CHOP upregulation. Teneurin-4 (TENM4), a trans-membrane protein that we identified as pro-tumorigenic in triple negative breast cancer, is known to be induced by CHOP. However, the role and involvement of TENM4 in ER stress and chemoresistance still remain unknown.

**Material and Methods**

4T1 and MDA-MB231 cells deficient for TENM4 were obtained with shRNA technology. Annexin/DAPI and DCFH-DA staining were assessed by FACS to evaluate cell viability and ROS production upon doxorubicin (doxo) treatment. ER stress, as CHOP, ATF4 and P-ELF2α expression, and autophagy as LC3B, P-AKT and p62 expression, were evaluated by western blot. For the in vivo experiments, mice were subcutaneously injected with 4T1 TENM4-deficient or WT cells. When the tumors reached 2 mm, mice were treated with doxo and tumor growth was monitored.

**Results and Discussions**

A higher chemosensitivity to doxo and ROS production were observed in TENM4-deficient compared to WT cells. Since increased CHOP and TENM4 expression were observed upon ER stress induction with tunicamycin, we hypothesized that the higher chemosensitivity observed was related to ER stress. Notably, TENM4-deficient cells exhibited higher ER stress as CHOP and ATF4 expression upon doxo treatment, suggesting a role of TENM4 in mitigating ER stress. Conversely, P-Elf2α, also associated with ER stress and required for autophagy induction, was downregulated in TENM4-deficient cells. Interestingly, TENM4-deficient cells also exhibited lower levels of the autophagy markers p62, LC3B and P-AKT. Being autophagy known to alleviate ER stress, these results suggest that TENM4, by influencing Elf2α phosphorylation, induces autophagy, which consequently attenuates ER stress, contributing to chemoresistance. Moreover, supporting the role of TENM4 in chemoresistance, 4T1 doxo-resistant cells show higher level of TENM4 and lower CHOP expression.

**Conclusion**

Overall, these data demonstrate an involvement of TENM4 in ER stress and chemoresistance. A better investigation of the ER stress pathways in which TENM4 is involved could provide a novel target to maximize ER stress and cell death, thus contributing to overcome chemoresistance.

### EACR2024-0457
### LAMP2A-Mediated Chaperone-Mediated

© 2024 The Authors. Molecular Oncology is published by John Wiley & Sons Ltd on behalf of Federation of European Biochemical Societies

### Autophagy as a Target for Overcoming Platinum Resistance in Ovarian Clear Cell Carcinoma

*G. Yılmaz Demir*[1], *İ. Durmaz Şahin*[1,2], *Ö. Öktem*[2,3], *Y. Esmaeilian*[2]
[1]*Koç University, Graduate School of Health Sciences, Istanbul, Turkiye*
[2]*Koç University, Research Center for Translational Medicine, Istanbul, Turkiye*
[3]*Koç University, Department of Obstetrics and Gynecology, Istanbul, Turkiye*

**Introduction**

Ovarian clear cell carcinoma (OCCC) is diagnosed in its early stages, but these cells respond poorly or not at all to chemotherapy. This resistance to chemotherapy results in relapse or death in patients due to inadequate response to platinum group chemotherapeutic agents.In this context, alternative treatment strategies should be developed. Chaperone-mediated autophagy (CMA), one of the subtypes of the autophagy mechanism that plays an important role in the pathophysiology of cancer cells, degrades a specific protein at certain times. Many cancer cells activate the CMA mechanism to increase their tumorigenic potential and survive longer. Therefore, understanding the effect of lysosomal associated protein 2A (LAMP2A), which plays a fundamental role in CMA, on drug resistance in OCCC is important to shed light on current therapeutic strategies.

**Material and Methods**

Ovarian clear cell carcinoma cell lines JHOC-5 and TOV-21G were used. To understand the effect of LAMP2A on the resistance of platinum group drugs in these cells, a guide RNA specific to the LAMP2A gene was designed and knocked out (KO) using the CRISPR/Cas9 system. The knockout of this gene was confirmed by QPCR and Western Blot methods. After inhibition of CMA, response analyses to cisplatin and carboplatin drugs in parental, plasmid control, and knockout cells were determined using the Sulforhodamine B (SRB) method. We also aim to identify the apoptosis proteins that change the resistance mechanism by inhibiting LAMP2A.

**Results and Discussions**

Through cytotoxicity assay analysis, we discovered that in ovarian clear cell carcinoma cells resistant to platinum group drugs, decreased expression of LAMP2A, the fundamental protein of chaperone-mediated autophagy, causes sensitivity to cisplatin and carboplatin. As a result of this analysis, we showed that the IC50 values against cisplatin and carboplatin drugs decreased in JHOC-5 and TOV21-G cells. The cell cycle analyses and the changes in apoptosis proteins that occur in this resistance mechanism by suppressing LAMP2A were performed.

**Conclusion**

Inhibition of LAMP2A appears to be a promising anticancer strategy in cisplatin and carboplatin-resistant ovarian clear cell carcinoma. The process of chaperone-mediated autophagy seems to be involved in maintaining drug resistance sensitivity. We believe that elucidating this mechanism in the drug-resistant subgroup of ovarian cancer will contribute to treatment strategies in the future.

### EACR2024-0458
### Role of intra-tumor heterogeneity in chemotherapy resistance in triple-negative breast cancer

*N. Sánchez Collantes*[1], *R. Blanco*[1], *I. Garrido-Cano*[2], *S. Torres-Ruiz*[2], *B. Cánovas*[3], *F. Calvo*[1], *Á.R. Nebreda*[3], *J.M. Cejalvo*[2], *I. Varela*[1]
[1]*Instituto de Biomedicina y Biotecnología de Cantabria IBBTEC- Universidad de Cantabria-CSIC, Cellular and Molecular Signalling, Santander, Spain*
[2]*INCLIVA Biomedical Research Institute- Hospital Clínico Universitario Valencia- University of Valencia, Oncology, Valencia, Spain*
[3]*Institute for Research in Biomedicine IRBBarcelona- The Barcelona Institute of Science and Technology, Cancer, Barcelona, Spain*

**Introduction**

Breast cancer clinical subtypes are defined by the expression of estrogen receptor (ER), progesterone receptor (PR) and the amplification of human epidermal growth factor receptor 2 (HER2). Current targeted therapies are directed against any of these receptors, which activate cancer-related pathways and sustain tumor progression. However, triple-negative breast cancer (TNBC) subtype does not express any marker and, thus, is not susceptible to these therapies. Neoadjuvant chemotherapy based on taxanes and anthracyclines, in combination with anti-PD1 immunotherapy, is the standard of care against TNBC. Unfortunately, around 50% of patients become resistant to this treatment. This project looks at chemoresistance beyond the genomic landscape of TNBC tumors. We hypothesize that a comprehensive study of the tumor transcriptional plasticity and its microenvironment can unravel key mechanisms of treatment resistance in TNBC.

**Material and Methods**

We apply single-cell RNA sequencing on primary samples from 15 sensitive and 15 resistant TNBC patients before and after neoadjuvant chemotherapy with/without immunotherapy. We look for differences between responders and non-responders in terms of transcriptional profile, cellular composition of the tumor and its microenvironment, as well as crosstalk between tumor and immune subpopulations.

**Results and Discussions**

Transcriptional-based cell clustering of pre-treated tumors allows to identify and independently study distinct cell-type populations within tumors. We identify tumor and immune transcriptional populations present at different ratios in resistant and sensitive tumors. Preliminary data suggests that tumor subpopulations with intense ribosome biogenesis are more abundant in resistant tumors before treatment administration, whereas highly-cycling tumor subpopulations are more prevalent in sensitive tumors. Regarding the crosstalk between the tumor and its microenvironment, we are able to identify intense communication between tumor and B-cells before treatment administration through specific signaling pathways, which may play a role in the different cell composition observed between sensitive and resistant tumors, prior to chemotherapy.

**Conclusion**

© 2024The Authors. Molecular Oncology is published by John Wiley & Sons Ltd on behalf of Federation of European Biochemical Societies

The transcriptional profiles identified inside primary pre- and post-treated tumors will shed light on cell populations and molecular pathways involved in treatment resistance and may be useful markers for patient stratification and prediction of treatment response, improving tailored therapy in TNBC patients.

### EACR2024-0479
### Regulation of oxidative stress and ferroptosis in ovarian cancer models by Semaphorin 4C: implications for therapy responsiveness

*R. Mastrantonio[1], C. Palazzo[2], C. Ruscetta[1], E. Testa[2], M. Buttarelli[2], C. Nero[2], M.T. Viscomi[1,2], L. Tamagnone[1,2]*
*[1]Università Cattolica del Sacro Cuore, Department of Life Science and Public Health- section of Histology and Embryology, Rome, Italy*
*[2]Fondazione Policlinico Universitario Agostino Gemelli I RCCS, Dipartimento per le Scienze della salute della donna e del bambino e di sanità pubblica, Rome, Italy*

**Introduction**
The dismal prognosis of ovarian cancer is largely due to the paucity of available therapeutic options in the advanced stage, and especially to the frequent onset of resistance to main stand platinum-based chemotherapy. Notably, alterations in redox balance and deregulated redox signaling are common hallmarks of cancer progression and resistance to therapy, including in ovarian cancer. Our lab has previously demonstrated the importance of Semaphorin 4C (SEMA4C) in breast cancer progression; indeed, SEMA4C is highly expressed in high grade advanced ovarian cancers, although its putative mechanistic role in this context has not been assessed. The aim is to elucidate the functional role and underlying signaling mechanism of SEMA4C in ovarian cancer cells, and its potential relevance as molecular target to improve therapy responsiveness.
**Material and Methods**
In order to investigate the role of SEMA4C in therapy responsiveness a panel of ovarian cancer cell lines were transduced by lentiviral vectors and transfected with siRNA to achieve the overexpression and the silencing of SEMA4C, and then subjected to platinum-based treatments. The mechanisms of chemoresistance studied on cell lines were also validated in primary cultures of cancer cell spheroids derived from the ascitic fluids of patients that experience high grade serous ovarian carcinoma.
**Results and Discussions**
Our data indicate a so far unknown role of SEMA4C in the regulation of NRF2, which is a key player of the antioxidant response, controlling refractoriness to platinum therapy. NRF2 in known to be involved in the modulation of ferroptosis regulating proteins, indeed the depletion of SEMA4C impacts on ROS and lipid ROS production, and, consequently, on viability of cells subjected to platinum-based treatments. In this context, the strengthening of oxidative stress and then, ferroptosis, can overcome the chemoresistance displayed by ovarian cancer cells.
**Conclusion**
The discovery of SEMA4C as a new player in oxidative stress-controlling chemoresistance could pave the ground for new approaches, improving ovarian cancer responsiveness to therapy.

### EACR2024-0484
### Mitocurcumin serves as a potential drug candidate for the treatment of therapy-resistant non-small cell lung cancer through modulating glutathione redox cycle

*G.C. Panigrahi[1], K. Gandhi[2], A. Joshi[1], V. Gota[1]*
*[1]Advanced Centre for Treatment- Research and Education in Cancer- HBNI, Clinical Pharmacology, Navi Mumbai, India*
*[2]Advanced Centre for Treatment- Research and Education in Cancer- HBNI, Clinician Scientist Lab, Navi Mumbai, India*

**Introduction**
Lung cancer is the world most leading cancer type in terms of mortality in which non-small cell lung cancer (NSCLC) is the major subtype contributing 85% of total lung cancer. It has low survival rates because of chemoresistance and complex tumor immune micro-environment. Here, we provide the novel therapeutic intervention for the better treatment of therapy-resistant NSCLC.
**Material and Methods**
Cisplatin resistant A549 cell line was developed by pulse treatment method to mimic the clinic scenario. The anti-tumor activity of mitocurcumin (mitoC) was established in various pre-clinical models such as A549 cisplatin sensitive and resistant xenografts, and disseminated LLC1 syngeneic model. Mechanistic study involving lentiviral shRNA based knockdown and overexpression studies was carried out to validate the potential target responsible for drug resistance. Biochemical enzyme inhibition and insilico docking studies were done to provide the insights into target-inhibitor interaction. Moreover, pharmacokinetics study of mitoC in mice was done by LC-MS.
**Results and Discussions**
MitoC exhibited five-fold more cytotoxicity towards cisplatin resistant NSCLC cells as compared to the parental cells. Glutathione reductase (GSR) was found to be highly upregulated in A549 cisplatin resistant (A549R) cells and when knocked down reversed drug resistance and decreased migration and invasion in vitro. However, overexpression of GSR in A549 cells led to cisplatin resistance and increased migration and invasion potential. GSR was inhibited by mitoC in cell free and cell-based system and bound to GSR other than the active site. MitoC showed mixed-II type of inhibition with GSR. It increased cellular and mitochondrial ROS in dose dependent manner in A549 and A549R cells. The prolonged exposure to mitoC decreased intracellular GSH pool thereby induced ROS mediated cytotoxicity. Mice xenograft study showed mitoC alone and in combination with docetaxel had better therapeutic potential for treatment of cisplatin resistant NSCLC tumors. It regressed non-immunogenic lung tumor by increasing tumor infiltrating cytotoxic T-cells. MitoC

© 2024 The Authors. Molecular Oncology is published by John Wiley & Sons Ltd on behalf of Federation of European Biochemical Societies reached its $C_{max}$ at 2h and get fully eliminated by 48h from the body.

**Conclusion**

MitoC exhibits higher cytotoxic effect in cisplatin resistant NSCLC. It reverses GSR mediated drug resistance in NSCLC by blocking glutathione system. It has low toxicity and high efficacy at its therapeutic dose. Hence, mitoC can be a potential standard of care drug for treatment of advanced NSCLC including cold tumors.

### EACR2024-0496
### KRAS mutations drive trifluridine resistance in colorectal cancer by regulating multidrug transporters

*X. Ma[1], P. van der Helm[1], J. Catsman[1], N. de Vries[2], H. Rosing[2], M. Parra Martinez[1], L. Leek[1], A. Huitema[2], J. van de Haar[1], E. Voest[1]*
*[1]The Netherlands Cancer Institute, Molecular Oncology & Immunology, Amsterdam, The Netherlands*
*[2]The Netherlands Cancer Institute, Pharmacy and Pharmacology, Amsterdam, The Netherlands*

**Introduction**

Genomic biomarkers to predict the outcome of chemotherapy are an unmet need. We recently reported that KRAS codon G12 ($KRAS^{G12}$) mutations could be used to predict failure to respond to trifluridine/tipiracil (FTD/TPI) in patients with metastatic colorectal cancer (mCRC). In isogenic cell lines and a panel of patient-derived mCRC organoids, $KRAS^{G12}$ mutations were associated with increased resistance to FTD, but the underlying resistance mechanism remained unknown.

**Material and Methods**

KRAS mutant cell lines with codon-specific mutations were engineered using CRISPR ctRNP technology. Intracellular FTD levels were assessed by immuno-fluorescence and mass spectrometry analyses. Cell viability was determined using IC50 measurements and colony formation assays. The expression of ATP-binding cassette (ABC) transporters was evaluated via qRT-PCR, FACS analysis, and western blot. Additionally, DNA damage was assessed through western blot.

**Results and Discussions**

We discovered that $KRAS^{G12}$ mutations induced resistance to FTD by reducing the intracellular concentration of FTD in $KRAS^{G12}$ mutant cells compared to $KRAS^{WT}$ cells. Mutations within KRAS were found to stimulate the expression of ATP-binding cassette (ABC) transporters in a cell line-dependent manner. Among these transporters, ABCB1 and ABCG2 emerged as the most commonly heightened drug transporters. This observation was validated in patient-derived mCRC organoids. Moreover, we noted a significant association between the KRAS G12D mutation and the upregulation of the multidrug-resistance-associated protein ABCC3 in cell lines. This finding was further substantiated in a patient cohort obtained from TCGA dataset. The escalated ABC transporters functioned as an efflux transporter of FTD, leading to reduced intracellular accumulation of FTD and contributing to drug resistance in $KRAS^{G12}$ mutant cells. Knocking down ABC transporters in codon-specific KRAS mutant cell lines respectively resulted in increased intracellular FTD concentrations and greater FTD-related DNA damage.

**Conclusion**

Our results implicate a direct link between KRAS mutations and drug transporters and provide a molecular mechanism for the observed resistance to FTD in $KRAS^{G12}$ mutant colorectal cancer cells.

### EACR2024-0497
### MYC Upstream Region Conferes Resistance to PI3K Inhibitors in Colorectal Cancer and Burkitt Lymphoma through MEK1-Mediated Autophagic Adaptation

*R. Bordone[1], D.M. Ivy[1], R. D'Amico[1], M. Barba[1], F. Di Pastena[2], B. Cesaro[3], A. Fatica[3], S. Coni[1], L. Di Magno[1], G. Canettieri[1]*
*[1]Sapienza University of Rome, Department of Molecular Medicine, Rome, Italy*
*[2]McMaster University, Department of Medicine, Hamilton ON, Canada*
*[3]Sapienza University of Rome, Department of Biology and Biotechnology "Charles Darwin", Rome, Italy*

**Introduction**

The MYC oncogene is frequently overexpressed in tumors and in particular in most of Colorectal Cancers (CRCs) and Burkitt Lymphomas (BLs), where inhibition of its translation is considered an attractive therapeutic opportunity. An internal ribosome entry site (IRES) was identified in MYC Upstream Region (MYC UR) and was suggested to play a leading role in maintaining elevated MYC content when global cap-dependent translation is inhibited, to allow survival of cancer cells during stress and resistance to chemotherapy. However, despite a quite high number of publications supporting this hypothesis, there have been conflicting reports arguing against IRES translation.

**Material and Methods**

Using a gene editing approach, we removed MYC UR from CRC cell lines and analyzed them combining in vitro and in vivo approaches. We also used different BL cell lines harboring or not MYC UR along with MYC open reading frame depending on the t(8;14) translocation breakpoint.

**Results and Discussions**

We demonstrated that the MYC UR does not facilitate cap-independent translation under cellular stress but, instead, it orchestrates resistance to PI3K inhibitors by acting as a central enhancer of the autophagic flux. We provide evidence that genomic deletion of MYC UR does not affect MYC protein content or CRC cell survival under basal conditions or in response to different types of stress. Conversely, when cells are exposed to Pi3K inhibitors, MYC UR mediates a MEK1-ERK1 dependent transcriptional upregulation of MYC and drug resistance, while cells lacking MYC UR are more vulnerable to Pi3K inhibitors and undergo programmed cell death. Mechanistically, this resistance is mediated by enhanced autophagic flux, governed by MYC, which promotes the expression of a set of autophagy-related target genes. Of note, we demonstrate that Burkitt Lymphoma cells lacking the chromosomal translocation of MYC UR are sensitive to Pi3K inhibitors, whereas cells in which this region is translocated respond to this drug only if autophagy is blocked.

© 2024 The Authors. Molecular Oncology is published by John Wiley & Sons Ltd on behalf of Federation of European Biochemical Societies

**Conclusion**
These findings challenge previous notions regarding IRES-mediated translation and highlight a promising strategy to overcome resistance to PI3K inhibitors in MYC-driven malignancies, offering potential clinical implications for Colorectal Cancer and Burkitt Lymphoma treatment.

### EACR2024-0506
### A Dual Role of Proline Dehydrogenase in Lung Cancer

P. Chiofalo[1], E. Berno[1], R. Cinquetti[1], L. Pulze[1], S. Valci[2], V. Guzzeloni[2], G. Foggetti[2,3], P. Campomenosi[1]
[1]*University of Insubria, Department of Biotechnology and Life Sciences, Varese, Italy*
[2]*IRCCS San Raffaele Scientific Institute, Department of Medical Oncology and Division of Experimental Oncology, Milan, Italy*
[3]*Vita-Salute San Raffaele University, Milan, Italy*

**Introduction**
Lung cancer is still a global health problem. Present therapeutic approaches, among which Tyrosine Kinase Inhibitors (TKIs), have improved outcomes of patients with lung adenocarcinoma (LUAD). However, resistance mechanisms occur. Proline dehydrogenase (PRODH) could be a novel target and/or biomarker of therapy response in EGFR mutant LUADs. In mitochondria, PRODH oxidizes proline to pyrroline 5-carboxylate. The electrons produced during the reaction can be used to generate ATP or ROS species, thus affecting several cellular processes, such as survival, senescence, and apoptosis, playing a critical role in cancer. Using different cellular models, we have shown a negative correlation between EGFR and PRODH expression, which appears to be mediated by STAT3. We hypothesized that STAT3 decreases *PRODH* expression, by recognizing specific response elements (REs) in the *PRODH* gene (Aim 1).Concurrently, we observed an increase in PRODH expression in EGFR mutant LUAD cell lines with in vitro acquired resistance to EGFR TKIs, compared to their parental cell lines, and asked if PRODH could play a role in acquired resistance (Aim 2).
**Material and Methods**
Luciferase assays were carried out in different cell lines using constructs carrying *PRODH* intronic regions containing putative STAT3 binding sites identified bioinformatically. To investigate PRODH role in acquired resistance to TKIs, we carried out PRODH silencing or inhibition in HCC827 cells, which carry EGFR exon 19 p.E746-A750del, and in its derivative HCC827-GR5 (2$^{nd}$ gen. TKI, gefitinib-resistant) cells, grown in 2D or 3D, and evaluated viability, and apoptosis.
**Results and Discussions**
Results 1. We observed luciferase activity upon STAT3 inhibition in DU145 cells compared to those treated with vehicle. Luciferase activity instead decreased when we ectopically expressed STAT3 in NCI-H1299 cells. Our data suggest that STAT3 may directly bind its specific REs in the *PRODH* gene to repress its expression and favor cell growth. Results 2. We observed that PRODH inhibition strongly impaired 2D and 3D growth of EGFR mutant, resistant LUAD cells but instead favored the growth of the parental cell line, in line with previous data. Interestingly, in TKI-resistant cells, PRODH inhibition determined an increased expression of cell cycle arrest (p21) and apoptotic (PUMA, caspase 8) markers.
**Conclusion**
From the data shown here, PRODH may exert different roles in early- and late-stage lung cancer and upon acquired drug resistance, based on the activated signaling pathways.

### EACR2024-0523
### BRK'ing down Replication Stress Response in Ovarian Cancer to Improve Platinum Therapy

K. Yap[1]
[1]*National University of Singapore, Department of Pharmacology, Singapore, Singapore*

**Introduction**
High-grade Serous Ovarian Cancer (HGSOC) is the most aggressive subtype of ovarian cancer, and is notorious for high relapse rates and platinum chemoresistance. Most HGSOC patients, which are BRCA wild-type, do not respond well to PARP inhibitors and have limited treatment options, thus warranting the urgent need to develop novel therapeutic approaches to improve their treatment outcomes. Recently, Protein Tyrosine Kinase 6 (PTK6/BRK) was found to be highly expressed in 70% of HGSOC patients and has been linked to DNA damage and chemoresistance in other cancers, but its role in ovarian cancer is unknown. The present study investigated the underlying mechanism behind PTK6-mediated platinum resistance and PTK6's potential as a therapeutic target in HGSOC.
**Material and Methods**
Bioinformatics analyses of ovarian cancer patient data from CSIOVDB and TCGA databases were carried out to evaluate PTK6 expression on patient survival, platinum resistance and Replication Stress Response (RSR) function. Gain- and loss-of-function in-vitro experiments were performed to determine how PTK6 mediates platinum resistance through the RSR pathway.
**Results and Discussions**
In-silico patient data revealed that high PTK6 expression was associated with shorter overall and progression-free survival, platinum resistance, and functional RSR in HGSOC patients. In-vitro studies showed that PTK6 depletion in BRCA-proficient HGSOC cells impaired their ability to repair cisplatin-induced DNA damage, leading to increased cisplatin sensitivity. Moreover, following cisplatin exposure, PTK6-depleted cells exhibited reduced nuclear levels of Replication Protein A 32 (RPA32) and RAD51, and reduced levels of RSR activation markers, pRPA32 S33 and pRPA32 S4/S8. We also show that the kinase activity of PTK6 is responsible for promoting the cisplatin-induced nuclear accumulation of RPA. Additionally, cisplatin stimulated the co-localisation of PTK6 and RPA transporter protein, human RPA-interacting protein (hRIP), in the nuclei of HGSOC cells, indicating that PTK6 promotes the cisplatin-induced nuclear import of RPA through hRIP, leading to RPA nuclear accumulation and RSR activation.
**Conclusion**

© 2024 The Authors. Molecular Oncology is published by John Wiley & Sons Ltd on behalf of Federation of European Biochemical Societies

Our study elucidates PTK6 as a crucial mediator of platinum resistance in HGSOC, by promoting the activation of the RSR through the hRIP-mediated nuclear import of RPA. Thus, our findings potentiate PTK6 inhibition as a novel strategy to enhance the efficacy of platinum chemotherapy, potentially transforming the ovarian cancer treatment landscape.

### EACR2024-0537

### Combinatorial therapeutic targeting of PI3K and CDK4/6 displays synergistic antitumor effects in PPGL

*F. Gentile*[1], *B. Karna*[2], *S. Gulde*[2], *D. De Martino*[2], *K. Ganesan*[2], *J. Maurer*[3], *H. Mohr*[2], *S. Nölting*[3,4], *N.S. Pellegata*[1,2]

[1]*University of Pavia, Department of Biology and Biotechnology "L. Spallanzani", Pavia, Italy*

[2]*Helmholtz Zentrum München, Institute for Diabetes and Cancer, Neuherberg, Germany*

[3]*Ludwig-Maximilians-University Munich, Department of Medicine IV- University Hospital, Munich, Germany*

[4]*University Hospital Zurich USZ and University of Zurich UZH, Department of Endocrinology- Diabetology and Clinical Nutrition, Zurich, Swaziland*

**Introduction**

Paragangliomas (PPGLs) are rare neuroendocrine tumors deriving from chromaffin cells located in para-sympathetic paraganglia or in the adrenal medulla (pheochromocytomas). Surgery represents the curative treatment for localized PPGLs. For aggressive and metastatic PPGLs, effective therapeutic options are lacking. Thus, there is an urgent clinical need to identify novel treatments for PPGLs. This study aims at evaluating the potential antitumor effects of targeting PI3K signaling and cell cycle regulation processes in PPGLs. We selected buparlisib (BKM120), a highly specific PI3K inhibitor, and ribociclib (LEE011), a CDK4/6 inhibitor.

**Material and Methods**

BKM120 and LEE011 were used alone and in combination on PC12 cells (rat PPGL). Proliferation, viability, migration, invasion, and apoptosis assays were conducted in both 2D and 3D settings. RNASeq was performed on treated PC12 cells. To identify mediators of the response to the various treatments, bioinformatics analysis was conducted using R software and DESeq2, GO, GSEA, EnrichR and DecoupleR pipelines.

**Results and Discussions**

In vitro experiments showed that the combination was more effective at inhibiting the viability, migration and invasion of PC12s than the single drugs. Bioinformatics analyses showed a strong downregulation of PI3K-AKT-MTOR, G2M checkpoint, E2F targets and MTORC signaling pathways upon combination therapy. The GSEA category “mitotic spindle” was uniquely enriched in combination-treated cells. We then focused on the transcription factors (TFs) regulating the differentially expressed genes in combination treatment vs untreated cells. TF analyses identified Foxm1 as a potential target of both BKM120 and LEE011: both its expression and its activity were significantly downregulated especially in the combination treatment. Foxm1 and its downstream targets are essential for mitotic entry and spindle checkpoint functioning. A decrease in Foxm1 function upon combination treatment could lead to mitotic catastrophe and cell death. Experimental data support such mechanism of action of the drug combination.

**Conclusion**

Our study demonstrates that the combination of BKM120 and LEE011 has higher antitumor activity against PPGL cells when compared to the individual drugs. Bio-informatics analysis highlighted a significant suppression of cell cycle regulatory pathways in combination-treated cells and identified Foxm1 as potential mediator of the synergistic effect of BKM120 and LEE011 in PPGLs.

### EACR2024-0601

### Modeling and treating cetuximab resistance using ex vivo colorectal tumor slices

*S. Asmanidou*[1], *W. Aulitzky*[2], *M. Dong*[2], *J. Thiel*[2], *S. Johnsen*[3], *T. Ekstrom*[3], *J. Schüler*[4], *E. Oswald*[4], *R. Kontermann*[1], *M. Olayioye*[1]

[1]*University of Stuttgart, Institute of Cell Biology and Immunology, Stuttgart, Germany*

[2]*Bosch Health Campus, Dr. Margarete Fischer-Bosch-Institute of Clinical Pharmacology, Stuttgart, Germany*

[3]*Bosch Health Campus, Robert Bosch Center for Tumor Diseases, Stuttgart, Germany*

[4]*Charles River Laboratories Inc, Charles River Discovery Research Services Gemany GmbH, Freiburg im Breisgau, Germany*

**Introduction**

Despite recent medical advances in treatment options, colorectal cancer (CRC) still remains the second-leading cause of carcinogenic death worldwide. Patients with RAS wild-type metastatic CRC are treated with the monoclonal antibody cetuximab that targets the epidermal growth factor receptor (EGFR). Unfortunately, therapy success is usually limited by the development of drug resistance within several months. To explore new therapeutic strategies targeting treatment-resistant cancer cells, we utilize ex vivo tissue slices from patient-derived xenografts (PDX) to model the complexity and heterogeneity of CRC tumors.

**Material and Methods**

As a cell line model, the K-Rras wild-type human CRC cell line LIM1215 was subjected to gradual cetuximab dose escalation over six months to establish cetuximab-resistant cell populations. In these cells, putative drug targets were identified by transcriptome profiling combined with molecular pathway analysis. As human CRC tumor models, PDX tumors were selected based on cetuximab sensitivity scores and K-Rras mutational status. Upon excision, viable PDX tumors were cut into 250 μm thick slices using a vibratome. Tissue slices were cultivated on Millipore filters and treated with specific drug combinations followed by immunocytochemical stainings.

**Results and Discussions**

Molecular profiling and transcriptome analysis of cetuximab-resistant LIM1215 cell populations revealed that the cells remained wild-type for K-Ras while apoptosis-related processes were upregulated. The predicted sensitivity towards cell death-inducing agents could be verified by flow cytometry and western blot

© 2024 The Authors. Molecular Oncology is published by John Wiley & Sons Ltd on behalf of Federation of European Biochemical Societies analysis of apoptotic markers after treating the cells with a set of BH3-mimetic drugs. These cell death-inducing agents were applied on ex vivo tissue slice cultures derived from cetuximab-resistant PDX tumors. Immunocytochemical stainings proved potent induction of apoptosis of the cancer cells in three different PDX tumor slice models, validating the results derived from the cetuximab-resistant cell line models.

**Conclusion**

Here we provide evidence that heterogenous K-Ras wild-type human CRC models that fail to respond to cetuximab remain sensitive towards apoptosis-inducing drugs. Our work thus holds promise for defining effective second-line treatments for cetuximab-resistant CRC.

### EACR2024-0692
### Implications of SWI/SNF complex alteration and its role in sensitivity to lung cancer treatments

*R. Marqueño Navarro[1], D. Martín[1], L. García[1], R. Blanco[1], I. Varela[1]*
*[1]Instituto de Biomedicina y Biotecnología de Cantabria IBBTEC, Señalización Celular y Molecular, Santander, Spain*

**Introduction**

Lung cancer stands as the most common cause of cancer-related deaths worldwide, representing 1 out 5 deaths. Over the last ten years, the ATP-dependent SWI/SNF chromatin remodeling complex have emerged for its role in tumour suppression. In lung cancer, different SWI/SNF genes are recurrently found mutated.SWI/SNF complex is a key regulatory element that use the energy of the ATP hydrolysis for the displacement or disruption of nucleosomes. Consequently, it plays an important role in DNA accessibility and transcriptional regulation. Nevertheless, the consequences of its alteration in cancer are still unclear.

**Material and Methods**

Non-small cell lung cancer cell lines were modified through CRISPR/Cas9 technology for the generation of knock-out (KO) models for *ARID1A*, *ARID2* and *SMARCA4*, the SWI/SNF genes with the highest rate of mutation in lung cancer. Gene editing were confirmed at the genetic and protein level by sequencing and western blot, respectively. Once validated, the different cell models were treated with dacomitinib, an EGFR inhibitor, at different concentrations to evaluate differences in the response to this drug. Additionally, we performed co-cultures of activated cytotoxic T-cell to assess differences in the sensitivity to the immune response.

**Results and Discussions**

It is known that the disruption of SWI/SNF complex can lead alterations in the pattern of gene expression which may result in changes at many levels. We, and others, have observed alterations in the EGFR pathway target genes in the context of mutated SWI/SNF complex. Our results show a higher sensitivity to dacomitinib after 48h of treatment in the KO cell lines compared to the WT. Moreover, it seems that this behaviour may not be subunit-specific but rather a common feature of SWI/SNF deficiency.In relation to the role of SWI/SNF in the response to immunotherapy, our preliminary results of the cell viability assays seem to show that the KO cell lines present a higher sensitivity to the cytotoxic T-cells compared to the WT. Being this effect stronger in the *SMARCA4* KO cell lines.

**Conclusion**

Our results suggest that SWI/SNF deficiency could lead to an alteration in the EGFR pathway, resulting in a higher sensitivity to inhibitors of this pathway, independently of the truncated subunit. Moreover, alterations in this complex may be involved in a higher sensitivity to activated cytotoxic T-cells. Although further research is needed to confirm these results, they can open new venues to improve patient stratification for immunotherapy.

### EACR2024-0702
### Anti-leukemic Efficacy of Azacitidine relies on AML-intrinsic RNA Receptor Signaling, and secondary resistance can be overcome by pathway modulating all-trans-retinoic acid

*N. El Khawanky[1,2], T. Nedelko[1,2], Y. Barth[1,2], M. Rosenbaum[3], S. Enßle[1,2], C. Weiß[2,3], J. Ruland[2,3], F. Bassermann[1,2], S.M. Hedeigger[1,2]*
*[1]Klinikum rechts der Isar- Technical University of Munich, Department of Medicine III Hematology/ Oncology- School of Medicine, Munich, Germany*
*[2]Technical University of Munich, TranslaTUM- Center for Translational Cancer Research, Munich, Germany*
*[3]Institute of Clinical Chemistry and Patho-biochemistry- Technical University of Munich, School of Medicine, Munich, Germany*

**Introduction**

Acute myeloid leukemia (AML) patients are commonly treated with Azacitidine (AZA). However, the molecular mechanisms by which AZA modulates anti-leukemic immunity against AML remains unclear. Our findings suggest that AZA-triggered anti-leukemic immune responses are the result of activated innate nucleic RNA-sensing pathways. RIG-I/MAVS and subsequent type I interferon (IFN-I) signaling seem to trigger the transcriptional upregulation of, otherwise, silenced endogenous retroviruses (ERVs). Here, we further investigated how the above-mentioned processes are affected during acquired AZA resistance (AZA-R). All-trans-retinoic acid (ATRA) was previously identified to upregulate RIG-I expression in acute promyelocytic leukemia. We, therefore, examined whether it could be agonistically exploited to overcome AZA-R AML.

**Material and Methods**

AZA-R cells were generated from wildtype (WT) murine (c1498 and Wehi-3B) and human (MOLM-13) AML cell lines and were exposed to AZA in vitro and in vivo. Expression of ERVs, transcriptional and protein expression of components from the RIG-I/MAVS pathway, and its role in AZA-induced immuno-surveillance were comprehensively studied. AZA-R cells were treated with ATRA to determine whether they could be re-sensitized to anti-leukemic immunity through RIG-I/MAVS.

**Results and Discussions**

© 2024 The Authors. Molecular Oncology is published by John Wiley & Sons Ltd on behalf of Federation of European Biochemical Societies

Transcriptional and protein expression of ERVs and downstream constituents of the RIG-I/MAVS pathway were abrogated in AZA-R AML cells. AZA-R AML bearing mice (c1498 and Wehi-3B) failed to show anti-leukemic activity following AZA treatment where recipient animals rapidly succumubed to disease. In depth CyTOF analysis revealed that T- and NK cell infiltration was crucial for mediating anti-leukemic response to AZA treated WT AML bearing mice. This was not observed in AZA-R AML bearing mice. Treatment of AZA-R AML in vitro and in vivo with ATRA re-induced RIG-I/MAVS expression mediating IFN-I independent apoptosis, re-boosting anti-leukemic activity. Further molecular mechanisms of RIG-I dependent ATRA efficacy in AZA-R AML are currently being investigated.

**Conclusion**

We show that AML-intrinsic RIG-I/MAVS signaling is imperative for AZA efficacy and immunosurveillance in AML, which is defective in AZA-R AML. Elucidating the molecular mechanisms of how AML-intrinsic nucleic acid sensors shape AZA treatment efficacy may guide development of systematic treatment strategies for AZA-R AML. We identify ATRA as a therapeutic strategy for AZA-R AML that re-ignites RIG-I/MAVS and anti-leukemic effects.

### EACR2024-0718
### Targeting SERCA2 calcium pumps sensitizes chemoresistant ovarian carcinoma cells and patient-derived tumor organoids to the BH3-mimetic ABT-737

*S. Messaoudi[1], R. Florent[2], L.B. Weiswald[1,2,3], E. Abeilard[1,3], M. Guillamin[4], G. Desmartin[2,3], L. Lecouflet[2,3], J. Divoux[2,3,4], L. Poulain[1,2,3,4], M. N'Diaye[1]*
*[1]Université de Caen Normandie, INSERM- ANTICIPE U1086- Interdisciplinary Research Unit for Cancer Prevention and Treatment- Comprehensive Cancer Center F. Baclesse, Caen, France*
*[2]Université de Caen Normandie, Services Unit PLATON "support platforms for preclinical and translational research in oncology"- ORGAPRED core facility, Caen, France*
*[3]UNICANCER, Comprehensive Cancer Center F. Baclesse, Caen, France*
*[4]Université de Caen Normandie, Services Unit PLATON "support platforms for preclinical and translational research in oncology"- IsoCELL, Caen, France*

**Introduction**

Ovarian cancer has a poor clinical prognosis due to innate or acquired chemoresistance following carboplatin/paclitaxel treatment. PARP inhibitors have revolutionised patient management, but they are only offered to patients with Homologous Recombination Deficiency who have responded to carboplatin treatment. There are therefore no effective options for patients who have not responded to chemotherapy. The chemo-resistance can be explained by an unbalanced ratio of anti-apoptotic (Bcl-$x_L$, Mcl-1) to pro-apoptotic (Bim, Puma, Noxa) Bcl-2 family members that prevents cell apoptosis. Calcium signaling deregulation is strongly involved in carcinogenesis and is involved in Bcl-2 family members expression in several cancer models. In this context, SERCA2 pumps play a major role because they control the flow of apoptotic calcium to the mitochondria but also, if they are inhibited, the UPR response at the origin of ER stress, which, if prolonged, triggers apoptosis. Therefore, we evaluated the interest of SERCA2 pumps inhibition to sensitize chemoresistant ovarian cancer cells to ABT-737, a BH3-mimetic that targets Bcl-$x_L$.

**Material and Methods**

The platinum-resistant cell line, OAW42-R, was treated with anti-SERCA2 strategies (siRNA or thapsigargin), in combination with ABT-737. Bcl-2 family members expression, induction of ER stress and apoptosis were studied by western blot, trypan blue counting and flow cytometry). Functional studies were carried on using siRNA and pharmacological inhibitors. Finally, the therapeutic efficacy of the combination in the clinic was evaluated on a 3D-model: patient-derived tumor organoids (PDTO). Three chemoresistant ovarian PDTO lines were treated with the thapsigargin/ABT-737 combination, and viability was assessed using CellTiterGlo assay.

**Results and Discussions**

These co-treatments induced a strong Noxa- and caspase-dependent apoptosis that involved UPR response and more precisely ATF4 transcription factor activation. Furthermore, thapsigargin/ABT-737 treatment induced a massive destructuration and a diminution of PDTO viability.

**Conclusion**

Our study shows for the first time that targeting SERCA2 calcium pump and inducing UPR response could be a relevant strategy in ovarian cancer treatment, offering new hope for patient management. The use of PDTO supported the results obtained in 2D culture and improved their relevance. As it turns out, compounds enabling the induction of ER stress are currently in clinical trials in various cancers, consolidating the therapeutic interest of our study.

### EACR2024-0725
### HDAC6 Targeted Therapy Approaches to Reverse Taxane Resistance in Castration Resistant Prostate Cancer

*A. Işıklar[1], İ. Bulut[1], B. Cevatemre[2], B. Yildirim[1], B.M. Kalkan[2], D. Ruzic[3], A. Lee[4], A. Ganesan[4], K. Nikolic[3], C.A. Ayhan[2,5]*
*[1]Koc University, Graduate School of Health Sciences, Istanbul, Turkiye*
*[2]Koc University, Research Center for Translational Medicine, Istanbul, Turkiye*
*[3]University of Belgrade, Department of Pharmaceutical Chemistry, Belgrade, Serbia*
*[4]University of East Anglia, School of Pharmacy, Norwich, United Kingdom*
*[5]Koc University, School of Medicine, Istanbul, Turkiye*

**Introduction**

Primary prostate cancers (PCa) can be treated with radiotherapy, but treatment options are limited for metastatic "castration resistant" (CR) phases. While some initially benefit from taxane treatment, resistance can develop, often driven by tumor epigenetic alterations,

© 2024 The Authors. Molecular Oncology is published by John Wiley & Sons Ltd on behalf of Federation of European Biochemical Societies particularly involving histone deacetylase (HDAC). Among these, HDAC6 stands out, as its knockout in mice is non-lethal and reduces tumor size, presenting a promising therapeutic avenue. This research endeavors to identify novel HDAC6 inhibitors that synergize with taxanes or counteract their resistance in CRPCa cells.

**Material and Methods**

A total of 42 HDAC6 inhibitors were synthesized and evaluated for efficacy against CRPCa and taxane-resistant CRPCa cells. Initial screening through SRB and CTG assays identified 11 inhibitors for further evaluation. In order to test their efficacy; HDAC6 enzyme activity assays are used, and specificity was determined by inhibition of HDAC1 and HDAC8 activity. The molecules, which failed to successfully inhibit HDAC6 were eliminated. Target engagement of HDAC6 inhibitors in cells was assessed via CETSA, while their impact on invasion and migration was evaluated through wound healing and Matrigel invasion assays. Western blotting and qPCR were employed to investigate HDAC6 inhibitor effects on nuclear and cytosolic targets, with RNA-seq analysis delving into their mechanism of action. In vivo studies examined their impact on tumor growth and lethality.

**Results and Discussions**

In summary, among 42 HDAC6i, 9b was the most potent, based on $IC_{50}$, specificity, selectivity, and exhibition of the expected phenotypes. Interestingly, targeting CRPCa cells with 9b successfully resensitized resistant cells to taxanes without affecting the sensitive parental lines. Ongoing investigations are focused on elucidating the mechanism of action of 9b and its efficacy in reducing tumor size in murine models. These findings hold promise for the development of novel treatment modalities for taxane-resistant patients.

**Conclusion**

In conclusion 9b successfully synergies with taxanes without affecting parental cell lines. So 9b could be promising for HDAC targeted therapies to revert chemoresistance in castration resistant prostate cancer.

### EACR2024-0730
### Metabolic changes occurring in BRAF/MEK inhibitors resistant melanoma cells

*A. Simiczyjew*[1], *M. Kot*[1], *K. Pietraszek-Gremplewicz*[1], *D. Nowak*[1]
[1]*Faculty of Biotechnology- University of Wroclaw, Department of Cell Pathology, Wroclaw, Poland*

**Introduction**

One of the most often used methods for melanoma treatment is a therapy based on the BRAF (v-raf murine sarcoma viral oncogene homolog B1)/MEK (mitogen-activated protein kinase kinase) inhibitors. Unfortunately, patients develop resistance to this form of therapy quite quickly and the mechanisms underlying this phenomenon are not yet fully understood. We have obtained two melanoma cell lines (WM9 and Hs294T) resistant to BRAF/MEK inhibitors (vemurafenib and cobimetinib, respectively), and thoroughly characterized them, among others, in terms of occurring within cells metabolic changes.

**Material and Methods**

The real-time PCR method was used to examine the expression level of selected genes related to cellular metabolism. The level of specific proteins was examined by Western blotting. Lipid droplets were stained with Lipid Spot 488 reagent, while their number and size were determined using an OPERA high-throughput microscope. Selected aspects of cell metabolism were examined using commercial kits.

**Results and Discussions**

Altered levels of expression of genes related to the transport of glucose (GLUT1 and GLUT3), lactate (MCT4), and $Na^+/H^+$ ions (NHE1) were observed in resistant cells compared to control ones. Moreover, the number of lipid droplets in resistant cells was increased, while their size was decreased, which was accompanied by a lower level of perilipin 2 (a protein present in the lipid droplet envelope). We also noticed elevated expression of fatty acid synthase (FASN) whereas the expression of hormone-dependent lipase (LIPE) was reduced in resistant melanoma cells. Moreover, the expression of PPARγ was decreased and the expression of caveolin 1 was increased in resistant cells, which was related to the metabolic changes observed in cells. Additionally, we performed several metabolic tests on the examined cells and a reduced level of lactate secreted by resistant cells was observed.

**Conclusion**

Melanoma resistance to therapy based on BRAF/MEK inhibitors is one of the main obstacles to the effective treatment of this cancer. It is therefore necessary to understand the molecular basis of this resistance to find tools to overcome it. As the metabolism of melanoma cells impacts cancer progression, we decided to focus on changes appearing in this aspect of resistant cells' functioning.

*This research was funded by the National Science Centre, Poland, with a grant received by Aleksandra Simiczyjew (SONATA 16, 2020/39/D/NZ5/02330).*

### EACR2024-0733
### Proteolysis strongly supports the invasion of metastatic melanoma cells resistant to treatment with BRAF/MEK inhibitors

*M. Kot*[1], *A. Simiczyjew*[1], *J. Wądzyńska*[1], *D. Nowak*[1]
[1]*Faculty of Biotechnology- University of Wroclaw, Department of Cell Pathology, Wroclaw, Poland*

**Introduction**

Melanoma is a very aggressive type of skin cancer. Due to occurring in around 50% of patients with BRAFV600E mutation, which makes BRAF kinase constantly active, BRAF/MEK inhibitors are routinely used in melanoma therapy. Unfortunately, the treatment is not always effective due to the emergence of drug resistance. The features accompanying the mentioned drug resistance have not been well understood yet. We decided to focus on the evaluation of the migration and invasion of BRAFi/MEKi-resistant cells and the mechanisms that support these phenomena.

**Material and Methods**

2D and 3D wound healing assays were performed to assess the migratory and invasive abilities of control and resistant melanoma cells. The activity of matrix

© 2024 The Authors. Molecular Oncology is published by John Wiley & Sons Ltd on behalf of Federation of European Biochemical Societies metalloproteinases (MMPs) was evaluated with gelatin zymography. To measure protein level Western Blot analysis was performed. To explore the expression level of selected genes qRT-PCR analysis was conducted. The Human Protease Array Kit was used to determine the composition of secreted by cells proteases.

**Results and Discussions**

Resistance to the treatment with BRAF and MEK inhibitors (vemurafenib and cobimetinib, respectively) was successfully developed in two metastatic melanoma cell lines, WM9 and Hs294T. Obtained resistant cells exhibited increased migration and, even more elevated, invasion in comparison to the control ones. It was also accompanied by a significant increase in proteolytic activity of resistant cells detected as elevated level of selected MMPs in conditioned medium derived from resistant and control ones as well as increased activity of MMP-2 and MMP-9. Additionally, changes in the level of MMPs' inhibitors have been also noticed. Moreover, the human Protease Array Kit revealed the elevated level of several cathepsins and MMPs in resistant melanoma cells.

**Conclusion**

The emergence of resistance in human melanoma cells is accompanied by several changes that occur after BRAFi/MEKi treatment. A better understanding of the mechanisms underlying drug resistance, including processes as important for carcinogenesis as migration and invasion, could certainly contribute to attempts to improve the therapeutic strategy and, as a result, have an impact on better prognosis for patients with advanced metastatic or unresectable melanoma.

*This research was funded by the National Science Centre, Poland, with a grant received by Aleksandra Simiczyjew (SONATA 16, 2020/39/D/NZ5/02330).*

### EACR2024-0885
### MicroRNA-31 as a novel therapeutic target to reverse chemo-resistance in pancreatic ductal adenocarcinoma

*D. Hackett[1], J. McGrath[1], X. Lin[2], R. Hilger[3], J. Stoof[1], B. Bibby[4], A. Buckley[1], A. Heeran[1], S. Maher[1]*
*[1]Trinity St. James's Cancer Institute- Trinity Translational Medicine Institute- Trinity College Dublin, Department of Surgery, Dublin, Ireland*
*[2]Royal College of Surgeons in Ireland RCSI, School of Pharmacy & Biomolecular Sciences, Dublin, Ireland*
*[3]University Hospital Essen, Department of Medical Oncology, Essen, Germany*
*[4]NHS Humber and North Yorkshire Integrated Care Board, Integrated Care Board, Hull, United Kingdom*

**Introduction**

Pancreatic ductal adenocarcinoma (PDAC) has a dismal long-term patient survival rate. This poor prognosis is in part due to poor patient responses to current standard-of-care chemotherapy regimens, including FOLFIRINOX (folinic acid, 5-FU, irinotecan and oxaliplatin) and Gem-Abraxane (Gemcitabine/nab-paclitaxel). Platinum agents are used in the treatment of a wide variety of solid malignancies. However, patients receiving platinum-based chemotherapy eventually succumb to treatment resistance. MicroRNAs (miRNAs) are small non-coding RNAs that may play an important role in modulating multiple cellular processes involved in chemo-resistance. We have previously demonstrated in several cancers, including PDAC, that loss of miR-31 influences cellular sensitivity to chemotherapy and radiotherapy. In silico analysis reveals ATOX1, a cytoplasmic copper (Cu) chaperone and transcription factor, as a predicted target of miR-31. There is evidence that cisplatin binds to the Cu(I)-ATOX1 complex. This interaction may facilitate cisplatin shuttling to target DNA in the nucleus.

**Material and Methods**

A pCMV-miR vector containing a miR-31 mimic was stably expressed into a miR-31-deficient PDAC cell line, BxPC-3. Additionally, a pmiRZip lentivector suppressing miR-31 was stably expressed in a miR-31 abundant PDAC cell line, Panc-1. ICP-MS post cisplatin treatment was used to measure levels of $Pt_{195}$ within the cytoplasmic and nuclear compartments of miR-31-manipulated PDAC cell lines.

**Results and Discussions**

We found that miR-31 expression inversely correlated with the nuclear accumulation of $Pt_{195}$ in PDAC cells. Our results show that overexpressing miR-31 significantly reduced ATOX1 expression in PDAC cells. Kaplan-Meier survival analysis of PDAC patients showed that patients with low miR-31 expression and high ATOX1 expression have significantly improved overall survival. Direct, miR-31-independent over-expression of ATOX1 expression in Panc-1 cells resulted in a significant increase in sensitivity to cisplatin. In both PDAC and oesophageal adeno-carcinoma isogenic models of cisplatin resistance, we found that cisplatin-resistant cells had significantly reduced expression of ATOX1 compared to matched parental cell lines. This loss of ATOX1 expression following acquisition of cisplatin resistance potentially suggests a conserved ATOX1-mediated resistance mechanism.

**Conclusion**

Our study demonstrates, for the first time, that manipulating miR-31 alters ATOX1 expression, promoting either a chemo-resistant or chemo-sensitive phenotype.

### EACR2024-0914
### Targeting Nrf2-Regulated Antioxidant Systems to Enhance Chemotherapy Response and Ferroptosis Induction in Chemotolerant Medulloblastoma Cells

*L. Manfreda[1], F. Rruga[1], C. Marchioro[1], M. Canton[1], E. Rampazzo[1], E. Mariotto[1], L. Persano[1], G. Viola[1], R. Bortolozzi[2]*
*[1]University of Padova, Department of Women's and Children's Health, Padova, Italy*
*[2]University of Padova, Department of Pharmaceutical and Pharmacological Sciences, Padova, Italy*

**Introduction**

Medulloblastoma (MB) is the most common brain tumor of childhood associated with high frequency of relapse after treatments and aggressive chemotherapy-dependent neurocognitive and endocrine dysfunctions. To deepen complex dynamics among tumor treatment, the emergence of drug resistance, and the initiation of tumor recurrence, we established an in vitro model of MB

© 2024 The Authors. Molecular Oncology is published by John Wiley & Sons Ltd on behalf of Federation of European Biochemical Societies resistance by exposing MB cells to a combination of Vincristine, Etoposide, Cisplatin, Cyclophosphamide (VECC). Multiomic profiling of MB-resistant models, besides the identification of transcriptional changes affecting cellular pathways involved in the control of cell growth and differentiation, highlighted the improvement of the mechanisms of control of redox homeostasis.

**Material and Methods**

The REDOX status of MB-resistant cells was evaluated by flow cytometry using DCFDA, for reactive oxygen species (ROS) detection and monobromobimane (mBBr), for GSH evaluation. The enhancement of the Nrf2 pathway was evaluated by protein expression detection and by Antioxidant Response Element luciferase reporter assays. Cell viability assay and flow cytometry techniques were used to assess the effects of Nrf2 knockdown and the combination of chemotherapy with ferroptotic inducers.

**Results and Discussions**

Our study focuses on antioxidant defense systems and their role in MB-resistance to chemotherapy. We found that MB-resistant cells exhibit increased transcriptional activation of Nrf2, leading to enhanced ROS scavenging capacity and improved tolerance to ferroptosis inducers, which are able to disrupt redox homeostasis, resulting in fatal lipid peroxide accumulation in cells. The inhibition of Nrf2 synergizes with both chemotherapy and ferroptosis inducers by reducing GSH levels in cells. Notably, preliminary analysis indicates that GSH reduction, mediated by Erastin-induced inhibition of cystine uptake, and GPX4 inhibition, mediated by RSL3, significantly enhance the efficacy of chemotherapy (VECC) in resistant cells. Furthermore, we observed that the overactivation of Nrf2 pathway correlates with worse survival in MB patients, underscoring the importance of targeting Nrf2 to prevent and treat chemoresistance in medulloblastoma

**Conclusion**

Taken together this data strongly supports the hypothesis that MB-resistant cells rely on GSH-dependent systems activated by Nrf2 and that affecting the complex antioxidant balance, could be exploited as a tool to shut down the defense systems of MB-resistant cells.

### EACR2024-0921
### POSTER IN THE SPOTLIGHT
### Pooled CRISPR Screening Identifies AP-1 Transcription Factor as a Druggable Driver of Osimertinib Resistance in Non-Small Cell Lung Cancer

*B. Dayanc[1,2], S. Eris[1,2], E. Cakiroglu[1,2], G. Ozden-Yilmaz[1,2], N.E. Gulfirat[1,2], O.S. Coskun-Deniz[1,2], G. Karakülah[1,3], S. Erkek-Ozhan[2], S. Senturk[1,2]*
*[1]Izmir International Biomedicine and Genome Institute-Dokuz Eylul University, Genomics and Molecular Biotechnology, Izmir, Turkiye*
*[2]Izmir Biomedicine and Genome Center, Basic and Translational Research Program, Izmir, Turkiye*
*[3]Izmir Biomedicine and Genome Center, Technological Research Program, Izmir, Turkiye*

**Introduction**

Non-small cell lung cancer (NSCLC) poses a formidable challenge in oncology, with EGFR-activating mutations being a driver mechanism. Although EGFR-Tyrosine Kinase Inhibitors (EGFR-TKIs),such as the third-generation irreversible Osimertinib,show promise in treating advanced NSCLC,drug resistance remains a major obstacle.Here,by leveraging a combination of pooled CRISPR screening,transcriptomic analysis and chromatin landscape profiling,we interrogate the significance of non-genetic synthetic lethal interactions and related transcriptional networks in resistance to Osimertinib.

**Material and Methods**

Osimertinib-resistant HCC827 subclone,devoid of secondary EGFR mutations,was established by dose-escalation strategy.Non-genetic regulatory mechanisms underlying Osimertinib resistance were explored via CRISPR screening using a gRNA library enriched for epigenetic and transcription factors.The resistance mechanisms were further investigated by employing high-throughput techniques, such as RNA-seq,ChIP-Seq and ATAC-Seq.Furthermore,a series of experiments were performed to investigate the role of AP-1 transcription factor in Osimertinib resistance.These included cell proliferation,survival and invasion assays performed in FOSL1 and JUN knock-out cells and upon pharmacological inhibition of AP-1 using SR11302.

**Results and Discussions**

HCC827-OsiR cells exhibited diminished activity in the EGFR pathway,with concomitant increase in AKT,ERK and STAT3 signaling.CRISPR screening identified epigenetic and transcription factors as modulators of resistance.Among these,AP-1 transcription factor complex,specifically FOSL1 and JUN,emerged as a high-confidence druggable hit.Depletion of AP-1 through genetic manipulation or pharmacological inhibition reinstated Osimertinib sensitivity,as evidenced by reduced colony formation,diminished proliferative capacity and restoration of EGFR pathway responses. Global transcriptome profiling between Osimertinib sensitive and resistant states,along with AP-1 knock-out in resistant cells,unveiled gene regulatory networks associated with AP-1-mediated resistance.ATAC-Seq and ChIP-Seq studies further unveiled potential AP-1 target genes modulated by FOSL1 and JUN.

**Conclusion**

This study underscores the significance of the druggable AP-1 complex in modulating Osimertinib resistance and its synthetic lethal interactions associated with the resistance phenomenon.Ongoing research will help to uncover the role of AP-1 downstream signaling in Osimertinib resistance,providing potential therapeutic implications.

### EACR2024-0933
### Cooperative and antagonistic interactions between therapy resistant melanoma sub-clones

*K. Schlegelmilch[1], V. Hollek[1], G. Giangreco[1], S. Strohbuecker[2], S. Hooper[1], S. MacFarlane[1], E. Sahai[1]*
*[1]The Francis Crick Institute, Tumour Cell Biology, London, United Kingdom*
*[2]The Francis Crick Institute, Bioinformatics and*

© 2024 The Authors. Molecular Oncology is published by John Wiley & Sons Ltd on behalf of Federation of European Biochemical Societies

*Biostatistics, London, United Kingdom*

**Introduction**

Melanoma is composed of diverse populations of cells at the genetic, epigenetic and phenotypic level. This intratumoural heterogeneity facilitates tumour evolution and represents a major challenge in cancer therapy as acquired resistance to BRAF and MEK targeted inhibition (BRAFi+MEKi) still results in poor patient prognosis for late stage melanoma today. Despite this, in vivo models that explore the dynamics of clonal interaction in a tumour containing heterogeneous resistance mechanisms during treatment with targeted therapy are currently lacking.

**Material and Methods**

We therefore generated human BRAF-mutant melanoma models representing genetic ($NRAS^{Q61K}$), non-genetic (active YAP1) as well as spontaneously arising resistance mechanisms to BRAFi+MEKi. Using RNA sequencing we investigated which signalling pathways are differentially affected in the different melanoma cells. A targeted compound screen was used to identify targets that are unique for individual resistance mechanisms and those that are common across multiple resistance mechanisms.

**Results and Discussions**

The RNA sequencing analysis demonstrated transcriptomic differences for melanoma cells with different resistance mechanisms. Our screening approach identified that each resistant state has distinct pharmacological vulnerabilities, which poses significant challenges for the design of strategies aiming to prevent the emergence of resistance entirely. Using an innovative in vivo approach to investigate intratumoural heterogeneity we are assessing if subclones with different resistance mechanisms compete or cooperate, and how they respond to treatment with inhibitors identified in our screens.

**Conclusion**

Development of various resistance mechanisms to targeted therapy within a tumour is common and hinders therapy success. We need to understand the molecular mechanisms and clonal dynamics within a tumour with multiple resistance mechanisms. Our results will aid the development of more effective therapies for melanoma.

### EACR2024-0938

### Reporter-assisted classification of resistance mechanisms to broad-spectrum RAS inhibition in KRAS-mutant colorectal cancer

*O. Aust*[1], *E. Blanc*[2], *V. Hollek*[1], *B. Papke*[1,3,4], *D. Horst*[1,3], *N. Blüthgen*[1,3], *C. Sers*[1,3], *C.J. Der*[4], *M. Morkel*[1,2,3]

[1]*Charité - Universitätsmedizin Berlin, Institute of Pathology, Berlin, Germany*

[2]*Charité - Universitätsmedizin Berlin, Berlin Institute of Health, Berlin, Germany*

[3]*German Cancer Research Center, German Cancer Consortium Partner Site Berlin, Berlin, Germany*

[4]*University of North Carolina at Chapel Hill, Lineberger Comprehensive Cancer Center, Chapel Hill, USA*

**Introduction**

KRAS activating mutations are observed in 40% of colorectal cancer (CRC). Recently, RAS inhibitors targeting a limited spectrum of mutations have demonstrated preclinical efficacy in RAS-mutant cancer models and promising results in the clinic. However, emerging resistance to RAS inhibition limited long-term efficacy. Here, we aim to identify and categorize mechanisms of resistance to a broad-spectrum active-state RAS inhibitor (hereafter termed RASi) in CRC cell lines.

**Material and Methods**

To this end, we employ a compartment-specific dual-color ERK activity reporter system to classify emerging resistant cell populations by KRAS effector pathway reactivation, and characterize mechanisms of resistance in sorted cell subpopulations.

**Results and Discussions**

We found that KRAS-mutant CRC cell lines are universally sensitive to RASi, as the treatment halted proliferation, which was in some instances accompanied by apoptosis. RASi treatment also resulted in inhibition of the RAS-RAF-MEK-ERK axis, as we demonstrated by western blot analysis and the ERK reporter. Long-term dose escalation of RASi in stably ERK reporter-expressing CRC cancer cell lines revealed multiple patterns of ERK reactivation in emerging resistant cell populations, which we could validate by assessing activities of compartment-specific ERK targets. Cells sorted according to their reported ERK activity patterns were characterized on the genome, phosphoprotein and transcriptome levels by exome sequencing, mass cytometry, and single cell RNA-sequencing, respectively. The resistant subpopulations showed distinct mutational patterns, as well as heterogeneous signal network states and transcriptomic signatures, casting light on the resistance mechanisms.

**Conclusion**

In summary, our observations validate reporter-assisted screening together with single cell analyses as a powerful approach for dissecting the complex landscape of therapy resistance. The strategy offers new opportunities to develop clinically-relevant combinatorial treatments to compete with the emergence of resistant cancer cells.

### EACR2024-0956

### Developing and characterising a T-DXd-resistant breast cancer cell line

*N. Ashfield*[1], *A.F. Mahdi*[1,2], *N.T. Conlon*[3], *J. Crown*[1,4], *D.M. Collins*[1]

[1]*Dublin City University, Cancer Biotherapeutics Research Group, School of Biotechnology, Dublin, Ireland*

[2]*University of Limerick, School of Medicine, Limerick, Ireland*

[3]*Dublin City University, School of Biotechnology, Dublin, Ireland*

[4]*Saint Vincent's University Hospital, Department of Medical Oncology, Dublin, Ireland*

**Introduction**

Trastuzumab deruxtecan (T-DXd) is a HER2-targeting antibody-drug conjugate (ADC) consisting of trastuzumab conjugated to a topoisomerase I inhibitor. T-DXd has been approved for the treatment of HER2-

© 2024 The Authors. Molecular Oncology is published by John Wiley & Sons Ltd on behalf of Federation of European Biochemical Societies overexpressing (HER2+) breast and gastric cancers, HER2-mutant non-small cell lung cancer and breast cancers with low HER2 expression. However, as with any other targeted treatment, patients are likely to develop resistance to T-DXd over time. This study aimed to develop T-DXd-resistance in the HER2+/oestrogen receptor (ER)-negative HCC1569 cell line and characterise these cells to elucidate potential resistance mechanisms.

**Material and Methods**

For six months, T-DXd-resistant HCC1569 cells (HCC1569-TDXd) were fed twice a week with medium containing increasing concentrations of T-DXd. Parental HCC1569 cells were used as an age-matched control with no drug treatment. 5-day acid phosphatase (AP) assays were used to assess antiproliferative effects of anti-HER2 ADCs T-DXd and T-DM1, HER-targeting tyrosine kinase inhibitors (TKIs; afatinib, lapatinib, neratinib, tucatinib), SN38 (T-DXd payload) and mertansine (T-DM1 payload). Western blots were performed on HCC1569 and HCC1569-TDXd cell protein lysates to assess changes in expression of total and phosphorylated HER2, EGFR, Akt, ERK, as well as topoisomerase I expression levels. Student's t-tests were used to analyse differences between IC50 values and protein expression levels of HCC1569 and HCC1569-TDXd. $p \leq 0.05$ was considered significant.

**Results and Discussions**

After six months, the HCC1569-TDXd cells were growing in 50ng/mL of T-DXd. AP assays confirmed at least 800-fold resistance in the HCC1569-TDXd cells (T-DXd IC50: HCC1569 = 11.98±2.36ng/mL; HCC1569-TDXd >10000ng/mL). No significant cross-resistance to T-DM1 was observed. The IC50 value for SN-38 was slightly higher in HCC1569-TDXd than in HCC1569 but this difference was not significant (HCC1569 = 1.85± 0.49nM; HCC1569-TDXd = 3.44±1.23nM; p=0.14). The IC50 value of afatinib was lower in HCC1569-TDXd than in HCC1569 (HCC1569 = 11.86±0.37nM; HCC1569-TDXd = 6.63±2.34nM; p=0.06). Western blotting showed upregulation of EGFR and Akt and downregulation of phosphorylated HER2 (pHER2) and phosphorylated Akt (pAkt) in HCC1569-TDXd compared to HCC1569.

**Conclusion**

We have successfully developed HCC1569-TDXd, a T-DXd-resistant cell line derived from the HER2+/ER- HCC1569. Work is ongoing to further characterise these cells and identify relevant resistance mechanisms.

### EACR2024-0965
### Study of the role of IFITM1 and other interferon-regulated genes in drug resistance in tumors

*J.M. Berna Belmonte[1], I. Pachón Bermúdez[2], M. Fuentes Baile[1], M.D.P. Garcia Morales[2], M. Saceda Sanchez[2], M.D.C. De Juan Romero[2]*
*[1]Unidad de Investigación- Fundación para el Fomento de la Investigación Sanitaria y Biomédica de la Comunidad Valenciana FISABIO- Hospital General Universitario de Elche- Camí de la Almazara 11- Elche- 03203 Alicante- Spain, Molecular Oncology, Elche, Spain*
*[2]Instituto de Investigación- Desarrollo e Innovación en Biotecnología Sanitaria de Elche IDiBE- Universidad Miguel Hernández- Avda. Universidad s/n- Ed. Torregaitán- Elche- 03202 Alicante- Spain, Molecular Neurobiology and Cancer, Elche, Spain*

**Introduction**

We have previously identified, by differential gene expression assays, a set of genes implicated with the response to interferon. Their expression changed in a statistically significant way during the processes of acquisition of resistance to 5-Fluorouracil (5-FU) in colon carcinoma cell lines (COAD) and to Paclitaxel in breast carcinoma cell lines. During the acquisition of resistance, interferon-induced transmembrane 1 and 3 (IFITM1 and IFITM3) were among the differentially expressed genes. IFITM1 and IFITM3 are proteins that are overexpressed in several types of cancer, including COAD and glioblastoma (GBM). They are crucial in tumor proliferation, metastasis, and invasion and inhibit apoptosis. In addition, they play a key role in resistance acquisition against different therapies; however, their mechanism of action remains unknown.

**Material and Methods**

This study was carried out using cell proliferation and survival assays, as well as cell cycle study by flow cytometry. QPCR and gene silencing by specific small interferent RNAs were also performed in different cell models of colon and GBM to analyze differential gene expression. Cells were treated or not with different therapeutic agents, to evaluate the relationship between the expression of our genes of interest with resistance to different treatments.

**Results and Discussions**

The expression levels of IFITM1 and some of the other genes of interest correlate with the degree of intrinsic resistance to the different treatments tested in our cell models. On the other hand, when selective pressure with drugs was applied to obtain resistant cell models, parallel changes in the expression of our genes of interest were observed. The silencing of IFITM1 did not seem to be sufficient to produce the reversion of resistance in the newly generated resistant cell lines. In this study, we got some insights into the molecular mechanisms that relate the acquisition of chemoresistance to the expression of these genes in COAD and GBM cell lines treated with 5-FU and carmustine (BCNU).

**Conclusion**

Our results indicate that an increase in the expression of a group of interferon-related genes correlates with resistance acquisition to different treatments in COAD and GBM cell models. This study opens the venue to the development of possible new therapies targeting these genes. Nevertheless, further studies are required to clarify the involvement of target genes in the acquisition of chemoresistance.

### EACR2024-0986
### SHIP2 regulates esophageal squamous cell carcinoma growth and response to therapy

*B. Beck[1], R. Ramos[2], G. Bregni[2], C. Bodart[2], N. Gillet[2]*
*[1]FNRS - Université libre de Bruxelles, IRIBHM, Brussels, Belgium*

© 2024 The Authors. Molecular Oncology is published by John Wiley & Sons Ltd on behalf of Federation of European Biochemical Societies

[2]ULB, faculty of medicine, Brussels, Belgium

**Introduction**

Esophageal squamous cell carcinoma (eSCC) is among the deadliest cancers worldwide. Current management, consisting of surgery preceded by neo-adjuvant radio-chemotherapy, is poorly efficient. Its relapse carries a short-term grim prognosis because of rapid resistance to therapy and the limited number of efficient drugs available. In this context, it is crucial to develop novel targeted therapies against eSCC. The role of PIP3, the product of phosphoinositide (PI) 3-kinase (PI3K) is often considered oncogenic, but other PIs such as PI(3,4)P2 are also critical signal molecules. SHIP2 (SH2 domain-containing PI-3,4,5-trisphosphate 5-phosphatase 2) is an enzyme able to catalyze the formation of PI(3,4)P2 and plays a crucial role in AKT activation. Phosphorylation of the AKT protein, one of the mechanisms leading to the activation of the pathway, shows higher expression in eSCC than in corresponding normal tissue, supporting the relevance of the PI3K/AKT path in eSCC development. However, virtually nothing is known about the role of SHIP2 in esophageal cancer.

**Material and Methods**

In this study, we used publicly available transcriptomics and genomics dataset, a panel human eSCC cell lines, siRNA and pharmacological inhibitors, xenografts, histology, and RNA sequencing.

**Results and Discussions**

By analyzing data from the Cancer Genome Atlas, we discovered that esophageal squamous cell carcinoma (eSCC) is the most prevalent cancer type exhibiting amplification of INPPL1, which codes for the SHIP2 enzyme. SHIP2 is the 5-phosphatase with the highest expression in human eSCC cells. Our investigation revealed that siRNA-mediated SHIP2 knockdown impedes cell growth across multiple eSCC lines. Similarly, pharmacological inhibition of SHIP2 results in decreased eSCC cell survival in both in vitro and in vivo settings. The inhibition of SHIP2 decreases AKT phosphorylation levels, suggesting that SHIP2 modulates eSCC cell growth through the regulation of the AKT pathway. By profiling eSCC cells, we identified several transcripts coding for druggable targets that are downregulated following SHIP2 inhibition. This led us to hypothesize that SHIP2 inhibition might enhance the sensitivity of cells to targeted therapies. Subsequently, we administered a combination of SHIP2 inhibitors and FDA-approved drugs targeting these identified candidates, confirming synergies in drug effects on eSCC cells.

**Conclusion**

In conclusion, SHIP2 regulates eSCC cell growth and sensitizes cancer cells to chemotherapy and targeted therapy.

### EACR2024-0989
### Investigating Mechanisms of Resistance to Osimertinib in EGFR-Driven Lung Adenocarcinoma

*S. Valci*[1], *S. Nerina*[1], *V. Guzzeloni*[1], *P. Chiofalo*[2], *P. Campomenosi*[2], *G. Foggetti*[1]
[1]*IRCCS San Raffaele Scientific Institute - Vita-Salute San Raffaele University, Experimental Oncology-Medical Oncology, Milan, Italy*
[2]*University of Insubria, Biotechnology and Life Science, Varese, Italy*

**Introduction**

Lung adenocarcinoma (LUAD) is the most frequent histological subtype of lung cancer and EGFR-driven LUAD represents ~15% of cases. Osimertinib, a 3rd gen. tyrosine kinase inhibitor (TKI), is the frontline therapy for advanced stages of this disease. Despite a longer overall survival in patients treated with osimertinib compared to previous gen. TKIs (38.6 vs. 31.8 months), drug resistance inevitably emerges posing an important clinical challenge due to the lack of second-line therapeutic option availability. Clinical trials designed to target known on-target or off-target mechanisms of osimertinib resistance (OR) are currently ongoing. However, 50% of resistance mechanisms that emerge upon therapy remain unknown highlighting the need to better understand the landscape of OR mechanisms to define alternative approaches. Modeling OR in vitro could help investigate uncovered mechanisms promoting resistance thus enabling to identify new biomarkers associated with loss of TKI sensitivity.

**Material and Methods**

To generate OR-cell lines, we plated and treated EGFR mutant cell lines (HCC-827 and HCC-4006) until we detected proliferation independently of drug exposure. To evaluate OR, we measured osimertinib IC50 at 72h of exposure via a luminescence-based assay and compared it to the corresponding parental cell line.

**Results and Discussions**

After ~15 weeks of treatment, we generated three and five OR-cell lines, from HCC-827 and HCC-4006 respectively, observing on average >120-fold higher IC50 in OR-cell lines compared to the parental ones. We investigated the presence of common OR mechanisms to focus on OR-cell lines lacking known OR mechanisms and excluded the presence of secondary EGFR and KRAS alterations via Sanger sequencing. Furthermore, we analyzed the EGFR downstream signaling pathway confirming that resistance to osimertinib in OR cell lines rely on off-target mechanisms of drug resistance.
We successfully established OR-cell lines that do not depend on mutant EGFR. Currently, we are evaluating the presence of gene amplification that may promote drug resistance via ddPCR (i.e., MET or ERBB2 amplification). Future analysis will focus on exploring enriched pathway expression and/or alterations in OR-cell lines compared to the parental cell lines via WES and RNA-seq.

**Conclusion**

By uncovering novel biomarkers linked to loss of TKI sensitivity, our studies will contribute to develop novel strategies to improve the outcomes of patients who no longer benefit from first-line osimertinib therapy.

### EACR2024-1017
### Loss of CDX1/CDX2 expression identifies cetuximab-resistant patients and offers novel therapeutic alternatives in metastatic colorectal cancer

*A. Rio Vilariño*[1], *M.I. Schlaepfer*[1], *L. García-Hernández*[1],

© 2024 The Authors. Molecular Oncology is published by John Wiley & Sons Ltd on behalf of Federation of European Biochemical Societies

J. García-Foncillas[1], A. Cebrián[1]
[1]Instituto de Investigaciones Sanitarias Fundación Jiménez Díaz, Translational Oncology, Madrid, Spain

**Introduction**

Despite the significant advancements in targeted therapies for RAS/RAF wild-type metastatic colorectal cancer patients, approximately 40% face primary resistance, underscoring the urgent need for novel biomarkers to guide treatment decisions and enhance survival rates.

**Material and Methods**

A bioinformatics analysis of the GSE59857 transcriptomic dataset, annotated for RAS/RAF status and cetuximab responsiveness in CRC cell lines, identified differentially expressed genes between sensitive and resistant groups. Candidate biomarkers, CDX1 and CDX2, were validated in patient datasets (GSE5851 and CPCT-02). Two cetuximab-sensitive and two primary-resistant CRC cell lines were chosen for evaluating CDX1 and CDX2 levels through western blot and RT-qPCR. Methylation analysis via pyrosequencing assessed CDX1 and CDX2 methylation in all cell lines. Forced expression of CDX1 and CDX2 was induced in resistant cell lines, and cell proliferation after cetuximab treatment was examined. Additionally, non-transduced resistant cells were treated with the demethylating agent Decitabine to assess its ability to restore CDX1 and CDX2 expression and cetuximab sensitivity. Immuno-histochemistry studied CDX1 and CDX2 in tumor samples with known anti-EGFR therapy responses.

**Results and Discussions**

Bioinformatic analysis identified CDX1 and CDX2 as significantly downregulated genes during primary cetuximab resistance. Validation in mCRC patient datasets (GSE58957 and CPCT-02) confirmed that low CDX1 and CDX2 expression correlated with anti-EGFR therapy resistance and significantly reduced progression-free survival post-cetuximab treatment. Resistant CRC cell lines and patients exhibited substantially lower CDX1 and CDX2 levels compared to sensitive counterparts. Overexpressing both genes, but not individually, restored cetuximab efficacy in resistant cell lines. Promoter hypermethylation was observed in both genes in resistant cell lines and hypomethylation in sensitive ones. Combining Decitabine pre-treatment with cetuximab reinstated efficacy along with CDX1 and CDX2 expression.

**Conclusion**

CDX1 and CDX2 expression and methylation emerge as promising predictive biomarkers for cetuximab resistance. The incorporation of demethylating agents presents a potential therapeutic strategy to restore cetuximab efficacy in this subset of patients.

### EACR2024-1038
### Unraveling the Role of Store-Operated Calcium Entry to Overcome Melanoma Drug Resistance

D.M. Vega Gutierrez[1], M. Tetsi Nomigni[1], K.L. Laius[2], B.A. Niemeyer[2], P. Antony[1], S. Kreis[1], S. Bréchard[1]
[1]University of Luxembourg, Department of Life Sciences and Medicine, Belvaux, Luxembourg
[2]Saarland University, Molecular Biophysics, Homburg, Germany

**Introduction**

Cutaneous melanoma (CM) is an aggressive skin cancer associated with UV-induced mutations in melanocytes, which occur in the BRAF and RAS genes in 50% and 25% of the cases, respectively. Treatment of CM in advanced stages is hindered by a rapid emergence of resistance to available therapies resulting in a poor overall 5-year survival rate for stage III/IV patients. Accumulating evidence indicates that store-operated $Ca^{2+}$ entry (SOCE) is a key oncogenic $Ca^{2+}$ signaling pathway driving cancer progression, however, its exact role and targetability in drug resistance is not yet defined. We propose here to investigate novel mechanisms regulated by or influencing SOCE in order to identify novel therapeutic options which, in combination with current treatment, could contribute to overcome melanoma drug resistance

**Material and Methods**

In this study, we used different melanoma cell lines with activating *BRAF*$^{V600E}$ *and NRAS* mutations. Drug-resistant melanoma cells were generated in our lab from parental cells by long-term exposure to targeted inhibitors. Experiments were performed in monolayer 2D and spheroid 3D models to strengthen the biological relevance of our data. Measurements of SOCE were recorded in drug sensitive and resistant melanoma cells. The role of SOCE in the regulation of gene expression and cytokine secretion was also analyzed in the different cell lines.

**Results and Discussions**

We show that SOCE is associated to the proliferation process by regulating the expression of melastatin $Ca^{2+}$ channel TRPM1. Moreover, SOCE is able to control cytokine production through the regulation of NF-kb factor as well as cytokine secretion. Down-regulation, in drug resistant melanoma cells, of key players of SOCE results to the dysregulation of TRPM1 expression and cytokine mobilization with the potential to shape the tumor immune landscape towards an anti-tumoral environment rendering tumors more sensitive to targeted and/or immunotherapies.

**Conclusion**

A better understanding of the role of SOCE/TRPM1 in the acquisition of drug resistance may facilitate the development of 2nd and 3rd line inhibitors to allow for more persistent tumor control by combining different treatments.

### EACR2024-1053
### Integrin-linked kinase supports drug-tolerant-persister cell survival and EMT-mediated drug resistance by upregulating YAP during EGFR TKI treatment of lung adenocarcinoma

R. Shi[1,2], D. Farnsworth[1,2], T. Atwal[1], J. Chow[1], R. Sheena[1], S. Dedhar[1], K. Bennewith[1,3], W. Lockwood[1,3]
[1]BC Cancer Research Institute, Integrative Oncology, Vancouver, Canada
[2]University of British Columbia, Interdisciplinary Oncology Program, Vancouver, Canada
[3]University of British Columbia, Pathology and

© 2024 The Authors. Molecular Oncology is published by John Wiley & Sons Ltd on behalf of Federation of European Biochemical Societies

*Laboratory Medicine, Vancouver, Canada*

**Introduction**

Lung cancer is the leading cause of cancer-related deaths world-wide. The 3rd-generation epidermal growth factor receptor (EGFR) tyrosine kinase inhibitor (TKI), osimertinib (Osi), has extensively improved patients outcomes. However, patients inevitably develop resistance against Osi. Epithelial-mesenchymal transition (EMT) is an established non-genetic resistance mechanism that also associated with increased metastatic potential of tumours. The progression towards EMT-mediated resistance exist a key transitory state of drug-tolerant-persistence (DTP), in which increased phenotypic plasticity permits survival during drug treatment. Integrin-linked kinase (ILK), an important regulator of integrin signaling, has been implicated in the pathogenesis of other cancers through the promotion of EMT. Moreover, a recent study in patients treated with EGFR-TKIs found that high ILK expression was correlated with worse prognosis. Therefore, we hypothesize that ILK may be important for DTP survival and EMT-mediated Osi resistance in EGFR mutant LUAD.

**Material and Methods**

Gene Set Enrichment Analysis was performed on LUAD patient databases. Genetic and pharmacological manipulations of ILK were used to regulate ILK's function. Cell viability and clonogenic assays were used to evaluate Osi sensitivity. Osi-resistant (OsiR) cells were made by dose-escalation. RNAseq and western blots were performed to assess expression levels. Confocal microscopy was used to evaluate YAP activity.

**Results and Discussions**

High ILK expression was found to be significantly correlated with an EMT expression signature in patients. ILK knockdown in HCC4006 cells, a line with high basal ILK expression, limited EMT progression and reduced the viability of DTP cells by impairing YAP activation, ultimately improving Osi response. Importantly, these ILK high DTP cells were able to persist long enough to acquire additional mutations that maintained their mesenchymal features and their insensitivity to Osi independent of ILK. Lastly, we show that pharmacological inhibition of ILK suppressed EMT and improved Osi response in LUAD cells, providing evidence that combination therapy of targeting ILK and EGFR is a feasible strategy in combating tumour persistence and resistance.

**Conclusion**

Our results show that ILK is important in promoting EMT and DTP survival during EGFR TKI treatment and that co-targeting ILK may help limit persistent tumour formation and drug resistance in lung cancer.

### EACR2024-1063
### Investigating the role of SGK signalling in prostate cancer

*D. Turnham[1], C. Green[1], A. Richards[1], T. Cunliffe[1], W. Van Veerden[2], G. Risbridger[3], W. Phillips[4], H. Pearson[1]*
*[1]Cardiff University, European Cancer Stem Cell Research Institute, Cardiff, United Kingdom*
*[2]Erasmus University Medical Center, Experimental Oncology, Rotterdam, United Kingdom*
*[3]Monash University, Monash Biomedicine Discovery Institute, Melbourne, Australia*
*[4]Peter MacCallum Cancer Centre, Department of Oncology, Melbourne, Australia*

**Introduction**

The PI3K/AKT/mTOR signalling pathway is frequently activated in prostate cancer (PCa), presenting a valuable therapeutic target. Despite a range of targeted therapies being explored clinically, therapeutic resistance is common owing to feedback loops that prevent complete suppression of the pathway. Thus, new approaches to overcome resistance to PI3K/AKT/mTOR directed therapies are needed. Serum-and glucocorticoid-regulated kinases (SGKs) have been shown to regulate AKT substrates in the presence of AKT inhibitors to facilitate resistance in breast cancer. Here, we aim to define the prostate-specific role of SGKs during normal tissue homeostasis, tumour progression and AKT-inhibitor resistance, with a view to identifying novel treatment strategies that aid patient care.

**Material and Methods**

Conditional Cre-LoxP technology was used to develop transgenic mice with *Sgk1* deletion specifically in prostate luminal epithelial cells (*PSACre-ERT2; Sgk1$^{fl/fl}$*), with and without additional *Pten$^{fl/fl}$* deletion. Mice were aged and prostate tissue analysed by IHC, western blot and proteomics. To assess the therapeutic potential of combined SGK/AKT inhibition a range of 2D/3D models were tested, before patient-derived xenograft (PDX) and transgenic mouse models were tested in vivo.

**Results and Discussions**

*Sgk1* depletion in murine prostate revealed Sgk1 is indispensable for normal prostate tissue homeostasis and associated with apoptosis evasion. In *Pten*-deficient PCa, *Sgk1* biallelic loss did not impact tumour burden. Additional analysis of SGK1 suppression in PCa cell lines demonstrated a significant reduction in colony formation, suggesting that SGK1 may also have a role in mediating stem/progenitor cell activity. Co-inhibition of AKT/SGK significantly reduced cell viability in PTEN-deficient human PCa cell lines and murine PCa organoids relative to either monotherapy or vehicle. Combined AKT/SGK inhibition in different mCRPC PDX models significantly reduced tumour growth compared to vehicle treatment irrespective of PTEN status. A reduction in tumour growth was also observed compared to AKT alone, which was associated with more potent suppression of AKT substrates.

**Conclusion**

SGK1 has a role in regulating apoptosis in prostate epithelial cells but is not required for PCa growth driven by PTEN loss. Co-targeting AKT and SGK is more efficacious in PTEN-deficient models, however further work is needed to identify biomarkers that predict response to AKT +/- SGK inhibitors and to determine the long-term benefits of this strategy.

### EACR2024-1090
### PPARγ agonist, Pioglitazone, in combination with bcr/abl TKI in patients of chronic myeloid leukemia in chronic

© 2024 The Authors. Molecular Oncology is published by John Wiley & Sons Ltd on behalf of Federation of European Biochemical Societies

### phase with suboptimal molecular response to Imatinib

H. Malhotra[1], L.M. Sharma[2], A. Yadav[3], N. Gupta[4]
[1]Sri Ram Cancer Center- Mahatma Gandhi Medical College Hospital-, Medical Oncology, Jaipur, India
[2]Mahatma Gandhi Medical College Hospital- Jaipur- India, Clinical Hematology, Jaipur, India
[3]Mahatma Gandhi Medical College Hospital- Jaipur- India, Medical Oncology, Jaipur, India
[4]Mahatma Gandhi Medical College Hospital, Clinical Hematology, Jaipur, India

**Introduction**

About 10 to 20% patients of Chronic Myeloid Leukemia in chronic phase (CML CP) have suboptimal molecular response (MR) to first line Imatinib maleate (IM) at the dose of 400 mg. once daily. Treatment options include IM dose increase or switch to a 2nd generation tyrosine kinase inhibitor (TKI). Synergy has been demonstrated when PPAR γ agonists are added to TKIs. We describe our initial results of 20 patients of CML CP with sub-optimal molecular response (bcr/abl ratio >10% at 6 months) treated with the combination of IM and pioglitazone, a PPAR γ agonist.

**Material and Methods**

A total of 172 patients of CML-CP, receiving first-line Imatinib 400 mg.OD, were prospectively studied. All patients had bcr/abl testing by standard RT-PCR with reporting on IS every 3 monthly. There were 110 males and 62 females with median age of 38 years (range 14 to 71). Majority of patients were intermediate/high Sokal risk. Twenty patients of CML CP with suboptimal MR (bcr/abl:abl RQ-PCR >10%) at 6 months of IM treatment were recruited in this study. Because of economic considerations, these patients were not candidates for 2nd gen. TKIs. Patients were given IM 400 mg/day and pioglitazone 30 mg/day. Institutional Ethics Committee permission was obtained before starting the trial. At the time of analysis, median follow-up was 18 months (range: 12 to 36 months)

**Results and Discussions**

Sixteen of the 20 patients achieved MR2 (bcr/abl <1.0) at 12 months, 5/16 achieving MR3 (bcr/abl <0.1); while 4/20 did not show optimal response and were advised a 2nd gen TKI. Treatment with pioglitazone was well tolerated. One patient was not analysed due to intolerance to pioglitazone after first dose. Two patients had grade 1 elevation of hepatic enzymes which returned to normal after one week treatment cessation. No patient reported hypoglycemia. No patients discontinued either drug because of adverse events.

**Conclusion**

We conclude that the combination of imatinib with pioglitazone is effective and well tolerated in patients with a sub-optimal MR to TKI in patients with CML-CP with a manageable toxicity profile. The combination could be a cost-effective strategy in treating imatinib suboptimal responders in the developing world. The combination needs further and larger trials for confirmation and more studies to evaluation of the mechanisms of the synergy.

### EACR2024-1123
### Generation of TAT-Cx43266-283- and temozolomide-resistant glioma stem cells

E. Jiménez-Madrona[1], L. García-Vicente[1], A. Álvarez-Vázquez[1], A. Tabernero[1]
[1]Instituto de Neurociencias de Castilla y León INCyL-Universidad de Salamanca, Instituto de Investigación Biomédica de Salamanca IBSAL, Biochemistry and Molecular Biology, Salamanca, Spain

**Introduction**

Glioblastoma is the most common malignant primary brain tumor. Despite maximum safe tumor resection, radiotherapy and adjuvant chemotherapy with temozolomide (TMZ), patients diagnosed with glioblastoma have a median survival of 10 to 15 months. Glioblastoma stem cells (GSCs) have the ability to withstand current treatment standards and self-renew, causing fatal relapses. Our lab designed the cell penetrating peptide TAT-Cx43266-283 (TAT-Cx43), which has shown promising results in preclinical GBM models, targeting GSCs and increasing glioblastoma-bearing mouse survival. Because tumor cells inevitably acquire resistance to most treatments, in this study we aim to develop and study TAT-Cx43- and TMZ-resistant GSCs.

**Material and Methods**

For this purpose, we used mouse GL261- and SB28-GSC subpopulations and treated them with growing concentrations of TAT-Cx43 (6.25 – 200 µM) and TMZ (25 – 1600 µM) for 6 to 8 passages (4-5 months). Dose-response was assessed in basal, TAT-Cx43- and TMZ-resistant GSCs. EC50 values were calculated at 72 and 144 h of treatment in at least 3 independent experiments.

**Results and Discussions**

Our results show that both SB28- and GL261-GSCs acquired resistance to TAT-Cx43 and TMZ after prolonged treatment with increasing concentrations. We are currently studying TMZ-resistant GSCs. Regarding TAT-Cx43-resistant cells, we found that EC50 values increased 5.36 (27.19 – 145.8 µM) and 1.96 times (62.19 – 121.8 µM) in TAT-Cx43-resistant compared to basal SB28-GSCs, at 72 and 144 h of treatment, respectively. The same trend was observed in GL261-GSCs. Thus, EC50 values for TAT-Cx43 increased 5.11 (34.36 – 175.5 µM) and 4.23 times (21.58 – 91.30 µM) in TAT-Cx43-resistant compared to basal GL261-GSCs, at 72 and 144 h of treatment, respectively. Interestingly, TAT-Cx43-resistant SB28-GSCs seemed to be more sensitive to TMZ. Thus, EC50 values for TMZ decreased 1.80 (2032 – 1126 µM) and 1.29 times (891.9 – 690.3 µM) in TAT-Cx43-resistant compared to basal SB28-GSCs at 72 and 144 h, respectively. Surprisingly, EC50 values for TMZ decreased 7.37 (6606 – 896.5 µM) but increased 6.54 times (47.89 – 313.6 µM) in TAT-Cx43-resistant compared to basal GL261-GSCs at 72 and 144 h, respectively. Although these results are intriguing, they should be interpreted cautiously, since non-lineal adjustment was not optimal for TMZ-EC50 in GL261-GSCs.

**Conclusion**

We generated TAT-Cx43- and TMZ-resistant SB28- and GL261-GSCs by exposing them to increasing concentrations of treatment for extended periods of time. Our results show that TAT-Cx43 might sensitize SB28-GSCs to TMZ. However, this trend of TMZ sensitization

© 2024 The Authors. Molecular Oncology is published by John Wiley & Sons Ltd on behalf of Federation of European Biochemical Societies was not reliably shown in TAT-Cx43-resistant GL261-GSCs. Furter studies are required to confirm these results and test the potential benefits of TMZ and TAT-Cx43 combination.

*COI: TAT-Cx43266–283 is a patent (ID: ES2526109B1) from the University of Salamanca*

### EACR2024-1145
### POSTER IN THE SPOTLIGHT
### Behave or die: Overactivation of oncogenic signaling steers cancer cells toward less malignant phenotypes

*M. dos Santos Dias[1], C. Papagianni[1], A. Friskes[2], S. Wang[3], J.M. Fernandes Neto[1], F. van Gemert[4], S. Mourragui[5], A. Villanueva[6], R. Medema[2], R. Bernards[1]*
*[1]The Netherlands Cancer Institute, Division of Molecular Carcinogenesis, Amsterdam, The Netherlands*
*[2]The Netherlands Cancer Institute, Division of Cell Biology, Amsterdam, The Netherlands*
*[3]Shanghai Cancer Institute, State Key Laboratory of Oncogenes and Related Genes, Shanghai, China*
*[4]The Netherlands Cancer Institute, Division of Tumor Biology & Immunology, Amsterdam, The Netherlands*
*[5]Hubrecht Institute-KNAW, University Medical Center, Utrecht, The Netherlands*
*[6]Catalan Institute of Oncology ICO, Chemoresistance and Predictive Factors Group, Barcelona, Spain*

**Introduction**
Multiple targeted inhibitors of oncogenic signaling have been developed in the last decades. Unfortunately, long-lasting control of advanced cancers with these agents remains virtually elusive due to the emergence of resistance. The pathological oncogenic signaling in cancer cells results in multiple cellular stresses and demands an increased mobilization of stress response pathways to keep cancer cells viable. We investigated here how the therapeutic overactivation of oncogenic signaling can be combined with drugs targeting stress response pathways to kill cancer cells. Furthermore, cancer cells often acquire resistance to targeted therapies by gaining further oncogenic alterations and becoming more aggressive. Given the opposite mechanism of toxicity, we hypothesized that resistance to the overactivation of oncogenic signaling should select for suppression of deregulated oncogenic pathways to evade the stressful state

**Material and Methods**
We used the Protein Phosphatase 2A (PP2A) inhibitor LB-100 to overactivate oncogenic signaling in colon cancer cells; compound and CRISPR screens to identify synthetic lethalities that were also validated in pancreatic and cholangiocarcinoma cancer cell panels; live-cell microscopy, DNA combing, and flow cytometry to address the mechanisms of toxicity; orthotopic PDX models to address the therapeutic window *in vivo;* and a multi-omics approach to investigate the phenotype of resistant cells

**Results and Discussions**
We found that LB-100 hyperactivates multiple oncogenic pathways, engaging stress responses and restraining the viability of colon cancer cells. Sublethal doses of LB-100 and the Wee1 inhibitor adavosertib combine to collapse DNA replication and trigger premature mitosis followed by cell death. The combination was synergistic in multiple cancer models in vitro and well tolerated in vivo, suppressing the growth of patient-derived colon tumors. Remarkably, acquired resistance to this drug combination in colon cancer cells led to a reduction of aneuploidy, transcriptional suppression of oncogenic signaling pathways, and increased expression of tumor suppressor genes. Consequently, acquired resistance to the combination restrained the ability of colon cancer cells to form tumors in vivo

**Conclusion**
These data show that overactivation of oncogenic signaling targets the pathological behavior of cancer cells and can be explored therapeutically. Moreover, the data suggest that this approach may force cancer cells to give up malignant traits and “behave” in order to survive

### EACR2024-1181
### POSTER IN THE SPOTLIGHT
### Proteasomal inhibition enhances the efficacy of BCMA-targeting CART-cell therapy in multiple myeloma by preventing BCMA degradation

*L. Rieger[1,2], K. Irlinger[1,2], F. Fuechsl[1,2], L. Pfeuffer[2,3], M. Tietje[1,2], L. Besse[4], P. Giansanti[2,5], J. Hecker[1,2], A. Krackhardt[2,6], F. Bassermann[1,2,6]*
*[1]University Hospital rechts der Isar of the TU Munich, Department of Oncology and Hematology, Munich, Germany*
*[2]Technical University Munich, TranslaTUM- Center for Translational Cancer Research, Munich, Germany*
*[3]TUM School of Medicine and Health-Technical University of Munich, Institute of Clinical Chemistry and Pathobiochemistry, Munich, Germany*
*[4]Kantonsspital St Gallen, Department of Medical Oncology and Hematology, Sankt Gallen, Switzerland*
*[5]University Hospital rechts der Isar of the TU Munich, Bavarian Center for Biomolecular Mass Spectrometry, Munich, Germany*
*[6]DKTK, German Cancer Consortium of Translational Cancer Research, Heidelberg, Germany*

**Introduction**
Despite initial high response rates of immune cell therapies in multiple myeloma (MM) including chimeric antigen receptor T-cell (CART) targeting the B-cell maturation antigen (BCMA), most patients eventually relapse. This highlights the need to enhance therapy efficacy. Although loss of BCMA expression marks one reason for therapy failure, little is known about the mechanistic means of antigen escape. Here we investigate the ubiquitin-proteasome system (UPS) in the regulation of BCMA expression in MM.

**Material and Methods**
BCMA regulation by the UPS was assessed using half-life analyses, proteasomal inhibition (PI) and immunoblotting. Primary $CD138^+$ cells were obtained from bone marrow aspirations and MACS® cell separation. BCMA ubiquitination was confirmed by tandem-ubiquitin binding entities (TUBE) assays and image-based analysis. Fractionation was used to assess plasma membrane bound BCMA levels, and antigen surface presentation was determined by FACS analysis.

© 2024 The Authors. Molecular Oncology is published by John Wiley & Sons Ltd on behalf of Federation of European Biochemical Societies

Co-culture assays were used to determine the effect of PI on CART cell efficacy in vitro and MM xenograft models were used in vivo. Patients were assessed for the reduction in free light chain levels, sBCMA, and $BCMA^+$ cells in the bone marrow.

**Results and Discussions**

In this study, we established that BCMA is a short-lived protein. Its degradation was abrogated upon PI treatment, proposing a role of the UPS the BCMA degradation. Plasma membrane localized BCMA was ubiquitylated in MM cells and K48-linkage specific poly-ubiquitylation of BCMA lead to proteasomal degradation. This process was increased upon stimulation of MM cells with the BCMA ligand April. In both, MM cell line models and primary $CD138^+$ cells of PI refractory MM patients, PI induced BCMA cell surface accumulation enhanced BCMA directed CART cell efficacy in co-culture models. Moreover, combined PI and BCMA CART-cell treatment led to a decrease in tumor burden in vivo and MM patients refractory to BCMA-targeted CART-cell therapy, but with residing effector cells, responded with a in decrease in serum free light chains and a decrease in $CD138^+/BCMA^+$ MM cells.

**Conclusion**

Here we show that BCMA is ubiquitylated and degraded by the UPS in MM. PI-induced increase of BCMA enhances BCMA-targeting CART cell efficacy in vitro and in vivo. This opens a rationale for combinatorial PI/CART therapy for patients refractory to BCMA-targeted immunotherapy and highlights the possibility to exploit the UPS to prevent BCMA antigen escape.

### EACR2024-1184
### Unraveling mechanisms of TKI resistance in NSCLC: insights from PDX models

*N. Mfonfu[1,2], F. Braye[1], G. Boldina[2], A. Yurchenko[1], L. Friboulet[1], S. Nikolaev[1], M. Cesaroni[2]*

*[1]Gustave Roussy, Molecular predictors and new targets in oncology, Villejuif, France*

*[2]Sanofi, Precision Medicine and Computational Biology, Vitry-sur-Seine, France*

**Introduction**

Targeted therapies like tyrosine kinase inhibitors (TKIs) targeting EGFR and ALK are often first-line treatment for Lung Cancer. However, after initial response, rare drug-tolerant cancer cells (persister cells) frequently remain and lead to the emergence of TKI resistance. Characterisation of the population of persister cells and investigation of the mechanisms of treatment evasion may improve understanding of the mechanisms of TKI resistance.

**Material and Methods**

Two patient derived PDX models from pretreated cancers were generated. One PDX model had EGFR and ALK mutations and responded to treatment with Osimertinib and Alectinib, and the other – had ALK mutations, and responded Lorlatinib. Each tumor was grafted into 5 mice, 3 of which were treated till emergence of the stable residual disease, and 2 – used as a control without treatment. snRNA-seq was performed to characterize in comparative framework the untreated and persister cancer cells.

**Results and Discussions**

Majority of persister cells were in the G1 phase of the cell cycle and suppressed most canonical cancer pathways, however a notable upregulation of the ciliated pathway was observed in both models. Transcription factors MEOX2 and MLX were identified as activated, indicating their roles in resistance mechanisms and inhibition of proliferation, cell growth, and mobility. Furthermore, distinct mechanisms were uncovered in different tumor models: the ALK PDX model exhibited enrichment of Epithelial-Mesenchymal Transition (EMT) signatures in Persister cells, while the EGFR ALK PDX model showed suppression of canonical pathways like MYC and Interferon responses.

**Conclusion**

PDX models proved to be an informative tool to study cancer persistence. TKI treatment evasion mechanisms in lung cancer might be intermediated by activation of the ciliated pathway.

### EACR2024-1186
### Unraveling the mechanisms leading to acquired resistance after treatment with immune checkpoint inhibitors

*A.Z. Johansen[1], H. Linder[1], C.Ø. Madsen[1], L. Grøntved[2], D.H. Madsen[1]*

*[1]National Center for Cancer Immune Therapy, Department of Oncology- Copenhagen University Hospital – Herlev and Gentofte, Herlev, Denmark*

*[2]Department of Biochemistry and Molecular Biology, University of Southern Denmark, Odense, Denmark*

**Introduction**

Cancer immunotherapy, particularly immune checkpoint inhibitors (ICIs), has become a cornerstone for treating various cancers by 'releasing the brakes' on T cells, allowing a better cancer-killing response. Despite the remarkable success of ICIs resulting in unprecedented durability of responses, acquired resistance leads to disease progression in initially responsive patients. Understanding the mechanisms of acquired resistance is a key challenge to lowering the number of patients who acquire resistance to ICIs.

**Material and Methods**

Mice inoculated with the immunogenic MC38 colon cancer cell line were treated with an ICI combination consisting of anti-CTLA-4 and anti-PD-1 antibodies. Distinct growth patterns, including complete response, acquired resistance, and primary resistance, were characterized based on tumor regression and survival metrics. Verification of resistance involved engrafting tumor fragments or FACS-sorted cancer cells into naïve mice, replicating the ICI treatment. Mechanistic insights into acquired resistance were gained through comprehensive assessments, including RNAseq, FACS analysis, cytotoxicity assays, and metabolic profiling.

**Results and Discussions**

Treatment with anti-PD-1 and anti-CTLA-4 antibodies revealed diverse response patterns - complete regression, acquired resistance, and primary resistance - despite the use of a homogeneous cancer cell line. Resistance verification through engraftment of tumor fragments or cancer cells from ICI-treated tumors with acquired resistance further validated the model. Classical mechanisms such as loss of IFNγ sensitivity and

© 2024 The Authors. Molecular Oncology is published by John Wiley & Sons Ltd on behalf of Federation of European Biochemical Societies downregulation of antigen presentation machinery were not implicated in the development of acquired resistance. Instead, TNFα signaling via NF-κB, epithelial-mesenchymal transition, and hypoxia signatures were observed in cancer cells from tumors with acquired resistance. Additionally, these cancer cells exhibited elevated mitochondrial respiration rates.

**Conclusion**

The syngeneic mouse model provided valuable insights into acquired resistance to ICIs. Acquired resistance was validated by the observation of primary resistance upon tumor fragment and cancer cell engraftment into naïve mice. The identified characteristics associated with altered metabolism, ECM remodeling, and hypoxia highlight potential targets to mitigate or reverse immunosuppression during the development of acquired resistance to ICIs.

### EACR2024-1201
### SRPK1: a novel mechanism for tumour chemoresistance

*D. Duzgun*[1], *S. Oltean*[2]
[1]*University of Exeter, Department of Clinical and Biomedical Science, exeter, Turkiye*
[2]*University of Exeter, Department of Clinical and Biomedical Sciences, Exeter, United Kingdom*

**Introduction**

Resistance to chemotherapeutic drugs is a major setback in cancer therapy, which leads to a high proportion of relapses and poor survival outcomes in cancer patients. Chemoresistance is frequently elicited by abnormal alternative splicing (AS), regulated by crucial kinases such as the serine-arginine protein kinase 1 (SRPK1). While SRPK1 has been implicated recently in chemo-resistance in several tumours, the molecular mechanisms of this process are not known. In the present study, we aimed to investigate whether chemotherapeutic drugs affect the expression and function of SRPK1 in various cancer cells as well as the mechanism through which SRPK1 is involved in chemoresistance.

**Material and Methods**

A cell culture technique was used to establish the drug-resistant cell lines from their parental cells. Molecular and cellular biological techniques including MTT assay, immunofluorescence, western blotting, gene knockdown, immunoprecipitation, and RT-PCR were used to characterize in the drug-resistant cell lines.

**Results and Discussions**

We selected two cisplatin (CDDP)-resistant cell lines (breast cancer MDA-MB-231R and colon cancer HCT-116R) and a docetaxel (DTX)-resistant (prostate cancer PC-3R) cell line by continuous exposure (over a period of 6 months) of PC-3, MDA-MB-231 and HCT-116 cells to sub-lethal, stepwise increasing concentrations of drugs. As indicated by IC50 value, drug-resistant cells were about five to seventeen times more resistant to drugs than parental lines. Immunoblotting showed that while there was a decrease in SRPK1 protein level in CDDP-treated HCT-116 cells, SRPK1 expression was upregulated by the DTX in PC-3 cells. Interestingly, SRPK1 expression was not affected by the treatment in all resistant cell lines, but was generally higher in MDA-MB-231R and PC-3R than their parental lines, and lower in HCT116R than HCT-116. Also, IC50 values significantly decreased in PC-3R and MDA-MB-231R cells with knockdown of SRPK1, while its downregulation showed markedly increase in IC50 in HCT-116R, implying that the expression level of SRPK1 may be major determinant of chemotherapeutic drugs responsiveness. To confirm the resistance status of the derived cell lines, immunostaining of phosphorylated H2AX (pH2AX) showed that while CDDP dramatically increased pH2AX in MDA-MB-231 and HCT-116 cells, but not in MDA-MB-231R and HCT116R, it was potently switched on by the addition of SPHINX31 in MDA-MB-231R, but not HCT-116R. In parallel study, we also performed immunostaining analysis in PC-3 cells to assess the role of class-III- β tubulin in DTX-resistance. We found that class-III β tubulin was overexpressed in PC-3R cells and siRNA knockdown of SRPK1 showed that a dramatic decrease in class-III β tubulin, which may be a target for over-coming resistance to DTX. As marker of apoptosis, CDDP-induced and DTX-induced activation of caspase-8, caspase-3 and cleavage of PARP was marked in PC-3 and MDA-MB-231 cells, but not in PC-3R and MDA-MB-231R ones. Additionally, combination treatment increased cleavage of caspase -8, caspase-3 and PARP in PC-3R and MDA-MB-231R cells compared with DTX, CDDP and SPHINX31 alone conditions; the opposite effects were observed in SRPK1-downregulated HCT-116R cells. These results suggested that inhibiting SRPK1 expression restored the sensitivity of drug-resistant cells and the anti-apoptotic effect of SRPK1 are related to its expression levels. We further studied the splicing of two members of the BCL2L1 (Bcl-x). For Bcl-x genes, the long isoform (Bcl-xL) possesses the anti-apoptotic activity while the short isoform (Bcl-xS) function as pro-apoptotic factor. By RT-PCR, we found that the relative abundance of Bcl-xS was increased by docetaxel in PC-3 but decreased in PC-3R in a dose-dependent manner. Our results suggest that SRPK1-mediated AS of Bcl-X Is involved in promoting DTX resistance in PC-3R cells. Collectively, these results indicated that aberrant SRPK1 expression induces CDDP and DTX resistance by inhibiting apoptosis in various cancer cells.

**Conclusion**

Our research could reveal a key role of SRPK1 in the development of drug resistance in various cancer cells, suggesting a potential therapeutic avenue for overcoming chemotherapy resistance.

### EACR2024-1208
### AKR1C3: the way to overcome drug resistance in osteosarcoma models

*C. Tucciarello*[1], *G. Giordano*[2], *S. Oliaro Bosso*[3], *D. Boschi*[4], *M. Lolli*[4], *C. Peraldo Neia*[5], *A. Pippione*[4], *Y. Pignochino*[6]
[1]*IRCCS- Insitute for Cancer Research and Treatment, Department of Clinical and Biological Sciences-University of Turin- Orbassano- TO- Italy- Department of Oncology- University of Turin- TO- Italy, Candiolo-Turin, Italy*
[2]*Candiolo Cancer Institute- FPO-IRCCS- Candiolo- TO-Italy-, Department of Oncology- University of Turin- TO-*

© 2024 The Authors. Molecular Oncology is published by John Wiley & Sons Ltd on behalf of Federation of European Biochemical Societies

Italy-, Turin- TO- Italy-, Italy
[3]Univeristy of Turin, Department of Drug Science and Technology of University of Turin, Turin- TO, Italy
[4]University of Turin, Department of Drug Science and Technology of University of Turin, Turin- TO, Italy
[5]University of Turin, Genomic Lab- Fondazione Edo ed Elvo Tempia Valenta, Turin- TO, Italy
[6]Candiolo Cancer Institute- FPO-IRCCS- Candiolo- TO-Italy, Department of Clinical and Biological Sciences-University of Turin- Orbassano- TO- Italy-, Turin- TO, Italy

**Introduction**

Osteosarcomas (OSs) are rare tumors of mesenchymal origin. Polychemotherapy regimens are the standard treatment for advanced OSs however drug resistance is a major challenge. The aldo-keto reductase 1 C3, (AKR1C3) is involved in the metabolism of several drugs among which doxorubicin (DOX). We explored AKR1C3 as a potential target to overcome drug resistance in OS.

**Material and Methods**

Four OS cell lines (HOS, KHOS, MG63, SAOS2) and two non-tumor ones (MRC5, HaCaT) were purchased from ATCC and culture as recommended. AKR1C3 was tested by western blot. Single-cell clone expansion of HOS and MG63 were subdued to increasing concentration of DOX, starting from suboptimal concentration, to obtain resistant clones. Cell viability and cell growth were tested after 72 hour treatment with AKR1C3 inhibitors (cpd1-6) both as single agents and in combination with DOX (CB). Apoptosis was measured after annexin V-PI staining by flow cytometry analysis.

**Results and Discussions**

AKR1C3 is variably expressed by OS cells (SAOS2 showed the highest expression and HOS the lowest one) and not in healthy control. At 3.125-6.25 µM, the cpds tested are non-toxic (cell viability >60%) on healthy cell lines and show a AKR1C3 expression-correlated response on OS cells, displaying synergistic effects (CI<1) when combined with DOX. Cell growth assays show a statistically significant decrease, up to 8-fold on SAOS-2, and 3-fold on HOS, of the area occupied by OS cells when treated with the CB if compared to DOX alone. Among all, one compound (cpd2) showed synergistic effect on all cell lines, with an IC50 of 3.6 µM and 6.2 µM in HOS and SAOS2, respectively. So we tested it on Dox resistant models. A 3-fold decrease in cell viability was observed in both HOS and SAOS2 resistant cells treated with cpd2:Dox CB, if compared to DOX alone. The mechanism behind synergistic effect lead to apoptosis induction.

**Conclusion**

AKR1C3 inhibitors improve DOX effect in vitro. Targeting AKR1C3 is a good strategy to overcome drug resistance in OSs and deserve further investigation in vivo.

### EACR2024-1220

### Unraveling the secretome of pancreatic stellate cells to improve pancreatic cancer drug response

*R. Rebelo[1], C. Xavier[2], E. Giovannetti[3], M.H. Vasconcelos[4]*
[1]i3S - Institute for Research and Innovation in Health/ Cancer Center Amsterdam- Amsterdam-UMC, Cancer Drug Resistance/Medical Oncology, Porto, Portugal
[2]i3S - Institute for Research and Innovation in Health, Cancer Drug Resistance, Porto, Portugal
[3]Cancer Center Amsterdam, Medical Oncolgy, Amsterdam, The Netherlands
[4]i3S - Institute for Research and Innovation in Health, Cancer Drug Resistance Group, Porto, Portugal

**Introduction**

Pancreatic ductal adenocarcinoma (PDAC) is a highly desmoplastic cancer characterized by one of the lowest survival rates and notable chemoresistance features. Pancreatic stellate cells (PSCs) and cancer-associated fibroblasts (CAFs) are key modulators in desmoplasia, and their secretome participates in different oncogenic processes in PDAC. Several pro-tumorigenic factors have been recently found in the secretome of PSCs and CAFs, either as soluble proteins or associated with Extracellular Vesicles (EVs). The purpose of this study is to identify mediators of PDAC drug resistance within the secretome of PSCs, particularly in their EVs.

**Material and Methods**

For this, we selected the human primary pancreatic stellate cell line HPaSteC and the human dermal fibroblast cell line HFF-1 (negative control). EVs secreted by these cell lines were isolated by differential ultracentrifugation and characterized using transmission electron microscopy, nanoparticle tracking analysis, and Western blot (WB) testing for EV biomarkers. An analysis of the proteome of the EVs was conducted using liquid chromatography-mass spectrometry and is being further validated by WB.

**Results and Discussions**

The EVs isolated from HPaSteC and HFF-1 cells present the expected morphology (cup-shape) and a size typical of small EVs. EVs markers LAMP1 and Annexin XI were detected in both EVs samples. Moreover, ApoB, actinin-4, and GRP78 were not enriched in the isolated EVs compared to respective cells. HFF-1 and HPaSteC EVs' samples show different proteomic profiles. Numerous proteins (~454) were found differently expressed in HPaSteC EVs compared to HFF1 EVs. GO Slim and STRING analysis show an enrichment of proteins involved in important biological processes (e.g., regulation, metabolism, establishment of cellular localization), and a significant enrichment in trans-membrane proteins and glycoproteins. Ongoing studies are validating the identified proteins in another human PSC line (RLT-PSC). Using data from The Cancer Genome Atlas, the expression of proteins identified is also being correlated with PDAC patients' overall survival.

**Conclusion**

Future work will assess the contribution of PSCs-derived EVs on the viability and drug response in PDAC 2D and 3D cell models. Furthermore, the prognostic values of the top targets will be evaluated in clinically annotated specimens from PDAC patients. With this work, we hope to identify key proteins involved in PSCs-mediated drug response in PDAC, with potential as biomarkers and therapeutic targets.

© 2024 The Authors. Molecular Oncology is published by John Wiley & Sons Ltd on behalf of Federation of European Biochemical Societies

### EACR2024-1247
### Potential Role of TXNIP as a mediator of FoxO3a antitumor activity in Tamoxifen-sensitive and Resistant Breast Cancer Cells

C. Morelli[1]
[1]*University of Calabria, Department of Pharmacy-Health and Nutritional Sciences, Rende, Italy*

**Introduction**

Resistance to endocrine therapy is one of the main obstacles in the treatment of estrogen receptor α positive (ER+) breast cancer (BC) patients. The transcription factor Forkhead box class O 3 (FoxO3a) acts as an oncosuppressor and seems to be a positive prognostic marker in ER+ and Tamoxifen resistant BC. Thioredoxin interacting protein (TXNIP) is a tumour suppressor gene whose levels are frequently low in several cancers, including BC, and its downregulation is associated to a poor prognosis. Since a strong positive correlation between FoxO3a and TXNIP in ER+ BC patients has emerged from large-scale gene expression data sets, aim of the study is to assess TXNIP involvement in mediating FoxO3a antitumor activity in ER+ BC cells (BCCs) and in their Tamoxifen resistant (TamR) counterparts.

**Material and Methods**

ER+ MCF-7 BCCs and the derived TamR cells, developed through prolonged exposure to Tam, were employed as experimental models. Data from TamR cells were also confirmed in TamR/TetOn-AAA BCCs, a doxycycline (Dox) inducible system, expressing the constitutively active FoxO3a gene, and the relative control TamR/TetOn-V. Western blot (WB), qRT-PCR, siRNA techniques, Chromatin Immunoprecipitation (ChIP), cell proliferation, migration, and invasion assays were used to assess the functional interaction between FoxO3a and TXNIP.

**Results and Discussions**

FoxO3a overexpression increased TXNIP mRNA and protein levels in both Tam-sensitive and -resistant BCCs, and FoxO3a silencing led to its decrease, demonstrating that TXNIP is transcriptionally regulated by FoxO3a in BC. ChIP experiments evidenced a significant recruitment of FoxO3a on the TXNIP promoter region containing a Forkhead Responsive Element (FHRE) motif in MCF-7, TamR cells and in Dox-inducible stable cell lines, further showing FoxO3a involvement in TXNIP gene regulation. Moreover, TXNIP silencing abrogated the inhibitory effects of FoxO3a on cell proliferation, motility and invasiveness in MCF-7 and TamR BCCs, highlighting the role of TXNIP in mediating FoxO3a antitumor activity.

**Conclusion**

Taken together, our data show how TXNIP regulation represents an additional mechanism through which the oncosuppressor FoxO3a exerts its inhibition on ER+ BCC growth and progression, reinforcing the idea that FoxO3a could represent a suitable target to be exploited in the treatment of ER+ tumours, including those resistant to endocrine therapies.

### EACR2024-1261
### Identification of resistance mechanisms to the combination of SHP2 + ERK inhibitors and SHP2 + RAS-Multi-ON inhibitors in KRAS-mutant pancreatic cancer

*A. Mulero-Sánchez[1], A. Kanhailal[1,2], A. Bosma[1], B. Visuvasam[1], C. Lieftink[1], R. Beijersbergen[1], F. Opdam[2], R Bernards[1], S. Mainardi[1]*
[1]*Oncode Institute, Division of Molecular Carcinogenesis, The Netherlands Cancer Institute, Amsterdam, The Netherlands*
[2]*Department of Medical Oncology, The Netherlands Cancer Institute - Antoni van Leeuwenhoek, Amsterdam, Netherlands*

**Introduction**

Over 90% of pancreatic tumors carry a driving mutation in the KRAS oncogene, which is also altered in 30-40% of lung and colorectal cancers, thus representing an ideal target for precision oncology. Nevertheless, inhibitors targeting the most frequent KRAS mutants identified in pancreatic ductal adenocarcinoma (PDAC) are not clinically available yet. We previously discovered, using experimental mouse models, that tumors carrying activating KRAS mutations are sensitive to the inhibition of SHP2, a phosphatase helping the transmission of the growth-promoting signals from the cell surface receptors to RAS. Moreover, SHP2 inhibitors cooperate with inhibitors of the RAS downstream effectors MEK and ERK to achieve superior disruption of the MAPK pathway, increased apoptosis and better tumor growth control, in the absence of in vivo toxicity. Based on those compelling results, we begun to investigate the combination of the SHP2 inhibitor RMC4360 with the ERK inhibitor LY3214996, in a Phase I/Ib clinical trial (SHERPA, SHP2 and ERK inhibition in pancreatic cancer, NCT04916236). In the meantime, we would like to anticipate possible mechanisms of resistance, both to the described combination, as well as to the combination of SHP2 inhibitors with the more recent Multi-RAS-ON inhibitor RMC6236.

**Material and Methods**

In order to identify dynamic biomarkers of resistance, we performed a series of unbiased CRISPR-based resistance screenings, and generated PDAC cell lines that are spontaneously resistant both to the combination of SHP2+ERK inhibitors and SHP2+Multi-RAS-ON inhibitors. Functional experiments including over-expression, knockdown or pharmacological inhibition of candidate mediators of resistance have been conducted to validate the putative hits.

**Results and Discussions**

The CRISPR-based resistance screenings led to the identification of mTOR and c-JUN hyperactivation as two mechanisms allowing pancreatic cancer cells to overcome the pharmacological complete suppression of the MAPK pathway. In parallel, monoclonal spontaneous resister cell lines also demonstrated increased mTOR pathway and contemporary c-JUN activation. Finally, we begun to mechanistically elucidate the interconnection between the mTOR and JUN pathways and how they cooperate to drive resistance to MAPK inhibition.

**Conclusion**

Alteration of nodes in the PI3K/AKT/mTOR and in the c-JUN pathway may serve as markers for sensitivity/

© 2024 The Authors. Molecular Oncology is published by John Wiley & Sons Ltd on behalf of Federation of European Biochemical Societies resistance to the drug combination investigated in the SHERPA trial, as well as to the possible alternative combination of SHP2 + Multi-RAS-ON inhibitors.

### Epigenetics

### EACR2024-0123
### Dynamic expression of miR-7974 favors colorectal cancer growth and metastasis

*Y.T. Chen*[1], *B.C.M. Tan*[1], *H. Liu*[2]
[1]*Chang Gung University, Department of Biomedical Sciences, Taoyuan, Taiwan*
[2]*Chang Gung University, Department of Cell and Molecular Biology, Taoyuan, Taiwan*

**Introduction**
Colorectal cancer (CRC) is one of the most commonly diagnosed malignancies, and whose frequent metastasis and recurrence leads to high mortality rate. MicroRNAs (miRNAs) are small noncoding RNAs, mediating target mRNA degradation and translational depression, to regulate biological process and carcinogenesis mechanism. Consequently, we reviewed the CRC miRNome and TCGA profiles to search novel miRNAs associated with CRC malignancy, and performed experimental validations to decipher their oncogenic activity and the underlying molecular regulations.
**Material and Methods**
Sequencing profiles from in-house data and TCGA were integrated to search CRC-associated expressed miRNAs. In vitro cell-based assays and xenograft model were performed to investigate the association of target miRNA with cancer-associated abilities. Luciferase reporter assays and rescuing experiments were conducted to decipher the molecular interplays and functional consequences.
**Results and Discussions**
CRC miRNome profiling uncovered the tumor-biased expression of miR-7974, and relevant experiments recognized its targeting CDKN1A expression to promote cancer growth. Intriguingly, we concurrently found the lower miR-7974 expression of late-stage tumors comparing to early-stage tumors, and miR-7974 expression surprisingly predicted unfavorable CRC patient survival. Likewise, miR-7974 was verified to suppress metastasis by inhibiting MYO1E expression. We surmised that lower miR-7974 expression in metastatic CRC alternatively enhanced metastatic potential. This hypothesis was supported by the lower miRNA expression of metastasis-derived SW620 cells compared to the primary-derived SW480 cells. Further, Transwell-based developing migration cell model provided consistent results to link the variable miR-7974 expression to CRC metastatic potential. That is to say, upregulated miR-7974 expression in early-stage cancers promotes CRC growth and expansion, while the low miR-7974 expression in advanced cancers alternatively displayed higher metastatic potential.
**Conclusion**
Our findings demonstrate the dynamic miR-7974 expression, coupling target mRNA repression and derepression, provides a favorable outcome for cancer cells acquiring proliferative and metastatic potentials. This miRNA delivering stage-dependent molecular regulation illustrates the significance of miRNome during CRC development and progression, and further reveals whose translational potential in CRC diagnosis and prognosis.

### EACR2024-0239
### Uncovering the role of epigenetics in LACTB's tumor suppressive landscape

*G.M. Rodriguez Gomez*[1], *J.M. Gonzalez-Morena*[1], *C.W. Phang*[1], *K. Čížek*[2], *P. Jakoubě*[1], *Z. Kečkéšová*[1]
[1]*Institute of Organic Chemistry and Biochemistry, Biochemistry and Molecular Biology, Prague, Czech Republic*
[2]*Institute of Organic Chemistry and Biochemistry, Mass Spectometry, Prague, Czech Republic*

**Introduction**
Tumor suppressors are proteins which act as "brakes" to prevent oncogenic mechanisms, and their dysregulation through mutations and/or inactivation, contributes to the development of cancer. Several tumor suppressors like P53, BRCA1-2, PTEN, etc, have been described in human cells and their mechanism of action uncovered fundamental molecular pathway dependencies of cancer cells, the knowledge of which is being used in the development of effective cancer treatments. β-lactamase-like protein (LACTB) is a recently discovered mitochondrial tumor suppressor protein operating in many different types of tissues. It has been shown that LACTB can downregulate pro-survival pathways in cancer cells such as Akt and Wnt/β-catenin, can induce cell cycle arrest, apoptosis, differentiation as well as induce autophagy and suppress epithelial-mesenchymal transition. However, the specific upstream circuits involved in the regulation of these LACTB-induced mechanisms are currently unknown.
**Material and Methods**
In this study, we employed proximity biotinylating *in vivo* screening, coimmunoprecipitation and proximity ligation assays with a wide range of additional molecular biology techniques. This led to the identification of several mitochondrial proteins to be involved in LACTB biology in breast cancer models.
**Results and Discussions**
One of the proteins that interact with and binds LACTB is the mitochondrial serine hydroxymethyl transferase 2 (SHMT2), which catalyzes the conversion of serine to glycine. We found that LACTB expression leads to downregulation of SHMT2, and this downregulation does not occur at a transcriptional level. Since LACTB contains a catalytic domain, our results suggest that LACTB might promote enzymatic degradation of SHMT2 through their interaction. Additionally, we found that LACTB -induced decrease of SHMT2 promotes a decrease in the levels of intracellular glycine. During the conversion of serine to glycine, SHMT2 generates a byproduct called 5,10 methylenetetrahydrofolate. This byproduct is a methyl donor that can fuel the production of activated methyl groups (S-adenyl methionines), which are essential in the regulation of epigenetic mechanisms, such as DNA methylation. In this way, we

© 2024 The Authors. Molecular Oncology is published by John Wiley & Sons Ltd on behalf of Federation of European Biochemical Societies found that the decrease in SHMT2 induced by the overexpression of LACTB leads to decrease in the global DNA methylation.

**Conclusion**

Together, our findings reveal that the LACTB-SHMT2 interaction regulate the protein levels of SHMT2 and in this way regulate the DNA patterns of methylation in breast cancer cells.

### EACR2024-0347
### Chromatin and metabolic adaptions provide survival advantage to drug-tolerant persister cells during cisplatin exposure

A. Singh[1], S. Gupta[1]
[1]*Advanced Centre for Treatment- Research and Education in Cancer, Tata Memorial Centre, Epi-genetics and Chromatin Biology Group, Navi mumbai, India*

**Introduction**

Drug-tolerant persister (DTP) cells represent a population of cancer cells that survive after the therapy. These small subsets of cells act as a reservoir for resistant populations and contribute to disease relapse. Understanding the mechanisms underlying DTP cell survival and transition to stable drug-resistant cells (DRC) is imperative for developing effective therapeutic strategies for DTP cells. Here, we investigated the differential alterations in DTP and DRC cells focusing on their metabolic and epigenetic profiles, following cisplatin exposure.

**Material and Methods**

Liver cancer cell lines were cultured and treated with cisplatin and 5-10% of the surviving population are DTP cells. To understand the differential mechanism between parental, DTP, and resistant cells following experiments, RNA sequencing analysis, cell proliferation, cell cycles, apoptosis assay, and TEM were performed. Moreover, chromatin and mitochondria-mediated alteration, and metabolic assays were also performed.

**Results and Discussions**

RNA sequencing analysis unveiled significant downregulation of pathways associated with cell cycle, replication, transcription, and euchromatin maintenance, alongside upregulation of cell-cell communication and cytokine signaling pathways in DTP cells compared to parental and resistant cells. Ultrastructural analysis depicted heterogeneity in cell size, increased nuclear size, lipid accumulation, elevated mitochondrial copy number, and structural alterations in mitochondria. Metabolomic profiling revealed increased levels of citric acid, alpha-ketoglutarate, and pyruvate in DTP cells, along with increased levels of PGC1α and TFAM indicating mitochondrial biogenesis and metabolic reprogramming. DTP cells exhibited elevated levels of heterochromatin markers H3K9me3 and H3K27me3, accompanied by increased expression of their respective methyl-transferases EHMT2 and EZH2, and decreased expression of the demethylase KDM3 targeting H3K9me3. Pre-treatment with chromatin modifier inhibitors followed by cisplatin significantly reduced the number of DTP cells compared to cisplatin alone.

**Conclusion**

Our findings underscore the therapeutic potential of targeting epigenetic and metabolic alterations in combination with chemotherapy to mitigate the survival advantage of DTP cells and prevent the emergence of drug resistance and tumor relapse.

### EACR2024-0369
### Sirtuin 6 deficiency induces apoptosis in low-H3K9ac expressing ovarian cancer cells via impairing DNA repair and survival pathways

A. Karna[1], K. Manoj[2], A. Pallavi[1]
[1]*Amity Institute of Molecular Medicine and Stem Cell Research, Aimmscr, Noida, India*
[2]*Amity Institute of Genome Engineering, Aige, Noida, India*

**Introduction**

Epigenomic-based therapies targeting histone modifications offer new therapeutic approaches for various cancers including ovarian cancer. It has been shown that high grade serous ovarian cancer (HGSOC) patients with homologous recombination deficiency show global decrease in histone H4 acetylation. While histone H4 acetylation is well studied in ovarian cancer, the potential of therapeutic targeting of H3K9 acetylation remains largely unstudied. In the present study, we have conducted in-depth characterization of epigenetic enzymes that acetylate/deacetylate histone H3K9 residues in ovarian cancer cells. This led to identification of Sirtuin 6 (Sirt6), NAD+-dependent class III histone deacetylase (HDAC), as a potential oncogene that may serve as a therapeutic target in ovarian cancer.

**Material and Methods**

We have chosen three ovarian cancer cells i.e. HGSOC OVSAHO cells with global low H3K9ac, HGSOC OVCAR8 cells with high H3K9ac; and non-HGSOC A2780 cells with high H3K9ac. Loss of function studies were performed by siRNA-based gene silencing and cellular assays including cell viability, cell cycle analysis, RNA/protein expression analysis, and molecular assays.

**Results and Discussions**

SIRT6 that deacetylate H3K9ac was knocked down and a strong induction of apoptosis was observed only in low-H3K9ac OVSAHO compared to high-H3K9ac OVCA cells. We find that SIRT6 deficient OVSAHO cells accumulate double strand breaks (increased gamma-H2AX), display enhanced ChK1 (S345) phosphorylation, which induces p73-mediated apoptosis. In addition to defective DSB repair pathway, the PI3K-AKT survival pathway was also impaired with reduction in AKT1/2 phosphorylation due to upregulation of serine/threonine phosphatase PHLPP1. This suppression of AKT1/2 pathway induced BAX mediated apoptosis in OVSAHO cells. Notably, we observed minimal apoptotic induction upon SIRT6 ablation in high H3K9ac-OVCAR8 and A2780 cells.

**Conclusion**

Collectively, we elucidate that ovarian cancer cells with low histone H3K9 acetylation get sensitized to Sirtuin 6 deficiency, leading to apoptosis via impairing DNA repair and survival pathways. Thus, we propose that

© 2024The Authors. Molecular Oncology is published by John Wiley & Sons Ltd on behalf of Federation of European Biochemical Societies targeting SIRT6 has major therapeutic potential for low-H3K9ac expressing HGS ovarian cancer.

### EACR2024-0409
### Strengthening the epigenetic and signaling axis: Dual HDAC/ROCK inhibitors as a new therapeutic strategy in cancer treatment

*M. Beljkaš[1], M. Petković[2], D. Ruzic[1], S. Oljacic[1], K. Nikolic[1], P. Arimondo[3], T. Srdic-Rajic[4], A. Vuletic[4], A. Jelic Mariokov Djuric[4]*
*[1]Faculty of Pharmacy- University of Belgrade, Department of Pharmaceutical Chemistry, Belgrade, Serbia*
*[2]Faculty of Pharmacy- University of Belgrade, Department of Organic Chemistry, Belgrade, Serbia*
*[3]Epigenetic Chemical Biology, Institut Pasteur, Department of Structural Biology and Chemistry, Paris, France*
*[4]Institute for Medical Research- University of Belgrade, Group for Molecular Oncology, Belgrade, Serbia*

**Introduction**
Histone deacetylases (HDACs) have exhibited limited efficacy in single-agent cancer therapy, so that combination therapies had to be explored. However, the clinical use of pan-HDAC inhibitors has been limited by their toxicity and side effects. To improve efficacy and safety, the pursuit of multi-target strategies and the development of isoform-selective HDAC inhibitors have attracted considerable attention. Recent research by Djokovic N. et al. has uncovered a novel synergistic relationship between Rho-associated protein kinases (ROCK) inhibitors and HDAC inhibitors for the treatment of pancreatic ductal adenocarcinoma (PDAC) through computational and experimental studies. This discovery suggests that concurrent inhibition of HDACs and ROCKs by a single agent may enhance anti-tumor efficacy of just HDAC or ROCK inhibitors.
**Material and Methods**
In the development dual HDAC6/ROCK inhibitors, molecular docking studies were performed using Gold Software 2022. For the optimization of these inhibitors, the semi-empirical PM3 and Hartree-Fock methods were used with the 3-21G basis set. The crystal structures of HDAC1 (PDB:5ICN), HDAC6 (PDB:5EDU), ROCK1 (PDB:6E9W) and ROCK2 (PDB:7JNT) were retrieved from the Protein Data Bank and prepared for docking studies. The synthesized inhibitors were evaluated against ROCK1, ROCK2, HDAC1 and HDAC6 in 10-dose IC50 mode, besides evaluating cytotoxicity in breast and pancreatic cancer cell lines via MTT assay.
**Results and Discussions**
Molecular docking studies identified a solvent-exposed region in the known ROCK inhibitor fasudil that served as a target site for integrating structural features of HDAC6 inhibitors, leading to the development of dual HDAC6/ROCK inhibitors. The synthesis of these inhibitors was confirmed by nuclear magnetic resonance and mass spectroscopy. Enzyme assays revealed their inhibitory activity against ROCK and HDAC, with one compound (compound 1) exhibiting selectivity over HDAC6. Compound 1 showed a superior anti-proliferative effect on the MDA-MB-231, MiaPaCa-2 and Panc-1 cell lines compared to the synthesized compounds and the positive controls (fasudil and tubastatin).
**Conclusion**
In this study are presented novel multitarget anticancer agents that simultaneously inhibit epigenetic (HDAC) and ROCK enzymes, which is a pioneering endeavor in this field. These results provide the basis for the development of innovative chemotypes of dual HDAC6/ROCK inhibitors, which represent a promising poly-pharmacologic approach for further cancer treatment.

### EACR2024-0418
### Context-specific role of SNAI2 in modulating lineage-defining transcription factor networks in PDAC

*A. Kanakan[1], T. Ekstrom[2], R. Rosok[2], W. Ge[3], S. A. Johnsen[2], Z. Najafova[1]*
*[1]Robert Bosch Center for Tumor Diseases, Tumor Cell Plasticity, Stuttgart, Germany*
*[2]Robert Bosch Center for Tumor Diseases, Molecular Cancer Epigenetics, Stuttgart, Germany*
*[3]Robert Bosch Center for Tumor Diseases, Bioinformatics Unit, Stuttgart, Germany*

**Introduction**
Pancreatic ductal adenocarcinoma (PDAC) is one of the most aggressive types of cancer with a 5-year overall survival of less than 8% due to high metastasis and chemoresistance, making PDAC a pressing concern for global healthcare. Higher expression of *SNAI2*, which is an epithelial to mesenchymal transition (EMT) transcription factor (TF) is observed to have a strong correlation with poor prognosis in PDAC. Our previous investigations have unveiled a direct regulatory relationship between ΔNp63 and *SNAI2* within the basal subtype of PDAC. However, patient-derived data from single-cell RNA-seq (scRNA-seq) as well as transcriptome data from patient-derived xenograft models (PDX), show that expression of *SNAI2* extends beyond basal-like PDAC. Therefore, we aim to unravel the mechanistic frameworks dictating the context-dependent function of *SNAI2* in PDAC with the ultimate goal of delineating rational therapeutic interventions aimed at counteracting its malignant effects.
**Material and Methods**
To address the epigenetic context of SNAI2 and unravel TFs cooperating with it, we employed techniques such as chromatin immunoprecipitation (ChIP), assay for transposase accessible chromatin (ATAC-seq), chromatin topology analysis of transcriptionally active chromatin (H3K4me3-HiChIP).
**Results and Discussions**
Through this comprehensive transcriptome and epigenome-wide profiling in PDAC cell lines, we observe extensive differences in the function of *SNAI2* across the PDAC subtypes. In line with this, ATAC-Seq coupled with footprinting analysis complemented by H3K4me3-HiChIP, reveals the potential of *SNAI2* as both a transcriptional activator and a repressor contingent upon the TF networks with which it interacts. As a novel finding, we discover

© 2024 The Authors. Molecular Oncology is published by John Wiley & Sons Ltd on behalf of Federation of European Biochemical Societies that *SNAI2* exhibits its transcriptional-repressive functions in basal-like PDAC by impeding *FOXA1* and *GATA6* binding, while its activating role is predominantly associated with established cis-regulatory regions regulated by *ΔNp63*. Conversely, our findings from classical PDAC suggest a predominantly repressive role of *SNAI2* within the chromatin context associated with impeding *HNF4A*.

**Conclusion**

Altogether, the modulation of these endodermal lineage-defining TFs by *SNAI2* advocates for the existence of a common component in repressing the cell's native TF network and activating malignant cancer properties. Therefore, our findings offer valuable insights into the heterogeneity of PDAC and the potential of personalized therapeutic approaches targeting *SNAI2*-mediated pathways.

### EACR2024-0473
### Unraveling DNA Demethylation-Induced Senescence in Colon Cancer Cells

X. Chen[1], K. Yamaguchi[1], P.A. Defossez[1]
[1]Université Paris Cité/ CNRS UMR7216, Epigenetics and Cell Fate Center, Paris, France

**Introduction**

DNA methylation is an essential epigenetic mark in mammals, which regulates gene expression and maintains genomic stability. During DNA replication, DNA methylation patterns are primarily maintained by DNMT1 (DNA methyltransferase 1) and its key regulator UHRF1 (Ubiquitin like with PHD and RING finger domains 1). Aberrant DNA methylation patterns are a hallmark of cancer. In the clinic, targeting the DNA methylation process has been explored as a therapeutic strategy. However, the DNMT1 inhibitor that is used, such as decitabine, also causes DNA damage, making it difficult to separate the therapeutic effects of DNA demethylation from those of DNA damage. Additionally, UHRF1's overexpression in various cancers makes it an attractive target; yet its precise role in cancer cells remains unclear, as does whether directly disrupting UHRF1 could effectively combat cancer.

**Material and Methods**

To address these questions, we used an advanced chemical/genetic system, the auxin-inducible degron (AID) technology in colon cancer cell lines, whereby the degron-fused protein can be totally and rapidly degraded upon the addition of a small molecule, auxin. We successfully generated and confirmed four cell lines: parental wild-type (WT), UHRF1-AID, DNMT1-AID, and double mutant UHRF1-AID/DNMT1-AID.

**Results and Discussions**

Upon auxin treatment, the absence of UHRF1 or/and DNMT1 results in global DNA methylation loss as expected. Surprisingly, prolonged DNA demethylation triggers senescence instead of apoptosis in colon cancer cells. Mechanistically, this senescence occurs independent of DNA damage, and of the canonical p53 and p16/pRb pathways. Time-course transcriptome analysis revealed several early responses involving cell cycle, E2F targets, interferon signaling, secretory phenotype, and cytosolic DNA sensing pathway. As a key cytosolic DNA sensor, cGAS (cyclic GMP-AMP synthase) was derepressed due to DNA demethylation and activated in the cytoplasm. cGAS knockdown could partially attenuated senescence phenotype.

**Conclusion**

In conclusion, blocking DNMT1 or UHRF1 to induce DNA demethylation triggers senescence in cancer cells without DNA damage. This process is dynamic and evolves over time, with cGAS upregulation being crucial for maintaining senescence. A combined treatment with senolytics may then be able to eliminate the cancer cells that have become senescent. Importantly, this strategy would function even in the many tumors that lack functional p53 or Rb.

### EACR2024-0533
### Can DNA methylation biomarkers for the development of gastric metachronous lesions also be considered in an intermediate-risk Caucasian population?

C. Lopes[1], T. C. Almeida[2], C. Macedo-Silva[3], J. Costa[4], S. Paulino[4], C. Jerónimo[3], A. Rei[5], D. Libânio[2], M. Dinis-Ribeiro[2], C. Pereira[2]
[1]CINTESIS@RISE- Portuguese Institute of Oncology of Porto IPO-Porto/Porto Comprehensive Cancer Center Porto.CCC, Precancerous Lesions and Early Cancer Management Group, Porto, Portugal
[2]Research Center of IPO Porto CI-IPOP/Rise@CI-IPOP Health Research Group- Portuguese Institute of Oncology of Porto IPO Porto/Porto Comprehensive Cancer Center Porto.CCC, Precancerous Lesions and Early Cancer Management Group, Porto, Portugal
[3]Research Center of IPO Porto CI-IPOP/Rise@CI-IPOP Health Research Group- Portuguese Institute of Oncology of Porto IPO Porto/Porto Comprehensive Cancer Center Porto.CCC, Cancer Biology and Epigenetics Group, Porto, Portugal
[4]Portuguese Institute of Oncology of Porto, Department of Pathology, Porto, Portugal
[5]Centro Hospitalar Universitário de Santo António, Department of Gastroenterology, Porto, Portugal

**Introduction**

In patients with early gastric cancer (GC), endoscopic submucosal dissection (ESD) is as effective and associated with better quality of life when compared to gastrectomy. However, there is a non-despicable rate of metachronous lesions (ML) after therapy that requires surveillance. Several risk factors have been identified for the development of these lesions, but those do not explain the whole risk. Epigenetic alterations, namely aberrant DNA methylation, are deeply involved in carcinogenesis and it has been described, exclusively in Asian patients, that aberrant methylation of several genes is a promising cancer risk marker. No description was performed in Western Caucasian populations.

**Material and Methods**

This study aimed to validate the DNA methylation profile of *MIR124-3, MIR34b/c*, *NKX6-1, EMX1, MOS,* and *CDO1* as biomarkers for gastric ML development in Caucasian patients. A case-cohort study included individuals treated by ESD with at least 60 months of follow-up (n=264). All cases with metachronous lesions were selected (n=32), as well as 50 controls (without

© 2024 The Authors. Molecular Oncology is published by John Wiley & Sons Ltd on behalf of Federation of European Biochemical Societies

ML) matched for age, gender, *Helicobacter pylori* status, histology, and lesion site. DNA from the normal mucosa adjacent to the primary lesion was extracted, pre-amplified, treated with bisulfite, and used in quantitative methylation-specific PCR, in triplicates.

**Results and Discussions**

*MIR124-3, MIR34b/c* and *NKX6-1* were successfully optimized and able to be analyzed in a total of 72 samples. An increase in the *MIR124-3* gene methylation levels was observed in cases vs controls (81 vs 50; P=0.0084). In the stratified analysis according to clinicopathological factors, *MIR124-3* hypermethylation was found particularly in female patients (P=0.01), whereas an increase in *MIR124-3* and *NKX6-1* methylation levels was identified in individuals negative for HP infection (P=0.0080 and P=0.043, respectively). Patients with higher *MIR124-3* methylation levels were significantly associated with higher risk for developing ML (HR=2.48, P=0.021), while a trend was observed for the *NKX6-1* gene (HR=1.99, P=0.074). No differences were observed regarding the *MIR34b/c* gene.

**Conclusion**

This preliminary study suggests an aberrant methylation profile in the *MIR124-3* gene, as well as an association with risk for ML development. Although further and larger studies are warranted, the association between biomarkers and ML development carefully addressed, especially when considering different ethnic populations.

### EACR2024-0555
### Deciphering the Role of MicroRNAs in Radioresistance: A Study on Pediatric Anaplastic Ependymoma Patients

*M. Elzayat[1], O. Samir[1], M. Saad Zaghloul[2], H. Taha[3], A. Refaat[4], F. Heakl[5], S. Sabet[6], A. A. Sayed[1], M. Tantawy[1]*

*[1]Children Cancer Hospital-57357, Research, Cairo, Egypt*

*[2]Children Cancer Hospital- 57357, Radiotherapy, Cairo, Egypt*

*[3]Children Cancer Hospital-57357, Pathology, Cairo, Egypt*

*[4]Children Cancer Hospital-57357, Radiology, Cairo, Egypt*

*[5]Cairo University, Science, Cairo, Egypt*

*[6]Cairo University, Zoology, Zoology, Egypt*

**Introduction**

Anaplastic ependymoma (AEPN), a type of brain tumor, is currently treated by surgical removal followed by additional therapy such as radiation or specific chemotherapy. However, relapse rates are high, and the prognosis is generally poor, emphasising the need for more effective treatment strategies. Epigenetics, including the study of microRNAs (miRNAs), are being studied to better understand their role in treatment response and as potential biomarkers for AEPN diagnosis and prediction. This study aims to identify a unique miRNAs expression pattern that differentiate between the responder and non-responder to the treatment shedding light on treatment resistance, including radioresistance.

**Material and Methods**

This research is a retrospective study of 21 pediatric patients diagnosed with AEPN. These patients received a standardized treatment regimen that involved surgery followed by radiotherapy. The patients were categorized into two distinct groups based on their treatment outcomes. The first group consisted of patients who did not experience any negative events during or after the treatment for five years, while the second group comprised those who experienced an adverse event following the completion of the entire treatment. To investigate the miRNA expression pattern of these patients, RNA was extracted from the initial preserved tissue samples and assessed for concentration and purity. Next-generation sequencing technology was utilized to examine the expression patterns of around 2400 miRNAs.

**Results and Discussions**

The results showed significant variations in the expression of five miRNAs (miR-223-5p, miR-29b-3p, miR-29a-3p, miR-190b-5p, and miR-1260b) between the two groups, with a p-value of less than 0.05. Four of these miRNAs (miR-29a-3p, miR-29b-3p, miR-190b-5p, and miR-1260b) were downregulated in the event group compared to the non-event group, with fold changes of -1.59, -2.87, -4.55, and -2.89. While miR-223-5p was upregulated with a fold change of 6.16. Enrichment analysis revealed that the downregulated miRNAs (miR-29a-3p, miR-29b-3p, and miR-1260b) regulated genes such as Cyclin D2 (CCND2), which is associated with cancer progression and radioresistance through the activation of the JAK/STAT pathway, leading to resistance to radiotherapy in other cancer patients.

**Conclusion**

These findings shed light on the role of specific miRNAs associated with treatment response in AEPN; however, further research is needed to confirm their involvement in the pathways that lead to radioresistance.

### EACR2024-0580
### Aberrant Promoter-methylation of GALNT6 &14 induces drug resistance and stemness of Breast Cancer via Beta-catenin

*P. Purohit[1], A. Gadwal[1], J.R. Vishnoi[2], P. Pareek[3], R. Choudhary[4], P. Elhence[5], M. Banerjee[1], P. Sharma[6]*

*[1]AIIMS Jodhpur, Biochemistry, Jodhpur, India*

*[2]AIIMS Jodhpur, Surgical Oncology, Jodhpur, India*

*[3]AIIMS Jodhpur, Radiation Oncology, Jodhpur, India*

*[4]AIIMS Jodhpur, General Surgery, Jodhpur, India*

*[5]AIIMS Jodhpur, Pathology & Lab medicine, Jodhpur, India*

*[6]AIIMSJodhpur, Biochemistry, Jodhpur, India*

**Introduction**

N-acetylgalactosaminyltransferases (GALNTs) are polypeptides implicated in abnormal glycosylation, potentially leading to stemness and drug resistance in breast cancer (BC). However, the exact mechanism remains unclear. Studies have shown that GALNTs undergo epigenetic modifications, which contribute to the progression of BC. Currently, there is insufficient data from the Indian population regarding the epigenetic modifications of various GALNTs and their correlation with drug resistance and stemness in BC.

**Material and Methods**

© 2024 The Authors. Molecular Oncology is published by John Wiley & Sons Ltd on behalf of Federation of European Biochemical Societies

The study is hospital-based case-control, involving acquisition of tumour tissue samples from 30 BC pre-therapeutic and post-therapeutic patients, alongside adjacent non-tumour tissues. Gene expression analysis of GALNT6, GALNT14, SOX2, OCT4, ABCC5, beta-catenin were performed using RT-PCR. Samples underwent DNA extraction followed by bisulfite conversion. Global methylation patterns were analyzed using ELISA. Methylation-specific PCR was conducted for GALNT6 and GALNT14 promoters and percentage of methylation was calculated.

**Results and Discussions**

Significant upregulation of GALNT6, GALNT14, SOX2, OCT4, ABCC5, and Beta-catenin genes were found in tumour tissues compared to adjacent non-tumour tissues and in pre-therapeutic patients compared to post-therapeutic patients. The percentage of 5-methylcytosine (% 5mC) was lower in tumour tissues (median level $0.0865 \pm 0.05$) compared to adjacent non-tumour tissues ($p < 0.000$). The %5mC increased post-NACT in the study group (pre-therapy $0.099 \pm 0.04$) (post-therapy0.11 $\pm$ 0.05 ($p < 0.632$)). Significant correlation of %5mC was there with SOX2, Beta-catenin pre-therapy and OCT4, ABCC5 post-therapy ($p<0.001$) GALNT6 and GALNT14 promoters were hypomethylated in tumour tissues compared to adjacent non-tumour tissues, with greater hypomethylation in a pre-therapeutic group compared to the post-therapeutic group ($p < 0.005$), suggesting the hypomethylation patterns in BC lead to drug-resistance and stemness gene expression.

**Conclusion**

The study findings indicate an association between epigenetic modification of GALNT6 and GALNT14 and tumour aggressiveness in BC. We report for the first time promoter specific hypomethylation of GALNT6 and GALNT14 in BC tumour tissue resulting in their overexpression, contributing to drug resistance and stemness. Also percentage methylation of 5mC was significantly associated with drug resistance, stemness and beta-catenin. Consequently, GALNT6 and GALNT14 present promising targets for therapeutic intervention.

### EACR2024-0584

### Epigenetic silencing of MAFG is a potential prognosis biomarker for non-small cell lung cancer adenocarcinomas

*A. Garcia-Guede[1], O. Vera[2], C. Rodriguez-Antolin[1], O. Pernia[1], M. Burdiel[1], I. Losantos-Garcia[3], I. Esteban[4], V.M. Gonzalez[5], J. de Castro[6], I. Ibanez de Caceres[1]*

*[1]IdiPAZ, Experimental Therapies and Biomarkers in Cancer, Madrid, Spain*

*[2]Instituto de Investigación Sanitaria del Hospital la Paz-IdiPAZº, Experimental Therapies and Biomarkers in Cancer- INGEMM, Madrid, Spain*

*[3]University Hospital la Paz, Biostatistics, Madrid, Spain*

*[4]University Hospital la Paz, Pathology deparment, Madrid, Spain*

*[5]Ramón y Cajal Hospital-IRYCIS, Aptamers laboratory, Madrid, Spain*

*[6]University Hospital la Paz, Medical Oncogoly, Madrid, Spain*

**Introduction**

Non-small cell lung cancer (NSCLC) has one of the highest incidence and mortality rates among all cancers. This is in part due to it is usually diagnosed in advanced stages and these patients frequently develop resistance to chemotherapy treatment. We have recently found that *MAFG* derepressed after miR-7 hypermethylation to promote platinum resistance in NSCLC and ovarian cancer cell lines. While the role of MAFG as a regulator of oxidative stress is well known, an alternative role for MAFG as a methylator-phenotype regulator has been found in melanoma and colorectal cancer. However, how MAFG reshapes the lung cancer epigenome hasn't been determined yet.

**Material and Methods**

Here we studied the role of MAFG as a regulator of DNA methylation by combining deletion of *MAFG* by CRISPR/Cas9 and CpG-Methyl-Array followed by expression (qPCR) and methylation (qMSP) analysis in tumor cell lines. Our translational approach combined aptahistochemistry at early stages with specific aptamers of the MAFG protein in 127 patients, methylation analysis in 35 fresh frozen tumors and 40 FFPE samples as well as the use of methylation and expression TCGA databases of NSCLC patients.

**Results and Discussions**

Our results indicate that loss of *MAFG* reduces the promoter methylation of *LIF* and *MAFG* itself. Moreover, we found that reduced methylation of *MAFG* in TCGA and increased protein levels of *MAFG* are associated to worse prognosis. Finally, transcriptional levels of *MAFG* can predict *KRAS* mutated NSCLC adenocarcinomas surveillance.

**Conclusion**

In summary, our work shows that detection of MAFG mRNA, DNA methylation or protein levels are a potent prognostic biomarker for NSCLC, specifically adenocarcinomas.

### EACR2024-0586

### Disrupting DOT1L epigenetic activity as a therapeutic strategy to trigger glioblastoma stem cell differentiation towards a neuronal-like state

*S. Assaf[1], D. Bozek[1], O. Cseh[1], H. Artee Luchman[1], S. Weiss[1]*

*[1]Arnie Charbonneau Cancer Institute, Cell Biology and Anatomy- University of Calgary, Calgary, Canada*

**Introduction**

Glioblastoma is incurable and represents the leading cause of brain tumor-related death in adults. Through a genome-wide essentiality screen, we previously identified Disruptor of Telomeric Silencing 1-like (DOT1L) as one of the critical epigenetic regulators for glioblastoma growth in vitro. Pharmacologic inhibition of DOT1L was further found to prolong survival in vivo. However, the mechanism through which DOT1L regulates glioblastoma growth remains elusive, limiting therapeutic relevance.

**Material and Methods**

RNA-sequencing and Assay for Transposase-Accessible Chromatin with sequencing (ATAC-seq) were performed on glioblastoma stem cell (GSC) models to profile the

© 2024The Authors. Molecular Oncology is published by John Wiley & Sons Ltd on behalf of Federation of European Biochemical Societies transcriptional and chromatin accessibility landscape following DOT1L inhibition, respectively. A genome-wide CRISPR-Cas9 knockout screen was performed to identify genes required to promote GSC differentiation and growth arrest following disruption of DOT1L epigenetic activity.

**Results and Discussions**

ATAC-seq analysis revealed that DOT1L inhibition in GSCs results in increased chromatin accessibility at key genes involved in neural differentiation, synaptic assembly, and dopaminergic neurotransmission. This was correlated with upregulated RNA and protein expression of these genes. The transcriptional upregulation of neuronal genes was accompanied by reduced expression of stem cell markers and morphological alterations consistent with neurite-like outgrowths. Together, these data suggest that disrupting DOT1L epigenetic activity attenuates GSC stemness while promoting differentiation towards a neuronal-like state. Our genome-wide CRISPR-Cas9 knockout screen identified unique subunits from major transcriptional and epigenetic complexes, which are required to promote GSC differentiation and growth arrest following DOT1L inhibition. This suggests the presence of an intricately regulated mechanism that drives GSC differentiation and growth arrest, which is under investigation.

**Conclusion**

We show that disruption of the epigenetic activity of DOT1L reprograms the chromatin accessibility and transcriptional landscape of GSCs, decreasing stemness and proliferation while driving neuronal differentiation. These findings enhance our understanding of mechanisms governing directed neuronal differentiation in GSCs and highlights potential therapeutic strategies for glioblastoma.

### EACR2024-0713
### Targeting Post-Translational Protein Modifications As A Novel Therapeutic Strategy For Diffuse Midline Glioma

*A. Samadder[1], H. Holliday[2], J.B. Bertoldo[2], E. Hayden[2], D.S. Ziegler[3], B.S. Rayner[2]*
*[1]Children's Cancer Institute- University of New South Wales, Brain Tumour Group- School of Clinical Medicine- UNSW Medicine and Health, Kensington-Sydney, Australia*
*[2]Children's Cancer Institute- Lowy Cancer Centre-UNSW Sydney, School of Clinical Medicine- UNSW Medicine & Health, Sydney, Australia*
*[3]Children's Cancer Institute- Lowy Cancer Centre-UNSW Sydney- Kids Cancer Centre- Sydney Children's Hospital- Randwick- NSW 2031- Australia., School of Clinical Medicine- UNSW Medicine & Health, Sydney, Australia*

**Introduction**

Diffuse Midline Glioma (DMG) is a rare and aggressive paediatric brainstem tumour with a 5-year survival rate of less than 1%. Over 90% of DMGs are driven by the H3K27M mutation, leading to widespread loss of repressive H3 K27 trimethylation and epigenetic reprogramming affecting gene expression. Citrullination is a post-translational modification involving the conversion of arginine residues to citrulline, catalysed by the Peptidyl Arginine Deiminase (PAD) enzyme family. Histone proteins are key substrates for citrullination, and our preliminary research has shown that inhibiting PAD affects the DMG epigenome and activates tumour suppressive signalling pathways, ultimately leading to DMG cell death. The project aims at investigating therapeutic efficacy by targeting PAD-mediated citrullination in DMG.

**Material and Methods**

DMG patient-derived RA055 and SU-DIPGXVII cells were treated with a range of PAD inhibitors including Cl-amidine, BB-Cl-amidine, CAY10723, GSK484 and our novel compound in development, JBD-1 with cytotoxicity and colony formation assays performed to determine the pre-clinical efficacy of the compounds. PAD expression and resultant citrullination were determined by western blotting and lentiviral-based CRISPR/Cas9 was used to knockout PAD in DMG cells.

**Results and Discussions**

PAD inhibition resulted in decreased H3 citrullination at specific residues in a dose-dependent manner, as well as activation of antioxidant defence pathways and initiation of the unfolded protein response, leading to tumour cell death. Ongoing research includes epigenetic profiling using CUT&RUN to d determine which genes and pathways are epigenetically regulated by histone citrullination in DMG. In addition, PAD isoform-specific activity assays and CRISPR/Cas9-mediated PAD-knockout DMG models are being used to identify the PAD isoforms affecting DMG tumorigenesis. Furthermore, mass spectrometry will be employed to investigate how pharmacological and genetic targeting of PADs impact protein citrullination as well as the related signalling pathways involved in DMG tumour metabolism and growth.

**Conclusion**

Our results show that protein citrullination potentially plays a critical role in DMG tumorigenesis and targeting PAD-mediated citrullination may provide a novel therapeutic strategy for children with DMG.

### EACR2024-0716
### TGFβ1 Mediated Regulation of Global Gene Expression Through Changes in Landscape of H3K4me3 and H3K9me3 Mark in Prostate Cancer

*A. Naik[1], N. Dalpatraj[1], N. Thakur[1]*
*[1]Ahmedabad University, Biological and Life Sciences, Ahmedabad, India*

**Introduction**

Epigenetics modifications regulate critical cellular processes and biological responses such as cell proliferation, migration, apoptosis, invasion, and senescence. In addition, growth factors, such as TGFβ, are significant regulators of tumorigenesis. The processes by which transitory TGFβ stimulation results in persistent gene expression patterns are still unclear. Epigenetic marks like Histone H3 modifications are directly linked with gene expression and are vital in tumorigenesis. However, the upshot of TGFβ signaling on the genome-wide H3K4me3 and H3K9me3 landscape remains unidentified.

© 2024 The Authors. Molecular Oncology is published by John Wiley & Sons Ltd on behalf of Federation of European Biochemical Societies

**Material and Methods**
In this study, We performed ChIP-Sequencing to identify the genome-wide landscape alterations in H3K4me3 and H3K9me3 marks in response to TGFβ stimulation in cancer.
**Results and Discussions**
We show that TGFβ induces the H3K4me3 mark on its ligands like TGFβ, GDF1, INHBB, GDF3, GDF6, and BMP5, suggesting a positive feedback loop. Most genes were involved in the positive transcriptional regulation from the RNA pol II promoter in response to TGFβ. Other processes influenced were intracellular protein transport, EMT, angiogenesis, histone H4 acetylation, cell cycle arrest, and genes involved in mitotic G2 DNA damage checkpoints, indicating a crucial role of H3K4me3 mark in TGFβ mediated oncogenic functions and EMT program. Our results also point toward a positive association between the oncogenic function of TGFβ and the H3K9me3 mark and provide a context for the role of H3K9me3 in TGFβ-induced cell migration and cell adhesion. Interestingly, these roles of TGFβ through H3K9me3 mark regulation may depend on transcriptional activation in contrast to the conventionally known repressive nature of H3K9me3. The results indicate a positive association between the oncogenic function of TGFβ and the H3K9me3 mark. The co-enrichment of H3K4me3 and H3K9me3 marks also revealed critical genes associated with cellular processes such as cell cycle, cell division, and cell adhesion, suggesting evidence of crosstalk and dual modification readouts. We also discovered distinct Broad H3K4me3 signatures that were found to be associated with different pathways contributing to the EMT program.
**Conclusion**
Our results link TGFβ stimulation to regulation in gene expression through an epigenetic mechanism. These findings have broader implications on epigenetic bases of acute gene expression changes caused by growth factor stimulation.

### EACR2024-0766
### POSTER IN THE SPOTLIGHT
### Extracellular histones profiles as biomarkers of pediatric H3K27-altered diffuse midline glioma

*D. Buzova[1], L.L. Petrilli[2], J. Frohlich[3], D. Tsoneva[4], S.D. Bianco[5], A. Alisi[6], J. Cerveny[1], T. Mazza[5], M. Vinci[2], M. Vinciguerra[7]*
*[1]Czech Globe, Department of Adaptive Biotechnologies, Brno, Czech Republic*
*[2]Ospedale Pediatrico Bambin Gesu', Department of Onco-hematology- Gene and Cell Therapy, Rome, Italy*
*[3]St'Anne University Hospital, International Clinical Research Center, Brno, Czech Republic*
*[4]Medical University of Varna, Department of Medical Genetics, Varna, Bulgaria*
*[5]IRCCS Casa Sollievo della Sofferenza, Bioinformatics Unit, San Giovanni Rotondo, Italy*
*[6]Ospedale Pediatrico Bambin Gesu', Research Unit of Molecular Genetics of Complex Phenotypes, Rome, Italy*
*[7]Medical University of Varna, Department of Stem Cell Biology and Transplantology, Varna, Bulgaria*
**Introduction**
Diffuse midline glioma, H3 K27-altered (DMG) is a fatal tumour that arises in midline structures of the brain. When located in the pons it is named diffuse intrinsic pontine glioma(DIPG). DMG/DIPG is usually diagnosed when children are aged <10 years, and it has a median overall survival of <12 months after diagnosis. Radiological imaging is the gold standard for DIPG diagnosis while the use of invasive biopsy focuses on the understanding its molecular biology, such as the histone H3K27M mutation. The urgent need to improve the survival encourages the use of biofluids for optimizing molecular diagnoses in DMG/DIPG. Here, we propose a new, fast, imaging and epigenetics based approach to diagnose DMG/DIPG in the plasma of pediatric patients.
**Material and Methods**
A total of 20 healthy children (mean age:10.5] and 25 children diagnosed with DMG/DIPG (mean age: 8.5) were recruited. Individual histones (H2A, H2B, H3, H4, macroH2A1.1 and macroH2A1.2), histone dimers and nucleosomes were assayed in biofluids by means of a new advanced flow cytometry ImageStream(X)-adapted method.
**Results and Discussions**
We report a significant upregulation of circulating histone dimers and tetramers (macroH2A1.1/H2B vs control: p-value<0.0001; macroH2A1.2/H2B vs control: p-value<0.0001; H2A/H2B vs control: p-value<0.0001; H3/H4 vs control: p-value=0.008; H2A/H2B/H3/H4 vs control: p-value<0.0001) and a significant down-regulation of individual histones (H2B vs control: p-value<0.0001; H3 vs control: p-value<0.0001; H4 vs control: p-value<0.0001). Moreover, individual histones and histone complexes are also detectable in the CSF of a subset of DMG/DIPG patients, and in the supernatant of a DMG/DIPG cell line, with distinct patterns compared to the plasma.
**Conclusion**
In summary, we identified circulating histone signatures able to detect the presence of DMG/DIPG in biofluids of children, using a rapid and non-invasive ImageStream(X)-based imaging technology. The patterns observed in the blood suggest the differential involvement of histone chaperone complexes in histone extracellular release in DMG/DIPG children plasma.

### EACR2024-0797
### Genetic and Epigenetic study of Formalin-damaged (FFPE) DNA with 6-base sequencing

*A. Modat[1], R. Crawford[1], F. Puddu[1], R. Sethi[1], T. Beech[2], T. Charlesworth[3], P. Creed[4]*
*[1]biomodal, Customer Collaborations, Cambridge, UK*
*[2]biomodal, Product Management, Cambridge, UK*
*[3]biomodal, Marketing and Product Management, Cambridge, UK*
*[4]biomodal, Computational Technologies, Cambridge, UK*
**Introduction**
Formalin-fixed and paraffin-embedded (FFPE) specimens are a common source of long-term stored samples used for research or clinical settings in fields including immunohistochemistry, oncology, and

© 2024 The Authors. Molecular Oncology is published by John Wiley & Sons Ltd on behalf of Federation of European Biochemical Societies genomics. Genomic studies that incorporate epigenomic information such as cytosine modification status (5mC & 5hmC) add additional insight into fundamental pathways of gene activation or silencing. Such insight can enable earlier detection of cancer or other disease state. The ability to derive combined genomic and epigenomic data from FFPE samples enables a meaningful increase in biological insight from this ubiquitous sample type. However, DNA damage induced by formalin fixation (e.g., deamination, fragmentation or nucleic acid crosslinking) can lead to decreased genomic and epigenomic data quality when using next generation sequencing (NGS) approaches.

**Material and Methods**

Here we apply a recently announced 6-base sequencing method to DNA extracted from both formalin-compromised DNA (fcDNA, known standards with controlled formalin damage) as well as FFPE samples from colorectal, lung, stomach, urinary, ovarian and pancreatic cancers. This innovative 6-base sequencing approach offers an enzymatic single-workflow solution enabling the simultaneous detection of both cytosine modifications (5mC, 5hmC) alongside canonical bases (A, C, T, G) at single-base resolution and with a low nanogram input requirement. Simultaneous detection avoids information loss with other methods, while preserving the ability to discriminate important C-to-T transitions.

**Results and Discussions**

Increased levels of formalin damage to DNA corresponded to lower library yields and insert sizes, as has previously been observed. Also as previously observed for orthogonal techniques, higher relative duplication and lower coverage rates were associated with increasing damage. However, the genetic accuracy of six-base sequencing was largely preserved, and minimal effect was observed on variant allele frequency (VAF) calling for all formalin compromised DNA standards even with severe damage (DIN $\leq 2.0$). Comparing deep sequenced FFPE cancer samples to matched fresh frozen (FF) equivalents showed overall minimal impact on overall genetic and epigenetic information.

**Conclusion**

In conclusion we demonstrate compatibility of 6-base sequencing with formalin-compromised DNA, producing high accuracy genetic and epigenetic information from FFPE samples even at severe levels of DNA damage.

### EACR2024-0974

### Identification of a novel functional hotspot noncoding mutation in HER2+ breast cancer that occurs in a transcriptional enhancer region

*A. Cuy Saqués*[1], *G. Colley*[2], *L. Ivers*[2], *C. Monger*[3], *F. Matra*[3], *D. Nimmo*[3], *A. Armesto*[3], *A. Bracken*[3], *J. Crown*[4], *A. Eustace*[2]

[1]*Dublin City University, National Institute for Cellular Biotechnology, Dublin, Ireland*

[2]*Dublin City University, Faculty of Biotechnology, Dublin, Ireland*

[3]*Trinity College Dublin, Smurfit Genetics Institute, Dublin, Ireland*

[4]*St Vincent's University Hospital., Medical Oncology Department, Dublin, Ireland*

**Introduction**

70% of breast cancer(BC) patients present PI3K pathway gene mutations. PIK3CA mutations are used as biomarkers of response to PI3K inhibitors(PI3Ki), but mutant PIK3CA doesn't always correlate with response to PI3Ki. Noncoding mutations in the loci of PI3K pathway genes could play a role in maintaining pathway activation and predict therapy response in BC.

**Material and Methods**

Noncoding mutations surrounding PI3K pathway genes were obtained from whole genome sequencing data of 915 BC patients(Nik-Zainal *et al.*, 2016;Priestley *et al.*, 2019;Staaf *et al.*, 2019). The Activity by Contact(ABC) model(Fulco *et al.*, 2019) was used to predict interactions between regions containing the mutations and surrounding transcription start sites. Mutation frequency was validated in a cohort of HER2+ BC patients via MassArray and correlated with patient outcome. Impact on gene expression was confirmed through luciferase reporter assays. CCLE data(RNA, RPPA and drug sensitivity) enabled the correlation of our prioritised mutations with changes in protein/gene expression and sensitivity to treatment. Finally, phenotypic assays assessed the anti-cancer impact of novel combinations of PI3Ki and selected drugs.

**Results and Discussions**

PI3K pathway gene loci are enriched for recurrent noncoding mutations in BC patients(p=0.005). An A:TGC insertion in the gene loci of ERBB2 (chr17:39959177, 9177 mutation) was identified in HER2+ BC patients. Its presence was confirmed by MassArray analysis in 75% of HER2+ BC patients analysed(n=72) and it correlated with poor clinical response to trastuzumab(p=0.006) in the metastatic setting. The 9177 mutation is located inside the HER2 amplicon and has high transcriptional activity in HER2+ BC cells; its enhancer activity was confirmed via luciferase assays. In-silico, the 9177 mutation is associated with increased gene expression up to 300kb away from the mutation site and significant decreases in DNA-damage repair protein expression. In-vitro analysis demonstrated that 9177 mutant HER2+ BCs are sensitive to a combination of a PI3Ki and an IRAK4 inhibitor, which increases cell cycle arrest and has synergistic anti-proliferative effects.

**Conclusion**

A novel functional non-coding 3-base insertion was found in 75% of HER2+ patients, with prevalence in those with poor trastuzumab response. It is located inside the HER2 amplicon, is associated with high transcriptional activity and acts as an enhancer. Its presence is related with sensitivity to an IRAK-4 inhibitor that showed synergy in combination with PI3Ki.

### EACR2024-1071

### EpigenPlot.com: enabling large-scale analysis of gene-level methylation in colorectal cancer

*D. Müller*[1], *B. Győrffy*[2]

[1]*Semmelweis University, Bioinformatics Department,*

© 2024 The Authors. Molecular Oncology is published by John Wiley & Sons Ltd on behalf of Federation of European Biochemical Societies

*Budapest, Hungary*
[2]*Semmelweis University, Department of Bioinformatics, Budapest, Hungary*

**Introduction**

DNA methylation plays a crucial role in colorectal cancer (CRC) development. This study aimed to identify novel methylation biomarkers by analyzing a large-scale dataset integrating DNA methylation data from various sources. In order to enable comfortable examination of the database we also aim to create a web-based interactive platform.

**Material and Methods**

We constructed a database encompassing methylation data based on Illumina HM450K and EPIC arrays from 2646 samples across various tissues (normal mucosa, adenoma, and adenocarcinoma) from multiple datasets. We compared methylation patterns between tissues, identified differentially methylated regions (DMRs), and evaluated their potential as biomarkers using receiver operating characteristic (ROC) analysis. Finally, we established a web application using the shiny R package.

**Results and Discussions**

We identified a high number of DMRs, many located near proximal promoters (TSS200, 5'UTR, and first exon). We validated previously studied CRC methylation biomarkers and identified novel candidates with promising performance in differentiating between normal and cancerous tissues. Our analysis also identified novel candidate biomarkers, including genes TMEM240 and TM4SF19 (HM450K cvAUC: 0.89 and 0.89).

**Conclusion**

Our study highlights the value of analyzing DNA methylation patterns across regions for uncovering potential CRC biomarkers. The established database and web platform provide valuable resources for researchers exploring the role of DNA methylation in CRC and potentially other cancers.

### EACR2024-1203
### Systematic Review of DNA Methylation Patterns in Colorectal Cancer: Clinical Implications and Therapeutic Targets

*L.R. Rajmohan*[1], *P. p*[2], *P. d*[1]
[1]*ACS Medical College & Hospital, General Medicine, Chennai, India*
[2]*ACS Medical College & Hospital, Biochemistry, Chennai, India*

**Introduction**

Colorectal cancer (CRC) is a complex disease characterized by heterogeneous DNA methylation patterns across different stages, impacting prognosis and treatment response. Understanding these variations is crucial for personalized therapeutic interventions.

**Material and Methods**

We performed a systematic review of literature examining DNA methylation patterns in CRC stages using PubMed, Embase, and Scopus databases. Studies investigating therapeutic strategies targeting aberrant DNA methylation were also analyzed.

**Results and Discussions**

DNA methylation alterations vary significantly among CRC stages, with distinct patterns observed in early-stage adenomas compared to advanced carcinomas. Hypermethylation of tumor suppressor genes and hypomethylation of oncogenes are common, contributing to tumorigenesis and metastasis. Therapeutic approaches targeting DNA methylation include DNA methyltransferase inhibitors (DNMTis) and histone deacetylase inhibitors (HDACis). Clinical trials demonstrate efficacy in reversing epigenetic alterations and restoring normal gene expression, although adverse effects such as myelosuppression and gastrointestinal toxicity are noted.

**Conclusion**

DNA methylation patterns in CRC exhibit stage-specific alterations with prognostic implications. Targeted therapies aimed at modulating DNA methylation hold promise in CRC treatment, necessitating further research to optimize efficacy and minimize adverse effects.

### Experimental/Molecular Therapeutics, Pharmacogenomics

### EACR2024-0009
### Overexpression of Smac by an armed vesicular stomatitis virus potentiates antitumor immunity by inducing pyroptosis

*F. Chen*[1], *L. Lang*[2], *M. Luo*[3], *Y. Teng*[1]
[1]*Emory University, Department of Hematology and Medical Oncology, Atlanta, United States*
[2]*Augusta University, Georgia Cancer Center, Augusta, United States*
[3]*Georgia State University, Department of Chemistry, Atlanta, United States*

**Introduction**

Oncolytic therapy is an emerging antitumor strategy that harnesses natural or engineered oncolytic viruses (OVs) to target tumor tissues and suppress tumor progression. Despite reports of successful clinical cases, many tumors appear to be insensitive to OV infection.

**Material and Methods**

To remove the related bottlenecks, an armed vesicular stomatitis virus (VSV) was constructed by inserting a transgene to express Smac/DIABLO during viral infection (VSV-S). We used head and neck squamous cell carcinoma (HNSCC) cell lines and related animal models as a research platform to evaluate the therapeutic efficacy and underlying mechanism of VSV-S treatment. Molecular alterations were examined by RNA-sequencing, phospho-kinase profiling, Western blotting, ELISA and immunohistochemistry. Cancer cell proliferation and death were assessed by MTT, transmission electron microscope and flow cytometry.

**Results and Discussions**

Second mitochondria-derived activator of caspases (Smac) is a protein that plays a role in apoptosis by binding to inhibitor of apoptosis proteins and allowing caspases to be activated. Our previous report showed that endogenous Smac was dramatically suppressed in HeLa cells during VSV infection. Here, we report for the first time that elevation of Smac levels by VSV-S infection enhances antitumor immunity by inducing tumor

© 2024 The Authors. Molecular Oncology is published by John Wiley & Sons Ltd on behalf of Federation of European Biochemical Societies pyroptosis, an inflammatory cell death usually associated with inflammasome activation. In the orthotopic tongue tumor mice, VSV-S showed greater potential to limit tumor burden and progression than VSV, enhanced T-cell recruitment and activation in the tumor micro-environment (TME) and reversed the immunosuppressive state to promote T cell-mediated antitumor immunity. Mechanistic study further indicated that VSV-S induced tumor cell pyroptosis through activation of caspase-1-gasdermin E (GSDME) signaling cascade, which is independent of its-mediated apoptosis.

**Conclusion**

These novel and significant findings not only demonstrate a novel mechanism underpinning VSV-S-mediated antitumor efficacy, but also provide a potential therapeutic strategy to enhance the success of oncolytic therapy in cancer patients.

### EACR2024-0027
### Exposure to T-Consciousness Fields ameliorate hepatocellular carcinoma induced in mice by diethyl nitrosamine

*M. Ramazanzade[1], M.A. Taheri[1], A. Davallou[1], G. Shojaei[1], P. Houshang[1], N. Taheri[1]*
*[1]Cosmointel, Sciencefact R&D, Toronto, Canada*

**Introduction**

Cancer treatment using Taheri Consciousness Fields (T-CFs) is a novel approach which was introduced in 1980s by Mohammad Ali Taheri based on in vivo exposure to T-Consciousness Fields. These fields are subcategories of a Networked Universal Internet called the Cosmic Consciousness Network (CCN). The aim of the present study was to experimentally examine the effects of T-CFs in prevention and treatment of HCC induced by a chemical carcinogen.

**Material and Methods**

HCC was induced in C57bl6 mice after treatment with a single dose (50 mg/Kg BW) of diethylnitrosamine (DEN) followed by phenobarbitone (PB, 500 mg/Liter water) given in daily drinking water. HCC induction was started in mice when they were 14 days old and PB was given from day 28. Animals were divided into 4 groups, a control group; treated with DEN+PB, intervention groups (T-CFs-exposed) comprised on a Prevention group and a Treated group. In prevention group mice were briefly subjected to T-CFs prior to DEN treatment. In the case of Treatment group, mice were exposed to T-CFs, after tumor formation.. Animals were monitored at different time points and finally after 7 months all the animals were sacrificed and processed for liver histological examination and serum analysis.

**Results and Discussions**

Serum α-fetoprotein (AFP) was elevated by 2-3 folds in mice treated with DEN regardless of other interferences. Pathology revealed that in positive control (DEN+PB) there was extensive liver damage, cytoplasmic vesicles in most of hepatocytes, nuclear degeneration and anaplastic alteration. Presence of clear cells indicate the progressive malignancy in liver preparation. In mice subjected to TC-Fields before DEN (Prevention), there was no observable atypical and abnormal mitotic condition. Liver damage was limited to inflammatory reactions, fibrotic structures and coagulative necrosis. In Treatment group (mice subjected to T-CFs after DEN), the number of bio-transformed cells were significantly less as compared to that observed in positive control group (DEN-treated). However, infiltration of inflammatory cells and few mitotic cells were observed.

**Conclusion**

In vivo exposure of mice to T-CFs under experimental condition either before or after inducing HCC by DEN can ameliorate HCC progression. The efficiency of T-CFs on liver damage and HCC was more obvious in mice exposed to T-CFs before carcinogen suggesting that this treatment works as an important cancer preventive agent. More experiments are needed to better understand the effects(s) of action of T-CFs.

### EACR2024-0039
### Bulgarian patients with multiple myeloma treated with Daratumumab – preliminary data of the drug's efficacy and safety

*D. Nikolova[1,2], A. Yordanov[3], A. Maslarova[3], A. Radinov[3]*
*[1]Medical University - Sofia, Medical Genetics, Sofia, Bulgaria*
*[2]University Hospital "St.Ivan Rilski", Laboratory of genetics, Sofia, Bulgaria*
*[3]University Hospital "St.Ivan Rilski", Clinic of Hematology, Sofia, Bulgaria*

**Introduction**

Treatment is multiple myeloma (MM) aims to slow the disease progression, to prolong remissions and improve the quality of life. Darzalex (Daratumumab) is the first Anti-CD38 monoclonal antibody approved for patients with relapsed MM in 2016 by FDA. A substantial reduction in the amount of the antibodies in the blood/urine is usually considered a positive effect of the therapy, therefore those levels have to be monitored at regular intervals during the treatment process. In this study, we report preliminary results of Darzalex treatment of our patients with MM.

**Material and Methods**

This study included 32 patients with MM. We monitor the percentage of plasma cell populations in blood/urine, the amount of the secreted paraprotein before and after therapy, patients' general clinical condition, the existence of complete/very good/partial/no response, as well as the efficacy of the treatment (side effects). The patients also undergo clinical laboratory testing, bone marrow evaluation, cytogenetic testing, physical examinations.

**Results and Discussions**

The mean age of the patients is 67 years (range from 45 to 81, women: men= 12:20). The majority are on Drd treatment, 8 – on DVd and 3 – only Dd. 11 patients are with disease progression, with different percentage of plasma cell population before treatment (from 10-100%), two have complete response (CR) and the majority (19 patients) are newly diagnosed. After four courses of the executed therapeutic regimen, the percentage of plasma cell populations in the bone marrow and the amount of secreted paraprotein in the serum/urine have been re-estimated. 12 patients (38%) have decreased the plasma cell populations more than 10 times. Seven patients (22%) have decreased the amount of paraprotein in the

Molecular Oncology 18 (Suppl. 1) (2024) 1–495
© 2024 The Authors. Molecular Oncology is published by John Wiley & Sons Ltd on behalf of Federation of European Biochemical Societies urine/blood, while other five (16%) have no data for a detectable paraprotein levels. Up to now, 8 patients (25%) have a complete response (CR), 2 (6%) – partial response (PR), 4 (13%) – very good partial response (VGPR). Only one patient doesn't show evidence for treatment efficacy. The most common side effect of the treatment is agranulocytosis (10/32, 31%) and allergic reactions of different severity (3/32, 9%).

**Conclusion**

New targeted therapies like Darzalex are changing the future of multiple myeloma. It demonstrates rapid and long-lasting responses. In our patients with MM, Daratumumab shows high efficacy coupled with minimal side effects.

### EACR2024-0067
### Synergistic action of PI3K/AKT pathway small molecule inhibitors with chemotherapeutic agents in lung cancer cells

*C. Neophytou*[1], *M. Michael*[1], *M. Christou*[2]
[1]*European University Cyprus, Apoptosis and Cancer Chemoresistance Laboratory- Basic and Translation Cancer Research Center- Department of Life Sciences, Nicosia, Cyprus*
[2]*European University Cyprus, Tumor Microenvironment Metastasis and Experimental Therapeutics Laboratory- Basic and Translation Cancer Research Center- Department of Life Sciences, Nicosia, Cyprus*

**Introduction**

Lung cancer is the second in incidence and first in mortality malignancy worldwide. Despite the many therapeutic advances against lung cancer in the last two decades, there is still great need for effective treatments. Chemotherapeutic drugs, including cisplatin and 5-fluorouracil (5-FU), are widely used for the treatment of solid tumors. The combination of conventional drugs with small molecule inhibitors (SMIs), is an important strategy to sensitize cancer cells to treatment and reduce negative side effects. The PI3K/AKT pathway, controlling proliferation and survival, is often overactive in cancer. MK-2206, an AKT inhibitor and buparlisib (BKM120) a PI3K inhibitor, are being investigated in many clinical trials in combination with chemotherapy. In this study, we investigated the potential synergistic effects of MK-2206 and buparlisib in combination with cisplatin and 5-FU in lung cancer cells.

**Material and Methods**

H460 and A549 cells were treated with the agents alone and in combination and the MTT viability assay was applied. We estimated the IC50 of the agents when used in monotherapy and then applied the Chou-Talalay index to determine if they have synergistic, additive or antagonistic effects. Cell cycle analysis and apoptosis was evaluated by Flow Cytometry following cell staining with AnnexinV/Propidium iodide. In addition, we investigated by Real-Time PCR and Western Blot, whether the agents change the mRNA and protein levels of key genes implicated in survival and cell death pathways.

**Results and Discussions**

Based on the IC50 values, we determined that H460 cells were more sensitive to cisplatin, 5-FU and BKM120 but not to MK-2206, compared to A549 cells. The optimal synergistic effect was observed when H460 cells were treated with 10 μM 5-FU and 0.1 μM BKM120 at 48 hours (CI index=0.08). The combination treatment increased the percentage of late apoptotic cells and the subG1 phase of the cell cycle and increased the mRNA levels of the pro-apoptotic Bax gene in H460 cells. In addition, the treatment reduced the expression levels of the anti-apoptotic Bcl-2 protein and of phosphorylated AKT suggesting inhibition of the PI3K survival pathway.

**Conclusion**

In conclusion, the combination of 5-FU and BKM120 induced apoptosis in H460 cells. Since this cell line is known for its metastatic potential, the agents may be evaluated in the future for their ability to block cancer cell invasion. The combination of conventional drugs and AKT inhibitors may have potential usefulness in lung cancer therapy.

### EACR2024-0076
### Allicin therapeutic potential: Effects on migration, proliferation, and modulation of metastasis-associated traits in breast cancer cells

*E. Venegas*[1], *K. Cáceres*[1], *C. Gónzalez*[1], *A. García*[1], *P. Ortiz*[2], *V. Rosas*[1]
[1]*Instituto Tecnológico y de Estudios Superiores de Monterrey, Escuela de Medicina y Ciencias de la Salud, Zapopan, Mexico*
[2]*Instituto Mexicano del Seguro Social, Centro de Investigación Biomédica de Occidente, Guadalajara, Mexico*

**Introduction**

Breast cancer is the second cause of cancer death among women overall, but is the leading cause among Black and Hispanic women. It has an annual increase rate of 0.5%, emphasizing the urgency of understanding its complexities. A crucial aspect of unraveling tumor progression in breast cancer lies in comprehending the epithelial-mesenchymal transition (EMT). Research focused on this process holds the key to identifying targeted therapeutic strategies. Lately, anti-cancer research has been pointing to plant-derived compounds; notably, organo-sulfur compounds of garlic (Allium sativum) have potential anticancer properties. Allicin (ALC), a significant garlic-derived component, stands out for its anti-angiogenic and immunomodulatory effects. While promising in apoptosis, its role in the metastatic process is under research. This study aims to assess ALC's effects on proliferation, clonogenicity, migration, senescence, and EMT properties across Luminal A (MCF-7) and Triple-negative (HCC70) breast cancer cell lines.

**Material and Methods**

The study determined ALC's IC50 for each cell line (10, 45, 90 μM for MCF7; 1.5-20 μM for HCC70). Evaluations included cell proliferation and colony formation after 4 and 15 days, investigating clonogenic capacity. Cellular senescence was determined through the Bromodeoxyuridine (BrdU) incorporation conducted

© 2024The Authors. Molecular Oncology is published by John Wiley & Sons Ltd on behalf of Federation of European Biochemical Societies over a 72-hour time frame. Protein expression related to EMT (E-cadherin, CD44, CD24, and vimentin) was assessed, and qPCR was conducted for EMT gene expression after 4-24 hours of ALC treatment. Wound-healing assays measured migration at 0-36 hours under ALC treatment for 24h.

**Results and Discussions**

Results show a significant decrease ($p<0.05$) in proliferation in MCF-7 and HCC70, with anti-clonogenic effects in a dose-dependent manner. ALC exhibited no senescence induction but exhibited a senolytic effect. Upregulation of STAT3 and E-Cadherin was observed. ALC modulated the expression of specific traits, including CD24 and CD44, and induced E-Cadherin expression in HCC70, associated with EMT reversal. Although ALC decreased migration, no dose-dependent effect was apparent. Morphological assessment after 72 hours revealed cell elongation, cytoplasmic expansion, and cell death.

**Conclusion**

In conclusion, ALC displays antiproliferative, anti-clonogenic, senolytic effects, and anti-EMT properties on both cell lines. These findings underscore ALC's multifaceted effects, positioning it as a promising therapeutic agent in breast cancer treatment.

### EACR2024-0096
### Targeting GLI1 to inhibit the Hedgehog pathway for the development of anticancer drugs

*S. Covaceuszach[1], L.E. Rosso[1], D. Pirolli[2], L. Di Marcotullio[3], B. Botta[4], D. Lamba[1]*
*[1]Istituto di Cristallografia, Dipartimento di Scienze Chimiche e Tecnologie dei Materiali CNR, Trieste, Italy*
*[2]Istituto di Scienze e Tecnologie Chimiche, Dipartimento di Scienze Chimiche e Tecnologie dei Materiali CNR, Roma, Italy*
*[3]Dipartimento di Medicina Molecolare, Sapienza University, Roma, Italy*
*[4]Dipartimento di Chimica e Tecnologie del Farmaco, Sapienza University, Roma, Italy*

**Introduction**

The Hedgehog pathway is a crucial signaling cascade involved in vertebrate and invertebrate embryonic development, homeostasis and tissue regeneration. Its dysregulation is associated with developmental pathologies and various cancers, primarily linked to alterations of the GLI family of transcription factors. Targeting GLI proteins has emerged as a promising therapeutic approach, notably through the identification of a GLI1 inhibitor, glabrescione B (GlaB), via in silico screening of plant derived compounds. This isoflavone from *Derris Glabrescens* plant seeds has demonstrated effectiveness in limiting growth and inducing apoptosis in glioblastoma stem cells both in vitro and in vivo.

**Material and Methods**

Two fusion constructs were created to produce the GLI1 DNA binding domain (GLI1-ZF) in a soluble form. Differential Scanning Fluorimetry (DSF) and Grating-Coupled Interferometry (GCI) were employed to qualitatively confirm and quantitatively characterize the kinetics and affinity of the interactions of GLI1-ZF with GlaB and a derived collection of bioactive compounds. Computational protein-ligand docking studies highlighted the molecular determinants of the observed binding constants differences.

**Results and Discussions**

The study focuses on optimizing a biophysical platform to screen GLI1 inhibitors using GlaB as a reference compound. DSF and GCI experiments indicate specific interactions of GLI1-ZF with GlaB and two synthetic derivatives, NT8 and NT11. The former shows a positive effect on association kinetics and affinity, while the latter shows a decrease in affinity due to a faster dissociation kinetic. The results of in silico docking agreed well with the experimental affinity data, indicating NT8, characterized by a more compact structure than GlaB, to bind GLI1-ZF more stably. Conversely, the GLI1-ZF/NT11 complex appears to be less stable likely due to greater steric hindrance.

**Conclusion**

This study allowed setup an optimized biophysical platform for the screening and quantitative characterization of affinity and binding kinetics that will be used in the validation of a series of candidates, identified through a virtual screening of a library of 90,000 compounds targeting GLI1 and optimized for ADME. Moreover, by integrating these experimental analyses with in silico docking, we elucidated the impact of specific GlaB scaffold derivatizations on the interaction with GLI1, paving the way for the development of more effective anticancer drugs.

### EACR2024-0115
### Minor spliceosome small nuclear RNAs are novel therapeutic targets for cancer

*K. Girardini[1], A. Augspach[2], P. Nicholson[3], M. Rubin[2], R. Kanadia[1]*
*[1]University of Connecticut, Physiology and Neuro-biology Department, Storrs, United States*
*[2]University of Bern, Department of Biomedical Research, Bern, Switzerland*
*[3]University of Bern, Next Generation Sequencing Platform, Bern, Switzerland*

**Introduction**

Increased expression of minor intron-containing genes (MIGs) and the small nuclear RNAs (snRNAs) of its namesake the minor spliceosome (MiS) revealed their novel role in prostate cancer progression. Specifically, expression of snRNAs (U11, U12, U4atac, and U6atac) progressively increases across cell lines (LnCaP, C4-2, 22Rv1) and organoids (PM154) representing prostate cancer progression. We hypothesized that this increase in MIG and MiS components would result in increased efficiency of minor intron splicing. Indeed, we confirmed this hypothesis by a luciferase minor intron splicing reporter. We showed that inhibition of the MiS by siRNA mediated knockdown of U6atac blocked prostate cancer cell proliferation and survival. Thus, we nominated snRNAs of the minor spliceosome as novel therapeutic targets to treat cancer.

**Material and Methods**

Given that snRNAs are often encoded by multiple gene variants that are expressed in a tissue specific manner, we sought to identify snRNA gene variants in the human

© 2024 The Authors. Molecular Oncology is published by John Wiley & Sons Ltd on behalf of Federation of European Biochemical Societies genome. We designed a bioinformatics approach to leverage sequence similarity and secondary structure constraints to identify high-confidence minor spliceosome snRNA gene variants in the human genome. Since we could not detect expression of these snRNAs, which are not polyadenylated with an average size ~150 nucleotides, with normal Illumina RNAseq, we have designed a new method we call snRNAseq. This new method uses off the shelf technology to adapt illumina sequencing platform to capture the expression of these snRNA variants that are often differentially expressed across prostate cancer progression.

**Results and Discussions**

We have identified 6, 2, 16, 61 snRNA gene variants for U11, U12, U4atac, and U6atac, respectively in the human genome. Moreover, through our snRNAseq, we have identified disparate gene variants that are expressed in LnCaP, C4-2, 22Rv1, and PM154 samples. The dynamic changes in expression of the snRNA variants across prostate cancer progression is in line with tissue-specific expression of multi-copy genes. This discovery opens a new targeted therapeutic avenue, where we can silence expression of a specific snRNA gene variant to inhibit the minor spliceosome in tissue or stage specific cancer thereby reducing toxicity related to broad inhibition of the minor spliceosome.

**Conclusion**

In all, we show that snRNAs are novel therapeutic targets that can be leveraged to achieve tissue and cancer stage specificity.

### EACR2024-0116

### Unveiling the potential of Indole-tethered pyrazoline derivatives as a promising strategy for combating cancer via Topoisomerase inhibition

*S. Sharma[1], Y.R. Pokharel[1], K. Haider[2], M. Shahar Yar[2]*

*[1]South Asian University, Faculty of Lifesciences and Biotechnology, New Delhi, India*

*[2]Jamia Hamdard, Department of Pharmaceutical Chemistry- School of Pharmaceutical Education and Research, New Delhi, India*

**Introduction**

In drug discovery, indole-pyrazoline derivatives show promise as potent anticancer agents, targeting DNA topoisomerase IIα. These compounds are synthesized and evaluated for their antiproliferative activity against cancer cell lines, with a focus on their mechanism of action and safety profile. Our research combines experimental and computational analyses to advance understanding and development of effective anticancer therapies.

**Material and Methods**

Synthesized derivatives (7a-l) were tested on A431, HeLa, and MDA-MB-231 cancer cell lines using MTT assay. Mechanistic investigations included topo IIα inhibition assay, flow cytometry for cell death and cycle arrest, and western blot for protein regulation. In vivo assessments followed IAEC guidelines. Molecular dynamics simulations enhanced understanding of protein-ligand interactions.

**Results and Discussions**

ndole-bearing pyrazoline derivatives 7a and 7b demonstrated potent inhibitory activity against A431 cells, with IC50 values of 3.17 μM and 5.16 μM, respectively, compared to doxorubicin (IC50 2.63 μM). Due to their high cytotoxicity and selectivity index, 7a and 7b underwent mechanistic studies. Significant reduction in clonogenic growth and inhibition of A431 cell migration were observed with 7a and 7b. Cell cycle analysis revealed S-phase arrest induced by both compounds, with 7a triggering notable cell death in A431 cells. Western blot analysis showed decreased expression of procaspases 3 and 9, and anti-apoptotic protein Bcl-xL, along with upregulation of proapoptotic protein Bax. Both compounds exhibited significant inhibition of topo IIα-mediated relaxation, implicating topo IIα inhibition in their anticancer activity. Oral acute toxicity studies indicated LD50 values of >500 to >2000 mg/kg for 7a and 7b, compliant with OECD recommendations. Histopathological examination revealed normal tissue architecture in treated heart and liver tissues. Molecular docking studies highlighted strong binding patterns of 7a and 7b with the topo IIα receptor (PDB ID: 1ZXM), underscoring their potential as effective anticancer agents.

**Conclusion**

Novel indole-pyrazoline derivatives (7a and 7b) show potent anticancer effects against A431 cells, inhibiting colony formation, reducing cell migration, inducing S phase arrest, and triggering significant cell death. They also inhibit topo IIα, exhibit low toxicity, and interact strongly with the receptor, suggesting promising anticancer agents.

### EACR2024-0138

### Development of novel long-acting leuprolide acetates and the studies for their physicochemical and pharmacokinetic/pharmacodynamic (PK/PD) properties

*S. Lee[1], M. Kim[1], W. Shin[1], J.H. Kim[1]*

*[1]Inventagelab, Inventagelab, Seongnam-si, South Korea*

**Introduction**

Leuprolide acetate (LEUP) is an agonist of gonadotropin-releasing hormone receptors (GnRHR). Chronic administration of LEUP results in pituitary GnRHR desensitization and, eventually, a significant decline in testosterone (TT) production to castration levels which may inhibit androgen receptor-positive tumor progression. However, initial overstimulation of GnRHR could lead to TT production, which emphasizes the necessity of long-acting LEUP without initial burst release. Herein, 3 types of microsphere suspensions for extended-release of LEUP (IVL-LEUPs: IVL3008, 3009 and 3016) with i) constant release of the drug, ii) sustained PD profiles, and iii) no initial bursts are introduced.

**Material and Methods**

IVL3008/3009/3016 were prepared by IVL-DrugFluidic® platform, a microfluidics-based manufacturing technology developed by Inventage Lab. The morphology of the microspheres was examined by scanning electron microscopy (SEM). The size of the

© 2024 The Authors. Molecular Oncology is published by John Wiley & Sons Ltd on behalf of Federation of European Biochemical Societies microparticles was measured by a laser particle size analyzer (PSA). The pharmacological (PK/PD) studies of LEUP-loaded microparticles were conducted with the beagle dogs for 3, 1 and 6 months for IVL3008, 3009 and 3016, respectively.

**Results and Discussions**

The formulated microparticles of all three IVL-LEUPs had smooth spherical surfaces and uniform size according to SEM images and PSA results. In the preclinical PK study, the plasma concentrations of LA were maintained within therapeutic castration level for pre-determined durations. More importantly, the PK profiles showed no initial bursts, sustained drug release for the durations and lowered maximum concentration of the drug (0.077 - 0.001-fold) compared to FDA-approved LEUP depots. As shown in PD studies, the plasma TT level was below a castrated level (< 0.5 ng/mL) without the initial increase when treated by IVL-LEUPs, meanwhile, the reference drugs showed initial sharp surges of TT levels (6 - 14.4-fold to the castrated level).

**Conclusion**

IVL-DrugFluidic® technology allowed us not only to optimize various formulations but also to manufacture the microspheres with high uniformity and precisely controlled physicochemical properties. The analysis taken together with PK/PD studies indicates that IVL-LEUPs showed improved safety profiles and less probability of initial overproduction of TT compared to references, presumably due to precise control over their drug-releasing capabilities. Based on the positive results, Phase I IND-enabling studies are ongoing.

### EACR2024-0154

### Cuphoralix: Copper(I) Ionophore for Lung Cancer Therapy

*P. Lelievre[1], N. Renier[2], L. Sancey[1], J.L. Coll[1], I. Jabin[3], H. Valkenier[2], A. Deniaud[4], B. Busser[1]*

*[1]Univ. Grenoble Alpes - Inserm U1209 - CNRS UMR 5309 - Institute for Advanced Biosciences, Team: « Cancer targets and Experimental therapeutics », Grenoble, France*

*[2]Universite libre de Bruxelles - Ecole polytechnique de Bruxelles, Engineering Molecular NanoSystems, Bruxelles, Belgium*

*[3]Universite libre de Bruxelles - Faculty of Sciences, Laboratoire de Chimie Organique, Bruxelles, Belgium*

*[4]Univ. Grenoble Alpes - CNRS - CEA - Interdisciplinary Research Institute of Grenoble, Chemistry and Biology of Metals laboratory, Grenoble, France*

**Introduction**

Dysregulated copper (Cu) homeostasis in cancer cells makes targeting Cu metabolism a promising therapeutic approach in oncology.Our team developed Cuphoralix, a new Cu ionophore that disrupts lung cancer metabolism. Previously, we showed that this molecule blocks the cell cycle irreversibly and disrupts crucial intracellular signaling for cancer cell migration and proliferation.The synergistic association of Cuphoralix with Cu+ salts effectively blocks lung cancer proliferation, presenting an innovative and promising anti-cancer strategy. Given Cuphoralix's lipophilic nature, we focused on developing the most appropriate formulation to optimize drug delivery before commencing in vivo studies.

**Material and Methods**

We developed two liposomal formulations: endocytic (DOPE:Chol:PEG-PE) and fusogenic (DMPC:DOTAP: PEG-PE) liposomes. Both formulations included the addition of a fluorescent lipid (NBD-PC) to enable monitoring of their properties by microscopy (474nm/ 533nm). Characterization involved DLS and UPLC measurements to determine the degree of encapsulation. Liposome viability (IC50) was tested on lung cancer cell lines (H322, A549). Additionally, we investigated potential synergy with concomitant trace element treatments such as Cu, Zn, and Fe. We quantified the uptake of Cu, Zn, and Fe in the presence of Cuphoralix using ICPMS. Lastly, we monitored the variation in mRNA levels of specific metal stress proteins (met, hspa6, il-8) via qRT-PCR.

**Results and Discussions**

We synthesized fusogenic and endocytic neutral liposomes with a diameter of 130 nm.The endocytic liposomes exhibited a superior encapsulation rate compared to the fusogenic liposomes. We confirmed their ability to fuse with the plasma membrane or undergo endocytosis, respectively. Both formulations demonstrated toxicity in lung cancer cell lines. We induced metallic stress, which intensified when Cuphoralix was combined with Cu+ salts. Intracellular Cu levels increased with Cuphoralix, with no significant uptake of Zn or Fe observed. Additionally, synergistic antiproliferative effects on lung cancer cell lines were observed with Cu ion addition, but not with other trace elements.

**Conclusion**

*Cuphoralix* disrupts Cu uptake in cells and induces metal stress. The overall effects of *Cuphoralix* on cancer cells are only amplified in the presence of Cu salts. The various formulations obtained will allow us to conduct the following preclinical in vivo studies, initially on chicken embryos (eggs), and subsequently on mice with lung tumors.

### EACR2024-0175

### Converting a patient-derived xenograft biobank in patient-derived xenograft organoids (PDXO) as a new preclinical model for high-throughput drug screening

*S. Clerici[1], A. Beserra[1], L. Leite[2], S. Zequi[3,4], T.G. Santos[1,4], V.R. Martins[4,5]*

*[1]AC Camargo Cancer Center, International Research Center, São Paulo, Brazil*

*[2]AC Camargo Cancer Center, Medical Oncology, São Paulo, Brazil*

*[3]AC Camargo Cancer Center, Reference Center of Urology, SãoPaulo, Brazil*

*[4]AC Camargo Cancer Center, National Institute for Science and Technology in Oncogenomics and Therapeutic Innovation, São Paulo, Brazil*

*[5]University of São Paulo, Biomedical Sciences Institute, São Paulo, Brazil*

**Introduction**

Patient-derived organoids (PDO) and patient-derived xenografts (PDX) are known to recapitulate morphology,

© 2024 The Authors. Molecular Oncology is published by John Wiley & Sons Ltd on behalf of Federation of European Biochemical Societies genomic, genetic heterogeneity, and response to treatment of the patient's tumor. They represent potential models for new drug discovery. PDOs and PDXs present complementary characteristics, mimicking tumor evolution and metastasis, thus allowing drug screening. The numerous existing PDX biobanks show a remarkable opportunity to generate patient-derived xenograft organoids (PDXOs), allowing high-throughput screening without complex and expensive activities of identifying, recruiting, and biopsy of new patients. The AC Camargo Cancer Center PDX Biobank included several cases of renal cell carcinoma (RCC) and many cases of clear cell Renal Cell Carcinoma (ccRCC) with the patient's complete clinical history. The disease control of patients with locally advanced or metastatic RCC is challenging, and more efficient drugs are an unmet clinical need. The present work aims to establish and validate PDXO to predict tumor response to standard-of-care treatments.

**Material and Methods**

As a proof-of-concept, nine PDX samples were enrolled (fresh and snap-frozen in vitrification solution) derived from ccRCC patients. Tumor samples were enzymatically digested, cells recovered and seeded in growth factor reduced Geltrex® and, then cultured in a kidney organoid medium. PDXO were histological and immunohistochemical characterized. This approach prompted us to evaluate PDXO vulnerabilities as a powerful tool for drug repositioning and new drug discoveries. PDXOs were treated with stand-of-care drug (Sunitinib) and their sensitivity were evaluated in cell viability assays. The correlation of PDXO sensitiviy and patient clinical response were analyzed.

**Results and Discussions**

Our efficiency of generation, growth, and expansion of PDXO is approximately 40% and four PDXO lines were characterized. A comprehensive immunohistochemical and histological characterization demonstrated that PDX and PDXOs preserved the matched patient tumor's characteristics. PDXOs were treated with Sunitinib according to the therapy regimens of the matched patient's tumor and showed similar sensitivity to the patient clinical response.

**Conclusion**

These results indicate that PDX previously generated and deposited in biobanks represent an unprecedented opportunity to generate reliable and less expensive PDXO models, compared to PDO, that could quickly become applied to high-throughput drug screening studies.

### EACR2024-0187

### Ex vivo patient-derived tumour slices as a tool for cancer drug discovery

*F. Garcon[1], L. Dallaway[1], J. Hair[1], J. Eyles[1]*
*[1]AstraZeneca, Oncology R&D, Cambridge, UK*

**Introduction**

Immuno-oncology (IO) therapies for cancer have recently delivered step-change improvements in patient outcomes, but the major proportion of patients remain unresponsive or become refractory to IO treatment. Because IO-based approaches necessarily rely on the interplay of multiple cell types, including immune cells, and other elements that constitute the tumour microenvironment, innovative assays are critical to generate further translational insight. Indeed, results from in vivo mouse models and in vitro assays with human cell lines often do not correlate with clinical efficacy and as such, do not always represent relevant avatars.

**Material and Methods**

We have leveraged recent advances in patient-derived tumour slice culture (PDTSC) techniques to enable in vitro mechanistic and novel biomarker testing/ identification studies. This technique involves the culture of thick slices of fresh tissue prepared using a vibratome and is currently the only in vitro assay system which retains the 3-dimensional architecture of the tumour microenvironment, complete with cell-cell and cell-matrix interactions.

**Results and Discussions**

We found that a large variety of tumour type was amenable to slicing and culture for several days, giving us the opportunity to test several therapeutic modalities including modified RNA, bispecific antibodies, T cell engagers and cell therapy. So far, the data generated using this platform has informed the underlying biology and translational strategies of multiple drug candidates.

**Conclusion**

Overall, our results validated this platform for assessing drug responses in patient samples ex vivo, and its use as an additional tool for future cancer drug discovery and development workstreams.

### EACR2024-0200

### Natural compound as anticancer agent: in vitro experimental evidence in Intrahepatic Cholangiocarcinoma

*S. Paradiso[1], G. Carpino[1], B. Botta[2], D. Quaglio[2], C. Di Meo[2], P. Infante[3], L. Di Marcotullio[3], E. Gaudio[1], D. Alvaro[4], V. Cardinale[4]*
*[1]Sapienza - University of Rome, Department of Anatomical- Histological- Forensic Medicine and Orthopedics Sciences, Rome, Italy*
*[2]Sapienza - University of Rome, Department of Chemistry and Technologies of Drugs, Rome, Italy*
*[3]Sapienza - University of Rome, Department of Molecular Medicine, Rome, Italy*
*[4]Sapienza - University of Rome, Department of Translational and Precision Medicine, Rome, Italy*

**Introduction**

Intrahepatic cholangiocarcinoma (iCCA) constitutes a highly aggressive and heterogeneous biliary tract cancer bearing a fatal prognosis (5-years relative survival rate). Due to its aggressiveness, there is an urgent need for therapeutic alternatives since advances in iCCA therapy represent an unfulfilled need in cancer research. Its heterogeneity is mirrored by an innovative classification into small and large bile duct iCCA. The molecular pathogenesis of iCCA is very elaborate and embroils distinct molecular networks: among them, Hedgehog (Hh) pathway plays a critical role in tumor survival, proliferation, migration and epithelial-mesenchymal transition reprogramming. Evidence on the pathogenetic role of Hh in iCCA connotes the possibility of targeting this signaling pathway for therapeutic intents in iCCA. The main purpose of this study is to shed light on a new natural compound, named Glabrescione B (GlaB), able to

© 2024 The Authors. Molecular Oncology is published by John Wiley & Sons Ltd on behalf of Federation of European Biochemical Societies selectively inhibit Gli1 (Hh downstream transcriptional factor), in vitro in established and primary cell lines.

**Material and Methods**

The dose-response effect of free GlaB and hyaluronic acid (HA)-encapsulated GlaB (HA-GlaB) has been assessed by Trypan Blue Exclusion test. The target protein expression levels have been analysed by Western blot. The cell migratory activity has been evaluated by Wound healing assay. Colony formation has been evaluated by clonogenic formation assay. Cell death has been explored by Flow cytometry analyses. All results were confirmed in at least three independent experiments and all quantitative data were reported as the mean ± SD. Student's t-tests for unpaired samples were used to assess differences among two groups. A p-value of $<0.05$ was considered statistically significant (n.s., nonsignificant, $p > 0.05$; $*p < 0.05$; $**p < 0.01$; $***p < 0.001$).

**Results and Discussions**

Our research illustrates a decrease in iCCA cell rate viability, migration, colony formation and a Gli1 levels depletion in a dose- and time-dependent manner after both free GlaB and HA-GlaB treatments ($0.05<p<0.001$), leading to a significant reduction of invasiveness and aggressiveness of cancer cells. Eventually, flow cytometry preliminary data shows cell death induction, as a consequence of drug administration compared to controls.

**Conclusion**

Aberrant Hh pathway activation is widely known to be closely related with the development and progression of various cancers, including iCCA. These data represent the cornerstones for in vivo pre-clinical studies of HA-encapsuled GlaB in iCCA.

### EACR2024-0229
### Novel Covalent Ligand Modulates Proteasome Activity in Colorectal Cancer

H.Y.Y. Yip[1], T.Y. Koo[1], C.Y.S. Chung[1,2,3]
[1]The University of Hong Kong, School of Biomedical Sciences- Li Ka Shing Faculty of Medicine, Hong Kong, Hong Kong-China
[2]The University of Hong Kong, Department of Pathology- Li Ka Shing Faculty of Medicine, Hong Kong, Hong Kong-China
[3]Centre for Oncology and Immunology, Hong Kong Science Park, Hong Kong, Hong Kong-China

**Introduction**

The global prevalence of colorectal cancer (CRC) is on the rise, and coupled with the growing resistance towards treatment, it is imperative to develop novel anticancer compounds. The ubiquitin-protease system (UPS) regulates many proteins associated with carcinogenesis. As part of the system, unwanted proteins undergo degradation by the 26S proteasome. An inhibition of proteasome activity leads to the accumulation of polyubiquitinated proteins and proteasomal stress. Since cancer cells generally exhibit higher sensitivity to proteasomal stress compared to normal cells, proteasome inhibitors are effective in treating cancers. However, drug resistance has been observed in some cancer patients treated with current proteasome inhibitors that target the catalytic core in the 26S proteasome. Developing a new class of proteasome inhibitors with different mechanisms of action from existing ones would be advantageous in addressing drug resistance.

**Material and Methods**

From the screening of our in-house covalent compound library, we have identified the lead compound, 2F3, with promising killing of colorectal cancer cells HCT116, HT29 and SW620. Chemoproteomics experiment and covalent docking enable us to identify the protein target of 2F3. To further investigate its anticancer effects, we have performed cell viability, wound healing, and Boyden chamber assays to examine changes in proliferation, migration, and invasion, respectively, in CRC cells upon 2F3 treatment. Changes in proteasomal activity have been investigated in 2F3-treated cells by the Proteasome-Glo™ assay. Western blotting has been performed to study changes in K48-linked ubiquitination associated with proteasomal degradation.

**Results and Discussions**

Our results show that a 19S regulatory particle of proteasome is the protein target of 2F3. 2F3 treatment mediates a promising inhibition of proteasome activity in CRC cells and changes in ubiquitination levels of cellular proteins, resulting in a significant decrease in cancer cell viability, cell migration and cell invasion.

**Conclusion**

Through covalent ligand screening and the application of chemoproteomics, we have identified a new drug target and developed a novel covalent ligand to modulate proteasome activity for the treatment of CRC.

### EACR2024-0240
### Preclinical development of FTX-001: First-in-class inhibitor of the long non-coding RNA MALAT1

M.A. Neveu[1], A. Revenko[2], T.W. Kim[2], X. Luo[2], K. Youngsoo[2], J. Schmidt[2], A.R. MacLeod[1,2], A.E. Denker[3]
[1]Flamingo Therapeutics, Flamingo Therapeutics, Leuven, Belgium
[2]Ionis Pharmaceuticals Inc, Ionis Pharmaceuticals Inc, Carlsbad, United States
[3]Flamingo Therapeutics, Flamingo Therapeutics, Philadelphia, United States

**Introduction**

Long non-coding RNAs (lncRNAs) can act as oncogenes and are altered in various cancer types. Among them, Metastasis-Associated Lung Adenocarcinoma Transcript 1 (*MALAT1*) is highly expressed in multiple cancer types and associated with disease progression in patients. *MALAT1* is highly upregulated in metastatic lesions of breast cancer patients. In preclinical breast cancer models, both genetic and pharmacological inhibition of *MALAT1* lead to changes in tumor architecture and reduction in metastasis induced by changes in expression of genes involved in epithelial-to-mesenchymal transition. As a nuclear retained lncRNA, *MALAT1* is highly sensitive to RNaseH1-dependant antisense oligonucleotide (ASO) degradation, because RNaseH1 is also predominantly localized to the nucleus. Thus, *MALAT1* represents an attractive novel therapeutic target uniquely tractable to ASOs with the potential to impact metastatic cancer progression. Here, we describe the identification and characterization of FTX-001, a novel ASO targeting the

Molecular Oncology 18 (Suppl. 1) (2024) 1–495
© 2024 The Authors. Molecular Oncology is published by John Wiley & Sons Ltd on behalf of Federation of European Biochemical Societies human *MALAT1* lncRNA, leading to its selection as a clinical drug candidate for human cancer treatment.

**Material and Methods**

FTX-001 is a Gen 2.5 ASO with constrained ethyl (cEt) chemistry. Preclinical pharmacology, tolerability, pharmacokinetic (PK) and toxicity studies were conducted in rodents and monkeys to demonstrate its potency and safety. Finally, a plasma protein binding (PPB) assay was performed in mouse, monkeys and human plasma to inform on bioavailability.

**Results and Discussions**

FTX-001 showed a consistent potent inhibition of *MALAT1* RNA expression in human cancer cell lines in vitro as well as in human xenograft tumor models in vivo upon systemic delivery. Adequate exposure, tissue distribution and tolerability profile were observed in mice, rats and NHP after repeated subcutaneous (sc) administrations. Results from 6-week non-clinical toxicity testing in mice (sc administration up to 45mg/kg) and NHP (intravenous administration up to 24mg/kg) revealed no unexpected toxicity findings during both dosing and recovery phases. The No-Observed-Adverse-Effect Level was the top dose studied in both species. FTX-001 plasma PK tissue distribution and PPB results were consistent with that expected with ASOs in the tested species.

**Conclusion**

Taken together, these results demonstrate that FTX-001 has the potential to achieve target engagement (*MALAT1* knockdown) in tumors at tolerated doses in cancer patients and has a preclinical benefit-risk profile supportive of advancement to Phase 1 human trials.

### EACR2024-0245

### The lncRNA LINT interacts with DHX36 to regulate translation and mitochondrial function in melanoma

*A. Haller*[1], *G. Gambi*[2], *M. D'Agostino*[3], *G. Davidson*[1], *G. Mengus*[1], *A. Simonetti*[3], *E. Ennifar*[3], *I. Davidson*[1]

[1]*IGBMC, Functional Genomics and Cancer, Illkirch, France*

[2]*NYU Langone Medical Center, NYU Grossman School of Medicine, New York City, United States*

[3]*IBMC, RNA architecture and reactivity, Strasbourg, France*

**Introduction**

Melanoma represents only 1 % of skin cancers, but is responsible for the majority of skin cancer deaths. Immunotherapy and targeted therapies are current first line treatments for melanoma patients. Nevertheless, acquired and intrinsic resistance as well as adverse side effects challenge researchers in finding new targets and new therapies. Long noncoding RNAs (LncRNAs), represent novel potential therapeutic targets due to their highly specific expression patterns and the fact that they can be targeted with antisense oligonucleotides (ASOs). Several lncRNAs have been shown to drive cancer cell proliferation, survival and metabolism. In this context, we identified a LncRNA designated LINT strongly expressed in melanoma compared to other cancers or tissues.

**Material and Methods**

LINT was depleted in melanoma cell lines and in melanoma CDX in mice using ASOs. A selective interaction of LINT with DHX36 was identified by mass spectrometry after LINT pulldown under native and UV crosslinked conditions. DHX36 was localized within cells by immunofluorescence using specific antibodies. The impact of LINT on mRNA association with DHX36 was studied by RNA-sequencing after immuno-precipitation of DHX36. Effects of LINT depletion on translation were determined by polysome fractionation and immunoblot.

**Results and Discussions**

We found that LINT is highly expressed in melanocytic cells, but display low or no expression in mesenchymal cells. ASO targeting of LINT shows it is essential for melanoma proliferation, survival and tumour formation. We showed that LINT interacts with DHX36, a G4 helicase, pointing to a role in translation of mRNAs containing G-quadruplex. LINT and DHX36 associate with the 80S ribosome particle and localize to mitochondria in melanoma cells. LINT silencing led to increased association of a collection of mRNAs encoding mitochondrial proteins with DHX36 increasing their translation. Paradoxically, maximal oxidative phosphorylation metabolism is reduced, with the excess mitochondrial proteins accumulating in the cytoplasm to induce mitophagy and mPOS mediated apoptosis.

**Conclusion**

LINT is a novel LncRNA required for melanoma cell proliferation and survival. By interacting with the G4 resolvase DHX36, LINT finely regulates translation of mitochondrial proteins to promote mitochondrial respiration and inhibit mitophagy. The finding that the lncRNAs LINT, LENOX and SAMMSON all converge to optimize mitochondrial function, albeit by different mechanisms, underscores the critical role of mitochondria in melanoma.

### EACR2024-0251

### Development and biological evaluation of PROTAC molecules directed against the anti-apoptotic proteins Mcl-1 and/or Bcl-xL for the treatment of ovarian cancers

*J. Pezeril*[1], *M. Cornu*[2], *T. Lemaitre*[2], *F. Schwalen*[2], *H. Paysant*[1], *N. Guedeney*[2], *L.B. Weiswald*[1,3], *C. Kieffer*[2], *A.S. Voisin-Chiret*[2], *L. Poulain*[1,3]

[1]*Université de Caen Normandie, Inserm U1086 ANTICIPE, Unité de Recherche Interdisciplinaire pour la Prévention et le Traitement des Cancers, Caen, France*

[2]*Université de Caen Normandie, UR 4258 CERMN – Centre d'Etudes et de Recherche sur le Médicament de Normandie, Caen, France*

[3]*UNICANCER, Centre de Lutte Contre le Cancer François Baclesse, Caen, France*

**Introduction**

The development of new therapeutic strategies is a major challenge for improving the management of ovarian cancers. The anti-apoptotic proteins Mcl-1 and Bcl-$x_L$, whose expression is strongly correlated with resistance to treatments, are relevant targets and we have previously developed an original Mcl-1 inhibitor, Pyridoclax. But the development of pharmacological inhibitors of these

© 2024 The Authors. Molecular Oncology is published by John Wiley & Sons Ltd on behalf of Federation of European Biochemical Societies proteins is currently hampered by on-target toxicities: thrombocytopenia associated to Bcl-$x_L$ inhibition and cardiotoxicity associated to Mcl-1 inhibition. PROTAC (PROteolysis TArgeting Chimera) technology, which induces tissue-specific targeted degradation of proteins by the ubiquitin-proteasome system, could be used appropriately in this context. This strategy involves combining a protein-targeting ligand and a ligand targeting an E3 ubiquitin ligase, absent from the tissues to be avoided but present in the tumour. On the basis of this technology, we have developed PROTAC molecules based on Pyridoclax or its derivatives fused to ligands of VHL and CRBN E3 ubiquitin ligases (slightly or not expressed in platelets and heart but expressed in ovarian cancers).

**Material and Methods**

42 molecules (37 with CRBN ligand, 5 with VHL ligand) have been synthetized and evaluated in the ovarian chemoresistant tumor cell line, IGROV1-R10, and their effects on Bcl-$x_L$ and Mcl-1 protein expressions were assessed by western blot and quantified.

**Results and Discussions**

Among the 42 molecules evaluated, 6 molecules showed high Mcl-1 degradation activity, with degradation percentages ranging from 77% to 94% at nanomolar concentrations, after 48h of exposure. Regarding Bcl-$x_L$, the degradation activity of the selected molecules is generally low, excepted for 3 molecules showing high activity (with a maximum of 81% degradation at 10 nM after 48h exposure). Their degradation efficacy in other ovarian tumor lines as well as their ability to induce cell death in combination with Bcl-$x_L$ inhibition is currently evaluated. The use of a panel of patient-derived tumor organoids (PDTO) is also planned to validate these results.

**Conclusion**

We demonstrated the efficacy of original PROTAC molecules to induce a strong degradation of the anti-apoptotic Mcl-1 or Bcl-$x_L$ proteins in ovarian tumor cells. Further validation studies regarding their ex vivo (PDTO) and in vivo (PDX, patient-derived xenografts) activities are now required, as well as their interest to lead to cancer cell death, alone or in combination with other anticancer agents.

### EACR2024-0252

### PP2A triggers the cell death signaling cascade after treatment with PEP-010, a first-in-class clinical-stage pro-apoptotic peptide

*A. Lacroix[1], L. Lhoste[1], C. Brenner[2], J. Wiels[2], D. Germini[1]*

*[1]PEP-Therapy, Research and Development, Paris, France*

*[2]CNRS- Institut Gustave Roussy- Université Paris-Saclay, Umr9018- metabolic and systemic aspects of oncogenesis for new therapeutic approaches, Villejuif, France*

**Introduction**

PP2A is a serine/threonine phosphatase which regulates several cellular pathways such as apoptosis, cell proliferation and DNA damage response. It is functionally inactivated in many cancers. PP2A can act on different downstream targets by dephosphorylating them at specific sites, and thereby activates or inactivates them. Major direct or indirect targets of PP2A are (i) certain members of the BH3 family, tightly correlated to mitochondrial membrane integrity and consequently to apoptosis regulation, (ii) caspase-9, (iii) Akt, (iv) Erk and (v) Myc. PEP-010 is a first-in-class pro-apoptotic peptide, developed by PEP-Therapy, a French clinical-stage biotech company. Briefly, PEP-010 induces apoptosis in cancer cells by disrupting the interaction between caspase-9 and PP2A. This interaction prevents these proteins to play their individual physiological roles in the apoptotic pathway. PEP-010 is currently evaluated in a Phase Ib multicenter clinical trial for the treatment of Platinum-Resistant Ovarian Cancer and Pancreatic Ductal Adenocarcinoma.

**Material and Methods**

Cell models of different origins were treated with PEP-010 and Okadaic acid, an inhibitor of PP2A, to probe the role of PP2A. Annexin-V and Propidium Iodide staining followed by flow cytometry analysis was used to assess apoptosis. Different steps of the mechanism of action were demonstrated by specific staining and probes followed by flow cytometry and/or western blot analysis.

**Results and Discussions**

We demonstrated that PP2A works as an early trigger of the PEP-010-induced apoptosis both in solid and in liquid tumors. Exploiting leukemia models resistant to Venetoclax, where PEP-010 showed a high efficiency, we demonstrated that rapidly upon entry of PEP-010 into the cells, PP2A dephosphorylates specific members of the BH3 family leading to the loss of mitochondrial membrane potential and consequently caspases activation.

**Conclusion**

This study presents the first proof of concept of PP2A involvement in PEP-010-induced cell death and of PEP-010 efficacy in models of Venetoclax resistant hematological malignancies. Our work underlines the interest of PP2A as a therapeutic target and as a key protein for apoptosis regulation. Moreover, it paves the way to further in vivo studies which could grant for a possible extension of the therapeutic indication in PEP-010 clinical development. If conclusive, PEP-010 could represent a valid therapeutic opportunity for Venetoclax-resistant populations.

### EACR2024-0253

### Ovarian patient-derived tumor organoids: towards innovative tools to predict the response to chemotherapy and PARP inhibitors

*L. Thorel[1,2], N. Elie[3], R. Florent[4], C. Blanc-Fournier[1,5,6], F. Joly[1,7], M. Figeac[8], R. Leman[9], E. Dolivet[1,10], L.B. Weiswald[1,2,4], L. Poulain[1,2,4,5]*

*[1]Université de Caen Normandie, INSERM U1086 ANTICIPE- BioTICLA laboratory, Caen, France*

*[2]UNICANCER, Comprehensive Cancer Center François Baclesse, Caen, France*

*[3]Université de Caen Normandie, US PLATON-*

Molecular Oncology 18 (Suppl. 1) (2024) 1–495

© 2024 The Authors. Molecular Oncology is published by John Wiley & Sons Ltd on behalf of Federation of European Biochemical Societies

*VIRTUAL'HIS Platform, Caen, France*
[4]*Université de Caen Normandie, US PLATON-ORGAPRED core facility, Caen, France*
[5]*Université de Caen Normandie, US PLATON-Biological Resource Center 'OvaRessources', Caen, France*
[6]*UNICANCER, Comprehensive Cancer Center François Baclesse- Department of Biopathology, Caen, France*
[7]*UNICANCER, Comprehensive Cancer Center François Baclesse- Clinical Research Department, Caen, France*
[8]*University of Lille, CNRS- Inserm- CHU Lille- Institut Pasteur de Lille- US 41 - UAR 2014 - PLBS, Lille, France*
[9]*UNICANCER, Comprehensive Cancer Center François Baclesse- Department of Cancer Biology and G enetics, Caen, France*
[10]*UNICANCER, Comprehensive Cancer Center François Baclesse- Department of Surgery, Caen, France*

**Introduction**

Ovarian cancers are the second cause of death from gynecological cancers worldwide, due to a late diagnosis combined with the development of resistance to chemotherapy. However, half of these cancers present alterations in homologous recombination (HR), making them sensitive to inhibitors of the PARP protein (PARPi), involved in DNA repair. Nevertheless, identifying patients who respond to chemotherapy and selecting those eligible for PARPi remains a challenge for clinicians. In this context, the use of patient-derived tumor organoids (PDTO) for predictive functional testing represents an interesting prospect to guide 1st line and following therapeutic choices. The aim is to study the feasibility of a PDTO-based functional assay in order to evaluate its potential for clinical purposes.

**Material and Methods**

30 PDTO models were successfully generated from 27 ovarian cancer patients of various histological sub-types. The histological and molecular relevance of PDTO was assessed by comparing their features to the ones of the tumor of origin. Both immunohistochemical analyses and global approaches (CGH array and transcriptomic profiling) were performed. For direct exposure functional assay, PDTO models were exposed to 6 drugs belonging to the 1st and 2nd lines of treatment including PARPi. To further define the HR status of PDTO, we performed a functional assay evaluating the ability of PDTO to initiate HR (RECAP test) using an original automated histo-imaging quantitative analysis of RAD51 foci, as well as an NGS analysis based on the sequencing of an HR-related genes panel to obtain a genome instability score (GIS).

**Results and Discussions**

PDTO models showed histological and molecular characteristics close to the ones of their tumor of origin. We showed that the PDTO models identified by the predictive assay as sensitive to treatments mainly derived from responder patients. Moreover, we were able to correlate the results of RECAP test with NGS-based HR status, themselves partially correlated to the results of carboplatin/PARPi direct exposure assay. These results require further investigations to explain these discrepancies.

**Conclusion**

We generated a panel of ovarian PDTO of various histological subtypes that recapitulates the initial tumor features, can be used for functional assays and displays heterogenous responses to standard-of-care treatments in a clinical correlated manner. These results provide further arguments for the use of ovarian PDTO in the context of precision medicine.

### EACR2024-0256
### Adenosine receptor A2A as a potential target for diagnosis and therapy in pseudohypoxic Pheochromocytomas and Paragangliomas (PPGLs)

*B. Karna*[1], *H. Mohr*[1], *S. Gulde*[2], *N. Bechmann*[3], *F. Schilling*[4], *W. Deuther-Conrad*[5], *S. Herzig*[1], *N. Pellegata*[1]
[1]*Helmholtz Center Munich, Institute for Diabetes and Cancer, Munich, Germany*
[2]*CureVac, Analytical Development, Tübingen, Germany*
[3]*Institut für Klinische Chemie und Laboratoriummedizin, Universitätsklinikum Carl Gustav Carus an der Technischen Universität Dresden, Dresden, Germany*
[4]*Biomedical Magnetic Resonance, Nuclear Medicine-Klinikum Rechts der Isar, Munich, Germany*
[5]*Neuropharmaceuticals, Helmholtz-Zentrum Dresden-Rossendorf, Dresden, Germany*

**Introduction**

In the tumor micro-environment, adenosine signaling via the adenosine receptor A2a (A2A/Adora2a) has been shown to play a role in promoting immune system evasion. Activation of intrinsic A2A signaling is also associated with tumor cell migration, branching and proliferation. A2A expression can be induced by hypoxia inducible factors (HIFs). We found A2A to be over-expressed in experimental in vitro and in vivo models of pseudohypoxic PPGLs (p-PPGLs). These tumors are aggressive, orphan of effective therapies, and associated to high morbidity and mortality. Given the need to identify novel targets for p-PPGLs, we investigated the role of A2A in the progression of these tumors.

**Material and Methods**

mRNA and protein levels of A2A were determined in PC12 cells and pheochromocytoma tissues from MENX rats via qRT-PCR, immunostaining, and western blotting analysis. The role of A2A in cell proliferation was investigated in the presence/absence of the A2A specific antagonist istradefylline using viability assays. Adenosine levels were measured in cells and tissues. Istradefylline was injected in MENX rats and tumor size was monitored over time. A2A specific radiolabeled tracer was used for autoradiography of the rat p-PPGLs.

**Results and Discussions**

The expression of A2A and Hif2α (Epas1) was significantly higher in PC12 cells having a hypoxic signature than in PC12 cells not having it. Adenosine levels were higher in rat p-PPGLs compared to normal rat adrenal tissues. In advanced rat p-PPGLs, we found an increased expression of genes of the A2A-adenosine signaling pathway (e.g. Adora2a, Epas1 and Entpd1). Furthermore, inhibition of A2A decreased cell viability specifically in A2A-expressing PC12 cells.

© 2024 The Authors. Molecular Oncology is published by John Wiley & Sons Ltd on behalf of Federation of European Biochemical Societies

Mechanistically, inhibition of A2A signaling in p-PPGL cells leads to decreased CREB phosphorylation.

**Conclusion**

Our findings indicate a role of A2A in p-PPGL cell growth thereby suggesting that it might be suitable as a target in advanced p-PPGL. The availability of A2A-directed tracers and antagonists opens the possibility to translate our findings into novel imaging and treatment options for p-PPGLs.

### EACR2024-0289
### Inhibition of GCN2 by ALE-001 results in significant anti-tumor efficacy as a monotherapy and in combination with checkpoint inhibitors

*B. van Raam*[1], *J. Vacca*[2], *M. Albino*[1], *R. Missiaen*[3], *M. van Woensel*[3], *J. Swiercz*[1]
[1]*Alesta Therapeutics, Cell Biology, Leiden, The Netherlands*
[2]*Alesta Therapeutics, Chemistry, Leiden, The Netherlands*
[3]*Droia Ventures, Discovery, Zaventem, Belgium*

**Introduction**

Solid tumors grow in an environment with limited access to nutrients, including essential amino acids. Tumor cells have adapted to this environment by relying on the activity of the integrated stress pathways, which include the amino acid sensor GCN2 and the ER stress sensor PERK. Activation of these pathways enables increased uptake of crucial amino acids by boosting specific amino acid transporters, thus outcompeting tumor-infiltrating immune cells. This action prevents cell death caused by amino acid deprivation. In addition, certain tumors have acquired deficiencies in the rate-limiting enzyme of the urea cycle, ASS1, which uses aspartate to fuel the urea cycle, thus preventing the de novo synthesis of purines in favor of arginine production. Thus, these cells trade an increased access to purines, facilitating rapid cell division, for a chronic deficiency in arginine. This, too, leads to reliance on GCN2 to reprogram the cells to scavenge arginine from the tumor microenvironment, and to adapt to low arginine conditions. Such tumors have been shown to lead to worse clinical outcomes. To leverage these liabilities, we developed a novel inhibitor of GCN2 which is effective in killing GCN2-dependent tumor cells.

**Material and Methods**

We tested a series of GCN2 inhibitors based on a common scaffold to determine their specificity and efficiency. First, biochemical testing was conducted to determine whether compounds inhibited recombinant GCN2 and/or the kinase PERK. Then, the inhibitors were screened for their ability to inhibit GCN2 and PERK in cells using a fluorescent reporter cell line. Finally, the best compounds were tested for their ability to kill GCN2-dependent tumor cell lines over GCN2-independent cell lines in both 2D and 3D cell killing assays. In addition, our top candidate was validated in mouse tumor models.

**Results and Discussions**

Our novel compounds displayed superior selectivity compared to previously developed GCN2-inhibitors, as well as improved physico-chemical properties. Furthermore, our compounds were well tolerated and selectively killed GCN2-dependent tumors alone or in combination with SOCs.

**Conclusion**

We developed a clinical lead compound for the treatment of aggressive tumors that rely on GCN2 activity for their survival. Our molecule has potential for being the first-in-class, and validates GCN2 inhibition as a therapeutic approach for solid tumors.

### EACR2024-0294
### Multi-omics and drug response assessments in longitudinal patient avatars unveil patient-specific recurrence trajectories and drug susceptibilities in glioblastoma

*A. Lipsa*[1], *B. Nosirov*[1,2], *L. Ermini*[1], *A.C. Hau*[1], *Y. Yabo*[1], *M. Mittelbronn*[3], *F. Hertel*[4], *P.V. Nazarov*[2], *S.P. Niclou*[1,5], *A. Golebiewska*[1]
[1]*NORLUX Neuro-oncology Laboratory- Department of Cancer Research, Luxembourg Institute of Health LIH, Luxembourg, Luxembourg*
[2]*Multiomics Data Science Research Group- Department of Cancer Research, Luxembourg Institute of Health LIH, Luxembourg, Luxembourg*
[3]*National Center of Pathology NCP- Luxembourg Center of Neuropathology LNCP, Laboratoire national de sante LNS, Dudelange, Luxembourg*
[4]*Department of Neurosurgery, Centre Hospitalier Luxembourg CHL, Luxembourg, Luxembourg*
[5]*Faculty of Science- Technology and Medicine FSTM, University of Luxembourg, Belvaux, Luxembourg*

**Introduction**

Glioblastomas (GBMs) have the worst prognosis of all adult brain tumors. Despite the aggressive standard-of-care, including surgery, radio- and chemotherapy, tumors inevitably recur and median survival remains at 18 months. GBMs exhibit complex changes at recurrence, although no common evolutionary trajectory has been identified yet. Such molecular evolution may lead to functional changes in drug response patterns. Investigations of longitudinal heterogeneity of paired primary-recurrent tumors using high resolution multi-omics profiling combined with functional investigations in matched patient avatars can provide clinically-relevant insights into tumor evolution at the individual patient level.

**Material and Methods**

We applied a cohort of longitudinal patient tumors and preclinical models derived thereof, propagated as primary organoids and orthotopic xenografts. Our cohort includes a unique collection of paired primary-recurrent patient avatars derived from 8 patients during their standard-of-care disease trajectory. We performed a comprehensive multi-omics profiling, including transcriptomics (bulk and single cell RNA-seq), epigenomics (DNA methylation arrays), genomics (targeted DNA-seq) and proteomics (LC-MS) to identify molecular evolution of tumors at recurrence. We further carried out an ex-vivo functional drug screen with a pharmacologically diverse

Molecular Oncology 18 (Suppl. 1) (2024) 1–495
© 2024 The Authors. Molecular Oncology is published by John Wiley & Sons Ltd on behalf of Federation of European Biochemical Societies

1482 compound library targeting cancer pathways and epigenetic modifiers.

**Results and Discussions**

High-resolution multi-omics analysis of longitudinal patient tumors and patient avatars identified patient-specific evolution of the primary tumors at recurrence following surgical resection and treatment pressure. While certain GBMs recurred without major molecular adaptation, others showed significant (epi)genetic and transcriptomic evolution towards new genetic clones and/or transcriptomic states. Majority of drug responses were similar in primary and recurrent tumors and were patient-specific. Interestingly, we observed selective susceptibilities to several epigenetic modifiers, especially Histone Deacetylase (HDAC) and Aurora Kinase (AURK) inhibitors in certain primary tumors, which were lost at recurrence.

**Conclusion**

Our study demonstrates the importance of combined omics and functional profiling to reveal clinical implications of longitudinal evolutionary trajectories in GBMs. Our findings imply that the impact of potentially effective drugs may differ between newly diagnosed and recurrent GBMs that might have implications in precision therapy strategies.

### EACR2024-0301
### Retrograde Trafficking in TNBC Stratification: Molecular Insights and Therapeutic Implications

*R. Bingham*[1], *H. McCarthy*[1], *N. Buckley*[1]
[1]*Queen's University Belfast, School of Pharmacy, Belfast, United Kingdom*

**Introduction**

Triple Negative Breast Cancer (TNBC), an aggressive and heterogenous subtype of breast cancer (BC), accounts for 15-20% of BC cases but a disproportionate 40% of deaths. Response rates to chemotherapy vary and the effectiveness of first line treatment forecasts overall survival, with poor outcome for non-responders. Predicting response remains elusive and alternate treatment options need identified for those not gaining benefit from current standard of care (SoC). Gene expression analysis of a retrospective TNBC patient cohort has identified several genes linked to retrograde trafficking (RT), associated with outcome. RT, which describes trafficking of cargo in a plasma membrane to Endoplasmic Reticulum direction is known to be dysregulated in cancer and disease. Therefore, we aim to investigate the therapeutic potential of targeting RT or associated intracellular processes, as our data indicates patients with high RT may not respond to SoC chemotherapy.

**Material and Methods**

In silico analysis of in-house and publicly available datasets was performed, correlating relapse-free survival and mRNA expression. Gene set enrichment analysis (GSEA) and drug sensitivity analysis was performed to identify molecular pathways related to RT. In vitro assays were used to assess cancer phenotypes following modulation of RT gene expression or treatment with RT associated drugs. Identified RT related pathways were investigated, including ferroptosis.

**Results and Discussions**

A gene signature based on the combined expression of RT genes Rab6A, COPZ1, VPS35, Rab2A, ANKFY1 and FAM21A was established and validated in large publicly available datasets. A high RT gene signature score was shown to significantly predict poor outcome in TNBC patients, while a low score predicted good outcome. Modulation of individual RT gene expression impacted cancer phenotypes including proliferation. GSEA using the RT gene signature, alongside drug sensitivity analysis pointed to a potential therapeutic vulnerability of inducing ferroptosis in patients with high RT highlighting an alternative treatment approach for poor outcome patients. Cell line models chosen to represent low to high RT showed response to ferroptosis inducers including RSL3 and erastin was strongly linked to RT score, however the exact molecular mechanism linking RT and ferroptosis remains unclear.

**Conclusion**

Our RT gene signature effectively predicts outcome of TNBC patients and highlights the potential of targeting RT-related pathways for more tailored treatment strategies.

### EACR2024-0303
### Extracellular vesicles derived from breast cancer cells rich in miR-23b-3p, miR-126-3p, and GAS5 inhibited the tumor growth of zebrafish xenograft model

*I.A. Pelisenco*[1], *I. Grossi*[1], *D. Zizioli*[1], *F. Guerra*[2], *C. Bucci*[2], *L. Mignani*[1], *G. Girolimetti*[2], *E. Marchina*[1], *G. De Petro*[1], *A. Salvi*[1]
[1]*University of Brescia, Department of Molecular and Translational Medicine, Brescia, Italy*
[2]*University of Salento, Department of Biological and Environmental Sciences and Technologies, Lecce, Italy*

**Introduction**

Extracellular vesicles (EVs) are a group of nanoscale cell-derived membranous structures secreted by all cell types, which can commute biological cargoes for intercellular communication. They have notable roles in diverse physiological and pathological circumstances. Given their cargo, EVs as a mimic of "nature's delivery system" can be used to transport nucleic acids, proteins, and metabolites to target recipient cells. EVs offer a range of advantages over traditional synthetic carriers, thus paving the way for innovative drug delivery approaches.

**Material and Methods**

Here, we treated 4 different breast cancer cell lines (HCC 1937, MDA-MB-231, MCF-7, and MDA-MB-453) with sorafenib, which is a multikinase inhibitor. Then we collected their cognate EVs and characterized them using Western-Blot and Transmission electron microscopy (TEM) analysis. The levels of encapsulated miR-23b-3p, miR-126-3p, and GAS5 were quantified by Droplet Digital PCR (ddPCR). Moreover, to establish the role of the EVs as carriers of ncRNAs in vivo, we injected the MDA-MB-231 and MDA-MB-453 cells in zebrafish embryos and we treated the xenografts with two different types of EVs rich in miR-23b-3p, miR-126-3p and GAS5.

© 2024The Authors. Molecular Oncology is published by John Wiley & Sons Ltd on behalf of Federation of European Biochemical Societies

**Results and Discussions**
Results from ddPCR showed elevated levels of miR-23b-3p, miR-126-3p, and GAS5 following sorafenib treatment. Subsequently, utilizing EVs as carriers for these specific ncRNAs in breast cancer cell treatment led to a significant increase in the expression levels of all three ncRNAs, up to 7.5 times ($p<0.01$), along with a notable inhibition of cellular proliferation in vitro (up to 19%; $p<0.01$). In vivo experiments performed in zebrafish model demonstrated a remarkable reduction of xenograft tumor area (84%; $p<0.0001$, 24 hours post-treatment), suppression of angiogenesis, and decreased number of micrometastasis in the tails following the administration of EVs enriched with these ncRNAs.
**Conclusion**
Our findings indicate a new way to enrich EVs with specific tumor-suppressor ncRNAs by treating the cells with an anti-cancer drug; the role of EVs as vehicles of ncRNAs; the combined effect of miR-23b-3p, miR-126-3p and GAS5 in limiting the aggressive properties of breast cancer in vitro and in vivo. Our results may be useful to develop new potential molecular therapeutic strategies against breast cancer.

### EACR2024-0304
### Tumor-targeted extracellular vesicles carrying therapeutic siRNAs to suppress metastasis in medulloblastoma

*D. Duy Anh*[1,2], *C.L. Hsieh*[3,4], *T.T. Wong*[5], *S.Y. Sung*[1,3]
[1]*Taipei Medical University, International Ph.D. Program for Translational Science, Taipei, Taiwan*
[2]*Pham Ngoc Thach University of Medicine, Department of Physiology- Pathophysiology and Immunology, Ho Chi Minh City, Vietnam*
[3]*Taipei Medical University, The Ph.D. Program for Translational Medicine, Taipei, Taiwan*
[4]*Development Center for Biotechnology, Institute for Drug Evaluation Platform, Taipei, Taiwan*
[5]*Taipei Neuroscience Institute- Taipei Medical University Hospital, Division of Pediatric Neurosurgery-Department of Neurosurgery, Taipei, Taiwan*
**Introduction**
The landscape of fatality in medulloblastoma (MB) mainly involves post-treatment recurrences in the form of metastasis. We recently identified *LOXL1-AS1* as a significant pro-metastatic long non-coding RNA gene in the sonic-hedgehog (SHH) subgroup of MB. To target *LOXL1-AS1*, small-interfering (si)RNAs allow both effective and specific gene silencing. However, the delivery of such therapeutic materials to brain tumors is extremely challenging. To this end, extracellular vesicles (EVs) are a powerful tool to protect siRNAs from in vivo degradation, promote penetration across physical barriers, and enhance tumor-specificity via surface display of targeting molecules. Our study aimed to develop a gene therapy model using siRNA-carrying and MB-specific EVs to target SHH-MB metastasis.
**Material and Methods**
An immortalized line of bone marrow-derived human mesenchymal stem cells (3A6) was used for EV production. A sequence coding for MB-specific cell-penetrating peptide (CPP) or an epitope tag (V5-tag, as control) was fused with the membrane glycoprotein Lamp2b and then transduced to 3A6 cells to establish stable line. EVs isolated from 3A6-conditioned serum-free medium were transfected with *LOXL1-AS1*-targeting (siLOXL1-AS1) or negative control (siNC) siRNAs, followed by RNase treatment and clean-up. EV characterization was performed using nanoparticle tracking, electron microscopy, qRT-PCR, and western blotting. After incubation with labeled EVs, MB cells were evaluated for fluorescent signals using flow cytometry and confocal microscopy or subjected to functional assays, including wound-healing, transwell migration, and sphere formation.
**Results and Discussions**
Generated EVs were smaller than 200 nm in diameter with intact membrane structure and enrichment of exosomal markers, Lamp2b protein, and V5-tag. MB cells treated with Lamp2b-CPP-EVs demonstrated a high level of EV uptake compared to those treated with Lamp2b-V5-EVs or other non-MB cell lines. SiRNA-transfected EVs had an encapsulation of ~400 siRNA copies per EV particle. MB cells treated with siLOXL1-AS1-EVs showed a reduced *LOXL1-AS1* expression and a significant inhibition in cell migratory and cancer stem-like features. An in vivo study using orthotopic xenograft mice intravenously injected with EVs is being evaluated for biodistribution, biosafety, and therapeutic effects.
**Conclusion**
Our study provides a promising EV-based siRNA delivery model for specific targeting and effective silencing of pro-metastatic genes in SHH-MB.

### EACR2024-0305
### Targeting Copz1 in in vitro mouse models of Thyroid Cancer

*T. Di Marco*[1], *M. Mazzoni*[1], *S. Paglairdini*[1], *A. Greco*[1]
[1]*Fondazione IRCCS Istituto Nazionale dei Tumori, Department of Experimental Oncology, Milan, Italy*
**Introduction**
To sustain the neoplastic state cancer cells depend on normal genes, neither mutated nor aberrantly expressed, whose activity is not essential for normal cells. This dependency, known as non-oncogene addiction (NOA), could be exploited as a new strategy for cancer treatment, with the advantage of not affecting normal cells. We recently identified the coatomer protein complex ζ1 (Copz1) as an example of NOA for thyroid cancer (TC), for the aggressive forms of which no effective treatments are available. Copz1 vulnerability in TC is related to the down regulation of the Copz2 isoform. Our in vitro studies showed that Copz1 is essential for TC cells but not for normal ones. Copz1 silencing in TC cells induced endoplasmic reticulum stress that triggered inflammatory events and culminated in immunogenic cell death. To assess the effect and translational value of Copz1 depletion on cell-mediated immunity and inflammation, in vivo preclinical models are needed. Toward this aim, we have characterized the susceptibility to Copz1 inhibition of murine TC cell lines.
**Material and Methods**
We used the following murine TC cell lines: T4888M, T3531L, 3610R, 3868 and 3473. Real Time PCR (RT-PCR) was performed using TaqMan probes. Transient Copz1 silencing was performed by siRNAs transfection.

Molecular Oncology 18 (Suppl. 1) (2024) 1–495
© 2024 The Authors. Molecular Oncology is published by John Wiley & Sons Ltd on behalf of Federation of European Biochemical Societies

Cell proliferation was monitored by crystal violet assay. Protein expression was analysed by Western Blot. Cytokines expression was performed on conditioned medium by ELISA Array. For long term Copz1 silencing, 3610R and T3531L containing an inducible short hairpin RNA (shRNA) against Copz1 were produced by lentiviral transfection; Copz1 silencing was induced by doxocycline (Dox) treatment.

**Results and Discussions**

All mouse cell lines were suitable for Copz1 silencing, since RT-PCR analysis showed down regulation of the paralogue Copz2 gene, compared to a control cell line. In short term silencing of Copz1, growth inhibition was observed in four cell lines, but only in T3531L and 3610R cell lines up regulation of IFN pathway related genes and the release of several inflammatory cytokines were detected. Long term silencing of Copz1, upon Dox treatment, showed consistent results: the down regulation of the target maintained up to 7-10 days and an increase of cell death up to 7 days.

**Conclusion**

Our results show that Copz1 silencing in mouse TC cells has the same effects observed in human TC cells. The inducible Copz1 silencing cell models will be used for studying the immune anti-tumor properties of Copz1 depletion in in vivo preclinical models.

### EACR2024-0315

### Plectin as a target for suppression of hepatocellular carcinoma growth and metastasis

*Z. Outlá*[1], *K. Korelová*[1], *M. Přechová*[1], *G. Oyman-Eyrilmez*[1], *J. Kosla*[1], *D. Jirák*[2], *M. Heikenwälder*[3], *S.M. Meier-Menches*[4], *G. Wiche*[5], *M. Gregor*[1]

[1]*Institute of Molecular Genetics of the Czech Academy of Sciences, Laboratory of Integrative Biology, Prague, Czech Republic*

[2]*Institute for Clinical and Experimental Medicine, Department of Radiodiagnostic and Interventional Radiology, Prague, Czech Republic*

[3]*German Cancer Research Center, Division of Chronic Inflammation and Cancer, Heidelberg, Germany*

[4]*University of Vienna, Department of Analytical Chemistry, Vienna, Austria*

[5]*Department of Biochemistry and Cell Biology- Max Perutz Labs, University of Vienna, Vienna, Austria*

**Introduction**

The cytoskeleton plays a critical role in various cellular processes, including carcinogenesis. Plectin, a protein that crosslinks and shapes cytoskeletal networks, is upregulated in various cancers. This study investigates the role of plectin-mediated cytoskeletal crosstalk in the invasion and tumorigenic potential of hepatocellular carcinoma (HCC) cells. Additionally, it aims to validate plectin as a potential therapeutic target for HCC treatment.

**Material and Methods**

First, plectin fluorescence intensity levels were compared in tumor (T) and non-tumor (NT) areas of HCC patients´ liver tissue samples. Plectin was inactivated in human HCC cell lines either genetically using CRISPR/Cas-9 approach or pharmacologically using plecstatin. The effect of plectin depletion on tumorigenic and invasion potential of HCC cells was assessed using soft agar colony formation assay, xenograft tumor formation assay, spheroid invasion assay, and lung colonization assay. The hydrodynamic gene delivery of c-myc (Myc)-encoding element together with CRISPR/Cas9 construct targeting Tp53 (Myc;sgTp53) via tail vein injection was employed to test the therapeutic effect of plectin inactivation on HCC development.

**Results and Discussions**

Plectin fluorescence intensity levels were significantly elevated in T area when compared to adjacent NT area, suggesting plectin as novel HCC marker. Plectin-depleted HCC cells formed lower number of colonies in soft agar. Likewise, genetic and pharmacological plectin inactivation resulted in reduced xenograft tumor growth. These findings point towards plectin´s impact on HCC tumorigenic potential. Plectin-inactivated HCC cells showed reduced invasion potential in vitro. Moreover, mice receiving plectin-ablated HCC cells displayed markedly lower lung tumor burden when compared to mice injected with wild-type cells. Similar effect on lung metastatic load was observed upon plecstatin treatment of wild-type-injected mice. Furthermore, both genetic plectin ablation in $Ple^{\Delta alb}$ mice and plecstatin treatment of $Ple^{fl/fl}$ mice reduced HCC tumor burden upon Myc;sgTp53 treatment, implying potential of plectin targeting in HCC suppression.

**Conclusion**

Our findings show that plectin elevation in liver tumor tissue associates with HCC progression, which can be inhibited by plectin inactivation. Plectin ablation attenuates the oncogenic and invasion potentials of HCC cells, suggesting plectin´s contribution to HCC metastatic capacity. Together, our results identify plectin as a potential therapeutic target in HCC.

### EACR2024-0333

### Towards precision medicine: a preliminary collection of patient-derived organoids for the screening of personalised therapies in hepatocellular carcinoma

*E. Monti*[1,2], *I. Leoni*[1,2], *G. Galvani*[1,2], *C. Vianello*[1,2], *C. Giovannini*[1,3], *F. Vasuri*[4], *F. Piscaglia*[3,5], *C. Stefanelli*[2], *L. Gramantieri*[5], *F. Fornari*[1,2]

[1]*University of Bologna, Centre for Applied Biomedical Research CRBA, Bologna, Italy*

[2]*University of Bologna, Department for Life Quality Studies, Rimini, Italy*

[3]*University of Bologna, Department of Medical and Surgical Sciences, Bologna, Italy*

[4]*IRCCS Azienda Ospedaliero-Universitaria Di Bologna, Department of Pathology, Bologna, Italy*

[5]*IRCCS Azienda Ospedaliero-Universitaria Di Bologna, Division of Internal Medicine- Hepatobiliary and Immunoallergic Diseases, Bologna, Italy*

**Introduction**

Hepatocellular carcinoma (HCC) represents 90% of primary liver tumors and the third most lethal cancer. Due to multiple risk factors, aetiologies and genetic alterations, HCC is heterogeneous and characterized by limited response to therapies and acquired resistance. A

© 2024The Authors. Molecular Oncology is published by John Wiley & Sons Ltd on behalf of Federation of European Biochemical Societies major obstacle to clinical translation of preclinical findings has been the lack of appropriate in vitro and in vivo models. Patient-derived organoids (PDOs) may overcome this limitation, as they recapitulate the molecular and cellular features of the original tissue. Our aim is the generation of tumoral and non-tumoral PDOs from HCC patients to be used as reliable models for the screening of personalised therapies.

**Material and Methods**

PDOs were generated from HCCs and adjacent non-malignant tissues of patients undergoing liver resection at IRCCS Policlinico Sant'Orsola, Bologna. Tissues were processed by mechanical and enzymatic dissociation, seeded in Matrigel, and cultured with specific media provided by HepatiCult Organoid Kit (StemCell). Total RNA from organoids was extracted and analysed by RT-qPCR. DNA was extracted and sequenced by NGS. Drug treatment with sorafenib or lenvatinib was monitored with the live imaging system Incucyte.

**Results and Discussions**

We established several PDOs from HCC tumors with different aetiologies and surrounding liver (SL) tissue. Success rate was 70% for SL (n=8) and 60% for HCC (n=13). The morphology of SL PDOs was cystic, while that of HCC PDOs differed among patients, showing both dense and cystic phenotypes. RT-qPCR analysis revealed similar expression of stemness markers (AXIN2, SOX9) and increased expression of HCC-specific markers (AFP, GPC3, CK19) in HCC organoids compared to non-tumoral ones. A mutational analysis assessed the presence of common mutations in tissue and organoid specimens. We induced differentiation in both SL and HCC PDOs, as confirmed by lower stemness markers (LGR5, SOX9) and higher hepatocyte markers (ALB, CYP3A4) expression. A pilot drug-sensitivity test on PDO HCC26 showed strong response to sorafenib but no response to lenvatinib.

**Conclusion**

We established a preliminary collection of HCC PDOs that retain the expression of distinctive tumor markers. We observed a different sensitivity of tumoral organoids to Tyrosine Kinase Inhibitors, supporting the use of PDOs as reliable personalised models for drug screenings. Further characterization and expansion of the collection will be fundamental to obtain a comprehensive preclinical platform for the study of tailored treatments in HCC.

### EACR2024-0338
### Chalcones Illuminate the Path: From Cytotoxicity to Molecular Mechanisms in High-Grade Serous Ovarian Cancer

*İ.S. Canıtez[1], M.S. Christodoulou[2], İ. Durmaz Şahin[3]*
*[1]Koc University, Research Center for Translational Medicine KUTTAM, Istanbul, Turkiye*
*[2]University of Milan, Department of Food- Environmental and Nutritional Sciences DeFENS, Milan, Italy*
*[3]Koc University, School of Medicine, Istanbul, Turkiye*

**Introduction**

Advanced high-grade serous ovarian cancer (HGSOC) with distance metastasis does not respond to standard therapy. Patients eventually progress to an aggressive, chemoresistant stage, where conventional chemotherapeutics such as carboplatin and paclitaxel are no longer effective. Hence, novel small molecules targeting cancer-promoting mechanisms in HGSOC are promising. Chalcones demonstrate anti-cancer activities through mechanisms including apoptosis and cell cycle regulation. Our research focuses on investigating the anti-cancer potential of novel chalcone molecules against HGSOC, which may lead to innovative therapeutic strategies in HGSOC treatment.

**Material and Methods**

We synthesized 10 novel chalcone molecules and tested their efficacy on HGSOC and non-tumorigenic cell lines. Initial in vitro viability screening via SRB assay identified promising chalcones against HGSOC for further analysis, which were also evaluated against patient-derived and in vitro chemoresistant models of HGSOC cells. To investigate their action mechanisms, clonogenic assay, apoptosis assays, propidium iodide staining, immunofluorescence, wound healing assay, and western blotting were performed.

**Results and Discussions**

Among 10 molecules, MC013, MC030, and MC060 exhibited strong anti-proliferative activity against HGSOC cell lines and lower cytotoxicity to normal cells. Further assessment against an enlarged panel of HGSOC cells, including patient-derived and chemo-resistant cells, confirmed their high potential as anticancer therapeutics against HGSOC with diverse characteristics. Chalcone treatment reduced colony formation and induced significant changes in the cell cycle profiles of HGSOC cells, leading to G0/G1 arrest and subG1 increase. These molecules induced p53-regulated apoptotic cell death by activating caspase-3/7 and upregulated γH2AX levels, suggesting DNA damage induction. They also deregulated several apoptosis- and cell cycle-related proteins. Combining MC013 with carboplatin showed additive effects in an HGSOC cell line, OVCAR-3. Notably, molecules led to decreased wound closure patterns and Slug levels and increased E-cadherin levels in HGSOC cells, suggesting their role in EMT and inhibiting migratory and invasive properties of HGSOC cells.

**Conclusion**

Our findings indicate not only the potent anti-cancer role of promising chalcones against HGSOC but also reveal cellular mechanisms involved. By identifying their action mechanisms, we aim to establish novel therapeutic targets for HGSOC and enhance its standard therapy.

### EACR2024-0363
### Combined treatment with the AR inhibitor darolutamide and the pan-PI3K inhibitor copanlisib leads to superior efficacy in prostate tumor models

*E. Nevedomskaya[1], T. Sugawara[2], S. Grünewald[2], S. Heller[3], A. Böhme[3], O. von Ahsen[3], H. Nguyen[4], E. Corey[4], O. Politz[1], B. Haendler[1]*
*[1]Bayer AG, R&D Oncology, Berlin, Germany*
*[2]Nuvisan ICB GmbH, Therapeutic Oncology Research, Berlin, Germany*
*[3]Nuvisan ICB GmbH, Translational Research, Berlin, Germany*

© 2024 The Authors. Molecular Oncology is published by John Wiley & Sons Ltd on behalf of Federation of European Biochemical Societies

[4]*University of Washington, Department of Urology, Seattle, United States*

**Introduction**

Androgen receptor (AR) signaling plays a key role in prostate cancer and inhibitors targeting this pathway are approved. Unfortunately, therapy resistance often occurs, so that novel treatment strategies are urgently needed. The PI3K/AKT/mTOR pathway is also involved in prostate cancer and we evaluated the efficacy of a combined treatment with the AR inhibitor darolutamide and the pan-PI3K inhibitor copanlisib in prostate cancer models to determine whether simultaneous blockade represents a promising approach.

**Material and Methods**

The in vitro impact of darolutamide and copanlisib treatment on prostate cancer cells was compared by determining viability, cell cycle distribution, and global gene expression analysis. In vivo studies were performed in the patient-derived (PDX) LUCaP 35 prostate cancer model. Vehicle and castration arms were compared to darolutamide, copanlisib, and darolutamide plus copanlisib arms. Transcriptomic analysis was performed after 28 days of treatment.

**Results and Discussions**

Androgen-sensitive prostate cancer cell lines responded best to copanlisib treatment. Additional combination with darolutamide led to superior efficacy, as monitored in cell viability, apoptosis, and fluorescence-activated cell sorting assays. Transcriptomic analysis revealed that the dual treatment reversed the androgen response and mTOR transcriptional signatures. Anti-tumor efficacy was observed for the LUCaP 35 model in vivo upon darolutamide treatment, and this was significantly enhanced upon additional copanlisib treatment. Comparative transcriptomic analysis showed the androgen response hallmark signature to be strongly down-regulated in the darolutamide-treated group. In the combination treatment, genes involved in the MTORC1 pathway and in fatty acid metabolism were down-regulated. Immunohistochemistry revealed that staining for pro-apoptotic players was significantly higher and for proliferation markers was reduced, following combined treatment, in comparison to individual darolutamide or copanlisib treatment.

**Conclusion**

Superior efficacy was observed in prostate cancer models following combined treatment with darolutamide and copanlisib, and this was linked to a marked expression decrease of genes involved in androgen response, MTORC1 signaling, and cholesterol metabolism. In addition a significant increase of the levels of pro-apoptotic proteins was observed. These results demonstrate the superior efficacy of combining an AR inhibitor with a PI3K inhibitor, and support further evaluation in the clinic.

### EACR2024-0370
### Integrative multi-omics combined with functional pharmacological profiling in patient-derived preclinical models identifies personalized therapeutic vulnerabilities of high grade glioma subtypes

*L. Ermini[1], A. Lipsa[1], A.C. Hau[2], E. Klein[3], S. Fritah[1], A. Oudin[1], M. Mittelbronn[1], P. Nazarov[1], S. Niclou[3], A. Golebiewska[1]*
[1]*Luxembourg Institute of Health, Department of Cancer Research, Luxembourg, Luxembourg*
[2]*Goethe University, University Hospital Frankfurt, Frankfurt, Germany*
[3]*University of Luxembourg, Department of Life Sciences and Medicine, Luxembourg, Luxembourg*

**Introduction**

High grade gliomas are currently incurable displaying very short patient survival rates. High throughput omics profiling has considerably improved classification of gliomas and biomarker discovery for molecular diagnostics. Integration of high resolution multi-omics with high throughput functional profiling can further refine patient stratification towards effective personalized medicine approaches and improved treatment outcomes.

**Material and Methods**

We investigated a cohort of >45 well-established patient-derived tumor organoid and orthotopic xenograft models derived from high grade gliomas. We conducted omics profiling, including transcriptomics (bulk RNA-seq), epigenomics (DNA methylation arrays) and genomics (targeted DNA-seq). 27 organoid models were functionally screened ex-vivo with personalized 203 compound libraries targeting cancer pathways and epigenetic modifiers. Additional functional profiling included 16 organoid models and 1280 diversified FDA-approved drugs. To identify patient-specific vulnerabilities, we performed detailed statistical assessment and unsupervised multi-omics factor analysis on molecular and functional profiles.

**Results and Discussions**

Multi-omics analysis revealed a presence of diverse molecular profiles, representing various genetic, epigenetic and transcriptomic subtypes of high-grade gliomas observed in patients. Integrative data analysis using multi-omics and functional characterization identified a presence of three distinct groups with varying responses to drug treatments. In particular, we observed varying efficacy of drugs targeting histone methyl-transferases and Pim-kinases, which were linked to specific biomarkers at (epi)genetic and transcriptomic levels. Additional validation includes assessment of drug efficacy in time at different concentration ranges and biomarker validation in patient tumor tissue among different patient groups based on their individual omics profiles.

**Conclusion**

Our findings suggest that the integration of omics and functional readouts may improve defining precision medicine treatment strategies. We provide a rationale for identifying distinct patient subgroups in the preclinical phase allowing for patient-tailored therapeutic strategies in high grade gliomas.

### EACR2024-0390
### The PARP1 selective inhibitor saruparib elicits potent and durable antitumor activity in patient-derived BRCA1, BRCA2

© 2024 The Authors. Molecular Oncology is published by John Wiley & Sons Ltd on behalf of Federation of European Biochemical Societies

### and PALB2-associated cancer models

A. Herencia Ropero[1], A. Llop-Guevara[1], A.D. Staniszewska[2], J. Domènech-Vivó[3], H. Domènech[4], A. Lau[2], J.V. Forment[2], M. Albertella[2], J. Balmaña[5], V. Serra[4]
[1]Vall d'Hebron Institute of Oncology, Experimental Therapeutics Group, Barcelona, Spain
[2]AstraZeneca, Oncology R&D, Cambridge, UK
[3]Vall d'Hebron Institute of Oncology, Hereditary Cancer Genetics Group, Barcelona, Spain
[4]Vall d'Hebron Institute of Oncology, Experimental Therapeutics Group, Barcelona, Spain
[5]Vall d'Hebron Institute of Oncology, Hereditary Cancer Genetics Group, Barcelona, Spain

**Introduction**

PARP1/2 inhibitors (PARPi) are targeted therapies approved for homologous recombination repair (HRR)-deficient breast, ovarian, pancreatic and prostate cancers. Since inhibition of PARP1 is sufficient to cause synthetic lethality in tumors with homologous recombination deficiency (HRD), PARP1 selective inhibitors such as saruparib are being developed. It is expected that selective PARP1 inhibition leads to a safer profile that facilitates its combination with other DNA damage repair inhibitors. Here, we aimed to characterize the antitumor activity of saruparib in preclinical models compared to the first generation PARP1/2 inhibitor olaparib and to identify mechanisms of resistance.

**Material and Methods**

Thirteen previously characterized patient-derived tumor xenograft (PDX) models from breast, ovarian and pancreatic cancer patients harboring germline pathogenic alterations in *BRCA1*, *BRCA2* or *PALB2* were used to evaluate the efficacy of saruparib alone or in combination with carboplatin or an ATR inhibitor (ATRi) and compared it to olaparib. We performed DNA, RNA and protein-based assays.

**Results and Discussions**

Saruparib showed superior antitumor activity than olaparib in terms of preclinical complete response rate (75% *vs*. 37%). The median preclinical progression free survival was significantly longer in the saruparib-treated compared to the olaparib-treated group (>386 days *vs*. 90 days). Mechanistically, saruparib induced more replication stress and genomic instability in PARPi-sensitive tumors. All tumors at progression with either PARPi (39/39) showed restoration of HRR functionality by RAD51 foci formation, and the most prevalent mechanisms were the acquisition of reversion mutations in *BRCA1*/*BRCA2* and the accumulation of hypomorphic BRCA1. Saruparib elicited profound and durable responses when combined with carboplatin or ATRi in 3/6 and 5/5 models, respectively.

**Conclusion**

Collectively, these results show that the novel PARP1 selective inhibitor saruparib yields a potent antitumor response in PDXs with HRD and delays PARPi resistance alone or in combination with carboplatin or ATRi, which supports its use in the clinic as a new therapeutic option.

### EACR2024-0395
### Evaluating the Impact of Vascular Endothelial Growth Factor Targeted Treatment on Survival Outcomes and Adverse Events in Ovarian Cancer: An Updated Systematic Review and Meta-Analysis

M.K. Elfaituri[1], A. Khaled[1], H. Faraj[1]
[1]University of Tripoli, Faculty of Medicine, Tripoli, Libya

**Introduction**

The Vascular Endothelial Growth Factor (VEGF) pathway, critical in ovarian tumor angiogenesis and progression, has emerged as a potential therapeutic target. However, the implications of VEGF-targeted treatment remain uncertain. This study provides an updated systematic review and meta-analysis examining the impact of drugs targeting VEGF on survival outcomes and adverse event rates in ovarian cancer patients.

**Material and Methods**

We systematically searched PubMed, Embase, Cochrane Library, and Web of Science databases until December 2022 for randomized controlled trials (RCTs). We selected studies comparing overall survival (OS) and progression-free survival (PFS) as first-line treatment and in recurrent disease, and the incidence of adverse events (hemorrhagic events, thromboembolic events, hypertension) in patients treated with VEGF inhibitors or VEGF receptor inhibitors versus usual care. Statistical analyses, conducted using R software (version 4.0.3) with the 'metafor' and 'meta' packages, employed random-effect models. Survival outcomes were synthesized using hazard ratios (HR), while relative risk (RR) was used to evaluate adverse event rates.

**Results and Discussions**

Our analysis incorporated eleven RCTs involving 2,931 patients (1,487 in the VEGF/R inhibitor group and 1,444 in the control group). VEGF/R targeted treatment significantly improved PFS as a first-line treatment (HR 0.82, 95% CI 0.72-0.92, P<0.01, I2=65%) and in recurrent disease (HR 0.58, 95% CI 0.51-0.67, P<0.01, I2=60%). There was a non-significant trend towards improved OS in first-line treatment (HR 0.97, 95% CI 0.89-1.05, P=0.46, I2=0%), but in recurrent disease, the improvement in OS was statistically significant (HR 0.85, 95% CI 0.77-0.94, P<0.01, I2=0%). VEGF inhibitors increased the risk of hemorrhagic events (RR 2.31, 95% CI 1.08-4.93, P=0.03, I2=74%) and hypertension (RR 2.77, 95% CI 1.66-4.62, P<0.01, I2=0%), but not for thromboembolic events (RR 1.22, 95% CI 0.61-2.46, P=0.58, I2=70%).

**Conclusion**

VEGF inhibitors significantly enhanced progression-free survival for both first-line treatment and recurrent ovarian cancer. For overall survival, the benefit was not statistically significant in first-line treatment but was significant in recurrent disease. These results underscore the importance of individualized treatment, weighing the survival benefits against potential risks, and call for more extensive studies to further evaluate VEGF inhibitors' long-term survival advantages and adverse event profiles.

### EACR2024-0427
### DFF332, a novel potent and selective HIF2α transcription factor inhibitor for the

© 2024 The Authors. Molecular Oncology is published by John Wiley & Sons Ltd on behalf of Federation of European Biochemical Societies treatment of VHL-deficient clear cell renal cell carcinoma

C. Fritsch[1], T. Stachyra[1], M. Wartmann[1], C. Schnell[1], J. Otero[2], F. Hofmann[3], R. Fairhurst[4]
[1]Novartis Biomedical Research, Disease Area Oncology, Basel, Switzerland
[2]Novartis Biomedical Research, Translational Clinical Oncology, East Hanover, United States
[3]Pierre Fabre laboratories, Pierre Fabre Laboratories, Toulouse, France
[4]Novartis Biomedical Research, Global Discovery Chemistry, Basel, Switzerland

**Introduction**

The hypoxia-inducible factors (HIFs) HIF1α and HIF2α are transcription factors well characterized as master regulators of oxygen homeostasis that control transcriptional responses to reduced oxygen availability (hypoxia). In clear cell renal cell carcinoma (ccRCC), genetic alterations (e.g. mutation or silencing) in the Von Hippel-Lindau (VHL) gene lead to the accumulation of HIFα in the tumor independent of oxygen levels. In ccRCC, HIF2α has been described to be the key oncogenic driver while HIF1α displays a tumor suppressor role. Pre-clinical and clinical data reported for HIF2α selective inhibitors in ccRCC further support HIF2α as an attractive target for anti-cancer therapy. Here, we report the characterization of DFF332, a clinical-stage and orally bioavailable small molecule that potently and selectively inhibits HIF2α transcriptional activity in ccRCC pre-clinical models.

**Material and Methods**

The in vitro activity and selectivity profile of DFF32 have been established across a diverse panel of biochemical and cell-based assays. In vivo, DFF332 anti-tumor efficacy, tolerability and effects on tumor pharmacodynamics have been assessed in VHL-deficient ccRCC cell line derived (786-O and SKRCO-1), and patient-derived xenograft mouse-models.

**Results and Discussions**

DFF332 is a potent HIF2α inhibitor that binds selectively to the PAS-B domain of HIF2α vs HIF1α as demonstrated in biophysical and biochemical assays (e.g. $IC_{50}$ of 9 nM in scintillation proximity assay). The ability of DFF332 to potently and selectively inhibit HIF2α transcriptional activity is further confirmed in cell-based functional assays (e.g. $IC_{50}$ of 37 nM in suppressing HIF2α-dependent target gene EGLN3 expression in 786-O cells). In vivo, when administered orally once daily, DFF332 exhibited significant tumor regression in VHL-deficient ccRCC cell line derived xenograft mouse-models (786-O and SKRCO-1) at doses that are associated with HIF2α pathway inhibition and are well tolerated. DFF332 anti-tumor efficacy is further confirmed in VHL-deficient ccRCC patient-derived xenograft mouse models.

**Conclusion**

DFF332 is a novel potent, selective and orally bioavailable HIF2α inhibitor that is capable of eliciting significant antitumor activity associated with HIF2α pathway inhibition and good tolerability. DFF332 clinical profiling has been initiated in VHL-deficient ccRCC patients.

### EACR2024-0463
### Different modes of Bcl-xL inhibition, different outcomes?

J.L. Tan[1], A. Tighe[1], N. Cook[1,2], S. Taylor[1]
[1]University of Manchester, Division of Cancer Sciences, Manchester, United Kingdom
[2]The Christie NHS Foundation Trust, Experimental Cancer Medicine Team, Manchester, United Kingdom

**Introduction**

Cells become dependent on Bcl-xL for survival during prolonged mitosis. As a result, inhibition of Bcl-xL is synergistic with chemotherapy agents that delay progression through mitosis, e.g. paclitaxel. In ovarian cancer this presents a therapeutic opportunity, as agents such as paclitaxel are commonly used as systemic therapies in the management of patients. However, direct Bcl-xL inhibitors are problematic due to on-target platelet toxicity, causing thrombocytopaenia. Strategies to target Bcl-xL inhibitors to tumour cells such as PROTAC technology (Proteolysis Targeting Chimaera) are being explored. This research will compare different modes of Bcl-xL inhibition, with the goal of identifying potential biomarkers to predict patients who can benefit from treatment with a Bcl-xL inhibitor.

**Material and Methods**

We screened a panel of 22 established ovarian cancer cell lines and 83 patient-derived ovarian cancer models (OCMs) for their response to paclitaxel alone and in combination with different Bcl-xL inhibitors, including the direct inhibitors A-1155463/A-1331852 and the PROTAC DT-2216. We assessed metabolic capacity (CellTiter-Glo®) and clonogenic potential.

**Results and Discussions**

The screen revealed a subset of OCMs that were sensitised to paclitaxel with the addition of a single dose of A-1155463. Notably, another subset of OCMs were highly sensitive to A-1155463 alone, so we focussed on screening responses to Bcl-xL inhibitor monotherapy in the cell lines. The cell lines screened clustered into 4 groups: one sensitive to both A-1331852 and DT-2216, one only sensitive to DT-2216, one only sensitive to A-1331852, and one resistant to both drugs. Sensitivity to Bcl-xL inhibitors did not correlate with Bcl-xL expression but did correlate with low levels of Mcl-1, a factor commonly associated with Bcl-xL inhibitor resistance in solid tumours. Western blotting of cell lines sensitive to A-1331852 but not DT-2216 confirmed the expression of von Hippel-Lindau protein, which is required for PROTAC function.

**Conclusion**

Ovarian cancer cell lines display a spectrum of sensitivity to Bcl-xL inhibitor treatment. Our ongoing research will investigate the molecular factors behind sensitivity and resistance to these inhibitors, initially focussing on Mcl-1 inhibition in A-1331852 resistant cell lines and assessing VHL function in DT-2216 resistant cell lines, leading to the identification of potential biomarkers for validation in the clinic.

### EACR2024-0470
### Targeted therapy with a dual MEK and Aurora kinases inhibitor induces

© 2024The Authors. Molecular Oncology is published by John Wiley & Sons Ltd on behalf of Federation of European Biochemical Societies

### apoptosis and cell cycle arrest in an acute myeloid leukemia stem cell line

P. Beigi[1,2], P. Fallah[3], S. Kaviani[1]
[1]Tarbiat Modares University, Department of Haematology, Tehran, Iran
[2]Mazandaran University of Medical Sciences, Diabetes Research Center, Sari, Iran
[3]Checkup Clinical and Specialty Laboratory, Haematology, Karaj, Iran

**Introduction**

Despite the growing number of treatment options for acute myeloid leukemia (AML), the clinical outcome has remained unfavorable. Dysregulation of tyrosine kinase receptors, RAS/RAF/MEK/ERK pathway, and Aurora Kinases family (AURKs) have been frequently reported in AML, which are linked to AML development and progression. Therefore, we aimed to evaluate the effects of BI.847325, an orally bioavailable and selective dual MEKs and AURKs inhibitor, in an AML stem cell line model.

**Material and Methods**

The cytotoxic effects of BI.847325 on KG-1a cells were assessed by MTT [3-(4,5-dimethylthiazolyl-2)-2,5-diphenyltetrazolium bromide] assay in 24, 48, and 72 hours post-treatment. KG-1a cells were treated with IC50 value of BI.847325. Then, the BI.847325 ability to induce apoptosis/necrosis was evaluated by Annexin V/PI flow cytometry as well as measuring mRNA expression of *MCL1* and *BIM* by qRT-PCR. Moreover, alterations in cell cycle distribution after BI.847325 treatment were examined by DNA content analysis using flow cytometry, along with determining *AURK-A*, *AURK-B*, *c.Myc*, and *P21* expression levels.

**Results and Discussions**

Both RAS/RAF/MEK/ERK pathway and AURKs play crucial roles in cell survival and cell cycle. In our study, BI.847325 induced significant cytotoxic effects on KG-1a cells in a time and dose-dependent manner. Flow cytometry assay revealed a significant increase in apoptosis after BI.847325 treatment. Furthermore, DNA content analysis demonstrated a significant accumulation of the cells in G2/M phase of mitosis, accompanied by a significant decrease in the G0/G1 and S phases, and a significant increase in the SubG1 phase. BI.847325, a dual MEK/AURKs inhibitor, significantly decreased the expression levels of *AURK-A*, *AURK-B*, and *c.Myc* which are involved in mitosis progression, and *MCL.1* as an anti-apoptotic gene, possibly because these targets are regulated by MAPK pathway. However, *BIM* upregulation, a pro-apoptotic element, was not significant. The expression level of *P21,* a cell cycle inhibitor, was significantly upregulated after BI.847325 treatment as well.

**Conclusion**

The targeted therapy using BI.847325 induced significant apoptosis and cell cycle arrest in the AML stem cell line at both molecular and cellular levels. Our findings suggest that multi-targeting MEK and Aurora kinases by BI.847325 might be a promising option to overcome AML. However, further complementary assays and in vivo models are needed to shed light on the detailed regulatory mechanisms of this compound.

### EACR2024-0471
### Glucose transporter 1 inhibitors induce autophagy and impede growth and invasion in thyroid cancer cells

C.Y. Kuo[1], S.P. Cheng[1], Y.S. Li[2], Y.C. Hsu[3]
[1]MacKay Memorial Hospital, Department of Surgery, Taipei, Taiwan
[2]MacKay Memorial Hospital, Department of Medical Research, Taipei, Taiwan
[3]National Central University, Department of Biomedical Sciences and Engineering, Taoyuan, Taiwan

**Introduction**

Differentiated thyroid cancer generally exhibits indolent growth and low expression of glucose transporter 1 (GLUT1). As part of tumor progression, upregulation of GLUT1 and other glycolytic enzymes contributes to increased uptake of glucose and clinical utility of fluorodeoxyglucose-positron emission tomographic imaging. Several GLUT1 inhibitors have been developed and evaluated in preclinical studies. We aimed to evaluate the effects of GLUT1 inhibitors on the cell biology of thyroid cancer cells.

**Material and Methods**

Two papillary thyroid cancer cell lines, TPC-1 (with RET/PTC1 rearrangement) and B-CPAP (harboring BRAF V600E mutation), were treated with two different GLUT1 inhibitors, STF-31 and BAY-876. Cell Counting Kit-8, clonogenicity, transwell, and 3D spheroid invasion assays were conducted.

**Results and Discussions**

GLUT1 inhibitors impeded the growth of thyroid cancer cells in a dose-dependent manner. Activation of the AMPK pathway was accompanied by growth arrest in the absence of apoptosis. GLUT1 inhibitors provoked autophagy, and autophagy inhibition further reduced cell viability. Co-treatment with lenvatinib had synergistic effects as determined by Chou-Talalay combination indexes. Cell invasive capacity was significantly suppressed by GLUT1 inhibitors.

**Conclusion**

GLUT1 inhibitors oppose tumor progression and invasion and may represent a promising target for cancer combination therapy.

### EACR2024-0475
### Co-targeting of c-Met and DNA damage response elicits synergistic effects in pancreatic ductal adenocarcinoma

O. Firuzi[1], F. Moosavi[1], A. Jafari[1], M. Tavakkoli[1], M. Fazeli[2]
[1]Shiraz University of Medical Sciences, Medicinal and Natural Products Chemistry Research Center, Shiraz, Iran
[2]Shiraz University, Department of Basic Sciences, Shiraz, Iran

**Introduction**

Pancreatic ductal adenocarcinoma (PDAC) is still one of the most aggressive and lethal malignancies across the world. Aberrant activation of c-Met, an oncogenic receptor tyrosine kinase, has been observed in many tumors including PDAC. Poly (ADP-ribose) polymerases (PARPs) are a family of proteins involved in DNA

© 2024 The Authors. Molecular Oncology is published by John Wiley & Sons Ltd on behalf of Federation of European Biochemical Societies damage response (DDR) and recently a PARP inhibitor, olaparib, has been approved for maintenance treatment of BRCA-associated PDAC patients. We aimed to study the effect of co-targeting of c-Met kinase and PARP in PDAC cells, in hopes of inducing BRCA-ness in PDAC cells and finding synthetic lethal combinations.

**Material and Methods**

Cabozantinib, crizotinib and PHA665752 were tested as c-Met inhibitors in combination with olaparib and also doxorubicin, a cytotoxic agent that causes severe DNA damage. MTT assay was conducted to examine the antiproliferative effects of drug combinations against AsPC-1 and Suit-2 PDAC cells in monolayer cell cultures. Combination index (CI) was mathematically calculated by Calcusyn software. Colony formation assay was also performed to study the effect of combinations on cancer cell growth. Three-dimensional spheroid cell cultures of PDAC cells were generated based on the liquid overlay technique in 96-well plates. The growth inhibitory effect of drug combinations in 3D cultures was examined by acid phosphatase (APH) assay.

**Results and Discussions**

CI values calculated from combination experiments performed in monolayer cultures showed that all c-Met inhibitors when combined with olaparib resulted in synergistic effects with CI values as low as 0.60. Combined treatment of c-Met inhibitors with olaparib also decreased colony formation and led to significant reduction of cell viability in three-dimensional spheroid cultures compared to single treatments. Similar results were obtained when c-Met inhibitors were combined with doxorubicin in monolayer and 3D cultures.

**Conclusion**

The findings of this study show that combination of c-Met inhibitors with olaparib as well as doxorubicin could constitute a reasonable strategy for development of more efficacious therapeutic options in PDAC.

### EACR2024-0503

### Efficacy of combined Proteasome and HDAC inhibitors treatment in glioblastoma cells

*L. Costa Furtado*[1,2], *J.C. Lipreri da Silva*[1], *V. Feijoli Santiago*[3,4], *J.A. Machado-Neto*[1], *G. Palmisano*[3], *R. Parise-Filho*[5], *W. Fenical*[6], *F.A.E. Kruyt*[2], *L. Costa-Lotufo*[1]

[1]*University of São Paulo, Department of Pharmacology- Institute of Biomedical Sciences, São Paulo, Brazil*

[2]*University of Groningen, Department of Medical Oncology- University Medical Center Groningen, Groningen, The Netherlands*

[3]*University Of São Paulo, Department of Parasitology- Institute of Biomedical Sciences, São Paulo, Brazil*

[4]*University of Southampton, School of Human Development and Health- Faculty of Medicine, Southampton, United Kingdom*

[5]*University of São Paulo, School of Pharmaceutical Sciences, São Paulo, Brazil*

[6]*University of California San Diego, Scripps Institution of Oceanography, San Diego, United States*

**Introduction**

Glioblastoma is the most aggressive adult brain tumour with a high mortality rate and poor survival. New therapies are needed to increase patient survival (approximately 2 years) and the molecular vulnerabilities in glioblastoma can be the key to improving the prognosis of patients. Here, we set out to investigate the combined effects of the proteasome inhibitor marizomib and the histone deacetylase inhibitor vorinostat (Zolinza®) in glioblastoma (stem, GSCs) cells and underlying mechanisms of cytotoxicity.

**Material and Methods**

The antiproliferative potential of single and combined treatments of marizomib and vorinostat was evaluated in T98G, U87, U251, GG16 and GSC23 by Sulforhodamine B and MTS assays, and Compusyn and Combenefit analyses. Protein markers of proteasome and HDAC inhibition, and differential expression of proteins related to the unfolded protein response (UPR) were studied through immunoblotting. Quantitative mass spectrometry-based proteomic data were analyzed using MaxQuant and Perseus computational platforms. Bioinformatic analyses followed by Database for Annotation, Visualization, and Integrated Discovery (DAVID) and Search Tool for the Retrieval of Interacting Genes/Proteins (STRING) databases.

**Results and Discussions**

Doses of 16 and 80 nM of marizomib, and 0.4 and 2 µM of vorinostat inhibited 50% of the proliferation of glioblastoma cells. Synergistic effects of the combination were observed in T98G, U87, U251 and GG16 cells, while in GCS23 there antagonistic activity was seen. Levels of HSP70, acetylated α-tubulin and histone H3 were increased by marizomib and vorinostat treatment in T98G cells. Proteomic data analysis identified 68 differentially expressed proteins upon treatment, including proteins related to the UPR, regulation of transcription from RNA polymerase in response to stress, protein folding and regulation of proteasome subunits. Preliminary studies show combination treatment-induced modulation of XBP1s, CHOP, and p-eIF2α, indicative of UPR activation in T98G. Cleaved PARP, caspase 3 and p-histone H2Ax were detected, indicative of apoptosis activation and DNA damage as likely mediators of cytotoxicity.

**Conclusion**

Marizomib and vorinostat have a strong synergistic activity in T98G cells and GSCs and evidence suggests the involvement of the UPR as a mediator of cell death. More detailed studies are in progress to further study the potency and underlying mechanisms of action of this combined treatment.

### EACR2024-0508

### Development of an ex vivo image-based 3D platform for precision therapy in pancreatic ductal adenocarcinoma

*F. Sangkolah*[1], *D. Visan*[1], *T. Richardson*[1], *K. Nan*[1], *D. Wiskerke*[1], *E. Peerani*[1], *F. Iori*[1], *G. de Fraine*[1], *A. Kaffa*[1], *D. Ranatunga*[1]

[1]*Ourotech Ltd, Pear Bio, London, United Kingdom*

**Introduction**

Pancreatic ductal adenocarcinoma is a highly aggressive malignancy, with a 5-year survival rate of less than 5%. Emerging therapies including immunotherapies offer promising avenue, but the lack of reliable biomarkers

© 2024 The Authors. Molecular Oncology is published by John Wiley & Sons Ltd on behalf of Federation of European Biochemical Societies remains a challenge for personalised treatment selections. We developed a microphysiological platform derived from patients' tumour aimed to assess patients' response to diverse therapeutic strategies including standard-of-care chemotherapeutic regimens and more novel options such as immunotherapies.

**Material and Methods**

Our platform utilises *ex vivo* culture of patients' tumour extracted from surgical resection of the pancreas. Mixed populations of single cells were cultured in biomimetic hydrogel alongside immune cells from matched patients' peripheral blood mononuclear cells (PBMC) to mimic the tumour-immune microenvironment. These cultures were tested with approved therapies FOLFIRINOX and gemcitabine/nab-paclitaxel, and experimental approaches such as pembrolizumab (as monotherapy or in combination). We monitored functional metrics including cell viability and immune cell infiltration through daily confocal imaging up to 5 days to assess cytotoxicity of each drug in the 3D cultures. Other readouts include metabolic-based assays and end-point cell viability imaging.

**Results and Discussions**

We observed cytotoxicity to standard therapies FOLFIRINOX and gemcitabine/nab-paclitaxel in patient-derived cultures. These cultures also showed distinct response pattern to the tested therapies. Notably, the platform highlighted promising response to pembrolizumab especially in combination with other therapies, including approved chemotherapies (5-FU and Irinotecan) and more experimental small molecular inhibitors.

**Conclusion**

We are able to evaluate different therapeutic strategies for PDAC in our platform by monitoring functional response metrics to established regimens and emerging therapies. By capturing the inter-tumour heterogeneity, the platform aims to facilitate effective personalised treatment for patients. Further research will include refinement of the platform and molecular readouts to confirm mechanism of action of targeted therapies, as well as the capacity to test additional drug candidates.

### EACR2024-0509

### Vicinal Diaryl-Substituted Compounds: Promising Therapeutics for Breast Cancer

*E. Nalbat[1], S. Guntekin-Ergun[2], S. Turanli[3], D. Lengerli[3], B. Caliskan[3], E. Banoglu[3], R. Cetin-Atalay[4]*

*[1]Middle East Technical University METU, Graduate School of Informatics- Department of Health Informatics, Cancer System Biology Laboratory CanSyL, Ankara, Turkiye*

*[2]Hacettepe University- Faculty of Medicine, Department of Medical Biology, Ankara, Turkiye*

*[3]Gazi University, Faculty of Pharmacy- Department of Pharmaceutical Chemistry, Ankara, Turkiye*

*[4]University of Chicago, University of Medicine- Section of Pulmonary and Critical Care Medicine, Chicago, USA*

**Introduction**

Breast cancer stands as the primary diagnosed cancer in women and the most prevalent overall, characterized by the formation of malignant tumors in breast cells. Its global burden is anticipated to rise in the coming years, driven by factors like late menopause, hormonal stimulation, advancing age, obesity, as well lifestyles. Resistance to traditional chemotherapy ensues from gene mutations and dysregulation of pivotal signaling pathways in breast cancer. Therefore, there is a pressing need to devise innovative therapeutics targeting breast cancer. This study delves into unraveling the molecular mechanisms underlying the cytotoxicity of recently discovered vicinal diaryl-substituted isoxazole and pyrazole compounds against breast cancer.

**Material and Methods**

Following the assessment of compound cytotoxicity using Sulforhodamine B assay and real-time cell electronic sensing analysis, we conducted analyses on cell cycle progression, cell death mechanisms, and DNA damage employing flow cytometry. Transcriptomic changes were examined using a PanCancer panel comprising 770 genes associated with cancer. Senescence-associated β-galactosidase activity and oxidative stress levels were quantified through immunostaining techniques. A wound healing assay was performed. Additionally, western blot analyses were employed to elucidate cell cycle phases, senescence pathways, and oxidative stress responses. The anticancer efficacy of the compounds was further evaluated in vivo using a tumor xenograft assay.

**Results and Discussions**

Oout of more than 60 compounds tested, compounds 11 and 85 exhibited pronounced cytotoxicity against breast cancer cell lines at low doses, while there were no cytotoxic effects on normal-like cells. Analysis of the cancer panel revealed significant alterations in the cell cycle, transcriptional misregulation, DNA damage-repair mechanism, and associated genes, with notable changes also observed in the levels of several proteins following treatment. Treatment with these compounds resulted in time- and dose-dependent growth inhibition, attributed to oxidative stress-induced DNA damage leading to cellular senescence, G0/G1 phase arrest, and apoptosis. Migration of breast cancer cells was also inhibited after compound treatment. Moreover, administration of compounds 11 and 85 substantially reduced tumor size in nude mice.

**Conclusion**

The observed anti-tumor effects of compounds 11 and 85 validate their potential as promising anti-cancer agents for the treatment of breast cancer.

### EACR2024-0513

### New Dithiocarbamate-Copper Complexes as Inhibitors of p97/NPL4 in Cancer Cells

*M. Loffelmann[1], Z. Skrott[1], D. Majera[1], P. Starha[2], V. Krystof[3], M. Mistrik[1]*

*[1]Institute of Molecular and Translational Medicine, Faculty of Medicine and Dentistry- Palacky University Olomouc, Olomouc, Czech Republic*

*[2]Department of Inorganic Chemistry, Faculty of Science- Palacky University Olomouc, Olomouc, Czech Republic*

*[3]Department of Experimental Biology, Faculty of Science- Palacky University Olomouc, Olomouc, Czech Republic*

**Introduction**

Cancer cells are highly dependent on functional proteostatic systems that are responsible for the

© 2024 The Authors. Molecular Oncology is published by John Wiley & Sons Ltd on behalf of Federation of European Biochemical Societies degradation of mutated or incorrectly folded proteins and that also maintain the balance with proteosynthesis. The p97 pathway is one of these systems, which is mainly involved in protein degradation. Protein p97 binds with several binding partners and cofactors, where Nuclear protein localization homolog 4 (NPL4) is one of these cofactors that is responsible for binding to substrates meant for degradation via the proteasome. Therefore, inhibition of NPL4 could impair protein degradation and cause severe damage to cancer cells. We looked for potential NPL4 inhibitors in the group of dithio-carbamates (DTCs). DTCs are small organic compounds that possess metal chelation activity and can form complexes with metal ions, especially with copper. One of these DTCs-copper complexes - bis(diethyldithio-carbamate)-copper (CuET) – was recently identified as an NPL4 protein inhibitor causing its aggregation.

**Material and Methods**

We tested 20 DTCs-copper complexes on two cancer cell lines – U-2-OS and H1299. The screening was performed using a developed flow cytometry-based assay to determine the ability of tested compounds to immobilize NPL4 after pre-extraction by measuring the NPL4-GFP signal in a reporter U2OS-NPL4-GFP cell line. CuET was used as a positive control. For other evaluations, we used confocal microscopy, immunoblot, and XTT cytotoxicity assay.

**Results and Discussions**

Out of 20 prepared DTCs-copper complexes for their ability to immobilize NPL4, 13 scored positively. The phenotypes previously reported for CuET were also observed for the tested complexes: heat shock response, unfolded protein response, accumulation of poly-ubiquitinated proteins, and immobilization of p97/NPL4 proteins. It turned out that the proteotoxic phenotypes were not only associated with CuET but shared with other DTCs-copper complexes. Interestingly, the reported anticancer activity was observed in structurally different compounds. A cytotoxic screening confirmed the toxicity of positive compounds to cancer cells, and we found a positive correlation between cytotoxicity and NPL4 aggregation, showing it as a possible target in antitumour therapy.

**Conclusion**

These findings propose DTCs-copper complexes, inducing proteotoxic stress by disrupting the p97/NPL4 pathway in cancer cells, for further studies with possible anticancer drug development.

### EACR2024-0534
### Novel Substituted 6-Phenyl Purine Analogues Inducing Autophagy Mediated Apoptosis on High Grade Serous Ovarian Cancer Cell Lines

*D. Demirel*[1], *P. Kul*[2], *D. Altıparmak*[2], *M. Tuncbilek*[2], *İ. Durmaz Şahin*[3]
[1]*Koc University, Graduate School of Health Sciences – Cellular and Molecular Medicine, İstanbul, Turkiye*
[2]*Ankara University, Department of Pharmaceutical Chemistry - Faculty of Pharmacy, Ankara, Turkiye*
[3]*Koc University, School of Medicine, İstanbul, Turkiye*

**Introduction**

Ovarian cancer (OC) ranks as the fifth leading cause of cancer-related deaths among women. High-grade serous ovarian cancer (HGSOC) constitutes the predominant histological subtype and is notorious for its poor survival rates and limited treatment responses. While approximately 85% of HGSOC patients achieve clinical remission with a combination of surgery and platinum-based chemotherapy, a subset (15-20%) remains unresponsive to this approach, with 25% developing resistance within six months. Despite the emergence of combinational PARP inhibitor therapy over the past decade, resistance to these inhibitors poses a significant challenge. Given the need for novel anti-cancer agents in HGSOC treatment, purine analogues emerge as promising candidates due to their modifiable structures and potential for diverse mechanisms of action. While clinically approved purine analogues like cladribine, pentostatin, and fludarabine exist, their selectivity and application in OC warrant further exploration.

**Material and Methods**

In this study, cytotoxicity screening was conducted using the NCI-SRB assay on 18 novel purine/pyrimidine nucleobase analogues across HGSOC cell lines (OVCAR-3, OVSAHO, and Kuromachi) and a non-cancerous cell line (HGRC1). The results identified molecules *31* and *32* as the most active compounds, with subsequent analyses revealing their effects on cell cycle progression (PI staining), apoptosis (Annexin V and Caspase3/7 staining), and cell signaling (Western Blot). Autophagy inhibitors and assays were also employed to elucidate the mechanisms of action.

**Results and Discussions**

The results indicated an increase in SubG1 staining, suggestive of controlled cell death, alongside co-activation of apoptotic and autophagic markers upon treatment with molecules *31-32*. Inclusion of autophagy inhibitors in treatment decreased apoptotic markers as well, yielding apoptosis activation dependent on autophagy signaling. Moreover, a decrease in total PARP expression was observed, indicating a PARP inhibitor-like effect. Notably, these analogues demonstrated lower cytotoxicity towards non-cancerous cells, implying potential selectivity for cancer cells.

**Conclusion**

In conclusion, the newly synthesized purine analogues *31-32* exhibit intriguing potential as anti-cancer agents, activating apoptosis and autophagy, suggesting a dual therapeutic approach. Further investigations, including ex vivo, in vivo, and in silico techniques, are warranted to validate and expand upon these findings.

### EACR2024-0574
### Advancing RNA Therapeutics for Pancreatic Cancer: Harnessing Star Co-polymers for Safe and Efficient Delivery

*S. Miron Barroso*[1], *J. Correia*[2], *J. Clark*[1], *M. Lythgoe*[1], *A. Porter*[2], *T. K. Georgiou*[2], *J. Krell*[1]
[1]*Imperial College London, Surgery and Cancer, London, United Kingdom*
[2]*Imperial College London, Department of Materials, London, United Kingdom*

**Introduction**

© 2024The Authors. Molecular Oncology is published by John Wiley & Sons Ltd on behalf of Federation of European Biochemical Societies

Pancreatic cancer has the lowest survival rate of any cancer type and its poor response to current therapeutics options highlights the urgent unmet medical need for the development of new therapeutic strategies. Even though RNA therapeutics have become increasingly attractive for cancer treatment, safe and effective delivery vectors are still needed. Polymeric carriers have been widely studied due to their versatility, multi-functionality and relative low cost. Poly[2-(dimethylamino)ethyl methacrylate] (PDMAEMA) contains tertiary amines that interact with RNA, allowing endosomal escape and cellular internalisation. By introducing PEG-based monomers like oligo(ethylene glycol) methyl ether methacrylate (OEGMA), studies have reported decreased toxicity and prolonged circulation time.

**Material and Methods**

DMAEMA-OEGMA star co-polymers of different architectures (position of the cationic groups) were synthesised by group transfer polymerisation. Their physicochemical properties were characterised by assessing size, zeta potential, cloud points, pkas, composition, dispersity and molecular mass. Optimal ratios of polymer to RNA were determined by agarose gel retardation assay and polyplex stability determined by heparin displacement assay. Safety was evaluated in 2D and 3D models of pancreatic cancer. Off target effects of the polymers on the immune system were evaluated by macrophage pro-inflammatory cytokine release quantification using qPCR. Transfection efficiency was assessed using flow cytometry and fluorescence microscopy in comparison with benchmark polymers.

**Results and Discussions**

The synthesised star co-polymers had molar masses of ~100,000 g/mol and hydrodynamic diameters between 14-22 nm. The core diameters determined by electron microscopy were around 20 nm and zeta potentials were neutral for all polymers. Each star polymer could fully complex RNA at an NP ratio of 5 or lower. In terms of cell viability in pancreatic cancer spheroids, the star polymers had a higher IC50 than polyethylenimine, a standard for gene delivery. The star polymers didn't show cytotoxicity at the concentrations used for transfection. Most of the star co-polymers appeared to have significantly higher transfection efficiency than polyethylenimine, with efficiencies reaching up to 85%.

**Conclusion**

Our findings suggest that DMAEMA-OEGMA star-shaped co-polymers have a favourable safety and efficiency profile. Overall, our study highlights the therapeutic potential of star co-polymers as delivery systems for RNA therapeutics.

### EACR2024-0589

### Oleuropein and Hydroxytyrosol-loaded mesoporous silica nanoparticles eased Glioblastoma cell aggressive features and increased the temozolomide effect

*M. Ercelik[1], C. Tekin[1], A. Pamukcu[2], M. Gurbuz[1], D.S. Karaman[3], G. Tezcan[4], B. Tunca[1]*
*[1]Bursa Uludag University, Medical Biology, Bursa, Turkiye*
*[2]Izmir Katip Çelebi University, Biomedical Technologies, Izmir, Turkiye*
*[3]Izmir Katip Çelebi University, Biomedical Engineering, Izmir, Turkiye*
*[4]Bursa Uludag Univers, Fundamental Sciences, Bursa, Turkiye*

**Introduction**

Glioblastoma (GB) patients receive chemo-radiotherapy with Temozolomide (TMZ) after surgical resection. However, since this treatment is unsuccessful in most cases, there is a need for promising ingredients and the use of innovative technologies in the treatment of GB. Oleuropein (OL) and Hydroxytyrosol (HT) are flavonoids known for their anti-GB cell effects. This study aimed to design a nano-drug formula with OL or HT-loaded mesoporous silica nanoparticles (F-MSN) with multi-branched polypropylene imine (PPI) and investigate its effect on GB cells.

**Material and Methods**

F-MSN were synthesized by employing sol-gel approach using tetraethylorthosilicateas and hexadecyltri-methylammonium bromide. Multi-branched PPI groups were formed by F-MSN surface growth to ensure the OL and HT load. Scanning electron microscopy and dynamic light scattering were performed for characterization analysis of NPs. The effect of OL and HT-loaded F-MSN-PPIs on GB cell viability was investigated in T98G cells using a WST-1 assay. In addition, upon OL or HT-loaded F-MSN-PPIs and their combination with TMZ, the changes in cancer-characteristic features of T98G cells were analyzed by scratch-wounding, colony formation, and 3D organoid sphere formation assays. The safety of F-MSN-PPIs was tested on L929 fibroblast cells.

**Results and Discussions**

It was found that synthesized F-MSNs have spherical structure and particle size of 200 nm. The net surface charge of F-MSN-PPI (+34.15 ± 0.08 mV) has decreased gradually with increasing OL or HT loading degree. The IC50 of F-MSN-PPI-OL and F-MSN-PII-HT was 196.83 µg/mL and 190.23 µg/mL at 24h in T98G cells. F-MSN-PPI had no cytotoxic effect on L929 cells up to 200 µg/mL concentration. F-MSN-PPI-OL and F-MSN-PPI-HT attenuated the wound healing of T98G cells compared to untreated cells ($p<0.05$). Both F-MSN-PPI-OL and F-MSN-PPI-HT reduced the colony forming of T98G cells and reduced their sphere size ($p<0.05$). In addition, the involvement of F-MSN-PI-loaded flavonoids, OL or HT in TMZ had a stronger anti-cancer effect than TMZ alone in all applied functional assays ($p<0.05$).

**Conclusion**

OL or HT-loaded F-MSN-PPIs could be a new candidate for developing innovative tools to treat GB.

### EACR2024-0595

### Growth hormone receptor antagonism reduces melanoma tumour growth in vivo

*M. Kim[1], Y. Wang[1], R. Langley[2], K. Sousa[2], K.B. Tran[2], P. Shepherd[2], S. Jamieson[3], J. Perry[1]*
*[1]University of Auckland, Liggins Institute, Auckland, New Zealand*
*[2]University of Auckland, Department of Molecular*

© 2024 The Authors. Molecular Oncology is published by John Wiley & Sons Ltd on behalf of Federation of European Biochemical Societies

*Medicine and Pathology, Auckland, New Zealand*
*[3]University of Auckland, Auckland Cancer Society Research Centre, Auckland, New Zealand*

**Introduction**

Despite recent advances in treatment, metastatic melanoma is largely refractory to clinically available therapies, highlighting the need for novel therapeutics. Growth hormone receptor (*GHR*) mRNA expression is almost 50-fold higher in melanoma cell lines compared to other cancer cell lines. Characterization of a panel of 102 primary metastatic melanoma cell lines (NZM panel) demonstrated that the majority express *GHR* mRNA and are responsive to growth hormone (GH). GH has been shown to contribute to cell proliferation, survival, and chemoresistance. Pegvisomant, the only clinically available GHR antagonist, is difficult to access for research so novel GHR antagonists are needed to investigate the role of GHR signaling in melanoma. The aim of this study was to evaluate the efficacy of an in-house generated GHR antagonist (GHA2) as a monotherapy in melanoma tumor models and determine its potential therapeutic application.

**Material and Methods**

Recombinant GHA2 protein was expressed and purified from *E. coli* and conjugated with polyethylene glycol to extend the in vivo circulating half-life of the drug. In vitro bioactivity was confirmed using cell-based assays and inhibition of GHR-dependent signal transduction by western blot (STAT5 phosphorylation). Mouse melanoma B16-F10 syngeneic graft grown in C57BL/6J mice were treated daily with vehicle or GHA2 (30 mg/kg/day) for 2 weeks. Human melanoma NZM79 xenografts grown in immunodeficient NIH-III mice were treated daily with vehicle or GHA2 (30 mg/kg/day) ± human GH (2 mg/kg/day) for 2 weeks. Tumors and blood were collected 24 h after the final treatment. Serum insulin-like growth factor 1 (IGF-1) was measured by ELISA analysis as a surrogate biomarker for GHA2 activity.

**Results and Discussions**

GHA2 effectively inhibited GH-dependent cell viability in cell-based drug screening assays and STAT5 phosphorylation in melanoma cell lines. GHA2 treatment significantly reduced the serum IGF-1 concentration by 72% in C57BL/6J mice compared to vehicle control ($p<0.001$). A trend for decreased B16-F10 tumor volume was observed following GHA2 administration, although this was not statistically significant. In NIH-III mice, GHR antagonism with GHA2 significantly decreased the growth rate of NZM79 tumors versus vehicle or GH treatment ($p<0.05$).

**Conclusion**

GHA2 effectively antagonized GH signaling in melanoma cells and slowed melanoma tumor growth, highlighting its potential as a new therapeutic strategy for treating melanoma.

### EACR2024-0617
### Biochemical Precision: Optimizing RAF and MEK inhibitor combinations for effective RAS/MAPK driven cancer therapies

*C. Adamopoulos[1,2], A. Orive-Ramos[2], B. Gaire[2], B. Baars[2], J. Lam[2], J. Huang[2], S.A. Aaronson[2], P.I. Poulikakos[2]*
*[1]National and Kapodistrian University of Athens, Medical School- Department of Biological Chemistry, Athens, Greece*
*[2]Icahn School of Medicine at Mount Sinai, The Tisch Cancer Institute- Department of Oncological Sciences, New York, United States*

**Introduction**

Activating mutations of RAS and RAF proteins, two key components of the RAS-RAF-MEK-ERK (RAS/MAPK) signaling pathway, drive growth of several human cancer. Targeting the pathway using combinations of RAF and MEK inhibitors has been clinically successful for advanced melanoma and non-small cell lung cancer (NSCLC) patients harboring the BRAF(V600E) mutation. However, this combinatorial strategy has been ineffective for cancers driven by RAS mutations. The currently approved BRAF inhibitors, RAF monomer-selective inhibitors, target selectively and inhibit the monomeric form of BRAF(V600E) oncoprotein in cancer cells but not in normal cells where BRAF-wild type signals as a RAS dependent RAF dimer. Additionally, the development of RAF-dimerization mediated adaptive resistance limits therapeutic effectiveness of BRAF monomer-selective inhibitors. Next generation RAF inhibitors equipotent for BRAF monomers and dimers have been developed but they are predicted to have limited therapeutic index due to concomitant inhibition of dimeric wild-type BRAF in normal cells. We have previously identified and characterized a novel class of RAF dimer-selective inhibitors that preferentially bind and inhibit BRAF dimers over monomers. Moreover, their rational triple combination together with a RAF monomer-selective inhibitor and a MEK inhibitor led to effective targeting of therapy resistant BRAF(V600E) driven tumors.

**Material and Methods**

To rationally design such combinatorial approaches for RAS-mutated cancers, we explored the efficacy of RAF and MEK inhibitors belonging to distinct biochemical and structural classes, in RAS-mutant tumor and RAS-wild type normal cellular models.

**Results and Discussions**

Our findings revealed that RAF dimer-selective inhibitors can suppress MAPK signaling and cell growth more potently in RAS-mutant cells compared to normal cells. Further, certain RAF and MEK inhibitor combinations resulted in more potent MAPK inhibition and cell growth in RAS-mutant cancer cells compared to RAS wild type cells, showing increased “therapeutic synergy” and providing evidence for clinical evaluation. Conversely, certain combinations are more potent in normal cells than in tumor cells, showing "adverse synergy" and are predicted to be clinically unsuccessful.

**Conclusion**

In conclusion, rationally designed combinations of selected RAF and MEK inhibitors, based on their distinct biochemical properties, can unveil new precise therapeutic strategies for more effective targeting RAS/MAPK driven cancers.

© 2024 The Authors. Molecular Oncology is published by John Wiley & Sons Ltd on behalf of Federation of European Biochemical Societies

### EACR2024-0619
### Uncovering the Druggable Proteome of Aggressive Childhood Cancers for Target-Based Drug Discovery

J.P. Ong[1,2], J. Bertoldo[1,2]
[1]Children's Cancer Institute Australia, Chemical Biology and Target-Based Drug Design, Sydney, Australia
[2]UNSW, Faculty of Medicine and Health- School of Clinical Sciences, Sydney, Australia

**Introduction**
Despite advancements in cancer treatment, childhood cancers remain neglected and are often treated with adult drugs, leading to suboptimal efficacy and adverse effects. Addressing the urgent need for effective and safer treatments for children requires overcoming two major challenges: a lack of druggable targets in childhood cancer and lack of drugs specifically developed for the paediatric population. To address these needs, the research objective is to develop a pipeline that integrates chemoproteomics, which explores interactions between chemical probes and protein targets, with functional genomics data. This approach aims to uncover cancer-relevant targets specific to childhood malignancies and discover promising covalent probes targeting these cancer dependencies. This cancer-agnostic pipeline aims to be encapsulated in an accessible, user-friendly web application. The feasibility of this approach is tested in neuroblastoma (NB) and medulloblastoma (MB).
**Material and Methods**
Functional genomic and chemoproteomics datasets were analysed to identify chemical probes targeting novel cancer-specific NB and MB genetic dependencies. These chemical probes were screened in cell viability assays in multiple cancer and non-cancer cells. Functional validation methods were used to confirm target engagement, on-target inhibition, and anti-cancer activity for discovered chemical probes.
**Results and Discussions**
From an initial set of 16,000 genes and 1,000 chemical probes we identified 65 NB-specific and 20 MB-specific cancer dependencies each targeted by at least one selective chemical probe. We prioritised the top 3 chemical probe/target pairs in NB and MB and initially validated their anti-cancer activity/biological relevance. Notably, chemical probes CP174 targeting IGF2BP3 (NB) and CP344 targeting SAFB2 (MB) showed selectivity and anti-cancer effects in cell viability and clonogenic assays. Synergistic effects and on-target actions of CP174 were supported by reduced mRNA levels in RNA-immunoprecipitation assays and enhanced probe potency upon target depletion. The anti-cancer and selective features of these chemical probes highlight the feasibility of this pipeline to identify and target cancer dependencies effectively.
**Conclusion**
Our research highlights a multidisciplinary approach to discover new cancer-relevant proteins and chemical probes targeting these proteins. These probes show promising drug-like properties and on-target effects, supporting this method's potential in discovering novel treatments for cancers.

### EACR2024-0620
### Co-targeting BET, CBP, and p300 inhibits neuroendocrine signalling in androgen receptor-null prostate cancer

N. Choo[1], S. Keerthikumar[2], S. Ramm[3], D. Ashikari[4], K.J. Simpson[3], R.A. Taylor[1], G.P. Risbridger[1], M.G. Lawrence[1]
[1]Monash University, Biomedicine Discovery Institute, Melbourne, Australia
[2]Peter MacCallum Cancer Centre, Sir Peter MacCallum Department of Oncology, Melbourne, Australia
[3]Peter MacCallum Cancer Centre, Victorian Centre for Functional Genomics, Melbourne, Australia
[4]Nihon University School of Medicine, Department of Urology, Tokyo, Japan

**Introduction**
Diverse phenotypes of castration-resistant prostate cancer (CRPC), including neuroendocrine disease, have differing sensitivity to drug treatment. The efficacy of BET and CBP/p300 inhibitors in prostate cancer is attributed, at least in part, to their ability to decrease androgen receptor (AR) signalling. However, the activity of BET and CBP/p300 inhibitors in prostate cancers that lack the AR is unclear. Therefore, our goal was to use patient-derived models to investigate the response of AR-null tumours to BET and CBP/p300 inhibition.
**Material and Methods**
We performed immunohistochemical staining of BRD4, CBP, and p300 in 170 prostate cancer patient-derived xenografts (PDX) from the MURAL and Movember GAP1 consortium. We next treated diverse prostate cancer organoids in a high-content assay, as well as PDXs with neuroendocrine pathology, with NEO2734, a first-in-class dual inhibitor of BET and CBP/p300 proteins that is in phase 1 trials for CRPC. RNAseq analysis was performed on these tumours collected after acute and long-term NEO2734 treatment to investigate transcriptional responses.
**Results and Discussions**
Across large cohorts of PDXs, BRD4, CBP, and p300 were co-expressed in AR-positive and AR-null prostate cancer. NEO2734 reduced the growth of both AR-positive and AR-null organoids, as measured by multiple independent readouts of viability, size and composition, and caused consistent transcriptional downregulation of cell cycle pathways. In neuroendocrine models, NEO2734 treatment reduced *ASCL1* levels and other neuroendocrine markers, and reduced tumour growth in vivo
**Conclusion**
These results show that epigenome-targeted inhibitors cause decreased growth and phenotype-dependent disruption of lineage regulators in neuroendocrine prostate cancer, warranting further development of compounds with this activity in the cliic.

### EACR2024-0629
### Development of Abbapolin inhibitors of PLK1 PBD as potential cancer therapeutics and as novel probes of PLK1 conformation

Molecular Oncology 18 (Suppl. 1) (2024) 1–495
© 2024 The Authors. Molecular Oncology is published by John Wiley & Sons Ltd on behalf of Federation of European Biochemical Societies

G. Merhej[1], J. Stafford[1], D. Chapagai[1], R. Chintada Nageswara[1], M. Wyatt[1], C. McInnes[1]
[1]University of South Carolina, Drug Discovery and Biomedical Sciences, Columbia, United States

**Introduction**

Polo- like kinase 1 (PLK1) has different cellular functions, among which mitotic regulation is the most well studied. PLK1 is upregulated in many cancers and blocking PLK1 is strongly anti-proliferative in cancer. Inhibitors of PLK1 have been tested clinically but have not fulfilled preclinical promise. These inhibitors block the PLK1 catalytic site, which could explain, in part, the lack in specificity of these drugs. We have developed inhibitors that target the Polo Box Domain (PBD) of PLK1, rather than the Kinase Domain (KD). We studied the inhibition potential of those inhibitors in vitro, in cellular assays, and in vivo. Moreover, we used those inhibitors as well as the KD inhibitor BI2536 as probes to study the conformational dynamics of PLK1.

**Material and Methods**

REplacement with Partial Ligand Alternatives through Computational Enrichment (REPLACE), Fluorescence Polarization (FP) assay, MTT Assay, NCI-60 data, Immunoblotting, Immunofluorescence, Cellular Thermal Shift Assay (CETSA), Xenograft models of prostate cancer.

**Results and Discussions**

Our compounds, named Abbapolins, were shown by Fluorescence Polarization Assay (FP) to block PBD binding to peptide tracers. Abbapolins show anti-proliferative activity (NCI-60 panel), induce cellular degradation of PLK1, and show promising in vivo activity. In addition, we use abbapolins as probes to better understand the dynamics governing the conformation of PLK1. Though the conformational dynamics of PLK1 remain elusive, those changes could be critical determinants of the protein’s function, cellular localization, and response to therapy. Recently, we show using thermal stability experiments and FP experiments that the KD binding drug BI2536 induces an "open conformation" of PLK1, causing a reduced thermal stability, and renders PLK1 more accessible to abbapolin binding. Previous results suggest that the open conformation is required for catalytic activity, whereas the closed conformation is auto inhibitory. Interestingly, abbapolin binding to cellular PLK1 induces its degradation, whereas KD-binding molecules cause the intracellular accumulation of PLK1.

**Conclusion**

The inhibition potential of abbapolins and the novel conformational changes in PLK1 will have important implications in the context of cancer therapy. Furthermore, current work in our lab is focused on understanding PLK1 interaction with other proteins in the context of conformational changes in PLK1.

**Permission has been obtained from IACUC for animal studies*
**No financial interest to disclose*

### EACR2024-0632
### Targeting Telomerase: A Structure-Based Drug Design Approach for Investigating the Anti-Cancer Potential of FDA Approved Drugs and Novel Lead Candidates Using In-Silico and In-Vitro Assays

D. Kaur[1], M. Chopra[2], D. Saluja[3]
[1]Dr. B.R Ambedkar Center for Biomedical Research- Delhi University- Delhi- India, Medical Biotechnology Laboratory and Laboratory of Molecular Modelling and Anticancer Drug Development, Delhi, India
[2]Dr. B.R Ambedkar Center for Biomedical Research- Delhi University- Delhi- India, Laboratory of Molecular Modelling and Anticancer Drug Development, Delhi, India
[3]Dr. B.R Ambedkar Center for Biomedical Research- Delhi University- Delhi- India, Medical Biotechnology Laboratory, Delhi, India

**Introduction**

Replicative immortality caused by increased telomerase activity is an established hallmark of cancer malignancies, making it an attractive target to develop anti-cancer drugs. The complex, multi-subunit enzyme has long posed a challenge due to the unavailability of a high-resolution crystal structure of hTERT and narrow therapeutic window hindering approval of any small molecule inhibitor. Our study focuses on drug repurposing approach to investigate the therapeutic potential of FDA Approved drugs as well as identify novel leads that inhibit telomerase enzyme using a molecular dynamics-based structure guided approach followed by the experimental evaluation using an in-vitro TRAP assay.

**Material and Methods**

Due to unavailability of a co-crystalised structure of BIBR1532 with the catalytic hTERT thumb domain, we devised a Molecular dynamics-based method to identify the exact binding site of the inhibitor. Two pharmacophore models were generated for the putative and newly identified binding pockets, which were screened virtually through the Drug Bank and novel libraries. The models were validated on the basis of fit value of the BIBR1532 and selectivity value indicating the favourable feature set required. The top hits obtained were filtered using druglikliness parameters like Lipinski, ADME and TOPKAT followed by redocking into their binding site. Finally, lead drugs that were able to dock in the new pocket were validated using Molecular Dynamics (MD)simulation studies and their binding free energy was calculated using MM-PB(GB)SA calculations followed by their PCA analysis and FEL characterization.

**Results and Discussions**

The structural investigation, molecular docking studies, and confirmatory molecular dynamics revealed that the exact binding site of BIBR1532 is 8.4 Å away from the reported FVYL pocket, exhibiting clinically relevant and characteristic interactions conserved. We have identified five lead drugs and four novel lead compounds that demonstrated stability in the new binding pocket, as indicated by their MD trajectory analysis based on parameters such as RMSD, RMSF, H-bonds, Radius of Gyration, and MM-PB(GB)SA scores. PCA and FEL characterization further unveiled important interactions. These compounds are also undergoing evaluation using the in-vitro TRAP assay.

**Conclusion**

© 2024 The Authors. Molecular Oncology is published by John Wiley & Sons Ltd on behalf of Federation of European Biochemical Societies

The study identified five potential clinically safe FDA-approved drugs which can be repurposed directly and four novel lead compounds against telomerase for advancing cancer therapeutics in future after further validation studies.

### EACR2024-0634
### Targeted drug discovery in paediatric diffuse midline glioma using chemoproteomics

J. Zhu[1,2,3], M. Tsoli[2], D. Ziegler[2,3,4], J. Bertoldo[1,3]
[1]Children's Cancer Institute Australia, Chemical Biology and Target-Based Drug Design, Randwick, Australia
[2]Children's Cancer Institute Australia, Brain Tumour, Randwick, Australia
[3]UNSW Medicine and Health, School of Clinical Medicine, Kensington, Australia
[4]Kids Cancer Centre, Sydney Children's Hospital, Sydney, Australia

**Introduction**
Paediatric diffuse midline glioma (DMG) is an aggressive brain cancer with the worst prognosis among all childhood cancers. It is primarily driven by histone H3K27M mutations in the genes encoding either histone H3.1 or H3.3 which are considered 'undruggable'. We propose that utilising chemoproteomics to explore novel DMG dependencies, including 'undruggable' drivers like H3K27M oncohistones, could lead to breakthrough targeted therapies.
**Material and Methods**
We utilized chemoproteomic methods and molecular docking simulations to develop a small molecule compound of interest known as JNSY1. The in vitro efficacy of JNSY1 was assessed via cell viability assays and soft-agar colony formation assays. Proteomic analyses, including label-free mass spectrometry and Western blotting were conducted on treated cells to elucidate JNSY1's mechanism of action in DMG cells.
**Results and Discussions**
JNSY1, was identified as a compound that modulates histone H3.1. It exhibits ideal drug-like properties, is predicted to penetrate the BBB and comply with Lipinski's rule of five. JNSY1 showed potent activity at low micromolar concentrations, particularly against H3.1K27M cell lines over wild-type and H3.3K27M cell lines. Soft-agar colony formation assays also showed that H3.1K27M cells were more sensitive to JNSY1 than H3.3K27M cells. This suggests that JNSY1's mechanism of action selectively targets H3.1K27M DMGs. Western blotting revealed increased expression of cleaved PARP-1, confirming that activation apoptosis was responsible for causing cell death. Proteomic analyses found that JNSY1 induced distinct changes to protein expression between H3.1K27M and H3.3K27M mutant cells, including downregulation of histone binding partners, chaperones, and chromatin remodelers. Additionally, key effectors in the DNA damage response and DNA repair such as the BRAT1, POLD2 and FANCI were downregulated. These lines of evidence suggests that JNSY1 disrupts histone H3.1 function, which may trigger apoptotic cell death through epigenetic reprogramming and repression of DNA damage repair.
**Conclusion**
We have found a promising first-in-class small molecule that modulates histones with potent bioactivity against DMG cells with selective activity against H3.1K27M-mutant DMG. Further studies will be conducted to confirm target engagement and in vivo efficacy.

### EACR2024-0681
### Overcoming Therapeutic Resistance: Remodelling the Prostate Cancer Microenvironment using Liposomal Nanomedicines

K. Greenland[1], D. Leach[1], J. Hindley[2], A. Varela-Carver[1], O. Ces[2], C. Bevan[1]
[1]Imperial College London, Surgery and Cancer, London, UK
[2]Imperial College London, Chemistry, London, UK

**Introduction**
Prostate cancer (PCa) is the second most common cause of cancer-related death in men globally. It is now well established that epithelial-derived cancer cells of solid tumours do not exist in isolation but in a complex cancer ecosystem, termed the tumour microenvironment (TME). The prostate TME influences drug response and resistance through various means, including acting as a fibrotic/mechanical barrier that prevents drug penetration into the tumour. High intratumoral pressure caused by this barrier effect also results in tumour hypoxia and reduced T-cell infiltration, further driving drug resistance. Targeting the PCa tumour microenvironment could, therefore, improve the performance of approved drugs and synergise with immunotherapies. Moreover, emerging evidence suggests that blockade of androgen receptor (AR) signalling, the usual treatment for disseminated disease, may within the PCa tumour microenvironment, promote conditions favourable for cancer growth, progression, and metastasis. Consequently, systemic anti-AR therapies that disrupt this signalling may predispose PCa to malignant transformation and migration.
**Material and Methods**
Utilising 3D cell culture, patient explant culture and in vivo models in combination with membrane biophysical engineering we are developing a liposomal nanomedicine capable of selectively releasing remodelling compounds into the prostate TME.
**Results and Discussions**
Using the FDA-approved antihypertensive Losartan as a remodelling drug, we demonstrated cytotoxic effects on cancer epithelial cell lines and two immortalised prostate myofibroblast (stromal) lines. Our data suggest that Losartan can impede collagen synthesis and downregulate the expression of microenvironment markers associated with tumour progression. Further, in explant culture of PCa patient samples, in which the complex cancer cells and microenvironment interactions are maintained, Losartan treatment downregulated cell proliferation. In vivo, animal models demonstrated that our liposomal formulations are well-tolerated and detectable in blood plasma without systemic toxicity.
**Conclusion**

© 2024 The Authors. Molecular Oncology is published by John Wiley & Sons Ltd on behalf of Federation of European Biochemical Societies

These findings highlight the utility of targeting the renin-angiotensin system as a novel approach in PCa and, more specifically, using Losartan as a microenvironment remodelling compound.

### EACR2024-0685
### Tumour selective targeting of hypoxia-activated ATR eradicates treatment-resistant hypoxic cells in triple negative breast cancers

*F. Mprah Barnieh*[1], *G. Ribeiro Morais*[1], *P. Loadman*[1], *R. Falconer*[1], *S. El-Khamisy*[1]
[1]*University of Bradford, Institute of Cancer Therapeutics, Bradford, United Kingdom*

**Introduction**

Hypoxia adaptation of tumour cells is critical to treatment resistance and poor prognosis of triple negative breast cancers (TNBCs). ATR kinase is an established DNA damage repair (DDR) protein whose non-DDR functions have recently been demonstrated as key to the survival of hypoxic cancer cells. This positions hypoxia selective delivery of ATR inhibitors as a favourable approach in selectively eradicating hypoxic cancer cells whilst avoiding normal tissue toxicities associated with ATR inhibition.

**Material and Methods**

A series of hypoxia-activated prodrug (HAP) of AZD6738, an ATR inhibitor, were synthesised and purified by preparative HPLC. The metabolism and activity of synthesised compounds were evaluated using bactosomal human NADPH-CYP reductases and in TNBC tissues in hypoxic (0.1% $O_2$), and normoxic (21% $O_2$) conditions. The toxicities of the synthesised HAP we also evaluated in normal human cells. Finally, we evaluated the multicellular penetrating ability and activity of synthesised HAP in 3D TNBC spheroids

**Results and Discussions**

We report a newly developed hypoxia-activated prodrug (ICT10336) which is selectively metabolised in hypoxic TNBC cells to release AZD6738, an ATR inhibitor. ICT10336 was demonstrated to be metabolically stable in normal tissue homogenates, and non-toxic to normal human cells. However, in hypoxic conditions, ICT10336 was bio-reduced by CYPOR and subsequently metabolised by CD13 to release free AZD6738 in both TNBC cells and preclinical cancer tissue homogenates. ICT10336 inhibited ATR activation (T1989 and S428 phosphorylation) in hypoxic TNBC cells and subsequently abrogated HIF-1A mediated hypoxia adaptation mechanisms, thus inducing apoptosis and cell death in 2D and 3D TNBC models. Interestingly, compared to AZD6738, ICT10336 demonstrated superior and efficient multicellular penetration ability in TNBC spheroids and selectively eradicated cells at the hypoxic core.

**Conclusion**

We have developed a new, non-toxic HAP of AZD6738, which demonstrates a less toxic therapeutic strategy to target treatment-resistant hypoxic cancer cells. This preclinical data supports further development of hypoxia-activated prodrugs of ATR inhibitors for for a more beneficial clinical applications

### EACR2024-0687
### Newly established in vitro drug-testing platform reveals Ceritinib as an alternative treatment strategy for hepatoblastoma

*S. Demir*[1], *S. Cairo*[2], *E. Indersie*[3], *R. Kappler*[4]
[1]*Dr. von Hauner Children's Hospital- University Hospital- Ludwig-Maximilians-University of Munich, Department of Pediatric Surgery, München, Germany*
[2]*Champions Oncology Inc., Research Headquarters, Rockville, United States*
[3]*XenTech, R&D and Contract Research, Evry, France*
[4]*Dr. von Hauner Children's Hospital- University Hospital- Ludwig-Maximilians-University of Munich, Department of Pediatric Surgery, Münich, Germany*

**Introduction**

The overall survival rate of patients with hepatoblastoma (HB), the most frequent primary pediatric liver tumor, has significantly increased up to 80% due to advancements in surgical methodology and elaborate clinical risk stratification. However, there is still a pertinent subgroup of high-risk patients presenting with challenging features, such as distant metastasis and resistance to chemotherapy, whose outcome remains inferior. Therefore, investigating potential therapeutics to strengthen standard-of-care (SOC) is desperately required. Thus, we aimed to establish a comprehensive in vitro drug-testing platform to determine the anti-tumor effectivity of potential novel therapeutic agents

**Material and Methods**

Our platform comprised of 5 liver tumor cell lines, 7 HB patient-derived xenograft cultures and 3 non-cancerous cell lines as controls. We assessed 78 perturbagens, including the SOC medication, drugs used in the current pediatric hepatic international tumor trial (PHITT), as well as drugs targeting different cellular pathways. Response to drugs were assessed by viability assay, and validated by in vitro cellular assays and in vivo mouse experiments.

**Results and Discussions**

Our drug testing pointed considerable efficacy of a group of compounds targeting protein-tyrosine kinases (PTK); consequently, we identified Ceritinib (Ceri) to be the most effective PTK inhibitor against HB. Western blot analysis showed, that Ceri can intervene with multiple signaling pathways by decreasing phosphorylation of AKT, ERK1/2, MTOR and FAK proteins. The validation of Ceri revealed decreased cell viability with significantly lower half-maximal inhibitory concentrations (IC50) for HB cells (median IC50= 1 μM) as compared to healthy controls (IC50> 50 μM). Moreover, we observed loss of proliferation, hindered colony formation capability, and induction of apoptosis in HB cells upon Ceri exposure. Furthermore, utilizing 3-dimensional HB tumor models proved significant anti-tumor activity of Ceri, represented by smaller volumes of Ceri treated HB spheroids. Notably, the combination of Ceri and cisplatin revealed a strong synergistic effect, comparable to the one of cisplatin and doxorubicin, which is given to high-risk hepatoblastoma patients. Most importantly, Ceri significantly reduced tumor growth in a

© 2024 The Authors. Molecular Oncology is published by John Wiley & Sons Ltd on behalf of Federation of European Biochemical Societies subcutaneous xenograft transplantation mouse model, in vivo.

**Conclusion**

By using a newly established drug-testing platform we identified ceritinib as a novel therapeutic approach for HB, especially when combined with cisplatin.

### EACR2024-0694

### Exploring the Therapeutic Potential of 1,2-epoxysteroids in Pancreatic Cancer Treatment

*A.R. Gomes*[1,2], *M.C. Gaspar*[1,3], *S. Costa*[4], *C.L. Varela*[5,6,7], *A.M. Abrantes*[1,6,8], *R. Alves*[6,8,9], *M.F. Botelho*[1,6,8], *E. Tavares-da-Silva*[2], *A.S. Pires*[1,6,8], *F. Roleira*[2]

[1]*Univ Coimbra, Coimbra Institute for Clinical and Biomedical Research iCBR area of Environment Genetics and Oncobiology CIMAGO- Biophysics Institute of Faculty of Medicine, Coimbra, Portugal*

[2]*Univ Coimbra, CERES- Faculty of Pharmacy- Laboratory of Pharmaceutical Chemistry, Coimbra, Portugal*

[3]*Univ Coimbra, Faculty of Sciences and Technology- Coimbra- Portugal, Coimbra, Portugal*

[4]*Univ Coimbra, Faculty of Pharmacy- Laboratory of Pharmaceutical Chemistry, Coimbra, Portugal*

[5]*Univ Coimbra, CERES- Department of Chemical Engineering, Coimbra, Portugal*

[6]*Univ Coimbra, Center for Innovative Biomedicine and Biotechnology CIBB, Coimbra, Portugal*

[7]*Univ Coimbra, Coimbra Institute for Clinical and Biomedical Research iCBR, Coimbra, Portugal*

[8]*Clinical Academic Center of Coimbra CACC, Coimbra, Coimbra, Portugal*

[9]*Univ Coimbra, Coimbra Institute for Clinical and Biomedical Research iCBR area of Environment Genetics and Oncobiology CIMAGO- Laboratory of Oncobiology and Hematology and University Clinics of Hematology and Oncology- Faculty of Medicine, Coimbra, Portugal*

**Introduction**

Pancreatic cancer (PanC) has become one of the deadliest malignant tumors with a 5-year survival rate of about 5% despite the currently available therapeutic options, reinforcing the need for novel therapeutic approaches. The introduction of an epoxide function in the steroidal backbone, namely in the 1,2-position of the A-ring, has proven to increase the molecule's cytotoxicity in several cancer cell lines. Thus, and given the heavy burden of PanC, we intended to evaluate the potential anticancer activity of new 1,2-epoxysteroids in several PanC cell lines.

**Material and Methods**

Compounds EP1, EP2 and EP3 and their corresponding parent compounds OL1, OL2 and OL3 were synthesized, and their cytotoxicity was evaluated in MIA PaCa-2, PANC-1, and Hs 766T cancer cells through the SRB assay, 48h after treatment with the compounds (1 to 75 μM). Cell viability, cell death profile and alterations in cell cycle were evaluated by flow cytometry.

**Results and Discussions**

In general, all steroidal epoxides decreased MIA PaCa-2, PANC-1 and Hs 766T cancer cells proliferation in a dose-dependent manner. The most sensitive cell line to EP1, EP2 and EP3 was MIA PaCa-2 with $IC_{50}$ values of 11.91, 3.88 and 10.76 μM, respectively. On the other hand, in general, the parent compounds were much less cytotoxic to all the cell lines studied, compared to their corresponding epoxides, proving that the introduction of the epoxide function improved the cytotoxicity displayed. Flow cytometry studies with EP2 (the most active compound) revealed that it was able to decrease MIA PaCa-2 cancer cell viability, causing cell death by apoptosis and necrosis at higher doses. This was accompanied by a cell cycle blockage at phase $G_2$/M.

**Conclusion**

EP2 displayed a cytotoxic effect mediated by apoptosis and necrosis. These results encourage further studies on its mechanism of action and selectivity to discover new molecules for the treatment of PanC.

*Funding: CIBB strategic projects: 10.54499/UIDB/04539/2020 and 10.54499/UIDP/04539/2020 and Associated Laboratory funding 10.54499/LA/P/0058/2020. CERES strategic projects: 10.54499/UIDB/00102/2020, 10.54499/UIDP/00102/2020. All projects were supported by FCT. PhD Fellowship from FCT and European Social Funding of Ana R. Gomes (UI/BD/150865/2021).*

### EACR2024-0709

### Deciphering the anticancer activity of Cotinus coggygria extracts and compounds with high selectivity against myelogenous leukemia cells

*I. Pasic*[1], *I.Z. Matic*[1], *T. Stanojkovic*[2], *M. Novakovic*[3], *V. Tesevic*[4]

[1]*Institute for Oncology and Radiology of Serbia, Laboratory for Radiobiology and Experimental Oncology, Belgrade, Serbia*

[2]*Institution for Oncology and Radiology of Serbia, Laboratory for Radiobiology and Experimental Oncology, Belgrade, Serbia*

[3]*University of Belgrade - Institute of Chemistry- Technology and Metallurgy- National Institute of the Republic of Serbia- Belgrade- Serbia, Institute of Chemistry- Technology and Metallurgy- National Institute of the Republic of Serbia, Belgrade, Serbia*

[4]*University of Belgrade – Faculty of Chemistry- Belgrade - Serbia, Faculty of Chemistry- Belgrade- Serbia, Belgrade, Serbia*

**Introduction**

Three extracts and twelve compounds were isolated from *Cotinus coggygria*. This study explored anticancer effects of ethanol (E), methylene chloride/methanol (M), and water (W) extracts and twelve compounds (butin, butein, fisetin, sulfuretin, taxifolin, eriodictyol, fustin, cotinignan A, sulfuretin auronol, 3-O-methylepifustin, 3-O-methylfustin, sitosterol-3-O-β-D-glucoside). Among these, 3-O-methylepifustin, epitaxifolin, and sulfuretin auronol have been discovered for the first time in *C. coggygria*.

**Material and Methods**

Cytotoxic effects of extracts and compounds were evaluated against human malignant cell lines: cervical adenocarcinoma HeLa, malignant melanoma A375, triple-negative breast cancer MDA-MB-231, prostate carcinoma DU 145, prostate adenocarcinoma PC-3, and chronic myelogenous leukemia K562, as well as against normal human cell lines, keratinocytes HaCaT and lung fibroblasts MRC-5 by MTT assay. The influence of

© 2024 The Authors. Molecular Oncology is published by John Wiley & Sons Ltd on behalf of Federation of European Biochemical Societies extracts, butin, butein, and sulfuretin on the cell cycle phase distribution and induction of apoptosis were investigated by flow cytometry in HeLa cells. Their effects on ROS concentrations in MRC-5 cells were measured by flow cytometric analysis.

**Results and Discussions**

$IC_{50}$ value was as low for butein as 8.66 μM for HeLa and 13.91 μM for K562 cells. Butein showed the highest selectivity in the cytotoxic activity against HeLa cells (SI 9.91), followed by sulfuretin (SI 7.05) and cotinignan A (SI 3.32) against K562 cells, when compared with normal MRC-5 cells. Sulfuretin exerted the highest selectivity against K562 cells (SI 5.75) and butein against HeLa when compared with cytotoxicity on normal HaCaT cells (SI 4.21). Sulfuretin auronol displayed the highest selectivity against K562 cells. The extracts and compounds increased the percentages of HeLa cells in the subG1 phase. Extracts initiated apoptosis in HeLa cells through caspase 3, -8, and -9. Butin and butein induced apoptosis in HeLa cells by activating the caspase-8 and -9. The increase in early and late apoptosis was also significant for extracts and butin. Treatment with three extracts and butein and sulfuretin decreased ROS levels in MRC-5 cells. Butein, sulfuretin, E and M extracts exerted cytoprotective properties, demonstrated by a decrease in ROS levels in cells exposed to $H_2O_2$.

**Conclusion**

This study emphasizes the importance of further studying *C. coggygria* extracts and compounds to evaluate their anticancer effectiveness in vivo and as safer complementary agents in cancer therapy.

### EACR2024-0721
### Beyond conventional approaches: Cephaeline's multi-omics drug repurposing approach to reverse bladder cancer phenotype

*M. Hoffmann[1], M. Makridakis[2], T.F. Reese[1], A. Latosinska[3], H. Mischak[3], G. Niegisch[1], M. Roubelakis[4], A. Vlahou[2], M. Frantzi[3], M. Mokou[3]*
*[1]Heinrich Heine University Duesseldorf, Dept. of Urology- Medical Faculty, Duesseldorf, Germany*
*[2]Biomedical Research Foundation of the Academy of Athens, Biotechnology Division, Athens, Greece*
*[3]Mosaiques Diagnostics, Mosaiques Diagnostics, Hannover, Germany*
*[4]National and Kapodistrian University of Athens, Laboratory of Biology- Medical School-, Athens, Greece*

**Introduction**

Treatment resistance poses one major clinical challenge in managing muscle-invasive bladder cancer (MIBC), demanding for the exploration of new therapeutic options to limit tumor aggressiveness and progression. Drug repurposing, reusing existing drugs for new purposes, could accelerate translation into the clinic. Here, we seeked to identify novel drug candidates for the treatment of MIBC through the application of a molecularly-based and data-driven drug repurposing pipeline that analyses multi-omics integrated signatures from patients and cell lines. We further characterized the cellular and molecular effects of the top candidate cephaeline, an alkaloid traditionally used as emetic agent.

**Material and Methods**

Proteomics and transcriptomics data of cell lines and tumors from hundreds of patients were integrated to develop MIBC signatures. Data-driven drug repurposing analysis was performed using the Connectivity Map (CMap) tool. Dose response for cephaeline was performed for BC cell lines (BFTC-905, HT-1376, T24 and RT112) and cisplatin-resistant sublines (T24-LTT, J82-LTT) by MTT assay. Benign HBLAK cells and fibroblasts served as normal controls. The effect on long-term proliferation following $IC_{50}$ treatment was measured using clonogenic assays, induction of cell cycle arrest and cell death by flow cytometry. Molecular mechanisms were investigated through proteome analysis using LC-MS/MS technology. Protein co-expression profiles were built in R.

**Results and Discussions**

CMap analysis identified 13 drugs having potential to reverse the aggressive phenotype of MIBC. $IC_{50}$ concentrations of cephaeline in BC lines ranged between 3.6-6.6 nM. Cisplatin resistant LTT sublines exhibited similar sensitivity. Fibroblasts and benign urothelial HBLAK cells were less sensitive ($IC_{50}$ 12.8 nM and 14.3 nM, respectively). Cephaeline induced cell cycle arrest rather than cell death. Concurringly, long-term proliferation was significantly reduced. Proteomic analysis revealed candidate mechanisms of action.

**Conclusion**

We employed a novel drug repurposing approach to develop more specific and effective drugs for MIBC. Cephaeline emerged as a potent drug inhibiting the proliferation of BC cells, even in cisplatin-resistant settings, while being tolerated by benign control cells. The cytostatic cellular effect was further confirmed at the molecular level. Cephaeline´s impact on tumor growth in a xenograft mouse model is under investigation.

### EACR2024-0737
### FKBPL: A Potential Oncogene in Oesophageal Adenocarcinoma

*C. Duggan[1], G. Moore[1], A. Heeran[2], M.W. Baig[1], C. Kelly[1], S. Annett[1], J. O'Sullivan[2], T. Robson[1]*
*[1]RCSI University of Medicine and Health Sciences, School of Pharmacy and Biomolecular Sciences - 123 St. Stephens Green, Dublin, Ireland*
*[2]Trinity College Dublin and St. James's Hospital, Department of Surgery- Trinity St. James's Cancer Institute- Trinity Translational Medicine Institute, Dublin, Ireland*

**Introduction**

Prognosis for oesophageal adenocarcinoma (OAC) is poor, with 5-year survival rates of 15-25%. Barrett's oesophagus (BO) is a pathological precursor to OAC which may present an opportunity to identify patients at risk of OAC. The mechanisms underlying BO-OAC progression remain to be elucidated and could allow development of robust diagnostic techniques and targeted therapies for BO-OAC. FK506-binding protein-like (FKBPL) is an angiogenesis-regulating protein, which correlates with cancer risk and severity. FKBPL-derived peptides have been shown to alter tumour growth in breast and ovarian cancers. An FKBPL mimetic demonstrated an excellent safety and pharmacokinetic

© 2024The Authors. Molecular Oncology is published by John Wiley & Sons Ltd on behalf of Federation of European Biochemical Societies profile in a human Phase 1a clinical trial in ovarian cancer. Here, we investigated a role for FKBPL in the BO-OAC disease cascade.

**Material and Methods**

FKBPL expression was measured across a human cell line panel representing BO-OAC progression at the mRNA and protein levels using qPCR and Western blotting. Using siRNAs, FKBPL mRNA expression was transiently knocked down in OE33 (adenocarcinoma) cells. The effects of FKBPL knock down on proliferation in this cell line were investigated using the Bromodeoxyuridine (BrdU) incorporation assay. Downstream effects of FKBPL knockdown in this cell line on secreted proteins was investigated using a 52-plex human biomarker ELISA kit. Transcriptome-wide effects of FKBPL knockdown in this cancer cell line were investigated by performing RNAseq analysis.

**Results and Discussions**

An increase in FKBPL mRNA (Fold Change [FC] = 1.4, p = 0.03) and protein (FC = 4, p = 0.02) expression was observed across disease progression from metaplastic (QH) to adenocarcinoma (OE33) cell lines, suggesting a role of increased FKBPL in disease progression. siRNA-mediated FKBPL knockdown in OE33 (adenocarcinoma) cells inhibited cancer cell proliferation (BrdU assay; FC = -0.48, p = 0.0008) compared with control, and decreased expression of inflammatory mediators such as MCP-1 (FC = -0.86, p = 0.0084). RNAseq analysis revealed downregulation of key genes and pathways involved in DNA damage repair, interferon signalling, and cytoskeleton organisation (n=4, FDR ≥ 0.05).

**Conclusion**

Together, these data suggest an oncogenic role for FKBPL in the BO-OAC disease setting and highlight FKBPL as a potential biomarker for identifying BO patients at risk of developing OAC. Specifically, FKBPL may promote oncogenic signalling pathways and enhance DNA repair mechanisms in OAC. Targeting of FKBPL in early-stage disease may prevent BO-OAC progression.

### EACR2024-0767

### The combination of herbs and chemotherapeutic agents inhibits tumor progression via down-regulation of the TNFa-VEGFa/PI3K/Akt/NOS/COX-2-MMP-2 pathway in DMBA-induced breast cancer in rats

*N. Avtandilyan[1], M. Kocharyan[1], S. Hovhannisyan[1], S. Gevorgyan[2], H. Javrushyan[1], M. Ginovyan[1], A. Maloyan[3]*

*[1]Yerevan State University, Institute of Biology, Yerevan, Armenia*

*[2]DeNovo Sciences Inc., DeNovo Sciences Inc., Yerevan, Armenia*

*[3]Oregon Health & Science University, Center for Developmental Health- Knight Cardiovascular Institute, Portland- Oregon, United States*

**Introduction**

The continuous increase in cancer rates, and failure of conventional chemotherapies to control the disease clearly demand alternative approaches. Modulating biochemical and immune functions using medicinal plants combined with chemotherapeutic agents has recently become an accepted therapeutic approach. We hypothesize that phytoextracts, either single or in combination with chemotherapy compounds, may effectively modulate the immune system (TNFa/COX-2), inhibit angiogenesis and progression of metastasis (VEGFa/NOS/NO/MMP-2) via regulation of PI3K/Akt signaling pathway.

**Material and Methods**

We evaluated the effect of herbal extracts (*Rumex obtusifolius, Hypericum alpestre, Inula helenium*, and *Alchemilla smirnovii Juz.*) (2.5mg/kg/day) on TNFa - VEGFa/PI3K/Akt/NOS/COX-2-MMP-2 pathway separately and in combination with 5-FU (10mg/kg/day), nor-NOHA (3mg/kg/day), and L-NAME (30mg/kg/day) in the DMBA-induced (25mg/rat) breast cancer rat model (treatment model administered eight weeks after tumors progression every 4$^{th}$ day). The effect of herbs, either separately or in combination with drugs was examined in blood three times after the DMBA administration: at the beginning of the treatment and tumor development (week 5); post-treatment, and post-completion of the study (weeks 12 and 16 respectively). TNFa, VEGFa, PI3K, Akt, COX-2, and MMP-2 levels were detected in the tumor tissue, lungs, and bone marrow by ELISA.

**Results and Discussions**

The results obtained in the case of combinations of herbs and chemotherapeutic agents showed a decrease in TNFa and VEGFa in blood, tumor, lung, and bone marrow, and the quantity of NO, MDA, and activity of NOS increased in the tumor. The latter is indicative of ROS/RNS-mediated cytotoxicity of herbs in the tumor micro-environment. A decrease in COX-2 and MMP-2 was observed in the tumor and lungs under the influence of herb extracts and combinations, thus showing anti-metastatic effects. Quantitative reduction of total and phosphorylated PI3K and Akt was observed in the tumor under the influence of herb extract. LC-Q-Orbitrap-HRMS analysis identified more than 200 phytochemicals in the ethanol extract of these herbs. Further in silico analyses revealed that 4 of these compounds have a high affinity for PI3K and Akt.

**Conclusion**

By discovering the molecular mechanisms of anticancer effects, determining the most active phytochemicals, and clarifying the specific targets of these compounds, we can potentially inhibit, prevent, or delay the development of cancer with minimal side effects.

### EACR2024-0793

### Cancer cells can escape immune surveillance by altering lipid metabolism

*J. Epiney[1], A. Kasapoglu[1], A. Santamaria-Martinez[1], G. Dienes[1], P. Miranda Herrera[2], L. Abrami[3], G. van der Goot[3], Y. Aye[2], G. D'Angelo[4], E. Oricchio[1]*

*[1]EPFL, Swiss Institute for Experimental Cancer Research, Lausanne, Switzerland*

*[2]EPFL, Institute of Chemical Sciences and Engineering, Lausanne, Switzerland*

*[3]EPFL, Institute of Bioengineering and Global Health Institute, Lausanne, Switzerland*

*[4]EPFL, Institute for Implementation Science in Health*

© 2024 The Authors. Molecular Oncology is published by John Wiley & Sons Ltd on behalf of Federation of European Biochemical Societies

*Care, Lausanne, Switzerland*

**Introduction**

In recent years, the use of monoclonal antibodies (mAb) enhancing anti-tumor immunity through mechanisms such as antibody-dependent cellular phagocytosis (ADCP) has been adopted in several tumor types to improve cancer treatment. Nonetheless, the persisting emergence of therapy resistance and tumor relapse, often due to the upregulation of "don't-eat-me" signals or the loss of target antigens, highlights the critical demand for innovative therapeutic approaches.

**Material and Methods**

In our study, we employed a combination of lipidomics and proteomics to investigate the interactions between cancer cells and innate immune cells within the tumor microenvironment.

**Results and Discussion**

Our findings reveal that specific lipids play a pivotal role in stabilizing "don't eat me" signals on the cell surface membrane. Notably, alterations in lipid distribution within the plasma membrane significantly affect the arrangement of surface receptors. This alteration impacts the intercellular communication between cancer cells and innate immune cells, thereby influencing the immune response in the tumor microenvironment.

**Conclusion**

Overall, our research emphasizes the critical role of plasma membrane composition in facilitating effective communication between cancer cells and the surrounding microenvironment. Alteration in the membrane composition of cancer cells coud provide a mechanism for these cells to evade immune surveillance.

### EACR2024-0794

### Prognostic Modeling for Optimal Cancer Type Selection of an Anti-Cancer Agent Using Primary Tumor Origin-Derived Prognosis-Associated Gene Signature : Implications for HDAC Inhibitor Vorinostat

*S.M. Lee[1], J.W. Song[2], L. Dae Hoon[1], J.T. Cha[2], M.S. Kim[2], J.W. Choi[3]*

*[1]Kyung Hee University, Biomedical and Pharmaceutical Sciences, Seoul, South Korea*

*[2]Kyung Hee University, Pharmacology- Institute of Regulatory Innovation Through Science, Seoul, South Korea*

*[3]Kyung Hee University, College of Pharmacy, Seoul, South Korea*

**Introduction**

Determining effective drugs for a patient is one of the most critical factors in precision medicine. In cancer treatment, the effectiveness of drug can be converted as cancer cell killing capacity of drug. Consequently, measuring drug sensitivity through cell viability emerged as the most intuitive and useful parameter for research a proper indication of drug. Nowadays, In vitro cell line-based drug sensitivity and gene expression data were widely used for drug sensitivity prediction. In this study, we introduced an innovative approach to predict drug sensitivity, proposing a reasonable indication of a specific drug by gene prognostic score analysis. We selected a histione deacetylase inhibitor vorinostat as a target drug.

**Material and Methods**

We analyzed transcriptional expression change data of vorinostat in 20 cancer cell lines. Additionally, we developed a novel cancer patient-derived prognostic score analysis method. we applicated this method to prognostic score analysis of drug target genes. We measured cell viability and basal expression level of survival-associated genes in 9 lung cancer cell lines. Sensitivity marker genes were determined by prognostic score and basal expression in cell lines. Overexpression and knock-down of sensitivity marker genes were conducted to validate the effect of sensitivity marker gene in vorinostat resistance.

**Results and Discussions**

Validation of gene expression changes of vorinostat confirmed that the predicted drug target gene expression changes aligned with the experimentally measured results. The prognostic score of target genes were analyzed by overall survival data of 8 cancer type patients. Total 53 genes were selected as candidates of sensitivity marker gene. Sensitivity of vorinostat in lung cancer cell lines were measured by In vitro cell viability assay. The combination of candidate gene expression data and sensitivity data of cell lines led to identification of sensitivity marker genes. Finally, we identified NCAPH, TTK, MCM5, PRC1, SCPEP1, and ING4 as sensitivity marker genes for vorinostat in lung cancer.

**Conclusion**

This research introduced a predictive system for drug sensitivity based on a prognostic gene signature. We demonstrated that the expression levels of sensitivity marker genes play a crucial role in determining drug sensitivity. Through our analysis of vorinostat sensitivity marker genes in lung cancer, we established that the anti-cancer effect of vorinostat was attributed to transcriptional expression changes in these sensitivity marker genes.

### EACR2024-0808

### Ex vivo micro tumor testing to assess patient-specific sensitivity to targeted therapies

*L. Ceton[1], E. Koedoot[1], T. Sijsenaar[1], A. Drachman[1], M. Scholtes[2], C. Steendam[3], N. Ottevanger[4], D. van der Meer[1], W. Vader[1]*

*[1]VitroScan, Research & Development, Leiden, The Netherlands*

*[2]Erasmus Medical Centre Cancer Institute, Urothelial Oncology, Rotterdam, The Netherlands*

*[3]Catharina Cancer Institute, Pulmonary Oncology, Eindhoven, The Netherlands*

*[4]Radboud University Medical Centre, Medical Oncology, Nijmegen, The Netherlands*

**Introduction**

Targeted therapies have introduced personalized medicine in the clinic and brought great advances in treating cancer patients. However, accumulating evidence indicates that a direct correlation between a mutated cancer gene and a response to a given therapy is too simplistic. The response of individual tumors to therapy is determined by the overall genetic milieu, including any

© 2024 The Authors. Molecular Oncology is published by John Wiley & Sons Ltd on behalf of Federation of European Biochemical Societies alterations in connected signaling pathways. Therefore, advanced tools are needed that transcend standard genetic testing to further improve the stratification of patients for effective therapies.This study investigates ex vivo micro tumor testing as a solution to assess the response to targeted therapies.

**Material and Methods**

Fresh tumor tissue was collected from patients in multiple indications: non-small-cell lung cancer (LC), bladder cancer (BC), and high-grade ovarian cancer (OV). Tumor clusters were isolated, seeded, and exposed to single-agent tumor therapies, while preserving the TME. Targeted therapies osimertinib (EGFR), erdafitinib (FGFR3) and olaparib (PARPi) were included in the assay. Morphological features were extracted from high-throughput 3D imaging data. Sensitivity evaluation was based on AUC comparison of fitted dose-response curves.

**Results and Discussions**

In LC, an EGFR-mutated (exon 19 deletion) tumor, and a tumor with high-copy ERBB/HER2 amplification were more sensitive to osimertinib than EGFR-WT tumors (N=4). Osimertinib is an approved therapy only for the first mutation, while the second aberration, active in the same signaling pathway, induces a similar ex vivo sensitivity. In BC, higher ex vivo sensitivity to FGFR inhibitor erdafitinib was observed in FGFR3-Y375C (N=2) mutated tumor versus FGFR3-WT (N=17, $P < 0.01$), but not to FGFR3-S249C (N=3) mutations. Additionally, differential sensitivity was observed between WT patients. For OC, strong ex vivo sensitivity to olaparib was observed in 7% (6/81) of tested patients. Correlation with the patients mutation status of known HRD genes was insignificant, showcasing the potential added value of the assay.

**Conclusion**

This study evaluates ex vivo patient-specific sensitivity to targeted therapies in three different indications, showcasing reproducible responses in mutated and WT patients. The method integrates the overall complexity of the tissue and cellular pathways that elicit response to treatment. Ex vivo tumor testing is therefore an independent and additional tool to support personalized medicine and support effective development of novel targeted therapies.

### EACR2024-0821

### A Zebrafish Patient Derived Xenograft Model Predicts Tumor Malignancy and Cisplatin Resistance of Ovarian Cancer

*E. Ersoz-Gulseven[1], S. Kurt[2], C. Ulukus[3], G. Cakan Akdogan[4]*

*[1]İzmir Biomedicine and Genome Center, Zebrafish Models Laboratory, Izmir, Turkiye*

*[2]Dokuz Eylul University Faculty of Medicine, Obstetrics and Gynecology, Izmir, Turkiye*

*[3]Dokuz Eylul University Faculty of Medicine, Pathology, İzmir, Turkiye*

*[4]Izmir Biomedicine and Genome Center, Zebrafish Models Laboratory, Izmir, Turkiye*

**Introduction**

Epithelial ovarian cancer is the 7th leading cause of death in women, with 45% 5-year survival rate. Most patients are diagnoses at advanced stages of the disease. While the first-line chemotherapy agent is cisplatin, nearly a quarter of patients are resistant at the time of first administration which causes relapse within the first 6 months. There is a need to predetermine the drug response of the patient before administering the therapy. Zebrafish is a trending model to assess cancer metastasis and drug response. Here, a prospective patient derived xenograft model (zPDX) was used to test malignancy and cisplatin response of ovarian tumors.

**Material and Methods**

Patients with high level CA125 and prediagnosis with malignant ovarian cancer were included in the study with consent and with ethic permisson with protocol no DEU2020/04-52. Piece of tumor was collected at the surgery and cultured to obtain epithelial ovarian cancer cells. Upon reaching confluency, cells were fluorescently stained and injected to the yolk of 2 days old zebrafish larvae. Metastasis, apoptosis and tumor size was quantified 4 days post injection in cisplatin treated and untreated xenografts. Histopathology of tumors and zPDX were compared.

**Results and Discussions**

The malignancy of the tumors was determined after pathology evaluation, therefore the zPDX experiment was done blindly. The tissue culture process was optimized with use of an EOC specific medium. The tumors that did not proliferate under culture conditions turned out to be either benign, borderline or tumors of other origins. The cells of tumors diagnosed as serous epithelial tumor proliferated rapidly in culture conditions and were used to generate zebrafish PDX models for each patient. 0, 20 or 40 µM cisplatin was administered to the PDX larvae and tumor burden was evaluated at the end of 4-day treatment. The PDX larvae in which tumor metastasis rate and tumor size decreased in cisplatin treated groups were considered sensitive. The resistant zPDX correlated with relapse of the patient, sensitive zPDX correlated with disease-free survival of patients for at least 6 months.

**Conclusion**

The findings reported here suggests that in vivo zPDX models can effectively predict cisplatin sensitivity of EOC patients. The short time and small tissue size required for the zPDX test, makes it applicable within 15 days of surgery. The zPDX test has potential to become an advisory test that will support the oncologist for chosing a personalized chemotherapy regime for each patient.

### EACR2024-0828

### A new generation of G-Quadruplex binding compounds: from their synthesis to the characterization of their anticancer effects

*A. Froux[1,2], A. Monari[3], A. Terenzi[2], G. Barone[2], S. Grandemange[1]*

*[1]Centre de Recherche en Automatique UMR 7039 CRAN/ CNRS, Université de Lorraine, Vandoeuvre-lès-Nancy, France*

*[2]Università degli studi di Palermo, STEBICEF*

© 2024 The Authors. Molecular Oncology is published by John Wiley & Sons Ltd on behalf of Federation of European Biochemical Societies

*department- BIP laboratory, Palermo, Italy*
*[3]Itodys Laboratory, Université Paris Cité- CNRS, Paris, France*

**Introduction**

G-rich sequences in nucleic acid molecules can fold into non-conventional secondary structures, as G-quadruplexes (G4). Those motifs are significatively enriched at key sites along the genome as in genes' promoters, sustaining their crucial role in the regulation of many cellular processes. The identification of G4 in the promoters of numerous oncogenes place those structures as promising therapeutic targets for cancer. In that sense, we developed a transdisciplinary approach to bind and stabilize G4 in cancerous cells, with the aim of finding new selective stabilizers to control cancer progression.

**Material and Methods**

In this multidisciplinary study, we synthesized 12 new transition metal complexes, derived from the Salphen scaffold, coordinated with $Zn^{2+}$, $Ni^{2+}$, $Cu^{2+}$, $Pd^{2+}$ and $Pt^{2+}$ metals. DNA-binding behavior was evaluated in solution by spectroscopy techniques, jointly with molecular dynamic simulations. Anticancer activity was investigated in pancreatic and breast cancer cell lines (T3M4, MDA-MB-231 and T47D), by testing the effect on proliferation, viability, and genes expression modulation.

**Results and Discussions**

The in-solution studies of the 12 new complexes showed their ability to bind and stabilize G4 structures, exhibiting preferential selectivity for G4 structures over double-stranded DNA. Molecular dynamic results confirmed the stable interaction between the complexes and the *kRAS* G4, unraveling an unconventional interaction mode, since some molecules bind to the G4 loop. In cancer cells, we confirmed by immunofluorescence assay the ability of the ligands to induce an increase in the G4 number, suggesting the structure stabilization in a cellular environment. Moreover, we showed the ability of our compounds to early downregulate the expression of several G4-driven oncogenes, that could be related to the stabilization of G4s. Based on those promising results, we tested the effect on cell proliferation and viability of cancer cell lines and interestingly showed those molecules can negatively affect such cellular parameters.

**Conclusion**

In this study, we brought a large library of new molecules able to specifically stabilize G4s, exhibiting interesting anticancer properties. Further investigations will be conducted to hopefully allow a better understanding of the G4s' role in a cancerous and tumoral environment and bring further therapeutic prospects in cancerology.

### EACR2024-0865

### Targeting triple-negative breast cancer with a novel CD44 nuclease-resistant RNA aptamer

*A. Caliendo[1], L. Agnello[1], G. Pinto[2], L.E. Ibarra[3], M. Fedele[1], M. Cantile[4], A. Amoresano[2], A. Zannetti[5], S. Camorani[1], L. Cerchia[1]*
*[1]Institute of Experimental Endocrinology and Oncology "G. Salvatore", National Research Council, Naples, Italy*
*[2]University of Naples Federico II, Department of Chemical Sciences, Naples, Italy*
*[3]Institute of Environmental Biotechnology and Health and National Council for Scientific and Technological Research CONICET, National University of Rio Cuarto, Cordoba, Argentina*
*[4]Institutional Biobank-Scientific Directorate, National Cancer Institute INT-IRCCS Fondazione G. Pascale, Naples, Italy*
*[5]Institute of Biostructures and Bioimaging, National Research Council, Naples, Italy*

**Introduction**

Triple-negative breast cancer (TNBC) poses a considerable clinical challenge due to limited targeted therapies and high recurrence rates. Aptamers are short oligonucleotides that, similar to antibodies, recognize their protein target with high specificity and affinity. Several features, as low size, easy production and modification, and high stability, make them excellent candidates for the development of novel targeted anticancer therapies. Recently, we reported the discovery of the sTN58 aptamer, which has striking efficacy in targeting a series of cultured cell lines and clinical patient samples covering different TNBC subtypes. The aptamer, binding to a cytomembrane protein of mesenchymal-like chemoresistant TNBC cells, interferes with their growth as mammospheres and efficiently delivers drug-loaded nanoparticles to the cells. Here, we show that sTN58 specifically targets CD44, a cell surface receptor upregulated in mesenchymal subpopulations of cancer cells and recognized as a molecular marker for cancer stem cells.

**Material and Methods**

Biotinylated sTN58 was used to capture and purify its binding target from membrane protein fraction of cisplatin-resistant MDA-MB-231(Cis-Pt-R) cells through magnetic streptavidin beads. Mass spectrometry combined with aptamer binding assays to different cell lines identified CD44 as the target. Binding analyses on TNBC cell lines expressing CD44 at different extent, and cells depleted from the protein target by siRNA approach, as negative control, were performed by flow cytometry and confocal microscopy approaches. Tumor targeting and antitumor efficacy of sTN58 aptamer were assessed in a 4T1-BALB/c orthotopic breast cancer mouse model.

**Results and Discussions**

The sTN58 aptamer specifically binds to CD44 on Cis-Pt-R cells and accordingly, CD44 silencing results in reduced sTN58 binding. In vivo pre-clinical studies demonstrated both the high tumor targeting efficiency of the aptamer, intravenously injected in tumor bearing mice, and its ability to significantly inhibit tumor growth and lung metastases, with downregulation of PD-L1, in agreement with the recent evidence of the role of CD44 as a key positive regulator of PD-L1 expression in TNBC.

**Conclusion**

Our findings suggest the potential of sTN58 as a targeting reagent for the recognition and therapy of cancers overexpressing CD44.

### EACR2024-0878

### Reduced expression of Myosin Vb in

© 2024 The Authors. Molecular Oncology is published by John Wiley & Sons Ltd on behalf of Federation of European Biochemical Societies

### colorectal tumors as a novel therapeutic vulnerability

B. Brotons Morales[1], S.C.D. van Ijzendoorn[2], À. Martínez-Barriocanal[1], D. Arango[1]
[1]Group of Molecular Oncology, Institut de Recerca Biomèdica de Lleida (IRBLleida), Lleida, Spain
[2]Department of Biomedical Sciences of Cells and Systems, Section Molecular Cell Biology, University of Groningen, University Medical Center Groningen, The Netherlands

**Introduction**

Myosin Vb (MYO5B) is a molecular motor protein highly expressed in the small and large intestine. We have found that MYO5B is significantly reduced in colorectal tumors, leading to loss of cell polarity, differentiation, and tissue architecture. These are hallmarks of advanced colorectal carcinomas and strongly correlate with poor patient prognosis. Interestingly, we have preliminary data showing that loss of MYO5B leads to lysosomal damage and iron accumulation in CACO2 cells, a colorectal cancer (CRC) cell line model. We hypothesized that the lack of MYO5B could sensitize CRC cells to oxidative stress, a vulnerability that could be exploited therapeutically. We aimed to demonstrate if colorectal cancer tumors with low/absent MYO5B exhibit enhanced sensitivity upon treatment with pro-oxidant therapies.

**Material and Methods**

We treated parental and MYO5B CRISPR KO CACO2-BBE cells with increasing concentrations of hydrogen peroxide, the best-known oxidative stress inducer, and measured drug sensitivity by assessing cell viability and clonogenic capacity. We conducted combination treatments of hydrogen peroxide with the antioxidant N-Acetylcysteine (NAC) and the iron chelator deferoxamine (DFO).,We performed equivalent cell viability experiments in DIFI and CCK81 CRC cell lines with transient downregulation of MYO5B (siRNAs). As hydrogen peroxide cannot be used therapeutically, we also evaluated cell sensitivity upon treatment with agents that are known to lead to the production of reactive oxygen species (ROS). Finally, we assessed intracellular ROS levels and lipid peroxidation by cell staining with specific fluorescent probes.

**Results and Discussions**

CRC cells lacking MYO5B displayed higher levels of endogenous baseline ROS levels and increased lipid peroxidation compared to their parental counterparts. Moreover, upon treatment with pro-oxidant compounds, MYO5B deficient cells also accumulated higher levels of ROS. Cells with reduced expression of MYO5B showed increased cell death and enhanced sensitivity when cultured in the presence of pro-oxidant agents, compared to the corresponding parental or non-targeting cell lines. Importantly, this phenotype could be reverted upon treatment with NAC or DFO.

**Conclusion**

CRC cells lacking or with reduced levels of MYO5B exhibit a higher sensitivity to oxidative stress, which potentially represents a therapeutically actionable vulnerability.

### EACR2024-0900
### Derivatization of Caffeic Acid Phenethyl Ester identifies a potential new therapeutic molecule for aggressive T-cell lymphoma

C. Giordano[1], G. Timelthaler[2], D. Kirchhofer[2], J. Kendler[3], M. Sexl[3], S. Kollman[3], S.D. Turner[4], L. Kenner[1,5,6], M. Touaibia[7], O. Merkel[1]
[1]Medical University of Vienna, Department of Pathology, Vienna, Austria
[2]Medical University of Vienna, Center for Cancer Research, Vienna, Austria
[3]University of Veterinary Medicine of Vienna, Institute of Pharmacology and Toxicology, Vienna, Austria
[4]University of Cambridge, Division of Cellular and Molecular Pathology- Department of Pathology, Cambridge, United Kingdom
[5]Medical University of Vienna, Center for Biomarker Research in Medicine CBMed Core Lab 2, Vienna, Austria
[6]Medical University of Vienna, Christian Doppler Laboratory CDL for Applied Metabolomics, Vienna, Austria
[7]Université de Moncton, Chemistry and Biochemistry Department, New Brunswick, Canada

**Introduction**

Anaplastic Large Cell Lymphoma (ALCL) is an aggressive CD30+ non-Hodgkin T-cell lymphoma, which is treated with the classic polychemotherapy regimen based on cyclophosphamide, doxorubicin, prednisone, and vincristine, but around 30% of patients relapse. The natural compound found in bee glue Caffeic Acid Phenethyl Ester (CAPE) was shown to have potential anti-cancer activity in preclinical studies. Our aim was to test CAPE efficacy in ALCL and to improve its pharmacological activity via chemical derivatization, in order to identify a potential new therapeutic molecule for ALCL.

**Material and Methods**

CAPE and synthetic derivatives were tested for their effect on ALCL cell viability. Flow cytometry, cell cycle analysis, Western blot and RNA-Seq were used to elucidate the mechanism of cell death induction. A click chemistry-amenable alkyne derivative allowed intracellular localization by spinning-disk fluorescence microscopy and identification of interacting proteins using Streptavidin/Biotin pull-down and mass spectrometry.

**Results and Discussions**

Chemical derivatization of CAPE led to the identification of CM14, with considerably improved activity and systemic stability. Moreover, CM14 was able to overcome acquired ALK inhibitor resistance. Cell cycle analysis and RNA-seq after coincubation of ALCL cells with CM14 demonstrated arrest in the G2/M phase and altered expression of cell cycle-related genes. CM14 accumulated on a single spot per cell adjacent to the nucleus, which we identified as the centrosome, as shown by colocalization with γ-tubulin. To identify direct binding targets of CM14, we used an alkyne derivative to pull down CM14-interacting proteins using biotin-streptavidin enrichment and analyzing pulled-down

© 2024 The Authors. Molecular Oncology is published by John Wiley & Sons Ltd on behalf of Federation of European Biochemical Societies proteins via mass spectrometry. TUBGCP2, a centrosomal protein, was more than 20-fold enriched than in untreated cells, suggesting interference in centrosome´s function by CM14.

**Conclusion**

We identified a synthetic derivative of CAPE which reduces ALCL viability and overcomes ALK inhibitor resistance inducing apoptosis via impediment of the cell division machinery. These results may open novel treatment avenues in this and other aggressive lymphoma types.

### EACR2024-0916
### Development of p300-targeting PROTACs with enhanced selectivity and onset of degradation

*J. Cresser-Brown*[1], *G. Marsh*[1], *H. Maple*[1], *C. Ott*[2], *D. Bosnakovski*[2], *M. Kyba*[3]
[1]*Bio-Techne, Tocris Bioscience, Bristol, United Kingdom*
[2]*Harvard Medical School, Massachusetts General Hospital Cancer Center, Boston, United States*
[3]*University of Minnesota, Department of Pediatrics, Minneapolis, United States*

**Introduction**

CREB-binding protein (CBP, CREBBP, KAT3A) and E1A-binding protein (EP300, p300, KAT3B) are paralogous multi-domain proteins that act as chromatin regulators and transcriptional co-activators. They contain a histone acetyltransferase (HAT) domain that catalyzes the histone H3, lysine 27 acetylation (H3K27ac) mark at regulatory elements such as enhancers and promoters. Transcription factors associate with stretches of H3K27ac marks (known as 'super-enhancer' elements) and result in gene transcription that ultimately establishes cell identity and fate. They are implicated in cancer pathology, and small molecule inhibition of the bromodomain (BRD) or HAT domain of CBP/p300 are considered promising therapeutic strategies for a number of cancer types.

**Results and Discussions**

CBP and p300 are highly homologous but have distinct roles that have to date been hard to delineate, since small molecule inhibitors developed to date are unable to selectively target each protein independently. Additionally, small molecule inhibitors that target individual domains are unable to entirely abrogate the full functionality of CBP/p300. A bromodomain-recruiting dual CBP/p300 PROTAC Degrader 'dCBP1' was therefore recently developed to provide a chemical tool to explore the phenotypic consequences of CBP/p300 chemical knockdown. A further study demonstrated that it is possible to degrade p300 with some selectivity by converting a CBP/p300 dual HAT-domain inhibitor into a PROTAC, called 'JQAD1'.

**Conclusion**

We have used a different HAT-domain recruiting ligand to develop novel PROTACs that elicit proteasome-mediated degradation of p300 with significantly enhanced selectivity over CBP, compared with JQAD1. We additionally demonstrate a faster onset of degradation for lead PROTAC molecules and present data exploring the consequences of selective p300 degradation in CIC-DIX4 sarcoma.

### EACR2024-0922
### Therapeutic interest of an iron-based complex to improve treatments against pancreatic and triple negative breast cancers

*C. Neybecker*[1], *V. Besancenot*[1], *E. Brunner*[1], *G. Harlé*[1], *A. Monari*[2], *M. Bouche*[3], *P. Gros*[3], *N. Touche*[1], *S. Grandemange*[1]
[1]*Université de Lorraine and CNRS, Centre de Recherche en Automatique de Nancy UMR 7039, Vandoeuvre-lès-Nancy, France*
[2]*Université Paris Cité and CNRS, Itodys, Paris, France*
[3]*Université de Lorraine and CNRS, Laboratoire Lorrain de Chimie Moléculaire UMR 7053, Vandoeuvre-lès-Nancy, France*

**Introduction**

With 9,7 million people dying worldwide in 2022, cancer represents one of the leading causes of death, despite a better understanding of carcinogenesis and improved therapeutic strategies. In this context, our research field is focused in triple-negative breast cancers (TNBC) and pancreatic cancers, both of which are particularly aggressive and lack therapeutic solutions. Our current work is focused on highlighting the antitumor activity of new Iron-based complexes via their ability to bind DNA. Based on our preliminary data one complex called AIM3 has been selected.

**Material and Methods**

Antitumour effects were evaluated on non-cancerous cell lines MCF10A (Breast epithelial cells) and several cancerous models of which three cancerous cell lines MDA-MB-231 (TNBC cell line) T3M4 and BXPC-3 Pancreatic Ducal Adenocarcinoma Cell lines (PDAC lines). Proliferation and cytotoxic assay were performed. Moreover the coupling of AIM3 with common chemotherapeutic drugs used in the treatment of PDAC and TNBC (Gemcitabine® and Doxorubicine® respectively) were analyzed. Then, transcriptomic analyses were achieved to elucidate the action mechanism of these compounds.

**Results and Discussions**

Our results indicate that iron-based complexes, and particularly AIM3 compound, have a strong anti-proliferative effect on all cell lines tested. Interestingly, the treatment leads more or less quickly to an induction of apoptosis mainly in cancerous cells. Moreover, the antiproliferative effect observed was only reversible (when the treatment is removed after 72 h and replace by normal medium) in the non-cancerous cells. Combine treatments realized in MDA- MB-231 and T3M4 cell lines demonstrate that AIM3 potentiate the cytotoxic effect of doxorubicine and gemcitabine respectively. AIM3 effects are also observed in gemcitabine resistant T3M4 cells. Our molecular investigations highlighted the modulation of the transcript level of HIF1 related genes and genes involved in iron homeostasis regulation (NDRG1 and PPFIA4). To verify the involvement of these genes, we demonstrate that an addition of FeSO4 can reverse the observed AIM3 effects.

**Conclusion**

© 2024 The Authors. Molecular Oncology is published by John Wiley & Sons Ltd on behalf of Federation of European Biochemical Societies

Our results indicate that AIM3 could be a new antiproliferative option for aggressive cancers, but these effects have to be monitored in vivo. This antitumoral effect observed can be related to iron homeostasis as well as hypoxia regulation, a signaling that has to be clarified to clearly positioned AIM3 as a new therapeutic agent.

### EACR2024-0930
### Subverting the anticancer activity of colchicine derivatives from tubulin to PDK2 to create a new class of agent directed to mitochondria

*A. Dillon[1], M. Korpidou[1], S. Erhardt[1], A. Turnbull[1], D. Mincher[1]*
*[1]Edinburgh Napier University, School of Applied Sciences, Edinburgh, United Kingdom*

**Introduction**
Mitochondria have a crucial role in the regulation of cell death and the promotion of apoptosis. Mitochondria of tumour cells are viable targets due to an exploitable difference in the mitochondrial membrane potential (MMP, Ψm) between cancerous and healthy cells. Analysis of many types of cancer cells has revealed a depolarised membrane potential. Colchicine derivatives have potent antiproliferative activity but are too toxic and non-selective for clinical applications. Colchicines bind to tubulin proteins and mitochondrial membranes incorporate tubulin binding sites. Dichloroacetic acid (DCA) is a known inhibitor of mitochondrial PDK2 (pyruvate dehydrogenase kinase 2) but with low antitumour potency. Molecular hybridisation and fragment-based drug design were used to combine colchicine and DCA scaffolds into a single structure to create a library of novel agents.
**Material and Methods**
A series of novel C10-aminocolchicine-DCA conjugates was synthesised and characterised. The antiproliferative activity of compounds were determined in a panel of cancer cell lines, including the relatively resistant HCT-15 colon carcinoma cell line with high expression of P-glycoprotein efflux pumps. For selected compounds, AutoDock4 molecular modelling software determined in silico inhibitory activities for tubulin- or PDK2-binding at ATP-binding and allosteric sites using published crystal structures.
**Results and Discussions**
Judicial modification of the structure of hybrid aminocolchicine-DCA conjugates translated their biological targets from tubulin either to dual tubulin-PDK2 inhibition (MAK1) or selectively to PDK2 (A10). All tested compounds had antiproliferative activity against the HCT-15 cell line in low- or sub-μM concentrations. Cytotoxic potency in this cell line suggested they may be able to circumvent resistance mechanisms associated with P-glycoprotein expression. The acyclic conjugate MAK1 had an IC50 value of 0.43 μM ± 0.10 μM and cyclised conjugate A10, possessing an additional annealed ring, had an IC50 value of 0.16 μM ± 1.55 μM. Molecular modelling showed that A10 bound to PDK2 strongly in the ATP-binding site with mean binding energy and mean Ki values of -8.62 kcal/mol and 0.31 μM, respectively.
**Conclusion**
The creation of an entirely novel fused-tetracyclic (extended) colchicine ring system opens new chemical space for developing agents with anticancer potency and targeting selectively the intra-mitochondrial enzyme PDK2 overexpressed in many tumours. Compounds A10 and MAK1 merit preclinical development.

### EACR2024-0941
### Novel WRN Helicase Inhibitors Selectively Target Microsatellite Unstable Cancer Cells

*G. Picco[1], Y. Rao[2], A. Al Saedi[3], Y. Lee[4], C. Herranz-Ors[1], S. Walker[3], K. May[5], M. Coelho[3], J. Houseley[6], B. Schwartz[7], M. Garnett[3]*
*[1]Sanger Institute, Translation Cancer Genomics, Cambridge, UK*
*[2]GSK, Synthetic lethality, Upper Providence, PA- US 19426, USA*
*[3]Sanger Institute, Translational Cancer Genomics, Cambridge, UK*
*[4]GSK, Synthetic lethality, Upper Providence, USA*
*[5]Babraham Institute, Epigenetics, Cambridge, UK*
*[6]Babraham, Epigenetics, Cambridge, UK*
*[7]GSK, Synthetic lethality, Upper Providence, USA*

**Introduction**
Microsatellite-unstable (MSI) cancers require WRN helicase to resolve replication stress due to expanded DNA (TA)n-dinucleotide repeats. WRN is a promising synthetic lethal target for MSI tumours, and WRN inhibitors are in development.
**Material and Methods**
Here, we used CRISPR-Cas9 base editing to map WRN residues critical for MSI cells, validating the helicase domain as the primary drug target. Fragment-based screening led to the development of potent and highly selective WRN helicase covalent inhibitors.
**Results and Discussions**
These compounds selectively suppressed MSI model growth In vitro and In vivo by mimicking WRN loss, inducing DNA double-strand breaks at expanded TA-repeats and DNA damage. Assessment of biomarkers in preclinical models linked TA-repeat expansions and mismatch repair (MMR) alterations to compound activity. Efficacy was confirmed in immunotherapy-resistant organoids and patient-derived xenograft (PDX) models.
**Conclusion**
The discovery of potent, selective covalent WRN inhibitors provides proof of concept for synthetic-lethal targeting of WRN in MSI cancer and tools to dissect WRN biology.

### EACR2024-0980
### Discovery of a potential colorectal cancer inhibitor from imipramine pharmacophores

*A. Albaladejo González[1], A. Rodríguez-Martínez[2], D. Ribeiro[1], F. Postigo-Corrales[1], M.D. López-Abellán[1], M.A. Beltrán-Videla[1], A.B. Arroyo-Rodríguez[1], A.M. Hurtado-López[1], H. Pérez-Sánchez[2], P. Conesa-Zamora[1]*

© 2024 The Authors. Molecular Oncology is published by John Wiley & Sons Ltd on behalf of Federation of European Biochemical Societies

[1]Hospital General Universitario Santa Lucía, Department of Pathology, Cartagena, Spain
[2]Universidad Católica San Antonio de Murcia UCAM, Computer Engineering, Murcia, Spain

**Introduction**

Metastasis is the primary cause of cancer mortality and relies on carcinoma cells acquiring invasive capabilities through actin cytoskeleton rearrangement, leading to the formation of crucial cellular protrusions. Fascin, an essential protein in membrane protrusion, is often elevated in malignant tumors, correlating with poorer survival. Consequently, Imipramine, identified as a novel fascin inhibitor, exhibits anti-migratory and anti-invasive activity by binding to fascin, suggesting its potential as a therapeutic agent for fascin-positive cancers. It is necessary to search for antitumor drugs targeting fascin. In this study, in silico compound library screening was followed by in vitro assays with lymphoma, colorectal, and breast cancer cell lines. This evaluation focused on Imipramine and its analog, Z117, as new compound derivate from new screening compounds with binding affinity to fascin.

**Material and Methods**

The study screened 1368049 compounds using Ligand-Based Virtual Screening, selecting a compound from Imipramine for in vitro testing, Z117. It evaluated its anti-fascin, anti-migratory, and anti-invasive potential. Cell viability was assessed using an XTT assay in two cancer cell lines each, with high and low fascin expression. For colorectal cancer, HCT-116 and DLD-1 were used, for lymphoma L-428 and U-937, and for breast cancer MDA-MB-231 and MCF7. Wound healing assays in colorectal cancer cell lines examined the compounds' effect on cell migration, and confluence percentage was measured.

**Results and Discussions**

The analog of Imipramine, Z117, showed approximately half the IC50 value of Imipramine in colorectal cancer cell lines, suggesting improved efficacy. However, in breast cancer and lymphoma cell lines, Z117 did not significantly affect cell viability as its IC50 value was notably higher than Imipramine's. In cell migration assays, Imipramine significantly inhibited migration after 24 and 48 hours, whereas its Z117 did not show the same effect at tested concentrations. In summary, while Z117 influenced cell viability, it did not significantly affect cell migration.

**Conclusion**

Comparatively to the anti-migratory and viability effects of Imipramine in colorectal cancer cell lines, Z117 had an improved effect on cell viability regardless of fascin expression levels. At the same time, this compound had no evident impact on cell migration. Conversely, Z117 have not demonstrated a significant impact on cell viability in lymphoma and breast cancer cell lines.

### EACR2024-0990

### Pipeline synthesis and characterization of Aurora-A targeting chimeric degrader molecules for identification of lead compounds with in vivo activity

*L. Eing*[1], *E. Wegrzyniak*[2], *G. Valinciute*[3], *J. Mihalic*[2], *Y. Mossé*[4], *M.F. Roussel*[3], *C. Guiducci*[2], *G. Hansen*[2], *M. Eilers*[1], *E. Wolf*[5]
[1]Julius-Maximilians-University Würzburg, Department for Biochemistry and Molecular Biology, Würzburg, Germany
[2]Nurix Therapeutics- Inc., Nurix Therapeutics, San Francisco, United States
[3]St. Jude Children's Research Hospital, Department of Tumor Cell biology, Memphis, United States
[4]The Children's Hospital of Philadelphia, Division of Oncology and Center for Childhood Cancer Research, Philadelphia, United States
[5]Christian-Albrecht-University Kiel, Unit for Tumor Biochemistry, Kiel, Germany

**Introduction**

High expression of the *MYCN* oncogene is observed in particularly aggressive neuroblastomas. The mitotic kinase Aurora-A mediates MYCN stabilization by preventing its degradation, presumably during mitosis, thereby allowing oncogenic MYCN levels to persist throughout the cell cycle. Direct targeting of MYCN remains difficult due to the lack of targetable binding pockets for small molecules. Our goal was to develop chimeric degrader molecules targeting the oncogenic scaffolding function of Aurora-A and indirectly destabilize the MYCN oncogene to identify novel molecules with therapeutic potential for the treatment of pediatric cancer.

**Material and Methods**

Automated solid-phase synthesis was used to generate six series of in total > 1000 chimeric degrader molecules based on different Aurora-A ligands. To characterize compound potency and selectivity, we used an in vitro pipeline combining efficacy studies using luciferase-based high-throughput assays for Aurora-A degradation and Aurora-B degradation as an estimate for off-target effects in neuroblastoma cells. In addition, cells ectopically expressing Aurora-A were used in cell viability rescue screens to further validate the potency and selectivity of each compound. To study the effect on MYCN levels upon Aurora-A degradation, we used a luciferase-based in cellulo assay to perform kinetic measurements. Subsequently, several compounds were tested for pharmacokinetic behavior in murine xenograft models of neuroblastoma.

**Results and Discussions**

Systematic study of cellular degradation of Aurora-A by the compound library revealed that most tested Aurora-A ligands can drive potent degradation when linked to Cereblon-binding moieties. Strong Aurora-B degradation correlated with a low selectivity in cell viability rescue assays, demonstrating that Aurora-B degradation is a reliable measure for the selectivity of Aurora-A degraders. Highly selective compounds induced a slow, continuous depletion of MYCN, whereas non-selective degraders induced a rapid MYCN depletion within a few hours. Compounds from two series were orally bioavailable and resulted in rapid tumor regression in vivo.

**Conclusion**

Our automated synthesis platform, combined with a robust in vitro/in vivo pipeline, has enabled the generation and characterization of hundreds of chimeric degrader molecules and the identification of novel

© 2024 The Authors. Molecular Oncology is published by John Wiley & Sons Ltd on behalf of Federation of European Biochemical Societies compounds with improved anti-tumorigenic activity compared to known Aurora-A inhibitors.

*Disclosure statement: EW and ME are founders and shareholders of Tucana Biosciences.*

### EACR2024-1020
### Anticancer Activities of tin(IV) phosphinoyldithioformate complexes

*S. Sharma[1], M. Balogová[2], H.M. Ögmundsdóttir[2], K.K. Damodaran[1]*
*[1]University of Iceland, Department of Chemistry, Reykjavík, Iceland*
*[2]University of Iceland, Faculty of Medicine, Reykjavík, Iceland*

**Introduction**

Cisplatin and other platinum-based chemotherapeutic agents are extensively used for cancer treatment. However, their clinical application is hampered by significant side effects and drug resistance. Organotin(IV) organometallic complexes present a promising alternative due to their improved therapeutic profile and unique mode of action compared to existing platinum-based drugs. In this study, we synthesized mono- and bis-tin complexes using benzyl phosphinoyl-dithioformate ligands [$H\text{-}S_2CP(O)(CH_2Ph_2)_2$], and evaluated their anticancer activities against various cancer cell lines.

**Material and Methods**

Materials and methods are listed below:
(a) Synthesis: The tin complexes were synthesized by reacting ligand (having sulfur and phosphorus moieties) with an equimolar amount of $SnBr_2CH_3Ph$ and $SnPhCl_3$ (Scheme 1 b and c). The isolated product was characterized by standard analytical techniques and single-crystal X-ray diffraction was performed on the crystals to elucidate the structural information.
(b) Anti-cancer activities: Following manufactures protocol crystal violet assay is utilized for the determination of half maximum inhibitory concentration ($IC_{50}$). The mode of cell death (apoptosis and necrosis) investigated in cell lines via flow cytometry using the Annexin V and propidium iodide (PI) staining method. Analysis of cell cycle with flow cytometry performed to determine the changes in the cell cycle for each cell lines utilizing cisplatin as a reference drug.

**Results and Discussions**

Sn-DBPTF-1 displayed potential cytotoxic activity ($IC_{50}$ <10 µg mL−1) towards all the tested cancer cell lines but was markedly less active against the non-cancerous MCF-10 cell line. However, Sn-DBPTF-2 had limited cytotoxic effects. Sn-DBPTF-1 induced G2 arrest in cancer cell lines HCT116 + Chr.2 (MMR-deficient colon cancer) and HCT116 + Chr.3 (MMR-proficient colon cancer). Aspc-1 showed accumulation in the G1 phase, implying inhibition of DNA synthesis. Sn-DBPTF-1 enhanced apoptosis in a dose-dependent manner in all tested cancer cell lines except for OVCAR-3 (ovarian cancer). The apoptotic effect was observed in non-tumorigenic cell lines (MCF-10 and D492) was notably smaller.

**Conclusion**

Two tin(IV) complexes having mono and bis ligand were synthesized and fully characterize and showed excellent cytotoxic potential against a variety of cancer cell lines, but not active against non- malignant cell line. Basic mode of action showed tin complexes induced cell cycle arrest and apoptosis leading to cell death.

### EACR2024-1028
### Mesenchymal stem cells modified to secrete targeted therapeutics for cancer therapy

*S. Emir[1], İ. Bahhar[1], R. Kaşmer[1], M. Ekingen[1], O. Topcu[2], D. Uludağ[1], G. Baalbaki[1], H.T. Kocabey[1], N. Karakaş[1,3]*
*[1]İstanbul Medipol University, Research Institute for Health Sciences and Technologies (SABITA), Cancer Research Center, İstanbul, Turkiye*
*[2]İstanbul Medipol University, Graduate School for Health Sciences- Medical Biology and Genetics Graduate Program, İstanbul, Turkiye*
*[3]İstanbul Medipol University, International School of Medicine- Department of Medical Biology, İstanbul, Türkiye*

**Introduction**

Targeted therapeutics have been potential candidates for fightening against cancers. Stem cells are being used in preclinical studies as a carrier for targeted therapeutics due to their migration capacity towards the tumor sites even including microdeposits. Furthermore, stem cells can be collected individually and engineered to secrete multiple therapeutics continuously which can enable their clinical translation from bench side to bed side. In a previous pioneering study, human neural stem cells (hNSCs) secreting interleukin-13 fused p*seudomonas exotoxin; PE (IL13-PE)* showed therapeutic benefits via protein synthesis inhibition against the most malignant form of brain tumors, glioblastoma. Next, we reported that IL13-PE can be directed towards other tumors such as lung cancers. In this study, we aimed to establish a stem cell based delivery of IL13-PE for a variety of tumors using human mesenchymal stem cells (hMSCs), which are already in clinics against several diseases.

**Material and Methods**

To prevent endogenic toxin activity in stem cells, human mesenchymal stem cells (UE7T-13, RIKEN, JAPAN) were firstly engineered to be toxin resistant using mutant elongation factor-2 coding single stranded oligonucleotides and resistant cells were selected by subsequent purified toxin (5-1000ng/ml) treatments. To determine therapeutic efficacy of stem cell delivered targeted toxin IL13-PE, bioimagable cancer cell lines were cocultured with therapeutic hMSCs and the mixed tumor cells along with stem cells were injected subcutaneously into mice. Bioluminescence imaging assays were performed both in vitro and in vivo.

**Results and Discussions**

Tumor cells expressing cognate receptor (IL13Rα2) showed decreased cell viability when cocultured with therapeutic stem cells (hMSC-IL13-PE) and subcutaneous tumor mass was significantly diminished as compared to tumors with naked hMSCs. The clinical compability of hMSCs for both autologous and alleogenic transplantation may provide new aspects for their use as of toxin vehicles.

Molecular Oncology 18 (Suppl. 1) (2024) 1–495
© 2024 The Authors. Molecular Oncology is published by John Wiley & Sons Ltd on behalf of Federation of European Biochemical Societies

**Conclusion**
These preclinical studies will lead us to develop an onsite toxin delivery strategy by human mesenchymal stem cells against several cancers that will eventually be compatible with clinical trials.

### EACR2024-1030
### Exploring Atmospheric Plasma as a Potential Therapy for Breast Cancer: Insights into Cell Invasion and Cancer Stem Cells Modulation

*C. Almeida-Ferreira[1,2,3], F. Rodrigues[1,4], F. Duarte[1,5], R. Silva Teixeira[1,2,6], F. Caramelo[1,2,7], M.F. Botelho[1,2,7], M. Laranjo[1,2,7]*
*[1]Univ Coimbra, Coimbra Institute for Clinical and Biomedical Research iCBR area of Environment-Genetics and Oncobiology Research CIMAGO- Institute of Biophysics- Faculty of Medicine, Coimbra, Portugal*
*[2]Univ Coimbra, Center for Innovative Biomedicine and Biotechnology CIBB, Coimbra, Portugal*
*[3]Univ Coimbra, Faculty of Pharmacy, Coimbra, Portugal*
*[4]Univ Coimbra, Faculty of Medicine, Coimbra, Portugal*
*[5]Univ Coimbra, Faculty of Sciences and Technology, Coimbra, Portugal*
*[6]Hospital Center of Vila Nova de Gaia/Espinho- EPE, Department of Cardiology, Vila Nova de Gaia, Portugal*
*[7]Clinical Academic Center CAC, Coimbra, Coimbra, Portugal*

**Introduction**
The incidence of breast cancer (BC) in women has been increasing. Some patients present poor responses, and treatments are frequently associated with several adverse effects. Cancer stem cells (CSCs) play a critical role in tumor recurrence and metastasis due to their heightened self-renewal and invasive properties. Therefore, new treatment options are needed. Cold atmospheric plasma (CAP), a gas with an equal number of positive and negative particles, also known as the fourth state of matter, has been investigated as a promised anti-tumoral treatment. CAP mechanisms of action are complex and remain partially unclear. Our main goals were to investigate the invasion of BC cell lines and the modulation of vascular endothelial growth factor receptor (VEGFR) and vimentin in breast CSCs after CAP exposure.

**Material and Methods**
For the transwell invasion assay, two triple-negative (TN) cell lines (HCC1395 and HCC1806) were used and exposed to CAP for 60 or 120 sec. Breast CSCs were obtained from TN and MCF7 cell lines and exposed to treatment for 120 or 240 sec. Fluorescence microscopy was performed to evaluate the VEGFR and vimentin. All experiments were assessed 24 hours after CAP exposure.

**Results and Discussions**
The invasion of TN cells decreased after 60 sec of CAP exposure; however, the most significant reduction was observed after 120 sec on both cell lines. It was 49.67± 3.16%, p=0.0005 for HCC1395, and 36.46±2.64%, p=0.0002 for HCC1806 cells compared to the control group. For VEGFR and vimentin, our preliminary results suggest a tendency to increase the fluorescence levels of cell lines compared to control conditions.

**Conclusion**
Non-thermal plasma can reduce cell invasion on TN cells, mainly after 120 sec. CAP seems to modulate CSC phenotype, increasing vimentin fluorescence and being a key biomarker of epithelial-mesenchymal transition (EMT). These findings suggest that CAP may be a promising therapy for BC disease, encouraging new studies.

*Funding: FCT (Portuguese National Funding Agency for Science and Technology) supports the CIBB through the Strategic Projects UIDB/04539/2020 (doi.org/10.54499/UIDB/04539/2020) and UIDP/04539/2020 (doi.org/0.54499/UIDP/04539/2020) and the Associated Laboratory funding LA/P/0058/2020 (doi.org/10.54499/LA/P/0058/2020). Project CARBONCT, 2022.03596, supported this work. PTDC (doi.org/10.54499/2022.03596.PTDC) and PhD grant from FCT, 2022.12228.BD.*

### EACR2024-1050
### Exploring the Efficacy of Monastrol Derivatives as Potential Fascin Inhibitors in Cancer Therapy: A Comprehensive In vitro Evaluation

*F. Postigo-Corrales[1], A. Rodríguez-Martínez[2], A. Albaladejo-González[3], M.D. López-Abellán[4], D. Ribeiro[4], A.B. Arroyo-Rodríguez[4], A.M. Hurtado-López[5], G. Luengo Gil[3], H. Pérez-Sánchez[2], P. Conesa-Zamora[3]*
*[1]Hospital General Universitario Santa Lucía- Universidad Católica San Antonio de Murcia UCAM, Laboratorio de Diagnóstico Molecular- Servicios de Anatomía Patológica y Análisis Clínicos, Cartagena, Spain*
*[2]Universidad Católica de San Antonio de Murcia, Computer Engineering Department, Murcia, Spain*
*[3]Hospital Universitario Santa Lucía- Universidad Católica de San Antonio de Murcia, Laboratorio de Diagnóstico Molecular- Servicios de Anatomía Patológica y Análisis Clínicos, Cartagena, Spain*
*[4]Hospital Universitario Santa Lucía, Laboratorio de Diagnóstico Molecular- Servicios de Anatomía Patológica y Análisis Clínicos, Cartagena, Spain*
*[5]Hospital Universitario Santa Lucía- Universidad Católica de San Antonio de Murcia, Laboratorio de Diagnóstico Molecular- Servicios de Anatomía Patológica y Análisis Clínicos, Murcia, Spain*

**Introduction**
Metastasis is the primary cause of global cancer-related deaths. Fascin, an actin-bundling protein often over-expressed in aggressive cancers like triple-negative breast cancer (TNBC) and serrated adenocarcinoma (SAC), plays a crucial role in their invasive phenotype. Inhibiting fascin is crucial, therefore Monastrol was identified for its ability to bind to the mitotic kinesin Eg5, a protein crucial for microtubule rearrangement during cell division. By binding to Eg5, Monastrol inhibits its activity, disrupting the formation of the mitotic spindle and causing cell cycle arrest. Previous research has demonstrated the antimigratory and anti-invasive effects of Monastrol on colorectal tumor cells, suggesting a potential interaction with fascin, also involved in microtubule dynamics. This study screened Monastrol and three analogs from the Enamine library for viability impact on colorectal cancer, breast cancer, and lymphoma cell lines.

© 2024 The Authors. Molecular Oncology is published by John Wiley & Sons Ltd on behalf of Federation of European Biochemical Societies

**Material and Methods**
Firstly, the study involved the screening of a library containing 1.368.049 compounds using Ligand-Based Virtual Screening calculations. Finally, 3 compounds derived from Monastrol were selected and tested in vitro on various cell lines with varying levels of fascin expression, including HCT-116 and DLD-1 (colorectal), MDA-MB-231 and MCF7 (breast), and L-438 and U937 (lymphoma). The cells were seeded and treated for 72h with the drugs at different concentrations to subsequently calculate their minimum inhibitory concentration (IC50). Cell viability was assessed using either cell counting or the XTT kit (Biotium, Avantor).
**Results and Discussions**
The results showed that only the analog Z144 exhibited an IC50 lower or equal to that of Monastrol in both lymphoma cell lines, as well as in the MDA-MB-231 breast cancer line and the HCT-116 colon line, suggesting potentially greater effectiveness. In contrast, the derivatives Z625 and Z195, in general, did not display IC50 values lower than the original compound in any of the lines, except for Z625, which showed a similar IC50 to Monastrol in HCT-116, and Z195 in MDA-MB-231.
**Conclusion**
Cell lines that overexpressed fascin seemed to respond more effectively to the derivative Z144, as they reduced their IC50 compared to Monastrol. This suggested a potential new drug, and further studies on migration and invasion were recommended for a comprehensive evaluation.

### EACR2024-1064
### Strategic Synergy: PARPi and DNA-DA Combinations Reshaping Pancreatic Cancer Treatment

*E. Pereira[1,2,3], G. Campos[1], J. Marques[1], A.R. Gomes[1,2,3], A.M. Abrantes[1,2,3], P. Silva-Vaz[1,2,3,4], M.F. Botelho[1,2,3], A.S. Pires[1,2,3]*
*[1]Univ Coimbra, Coimbra Institute for Clinical and Biomedical Research iCBR area of Environment Genetics and Oncobiology CIMAGO- Institute of Biophysics- Faculty of Medicine, Coimbra, Portugal*
*[2]Univ Coimbra, Center for Innovative Biomedicine and Biotechnology CIBB, Coimbra, Portugal*
*[3]Clinical Academic Center of Coimbra CACC, Coimbra, Coimbra, Portugal*
*[4]Unidade Local de Saúde ULS de Coimbra, Centro Hospitalar e Universitário de Coimbra CHUC- General Surgery Department, Coimbra, Portugal*

**Introduction**
Pancreatic Cancer (PanC) ranks among the most lethal cancers globally, with a 5-year survival rate of only 5%, requiring novel and more effective treatment strategies. Targeting Poly(ADP-ribose) Polymerase (PARP) inhibition has emerged as a promising approach to hamper cancer cells' DNA repair mechanisms. Consequently, combining PARP inhibitors (PARPi) with DNA-damaging agents (DNA-DA) is proposed to escalate DNA damage, overwhelm repair mechanisms, thereby enhance treatment efficacy while reducing adverse effects. Our research group has been exploring synergistic therapeutic approaches, specifically the combination of PARPi with DNA-DA, aiming to optimize treatment outcomes and mitigate side effects in PanC. This study presents preliminary results on the chemosensitizing potential of a PARPi, Olaparib (OLA), when combined with different DNA-DA.
**Material and Methods**
We used OLA as the PARPi and either irinotecan (IRI) or oxaliplatin (OXA) as DNA-DA. The impact of these combinations was assessed on the MIA PaCa-2 and PANC-1 cell lines. Each DNA-DA was combined with OLA at a fixed non-cytotoxic concentration (1μM), and the SRB assay was used to evaluate chemosensitization at 24, 48, and 72h post-treatment. The chemosensitization effect was quantified using the sensitization enhancement ratio (SER).
**Results and Discussions**
We have previously demonstrated the synergistic effect of the OLA and IRI (OLA administered simultaneously or 24h after IRI), as well as OLA and OXA (OLA administered 24h after OXA) combinations at a fixed ratio based on each IC50. In this study, we present preliminary results indicating the OLA chemo-sensitization when combined with IRI or OXA. We observed that combining a fixed non-cytotoxic concentration (1μM) of OLA, which inhibits PARP, with IRI or OXA resulted in a concentration decrease of IRI and OXA (compared to both in monotherapy) with a significant SER (1–4) at 24, 48, and 72h after treatment in MIA PaCa-2 and PANC-1 cells.
**Conclusion**
Our study presents preliminary evidence of the chemosensitizing potential of OLA when combined with IRI or OXA, supporting further exploration of this combination strategy as a potential avenue for enhancing treatment efficacy and minimizing adverse effects in PanC therapy.
*Funding: CIBB strategic projects 10.54499/UIDB/04539/2020 and 10.54499/UIDP/04539/2020, and Associated Laboratory funding 10.54499/LA/P/0058/2020 from FCT. Scholarship grant from FCT and European Social Funding 2021.05543.BD.*

### EACR2024-1073
### Investigating integrin-linked kinase as a novel therapeutic vulnerability in glioblastoma

*M. Romano[1], R. Elliott[1], A. Loftus[1], J. Birch[2], N. Carragher[1]*
*[1]Institute of Genetics and Cancer- University of Edinburgh, Cancer Research UK Scotland Centre Edinburgh, Edinburgh, United Kingdom*
*[2]Wolfson Wohl Translational Cancer Research Centre, School of Cancer Sciences- University of Glasgow, Glasgow, United Kingdom*

**Introduction**
Glioblastoma (GBM) is the most common and lethal brain tumour in adults. GBM treatment entails surgery, chemo-, and radiotherapy, but patient survival remains poor and better treatment options are urgently needed. Integrin-linked kinase (ILK) is a cell-matrix adhesion protein overexpressed in several cancers and has previously been identified as a therapeutic target contributing to malignant features of GBM. As cancer cells rarely respond robustly to loss of function of a

© 2024 The Authors. Molecular Oncology is published by John Wiley & Sons Ltd on behalf of Federation of European Biochemical Societies single protein target and GBM stem cells in particular contribute to drug resistance and relapse, the aim of this project is to exploit ILK as a therapeutic vulnerability in GBM stem cells, identifying novel combination strategies with ILK-targeting compounds.

**Material and Methods**

Chemical-genetic screens were performed using two target-annotated small-molecule compound libraries, Library of Pharmacologically Active Compounds (LOPAC®1280; 1280 compounds) and Comprehensive anti-Cancer Chemical library (C3L; 789 compounds) at four concentrations (30 nM, 100 nM, 300 nM, 1 µM) on an isogenic paired model of ILK-expressing and ILK-null murine GBM stem cells. Cell-based screening was performed in 384-well plate format using the PrestoBlue™ reagent (Invitrogen™) and cell viability as the primary assay endpoint. Compounds which demonstrated increased sensitivity with ILK-deficiency were validated by means of dose-response curves on resupplied compound material. These validated hits were examined in combination with radiotherapy (CIX1 X-Ray cabinet).

**Results and Discussions**

We identified 13 hit compounds which enhance the biological effect of ILK loss and show promise as therapeutic agents to be used in combination with ILK-targeting compounds. These compounds have been validated to demonstrate therapeutic vulnerability of ILK depletion in a GBM stem cell model and their mechanism of action is under investigation at transcriptomic, post-translational pathway and phenotypic levels. Following validation, the compounds have been tested in combination with clinically-relevant doses of radiation, as this is the standard-of-care for patients with GBM.

**Conclusion**

We identified compounds that synergise with loss of ILK, which will be further validated in combination with radiotherapy. Validated compounds with suitable pharmacokinetic and safety profiles will be tested further in vivo to determine translational potential.

### EACR2024-1097

### Predicting clinical outcomes using an in vitro drug sensitivity test based on pharmacokinetic data. A colorectal cancer example

*A. Poloznikov*[1], *B.R. Britt*[1], *S. Rodin*[2], *K. Grinnemo*[2], *M. Woywod*[3]
[1]*Mimi-Q GmbH, Research and development, Potsdam, Germany*
[2]*Uppsala University, Cardio-Thoracic Translational Medicine CTTM Lab- Department of Surgical Sciences, Uppsala, Sweden*
[3]*Mimi-Q GmbH, Product development, Potsdam, Germany*

**Introduction**

The development of an in vitro assay to predict clinical outcomes is fundamental to both drug development and personalized medicine. Conventional in vitro methods poorly replicate the physiological pharmacokinetics of drugs. This hampers the translation of in vitro parameters into clinical estimations. The exposure time and drug concentrations presented in the published studies often exceed the clinically relevant values. The effect of the sequence of drug administration is also rarely addressed. We hypothesized that imitation of the pharmacokinetic profile of a drug or drug combination according to the treatment scheme could have a positive impact on the clinical validity of the in vitro results. In this study, we have analyzed how exposure time affects the sensitivity status of the cells. Based on available PK data we designed in vitro protocols to mimic clinically relevant exposure to standard-of-care colorectal cancer treatment and compared the in vitro results with the individual clinical response and clinical trial data.

**Material and Methods**

For data analysis we used published NCI data. In addition, we performed categorical clustering to derive drug clusters to which the tested cell lines preserved the response status across different incubation times. Primary colorectal cancer cell cultures (N=6) were profiled in chemosensitivity assays to evaluate $IC_{50}$ and $GI_{50}$ values. Alternatively, these cells along with HT-29, HCT 116, COLO 205, SW480 and SW620 cancer cell lines were treated according to the developed the mFOLFOX-6, CapOx and FOLFIRI in vitro protocols.

**Results and Discussions**

According to our data analysis we identified that even small changes in incubation time leads to significant changes in the order of cell line sensitivities. We were not able to identify any meaningful mechanism of action-related clusters. PK-based test demonstrated the best performance for classifying 'responders' and 'non-responders' in a small cohort of primary colorectal cancer cells among all the methods used. $IC_{50}$ and $GI_{50}$ metrics were not univocal for different drugs and were particularly inaccurate for 5-FU. Incubation with clinically reachable concentrations misclassified only one case. PK-based test results were also consistent with the historical clinical data on similarities of mFOLFOX-6 and CapOx schemes.

**Conclusion**

Our study results along with findings from other groups, suggest that the PK-based testing approach may have considerable potential in fields of drug development and personalized treatment.

### EACR2024-1108

### Depletion of PDGFRß+ stromal cells by a protein-based targeted nanotoxin reduces tumour growth and sensitizes cells to chemotherapy

*L. Alba-Castellon*[1,2,3], *E. Volta-Duran*[3,4], *U. Unzueta*[1,2,3], *A. Villaverde*[3,4], *E. Vazquez*[3,4], *R. Mangues*[1,2,3]
[1]*Institut de Recerca Sant Pau, Oncogenesis and Anti-tumor Drugs, Barcelona, Spain*
[2]*Josep Carreras Leukaemia Research Institute, Campus Sant Pau, Barcelona, Spain*
[3]*Centro de Investigación Biomédica en Red, Bioingeniería- Biomateriales y Nanomedicina CIBER-BBN, Barcelona, Spain*
[4]*Universitat Autònoma de Barcelona, Institut de Biotecnologia i de Biomedicina and Departament de*

© 2024 The Authors. Molecular Oncology is published by John Wiley & Sons Ltd on behalf of Federation of European Biochemical Societies

*Genètica i de Microbiologia, Bellaterra, Spain*
**Introduction**
There is an urgent need to identify novel therapeutic strategies aimed at addressing the metastatic process and overcoming drug resistance in patients with cancer. The tumour microenvironment (TME) plays a pivotal role in supporting cancer progression, with cancer-associated fibroblasts (CAFs) being a crucial component. CAFs remodel the extracellular matrix and release cytokines and growth factors, influencing both cancer cell behaviour and the tumour microenvironment. This study focused on creating a protein-based nanoparticle designed to target and eliminate protumorigenic CAFs, thereby promoting an anti-TME.
**Material and Methods**
Nanoparticles were produced in *E. coli* and purified by IMAC affinity chromatography. Internalization and specificity for PDGFRβ-expressing CAFs was assessed by confocal microscopy, flow cytometry and western blot. Cytotoxicity was evaluated by XTT assay kit. In order to evaluate nanoparticles and a nanotoxin's performance in vivo, we use a syngeneic subcutaneous mouse model of colorectal cancer and head and neck squamous cell carcinoma.
**Results and Discussions**
The self-assembly scaffold nanoparticle, PDGFD-GFP-H6, was selectively internalized into fibroblasts expressing PDGFRβ, including primary human CAFs, without compromising their viability. This internalization was dependent on receptor expression, as demonstrated by the impaired internalization in the presence of a competitor. Conversely, nanoparticles were not detected in PDGFRβ-negative epithelial tumour cells. When administered in vivo, PDGFD-GFP-H6 was distributed to the tumor stroma, but not to the kidney or liver. To target and eliminate PDGFRβ-positive CAFs, a bacterial cytotoxic domain was incorporated into the nanoparticles to form a targeted nanotoxin, PDGFD-NT-H6. This nanotoxin eliminate fibroblasts in vitro, while sparing cancer cells. Furthermore, in vivo administration effectively impaired tumor growth and induced changes in the histological characteristics of the TME, including necrotic areas, immune cell recruitment, and blood vessel density. Importantly, animals treated with this stroma-targeted nanotoxin exhibited increased sensitivity to chemotherapy.
**Conclusion**
Depletion of PDGFRβ-expressing CAFs by the targeted nanotoxin PDGFD-NT-H6 reduces tumor growth rate concomitant with histological changes which may contribute to a less aggressive tumor. These findings underscore the significance of considering the entire tumour ecosystem to advance cancer treatment.

### EACR2024-1146
### Exploring the role of KSR as a therapeutic target to overcome resistance to KRAS inhibition

*O. Brehey Lyakhovych[1], G. Paniagua[1], H. K C Jakob[1], S. García-Alonso[1], C. G. Lechuga[1], M. Drosten[1], M. Barbacid[1]*
*[1]Spanish National Cancer Research Center CNIO, Molecular Oncology, Madrid, Spain*
**Introduction**
KSR proteins have long been considered only as scaffold proteins required for optimal mitogen-activated protein kinase (MAPK) pathway signaling. However, recent evidence suggests that they play a more complex role within this pathway with profound implications for cancer therapy.
**Material and Methods**
To explore the activity of KSR in MAPK pathway activation, we generated different mutants of KSR1 and the RAF family. Using the BioID assay, we aimed to identify new protein interactors of KSR1. To assess the potential therapeutic efficacy of targeting KSR, we established Sotorasib-resistant human lung cancer cell lines with KRASG12C mutations. Moreover, we utilized CRISPR-Cas9 for the genetic elimination of KSR1 to reverse the resistance, validated through proliferation assays
**Results and Discussions**
Ectopic expression of KSR1 or KSR2 was sufficient to activate the MAPK pathway and to induce cell proliferation in the absence of RAS proteins. KSR1 requires dimerization with at least one member of the RAF family to stimulate proliferation, resulting in translocation of the heterodimerized RAF protein to the cell membrane. This activity also required efficient ATP binding. We further show that Sotorasib, KRASG12C inhibitor, is less effective when KSR1 expression levels are elevated in human cancer cell lines. In agreement with these results, when we silence KSR the response upon Sotorasib treatment improves considerably.
To get further insights into the mechanisms of KSR activity, we performed a BioID assay and identified known KSR interactors along with several subunits of the PP6 phosphatase never been described before that could play a key role in the modulation of KSR activity.
**Conclusion**
KSR induces RAS-independent proliferation by the activation of MAPK pathway and reduces the effectiveness of KRAS inhibition. Thus suggesting that increased levels of expression of KSR may make tumor cells less dependent on KRAS oncogenic signaling, providing an alternative strategy for targeting KRAS-driven tumors.

### EACR2024-1149
### Analyses of tumor gene expression profile implicate specific metabolic and signaling signatures in pancreatic cancer

*D.G. Conde[1], Z. Nwosu[1]*
*[1]Cornell University, Molecular Biology and Genetics, Ithaca, United States*
**Introduction**
Pancreatic ductal adenocarcinoma (PDAC) is one of the deadliest malignancies and is characterized by profound metabolic alterations. To facilitate PDAC drug target identification and biomarker discovery, we aimed to determine genes and pathways consistently underlying this disease.
**Material and Methods**
We analyzed differential gene expression in five published PDAC microarrays and defined a gene as

Molecular Oncology 18 (Suppl. 1) (2024) 1–495
© 2024 The Authors. Molecular Oncology is published by John Wiley & Sons Ltd on behalf of Federation of European Biochemical Societies

‘consistent’ if ‘upregulated’ or ‘downregulated’ in four of the five cohorts (adjusted P<0.05). We further analyzed the consistent genes in >20 additional datasets, including tumor microarrays, bulk tumor and single cell RNA sequencing data, gene expression data of normal tissues, and in multiple cell lines.

**Results and Discussions**

We found that cell cycle, extracellular matrix receptor interaction, focal adhesion, p53 are among the topmost upregulated pathways in PDAC. The most upregulated metabolic pathways included glycolysis, cholesterol metabolism, redox metabolism, and lipogenesis, whereas signaling alterations included PI3K-AKT, p53, TGF-β, Hippo, MAPK and HIF1A signaling.

**Conclusion**

Our study has pinpointed clusters of metabolic and signaling genes/pathways that underlie pancreatic cancer, thus paving the way for improved detection, mechanistic insights, and therapy.

### EACR2024-1152
### SRI-41302 in combination with Anti-PD-1 Antibody Induces a Memory Response in MC-38 Colon Adenocarcinoma

*R. Boohaker*[1], *O. Moukha-Chafiq*[2], *C. Augelli-Szafran*[3]
[1]*Southern Research, Oncology- Scientific Platforms, Birmingham, United States*
[2]*Southern Research, Chemistry- Scientific Platforms, Birmingham, United States*
[3]*Southern Research, Scientific Platforms, Birmingham, United States*

**Introduction**

Colorectal cancer has the fourth highest incidence rate and second highest mortality rate in the United States of America. While incidence rates have dropped since the 1980s due to earlier detection, about a quarter of newly-diagnosed cases have distant metastasizes, resulting in a 14.7% 5-year relative survival rate compared to a 90.6% 5-year survival for patients with localized disease. Tumor resection is the standard-of-care for localized disease, with combination chemotherapy/surgery therapy for metastatic cases typically involving fluorouracil-based chemotherapies. Immune checkpoint inhibitor (ICI) therapy, such as PD-1 inhibition, have found a niche benefit in mismatch repair deficient and microsatellite instability high (MSI-H/dMMR), where neoantigens induced by genomic mutations are prevalent in the tumor microenvironment. Unfortunately, microsatellite stable with proficient mismatch repair (MSS/pMMR) have failed to date to see any benefit to ICI therapy.This can be overcome with priming doses of nucleosides therapy.

**Material and Methods**

Herein we demonstrate how induction of apoptosis with nucleoside analogues generates novel antigens needed for ICI treatment to be effective in MSS/pMMR cancer. Nucleoside analogues Thiarabine and its protide analog SRI-41302induced apoptosis in MC-38 cells. Conditioned media from treated MC-38 cells induced an upregulation in activation/exhaustion markers on both T cytotoxic cells and T helper cells. In vivo, utilizing humanized PD1/PDL1 GEMM mice, monotherapy with either Thiarabine or SRI-41302 demonstrated early signs of exhaustion and recruitment of cytotoxic T cells to the tumor.

**Results and Discussions**

Combination ICI therapy with nucleoside analogues and Anti-PD-1 Antibody resulted in tumor reduction, lack of tumor regrowth, and prevented tumor engraftment at a secondary site, indicating a memory phenotype.

**Conclusion**

The mechanism of action of Thiarabine is poorly understood and while it closely resembles the function of Cytarabine, not much can be attributed to its potency of the compound past the metabolic changes to the compound itself. Based on previous FDA allowances for the use of the Keytruda checkpoint inhibitor in the presence of MSS or chronic MSI, regardless of PD1/PDL1 status, we devised a series of in vitro and in vivo experiments to determine if nucleosides could perturb an otherwise inert tumor environment such that an immune response would be mounted to induced DNA damage.

### EACR2024-1187
### Exploring the Cytotoxic Potential of Snake-Derived Peptides: Targeting Breast Cancer and Ovarian Clear Cell Carcinoma

*V. Gallego Londoño*[1,2], *M. Manrique-Moreno*[3], *G.A. Santa-González*[4], *G.J. Meersma*[1], *B.A. Wisman*[5], *S. de Jong*[1]
[1]*University Medical Center Groningen- UMCG, Medical Oncology, Groningen, The Netherlands*
[2]*University of Antioquia, Chemistry Institute, Medellin, Colombia*
[3]*University of Antioquia, Chemistry Institute, Medellín, Colombia*
[4]*Instituto Tecnológico Metropolitano, Facultad de Ciencias Exactas y Aplicadas, Medellin, Colombia*
[5]*University Medical Center Groningen- UMCG, Gynecologic Oncology, Groningen, The Netherlands*

**Introduction**

Bioactive peptides (BAPs) have emerged as an attractive alternative for cancer treatment, mainly due to their ability to target cancer cells through common membrane features, thereby avoiding the challenges posed by intratumoral heterogeneity. In this study, we explored the cytotoxic activity of peptides derived from snake venoms, specifically Crotalicidin (Ctn) from *Crotalus durissus*, and NA-CATH-ATRA-1-ATRA-1 from *Naja atra*. Notably, NA-CATH-ATRA-1-ATRA-1 (NA) has not been previously evaluated in tumor cells. Our investigation encompassed breast carcinoma (BC) and ovarian clear cell carcinoma (OCCC), both characterized by high recurrence rates and limited response to chemotherapy.

**Material and Methods**

Cytotoxicity was evaluated in 2D models using MTT viability assay for 24 hours in breast cancer (MDA-MB-231, MCF-7) and OCCC, (OVCA429, TOV21G) cell lines. Real-time analysis was performed using IncuCyte Imager fluorescence microscopy with propidium iodide (PI) staining over 48 hours. Cytotoxicity was also assessed on established spheroids growing as 3D cells in matrigel. After a 24 h treatment, images of Hoechst/Calcein/PI staining of spheroids post-treatment were

© 2024The Authors. Molecular Oncology is published by John Wiley & Sons Ltd on behalf of Federation of European Biochemical Societies captured and analyzed using EVOS 5000 microscopy. As a reference, we utilized the peptide LTX-315, a BAP currently analyzed in clinical trials. Peptide stability testing was conducted in media with 10% fetal calf serum (FCS).

**Results and Discussions**

Cytotoxicity assessment revealed that OCCC cells exhibited higher sensitivity to Ctn and NA compared to BC cells, with $IC_{50}$ values approximately 2.0 to 3.6 times lower. Furthermore, OCCC and BC cells demonstrated increased sensitivity to Ctn and NA compared to LTX-315. NA's cytotoxicity exceeds Ctn's across all cell lines. Notably, all peptides showed rapid membranolytic effect within an hour, with no observed apoptosis, indicated by the absence of caspase-3 and PARP activation. Peptides at concentrations of 25 µM-50 µM exhibited the ability to penetrate and diffuse through the extracellular matrix of spheroids, leading to a decrease in viability. The presence of FCS resulted in inactivation in time of all peptides tested.

**Conclusion**

Snake-derived peptides demonstrate potent cytotoxicity primarily through membranolytic mechanisms and show promising efficacy in targeting cancer cells, surpassing the effectiveness of the clinical-phase control peptide LTX-315. Future work aims to explore additional cell death mechanisms and enhance bioactive peptide efficacy as complementary cancer therapy.

### EACR2024-1190

### Design of new anticancer drugs by selectable intracellular organelle targeting: make for the mitochondria or turn left for the lysosome?

*D. Mincher[1], M. Kamel[2], O. Mohammed[2], J. MacCallum[2], A. Turnbull[2]*

*[1]Edinburgh Napier University- School of Applied Sciences, Centre for Biomedicine and Global Health, Edinburgh, United Kingdom*

*[2]Edinburgh Napier University, School of Applied Sciences, Edinburgh, United Kingdom*

**Introduction**

Defining structural features that can control and pinpoint the delivery of drugs to specific sub-cellular organelles of cancer cells offers the prospect of designing more potent and selective drugs that negate drug resistance, with less detrimental side-effects for the patient. Several vectors, including cationic peptides, are known to transport drug cargo efficiently to mitochondria. Utilizing a disubstituted anthraquinone template, this study combines the vector triphenylphosphonium (TPP) and the mitochondrial-active dichloroacetic acid (DCA) synergistically to achieve selective targeting of cancer cell mitochondria, by exploiting mitochondrial membrane potential differences.

**Material and Methods**

A library of novel spacer-linked, anthraquinone-TPP-DCA-conjugates (code-named MK) was synthesized and characterized. The antiproliferative activity of compounds were determined in a panel of cancer cell lines including PC3 prostate adenocarcinoma, MCF7 breast carcinoma, and HCT-15 colon carcinoma cell lines. Morphological changes, cell-based assays of induction of apoptosis and sub-cellular localisation of compounds defined by confocal microscopy were performed.

**Results and Discussions**

Notably, the conjugates were equally potent at low or sub micromolar concentrations in cell lines with low (MCF7), moderate (PC3) and high (HCT-15) levels of P-gp expression, suggesting circumvention of P-gp mediated drug efflux. In HCT-15, two lead conjugates (MK39 and MK44) had IC50 values of 3.2 µM ± 0.09 and 3.7 ± 0.02 µM respectively, induced cell shrinkage, nuclear contraction and plasma membrane blebbing (1 µM at 4h); whereas mitoxantrone (1 µM at 4h) had no effect. MK39 and MK44 localized exclusively in mitochondria and did not enter the nucleus, with the prospect of averting genotoxicity. Significantly, by addition of a polyamine spacer group between the anthraquinone and the TPP vector, we demonstrated that agents can be ‘diverted’ exclusively to lysosomes.

**Conclusion**

Novel compounds have been designed that can be targeted exclusively to cancer cell mitochondria. Furthermore, pharmacophores have been identified for insertion into a single scaffold to re-route the compounds to the lysosomes. The ability to exert control over sub-cellular destinations and trigger apoptosis has potential for new anticancer drug development.

### EACR2024-1256

### Ziziphus nummularia crude and fractionated extracts attenuate the malignant phenotype of human triple negative breast cancer cells

*A.A. Shaito[1], R. Abdallah[2], M. Sobeh[3], N. Sahri[3], J.E. Mesmar[2], E. Baydoun[2],*

*[1]Biomedical Research Center, Qatar University, Doha, Qatar*

*[2]Department of Biology, American University of Beirut, Beirut, Lebanon*

*[3]Agrobiosciences Program, College for Agriculture and Environmental Science, Mohammed VI Polytechnic University, BenGuerir, Morocco*

**Introduction**

Triple negative breast cancer (TNBC) is the most aggressive subtype of breast cancer and has limited therapeutic options. Effects of Ziziphus nummularia against TNBC have not been investigated yet.

**Material and Methods**

An ethanolic extract of Z. nummularia (ZNE) was prepared and chromatographically fractionated. Phytochemical composition of ZNE and its chromatographically isolated fraction (F6) was identified both qualitatively by spectrophotometric assays and analytically by HPLC-PDA-MS/MS. Effects of ZNE and F6 on the viability of several cancerous cell lines were tested by MTT assay. The anti-cancerous potential of ZNE and F6 was tested in vitro in MDA-MB-231 cells, a TNBC cell line. ZNE and F6 radical scavenging capacity was tested using DPPH assay, and their effects on reactive oxygen species (ROS) generation in vitro in cells by DCFDA staining. Propidium iodide-based FACS

© 2024 The Authors. Molecular Oncology is published by John Wiley & Sons Ltd on behalf of Federation of European Biochemical Societies analysis was used for cell cycle analysis. Scratch wound healing and trans-well migration chamber assays were used to assess MDA-MB-231 cell migration and invasion. Western blotting analysis was used to analyse changes in the levels of cell cycle, apoptosis and autophagy proteins.

**Results and Discussions**

Findings showed that ZNE and F6 reduced the viability of several cancerous cell lines including MDA-MB-231 cells. F6 decreased MDAMB-231 viability more than crude ZNE or F6. ZNE and F6 are rich in phytochemicals and HPLC-PDA-MS/MS analysis identified several metabolites that were previously reported to have anti-cancerous effects. Both ZNE and F6 showed potent antioxidant capacity in the DPPH assay, but promoted reactive oxygen species (ROS) production in MDA-MB-231 cells; an effect which was blunted by the antioxidant N-acetyl cysteine (NAC). NAC also blunted ZNE- and F6-induced reduction in TNBC cell viability. We also demonstrated that ZNE and F6 induced an arrest of the cell cycle, and triggered apoptosis- and autophagy-mediated cell death. ZNE and F6 inhibited metastasis-related cellular processes by modifying cell migration, invasion, and adhesion.

**Conclusion**

Collectively, our findings reveal that Z. nummularia is rich in metabolites that can attenuate the malignant phenotype of TNBC and may provide novel approaches for the discovery of new drug leads for treatment of TNBC and other cancers.

### EACR2024-1263
### Aurora Kinases as Novel Targets for the Treatment of Aggressive Meningiomas

M. Alaa Eddine[1], T. Yu[1], R. Warta[1], S. Krieg[1], A. Unterberg[1], C. Herold-Mende[1], G. Jungwirth[1]
[1]*Division of Experimental Neurosurgery- University of Heidelberg, Department of Neurosurgery, Heidelberg, Germany*

**Introduction**

Management of clinically aggressive Meningiomas (MGMs) remains challenging due to the absence of efficient systemic therapies. Aurora A (*AURKA*) and Aurora B (*AURKB*) are essential regulators of the mitotic spindle assembly and have been previously shown to be highly overexpressed in high-grade MGMs and linked to shorter progression free survival. Therefore, we investigated the efficacy of *AURKA*i alisertib, *AURKB*i barasertib, and the pan-Aurora kinase inhibitor danusertib as potential therapeutics for the treatment of MGMs.

**Material and Methods**

RNAi-mediated knockdown experiments were conducted in benign (Ben-Men-1) and anaplastic MGM cell lines (NCH93). mRNA levels of *AURKA* and *AURKB* were quantified by qPCR. Cell viability or cell proliferation were evaluated by crystal violet assay or manual counting. Migration assays were performed after treatment with all three compounds. Our top compound was administered to nude mice with subcutaneous xenograft transplants.

**Results and Discussions**

Analyses of siRNA mediated gene knockdown of *AURKA* and *AURKB* decreased cell growth up to 94.62% (P<.001), suggesting being essential for cell growth. Double knockdown of both Aurora kinases even decreased cell growth up to 99.38% (P<.001). Dose-response curves of small molecule inhibitors alisertib, barasertib and danusertib revealed $IC_{50}$ values of 53.99, 7.64, and 61.58 nmol/l, respectively. Treatment with each of these compounds decreased cell growth up to 94.05% (P<.001) and cell migration up to 71.2% (P<.001). Alisertib treatment of xenografted mice inhibited tumor volume by 74.01% (P<.001), while being well-tolerated.

**Conclusion**

*AURKA/B* seem to be essential for cellular growth and migration. All three inhibitors demonstrated strong anti-cancer effects in vitro. Alisertib demonstrated substantial in vivo anti-tumor effects while showing a high tolerability. These novel data suggest that targeting Aurora kinases could offer new therapeutic options for patients with aggressive MGMs in future years.

### Immunotherapy

### EACR2024-0030
### Deciphering the role of oxidative stress markers in the management of bladder cancer

M.K. Singh[1], V. Singh[2], A. Kumar[2]
[1]*King George Medical University, Urology, Lucknow, India*
[2]*King George's Medical University, Urology, Lucknow, India*

**Introduction**

The influence of environmental chemicals on oxidative stress markers is well-documented and has significant implications for the development of bladder cancer. However, knowledge concerning the role of oxidative stress parameters in the response of non-muscle invasive bladder cancer (NMIBC) patients to therapy remains neglected. In this study, we delve into the role of oxidative stress parameters in the context of BCG immunotherapy for NMIBC patients.

**Material and Methods**

Here, a cohort of 140 NMIBC patients who underwent BCG treatment were selected from the urology department of a tertiary care centre. These patients were categorized into two distinct groups based on their response to BCG therapy: 60 patients fell under the category of BCG-responsive (BCG-R), while 80 were classified as BCG-non-responsive (BCG-N). BCG-R patients displayed no signs of tumor recurrence or progression following one year of BCG immunotherapy, whereas BCG-N patients experienced tumor recurrence after 3 to 6-month cycles of BCG instillation, as confirmed through cystoscopy. Levels of oxidative stress markers- malondialdehyde (MDA), nitric oxide (NO), superoxide dismutase (SOD), thiol group and catalase (CAT) was measured in all studied participants. Additionally, study protocol was approved from the ethics committee of the institution and participants were enrolled after getting written informed consent from them

© 2024 The Authors. Molecular Oncology is published by John Wiley & Sons Ltd on behalf of Federation of European Biochemical Societies and/or their attendants. Data was analysed by using GraphPad Prism and SPSS to derive significant results.

**Results and Discussions**

This study uncovered marked differences in the levels of oxidative stress markers, including MDA (1.89±0.14 to 3.60±0.18 (p <0.012), NO (7.35±0.39 to 49.71± 2.15 (p<0.001)) and SOD (154.49±2.14 to 35.12 ± 2.66 (p<0.002), thiol (P < 0.032)) when comparing the BCG-N and BCG-R groups. Additionally, our data elucidated a significant correlation between oxidative stress markers and key NMIBC characteristics, particularly T1 high-grade tumours and tumours >2.5 cm in size. Notably, we did not detect any statistically significant disparities in CAT levels among the studied groups (0.61±0.02 to 0.66±0.01 (P= 0.44)).

**Conclusion**

Our findings significantly underscore the pivotal role of oxidative stress markers in the progression and recurrence of NMIBC linked to BCG-N. Consequently, effective management strategies for individuals with T1 high-grade tumors and tumors >2.5 cm are paramount for delivering antioxidant protection within the context of NMIBC.

### EACR2024-0048

### Lactobacillus metabolites relieves the reactive cutaneouscapillary endothelial proliferation (RCCEP) induced by Checkpoint inhibitors: a case report and review of literature

Y. Huang[1], M. Chen[2]

[1]*Yunnan Provincial Corps Hospital of Chinese People's Armed Police Forces, Outpatient Department, Kunming, China*

[2]*Chongqing University Cancer Hospital, Department of Clinical nutrition, Chongqing, China*

**Introduction**

Anti-programmed cell death protein 1 (PD-1) has been successfully used in nonsmall-cell lung cancer (NSCLC) treatment. However, it causes significant adverse effects (AEs), including reactive cutaneouscapillary endothelial proliferation (RCCEP). We reported a case of Lactobacillus metabolites relieves the RCCEP induced by Checkpoint inhibitors and review the current literature.

**Material and Methods**

Case report and literature review. The patient's medical record was reviewed for demographic and clinical data. For literature review, all case reports or other publications published in English literature were identified using PUBMED. We characterised the gut microbiota by 16s rRNA gene sequencing before and after the treatment of lactobacillus metabolites.

**Results and Discussions**

A 55-year-old female was diagnosed with drive gene negative stage IV left lung adenocarcinoma cT1N3M1. She treated with four cycles of Carilizumab combined with chemotherapy, as well as with the used of lactobacillus metabolites (Po. 2.5g Tid.). After 1 cycle treatment, RCCEP appeared, and the score is level 1. When she stop to use lactobacillus metabolites, RCCEP increase significantly, the score turned to level 3 (previously reported incidence of RCCEP level 3 only 0.6~0.8%). lactobacillus metabolites reshape the gut microbiota, with an increase in *Lachnospiraceae* and *Veillonella*, and a decrease in *Escherichia-Shigella*. Stop using lactobacillus metabolites, patient gut microbiota restored to the status of no used lactobacillus metabolites.

**Conclusion**

Lactobacillus metabolites, ameliorate the gut microbiota structure of the patients, may alleviate the symptoms of RCCEP induced by Checkpoint inhibitors.

### EACR2024-0102

### Enhancing Anti-Tumor Immunity through Talaporfin Sodium Photodynamic Therapy (TS-PDT) and Anti-Programmed Death 1 (Anti-PD-1) Antibody Combination Therapy

H. Kataoka[1], M. Sasaki[1], T. Mamoru[1]

[1]*Nagoya City University Graduate School of Medical Sciences, Departments of Gastroenterology and Metabolism, Nagoya, Japan*

**Introduction**

Photodynamic therapy (PDT) utilizing a photosensitizer exposed to specific light irradiation is a promising non-invasive anticancer treatment. In Japan, second-generation PDT employing talaporfin sodium (TS) and diode LASER is approved for lung cancer, brain tumor, and esophageal cancer post-chemoradiotherapy (CRT) failure. Radiotherapy at one site occasionally induces regression of non-irradiated metastatic tumors, known as the abscopal effect, observed also in PDT. PDT triggers direct cell death and augments tumor immunity via immunogenic cell death (ICD) by inducing damage-associated molecular patterns (DAMPs), though the precise mechanisms are unclear. This study aims to elucidate the anti-tumor effects of TS-PDT and its synergy with the immune checkpoint inhibitor anti-programmed death 1 (anti-PD-1) antibody.

**Material and Methods**

Cell death mechanisms induced by TS-PDT were investigated via cell viability assays and flow cytometric analysis for annexin V and activated caspase-3. Autophagy was assessed by LC3 and DAP green dye. DAMP induction by TS-PDT was measured using ELISA (HMGB1 and ATP) and immunofluorescence staining (calreticulin and HSP90) in vitro. A syngeneic mouse model with bilateral flank tumors established using MKN45 cells confirmed the abscopal effect enhancement.

**Results and Discussions**

TS-PDT induced apoptosis, necrosis, and autophagy-associated cell death in vitro and triggered DAMP release and/or expression. In vivo, TS-PDT with anti-PD-1 antibody combination significantly inhibited tumor growth in irradiated and non-irradiated tumors compared to single or no treatment. The migration of CD4+ and CD8+ T cells was significantly increased in the TS-PDT+ anti PD-1 antibody combination group with significantly increased Granzyme B mRNA expression in non-irradiated tumors.

**Conclusion**

TS-PDT enhances anti-tumor immunity via DAMP release and/or expression through ICD induction. The

© 2024 The Authors. Molecular Oncology is published by John Wiley & Sons Ltd on behalf of Federation of European Biochemical Societies combination therapy of TS-PDT and anti-PD-1 antibody holds promise for developing effective anti-tumor strategies.

### EACR2024-0129
### IL4 receptor targeted nab-paclitaxel enhanced M2 macrophage reprogramming via ROS-HMGB1-TLR4 activation and inhibited tumor growth and metastasis

*P.V. Sri Murugan*[1], *G. Gowri Rangaswamy*[1], *B. Lee*[1]
[1]*Kyungpook National University, Biochemistry and Cell Biology, Daegu, South Korea*

**Introduction**

Tumor-associated macrophages (TAMs) play a vital role in tumor progression and metastasis. Thus, M2-type macrophages in tumor tissues represent a promising target for cancer therapy. An albumin-bound paclitaxel nanoparticle called nab-paclitaxel (trade name Abraxane, hereafter Abx) has been used in treating tumor. Abx is internalized into macrophages via macropinocytosis and drives pro-inflammatory M1 macrophage polarization that demonstrates antitumor activity. However, the macrophage activation mechanism by Abx remains poorly explored.IL-4Receptor is expressed in M2 macrophages at higher levels than in M1 macrophage. We have identified the IL4RPep-1 peptide consisting of CRKRLDRNC amino acid sequence using phage display, that has been successfully used as a tumor-homing peptide. This study aimed to elucidate the selective delivery of Abx to M2-type macrophages by targeting IL4R and reprogramming them into an anti-tumoral M1-type. Furthermore, the pathways included in the reprogramming of M2 macrophages through the IL4R-targeted Abx were explored.

**Material and Methods**

Abx was conjugated with the IL4RPep-1 peptide using click chemistry (IL4R-Abx). Cellular internalization, cytotoxicity assays, macrophage reprogramming studies and signal pathways leading to macrophage reprograming was studied in vitro. The In vivo and Ex vivo bio distribution of the peptide conjugated drug was examined and the effect on tumor growth and metastasis by IL4R-Abx were evaluated in various tumor models. Variation in immune population were examined at the end of therapy.

**Results and Discussions**

IL4R-Abx was internalized into M2 macrophages more efficiently, which was primarily inhibited using an anti-IL4R antibody and a receptor-mediated endocytosis inhibitor compared with a macropinocytosis inhibitor. IL4R-Abx reprogrammed the M2-type macrophages into M1-like phenotype and increased reactive oxygen species (ROS) levels and extracellular release of high mobility group box 1 (HMGB1) in M2 macrophages at higher levels than Abx and Ctrl-Abx. IL4R-Abx accumulated at tumors, heightened immune-stimulatory cells while reducing immune-suppressing cells, and hampered tumor growth and metastasis in mice more efficiently than Abx and Ctrl-Abx.

**Conclusion**

These results indicate that IL4R-targeting allows enhancement of M2-macrophage shaping into M1-like phenotype by Abx through the ROS-HMGB1-TLR4 axis, improvement of antitumor immunity, and thereby inhibition of tumor growth and metastasis, presenting a new approach to cancer immunotherapy.

### EACR2024-0140
### Leveraging optimized drug combinations to enhance immunotherapy efficacy in colorectal carcinoma

*V. Benboubker*[1], *G. Mourad Ramzy*[1], *P. Nowak-Sliwinska*[1]
[1]*School of Pharmaceutical Sciences- University of Geneva, Molecular Pharmacology Group, Geneva, Switzerland*

**Introduction**

Colorectal carcinoma (CRC) remains a significant contributor to cancer-related mortality worldwide, with current therapeutic strategies hampered by toxicities and drug resistance, particularly in advanced stages. Immunotherapies, notably immune checkpoint blockade (ICB), show promise in CRC, yet only a minority of patients benefit from. These therapeutic challenges underscore the need for combined approaches. Here, we aim to assess the strong potential of low-dose optimized drug combinations (ODCs), previously optimized and validated in our lab, to enhance the anti-tumor immune response in CRC.

**Material and Methods**

We validated the efficacy of four distinct ODCs consisting of different tyrosine kinase inhibitors in an innovative organoid co-culture model consisting of AKP CRC organoids ($APC^{-/-}$, $Kras^{G12D}$, $TP53^{-/-}$), tumor associated endothelial cells, and splenocytes (ccAKP). We further explored the safety and efficacy of the ODC *in vivo* in immunocompetent mice model, grafted subcutaneously with AKP organoids. We evaluated organoids viability, tumor growth control, tumor immunogenicity enhancement, and modulation of the tumor microenvironment through flow cytometry (FACS) analysis and immunohistochemistry (IHC) studies. Furthermore, we investigated the endothelial cell anergy inhibition through in vitro tube formation assay and qPCR gene expression.

**Results and Discussions**

One ODC consisting of (regorafenib, vemurafenib, selumetinib and erlotinib) emerged as particularly promising, significantly suppressing ccAKP growth and viability by 90% compared to oxaliplatin at clinically used dose. In addition, FACS analysis showed an increased T cell infiltration and activation within ccAKP, which potentially sensitizes them to anti-PD1 therapy. In the syngeneic mouse AKP model, our ODC significantly reduced tumor size by 70% compared to vehicle control, with no observed toxicity. FACS analysis of the isolated tumors showed an increased T cell infiltration. Moreover, ODCs were found to upregulate endothelial adhesion molecules expression, reduce neovascularization, and normalize vessel formation, crucial for facilitating T cell infiltration.

**Conclusion**

© 2024The Authors. Molecular Oncology is published by John Wiley & Sons Ltd on behalf of Federation of European Biochemical Societies

Our study highlights the promise of Optimized Drug Combinations in enhancing the immune response against CRC, offering new avenues for combination strategies with immunotherapies. These findings hold significant implications for improving treatment outcomes in CRC patients and advancing CRC treatment.

### EACR2024-0145
### The potential use of CAR EV's as a therapeutic strategy for solid tumors

*E. Buderovsky[1,2,3], G. Horn[2,4], A. Globerson Levin[2,4], A. Aharon[1,3]*
*[1]Tel Aviv University, The Faculty of Medicine, Tel Aviv, Israel*
*[2]Department of R&D- TASMC, Immunology and Advanced CAR-T therapy, Tel Aviv, Israel*
*[3]Hematology Division- TASMC, Hematology Research Laboratory, Tel Aviv, Israel*
*[4]TASMC and Tel Aviv University, Dotan Center for Advanced Therapies, Tel Aviv, Israel*

**Introduction**
Solid tumors are a leading cause of cancer-related mortality in developed countries, primarily due to late-stage diagnoses resulting in poor prognosis. Chimeric Antigen Receptor (CAR) T-cells, engineered to recognize tumor-associated antigens, have shown success in hematological cancers. However, their effectiveness is limited in solid tumors due to challenges in infiltrating the tumor niche, possibly influenced by the immuno-suppressive tumor microenvironment. Immunotherapy based extracellular vesicles (EVs) may overcome some of these limitations. EVs, nano-metric membraned vesicles originating from various cells, express antigens/proteins and genetic molecules reflects their parental cells. EVs interact with target cells and transfer diverse cargo, to recipient cells. CAR EVs represent a novel approach combine the benefits of both EVs and CAR T cells. CAR EVs has the recognition of the CAR to the cancer cell, while the content has all that is needed to kill a cancer cell. This study aimed to explore the in-vitro and in-vivo mechanisms of action of CAR EVs against solid tumors, and their potential to facilitating solid tumor infiltration while minimizing side effects and toxicity.
**Material and Methods**
High-expression EGFR and CD276 cancer cell lines were carefully selected using Flow cytometry analysis. Four CAR retro constructs (anti-CD276.1/2 and anti-EGFR.1/2) were transduced into T cells, and their efficiency was assessed using cytotoxic assays. After stimulating cells with antigen coated beads (CD276 or EGFR), CAR EVs were isolated using the ultracentrifuge method. The size and protein content of the EVs were characterized, and their impact on cancer cells was subsequently evaluated.
**Results and Discussions**
Higher transduction were found in anti-CD276.1 and anti-EGFR.1. Cytotoxic abilities of these CAR-T cells were tested on three lung cancer cell lines, anti-CD276.1CAR and anti-EGFR.1CAR exhibit superior performance across different effector-to-target ratios and higher IFNγ secretion. Additionally, anti-CD276.1 and anti-EGFR.1 CAR EVs exhibit high cytotoxicity against lung cancer cells. These findings suggest the potential of these CAR-T cells and EVs as effective therapies for specific lung cancer subtypes.
**Conclusion**
Next, we will assess the CAR EV's potential in vivo, using a mouse model. We are confident that CAR EVs have the potential to revolutionize solid tumor therapy by enhanced and efficient delivery to the tumor niche, while maintaining a comparable killing effect to traditional CAR-T cells.

### EACR2024-0155
### A 3D ECM-embedded tumoroid platform for testing antibody drugs and engineered TCRs for immune oncology

*E. Danen[1], A. Liao[2]*
*[1]Leiden University, LACDR: Leiden Academic Center for Drug Research, Leiden, The Netherlands*
*[2]Leiden University, Leiden Academic Center for Drug Research, Leiden, The Netherlands*

**Introduction**
Clinical use of immune checkpoint inhibitors is established, but response is observed in fewer than 15% of the cases. Additional strategies have entered the clinic including bsAbs and adoptive T cell therapy. Success of such therapies for the treatment of solid tumors is still limited and optimal strategies need to be selected.
**Material and Methods**
We developed a screening platform for immune oncology using an assay based on automated image guided injection of tumoroids in multi-well plates preloaded with ECM. The identical x-y-z position of the ECM-embedded tumoroids in each well facilitates automated imaging to generate quantitative data for T cell recruitment to tumoroids and killing of tumoroids by T cells.
**Results and Discussions**
We applied this to screening a panel of CD3:Her2 bsAbs binding with different affinities to CD3 or Her2 or targeting different epitopes on Her2. Exposure to non-activated PBMC derived T cells shows an initial phase of random T cell movement throughout the ECM followed by a bsAb-dependent phase of active T cell recruitment to tumoroids (day 2-4) and a subsequent phase of tumoroid killing (day 4-6). We show that the wave of T cell recruitment following initial T cell-tumoroid contact involves chemotactic signaling. Decreased affinity at the Her2 or CD3 arm can be compensated for by increasing bsAb concentrations. However, we detect major differences in efficacy for different high affinity epitopes. I.e., of two bsAbs interacting with high affinity with distinct Her2 epitopes and each causing effective tumor cell killing in 2D co-culture, only one was able to trigger a wave of T cell recruitment and subsequent tumoroid killing in 3D. We applied the same setup to testing the efficacy of T cells expressing engineered TCRs aimed at application in adoptive T-cell therapy. Kinetics of experiments using these activated engineered T cells are considerably shorter (24 hours instead of ~6 days) but show a similar pattern of T cell recruitment and subsequent tumoroid killing. We demonstrate successful generation of quantitative data for T cell recruitment and tumoroid killing for engineered T cells targeting tumor

© 2024 The Authors. Molecular Oncology is published by John Wiley & Sons Ltd on behalf of Federation of European Biochemical Societies antigens expressed on TNBC and uveal melanoma tumoroids.

**Conclusion**

A new screening platform using 3D ECM-embedded tumoroids provides quantitative data for T cell recruitment to tumoroids and killing of tumoroids by T cells. This platform is used for selection of bsAbs and engineered TCRs and provides mechanistic insight into the mode of action of successful T cell engaging strategies.

### EACR2024-0172

### ADAR1 loss impairs tumor growth and extends survival in immunocompetent preclinical models of glioblastoma

*A. Alvarez-Prado[1], L. Rentsch[1], J. Joyce[1]*

*[1]Ludwig Institute for Cancer Research and University of Lausanne, Department of Oncology, Lausanne, Switzerland*

**Introduction**

Glioblastomas (GBs) represent the most frequent and lethal form of primary brain tumors, with a median survival of 14.6 months and 15,000 newly diagnosed patients per year in Europe and the US. Current therapies invariably fail, very likely due to the extreme genetic heterogeneity of glioblastomas and the presence of a highly immunosuppressive tumor microenvironment. In this study, we exploited an innate immunity checkpoint – RNA sensing – with the aim of simultaneously targeting cancer cells and their supporting microenvironment. Adenosine Deaminase Acting on RNA 1 (ADAR1) is a central component of the RNA sensing pathway. It edits endogenous self dsRNAs, which would otherwise be recognized as foreign and trigger an aberrant innate immune response. Sensing of foreign nucleic acids results in interferon production which leads to cell-growth arrest, inflammation and immune cell infiltration through the expression of interferon-stimulated genes (ISGs). ADAR1 has recently emerged as a promising immuno-oncology target, with evidence pointing towards ADAR1 loss representing a novel vulnerability of ISG-expressing cancer cells. Despite expressing ISGs, glioblastoma tumors have not been evaluated for sensitivity to ADAR1 inhibition to date in immunocompetent models.

**Material and Methods**

We combined genetic and pharmacologic approaches to inhibit ADAR1 in a collection of patient-derived GB cancer cell lines and developed several immuno-competent pre-clinical mouse models of glioblastoma allowing tumor-specific homozygous genetic deletion of *Adar1* in early stages of tumor development and inducible deletion in established tumors.

**Results and Discussions**

Our data revealed that: (i) genetically distinct human GB cancer cells are sensitive to genetic *ADAR1* down-regulation and ADAR1 chemical inhibition; (ii) homozygous deletion of *Adar1* in early stages of tumor development and in established tumors resuts in delayed tumor growth and prolonged survial in immuno-competent mouse models.

**Conclusion**

This study showed that genetic deletion of *Adar1* in different immunocompetent preclinical mouse models of glioblastoma leads to delayed tumor growth and extended survival, indicating new opportunities for the treatment of this deadly disease.

### EACR2024-0309

### Tumor-infiltrating immune cells and HLA expression as potential biomarkers predicting response to PD-1 inhibitor therapy in stage IV melanoma patients

*B. Hegyi[1], K.G. Csikó[1], T. Balatoni[2], A.R. Neumark[3], K. Bőcs[4], E. Tóth[5], G. Fröhlich[6], G. Liszkay[7], S. Ferrone[8], A. Ladányi[9]*

*[1]National Institute of Oncology, Department of Chest and Abdominal Tumors and Clinical Pharmacology, Budapest, Hungary*

*[2]National Institute of Oncology, Department of Oncodermatology, Budapest, Hungary*

*[3]Semmelweis University, Faculty of Medicine, Budapest, Hungary*

*[4]National Institute of Oncology, Department of Diagnostic Radiology, Budapest, Hungary*

*[5]National Institute of Oncology, Department of Surgical and Molecular Pathology, Budapest, Hungary*

*[6]National Institute of Oncology, Center of Radiotherapy, Budapest, Hungary*

*[7]National Institute of Oncology, Department of Oncodermatiolgy, Budapest, Hungary*

*[8]Massachusetts General Hospital- Harvard Medical School, Department of Surgery, Boston, United States*

*[9]National Institute of Oncology, Department of Experimental Pharmacology, National Tumor Biology Laboratory, Budapest, Hungary*

**Introduction**

Immune checkpoint inhibitors, especially PD-1 blocking agents are known to be effective in metastatic melanoma, however, a considerable proportion of patients fail to respond to therapy, necessitating the identification of predictive markers. Our previous studies proved the predictive role of HLA class I expression, and infiltration by several immune cell types in melanoma patients receiving ipilimumab therapy. The aim of the present study was to examine the associations of tumor cell HLA-I and -II expression, and immune cell infiltration with the response to PD-1 inhibitor therapy.

**Material and Methods**

Archived paraffin blocks of pretreatment surgical samples (70 lymph node and 42 skin/s.c. metastases) from stage IV melanoma patients receiving nivolumab (n=17) or pembrolizumab therapy (n=23) were selected. Using immunohistochemistry, we determined melanoma cell expression of HLA-I molecules (using 4 antibody clones with different specificities), HLA-II, as well as the intratumoral density of immune cells expressing the following markers: CD8, CD45R0, FOXP3, CD20, NKp46, CD103, CD134, CD137, PD-1, and PD-L1. The associations of the above parameters with treatment response were analyzed.

**Results and Discussions**

Of the 40 patients 28 exhibited complete or partial response. Among the responders the ratio of patients

© 2024The Authors. Molecular Oncology is published by John Wiley & Sons Ltd on behalf of Federation of European Biochemical Societies showing melanoma cell HLA-II expression was higher compared to non-responders (17/28 vs. 2/12, p=0.0158), and similar results were obtained in the case of 2 anti-HLA-I antibodies (HC10 and EMR8-5), with significantly less cases showing decreased expression in responders. A combined score of HLA-I and -II expression could also predict treatment response (p=0.0019). With the exception of CD137 and NKp46, the ratio of patients showing strong immune cell infiltration was higher in the responders compared to non-responders. The most significant differences were observed in the case of PD-1 (23/28 vs. 3/12, p=0.0010) and PD-L1 (19/28 vs. 1/12, p=0.0012). Interestingly, PD-L1 expression (≥1%) by tumor cells did not predict treatment response significantly, in contrast to its expression by immune cells.

**Conclusion**

Our findings corroborate previous results indicating the importance of immune cell infiltration and tumor cell HLA-II expression in the efficacy of PD-1 inhibitor treatment in a "real world" patient cohort and suggest the potential predictive role of HLA-I expression, for which controversial results were obtained in previous studies.
The work was supported by NKFIH grant ANN 128524.

### EACR2024-0368
### CHL-4, a Novel Small Molecule Induces Interferon Response via RIG-I/MAVS Pathway: Potential Combination Therapy with Immunotherapy

*E.H.C. Lee*[1], *A.P. Kumar*[1]
[1]*National University of Singapore, Department of Pharmacology, Singapore, Singapore*

**Introduction**

Triple negative breast cancer (TNBC) is a highly aggressive subtype of breast cancer with poor prognosis. Despite entering an era of targeted and immunotherapy, TNBC patients remain largely limited to conventional treatments of chemotherapy and surgery. Our group has identified a novel small molecular inhibitor, CHL-4, and established that its anti-tumour effects in vitro and in vivo function through Lyn kinase, a member of the Src family kinases. This current study further identifies and characterises a novel immunomodulatory role for CHL-4, emphasizing its potential as a novel treatment option for TNBC.

**Material and Methods**

Transcriptomic analysis of CHL-4 treated TNBC cell line MDA-MB-231 was carried out to identify upregulated gene sets upon treatment. qPCR, Western blot and ELISA was performed to validate and examine expression of key transcripts and proteins involved in the interferon (IFN) signalling pathway. Next, using knockout cell lines generated with CRISPR-Cas9 technology, the contribution of various upstream signalling components in the IFN pathway was evaluated. Development of an in vitro 3D vascularised TNBC model is also underway, as well as in vivo testing of CHL-4 in a mouse model.

**Results and Discussions**

Gene expression profiling revealed that CHL-4 treatment caused upregulation of inflammatory and IFN signalling pathways. We validated in a range of TNBC cell lines that gene and protein expression of Type I/III IFNs is induced upon CHL-4 treatment, which is attributed to increased phosphorylation of IRF3. Using MDA-MB-231 knockout cell lines, we found that the RIG-I/MAVS cytosolic RNA-sensing pathway is indispensable for IFN signalling, as IFN production is abrogated upon knockout of either RIG-I or MAVS. CHL-4 also induces IFN response in the mouse TNBC cell line 4T1, allowing for drug testing in vivo. We have also successfully optimised the formation of a 3D vascularised TNBC model which will be a useful platform for testing human tumours in a setting that closely mimics the tumour microenvironment.

**Conclusion**

Breast cancer is typically considered immunologically "cold". CHL-4 induced activation of IFN response in TNBC suggests that our inhibitor has potential to stimulate anti-tumour immunity, especially in combination with immunotherapy such as anti-PD-L1. Further characterisation of CHL-4's effects in vivo and in vitro, especially on immune cell function and activation will provide stronger understanding of its mechanism of action and better position it as a novel therapy for TNBC.

### EACR2024-0375
### Cytokine-induced killer lymphocytes engineered with chimeric antigen receptors for a novel immunotherapy hitting malignant pleural mesothelioma

*A.M. Lombardi*[1], *V. Leuci*[1,2], *A. Massa*[1], *C. Donini*[1], *A. Proment*[1], *L. Vitali*[1], *A. Merlini*[1], *G.V. Scagliotti*[1], *D. Sangiolo*[1], *E. Vigna*[1]
[1]*University of Turin, Oncology, Turin, Italy*
[2]*Candiolo Cancer Institute FPO-IRCCS, Oncology, Candiolo TO, Italy*

**Introduction**

Malignant pleural mesothelioma (MPM) originates from the mesothelial cells of the pleura. Even considering the improvements obtained with chemotherapy and immunotherapy with checkpoint inhibitors, the prognosis in advanced/metastatic stages of MPM remains dismal. Therefore, new effective therapeutic approaches are urgently needed. To address this issue, Chimeric Antigen Receptor (CAR)-based cellular immunotherapy has been recently explored. In most of the cases, the CAR target of choice was Mesothelin (MSLN). However, the variable MSLN expression levels across different MPMs prompted the evaluation of novel targets. MET receptor is one of the key oncogenes involved in the onset and progression of solid tumors. Thus, MET could represent a promising therapeutic target for MPM treatment.

**Material and Methods**

The experimental platform is based on Cytokine Induced Killer lymphocytes (CIKs) as immune effectors. CIKs are characterized by a T/NK phenotype, a high proliferation rate in vitro, and excellent trafficking to the tumor site, where they exert a NKG2D receptor-mediated/MHC-unrestricted killing activity. CAR-CIKs were generated by engineering CIK obtained from patients' PBMC with lentiviral vectors expressing a MET-CAR or a MSLN-CAR. The killing activity of the two different CAR-CIKs

© 2024 The Authors. Molecular Oncology is published by John Wiley & Sons Ltd on behalf of Federation of European Biochemical Societies has been evaluated by co-culturing CAR-CIKs and MPM cells, at different ratios.

**Results and Discussions**

We evaluated NKG2D ligands, MET, and MSLN surface expression by flow cytometry in a panel of MPM cell lines with different histological classifications. NKG2D ligands expression predicted a good CIK-killing activity in most of the models. MET was expressed by a very high percentage of all the analyzed cell lines. On the contrary, MSLN expression is more frequently restricted to a sub-fraction of the cell populations. In the MPM models with high MET density, the killing activity of MET-CAR-CIKs was potent, even at low effector/target ratios. In MPM models with low MET density, CIKs activity was CAR-independent. Moreover, MET-CAR-CIKs did not attack normal pleural mesothelial cells, envisaging the lack of on-target/off-tumor toxicity. The response to MSLN-CAR-CIK was dependent on the percentage of MSLN-positive cells present in each MPM model.

**Conclusion**

MET- and MLSN- CAR-CIK effectively killed MPM cells in vitro, depending on the density or frequency of the target, respectively. The use of the two CARs in combination could represent a promising strategy to enhance CAR-CIK efficacy in this challenging setting.

### EACR2024-0403
### Phenotypic plasticity and emerging epigenomic signatures of immuno-supression in SCLC

*O. Bartley[1], K. Brennan[1], V. Fife[1], S. Hood[1], K. Simpson[1], B. Fiona[2], E. Kilgour[1], C. Dive[1], D. Millrine[1]*
*[1]CRUK, National Biomarker Centre, Manchester, UK*
*[2]Christie NHS Foundation Trust, Department of Medical Oncology, Manchester, UK*

**Introduction**

Small cell lung cancer (SCLC) is an aggressive neuroendocrine (NE) tumour. Introduction of immune checkpoint blockade (ICB) has brought some durable response in a minority of patients, but the majority do not respond, despite a high mutational burden. Consistent with the aggressive trajectory of SCLC, patients often present with extensive stage disease where surgical resection is rarely feasible. We pioneered development of circulating tumour cell (CTC)-derived explant (CDX) models to circumvent the paucity of biopsies for research. CDX models recapitulate the inter-tumoral heterogeneity observed in SCLC and can be generated longitudinally, enabling disease progression monitoring. CDX models also replicate the intra-tumoral plasticity of SCLC with NE to non-NE (NNE) transition observed. We sought to investigate how epigenetic regulators dictate lineage plasticity and tumour-intrinsic immunogenicity during disease progression, specifically focusing on polycomb repressive complex 2 (PRC2).

**Material and Methods**

CTCs enriched from patient-derived blood drawn at either baseline or disease progression timepoints were implanted into immunocompromised mice to generate CDX models. Following ex vivo culture, NE and NNE cells were physically isolated from individual CDX tumours and subjected to FACS-based profiling. Parallel bulk RNAseq and ATACseq profiling were then applied to assess how PRC2 inhibition shapes IFN-γ sensitivity during disease progression.

**Results and Discussions**

NE cells exhibited transcriptional repression of HLA loci and transition to NNE cells coincided with epigenomic reprogramming, restored IFN-γ activity and antigen presentation. PRC2 components were highly enriched in NE cells. PRC2 inhibition augmented IFN-γ responses and induced up to 11-fold increases in surface MHC-I within NE cells derived from baseline CDX models (P=0.01). However, this immunomodulatory effect was significantly dampened in NE cells derived from matched progression models (P=0.03). Meanwhile, PD-L1 levels remained consistent. Biochemical analysis alongside parallel RNAseq and ATACseq profiling revealed PRC2 inhibition and IFN-γ initiate chromatin remodelling at disparate sites to synergistically modify gene expression linked to tumoral immunity.

**Conclusion**

Our findings elucidate mechanisms underlying the combined immunomodulatory activity of PRC2 inhibition and IFN-γ. We highlight the importance of inter- and intra-tumoral heterogeneity to the inflammatory output of this signalling paradigm.

### EACR2024-0422
### A Versatile Monoclonal Antibody for the Characterization of Cells Expressing scFv-Based Chimeric Antigen Receptors

*J. Hillger[1], A. Singh[2], R. Sinapius[3], S. L'Heureux[3], J. Fisher[3]*
*[1]Cell Signaling Technology, Technical Support, Leiden, The Netherlands*
*[2]Cell Signaling Technology, Immunology, Danvers, USA*
*[3]Cell Signaling Technology, Cytometry - Conjugation, Danvers, USA*

**Introduction**

Chimeric Antigen Receptor (CAR)-T cell therapy is a groundbreaking therapeutic modality that has proven to be successful in the treatment of multiple types of hematological malignancies. As this therapeutic platform continues to evolve, there is a need for reagents that can be leveraged to selectively interrogate multiple aspects of CAR-T cell biology. Here, we report on a highly versatile recombinant rabbit monoclonal antibody raised against the $Gly_4Ser$ peptide linker, which is a ubiquitous component of single-chain variable fragment (scFv)-based CARs. This antibody can be leveraged for flow cytometry-based CAR detection, bead-based CAR-T cell sorting, and activation of CAR transduced cells.

**Material and Methods**

The recombinant monoclonal antibody, E7O2V, was generated by immunizing rabbits with a synthetic peptide containing multiple, tandem repeats of the $Gly_4Ser$ core pentapeptide. E7O2V was directly conjugated to a panel of fluorophores and validated for specificity in a live cell flow cytometry assay using primary human CAR-T cells. To assess its utility bead-based sorting of CAR transduced cells, a biotinylated conjugate of E7O2V was used in combination with magnetic beads coated with streptavidin via a cleavable linker to immunoaffinity purify CAR transduced cells from a mixed population.

© 2024 The Authors. Molecular Oncology is published by John Wiley & Sons Ltd on behalf of Federation of European Biochemical Societies

Finally, a plate-bound antibody stimulation assay was leveraged to assess the ability of E7O2V to activate CAR transduced cells.

**Results and Discussions**

Live cell flow cytometric analysis revealed that E7O2V could detect surface expressed CARs and no specific staining was observed on non-transduced cells. Furthermore, flow cytometric analysis of bead-free, purified cells revealed that E7O2V could facilitate enrichment of CAR transduced cells to a high degree of purity. E7O2V was found to selectively activate CAR transduced cells as demonstrated by upregulation of cell surface activation markers, including CD69.

**Conclusion**

Exploiting the ubiquitous $Gly_4Ser$ linker of scFv-based CARs for antibody discovery, we identified a novel and highly versatile monoclonal antibody, E7O2V. This antibody can be leveraged in multiple assay formats to interrogate various attributes of CAR transduced cells, including CAR expression, CAR signaling, and molecular analysis of rare CAR-T subsets enabled by bead-based enrichment.

### EACR2024-0439
### ICOS immunotherapy – Towards understanding the mechanism of action

*K. Reshef[1], S. Amar[1], R. Sheinin[1], M. Monsonego[1], T. Weiss[1], E. Goldschmidt[1], A. Kaminitz[1], E. Shifrut[1,2,3], A. Madi[1]*

*[1]Tel Aviv university, Pathology, Tel Aviv, Israel*

*[2]Tel Aviv university, The School of Neurobiology-Biochemistry and Biophysics, Tel Aviv, Israel*

*[3]Tel Aviv Sourasky Medical Center, Dotan Center for Advanced Therapies, Tel Aviv, Israel*

**Introduction**

Patient response to checkpoint blockade therapy in the clinic isn't optimal, as a significant portion of patients doesn't benefit from it. T regulatory cells (Tregs) which under normal circumstances are responsible for maintaining immune homeostasis by repressing the immune system activity are enriched in the tumor microenvironment (TME). Unfortunately, it seems that in most cancers, CD8 T cells function poorly while the Tregs suppressive activity is reserved and sometimes elevated. Thus, to improve patients' response to immunotherapy, targeting Tregs and impairing their suppressive activity would potentially lead to improving overall anti-tumor immunity. It was previously shown that inducible T cell co-stimulatory (ICOS) is highly expressed on tumor-infiltering Tregs and therefore, might serve as a good target. Indeed, ICOS immunotherapy is already in clinical trials, but the mechanism of action is not yet clear.

**Material and Methods**

To deeper our understanding, we performed a single-cell RNA-seq of tumor-infiltrating immune cells enriched with Tregs from the TME 24 hours following a single injection of αICOS agonist to MC38 tumor-bearing mice.

**Results and Discussions**

Our preliminary results show that αICOS treatment leads to an elevation in the CD8 T cells population. In addition, we see a trend of decrease in the Tregs population and an overall less suppressive Treg phenotype reflected by the reduction of molecules like 41BB (CD137, TNFRS9) and Granzyme B. When performing a suppression assay using tumor-infiltrating Tregs from tumor-bearing mice, Tregs sorted from the αICOS agonist treated mice, showed an inferior suppression capability as compared to isotype control treated mice. Moreover, we validated this result using human Tregs. These results imply that although ICOS is considered a co-stimulatory receptor, under some conditions it can suppress Tregs activity. Interestingly, our single-cell RNA-seq data shows a reduction in CCAAT Enhancer Binding Protein Beta (Cebpb) following αICOS administration. Cebpb is known to dampen the inhibitory effect of IFNg on Tregs.

**Conclusion**

We thus hypothesize that αICOS agonist causes a reduction in Cebpb levels which synthesizes Tregs to IFNg, resulting in enhanced Treg fragility and reduced suppressive function.

### EACR2024-0442
### POSTER IN THE SPOTLIGHT
### CD8-targeted IL2 reinvigorates dysfunctional T cells to elicit anti-tumor immune responses in human cancer tissue

*P. Kaptein[1], N. Slingerland[1], C. Metoikidou[1], F. Prinz[2], M. Machuca-Ostos[1], T. Schumacher[3], Y. Yeung[4], K. Moynihan[5], I. Djuretic[6], D. Thommen[1]*

*[1]Netherlands Cancer Institute, Molecular Oncology & Immunology, Amsterdam, The Netherlands*

*[2]Medical University of Graz, Internal Medicine, Graz, Austria*

*[3]Oncode Institute, Molecular Oncology & Immunology, Amsterdam, The Netherlands*

*[4]Asher Biotherapeutics- Inc, Asher Biotherapeutics, South San Francisco, United States*

*[5]Asher Biotherapeutics- Inc.-, Asher Biotherapeutics, South San Francisco, United States*

*[6]Asher Biotherapeutics- Inc., Asher Biotherapeutics, South San Francisco, United States*

**Introduction**

Cancer patients can experience profound responses to immune checkpoint blockade (ICB), however the majority does not therapeutically benefit from this treatment. Thus, it becomes crucial to explore alternative strategies that effectively activate anti-tumor immunity. Interestingly, emerging evidence suggests that the PD-1/PD-L1 axis is not the sole impediment for activating T cells within tumors. This observation has prompted us to investigate alternative stimuli capable of inducing anti-tumor immune responses.

**Material and Methods**

To investigate the therapeutic reactivation of T cells in their original tissue context, we used the patient-derived tumor fragment (PDTF) platform developed by our lab. Previous work has demonstrated that PDTFs display immune responses to ex vivo PD-1 blockade replicating the clinical response of the same patient. To modulate antitumor immunity in a controlled manner, we here employed a novel interleukin-2 variant cis-targeted to CD8 T cells (CD8-IL2), which allows to specifically activate this subset.

Molecular Oncology 18 (Suppl. 1) (2024) 1–495

© 2024 The Authors. Molecular Oncology is published by John Wiley & Sons Ltd on behalf of Federation of European Biochemical Societies

**Results and Discussions**

Upon treating PDTFs from different cancer types with CD8-IL2, we observed the broad induction of proliferation and activation markers in tumor-resident CD8 T cells across samples. A proinflammatory cytokine and chemokine response was however only detectable in a fraction of tumors. Perturbation studies revealed that TCR signaling is imperative for a productive immune response following CD8-IL2 treatment. To identify the underlying mechanisms, we performed single-cell RNA/TCR-seq of PDTFs. This revealed that CD8-IL2 broadly induced enhanced effector capacity in intratumoral CD8 T cells, thereby specifically enabling reinvigoration of the dysfunctional T cell pool to elicit potent immune activity. Notably, the revival of dysfunctional T cells to mediate effector activity by CD8-IL2 depended on simultaneous antigen recognition and surpassed both quantitatively and qualitatively the outcomes achieved by PD-1 blockade. Finally, CD8-IL2 demonstrated the ability to functionally reinvigorate T cells in tumors resistant to anti-PD-1, highlighting its potential as a novel treatment strategy for cancer patients.

**Conclusion**

Collectively, our results align with a model in which IL2 can 'arm' T cells with effector capacity, preparing them to undergo full functional reactivation upon encountering their target antigen. This mechanism enables these T cells to overcome resistance to ICB, positioning CD8-IL2 as a promising treatment strategy for cancer patients.

### EACR2024-0466
### Effect Monitoring and Targeted Treatment Strategies in Advanced Gastric Cancer

N. Guo[1]

[1]*Chongqing University Three Gorges Hospital, Department of gastrointestinal surgery, Chongqing, China*

**Introduction**

The purpose of this study is to assess recent research advances and monitoring methods in the treatment of advanced gastric cancer. The focus is on molecular targeted therapy, immunotherapy, and precision medicine, aiming to provide updated treatment strategies and monitoring techniques for clinical practice.

**Material and Methods**

Molecular Targeted Therapy: Through literature reviews and analysis of clinical trial data, researchers systematically investigate key molecular targets in the gastric cancer mechanism, particularly EGFR and VEGFR. Novel molecular targeted drugs, such as cetuximab and afatinib, are employed for treatment, and their efficacy and tolerability are evaluated. Immunotherapy: Utilizing extensive clinical trial data, researchers conduct in-depth analyses of the role of anti-PD-1/PD-L1 inhibitors in the treatment of advanced gastric cancer. The modulation of patients' immune systems and treatment tolerability are explored to better understand the advantages and limitations of immunotherapy. Precision Medicine: Employing genomics and transcriptomics technologies, researchers conduct individualized analyses of patients with advanced gastric cancer to identify tumor characteristics. Special attention is given to patients with HER2 overexpression. Effect Monitoring: Integrating the development of imaging technology, new techniques such as radionuclide imaging and magnetic resonance imaging are employed for comprehensive patient monitoring.

**Results and Discussions**

Molecular targeted therapy demonstrates significant efficacy on EGFR and VEGFR targets, offering new treatment options for gastric cancer patients. Immunotherapy, through anti-PD-1/PD-L1 inhibitors, substantially improves patient survival rates and enhances overall quality of life. Precision medicine's individualized treatment strategies are successful, particularly within the HER2 overexpression subgroup. The application of imaging technology and liquid biomarkers provides more accurate and comprehensive means for monitoring treatment effects.

**Conclusion**

Recent research indicates substantial progress in molecular targeted therapy, immunotherapy, and precision medicine for advanced gastric cancer. These treatment approaches not only enhance efficacy but also offer more individualized and precise treatment choices for patients.

### EACR2024-0481
### A T-cell monitoring approach using a single-domain antibody directed against the pan-T-cell marker CD2 as potential immunotherapy tracer

S. Bissenberger[1], A. Krackhardt[1], G. Dario[1], C. D'Alessandria[2], L. Russelli[2], W. Weber[2]

[1]*Klinikum rechts der Isar- Technische Universität München, Klinik und Poliklinik für Innere Medizin III, Munich, Germany*

[2]*Klinikum rechts der Isar- Technische Universität München, Klinik und Poliklinik für Nuklearmedizin, Munich, Germany*

**Introduction**

Cancer research is focusing on the development of immunotherapies tailored for various tumor types, offering the potential for effective and targeted treatment modalities. However, a critical gap exists in the absence of adequate imaging techniques to comprehensively monitor treatment responses. Previous research employing F(ab')$_2$ fragments against CD2 has suggested the target's potential. Nevertheless, the advent of single-domain antibodies (sdAb) emerges as a promising avenue, given their compact size and advantageous attributes for clinical translation.

**Material and Methods**

Following the production in E.coli, the purity of sdAbs underwent validation through SDS-page and HPLC. Target specificity was evaluated by FACS using T cells, antigen-transduced tumor cells, and a CD2-knockout model. Potential impacts on T-cell functionality post-binding were evaluated by investigating tumor cell killing and cytokine secretion after coculturing tumor cells with T cells by ELISA. In a mouse model based on an adoptive T-cell transfer approach utilizing the same effector and target cells, potential in vivo effects on T-

© 2024 The Authors. Molecular Oncology is published by John Wiley & Sons Ltd on behalf of Federation of European Biochemical Societies cell functionality were conclusively ruled out, demonstrated by tumor rejection after T-cell and antibody injection. ImmunoPET proof of concept was established by employing the sdAb labeled with $Ga^{68}$ in a setup similar to the rejection study, followed by PET-MRI imaging.

**Results and Discussions**

Specific binding to human T cells with no significant functional impairment was observed in in-vitro studies. In the subsequent adoptive T-cell transfer model, our sdAb demonstrated no compromise in rejection capacity. Furthermore, specific accumulation of the labeled antibody was evident in a preliminary imaging experiment. Future experiments will delve into diverse immunotherapy approaches, correlating detailed PET images with ex vivo analysis to pinpoint patterns of heightened T-cell density within tumors, paving the way for the development of an imaging-based biomarker.

**Conclusion**

The presented sdAb facilitates straightforward and specific tracking of in vivo T-cell activity without compromising their ability to reject tumors. This data introduces the prospect of basing decisions regarding immunotherapies on prospective biomarkers post further development.

### EACR2024-0517
### Breaking Chemotherapy Resistance in Gastric Adenocarcinoma: Immunogenic Cell Death Induction by Carbonic Anhydrase IX Targeting

*E. Andreucci[1], A. Biagioni[1], S. Peri[2], G. Versienti[1], F. Cianchi[2], C.T. Supuran[3], L. Calorini[1], L. Magnelli[1], L. Papucci[1]*

*[1]University of Florence, Department of Experimental and Clinical Biomedical Sciences "Mario Serio", Florence, Italy*

*[2]University of Florence, Department of Experimental and Clinical Medicine, Florence, Italy*

*[3]University of Florence, Department of NEUROFARBA, Florence, Italy*

**Introduction**

Gastric cancer (GC) is the fifth most frequently diagnosed malignancy and the fourth leading cause of cancer-related death worldwide. When surgery is not pursuable, multimodal perioperative chemotherapy (pCT) is used to improve patients' OS, but the progressive gain of chemoresistance inevitably limits therapies. We recently identified carbonic anhydrase IX (CAIX) as a promising target in GC. Its expression correlates with pCT resistance in GC patients and its inhibition through SLC-0111 improves therapy response even in resistant cells. The ongoing study aims to investigate the mechanisms of SLC-0111-induced cytotoxicity and possible induction of immunogenic cell death in GC that may prompt an anti-cancer immune response.

**Material and Methods**

The CAIX inhibitor SLC-0111 and the standard European pCT FLOT (*i.e.* Leucovorin, 5-Fluouracil, Docetaxel, and Oxaliplatin) were used as single and combined treatments on wild-type (WT) and chemically-induced FLOT-resistant (FLOTr) GC cell lines. Optimal doses and timing of treatments to induce GC cell death were chosen based on viability analyses. Cancer cell death characterization was performed by flow cytometer and western blot analyses to identify key pathways and regulators involved in SLC-0111/FLOT-induced cytotoxicity. The expression/release by GC cells of DAMPs like Annexin A1 (ANXA1), Calreticulin (CALR), High Mobility Group Box 1 (HMGB1), extracellular ATP, and cytosolic dsDNA was evaluated in flow cytometry, ELISA, luminometry and immunofluorescence following the SLC-0111/FLOT treatment.

**Results and Discussions**

The SLC-0111/FLOT treatment triggers apoptotic and non-apoptotic cell deaths known to be endowed with immunogenic potential. In particular, the increased expression of Glutathione Peroxidase 4 (GPX4), reduced Nuclear Factor Erythroid 2–Related Factor 2 (NRF2) and augmented Divalent Metal Transporter 1 (DMT1) accompanied by high ROS production and lipid peroxidation suggests that SLC-0111-FLOT treatment induces ferroptosis, one of the immunogenic cell deaths to date identified. DAMPs analysis revealed increased levels of cytoplasmic dsDNA, expression of CALR and ANXA1, and increased release of HMGB1 and ATP by GC cells treated with SLC-0111/FLOT compared to control.

**Conclusion**

Overall, our data suggest that the SLC-0111/FLOT combination therapy not only strengthens therapy response and re-sensitizes resistant GC cells to therapy, but could also trigger immunogenic cell death that should prompt an anti-cancer immune response against tumor advancement.

### EACR2024-0546
### A chimeric Human/Dog vaccine against CSPG4 protein reveals potential therapeutic effects in comparative models of melanoma and osteosarcoma

*L. Tarone[1], D. Giacobino[2], L. Parisi[2], L. Maniscalco[2], S. Iussich[2], C. Montana[1], E. Morello[2], P. Buracco[2], F. Riccardo[1], F. Cavallo[1]*

*[1]University of Turin, Department of Molecular Biotechnology and Health Sciences, Turin, Italy*

*[2]University of Turin, Department of Veterinary Sciences, Grugliasco, Italy*

**Introduction**

Malignant melanoma (MM) and osteosarcoma (OSA) pose significant challenges in treatment due to poor efficacy of standard therapies against recurrences and metastasis, along with severe side effects and resistance mechanisms. Immunization exploiting plasmid DNA encoding hybrid tumor antigens to break tolerance, emerges as a promising strategy to stimulate the patient's own immune system, eliciting a specific and durable anti-tumor immune response. The Chondroitin Sulfate Proteoglycan (CSPG)4 arises as an ideal immuno-therapeutic target, given its high expression in MM and OSA and a key oncogenic role. Thanks to the similarities between human and canine MM and OSA, and the high homology between the corresponding CSPG4 sequences,

Molecular Oncology 18 (Suppl. 1) (2024) 1–495

© 2024 The Authors. Molecular Oncology is published by John Wiley & Sons Ltd on behalf of Federation of European Biochemical Societies we availed of dogs with spontaneous tumors, other than murine models, to assess the efficacy of a chimeric anti-CSPG4 DNA vaccination.

**Material and Methods**

Since the CSPG4 is a non-mutated self-antigen, and as such it is poorly immunogenic, we generated a hybrid plasmid derived partly from the human (Hu) and partly from the dog (Do)-CSPG4 (HuDo-CSPG4), to overcome host's unresponsiveness. We evaluated the safety, immunogenicity and anti-tumor potential of the chimeric HuDo-CSPG4 DNA vaccine, combined with electroporation, in comparative systems in vivo, including mice challenged with transplantable tumors and canine patients with spontaneous CSPG4$^{+}$ MM and OSA. We conducted human-surrogate cytotoxic assays using dendritic cells derived from healthy donors to stimulate autologous lymphocytes in vitro

**Results and Discussions**

HuDo-CSPG4 electrovaccination was immunogenic and exhibited anti-tumor potential in both MM and OSA mouse models. Adjuvant HuDo-CSPG4 proved to be safe in MM- and OSA-affected dogs, eliciting both humoral and cellular immunity. Clinically, the vaccination resulted effective as immunized dogs displayed prolonged overall survival compared to those receiving standard treatments alone. Furthermore, HuDo-CSPG4 vaccine-induced antibodies exerted a mechanistic effect, impairing functionality in CSPG4$^{+}$ MM and OSA cells. HuDo-CSPG4 induced a cytotoxic response in a human-surrogate setting in vitro.

**Conclusion**

HuDo-CSPG4 vaccination arises as an appealing strategy to induce a specific anti-tumor immunity, offering a strong rationale for its application in effectively treating CSPG4$^{+}$ MM and OSA patients. On this basis, we are advancing towards combinatorial approaches to overcome resistance and target tumor cells from multiple angles, aiming for a durable clinical response.

### EACR2024-0553
### Exploring the potential of Cathepsin V inhibition in glioblastoma- effects on cancer cell proliferation and NK cell cytotoxicity

*E. Senjor[1,2], A. Mitrović[1,2], M. Jukič[2], M. Proj[2], S. Gobec[2], J. Kos[1,2], M. Perišić Nanut[1]*
*[1]Jožef Stefan Institute, Department of Biotechnology, Ljubljana, Slovenia*
*[2]University of Ljubljana, Faculty of Pharmacy, Ljubljana, Slovenia*

**Introduction**

Cathepsin V (CTSL2), a lysosomal cysteine peptidase, exhibits distinct tissue distribution, substrate specificity, and functions. Elevated levels of CTSL2 have been linked to various pathological processes, including cancer. Notably, its expression correlates with the dysregulation of cell cycle and growth regulatory genes in cancer, suggesting its involvement in tumor progression. Recent evidence shows that CTSL2 expression correlates with poorer prognosis across multiple cancer types. Natural killer (NK), cells pivotal in the cancer immune surveillance, employ the granzyme-perforin pathway for eliminating cancer stem cells, a mechanism regulated by proteolytic activation mediated by cathepsins C, H, and L. However, their cytotoxic function is susceptible to immunosuppressive factors within the TME, including cystatin F (CF), an inhibitor of cathepsins C, H, and L. CF is upregulated in glioblastoma (GBM), correlating with reduced survival. This upregulation compromises NK cell cytotoxicity. CF's activity is regulated by various factors- expression levels, N-glycosylation, and proteolytic activation. In the lysosomes, CF is activated from inactive dimeric form to active monomer and CTSL2 was shown to be the key peptidase involved in the activation of CF.

**Material and Methods**

Compound 7 was identified as a reversible, selective, and potent inhibitor of CTSL2, through molecular docking of small molecular compounds from commercial libraries with CTSL2 and enzyme kinetics for the enzyme inhibition, selectivity, and reversibility of binding. Western blot analysis determined the conversion of CF to its active form, while NK-mediated cytotoxicity against GBM cells was assessed using flow cytometry and confocal microscopy. The effects of compound 7 on GBM cell proliferation were evaluated through flow cytometry and confocal microscopy.

**Results and Discussions**

CTSL2 inhibition decreased the conversion of CF from dimer to active monomer form in primary NK cells. Furthermore, primary NK cells treated with CTSL2 inhibitor had increased cytotoxicity against glioblastoma stem cells compared to untreated NK cells. Compound 7 treatment decreased the proliferation of cancer cells cultured in suspension and in the spheroid model.

**Conclusion**

Targeting CTSL2 presents a promising strategy to mitigate the effects of CF on NK cell function and enhance their cytotoxicity against cancer cells. In addition, CTSL2 inhibition also decreased proliferation of cancer cells offering a potential dual-targeting approach for modulating the TME.

### EACR2024-0592
### Targeting TLR2 as a novel strategy to improve the efficacy of breast cancer immunotherapy

*A. Di Lorenzo[1], C. Cossu[1], E. Bolli[1], E. Di Gregorio[1], G. Ferrauto[1], C. Romiti[1], A. Amaolo[1], G. Donofrio[2], F. Cavallo[1], L. Conti[1]*
*[1]University of Turin, Department of Molecular Biotechnology and Health Sciences, Turin, Italy*
*[2]University of Parma, Department of Veterinary Medical Sciences, Parma, Italy*

**Introduction**

Despite significant progress in its treatment, breast cancer (BC) remains the leading cause of cancer death in women. Therefore, the development of combination therapies able to target key cancer-inducing or cell-sustaining pathways is needed. We have previously demonstrated that Toll-like receptor 2 (TLR2) promotes CSC self-renewal, breast cancer progression and resistance to chemotherapy due to DAMPs binding. TLR2 inhibition restores and potentiates doxorubicin anti-tumor effect in vitro and in vivo. In addition, TLR2

© 2024 The Authors. Molecular Oncology is published by John Wiley & Sons Ltd on behalf of Federation of European Biochemical Societies seems to regulate the expression of the cystine/glutamate antiporter xCT, which controls redox balance and mediates several pro-tumoral mechanisms. The role of xCT in breast cancer is largely studied by our group and different vaccines have been developed to target this antigen with promising results in pre-clinical models. In this study, TLR2 targeting is exploited to improve the efficacy of both chemo- and immunotherapy as a new strategy for breast cancer treatment.

**Material and Methods**

We analyzed the effects of TLR2 inhibitors in combination with chemotherapies or inhibitors of the cystine-glutamate antiporter xCT, a promising BC target. 4T1 tumor-bearing mice were treated with nanosystems delivering TLR2 inhibitors in combination with chemotherapy and xCT immunotargeting.

**Results and Discussions**

TLR2 conferred resistance to immunogenic cell death inducers such as some chemotherapies or xCT inhibitors in BC cells. Treatment with TLR2 inhibitors induced apoptosis of BC cells, and a synergistic effect was observed with chemotherapy or xCT inhibitors. Nanoparticle-mediated delivery of TLR2 inhibitors significantly impaired tumor progression in vivo, and their combination with chemotherapy or xCT immunotargeting further improved these effects.

**Conclusion**

In conclusion, TLR2 mediates several pro-tumoral mechanisms including chemoresitance. TLR2 inhibition improves the efficacy of chemo- and immunotherapies opening new perspectives for the treatment of breast cancer.

### EACR2024-0624
### Cytokine induced killer cell therapy as a treatment strategy for colorectal cancer liver metastases

*K. Fenix[1], C.M.Y. Li[1], G. Maddern[1]*
*[1]The University of Adelaide, Discipline of Surgery-Adelaide Medical School, Adelaide, Australia*

**Introduction**

Colorectal cancer (CRC) is the second leading cause of cancer-related death worldwide, with low response rates to current immunotherapies. Cytokine-induced killer (CIK) cell therapy is a cellular immunotherapy reported to have strong anti-tumour activity across a range of solid tumours. Our previous study showed that CIK cell therapy provides significant clinical benefit to CRC patients. In this study, we aim to develop a protocol for generating CIK cells for the treatment of CRC liver metastases.

**Material and Methods**

We compared the generation of CIK cell therapy products under good manufacturing protocols. The generation of CIK cells were validated by flow cytometry and its efficacy was measured using cancer cell lines and autologous patient derived organoids (PDOs) from CRC liver metastasis biopsies.

**Results and Discussions**

Our results suggest that CIK cells can be consistently generated from liver metastatic CRC patients. Donor attributes such as age, sex, or prior chemotherapy exposure had no significant impact on CIK cell numbers or function. CIK cells are functionally effective, capable of releasing inflammatory cytokines and are able to induce cytotoxic activity against matched PDOs from liver metastatic CRC patients.

**Conclusion**

Our results provide evidence that CIK cell therapy can be developed for wider adoption as a treatment strategy for liver metastatic CRC.

### EACR2024-0644
### Dual blockage of both PD-L1 and CD47 enhances the therapeutic effect of oxaliplatin and FOLFOX in CT-26 mice tumor model

*M. Alimohammadi[1], R. Alimohammadi[2], H. Ghaffari-Nazari[3], S.A. Jalali[2]*
*[1]Tehran University of Medical Sciences, Immunology, Tehran, Iran*
*[2]Shahid Beheshti University of Medical Sciences, Immunology, Tehran, Iran*
*[3]Semnan University of Medical Sciences, immunology-, Semnan, Iran*

**Introduction**

Colorectal cancer (CRC) is known as tumor with poor immunogenicity. By using immunological cell death (ICD) inducing chemotherapy agents we can improve the immunogenicity of CRC. Beside the bifacial effect of ICD inducers, they may increase the expression of inhibitory checkpoint receptor on tumor cells (mainly CD47 and PD-L1), which leads to inhibition the immune system activation. In this study we examined the therapeutic effect of Oxaliplatin and FOLFOX as ICD inuder regimen in combination with blocking antibodies against CD47 and PD-L1.

**Material and Methods**

The cell surface expression of CD47 and PD-L1 were measured in CT-26 cell line following OXP and FOLFOX treatment invitro. To evaluate the invivo therapeutic effect, the subcutaneous CT-26 tumor model was established. Seven days after tumor inoculation, the tumor-bearing mice were randomly assigned into treatment groups: Control , OXP (Oxaliplatin, 6 mg/kg), FOLFOX (Oxaliplatin, 6 mg/kg + 5FU, 50 mg/kg + Flavinin 90 mg/kg), OXP + anti-CD47 (100 μg per mice), OXP + anti-PD-L1 (200 μg per mice), OXP + anti-CD47 + anti-PD-L1 and FOLFOX + anti-CD47 + anti-PD-L1. Tumor tissues were collected to analyze the antitumor mechanisms and tumor microenvironment changes following different therapies. Tumor volume and survival rate were evaluated during 90 days.

**Results and Discussions**

FOLFOX and Oxaliplatin treatment leads to increase in CD47 and PD-L1 expression on CT-26 cells invitro and invivo. Combining blocking Antibodies against CD47 and PD-L1 with FOLFOX may lead to significant increase in survival and decrease in tumor size. This triple combining regimen also leads to a significant decrease in Treg and MDSC and significant increase in CD8+ INF-γ+ lymphocytes and M1/M2 macrophage ratio in tumor microenvironment.

**Conclusion**

© 2024 The Authors. Molecular Oncology is published by John Wiley & Sons Ltd on behalf of Federation of European Biochemical Societies

Our study showed triple combining therapy with FOLFOX, CD47 and PD-L1 is an effective treatment regimen in CT-26 mice tumor model and may consider as a potential to translate to clinic.

### EACR2024-0659
### Ly6C+ monocytes and monocyte-derived dendritic cells enhance efficacy of adoptive T cell therapy in murine lymphoma

*M. Clark[1], R. Cederberg[2], J. Oh[3], A. Patterson[4], R. Klein Geltink[5], K. Bennewith[6]*
*[1]BC Cancer Research Centre, Interdisciplinary Oncology Program- UBC, Vancouver, Canada*
*[2]BC Cancer Research Centre, Department of Pathology and Laboratory Medicine- UBC, Vancouver, Canada*
*[3]BC Children's Hospital Research Institute, Department of Pathology and Laboratory Medicine- UBC, Vancouver, Canada*
*[4]BC Children's Hospital Research Institute, Department of Pathology and Laboratory Medicine- UBC, Vancouver, Canada*
*[5]BC Children's Hospital Research Institute, Interdisciplinary Oncology Program- Department of Pathology and Laboratory Medicine- UBC, Vancouver, Canada*
*[6]BC Cancer Research Centre, Interdisciplinary Oncology Program- Department of Pathology and Laboratory Medicine- UBC, Vancouver, Canada*

**Introduction**
In recent years, immunotherapy has revolutionized the field of oncology by prolonging survival in patients with otherwise rapidly fatal cancers. In particular, adoptive T cell therapies (ACT) demonstrate efficacy against hematological malignancies; however, their effectiveness diminishes in solid tumours due to the hostile tumour microenvironment that limits anti-tumour immune responses. The heterogeneous immune compartment in solid tumours contains various myeloid subsets, including Ly6C$^+$ monocytes, that can either hinder or promote anti-tumour immunity. Ly6C$^+$ monocytes can differentiate into tumour-associated macrophages (TAMs) or monocyte-derived dendritic cells (Mo-DCs), leading to T cell suppression or activation, respectively. We hypothesize that Ly6C$^+$ monocytes improve CD8$^+$ T cell function in the context of ACT, and that harnessing these Ly6C$^+$ monocytes by modulating the tumour microenvironment may improve ACT response.

**Material and Methods**
CD45.1$^+$ BoyJ mice were implanted subcutaneously with EL4-OVA tumour cells. Tumour-bearing mice were infused with OT-I CD8$^+$ T cells, followed by anti-Ly6C$^+$ antibody treatment to deplete Ly6C$^+$ monocytes (or isotype control).

**Results and Discussions**
Intertumoural infiltration of donor T cells was consistently higher in mice that underwent tumour regression following adoptive transfer of OT-I CD8$^+$ T cells. Interestingly, response to ACT was correlated with increased OVA peptide antigen presentation by Ly6C$^+$ monocytes, potentially enhancing donor T cell function. Anti-Ly6C treatment depleted both Ly6C$^+$ monocytes and Mo-DCs (but not TAMs) and did not impact EL4-OVA tumour growth in the absence of ACT. However, anti-Ly6C treatment following adoptive transfer of OT-I CD8$^+$ T cells accelerated tumour growth compared to mice treated with an isotype control. Importantly, Ly6C$^+$ monocyte and Mo-DC depletion following ACT was correlated with decreased infiltration of donor T cells and increased expression of exhaustion markers, such as PD-1, suggesting that Ly6C$^+$ monocytes and Mo-DCs may enhance donor CD8$^+$ T cell effector function in ACT. We are currently working to increase Ly6C$^+$ monocyte and Mo-DC function by modulating the tumour microenvironment, in combination with ACT, to achieve more robust tumour clearance.

**Conclusion**
These data identify a supportive role for Ly6C$^+$ monocytes and Mo-DCs in ACT. Ultimately, increasing Ly6C$^+$ monocyte recruitment or antigen presentation in solid tumours may lead to more durable clinical responses for patients treated with ACT.

### EACR2024-0690
### PBMCs as tool for identification of novel immunotherapy biomarkers in lung cancer

*C. DE ROSA[1], F. Iommelli[2], V. De Rosa[2], C. Tuccillo[1], L. Amato[1], F. Cimmino[3], F. Ciardiello[1], F. Papaccio[4], F. Morgillo[1], C.M. Della Corte[1]*
*[1]University of Campania Luigi Vanvitelli, Department of Precision Medicine, Naples, Italy*
*[2]National Council of Research, Institute of Biostructures and Bioimaging, Naples, Italy*
*[3]Hospital, "Martiri Di Villa Malta", Sarno, Italy*
*[4]University of Salerno, Department of Medicine- Surgery and Dentistry- "Scuola Medica Salernitana", Baronissi, Italy*

**Introduction**
Lung cancer, including both non-small (NSCLC) and small (SCLC) subtypes, is currently treated with combination of chemo- and immune-therapy. However, predictive biomarkers to identify high-risk patients are needed. Our hypothesis was that certain biological parameters potentially detectable in peripheral blood could be used as surrogate biomarkers to predict tumor response. In the present work, we propose an innovative strategy for monitoring and predicting response to immunotherapy using peripheral blood immune cells (PBMCs) as a tool to study the innate immune response and as a source for genetic testing.

**Material and Methods**
We enrolled patients (n=20) with diagnosis of LC receiving cisplatin and/or an anti-PD-L1 antibody. We analyzed the expression of the cGAS-STING pathway in PBMC isolated from LC patients divided into best responders (BR), responders (R) and non-responders (NR). We screened cGAS/STING expression by RT-qPCR and ELISA. The PBMCs were whole exome sequenced (WES) for DNA Damage Response (DDR) panel. We performed an analysis of germline variants which lead to loss of function: gain of stop codon, loss of initiation codon, frameshift, deletion of single exon, missense change and inframe del predicted by in silico tools as deleterious (poliphen-2 and SIFT) and reported

© 2024 The Authors. Molecular Oncology is published by John Wiley & Sons Ltd on behalf of Federation of European Biochemical Societies in ClinVar database as pathogenic or uncertain significance variants.

**Results and Discussions**

We found a significant reduction of CXCL10 and CCL5 in NR serum as compared to BR and R cohorts. The mRNA expression levels of *STING*, *cGAS*, *CXCL10* and *CCL5* in were significantly higher in the BR cohort. In the NR, the decrease in mRNA fold change was associated with poor response to anti-PD-L1 therapy. From WES, each subject had at least 1 germline alteration in a DDR gene. We found germline *POLE*, *POLD1*, *RAD51B*, *CHEK1*, and *ATM* pathogenic variants in BR SCLC patients. Whereas NSCLC BR patients harboured germline pathogenic variants in *BARD1* and *ATM*. This trend may suggest that deleterious DDR variants in the PBMCs of LC patients might have an impact on clinical response to immunotherapy. Thus, we tested the effect of DDR inhibitors (DDRi) in PBMCs, and we found that DDRi strongly increased cGAS-STING expression and tumour infiltration ability. in NR and R patients.

**Conclusion**

PBMCs may be a useful tool for investigating features of immunotherapy responsiveness. Moreover, PBMCs allow to perform functional studies of immune system activation ex vivo, as exemplified by the gene expression profile and immune cells infiltration.

### EACR2024-0719
### Development of activated CAR macrophages for an anti-tumor immunotherapy strategy

*L. Ziane Chaouche[1], A. Raffo-romero[2], N. Hajjaji[3], F. Kobeissy[4], A. Cozzani[5], S. Mitra[5], D. Cizkova[6], I. Fournier[1], M. Salzet[1], M. Duhamel[1]*

*[1]PRISM laboratory. Inserm 1192, Biology, Villeneuve-d'Ascq, France*

*[2]PRISM laboratory. Inserm1192, Biology, Villeneuve-d'Ascq, France*

*[3]Breast Cancer Unit, Oscar Lambret Center, Lille, France*

*[4]Faculty of Medicine, Department of Biochemistry and Molecular Genetics, Beirut, Lebanon*

*[5]Inserm UMR1277- CNRS UMR9020-CANTHER, Lille University Hospital, Lille, France*

*[6]Institute of Neuroimmunology, Slovak Academy of Sciences, Bratislava, Slovakia*

**Introduction**

There is increasing interest in immunotherapeutic approaches to cancer. Chimeric antigen receptor (CAR) T-cell therapy has proven effective in the treatment of hematological tumors; however, its efficacy in the treatment of solid tumors is hampered by lower intra-tumor infiltration of CAR T cells and tumor-induced immunosuppression. Macrophages, which constitute a significant part of the tumor environment, play multiple roles in tumor development and represent promising therapeutic targets. Macrophages can infiltrate solid tumor tissue, interact with various cellular components in the tumor microenvironment, and promote a direct anti-tumor response by phagocytosing tumor cells.

**Material and Methods**

In this study, we developed macrophages expressing a CAR receptor against the HER2 antigen. The CAR receptor has an intracellular domain CD3ζ with homology to the protein FcεRI-γ. When activated by the antibody-antigen recognition complex, this domain induces the phagocytic activity of macrophages.

**Results and Discussions**

Approximately 30% of macrophages express the CAR after transduction. CAR-M exhibited significantly enhanced phagocytosis of HER2-coated beads compared to wild-type (WT) macrophages. CAR-M also demonstrated the ability to phagocytose HER2+ cancer cell lines. Co-culture experiments with breast cancer tumoroids (HER2+ or HER2-) confirmed the efficacy of CAR-M in a more complex environment. However, within the tumor microenvironment, macrophages tend to adopt an anti-inflammatory phenotype, which reduces their anti-tumor activities. To address this issue, we implemented a dual strategy. The combined strategy resulted in increased pro-inflammatory markers and sustained macrophage activation in the presence of cancer cells. In addition, this strategy showed increased phagocytic activity against HER2+ beads or HER2+ tumors. . We also showed that the activated CAR-M secreted factors enhancing T-cell proliferation and could; therefore, modulate the tumor microenvironment.

**Conclusion**

Our therapeutic strategy is based on the dual activation of tumor-infiltrating macrophages. The first activation involves enhancing the phagocytic activity of macrophages by expressing a CAR receptor targeting a tumor antigen. The second activation involves reprogramming macrophages towards a pro-inflammatory phenotype. The ultimate goal of this therapy is to extend its use to other solid tumors, particularly glioblastoma, an aggressive CNS tumor with limited therapeutic options.

### EACR2024-0723
### POSTER IN THE SPOTLIGHT
### Immunophenotypes in mismatch repair proficient colorectal cancer identify a novel biomarker of immunotherapy response

*K. Clayton[1], A. Acha[1], P. Andrei[1], E. Fontana[2], M. Rodriguez-Justo[3], F. Ciccarelli[4]*

*[1]Francis Crick Institute, Ciccarelli Group, London, UK*

*[2]Sarah Cannon Research Institute, Na, London, UK*

*[3]University College Hospital, Pathology, London, UK*

*[4]Francis Crick Institute, Ciccarelli Grouo, London, UK*

**Introduction**

Only a fraction of colorectal cancer (CRC) patients, those with a high tumour mutational burden (TMB) due to deficient mismatch repair (dMMR) machinery, receive immunotherapy. Still fewer of these clinically respond. Most CRCs are MMR proficient (pMMR) and are resistant to immune checkpoint inhibitors (ICIs).

**Material and Methods**

We performed multi-regional RNA sequencing of microdissected stroma and tumour epithelium from dMMR and pMMR CRCs to compare their intrinsic (*i.e.* constitutive of tumour biology) and extrinsic (*i.e.*

Molecular Oncology 18 (Suppl. 1) (2024) 1–495

© 2024 The Authors. Molecular Oncology is published by John Wiley & Sons Ltd on behalf of Federation of European Biochemical Societies related to the tumour microenvironment, including intra-epithelial immune infiltration) properties. Using mouse models, we confirmed that pMMR CRCs expressing high levels of the marker indeed respond to ICI.

**Results and Discussions**

Cytotoxicity, NK cells and M1 macrophages, alongside interferon responses, were descriptive of dMMR versus pMMR subtypes in both tissue compartments. High correlation between the stromal and intra-epithelial enrichments is suggestive of dynamic co-infiltration and recruitment between tissue compartments. We used these enrichment profiles to further dissect CRC phenotypes within their subtype. We found highly parallel immunophenotypes between MMR subtypes representing highly infiltrated to lowly infiltrated tumours suggesting that TMB is not indicative of immune infiltration status in CRC. Most importantly cases with favourable immunotherapy responses were over-represented in clusters with high enrichment of these immuno-phenotypes. Global clustering found that infiltrated pMMR CRCs co-clustered with infiltrated dMMR CRCs, the latter having favourable anti-PD1 responses. We proposed that these pMMR patients possess the immune infiltrate that correlates with and is necessary for clinical response. We confirmed in pMMR mouse models that those with ICI-responsive syngeneic tumours express high levels of the marker protein. Our previous work shows that CRCs with durable response to anti-PD1 immunotherapy show rich cytotoxic T-cell and PDL1+ macrophage infiltrates. Moreover, we show, via immuno-histochemistry, that stromal marker expression is capable of delineating responders from non-responders and is also significantly raised in putative pMMR responders.

**Conclusion**

We have shown in a dMMR and pMMR CRC cohort that ICI responses can be characterised by immunophenotypic status and single marker expression. There exists a subset of pMMR CRC cases, currently untreated, also high for this marker and would likely benefit from immuno-therapeutic intervention.

### EACR2024-0742

### Targeting inflammatory programs to unleash CD8 T cell actions against tumors

*M. Tredicine[1], G. Ferri[1], S. D'Orazio[1], M. Mucci[1], A. Gaimari[2], B.D.S. Patricia[2], M. Mazza[3], S. Chiocca[4], A. Recchiuti[1], D. Mattoscio[1]*

*[1]University of Chieti, Dsmob Cast, Chieti, Italy*

*[2]IRCCS Istituto Romagnolo per lo Studio dei Tumori IRST "Dino Amadori",Immunoterapia- Terapia Cellulare e Biobanca ITCB, Meldola, Italy*

*[3]IRCCS Istituto Romagnolo per lo Studio dei Tumori IRST "Dino Amadori", Immunoterapia- Terapia Cellulare e Biobanca ITCB, Melodola, Italy*

*[4]European Institute of Oncology, Experimental Oncology, Milano, Italy*

**Introduction**

CD8 T cells are the key anticancer effectors and strategies to reinvigorate their function constitute the backbone of immunotherapies. However, recent evidence suggests that inflammatory signals released in the tumor microenvironment (TME) alter T CD8 actions and promote their differentiation into an exhausted phenotype (Tex), that hamper anticancer immunity and response to therapy. Thus, stimulating antitumor functions of CD8 T cells by inferring with cancer associated inflammation is overtly important. Along these lines, we recently reported that re-invigorating resolution (the ideal outcome of inflammation) with resolvins (RvD), shape immune cell plasticity in TME restoring antitumoral functions of innate immune cells. However, if the stimulation of an appropriate resolution program of cancer inflammation impacts on T CD8 functions is still unclear.

**Material and Methods**

To address this, we used head and neck cancer (HNC) positive for human papilloma virus as experimental system due to the immunosuppressive micro-environments despite a robust intratumoral immune infiltration, suggestive of failure in immune cell activation. Bioinformatic analysis of TCGA patient tumors, coupled with in vitro and in vivo preclinical models exposed to RvD were used to dissect how dampening inflammation and restoring resolution reinvigorate T CD8.

**Results and Discussions**

In patients, both killing capacity and Tex were markedly enriched in immunomodulatory, chemotactic and cytokine signaling pathways, thus indicating that inflammatory programs drive T CD8 antitumor functions. Consistent with this, among the tested resolvins, exposure to RvD5 reduced HNC cell proliferation by selectively enhancing T CD8 cell activity in vitro. Mechanistically, RvD5 stimulated T CD8 to block cancer cells in the G2/M phase of the cell cycle and downregulated markers of exhaustion both in CD8+ T (PD-1) and in tumor cells (PD-L1). In preclinical models of HNC in vivo, RvD5 reduced cancer growth by lowering the expression of PDL-1 in tumor cells and PD-1 on tumor-infiltrated CD8 T cells. Cytokine analysis and lipidomic showed an increased release of mediators involved in antitumor response in TME of mice treated with RvD5.

**Conclusion**

Thus, RvD5 reduces tumor growth by modulating inflammation and delaying exhaustion of CD8 T cells in the TME. Therefore, inflammatory signals in TME are crucial to shape T CD8 actions against cancer cell and their targeting could represent a new therapeutic strategy to potentiate antitumor immunity.

### EACR2024-0800

### Development of a novel anti-TIGIT IL-15 fusion antibody for enhanced anti-tumour therapy

*R. Etherington[1], S. Cheon[2], W. Kang[2], T. Bhatt[1], J.H. Kim[1], S. Nielsen[1], C. Willberg[1], Y. Kang[3], H. Jo[3]*

*[1]MediMab Biotherapeutics Ltd., Research and Development, Oxford, United Kingdom*

*[2]MediMabBio Inc., Research and Development, Gyeonggi-do, South Korea*

*[3]MediMabBio Inc., Founder/CEO, Gyeonggi-do, South Korea*

**Introduction**

Immune checkpoint inhibitors have revolutionised the field of cancer immunotherapy, but current options do not prove effective in all indications. Pro-inflammatory

© 2024 The Authors. Molecular Oncology is published by John Wiley & Sons Ltd on behalf of Federation of European Biochemical Societies cytokines, such as IL-15, produce potent immune responses, but are cleared rapidly and carry a high risk of systemic toxicity. To address this, we have developed a fusion antibody combining a potent anti-TIGIT (T cell immunoreceptor with Ig and ITIM domains) blocking antibody with IL-15.

**Material and Methods**

We identified an optimal anti-TIGIT antibody with strong binding to human TIGIT and potent TIGIT-CD155 blocking activity, as determined by TIGIT-CD155 blocking bioassay. After selection of our antibody candidate, we generated an anti-TIGIT-IL-15 fusion antibody (MFA011). IL-15 binding ability was determined by ELISA and IL-15 receptor (IL-15R) signalling was detected by pSTAT5 phospho-staining. In vitro efficacy was assessed in human PBMC from healthy donors using cytotoxicity assays (IncuCyte, Sartorius), ELISA, and flow cytometry-based assays, including intracellular cytokine staining. In vivo efficacy was assessed in CT26 tumour-bearing BALB/c-hTIGIT mice with i.v. dosing of MFA011.

**Results and Discussions**

MFA011 retained a strong ability to both bind human TIGIT and block TIGIT-CD155 interactions, similarly to our anti-TIGIT monoclonal. Furthermore, MFA011 was able to induce potent IL-15R signalling on TIGIT-expressing cells, resulting in sustained activation compared to free IL-15. Production of pro-inflammatory cytokines, such as IFNg and IL-2, by T cells and NK cells was increased by MFA011, which translated to increased killing of target cancer-derived cell lines in in vitro cytotoxicity assays compared to anti-TIGIT blocking alone. MFA011 was able to reduce the immunosuppressive effects of regulatory T cells (Treg) both through depletion of Treg by antibody-dependent cellular cytotoxicity (ADCC), and direct stimulation of CD8+ T cells resulting in increased proliferation. Although IL-15 has strong potential for immuno-toxicity, in vitro and in vivo assays have demonstrated minimal toxicity and effective targeting of MFA011. In accordance with in vitro data, MFA011 also demonstrated enhanced immune responses in vivo as demonstrated by a reduction in tumour volume in CT26-bearing mice.

**Conclusion**

Dual targeting of TIGIT and IL-15 signalling through MFA011 effectively enhances anti-tumour immune responses while limiting the off-target effects of IL-15 signalling.

### EACR2024-0836
### Targeting-specificity of antibody-IL-15 fusion proteins impact T cell infiltration and distribution in homo- and heterotypic tumor spheroids

*A. Möller[1], E. Müller[1], N. Hecke[1], D. Müller[1]*
*[1]Institute of Cell Biology and Immunology- University of Stuttgart, Biomedical Engineering, Stuttgart, Germany*

**Introduction**

IL-15 holds promising potential for cancer immunotherapy; however, systemic administration is restricted by dose-limiting toxicity. Tumor- associated antigen (TAA)-directed antibody-IL-15 fusion proteins are being developed to target IL-15 into the tumor and achieve effective concentrations at lower dosage. This approach is challenged by the heterogeneity in TAA expression and composition in solid tumors, which needs to be addressed for effective implementation. Here, we established homo- and heterotypic tumor spheroids with different target expression patterns and investigated the dynamics of antibody-fusion protein targeting and subsequent immune cell recruitment and distribution in 3D.

**Material and Methods**

Antibody-IL-15 fusion proteins directed against clinically relevant targets (e.g., EGFR, FAP) were produced in HEK293-6E cells. Homo- and heterotypic spheroids were generated in ultra-low attachment plates using cancer cell lines and fibroblasts with defined TAA expression profile. Infiltration and distribution of antibody-IL-15 fusion proteins and PBMCs into spheroids were assessed at different time points. The spheroids were i.) shock-frozen, cyrosectioned, stained, and imaged with confocal laser scanning microscopy ii.) directly mounted and imaged with multiphoton confocal laser scanning microscopy iii.) singularized cells stained to determine the immune cell profile, and analyzed using flow cytometry and FlowJo$^{TM}$ software. Image analyses were conducted using ImageJ and CellProfiler$^{TM}$.

**Results and Discussions**

The antibody-IL-15 fusion proteins penetrated gradually and distributed into homo- and heterotypic spheroids in a target-specific manner. At high antigen expression, strong retention and accumulation at the rim suggested a "binding site barrier effect", while lower antigen expression was associated with a rather uniform distribution pattern. Furthermore, target-specific retention of fusion proteins related to the infiltration and distribution of immune cells, which were mainly identified as activated T cells.

**Conclusion**

In our model system, target expression and density dictated binding and distribution of antibody-IL-15, which related to the activation and recruitment pattern of T cells into tumor spheroids. Further studies are now required to corroborate these findings in models of higher complexity. Thus, insights from 3D-models will contribute to evaluating and improving the targeting design of the antibody-IL-15 strategy.

### EACR2024-0854
### Unravelling heterogeneity in response to engineered T-cell immunotherapy using cancer patient derived organoids

*B. Vervoort[1], E. van Vliet[1], A. Zeeman[1], A. Cleven[2], T. Aarts-Riemens[2], J. Kuball[2], Z. Sebestyen[2], M. Alieva[3], A. Rios[1]*
*[1]Princess Maxima Center, Research, Utrecht, The Netherlands*
*[2]University Medical Center Utrecht, Center for Translational Immunology, Utrecht, The Netherlands*
*[3]Instituto de Investigaciones Biomedicas Alberto Sols, research, Madrid, Spain*

**Introduction**

© 2024 The Authors. Molecular Oncology is published by John Wiley & Sons Ltd on behalf of Federation of European Biochemical Societies

Engineered T-cell therapy has revolutionized the field of cancer therapy, specifically for blood-based cancers. This success has yet to be translated to solid tumors, where numerous challenges hamper the infiltration, recognition, and killing capacity of therapeutic immune cells. Next to resistance mechanisms related to the tumor micro-environment, tumor intrinsic heterogeneity between patients and even within the same tumor, challenges the achievement of consistent clinical outcomes. Here, we study the molecular heterogeneity of breast cancer-derived organoids and the precise determinants of response to engineered T-cell therapy.

**Material and Methods**

To explore this heterogeneity in tumor response, we previously developed an organoid-based co-culture system. Using dynamic live imaging, we identified variations in response at the individual organoid level using the analytical tool called BEHAV3D[1]. To explore how this molecular heterogeneity at single organoid level impacts response to engineered T-cell therapy, we mapped the therapy response and transcriptomic profiles of up to 100 clonal organoid lines derived from 4 patients.

**Results and Discussions**

Using this approach, we uncovered variations in how individual organoids respond to T-cell therapy that we could link to inherent biological diversity among organoids derived from the same patient. These findings highlight the need to better understand intra-tumoral heterogeneity for guiding personalized treatment development. We present FUN-CLON: a pipeline for transcriptomics analysis of FUNctionally annotated CLONal organoids to identify gene signatures associated with different T-cell resistance or sensitivity mechanisms shared among patients. Validation of these signatures across publicly available sequencing datasets and spatial transcriptomics data showed that they were not only differentially expressed between and within patients but also predictive of T-cell infiltration in these tumors. Furthermore, FUN-CLON allowed for discriminating between general and engineered T-cell specific resistance mechanism, uncovering a multifaceted landscape of resistance to T-cell therapy.

**Conclusion**

With our pipeline we identified heterogenous molecular signatures affecting T-cell therapy sensitivity, we can distinguish tumor patient groups for tailored T-cell immunotherapy and provide promising leads for the optimization of engineered T-cell therapy in solid tumors.

### EACR2024-0875

### Antisense oligonucleotides (ASOs) targeting Neuropilin-1 (NRP1) in combination with anti-PD-(L)1 antibodies as a novel approach to treat cancer

*A. Maaske[1], N. Kirchhammer[2], J. Festag[1], M. Buchi[2], M. Schell[1], S. Raith[1], S. MIchel[1], A. Zippelius[2,3], F. Jaschinski[1], R. Klar[1]*

*[1]secarna pharmaceuticals GmbH & Co. KG, Immune oncology, Planegg, Germany*

*[2]University Hospital Basel, Cancer Immunology- Department of Biomedicine, Basel, Switzerland*

*[3]University Hospital Basel, Medical Oncology, Basel, Switzerland*

**Introduction**

NRP1 takes part in many processes that are of relevance to tumors, including migration and polarization of macrophages, phenotypic stability of regulatory T-cells, exhaustion of effector T-cells and angiogenesis. These functions are mediated by different domains of NRP1, strongly indicating a possible advantage in the knockdown of the entire protein, over treatment modalities, which block the function of individual domains. On the other hand, antibodies don't seem to face the same issues when targeting surface proteins with singular functionality like anti-PD-1 and anti-PD-1. Nevertheless, the majority of patients doesn't benefit from this treatment, leading to a still unmet medical need. Here we show that locked nucleic acid (LNA) modified third generation ASOs specific for NRP1 have therapeutic efficacy as monotherapy and in combination with anti-PD-(L)1 antibodies in murine cancer models.

**Material and Methods**

Both human and murine surrogate LNAplus™ ASOs specific for NRP1 were designed with our in-house Oligofyer™ bioinformatics system, achieving knockdown of more than 85% in vitro. In vivo knockdown was analyzed on mRNA- and protein level. Different syngeneic tumor cells were implanted into the mammary fat pads of mice. Upon reaching an adequate tumor size, mice were randomized and systemically treated with NRP1-specific ASOs, anti-PD-1 or anti-PD-L1 antibodies, or a combination of these. Tumors were excised shortly after treatment initiation for further analysis, or continuously monitored regarding growth and subsequent survival of the mice.

**Results and Discussions**

Already on day three of systemic treatment with NRP1-specific ASOs, robust knockdown of NRP1 was observed in several intra-tumoral cell types, including infiltrating macrophages and T-cells. This knockdown led to delayed tumor growth and even complete eradication of tumors in some animals, resulting in a survival benefit in comparison to vehicle-treated animals. Furthermore, we observed a strong on top effect on anti-PD-(L)1 antibodies, leading to complete tumor elimination in up to 73% of mice.

**Conclusion**

ASOs targeting NRP1 are a novel therapeutic modality that has potent anti-tumor efficacy as monotherapy and in combination with checkpoint inhibitors in murine tumor models. Encouraged by the promising results, preparation of a clinical trial to treat patients with solid cancers has been started.

*The following authors are employees of secarna pharmaceuticals: AM, JF, MS, SR, SM, FJ, RK The following authors either hold patents or are part of filed patent applications pertaining to inventions mentioned in the present abstract:AM, JF, SM, FJ, RK*

### EACR2024-0905

### POSTER IN THE SPOTLIGHT

### Developing personalized immunotherapy strategies using an ex vivo tumor fragment platform

© 2024 The Authors. Molecular Oncology is published by John Wiley & Sons Ltd on behalf of Federation of European Biochemical Societies

T. Reissig[1,2], M. van Ingen[1,2], C. Spooner[3], P. Vitorino[3], S. Liang[3], C. Garnett-Benson[3], D. Thommen[1,2]
[1]The Netherlands Cancer Institute, Division of Molecular Oncology and Immunology, Amsterdam, The Netherlands
[2]Oncode Institute, Oncode Institute, Utrecht, The Netherlands
[3]Bristol Myers Squibb, Research and Development, Princeton- New Jersey, United States

**Introduction**

PD-1/PD-L1 blockade has improved survival in cancer patients, but long-term responses are limited. To improve outcomes, combination therapies might be key, however the development of rational and personalized combinations is challenging. We here exploit a patient-derived tumor fragment (PDTF) platform to identify new synergistic immunomodulatory treatment combinations targeting non-redundant suppressive pathways.

**Material and Methods**

PDTFs preserve the human tumor microenvironment (TME) and show immune activity upon *ex vivo* PD-1 blockade that predicts clinical anti-PD-1 response. We cultured PDTFs from four cancer types (kidney, lung, skin, ovary) that were either left untreated or exposed to different agents including anti-PD-1 (nivolumab), anti-LAG3 (relatlimab), anti-ILT4 (BMS-986406), anti-CCR8 (BMS-986340) and/or JNK inhibitor (BMS-986360). After culture, soluble mediators were assessed, and immune cell activation was measured by flow cytometry. In addition, comprehensive TME profiling, focused on myeloid and T cell states, and target expression, was performed, allowing to link this information to the treatment-induced effects.

**Results and Discussions**

To make optimal use of limited cancer tissue and to identify treatment combinations in a rational way, we developed a phased approach. In phase 1, tumors were evaluated for response to single or dual checkpoint inhibition (anti-PD-1±anti-LAG3). Non-responding tumors were then tested for targeting of multiple pathways including anti-PD-1+anti-LAG3 with either anti-ILT4, JNKi or anti-CCR8 (phase 2). In phase 3, the best responding triple combination from phase 2 was validated and compared against single and dual combinations to confirm synergy and understand contribution of the compounds. As clinical data are not yet available for these combinations, we assessed soluble mediator production and immune cell activation as indicators of immunological response. Whereas in some tumors single or dual checkpoint blockade could enhance immune activity, most tumors required targeting of additional pathways to overcome immune resistance. Notably, individual tumors showed response to distinct treatment combinations, highlighting the need for developing personalized immunotherapy strategies.

**Conclusion**

Translational studies using patient-derived tumor models may help to identify rational immunotherapy combinations overcoming anti-PD-1 resistance and their biomarkers, and to prioritize them for clinical development.

### EACR2024-0996
### Assessment of an in vitro potency assay for evaluation of immune cell-mediated cytotoxicity using the Omni Pro 12 automated imaging platform

D. Sullivan[1], L. Stemkens[2], I. Thijssen[2], S. Meiler[3], M. Vermeulen[3], D. Califano[4], S. Chvatal[4], D. Millard[1]
[1]Axion BioSystems, Applications, Atlanta, United States
[2]Axion BioSystems, Applications, Eindhoven, The Netherlands
[3]Axion BioSystems, Product Managment, Eindhoven, The Netherlands
[4]Axion BioSystems, Product Managment, Atlanta, United States

**Introduction**

The development of immunotherapies relies on the use of in vitro potency assays—which are key for understanding complex interactions between immune cells and cancer cells. Immune effector T-cells are a promising cancer therapy due to their innate cytotoxicity. In particular, CAR T-cell therapy uses genetically engineered T-cells that express a chimeric antigen receptor that binds to a specific antigen on tumor cells. Assessing the efficacy and potency of CAR T-cell therapies in vitro and at high throughputs is vital for the preclinical development of these promising therapies. Here, we describe an in vitro potency assay that uses an automated imaging platform to quantify immune cell-mediated cytotoxicity of cancer target cells by immune effector cells.

**Material and Methods**

A GFP fluorescent and HER-2 expressing lung cancer cell line, A549, was seeded into a 96-well cell culture plate. Then, HER-2 CAR T-cells were added at 24 hours post target cell seeding at various E:T ratios (1:10, 1:5, 1:2, 1:1, and 5:1). Fluorescent images of the target cells were captured every 6 hours by the Omni Pro 12, an automated, high-throughput, live-cell analysis platform designed for continuous multi-well imaging inside an incubator. Automated image analysis was performed using the Confluency Module in the Axion Portal to view attachment and proliferation and quantify cytolysis of fluorescent A549 target cells. Percent cytolysis of the target cells was calculated by comparing the green fluorescent confluency of treated wells to no treatment control wells.

**Results and Discussions**

A549-GFP cells showed a dose-dependent decrease in confluency over time that correlated with increasing amounts of CAR T-cells. At 92 hours post HER-2 CAR T-cell addition, the 5:1 ET group demonstrated approximately 91.8% +/- 2.0% cytolysis of A549-GFP cells, while the 1:10 group demonstrated approximately 69.2% +/- 3.0% cytolysis.

**Conclusion**

Overall, the Omni Pro 12 platform enables continuous quantification of the potency and kinetics of immune cell-mediated cytolysis. Future work will evaluate differences in CAR T-cell potency when co-cultured with target cells that express different levels of HER-2.

© 2024 The Authors. Molecular Oncology is published by John Wiley & Sons Ltd on behalf of Federation of European Biochemical Societies

### EACR2024-1029
### Validation of an impedance-based in vitro potency assay for repeatability and precision

*D. Califano[1], J. Jiang[2], D. Windgassen[3], C. De La Flor[4], Z. Yu[5], S. Chvatal[1], D. Sullivan[6], D. Millard[1]*
*[1]Axion BioSystems, Product Managment, Atlanta, USA*
*[2]Miltenyi Biotec, Analytical Development, Sunnyvale, USA*
*[3]Miltenyi Biotec, Analytical Development, San Jose, USA*
*[4]Miltenyi Biotec, Product Managment, San Diego, USA*
*[5]Axion BioSystems, Business Development, Eindhoven, The Netherlands*
*[6]Axion BioSystems, Applications, Atlanta, USA*

**Introduction**

Cell-based immunotherapies, such as CAR T cells, are emerging as a promising approach to therapeutic intervention against cancer. Developing cell and gene therapy products requires reliable and reproducible methods of quantifying critical quality attributes, such as potency, to ensure product strength and consistency. A variety of assays can be used to assess potency; however, it is crucial that these tests are qualified for assay performance to ensure intra- and inter-assay precision. Biological assays, although inherently variable, are often the best methods to investigate a product's mechanism of action and predict clinical outcomes. Previous work has shown that impedance-based potency assays provide a non-invasive, real-time measure of effector cell-mediated cytotoxicity, thus reducing cell manipulation that impacts data reliability.

**Material and Methods**

Effector cell-mediated cytotoxicity was performed using CD19-positive liquid tumor cell line (Raji) that was tethered to the well surface using an anti-CD40 antibody. CD19-specific CAR T effector cells were added 24 hours after the target cells and co-cultured with the target cells across a range of E: T ratios and cytolysis was monitored for six days. Real-time measurement of cell-mediated cytotoxicity also allowed for the calculation of kill time 50 (KT50), defined as the time required for 50% cytolysis of the target cells.

**Results and Discussions**

The coculture experiment assay was performed in duplicate by a second analyst and on subsequent days to evaluate inter-assay precision. In addition to this, CAR T-mediated cell killing in a multiplate impedance system was also investigated for plate-to-plate variability. The percent coefficient of variation (% CV), a statistical measure that describes the precision and repeatability of an assay, was calculated to assess variability between assay replicates, operators, and plate replicates. In all conditions tested, the percent CV for all E:T ratios was below 20% illustrating the repeatability and precision of the assay.

**Conclusion**

Here, we describe an in vitro potency assay that demonstrates low variability and high precision across replicates, analysts, and days. These data support the use of an impedance-based potency assay for the evaluation and characterization of immunotherapy products.

### EACR2024-1062
### NEO-TIL Developing durable novel TIL therapies for lung cancer

*A. Tomar[1], R. Li[1], A. Soo[2], A.M. Quinn[3], P. Loftus[4], M. Kerin[1], L. Barkley[1]*
*[1]Lambe Institute for Translational Research- University of Galway, Department of Surgery, Galway, Ireland*
*[2]University Hospital Galway, Department of Cardiothoracic Surgery, Galway, Ireland*
*[3]University Hospital Galway, Department of Anatomic Pathology, Galway, Ireland*
*[4]Orbsen Therapeutics Ltd., Upstream Process Development, Galway, Ireland*

**Introduction**

Lung cancer is among the leading causes of cancer mortality worldwide. The advancement in therapies using monoclonal antibody-based immune checkpoint blockade (ICB) has shown minor improvements in overall survival. Regardless of these improvements, the majority of patients suffer cancer progression either after first-line treatment only or combined treatment with ICB. The development of novel personalized treatment is an urgent need for prolonged tumour regression to enhance the survival rates of lung cancer patients. Adoptive Cell Transfer of polyclonal tumour infiltrating lymphocytes *(ACT-TIL)* is a potential treatment option for lung cancer patients, due to lung cancers' high mutational load. This study aims to develop a process of successful TIL isolation and expansion from lung cancer tissue and generate a biobank of patient-specific tumour-reactive TILs.

**Material and Methods**

Ethical approval is in place to obtain cancer tissue from lung cancer patients undergoing surgery at University Hospital Galway. Lung tumour tissue is sliced into small fragments of 1-2 $mm^2$ and seeded into TIL media supplemented with interleukin-2 (IL-2). The TILs are grown for 15 days, cell counts are performed. After expansion, TILs are characterized by flow cytometry analysis to measure T-cell markers (CD3, CD4, and CD8) and T-cell exhaustion markers (PD-1, TIM-3, and LAG-3). Autologous tumour cell lines (TCLs) are established from the same piece of lung cancer tissue. Co-culture experiments with TILs and autologous TCLs are performed and IFN-ϒ release is measured (ELISA) to assess TIL tumour reactivity.

**Results and Discussions**

Using this process, we have isolated TILs from 28 lung cancer patients. We have determined the growth kinetics of these TIL populations. These cells are positive for T cell markers CD3, CD4, and CD8. They also express inhibitory receptors PD-1, LAG-3, TIM-3, which are typically expressed on chronically stimulated CD8+ T cells and would suggest these TILs are tumour reactive. Indeed, initial co-culture experiments show an increase in IFN-ϒ release when TILs are co-cultured with autologous tumour cells, indicating TILs are tumour reactive.

**Conclusion**

We have developed a research-based process that enables the isolation and growth of TILs from lung cancer tissue. Future studies will advance this TIL expansion process to a GMP-compliant process with the aim of developing a

© 2024 The Authors. Molecular Oncology is published by John Wiley & Sons Ltd on behalf of Federation of European Biochemical Societies precision medicine for Irish lung cancer patients in the West of Ireland.

### EACR2024-1111
### CAR T cell-mediated cytotoxicity and cytokine release in response to varying levels of antigen expression on target cells

*S. Barnes[1], D. Sullivan[2], D. Lazar[3], K. Haupt[4], Z. Yu[5], D. Califano[6], S. Chvatal[7], D. Millard[2]*
*[1]Axion BioSytems, Applications, Atlanta, USA*
*[2]Axion BioSystems, Applications, Atlanta, USA*
*[3]Promega, Cell Analysis, Madison, USA*
*[4]Promega, Product Managment, Madison, USA*
*[5]Axion BioSytems, Business Development, Eindhoven, The Netherlands*
*[6]Axion BioSytems, Product Managment, Atlanta, USA*
*[7]Axion BioSystems, Product Managment, Atlanta, USA*

**Introduction**
The development of immunotherapies relies on the use of in vitro potency assays, which are crucial for understanding the complex interactions between immune cells (effectors) and cancer cells (targets). Chimeric antigen receptor (CAR) T-cell therapy is an immuno-therapeutic approach that exhibits promise; however, varying levels of antigen expression on the surface of tumor cells can influence the efficacy of CAR T cell-mediated killing. To fully understand the impact of target antigen density on CAR T cell activity, we utilized a matrix approach to assess both target cell death, using an impedance-based in vitro potency assay to quantify CAR T cell-mediated cytotoxicity, as well as cytokine release with Lumit™ immunoassays.
**Material and Methods**
SKOV3 (high HER2 expression), A549 (low HER2 expression), or MDA-MB-231 (no HER2 expression) target cells were seeded into a 96-well microplate with embedded electrodes in the substrate that detect the attachment and proliferation of target cells. HER2 CAR T cells were added after 24 hours at effector-to-target (E:T) ratios of 1:5 or 1:1 and target cell cytolysis was recorded continuously by the Maestro Z for 72 hr.
**Results and Discussions**
At 72 hours post CAR T cell addition, complete killing was observed in SKOV3 cells at the 1:1 ratio, while A549 cells exhibited only 80% cytolysis. MDA-MB-231 cells showed 20% cytolysis, likely due to nonspecific killing by non-engineered T cells. To assess release of pro-inflammatory cytokines TNF-α and IFN-γ, we collected supernatant at 24, 48, and 72 h. At the 1:1 E:T ratio, CAR T cells co-cultured with target cells expressing low or high levels of HER2 had the highest TNF-α production at 24 hours, with CAR T cells co-cultured with SKOV3 cells releasing 30.6% more TNF-α when compared to A549 cells. The highest levels of IFN-γ were detected in A549 and SKOV3 groups at the 1:1 E:T ratio, with the highest levels observed from CAR T cells co-cultured with SKOV3 cells at 72 hr, releasing approximately 1459.6 +/- 357.3 pg/mL of IFN-γ, compared to 1093.8 +/- 387.5 pg/mL IFN-γ released by CAR T cells co-cultured with A549 cells. As expected, CAR T cells co-cultured with MDA-MB-231 cells did not release any detectable TNF-α or IFN-γ at any E:T ratio.
**Conclusion**
Continuous monitoring of CAR T cell-mediated killing and measurement of cytokine release was demonstrated against different target cancer cell lines with varying HER-2 antigen densities and across different E:T ratios, illustrating the importance of target antigen density selection.

### EACR2024-1125
### Analysis of the Mechanisms of Heavy-Chain Ferritin Uptake by Macrophages - Implications for Drug Delivery and Immunotherapy

*M. Kubiak[1], P. Kucharzewska[1], M. Gorczak[1], A. Boffi[2], M. Krol[1], L. Krzeminski[3]*
*[1]Warsaw University of Life Sciences, Cellular Immunotherapy Center, Warsaw, Poland*
*[2]Sapienza University of Rome, Department of Biochemical Sciences "Alessandro Rossi Fanelli", Rome, Italy*
*[3]Biosens Labs, Sp. z o.o, Warsaw, Poland*

**Introduction**
Macrophages (MQ) are one of the most abundant non-malignant cells in the tumor microenvironment and for this reason, they are one of the main targets of tumor immunotherapy. Due to the physiological interaction between cancer and MQ, the use of these cells as drug carriers could allow an active targeting and controlled drug release. Most of the currently used anti-cancer therapies use the highly cytotoxic substances, therefore, to avoid the pre-mature death of carrier cells, it is necessary to use a drug-binding transporter such as ferritin (Ft) which seems to be an ideal choice due to its exceptional properties. The aim of the study was to analyze the mechanisms and to find the receptor responsible for heavy-chain ferritin (HFt) uptake by human MQ to help developing the pioneering drug delivery system.
**Material and Methods**
The involvement of various endocytic pathways was examined using well-known chemical inhibitors. To examine the role of Macrophage Scavenger Receptor 1 (MSR1) we used various methods such as: siRNA-mediated knockdown, overexpression, competition assays and colocalization studies with multiple receptor-specific ligands. The interaction of HFt with MSR1 was further verified by kinetic binding analysis using cutting-edge techniques such as AlphaScreen.
**Results and Discussions**
In this study we confirmed the important role of the clathrin-dependent endocytosis in HFt uptake by MQ; however, there are also non-specific endocytic pathways such as macropinocytosis involved in this process. Moreover, the results of this project provided a clear confirmation of the key role of MSR1 in HFt uptake in MQ. Our findings are consistent with the previously published research indicating the possible interaction between MSR1 and HFt; however, this is the first time when the existence of the binding between these two proteins was proved for cells naturally expressing MSR1.

© 2024 The Authors. Molecular Oncology is published by John Wiley & Sons Ltd on behalf of Federation of European Biochemical Societies

**Conclusion**
Our research provides a comprehensive understanding of the mechanism of HFt uptake by human MQ with the potential to utilize it to enhance the precision and efficacy of cancer therapies while minimizing unintended cytotoxicity. It can contribute to the development of new therapeutic strategies based on a) HFt as a drug nanocarrier and its ability to target cells with increased expression of MSR1 (such as tumor-associated MQ) and b) macrophage-mediated drug delivery in which the combination of MQ ability to internalize HFt and the encapsulation of drugs inside HFt could solve the problem of side effects of anti-cancer therapeutics.

### EACR2024-1129
### Targeting IL1R2+ tumor-infiltrating regulatory T cells with an anti-IL1R2 nanobody construct induces a strong anti-tumor effect in combination with anti-PD1 therapy

*S. Arnouk[1,2], D. Kancheva[1,2], J. Van Craenenbroeck[1,2], H. Van Damme[1,2], Y. Elkrim[1,2], E. Lebegge[1,2], T. De Groof[3], G. Raes[1,2], D. Laoui[1,2], J. Van Ginderachter[1,2]*
*[1]Vrije Universiteit Brussel, Brussels Center for Immunology, Brussels, Belgium*
*[2]VIB Center for Inflammation Research, Myeloid Cell Immunology Lab, Brussels, Belgium*
*[3]Vrije Universiteit Brussel, In vivo Cellular and Molecular Imaging Laboratory- Department of Medical Imaging, Brussels, Belgium*

**Introduction**
Immune checkpoint blockade (ICB) has shown success in treating certain cancers, but the overall response rates remain low, particularly for cancers with low mutational burden. There are several pieces of evidence that implicates immunosuppressive cell types, such as tumor-infiltrating regulatory T cells (tiTregs), in the lack of efficacy of ICB. Therefore, targeting those cells is very promising. However, peripheral Tregs are important for maintaining tolerance and immune homeostasis. Hence, the targeting needs to be only directed towards the tiTregs, which can be done by targeting a tiTreg-specific marker.
**Material and Methods**
Analysis of publicly available scRNAseq and CITEseq datasets, flow cytometry, in vitro assays, nanobody (Nb) generation and production.
**Results and Discussions**
IL1R2 has been suggested by us and others to be one of the tiTreg-specific markers. Therefore, we verified that IL1R2 protein is absent from peripheral Tregs and from anti-tumoral immune cells infiltrating mouse tumor models. We also showed that IL1R2 marks a highly activated and suppressive population of tiTregs. However, a deficiency of this receptor, either in the whole body or specifically within Treg, did not affect tumor characteristics nor tiTreg infiltration or phenotype, suggesting that this receptor is not functionally important on these cells in a cancer setting. Next, we generated Nbs, the smallest naturally occurring antigen-binding fragments, against IL1R2 and functionalized them with the Fc region of mouse IgG2a, known for its capacity to mediate antibody-dependent cell-mediated cytotoxicity (ADCC). This construct could indeed successfully deplete a proportion of the tiTregs which, in combination with ICB, caused a significant delay in mammary tumor growth. Furthermore, we enhanced the efficacy of the Nb construct by introducing point mutations in the Fc region known to potentiate the ADCC activity. We showed that the combination of ICB with this potentiated construct was superior to the combination with the original construct, causing full regression of 60% of the mammary carcinoma tumors with maintenance of immunological memory against a later challenge with the same cancer cells.
**Conclusion**
IL1R2 is a validated marker for activated suppressive tiTregs. Depleting tiTregs with anti-IL1R2 Nb-Fc constructs yields a potent therapeutic effect when combined with immune checkpoint inhibitors. Surface IL1R2 is found on tiTregs across various human cancer types, supporting the potential clinical application of our therapy.

### EACR2024-1136
### Anti-MIF nanobodies as a novel tool to improve cancer therapy

*R. Mora[1], B. Stijlemans[1], J. Van Ginderachter[1]*
*[1]Vrije Universiteit Brussels, Brussels Center of Immunology, Brussels, Belgium*

**Introduction**
The macrophage migration inhibitory factor or MIF is a multifunctional cytokine that has been implicated in 9 out of the 10 Hallmarks of cancer. It can bind to CD74, its main receptor, but also to CXCR2, CXCR4, and CXCR7. MIF's protumoral effects have already been studied and described, and most importantly, its blockade shows encouraging results as a possible alternative to improve cancer therapy. On this note, we have developed and characterized cross-reactive anti-MIF nanobodies (Nbs). They successfully showed to be able to decrease inflammation, specifically reducing TNF expression of murine and human LPS-stimulated cells.
**Material and Methods**
Subsequently, generating a trivalent version of 2 anti-MIF Nbs coupled with an anti-albumin Nb, to increase the half-life, gave better results in-vivo compared to the monovalent construct significantly improving the protection in a murine endotoxemia model and increasing mice survival. Based on these results and taking into consideration MIF's pro-tumoral role, we tested the trivalent constructs in MC-38 tumor-bearing mice, alone or in combination with anti-PD1 therapy.
**Results and Discussions**
We found that the anti-MIF monotherapy significantly reduces tumor growth compared to the untreated and the anti-PD-1-only treated group. The anti-MIF + anti-PD-1 therapy caused slower tumor growth compared to the untreated group and the anti-PD-1 group. However, there are no significant differences in tumor growth between the anti-MIF monotherapy and anti-MIF + anti-PD1 combinatorial treatment, suggesting no additional contribution of the anti-PD-1 treatment. Within the tumor microenvironment, we found a significant increase of

© 2024 The Authors. Molecular Oncology is published by John Wiley & Sons Ltd on behalf of Federation of European Biochemical Societies

CD45+cells and IFN-γ CD8+ producing cells in the anti-MIF + anti-PD-1 treated group compared to the other groups.

**Conclusion**

This information confirms that blocking MIF partially improves the outcome. However, these results could be further improved by modifying the delivery system and directing the therapy toward the tumors to increase its accumulation and prevent its sink in the periphery due to circulating MIF.

### EACR2024-1147
### Generation of a novel anti-EGFR monoclonal antibody CC4-B3-G3-H10 against Epidermal Growth Factor Receptor dimerization domain

*O. Bender[1], H. Gurdal[2], E. Yilmaz[1], D. Engin[1], A. Atalay[1]*
*[1]Ankara University, Biotechnology Institute, Ankara, Turkiye*
*[2]Ankara University, Faculty of Medicine- Medical Pharmacology, Ankara, Turkiye*

**Introduction**

EGFR is a transmembrane glycoprotein that constitutes one of four members of the ErbB family of tyrosine kinase receptors. Binding of EGFR to its ligands leads to autophosphorylation of the receptor tyrosine kinase and subsequent activation of signaling pathways involved in the regulation of physiological events such as cellular proliferation and differentiation. The sophisticated oncogene features of EGFR have made it an attractive target. Efforts to develop more effective therapeutic or diagnostic agents against EGFR are constantly ongoing. This study aimed to develop, characterize, and examine the effectiveness of a novel anti-EGFR monoclonal antibody against the EGFR dimerization region. Thus, even if the ligand binds there will be no dimerization and activation of EGFR and downstream pathways.

**Material and Methods**

Mice were immunized with a 20.24 kDa recombinant antigen of 824-1251 nucleotides encoding EGFR domain 2. Hybridomas were grown and selected by using a methylcellulose-based semi-solid selective medium system. During the selection process, anti-EGFRd2-producing mAb was demonstrated with a unique workflow by multistep immunoblotting and ELISA assays. The mAb was purified and then subjected to isotyping and affinity testing. Biological activity was monitored by real-time cell analyses to show the antibody's cellular inhibition efficacy on various cancer cells. Relative levels of 17 different phosphorylated amino acids were identified in EGFR and other ErbB family members with human EGFR phosphorylation antibody array.

**Results and Discussions**

Soluble recombinant EGFRd2 protein was produced with a high yield (0.5 mg/mL) and purity. 21 out of 209 hybridomas were detected specifically against EGFRd2. After further passages, four hybridomas were identified as stable and potent secretors. Then a stable clone was developed which produced the IgG1 kappa type anti-EGFR CC4-B3-G3-H10 mAb against EGFR dimerization site that binds its antigen with high affinity. It also has been shown to recognize endogenous EGFR. Cellular inhibition tests showed that it was at least as effective as Cetuximab. It was observed that EGFR (Tyr845), ErbB2 (Tyr1112), and ErbB2 (Ser1113) amino acids were endogenously phosphorylated in Caco-2 cells and Tyr845 and Tyr1112 were significantly decreased and there was no significant change at the Ser1113 level. Non-endogenous ErbB2 (Tyr877) increased significantly.

**Conclusion**

We conclude that novel CC4-B3-G3-H10 mAb has capacity for diagnostic applications and promising for therapeutic area.

### EACR2024-1148
### The endogenous T cell landscape is orchestrated by CAR-T cells and predictive of treatment response

*J. Poller[1], J. Frede[1], K. Shi[1], H. Stuart[1], N. Sotoudeh Chafi[1], K. Lim[1], C. Havig[2], T. Vijaykumar[1], B. Knoechel[2], J. Lohr[1]*
*[1]Dana-Farber Cancer Institute, Medical Oncology, Boston, United States*
*[2]Dana-Farber Cancer Institute, Pediatric Oncology, Boston, United States*

**Introduction**

T-cell redirecting immunotherapies, especially tumor-specific chimeric antigen receptor modified-T (CAR-T) cells, have transformed the treatment landscape for patients with hematological malignancies including multiple myeloma (MM). However, durable responses are observed in only a fraction of patients. To overcome treatment resistance and enhance the efficacy of CAR-T cells, a detailed understanding of their co-evolution with other components of the immune microenivronment is needed.

**Material and Methods**

We dissected the longitudinal CAR-T cell interactions that critically alter CAR-T cell function by investigating the immune microenvironment before, 1 month and 6 month after CAR-T cell treatment. We focused on anti-BCMA CAR-T cells in patients with MM as a model system, as the vast majority of this patient population experiences relapses. We used single cell transcriptome profiling to define the transcriptional states in CAR-T and endogenous T cells in combination with immune profiling and VDJ sequencing.

**Results and Discussions**

By dissecting the alterations in CAR-T cells and the CD8+ T cell compartment over time we observed persistent depletion of naïve-like CD8+ T cells, resulting in reduced differentiation potential and plasticity. We observed state-specific clonal expansion in the activated effector subset and identified pre-existing clones (39%), which we tracked over time and cell states. We defined novel transitional T cell states with an intermediate role in the T cell differentiation process. Transitional T cells T1 were associated with poor response, thus representing a potential target cell group to increase CAR-T cell efficacy. Additionally, we predicted TIM3/GAL9 as a highly specific interaction between endogenous T cells and CAR-T cells. TIM3 was highly specific to CAR-T cells by RNA and surface and its expression was correlated with worse clinical outcome. Our data indicate

© 2024 The Authors. Molecular Oncology is published by John Wiley & Sons Ltd on behalf of Federation of European Biochemical Societies

GAL9 as an important regulator of anti-tumor immune response in MM and as a promising therapeutic target in MM to optimize T-cell based therapies.

**Conclusion**

We identify a novel transitional CD8+ T cell population that coincides with sustained depletion of the tumor-reactive T cell repertoire, compositional evolution of functional T cell subsets and a diminishing potential for self-renewal. Furthermore, we determine GAL9 as a potential therapeutic target.

### EACR2024-1155
### Innovative immunotherapy based on commensal-derived peptides for enhancing CD8+ T cell activation against Tumor-Associated Antigens

A. Talpin[1], J.M. Carpier[1], L. Aubergeon[1], J. Kervevan[1], C. Gaal[2], A. Maia[3], C. Gouttefangeas[3], L. Chene[1], J. Gamelas Magalhaes[1]

*[1]Enterome, Drug Discovery, Paris, France*
*[2]Enterome, Data Science, Paris, France*
*[3]Institute for Immunology and Cluster of Excellence iFIT EXC2180- Eberhard-Karls-University Tübingen, Image-Guided and Functionally Instructed Tumor Therapies, Tübingen, Germany*

**Introduction**

Peptide-based immunotherapy offers significant potential against cancer by leveraging the body's immune system to eliminate cancer cells, targeting tumor-associated antigens (TAAs) or tumor-specific antigens (TSAs). The OncoMimics™ approach innovatively uses commensal-derived peptide antigens mimicking TAA-derived peptides (TAAps) to elicit a cross-reactive T cell response against tumors.

**Material and Methods**

We present a novel peptide-based immunotherapy relying on the concept of molecular mimicry and cross-reactivity between commensal-derived peptides called OncoMimics™ peptides (OMPs) and TAAps to induce strong cytotoxic T cell responses against tumors. Our approach involves the selection and validation of HLA-A2 restricted peptides derived from the microbiota, capable of inducing a robust anti-tumor response. These peptides were assessed in humanized mouse models and evaluated ex vivo for their ability to activate cytotoxic human cross-reactive T cells.

**Results and Discussions**

In humanized HLA-A2 murine models, OMPs trigger the expansion of cross-reactive OMP-/TAAp- specific $CD8^+$ T cells with specific cytotoxic activity against tumor cells. Experiments conducted on HLA-$A2^+$ healthy human peripheral blood mononuclear cells (PBMCs) reveal a high prevalence of cross-reactive OMP-/TAAp-specific $CD8^+$ T cells when stimulated in vitro. In addition, those cross-reactive $CD8^+$ T cells demonstrate ability to exert cytolytic activity against tumor cells presenting TAAps.

**Conclusion**

The OncoMimics™ strategy illustrates the capacity of bacterial antigen-derived peptides to elicit anti-tumor immune responses, highlighting a new avenue for cancer immunotherapy.

### EACR2024-1156
### Investigating the Apoptotic Effect of NK-92 Cells on the Du145 Prostate Cancer Cell Line Post-Stimulation with Anti-KIR2DL4

M. Dastouri[1], N. Kilic[2], H. Yilmaz[3]

*[1]Ankara Medipol University, Medical Biology- School of medicine, Ankara, Turkiye*
*[2]Ankara University- Faculty of Science, Department of Biology, Ankara, Turkiye*
*[3]Ankara Yıldırım Beyazıt Üniversitesi- Department of Medical Biology, Institute of Health Sciences, Ankara, Turkiye*

**Introduction**

The growing interest in utilizing immune cells for cancer therapy has led to a shift towards exploiting the body's immune system as a novel approach to combat cancer, with a focus on selectively targeting cancer cells while preserving normal ones. NK-92 cell-based therapies, renowned for their potent cytotoxicity, have emerged prominently in this arena, where the balance of activator and inhibitor receptors within NK cells governs their cytotoxic effectiveness. While studies suggest that anti-KIR2DL4 acts as an activator, its impact on DU145 prostate cancer cells when stimulating NK-92 cells remains unexplored. Our study uniquely examines the anti-cancer effects of NK-92 cells stimulated with anti-KIR2DL4 on the DU145 cancer cell line.

**Material and Methods**

Experimental setups involved culturing stimulated and unstimulated NK-92 cells with DU145 prostate cancer cells and normal prostate PNT1A cells at cell ratios of 1:5 and 1:10. Cellular cytotoxicity was assessed using WST-1 to determine the most effective cell ratio. Once the optimal cell ratio was determined, the synthesis of apoptosis pathway-related proteins, including Caspase-3, Caspase-8, Caspase 9, and BAX, was analyzed through immunostaining.

**Results and Discussions**

Results demonstrated that stimulated NK-92 cells exhibited greater cytotoxicity towards DU145 cancer cells compared to PNT1A normal prostate cells. Among all NK-92 cell groups stimulated with the agonistic antibody anti-KIR2DL4, the most effective response was observed against the DU145 prostate cancer cell line at a ratio of 1:10 (Target: Effector). Immunostaining revealed a notable increase in apoptosis pathway-related proteins in groups cultured with induced NK-92 cells.

**Conclusion**

Our study has shown that NK-92 cells when stimulated with antibody anti-KIR2DL4, display enhanced cytotoxicity against DU145 prostate cancer cells compared to normal prostate cells. Additionally, immunostaining analysis indicated a significant increase in apoptosis pathway-related proteins in NK-92 cell groups subjected to stimulation. These findings underscore the potential of anti-KIR2DL4-stimulated NK-92 cells as a promising therapeutic strategy for targeting prostate cancer. Moreover, they lay a solid foundation for further investigation in subsequent preclinical and clinical studies, potentially paving the

© 2024 The Authors. Molecular Oncology is published by John Wiley & Sons Ltd on behalf of Federation of European Biochemical Societies way for the development of more effective cancer treatment modalities

### Molecular and Genetic Epidemiology

### EACR2024-0436

### Impact of weight loss on cancer-related proteins in serum: results from a cluster randomised controlled trial of individuals with type 2 diabetes

*E. Hazelwood[1], C. Bull[1], D. Legge[2], L. Corbin[1], T. Richardson[1], N. Sattar[3], N. Murphy[4], M. Gunter[5], N. Timpson[1], E. Vincent[2]*

*[1]University of Bristol, Population Health Sciences, Bristol, United Kingdom*

*[2]University of Bristol, Translational Health Sciences, Bristol, United Kingdom*

*[3]University of Glasgow, School of Cardiovascular and Metabolic Health, Glasgow, United Kingdom*

*[4]International Agency for Research on Cancer, Section of Nutrition and Metabolism, Lyon, France*

*[5]Imperial College London, Department of Epidemiology and Biostatistics, London, United Kingdom*

**Introduction**

Type 2 diabetes (T2D) is associated with higher risk of several cancers. However, the biological intermediates driving this relationship are not fully understood. As novel interventions for managing T2D become increasingly available, whether they also disrupt the pathways leading to increased cancer risk is unknown. We investigated the effect of a T2D intervention, involving intentional weight loss, on circulating cancer-associated proteins to gain insight into potential mechanisms linking T2D and adiposity with cancer development.

**Material and Methods**

Fasting serum samples from participants with diabetes enrolled in the Diabetes Remission Clinical Trial (DiRECT) receiving the Counterweight-Plus weight-loss programme (intervention, N = 117, mean weight-loss 10 kg, 46% diabetes remission) or best-practice care by guidelines (control, N = 143, mean weight-loss 1 kg, 4% diabetes remission) were analysed using the Olink Oncology-II platform. To identify proteins which may be altered by the weight-loss intervention, the difference in protein levels between groups at baseline and 1 year was examined using linear regression. Mendelian randomization (MR) was performed to extend these results to evaluate cancer risk and elucidate possible biological mechanisms linking T2D and cancer development. MR analyses were conducted to estimate potential causal relationships between proteins modified during intentional weight loss and the risk of colorectal, breast, endometrial, gallbladder, liver, and pancreatic cancers.

**Results and Discussions**

Nine proteins were modified by the intervention: glycoprotein Nmb; furin; Wnt inhibitory factor 1; toll-like receptor 3; pancreatic prohormone; erb-b2 receptor tyrosine kinase 2; hepatocyte growth factor; endothelial cell specific molecule 1 and Ret proto-oncogene (Holm corrected *P*-value <0.05). MR analyses indicated a causal relationship between predicted circulating furin and glycoprotein Nmb on breast cancer risk (odds ratio (OR) = 0.81, 95% confidence interval (CI) = 0.67–0.99; and OR = 0.88, 95% CI = 0.78–0.99 respectively), though these results were not supported in sensitivity analyses examining violations of MR assumptions.

**Conclusion**

Intentional weight loss among individuals with recently diagnosed diabetes may modify levels of cancer-related proteins in serum. Further evaluation of the proteins identified in this analysis could reveal molecular pathways that mediate the effect of adiposity and T2D on cancer risk.

### EACR2024-0667

### Polymorphisms of cell cycle regulators: risk factor for prostate cancer

*M. Knoško Brožová[1], J. Jurečeková[1], M. Mečiaková[1], D. Evin[2], J. Kliment[3], R. Dušenka[3], M. Vilčková[1], M. Kmeťová Sivoňová[1]*

*[1]Jessenius Faculty of Medicine in Martin- Comensius University in Bratislava, Department of Medical Biochemistry, Martin, Slovakia*

*[2]University Hospital Martin, Clinic of Nuclear Medicine, Martin, Slovakia*

*[3]University Hospital Martin, Urological Clinic, Martin, Slovakia*

**Introduction**

Prostate cancer (PCa) is the second most prevalent cancer in men worldwide, representing 15% of all cancers diagnosed in males with a high incidence, complex and multifocal features and atypical early clinical symptoms. In addition, PCa harbors numerous genetic and epigenetic abnormalities that drive unrestricted cellular growth and increased metabolic demands. The progression of the cell cycle is well controlled by the regulators. One of them are cyclin dependent kinases (CDKs). CDKs are serine/threonine kinases that form a complex with cyclin proteins and their phosphorylation leads to cell cycle progression. Second one is their inhibitors such as cyclin-dependent kinase inhibitor 1 ($p21^{Waf1}$) that has a significant role in modulating DNA repair processes by inhibiting cell cycle progression.

**Material and Methods**

We decided to study the association between selected single nucleotide polymorphisms (SNP) of *CDK1* (intron A/G, rs2448343) and $p21^{Waf1}$ (intron G/C, rs730506) and the risk of developing prostate cancer and with clinico-pathological characteristics (serum PSA, Gleason score and pathological T stage). Using PCR-RFLP and Real-time PCR we analyzed samples of patients with histologically verified prostate cancer and healthy individuals.

**Results and Discussions**

We can confirm a significant risk of developing prostate cancer in individuals with a mutant genotype in the p21 polymorphism (p=0,008). After analysis of the associations of clinical status, the p21 polymorphism showed a positive association with a higher Gleason score (>7) (p=0,005). No significant associations were found for the CDK1 but we found out for the combined

Molecular Oncology 18 (Suppl. 1) (2024) 1–495
© 2024 The Authors. Molecular Oncology is published by John Wiley & Sons Ltd on behalf of Federation of European Biochemical Societies genotypes of CDK1 (*AA*) and p21 (*GC*) significantly increase the risk of PCa compared to individuals with wild-type genotypes (p=0,05).

**Conclusion**

We assume that this finding of single nucleotide polymorphisms could be one of the genetic markers of prostate cancer.

### EACR2024-0726

### The mediating role of mammographic density in the protective effect of early-life adiposity on breast cancer risk

*M. Vabistsevits*[1], *G. Davey Smith*[1], *T. Richardson*[1], *R. Richmond*[1], *W. Sieh*[2], *J. Rothstein*[2], *L. Habel*[3], *S. Alexeeff*[3], *B. Lloyd-Lewis*[4], *E. Sanderson*[1]

[1]*University of Bristol, MRC Integrative Epidemiology Unit, Bristol, United Kingdom*

[2]*Icahn School of Medicine at Mount Sinai, Department of Genetics and Genomic Sciences, New York, United States*

[3]*Kaiser Permanente Northern California, Division of Research, Oakland, United States*

[4]*University of Bristol, School of Cellular and Molecular Medicine, Bristol, United Kingdom*

**Introduction**

Observational studies suggest that mammographic density (MD) may have a role in the unexplained protective effect of childhood adiposity on breast cancer risk. In this work, we investigated a complex and interlinked relationship between puberty onset, adiposity, MD, and their effects on breast cancer using Mendelian randomization (MR).

**Material and Methods**

We estimated the effects of childhood and adulthood adiposity, and age at menarche on MD phenotypes (dense area (DA), non-dense area (NDA), percent density (PD)) using MR and multivariable MR (MVMR), allowing us to disentangle their total and direct effects. Next, we examined the effect of MD on breast cancer risk, including risk of molecular subtypes, and accounting for genetic pleiotropy. Finally, we used MVMR to evaluate whether the protective effect of childhood adiposity on breast cancer was mediated by MD.

**Results and Discussions**

Childhood adiposity had a strong inverse effect on mammographic DA, while adulthood adiposity increased NDA. Later menarche had an effect of increasing DA and PD, but when accounting for childhood adiposity, this effect attenuated to the null. DA and PD had a risk-increasing effect on breast cancer across all subtypes. The MD single-nucleotide polymorphism (SNP) estimates were extremely heterogeneous, and examination of the SNPs suggested different mechanisms may be linking MD and breast cancer. Finally, MR mediation analysis estimated that 56% (95% CIs [32% - 79%]) of the childhood adiposity effect on breast cancer risk was mediated via DA.

**Conclusion**

In this work, we sought to disentangle the relationship between factors affecting MD and breast cancer. We showed that higher childhood adiposity decreases mammographic DA, which subsequently leads to reduced breast cancer risk. Understanding this mechanism is of great importance for identifying potential targets of intervention, since advocating weight gain in childhood would not be recommended.

### EACR2024-0843

### Comprehensive Analysis of Early-onset Ovarian Cancer Genetic Predispositions

*K. Horackova*[1], *P. Zemankova*[1], *P. Nehasil*[1], *M. Hovhannisyan*[1], *M. Janatova*[1], *P. Kleiblova*[1], *B. Stastna*[1], *Z. Kleibl*[1], *J. Soukupova*[1]

[1]*Institute of Medical Biochemistry and Laboratory Diagnostics- First Faculty of Medicine- Charles University and General University Hospital in Prague- Prague- Czech Republic, Laboratory of Oncogenetics, Prague, Czech Republic*

**Introduction**

Ovarian cancer (OC) is the leading cause of cancer-related death in women, with a typical onset after the age of 60. Characteristics of OC include a high frequency of germline pathogenic variants (GPV); however, a specific subgroup of OC patients diagnosed before 30 years of age is prominent with distinguishing features including a lack of GPV in established genes associated with hereditary breast, ovarian, and pancreatic cancer (HBOPC) predisposition.

**Material and Methods**

The peripheral blood-derived DNA and RNA from 123 early-onset OC patients were analyzed using whole exome sequencing, with additional human leukocyte antigen (HLA) typing, polygenic risk score (PRS) and survival analysis, compared to histology/stage-matched late-onset and unselected OC patients, and population-matched controls.

**Results and Discussions**

We identified only 6/123 (4.9%) early-onset OC patients carrying GPV in established high-penetrance HBOPC genes; however, two other distinct trajectories of so far missing heritability of early-onset OC stood out. First, we propose an association of early-onset OC predisposition with breast cancer (BC), highlighted by the enrichment of *CHEK2* GPV ($p$=1.2×10$^{-4}$) and a significant stratification of early-onset OC patients from controls by the $PRS_{313}$ SNP set ($p$=0.03), which is considered to be BC-specific. Second, impaired anti-tumor immunity may be involved, as we observed a significant frequency of HLA homozygotes ($p$=3×10$^{-7}$) and carriers of some HLA risk alleles (HLA-A*36:01, B*53:01, DQA1*01:03 DRB1*11:01; $p<0.05$) in the early-onset OC patients along with the enrichment of GPV in the antigen presentation-related gene *LY75-CD302* ($p$=8.3×10$^{-4}$) compared to controls. In addition, early-onset OC patients had a significantly higher germline mutation burden compared to controls and showed a survival advantage compared to both *gBRCA1/2*-negative age-unselected and histology/stage-matched late-onset OC patients.

**Conclusion**

The genetic predisposition to early-onset OC (<30 years) does not appear to follow the classical Mendelian monogenic inheritance but may be associated with an atypical BC-like inheritance and impaired anti-tumor

© 2024 The Authors. Molecular Oncology is published by John Wiley & Sons Ltd on behalf of Federation of European Biochemical Societies immune response due to impaired HLA-mediated antigen-recognition and higher germline mutation burden.

*Supported by Ministry of Health of the Czech Republic: NU23-03-00150, NW24-03-00092, DRO-VFN-64165; Charles University projects: COOPERATIO, SVV260631, UNCE/24/MED/022; Ministry of Education Youth and Sports of the Czech Republic grant EXCELES no. LX22NPO5102 and LM2023033.*

### EACR2024-0844
### Germline pathogenic FANCG variants are not predisposing to hereditary breast and ovarian cancer

*B. Stastna[1], J. Soukupova[1], P. Kleiblova[1], P. Zemankova[1], K. Horackova[1], M. Hovhannisyan[1], L. Macurek[2], Z. Kleibl[1]*
*[1]Institute of Medical Biochemistry and Laboratory Diagnostics- First Faculty of Medicine- Charles University and General University Hospital in Prague- Prague- Czech Republic, Laboratory of Oncogenetics, Prague, Czech Republic*
*[2]Institute of Molecular Genetics of the Czech Academy of Sciences- Prague- Czech Republic, Laboratory of Cancer Cell Biology, Prague, Czech Republic*

**Introduction**
Heterozygous germline pathogenic variants (gPV) in several Fanconi anemia (FA) genes (including *BRCA1/FANCS*, *BRCA2/FANCD1*, *PALB2/FANCN*, *BRIP1/FANCJ*, *RAD51C/FANCO*) predispose to breast and/or ovarian cancer. However, the risk associated with gPV in other FA genes is not yet fully understood. We investigated the association between gPV in *FANCG* and the risk of breast and ovarian cancer.
**Material and Methods**
We compared the frequency of *FANCG* gPV (truncations and missense) in 10,204 breast cancer patients and 2,966 ovarian cancer patients obtained from the CZECANCA database with that in 3,250 population-matched controls analyzed by panel NGS. Additionally, we analyzed loss-of-heterozygosity (LOH) in five tumors samples from gPV carriers and we performed an in vitro functional assay of selected rare but recurrent missense variants to complement the activity of FA core complex in U2OS-FANCG knock-out cells.
**Results and Discussions**
The frequency of *FANCG* gPV did not differ between breast cancer (20/10,204; 0.20%), or ovarian cancer (8/2,966; 0.27%) patients and controls (3/3,250; 0.18%). In addition, we detected LOH of wt-alleles of *FANCG* in only one in five tumors analyzed. Furthermore, the performed functional in vitro analysis showed that none of the nine tested missense variants affected FANCG capacity in DNA repair (FANCD2 mono-ubiquitination and FANCD2 foci formation upon the DNA damage), in contrast to all tested *FANCG* truncations.
**Conclusion**
Our results suggest that heterozygous gPV in *FANCG* do not likely contribute in a clinically significant way to the development of breast and ovarian cancer and that information about gPV in *FANCG* is of low prognostic and predictive value and thus of limited clinical utility.

Supported by grants: Ministry of Health of the Czech Republic: NU23-03-00150, NW24-03-00092, DRO-VFN-64165; Charles University projects: COOPERATIO, SVV260631, UNCE/24/MED/022; and the Ministry of Education Youth and Sports of the Czech Republic grant EXCELES no. LX22NPO5102 and LM2023033.

### EACR2024-0863
### Pan-cancer analysis of germline CHEK2 pathogenic variants in 17,654 cancer patients

*B. Stastna[1], L. Stolarova[2], P. Kleiblova[1], P. Zemankova[1], J. Soukupova[1], K. Horackova[1], M. Hovhannysian[1], L. Macurek[2], Z. Kleibl[1]*
*[1]Institute of Medical Biochemistry and Laboratory Diagnostics- First Faculty of Medicine- Charles University and General University Hospital in Prague- Prague- Czech Republic, Laboratory of Oncogenetics, Prague, Czech Republic*
*[2]Institute of Molecular Genetics of the Czech Academy of Sciences- Prague- Czech Republic, Laboratory of Cancer Cell Biology, Prague, Czech Republic*

**Introduction**
The *CHEK2* gene, encodes the nuclear serin/threonine checkpoint kinase CHK2, which is activated in response to DNA damage and other stress signals. Germline pathogenic *CHEK2* variants (gPV) confer moderate risk of breast cancer. In addition to truncating/spliceogenic/CNV gPV, dozens of rare missense *CHEK2* variants, which have been shown to impair CHK2 function, increase the breast cancer risk similarly. Because *CHEK2* gPV has been associated with other cancer types, we performed a pan-cancer analysis of gPV in *CHEK2* to assess their associations with different cancer types in a large, homogeneous population of Czech cancer patients.
**Material and Methods**
We compared the frequency of *CHEK2* gPV (truncations and missense) in 17,654 cancer patients and 1,662 population-matched controls analyzed by panel NGS. Using Fisher's exact test, we determined the association of *CHEK2* gPV (truncating/spliceogenic/CNV and pathogenic/likely pathogenic missense) in the Czech population with different types of cancer.
**Results and Discussions**
Out of a total 306 *CHEK2* gPV carriers, 243 (79.4%) carried truncating/spliceogenic/CNV variants (1.4% patients), and 63 (20.6%) were functionally-impaired missense gPV (0.4% patients). Carriers of *CHEK2* gPV had significantly increased risk of gastrointestinal cancer (OR=4.39; 95%CI:1.38-13.37), male genital cancer (OR=3.21; 95%CI:1.12-9.19), kidney cancer (OR=3.82; 95%CI:1.27-11.25), thyroid cancer (OR=4.68; 95%CI:1.22-15.74), hematological malignancies (OR=4.37; 95%CI:1.14-14.68) and with female (OR=3.25 ; 95%CI:1.67-7.25) and male (OR=8.06; 95%CI:2.52-24.7) breast cancer. A marginal association of *CHEK2* gPV was observed with lung cancer.
**Conclusion**
These results are consistent with the previous studies showing that heterozygous *CHEK2* gPV contributes to the development of various cancer types and indicates that about 20% of gPV carriers represent individuals with missense gPV.

*Supported by grants: Ministry of Health of the Czech Republic: NU23-03-00150, NW24-03-00092, DRO-VFN-64165; Charles University projects: COOPERATIO, SVV260631, UNCE/24/MED/022; and the Ministry of Education Youth and Sports of the Czech Republic grant EXCELES no. LX22NPO5102 and LM2023033.*

© 2024 The Authors. Molecular Oncology is published by John Wiley & Sons Ltd on behalf of Federation of European Biochemical Societies

### EACR2024-0950
### Polygenic Risk Score and Breast Cancer Risk Prediction in the Czech Population

M. Hovhannisyan[1], P. Zemánková[1,2], P. Nehasil[1,2,3], K. Matějková[1,4], K. Horáčková[1], B. Šťastná[1,5], J. Soukupová[1], P. Kleiblová[1,6], Z. Kleibl[1,2], M. Janatová[1]
[1]Institute of Medical Biochemistry and Laboratory Diagnostics, First Faculty of Medicine- Charles University and General University Hospital in Prague, Prague, Czech Republic
[2]Institute of Pathological Physiology, First Faculty of Medicine- Charles University, Prague, Czech Republic
[3]Department of Paediatrics and Inherited Metabolic Disorders, First Faculty of Medicine- Charles University and General University Hospital in Prague, Prague, Czech Republic
[4]Department of Genetics and Microbiology, Faculty of Science- Charles University in Prague, Prague, Czech Republic
[5]Department of Biochemistry, Faculty of Science- Charles University, Prague, Czech Republic
[6]Institute of Biology and Medical Genetics, First Faculty of Medicine- Charles University and General University Hospital in Prague, Prague, Czech Republic

**Introduction**

Polygenic Risk Score (PRS) quantifies the effect of low-penetrance alleles present in an individual's genome. Currently, several sets of single nucleotide polymorphisms (SNPs) have been constructed to assess breast cancer (BC) risk.

**Material and Methods**

In this retrospective study, we aimed to evaluate the performance of two widely used sets, PRS313 and PRS77, on the population of 1329 Czech breast cancer patients and 1324 non-cancer controls. All participants had no pathogenic variants in the HBOC predisposition genes. NGS was employed to genotype the participants for both sets of SNPs.

**Results and Discussions**

PRS313 outperformed PRS77 in both categorical and continuous PRS analyses. Patients in the top 2.5% of PRS313 had a threefold increased risk of developing BC ($p=1.76\times10^{-4}$); no significant risk increase was observed in the top 2.5% using PRS77. The HR per SD was 1.64 (95%CI=1.49–1.81; $p<2.0\times10^{-16}$) for PRS313 compared to 1.40 (95%CI=1.28–1.53; $p=3.89\times10^{-14}$) for PRS77.

**Conclusion**

While both sets were able to stratify women according to their PRS, PRS313 better discriminated between the patients and the controls. Subsequently, the incongruent categorisation between the sets indicates possible errors in risk assessment when using PRS77.

*Acknowledgement: This work was supported by Charles University project grants COOPERATIO, SVV260631, UNCE/24/MED/022, and the EXCELES no. LX22NPO5102 grant from the Ministry of Education, Youth and Sports of the Czech Republic.*

### EACR2024-1165
### Risk of colorectal adenomas in celiac disease patients: Evidence from real-world studies

R.N. Jahan[1], Y. Sultana[1]
[1]School of Pharmaceutical Education and Research- Jamia Hamdard, Pharmaceutics, New Delhi, India

**Introduction**

Celiac disease (CD) is an autoimmune disorder that causes an immune-based reaction to gluten. Uncontrolled and untreated CD can lead to other autoimmune disorders including gastrointestinal cancers. However, the increased risk of colorectal adenomas in patients with CD has not been extensively estimated. Therefore, we aimed to estimate the risk of colorectal adenomas in patients with CD.

**Material and Methods**

A systematic literature search was performed on PubMed/Medline and Cochrane Library using relevant pairing keywords. Eligible studies are identifying observational studies to estimating the risk and events of colorectal adenoma/neoplasia and advance colorectal adenoma in patients with CD. The outcomes were estimating the odds ratios (OR) with 95% confidence intervals (CI) using a random-effect model. A meta-analysis was carried-out using Review Manager (RevMan) V 5.3 software.

**Results and Discussions**

We identified a total of four eligible studies including 424 cases of CD and 800 controls of non-CD. The mean age of participants were 57.60 years. Results from the meta-analysis showed no significant difference between CD patients and risk of developing colorectal adenomas (OR= 1.27, 95% CI, 0.72, 2.26; p= 0.41). Similarly, the progression of advance adenomas risks also showed non-significant difference between CD patients and control group (OR= 1.05, 95% CI, 0.58, 1.90; p= 0.86).

**Conclusion**

The current meta-analysis suggests that the risk of developing colorectal adenomas and advance adenomas were not significantly associated with CD patients. However, due to the scarce of published evidence, more real-world studies warranted to determine the causal association and make this finding more robust.

### EACR2024-1264
### The contribution of moderate- and high-penetrance cancer genes to the risk of gastric cancer

J. Guerra[1], A. Estrada Florez[2], P. Lott[2], G. Hispanic Gastric Cancer Genetics Collaborative[2], C. Sanabria[3], M. Teixeira[1], L. Carvajal Carmona[4]
[1]Portuguese Oncology Institute of Porto, Cancer Genetics Group, Porto, Portugal
[2]University of California, Genome Center, Davis- California, United States
[3]Instituto Nacional de Cancerología, Programa de Cáncer Hereditario, Bogota, Colombia
[4]University of California- Davis, Biochemistry and Molecular Medicine, Davis- California, United States

**Introduction**

Approximately 10% of gastric cancer cases exhibit familial clustering, yet only 1-3% can be explained by a known hereditary syndrome, being the Hereditary Diffuse Gastric Cancer (HDGC) the most well-known hereditary syndrome of gastric cancer. The other gastric cancer hereditary syndrome associated with intestinal

© 2024The Authors. Molecular Oncology is published by John Wiley & Sons Ltd on behalf of Federation of European Biochemical Societies gastric cancer is the familial intestinal gastric cancer (FIGC), however, this remains widely understudied and genetically unexplained. Likewise, the heritability of families with mixed GC clustering remains to be uncovered.

**Material and Methods**

We evaluated the contribution of pathogenic and likely pathogenic (P/LP) variants in 13 well-established and multi-organ cancer predisposition genes (*APC*, *ATM*, *BRCA2*, *BRCA1*, *PALB2*, *RAD51D*, *RAD51C*, *CHEK2*, *MLH1*, *MSH2*, *MSH6*, *PMS2*, and *TP53*) to the risk of GC, in 667 GC patients recruited in three independent studies in Spain, Portugal and Latin America.

**Results and Discussions**

We identified 39 patients (6%) with P/LP variants in *ATM*, *BRCA2*, *BRCA1*, *PALB2*, *TP53*, *RAD51D*, *RAD 51C*, *CHEK2*, *MLH1*, and *PMS2*. ATM and BRCA2 were mutated in 13 and 11 patients, respectively and were the most mutated genes. We found a P/LP variant prevalence of 9.3% (18/193), 4.9% (5/103), and 4.2% (14/334) in patients with intestinal mixed and diffuse tumors respectively. The average age of diagnosis in the P/LP variant carriers was 54 years (y), with an average age of diagnosis of 52y in patients with diffuse GC, 54y in patients mixed tumors and 56y in patients with intestinal tumors. We found a significantly lower age of onset among female P/LP variant carriers (53y vs. 56y in men, P<0.05).

**Conclusion**

To our knowledge, we are the largest study on the contribution of known cancer genes to the risk of GC. While the P/LP prevalence was 6% in our study, one in 10 of the cases with intestinal tumors had a causal germline mutation. As most of the mutated genes play a crucial role in DNA repair, our findings are of dual importance for GC prevention and treatment as carriers of these variants likely respond to immunotherapies or PARP inhibitors. Our results show that genes known to predispose to cancer should also be considered for testing in GC patients and families, regardless of histology type.

### Prevention and Early Detection

### EACR2024-0047
### Nutritional assessment for 891 patients with common cancer in a cancer hospital of Southwest China

Y. Huang[1], M. Chen[2]
*[1]Yunnan Provincial Corps Hospital of Chinese People's Armed Police Forces, Outpatient Department, Kunming, China*
*[2]Chongqing University Cancer Hospital, Department of Clinical nutrition, Chongqing, China*

**Introduction**

To investigate the nutritional status of hospitalized patients with common malignant tumor in a cancer hospital of Southwest China.

**Material and Methods**

From April 2017 to May 2021, we enrolled 891 patients with cancer hospitalized for treatment in Chongqing university Cancer Hospital. These patients were diagnosed with one of the following 16 different types of malignant tumors: lung cancer, gastric cancer, liver cancer, colorectal cancer, breast cancer, esophageal cancer, cervical cancer, endometrial cancer, naso-pharyngeal carcinoma, malignant lymphoma, leukemia, pancreatic cancer, ovarian cancer, prostate cancer, bladder cancer and brain tumor. Patient-generated subjective global assessment (PG-SGA), anthropometric measurements, and laboratory examination were used to evaluate the nutritional risk or nutritional status.Cancer pain status were assessed with the Numerical Rating Scale (NRS). We also investigated the nutritional therapy of these cancer patients.

**Results and Discussions**

According to the PG-SGA score, 48.7% (434/891) of the cancer patients were severe malnutrition (PG-SGA≥9), 31.2% (312/891) were moderate malnutrition (8≥PG-SGA≥4), 14.7% (131/891) were mild malnutrition (PG-SGA 2~3), and only 5.4% (48/891) patients were no malnutrition (PG-SGA 0~1) . The rate of malnutrition for gastrointestinal cancer patients is higher than Nongastro-intestinal cancer patients (67.3% vs. 44.6%, $\chi 2=31.48$, P <0.001). Multiple linear regression analysis, PG-SGA scores and body mass index (P<0.001), serum total protein(P<0.001), hemoglobin serum(P<0.001), albumin (P<0.001), prealbumin (P<0.001), calf circumference (left side, P=0.001) were correlated. Age(≥65 years), albumin(<40g/L), prealbumin (<150mg/L) and cancer pain (NRS≥4) are the risk factors of severe malnutrition. However, only 26.8% (200/746) of all the moderately and severely malnourished patients received nutritional therapy.

**Conclusion**

94.6% of the common malignant tumor patients enrolled in the present study were malnutrition. Nutritional therapy of malignant tumor patients with malnutrition is very low. Suggestions for patients with malignant tumor after admission nutritional risk screening, and comprehensive nutritional evaluation, including PG-SGA score, and to give the right nutritional therapy.

### EACR2024-0053
### Analysis of the expression of TPM5 and WASF1 in gastric cancer and their predictive value for patient prognosis

H. Qiu[1]
*[1]Chongqing Traditional Chinese Medicine Hospital, Department of gastroenterology, Chongqing, China*

**Introduction**

To explore the expression of tropomyosin 5 (TPM5) and WASF1 in gastric cancer tissues and their predictive value for the prognosis of gastric cancer patients.

**Material and Methods**

A total of 90 gastric cancer patients admitted to our hospital from February 2016 to February 2018 were selected. The surgically resected gastric cancer and adjacent cancer tissues were collected, and the expressions of TPM5 and WASF1 were detected by immune-histochemistry. The difference of positive expression rates of TPM5 and WASF1 in gastric cancer tissues with different clinicopathological characteristics was compared, the factors affecting prognosis of patients

Molecular Oncology 18 (Suppl. 1) (2024) 1–495
© 2024 The Authors. Molecular Oncology is published by John Wiley & Sons Ltd on behalf of Federation of European Biochemical Societies with gastric cancer were analyzed by *Logistic* regression, and the value of TPM5 and WASF1 in predicting adverse outcomes of patients with gastric cancer was analyzed by subject work characteristic curve (ROC).

**Results and Discussions**

The positive expression rate of TPM5 in gastric cancer tissues was lower than that in adjacent cancer tissues ($P< 0.05$), and the positive expression rate of WASF1 was higher than that in adjacent cancer tissues ($P< 0.05$). The positive expression rate of TPM5 in gastric cancer tissues with low differentiation, subserous membrane and beyond, stage III and lymph node metastasis was lower than that of medium and high differentiation, infiltration into mucosa and submucosa, stage I to II and no lymph node metastasis ($P< 0.05$), and the positive expression rate of WASF1 was higher than that of medium and high differentiation, infiltration into mucosa and submucosa, stage I to II and no lymph node metastasis ($P< 0.05$). *Logistic* regression analysis showed that late TNM stage, lymph node metastasis and WASF1 positive expression were risk factors for poor prognosis in gastric cancer patients, and TPM1 positive expression was protective factor ($P< 0.05$). The combination of TPM5 and WASF1 in predicting the death of gastric cancer patients was 0.867, which was higher than 0.723 and 0.746 predicted separately ($P< 0.05$).

**Conclusion**

In gastric cancer tissues, the positive expression rate of TPM5 decreased, and the positive expression rate of WASF1 increased. The negative expression of TPM5 and positive expression of WASF1 were related to the malignant clinicopathological characteristics of gastric cancer and the low survival rate.

### EACR2024-0055

### Impact of COVID-19 pandemic on liver cancer treatments in Japan: A nationwide observational study using an interrupted time-series analysis

*M. Fujita[1,2], K. Nagashima[3], T. Fujisawa[1], K. Suzuki[1], T. Kasai[1], H. Hashimoto[1], K. Yamaguchi[1], Y. Onouchi[2], D. Sato[4], A. Hata[1]*

*[1]Chiba Foundation for Health Promotion and Disease Prevention, Department of Health Research, Chiba, Japan*

*[2]Chiba University Graduate School of Medicine, Department of Public Health, Chiba, Japan*

*[3]Keio University Hospital, Biostatistics Unit- Clinical and Translational Research Center, Tokyo, Japan*

*[4]Fujita Health University Graduate School of Medicine, Hospital and Health Administration, Toyoake, Japan*

**Introduction**

The onset of the COVID-19 pandemic compelled healthcare providers to deviate from established standards of care. While previous studies have reported the impact of the pandemic on cancer treatments, comprehensive nationwide studies are scarce. We aimed to identify the changes in the number of treatments for liver cancer during the pandemic using a nationwide insurance claims data in Japan.

**Material and Methods**

We used datasets from January 2015 to January 2021, which were prepared by Ministry of Health, Labour and Welfare by randomly extracting from a nationwide database. Targeted treatments for liver cancer included surgical resection, microwave coagulation therapy or radiofrequency ablation, embolization, and stereotactic body radiotherapy (SBRT). Using an interrupted time series analysis, we estimated changes in the number of treatments and counterfactual number, which was extrapolated from the trends in pre-pandemic numbers. This study was approved by the appropriate ethics review board and informed consent was not required owing to anonymized data. No competing interests exist.

**Results and Discussions**

The number of surgical resections for liver cancer significantly decreased in July 2020 (-218; 95% CI: -381 to -56). Conversely, the number of SBRTs significantly increased in both July 2020 (37; 95% CI: 6 to 68) and October 2020 (47; 95% CI: 16 to 78). Additionally, while the number of embolizations increased in July 2020, the change was not statistically significant. These trends suggest that SBRT and embolization partly replaced surgical resection for liver cancer. Consequently, the total number of treatments remained unchanged during the pandemic and was similar to the counterfactual numbers. This suggests that the number of treatments for liver cancer was maintained during the pandemic. In general, liver cancer is detected through follow-up examinations in patients infected hepatitis C virus (HCV) or hepatitis B virus (HBV), which are the primary causes of liver cancer. Considering the diagnostic route for liver cancer, the consistent number of liver cancer treatments observed in this study suggests that management of patients diagnosed with HCV or HBV infections before the pandemic continued as usual.

**Conclusion**

In Japan, replacement of surgical resection with alternative treatments for liver cancer appears to have been adopted to overcome the challenges posed by the pandemic, resulting in the maintenance of the number of treatments.

### EACR2024-0165

### Associations between peripheral whole blood cell counts derived indexes and cancer prognosis: An umbrella review of systematic reviews with meta-analyses of cohort studies

*Y. Zhang[1], F.H. Liu[1], Y.L. wang[1], J.X. Liu[1], C. Liu[2], H.H. Wang[3], T.T. Gong[2], Q.J. Wu[1]*

*[1]Shengjing Hospital of China Medical University, Department of Clinical Epidemiology, Shenyang, China*

*[2]Shengjing Hospital of China Medical University, Department of Obstetrics and Gynecology, Shenyang, China*

*[3]Shengjing Hospital of China Medical University, Department of Hematology, Shenyang, China*

**Introduction**

Although several studies have explored the associations between whole blood cell count (WBCC) derived indexes (neutrophil-to-lymphocyte ratio [NLR], platelet-to-lymphocyte ratio [PLR], and lymphocyte-to-monocyte

© 2024 The Authors. Molecular Oncology is published by John Wiley & Sons Ltd on behalf of Federation of European Biochemical Societies ratio [LMR]) and cancer prognosis, a summary of the accumulated evidence from these systematic reviews and meta-analyses is lacking. We aimed to consolidate evidence from systematic reviews and meta-analyses investigating the association between above peripheral WBCC derived indexes and cancer prognosis and assess the strength and validity of evidence.

**Material and Methods**

We systematically searched PubMed, EMBASE, Web of Science, and Cochrane Library from inception up to 9 March 2023 for systematic reviews with meta-analyses of cohort studies that investigated the associations between WBCC-derived indexes (NLR, PLR, and LMR) and cancer prognosis. The protocol was registered in PROSPERO (CRD42023487176). For each of the meta-analysis, random-effects models were applied to estimate summary effect sizes and 95% confidence intervals (CIs). The strength of the evidence was categorized into five levels (convincing, highly suggestive, suggestive, weak, and not significant).

**Results and Discussions**

A total of 694 meta-analyses from 224 articles were included in this umbrella review. There were 219 (97.8%) articles rated as moderate-to-high quality according to AMSTAR. There were four associations supported by convincing evidence, specifically, the association between PLR and overall survival in non-metastatic colorectal cancer (pooled hazard ratio [HR]=1.59; 95% CI: 1.32, 1.91), gastric cancer (HR=1.60; 95% CI: 1.41, 1.82), and esophageal cancer (HR=1.29; 95% CI: 1.18, 1.42), and the association between NLR and event-free survival in esophageal squamous cell carcinoma (HR =1.36; 95% CI: 1.21, 1.52). Meanwhile, there were 165 associations supported by highly suggestive evidence and 164 associations supported by suggestive evidence.

**Conclusion**

Our umbrella review summarized the existing evidence on the WBCC-derived indexes (NLR, LMR, and PLR) and cancer prognosis. Although these indexes are promising as markers of cancer prognosis, the direction of effect sizes is not completely consistent between studies, and further research is needed to assess causality and provide firm evidence.

### EACR2024-0182

### Plant-based diet indices and their interaction with ambient air pollution on the ovarian cancer survival: A prospective cohort study

*F. Cao[1], R. Wang[1], L. Wang[2], Y.Z. Li[1], Y.F. Wei[1], G. Zheng[1], Y.X. Nan[1], X. Meng[3], T.T. Gong[2], Q.J. Wu[1]*
*[1]Shengjing Hospital of China Medical University, Department of Clinical Epidemiology, Shenyang, China*
*[2]Shengjing Hospital of China Medical University, Department of Obstetrics and Gynecology, Shenyang, China*
*[3]Fudan University, School of Public Health- Key Lab of Public Health Safety of the Ministry of Education and N HC Key Lab of Health Technology Assessment, Shanghai, China*

**Introduction**

Ambient air pollution might serve as a prognostic factor for ovarian cancer (OC) survival, yet the relationships between plant-based diet indices (PDIs) and OC survival remained unclear. We aimed to investigate the associations of comprehensive air pollution and PDIs with OC survival and explored the effects of air pollution-diet interactions.

**Material and Methods**

The present study encompassed 658 patients diagnosed with OC. The overall plant-based diet index (PDI), the healthful PDI, and the unhealthful PDI (uPDI) were evaluated by a self-reported validated food frequency questionnaire. In addition, an air pollution score (APS) was formulated by summing the concentrations of particulate matter with a diameter of 2.5 microns or less, ozone, and nitrogen dioxide. Cox proportional hazard models were applied to calculate hazard ratios (HRs) and 95% confidence intervals (CIs) of overall survival (OS). The modifying effect of PDIs on the relationships between APS and OS was further examined by incorporating interaction terms.

**Results and Discussions**

Throughout a median follow-up of 37.60 (interquartile: 24.77–50.70) months, 123 deaths were confirmed. Comparing extreme tertiles, higher uPDI was associated with lower OS of OC (HR=2.06, 95%CI=1.30, 3.28; *P*-trend<0.01), whereas no significant association was found between overall PDI as well as hPDI and OC survival (*P*-trend > 0.05 for both). Higher APS ($HR_{\text{for per interquartile range}}$=1.27, 95%CI=1.01, 1.60) were significantly associated with worse OC survival, and the associations could be exacerbated by adhering to uPDI. Notably, an additive interaction was identified between combined air pollution and uPDI (*P* < 0.005 for high APS and high uPDI). We also found that adherence to overall PDI aggravated associations of air pollution with OC survival (*P*-interaction=0.006).

**Conclusion**

Joint exposure to various ambient air pollutants was significantly associated with lower survival among patients with OC, particularly for those who predominantly consumed unhealthy plant-based food.

### EACR2024-0209

### Racial and Gender Disparities in the Incidence of Polycythemia Vera: A Nationwide Analysis

*S. Javaid[1], K. Frasier[2], V. Li[3], J. Vinagolu-Baur[4], N. Yaguachi[4], E. Rodriguez[5], O. Castillo[6], R. Batista[4], K. Jonas[7], L. Palma[4]*
*[1]Wyckoff Heights Medical Center, Internal Medicine, Brooklyn, United States*
*[2]Nuvance Health/Vassar Brothers Medical Center, Medicine, Poughkeepsie, United States*
*[3]Lake Erie College of Osteopathic Medicine, Medicine, Erie, United States*
*[4]State University of New York- Upstate Medical University, Medicine, Syracuse, United States*
*[5]University of Missouri-Columbia- School of Medicine, medicine, Columbia, United States*
*[6]Dutchess Community College, Medicine, Beacon, United States*

© 2024 The Authors. Molecular Oncology is published by John Wiley & Sons Ltd on behalf of Federation of European Biochemical Societies

[7]University of Missouri-Columbia- School of Medicine, Medicine, Columbia, United States

**Introduction**

Polycythemia vera is a rare type of blood cancer characterized by the overproduction of blood cells in the bone marrow. Left untreated, it can lead to severe complications, recurrent hospitalizations, and reduced life expectancy. Limited research exists on gender, ethnic, and racial disparities in PV occurrence, prompting our analysis across diverse populations.

**Material and Methods**

We used the National Inpatient Sample 2020 to identify patients admitted with a primary or secondary diagnosis of Polycythemia vera. We analyzed baseline characteristics, including age, gender, income status, and certain comorbid conditions, to evaluate risk associated with PV. We employed a Log Binomial regression model for calculating the Risk ratios while accounting for confounding variables such as demographic characteristics of patients

**Results and Discussions**

A total of 20870 patients had PV. The mean age of patients with and without PV was 70+/-13 and 49+/-27 years, respectively. Females and males were 44.25% and 55.74%, respectively, p<0.001. Males had an increased risk of PV compared to females (RR=1.54(1.45-1.64); p<0.001). Compared to white females, white males had an increased risk of PV (RR=1.28(1.19-1.37); p<0.001). Black males (RR=0.69(0.58-0.82);p<0.001) and females (RR=0.46(0.38-0.55); p<0.001) as well as Hispanic males (RR=0.81(0.67-0.98); p=.03) and females (RR=0.44 (0.34-0.56); p<0.001) had a decreased Risk of PV compared to white females. Females of other races (RR=0.66(0.46-0.93); p=0.02) had a decreased risk, while males of other races (OR=1.21(0.93-1.56); p=0.14) and Asian males (RR=1.40(0.79-2.47); p=0.24) and females (RR=0.97(0.52-1.82); 0.94) had no difference in the risk of PV. Risk of PV increased with age (36-45: RR(3.85(2.82-5.26), p<0.001; 46-64: RR=7.14(5.42-9.39), p<0.001; >65: RR=9.95(7.49-13.21); p<0.001. Risk of PV also increased with higher Charlson comorbidity index (CI 1: RR=1.46(1.30-1.64); p<0.001); CI 2: RR=1.71(1.52-1.93), p<0.001; CI >/=3: RR=1.89 (1.68-2.12); p<0.001. Patients with Hypertension had a high risk of PV (RR=1.11(1.03-1.19); p=0.006) while patients with Diabetes (RR=0.68(0.63-0.74); p<0.001) and End Stage Renal Disease (RR=0.47(0.37-0.59); p<0.001) had lower risk of PV

**Conclusion**

White males have a higher risk of PV than other races, and the risk increases with age. Persistent disparities in PV are linked to factors such as lack of awareness and unequal access to healthcare. Identifying at-risk populations is crucial for reducing these disparities efficiently.

### EACR2024-0211
### Analyzing the Influence of Ethnicity and Age on Penile Cancer and the Risk factors analysis: A Nationwide Analysis of Inpatient sample

*S. Javaid[1], K. Frasier[2], V. Li[3], J. Baur[4], N. Yaguachi[4], E. Rodriguez[5], O. Castillo[6], R. Batista[4], K. Jonas[7], L. Palma[4]*

[1]Wyckoff Heights Medical Center, Internal Medicine, Brooklyn, United States
[2]Nuvance Health/Vassar Brothers Medical Center, Medicine, Poughkeepsie, United States
[3]Lake Erie College of Osteopathic Medicine, Medicine, Erie, United States
[4]State University of New York- Upstate Medical University, Medicine, Syracuse, United States
[5]University of Missouri-Columbia- School of Medicine, Medicine, Columbia, United States
[6]Dutchess Community College, Medicine, Beacon, United States
[7]University of Missouri-Columbia- School of Medicine-, Medicine, Columbia, United States

**Introduction**

Penile cancer is an extraordinarily infrequent and aggressive form of cancer that accounts for less than one percent of all male cancers and can frequently lead to hospitalizations. Despite this, limited research has been conducted on this topic in recent years, and there is a scarcity of data regarding age and ethnic disparities in penile cancer. Our study aimed to analyze the racial disparities and comorbidities associated with the risk of penile cancer.

**Material and Methods**

We used the National Inpatient Sample 2017-2020 to identify patients admitted with a primary diagnosis of penile cancer. We analyzed baseline characteristics, including age, income status, race, and certain comorbid conditions, to evaluate the risk associated with Penile cancer. We used a Log Binomial regression model for calculating the risk ratios after adjusting for confounding variables.

**Results and Discussions**

A total of 27,820,162 patients were assessed, and 2,710 had penile cancer. The mean age of patients with and without penile cancer was 65+/-13 and 49+/-27 years, respectively, p<0.001. White (59.66%) and Hispanics (21.78%) had the greatest incidence, followed by Black (9.85%), Asian (2.27%), Native Americans (0.95%), and other races (5.49%), p<0.001. Compared to white, Hispanic (RR=2.70(2.14-3.4); p<0.001) and males of other races (RR=2.29(1.53-3.43); p<0.001) had increased risk of Penile cancer. No difference was noted in the Black (RR=0.80(0.58-1.10); p=0.17, Asian (RR=1.06 (0.58-1.95); p=0.83) and Native Americans (RR=1.70 (0.63-4.58); p=0.29). The risk of penile cancer increased with age (36-45: RR=4.43(1.71-11.50), p=0.002; 46-64: RR=5.18(2.12-12.69), p<0.001; >65: RR=8.50(3.41-21.14), p<0.001. Obese patients had higher risk of developing penile cancer (RR=1.62(1.28-2.05); p<0.001), while patients with Diabetes (RR=0.52(0.42-0.63); p=0.001), Heart Failure (RR=0.25(0.18-0.35); p<0.001), Coronary Artery Disease (RR=0.44(0.34-0.56); p<0.001), and Hyperlipidemia (RR=0.68(0.56-0.84); p<0.001) had lower risk of penile cancer.

**Conclusion**

Racial and age-based disparities in penile cancer are multifaceted, stemming from a complex interplay of factors such as lack of awareness, unequal access to healthcare, and numerous other underlying causes. Recognizing the at-risk populations is crucial to develop

© 2024 The Authors. Molecular Oncology is published by John Wiley & Sons Ltd on behalf of Federation of European Biochemical Societies targeted interventions that can effectively reduce the incidence of these disparities. Further prospective studies are needed to validate the findings of our study.

### EACR2024-0221
### Exploring Ethnic and Sociodemographic Factors Impacting Access to CAR-T Therapy: A cross-sectional Study of Nationwide Data

*S. Javaid[1], K. Frasier[2], E. Rodriguez[3]*
*[1]Wyckoff Heights Medical Center, Medicine, Brooklyn, United States*
*[2]Nuvance Health/Vassar Brothers Medical Center, Medicine, Poughkeepsie, United States*
*[3]University of Missouri-Columbia- School of Medicine, Medicine, Columbia, United States*

**Introduction**

Chimeric antigen receptor T-cell therapy (CAR-T) has emerged as a promising approach for hematological malignancies, involving programming T-cells to target tumor-associated antigens. Limited research exists on cost estimates, affordability, and disparities in access across different population subgroups. We conducted an analysis to determine CAR-T availability and utilization disparities

**Material and Methods**

We utilized the National Inpatient Sample (2017-2020) to identify patients admitted for CAR-T. We analyzed the baseline characteristics like age, gender, insurance, income status, and several comorbidities to determine any disparities in the prevalence of CAR-T. Multivariate logistic regression analysis was utilized to determine the disparities in the likelihood of undergoing CART across various population subgroups.

**Results and Discussions**

A total of 5835 patients had CAR-T therapy. The mean age of patients was 55 years. Males and females were 59.13% vs 40.87%, respectively, p<0.001. Females were less likely to have CAR-T (OR=0.75(0.66-0.86), p<0.001. Compared to younger patients, older patients had a decreased likelihood of CAR-T (36-45: OR=0.57 (0.40-0.81), p=0.002; >65: OR=0.69(0.52-0.92), p= 0.012). A greater percentage of white patients had CAR-T as compared to blacks and Hispanics (75.12% vs. 67.08%, 16.03% vs. 6.64%, and 13.19% vs. 12.82%, respectively, p=0.006). Compared to white patients, black patients had a decreased likelihood of CAR-T (OR=0.47 (0.36-0.62), p<0.001), while Hispanics had an increased likelihood (OR=1.24(1.02-1.54), p=0.04). A greater percentage of patients with private insurance had CAR-T, while fewer patients with Medicare and Medicaid had CAR-T (52.85% vs. 30.13%, 33.87% vs. 41.98%, 10.16% vs. 23.6%, respectively, p<0.001). Compared to Medicare, patients with private insurance had an increased likelihood of CAR-T (OR=2.78(2.32-3.35), p<0.001). Patients in Large and teaching hospitals had an increased likelihood of CAR-T compared to smaller and nonteaching hospitals (OR=2.09(1.73-2.53), p<0.001) and (OR=30.27(14.38-63.72), p<0.001 respectively).

**Conclusion**

In conclusion, female and older patients had a decreased likelihood of CAR-T. White patients and patients with private insurance had an increased likelihood of CAR-T. Disparities exist in the incidence of CART therapy. It is crucial to address this issue, given the increasing utilization and wide-ranging benefits for various types of cancers, while also striving to ensure equity in healthcare.

### EACR2024-0265
### Unveiling the Varied Prevalence of Chronic Myeloid Leukemia (CML) in Hospitalized Patients: A Population Subgroup Analysis of National Inpatient Data

*S. Javaid[1], K. Frasier[2], K. Jonas[3]*
*[1]Wyckoff Heights Medical Center, Medicine, Brooklyn, United States*
*[2]Nuvance Health/Vassar Brothers Medical Center, Medicine, Poughkeepsie, United States*
*[3]University of Missouri-Columbia- School of Medicine, Medicine, Columbia, United States*

**Introduction**

Chronic myeloid leukemia (CML) is a myeloproliferative neoplasm that affects the blood and bone marrow and is characterized by increased granulocyte production. However, there is limited information about its prevalence across different population subgroups and identifying high-risk patient populations requiring hospitalization.

**Material and Methods**

We utilized the National Inpatient Sample (2019-2020) to identify the hospitalized patients with a primary diagnosis of CML. We compared the baseline patients' characteristics including age, sex, insurance status, income, race, and hospital characteristics to determine the disparities in the prevalence of CML. Multivariate regression analysis was used to determine the disparities in the odds of CML across different patient populations and the implicated associated risk factors.

**Results and Discussions**

A total of 6,280 patients were hospitalized with CML. The mean age of patients with CML was 52.94+/-19. Males and females with CML were 61.15% vs 38.85%, respectively, p<0.001. Females were less likely to have CML (OR=0.62(0.54-0.70), p<0.001). Compared to Medicare, patients with private insurance (OR=1.54 (1.28-1.84), p<0.001) and no insurance (OR=1.49(1.11-1.98), p=0.007) were more likely to be admitted with CML while no difference was noted for patients with Medicaid (OR= 1.13(0.91-1.40), p=0.23). Compared to Whites, Hispanics (OR=1.56(1.30-1.85), p<0.001) and patients of other races (OR=1.48(1.09-2), p=0.01) were more likely to have CML, while no difference was noted for Blacks (OR=1.02(0.86-1.22), p=0.77). Patients in large, urban, and teaching hospitals were more likely to be admitted with CML compared to small, rural, and nonteaching hospitals (OR=1.58(1.33-1.87),p<0.001; OR=1.69(1.08-2.64),p=0.02; OR=2.04(1.65-2.53), p<0.001 respectively). Patients with Hypertension, Diabetes, and Coronary Artery Disease had a decreased likelihood of being admitted with CML (OR=0.71(0.62-0.83), p<0.001; OR=0.31(0.26-0.37), p<0.001; OR=0.53 (0.43-0.65), p<0.001 respectively).

**Conclusion**

© 2024 The Authors. Molecular Oncology is published by John Wiley & Sons Ltd on behalf of Federation of European Biochemical Societies

Hispanics and other races had a higher risk of admission with CML compared to Whites and Blacks. Patients in large, urban, and teaching hospitals were more likely to be admitted with CML, as well as have private insurance. There are significant disparities in the prevalence of hospitalized CML patients, highlighting the need for equitable access to healthcare for this population.

### EACR2024-0267
### Accessibility to surgical resections of pancreatic cancers and the disparities across various population subgroups in the healthcare system

*S. Javaid[1], K. Frasier[2], E. Rodriguez[3]*
*[1]Wyckoff Heights Medical Center, Medicine, Brooklyn, United States*
*[2]Nuvance Health/Vassar Brothers Medical Center, Medicine, Poughkeepsie, United States*
*[3]University of Missouri-Columbia- School of Medicine, Medicine, Columbia, United States*

**Introduction**
Pancreatic cancer is the fourth leading cause of cancer-related deaths in the United States. Surgical resection is the only curative option, but it's limited to a small percentage of patients due to advanced and metastatic disease. Access to surgical resections for pancreatic cancers across patient populations and healthcare systems is poorly understood

**Material and Methods**
We used the National Inpatient Sample (2019-2020) to identify the patients hospitalized for the surgical resection of Pancreatic cancer (both local and advanced). We compared the baseline patients' characteristics, including age, sex, insurance status, income, race, and hospital characteristics, to determine the disparities in the prevalence of pancreatic resections. Multivariate regression analysis was used to determine the disparities in the odds of surgical procedures across different patient populations and the implicated associated risk factors.

**Results and Discussions**
A total of 35650 patients with Pancreatic cancer underwent surgical resection . The mean age of patients with surgery was 66+/-11. Males and females were 49.72% vs 50.28% respectively, p<0.001. Compared to males, females had a higher likelihood of undergoing surgery (OR=1.10(1.05-1.16),p<0.001). Compared to white patients, Blacks (OR=0.63(0.58-0.69),p<0.001) and Hispanic (OR=0.90(0.82-0.99),p<0.001) had decreased likelihood of surgery . Compared to Medicare, patients with Medicaid had a decreased likelihood of undergoing surgery (OR=0.71(0.63-0.80),p<0.001, while patients with private insurance had an increased likelihood (OR=1.99(1.86-2.13),p<0.001). The likelihood of surgery increased with the increase in income ( $65,000-$85,999: OR=1.34(1.24-1.44),p<0.001; >$86,000: OR=1.64(1.52-1.77),p<0.001). Patients in the medium and large hospitals had an increased likelihood of surgery compared to small hospitals (OR=2.13 (1.90-2.39), p<0.001 and OR=5.10(4.60-5.64),p<0.001, respectively). Similarly, patients in the urban and teaching hospitals had a higher likelihood of surgery as compared to rural and nonteaching hospitals (OR=3.15(2.35-4.22),p<0.001 and OR=4.44(3.99-4.93),p<0.001) respectively.

**Conclusion**
Females, white, older patients, and those with private insurance were more likely to undergo surgical resection for pancreatic cancer, and they were also more likely to be admitted to medium and large urban teaching hospitals. Access to surgical treatments raises concerns about equitable healthcare and resource utilization for ensuring equal care.

### EACR2024-0337
### Detection of colorectal cancer by tumour-homing probiotic bacteria

*G. Radford[1], J. Ng[1], J. Goyne[2], J. Wright[2], L. Vrbanac[1], S. Hasan[1], K. Barratt[1], E. Thomas[1], D. Worthley[2], S. Woods[1]*
*[1]The University of Adelaide, School of Medicine, Adelaide, Australia*
*[2]South Australian Health and Medical Research Institute, Precision Medicine, Adelaide, Australia*

**Introduction**
Colorectal cancer (CRC) is the 2nd leading cause of cancer deaths worldwide. Contributing to lethality of advanced disease are shortfalls in diagnosis. Genetically modified, tumour-homing probiotic bacteria, *Escherichia coli Nissle* 1917 (EcN), present a novel potential solution to improve early detection of CRC. Over the last decade, convincing preclinical studies have shown that EcN selectively colonise tumour tissue, however, how or why this occurs is yet to be elucidated. We've shown, in our clinically relevant orthotopic model of CRC pre-disposition, with orally administered EcN, that size matters. Here, to investigate colonization determinates, we expand to a breast tumour model with variation in oxygenation status across the tumour and bacteria with different oxygen requirements. We envisage a mechanistic understanding of colonization will enable the optimization of bacteria for tumour detection.

**Material and Methods**
Mice were orthotopically injected with CRC organoids, with colonoscopies utilised to grade tumour growth. Mice were orally gavaged with $10^{10}$ colony forming units (CFU) of luciferase-expressing EcN twice over a three-day period. Five-days post-injection mice were imaged in vivo, injected with Hypoxyprobe, and tissues were harvested. In a separate cohort, mice were subcutaneously injected with mammary carcinoma cells. Once tumours reached 500mm$^3$, mice were intravenously injected with 5 x $10^8$ CFU of luciferase-expressing *Pseudomonas aeruginosa*. At seven-days post-injection mice were imaged in vivo, injected with Hypoxyprobe, and tissues were harvested. Controls included PBS-treated tumour-bearing mice.

**Results and Discussions**
CFU assays quantifying live bacteria confirmed CRC tumours (grade 3/4, median tumour diameter of 2mm +/-1.2mm) had significantly greater colonisation compared to smaller tumours (grade 1/2). Similarly for the mammary carcinoma model, CFU assays confirmed significant colonization in PA-treated tumour compared to controls. In both models, bacterial localisation and

© 2024 The Authors. Molecular Oncology is published by John Wiley & Sons Ltd on behalf of Federation of European Biochemical Societies degree of hypoxia was visualised through immuno-fluorescent staining and confirmed via RNAscope.

**Conclusion**

Our results demonstrate that size dictates colonization in a clinically relevant CRC model. Furthermore, we are the first to show aerobic bacterium, Pseudomonas aeruginosa, can colonise tumours. For mechanistic understanding, we explored the relationship between hypoxia and bacteria location. We envisage this knowledge could help to optimize engineered bacteria for use as a screening tool for cancer patients.

### EACR2024-0380

### Evaluation of the Predictive Power of the Assessment of Different NEoplasias in the adneXa (ADNEX) Model for Preoperative Ovarian Cancer Risk in Adnexal Masses: A Diagnostic Accuracy Meta-analysis

*M.K. Elfaituri*[1], *A. Khaled*[1], *H. Faraj*[1]

[1]*University of Tripoli, Faculty of Medicine, Tripoli, Libya*

**Introduction**

Ovarian cancer is recognized as one of the most lethal gynecological malignancies, emphasizing the necessity for early and accurate identification. The precise differential diagnosis of adnexal masses is pivotal in this context. The Assessment of Different NEoplasias in the adneXa (ADNEX) model, created by the International Ovarian Tumor Analysis (IOTA), is designed to classify these masses preoperatively. This study aims to examine the performance and predictive accuracy of the ADNEX model in preoperative ovarian cancer risk estimation within adnexal masses.

**Material and Methods**

We systematically reviewed studies published in PubMed, EMBASE, and Cochrane Library databases until June 2023, utilizing the ADNEX model to predict ovarian cancer in adnexal masses. For the statistical treatment of data, we used R software (version 4.0.3) with the mada package, allowing for the pooling of metrics such as sensitivity, specificity, estimates of the false-positive rate, the diagnostic odds ratio, along with the positive and negative Likelihood Ratios (LRs).

**Results and Discussions**

The meta-analysis comprised 8 studies, with a total of 4985 patients, of whom 1551 were confirmed diagnosed with ovarian cancer. The ADNEX model demonstrated a high pooled sensitivity of 92.5% (95% CI: 89.5-94.6%, I2=59.7%), indicating strong accuracy in correctly identifying patients with ovarian cancer. Conversely, the false-positive rate estimate was relatively low at 17.3% (95% CI: 13.4-22.1%), showing the model's efficiency in minimizing incorrect cancer diagnosis in patients without the disease. The second part of our analysis revealed a moderate-to-high specificity of 82.7% (95% CI: 77.9-86.6%, I2=88%), albeit with considerable heterogeneity across studies. The ADNEX model displayed high discriminative performance, as evidenced by a diagnostic odds ratio of 58.76 (95% CI: 36.69-94.09). Positive and negative Likelihood Ratios (LRs) were found to be 5.35 (95% CI: 4.16-6.88) and 0.09 (95% CI: 0.06-0.13), respectively, suggesting the model's effectiveness in modifying the probability of having ovarian cancer based on a positive or negative result.

**Conclusion**

The findings of this study underscore the substantial predictive capability of the ADNEX model in offering a dependable preoperative risk estimation of ovarian cancer in patients presenting with adnexal masses. This prediction accuracy could prove instrumental in guiding clinical decision-making and developing patient-specific treatment strategies.

### EACR2024-0398

### Familial risk of colorectal cancer: Frequency of colorectal polyp diagnoses in relatives as important as number of relatives with polyp

*Y. Hu*[1,2], *E. Kharazmi*[1,3], *Q. Liang*[1,2], *K. Sundquist*[4,5], *J. Sundquist*[3,5], *M. Fallah*[1,3,6]

[1]*German Cancer Research Center DKFZ, Division of Preventive Oncology, Heidelberg, Germany*

[2]*Heidelberg University, Medical Faculty Heidelberg, Heidelberg, Germany*

[3]*Lund University, Center for Primary Health Care Research, Malmö, Sweden*

[4]*Lund University, Center for Primary Health Care Research-, Malmö, Sweden*

[5]*Shimane University, Center for Community-based Healthcare Research and Education CoHRE, Izumo, Japan*

[6]*University of Bern, Institute of Primary Health Care BIHAM, Bern, Switzerland*

**Introduction**

The weak recommendation for screening relatives of patients with benign colorectal polyp is based on low- or very low-quality evidence. We aimed to provide high-quality evidence on the risk of colorectal cancer (CRC), especially early-onset CRC (before age 50), by the frequency of polyp diagnoses in family members, which has not been studied.

**Material and Methods**

We leveraged data from nationwide Swedish family-cancer datasets (1964-2018) to calculate standardized incidence ratios (SIRs) for individuals with a family history of polyp, stratified by frequency of polyp diagnosis (once, ≥2 times of polyp diagnoses), number of relatives with colorectal polyp, and youngest age at polyp diagnosis in relatives.

**Results and Discussions**

We followed up 12,411,107 individuals for up to 54 years (median=31 years). Compared with the risk in individuals with no family history (N=142,234), the risk of lifetime CRC was 1.35 times in those with 1 first-degree relative (FDR) with one-time colorectal polyp diagnosis (95%CI=1.32-1.38, N=11,035; early-onset CRC, SIR=1.44, 95%CI=1.34-1.55, N=742). The risk was significantly higher in individuals with 1 FDR with ≥2 times of polyp diagnoses (lifetime SIR=1.82, 95%CI=1.76-1.88; early-onset SIR=2.27, 95%CI=1.99-2.58). A rather similar risk was observed for individuals with ≥2 FDRs with one-time colorectal polyp diagnosis (lifetime SIR=1.89, 95%CI=1.73-2.06; early-onset SIR= 2.16, 95%CI=1.55-2.93). Individuals with ≥2 FDRs with

© 2024 The Authors. Molecular Oncology is published by John Wiley & Sons Ltd on behalf of Federation of European Biochemical Societies

≥2 times of polyp diagnoses had a 2.44-fold lifetime risk (95%CI=2.20-2.69) and a 3.92-fold early-onset risk (95%CI=2.83-5.30). Younger age at polyp diagnosis in FDRs was associated with an increased risk of CRC. A family history of polyp in second-degree relatives was important only when there were multiple polyp diagnoses.

**Conclusion**

Frequency of colorectal polyp diagnosis in relatives is associated with risk of CRC, particularly early-onset CRC, independent of number of relatives with polyp and youngest age at polyp diagnosis. Frequency of colorectal polyp diagnosis in relatives should be considered as important as number of relatives with colorectal polyp when developing CRC screening strategy.

### EACR2024-0465
### Utility of Liquid Biopsy Based HPV (Human Papilloma virus) cell free (cf) DNA detection for the prognosis and diagnosis of Cervical Cancer

*A. Gupta[1], R. Chauhan[2], A. Shankar[3], D.N. Sharma[3], V. Suri[4], M. Singh[2]*
*[1]All India Institute of Medical Sciences, Medical Oncology, New Delhi, India*
*[2]All India Institute of Medical Sciences- New Delhi, Medical oncology, New Delhi, India*
*[3]All India Institute of Medical Sciences- New Delhi, Radiation Oncology, New Delhi, India*
*[4]All India Institute of Medical Sciences- New Delhi, Department of Neuropathology, New Delhi, India*

**Introduction**

Recent advancements in analyzing blood samples for circulating tumor cells or cell-free circulating tumor DNA (ctDNA) suggest that liquid biopsies hold promise for cancer detection and monitoring. Human papillomavirus (HPV) cell free (cf) DNA emerges as a distinctive marker for prognostic assessment in high-risk HPV-related cancers. However, the detection of circulating markers for cervical cancer (CC) demands highly sensitive techniques capable of quantifying circulating human papillomavirus DNA. In this study, we employed the highly sensitive droplet digital PCR (ddPCR) approach to detect and quantify circulating human papillomavirus DNA in plasma from cervical cancer patients at baseline (chemo/radiotherapy naïve) and subsequent follow-ups, aiming to validate its utility as a prognostic marker in CC.

**Material and Methods**

Blood sera were obtained from 60 patients diagnosed with cervical cancer (Stage I-IV) at AIIMS Delhi, both at baseline and after a 3-month treatment period. Additionally, 10 healthy controls were included. Plasma was separated out and stored at -80°C. cf DNA was extracted from 1 ml of plasma using Promega kit. Furthermore, cfDNA from 35 patients were analyzed to identify the presence of circulating high-risk Human Papillomavirus (HPV), specifically HPV16 and HPV18 cfDNA, using ddPCR.

**Results and Discussions**

The baseline median concentration of cf DNA in CC patients was approximately 9.2 ng/uL. After 3 months of treatment, there was a decrease in cfDNA levels, with a median concentration of 7 ng/ul. In comparison, healthy controls have a median ccf DNA concentration of 6.95 ng/uL. To identify low-copy-number HPV cfDNA, we utilized ddPCR-based screening on processed samples. The result showed a positive detection rate of 56% for circulating HPV18 DNA and 60% for circulating HPV16 DNA in CC patient's sera. We also found that HPV16 is more prevalent as compared to HPV18. Quantitative evaluation showed significant correlations between ccfHPV16 DNA level and tumor size demonstrating its utility as a promising marker to detect disease burden.

**Conclusion**

Based on these findings, we suggest that low copy number of HPV cell free DNA can predict chemo-radiotherapy outcome in CC patients across all stages.

### EACR2024-0532
### GRP78 as a predictive marker for invasiveness in triple-negative breast cancer

*M.B. Alvarez-Elizondo[1], A. Raiter[2], R. Yerushalmi[2], D. Weihs[3]*
*[1]Technion - Israel Institute of Technologu, Faculty of Biomedical Engineering, Haifa, Israel*
*[2]Tel Aviv University, Faculty of Medicine, Tel Aviv, Israel*
*[3]Technion-Israel Institute of Technology, Biomedical Engineering, Haifa, Israel*

**Introduction**

Metastasis continues to be the leading cause (i.e. 90%) of cancer-related deaths. Chemoresistance contributes significantly to this statistic, especially in metastatic, triple-negative breast cancer (TNBC). Improved understanding of molecular drivers of the metastatic cascade is crucial. Current chemotherapy regimens for breast cancer include doxorubicin and paclitaxel that induce various effects, such as the unfolded protein response (UPR) that is regulated by the 78-kDa glucose-regulated protein (GRP78). The GRP78 localizes to the surface of tumor cells, and may be secreted to the tumor microenvironment. The GRP78 is also associated with metastatic disease, yet the connection between expression level and invasiveness is yet unknown.

**Material and Methods**

We evaluate chemotherapy effects on TNBC cell-surface GRP78 expression and on cell mobility and invasiveness. For this purpose, we combine the in vitro Boyden chamber migration assay with our innovative, clinically relevant, mechanobiology-based invasiveness assay to evaluate the effect of doxorubicin on the invasiveness of GRP78 expressing TNBC cells.

**Results and Discussions**

Our results show concentration-dependent chemotherapy-induced enhancement of the cell-surface GRP78 expression in two TNBC cell types. We demonstrate reduction in sample mobility (via Boyden chamber assay) following treatment with 1 µg/ml doxorubicin for 24 hours. Furthermore, the treatment produced GRP78-positive and negative subpopulations, where the GRP78-negative cells were notably more invasive than GRP78-positive cells, likely indicating their higher metastatic potential. Our findings reveal that cell-surface GRP78

© 2024 The Authors. Molecular Oncology is published by John Wiley & Sons Ltd on behalf of Federation of European Biochemical Societies expression after chemotherapy is a potentially promising, prognostic indicator for increased metastasis in TNBC.

**Conclusion**

The GRP78 may provide a predictive marker for chemoresistance and invasiveness, which could improve therapy choices and outcomes in cancer patients.

### EACR2024-0599
### Finding patient-reported deterrents to adjunct Breast Cancer screening among patients with dense breast tissues. A cross sectional study in Pakistan

*A. Fatima*[1]
[1]*Sir GangaRam Hospital, Department of Oncology, Lahore, Pakistan*

**Introduction**

Women with dense breast tissues are at a greater risk of developing cancer because of reduced sensitivity of mammography. In order to address this issue, theres need to incorporate adjunct screening modalities for this particular population. We aim to identify patient's characteristics such as age, socioeconomic status, personal risk of breast cancer etc associated with patient cited concerns about adjunct breast screening modalitie

**Material and Methods**

A cross sectional study was conducted from June 2019 to August 2020. The preferences and attitudes of women with dense breasts toward adjunct breast cancer screening were evaluated. Patient survey responses regarding whether various factors would deter patients from adjunct breast cancer screening and regarding which of three hypothetical breast screening examinations they would prefer were extracted from the survey data. Patient demographic and clinical data were obtained by re-view of patient medical records.

**Results and Discussions**

Surveys were completed by 700 women (median age, 53.0 years) with dense breasts. Lower con dence in the sensitivity of mammography of dense breasts was independently associated with lesser concern about adjunct screening examination time (1 divided by adjusted odds ratio [1/AOR], 0.55 [95% CI, 0.34–0.89]), additional imaging that could result (1/AOR, 0.51 [95% CI, 0.31–0.85]), and greater preference for a more sensitive hypothetical screening examination (1/AOR, 1.85 [95% CI, 1.20–2.86]). Concern about examination cost, the most commonly cited deterrent to adjunct screening (66.9%), was independently associated with younger age (1/AOR, 1.45 [95% CI, 1.01– 2.08]) but not with imputed socioeconomic variables or other tested variables. Younger age was also associated with lesser concern about pain (1/AOR, 0.69 [95% CI, 0.48–0.99]), additional imaging that could result (1/AOR, 0.48 [95% CI, 0.31–0.76]), and IV contrast administration (1/AOR, 0.56 [95% CI, 0.37–0.83]).

**Conclusion**

Our study exhibits that patient concerns about adjunct breast cancer screening may be mitigated by educating patients about the limitations of the sensitivity of mammography of dense breasts and by exploring age-specific ways to address the financial impact of adjunct screening.

### EACR2024-0849
### Brain Metastasis is Fatal: A Review of Mortality in a Cohort of Patients with Metastatic Breast Cancer in South West Nigeria

*A. Alabi*[1], *A. Lawal*[2], *O. Fatiregun*[3], *T. Falowo*[4], *A. Joseph*[1], *B. Adegboyega*[1], *A. Sowunmi*[1]
[1]*NSIA-LUTH Cancer Centre, Department of Radiotherapy & Oncology, Lagos, Nigeria*
[2]*College of Medicine- University of Lagos, Department of Surgery, Lagos, Nigeria*
[3]*Lagos University Teaching Hospital, Department of Psychiatry, Lagos, Nigeria*
[4]*CancerAware Nigeria, N/a, Lagos, Nigeria*

**Introduction**

Breast cancer accounts for the highest age-standardized mortality in Western Africa according to GLOBOCAN 2020. The mortality rate in metastatic breast cancer (MBC) depends on various factors, including the individual's age, overall health, tumor characteristics, treatment options, and access to healthcare. The study aims to assess the causes of mortality in patients with metastatic breast cancers in NSIA-LUTH Cancer center in Lagos.

**Material and Methods**

This prospective cohort study was conducted among 313 patients with metastatic breast cancer between 2021 and 2022. Sociodemographic and Clinical information was collected and entered into SPSS version 22. Patients were monitored for 1 - 3 years following recruitment and data analysed. The patients were followed up during the period of study by a patient navigation program via communication by phone and or clinic attendance. We calculated adjusted hazard ratios (HRs) and 95% confidence intervals (CIs) using Cox proportional hazards regression for mortality. Ethical approval was obtained from institutional HREC.

**Results and Discussions**

A total of 313 patients with metastatic breast cancer were recruited and followed up for 1-3 years. The mean age of patients was 49.8 ± 13.5 years. Majority of participants 309 (98.7%) were female. One hundred and sixty-two (51.8%) had Triple Negative Breast cancer with majority (66%) seen in ages older than 40years. Metastases in the Lungs occurred in 168(53.8%) followed by 152 (48.7%) with Spine metastasis. The brain was the site with the highest recorded death, with 68.3% of all patients with brain metastasis dead within 1 year of diagnosis. The Pearson coefficient was 0.13. More than half of the patients 13 (61.9%) with Her 2- enriched histologic type died within the study period. More than half of the patients 175(56%) died during the period of study due to disease progression. Comorbidities such as Hypertension and Diabetes Mellitus were not seen to have affected the risk of death as compared to studies by Misganaw et al that reported having one or more co-morbidities being linked to significantly increasing mortality rate.

**Conclusion**

The mortality rate in this study was high owing to several factors. Treatment options are now evolving targeting the use of novel drugs to focus on managing advanced

© 2024 The Authors. Molecular Oncology is published by John Wiley & Sons Ltd on behalf of Federation of European Biochemical Societies disease, thereby extending survival, and improving quality of life.

### EACR2024-0871
### Metabolomic risk prediction of incidence of 17 cancers in half a million individuals

K. Schut[1], S. Kerminen[1], L. Jostins-Dean[1], P. Würtz[1], J. Barrett[1]
[1]*Nightingale Health Plc., Health Insights, Helsinki, Finland*

**Introduction**
Metabolomic profiles are increasingly being used in cancer prediction and aetiology studies. We recently completed metabolomic profiling of blood samples from half a million UK Biobank participants. In this study, we use these newly available data to evaluate how well metabolomic risk scores predict the incidence of 17 different cancers and compare them to standard risk factors.

**Material and Methods**
We considered incident cancers in the UK Biobank with at least 500 cases within 10 years of the blood sample. In half of the data, we trained predictive incidence models using Cox proportional hazards regressions and LASSO regularisation with 10-fold cross-validation on combinations of age, sex, 38 nuclear magnetic resonance-based metabolomic biomarkers, and a set of standard risk factors: body mass index (BMI), weekly alcohol intake and pack years of smoking. In the test set, we calculated metabolomic risk scores and computed correlations between them. To assess the performance of the scores, we computed area under the receiver operating characteristic (ROC) curves (AUC) and hazard ratios (HR) for 1, 2, 5 and 10 years of follow-up.

**Results and Discussions**
Out of the 17 evaluated cancer types, 12 were significantly predicted by metabolomic risk scores, independent from age and sex. Within a 1-year follow-up, individuals in the highest risk decile of the scores for kidney cancer and lung cancer had a HR of 8.99 (95% CI = 4.40 – 18.37, p = 1.75E-09) and 7.21 (95% CI = 5.02 – 10.34, p = 8.72E-27) compared to the remaining 90%, respectively. Within 2 years, for multiple myeloma and leukaemia, these HRs were 5.92 (95% CI = 3.08 – 11.40, p = 1.00E-07) and 5.90 (95% CI = 3.60 – 9.67, p =1.92E-12), respectively. The metabolites increased AUCs on top of standard risk factors for several cancer types, including kidney cancer (+0.02), liver cancer (+0.07), leukaemia (+0.03), multiple myeloma (+0.07) and non-Hodgkin lymphoma (+0.03) 5 years into the future. We observed weak to no metabolomic prediction for incident prostate, ovarian, brain or breast cancer, or malignant melanoma.

**Conclusion**
Metabolomic risk scores can be used as risk predictors in a range of cancers, independent from age, sex, BMI, alcohol use and smoking behaviour. Such scores also may aid in screening, monitoring and personalised treatment decision-making.

### EACR2024-0966
### A systematic review on tobacco use among Indian tribal population

D.L. Francis[1], P.K. Jena[2]
[1]*School of Public Health- KIIT University- Odisha-India, Public Health, Bhubaneswar, India*
[2]*School of Public Health- KIIT University, Public Health, Bhubaneswar, India*

**Introduction**
Tobacco use is primarily responsible for the global economic burden of death and disease, which is currently connected to around 8,000,000 deaths per year, with approximately 80% of these fatalities occurring in low- and middle-income economies. The goal of this study was to conduct a comprehensive assessment of current literature on tobacco use among Indian tribal populations in order to determine the prevalence, distribution, and factors influencing tobacco use. These data are required to develop and adapt control methods targeted at reducing tobacco use among this disadvantaged population and preventing long-term illnesses.

**Material and Methods**
A systematic review of evidence on tobacco usage among Indian tribal populations was done. This study used relevant articles published between 2005 and 2023 in PubMed, Crossref, Google Scholar, and Web of Science. We considered studies that reported the prevalence of tobacco use among Indian tribes in our review using the PRISMA standards.

**Results and Discussions**
A total of 14 studies were reviewed, with a total population of 28,572 people. Tobacco consumption rates ranged from 12% to 42.5%. In gender-specific research, female smoking tobacco use ranged from 1.2% to 12%, whereas male smoking tobacco use ranged from 15% to 52%. Smokeless tobacco consumption, on the other hand, was quite common among the indigenous community. Lack of information about oral health, deeply ingrained dental beliefs, and restricted access to health services were the most significant risk factors for tobacco use.

**Conclusion**
In order to combat the tobacco use, further research into better methods and research-based regulations and workable quitting strategies must be prioritized. Campaigns promoting tobacco cessation and abstinence are advised in this review as a definite way to lessen the negative effects of cigarette smoking and tobacco misuse.

### EACR2024-1103
### Assessing the impact of additional rounds of breast cancer screening after the age of 64 on health outcomes using NHS Breast Screening Programme data

H. Russell[1], S.M. Ingle[1], B. Morrison[2], K. Freeman[3], R. Given-Wilson[4], M.G. Wallis[5], J.A.C. Sterne[1,6,7], S. Taylor-Phillips[3]
[1]*University of Bristol, Bristol Medical School Population Health Sciences, Bristol, United Kingdom*
[2]*University of Birmingham, Institute of Applied Health Research, Birmingham, United Kingdom*
[3]*University of Warwick, Warwick Medical School, Coventry, United Kingdom*
[4]*St George's University Hospitals NHS Foundation*

© 2024 The Authors. Molecular Oncology is published by John Wiley & Sons Ltd on behalf of Federation of European Biochemical Societies

Trust, Radiology Department- St George's Hospital, London, United Kingdom
[5]Cambridge University Hospitals NHS Trust, Cambridge Breast Unit and NIHR Cambridge Biomedical Research Centre, Cambridge, United Kingdom
[6]Health Data Research UK, South-West, Bristol, United Kingdom
[7]NIHR, Bristol Biomedical Research Centre, Bristol, United Kingdom

**Introduction**

In England, the NHS Cancer Plan extended the age range of breast screening from age 50-64 years to 50-<71 years between 2000-2006. However, we do not know which strategy gives most benefit and least harm. Randomised trials would provide strongest evidence for the causal effect of additional screening on outcomes, but are impractical and costly. In the absence of trial data, we used modern methods for emulating trials within observational data to assess the effect of extending screening age on outcomes.

**Material and Methods**

Data from the English breast screening programme were used to emulate the target trial evaluating the effect of two additional rounds of routine screening after the age of 64, on outcomes including breast cancer diagnosis and breast cancer mortality. Follow-up was available up to 2018. Women were eligible for inclusion in the target trial when aged 64-68, if they had previously been invited to screening. We assessed eligibility among women in the database for each person-month between 2001-2008. Women were assigned to the screening strategy that was compatible with her data. The effect of additional screening on outcomes was assessed via pooled logistic regression with stabilised inverse probability weights for the probability of being invited to additional screening. This weighting procedure balances baseline and time-varying covariates between the screening groups.

**Results and Discussions**

267,512 women were eligible for inclusion. 120,840 received an invitation to screening between their first date of eligibility and their 68$^{th}$ birthday (45.2%). Those who received additional invitations were slightly younger, attended more of their prior invitations to screening and were less likely to have a prior diagnosis of breast cancer. Among those who received additional invitations (median follow-up 12.1 years; interquartile range (IQR) 5.7-13.5), there were 4,722 diagnoses of breast cancer (3.9%) and 894 breast cancer deaths (0.7%). Among those who received no additional invitations (12.8 years; IQR 1.5-14.0) there were 4,768 diagnoses (3.3%) and 1,238 breast cancer deaths (0.8%). Compared with an upper age limit of 64 for invitations to screen, the weighted hazard ratios (95% confidence interval (CI)) for an increased upper age limit of 70 were 1.21 (1.16, 1.26) for breast cancer diagnosis, and 0.97 (0.90, 1.06) for breast cancer death.

**Conclusion**

Increasing the upper age limit for invitations to screening increased breast cancer diagnoses, though did not have a marked effect on breast cancer death.

### EACR2024-1117
### Untargeted metabolomic signature of dairy consumption is associated with reduced rate of colorectal cancer

D. Wu[1], E. Courtois[1], C. Chatziioannou[1], P. Keski-Rahkonen[1], M. Gunter[2], P. Ferrari[1], M. Jenab[1], V. Viallon[1]
[1]International Agency for Research on Cancer, Nutrition and Metabolism Branch, Lyon, France
[2]Imperial College London, Faculty of Medicine, London, United Kingdom

**Introduction**

Colorectal cancer (CRC) incidence ranks third in Europe and is projected to rise. Modifiable dietary patterns such as dairy consumption have been associated with a reduced risk of CRC, but the mechanisms behind this association are not well understood. Current hypotheses include chemoprotective actions of calcium, or the effect on the initiation of CRC through fatty acids or bile acids. Metabolomics techniques are a promising tool for the discovery of novel biomarkers of dietary exposures and associated metabolic mechanisms. In this study, we used available untargeted metabolomics data to identify a signature of dairy consumption and study its association with risk of CRC.

**Material and Methods**

Untargeted metabolomics (LC-MS/MS) was measured in the plasma (serum) obtained prior to CRC diagnosis of 1112 individuals and matched cancer-free participants of an established nested case-control study of colorectal cancer within the European Prospective Investigation into Cancer and Nutrition cohort. Controls were matched on study centre, sex, age, follow-up time, and blood sample time. A metabolic signature of dairy consumption was constructed with l1-penalized regression and selected using 5-fold cross-validation, after adjusting for age, BMI, country of residence, fasting status, smoking, sex, and height. Hazard ratios and 95% confidence intervals (CIs) for the association of the overall metabolic signature and individual metabolites with CRC were assessed with conditional logistic regression controlled for age, sex, dairy products consumption, BMI, smoking, physical activity, and level of education.

**Results and Discussions**

Using measurements on 930 metabolic features acquired in positive mode in the nested CRC study, we identified a metabolic signature of dairy consumption that comprised eight features and was moderately correlated with dairy (r = 0.28, 95% CI [0.16, 0.40]). Individual features were not strongly associated with CRC, but the composite signature was significantly inversely associated with risk of CRC (HR = 0.86, 95% CI [0.75, 0.99]). The metabolic signature included 2-hydroxy-3-methylbutyric acid which was found to be negatively associated with dairy intake and has been previously found to be positively associated with alcohol intake, risk of hepatocellular carcinoma, and pancreatic cancer.

**Conclusion**

An eight-metabolite signature of total dairy intake may comprise candidate metabolites which underlie metabolic mechanisms of the inverse association of dairy consumption on CRC.

### EACR2024-1153
### Efficient enrichment of plasma cells for

© 2024 The Authors. Molecular Oncology is published by John Wiley & Sons Ltd on behalf of Federation of European Biochemical Societies

### disease monitoring at MGUS and MRD stages

A. Johni[1], L. Yu[2], S. Sa[2]
[1]Applied Cells Inc., Research and Development, Southwater, United Kingdom
[2]Applied Cells Inc., Research and Development, Santa Clara, United States

**Introduction**

Plasma cell characterization in bone marrow is crucial for diagnosing and monitoring hematologic cell malignancies like multiple myeloma (MM). Plasma cells in MM patients vary in proportion from <0.01% up to 100%, depending on the disease stage. The accurate detection and analysis of plasma cells in both MGUS (Monoclonal Gammopathy of Undetermined Significance) and MRD (Minimal Residual Disease) stages are especially important for understanding disease progression, tailoring treatment strategies, and predicting patient outcomes. Additionally, for less invasive sample collection, circulating plasma cells (CPCs) in peripheral blood have become an important prognostic marker in MM patients, even though the CPC burden in peripheral blood is reported to be >100-fold lower than in bone marrow.

**Material and Methods**

Current methods for plasma cells enrichment, including FACS or MACS, face challenges due to their time-consuming workflows and considerable cell loss. Both techniques require large starting sample volumes to recover a sufficient number of cells for downstream analysis. Here we present a new, automated workflow to isolate CD138+ plasma cells directly from bone marrow or peripheral blood with high cell recovery, reducing the required starting sample volume. The MARS® column-free in-flow magnetic cell separation allows enrichment of plasma cells from a very low starting %. In a pilot study, CD138-expressing MM.1S cell line cells were spiked into peripheral blood from 0.0001% to 5.0% of the white blood cell frequency range. With the optimized running conditions (including flow rates) we achieved over 70% purity and 80% recovery of the spiked cells.

**Results and Discussions**

This method has been validated on bone marrow and peripheral blood samples from MM patients. The MARS® workflow is compatible with FISH (Fluorescence In Situ Hybridization), next-generation and single-cell sequencing, and flow cytometry analysis. Additionally, the simplicity and automation of this method make it a potential candidate for standardizing clinical procedures in MM disease monitoring.

**Conclusion**

Efficient enrichment of plasma cells for disease monitoring at MGUS and MRD stages is essential for effective management of multiple myeloma. The presented automated workflow offers high cell recovery and reduced starting sample volumes, making it a promising approach for standardized clinical procedures and improving patient outcomes.

### EACR2024-1189
### Increasing incidence of colorectal cancer in young adults aged <50 years in England over the last 20 years

H. Al-Maliki[1], Y. Salari[1], S. Edensor[1], A. Soultati[2], A. Memon[1]
[1]Brighton and Sussex Medical School, Primary Care and Public Health, Brighton, United Kingdom
[2]University Hospitals Sussex NHS Foundation Trust, Department of Oncology, Brighton, United Kingdom

**Introduction**

Worldwide, colorectal cancer (CRC) is the 3$^{rd}$ most common cancer and the 2$^{nd}$ leading cause of cancer mortality – with an estimated 1.9 million new cases (10% of all cancers) and 935,000 deaths (9.4% of all cancers) in the year 2020. In recent decades, the incidence of early-onset CRC, (i.e., in persons aged <50 years) has been rising in many countries, whereas in older adults the incidence has stabilized. We conducted a retrospective population-based cohort study to examine changes in the incidence of CRC in England over the last four decades.

**Material and Methods**

Individual-level data for patients diagnosed with CRC during 1985-2019 were obtained from Public Health England. Average annual incidence rates (AAIRs) were calculated by sex and two age categories (<50 and 50+ years) during the seven 5-year time periods (1985-89 to 2015-19).

**Results and Discussions**

During the 35-year study period, a total of 1,048,935 patients (54% males, 46% females) with CRC were registered in England. In young adults aged <50 years, the average annual number of cases increased from 1224/year in 1985-89 to 2209/year in 2015-19 (+80%); the AAIRs increased from 3.8/100,000 in 1985-89 to 6.3/100,000 in 2015-19 (+66%). This increase accelerated from 2000-04 onwards in both sexes. Whereas in older adults aged 50+ years, the AAIRs remained fairly stable during the study period with a nominal increase of 4%. During the recent 2010-17 period, 62% of the patients aged <50 years were diagnosed at an advanced Stage 3 or 4, compared to 55% of those aged 50+ years. There were relatively higher proportion of older adults aged 50+ years diagnosed at an early Stage 1 or 2, compared with those aged <50 years – suggesting that the benefit of early diagnosis due to screening/treatment of pre-malignant lesions is mainly in older adults aged 50+ years currently included in the screening criteria.

**Conclusion**

This large population-based study of over 1 million patients shows that the incidence of CRC in older adults aged 50+ years in England has stabilized (or levelled off) during the last 20 years, whereas there has been a steady increase in incidence in young adults aged <50 years during this period. It is suggested that the rising incidence of early-onset CRC in young adults aged <50 years may be attributed to diet, lifestyle factors, microbiome composition and genetic factors. This study supports the debate for lowering the bowel cancer screening age to facilitate early diagnosis and treatment of pre-malignant lesions in young adults aged <50 years.

### EACR2024-1215
### Systematic Review of Lifestyle Factors and Hereditary Risk Factors for Pancreatic

© 2024The Authors. Molecular Oncology is published by John Wiley & Sons Ltd on behalf of Federation of European Biochemical Societies

### Cancer: Implications for Prevention and Early Detection Strategies

L.R. Rajmohan[1], P. p[2], P. d[1]
[1]ACS Medical College & Hospital, General Medicine, Chennai, India
[2]ACS Medical College & Hospital, Biochemistry, Chennai, India

**Introduction**

Pancreatic cancer remains one of the most lethal malignancies, necessitating a deeper understanding of its etiology for effective prevention and early detection. This systematic review investigates the association between lifestyle factors, hereditary risk factors, and pancreatic cancer incidence, aiming to elucidate their roles in disease development.

**Material and Methods**

A comprehensive search of PubMed, Embase, and Scopus databases was conducted to identify studies exploring the relationship between lifestyle factors, hereditary risk factors, and pancreatic cancer risk. Articles reporting on epidemiological studies, genetic analyses, and risk assessment models were included. Data synthesis and analysis were performed to evaluate the contribution of identified risk factors to disease incidence.

**Results and Discussions**

Multiple lifestyle factors, including smoking, obesity, and high-fat diet, are consistently associated with an increased risk of pancreatic cancer. Additionally, hereditary factors such as family history of pancreatic cancer and germline mutations in BRCA1/2 and CDKN2A genes contribute significantly to disease susceptibility. Understanding these risk factors can inform the development of targeted prevention strategies and screening programs for high-risk individuals.

**Conclusion**

The findings highlight the importance of lifestyle modifications and genetic counseling in mitigating pancreatic cancer risk. Implementation of tailored prevention interventions and early detection strategies based on identified risk factors has the potential to reduce the burden of this deadly disease.

### EACR2024-1221

### Determining The Right Formula For Neoadjuvant Chemotherapy To Achieve Complete Pathologic Response In A Black Population: An Audit Of Clinical Practice

A. Alabi[1], A. Lawal[2], B. Adegboyega[1], A. Joseph[1], A. Sowunmi[1]
[1]NSIA-LUTH Cancer Centre, Department of Radiotherapy & Oncology, Lagos, Nigeria
[2]College of Medicine- University of Lagos, Department of Surgery, Lagos, Nigeria

**Introduction**

Breast cancer (BC) is a complex, heterogeneous disease classified into hormone-receptor-positive, human epidermal growth factor receptor-2 overexpressing (HER2+) and triple-negative breast cancer (TNBC) subtypes. These tumour characteristics play a major role in determining treatment options and clinical outcomes. Neoadjuvant chemotherapy (NACT) offers a unique opportunity to reduce tumour size in patients with locally advanced breast cancer, this has proven to be effective in the management of locally advanced BC. Late-stage presentation remains a dilemma for cancer patients in LMIC contributing to a significant proportion requiring NACT. Currently, limited knowledge exists on the most effective NACT for the black population due to a lack of clinical trials and paucity of data in the management of BC. This study aims to review predictors of response to NACT amongst black African women.

**Material and Methods**

This is a retrospective study of histologically confirmed BC patients who received NACT between 2011 and 2015 at the Lagos University Teaching Hospital, Lagos State. Sociodemographic and Clinical information was collected and analysed into 2 subgroups: Group 1 Age $\leq$ 40 years and Group 2 age > 40 years. Medical case notes of these patients were retrieved to assess socio-demographic data, clinical history, treatment response including clinical, radiologic, and pathologic parameters, pathologic complete response rates (pCR), and survival measurements. Data was analysed using SPSS version 26. Comparison was by chi-square test of independence at p value 0.05. Ethical approval was obtained from institutional HREC.

**Results and Discussions**

351 case records were reviewed. Mean age was 49.9 ± 12.1 (range 28–85). NACT was either anthracycline based regimen, taxanes only or taxanes based regimen. Median chemotherapy dose was 4 (range 2–8) cycles. The widest diameter of tumours was 30mm to 300mm. Ninety-eight (28%) had cPR following a minimum of 4 cycles of chemotherapy, this was also seen with Luminal A subtypes. TNBC had more partial responses 86(25%). Younger patients had better tumour response in stages I and II while older patients 134(38%) had better responses in Stage III.

**Conclusion**

The goal of NACT in achieving pCR was achieved in a subset of this patient resulting in better survival outcomes. It is important to carry out a larger prospective study to emphasize the importance of using NACT to improve treatment-related outcomes for all subtypes.

### Radiobiology/Radiation Oncology

### EACR2024-0127

### TIGIT blockade combined with local radiotherapy and PD-1 blockade in a syngenic murine breast cancer model

I.A. Kim[1], S. Kim[2], S.H. Jeon[3]
[1]Seoul National University Bundang Hospital- Seoul National University College of Medicine, Radiation Oncology, Seoul, South Korea
[2]Seoul National University Graduate School of Medicine, Tumor Biology, Seoul, South Korea
[3]Seoul National University Bundang Hospital, Radiation Oncology, Seongnam, South Korea

**Introduction**

Molecular Oncology 18 (Suppl. 1) (2024) 1–495
© 2024 The Authors. Molecular Oncology is published by John Wiley & Sons Ltd on behalf of Federation of European Biochemical Societies

The efficacy of Immune checkpoint inhibitor (ICI) alone showed limited efficacy in many tumors such as breast cancer. Radiation therapy (RT) is a promising combination partner of ICI as a strong immune stimulator; however, it also can increase immune suppressive repertoires. TIGIT (T cell Immunoglobulin and ITIM domain) is an inhibitory receptor expressed on activated lymphocytes. We evaluated the effects of TIGIT blockade in addition to local RT and PD-1 blockade as a strategy to overcome therapeutic resistance of ICI in a syngenic murine breast cancer model.

**Material and Methods**

4T1-Luc tumors were treated with various strategies including RT, anti-PD-1, anti-TIGIT. 24 Gy in 3 fractions was delivered to the tumor at hindlimb while the tumor at flank was left unirradiated. Anti-PD-1 blocking antibody (10 mg/kg) and anti-TIGIT blocking antibody (10 mg/kg) were intraperitoneally injected for 6 times for 2 weeks. Flow cytometry, IHC staining, and ELISA were performed to assess the immunologic response after each treatment modality.

**Results and Discussions**

The triple combination therapy (TCT) group showed the most superior growth delay of primary and secondary tumor among treatments. The number of metastatic lung nodule was significantly reduced by TCT as well. Plasma levels of interferon-βand interferon-γ were the highest after TCT. Proportion of $CD8^+$ dendritic cells among immune cells in spleen as well as tumor-draining lymph node was significantly increased by TCT. Moreover, TCT significantly increased effector-memory ($CD44^+$ $CD62L^-$) cells among splenic $Foxp3^-$ non-regulatory $CD4^+$ and $CD8^+$ T cells, while decreasing proportion of regulatory T cells. TCT also increased the infiltration of $CD8^+$ T cells in primary and secondary tumors. Furthermore, increased expression of CD226, an activating counter-receptor of TIGIT, on $CD8^+$ T cells following TIGIT blockade was observed in both spleen and primary tumor, possibly suggesting the activation of anti-tumor immune response by addition of TIGIT blockade to local RT and PD-1 blockade. Intratumoral myeloid-derived suppressor cells were significantly decreased by TCT.

**Conclusion**

TIGIT blockade elicits local and systemic antitumor immune responses in a murine triple-negative breast cancer model. The tumor growth delay and inhibition of lung metastasis were most prominent in TCT. These results suggest that TIGIT blockade could be a viable approach to increase the efficacy of RT and ICIs in breast cancer which is a relatively immunologically cold tumor.

*Grant#2023R1A2C3003782*

### EACR2024-0178

### HLA expression after carbon-ions irradiation in human cervical cells

*S. Hasegawa[1], N. Izumi Nakajima[1]*
*[1]National Institutes for Quantum Science and Technology, Charged Particle Therapy Research, Chiba, Japan*

**Introduction**

Human leucocyte antigen (HLA) is a major histo-compatibility complex (MHC) class I molecule in human, being critical for anti-tumor immunity in the host. Lack of HLA expression is often observed in many cancers including human cervical cancer. This study aimed to determine the impact of carbon-ions (C-ions) irradiation on HLA expression in human cervical cancer cells.

**Material and Methods**

Human cervical cancer cell lines (SiHa, CaSki, HeLa, ME-180), NB1RGB human normal fibroblast cells, and HEK293 cells stably overexpressing human papilloma virus (HPV)-E6 or E7 gene were used. Exposure to C-ions irradiation (290 MeV/n; LET, ~70 keV/μm, mono peak) was performed at the Heavy-Ion Medical Accelerator facility of the National Institutes for Quantum Science and Technology. HLA-ABC expression in cell membrane was evaluated using a spectral cell analyzer.

**Results and Discussions**

Relative HLA expression was downregulated in HPV-positive human cervical cancer cells compared to HPV-negative normal fibroblast NB1RGB cells. Relative HLA expression was downregulated in HEK293 cells stably overexpressing HPV-E6 or E7 genes compared to HEK293 cells. C-ions irradiation (up to 10 Gy) upregulated relative expression of membrane HLA in human cervical cancer cells and HEK293 cells overexpressing HPV-E6 or E7 protein in a dose-dependent manner.

**Conclusion**

C-ions irradiation upregulates HLA expression in human cervical cancer cells.

### EACR2024-0198

### A novel role of exostosin glycosyltransferase 2 (EXT2) in glioblastoma cell metabolism, radiosensitivity and ferroptosis

*R. Matesanz-Sanchez[1], M. Peitzsch[2], M. Seifert[3], N. Cordes[1,4,5,6], A. Vehlow[1]*
*[1]OncoRay - National Institute for Radiation Research in Oncology, Medizinische Fakultät Carl Gustav Carus-Technische Universtät Dresden, Dresden, Germany*
*[2]Institute of Clinical Chemistry and Laboratory Medicine, University Hospital Carl Gustav Carus-Technische Universität Dresden, Dresden, Germany*
*[3]Institute of Medical Informatics and Biometry IMB, Medizinische Fakultät Carl Gustav Carus-Technische Universität Dresden, Dresden, Germany*
*[4]German Cancer Consortium DKTK-Partner Site Dresden, German Cancer Research Center DKFZ, Heidelberg, Germany*
*[5]Institute of Radiooncology – OncoRay, Helmholtz-Zentrum Dresden - Rossendorf, Dresden, Germany*
*[6]Department of Radiotherapy and Radiation Oncology, University Hospital Carl Gustav Carus-Technische Universität Dresden, Dresden, Germany*

**Introduction**

Inherent resistance of glioblastoma (GBM) cells to therapy contributes significantly to the dismal prognosis of glioblastoma patients. Besides genetic and epigenetic alterations, metabolic reprogramming appears fundamental in this aspect. Recent studies suggest a connection between therapy resistance and ferroptosis, an

© 2024 The Authors. Molecular Oncology is published by John Wiley & Sons Ltd on behalf of Federation of European Biochemical Societies emerging cell death mechanism associated with lipid peroxidation. Nevertheless, the causal circuits are only partially understood. Here, we identify exostosin glycosyltransferase 2 (EXT2) as novel regulator of GBM therapy resistance and characterize the underlying mechanisms.

**Material and Methods**

Novel regulators of GBM therapy resistance were identified through combined transcriptome analysis of a panel of 9 human GBM cell models and TCGA GBM patient datasets. Cell viability, residual DNA double strand breaks, autophagy and apoptosis were quantified in by conducting a RNA interference-mediated screen of the top 12 differentially expressed genes associated with poor survival of GBM patients. GBM cell basal clonogenic and clonogenic radiation survival were quantified upon EXT2 siRNA. To link EXT2 to cellular metabolism, untargeted and targeted metabolome analyses followed by metabolite classification and pathway mapping were performed upon EXT2 knockdown. EXT2 dependent ferroptosis was assessed by quantification of lipid peroxidation without and in combination with irradiation.

**Results and Discussions**

Among the top 12 differentially expressed genes, we discovered exostosin glycosyltransferase 2 (EXT2) most potently to reduce cell viability and induce cell death. In addition, untargeted and targeted metabolome analyses detected that EXT2-depleted GBM cells exhibit a differential abundance of S-adenosylmethionine (SAM) as well as SAM-associated metabolites particularly in the transsulfuration pathway. Considering these metabolic changes in mitigating ferroptosis, we found lipid peroxidation increased across the panel of non-irradiated and irradiated EXT2-depleted GBM cell models. Moreover, pretreatment with the ferroptosis inducer Ferrostatin-1 reversed lipid peroxidation and counteracted EXT2 depletion-related radiosensitization.

**Conclusion**

Collectively, our results uncover a novel role of EXT2 in survival of GBM cells, their radiation response and specific metabolic pathways linked to ferroptotic cell death.

### EACR2024-0325

### Radiation-induced lymphopenia influences anti-cancer treatment outcome in a preclinical model of lung cancer

*E. Prades Sagarra[1], N.G. Lieuwes[1], R. Biemans[1], L. Schuitmaker[1], S. van Hoof[2], P. Lambin[1], F. Verhaegen[2,3], A. Yaromina[1], L.J. Dubois[1]*

*[1]Maastricht University, The M-Lab - Department of Precision Medicine - GROW Research Institute for Oncology & Reproduction, Maastricht, The Netherlands*

*[2]SmART Scientific Solutions BV, SmART Scientific Solutions BV, Maastricht, The Netherlands*

*[3]MAASTRO Clinic, Radiotherapy Division - GROW Research Institute for Oncology & Reproduction – Maastricht University Medical Centre+, Maastricht, The Netherlands*

**Introduction**

Development of radiotherapy-induced lymphopenia (RILP), i.e. >20% loss in absolute lymphocyte counts, is observed in approximately half of the treated cancer patients and has been associated with worse treatment response. Retrospective studies reported associations between the lymphopenia grade and the radiation dose to major blood-carrying structures or thoracic vertebra. However, investigations of the causal relationship between RILP and worse treatment outcome are currently lacking. In this project, we developed murine RILP models and tested whether lymphopenia influenced tumor growth in vivo after radiotherapy and immunotherapy.

**Material and Methods**

Non-tumor bearing female C57Bl/6J mice received 10Gy irradiation at the heart, large blood vessels (LBV) or thoracic vertebra, using a CT image-guided irradiator, to induce lymphopenia. White blood cell (WBC) subtype counts were measured before and weekly after irradiation. Another group of animals were inoculated subcutaneously with Lewis Lung Carcinoma cells before lymphopenia induction. When tumors reached 208 ± 52mm$^3$, tumors were locally irradiated (10Gy) and animals were injected with the immunocytokine L19-IL2 (1 mg/kg, 3 times QOD) or vehicle (PBS) intravenously. Tumor growth was followed via caliper measurements until reaching >4 times starting volume.

**Results and Discussions**

Irradiation treatment plans were optimized in silico to ensure maximal target coverage, while minimizing the dose to normal tissues. In non-tumor bearing animals, LBV and vertebra irradiation led to grade 2 RILP. Heart irradiation failed to induce grade 2 RILP and therefore was not taken along in the subsequent study with tumor-bearing animals. Combination of radiotherapy and L19-IL2 induced a tumor growth delay compared to radiotherapy alone. Grade 2 RILP was confirmed in tumor-bearing animals treated with radiotherapy only, but did not affect treatment outcome. Surprisingly, vertebra and LBV irradiation increased the median survival in animals treated with radiotherapy and L19-IL2 (13.8 and 12.6 days for LBV and vertebra irradiation vs 10.5 days for sham irradiation). Especially in the vertebra group, L19-IL2 combined with radiotherapy restored and increased lymphocyte and eosinophil counts.

**Conclusion**

We established an irradiation method to induce grade 2 RILP in mice by precise irradiation of LBV and vertebra. RILP did not affect treatment outcome in animals treated with radiotherapy only. L19-IL2 could be a promising strategy to restore lymphocyte counts and revert RILP.

### EACR2024-0359

### Physical and biological tumor characteristics evaluation in an orthotopic glioblastoma model

*M.S. Kumaravadivel[1,2], E. Ulbricht[3], T. Rassamegevanon[1,2], M.H.H. Schmidt[4], M. Schmitz[5], M. Krause[1,2,6,7,8], A. Taubenberger[3], A. Dietrich[1,2], R. Bütof[2,6,7,8]*

*[1]German Cancer Consortium DKTK- Partner Site Dresden, and German Cancer Research Center DKFZ, Heidelberg, Germany*

*[2]OncoRay – National Center for Radiation Research in Oncology, Faculty of Medicine and University Hospital*

© 2024 The Authors. Molecular Oncology is published by John Wiley & Sons Ltd on behalf of Federation of European Biochemical Societies

Carl Gustav Carus- TUD Dresden University of Technology- Helmholtz-Zentrum Dresden – Rossendorf, Dresden, Germany
[3]Biotechnology Center, Technische Universität Dresden, Dresden, Germany
[4]Institute of Anatomy, Medical Faculty Carl Gustav Carus-Technische Universität Dresden School of Medicine, Dresden, Germany
[5]Institute of Immunology, Faculty of Medicine Carl Gustav Carus-Technische Universität Dresden, Dresden, Germany
[6]Department of Radiotherapy and Radiation Oncology, Faculty of Medicine and University Hospital Carl Gustav Carus-TUD Dresden University of Technology, Dresden, Germany
[7]Helmholtz-Zentrum, Dresden - Rossendorf, Dresden, Germany
[8]National Center for Tumor Diseases Dresden NCT/ UCC, Germany; German Cancer Research Center DKFZ, Heidelberg, Germany; Faculty of Medicine and University Hospital Carl Gustav Carus-TUD Dresden University of Technology, Dresden, Germany; Helmholtz -Zentrum Dresden-Rossendorf HZDR, Heidelberg, Germany

**Introduction**

Glioblastoma multiforme (GBM) is the most common form of cancer in the adult brain with a poor median overall survival of 15 months. Treatment includes surgery and postoperative radio-chemotherapy. Target volume margins for irradiation accounting for microscopic tumor extension are uncertain and therefore population-based margins are currently used in the clinical routine. These could either be too small leading to increased risk of loco-regional control or too large and thus, enhancing the probability of normal tissue toxicity. Of note, the effects of irradiation on these margins and surrounding regions of the tumor are not well characterized so far.

**Material and Methods**

To overcome this knowledge gap, we investigated an orthotopic GBM model (U87MG-mCherry) for the cellular and mechanical properties of its tissue at the cellular scale. Orthotopic tumor growth was monitored through magnetic resonance imaging (MRI). After reaching a diameter of 2 – 3 mm on the MRI, tumors were irradiated with 3 fractions of 3 Gy by an in-house developed small animal image guided radiotherapy (SAIGRT) system. To quantitatively assess mechanical properties of brain tissue and tumor, 24 hours post the last irradiation fraction, tissue sections were probed using a combined atomic force microscopy (AFM)–light microscopy setup within the tumor, the surrounding tissue and contralateral regions.

**Results and Discussions**

Non-irradiated control samples showed similar mechanical properties within the tumor region and its surrounding tissue. In contrast, the tissue region adjacent to the tumor underwent significant stiffening upon irradiation. Irradiation had no clear effect on the tumor itself nor on the contralateral side. To further characterize the mechanical and biochemical changes, multiplexed histology with a panel of six markers specific to early irradiation changes is ongoing.

**Conclusion**

These findings will eventually contribute to a better understanding of mechanical responses to irradiation and to a more individualized target volume definition for an optimized radio-oncological treatment.

### EACR2024-0405
### Exploring FOXM1 as a candidate radiosensitiser in meningioma treatment

J. Na[1], W.K.V. Chan[2], A. Hart[3], C.O. Hanemann[1]
[1]University of Plymouth, Peninsula Medical School, Plymouth, UK
[2]Exeter University, Medical Science, Exeter, UK
[3]Bath University, Biomedical Science, Bath, UK

**Introduction**

The urgent need for developing and evaluating potential radiosensitizers arises due to the absence of efficient chemotherapy options for meningioma. Through comprehensive molecular profiling, Forkhead Box M1 (FOXM1) has been identified as a pivotal transcription factor driving proliferation in meningioma. Diminished FOXM1 expression is recognised for its role in increasing sensitivity to radiation-induced cell death across diverse tumour types. Nevertheless, limited information exists regarding the behaviour of FOXM1 expression in meningioma. Thus, this study aims to specifically investigate FOXM1 as a potential radiosensitizer in meningioma.

**Material and Methods**

By inhibiting FOXM1 using Siomycin A or shRNA in conjunction with radiation treatment, we investigated their impact on survival, apoptosis, and cell cycle progression in two immortalized meningioma cell lines (IOMM-Lee and KT21-MG1).

**Results and Discussions**

Our results demonstrate that inhibition of FOXM1 synergistically reduces cell survival in combination with radiation therapy. FOXM1 knockdown (KD) amplifies radiation-induced apoptosis and augments DNA double-strand breakage. While cellular proliferation displayed a decreasing trend with FOXM1 inhibition, statistical significance was not achieved. It was observed that FOXM1 KD increased the level of phosphorylation (Thr600) in response to radiation, aligning with the data on cell cycle arrest that indicates an augmentation in G2/M arrest.

**Conclusion**

We propose FOXM1 as a potential radiosensitiser in meningioma, aimed at reducing the requisite radiation dosage for effective therapy while mitigating associated adverse effects.

### EACR2024-0406
### HDAC inhibitor, LAQ824, enhanced radiosensitisation in meningioma via potent DNA damage

S. Shaji[1], J. Na[1], C.O. Hanemann[1]
[1]University of Plymouth, Peninsula Medical School, Plymouth, United Kingdom

**Introduction**

Meningiomas are the most common intracranial neoplasm. Despite the recent progress in molecular profiling and classification of meningioma, the mainstays

© 2024The Authors. Molecular Oncology is published by John Wiley & Sons Ltd on behalf of Federation of European Biochemical Societies of treatment are confined to safe resection and radiation therapy. Currently, no standard chemotherapy is administrated for meningioma. Factors such as radiation resistance, incomplete resection and the invasive behaviour of the tumour are thought to contribute to meningioma recurrence. These challenges underscore the need for further research and the development of non-invasive, targeted therapies to improve meningioma management. A recent study identified the potential of epigenetic compounds like Panobinostat, LAQ824 and HC toxin in reducing the cell viability in patient-cultured meningioma (Tatman, Wroblewski et al. 2021). The early phase clinical studies of AR-42, a histone deacetylase inhibitor (HDACi) for meningiomas have shown promising results (Welling, Collier et al. 2021). In the initial screening of our current study LAQ824, a pan HDACi outperformed other HDACis and currently no clinical trials are ongoing with LAQ824.So, we attempted to increase the radiation efficacy by the addition of LAQ824 in high-grade meningiomas.

**Material and Methods**

Immortalised cell lines IOMM-Lee, KT21-MG1, and patient-derived grade 2 meningioma cells (BTB0380 and BTB0017) were used. Cell survival, cell viability, HDAC activity, DNA-double strand breaks (DNA-DSBs), apoptosis and senescence were measured. 3D spheroids were developed, and their growth and viability were measured.

**Results and Discussions**

We found the combination treatment resulted in a significant (p-value <0.0001) synergistic reduction of survival fraction by 10-fold in IOMM-lee and 6-fold in KT21-MG1. Also, the radiation-induced HDAC activity is altered by the action of LAQ824. LAQ824 increased the radiation-induced DNA-DSBs (8.7±0.6 and 15.1±2.1 γH2AX foci/nucleus) compared to the irradiation alone (3.8±0.4 and 6.7±0.3 γH2AX foci/nucleus) in IOMM-Lee and KT21-MG1 followed by a significant (p-value< 0.0001) apoptotic cell death (47%) and senescence (42.5%) in KT21-MG1, but not in IOMM-Lee. In addition, the dead cells in 3D spheroids derived from IOMM-Lee and KT21-MG1 were significant after the combination treatment reaching 16.5% and 0.8% on average, respectively. So far, BTB0380 has shown a synergistic effect and BTB0017 was sensitive to both treatments and showed no synergism.

**Conclusion**

Our findings showed promising preliminary results for LAQ824 as a radiosensitiser in meningioma management.

### EACR2024-0416
### Radioresistant Murine Pheochromocytoma Cell Lines

S. Lemm[1,2], S. Richter[3], M. Ullrich[1], J. Pietzsch[1,2]
*[1]Helmholtz-Zentrum Dresden-Rossendorf, Institute of Radiopharmaceutical Cancer Research, Dresden, Germany*
*[2]Technische Universität Dresden, Faculty of Chemistry and Food Chemistry, Dresden, Germany*
*[3]University Hospital Carl Gustav Carus, Institute for Clinical Chemistry and Laboratory Medicine, Dresden, Germany*

**Introduction**

Treatments for patients with metastatic pheochromocytoma and paraganglioma (PCC/PGL) are often limited. Peptide receptor radionuclide therapy (PRRT) with $\beta^-$ particle emitters is usually an option for slow-growing lesions. During the low-dose phase after decay of the lethal dose individual tumors cells can become radioresistant. In-depth characterization of the underlying mechanisms involved in this radioresistant phenotype still remains to be elucidated. To address this issue, we generated radioresistant (rr) murine pheochromocytoma cell lines (MPC) and characterized them in comparison to non-irradiated (noIR) control cells.

**Material and Methods**

For the generation of rr cells two different parental cell lines were chosen: genetically modified MPC cells expressing a codon-optimized *EPAS1* gene (HIF-2α) to assess the effects of pseudohypoxia on radioresistance and MPC control cells harboring the empty expression vector (EV). Both cell lines underwent two different irradiation regimes: (I) daily irradiation with increasing X-ray doses from 0.5 to 2.0 Gy and (II) intermitted X-ray treatment with 2 Gy. Growth of cells and corresponding subcutaneous tumor allografts was examined. Radiosensitivity was analyzed in an irradiation dose response assay with spheroids subjected to single doses of 0−40 Gy.

**Results and Discussions**

Following the irradiation protocol (I) cells accumulated 201 Gy, whereas protocol (II) led to a total dose accumulation of 60 Gy. Morphological changes were observed when cells were irradiated at a daily basis, resulting in a more clustered and spheroidal growth. Upon daily irradiation with 2 Gy monolayer H2A cells stopped proliferating more often compared to EV cells. Growth of rr cells and the corresponding tumor allografts decreased compared to noIR cells, with a more pronounced effect in EV compared to HIF 2α-positive lineages. Assessing the response to a series of increasing X-ray doses, tumor spheroids grown from HIF-2α-positive rr cells showed increased survival compared to irradiated EV and noIR cells.

**Conclusion**

The irradiation protocols using low doses of X-rays resulted in a novel model for radioresistant PCC/PGL that arise during later stages of PRRT treatment. Especially HIF-2α mediated the radioresistant phenotype, but EV cells were more robust against daily irradiation in monolayer. MPCrr will be used to further investigate genetic and metabolic characteristics of radioresistant phenotypes in PCC/PGL in vitro and in vivo.

### EACR2024-0494
### Irradiation of extracellular matrix proteins affects their molecular structure and mediates epithelial cell behavior

Y. Meshram[1], S. Cartmell[2], C. Kirwan[3], R.J. Tuieng[4], M.J. Sherratt[4]
*[1]Division of Cell Matrix Biology & Regenerative Medicine, The University of Manchester, Manchester, United Kingdom*

© 2024 The Authors. Molecular Oncology is published by John Wiley & Sons Ltd on behalf of Federation of European Biochemical Societies

[2]The Henry Royce Institute- Royce Hub Building-, Department of Materials- School of Natural Sciences- Faculty of Science and Engineering- The University of Manchester, Manchester, United Kingdom
[3]Manchester Cancer Research Centre, Division of Cancer Sciences- School of Medical Sciences - Faculty of Biology- Medicine and Health- The University of Manchester, Manchester, United Kingdom
[4]Division of Cell Matrix Biology and Regenerative Medicine, The University of Manchester, Manchester, United Kingdom

**Introduction**
Radiotherapy is an important treatment for breast cancer treatment, but exposure of healthy tissues to therapeutic X-ray doses is associated with side effects including secondary cancers and fibrosis. Whilst the impact of X-ray exposure on cells is well characterized, the interactions between ionizing radiation and the surrounding extracellular matrix are poorly defined. This study aimed to use a model extracellular matrix (ECM) system Matrigel® to characterize the impact of therapeutic X-ray doses on ECM structure and downstream cell behavior.

**Material and Methods**
Matrigel®, a reconstituted basement membrane matrix derived from Engelbreth-Holm-Swarm (EHS) mouse sarcoma was exposed to X-ray radiation (doses of 50Gy or 100Gy). The impact of X-ray exposure on Matrigel® composition, protein abundance was assessed by mass spectrometry (LC-MS/MS). Irradiation induced changes in protein structure were identified by peptide location fingerprinting. The influence of irradiated Matrigel® on the behavior of immortalized mouse mammary epithelial cells (EpH4) was assessed by live cell imaging (cell adherence, viability, and proliferation) and by transcriptomic analysis of extracted mRNA.

**Results and Discussions**
Exposure to therapeutic X-ray doses (50 and 100 Gy) had no effect Matrigel® protein identity or relative abundance but did induce significant changes in localized tryptic peptide yield for the basement membrane proteins laminin, collagen IV, nidogen, and heparan sulfate proteoglycans. Epithelial cell adhesion was significantly reduced on irradiated Matrigel® (at both X-ray doses). Hierarchical clustering of the top 50 differentially expressed genes identified both dose dependent upregulation (17 genes) and downregulation (33 genes) following exposure of cells to 100Gy irradiated Matrigel®. Significantly affected GO processes included cell growth and shape and proliferation.

**Conclusion**
This study demonstrates that exposure of ECM proteins to therapeutic X-ray doses can significantly alter protein structure and in the behavior of adhered cells. We conclude that therapeutic X-ray regimes have the potential to influence normal tissue function as a consequence of cell/matrix interactions.

### EACR2024-0650
### Tumor immune microenvironment changes in response to different radiotherapy regimens in a bilateral tumor model

M. Alimohammadi[1], H. Ghaffari-Nazari[2], R. Alimohammadi[3], M. Bakhshandeh[4], S.A. Jalali[3], N. Rezaei[1]
[1]Tehran University of Medical Sciences, Immunology, Tehran, Iran
[2]semnan university of medical sciences, Immunology, semnan, Iran
[3]Shahid Beheshti University of Medical Sciences, Immunology, Tehran, Iran
[4]Shahid Beheshti University of Medical Sciences, radiology technology, tehran, Iran

**Introduction**
The effect of Radiotherapy on immune response may vary with dose and fractionation schedule. Combining radiotherapy with immunotherapy could be a promising strategy for synergistic enhancement of treatment efficacy, however, the selection of the best matched combination of immunotherapy with each radiotherapy scheme remain to be addressed. In the present study, we evaluated the impact of different radiotherapy schemes on immune microenvironment of treated and distal tumors while assessing the systemic anti-tumor immune response aiming to help for designing more rational RT combinations.

**Material and Methods**
In a bilateral CT26 tumor model, one tumor site was irradiated with three distinct radiotherapy regimens, including hypofractionated (1 x 16Gy , 2 x 10Gy), and conventional (10 x 3Gy) under the same biologically effective dose. The Immunomonitoring experiments were performed at different time points by flowcytometry. Tumor volume and survival rate were evaluated during 90 days.

**Results and Discussions**
Compared to the control group, only ablative radiation increased $CD8^+$ T cells population in both the treated and distal tumors. While conventional RT enhanced the recruitment of MDSCs into the irradiated tumors, the Ablative and hypo schemes induced Treg enrichment. The two hypofractionated regimens enhanced the expression of CD47 as well as the PD-1/PD-L1 axis, and a long-term survival was observed following them. Combination of Ablative therapy with anti-PD-L1 increased $CD8^+$ cells number in treated and distal tumors, which is consistent with better tumor control and increased survival following this scheme.

**Conclusion**
It seems that hypofractionated radiotherapy regimens, are the most effective methods in inducing the systemic (abscopal) effect and immune checkpoint blockers can promote them. The results suggest targeting the recruitment of MDSC and Treg are, respectively, crucial for therapeutic efficacy of conventional and hypofractionated RT regimens, and dual checkpoint blockade of CD47 and PD-L1 seems necessary to maximize the anti-tumor immune responses following the hypofractionated schemes.

### EACR2024-0746
### Evaluating the immune response activation by targeted radionuclide

© 2024 The Authors. Molecular Oncology is published by John Wiley & Sons Ltd on behalf of Federation of European Biochemical Societies

### therapy: towards a correlation between dose and response

J. Perrin[1], G. Tamborino[1], N. Overdevest[1], J. Nonnekens[1]
[1]Erasmus MC, Molecular genetics and Radiology & Nuclear medicine, Rotterdam, The Netherlands

**Introduction**

Targeted radionuclide therapy (TRT) consists of the injection of a radiolabeled vector targeting cancer cells. This allows the irradiation of the primary tumor and metastases while mostly sparing the healthy tissues. This led to clinical successes in neuroendocrine and prostate cancer for a proportion of the patients, but current treatments do not lead to cure. Furthermore, there is still little known about the immunogenic potential of TRT and thus whether it could synergize with immunotherapies which has shown potential for external beam radiotherapy.

**Material and Methods**

The aim of our study is to assess the immunogenicity of TRT using available literature data. Among the 1074 studies included in our search, 25 met inclusion criteria. To compare the large variety of TRT strategies used, the dose (rate) delivered at the tumor site in vivo or to cultured cells in vitro was calculated. Next, the correlation between the delivered dose (rate) and the % of change in various immune parameters between the control and TRT condition was evaluated.

**Results and Discussions**

A strong, significant correlation (R>0.7, p<0.05) was observed between early factors of the immune response, such as calreticulin tumor expression and TNF-a secretion, and the absorbed dose of TRT. Interestingly, a stronger correlation was observed when the dose rate was taken into account rather than the cumulated dose, suggesting the dose rate is better suited to predict TRT immunogenicity. Immune markers related to the tumor specific lysis also seemed to show a dose rate-dependent increase, however not enough data was available to establish a correlation. At the opposite, later immune parameters like CD8 or CD4 T cells tumor infiltrate did not show any correlation with the absorbed dose or dose rate.

**Conclusion**

This highlights that TRT can be immunogenic, and a correlation with the dose (rate) is observed for some elements of the immune response. However, the various way of measuring each immune markers and the different time points used in literature introduce a bias in our analysis. This might explain why, even though some parameters are increased after TRT, no correlation with the dose is observed. Nevertheless, increased knowledge on which dose (rate) is necessary for TRT to modulate the immune microenvironment is crucial to move forward with immunotherapy combination. Further experiments aiming to understand the underlying mechanisms of the observed correlations will allow to identify which dose is best suited to induce immunogenic effects.

### EACR2024-0807
### Identification of replication stress-associated DNA repair processes driven radioresistance in triple-negative breast cancer

M. Vogt[1], E.L. González[2], S. Classen[1], K. Rothkamm[1], C. Petersen[3], N. Cordes[2,4,5,6,7], K. Borgmann[1]
[1]Laboratory of Radiobiology and Experimental Radiooncology, University Medical Center Hamburg-Eppendorf UKE, Hamburg, Germany
[2]OncoRay - National Center for Radiation Research in Oncology, Faculty of Medicine Carl Gustav Carus-Technische Universität, Dresden, Germany
[3]Clinic for Radiotherapy and Radiation Oncology, University Medical Center Hamburg-Eppendorf UKE, Hamburg, Germany
[4]Department of Radiotherapy and Radiation Oncology, University Hospital Carl Gustav Carus, Dresden, Germany
[5]National Center for Tumor Diseases NCT- Partner Site Dresden, German Cancer Research Center DKFZ, Heidelberg, Germany
[6]German Cancer Consortium, Partner Site Dresden: German Cancer Research Center DKFZ, Heidelberg, Germany
[7]Institute of Radiooncology - OncoRay, Helmholtz-Zentrum Dresden-Rossendorf HZDR, Dresden, Germany

**Introduction**

Prognosis of breast cancer strongly depends on the molecular subtype, with triple-negative breast cancer (TNBC) having the worst prognosis. TNBC initially respond well to therapy, but often develop resistance to therapy due to an adaption to replication stress. The aim of this study was to identify replication stress-associated DNA repair processes to overcome therapy resistance in TNBC.

**Material and Methods**

Identification of relevant genes using a siRNA screen of 44 genes based on colony forming ability after 6 Gy (IR) in 3D cell culture in MDA-MB-231 cells. Verification of sensitivity in MDA-MB-468 cells and/or after Novobiocin treatment. Determination of DNA damage: general double strand repair (DSB) by 53BP1/ gH2AX, homologous recombination specific repair by Rad51, single-stranded DNA damage by RPA and S phase-specific repair by PCNA with corresponding immuno-fluorescence staining. Determination of replication processes with the DNA fiber assay.

**Results and Discussions**

Significant differences in radiosensitivity were observed after knockdown of all 44 genes associated with replication stress. Sensitization due to decreased survival was observed in 70% of genes, reaching up to 36%. The most radiosensitizing genes were *MSH2* (p<0.001), *POLK* (p<0.01), *CLK2* (p<0.001), *ISG15* (p<0.01) and *POLQ* (p<0.01). POLQ avoids replication stress by participating in micro-homology-mediated end joining of DSBs (MMEJ/TMEJ) and acting as an error-prone polymerase in translesion synthesis (TLS) and was the focus of further studies. The radiosensitizing effect after siRNA to POLQ could be confirmed by inhibition with novobiocin (SER=1.1-1.3/SER=1.1-1.4) and was validated in two cell lines. Confirming the radiation sensitization, a significant increase in DSBs was observed after POLQ inhibition, with a maximum in S phase of 30 %. Replication stress was manifested in the

© 2024 The Authors. Molecular Oncology is published by John Wiley & Sons Ltd on behalf of Federation of European Biochemical Societies increased formation of RPA and Rad51 foci and a slowed progression of DNA replication. Current studies are investigating the contribution of the POLQ-mediated processes TMEJ and TLS to radiosensitivity.

**Conclusion**

The use of inhibitors to overcome tolerance to replication stress resulting in radiosensitization of TNBC represents promising targets or new therapeutic options, especially in tumors that carry a BRCA1 mutation with resistance to PARP inhibitors.

*Acknowledgments: DFG grant no. 467414153.*

### EACR2024-0988
### Anti-L1CAM Terbium-161-based radioimmunotherapy eliminates ovarian cancer stem cells

*T. Todorov[1], R. Coelho[2], S. Dellea[3], F. Jacob[2], V. Heinzelmann-Schwarz[4], P. V. Grundler[5], N. P. van der Meulen[5], R. Schibli[1], M. Béhé[3], J. Grünberg[3]*

*[1]ETH Zurich and Paul Scherrer Institute, Center for Radiopharmaceutical Sciences and Department of Chemistry and Applied Biosciences, Zurich – Villigen-PSI, Switzerland*

*[2]University Hospital Basel and University of Basel, Department of Biomedicine, Basel, Switzerland*

*[3]Paul Scherrer Institute, Center for Radio-pharmaceutical Sciences, Villigen-PSI, Switzerland*

*[4]University Hospital Basel and University of Basel, Department of Gynecology and Gynecological Oncology - Hospital for Women and Department of Biomedicine, Basel, Switzerland*

*[5]Paul Scherrer Institute, Center for Radio-pharmaceutical Sciences and Laboratory of Radio-chemistry, Villigen-PSI, Switzerland*

**Introduction**

Cancer stem cells (CSCs) are a self-renewable and highly tumorigenic cell population which is a key player in therapy resistance, tumor relapse and metastasis formation in various malignancies. Our previous work verified in vitro and in vivo L1CAM+/CD133+ cells as radioresistant CSCs in ovarian cancer (OC). Anti-L1CAM radioimmunotherapy (RIT) with the monoclonal antibody chCE7 and Terbium-161 ($^{161}$Tb), emitting $\beta^-$ radiation and highly cytotoxic Auger electrons (AE), was validated against bulk L1CAM-expressing tumors in comparison to the clinically applied $\beta^-$ emitter Lutetium-177 ($^{177}$Lu). Here, to address the demand for CSC-targeted therapies, we describe a novel treatment approach by eliminating L1CAM+/CD133+ ovarian CSCs and tumor cells differentiated form them comparing $^{161}$Tb-based anti-L1CAM RIT to $^{177}$Lu.

**Material and Methods**

L1CAM+/CD133+ cells were evaluated in an OC patient cohort by immunofluorescence. A cell uptake assay and MTT proliferation assay in bulk OC cell lines were used to determine the membrane-bound and internalized uptake as well as the radiocytoxicity of chCE7-based radioimmunoconjugates (RICs). L1CAM+/CD133+ ovarian CSCs were sorted via fluorescence-activated cell sorting (FACS) from OC cell lines and inoculated into CD-1 immunodeficient mice. The mice were treated with $^{161}$Tb-chCE7 or $^{177}$Lu-chCE7 to study the potential of $^{161}$Tb to eradicate CSCs and their derivative cells compared to $^{177}$Lu.

**Results and Discussions**

L1CAM+/CD133+ cells of 0.3%-21% were confirmed in chemonaïve and relapse OC patient samples. The RICs had similar uptake of 50%-75% in the tested OC cell lines. The highly cytotoxic AE emitted by $^{161}$Tb resulted in significantly increased cytotoxicity in vitro and eradicated all tumor cells including the L1CAM+/CD133+ ovarian CSCs in vivo resulting in no tumor development. In contrast, the $\beta^-$ emission of $^{177}$Lu was not sufficient to eradicate the CSCs leading to tumor formation. Follow-up immunofluorescence analysis of the tumors confirmed the major CSC property of the inoculated L1CAM+/CD133+ ovarian CSCs to recapitulate the tumor heterogeneity by regenerating 14%-99% L1CAM+ and 1%-17% CD133+ cells together with populations negative for both CSC biomarkers

**Conclusion**

Anti-L1CAM $^{161}$Tb-based RIT represents a novel therapeutic modality to meet the urgent need of CSC-targeted therapies for clinical use. This research establishes a solid foundation for the translation of the reported RIT into clinical application.

### Signalling Pathways

### EACR2024-0010
### The complex interplay between GLS1 and c-Myc drives head and neck cancer metastasis

*Y. Teng[1], J. Yang[1], F. Yang[1], F. Chen[1]*

*[1]Emory University, Department of Hematology and Medical Oncology, Atlanta, United States*

**Introduction**

c-Myc is known to promote glutamine utilization by upregulating glutaminase 1 (GLS1), which converts glutamine to glutamate, which is catabolized in the TCA cycle. However, the regulation between these two molecules in head and neck cancer (HNC) remains largely unknown.

**Material and Methods**

Molecular alterations were examined by RNA-sequencing, ChIP, Western blotting, real-time RT-PCR, immunoprecipitation and immunohistochemistry. Cancer cell phenotypes were assessed by MTT, glucose consumption, migration and invasion assays. The orthotopic mouse model of HNC metastasis was used to evaluate the metastatic potential of tumor cells after the indicated gene modulations.

**Results and Discussions**

Both GLS1 and c-Myc show increased expression in primary HNC tumors, with further upregulation in metastatic HNC tumors. Bioinformatic analysis of the TCGA HNC dataset revealed a robust correlation between the expression of c-Myc and GLS1. We then elucidated the significance of the reciprocal regulation between c-Myc and GLS1. Specifically, c-Myc protein binds directly to the promoter of the GLS1 gene, resulting in transcriptional upregulation. Interestingly,

© 2024 The Authors. Molecular Oncology is published by John Wiley & Sons Ltd on behalf of Federation of European Biochemical Societies when we inhibited GLS1 signaling in HNC cells by lentiviral shRNA knockdown or treatment with the GLS1 inhibitor CB-839, this resulted in the downregulation of USP1, a well-characterized human deubiquitinating enzyme. Subsequently, reduced USP1 levels result in decreased c-Myc protein stability via the ubiquitin-proteasome pathway. Most importantly, this newly identified GLS1-c-Myc positive feedback loop played a critical role in driving HNC to lymph node metastasis in our orthotopic mouse models of HNC, and its blockade by targeted pharmaceutical agents significantly suppressed metastasis.

**Conclusion**

Given the continued challenge in treating HNC, these groundbreaking discoveries offer a molecular foundation for the potential combination of GLS1-specific inhibitors with therapies targeting c-Myc in the treatment of patients with metastatic HNC.

### EACR2024-0117

### Characterizing the interplay between neddylation and isomerization in the Ubiquitin-Proteasome System regulation

D. kantar[1], K. Bachiri[1], E. Laurent[1], E. Coyaud[1]
[1]PRISM, Interactomics, Villeneuve d'asq, France

**Introduction**

Merkel cell carcinoma (MCC) is a rare and highly malignant neuroendocrine cancer that is often associated with the Merkel cell polyomavirus (MCPyV). Current treatment options, including surgery and immunotherapy, are limited due to tumor resistance. The purpose of this study was to explore alternative therapeutic options by investigating the interaction between MCPyV large T antigen (LT) and PIN1, a protein that plays a role in cancer progression and resistance to therapy. Additionally, we examined the role of PIN1 in regulating protein ubiquitination via neddylation in key cancer-related pathways. By understanding the molecular mechanisms underlying these processes, we aim to identify novel targets for more effective treatment strategies for MCC.

**Material and Methods**

First, we inhibited PIN1 and NEDD8 in cells and assessed their effects on SCF complex substrates. Mutants of PIN1, RBX1, and NEDD8 were generated to investigate their interactions. Techniques such as Immunoprecipitation-Mass Spectrometry, proximity-dependent biotinylation, proximity ligation assay, and fluorescence resonance energy transfer will be employed to analyze protein interactions and conformational changes in the SCF complex. Additionally, loss-of-function experiments using siRNAs and commercially available drugs against PIN1 and NEDD8 will further elucidate their functional roles.

**Results and Discussions**

The results of proximity interaction assays indicate that PIN1 is situated in close proximity to the LT and the components of the SCF/C complex, particularly RBX1, a component of the SCF/C complex. Our objective is to establish that PIN1 interacts with RBX1 via NEDD8, a post-translational modification of the Cullin protein, which activates the complex. Additionally, we are examining whether PIN1 causes a conformational change in the complex and whether its depletion impairs the turnover of SCF/C targets within the cell.

**Conclusion**

In conclusion, this study presents a unique mechanism that connects the isomerization of proteins and the neddylation system. To the best of our knowledge, this is the first time that such a link has been suggested. This study aims to shed light on the mechanism of action of virus-positive MCCs and provide new insights into the oncogenic mechanisms of MCCs. The findings from the PIN1 project are expected to make significant contributions to both fundamental and tumor biology research.

### EACR2024-0206

### Assessment of novel therapeutic treatments in KRAS mutant Colorectal Cancer using Patient-Derived Organoids

M. Lazzari[1], C. Esposito[2], D. Liberati[2], K. Guja-Jarosz[2], M. von Strauss[3], R. Peterli[3], B. Müller[3], L.M. Terracciano[4], M. Llerena-Coto[2], S. Piscuoglio[5]
[1]Humanitas University, Biomedical Science- Precision Medicine research laboratory, Milano, Italy
[2]Visceral Surgery and Precision Medicine Research Laboratory- University of Basel, Department of Biomedicine, Basel, Switzerland
[3]Clarunis- Department of Visceral Surgery- University Center for Gastrointestinal and Liver Diseases, St. Clara Hospital and University Hospital, Basel, Switzerland
[4]Anatomic Pathology Unit, Humanitas Research Hospital, Milano, Italy
[5]Precision Medicine research laboratory, Humanitas Research Hospital, Milano, Italy

**Introduction**

Colorectal Cancer (CRC) defines a complex family of tumors of the colon and the rectum that differ for localization, morphology, genomic stability/instability, and set of somatic mutations in *APC*, *KRAS*, *TP53/ SMAD2/4*. The acquisition of mutations in *KRAS* oncogene, especially at codon 12 and 13, leads to the constitutive activation of the KRAS/RAF/MEK/ERK pathway in 35-45% of CRC, favoring tumor progression and metastasis. Patient-derived organoids (PDOs) are 3D in vitro cultures that have been shown to be a valuable morphological and molecular surrogate of their tissue of origin and their response to drugs to be broadly resembling the clinical response. Considering that the biology of *KRAS*-targeting in CRC is not yet clarified, in this work we exploited PDOs to study changes in the molecular landscape of CRC following direct or indirect *KRAS*-targeting.

**Material and Methods**

We performed a drug screening using a targeted drug panel of 8 different compounds active on KRAS pathway at 0.1/0.5nM-10/30uM concentration range, and we evaluated the cell viability after 72 hours to calculate the $EC_{50}$ of each compound. We firstly treated the 2D normal colon mucosa NCM460D cells and the DiFi (*KRAS* WT), HCT116 ($KRAS^{G13D}$) and LS-174T ($KRAS^{G12D}$) CRC cells, then we repeated the drug screening on a cohort of 14 CRC PDOs harboring $KRAS^{G12D}$, $KRAS^{G12A}$, $KRAS^{G13D}$, $BRAF^{V600E}$ and *KRAS* WT.

Molecular Oncology 18 (Suppl. 1) (2024) 1–495
© 2024 The Authors. Molecular Oncology is published by John Wiley & Sons Ltd on behalf of Federation of European Biochemical Societies

**Results and Discussions**
We observed that CRC PDOs responded to the drug treatment similarly to 2D cell lines. However, the $EC_{50}$ value of each compound in CRC PDOs was shifted towards a lower or higher effective concentration. Interestingly, the novel $KRAS^{G12D}$ inhibitor MRTX1133 showed an effective $EC_{50}$ 20 times lower (50 nM) in $KRAS^{G12D}$ PDO compared to that in LS-174T cells (1 uM). In addition, CRC PDOs carrying the same $KRAS^{wt/mut}$ displayed a comparable dose-response curve profile towards a given compound, but different $EC_{50}$ value. The variation in drug responses in the same *KRAS* status might be possibly explained by the coexistence of different patient-specific tumor cell clones in the CRC PDOs, that ultimately affect the overall CRC PDOs behavior.
**Conclusion**
Our preliminary data greatly support PDOs as a more reliable platform to explore new therapeutic strategies in CRC, since they maintain the patient inter/intra-tumor heterogeneity. Next, we plan to integrate our data with omics profiling and single-cell resolved multiplexed protein readout to deepen our understanding in KRAS perturbed signaling network following targeted drug treatment in CRC.

### EACR2024-0272
### Expression modulation in PI3K/AKT pathway genes induced by a 3rd generation alkyl-phospholipid (erufosine) in breast cancer cells

*A. Pervaiz[1], A. Shahzad[1], K. Umer[1]*
*[1]University of Health Sciences, Institute of Biomedical and Allied Health Sciences, Lahore, Pakistan*
**Introduction**
PI3K/AKT is a frequently de-regulated pathway in cancers and promote the active proliferation of transformed cells. Considering this, exploring novel anti-cancer agents to target this pathway is inevitable. Erufosine is a 3rd generation alkyl-phospholipid and interacts with the endogenous phospholipids of cell membranes to induce antineoplastic affects. The current study was designed to explore expressional changes induced by erufosine in PI3K/AKT pathway related genes in the breast cancer cells.
**Material and Methods**
Toxic effects of erufosine on the breast cancer cell lines (MDA-MB-231 and MCF-7) were assessed via MTT dye reduction assay and inhibitory concentrations (IC) were determined by using GraphPad Prism 8 software. Afterwards, the cells were exposed to $IC_{70}$ (20-40μM) concentration of erufosine for 48h followed by RNA extraction and cDNA synthesis. Expression changes in 84 PI3K/AKT related genes were investigated by using a ready-made $RT^2$ real-time PCR panel (Qiagen, PAHS-058Z). Results were compared with the untreated cells grown in parallel and fold changes were determined by using Livak method. Ingenuity Pathway Analysis was used to design the signalling cascade by using expressional data sets.
**Results and Discussions**
Erufosine substantially altered the expression of PI3K/AKT pathway related genes as shown by a de-regulation (≥2fold) of 26/84 (31%) and 38/84 (45%) genes in MDA-MB-231 and MCF-7 cells, respectively. Overall, a total of 10/84 (12%) and 74/84 (88%) genes were up- and down-regulated in MDA-MB-231 cells, respectively. In MCF-7 cells, 29/84 (35%) and 55/84 (65%) genes were up- and down-regulated, respectively. As far as most extreme de-regulations are concerned, GRB10 (2.3) and TLR4 (16) were the up-regulated, while CDKN1B (-4.6) and PIK3R2 (-5.6) were the down-regulated genes in MDA-MB-231 and MCF-7 cells, respectively. Ingenuity Pathway Analysis showed distinct positioning of the genes in software based designed PI3K/AKT pathway.
**Conclusion**
Erufosine showed substantial potential to de-regulate PI3K/AKT pathway related genes in breast cancer cells. Receptor positive (MCF-7) were more sensitive towards erufosine exposure as compared to receptor negative breast cancer cells (MDA-MB-231). Further studies are needed to understand the fine tuning being operated by erufosine in breast cancer cells to exploit its clinical utilization overtime.

### EACR2024-0273
### Effects of a 3rd generation alkyl-phospholipid (erufosine) on apoptosis related multiple genes in breast cancer cells

*A. Shahzad[1], A. Pervaiz[2], A. Javed[2], K. Umer[2]*
*[1]University of Health Sciences, Institute of Biomedical & Allied Health Sciences, Lahore, Pakistan*
*[2]University of Health Sciences, Institute of Biomedical and Allied Health Sciences, Lahore, Pakistan*
**Introduction**
Apoptosis is a major hallmark of cancer, where transformed cells modify the associated pathways for long-term survival. Search for anti-cancer agents with substantial potential to induce apoptosis in tumor cells is a continuous process. Erufosine, a 3rd generation alkyl-phospholipid molecule, has shown cytotoxic effects against various cancers including breast cancer in pre-clinical settings. Erufosine interacts with the endogenous lipids of cell membrane and induces anti-neoplastic effects. Present study was designed to understand the expression modifications in apoptosis related multiple genes induced by erufosine in breast cancer cells.
**Material and Methods**
Two distinct breast cancer cell lines (MDA-MB-231: receptor negative, MCF-7: receptor positive) were cultured and exposed to already known $IC_{70}$ concentration (20-40μM) of erufosine for 48h. Following the exposure period, total RNA was extracted and cDNA were synthesized from the treated and untreated cells. Expressional modulations in the 84 apoptosis related genes were determined by using a real-time PCR based ready-made panel (Qiagen, PAHS-020Z).). After the normalization of data, fold changes were determined by Livak method. Ingenuity Pathway Analysis was applied to design a proposed signaling pathway by using the expressional data sets.

© 2024 The Authors. Molecular Oncology is published by John Wiley & Sons Ltd on behalf of Federation of European Biochemical Societies

**Results and Discussions**
Among the two cell lines, receptor positive MCF-7 cells were more sensitive towards erufosine as compared to receptor negative MDA-MB-231 cells. Considering a substantial de-regulation (≥2fold), a total of 40/84 (48%) genes were altered in MCF-7 cells as compared to 15/84 (18%) in MDA-MB-231 cells. Overall, 48/84 (57%) genes showed up- and 36/84 (43%) down-regulation in MCF-7 cells. Likewise, 28/84 (33%) genes showed up- and 58/84 (67%) down-regulation in MDA-MB-231 cells. When considered the most extreme de-regulations, BIRC3 (21fold) and BCL2A1 (3.3fold) were the most up-regulated genes in MCF-7 and MDA-MB-231 cells, respectively. Similarly, TNFSF8 (-14fold) and DAPK1 (-5.3fold) were the most down-regulated genes in MCF-7 and MDA-MB-231 cells, respectively. The designed pathway showed discrete positions of the genes in signaling cascades.
**Conclusion**
Erufosine showed a remarkable potential to de-regulate multiple apoptosis related genes in breast cancer cells. Expression alterations were distinct in the two breast cancer cell lines. Further investigations are needed to understand reasoning behind the imposed de-regulations and their subsequent clinical utilization in breast cancer therapeutics.

### EACR2024-0300
### Communication between tumor cells and fibroblasts triggers a persister phenotype in triple negative breast cancer

*S. Wiemann[1], V. Golde[1], I.U. Zabala[1], E.I. Vlachavas[1], M. Vetter[2], M. Berdiel-Acer[1], M. Bauer[3], K. Schüler[3], C. Thomsson[2], A. Maia[1]*
*[1]German Cancer Research Center DKFZ, Division of Molecular Genome Analysis, Heidelberg, Germany*
*[2]Martin Luther University Halle-Wittenberg, Department of Gynaecology, Halle Saale, Germany*
*[3]Martin Luther University Halle-Wittenberg, Department of Pathology, Halle Saale, Germany*

**Introduction**
Neoadjuvant chemotherapy (NACT) is the standard of care for the treatment of triple negative breast cancer (TNBC), however, patient outcome is variable. Tumor physiology and response to therapy are orchestrated by an intricate interplay between cancer and stromal cells collectively forming the tumor microenvironment. We have previously uncovered a feedback mechanism of tumor cells and fibroblasts, involving IFNB1 signaling, which supports tumor cells in the recovery from chemotherapy-induced stress. Clinical relevance was established as expression of target proteins of IFNB1 signaling correlates with therapy success, i.e. pathological complete response (pCR), in TNBC patients after NACT.
**Material and Methods**
Patient-derived fibroblast cell lines were established and co-cultured with various breast cancer cell lines that had previously been incubated with different chemotherapeutic drugs. Drug concentrations were selected to kill the vast majority of cells. Colony formation capacity of persisting tumor cells was measured when kept in mono- or co-culture. Various genes were knocked down in tumor cells and fibroblasts to obtain mechanistic knowledge on the feedback mechanism. Expression of interferon stimulated genes was recorded as proxy of an interferon response. Following NACT, surgical material (tumor bed/residual disease) was fixed and embedded in paraffin. IHC was performed with antibodies targeting interferon stimulated proteins and expression correlated with pCR/non-pCR.
**Results and Discussions**
The introduction of carboplatin has substantially improved pCR rates in TNBC patients. This drug neither triggers an interferon response nor tumor cell recovery in vitro. Mechanistically, IFNB1 is induced by several classes of chemotherapeutic drugs some of which are applied according to current treatment algorithms (AGO). Expression of IFNB1 seems to not involve classic nucleic acid-sensing pathways. IFNB1 then elicits JAK/STAT signaling as well as an antiviral response in fibroblasts and in tumor cells via paracrine and autocrine signaling, respectively. While IFNB1 signaling in fibroblasts is prerequisite for recovery of persisting tumor cells, this pathway seems not to be relevant in the tumor cells in the context of the recovery from chemotherapy.
**Conclusion**
Mechanistic knowledge could have impact on the choice of therapeutic drugs applied in NACT of TNBC patients. These drugs should not elicit an interferon-response, like carboplatin, or interfere with the feedback loop between tumor cells and fibroblasts.

### EACR2024-0314
### Inhibition of notch signaling in non-small-cell lung cancer attenuates tumoral phenotype

*M.G. Stinson[1], M.J. Mosna[1], F.J. Garde[1], C.D. Pastore[1], M.F. Ogara[2], A. Sabater[1], A.L. Carcagno[1]*
*[1]Instituto de Química Biológica de la Facultad de Ciencias Exactas y Naturales IQUIBICEN- CONICET-UBA, Departamento de Química Biológica- Facultad de Ciencias Exactas y Naturales- Universidad de Buenos Aires, Ciudad Autónoma de Buenos Aires, Argentina*
*[2]Instituto de Fisiología, Biología Molecular y Neuro-ciencias IFIBYNE- CONICET-UBA, Facultad de Ciencias Exactas y Naturales- Universidad de Buenos Aires, Ciudad Autónoma de Buenos Aires, Argentina*

**Introduction**
Lung cancer is the second most frequent cancer worldwide, and it is also the leading cause of cancer-related deaths globally. Non-small-cell lung cancer (NSCLC) represents approximately 85% of all lung cancer cases. 55% of NSCLC patients receive a diagnosis at advanced disease stages, with presence of distant metastases, and have a 5-year relative survival rate of only 9%. Accumulating evidence points to the relevance of developmental pathways in carcinogenic processes. Notably, intercellular communication via the Notch signaling pathway is implicated in several developmental processes. During development of the lung, Notch signaling plays crucial roles in processes such as proximo-distal patterning, cell fate choice, cell proliferation, and apoptosis. This work aims to evaluate the role of Notch signaling in the tumorigenicity of NSCLC cells.

Molecular Oncology 18 (Suppl. 1) (2024) 1–495
© 2024 The Authors. Molecular Oncology is published by John Wiley & Sons Ltd on behalf of Federation of European Biochemical Societies

**Material and Methods**
We used DAPT, a pharmacological inhibitor of the gamma-secretase, to downregulate the activity of the Notch pathway in A549 human lung adenocarcinoma cells as a model for NSCLC. A549 cells were treated for 7 to 9 days with 40 µM DAPT. Cellular viability was evaluated by Trypan Blue exclusion assay. MTT reduction assay was used as a measure of metabolic state and/or proliferation, depending on the conditions in which the experiments were conducted. Cellular adhesion to the culture plate was evaluated by staining adhered cells with Crystal Violet, re-solubilizing the dye, and measuring its absorbance. Migration was evaluated through wound healing assay, proliferation through confluence assay, and clonogenicity through 2D clonogenic assay.
**Results and Discussions**
Notch inhibition was confirmed through RT-qPCR against Hes1, a canonical target of the Notch signaling pathway. Treated cells are larger than control cells, show reduced adhesion time to the culture plate, decreased proliferative and clonogenic capacities, and impaired migration. However, trypan blue exclusion experiments show no significant differences in viability between treated and control cells. Furthermore, spheroids of treated A549 cells are larger and appear to be less densely packed than control spheroids. Spheroids were also grafted onto the chorioallantoic membrane of chick embryos, where their original differences in dimensions were lost.
**Conclusion**
These results suggest that Notch signaling is relevant to the tumorigenicity of NSCLC cells, indicating its potential as a therapeutic target for lung cancer treatment.

### EACR2024-0339
### Natural coumarins derived from Magydaris tomentose exhibit anti-angiogenic properties via targeting VEGF-VEGFR signaling

*M.J. Hsu[1], S.W. Huang[1]*
*[1]Taipei Medical University, Department of Pharmacology, Taipei, Taiwan*
**Introduction**
Recent developments in drug discovery have highlighted plant-derived natural coumarins. It exhibits broad pharmacological properties such as anti-inflammatory, anti-coagulant, anti-bacterial, antioxidant, and anti-cancer activities. Given the established low toxicity and occurrence of coumarins in various herbal remedies, further exploring their bioactivities and underlying mechanisms appears prudent. From the seeds of *Magydaris tomentosa*, we have extracted and isolated a series of natural coumarins known as MT compounds (MT102~MT116). In this study, we aim to explore MT compounds' anti-angiogenic and anti-lymphangiogenic mechanisms.
**Material and Methods**
Effects of MT compounds on endothelial cell proliferation, migration, invasion, and activation of signaling molecules induced by VEGF, were analyzed by immunoblotting, MTT, BrdU, migration, and invasion assays. We performed a tube formation assay, aorta ring sprouting assay, and a mouse metastasis model to evaluate MT compounds' ex vivo and in vivo anti-angiogenic effects.
**Results and Discussions**
MT compounds inhibited VEGF-A-induced cell proliferation, migration, invasion, and endothelial tube formation of human umbilical vascular endothelial cells (HUVECs). MT compounds also reduced VEGF-C-induced cell proliferation, invasion, and endothelial tube formation of lymphatic endothelial cells (LECs). MT116 suppressed VEGF-A-induced microvessel sprouting ex vivo and reduced B16F10 melanoma lung metastasis. In addition, MT116 inhibited the phosphorylation of VEGFR-2 and its downstream signaling molecules in VEGF-A-stimulated HUVECs.
**Conclusion**
Together, MT116 may inhibit endothelial remodeling and suppress angiogenesis by targeting VEGF-VEGFR signaling. These results also suggest MT116 as a potential lead compound in developing novel agents in treating cancer and angiogenesis-related diseases.

### EACR2024-0352
### Natural plant extracts, MC, interfere with the VEGF-A/VEGFR2 pathway to suppress the angiogenesis process

*S.W. Huang[1], M.J. Hsu[1]*
*[1]Taipei Medical University, Department of Pharmacology, Taipei, Taiwan*
**Introduction**
Angiogenesis, the process that forms new capillaries from pre-existing blood vessels, is a prerequisite for tumor progression and metastasis. Among all the angiogenic factors, vascular endothelial growth factor (VEGF)-A plays the most critical role in physiological and pathological angiogenesis via activating its receptor VEGFR-2. Targeting VEGF-A/VEGFR-2 signaling, therefore, represents an effective therapeutic strategy in treating angiogenesis-related diseases, including cancer. Exploring natural products as drug leads remains a pivotal avenue in drug discovery. In this study, we are interested in investigating the bioactive natural products of *Muscari comosum,* which constitutes a prevalent botanical component in the Southern Italian diet.
**Material and Methods**
*Muscari comosum* extract (MC) isolated from MC plant were explored for their effects on endothelial cell proliferation, migration, invasion, tube formation, and signaling molecules activated by VEGF-A using the BrdU, migration, invasion, tube formation assays, and immunoblotting. An aorta ring sprouting assay and a mouse metastasis model were used to evaluate ex vivo and in vivo anti-angiogenic activities of MC compounds.
**Results and Discussions**
MC concentration-dependently suppressed VEGF-A-induced endothelial cell proliferation, migration, invasion, and tube formation of HUVECs. Ex vivo and in vivo animal models demonstrated that systemic administration of MC significantly suppresses microvessel sprouting and neovascularization. In addition, we found that MC suppresses the activation of

© 2024 The Authors. Molecular Oncology is published by John Wiley & Sons Ltd on behalf of Federation of European Biochemical Societies

VEGFR-2 and its downstream signaling cascades in VEGF-A-stimulated HUVECs.

**Conclusion**

Our findings showed that MC compounds inhibit angiogenesis by suppressing the VEGF-A/VEGFR2 signaling. These observations also support the role of MC compounds as potential drug candidates or using them as a lead for developing novel anti-angiogenic agents against angiogenesis-related diseases such as cancer.

### EACR2024-0407
### High-resolution analysis of protein interactions by Enzyme-activated Proximity of Oligonucleotide Sensing (EPOS)

*L. Wenson*[1]*, J. Heldin*[1]*, O. Söderberg*[1]
[1]*Uppsala University, Department of Pharmaceutical Biosciences, Uppsala, Sweden*

**Introduction**

Protein-protein interactions (PPIs) are essential for all processes in a cell, from signal transduction to proliferation. Dysregulated PPIs are known to be involved in cancer and other diseases and thus monitoring PPIs is pivotal in understanding cancer development and further in developing new treatments and drug targets. Our research group has focused on developing various methods for analysis of protein interactions such as in situ proximity ligation assay (in situ PLA). The PPIs detected by PLA are visualised by fluorescently tagged DNA strands created by rolling circle amplification (RCA), allowing single molecule detection of endogenous PPIs. Such RCA products have a size of approximately 1 µm in diameter. Consequently, the dynamic range in the analysis is limited and the resolution of the images is compromised as every molecule will be detected as a 1 µm object. To overcome these limitations, we developed enzyme-activated proximity of oligonucleotide sensing (EPOS) a method that is based on hybridization chain reaction (HCR) instead of RCA to generate a signal. This results in better resolution, improved efficiency of targeting PPIs, a more stringent threshold for detection at a lower cost.

**Material and Methods**

EPOS utilises proximity probes consisting of DNA hairpins that are conjugated to antibodies to detect PPIs. The hairpins are partially degraded by the enzymes Uracil-DNA glycosylase and EndonucleaseIV. This step transforms the oligonucleotides to expose single stranded elements, allowing the two different probes to hybridize together if the antibodies are bound in close proximity, i.e., bound to a protein complex. As a consequence of the hybridization between proximity probes, an initiator sequence will be exposed and initiate HCR of fluorophore-labelled hairpins. The HCR products, consisting of multiple fluorophore-labelled hairpins, will generate a long, nicked double-stranded DNA molecule attached to the proximity probes that can be visualised under a fluorescent microscope.

**Results and Discussions**

We benchmarked EPOS against in situ PLA using different, well-known PPIs such as E-cadherin and β-catenin or SMAD3 and SMAD4. As the HCR product is shorter, but with increased numbers of fluorophores, every molecule detected is be smaller and no longer detected as defined objects. This increases the resolution in PPI detection and provides an improved dynamic range.

**Conclusion**

Here we show that EPOS can be used to detect PPIs and furthermore delivers superior results compared to in situ PLA.

### EACR2024-0438
### Crosstalk between oncogenic signaling pathways as drivers of therapy resistance against small molecule inhibitors in Glioblastoma

*M. van Heumen*[1]*, L. Hoosemans*[2]*, F. Rapino*[3]*, A. Hoeben*[4]*, M. Vooijs*[2]
[1]*Maastro Clinic/Maastricht University, Radiotherapy, Maastricht, The Netherlands*
[2]*Maastricht University, Radiotherapy, Maastricht, The Netherlands*
[3]*University of Liege, Giga Cancer, Liege, Belgium*
[4]*MUMC, Medical Oncology, Maastricht, The Netherlands*

**Introduction**

Glioblastoma (GBM) is the most common malignant primary brain tumor in adults, with a poor prognosis of ~15 months. Despite multimodal treatment schedules including surgery, radio- and chemotherapy, tumor recurrence is inevitable. An important challenge in GBM treatment involves intratumoral heterogeneity, characterized by molecularly, phenotypically, and clinically distinctive GBM subtypes within a single tumor. This emphasizes the need for a more individualized precision targeting treatment approach. Different small molecule inhibitors (SMI) have been designed to inhibit key signaling proteins in oncogenic driver pathways, including EGFR, PDGFR, ME, CDK4, and PI3K/Akt. However, these SMI's have been unsuccessful in improving GBM patient outcome. Here, we investigate if crosstalk and redundancy in signaling pathways are responsible for single-agent resistance using molecular defined patient-derived glioblastoma 2D stem cell cultures (pdGBM).

**Material and Methods**

Eight BBB-penetrable and clinically approved SMI's were used to target key GBM driver genes. Cell viability was measured via CellTiter-Glo and the IC50 for each inhibitor was defined for six molecularly distinct pdGBMs. Changes in phosphokinase activity after SMI therapy was assessed via WB and phosphokinase screening. Based upon this, SMI combinations were made, targeting compensatory pathways driving treatment resistance. Bliss scores were used to test synergistic SMI combinations.

**Results and Discussions**

Targeting downstream effector kinases (MEK, PI3K, CDK4; IC50 70nM-1µM) or membrane-bound tyrosine kinase receptors (IC50 1-15µM) reduced cell viability, however remaining cell proliferation was seen in all pdGBM using the SMIs as single agent. WB analysis demonstrated on-target SMI drug effects and

© 2024 The Authors. Molecular Oncology is published by John Wiley & Sons Ltd on behalf of Federation of European Biochemical Societies compensatory upregulation of related signaling pathways (MEK–PI3K crosstalk). Phosphokinase screen profiling showed significant upregulation of specific and common kinases, such as GSKβ upon MEK-, PI3K- or CDK4-inhibition. SMI combinations (EGFRi+MEKi+CDK4i) based on molecular subtypes showed synergistic effects in $EGFR_{wt}$ (bliss>10) but not in $EGFR_{mut}$ pdGBMs (bliss<10), showing the vital role of tumour mutational burden and intratumoral heterogeneity in SMI resistance.

**Conclusion**

This study presents a mechanistically driven selection of tumor tailored combination treatments to overcome resistance to SMI monotherapy in pdGBM. The efficacy of these novel SMI combinations will be challenged next in combination with standard of care radio- and chemotherapy.

### EACR2024-0464
### Canonical transient receptor potential isoform 3 (TRPC3) promotes triple negative breast cancer migration through regulating TRPC3-NFATc1-GPC6-vinculin signaling

*S.Y. Tsang*[1], *Y. Wang*[1], *X. Zhuang*[1]
[1]*The Chinese University of Hong Kong, School of Life Sciences, Hong Kong, Hong Kong- China*

**Introduction**

Canonical transient receptor potential isoform 3 (TRPC3), a calcium-permeable non-selective cation channel, has been reported to be upregulated in breast cancer. However, whether TRPC3-mediated calcium influx would affect the migration of triple negative breast cancers (TNBC) cells, and, if yes, the underlying mechanisms involved, remain to be investigated.

**Material and Methods**

Immunostaining followed by confocal microscopy and western blotting were used to detect the expression and/or subcellular localization of protein. Lentiviral vector was used for stable gene knockdown. Chromatin immunoprecipitation was used to detect transcription factor and DNA interaction. Co-immunoprecipitation was used to detect protein-protein interaction. Wound healing was used to assess cancer cell migration.

**Results and Discussions**

TNBC lines MDA-MB-231 and BT-549 were both found to express TRPC3 on their plasma membrane while $ER^+$ line MCF-7 and $HER2^+$ line SK-BR3 do not. Blockade of TRPC3 by pharmacological inhibitor Pyr3 or stable knockdown of TRPC3 by lentiviral vector both inhibited cell migration. Importantly, blocking TRPC3 or knockdown of TRPC3 both caused the translocation of NFATc1 from the nucleus to the cytosol. Interestingly, NFATc1 was found to bind to the promoter of glypican 6 (GPC6). Consistently, knockdown of TRPC3 decreased the expression of GPC6. In addition, GPC6 protein was found to physically interact with vinculin. Intriguingly, knockdown of TRPC3 or knockdown of GPC6 all induced larger, stabilized actin-bound peripheral focal adhesion (FA) formations in TNBC cells as determined by co-staining of actin and vinculin. These large, stabilized actin-bound peripheral FAs indicated a defective FA turnover, and were reported to be responsible for impairing polarized cell migration.

**Conclusion**

Our results suggest that, in TNBC cells, calcium influx through TRPC3 channel positively regulates NFATc1 nuclear translocation and GPC6 expression, which maintains the dynamics of FA turnover and optimal cell migration. Our study reveals a novel TRPC3-NFATc1-GPC6-vinculin signaling cascade in maintaining the migration of TNBC cells.

### EACR2024-0504
### Identification and Validation of a Disease-modifying Target for Aberrant PI3K/AKT Activation in Gastric Cancer

*H.Y. Lam*[1], *S.S. Shikha*[1], *M.K. Shanmugam*[1], *T.Z. Tan*[2], *Y. Ito*[2], *S.L. Chan*[3], *P. Tan*[4], *G. Sethi*[1], *A.P. Kumar*[1]
[1]*National University of Singapore, Department of Pharmacology, Singapore, Singapore*
[2]*National University of Singapore, Cancer Science Institute of Singapore, Singapore, Singapore*
[3]*National University of Singapore, Department of Surgery, Singapore, Singapore*
[4]*Duke-NUS Medical School, Program in Cancer and Stem Cell Biology, Singapore, Singapore*

**Introduction**

Despite the advancements in treatment strategies, major limitations in gastric cancer treatment include the lack of prognostic and predictive markers, high-dose toxicity, eventually declining the overall survival rates of gastric cancer patients. The PI3K pathway is frequently mutated and aberrantly activated in gastric cancer and many other cancers and plays a central role in tumor cell proliferation and survival, making it a rational therapeutic target. However, results from clinical trials with PI3KCA inhibitors in solid tumors have been largely disappointing. Some of the challenges include the lack of biomarker predictive of clinical response, suboptimal patient selection in trials, and drug-related toxicities. Our lab has previously identified DP103, a DEAD-box RNA helicase, as a prognostic and predictive marker in breast cancer. We hypothesize that DP103 may similarly serve as a novel therapeutic target for gastric cancer.

**Material and Methods**

We performed tissue microarray analyses and immuno-histochemistry staining to examine the expression of DP103 in gastric carcinoma patient samples. Gastric cancer cells were transfected with small interfering RNAs (siRNAs) targeting DP103 to investigate its roles in gastric cancer in vitro. For in vivo investigation of DP103's roles in tumorigenesis, we utilized the xenograft mouse model and the drosophila model.

**Results and Discussions**

We first demonstrate the upregulated DP103 protein levels in gastric cancer, where a relatively high proportion of patients are known to harbor gain-of-function PIK3CA mutations. Our clinical data indicate a positive correlation between DP103 expression and PIK3CA (the PI3K/Akt/mTOR pathway-associated gene that encodes for p110α). Additionally, we show that DP103 physically interacts with PI3KCA to bring about constitutive activation of the PI3K/Akt activity and

© 2024 The Authors. Molecular Oncology is published by John Wiley & Sons Ltd on behalf of Federation of European Biochemical Societies recruits the PI3K complex to the membrane in gastric cancer.

**Conclusion**

This study has provided evidence for the clinical use of DP103 expression levels as selection criteria for patients in PI3K/Akt inhibitor trials, paving the way for precision medicine and improved clinical outcomes. Exploring strategies either targeting DP103 helicase activity or using stapled peptides to disrupt the DP103-p110α axis hold immense potential for advancing treatment efficacy.

### EACR2024-0512
### Validated GPCR recombinant monoclonal antibodies for cancer research

*A. Ball[1], Y.L. Hsieh[2], C.K. Huang[2], S. Huang[2], S.R. Wu[2], J. Liu[3], P.S. Peng[2], C.Y. Lin[4]*
*[1]GeneTex- Inc., Product Development, Irvine, USA*
*[2]GeneTex- Inc., Product Development, Hsinchu, Taiwan*
*[3]GeneTex- Inc., Information Technology, Hsinchu, Taiwan*
*[4]GeneTex- Inc., Chief Operating Officer, Hsinchu, Taiwan*

**Introduction**

Recombinant monoclonal antibodies (rAbs) are vital reagents for cancer biology research as they offer reproducible performance and scalable supply. A major goal of antibody manufacturers is to utilize rAb technology to create new reagents against difficult targets for which there are few or no reliable products. G protein-coupled receptors (GPCRs) are seven-pass transmembrane domain receptors involved in a plethora of physiological and pathophysiological processes that contribute to various chronic diseases, including many malignancies. Approximately 60-85% of therapeutically relevant GPCRs have no agents directed against them, including GPCRs that may be involved in oncogenesis, metastasis, and chemotherapy resistance as well as those whose connection to cancers has already been reported. The development and validation of high-quality, specific antibodies against GPCRs is essential for advancing understanding of GPCR biology and for evidence-based antineoplastic drug design.

**Material and Methods**

GeneTex is combining its recombinant antibody production platform with enhanced validation protocols to manufacture novel monoclonal antibodies against GPCRs with direct relevance to cancer. GeneTex employs a multi-parameter FACS-based method to select antigen-specific IgG+ memory B cells from an immunized rabbit (Starkie et al., 2016). The heavy and light chain variable region genes from single cells that produce an IgG with reactivity to the targeted GPCR are amplified, cloned, and co-expressed in mammalian cells to generate the functional antibody. Application-specific testing is performed in conjunction with knockout/ knockdown (KO/KD), differential expression comparison in cells and tissues, cell fraction enrichment, and other testing. Antibodies directed against extracellular GPCR domains are validated for specificity using GPCR arrays (CDI Labs, Baltimore, MD).

**Results and Discussions**

This process has resulted in the production of well-characterized rAbs for multiple GPCRs studied by cancer biologists. Among these are new monoclonal reagents against the dopamine D2 receptor (DRD2), retinoic acid-induced protein 3 (RAI3), chemokine receptors CXCR2, CXCR4, and CXCR7, melanocortin receptor 1 (MC1-R), and somatostatin receptor type 3 (SSTR3). Antibody specificity was demonstrated with KO/KD, antibody comparison, and/or testing on the GPCR arrays.

**Conclusion**

The approach described above has successfully generated a series of reliable GPCR monoclonal antibodies and will be applied to the remaining non-olfactory GPCRs.

### EACR2024-0559
### Comparative transcriptomics on tumoral, adjacent and normal prostate tissues

*C. Payan[1], H.M. Guevara-Nieto[2], D. Rodriguez-Morales[3], E. Vargas[3], S. Ramirez-Clavijo[4], R. Parra-Medina[5]*
*[1]Universidad Nacional de Colombia, Biology, Los Robles La Paz, Colombia*
*[2]Instituto Nacional de Cancerologia, Cancer Biology Research Group, Bogotá, Colombia*
*[3]Instituto Nacional de Cancerologia, Pathology, Bogota, Colombia*
*[4]Universidad del Rosario, Biology, Bogota, Colombia*
*[5]Instituto Nacional de Colombia, Pathology, Bogota, Colombia*

**Introduction**

Prostate cancer presents a significant global health concern, with its progression, treatment response, and metastasis greatly influenced by the tumor micro-environment. However, there's a gap in research directly comparing adjacent tissue behavior with both tumor and normal tissue. To address this, we developed an analytical approach to identify gene alterations in adjacent tissue without requiring comparison to tumor or normal tissue.

**Material and Methods**

We conducted searches for datasets containing information on adjacent tumor and normal tissue, as well as transcriptomic data from the prostate cancer project in TCGA for adjacent tumor samples. Differentially expressed genes (DEGs) were identified from the dataset to directly compare adjacent tissue with normal tissue. These DEGs were then used as probes to identify other genes of interest within the TCGA database. Our hypothesis posited that genes exhibiting correlated expression with the DEGs would also show altered expression in adjacent tissue within TCGA. Subsequently, a co-expression network was constructed using all genes displaying a correlation coefficient greater than 0.6 with at least one of the DEGs, followed by network analysis.

**Results and Discussions**

Among the datasets examined, GSE6919 was the only one enabling direct comparison of adjacent tissue to prostate cancer with normal tissue. From this dataset, six differentially expressed genes (DEGs) were identified: TRIB1, C18orf54, ARF1, NFKBIZ, LINC00662, and MAFB. Analyzing the transcriptomes of 54 adjacent tissues to prostate cancer from TCGA revealed that the expression profiles of 3,316 genes had a Pearson correlation coefficient greater than 0.6 with at least one

© 2024 The Authors. Molecular Oncology is published by John Wiley & Sons Ltd on behalf of Federation of European Biochemical Societies of the six DEGs. Using these genes, a co-expression network was constructed, retaining 3,254 genes. Two distinct networks emerged: one comprising 2,234 genes enriched with genes related to protein synthesis and vesicle transport from the endoplasmic reticulum to the Golgi apparatus, and another consisting of 395 genes enriched with genes from the IL17 and TNF signaling pathways.

**Conclusion**

The method proposed demonstrated efficacy in identifying genes potentially altered in adjacent tissue to the tumor, even without normal tissue for comparison. Several pathways identified have known associations with cancer progression, metastasis, and treatment response. Further investigation is needed to ascertain whether the behavior of these pathways, since adjacent tissue holds clinical significance for prostate cancer.

### EACR2024-0662
### Tumor-immune hybrid cells evade immune and potentiate liver metastasis of colorectal cancer via CTLA4

*P. Tanjak[1], A. Chaiboonchoe[2], T. Suwatthanarak[1], K. Thanornjit[1], O. Acharayothin[3], J. Chanthercrob[2], T. Parakonthum[3], J.M. Fischer[4], M.H. Wong[5], V. Chinswangwatanakul[3]*

*[1]Faculty of Medicine Siriraj Hospital- Mahidol University, Siriraj Cancer Center, Bangkok, Thailand*
*[2]Faculty of Medicine Siriraj Hospital- Mahidol University, Siriraj Center of Research Excellent for Systems Pharmacology, Bangkok, Thailand*
*[3]Faculty of Medicine Siriraj Hospital- Mahidol University, Department of Surgery, Bangkok, Thailand*
*[4]Oregon Health & Science University, Cancer Early Detection Advanced Research Center, Portland, USA*
*[5]Oregon Health & Science University, Knight Cancer Institute, Portland, USA*

**Introduction**

Immune evasion is one of cancer hallmarks. The tumor-immune hybrid cell is hypothesized as a subset of cells that evolves the capacity to disseminate and seed metastases.

**Material and Methods**

To explain the mechanism of metastasis by tumor-immune hybrid cells, we investigated circulating tumor hybrid cells in the peripheral blood of patients with stage IV colorectal cancer. Then, we analyzed the presence and molecular mechanism of tumor-immune hybrid cells in tissues of primary colorectal cancers and their liver metastasis sites by using several techniques: immuno-fluorescence, spatial proteomic, spatial transcriptomic, molecular classification, and molecular pathway analysis.

**Results and Discussions**

Our finding indicated a high prevalence of circulating tumor hybrid cells in stage IV colorectal cancer patients. Circulating tumor hybrid cell surface signature (CK+/EpCAM+/CD45+) allowed to identify tumor-immune hybrid cells in primary colorectal cancer and matched liver metastasis tissues. Tumor-immune hybrid cells showed the strongest relation to induce CTLA4 in primary colorectal cancer lesion that might influence to up-regulate CD68, CD4 and HLA-DR in metastatic liver lesions. Additionally, the upregulation of genes, *CD68*, *CD4,* and *CLA4*, highly found in the consensus molecular subtype 1 primary colorectal cancer tissue. These gene features were used to determine the critical molecular pathways of tumor-immune hybrid cells in primary colorectal cancers and their metastases. The results of pathway analysis suggested that the features of tumor-immune hybrid cells associated with neutrophils, both neutrophils extracellular trap signaling (NET), and neutrophil degranulation pathways.

**Conclusion**

These data provide the critical molecular pathways of tumor-immune hybrid cells in immune evasion and support neutrophils, which may facilitate colorectal cancer cells into different tissues. Tumor-immune hybrid cells and their molecules may represent a potential target for immunotherapy in colorectal cancer.

### EACR2024-0700
### Stratification of wild-type RAS cancer cells by RAS/pathway activity using imaging-based protein-interaction analysis informs next-gen RAS-inhibitor therapy strategy

*D. Koschut[1], A. Venkitaraman[1,2]*

*[1]Agency for Science Technology and Research ASTAR, Institute of Molecular and Cell Biology IMCB, Singapore, Singapore*
*[2]National University of Singapore NUS, Cancer Science Institute CSI, Singapore, Singapore*

**Introduction**

RAS is a notoriously challenging drug target, but next-generation drugs can aim for specific KRAS-mutants while also new pan-RAS inhibitors are in development. Mutations in RAS can lead to increased RAS-GTP levels, however, also abnormal signalling upstream can lead to wt RAS activation. Nevertheless, most patient stratifications rely on identifying RAS mutations in tumors. Cancer types with high RAS mutation frequencies include prevalent cancers such as colorectal and lung carcinoma. Importantly, other actionable mutations in these cancers are often upstream or downstream of RAS, while 20-40% of these cancers have no actionable mutation and unknown RAS activity status.

**Material and Methods**

Cell lines with no mutations in any RAS/downstream protein were grouped by highest or lowest KRAS knockout effect to establish a model system. Cell lines of each group were cultured to characterize their RAS pathway activity using a high-content imaging (HCI) approach to preserve spatial quantification. Proximity-ligation assay (PLA) and HCI were combined to screen for endogenous protein-protein-interactions (PPI) in RAS:RAS-effector proteins suitable as biomarkers for RAS activity. Furthermore, immuno-fluorescence (IF) of multiple RAS-related pathway proteins - and their activity - allowed to quantify contextual and spatial effects of elevated (or blocked) RAS activity in the same sample.

**Results and Discussions**

Analysis of 1095 cell lines uncovered 5 cancers in which cell lines wt for RAS showed increased KRAS-dependency. In cells of these cancers, a screen for PLA

© 2024 The Authors. Molecular Oncology is published by John Wiley & Sons Ltd on behalf of Federation of European Biochemical Societies pairs identified at least 3 biomarkers to measure RAS interactions. Following HCI-PLA/IF, a principal component analysis revealed the most relevant features to distinguish between cells with high and low RAS/ pathway activity — independent of mutation status. Notably, the wt RAS cells grouped for high RAS activity were more sensitive to RMC-6236 (RAS-IN-2) pan-RAS inhibitor and elevated mTOR signalling was visualized as compensatory drug response. The identified biomarkers, e.g. PTPN11:RAS PPI level, are highly translatable: Only a small number of cells and no genetic manipulation are required, allowing to detect RAS activity in patient cells and histological tumor slices with no RAS mutation, improving cancer patient stratification.

**Conclusion**

Stratification of wt RAS cancers by RAS activity can identify more patients eligible for treatment with RAS inhibitors and may also inform RAS treatment resistance/ relapse strategies for both wt and mutated RAS tumors.

### EACR2024-0735
### A personalized HER2-targeted therapy, Neratinib can be repurposed as a therapeutic approach for triple-negative breast cancer by suppressing the STAT3/EGFR signaling axis

M. Joshi[1,2], P. Dey[1,2], S. Dimri[1,2], S. Ghosh[1,2], A. De[1,2]

*[1]Advanced Centre for Treatment Research and Education in Cancer ACTREC- Tata Memorial Centre- Kharghar- Navi Mumbai - 410210- India, Molecular Functional Imaging Lab, Navi Mumbai, India*

*[2]Homi Bhabha National Institute- Mumbai- India, Life Science, Mumbai, India*

**Introduction**

Triple-negative breast cancer (TNBC) is the most aggressive subtype of breast cancer, due to its molecular heterogeneity, high metastatic potential, and lack of recognized molecular targets for therapy. Previous work from the lab shows that non-canonical (pS727) STAT3 activation is predominantly expressed in TNBC patients and is independent of canonical (pY705) activation, which can be a potential target for TNBC treatment. In this study, we aimed to target the EGFR-STAT3 signaling axis as EGFR is known to regulate S727-phosphorylation of STAT3, using an FDA-approved drug, Neratinib (a pan-HER family inhibitor).

**Material and Methods**

TCGA data was used to analyze EGFR expression and patient survival in TNBC cohorts. The RNA expression was measured with Q-PCR and the protein levels were measured by western blot and immunofluorescence. EGFR protein knockdown was done using TRIM-Away. MTT assay, clonogenic assay, and flow cytometry were done to examine cell viability, cell proliferation, and apoptosis, respectively. BRET (Bioluminescence energy transfer assay) was used to assess STAT3 dimerization and activation in the live cells. Wound-scratch assay and Boyden-chamber assay were done to check the migratory and invasive properties of TNBC cells, respectively.

**Results and Discussions**

We found that EGFR is overexpressed in TNBC patients and patients having high EGFR expression show poorer outcome survival. EGFR overexpression was also validated in cell lines and cells having EGFR overexpression showed STAT3 overexpression and activation. Targeting EGFR signaling with EGFR inhibitors (Neratinib and Gefitinib) or EGFR knockdown led to a decrease in both canonical (Y705) and non-canonical (S727) activation of STAT3. Next, we identified that Neratinib exhibits an inhibitory effect on STAT3 activation in TNBC by targeting the EGFR-ERK-STAT3 signaling axis. Neratinib inhibited STAT3 dimerization and nuclear localization, thus down-regulating its target genes, such as (VEGF, vimentin, and c-myc). Further, Neratinib-mediated STAT3 inhibition suppressed growth and proliferation, induced apoptosis, and decreased migration and invasiveness of TNBC cells.

**Conclusion**

This study provides the first evidence that targeting EGFR signaling with Neratinib decreases non-canonical STAT3 activation and suppresses its tumor-promoting characteristics in TNBC. As EGFR is overexpressed in a subtype of TNBC patients, we propose that Neratinib has the potential as a novel EGFR/STAT3 inhibitor for treating TNBC in the future.

### EACR2024-0741
### The molecular background determines LSD1 inhibition sensitization of AML cells to gilteritinib and other kinase inhibitors

P. Casado Izquierdo[1], N. Afroz Nishat[1], S.F. Pedicona[1], S. Yasmin[1], S.M. Martin-Guerrero[1], V. Rajeeve[1], P.R. Cutillas[1]

*[1]Barts Cancer Institute / Queen Mary University of London, Centre for Genomics and Computational Biology, London, United Kingdom*

**Introduction**

Acute myeloid leukaemia (AML) is a disease with adverse prognosis. Epigenetic and kinase signalling are frequently dysregulated in AML and drugs targeting epigenetic enzymes and kinases have been tested in clinical trials to improve AML treatment. However, an incomplete understanding of their molecular effects in specific patient populations has resulted in modest clinical efficacy of drugs targeting such enzymes. Here, we hypothesise that the molecular background of AML determines the precise combinations of epigenetic and kinase targeting drugs that more effectively disrupt the dependencies that drive AML.

**Material and Methods**

Mega-erythroid (HEL, KMOE2 and CMK) and monocytic (MV4-11, P31 and NOMO1) AML cells were treated with the LSD1 inhibitor (LSD1i) GSK-2879552 for five days, followed by treatments with inhibitors of FLT3 (midostaurin and gilteritinib), MEK (trametinib), and PI3K (alpelisib, TGX-221, duvelisib, idelalisib and pictilisib aimed for α, β, δ/γ, δ and all isoforms) for three days. We subsequently measured synergistic reductions of cell viability. Additionally, proteomics and phospho-proteomics based on mass spectrometry was used to quantify protein expression and phosphorylation in cells treated with different inhibitor combinations.

**Results and Discussions**

LSD1i made MV4-11 cells more dependent on FLT3 inhibitors and compounds that target kinases (MEK and

Molecular Oncology 18 (Suppl. 1) (2024) 1–495

© 2024 The Authors. Molecular Oncology is published by John Wiley & Sons Ltd on behalf of Federation of European Biochemical Societies

PI3Kδ) acting downstream of FLT3. Phosphoproteomics showed that LSD1i increased the phosphorylation of the stress response kinases MAP3K forms 7, 11, and 20, MAP4K4, RIPK3, STK3 and TAOK2. LSD1i also sensitized the FLT3-ITD negative cell lines HEL and KMOE2 to gilteritinib LSD1i sensitized HEL cells to alpelisib and pictilisib and P31 cells to trametinib. LSD1i induced monocytic markers in both cell lines, but reduced erythroid markers and increased the expression of energy metabolism enzymes in HEL cells. LSD1i reduced histone H3K79 methylation only in P31 cells and inhibitors of DOT1L, the enzyme that methylates H3K79, sensitized P31 cells to MEKi but not HEL cells to PI3Ki. These data suggest that the LSD1i induced sensitization to MEKi in P31 cells involves DOT1L, while the sensitization to PI3Kis in HEL cells may involve the lost of erythroid differentiation and energy metabolism alterations in a DOT1L independent manner.

**Conclusion**

LSD1 inhibition sensitizes AML cells to kinase inhibitors in a model-dependent manner with DOT1L being involved in AML cells with specific molecular backgrounds.

### EACR2024-0860
### ERBB2 circumvents negative feedback mechanisms induced by EGFR overactivation in basal-like/triple-negative breast cancer cells

*C. Miano[1,2,3], F. Sacchi[1,2,3], S. Da Pra[1,3], I. Del Bono[1,3], C. Bongiovanni[1,2,3], S. Boriati[1,3], S.L. Siragusa[1,3], M. Lauriola[1], G. D'Uva[1,2,3]*
*[1]University of Bologna, Department of Medical and Surgical Sciences, Bologna, Italy*
*[2]National Institute of Biostructures and Biosystems INBB, National Laboratory of Molecular Biology and Stem Cell Engineering, Bologna, Italy*
*[3]University of Bologna, Centre for Applied Biomedical Research CRBA, Bologna, Italy*

**Introduction**

Breast cancer is the most widespread malignancy affecting women worldwide. While targeted therapies have been effectively utilized in clinics for hormone-responsive and HER2+ subtypes, therapeutic options for triple-negative/ basal-like breast cancer subtypes are still limited to date. High levels of epidermal growth factor receptor (EGFR) expression have been linked to these subtypes; however, efforts to target this receptor have yielded disappointing outcomes. This study aimed to evaluate the impact of chronic EGFR activation due to long-term exposure to various doses of EGF in quasi-normal basal-like/triple-negative breast cells.

**Material and Methods**

Quasi-normal basal-like/triple-negative MCF10A breast cells and their derivative cells with ERBB2 over-expression (MCF10A-HER2) were employed. These cell models were chronically stimulated with varying doses of EGF. The expression, activation, and dimerization of EGFR and ERBB2 were characterized, along with cell proliferation, differentiation, migration, anchorage-independent growth, and responsiveness to anti-EGFR and anti-HER2 targeting agents.

**Results and Discussions**

Prolonged EGFR activation induced by high EGF doses reduced the abundance of EGFR and ERBB2 proteins, and decreased cell proliferation, migration, and anchorage-independent growth. ERBB2 overexpression circumvented the feedback mechanism activated by chronic exposure to high EGF doses, partially preventing the downregulation of EGFR abundance and activation. High EGF doses failed to inhibit the proliferation and migration of HER2-overexpressing cells and further increased their ability to grow in suspension. EGFR and ERBB2 drove anchorage-independent growth of HER2+ basal-like/triple-negative breast cancer cells. However, the efficacy of anti-EGFR agents in suppressing this process appeared to be dose-dependently reduced by EGF and completely impaired by high EGF doses, whereas the administration of anti-ERBB2- agents efficiently blocked this ability even upon treatment with high EGF doses.

**Conclusion**

Our data suggest that ERBB2 provides the ability to bypass negative feedback mechanisms activated by high EGF doses, enhancing anchorage-independent growth. We propose that targeting ERBB2, alone or in combination with anti-EGFR agents, could be an effective approach to inhibit the metastatic progression of basal-like/triple-negative breast cancer cells.

### EACR2024-0928
### Escape from TGF-β-induced Senescence Promotes Aggressive Cancer Hallmarks in Epithelial Hepatocellular Carcinoma Cells

*M. Kalyoncu[1], D. Demirci[1], S. Eris[1,2], B. Dayanc[1,2], E. Cakiroglu[1,2], M. Basol[2,3], M. Uysal[1,2], G. Cakan-Akdogan[3,4], G. Karakülah[2,5], S. Senturk[1,2,6]*
*[1]Izmir Biomedicine and Genome Center, Basic and Translational Research Program, Izmir, Turkiye*
*[2]Izmir International Biomedicine and Genome Institute/ Dokuz Eylul University, Department of Genomics and Molecular Biotechnology, Izmir, Turkiye*
*[3]Izmir Biomedicine and Genome Center, Industrial R&D Program, Izmir, Turkiye*
*[4]Dokuz Eylul University School of Medicine, Department of Medical Biology, Izmir, Turkiye*
*[5]Izmir Biomedicine and Genome Center, Technological Research Program, Izmir, Turkiye*
*[6]Bilkent University, Department of Molecular Biology and Genetics, Ankara, Turkiye*

**Introduction**

Transforming growth factor-β (TGF-β) signaling and cellular senescence are key hallmarks of hepatocellular carcinoma (HCC) pathogenesis.Despite acting as a tumor-suppressor by inducing senescence-associated growth arrest in epithelial HCC cells,chronic elevation of TGF-β correlates paradoxically with aggressiveness and poor prognosis in advanced HCC.The mechanisms underlying this transition and the role of senescence escape in this context remain understudied.

**Material and Methods**

TGF-β resistant cells (Huh7-TR) were generated by chronic exposure of Huh7 cells to TGF-β.Smad knock-down cells were created via retroviral RNAi system.The loss of TGF-β sensitivity was confirmed by SA-β-

© 2024 The Authors. Molecular Oncology is published by John Wiley & Sons Ltd on behalf of Federation of European Biochemical Societies

Gal,BrdU incorporation,cell cycle,FUCCI reporter and luciferase reporter assays.The relationship between TGF-β resistance and aggressive traits was demonstrated by EMT-related gene expression profiling,2D and3D growth assays, as well as invasion and metastasis assays.Gene expression profiles of TGF-β sensitive and resistant states were generated through global transcriptome analysis.Restoration of cytostatic responses to TGF-β activity were explored through targeted depletion of genes using CRISPR/Cas9 or RNAi techniques. Subcellular signaling dynamics of Smad molecules and TGF-β/Smad3 transcriptional activity were assessed by immunofluorescence,BrdU incorporation and luciferase assays.

**Results and Discussions**

Chronic TGF-β exposure promotes escape from Smad3-mediated senescence and induces TGF-β resistance in Huh7 cells.Escaping senescence was associated with the acquisition of mesenchymal features,while loss of TGF-β sensitivity is linked to defective TGF-β/Smad signaling. Due to significant variations in transcriptome profiles between Huh7 and Huh7-TR cells,we conclude that TGF-β resistance involves the simultaneous activation of multiple genes or pathways. Through genetic ablation and molecular studies,we identified GRM8 (Glutamate Metabotropic Receptor 8) as a critical modulator of the resistance phenomenon,potentially by disrupting subcellular dynamics of Smad activity.

**Conclusion**

Our findings unveil a novel phenomenon wherein epithelial HCC cells may exploit senescence evasion as a mechanism to oppose TGF-β anti-tumor responses and progress towards more aggressive HCC phenotypes. Further studies supported by high-throughput functional assays such as drug or CRISPR screens are warranted to help inform a better understanding of how these,and potentially other genes,contribute to escaping senescence and committing to resistance.

### EACR2024-0939

### Targeting HER3 in Cancer Therapy: Pioneering Approaches through DNA Vaccination and Monoclonal Antibody Development

*L. Luberto[1], A. Muzi[2], F.F. Ferrara[2], R. Arriga[2], M. Cappelletti[2], A. Conforti[3], E. Marra[1], L. Aurisicchio[4], G. Roscilli[2]*

*[1]Takis srl, Preclinical Disease models for Infection-Cancer and Therapeutics, Rome, Italy*

*[2]Takis srl, Monoclonal antibodies development, Rome, Italy*

*[3]Evvivax, Cancer vaccines, Rome, Italy*

*[4]Takis srl, Cancer Immunotherapy, Rome, Italy*

**Introduction**

The human epidermal growth factor receptor 3 (HER3), a pivotal member of the ErbB receptor family, is integral to cellular regulation and oncogenesis, particularly in mediating drug resistance. This positions HER3 as a strategic target for innovative cancer therapies. Our research introduces a dual-pronged approach targeting HER3 through a therapeutic vaccine and a novel monoclonal antibody strategy, with potential extensions to vectorized antibody expression and advanced antibody-based therapeutics.

**Material and Methods**

We developed a genetic cancer vaccine employing DNA vectors encoding either the full length of HER3 (HER3_FL) or its extracellular/transmembrane domains (HER3_ECD-TM), delivered via DNA electro-gene-transfer (DNA-EGT) into BALB/NeuT mice. This method is designed to induce a specific immune response against tumor cells, establishing long-term immunologic memory to prevent cancer relapse. Concurrently, we explored a monoclonal antibody strategy, generating a series of hybridomas against human ErbB3 through DNA-EGT. These antibodies were selected for their ability to inhibit the ErbB3-mediated signalling pathway effectively.

**Results and Discussions**

Our results show that DNA-EGT vaccination elicited a robust immune response against HER3, significantly preventing cancer onset in prophylactic settings and slowing tumor progression in therapeutic models. Moreover, the anti-ErbB3 monoclonal antibodies demonstrated in vitro growth modulation of cancer cells and in vivo antitumor properties across various cancer models.

**Conclusion**

Crucially, our approach opens pathways for further innovations, including the development of vectorized antibody expression systems for sustained in vivo antibody production. Additionally, our anti-ErbB3 monoclonal antibodies hold promise as the foundation for creating antibody-drug conjugates (ADCs) or T cell-engaging bispecific antibodies, offering targeted and potent therapeutic options against HER3-expressing tumors. This comprehensive dual-strategy approach not only underscores the therapeutic potential of targeting HER3 but also illustrates the versatility and expansiveness of our methodologies. By pioneering these strategies, our work lays the groundwork for novel cancer treatment paradigms, demonstrating the significance of HER3 as a therapeutic target and the effectiveness of innovative therapeutic strategies in the fight against cancer.

### EACR2024-0952

### Hypoxia and ER signalling regulate the breast cancer growth- and metastasis-associated glycerophosphodiesterase EDI3

*K. Rohlf[1], A. Glotzbach[1], K. Edlund[1], R. Marchan[1]*

*[1]Leibniz Research Centre for Working Environment and Human Factors at the TU Dortmund IfADo, Toxicology, Dortmund, Germany*

**Introduction**

EDI3 (*GPCPD1*) is highly expressed in human ER-HER2+ breast cancer tissues and cell lines compared to other subtypes. Silencing EDI3 in ER-HER2+ cells reduced cell viability, adhesion and anoikis resistance in vitro, and slowed tumour growth and metastasis in mice. However, little is known about EDI3's regulation in cancer. Our initial studies revealed that EDI3 is regulated downstream of HER2 via PI3K/Akt/mTOR and GSK3β and identified potential transcription factors that impact

Molecular Oncology 18 (Suppl. 1) (2024) 1–495
© 2024 The Authors. Molecular Oncology is published by John Wiley & Sons Ltd on behalf of Federation of European Biochemical Societies

EDI3 expression, including HIF1α. Since HIF1α is a key regulator of the cell's hypoxic response, we explored how hypoxia affects EDI3 expression, and the impact of HIFs on EDI3 expression under hypoxia and normoxia. EDI3 expression is especially high in HER2+ tumours that are also ER- and ER signalling is reported to influence HIF1α expression; therefore, we also investigated a role of ERα in EDI3 regulation.

**Material and Methods**

Chromatin immunoprecipitation and the electrophoresis mobility shift assay were used to investigate binding of candidate transcription factors, including HIF1α, to the promoter region of EDI3. Breast cancer cells, including SKBR3, HCC1954 and MCF7, were exposed to 1% hypoxia, or treated with hypoxia mimetics, and EDI3 mRNA expression was analysed with qRT-PCR. *ESR1* or *HIF1A* expression was silenced using siRNA and ER was inhibited with fulvestrant.

**Results and Discussions**

EDI3 expression was consistently reduced under hypoxic or hypoxia-mimicking conditions in all breast cancer cell lines tested, regardless of molecular subtype. This supports our previous finding that inhibiting GSK3β reduces EDI3 expression, since GSK3β is reported to phosphorylate HIF1α, causing its inactivation. Silencing *HIF1A* increased EDI3 expression, even in normoxia, suggesting that the regulation of EDI3 by HIF1α may be independent of its role in hypoxia. Furthermore, knocking down EDI3 did not affect *HIF1A* expression but reduced the expression of HIF2α (*EPAS1)*. Finally, we show that downregulating ERα expression in an ER+ breast cancer cell line resulted in upregulation of EDI3 expression.

**Conclusion**

EDI3 expression decreases under hypoxic conditions, suggesting that its tumour promoting effect is oxygen dependent. High EDI3 expression may support survival under oxygenated conditions such as during bloodstream dissemination. The complex interplay among ER, HER2 and hypoxia-related factors in relation to EDI3 in breast cancer will be further investigated.

### EACR2024-0959

### Therapeutic Potential of Novel Tetrahydroquinoline Derivatives: Targeting EGFR Signaling to Modulate the ROS-mediated mitochondrial pathway in Glioblastoma Multiforme

*S. Koochakkhani*[1], *D. S. N. Branco*[2], *A. Murugesan*[1], *N. R. Candeias*[2], *M. Kandhavelu*[1]

[1]*Tampere University, Molecular Signaling Group- Faculty of Medicine and Health Technology, Tampere, Finland*

[2]*University of Aveiro, LAQV-REQUIMTE- Department of Chemistry, Aveiro, Portugal*

**Introduction**

Current treatment for Glioblastoma Multiforme (GBM) is not efficient due to its aggressive nature, infiltrative behavior, and chemotherapy resistance. Novel therapeutic strategies are immediately required to address these challenges effectively. This study explores the cytotoxic potential of eight newly developed tetrahydroquinoline derivatives targeting the Epidermal Growth Factor Receptor (EGFR) signaling pathway in GBM cells.

**Material and Methods**

Tetrahydroquinoline derivatives were designed and synthesized using the Petasis borono-Mannich reaction. The cytotoxicity of the synthesized derivatives was evaluated using trypan blue exclusion assays on GBM cell lines. Apoptosis induction and the mechanism of action were explored through measurements of reactive oxygen species (ROS) levels, propidium iodide (PI) and annexin V staining, Caspase-3/7 activity assays, mitochondrial membrane potential (ΔΨm) assays, and calcium ion measurements.

**Results and Discussions**

Among eight derivatives, the 4-trifluoromethyl substituted derivative (4ag) was identified as a potent derivative against GBM cells, demonstrating its efficacy superior to the standard chemotherapy agent temozolomide (TMZ), with $IC_{50}$ values of 38.3 µM and 40.6 µM in SNB19 and LN229 cell lines, respectively. We observed that 4ag triggers the accumulation of ROS upon EGFR inhibition, leading to mitochondrial dysfunction and ultimately apoptosis. This process involves an increase in the intracellular ROS, further elevating the mitochondrial ROS level, ultimately inducing cell death by activating Caspase-3/7 and disrupting ΔΨm. Moreover, while 4ag effectively modulates ROS levels and mitochondrial function, it exerts no significant impact on cellular calcium dynamics in GBM cells.

**Conclusion**

These results highlight the therapeutic potential of tetrahydroquinoline derivative targeting EGFR signaling, offering insights for innovative GBM treatment strategies.

### EACR2024-1079

### FAT1 is a key regulator of resistance to therapy -induced ferroptosis in head and neck squamous cell carcinoma

*S. Stoiber*[1,2], *O. Boras*[1,2], *L. Kadletz-Wanke*[3], *L. Kenner*[1,2,4,5]

[1]*Medical University of Vienna, Department of Pathology, Vienna, Austria*

[2]*Medical University of Vienna, Christian Doppler Laboratory for Applied Metabolomics CDL-AM, Vienna, Austria*

[3]*Medical University of Vienna, Department of Otorhinolaryngology- Head and Neck Surgery, Vienna, Austria*

[4]*CBmed GmbH, Center for Biomarker Research in Medicine, Graz, Austria*

[5]*University of Veterinary Medicine, Unit of Laboratory Animal Pathology, Vienna, Austria*

**Introduction**

Head and neck squamous cell carcinoma (HNSCC), the sixth most common cancer worldwide, takes the lives of approximately 500,000 people annually. Traditional therapies, such as radiotherapy (RT), show limited success, especially in a patient sub-group. Researchers have independently shown that ferroptosis, a type of regulated cell death, can sensitize therapy-resistant

© 2024 The Authors. Molecular Oncology is published by John Wiley & Sons Ltd on behalf of Federation of European Biochemical Societies tumors to RT and that the loss of *FAT1*, a member of the cadherin family that is activated upon cell-to-cell contact, might contribute to the treatment-resistance of HNSCC. This study aims to explore the role of FAT1 in RT- and Erastin-, a potent SLC7A11 inhibitor, induced ferroptosis.

**Material and Methods**

Publicly available and in-house sequencing datasets were mined to interrogate the connection of *FAT1* loss/ expression and treatment response and survival. Furthermore, the role of ferroptosis in preclinical models of RT was studied and connected to the mutational state of *FAT1* in HNSCC cell lines (*FAT1* wildtype (WT): Cal27 and SCC25; *FAT1* loss: FaDu). A doxycycline-inducible FAT1 knockdown (KD) was employed to study the molecular changes in Cal27 and SCC25 cells upon FAT1 KD and their response to Erastin / RT was investigated.

**Results and Discussions**

Mining of patient data revealed frequent mutations in *FAT1* (~15%) which correlated with shorter survival times. FaDu (*FAT1*-deficient) showed increased resistance to RT and Erastin in comparison to Cal27 and SCC25 (*FAT1* WT). The observed resistance of FaDu was highly associated with cell confluency – the denser the monolayer, the more resistant FaDu became. Intriguingly, this phenomenon was not present in the other cell lines, which might indicate that FAT1 activation upon cell contact sensitizes cells to ferroptosis. FaDu also showcased an increased expression of SLC7A11, a major counterplayer of ferroptosis, at higher cell confluency, which might be linked to increased activity of the MAPK/ERK pathway, which is known to modulate SLC7A11 levels. Indeed, we observed increased levels of p-ERK in FaDu. Accordingly, FAT1 KD in Cal27 and SCC25 led to increased RT/Erastin resistance at higher confluency, hyperactivation of the MAPK/ERK pathway, and increased expression of SLC7A11.

**Conclusion**

Our data demonstrates that the inferior survival of *FAT1*-mutant patients might be due to FAT1's role in ferroptosis regulation, thereby influencing RT efficacy. Hence, we propose to screen for changes in *FAT1* or downstream targets, such as p-ERK or YAP/TAZ, to identify patients which might benefit the most from RT.

### EACR2024-1080

### Integration of multi-omics approaches in Head and Neck Cancer and analysis of circadian rhythm and hypoxia role in tumor progression

*D. Lenoci*[1], *M. Terramagra*[2], *M. Lucchetta*[2], *E. Torchia*[3], *S. Cavalieri*[3], *D. Mattavelli*[4], *P. Bossi*[5], *D. Ferrari*[6], *L. Licitra*[3], *L. De Cecco*[2]

[1]*Fondazione IRCSS Istituto Nazionale dei tumori, Department of Research- Integrated biology of rare tumors, Milan, Italy*

[2]*Fondazione IRCCS Istituto Nazionale dei Tumori, Department of Research- Integrated biology of rare tumors, Milan, Italy*

[3]*Fondazione IRCCS Istituto Nazionale dei Tumori di Milano, Head and Neck Medical Oncology Department, Milan, Italy*

[4]*Spedali Civili di Brescia and University of Brescia, Department of Otorhinolaryngology Head and Neck Surgery, Brescia, Italy*

[5]*IRCCS Humanitas Research Hospital, Medical Oncology and Hematology Unit, Milan, Italy*

[6]*Ospedali Santi Paolo e Carlo, Medical Oncology, Milan, Italy*

**Introduction**

Head and Neck squamous cell carcinoma (HNSCC) is the 6th most deadly tumor worldwide. The majority of patients are detected in late stages. The whole transcriptome, transcrition factors (TF) and miRNA allow to improve our knowledge on the disease. The hypothesis is that the integration of gene-miRNA expression may disclose the biological differences in different stages in order to construct the natural history of the disease and improve prognostic prediction and guidance of the optimal treatment choice.

**Material and Methods**

The study population was composed by late stages, 370 from BD2Decide(NCT02832102) and 123 from B490 (2011-002564-24) and early stages, 171 (INT239/20). RNA was isolated from FFPE using FFPE kit (Qiagen). GE and ncRNA libraries were prepared using Lexogen and miRNAKit(Qiagen), respectively and sequenced on a NextSeq500. Only HPV negative were considered. The integration of gene-miRNA expression data was performed. The differential expression analyses (DE) was carried out using DESeq2 and the enrichment using Reactome. Mirnetwork was used to evaluate the network with a topological-structural level.

**Results and Discussions**

Circadian rhythm was considered because poorly studied in HNSCC. It was upregulated in Stage I/II and down-regulated in B490. In Stage I/II, HIF1A composed the main network, targeted by 14 miRNA, and it controlled different TF. HIF1A was linked with CLOCK by BHLHE40 and CLOCK with CRY1, CRY2, PER3, PER2, NPAS2. In the BD2, HIF1A create big network and was targeted by 7 miRNA and controlled TF. Only miR-3609 which target HIF1A, was in common in StageI/II and BD2. PER2,CRY1,CRY had nodes directly with NPAS2. CLOCK was not expressed in BD2. In B490, BHLHE40 built the biggest node and, among a big number of TF, controlled CLOCK and HIF1A. PER2, CRY1, CRY2 were not expressed. ATF2 was in common in the three cohort. Survival analyses using genes and miRNA involved in circadian rhythm and hypoxia, in BD2 and B490 cohorts was performed. In BD2, the genes and miRNA with prognostic role were CRY2, ESR1, PER2, miR-33a-5p, miR-196b-5p, miR-328-3p, miR-421, miR-432-5p and miR-1299. In B490, the prognostic genes and miRNA were ACAP3, CORO28, ETS1, HDAC9, IRF1, MMP2, TP53, ZEB2, let-7g-3p and miR-362-3p.

**Conclusion**

Hypoxia was linked to circadian rhythm and the network of some miRNA and genes in late stages were present in Stage I/II and they changed target among the stages. Thus, allow us to detect the malignant progression of tumor already in Stage I/II and find future new target therapy.

© 2024 The Authors. Molecular Oncology is published by John Wiley & Sons Ltd on behalf of Federation of European Biochemical Societies

### EACR2024-1141
### Role of proteins involved in epithelial integrity and polarity, regulated by (de)phosphorylation by Syk or PTPN13, in mammary tumour invasion

*F. Lakis*[1,2], *P. Barraja*[1], *G. Freiss*[1], *P. Coopman*[1], *F. Abdel-Sater*[2], *M. Peter*[1]
[1]*IRCM- Institut de Recherche en Cancérologie de Montpellier, Inserm U1194- University of Montpellier-ICM- CNRS, Montpellier, France*
[2]*Laboratory of Cancer Biology and Molecular Immunology, Faculty of Sciences-I- Lebanese University, Hadath, Lebanon*

**Introduction**

The spleen tyrosine kinase (Syk) and tyrosine-protein phosphatase non-receptor type 13 (PTPN13) were described in hematopoietic cells with pro-oncogenic effects. Our team and others observed, however, that Syk and PTPN13 are also expressed in non-hematopoietic cells and established their role as tumor suppressors in mammary epithelial cells. To better understand their negative role in breast cancer, our team recently demonstrated a role of these proteins in cell junction stabilization (Hamyeh et al., Theranostics, 2020; Kassouf et al.,Cancers, 2019). However, the downstream signaling effectors of Syk and PTPN13 remain largely unknown.

**Material and Methods**

Using phosphoproteomics and interactomics approaches, we identified Rho guanine nucleotide exchange factor 7 (ARHGEF7) and Par-3 family cell polarity regulator (PARD3) as common putative effectors of Syk and PTPN13. In this study, we aim to investigate whether these proteins are direct partners or substrates of Syk and PTPN13. Their function, consequences of their (de)phosphorylation and role in epithelial integrity in 2D/3D models are being studied. In addition, we will investigate these proteins of interest in genetically-engineered mouse models in order to unravel their function in vivo. Based on the obtained results, clinical studies will later on be performed on breast cancer biopsies.

**Results and Discussions**

We showed that PARD3 and ARHGEF7 co-immunoprecipitate with Syk and PTPN13. Immuno-fluorescence analysis on MCF7 cells revealed the colocalization of Syk and PTPN13 with PARD3 and ARHGEF7 at cell-cell junctions. Notably, enhanced Syk expression and activation increased PARD3 recruitment at cell-cell junctions. Moreover, we developed various assays allowing us to explore PARD3 and ARHGEF7 functions and extended our studies to an additional breast cancer cell line, MCF10DCIS, clinically relevant for cancer research and drug development.

**Conclusion**

This study will characterize new signaling effectors through which Syk and PTPN13 exert their anti-proliferative and anti-invasive effects in epithelial cells, which may ultimately aid to identify novel biomarkers and therapeutic targets.

### EACR2024-1227
### Systematic Review of Intracellular Signaling Pathways in Prostate Cancer: Potential Therapeutic Targets and Biomarkers

*L.R. Rajmohan*[1], *P. p*[2], *P. d*[1]
[1]*ACS Medical College & Hospital, General Medicine, Chennai, India*
[2]*ACS Medical College & Hospital, Biochemistry, Chennai, India*

**Introduction**

Prostate cancer is a heterogeneous disease characterized by dysregulated intracellular signaling pathways contributing to disease progression and treatment resistance. Understanding these pathways is crucial for identifying potential therapeutic targets and biomarkers.

**Material and Methods**

A systematic review was conducted to identify key intracellular signaling pathways implicated in prostate cancer progression and treatment resistance. PubMed, Embase, and Scopus databases were searched for relevant studies. Studies investigating the role of signaling pathways in disease progression and treatment response were included.

**Results and Discussions**

Several intracellular signaling pathways, including the PI3K/AKT/mTOR pathway, the androgen receptor (AR) signaling pathway, and the MAPK pathway, are dysregulated in prostate cancer. Dysregulation of these pathways promotes tumor cell proliferation, survival, and invasion, leading to disease progression and treatment resistance. Novel therapeutic targets, such as PI3K, AKT, mTOR inhibitors, and AR pathway inhibitors, have emerged from the study of these signaling pathways. Additionally, biomarkers, including PTEN loss, TMPRSS2-ERG fusion, and AR-V7 splice variant, have shown clinical relevance for predicting treatment response and patient outcomes.

**Conclusion**

Understanding the dysregulated intracellular signaling pathways in prostate cancer provides opportunities for the development of targeted therapies and biomarkers to improve patient outcomes. Further research into these pathways is warranted to optimize treatment strategies and personalize patient care.

### Translational Research

### EACR2024-0005
### Spontaneous pneumothorax as the presentation of small cell lung carcinoma: A case report

*J. Buchanan*[1], *M. Shatila*[2]
[1]*University of Birmingham, Medical School, Birmingham, United Kingdom*
[2]*University Hospitals Birmingham, Department of Thoracic Surgery, Birmingham, United Kingdom*

**Introduction**

© 2024 The Authors. Molecular Oncology is published by John Wiley & Sons Ltd on behalf of Federation of European Biochemical Societies

A pneumothorax is a non-physiological collection of air in the pleural space. Pneumothoraces can be broadly divided into Primary, Secondary, and Traumatic. Cancer of the lung is a known cause of secondary pneumothorax in both primary and metastatic lesions, however, a pneumothorax as the presentation of lung cancer is exceedingly rare. Non-small cell lung carcinoma (NSCLC) has been reported in the literature to present with a pneumothorax, particularly in adeno/squamous cell carcinomas. It is almost completely unheard of for small cell lung carcinoma (SCLC) to present with a pneumothorax.

**Material and Methods**

An extensive literature review was conducted and analysis of, to the best of our knowledge, the fourth case of SCLC presenting initially with pneumothorax is presented.

**Results and Discussions**

We present the case of a 62-year-old male patient, presenting twice in two months with spontaneous pneumothorax. The initial management involved admission and chest drain insertion. The patient has a past medical history of chronic obstructive pulmonary disease (COPD) and a significant smoking history. On the second admission, he underwent a video-assisted thoracoscopic (VATS) bullectomy and talc pleurodesis. Intraoperative histology confirmed SCLC with extensive infiltration. No gross evidence of metastatic spread was present on computed tomography (CT). Due to the R1 resection and significant risk of recurrence, the management plan included four cycles of adjuvant chemotherapy with carboplatin and etoposide, and radiotherapy as a consideration upon completion.

**Conclusion**

Pneumothorax as the presentation of lung cancer imparts a very poor prognosis, however the reasons for this are largely unknown. Furthermore, the mechanisms underlying spontaneous pneumothorax in lung cancer are also not well understood. To the best of our knowledge, three cases have been previously reported of SCLC presenting with pneumothorax, making this the fourth. We hypothesise that, in the present case, oncogenesis occured due to accumulation of carcinogens in a peripheral bullous airspace, resulting in the presentation of recurrent spontaneous pneumathoraces.

### EACR2024-0015

### Irradiation of tumor tissues with non-thermal and non-ionizing microwave radiation increases the degree of binding of antitumor drugs to them

V. Kalantaryan[1], R. Martirosyan[1], Y. Babayan[2]
*[1]Yerevan State University, Radiophysics and Electronics, Yerevan, Armenia*
*[2]National University of Architecture and Construction., dept.Physics, Yerevan, Armenia*

**Introduction**

As known super and extremely high-frequency electromagnetic radiation (centimeter and millimeter waves) at a proper selection of exposure regimes can result in distinct and stable antitumor effects. The aim of the studies was to find out whether low-intensity electromagnetic radiation can affect the degree of binding of an anticancer drug to the affected tissues.

**Material and Methods**

DNA released from sarcoma 45 tumor (tDNA) and healthy rats (hDNA) in water-saline solution was irradiated during 90 min by frequencies at both resonances for oscillations of water molecular structures (at 64.5 GHz for triads and 50.3 GHz for hexagons) and non-resonance (48.3 GHz). The absorption spectra of non-irradiated and irradiated complexes of DNA with anti-tumorous drugs doxorubicin (DX) and netropsin (NT) were obtained by spectroscopic method. From the absorption spectra, binding constants K at 290K, 300K, and 310K temperatures have been determined.

**Results and Discussions**

When tumor DNA is irradiated at resonant for water molecular structures frequencies of 64.5 GHz and 50.3 GHz, anticancer drug forms more stable (much stronger) complexes, the binding constant K to DX and NT is almost an order of magnitude higher than for the non-irradiated DNA (for irradiated at 64.5 GHz frequency tumor DNA-DX complexes at 300K $K = 57.4 \cdot 10^{-5}\ M^{-1}$, and for non-irradiated $K = 7.4 \cdot 10^{-5}\ M^{-1}$). This indicates that the same antitumor effect can be achieved with much smaller doses of drugs, which is actually equivalent to increasing their effectiveness.

**Conclusion**

It is necessary to compare the dynamics of changes in tumor growth in three groups of animals: in the control group without irradiation, where animals receive the usual course of treatment, and in two (or more) experimental ones, where they receive smaller doses and are simultaneously irradiated. If the therapeutic effect is the same in all groups, then this will unequivocally confirm our preliminary assumption about the increase in the degree of binding of drug to tumor tissue when the latter is irradiated. This is important from the point of view of using gentle methods of treatment for patients and reducing the costs associated with the purchase of expensive medicines.

### EACR2024-0019

### Can nurses successfully do cancer Patient Advocacy : need in resource-poor settings

S. Pal[1], K. Gupta[2]
*[1]society for cancer care and prevention, oncology, meerut, India*
*[2]SFCCP, oncology, meerut, India*

**Introduction**

essence of Lung cancer patient advocacy & education is in providing specific knowledge about human rights of patients and in educating family on home based care. This profile suits very well for nurses. Informed and educated patients are able to save self-esteem, to establish good relationship with social environment and to achieve better social participation. The aim of this study was to make a public health policy paper on role of nurses in Patient Advocacy. resource poor settings patient advocacy is least priority in public health budget/planning lung cancer sufferers who return to villages in rural parts of India after taking chemotherapy/ surgery in city hospitals need this assistance.

© 2024 The Authors. Molecular Oncology is published by John Wiley & Sons Ltd on behalf of Federation of European Biochemical Societies

Community NGO's can play key role in providing cancer care including patient & family education.

**Material and Methods**

82 lung cancer patients (60 men & 22 women, age 50-60 yrs) included in analysis ( n=82), all matched criteria regarding age and cultural/educational level. 8 nurses having keen interest and aptitude for patient advocacy included and trained. Patients/relatives answered questionnaire specifically designed to assess image of Lung cancer patients and relationship with nurses. Impact of nurses on family support & rights of patient knowledge. Cancer advocacy/awareness efforts must be devised suitable to local communities. Due to lack of resources , this issue in lung cancer care has been neglected for last decade in asia.

**Results and Discussions**

cancer advocacy/education significantly improved self-image in lung cancer sufferers group when compared to control group (P<0,03). There was no significant difference after education between these group with regard to social relationship (P>0,03). Due to resource constraints sample size & evaluation parameters. Our NGO planning multi-institutional-collaborations to pilot project on this unexplored issue

**Conclusion**

Our cancer NGO has taken initiative on this front of lung-cancer patients advocacy/education. Nurses are important in establishing self management approach in which patients learn their rights, for changing their environment, and for planning their future. For successful Lung cancer patients' psychosocial adaptation and social participation, it is necessary that whole society provides more resources for nurses to become patient advocates. This is low cost approach to improve care outcomes in resource constrained settings. This issue is fertile ground for further studies by EACR interaction/participation.

### EACR2024-0021
### National oncology Registry development for lung cancer. Experiences by NGO nurses from Asia

*S. Pal*[1], *T. Roy*[2]

[1]*society for cancer care and prevention, oncology, meerut, India*

[2]*SFCCP, oncology, meerut, India*

**Introduction**

oncology Registry provides detailed information on incidence ,trend survival statistics. national level oncology registries are population-based & seek to describe incidence, rates, trends of oncology issues within set populations. provides info on staging, treatment, allied clinical data required to monitor clinical care/outcomes. Development of comprehensive database on lung cancer is long awaited in asia. Our NOT-FOR-PROFIT FORUM developed primary plan in consultation with four divisional hospitals & Health ministry. Aim to establish platform for multi-clinician, multi-centric collation of oncology datasets. Proposal of intent has been approved at national level.

**Material and Methods**

we volunteer nurses of this oncology forum relate our experience of initiative aimed at establishing methodology, statistical analysis & supportive control center for multicollaborator oncology disorders data collection, aiming to establish national oncology data repository. 6 IT college students developed this basic framework of this registry by creating online platform. Developed it in 5 months and data entry completed in 14 months.

**Results and Discussions**

Initiated from four sites, modern technology of data collection, storage, analysis & distribution is optimized towards implementation of sustained comprehensive and multi-collaborator data registry. Need for minimum datasets, customization of technology to suit needs, data capture, storage/retrieval. These can be leveraged to inform future direction of initiatives: expanding scope of database, optimizing variables for data analysis and addressing data privacy, security and ownership concerns. We developed our model database but need participation of private oncology institutes. Total participants projected by 2025 are 846.

**Conclusion**

Our experience with this initiative over past 3 years show :data be collated centrally in secure/private manner. Multicentre, multi-clinician collaboration is possible with Collaborative efforts with senior EACR researchers. major concern is haphazard data/protocol maintenance by private entities. Most difficult data outsourcing was about survival statistics. NATIONAL lung cancer Registry is distant dream in resource-poor-nations. But we youth volunteers have taken step in forward direction on this burning issue in Asian-settings.

### EACR2024-0045
### Knowledge, attitudes, and Current practices toward lung cancer palliative care management in China: a national survey

*M. Chen*[1], *H. Yang*[2], *J. Zhang*[3], *H. Yu*[4]

[1]*Chongqing University Cancer Hospital, Department of Clinical nutrition, Chongqing, China*

[2]*Chongqing University Cancer Hospital, Department of Geriatric Oncology and Department of Palliative care-, Chongqing, China*

[3]*Chongqing University Cancer Hospital, Department of Geriatric Oncology and Department of Palliative care, Chongqing, China*

[4]*yhqdyx@cqu.edu.cn, Department of Geriatric Oncology and Department of Palliative care, Chongqing, China*

**Introduction**

To demonstrate the status and differences in knowledge, attitudes, and practices (KAP) of lung cancer palliative care (LCPC) management, and to measure patient controlled analgesia (PCA) in cancer pain management in of China.

**Material and Methods**

A questionnaire on LCPC management was used in this study, which involved a total of 2093 participants from 706 hospitals in China. Seven major components make up the questionnaire, including chi-square tests or Fisher exact probabilities to measure the differences in KAP between hospitals grades. Comparing distributions of ordered data between groups was done using the Kruskal-Wallis H test or the Mann-Whitney U test. Multiple

© 2024 The Authors. Molecular Oncology is published by John Wiley & Sons Ltd on behalf of Federation of European Biochemical Societies choice questions use multiple response cross analysis. Correlation was evaluated by the Spearman correlation coefficient.

**Results and Discussions**

84.2% participants believed that anti-tumor therapy is equally important as palliative care. The satisfaction rate of participants from grade 3 hospitals, which was significantly higher than that of grade 2 and grade 1 hospitals ($\chi^2$=27.402, $P$=0.002). The most common symptoms requiring LCPC was pain. The major barriers toward to LCPC were “Patients and families are concerned about the safety of long-term use of palliative care related drugs”. The most common reasons for the use of PCA treatment were 31.1% participants thought “Patients with systemic application of large doses of opioids or adverse reactions to opioids that cannot be tolerated”. The top three barriers toward PCA treatment of cancer pain were (i) worry about adverse reactions of drug overdose, (ii) worry about opioid addiction, and (iii) increase of patients' economic burden. In the past 24 months, 33.9% of the participants had not participated in online or offline training related to palliative care of lung cancer.

**Conclusion**

Chinese healthcare workers are in need of training for lung cancer palliative care and, in particular, for controlling cancer pain symptoms.

### EACR2024-0049
### Lactobacillus metabolites relieves the rash and diarrhea adverse reaction induced by EGFR TKI: two case reports and literature review

Y. Huang[1], M. Chen[2]
*[1]Yunnan Provincial Corps Hospital of Chinese People's Armed Police Forces, Outpatient Department, Kunming, China*
*[2]Chongqing University Cancer Hospital, Department of Clinical nutrition, Chongqing, China*

**Introduction**

The association between gut microbiota and molecular targeted therapy for lung cancer has been attracting increasing interest. Rash and diarrhea are the most common adverse events caused by EGFR tyrosine kinase inhibitors (TKI) in patients with EGFRm advanced non-small cell lung cancer (NSCLC), which occurring within several days to weeks after treatment with EGFR TKI. Some patients have to stop taking EGFR TKI due to these adverse events, which further affected their survival and quality of life.

**Material and Methods**

Case report and literature review. The patient's medical record was reviewed for demographic and clinical data. For literature review, all case reports or other publications published in English literature were identified using PUBMED.

**Results and Discussions**

Herein, we report two cases of EGFRm advanced NSCLC with diarrhea and rash during EGFR TKI, followed by the treatment of lactobacillus metabolites, JK-5G and JK-21, and both their diarrhea and rash were relieved. Lactobacillus metabolites supplementation could improved the patients nutrition status and quality of life. This report emphasizes the lactobacillus metabolites could relieves the rash and diarrhea induced by EGFR TKI.

**Conclusion**

We first report that lactobacillus metabolites could improve patients nutrition and quality of life.

### EACR2024-0060
### Schwann Cell Insulin-like Growth Factor Receptor Type-1 Mediates Metastatic Bone Cancer Pain in Mice

F. De Logu[1], R. Nassini[2]
*[1]University of Florence, Department of Heakth Sciences, Firenze, Italy*
*[2]university of florence, department of health sciences, Florence, Italy*

**Introduction**

Pain is a recurrent symptom of cancer that becomes more frequent and debilitating in the presence of bone metastases, which is a common consequence of many primary tumors, including breast cancer (Rades et al., 2010). Treatment of metastatic bone cancer pain (MBCP) represents a major medical challenge, as current therapies are insufficient, resulting in psychological distress, anxiety, and significantly reduced quality of life (von Moos et al., 2017). The mechanism of MBCP involves several interactions between tumor and bone cells, activated inflammatory cells, and bone-innervating neurons (Andriessen et al., 2021; Zajaczkowska et al., 2019). Although bone resorption associated with the invasion of cancer cells has been considered as the primary cause of MBCP (Mantyh, 2006; Mantyh, 2014), knowledge of the cellular and molecular mechanisms underlying MBCP is limited.

**Material and Methods**

Female mice were used throughout (25–30 g, 5–8 weeks old). To generate mice in which the Trpa1 gene was conditionally silenced in Schwann cells, homozygous 129S-Trpa1$^{tm2Kykw/J}$ (floxed Trpa1, *Trpa1*$^{fl/fl}$,) were crossed with hemizygous B6.Cg-Tg(Plp1-CreERT) 3Pop/J mice (Plp-Cre$^{ERT}$, ) expressing a tamoxifen-inducible Cre in their Schwann cells (Plp1, proteolipid protein myelin 1) (De Logu et al., 2017). Human Schwann cells (HSCs) were used

**Results and Discussions**

In the present MBCP mouse model, E0771 breast cancer cells inoculated into the mouse mammary gland metastasize to the femur head and, via IL-8, subjugate osteoclasts to release osteolytic biomarkers, including IGF-1. The unexpected and major finding of the study is that the entire panel of pain-like behaviors evoked by bone metastasis depends on osteoclast-derived IGF-1, which, targeting the Schwann cell IGF-1R, converts the glial cell into an active proalgesic phenotype. IGF-1R activation in Schwann cells *via* a series of intracellular mediators and TRPA1 releases M-CSF that, promoting endoneurial rMΦs expansion, sustains pain-like behaviors.

**Conclusion**

We propose that the humoral communication between osteoclasts and Schwann cells converges in a final common pathway implicating a role of NO/TRPA1/

© 2024 The Authors. Molecular Oncology is published by John Wiley & Sons Ltd on behalf of Federation of European Biochemical Societies oxidative stress that is observed in different mouse models of cancer or neuropathic pain. However, this conclusion does not implicate that different proalgesic pathways contribute to MBCP. Glial cell-selective targeting of the various mediators that sustain proalgesic signals initiated by Schwann cell IGF-1R activation could be a promising area for future treatments against MBCP.

### EACR2024-0104
### Ribosome Inactivating Proteins: The potent modulator of tumor suppressors and oncogenes in breast cancer cells

*A. Pervaiz[1], K. Umer[1], S. Saeed[1]*
*[1]University of Health Sciences, Institute of Biomedical and Allied Health Sciences, Lahore, Pakistan*

**Introduction**
Ribosomal inactivating plant proteins (RIPs) target the translational machinery of target cells, interfere with the protein synthesis and induce cytotoxic and cytostatic effects. Riproximin is a recently identified class II RIP and was extracted/ purified from a plant "*Ximenia Americana*". The protein has demonstrated substantial anti-neoplastic effects against cancers during in vitro and in vivo investigations. Most importantly, the protein has shown negligible cytotoxic effects against normal cells during pre-clinical investigations. In present study, we have investigated the impact of riproximin exposure on expressional modulations of 84-tumour suppressors and oncogenes in breast cancer cells.
**Material and Methods**
Human breast cancer cell lines (MDA-MB-231: triple negative, MCF-7: receptor positive) were cultured at standard conditions and exposed to already determined $IC_{50}$ concentration of riproximin for 48h. Following exposure period, total RNA was extracted and cDNA were synthesized by using commercially available kits. Expressional modulations in the 84-tumour suppressor and oncogenes were determined by using a real-time PCR based ready-made panel (Qiagen, PAHS-502Z). Fold changes were determined by Livak method and the data sets were compared with untreated control cells grown in parallel.
**Results and Discussions**
Among the 84-tumour suppressor and oncogenes, comparable de-regulations (≥2fold) were observed in the two breast cancer cell lines (MDA-MB-231: 45/84, MCF-7: 49/84). More specifically, a total of 70/84 (83%) genes of the panel were up-regulated, while 14/84 (17%) were down regulated in MDA-MB-231 cells. In similar pattern, 59/84 (70%) genes of the panel were up-regulated, while 25/84 (30%) were down regulated in MCF-7 cells. When considered substantial de-regulation (≥10fold), FOS, RASSF1 and KITLG were most de-regulated genes in MDA-MB-231 cells, while WT1, TNF and FOS were most altered genes in MCF-7 cells.
**Conclusion**
Riproximin showed a remarkable potential to de-regulate multiple tumor suppressors and oncogenes in breast cancer cells. However, based on molecular features and other cell specific features, these alterations were distinct in the two breast cancer cell lines. Further investigations are warrant to understand the mechanistic reasoning behind the imposed de-regulations and their subsequent clinical utilization in breast cancer regime.

### EACR2024-0133
### Nanodevices targeting cancer-associated fibroblasts to modulate the tumor microenvironment: a tool for triple-negative breast cancer treatment

*A. Lameirinhas[1,2], P. Díez[2,3,4], F.J. Hicke[2,3,4], A. Ágreda-Roca[1], S. Torres-Ruiz[1], A. Lluch[1,5,6,7], J.M. Cejalvo[1,6,7], P. Eroles[1,5,6], I. Garrido-Cano[1,2,4], R. Martínez-Máñez[2,3,4,8]*
*[1]Biomedical Research Institute INCLIVA, Breast Cancer Biology Research Group, Valencia, Spain*
*[2]Universitat Politècnica de València, Instituto Inter-universitario de Investigación de Reconocimiento Molecular y Desarrollo Tecnológico IDM, Valencia, Spain*
*[3]Universitat Politècnica de València, Unidad Mixta UPV-CIPF de Investigación en Mecanismos de Enfermedades y Nanomedicina, Valencia, Spain*
*[4]CIBER-BBN, CIBER de Bioingeniería- Biomateriales y Nanomedicina, Madrid, Spain*
*[5]University of Valencia, Department of Physiology, Valencia, Spain*
*[6]CIBERONC, Centro de Investigación Biomédica en Red de Cáncer, Valencia, Spain*
*[7]Hospital Clínico Universitario de Valencia, Clinical Oncology Department, Valencia, Spain*
*[8]Universitat Politècnica de València- Instituto de Investigación Sanitaria La Fe, Unidad Mixta de Investigación en Nanomedicina y Sensores, Valencia, Spain*

**Introduction**
Most of the BC therapies currently used in clinical practice are focused on targeting tumor cells. However, the tumor microenvironment (TME), specifically the cancer-associated fibroblasts (CAFs), plays an important role in tumor progression, tumor immunity modulation, and therapy resistance. Hence, we design a nanodevice to target CAFs and improve BC treatments.
**Material and Methods**
A nanoparticle was synthesized using mesoporous silica nanoparticles as support, loaded with doxorubicin, and functionalized with an FAP-α ligand peptide (NP-FAP-DOX). NP-FAP-DOX's characterization and cargo release studies were performed. NP-FAP-DOX's targeting efficacy, cellular cytotoxicity, and tumor penetration were evaluated in BC cell lines, CAFs derived from triple-negative BC patient biopsies, and BC patient-derived organoids. The in vivo efficacy was evaluated in a murine triple-negative BC model.
**Results and Discussions**
The NP-FAP-DOX showed controlled cargo release. The nanoparticles efficiently targeted and produced cytotoxic effects in FAP-α positive cells. Moreover, the MSNs probed to be able to penetrate BC 3D models, maintaining the targeting effect. Additionally, the preliminary in vivo studies with a murine model of BC demonstrated promising results in tumor growth inhibition while promoting tumor lymphocyte infiltration and decreasing the M2-like macrophages.

© 2024 The Authors. Molecular Oncology is published by John Wiley & Sons Ltd on behalf of Federation of European Biochemical Societies

**Conclusion**
Overall, these results demonstrated the potential of the designed nanodevices as a new targeted drug delivery system for BC treatment. These nanodevices can improve drug delivery efficacy, overcome adverse side effects, and enhance therapy efficacy through modulation of TME.

### EACR2024-0142
### Potential biomarkers for oral squamous cell carcinoma identified in blood plasma using quantitative lipidomics

*A. Leopoldino[1], J.S.F. Alves[1], G. Silva[1], J. Potenza[1], R. Lacchini[2], L.L. Matos[3], N.P. Lopes[4]*
*[1]University of São Paulo/Pharmaceutical Sciences of Ribeirão Preto, Clinical Analyses- Toxicology and Food Sciences, Ribeirao Preto, Brazil*
*[2]University of São Paulo/Ribeirão Preto College of Nursing, Psychiatric Nursing and Human Sciences, Ribeirão Preto, Brazil*
*[3]Instituto do Câncer do Estado de São Paulo- Hospital das Clínicas- Faculdade de Medicina da Universidade de São Paulo ICESP HCFMUSP, Head and Neck Surgery Department, Ribeirao Preto, Brazil*
*[4]University of São Paulo/Pharmaceutical Sciences of Ribeirão Preto, Biomolecular Sciences, Ribeirao Preto, Brazil*

**Introduction**
Oral squamous cell carcinoma (OSCC) is typically detected at advanced stages through histopathology. Certain research studies have suggested that the metabolism of sphingolipids (SL) is changed in oral squamous cell carcinoma (OSCC). Our study aimed to discover OSCC sphingolipid biomarkers in blood plasma using targeted lipidomics based on liquid chromatography coupled to triple quadrupole mass spectrometry (LC-QqQ).
**Material and Methods**
The Ethical Committees in Human Research of the University of São Paulo have authorized the study. Subsequently, all patients with OSCC ($n$ = 50) and healthy volunteers ($n$ = 30; control group) who agreed to participate signed a free and informed consent and donated blood samples. The clinic-pathological and sociodemographic data were obtained. Concurrently, the development and validation of the LC-QqQ method followed the Food and Drug Administration bioanalytical validation guidelines. Quantitative analysis of 28 sphingolipid species in blood plasma was performed on both OSCC patients and control groups. A commercial mixture of SL and an internal control were included. Univariate and multivariate statistical analyses were performed using SL and clinicopathological data by RStudio software (v1.3.959).
**Results and Discussions**
Targeted lipidomic of blood plasma showed that the levels of thirteen species of sphingolipids were positively regulated in OSCC ($p< 0.05$) compared to the control group. Moreover, ten SL species were associated with tumor size ($p< 0.05$), four SL with intratumoral infiltrate ($p< 0.05$), two SL with angiolymphatic invasion ($p< 0.05$), and one SL with lymph node metastasis ($p< 0.05$). The sphingolipids altered in the plasma of OSCC patients belong to the subclasses of ceramide, glycosylceramide, lactosylceramide, dihydroceramide, sphingomyelin, dihydrosphingomyelin, and sphingosine-1-phosphate. Our findings provide new clinical associations on 28 plasma sphingolipid species in patients with oral squamous cell carcinoma (OSCC), revealing positive regulation of sphingolipid species in advanced stages of OSCC.
**Conclusion**
We propose using the SL species for a more accurate diagnosis of OSCC. We also validate a targeted lipidomics strategy for rapid diagnosis and prognosis using liquid biopsies.

### EACR2024-0164
### Targeting alpha-synuclein to improve melanoma treatments

*A. Mohan[1,2], O.J. Russell[1,2], N. Coles[1,2], C. Tarzi[3,4], V.P. Lenis[1,2], C. Jennings[2], C. Angione[2,3,4], T.F. Outeiro[5,6,7,8], A.A. Khundakar[1,2,5], P. Filippou[1,2]*
*[1]Teesside University, School of Health & Life Sciences, Middlesbrough, United Kingdom*
*[2]Teesside University, National Horizons Centre, Darlington, United Kingdom*
*[3]Teesside University, School of Computing Engineering & Digital Technologies, Middlesbrough, United Kingdom*
*[4]Teesside University, Centre for Digital Innovation, Middlesbrough, United Kingdom*
*[5]Newcastle University, Translational and Clinical Research Institute- Faculty of Medical Sciences, Newcastle upon Tyne, United Kingdom*
*[6]Max Planck Institute, for Multidisciplinary Sciences, Göttingen, Germany*
*[7]Scientific employee with honorary contract, Deutsches Zentrum für Neurodegenerative Erkrankungen, Göttingen, Germany*
*[8]University Medical Center, Department of Experimental Neurodegeneration- Center for Biostructural Imaging of Neurodegeneration, Göttingen, Germany*

**Introduction**
Recent evidence has suggested an overlap in the molecular mechanisms involved in melanoma and Parkinson’s disease (PD), including those associated with protein aggregation. In PD, the protein alpha-synuclein (α-syn) aggregates in patient's brains and, strikingly, the levels of α-syn are significantly increased in melanoma. Chemotherapy's effectiveness for metastatic melanoma may be limited due to chemoresistance. Therefore, it is important to consider alternative mechanisms and targets to improve prognosis and therapeutic interventions.
**Material and Methods**
Melanoma cellular models (A375 and Malme-3M) were cultured as monolayers and spheroids. α-syn expression levels were measured via RT-qPCR and ELISA. Immunocytochemistry (ICC) confirmed the presence of aggregated α-syn in Malme-3M cells. Immunocapture assays, followed by proteomic analysis and western blotting, were employed to identify potent variants of aggregated and/or phosphorylated α-syn. Dacarbazine (DTIC), a chemotherapeutic drug and thymoquinone (TMQ), a natural compound, were used for drug treatments. MTT assays and molecular docking assessed

Molecular Oncology 18 (Suppl. 1) (2024) 1–495
© 2024 The Authors. Molecular Oncology is published by John Wiley & Sons Ltd on behalf of Federation of European Biochemical Societies cytotoxicity and binding affinities respectively. The impact of treatments on α-syn was assessed using RT-qPCR and ELISA.

**Results and Discussions**

Malme-3M melanoma metastatic cells exhibited higher levels of α-syn compared to A375 malignant cells. ICC confirmed aggregated α-syn in Malme-3M cells, consistent with observations in spheroid secretome and our previous findings in metastatic melanoma lymph nodes. Variations in α-syn post-translational modifications, particularly phosphorylation (e.g. pS129), were noted in pathological α-syn aggregates. To assess the levels of α-syn upon drug treatments, TMQ, an emerging anticancer natural compound was chosen due to its minimal toxicity towards normal cells, along with DTIC. TMQ and DTIC displayed varying cytotoxic responses in both cellular models, with more pronounced effects in non-metastatic melanoma models, implying differential modulation of α-syn levels. Molecular docking revealed differential binding of α-syn to each drug. Mechanisms underlying α-syn involvement in drug response may differ between TMQ and DTIC treatments.

**Conclusion**

Elevated α-syn expression and aggregation in metastatic melanoma cells, along with differential cytotoxicity responses to TMQ and DTIC, suggests a potential role for α-syn in influencing therapeutic outcomes. These findings highlight the need for deeper understanding of α-syn mediated mechanisms in melanoma and treatment response.

### EACR2024-0195

### The synergistic anti-tumor activity of DN200434 (ERRγ inverse agonist) and MK2206 (Akt inhibitor) in anaplastic thyroid cancer cells and radioiodine refractory papillary thyroid cancer cells

*D. Gulwani[1], T.D. Singh[1]*

*[1]All India Institute of Medical Sciences, Medical Oncology, New Delhi, India*

**Introduction**

Estrogen Related Receptors (ERRs) were the first orphan nuclear receptors identified based on sequence similarity with the classical estrogen receptors. Numerous unbiased investigations have connected ERRγ to the initiation and advancement of various cancers, including thyroid carcinoma. Furthermore, ERRγ is known to play a distinct role in modulating effectors of metabolism, cell cycle progression, and apoptosis in malignancies of various origins. We evaluated the expression levels of Estrogen Related Receptor Gamma (ERRγ) in various thyroid cancer cell lines to assess its potential as a therapeutic target. Additionally, we tested the anti-tumor effect of DN200434 (inverse agonist of ERRγ) in combination with MK2206 (Akt inhibitor)

**Material and Methods**

ATC cells (HTh7, 8505c) and PTC cells (KTC-1, TPC-1) were cultivated in their corresponding mediums. A CCK-assay was used to measure the IC50 values of DN200434 and MK2206 in ATC/PTC cells after 24 hours. Cells were treated with drugs alone or in combination for 24 hours to assess their inhibitory effect on viability, apoptosis, and cell cycle using flow cytometry. Additionally, western blot was used to enumerate the protein levels implicated in the cell cycle and apoptotic pathways. Following combination treatment, the colony formation assay was carried out in six-well plates with 500 cells per well. Crystal violet staining was followed by a count of large colonies with up to 50 cells

**Results and Discussions**

We observed that the IC50 values of DN200434 and MK2206 were 30-45 µM and 10-20 µM in ATC/PTC cells at 24 h, respectively. The combination of DN200434 and MK2206 in ATC/PTC cells synergistically enhanced cell cytotoxicity up to 70-75% as compared to DN200434 (5-15%) and MK2206 (10-20%) alone. Flow cytometry results showed significant apoptosis and cell cycle arrest at G0/G1 phase in the combination drug-treated cells, respectively. Western blot data confirmed significant apoptosis in combination treated cells with an increased level of cleaved caspase-3 and cleaved-PARP. A significant reduction (70-80%) in the colony forming ability was observed in combination treated cells

**Conclusion**

Our results demonstrate that the combination of DN200434 and MK2206 can be used as a therapeutic approach for managing anaplastic and radioiodine refractory papillary thyroid cancer. Further evaluation of combination therapy in animal models or 3D spheroid models is required before clinical trials of this potent combination

### EACR2024-0197

### Differential Immunomodulatory effects of Anthracyclines and taxanes in breast cancer patients

*R. Oehler[1], K. Wimmer[1], M. Sachet[1], C. Ramos[1], C. Brostjan[1], Z. Bago-Horvath[2], R. Bartsch[3], M. Filipits[4], M. Gnant[5], C.F. Singer[5]*

*[1]Medical University of Vienna, Department of General Surgery, Vienna, Austria*

*[2]Medical University of Vienna, Department of Pathology, Vienna, Austria*

*[3]Medical University of Vienna, Department of Medicine 1, Vienna, Austria*

*[4]Austrian Breast & Colorectal Cancer Study Group ABC SG, Medical University of Vienna- Center for Cancer Research, Vienna, Austria*

*[5]Austrian Breast & Colorectal Cancer Study Group ABC SG, Medical University of Vienna- Comprehensive Cancer Center, Vienna, Austria*

**Introduction**

Anthracyclines and taxanes are chemotherapeutic agents commonly used in a sequential regimen in the neoadjuvant treatment of early, high-risk or locally advanced breast cancer (BC). Novel approaches to increase the response rate combine this treatment with immunotherapies such as PD-1 inhibition. However, the expected stimulatory effect of on lymphocytes may depend on the chemotherapy backbone. Therefore, we separately compared the immunomodulatory effects of the anthracycline epirubicin and the taxane docetaxel in the setting of a randomized clinical trial.

**Material and Methods**

© 2024The Authors. Molecular Oncology is published by John Wiley & Sons Ltd on behalf of Federation of European Biochemical Societies

Tumour and blood samples of 154 patients from the ABCSG-34 trial were available (76 patients received four cycles of epirubicin/cyclophosphamide (EC) followed by four cycles of docetaxel (D); 78 patients get the reverse treatment sequence). Tumour-infiltrating lymphocytes, circulating lymphocytes and 14 soluble immune mediators were determined at baseline and at drug change. Furthermore, three BC cell lines were treated with E or D and co-cultured with immune cells.

**Results and Discussions**

Initial treatment with four cycles of EC reduced circulating B and T cells by 94% and 45%, respectively. In contrast, no comparable effects on lymphocytes were observed in patients treated with initial four cycles of D. Most immune mediators decreased under EC whereas D-treatment resulted in elevated levels of CXCL10, urokinase-type plasminogen activator (uPA) and its soluble receptor (suPAR). Accordingly, only the exposure of BC cell lines to D induced similar increases. While treatment of BC cells with D was associated with a necrotic cell death, E induced apoptosis.

**Conclusion**

The deleterious effect of epirubicin on lymphocytes indicate strong immunosuppressive properties of this chemotherapeutic agent. Docetaxel, in contrast, has no effect on lymphocytes, but triggers the secretion of stimulatory proteins in vivo and in vitro, indicating a supportive effect on the immune system. Underlying differences in the induced cell death might be causal. The divergent immunomodulatory effects of anthracyclines and taxanes should be considered when planning future combinations with immunotherapies in breast cancer

### EACR2024-0274

### The Difference of Transcriptomic Insights between Prurigo Nodularis, Atopic Prurigo Nodularis, and Atopic Dermatitis: Unraveling Neuronal Profiles and Immune Response

*J. Jang[1], Y.B. Lee[2], H.S. Park[1], N. Hong[3], H.D. Kook[3], J.I. Kim[1], J. Ahn[3]*

*[1]Seoul National University, Department of Biomedical Science, Seoul, South Korea*

*[2]Catholic University of Korea, Department of Dermatology, Gyeonggi-Do, South Korea*

*[3]National Medical Center, Department of Dermatology, Seoul, South Korea*

**Introduction**

Prurigo nodularis (PN) is a disease characterized by chronic itch with firm nodules like papules or nodules. The underlying pathophysiology of PN is still controversy, but it has been reported to be due to synergistic neural- and immune mediated mechanisms. The relationship between various inflammatory skin diseases and prurigo nodularis is widely known, and atopic dermatitis appears with the highest frequency. The prurigo nodularis accompanying atopic dermatitis has recently been called atopic prurigo nodularis (atopic PN). Our study aimed to compare this condition with typical atopic dermatitis (AD) and prurigo nodularis without atopic manifestations at the RNA transcriptomic level to uncover potential molecular distinctions and shared pathways.

**Material and Methods**

Skin punch biopsies of 4mm lesion sites were conducted on a cohort consisting of 27 patients and 4 healthy controls. Among the patients, 13 were diagnosed AD, 7 with PN, 7 with APN. The samples were preserved at -20°C using RNA*later*® solution and subsequently extracted using Trizol®. 3' mRNA sequencing method was utilized and sequenced using Hiseq2500. The fastq files underwent UMI extraction, trimming, alignment, and counting processes to be converted into count matrix. Differential gene expression analysis was then conducted using DESeq2 to identify differentially expressed genes (DEG).

**Results and Discussions**

In the comparative analysis between APN and PN against AD, both conditions consistently exhibited the proenkephalin (PENK) gene as a DEG. Moreover, APN and PN demonstrated an enhanced expression of transcripts related to neuronal activities such as neuropeptide expression, neuron proliferation, differentiation, migration and growth when compared to AD. APN showed upregulation of pathways related to hypoxia and epithelial-mesenchymal transition (EMT), which appear to be involved in dermal fibrosis, both of which were also noticeably upregulated in PN compared to AD. Immunologically, AD, APN and PN all exhibited higher type 1 and 2 immune gene signature scores compared to normal controls, with the differences among them being non-significant.

**Conclusion**

Our study elucidates that APN and PN manifest unique neuronal signatures and shared inflammatory responses compared to AD, underscoring distinct yet overlapping pathogenic pathways. In addition, it was confirmed that type 1 and 2 inflammation is increased in both PN and APN, so they can be targets of the same immunological treatment regardless of PN's accompanying diseases.

### EACR2024-0293

### Hypoxic and Angiogenic response to repeated modulated electrohyperthermia in murine Triple negative breast cancer model

*S.M.Z. Bokhari[1], K. Aloss[1], P.H.L. Viana[2], C.A. Schvarcz[3], B. Besztercei[1], N. Giunashvili[1], D. Bócsi[1], Z. Koós[1], Z. Benyó[1], P. Hamar[1]*

*[1]Semmelweis University, Institute of Translational Medicine, Budapest, Hungary*

*[2]Semmwleis University, Institute of Translational Medicine, Budapest, Hungary*

*[3]Semmelwies university, Institute of Translational Medicine, Budapest, Hungary*

**Introduction**

Modulated electro-hyperthermia (mEHT) is a novel complementary therapy where a 13.56 MHz radiofrequency current targets cancer cells selectively, inducing tumor damage by thermal and electromagnetic effects. We observed vascular damage in mEHT-treated tumors and investigated the potential synergism between

Molecular Oncology 18 (Suppl. 1) (2024) 1–495

© 2024 The Authors. Molecular Oncology is published by John Wiley & Sons Ltd on behalf of Federation of European Biochemical Societies mEHT and inhibition of vasculature recovery in our triple negative breast cancer (TNBC) model.

**Material and Methods**

4T1/4T07 isografts were treated three to five times with mEHT. Digoxin was administered daily by intraperitoneal injection to mice in the combination therapy group. Tumor growth was monitored with ultrasound and digital calipers. Tumor destruction histology, blood capillary damage, and molecular changes were detected using immunohistochemistry, flow cytometry, qPCR and western blot.

**Results and Discussions**

mEHT induced vascular damage four to twelve hours after treatment leading to tissue hypoxia detected at twenty-four hours. Hypoxia in treated tumors induced an angiogenic recovery twenty-four hours after the last treatment. Administration of the cardiac glycoside digoxin could synergistically augment mEHT-mediated tumor damage and could reduce tissue hypoxia signaling and consequent vascular recovery in mEHT-treated tumors.

**Conclusion**

Repeated mEHT induced vascular damage and hypoxic stress in TNBC which promoted vascular recovery. Inhibiting this hypoxic stress signaling enhanced the effectiveness of mEHT and may potentially enhance other forms of cancer treatment.

### EACR2024-0302

### PEP-010, a first-in-class clinical-stage pro-apoptotic peptide is efficient in vitro and in ovo on pancreatic cancer in monotherapy and in combination with different chemotherapies

*A. Lacroix[1], R. Farhat[1], L. Lhoste[1], L. Dadon[1], J. Wiels[2], C. Brenner[2], D. Germini[1]*

*[1]PEP-Therapy, Research and Development, Paris, France*

*[2]CNRS- Institut Gustave Roussy- Université Paris-Saclay, Umr9018- Metabolic and systemic aspects of oncogenesis for new therapeutic approaches, Villejuif, France*

**Introduction**

Pancreatic cancer (PC) poses a significant threat as it is often diagnosed late due to nonspecific symptoms and as a limited number of patients are eligible for surgical resection. The challenge in achieving therapeutic success in PC can be attributed to the malignant pancreatic cells' capability to evade apoptosis through diverse mechanisms and to acquire mutations. PEP-010 is a first-in-class pro-apoptotic peptide in clinical stage, developed by PEP-Therapy, a French clinical-stage biotech company. PEP-010 is a cell-penetrating and interfering peptide designed to specifically target the intracellular PP2A/caspase-9 interaction. This interaction prevents these two proteins from playing their role in apoptosis, thus PEP-010 acts by disrupting this interaction, releasing PP2A and caspase-9, restoring apoptosis in cancer cells. To support the design of the Phase Ib we aimed to show PEP-010 efficacy in inducing apoptosis in PC models both in monotherapy and in combination with gemcitabine, a Standard-of-Care for pancreatic cancer, or with paclitaxel.

**Material and Methods**

In cell lines, MTT assay or Annexin-V/Propidium Iodide staining and flow cytometry analysis were used to assess apoptosis after treatment with PEP-010 in monotherapy or in combination with paclitaxel or gemcitabine. In chicken eggs xenografted with PC cells, PEP-010 efficacy in monotherapy or in combination with gemcitabine was assessed by tumor weight after treatment. Cleaved caspase-3 expression was evaluated using immunofluorescence and fluorescent microscopy.

**Results and Discussions**

We demonstrated that PEP-010 penetrates cells rapidly and induces apoptosis in PC models both in vitro and in ovo. PEP-010 activity can be monitored by the expression of the cleaved caspase-3 which could be used as a pharmacodynamic biomarker of PEP-010. We also demonstrated through both in vitro and in ovo experiments that combining PEP-010 with standard chemotherapies such as gemcitabine results in an additive effect significantly superior to individual effects of the monotherapies. We have also observed in vitro a beneficial effect of using PEP-010 in combination with paclitaxel.

**Conclusion**

These preclinical data together with observed preliminary signal of antitumoral activity in one PC patient in combination with paclitaxel during the clinical trial dose-escalation phase (Phase Ia), support the clinical trial strategy and highlights the therapeutic interest of PEP-010 for this hard-to-treat disease.

### EACR2024-0311

### canSERV – providing cutting edge cancer research services across Europe

*J. Balogh[1], M. Pausan[2], J. Eriksson[3], S. Scollen[4], V.M. dos Santos[5], E. Medico[6], B. Stechmann[7], M. Hagn[8], C. Brockhaus[9], J.K. Habermann[10]*

*[1]BBMRI-ERIC, Finance and Project Management, Graz, Austria*

*[2]BBMRI-ERIC, Biobanking Development, Graz, Austria*

*[3]EURO-Bioimaging, Euro-BioImaging Statutory Seat, Turku, Finland*

*[4]ELIXIR/EMBL, Human Genomics and Translational Data, Hinxton, United Kingdom*

*[5]Wageningen University representing IBISBA, Bio Process Engineering, Wageningen, The Netherlands*

*[6]Universita degli Studi di Torino representing EuroPDX, Oncogenomics, Turin, Italy*

*[7]EU-OPENSCREEN, Operations & Scientific Strategy, Berlin, Germany*

*[8]ARTTIC, Consultant Department, Munich, Germany*

*[9]Instruct ERIC, Instruct Hub, Oxford, United Kingdom*

*[10]BBMRI-ERIC, Director General, Graz, Austria*

**Introduction**

canSERV is a € 15 Mio. project offering cutting-edge research services, enabling innovative R&D projects and fostering precision medicine for patients benefit. canSERV involves 18 leading organizations across Europe including Research Infrastructures, key organisations and oncology experts.

**Material and Methods**

© 2024 The Authors. Molecular Oncology is published by John Wiley & Sons Ltd on behalf of Federation of European Biochemical Societies canSERV's main objectives are: (i) offer at least 200 different unique Personalised Oncology relevant and valuable cutting-edge services; (ii) establish a single, unified, transnational access platform to request services and trainings; (iii) ensure oncology-related data provided will be fully compliant with FAIR principles and complement and synergise with other EU initiatives and (iv) ensure long-term sustainability beyond project duration. Furthermore, canSERV establishes the European Molecular Tumor Board Network (EMTBN) that is open for anyone to join. The EMTBN develops Molecular Tumor Board (MTB) consensus guidelines, an MTB outcome registry, and provides advice to scientists, clinicians, and MTBs.

**Results and Discussions**

canSERV offers a series of open and challenge calls for access to services in the amount of ~ € 9 Mio. The calls are designed to support researchers to develop innovative research projects that explore cutting-edge methodologies and target critical gaps in cancer research and care by providing funding to resources/services. By encouraging the submission of collaborative proposals, canSERV aims to foster transnational cooperation, support a vibrant scientific community, and help to accelerate knowledge gain and transfer through defragmenting the European Research Area.

**Conclusion**

canSERV presents an unparalleled opportunity to accelerate cancer research, drive innovation, and improve patient outcomes.

*canSERV is granted by the EU Horizon programme under #101058620. The abstract is submitted on behalf of the canSERV Consortium.*

### EACR2024-0322
### Introducing Sample Re-Interrogation for Progressive Development and On-line Analysis of High Parameter Spatial Biology Data

*O. Braubach[1], M. Ingalls[2], C. Jackson[2], C. Kayla[2], A. Christians[3], A. Brix[3], J.S. Schwarz[2]*

*[1]Canopy Biosciences- A Bruker Company, Research and Development, St Louis, United States*

*[2]Canopy Biosciences- A Bruker Company, Research and Development, St. Louis, United States*

*[3]Canopy Biosciences- A Bruker Company, Research and Development, Hannover, Germany*

**Introduction**

Utilizing spatial biology in cancer research, we can understand the tumor microenvironment, unravel responsiveness to drug treatments and cell therapies, and ultimately stratify patients for targeted treatment and clinical trial enrollment. Establishing spatial biology workflows can be a resource intensive exercise and may require a commitment of high-value patient samples. Further, the design of detection reagent panels used to study samples is not trivial and may require optimization for individual specimens. Here we present a novel solution that permits sample re-interrogation and real-time adjustments of reagent panels in our spatial biology workflow

**Material and Methods**

The CellScape™ platform for Precise Spatial Multiplexing paired with VistaPlex™ Assay Kits provide a ready-to-use cyclic multiplex immunofluorescence (IF) solution to interrogate fresh frozen and FFPE tissue samples. Tissues are mounted in enclosed fluidic chambers, while staining and imaging are conducted on the CellScape instrument. The experiment requires no additional autostainer equipment and is divided by rounds of staining and imaging. The user may therefore pause between rounds and conduct inter-round analyses, which can then guide decisions of whether and how to continue the experiment.

**Results and Discussions**

To illustrate the benefit of this approach, we present a use case in which we applied VistaPlex antibody panels to reveal the spatial distribution of major immune cell populations. Inter-round analysis and conclusions were then drawn from this preliminary phenotype map before successive rounds of rationally planned staining were conducted to further deepen the depth and breadth of spatial phenotyping.

**Conclusion**

The CellScape sample re-interrogation feature is a pragmatic spatial biology solution that optimizes the use of precious resources while allowing researchers to generate informed decisions about antibody panel designs in real time. This enhanced utility may reduce the resistance to spatial biology among researchers working with samples that are limited or irreplaceable.

### EACR2024-0327
### The Effect of Treatment on Bladder Cancer Ex Vivo Tissue Slices Through Single-Cell and Spatial Analysis

*M. Radic[1], F. La Manna[1], M. Minoli[1], P. Chouvardas[1], G. N. Thalman[2], R. Seiler-Blarer[3], B. Kiss[2], M. Kruithof-de Julio[1]*

*[1]University of Bern, Department for BioMedical Research, Bern, Switzerland*

*[2]Inselspital- Bern University Hospital, Department of Urology, Bern, Switzerland*

*[3]Spitalzentrum Biel- Hospital Center Biel, Department of Urology, Biel, Switzerland*

**Introduction**

Bladder cancer (BLCa) remains a prevalent malignancy with a relatively poor outcome that has shown minimal improvement in recent decades. Phenotyping the residual disease holds promise in detecting drug-resistant populations, thus facilitating the selection of tailored treatments, and significantly improving the long-term prognosis for BLCa patients.

**Material and Methods**

We utilized an ex vivo tissue slices platform that offers a faithful representation of intratumoral complexity to evaluate standard-of-care (SOC) chemotherapies alongside non-SOC targeted therapy agents. We validated the expression of proliferative and epithelial markers through immunohistochemistry staining and identified distinctive cell clusters in treated and untreated samples using flow cytometry (FCM). Furthermore, we conducted spatial transcriptomics using the 10x Genomics Visium CytAssist to evaluate the impact of treatment on tissue spatial diversity.

**Results and Discussions**

© 2024 The Authors. Molecular Oncology is published by John Wiley & Sons Ltd on behalf of Federation of European Biochemical Societies

A panel of 17 FCM markers enabled us to identify four cell clusters (epithelial, immune, endothelial, and stromal), as well as to classify tumor subtypes (basal and luminal), assess immune infiltration, and recognize hot-target expression. In our initial assessment, we validated the FCM panel on 10 BLCa samples, where we identified connections between the expression of specific markers and various clinical and pathological parameters, such as metastatic presence and heightened immune and stromal infiltration. The application of the FCM panel to ex vivo tissue treated with chemotherapy and targeted therapy agents from six patients demonstrated a shift in cluster expression post-treatment, particularly noticeable with chemotherapy. Notably, treatment with erdafitinib or lapatinib in targeted therapy decreased the proportion of epithelial cells expressing the pharmacological targets FGFR3 and EGFR. This treatment also changed the basal/luminal cell ratio, characterized by increased CD44, CD90, and CD239 markers. By merging single-cell expression patterns from FCM with spatial transcriptomics in a specific patient, we generated a comprehensive molecular map of cellular subtypes, unveiling dynamic alterations in the tumor microenvironment following treatment.

**Conclusion**

Our results emphasize the efficacy of FCM in identifying specific cellular clusters that were upregulated post-treatment, especially after chemotherapy. This underscores the translational potential for precise targeting of residual disease cell clusters after treatment.

### EACR2024-0354

### First-in-class clinical candidate ONC201 induces integrated stress response in cervical cancer cell lines that culminates via distinct phenotypic responses

<u>S. Manohar</u>[1], S. Pathak[2]

*[1]Sunandan Divatia School of Science- SVKM's NMIMS Deemed-to-be University, Biological Sciences, Mumbai, India*

*[2]Sunandan Divatia School of Science- SVKM's NMIMS Deemed-to-be University, Biological Sciences, Mumbau, India*

**Introduction**

Cervical cancer (CC) is a major public health problem in developing countries including India. Carcinogenesis of cervical epithelial cells is induced by infection with specific subtypes of Human Papilloma Virus (HPV) in almost all cases. Standard chemotherapy is the only treatment option for advanced stage disease. However, its efficacy is limited due to acquired resistance and systemic toxicity. ONC201 (also known as TIC10) is a first-in-class small molecule currently in Phase II/III clinical trials for various cancers. It has been shown to inhibit cell proliferation via induction of integrated stress response (ISR) in different types of cancers. Yet, there is no report on the potency of this versatile drug against cervical cancer. In the present study, we sought to test the in vitro efficacy and mechanism of action of ONC201 in HPV-positive CC cells.

**Material and Methods**

In vitro cytotoxicity of ONC201 was evaluated in HPV-positive cervical cancer cell lines viz. HeLa and SiHa by MTT assay. Further, its effect on clonogenic potential and cell cycle was tested using colony forming assay and flow cytometry, respectively. Apoptosis studies were performed by annexin-V/PI staining using flow cytometry. Gene and protein expression analysis was carried out for apoptotic and ISR markers by qRT-PCR and western blotting respectively.

**Results and Discussions**

ONC201 exerted significant cytotoxicity and inhibited clonogenic potential of both CC cell lines HeLa and SiHa. Although it induced integrated stress response as evidenced by increase in ATF4 and mitochondrial casinolytic protease (cIpP) levels in both the cell lines, it induced apoptosis in HeLa whilst it exerted only a cytostatic effect i.e. G2/M arrest in SiHa cells. Typical markers of extrinsic apoptosis were observed in ONC201-treated HeLa cells such as cleaved caspsase-8 and increased TRAIL and DR5 expression but not in SiHa. Cell cycle arrest and apoptosis were independent of p53.

**Conclusion**

This study provides a proof-of-concept for anticancer activity of versatile drug ONC201. It also highlights distinct phenotypic responses i.e. apoptosis versus growth arrest of two CC cell lines to ONC201-induced ISR. Future studies warrant delving deeper so as to understand what dictates such cell-line-dependent response of HPV-positive cervical cancer cells to ONC201.

### EACR2024-0373

### Influencing drug responses through in vitro tumor microenvironment characteristics

<u>H. Lanz</u>[1], M. Geyer[1], B. Kramer[1], J. Joore[1], K. Queiroz[1]

*[1]Mimetas, Model Development, Oegstgeest, The Netherlands*

**Introduction**

Pancreatic Ductal Adenocarcinoma (PDAC), the most common pancreatic cancer type, is projected to become become the second leading cause of cancer-related deaths by 2030. Mortality rates can reach up to 93%. Current standard-of-care for patients with PDAC includes chemotherapeutic regimens and pancreatic cancer surgery. Unfortunately, only 20% of the patients are eligible for surgery due to late diagnosis. Despite being te primary treatment, chemotherapeutic regimens have limitations, including the development of treatment resistance. PDAC tumors have a unique tumor micro-environment (TME) characterized by high-density stroma and hypovascularization, therefore these present high interstitial pressure and hypoxia. These features potentially interfere with the efficiency of chemo-therapeutic drugs and highlight the urgent need for novel PDAC screening platforms.

**Material and Methods**

Organ-on-a-chip technology has emerged as a promising tool for recreating the TME. In this context, we introduce the OrganoPlate®, a high-throughput 3D cell culture microfluidic platform. This innovative platform allows researchers to explore a wide range of flow and co-culture conditions, including the creation of

© 2024 The Authors. Molecular Oncology is published by John Wiley & Sons Ltd on behalf of Federation of European Biochemical Societies physiologically relevant models with a minimal requirement of cell material. Our goal was to faithfully model crucial phenotypic properties of PDAC by incorporating different aspects of the TME, such as interstitial pressure, hypoxia, vascularisation, and immune recruitment, through different culture set-ups.

**Results and Discussions**

In a PDAC TME model it was shown that interstitial pressure influences cancer cell morphology and causes chemoresistance through changes in gene expression. Hypoxic conditions strongly influenced the effect of chemotherapeutics on PDAC. The addition of the stromal component of the PDAC TME resulted in shielding of the tumor of different sets of immune cells. This opened up the opportunity to search for stromal targeting treatments to improve PDAC destruction.

**Conclusion**

The ability to create TME-on-a-chip models holds the potential for enhanced prediction of how both conventional and targeted therapies will perform. These models allow us to better understand treatment outcomes and optimize therapeutic strategies.

*COI: All authors are or were employees of Mimetas BV, the Netherlands, which is marketing the OrganoPlate. OrganoPlate is a trademark of Mimetas BV.*

### EACR2024-0387

### Utilizing translational platform GlioBank to identify novel molecular signatures of glioblastoma progression

*B. Breznik[1], M. Novak[2], B. Majc[3], M. Malavolta[4], A. Porčnik[5], J. Mlakar[6], M. Hren[7], A. Habič[2], M. Skoblar Vidmar[8], A. Sadikov[4]*

*[1]National Institute of Biology- Slovenia, Department of Genetoc Toxicology and Cancer Biology, Ljubljana, Slovenia*

*[2]National Institute of Biology, Department of Genetic Toxicology and Cancer Biology, Ljubljana, Slovenia*

*[3]National Institute of Biology, Department of Genentic Toxicology and Cancer Biology, Ljubljana, Slovenia*

*[4]University of Ljubljana, Faculty of Computer and Information Science, Ljubljana, Slovenia*

*[5]University Medical Centre Ljubljana, Department of Neurosurgery, Ljubljana, Slovenia*

*[6]University of Ljubljana, Institute of Pathology- Faculty of Medicine, Ljubljana, Slovenia*

*[7]Biosistemika- d.o.o., Biosistemika- d.o.o., Ljubljana, Slovenia*

*[8]Institute of Oncology Ljubljana, Institute of Oncology Ljubljana, Ljubljana, Slovenia*

**Introduction**

Glioblastoma (GBM) is one of the most lethal solid tumors in humans. Currently, GBM is considered incurable and there is a lack of progress in identifying clinically relevant biomarkers and effective therapeutic options. The aim of the study was to establish a translational platform in Slovenia to centrally collect biological material and all relevant clinical and research data and use GlioBank datasets to determine the molecular characteristics of GBM and identify biomarkers associated with poor GBM survival.

**Material and Methods**

Collection of tumor tissue and establishment of cellular models, including primary GBM cell cultures and organoids, were performed according to GlioBank standard operating procedures. Expression analysis of genes associated with GBM stem cells, epithelial-to-mesenchymal transition (EMT), immunosuppression and GBM subtypes was performed in a cohort of 91 patient tissues and 23 primary GBM cells by qPCR, followed by bioinformatic analyzes of available clinical and research data.

**Results and Discussions**

This study demonstrates the establishment of the Slovenian translation platform GlioBank and the demonstration of its utility for research and translation by identifying molecular signatures associated with GBM progression and survival. We found that high expression of *STAT3, S100A4* and *DAB2* in tumor tissue is associated with poor overall survival of GBM patients. In addition, novel differences in the molecular signature between the tumor core vs. rim on 27 patient-matched samples were detected, suggesting that differences between these tumor regions need to be considered in the search for therapeutic modalities targeting infiltrating tumor cells.

**Conclusion**

A centralized GlioBank built on a multidisciplinary network has been established to facilitate basic and clinically relevant research in the field of neuro-oncology. New molecular signatures of GBM have been found that are associated with invading tumor cells in patients as well as GBM survival.

### EACR2024-0404

### 3D cancer matrix-based models and Multi-Organ-on-Chip Platforms to Investigate in vitro tumour cells invasion, survival under fluid flow and infiltration in metastatic target

*S. scaglione[1,2], E. Palama'[3], M. Aiello[2,3]*

*[1]React4life, R&D, Genoa, Italy*

*[2]National Research Council, National Research Council IEIIT, Genoa, Italy*

*[3]React4life, Research, Genoa, Italy*

**Introduction**

Traditionally, human cancer modeling for both basic research and drug testing has heavily leaned on 2D cell cultures in static conditions, as well as xenografts animal models. However, critical questions remain about the metastatic journey of these cells from primary tumor to bone and the often-discrepant drug responses between clinical and preclinical studies.

**Material and Methods**

We employed an alginate-based hydrogel with adjustable stiffness to simulate the tumor microenvironment, facilitating the migration and expansion of MCF7 and MDA-MB-231 breast cancer cells, which vary in aggressiveness. The viability and migration behaviors of the cells embedded in the hydrogels were characterized, along with cytoskeletal dynamics (MTT assay, Dead Alive, Immunofluorescence). A multi-organ-on-chip (OOC) system was used to culture 3D breast cancer tissues and a bone tissue model in two interconnected

© 2024 The Authors. Molecular Oncology is published by John Wiley & Sons Ltd on behalf of Federation of European Biochemical Societies chambers, simulating the metastatic pathway. The bone niche was resembled by suing hydroxyapatite-enhanced polymeric structures. Computational fluid dynamics simulations established the flow velocities and shear stresses within the OOC's fluidic pathways, representing the circulatory transit of tumor cells from breast to bone.
**Results and Discussions**
The multi-organ OOC configuration allowed culture media sampling both in the breast and bone compartment, and also in the circulatory flow, facilitating the quantification of circulating tumor cells. Viability of CTCs was affected by different shear stresses regimes (1-20 dyn/cm2). Human cell-laden alginate hydrogels were successfully cultured for up to 120 days, displaying cytoskeleton reorganization resembling in vivo phenotype, consistent proliferation, and spontaneous migration out the matrix joining the systemic circulation, where their survival was cross-correlated to the shear stress. Interestingly tumor cells intravasation was resembled in vitro as wells and cells extravasation under specific biochemical signaling.
**Conclusion**
Notably, this technological approach enables to capture the key elements of the metastatic process and also to test new therapies addressed to the metastasis inhibition. Finally, due to its compatibility with the culture ex vivo of patient derived breast cancer biopsies, this OOC based approach open new scenario towards the personalized medicine.

### EACR2024-0410
### Ready to use Multiplex Immunofluorescence Panels Provide Reliable Deep Spatial Phenotyping for Diverse Immuno-Oncology Applications

*J. Rauer[1], C.E. Jackson[2], J. Boog[1], A. Christians[1], M.H. Ingalls[2], A. Northcutt[2], K.E. Cashion[2], K. Kwarta[3], J.S. Schwarz[2], O. Braubach[2]*
*[1]Canopy Biosciences- A Bruker Company, Research and Development, Hannover, Germany*
*[2]Canopy Biosciences- A Bruker Company, Research and Development, St. Louis, United States*
*[3]Canopy Biosciences- A Bruker Company, Product Management, St. Louis, United States*
**Introduction**
Multiplex immunofluorescence (mIF) is a powerful tool that enables deep phenotyping of immune and cancer cells across a wide variety of tissue types. The initial effort and cost required to develop high-plex mIF panels is significant and poses a considerable hurdle for adoption of spatial biology. To streamline high-plex assay development, we developed a process for creating and validating multiplex antibody panels for use in immuno-oncology applications that leverage CellScape™ Precise Spatial Multiplexing. Our multiplex assay development was a rigorous, multi-stage, iterative process to evaluate antibodies for suitability, specificity, and reproducibility. These assay panels are formulated as ready-to-use VistaPlex™ Assay Kits and are designed to enable rapid, reliable spatial phenotyping of key immune populations.
**Material and Methods**
To confirm the utility of VistaPlex, we deployed the Human FFPE Spatial Immune Profiling Panel to stain and image various FFPE tissue samples on the automated CellScape platform. With CellScape, tissues are mounted in enclosed fluidic chambers while on-instrument staining, imaging and signal erasure are conducted in iterative rounds. The panel presented here contains antibodies against 15 common biomarkers used in immuno-oncology applications, including CD3, CD4, CD8, CD20, and CD45 to detect cell lineages, PD-1 and PD-L1 to interrogate immune checkpoints, and markers that inform about cell state and activation.
**Results and Discussions**
Spatial immune profiling data were obtained from whole-biopsy human tonsil, colon, breast, and lung tissue samples, as well as multi-tissue TMAs. Deep immune phenotyping was performed using unsupervised clustering analysis and AI-assisted cluster labelling, and biomarker distributions were studied in the context of cellular neighborhoods and other proximity analyses.
**Conclusion**
Our data are robust and demonstrate that VistaPlex Assay kits for spatial profiling present an efficient and cost-effective solution for users that want to break into spatial biology.

### EACR2024-0417
### Brain tumoroids: revolutionizing treatment prediction and drug development with fast, reproducible and easy-to-use personalized models

*A. Tchoghandjian[1], A. Soubéran[1], C. Jiguet-Jiglaire[2], S. Toutain[1], N. Baeza-Kallee[1], R. Appay[2], T. Graillon[3], D. Figarella-Branger[1], E. Tabouret[4]*
*[1]Institute of Neurophysiopathology, Gliomagenesis and microenvironment, Marseille, France*
*[2]assistance Publique Hôpitaux de Marseille, Neuropathologie, Marseille, France*
*[3]Assistance Publique Hôpitaux de Marseille, Neurochirurgie, Marseille, France*
*[4]Assitance publique Hôpitaux de Marseille, Neuro-Oncologie, Marseille, France*
**Introduction**
The modeling of tumors has become an essential and instrumental support for research. Patient avatars, crucial tools in the advancement of personalized medicine and the discovery of novel therapeutic interventions, hold immense promise.
**Material and Methods**
We developed a step-by-step protocol for the culture of viable and standardized tumoroids. Tumor samples are automatically minced, selected, and pieces of tumors were cultured under agitation. We performed multiplexed spatial imaging to characterize tumoroids cellular composition and organization. We used methylome profiling and cytometry to determine the molecular signature and the quantity of immune cells throughout tumoroid culture, in comparison to the parental tumor. Finally, we tested whether tumoroids could reflect patient treatment response by applying a STUPP-like protocol, and were suitable to discovery new treatment by testing combinatory regimens.

© 2024 The Authors. Molecular Oncology is published by John Wiley & Sons Ltd on behalf of Federation of European Biochemical Societies

**Results and Discussions**
This protocol is a cheap, easy, fast, automatized, reproducible and efficient method to generate any type of brain tumoroids. It preserves native cytoarchitecture, cellular and molecular heterogeneity and specificities. Moreover, this model mimics patient's treatment response and is functional as we identified efficient combinatory treatments.
**Conclusion**
This tumoroid model represents a robust model for biological studies and new drug testing. These tumor avatars open perspectives for the establishment of a personalized medicine.

### EACR2024-0420
### High-resolution Analysis of Immune Checkpoint Activation Utilizing a Combined in situ Proximity Ligation Assay (isPLA) and Multiplex Immunofluorescence (mIF) Imaging Approach

*J. Boog[1], A. Christians[1], C.E. Jackson[2], M.H. Ingalls[2], J.S. Schwarz[2], S. Bodbin[3], A. Zieba-Wicher[4], S. Basu[4], O. Braubach[2]*
*[1]Canopy Biosciences- A Bruker Company, Research and Development, Hannover, Germany*
*[2]Canopy Biosciences- A Bruker Company, Research and Development, St. Louis, United States*
*[3]Navinci Diagnostics, Research and Development, Uppsala, Sweden*
*[4]Navinci Diagnostics, Management, Uppsala, Sweden*
**Introduction**
The immune checkpoint involving PD-1 and PD-L1 generates suppressive signals in T cells, which help to prevent autoimmunity by inducing a state of immune exhaustion. In the context of the tumor microenvironment (TME), however, cancer cells can manipulate these pathways to camouflage themselves from an immune attack. Blocking these checkpoints has thus emerged as a key immunotherapeutic tactic. Nonetheless, the success rate of immune checkpoint inhibitors (ICIs) has been mixed, even for patients who test positive for relevant diagnostic biomarkers. Patient stratification currently depends on immunohistochemical staining for checkpoint proteins like PD-L1, but these tests do not provide adequate stratification. A more comprehensive stratification, one that includes immune profiling of the TME plus an evaluation of immune checkpoint interactions, might provide better patient stratification and ICI responsiveness. Here we describe mIF that is augmented with a PD-1 and PD-L1 protein-protein interaction assay.
**Material and Methods**
Human formalin-fixed, paraffin-embedded (FFPE) tissue sections were subjected to standard histological processing and then incubated with primary antibodies against PD1 and PD-L1. Sections were then treated with oligonucleotide-modified secondary probes suitable for in situ Proximity Ligation Assay (isPLA), enabling the detection of PD1 and PD-L1 interactions. The PLA protocol utilized a fluorescent probe complementary to amplified nucleic acid to enhance the visibility of the protein interactions. The sample was then mounted in a closed microfluidic chamber and imaged on the CellScape™ platform. After imaging of the isPLA signal, the CellScape cyclic multiplex mIF staining approach was used to iteratively stain immune and structural markers on the tissue sample utilizing the VistaPlex™ Spatial Immune Profiling Assay Kit.
**Results and Discussions**
This proof-of-concept study demonstrates that high-plex mIF can be augmented with an isPLA on human FFPE samples. The combined application of both methods allows visualization of PD1/PD-L1 protein-protein interactions and integrates this interaction within the spatial context of the surrounding cell populations.
**Conclusion**
Spatial interactomics with single cell resolution allows for a more comprehensive insight into the interplay of different immune cell and non-immune cell populations during checkpoint activation processes in normal and neoplastic tissues.

### EACR2024-0441
### Multi-Exposure High Dynamic Range Microscopy Eliminates the Need for Exposure Optimization in High-Plex Spatial Biology Applications

*B. Berigan[1], J.S. Schwarz[2], M. Ingalls[2], X. Meshik[2], A. Northcutt[2], O. Braubach[2]*
*[1]Canopy Biosciences - A Bruker Company, Product Development, St. Louis, United States*
*[2]Canopy Biosciences - A Bruker Company, Research and Development, St. Louis, United States*
**Introduction**
High-plex immunofluorescence imaging allows researchers to perform deep cellular phenotyping with spatial context. Most high-plex imaging workflows require the end user to optimize individual antibody concentrations and image acquisition settings across several individual experiments for reproducible biomarker detection. Non-optimized antibody concentrations or exposure times result in under- or over-saturated images, both of which impact the accuracy of quantitative phenotyping data. As the number of markers used in spatial biology experiments increases, it becomes necessary to develop strategies that streamline or automate parameter optimization.
**Material and Methods**
The CellScape™ platform for Precise Spatial Multiplexing utilizes multi-exposure, high dynamic range (HDR) microscopy to collect in situ high-plex immunofluorescence imaging data. CellScape replaces the standard approach of acquiring one image using a single exposure time via an auto-acquisition process that acquires a series of images across a range of relevant exposure times. Each acquired image captures linear data, providing a series of images with signal intensities ranging from the dimmest to the brightest signals detectable by the system. As a result, a broader dynamic range of signal intensity is detectable relative to system's that utilize a single exposure time. Moreover, an 'optimal' exposure rate for each individual biomarker is collected during an experiment, thus overcoming the

Molecular Oncology 18 (Suppl. 1) (2024) 1–495
© 2024 The Authors. Molecular Oncology is published by John Wiley & Sons Ltd on behalf of Federation of European Biochemical Societies need for manual optimization of exposure times for each marker included in high-plex immunofluorescence assays.

**Results and Discussions**

Here we use CellScape to collect a high-plex immunofluorescence spatial biology dataset. Analysis of the dataset highlights both the extended linear dynamic range of the HDR image capture and a determination of the 'best-matched' exposure for each biomarker. Further, we extend this analysis to show how HDR can support biological variation and allow normalization that is not possible with single exposure imaging. Finally, we examine a reagent concentration series to demonstrate how HDR can be used to maximize the sensitivity of the assay by allowing increasing detection reagent concentration without compromising overall dynamic range for the signal.

**Conclusion**

Our data demonstrates that HDR microscopy supports the deployment of spatial biology as a tool to interrogate the tumor microenvironment, unravel responsiveness to drug treatments and cell therapies, and advance precision medicine.

### EACR2024-0449

### Detection of low frequency variants from cell free DNA samples using a high sensitivity cfDNA library preparation with enrichment method

*E. Bocian-Canepa[1], M. Cuadras[2], J. Beytebiere[2], R. Garcia[2], W. Jordan[2], M. Kellogg[2], A. Mai[2], V. Montel[2], A. Nicholson-Shaw[2], L. Watson[2]*

*[1]Illumina, Product Marketing, Cambridge, UK*

*[2]Illumina, Research and Development, San Diego, USA*

**Introduction**

Cell-free DNA from plasma combined with Next Generation Sequencing is an emerging tool for non-invasive evaluation of biomarkers in cancer. Genomic variants that arise in tissues are typically present at very low abundance in plasma and require detection methods with high sensitivity and specificity. We developed library preparation assay and analysis pipeline for detection of low frequency variants with high sensitivity and specificity from as little as 10 ng cfDNA input. In this study, we evaluate the sensitivity of the method for detection of SNV, CNV and fusion variants using a range of custom enrichment panels.

**Material and Methods**

The library preparation consists of end-repair of extracted cfDNA, followed by ligation of adapters containing UMIs for error correction, addition of indexes by PCR, and enrichment of regions of interest with a single hybridization step. Enrichment panels of sizes 55, 250 and 2000 kb were designed to target cancer-related genes of interest. Libraries were prepared from 20 ng of the following sample types: commercial cfDNA reference standard, cfDNA-like contrived sample (variants from cfDNA reference standard diluted in cfDNA), and nucleosome preparations (npDNA) from cell lines harboring variants of interest. To compare performance between 1-plex and 4-plex enrichment, individually enriched libraries were re-enriched using the same panel as 4-plex enrichment. Libraries were sequenced on Illumina NovaSeq™ 6000 or NextSeq™ 2000 sequencing systems. Alignment and variant calling were performed using an optimized DRAGEN™ integrated analysis pipeline on BaseSpace™ Sequence Hub.

**Results and Discussions**

The cfDNA samples achieved a high level of UMI-collapsed mean target coverage depth (>2000x), indicating high conversion efficiency from 20 ng cfDNA input. SNVs at 0.2% VAF, Indels and fusions at 0.5% VAF, and low abundance CNV were detected at ≥95% sensitivity. Evaluation of cfDNA from healthy donors showed ≥99.998% analytical specificity.

**Conclusion**

These results demonstrate that Illumina cfDNA Prep with Enrichment coupled with DRAGEN™ for ILMN cfDNA Prep with Enrichment App achieve high sensitivity for SNVs, Indels, gene fusions and CNVs from as little as 10 ng input. The assay is a versatile custom enrichment solution optimized for low input cfDNA and shows high concordance for variant detection between 1-plex and 4-plex enrichment formats. Illumina cfDNA Prep with Enrichment supports a range of panel sizes and is compatible with probe formats from Illumina or third-party providers.

### EACR2024-0450

### A Comprehensive Analytical Validation Framework for Immunofluorescence Assay Design Using CellScape Precise Spatial Multiplexing

*C. Jackson[1], A. Christians[2], J. Boog[2], M. Ingalls[1], X. Meshik[1], A. Northcutt[1], K. Cashion[1], S. Schwarz[1], O. Braubach[1]*

*[1]Canopy Biosciences - A Bruker Company, Research and Development, St. Louis, United States*

*[2]Canopy Biosciences - A Bruker Company, Research and Development, Hannover, Germany*

**Introduction**

Multiplex immunofluorescence (mIF) is a powerful immunology research tool that enables deep phenotyping of cells within the tissue microenvironment. Careful optimization of multiplex antibody panels for specificity, sensitivity and reproducibility is crucial to achieving robust data and is a requirement for assay repeatability and standardization. Here, we describe our validation procedure for multiplex assay panels.

**Material and Methods**

Experiments were performed using the CellScape™ platform for precise spatial multiplexing. The platform enables automated cyclic mIF for readily available fluorophore-conjugated antibodies compatible with fresh frozen or FFPE tissues. The use of high dynamic range imaging further facilitates quantitative phenotyping, while excellent imaging resolution provides single-cell discrimination with high accuracy. To design a ready-to-use panel for immune cell phenotyping, individual antibodies were first screened for specificity and then optimized for labeling sensitivity. Signal-to-noise, inter-assay reproducibility, and intra-assay precision were evaluated across serial sections in a multi-stage testing matrix to validate each antibody in isolation and

© 2024 The Authors. Molecular Oncology is published by John Wiley & Sons Ltd on behalf of Federation of European Biochemical Societies assembled into a panel. Resulting from this effort, we introduce a 15-plex ready-to-use VistaPlex™ immune profiling panel for human FFPE tissues.

**Results and Discussions**

Analysis of signal-to-noise ratios in the images across multiple sections and tissue types demonstrated robust performance of selected multiplexed antibodies. Measurements of both signal and quantitative immune phenotyping across technical replicates showed a high degree of intra-assay precision and inter-assay reproducibility. The 15-plex panel was validated on human tonsil, lung, breast, and colon tissues with the ability to positively identify epithelial cells and 22 cellular immune phenotypes most critical to understanding immune responses.

**Conclusion**

Our rigorous antibody validation protocol enables stringent and robust multiplex assay panel design. Pairing the CellScape platform with the comprehensively validated antibody panels streamlines deep immune profiling and enables this spatial biology workflow to be implemented across both basic and translational immuno-oncology research applications.

### EACR2024-0462
### Coordinated action of the nucleotide excision repair endonuclease ERCC1–XPF and TDP1–PARP1 in hepatitis B virus covalently closed circular DNA synthesis

*W. Huang*[1], *H.C. Hung*[2]
[1]*National Cheng Kung University, Medical Laboratory Science and Biotechnology, Tainan, Taiwan*
[2]*National Cheng Kung University, Basic Medical Sciences, Tainan, Taiwan*

**Introduction**

Chronic hepatitis B is a significant risk factor for hepatocellular carcinoma. The low rate of covalently closed circular DNA (cccDNA) clearance is a main cause of persistent viral infection. We previously found that nucleotide excision repair (NER) increased the formation of HBV cccDNA. Accordingly, we hypothesized that NER endonucleases are involved in resolving the viral rcDNA flap structure.

**Material and Methods**

Requirement of the NER 5' endonuclease ERCC1–XPF in HBV cccDNA synthesis in the liver cells was examined by quantitative PCR and Southern blotting analyses. Moreover, the chromatin immunoprecipitation and *in vitro* rcDNA cleavage assays were performed to detect the structural and functional associations of the ERCC1–XPF complex and tyrosyl DNA phosphodiesterase 1 (TDP1) with the HBV rcDNA flap. PARP1, which interacts with TDP1 to cleave the topoisomerase 1-DNA complex, was also investigated for its involvement in HBV cccDNA formation. Furthermore, the genetically epistatic relationship between the ERCC1–XPF and TDP1–PARP1 complexes in the cleavage of the rcDNA flap was evaluated. Finally, a digital PCR method to sensitively quantify cccDNA in human liver tissues was developed.

**Results and Discussions**

The NER 5' endonuclease ERCC1–XPF increased the cccDNA levels in HepAD38 and HepG2-NTCP cells. The ERCC1–XPF complex, as well as tyrosyl DNA phosphodiesterase 1 (TDP1), the primary enzyme that cleaves the topoisomerase 1-DNA complex, bound and cleaved the rcDNA flap to facilitate cccDNA synthesis. Inhibition of the enzymatic activity of PARP1, which interacts with TDP1 to cleave the topoisomerase 1-DNA complex, blocked cccDNA formation in a manner dependent on ERCC1, XPF, and TDP1. These findings suggested that the ERCC1–XPF and TDP1–PARP1 complexes are epistatic in the cleavage of the rcDNA flap and that PARP1 inhibitors have potential as therapeutic drugs to promote cccDNA clearance. Moreover, based on the digital PCR cccDNA quantification analysis, we found that the ERCC1, TDP1, and PARP1 levels correlated with the cccDNA levels in the liver.

**Conclusion**

The ERCC1–XPF and TDP1–PARP1 complexes function in concert to cleave the HBV rcDNA flap so that cccDNA synthesis can proceed. PARP1 inhibition provides a promising therapeutic strategy for cccDNA clearance.

### EACR2024-0480
### A functional role for highly glycosylated B7-H5/VISTA immune checkpoint protein in metastatic clear cell renal cell carcinoma

*M. Emaldi*[1], *P. Alamillo*[1], *E. Rey*[1], *L. Mosteiro*[2], *D. Lecumberri*[3], *R. Pulido*[1], *J.I. López*[1], *C.E. Nunes-Xavier*[1]
[1]*Biobizkaia Health Research Institute, Cancer, Barakaldo, Spain*
[2]*Cruces University Hospital, Department of Pathology, Barakaldo, Spain*
[3]*Hospital de Urduliz, Service of Urology, Urduliz, Spain*

**Introduction**

B7 family of immune checkpoint proteins control immune responses and protect healthy tissues from immune attack under physiological conditions. However, increased B7 protein expression hinders tumor elimination by the immune system. Immune checkpoint inhibitors targeting specific B7 proteins are used in clinical practice, but many patients do not respond or become resistant over time, making necessary the identification of novel response biomarkers and therapeutic targets. B7-H5 immune checkpoint protein is a promising candidate as an alternative immuno-therapeutic target in cancer treatment.

**Material and Methods**

We here performed a molecular, functional and clinical characterization of B7-H5 from renal cancer cells and metastatic ccRCC tumor specimens. We analyzed the expression and clinicopathological correlations of B7-H5 on tumor cells and tumor infiltrated leucocytes (TILs) in tumor tissue microarray samples from primary tumors and metastatic lesions from a cohort of 54 metastatic ccRCC patients.

**Results and Discussions**

mRNA and protein expression levels of B7-H5 were found upregulated in clear cell renal cell carcinomas

Molecular Oncology 18 (Suppl. 1) (2024) 1–495
© 2024 The Authors. Molecular Oncology is published by John Wiley & Sons Ltd on behalf of Federation of European Biochemical Societies

(ccRCC). Immunofluorescence assays revealed that B7-H5 is mainly expressed in the cell membrane, and Western blot analysis revealed that B7-H5 protein is heavily glycosylated. Mutagenic studies on B7-H5 identified the Asn(N) residues targeted by glycosylation as N49, N91, N108, N128, and N135, and revealed an impact of N-glycosylation on protein expression levels and protein localization. Knockdown of B7-H5 expression decreased 2D and 3D cell viability of renal cancer cells. B7-H5 expression on TILs correlated with stage, necrosis, disease free survival, overall survival, synchronous metastasis and metastasis to epithelial organs.

**Conclusion**

Our results provide insights into the molecular properties and clinical impact of B7-H5 in ccRCC, and support the suitability of B7-H5 as a new immunotherapeutic target in metastatic ccRCC.

### EACR2024-0488
### Eficacy of disitamab vedotin, trastuzumab emtansine, trastuzumab deruxtecan, and their combinations in HER2-positive breast cancer and gastric cancer: A preclinical study with mice models

*N. Pourjamal[1], V. Le Joncour[1], G. Vereb[2], C. Honkamäki[1], J. Isola[3], J.V. Leyton[4], P. Laakkonen[1], H. Joensuu[5], M. Barok[5]*
*[1]University of Helsinki, Faculty of Medicine, Helsinki, Finland*
*[2]University of Debrecen, Department of Biophysics and Cell Biology, Debrecen, Hungary*
*[3]Tampere University, Faculty of Medicine, Tampere, Finland*
*[4]University of Ottawa, Faculty of Medicine, Ottawa, Canada*
*[5]Helsinki University Central Hospital, Faculty of Medicine – Oncology Department, Helsinki, Finland*

**Introduction**

Most HER2-positive breast cancers or gastric cancers become resistant to the approved anti-HER2 antibody-drug conjugates (ADC) trastuzumab emtansine (T-DM1) and trastuzumab deruxtecan (T-DXd), and such patients have limited therapeutic options.

**Material and Methods**

We compared the efficacy of T-DM1, T-DXd, and disitamab vedotin (DV), a novel anti-HER2 ADC, as single agents and in dual combinations on cell survival in ten HER2-positive breast cancer or gastric cancer cell lines, of which seven were T-DM1-sensitive (SKBR-3, UACC-812, EFM-192A, N87, OE19, JIMT-1, BT-474) and three T-DM1-resistant (RN87, ROE19, SNU-216), in a HER2-negative breast cancer cell line Hs-578T (control). Then, we investigated the efficacy of these single agents and their dual combinations in T-DM1-sensitive, T-DM1-resistant, and T-DXd-resistant HER2-positive mouse xenograft models of HER2-positive breast cancer and gastric cancer.

**Results and Discussions**

T-DXd was less efficient than T-DM1 in vitro yet inhibited more efficiently than T-DM1 the growth of HER2-positive breast cancer and gastric cancer xenografts in vivo, including T-DM1-resistant RN87 and JIMT-1-tumors. DV was highly efficacious in vitro on T-DM1- and T-DXd-resistant HER2-positive cancer cell lines, and DV inhibited the growth of RN87 and JIMT-1 xenografts that had progressed on T-DM1 and T-DXd. The combinations of T-DM1 plus DV and T-DXd plus DV had greater anti-cancer efficacy than the corresponding single agents in multiple cell lines and in JIMT-1 and N87 mice xenograft models.

**Conclusion**

The results support the evaluation of DV and its combinations with T-DM1 and T-DXd in clinical trials in patients with HER2-positive breast cancer and gastric cancer who progress on T-DM1 and/or T-DXd.

### EACR2024-0500
### Patient-derived gastric cancer organoids mirror tumor features and display heterogeneous drug responses correlating with patient' clinical outcomes

*M. Cabeza-Segura[1], A. Marín-Alonso[1], A. González-Vilanova[1], A. Aguilar-Cifre[1], R. Villagrasa[2], B. Palomar[3], C. Martínez-Ciarpaglini[4], A. Cervantes[5], T. Fleitas[5], J. Castillo[6]*
*[1]INCLIVA Biomedical Research Institute, Medical Oncology, Valencia, Spain*
*[2]Hospital Clinico Universitario de Valencia, Gastro-enterology and Hepatology, Valencia, Spain*
*[3]INCLIVA Biomedical Research Institute, Pathology, Valencia, Spain*
*[4]INCLIVA Biomedical Research Institute- CIBERONC-Instituto de Salud Carlos III, Pathology, Valencia, Spain*
*[5]INCLIVA Biomedical Research Instituto- CIBERONC-Instituto de Salud Carlos III, Medical Oncology, Valencia, Spain*
*[6]INCLIVA Biomedical Research Institute- CIBERONC-Instituto de Salud Carlos III- University of Valencia, Biochemistry and Molecular Biology, Valencia, Spain*

**Introduction**

Gastric cancer (GC) is an aggressive and heterogenous malignancy frequently diagnosed at advanced stages. In this context, chemotherapy represents the main therapeutic option. The addition of targeted therapies (trastuzumab, zolbetuximab…) and immunotherapy had improved the outcomes for some specific profiles. Nevertheless, many patients do not respond or develop resistance. Therefore, reliable tumor models that recapitulate the heterogeneity and pathophysiology of patient tumors is an urgent need. Patient-derived gastric tumor organoids (GTO) represent an appropriate functional model to study tumor biology and to perform drug sensitivity assays in a personalized manner.

**Material and Methods**

A prospective biobank of GTOs derived from advanced GC patients was generated according to an in-house protocol from January 2019. Hematoxylin-eosin (H&E) staining and immunohistochemistry (IHC) evaluating HER2, MLH1, MSH2, MSH6 and PMS2 expression was performed in both GTOs and tumor tissues. To characterize the genomic profiles, whole exome

© 2024 The Authors. Molecular Oncology is published by John Wiley & Sons Ltd on behalf of Federation of European Biochemical Societies sequencing (WES) was carried out. Drug sensitivity assays to chemotherapy drugs and targeted agents were performed through cell viability experiments. Clinico-pathological data of the patients from whom the GTOs were generated were also collected.

**Results and Discussions**

From January 2019, 36 GTOs lines from 30 patients were established, encompassing all histological subtypes of GC as well as other models of clinical interest, such as those with amplifications of the *ERBB2* gene and those with microsatellite instability. This biobank also includes organoids derived from both treatment-naïve and pre-treated patients. H&E and IHC evaluation revealed that GTOs recapitulate the phenotypic features and the heterogenous HER2 and repair proteins expression from tumor tissues. On the other hand, WES analysis showed a relevant molecular concordance between GTOs and tumor biopsies. Furthermore, GTOs exhibited heterogeneous drug responses to 5-fluorouracil, oxaliplatin, cisplatin, paclitaxel, SN38, trastuzumab and lapatinib that correlate with patient clinical outcomes. Interestingly, those models generated from untreated tumors recapitulated the subsequent patient clinical response, while those GTOs established from tumors pre-treated with chemotherapy or HER2-targeted therapy showed greater resistance to that same treatment.

**Conclusion**

Patient-derived GTOs could become a potential tool for functional precision medicine as predictors of patient clinical outcomes in gastric cancer.

### EACR2024-0521
### Identification and positioning of an approved drug, MS-AP-031, to delay metastasis of primary colorectal cancer tumour patients in an adjuvant setting

*D. Roy Chowdhury[1], C. Amarakanth[1], S. Kannan[1], S. Wahid[1], J. Sanyal[1], R. Singh[1], J. Tayal[2], A. Mehta[3], D. Doval[4], A. Roy Chowdhury[1]*
*[1]Mestastop Solutions Pvt. Ltd., Core R&D, Bengaluru, India*
*[2]Rajiv Gandhi Cancer Institute and Research Centre, Bio-repository, New Delhi, India*
*[3]Rajiv Gandhi Cancer Institute and Research Centre, Molecular Diagnostics and Transfusion Services, New Delhi, India*
*[4]Rajiv Gandhi Cancer Institute and Research Centre, Medical Oncology, New Delhi, India*

**Introduction**

Metastasis is the cause of 90% fatality in cancer patients. The available medicines only focus on the proliferation of the primary or secondary tumours, not on abrogating the biology of metastasis. The failure of clinical candidates directed towards metastasis questions the relevance of target-based therapy and our understanding of the complex process. Our research addresses this question by focusing on a translational, phenotypic approach validated with retrospective clinical studies. We establish the relevance of critical steps in metastasis biology and how some drugs approved for non-oncology chronic indications can delay metastasis without impacting the primary tumour.

**Material and Methods**

We have created three proprietary platforms, METAssay, METSCAN and METVivo, which faithfully imitate the metastasis process in vitro, ex vivo patient primary tumour samples, and in vivo settings. From a retrospective clinical trial of colorectal cancer patients with a five-year follow-up, the METSCAN platform identified few approved drugs, resulting in increased progression-free survival of patients. We tested these drugs in our METAssay platform, and the compound that showed the most promising results in delaying metastasis without affecting the proliferation of primary tumour was tested in the METVivo platform.

**Results and Discussions**

All our in vitro studies were performed at non-cytotoxic concentrations. MS-AP-031, the most effective compound, surprisingly promoted epithelial-to-mesenchymal transition (EMT) and invasion. It inhibited both intravasation and extravasation and also reduced tumour cell-platelet binding. Our studies showed that the drug effectively pushed the cells towards the mesenchymal axis, increasing its cellular stiffness, senescence and plasticity ratio (PR). Translational studies with ex-vivo patient samples also supported this observation. Animal studies in drosophila and mice confirmed the ability of MS-AP-031 to reduce metastasis without having any significant effect on the primary tumour site at the concentrations used.

**Conclusion**

Our studies suggest that MS-AP-031 promotes EMT and inhibits the reverse mesenchymal to epithelial transition (MET), thereby preventing tumour growth in secondary tissue and inhibiting the most significant step in metastasis seeding, re-epithelization. Dose-dependent and PK/PD studies are currently underway to position MS-AP-031 as a drug-repurposing clinical candidate to delay metastasis in an adjuvant setting.

### EACR2024-0527
### Chemo-Immunotherapy of Epirubicine micelle and anti-PD-1 antibody for Reprogramming Tumor Microenvironment to Overcome ICI-Resistance

*H. Kinoh[1], S. Quader[1], H. Cabral[2], K. Kataoka[1]*
*[1]Inovation Center of Nanomedicine, Kataoka-Kinoh Laboratory, Kawasaki, Japan*
*[2]The University of Tokyo, Department of Bioengineering, Graduate School of Engineering, Tokyo, Japan*

**Introduction**

Cancer immunotherapy with antibodies (ICI) that block the PD-1/PD-L1 pathway of immune checkpoints has no effect on pancreatic cancer. One of the main reasons for this low efficacy is the thick stroma of pancreatic tumors. Previously, we developed epirubicin-encapsulated micelles (Epi/m) designed to accumulate in tumor tissues and release their payload at intratumoral pH, which allows avoiding drug leakage at physiological pH, sparing healthy tissues and increasing active dose in tumors.

**Material and Methods**

© 2024 The Authors. Molecular Oncology is published by John Wiley & Sons Ltd on behalf of Federation of European Biochemical Societies

Using Pan02(Kras wt, Trp53 wt) and KPC (LSL-KrasG12D/+, LSL-Trp53R172H/+) cells, we challenged the combination efficacy of Epirubicin micelles (Epi/m), anti-PD-1, Gemicitabine, and Nab-PTX. Furthermore, we also investigate the stroma degradation against these combination using immuno-histochemistry and CUBIC methods. Given the promising anti-tumor activity observed in angiosarcoma patients, US FDA has designated NC-6300 clinical development program for angiosarcoma as Fast Track Designation in 2021.

**Results and Discussions**

We found the combination of Epi/m and anti-PD-1 antibody induced MMP and hyaluronidase to degrade collagen and hyaluronic acid on tumor stroma, so that antibody drugs and CTLs can reach deep inside pancreatic cancer after systemic administration. The combination also promoted infiltration of dendritic cells and M1-like macrophages, but suppressed MDSCs into tumors. The exposure of the Epi/m induce IL6, TNFα, and IL1β in the tumor. Furthermore, the Epi/m reduced the levels of cytidine deaminase protein through reactive oxygen species-mediated degradation, resulting in the increased stabilization of gemcitabine. Thus, the combination of Epi/m, aPD1 and gemcitabine eradicated orthotopic pancreatic tumors (KPC model) (CR = 90%), inhibiting liver metastasis with long-term memory effects.We found the combination of Epi/m and anti-PD-1 antibody induced MMP and hyaluronidase to degrade ECM on tumor stroma, so that antibody drugs and CTLs can reach deep inside of pancreatic cancer. Furthermore, the Epi/m reduced the levels of cytidine deaminase and ATF4 protein through ROS-mediated degradation, resulting in the increased stabilization of gemcitabine. Thus, the combination of Epi/m, aPD1 and gemcitabine eradicated orthotopic pancreatic tumors (KPC model) (CR = 90%), inhibiting liver metastasis with long-term memory effects.

**Conclusion**

Our findings support the concept that suboptimal intratumoral concentrations of gemcitabine represent a crucial mechanism of therapeutic resistance in PDA.

### EACR2024-0530
### Rapid Mechanobiology-Based Diagnosis and Early Prognosis of Cancer Metastasis

*D. Weihs*[1]
[1]*Technion-Israel Institute of Technology, Biomedical Engineering, Haifa, Israel*

**Introduction**

Metastasis is a leading cause of cancer-related deaths, underscoring the need for early detection and prediction. Current methods rely on histopathology, disease statistics, or genetics, which can be inaccurate, time-consuming, and rely on known markers. We present an innovative, rapid (~2hr) mechanobiology-based approach for diagnosing cancer and predicting the invasiveness of cells, which correlates with clinical metastasis likelihood.

**Material and Methods**

We evaluate the invasiveness of 10 breast- and pancreatic cancer cell-lines, as well as >30 human pancreatic and skin tissue samples. Cells are seeded on physiological-stiffness gels, and within an hour, invasive cell subsets forcefully indent the gels, while non-invasive cells do not. The percentage of cells indenting and their depths, evaluated within 2 hours, provide the mechanical invasiveness of the sample. Cell line and tumor sample results were, respectively, validated against Boyden chamber migration assays and clinical histopathology and patient follow-up. Invasive cell subsets, such as cancer stem cells, were collected via FACS and separately evaluated. Machine learning models were developed for prediction.

**Results and Discussions**

The mechanical invasiveness, as determined by the percentage of indenting cells and their depths, demonstrates direct agreement with migratory capacity of cell lines and clinical invasiveness of tumors. Cancer stem cells, a small subset, were shown to be highly invasive, providing a prognostic predictor. Machine learning models accurately predicted invasiveness and metastatic risk with high sensitivity and specificity.

**Conclusion**

Metastasis is a primary driver of cancer-related mortality, emphasizing the need for early detection and early prognosis of metastatic potential. Our innovative mechanobiology-based assay rapidly and accurately identifies cancer and predicts the metastatic likelihood, providing cancer-type-specific prognostic thresholds. This approach can significantly impact patient-specific treatment strategies and disease management by providing timely information on metastatic risk.

### EACR2024-0563
### Gender-specific involvement of steroid hormones and their receptors in the progression of low- and high-grade gliomas

*A. Hirtz*[1], *A. Gantner*[1], *F. Rech*[1], *M. Thomassin*[1], *L. Taillandier*[1], *H. Dubois-Pot-Schneider*[1], *H. Dumond*[1]
[1]*University of Lorraine, CRAN UMR 7039 - BioSiS, Vandoeuvre lès Nancy, France*

**Introduction**

Gliomas are the most common primary brain tumors in adults. The WHO classifies these tumors according to their degree of malignancy and molecular subtype : astrocytoma (IDH mut, grade 2,3,4), oligodendroglioma (IDHmut, 1p19q codel, grades 2,3) and glioblastoma (IDH wt grade 4). The current treatment combines surgery, chemotherapy and radiotherapy. Few risk factors are clearly defined, but women are 1.5-fold less affected and survive longer than men. If gender differences in the natural history of the disease are widely described, their underlying mechanisms remain to be determined. Based on these observations, we hypothesized that female hormonal signaling have neuroprotective properties and addressed the key role of enzyme involved in oestrogens biosynthesis (aromatase), and nuclear (ERa, ERb, AR) or membrane (GPER) hormone receptors.

**Material and Methods**

We mined the transcriptomic and clinical data from public databases (TCGA and CGGA) to identify male-over-female differentially expressed genes and selected those associated with patient survival using univariate analysis, depending on the grade, and molecular status. The link between the expression levels of steroid

© 2024The Authors. Molecular Oncology is published by John Wiley & Sons Ltd on behalf of Federation of European Biochemical Societies biosynthesis enzyme or receptors of interest and survival was studied using the log rank test. A functional analysis of gender-specific correlated genes was performed. The in silico results are currently compared with experimental data from spatial transcriptomics.

**Results and Discussions**

Our analysis highlighted a link between the expression level of aromatase or hormone receptors with patient survival, depending on gender and molecular subtype. Low expression of aromatase, AR and nuclear ER were mainly associated with patient survival and their correlated genes were mainly related to immune response. However, high expression of GPER was associated to better survival, especially in females and correlated genes were related to angiogenesis. Moreover, HOX-related genes appeared to be differentially expressed between males and females in low grade glioma suggesting that these tumors could originate in perturbation of developmental signals.

**Conclusion**

Consideration of the tight control of steroid hormone production and signaling seems crucial for the understanding of glioma pathogenesis and emergence of future tailored therapies. Complementary approaches like spatial transcriptomics is on the way to better understand the hormonal impact on intratumor heterogeneity, often responsible for the systematic recurrence and mortality of these tumors.

### EACR2024-0577

### Eukaryotic translation initiation factor 4A3 inhibitors express differential effects on different subtypes of breast cancer

*J. Ling[1], A. Garrett[1], K. Chang[1], C. Joe[1], R. Mizojiri[2]*

*[1]California University of Science and Medicine, medical education, Colton, United States*

*[2]Takeda Pharmaceutical Company Ltd., Research, Fujisawa, Japan*

**Introduction**

Eukaryotic translation initiation factor 4A3 (eIF4A3) is a core component of exon junction complex (EJC) that plays regulatory roles in the whole process of mRNA metabolism, thus emerging as a promising target to regulate cancer development. Although eIF4A3 has been identified to express oncogenic activities in breast cancers (BC), targeting it for therapies remains unexplored. In this study, we utilized eIF4A3-selective inhibitors to examine the potential of modulating eIF4A3 for breast cancer therapies.

**Material and Methods**

To identify the specificity of eIF4A3 inhibitors (1a, 1o, and 1q) on BC cell growth, various subtypes of BC cell lines were used, including luminal A (e.g., MCF-7), luminal B, triple negative (TNBC) (e.g., MDA-MB-231), and HER2-enriched subtypes. A patient-derived organoid (PDO) was also tested. Western blot, cell-based assays, flow cytometry, confocal microscopy, and RNA-Seq were utilized to analyze the effects and mechanisms of the inhibitors.

**Results and Discussions**

Cell proliferation/ cytotoxicity assays identified that the inhibitors suppressed the growth of all BC cells with the order of efficacy as 1q>1o>1a. Interestingly, TNBC cells were more sensitive to these inhibitors than luminal subtypes, and the sensitivity of HER2-enriched subtype was in-between. Through a TNBC PDO model that better mimics BC pathology, we identified that 1q was very effective to inhibit the organoid growth with $IC_{50}$ at 1.202 μM. Treatment of MDA-MB-231 cells with 1q inhibited cell cycle progression at M phase and/or the transition from M to G1 phase, but MCF-7 cells were inhibited at S phase, showing a different responsive pattern. Western blot revealed that while the levels of three EJC components were not affected by 1q, MLN51(a peripheral component of EJC) was specifically cleaved, suggesting that eIF4A3 inhibitors may function through MLN51 to suppress BC cell growth. Indeed, we observed that MLN51 localization was altered upon 1q treatment, wherein it became more nucleus localized in MDA-MB-231cells but more defused in MCF-7 cells. Finally, RNA-seq results unveiled different patterns of gene expression between TNBC and luminal BC, providing specific biological pathways to enhance our further study on the druggability of eIF4A3.

**Conclusion**

This study identifies that eIF4A3 inhibitors are effective to suppress BC cell growth, especially with higher efficacy on TNBC cells, providing a novel strategy for the development of precision medicine for TNBC, one of the most aggressive BCs and unmet medical needs.

### EACR2024-0648

### Long-term humanization of NOG mice and next-generation NOG strains to induce lineage-specific differentiation of immune cells for assessment of checkpoint inhibitors and immune cell engagers

*M. Stecklum[1], L. Baskin[2], D. Behrens[1], J. Hoffmann[3]*

*[1]EPO GmbH, Preclinical Immunoncology, Berlin, Germany*

*[2]Taconic Biosciences, Research and Development, Rensselaer, United States*

*[3]EPO GmbH, Immunoncology Research, Berlin, Germany*

**Introduction**

The preclinical evaluation of novel immune therapies requires humanized immune system (HIS) mouse models. In previous studies we have demonstrated that either peripheral blood mononuclear cells or CD34+ hematopoietic stem cells (HSC) can be used to establish a HIS model. With the development of next-generation NOG mice, lineage-specific differentiation of immune cell sub-populations can be supported. Transplantation of cell line-derived (CDX) or patient-derived (PDX) tumor xenografts on HIS mice provides a full model for human tumors for the investigation of checkpoint inhibitors (CPI), as well as novel cell therapies and immune cell engagers.

**Material and Methods**

HSC-humanized mice were generated by i.v. transplantation of HSC to immunodeficient mice (single donors, mixed donor pool) . For humanization, NOG mice and next-generation NOG strains were used: NOG, NOG-EXL, hIL-2 NOG, hIL-6 NOG and FcResolv™

© 2024 The Authors. Molecular Oncology is published by John Wiley & Sons Ltd on behalf of Federation of European Biochemical Societies

NOG mice were compared to each other for lineage-specific differentiation. Long-term survival was monitored and engraftment of immune cells was analyzed by FACS of blood every 4 weeks. In humanized NOG mice, CDX and PDX from different entities were s.c. transplanted and used to evaluate CPI.

**Results and Discussions**

Humanized hIL-2 NOG mice showed significantly decreased survival after HSC transplantation in comparison to the other mouse strains and had to be sacrificed within the first 6-8 weeks after HSC transplantation followed by huNOG-EXL after 16-20 weeks. In all mouse strains, transplanted HSCs engrafted and differentiated mainly into B and T cells. NOG-EXL mice displayed the highest engraftment, with up to 80% of human cells in the blood, including a higher portion of myeloid cells. In humanized hIL-6 NOG mice a higher portion of monocytes could be determined. In FcResolv™ NOG mice engraft and differentiate HSCs similarly to the parent NOG strain. Long-term survival is still ongoing. In humanized NOG mice, selected CDX and PDX tumors successfully engrafted without significant differences in tumor growth compared to non-humanized mice. CPI treatments induced tumor growth delay.

**Conclusion**

Next-generation NOG mouse strains are characterized by a lineage-specific differentiation of immune cells depending on integrated human cytokines. Furthermore, we established a human tumor-immune-cell model for NOG mice using different entities of CDX or PDX in combination with CPI, which can be used for preclinical translational studies on tumor immune biology as well as evaluation of new therapies and drug combinations.

### EACR2024-0666

### Functional paclitaxel and eribulin assay for patient derived xenograft breast cancer tissue ex vivo correlates with in vivo sensitivity in mice

*D. van Gent[1], Z. Komar[1], N.S. Verkaik[1], E. Marangoni[2], A. Dahmani[2], A.B. Houtsmuller[3], R. Kanaar[1], A. Jager[4]*

*[1]Erasmus MC Cancer Institute, Molecular Genetics, Rotterdam, The Netherlands*

*[2]Institut Curie, Translational Research Department, Paris, France*

*[3]Erasmus MC, Erasmus Optical Imaging Center and Department of Pathology, Rotterdam, The Netherlands*

*[4]Erasmus MC Cancer Institute, Medical Oncology, Rotterdam, The Netherlands*

**Introduction**

Breast Cancer (BrC) response to chemotherapy is variable. Therefore, the need for predictive biomarkers for chemotherapy response is high. However, genetic or histological biomarkers are not sufficient to correctly predict therapy response. Therefore, we aimed to develop an *ex vivo* assay to predict chemotherapy response in BrC patients, using *ex vivo* and Cancer-on-Chip (CoC) approaches.

**Material and Methods**

Patient-Derived Xenograft (PDX) tumor with known in vivo paclitaxel sensitivity and primary BrC tumors were sliced and cultured in 6-well plates (referred to as ex vivo culture), or in the CoC platform (BI/OND). Tissue slices were treated with paclitaxel or eribulin under both culture conditions and Formalin-Fixed Paraffin-Embedded. 4 µM tissue sections were immunostained for proliferation, mitosis and apoptosis. Multiplex analysis was performed to detect multiple immune markers of the tumor microenvironment (TME).

**Results and Discussions**

Our study shows that both the PDX and primary BrC tumors stay proliferative and viable in the ex vivo and CoC culture systems. TME composition was identified and shown to remain approximately constant during the 3 days ex vivo culture. To assess paclitaxel-sensitivity we first investigated the effects on proliferation and cell death after 3-5 days in culture. Unexpectedly, we did not observe any effect on these parameters, even at the highest drug concentrations. Therefore, we developed an assay based on the ratio between mitotic and S-phase cells. Here, we found that cells arrested in mitosis, but apparently managed to proceed through the cell cycle in the long run. Interestingly, we found a strong correlation between the predicted ex vivo outcome of the mitotic arrest assay and the in vivo sensitivity tested in mice. Moreover, we found that the robustness and sensitivity of the assay was good.

**Conclusion**

In this study paclitaxel and eribulin sensitivity assays were optimized for BrC PDX and primary tumor material. Eventually, a clinical study will be set-up to validate the newly developed paclitaxel prediction assay. Time lapse live microscopic methods will be applied to further investigate the mechanism of paclitaxel and eribulin sensitivity.

### EACR2024-0745

### Cancer patient-derived organoids dependency map

*C. Herranz Ors[1], S. Bhosle[1], A. Beck[1], S. Barthorpe[1], H. Lightfoot[1], J. Gilbert[1], H. Rogers[2], C. Beaver[3], H.E. Francies[1], M.J. Garnett[1]*

*[1]Wellcome Sanger Institute, Cancer- Ageing & Somatic Mutation Programme, Hinxton, United Kingdom*

*[2]Wellcome Sanger Institute, Cellular Operations, Hinxton, United Kingdom*

*[3]Wellcome Sanger Institute, Scientific Operations, Hinxton, United Kingdom*

**Introduction**

Cancer in vitro cell culture models are ubiquitous in research and drug discovery. The current widely available set of ~1,000 human cancer cell lines has limitations including poor representation or the absence of cancer types, no germline data for accurately calling somatic variation, a lack of matched patient or clinical data, and genetic drift due to prolonged culturing. Tumour organoids are 3D in vitro cultures incorporating basement membrane extract and niche factors that can be generated from multiple cancer types and, to a varying extent, reflect the genetics, heterogeneity, histology and clinical responses of tumours. Nonetheless, to date, there have been few systematic efforts to derive large numbers of annotated organoids, and many organoid cultures are non-renewable, undergoing replicative senescence in

© 2024 The Authors. Molecular Oncology is published by John Wiley & Sons Ltd on behalf of Federation of European Biochemical Societies culture, and further limiting their use. Here we report, as Cell Model Network UK, the systematic creation of a highly-annotated renewable cancer patient-derived organoid biobank and demonstrate their utility for mapping cancer dependencies.

**Material and Methods**

More than 250 organoids were successfully derived and banked from colorectal, gastric, oesophagal, ovarian, and pancreatic tumours. These models were comprehensively annotated with clinical information, and whole-genome DNA and RNA sequencing were performed. Whenever possible, matched tumour samples were also sequenced. Genome-wide CRISPR-Cas9 screens were conducted on a subset of organoids and drug screenings were performed to validate some of the cancer dependencies found.

**Results and Discussions**

This biobank enables us to underscore the diverse and informative landscape of the organoid cohorts, capturing both common and rare genomic alterations across various tumour types. Organoids faithfully recapitulate the genomic diversity of the original lesions and exhibit stability over time in culture, confirming their suitability for clinically relevant research. Their high purity further highlights their advantage in exploring the diversity and evolutionary changes in cancer cells. In addition, organoids captured singularities related to patients' treatment. Functional data analyses identified colorectal organoid-specific cancer dependencies primarily associated with the EGFR-RAS-MAPK signalling pathway.

**Conclusion**

This fully annotated and accessible platform offers a powerful tool for personalised medicine approaches and allows large-scale screenings in complex and diverse 3D patient-derived models.

### EACR2024-0756

### Glucose oxygenase-functionalized nanomotors as a new therapeutic strategy for the treatment of breast cancer

*S. Torres Ruiz[1], P. Díez[2,3,4], A. Àgreda-Roca[1], A. Lameirinhas[1,2], M. Tapia[1,5], A. Lluch[1,5,6,7], B. Bermejo[1,5,6], J.M. Cejalvo[1,5,6], I. Garrido-Cano[1,2,3], R. Martínez-Máñez[2,3,4,8]*

*[1]Biomedical Research Institute INCLIVA, Breast Cancer Biology Research Group, València, Spain*

*[2]Universitat Politècnica de València, Instituto Inter-universitario de Investigación de Reconocimiento Molecular y Desarrollo Tecnológico IDM, València, Spain*

*[3]CIBER-BBN, CIBER de Bioingeniería- Biomateriales y Nanomedicina, Madrid, Spain*

*[4]Universitat Politècnica de València, Unidad Mixta UPV-CIPF de Investigación en Mecanismos de Enfermedades y Nanomedicina, València, Spain*

*[5]Hospital Clínico Universitario de Valencia, Clinical Oncology Department, València, Spain*

*[6]CIBERONC, Centro de Investigación Biomédica en Red de Cáncer, València, Spain*

*[7]University of Valencia, Department of Physiology, València, Spain*

*[8]Universitat Politècnica de València-Instituto de Investigación Sanitaria La Fe, Unidad Mixta de Investigación en Nanomedicina y Sensores, València, Spain*

**Introduction**

Breast cancer is one of the leading causes of cancer-related death among women worldwide. Due to this fact, numerous researchers are focused on improving the efficacy of actual therapies for breast cancer treatment. This work aimed to develop nanomotors (NMs)-based nanodevices and to evaluate their therapeutic efficacy for the treatment of breast tumors. NMs-based drug delivery systems are promising therapeutic tools in the cancer field that exhibit auto-propulsion by converting available fuels into mechanical force. These systems offer the potential to improve treatment outcomes while minimizing adverse effects associated with traditional therapies. In this sense, we design a glucose oxygenase (GOx)-functionalized NM that takes advantage of the high glucose consumption rate of cancer cells for efficient doxorubicin delivery.

**Material and Methods**

The NMs consist of Janus-type nanoparticles with two opposing faces, a platinum nanoparticle and a mesoporous silica nanoparticle loaded with the cytotoxic drug doxorubicin (DOX) and whose surface was functionalized with the active ($NM_{DOX\text{-}GOx}$) or inactive ($NM_{DOX\text{-}GOx\text{-}IN}$) GOx enzyme. An empty NM functionalized with active GOx ($NM_{GOx}$) was also synthesized. NMs were characterized by standard techniques. Motion and cargo release studies were performed, and anti-tumor properties were evaluated through cell cytotoxicity and tumor penetrability assays in patient-derived organoids (PDO) from triple-negative breast cancer patients.  PDO viability was determined by CellTiter-Glo assay, and penetrability was determined by confocal microscopy.

**Results and Discussions**

$NM_{DOX\text{-}GOx}$ showed an increased diffusive motion in the presence of rising glucose concentrations when compared to $NM_{DOX\text{-}GOx\text{-}IN}$. Cargo release studies confirmed the controlled delivery of DOX in the presence of protease. Furthermore, penetrability studies revealed the high efficiency of $NM_{DOX\text{-}GOx}$ to effectively deliver DOX in PDO. By contrast, treatment with $NM_{DOX\text{-}GOx\text{-}IN}$ exhibited a significantly lower efficacy in the delivery of DOX to PDO. These results were also confirmed by cytotoxicity assays, which revealed a significantly higher cytotoxic activity of $NM_{DOX\text{-}GOx}$ when compared to control NMs.

**Conclusion**

Altogether, these findings support the potential of the developed nanodevices as innovative and effective therapeutic agents for the treatment of breast cancer.

### EACR2024-0779

### Comprehensive spatial phenotyping across the discovery to clinical continuum

*A. Pratapa[1], N. Ma[1], A. Viratham[1], V. Goubert[1], N. Jhaveri[1]*

*[1]Akoya Biosciences, Applications, Marlborough, USA*

**Introduction**

© 2024 The Authors. Molecular Oncology is published by John Wiley & Sons Ltd on behalf of Federation of European Biochemical Societies

The transition from identifying predictive spatial biomarkers, known as spatial signatures, for immunotherapy responses to their clinical implementation requires a cohesive strategy that connects ultrahigh-plex discovery experiments with high-throughput translational investigations. This research focuses on integrating Akoya Biosciences' spatial multiplexed imaging technologies with advanced data analysis methods to achieve comprehensive spatial phenotyping from discovery to clinical applications.

**Material and Methods**

Human formalin-fixed, paraffin-embedded (FFPE) cancer tissues underwent profiling using ultrahigh-plex PhenoCode™ Discovery panels to assess cell lineage, immune activation, and checkpoint markers through the PhenoCycler®-Fusion spatial biology platform. Then, PhenoCode™ Signature panels were used to target specific biomarkers related to immune profile, immune contexture, tumor-infiltrating lymphocytes (TIL), macrophage polarization, and T cell status using the PhenoImager® HT platform. Whole slide image analysis was utilized for precise image analysis tasks, such as region of interest (ROI) segmentation, cell detection, classification, exploration of spatial interactions, and identification of distinct spatial signatures.

**Results and Discussions**

Our investigation unveiled unique spatial relationships across different tumor categories, providing quantification of immune cell distributions and their interconnections. The ultrahigh-plex data demonstrated strong correlation with high-throughput signature panel analyses, thereby paving the way for a streamlined approach to pinpointing and crafting predictive spatial signatures for immunotherapy efficacy.

**Conclusion**

Leveraging ultrahigh-plex discovery panels, high-throughput signature panels, and advanced deep-learning image analysis offers a holistic comprehension of cellular interactions within the tumor microenvironment. This integrated methodology, leveraging Akoya's end-to-end workflows, expedites the discovery of predictive spatial biomarker signatures across various human tissue samples.

### EACR2024-0816

### Assessing anti-tumor efficacy of tumor-dendritic cell reprogramming using immuno-competent 3D tumor microtissue models

*I. Agarkova[1], N. Rotankova[1], C. Veser[1], M. Rudnik[1], L. Laure-Anne[1], R. Andre[2], R. Emilie[3], P. Cristiana[2], R. Fábio F.[4], P. Carlos-Filipe[5]*

*[1]Insphero AG, Immuno-Oncology, Schlieren, Switzerland*
*[2]Asgard Therapeutics, Immuno-Oncology, Lund, Sweden*
*[3]Asgard Therapeutics, Oncology, Lund, Sweden*
*[4]Asgard Therapeutics, Research, Lund, Sweden*
*[5]Lund University, Molecular Medicine and Gene Therapy, Lund, Sweden*

**Introduction**

Despite the extensive use of mouse models in pre-clinical cancer research, their translatability is often limited by inter-species differences. Recent studies have reported that overexpression of the transcription factors PU.1, IRF8, and BATF3 (PIB) in cancer cells induces reprogramming into functional antigen-presenting type 1 conventional dendritic cells (cDC1s). This suggests a novel strategy for cancer immunotherapy based on the recreation of cDC1s′ functional properties in tumor cells by in-vivo reprogramming, forcing presentation of endogenous tumor neoantigens and inducing personalized anti-tumor immunity. Here, we evaluate efficacy of cDC1 reprogramming using innovative in-vitro 3D human immunocompetent tumor microtissue (TMT) models.

**Material and Methods**

TMTs were generated by co-aggregation of T98G or A375 human tumor cell lines with cancer-associated fibroblasts in proprietary AKURA™ 384 well plates. We transduced TMTs with PIB-mCherry-encoding lentiviral particles at different multiplicities of infection (MOIs) and profiled cDC1 reprogramming efficiency by immunofluorescence staining and high-content confocal imaging. We observed that cDC1 reprogramming progresses in 3D TMTs and is associated with expression of the dendritic cell markers CD45 and HLA-DR. Additionally, we observed that higher MOI is associated with higher transduction and reprogramming efficiency and decreased TMT size. To further investigate *ex vivo* antitumor efficacy, we co-cultured TMTs with HLA-matched PBMCs and profiled cytokine secretion and TMT size as readouts for T cell activation and cytotoxicity.

**Results and Discussions**

We observed increased secretion levels of IFNγ, TNFα, and Granzyme B, accompanied by reduced TMT size, in the co-cultures containing PIB-mCherry-transduced TMTs compared to those with mCherry-transduced TMTs. Importantly, both readouts were correlated with the percentage of reprogrammed tumor-cDC1s within the 3D TMT in a dose-dependent manner, suggesting that in situ cDC1 reprogramming promotes T cell activation and cytotoxicity.

**Conclusion**

In summary, we used 3D cocultures of human tumor, stromal, and immune cells in automation-compatible AKURA™ 384 well plates to demonstrate that cDC1 reprogramming progresses within TMTs, promoting antitumor immunity. Ultimately, these findings provide proof-of-principle for a novel cancer immunotherapy based on in situ cDC1 reprogramming and demonstrate the versatility of the AKURA™ platform for evaluating efficacy of novel anti-cancer therapies.

### EACR2024-0827

### Expanding the Landscape of Professional Project Management to Support Cancer Research in Serbia

*A. Djuric[1], M. Radulovic[1], A. Damjanovic[1], F. Remond[2], S. Castellvi-Bel[3], J. Zoidakis[4,5], M. Cavic[1]*

*[1]Institute for Oncology and Radiology of Serbia, Department of Experimental Oncology, Belgrade, Serbia*
*[2]The Netherlands Cancer Institute, Department of Pathology, Amsterdam, The Netherlands*
*[3]Fundació Clínic per la Recerca Biomèdica Institut d'Investigacions Biomèdiques August Pi iSunyer*

© 2024 The Authors. Molecular Oncology is published by John Wiley & Sons Ltd on behalf of Federation of European Biochemical Societies

*FRCB-IDIBAPS- Centro de Investigación Biomédica en Red de Enfermedades Hepáticas y Digestivas CIBERehd- Hospital Clínic- University of Barcelona, Gastroenterology Department, Barcelona, Spain*
[4]*Biomedical Research Foundation- Academy of Athens, Department of Biotechnology, Athens, Greece*
[5]*National and Kapodistrian University of Athens, Department of Biology, Athens, Greece*

**Introduction**

Research Management Offices (RMOs), separate organizational units within universities, research and healthcare centers, serve as advisory and supporting entities along the entire research process. Their purpose is to ensure and promote high-quality research thus contributing to excellence, sustainability and open science principles. This study aimed to analyze the advantages of the establishment and development of the first RMO at the Institute for Oncology and Radiology of Serbia (IORS) and to propose further R&D strategies for the efficient implementation of EU grants to ensure sustainability of research ecosystems in Serbia.

**Material and Methods**

Within the framework of the STEPUPIORS Horizon Europe project, the first RMO was established at IORS as a project delievarble. The initial steps were to define the scope of activities and to develop project management competencies through training, workshops and seminars. Human capacities for project management were built through intensive training and expert visits. The grant management offices from partner institutions provided focused training on pre-award and post-award processes with adequate on-site training in project management.

**Results and Discussions**

Good pre- and post-award grant management practices were established within the first 12 months of the project. The RMO organized 5 training events on omics analyses, project management and education capacities, reaching over 200 Serbian researchers from 7 institutions. A training module was established on the dedicated project website (https://www.stepupiors.eu/training-webinars/) following Open Science principles. The RMO coordinated the development of 2 newsletters and 1 patient brochure on genetic testing in hereditary colorectal cancer. Five manuscripts on colorectal cancer patient management were published with the deposition of 2 datasets. Seven new grant applications were submitted. One female project member enrolled in a Master's program for Management in the Health Care System. IORS guidelines for the management of international grants and depreciation of equipment were prepared.

**Conclusion**

The establishment of the IORS RMO induced a rise in cancer research excellence and professionalism in the implementation and coordination of their projects. The RMO will strive to support IORS staff in education, research and administration activities, with special reference to the digitalization of cancer research, open science and integrity and equity in the cancer research community in Serbia and the region.

### EACR2024-0893
### ctDNA profiling of HR+/HER2-low and HR+/HER2-0 metastatic breast cancer patients

*N. Dobrić[1], N. Dandachi[1,2], E.V. Klocker[1], C. Suppan[1], R. Graf[3], S. Hasenleithner[1], P.J. Jost[1], E. Heitzer[3,4], M. Balic[1]*
[1]*Division of Oncology, Department of Internal Medicine- Medical University of Graz, Graz, Austria*
[2]*Research Unit Epigenetic and Genetic Cancer Biomarkers- Medical University of Graz, Medical University of Graz, Graz, Austria*
[3]*Institute of Human Genetics, Diagnostic and Research Center for Molecular Biomedicine- Medical University of Graz, Graz, Austria*
[4]*Christian Doppler Laboratory for Liquid Biopsies for Early Detection of Cancer, Medical University of Graz, Graz, Austria*

**Introduction**

Despite the substantial progress in the treatment of hormone receptor-positive (HR+) breast cancer (BC), there is still a significant proportion of patients with lower benefit from endocrine-based treatments. A meaningful proportion of these patients have HER2-low disease. As a subset of HER2-negative BC, they have traditionally been thought not to benefit from anti-HER2 therapies. However, recent studies involving HER2-low advanced BC patients have shown significant clinical benefits from HER2-targeted antibody-drug conjugates (ADCs) just based on the low expression of the HER2 protein. We performed a circulating tumor DNA (ctDNA)-based analysis of tumor fraction and mutational profiles to potentially identify different clinical behavior and therapeutic targets within these two groups.

**Material and Methods**

113 plasma samples from 103 metastatic breast cancer patients (HR+/HER2-low, n=76; HR+/HER2-0, n=37) were collected either before starting 1$^{st}$ line or 2$^{nd}$ line treatment. Tumor fractions were assessed using an untargeted aneuploidy screening and expressed as z-scores (mFAST-SeqS). The mutational landscape of ctDNA was established using a 77-gene panel (AVENIO ctDNA Expanded). Tumor fractions, the number of somatic variants and variant allele frequencies (VAF) were compared between HER2-low and HER2-0 patients.

**Results and Discussions**

HER2-low patients had significantly higher z-scores compared to HER2-0 patients (median 2.96 vs. 1.58, rank-sum p-value 0.023). In contrast, neither the highest nor the average VAF differed significantly between the two groups. While both groups presented with a median of 3 detected variants (HER2-low range: 1-20, HER2-0 range: 1-12), a significant difference was observed in the number of clonal variants between HER2-low and HER2-0 patients (median 2 vs. 3, rank-sum p-value 0.035). In contrast to previous reports, *PIK3CA* mutations were more prevalent in HER2-0 patients (58.06%) compared to HER2-low patients (40%), whereas *TP53* mutations were identified with 32.26% in HER2-0 and 26.67% in HER2-low patients.

**Conclusion**

Our results suggest a significant difference in the tumor fractions in plasma between HER2-0 and HER2-low in our patient cohort. Additionally, HER2-0 patients had a

© 2024 The Authors. Molecular Oncology is published by John Wiley & Sons Ltd on behalf of Federation of European Biochemical Societies significantly higher number of detected clonal variants than HER2-low patients. The mutational landscape revealed differences from previous reports, indicating that further investigations are needed to elucidate and establish the distinct features of HER2-low breast tumors.

### EACR2024-0929
### Translational biomarkers to stratify aggressive HNSCC patients in the clinics

*P. Parimelazhagan Santhi*[1], *H. Irjala*[2], *J. Westermarck*[3], *S. Ventelä*[4], *J. Rantala*[5]
[1]*University of Turku, Turku Bioscience, Turku, India*
[2]*University of Turku, Department of Otorhinolaryngology, Turku, Finland*
[3]*University of Tukru, Turku Bioscience, Turku, Finland*
[4]*University of Turku, Department of Othorinolaryngology, Turku, Finland*
[5]*Misvik Biology, Misvik Biology, Turku, Finland*

**Introduction**
A major clinical challenge in Head and Neck Squamous Cell Carcinoma (HNSCC) is lack of diagnostic methods to predict metastatic behavior of the cancer. The most effective solution to this challenge is the development of a biomarker that could identify aggressive, i.e. metastatic, cancer at an early stage. Based on our recent research LIM domain and actin binding 1 (LIMA1) could potentially be used as a marker to identify patients having increased risk of metastasis during the follow- up. More interestingly, LIMA1 exists in two different isoforms i.e. LIMA1 α and LIMA1 β whose role in cancer aggressiveness or metastasis remains completely unexplored. This is because these isoforms could so far be studied only with the Western blot method, which is not widely used in clinics and hospitals. In this study, we have developed custom-made LIMA1 isoform specific antibodies and tested these in an immunohistochemistry (IHC) setting in a population validated tissue microarray (PV- TMA).

**Material and Methods**
The custom made LIMA1 isoform specific antibodies were validated by siRNA silencing, western blots, confocal imaging. Then the antibodies were used in immunohistochemical staining of a population validated tissue microarray (PV- TMA) of (n= 685) HNSCC patient samples.

**Results and Discussions**
Our study revealed that one specific isoform of LIMA1 i.e. LIMA1-α is significantly higher expressed ($p< 0.001$) in patients having poor disease specific survival. The isoform specific immunohistochemistry is a significant tool to stratify the aggressive metastatic HNSCCs from non-metastatic counterparts and the findings could unhinderedly be translated to the clinical practice.

**Conclusion**
This biomarker-based approach to identify aggressive and metastatic cancers will strikingly improve the survival rate of the HNSCC patients and decrease cancer treatment toxicities, since most of the cancer treatments cause severe or even fatal complications.

### EACR2024-0940
### A new deep learning model that accurately predicts exome-wide gene expression from histopathology slides

*L. Gugel*[1], *O. Tirosh*[1], *Y. Kinar*[1], *A. Michalska-Falkowska*[2], *J. Reszec-Gielazyn*[2], *W. Bauer*[2], *M. Niemira*[3], *B. Temkin*[4], *R. Aharonov*[1], *G. Dinstag*[1]
[1]*Pangea Biomed, Research, Tel Aviv, Israel*
[2]*Medical University of Bialystok, Biobank, Bialystok, Poland*
[3]*Medical University of Bialystok, Genomics and Epigenomics Laboratory, Bialystok, Poland*
[4]*Pangea Biomed, Software, Tel Aviv, Israel*

**Introduction**
The use of tumor molecular profiling in clinical settings has enhanced cancer diagnostics and the delivery of precision oncology. Recently, methods for predicting gene expression directly from Haematoxylin-and-Eosin-stained (H&E) histology images have offered a new way to leverage the easily obtainable and cost-effective H&E images for precision oncology applications. We previously introduced such a method – DeepPT – and demonstrated how we can leverage its imputed gene expression for successful prediction of drug response, using our ENLIGHT platform.

**Material and Methods**
We present DeepExpress, a new method for expression prediction from H&E slides, based on an improved architecture, including multi-task learning and a feature space based on a self-supervised pre-trained deep network. We tested DeepExpress, DeepPT and other leading expression prediction methods on patient data from 17 cancer types in TCGA. In addition, we obtained a new dataset collected at the Medical University of Bialystok (UMB) consisting of matched slide images and mRNA expression from 151 cases representing 7 cancer types, which serves as an independent test set.

**Results and Discussions**
DeepExpress shows statistically significant improvement compared with the other methods in terms of median Pearson correlation between actual and predicted mRNA expression, among top predicted genes - the only metric available for all methods. Notably, DeepExpress significantly improves upon DeepPT, demonstrating up to a 3-fold increase in the number of well-predicted genes (genes with Pearson rho > 0.4) in 14 of the 17 cancer types tested. On the external test data from UMB, DeepExpress improves expression prediction in 6 of 7 tested cancer types, with up to a 7-fold increase in the number of well-predicted genes. Notably, immune genes are particularly well predicted: on a set of 826 hallmark immune genes, DeepExpress exhibits up to 3.5-fold and 2-fold increase in the percentage of well-predicted genes in the TCGA and UMB data, respectively, compared to other genes.

**Conclusion**
DeepExpress significantly improves upon competing methods for predicting mRNA expression from H&E slides across diverse cancer types. Overall, it well predicts the expression of 600 to 3,800 genes in the TCGA cohorts and 500 to 3,200 genes in the independent test cohort. The method's strong ability to predict the expression of immune genes suggests a potential benefit in predicting response to immunotherapy.

© 2024The Authors. Molecular Oncology is published by John Wiley & Sons Ltd on behalf of Federation of European Biochemical Societies

### EACR2024-0958
### Patient-derived organoids as a reliable screening platform for assessing ADC efficacy and specificity

*M Silva*[1], *A. Buijs*[1], *D. Mori*[1], *V. Adegbenro*[1], *F. Morales*[1], *R. Overmeer*[1], *S. Lukovac*[1], *S. F Boj*[1], *M. Derksen*[1]
[1]*HUB organoids, research and development, Utrecht, The Netherlands*

**Introduction**
Antibody-drug conjugates (ADCs) are a new class of pharmaceutical drugs designed to deliver cytotoxic agents to cells expressing specific antigens selectively. Based on Paul Ehrlich's "magic bullet" concept, ADCs are designed to target cancer cells, minimizing systemic toxicity typical of conventional chemotherapy. ADCs structurally consist of a particular monoclonal antibody linked to a cytotoxic agent. Patient-derived Organoids (PDOs) provide a new tool for studying cancer biology and patient stratification. PDOs are adult stem cell-based culture systems maintaining the patient-specific genetic and phenotypic characteristics, including surface marker expression. HUB Organoids has generated a large biobank of PDOs characterized on the genomic and transcriptomic level.
**Material and Methods**
Organoids were selected based on their target-antigen (TA) expression, assessed by flow cytometry and RPKM. Organoid killing upon ADC treatment was assessed using a ATP-based viability readout (CellTiter Glo) after six days of treatment.
**Results and Discussions**
PDOs with high and low expression of TA were selected. The results of viability screening show a correlation between TA-targeting ADC-induced organoid killing and TA expression in the organoids.
**Conclusion**
The work presented here shows the suitability of the organoid screening platform for assessing ADC efficacy. The results reveal that PDOs hold value for the preclinical development of ADCs and for evaluating their tumor specificity. Moreover, PDOs have the potential to predict patient specific response to ADCs.

### EACR2024-0953
### SCOT: A method for testing response biomarkers before interventional clinical data is available

*Y. Kinar*[1], *R. E. Bell*[1], *D. S. Ben-Zvi*[1], *E. Elis*[1], *E. Maimon*[1], *T. Beker*[1], *R. Aharonov*[1], *G. Dinstag*[1]
[1]*Pangea Biomed, Research, Tel Aviv, Israel*

**Introduction**
A key challenge in precision oncology is to develop predictive markers that identify patients likely to benefit from a given treatment, ideally before clinical trials are performed so that they can be effectively used in clinical trial design. Even when early development of such putative markers is possible, a challenge remains in testing their predictivity in the absence of clinical interventional data. Here, we introduce a method termed the Surrogate Clinical Outcome Test (SCOT), which assesses, using non-interventional data, whether a drug-specific biomarker will be beneficial in clinical settings.
**Material and Methods**
SCOT assumes that patients with low drug target(s) expression can serve as a surrogate for patients receiving the investigated drug, in the sense that in these patients, the dependency of survival on the biomarker score is similar to the dependency in patients who actually receive the drug. Hence, a good predictive marker will have a negative hazard ratio (HR) in patients with low target expression. To exclude markers that are merely prognostic, SCOT requires that the negative HR is not/less significant when the target expression is high. We tested SCOT on predictive biomarkers for 7 drug classes: BRAFi, HER2i, AKTi, Imatinib, ERi, Aromatase inhibitors and IAPi. For the first 6 we developed transcriptomic response biomarkers using our ENLIGHT algorithm, and for IAPi we used a previously published biomarker. We also tested SCOT on KI67 and Proliferation Index, known prognostic biomarkers.
**Results and Discussions**
Of the 7 treatment response biomarkers, SCOT classified 5 as predictive to the respective drug. 4 of the 5 were confirmed as predictive on interventional data (prediction AUC > 0.6). Of the 2 markers SCOT classified as non-predictive, 1 was confirmed as such on the interventional data. Overall, SCOT's classification was confirmed for 5 biomarkers (71% accuracy). Moreover, SCOT correctly classified KI67 and Proliferation index as prognostic, non-predictive biomarkers.
**Conclusion**
Our work presents a novel method to test predictive markers for virtually any targeted cancer drug using big non-interventional clinical data. A key feature of this method is that it offers an independent validation of predictive markers for drugs, based on human data, even before the drug enters clinical development. Combined with our ENLIGHT platform for generating predictive biomarkers in the absence of direct response data, this creates a powerful pipeline for early biomarker development and validation.

### EACR2024-0967
### Novel Amplification Assay Enables Multiplex Imaging of Low Abundance Targets in the Tumor Microenvironment (TME)

*J. Ziello*[1], *Y. Nossin*[2], *D. Papalegis*[1], *L. Vu*[1], *K. Goldman*[1], *J. Loebelenz*[1], *S. Sponaugle*[1], *S. Tkachev*[1], *S. Klein*[1]
[1]*Cell Signaling Technology, Cell Signaling Technology, Danvers, United States*
[2]*Cell Signaling Technology, Field Support Team, Leiden, The Netherlands*

**Introduction**
The rise of numerous immunotherapy biomarkers and their significance in the spatial context of the tumor microenvironment (TME) has led to a growing demand for dependable and precise multiplex immuno-histochemistry (mIHC) assays. Many existing mIHC technologies lack amplification capabilities, resulting in the failure to detect targets with low expression levels, or involve the deposition and cycling of antibodies,

© 2024 The Authors. Molecular Oncology is published by John Wiley & Sons Ltd on behalf of Federation of European Biochemical Societies potentially leading to epitope masking and degradation. SignalStar$^{TM}$ Multiplex Immunohistochemistry (mIHC) from Cell Signaling Technology presents a new method for labeling, amplifying, and visualizing up to 8 targets in formalin-fixed, paraffin-embedded carcinoma (FFPE) tissue sections without requiring fluorophore deposition or antibody cycling.

**Material and Methods**

In this investigation, the SignalStar mIHC assay was employed to simultaneously label 8 targets, including TIM-3, PD-1, PD-L1, LAG3, CD3, CD68, CD8, and Pan-Keratin. A network of complementary, fluorescently labeled oligonucleotides was utilized to amplify the signal of the first 4 antibodies before tissue imaging. Subsequently, the fluorescent signal was removed, and the process was repeated. The resulting two 4-plex images were computationally aligned using QuPath.

**Results and Discussions**

This approach was compared with the Tyramide Signal Amplification (TSA) deposition assay, as well as with indirect and direct immunofluorescent detection, in sequential sections. Each mIHC strategy was further juxtaposed with the conventional chromogenic IHC assay. The fluorescent signal from the 8-plex SignalStar assay was removed, and the same tissue was stained with fluorescent direct conjugates to achieve an even higher plex.

**Conclusion**

Our results demonstrate that the SignalStar assay detected similar percentages of cells compared to chromogenic and TSA staining. Conversely, the SignalStar assay often exhibited superior performance over indirect and direct immunofluorescent detection methods. In conclusion, our study underscores SignalStar mIHC as a valuable tool for characterizing and analyzing the complex TME. SignalStar mIHC surpasses existing technologies by eliminating the need for deposition to amplify the signal of multiple co-localizing targets, including low abundance proteins, while maintaining dynamic range.

### EACR2024-0968

### Constructing comprehensive single-cell spatial atlases across multiple FFPE human tissues using a universal 6,000-plex RNA panel

*R. van Eijsden[1], M. Patrick[2], S. He[2], D. Kroeppler[2], P. Danaher[3], T. My Van[1], J. Preciado[2], H. Zhai[2], J. Reeves[3], J. Beechem[2]*

*[1]NanoString Technologies, Customer Experience, Seattle, United States*

*[2]NanoString Technologies, Research & Development, Seattle, United States*

*[3]NanoString Technologies, Bioinformatics, Seattle, United States*

**Introduction**

A high-plex RNA panel that has high coverage and works universally on various tissue types allows researchers to study distinct molecular characteristics at spatial single-cell resolution. Single-cell spatially resolved tissue is crucial to uncover underlying organ development processes and disease mechanisms. We have developed a universal 6K Discovery RNA panel that covers broad biological areas of interest with special emphasis on oncology, immunology, and neuroscience. This panel generates a high number of transcripts-per-cell in intact formalin-fixed paraffin-embedded (FFPE) human tissue from many tissue types.

**Material and Methods**

In this study, we used the 6K Discovery RNA panel to characterize eight different FFPE tissue types, including brain, skin, lung, breast, liver, colon, pancreas, and kidney from humans. Four protein markers and DAPI were co-detected on the same tissue slide to identify the morphology in tissues as well as to improve the accuracy of cell segmentation. Also, tertiary analysis algorithms were developed for cell typing, co-localization of genes and ligands, cell-cell interaction, and pathway analysis.

**Results and Discussions**

Hundreds of millions of transcripts were simultaneously detected with a Spatial Molecular Imager (SMI) with high sensitivity and specificity on an FFPE tissue section with up to 1 $cm^2$ scan area. Thousands of genes were detected above the limit of detection (LOD) across each tissue, with single-cell and subcellular resolution. We also constructed the methods to investigate sample-specific spatial neighborhoods, defined by cell types, cell states, nearly a full-reactome set of biological pathways, and over 160,000 ligand-receptor pairwise interactions in each tissue type. Finally, we created the cell type and spatial neighborhood atlas of eight tissue types. Included in this dataset was squamous Cell carcinoma, for which we showed seven distinct cell populations, averaging 1656 counts per cell with seven distinct cancer cell populations.

**Conclusion**

Single-cell spatial measurements at 6,000-plex in a large viewing area on archival tissue with the CosMx$^{TM}$ SMI, coupled with comprehensive tertiary analysis workflow, enable researchers in every field to gain a global perspective of the spatial transcriptional landscape across multiple FFPE tissue types, enabling the next level of biological discovery and translational research.

### EACR2024-0970

### A transcriptome-based response predictor identifies potential responders among patients with negative standard markers for response to immune checkpoint blockers

*G. Dinstag[1], E. Elis[1], D. S. Ben-Zvi[1], A. Elia[2], K. Aldape[3], R. Eytan[4], E. Pikarsky[2], A. Popovtzer[5], J. Arnon[6], R. Aharonov[1]*

*[1]Pangea Biomed, Research, Tel Aviv, Israel*

*[2]Hadassah Hebrew University, Department of Pathology, Jerusalem, Israel*

*[3]National Cancer Institute, Laboratory of Pathology, Baethesda, United States*

*[4]National Cancer Institute, Cancer Data Science Laboratory, Bethesda, United States*

*[5]Hadassah Hebrew University, Medical Center, Jerusalem, Israel*

*[6]Hadassah Hebrew University, Oncology, Jerusalem,*

© 2024 The Authors. Molecular Oncology is published by John Wiley & Sons Ltd on behalf of Federation of European Biochemical Societies

*Israel*
**Introduction**
Immune checkpoint blockers (ICB) are revolutionizing cancer treatment, and are being approved for an increasingly wide range of cancer types. The most common biomarkers currently in use to select patients for ICB are PD-L1 expression on tumor cells, as measured by IHC, and tumor mutational burden (TMB) and microsatellite instability (MSI), both measured by NGS. However, some patients that are negative for these markers still respond to ICB. This calls for complementary biomarkers to better identify responders to ICB, especially in patients that are negative to current biomarkers. Here, we focus on predicting response to anti-PD1 in patients with negative PD-L1, TMB or MSI.
**Material and Methods**
We employ ENLIGHT, our transcriptome-based precision oncology platform, which identifies and utilizes clinically relevant genetic interactions to predict a patient's response to a wide range of targeted drugs, including ICB. ENLIGHT generates an individual ICB response score calculated from a 10-gene expression signature - the ENLIGHT Matching Score (EMS). Patients with EMS above a predetermined threshold are considered matched by ENLIGHT to anti-PD-1 treatments. We have previously shown, based on more than 1000 cases analyzed retrospectively, that this signature can identify responders to anti-PD-1 with high accuracy. Here, we use ENLIGHT to perform a retrospective analysis of 125 cases from three different datasets, who had low PD-L1 presentation (<1%), low TMB (< 10) or microsatellite stable tumors (MSS), and were treated with anti-PD-1, to specifically assess ENLIGHT's performance in this biomarker-negative sub-population.
**Results and Discussions**
Patients who responded to anti-PD-1 treatments had significantly higher EMS in all three datasets. Correspondingly, ENLIGHT is highly predictive of response to anti-PD-1 in patients with negative ICB markers in the three datasets (ROC AUCs of 0.80, 0.84, 0.77, respectively, for low PDL1, low TMB and MSS). It is important to note that ENLIGHT was not trained on any of these datasets, and is applied as-is using previously published parameters. Overall, we find that patients who are ENLIGHT-Matched to anti-PD1 are significantly more likely to respond than unmatched patients (odds ratio [OR] = 3.55; 95% confidence interval [CI] [1.38, 9.11]; p =0.004).
**Conclusion**
ENLIGHT is a powerful tool for predicting response to anti-PD-1 treatment in patients with negative standard biomarkers for ICB, a currently unmet need with considerable clinical importance.

### EACR2024-0972
### Comparative biological discoveries between colon cancer and diseased tissues using a novel spatial multiomic approach and a comprehensive Immuno-Oncology Proteome Atlas and Whole Transcriptome Atlas

*I. Yujnovsky[1], A. Rosenbloom[2], E. Piazza[3], B. Filanoski[2], E. Ignacio[4], N. Nelson[5], T. Theisen[6], M. Hoang[6], J. Beechem[7]*
*[1]NanoString Technologies, Marketing, Marseille, France*
*[2]NanoString Technologies, Research Proteomics, Seattle, United States*
*[3]NanoString Technologies, Bioinformatics, Seattle, United States*
*[4]NanoString Technologies, Process Development, Seattle, United States*
*[5]Abcam plc, Commercial Strategy, Cambridge, United Kingdom*
*[6]NanoString Technologies, Research Genomics, Seattle, United States*
*[7]NanoString Technologies, Research and Development, Seattle, United States*
**Introduction**
The advancement of spatially resolved, multiplex proteomic and transcriptomic technologies has redefined the approaches to complex biological questions related to tissue heterogeneity, tumor microenvironments, cellular interactions and diversity, and therapeutic response. While spatial transcriptomics has traditionally led the way in plex, multiple studies have demonstrated a poor correlation between RNA expression and protein abundance, owing to transcriptional and translational regulation, target turnover, and most critically, post-translational protein modifications. Therefore, a more holistic, ultra-high-plex, and high-throughput proteomic atlas approach becomes critical for the next phase of discovery biology.
**Material and Methods**
A Digital Spatial Profiler platform is uniquely suited to support high-plex proteomics, allowing for the simultaneous analyses of proteins from discrete regions of interest in FFPE tissue sections while preserving spatial context. The assay relies upon abcam antibodies coupled to photocleavable DNA barcodes readout with NGS sequencing, allowing for theoretically unlimited plex. We introduce the Human Immuno-Oncology Proteome Atlas (IPA), a 570+ antibody-based proteomic discovery panel, compatible with immunohistochemistry on FFPE tissues with NGS readout. IPA is the highest-plex, most comprehensive, antibody-based multiomic panel to date focusing on key areas of immuno-oncology, oncology, immunology, epigenetics, metabolism, cell death, and signaling pathway regulation.
**Results and Discussions**
We demonstrate the performance of IPA on various cell lines and tissue. We also show the power of IPA, using the spatial multiomic assay along with the GeoMx® Whole Transcriptome Atlas (> 18,000 transcripts), a 30-plex custom antibody panel and microbiome-curated RNA custom spike-in (~42 transcripts) to evaluate 70 different colon disease samples across 5 pathologies including adenocarcinoma, hyperplasia, and chronic inflammation. This is the highest-plex multiomic (~610-plex proteins and >18,042 genes) study ever implemented for spatial biology.
**Conclusion**
We observed an upregulation of specific pathways associated with tumorigenesis and inflammation. We also observed distinct differences in proteomic and transcriptomic landscape between pathologies. The

© 2024 The Authors. Molecular Oncology is published by John Wiley & Sons Ltd on behalf of Federation of European Biochemical Societies cutting-edge, data-driven, expert-curated IPA panel is at the forefront of spatial proteomics, empowering the researcher for the acceleration of biological discoveries. FOR RESEARCH USE ONLY. Not for use in diagnostic procedures.

### EACR2024-0992
### Zebrafish Avatars achieve over 90% accuracy in predicting patient response to therapy across CRC, Breast, and Ovarian Cancer: results from 3 clinical studies

*R. Fior[1], B. Costa[1], R.V. Mendes[1], M. Estrada[1], C. Rebelo de Almeida[1], M. Fontes[1], F. Amorim[1], L.M. Fernandez[2], F. Silva[3], F. Cardoso[4]*
*[1]Champalimaud Foundation, Champalimaud Research- Experimental Clinical Research, Lisbon, Portugal*
*[2]Champalimaud Foundation, Champalimaud Clinical Centre- Colorectal Surgery Department, Lisbon, Portugal*
*[3]Champalimaud Foundation, Champalimaud Clinical Centre- Gynecology Unit, Lisbon, Portugal*
*[4]Champalimaud Foundation, Breast Unit- Champalimaud Clinical Center, Lisbon, Portugal*

**Introduction**
In recent years, many developments in cancer research have led to an array of apparently equivalent treatment options. However, the efficacy of each treatment varies between patients and there is NO TEST for predicting which option will be the best. As a result, patients often go through rounds of trial-and-error to find the most effective treatment, suffering unnecessary side effects and losing valuable time.

**Material and Methods**
Our Lab has been developing the zebrafish-patient-derived-xenografts or zAvatars to screen anti-cancer therapies for personalized medicine. This assay relies on the injection of patient’s tumor cells into zebrafish embryos to generate the zAvatars, which then are treated with the different therapies. The final results are obtained within 10 days. With zAvatars it is possible to rapidly screen anti-cancer therapies, including chemo, radio- and targeted therapies, as well as evaluation of angiogenesis and metastasis and innate immune profiling.

**Results and Discussions**
Here, we present the results of 3 clinical studies aimed at determining the predictive value of the zAvatar model, in colorectal cancer (CRC), breast and ovarian cancer.
Our results in CRC shows that the zAvatar-test has a positive predictive value of 91% and a negative predictive value of 90% (N=55 patients). Importantly, patients with a sensitive zAvatar-test exhibited a longer progression-free-survival (PFS) compared to those with a resistant test. In ovarian cancer (N=23 patients) the zAvatar-test reached 90% accuracy and 100% in breast cancer (N=18 patients). We also analyzed the metastatic capacity of the patient tumor cells and show that their metastatic potential in zAvatars may have a significant prognostic value.

**Conclusion**
These promising results are leading us to the next step: a multicentric randomized clinical trial to evaluate the PFS and OS of patients treated according to zAvatar-based therapeutic decision versus those treated according to clinical-choice (standard of care). In summary our results show that zAvatars can be a groundbreaking model for personalised medicine to help tailor treatments for each individual patient.

### EACR2024-1023
### Transcriptional profiling of epithelioid sarcoma discloses biologic features and diagnostic biomarkers defining proximal and classic subtypes

*L. Sigalotti[1], A.M. Frezza[2], M. Sbaraglia[3], E. Del Savio[1], D. Baldazzi[1], I. De Benedictis[1], E. Bellan[3], S. Stacchiotti[4], A.P. Dei Tos[5], R. Maestro[1]*
*[1]Centro di Riferimento Oncologico I.R.C.C.S., Oncogenetics and Functional Oncogenomics, Aviano, Italy*
*[2]Fondazione IRCCS Istituto Nazionale Tumori, Department of Medical Oncology- ISG EpisObs study coordinator, Milan, Italy*
*[3]Azienda Ospedale-Università Padova, Department of Pathology, Padua, Italy*
*[4]Fondazione IRCCS Istituto Nazionale Tumori, Department of Medical Oncology, Milan, Italy*
*[5]University of Padua School of Medicine, Department of Medicine, Padua, Italy*

**Introduction**
Epithelioid sarcoma (ES) is an ultra-rare and poorly characterized mesenchymal tumor hallmarked by loss of SMARCB1/INI1 expression. ES is morphologically classified into 2 subtypes: classic ES (C-ES), usually located in distal anatomic regions, and proximal ES (P-ES), frequently arising in proximal anatomic sites. This distinction has clinical relevance since P-ES tend to have a more aggressive behavior. The lack of unequivocal diagnostic markers makes P-ES and C-ES distinction prone to inter- and intra-observer variability. To identify subtype-specific features and biomarkers, we transcriptionally profiled 24 primary, untreated ES (14 C-ES and 10 P-ES).

**Material and Methods**
Diagnosis was confirmed by centralized pathology review. RNA sequencing was performed on FFPE tumor sections with tumor cell fraction ≥ 60%. Molecular groups were identified by unsupervised analyses. Genes differentially expressed between P-ES and C-ES were obtained using DESeq2. Functional annotation was performed by over-representation analysis and gene set enrichment analysis. Selected differentially expressed genes were orthogonally validated by IHC.

**Results and Discussions**
Unsupervised analyses of the transcriptomes evidenced a net separation of P-ES and C-ES, indicating that histological subtyping do identify molecularly distinct tumor variants. Functional annotation of differentially expressed genes highlighted enrichment of mesenchymal and vascular-associated signatures in C-ES. In contrast, P-ES were marked by pathways related to proliferation and chromatin regulation. Immunohistochemistry confirmed the selective expression in C-ES of the vascular transcription factor SOX17 (100% specificity

© 2024 The Authors. Molecular Oncology is published by John Wiley & Sons Ltd on behalf of Federation of European Biochemical Societies and sensitivity) and of N-cadherin (100% specificity, 67% sensitivity), suggesting a possible association with the endothelial lineage. Conversely, the chromatin remodeling-associated transcription factor GATA3 was exclusively expressed in P-ES (IHC: 100% specificity, 69% sensitivity).

**Conclusion**

P-ES and C-ES show a significantly different transcriptional profile. SOX17, N-cadherin and GATA3 stand out as biomarkers for objective ES subtype diagnosis.

### EACR2024-1042

### Ovarian cancer Zebrafish Avatars for personalized multiple drug testing

*M. Falcão Estrada[1], F. Amorim[1], C. Rebelo de Almeida[1], F. Silva[2], J. Casanova[2], R. Canas-Marques[3], M. Castillo Martin[3], S. Ferreira[2], H. Nabais[2], R. Fior[1]*

*[1]Champalimaud Foundation, Cancer Development and Innate Immune Evasion, Lisbon, Portugal*

*[2]Champalimaud Foundation, Gynecology Unit, Lisbon, Portugal*

*[3]Champalimaud Foundation, Anatomical Pathology Service, Lisbon, Portugal*

**Introduction**

Ovarian Cancer is the 1st cause of death amongst gynecological cancers. Treatments are chosen with no evidence of the tumor's response to therapy, exposing patients to unnecessary toxicities. Until now, there is no test to assess the best option for each patient. To solve this unmet need we developed the Ovarin cancer zebrafish Avatar-test (zAvatars).

**Material and Methods**

Cells were isolated from ovarian cancer patients, after obtaining their informed consent. zAvatars from 23 patients were generated by injecting cells in the perivitelline space of 2 days post fertilization zebrafish embryos. zAvatars were challenged with the same therapy as their donor patient as well as second line therapeutic options, to evaluate drug sensitivity, in a non-interventional retrospective clinical study.

**Results and Discussions**

Our data shows that, in the majority of patients that were sensitive to treatment, their matching zAvatars were also sensitive; whereas in the majority of patients that were resistant their zAvatars also showed resistance to treatment. To calculate the predictive power of the zAvatar model, we plotted the results in a confusion matrix and obtained a positive predictive value of 93% and a negative predictive value of 89%, which corresponds to an overall correlation of 91.3%. Analysis of the metastatic capacity showed that tumor cells from patients with accumulation of malignant ascitic or pleural effusion fluid, had a higher metastatic potential, regardless of being isolated from the peritoneal wall or the fluid. We also observed differential patterns of micrometastases distribution: tumor cells from patients without fluids mostly metastasized to 3 spots - head, tail and CHT; whereas tumor cells from patients with fluids were able to form metastases in 5 spots - head, tail, CHT, eyes and gills. These results are in line with the literature that considers that patients with fluids have poorer prognosis despite having the same follow up and treatment as patients without fluid accumulation.

**Conclusion**

With this clinical study we were able to demonstrate that zAvatars predict the patient response to therapy, and thus can be used as a screening method to guide patient therapy. As a next step, we will perform a Multicentric Randomized Clinical Trial, to demonstrate the clinical benefit of using the zAvatar test as a screening tool to guide patient therapy, and thus improve patient survival and wellbeing.

*This research received support from Champalimaud Foundation and FCT (FCT-PTDC/MEC-ONC/31627/2017).*

### EACR2024-1102

### A blind retrospective analysis of a novel predictive marker of ICB response in NSCLC, calculated from histopathological slides through inferred transcriptomics

*O. Tirosh[1], G. Leon[1], G. Dinstag[1], Y. Kinar[1], E. Anna[2], M. Tabi[2], E. Pikarski[2], A. Popovtzer[2], R. Aharonov[1], J. Arnon[3]*

*[1]Pangea Biomed, Research, Tel Aviv, Israel*

*[2]Hadassah Hebrew University, Department of Pathology, Jerusalem, Israel*

*[3]Hadassah Hebrew University, Medical center, Jerusalem, Israel*

**Introduction**

PD-1 inhibitors are in the forefront of contemporary clinical oncology and have become an integral part of treatment of many malignancies, including non-small cell lung cancer (NSCLC). Nevertheless, tumor response to PD-1 inhibition varies widely. Commonly used predictive markers such as PD-L1 expression and tumor mutational burden (TMB) have limited predictive value, which calls for the development of practical and more accurate tests. We present results of a blind retrospective analysis of a novel predictive marker of response to PD-1 inhibition relying solely on Hematoxylin and Eosin (H&E) slides.

**Material and Methods**

We obtained high resolution H&E slides from tumor-tissue samples of 50 cases of metastatic NSCLC treated with first-line PD-1 inhibitors (baseline response rate = 68%). We retrospectively applied our ENLIGHT-DP pipeline to generate, in a blinded manner, an individual response score to PD-1 inhibition for each slide. ENLIGHT-DP is composed of two main steps: (i) predict mRNA expression directly from an H&E slide using our digital-pathology based algorithm; and (ii) use these values as input to ENLIGHT, our transcriptome-based precision oncology platform, which generates a score that predicts response to targeted and immune therapies. We then unblinded the clinical outcome (RECIST1.1), and evaluated ENLIGHT-DP's performance vs. standard markers.

**Results and Discussions**

ENLIGHT-DP's score is predictive of response with ROC AUC = 0.69 ($p = 0.01$, one-sided permutation test). Using a predefined threshold for binary classification of response derived from independent data, ENLIGHT-DP achieved 100% PPV and 44% sensitivity. In comparison, predicting response according to PD-L1 > 1% achieves

© 2024 The Authors. Molecular Oncology is published by John Wiley & Sons Ltd on behalf of Federation of European Biochemical Societies

68% PPV and 62% sensitivity, while PD-L1 > 50% achieves 65% PPV and 38% sensitivity, i.e, both thresholds exhibit no predictive power (PPV <= baseline response rate). Patients with high TMB (>10) had 82% PPV and 26% sensitivity. ENLIGHT-DP was particularly good at stratifying patients with PD-L1 < 1% (18 patients, ROC AUC = 0.8, p = 0.03).

**Conclusion**

ENLIGHT-DP demonstrates high predictive power for response to PD-1 inhibition in NSCLC relying solely on H&E slides, outperforming the commonly used PD-L1 and TMB markers. It is also able to identify responders within patients with PD-L1 < 1%, for whom PD-1 inhibition is usually considered ineffective. Importantly, our approach does not require training on prior treatment outcomes, and can therefore be generalized to drugs for which such data is unavailable or scarce.

### EACR2024-1173
### Defining the role of the Activator protein 1 (AP-1) transcription factor subunit FOSL2 (FRA2) in the molecular adaptation to hypoxia in colorectal cancer

R. Mahmoud[1]
[1]*University of Nottingham, Translational Medical Sciences- School of Medicine, Nottingham, UK*

**Introduction**

Colorectal cancer (CRC) is the third most common cancer worldwide. Hypoxia (low oxygen) is frequently (~45%) seen in these tumours where it mediates therapy resistance, increased metastasis, and worse patient outcome. Hypoxia stabilizes the transcription factors HIF1α and HIF2α which enable molecular adaptation to the hypoxic insult at a transcriptional level, this is in cooperation with additional transcription factors. We have recently identified FOSL2 (FRA2), a subunit of the Activator protein 1 (AP-1) transcription factor, as a mediator of hypoxic CRC cell viability using a lentiviral shRNA screen. Therefore, we aimed to investigate its role in CRC molecular adaptation to hypoxia.

**Material and Methods**

Our experiments were conducted in colorectal cell lines in normoxia (20%O2) and hypoxia (1%O2). The impact of FOSL2 on hypoxia cell phenotypes was investigated using a variety of cell and molecular biology approaches including CRISPR Cas9 technology, cell count, FACS, Trans-well invasion assay, wound healing assay and others.

**Results and Discussions**

Analysing publicly available patients' datasets we demonstrated increased FOSL2 expression in the advanced compared to early CRC tumour stages (*p< 0.001*). We also identified FOSL2 (RFS, *p<0.001*) as possible poor prognostic marker in CRCs and mediators of tumour invasion. Our western-blot data confirmed that FOSL2 expression and phosphorylation is upregulated by hypoxia in CRC cell-lines. To investigate FOSL2 phenotypic role we generated HCT116 inducible CRISPR-CAS9 knockouts. Our data showed that FOSL2 knockdown reduced HCT116 cell count and 2D colony formation ability in hypoxia. FOSL2 knockdown also reduced HCT116 3D spheroids volume and growth rate. Also, FOSL2 knockouts xenografts IHC staining showed reduction in the proliferation marker Ki67 expression compared to the controls. Moreover, FACS analysis showed that FOSL2 knockdown induces apoptosis in hypoxic HCT116 cells. This was confirmed by increased expression of cleaved caspase3 in FOSL2 KO in hypoxia. Furthermore, wound healing and transwell-invasion assays showed that FOSL2 knockdown reduces HCT116 cells ability to migrate and invade (*p<0.015, p<0.003*) in hypoxia.

**Conclusion**

Our study suggests that FOSL2 is a key mediator of the molecular adaptation to hypoxia in CRC tumours and is a possible therapeutic target for the treatment of hypoxic therapy-resistant colorectal tumours. Therefore, we next aim to reveal its activation mechanism in hypoxic CR tumours to identify suitable targeting therapies.

### EACR2024-1195
### Novel organoid models enable pre-clinical chemotherapy screening for Mucinous Ovarian Carcinoma

O. Craig[1,2], C. Salazar[1], S. Abdirahman[1], N. Rajadevan[1,2,3], J. Luu[4], P. Wojtowicz[3], O. McNally[2,3,5], C. Scott[2,5,6], K. Simpson[2,4], K. Gorringe[1,2]
[1]*Peter MacCallum Cancer Centre, Laboratory Research, Melbourne, Australia*
[2]*The University of Melbourne, Sir Peter MacCallum Department of Oncology, Melbourne, Australia*
[3]*The Royal Women's Hospital, Gynaeoncology, Melbourne, Australia*
[4]*Peter MacCallum Cancer Centre, The Victorian Centre for Functional Genomics, Melbourne, Australia*
[5]*Peter MacCallum Cancer Centre, Gynaeoncology, Melbourne, Australia*
[6]*Walter and Eliza Hall Institute of Medical Research, Clinical Translation Centre, Melbourne, Australia*

**Introduction**

Mucinous Ovarian Carcinoma (MOC) is a rare form of ovarian cancer that is histologically distinct from other subtypes. When diagnosed at late stage, prognosis is dire as patients typically respond poorly to standard ovarian cancer platinum-based chemotherapy regimens. There is currently very limited pre-clinical or clinical trial drug response data available for this disease, and consequently there is no clear clinical consensus on the best systemic therapy to offer these patients. Our lab has generated a unique inventory of MOC patient-derived organoids and aimed to use these to generate in vitro efficacy data for a range of commonly prescribed therapeutics.

**Material and Methods**

Fresh and frozen patient tumour samples were disrupted using a combination of mechanical and enzymatic methods. Resultant cells were seeded into Matrigel domes and cultured with a specific cocktail of growth factors to stimulate and support organoid growth. DNA and RNA were extracted from matched organoid and parent tumour tissue pairs for sequencing and immuno-histochemistry of key diagnostic MOC markers was performed. Characterised organoids were then expanded, digested to single cells and seeded in 384 well plates before being treated with a panel of 14 agents across a 10-point dose range. Responses were quantified using

© 2024 The Authors. Molecular Oncology is published by John Wiley & Sons Ltd on behalf of Federation of European Biochemical Societies

CellTiter-Glo assay, Hoechst staining and brightfield imaging.

**Results and Discussions**

Characterisation of organoids revealed these models successfully recapitulated MOC parent tumours. While there was some variation between organoid lines, overall, this cohort demonstrated poor response to platinum-based therapies, as expected based on clinical outcomes. Organoids demonstrated a surprising yet consistent response to the taxanes Paclitaxel and Docetaxel. Promisingly, several agents were effective across a wide range of organoid lines, most notably Gemcitabine, Doxorubicin, Mitomycin C and the Topoisomerase I inhibitors Irinotecan and Topotecan.

**Conclusion**

This is the first cohort of MOC organoid models tested against a wide range of chemotherapeutic agents, providing crucial drug efficacy data to guide clinical practice. Our results support clinical observations of platinum-resistance, and suggest alternative therapies may be more effective in these patients. Future work aims to explore combinations of these agents and replicate results in vivo using novel patient-derived xenograft models, as well as correlate organoid line drug sensitivity back to genetic/gene expression profiles to identify biomarkers of response.

### EACR2024-1210
### Systematic Review of Molecular Pathology Signatures for Predicting Response to Immunotherapy in Lung Cancer

_L.R. Rajmohan_[1], P. p[2], P. d[1]
*[1]ACS Medical College & Hospital, General Medicine, Chennai, India*
*[2]ACS Medical College & Hospital, Biochemistry, Chennai, India*

**Introduction**

Immunotherapy has revolutionized lung cancer treatment, but response rates vary widely among patients. Identifying molecular pathology signatures as predictive biomarkers is crucial for optimizing patient selection and treatment outcomes. This systematic review aims to evaluate the predictive value of molecular signatures in lung cancer immunotherapy.

**Material and Methods**

A comprehensive literature search was conducted in PubMed, Embase, and Scopus databases for studies investigating molecular markers predictive of immunotherapy response in lung cancer. Studies reporting on the correlation between molecular signatures and clinical outcomes in immunotherapy trials were included.

**Results and Discussions**

Numerous molecular pathology signatures, including tumor mutational burden (TMB), programmed death-ligand 1 (PD-L1) expression, and specific gene mutations (e.g., EGFR, KRAS), have been identified as predictive biomarkers for immunotherapy response in lung cancer. These signatures show varying degrees of correlation with clinical outcomes, such as overall survival, progression-free survival, and treatment response.

**Conclusion**

Molecular pathology signatures offer valuable insights into patient selection and treatment response in lung cancer immunotherapy. Integration of these signatures into clinical practice could enhance personalized treatment strategies and improve patient outcomes.

### EACR2024-1230
### Implementation of a pain neuroscience education program for patients with oncologic pain

_L.T. Ordoñez-Mora_[1], R. Guil[2], I.D. Rosero[3]
*[1]Universidad Santiago de Cali, Doctorado en Ciencias Aplicadas, Cali, Colombia*
*[2]Universidad de Cadiz, Psicology, Cadiz, Spain*
*[3]Universidad Santiago de Cali, Programa de fisioterapia, Cali, Colombia*

**Introduction**

Within the processes of cancer intervention, pain appears as one of the most difficult to manage and disabling symptoms. The modern neuroscience of pain has improved the understanding of pain, including the role of sensitization in the processes of pain intervention. In this regard, it has been recommended to use educational modalities; in this sense, neuroscience education (PNE) is an intervention that provides elements of the neuro-physiology of pain that are transferred to the patient from examples or metaphors. The aim of this study was to examine the efficacy of NPE on pain and biopsychosocial variables in comparison with conventional management.

**Material and Methods**

A single-blind controlled clinical trial (Trial registration number NCT05581784), with 82 adults with the presence of oncologic pain due to prostate cancer in men and uterus and breast cancer in women in stages III and IV, from the execution of a baseline assessment considering pain and biopsychosocial variables, an assignment to one of two groups was generated: one corresponding to education in pain neuroscience (PNE), where the participants will perform 1 weekly session until completing 9 educational sessions each with a duration of 30 minutes; the second group (control) will not receive the educational intervention; the two groups will have the elements of conventional management estimated by the treating physician. The post-intervention evaluation will be carried out after 10 weeks for both groups. Statistical processing will consider analysis by protocol and by intention to treat according to the variables evaluated.

**Results and Discussions**

This study is in the process of recruiting patients according to the sample inclusion criteria. A mean pain has been established with the collection of 66 patients, corresponding to 5.8 +/- 1.9 for the intervention group and 5.3 +/- 2 $p = 0.4$.

**Conclusion**

It is expected that the intervention based on education in the neuroscience of pain has greater effectiveness in pain modulation processes with respect to patients who did not receive the intervention, and it is expected that the educational content generated can be used in patients with these characteristics.

© 2024 The Authors. Molecular Oncology is published by John Wiley & Sons Ltd on behalf of Federation of European Biochemical Societies

### EACR2024-1245
### High detection rate of somatic low frequency variants from FFPE samples using a versatile custom enrichment library preparation assay

A. Babayan[1], A. Dabuet[2], W. Jordan[2], S. de Rozieres[2], L. Watson[2], M. Cuadras[2]
[1]*Illumina, Cancer Research Marketing Europe, Berlin, Germany*
[2]*Illumina, Research and Development, San Diego, United States*

**Introduction**
The molecular profiling of solid tumors by Targeted Next Generation Sequencing (NGS) is an emerging practice for standard of care in oncology. Unfortunately, the process of fixation and paraffin embedding tissue samples can damage and/or modify the gDNA making tumor genetic analysis challenging, especially for detecting low abundant somatic mutations. In this study the efficacy of Illumina cfDNA Prep with Enrichment kit to detect low frequency variants in FFPE DNA was evaluated. The assay is a novel and flexible custom enrichment library preparation method that uses Unique Molecular Identifiers (UMI) for error correction.
**Material and Methods**
gDNA from FFPE samples was fragmented in a Covaris LE220 Model. The library preparation consisted of end-repair of fragmented FFPE DNA, followed by ligation of adapters containing unique molecular identifiers (UMIs), addition of indexes by PCR, and enrichment of regions of interest with a single hybridization step. Custom panels of sizes 300 kb and 2000 kb that target cancer-related genes, and Illumina Exome 2.5 panel were used for enrichment. Libraries were prepared from 10, 20 and 40 ng of commercial FFPE Cell lines and FFPE human tissues. Libraries were sequenced on Illumina NextSeq™ 550 and NovaSeq™ 6000 sequencing systems. Alignment and variant calling were performed using DRAGEN™ Enrichment App on BaseSpace™ Sequence Hub.
**Results and Discussions**
The high and mid quality FFPE samples achieved >70% read enrichment and a high level of mean target coverage depth (>100x), indicating high conversion efficiency from 10 ng input. The mean target coverage decreased with poor FFPE sample quality but was rescued with deeper sequencing and/or 40 ng input. Single nucleotide variants (SNVs) at 1%-2%, and Indels at 2%-3% variant allele frequency (VAF) were detected at ~90% sensitivity with custom and Exome 2.5 enrichment panels.
**Conclusion**
These results demonstrate the performance of Illumina cfDNA Prep with Enrichment in preparing sequencing-ready libraries from FFPE DNA that produce accurate data for downstream analyses, including somatic variant calling from as little as 10 ng input. The assay is a versatile library preparation kit compatible with user-supplied enrichment panels allowing researchers to tailor their experimental design based on their research needs.

### Tumour Biology

### EACR2024-0028
### The novel long intergenic non-coding RNA-RFC4 regulates chromosomal instability in cancer

R. Montiel-Manriquez[1], L.A. Herrera[2], C. Arriaga-Canon[1], L. Contreras-Espinosa[1], E. Fabian-Morales[3]
[1]*Instituto Nacional de Cancerología INCan-Instituto de Investigaciones Biomédicas- Universidad Nacional Autónoma de México UNAM, Unidad de Investigación Biomédica en Cáncer, Ciudad de México, Mexico*
[2]*Tecnológico de Monterrey, Escuela de Medicina y Ciencias de la Salud, Ciudad de México, Mexico*
[3]*Instituto Nacional de Cancerología INCan- Red de Apoyo a la Investigación RAI- Universidad Nacional Autónoma de México UNAM, Unidad de Aplicaciones Avanzadas en Microscopía ADMiRA, Ciudad de México, Mexico*

**Introduction**
Chromosomal instability (CIN) is hallmark of cancer associated to progression and aggressiveness. Among the key factors controlling CIN, long non-coding RNAs (lncRNAs) have shown to play important roles in regulating networks that maintain genome stability, disturbances in their expression induce genome instability and CIN, nevertheless, characterization of these transcripts and the networks they control to regulate CIN is still poorly understood, therefore, it is needed to explore their molecular functions in CIN regulation.
**Material and Methods**
RNA-seq data from the prostate cell lines: PrEC (stable), and LNCaP (unstable), was analyzed to find novel lncRNAs associated with CIN. Afterwards, experimental validation of the bioinformatic results in PrEC and LNCaP was performed with RT-qPCR and western blot. For the functional experiments, lncRNA knockdown (KD) was performed in the cells using antisense oligonucleotides (ASOs) targeting the lncRNA of interest, followed by karyotyping to assess CIN in this model. Confirmation of the lncRNA KD was performed by qPCR.
**Results and Discussions**
After analyzing and comparing RNA-Seq data from PrEC and LNCaP, the novel lncRNA-RFC4 was discovered in chromosome 3. This nuclear-retained transcript is adjacent to the protein coding gene RFC4, a gene associated to CIN that participates in DNA repair and replication. RT-qPCR analysis showed that while the lncRNA is upregulated in PrEC, RFC4 is downregulated, the opposite pattern was observed in LNCaP, suggesting the lncRNA is a cis-repressor of RFC4. After lncRNA KD in the stable cell line PrEC, an increase in RFC4 expression was observed, suggesting the possible role of the lncRNA-RFC4 as a transcriptional repressor. Karyotype analysis showed that a significant increase in tetraploid cells was induced after lncRNA-KD, suggesting this transcript as a regulator genome stability in this cellular model. To understand how the lncRNA regulates genome stability, RNA-Seq was performed in the KD experiments showing that lncRNA KD induced disruption of several pathways needed for proper DNA

© 2024 The Authors. Molecular Oncology is published by John Wiley & Sons Ltd on behalf of Federation of European Biochemical Societies replication and chromosome segregation, explaining CIN induction in PrEC.

**Conclusion**

Together our results indicate that the novel lncRNA-RFC4 could be a cis-repressor that maintains genome stability by regulating the adjacent coding gene RFCA and tran*s*-regulation of important pathways needed for proper DNA replication and repair in this cell model.

### EACR2024-0029
### HMGB1, a potential regulator of tumor microenvironment in KSHV-infected endothelial cells

*M.S. Lee[1], H.S. Kim[2]*
*[1]Eulji University, Department of Microbiology and Immunology, Daejeon, South Korea*
*[2]Inha University, Molecular Medicine, Incheon, South Korea*

**Introduction**

High-mobility group box 1 (HMGB1) is a protein that binds to DNA and participates in various cellular processes, including DNA repair, transcription, and inflammation. It is also associated with cancer progression and therapeutic resistance. Despite its known role in promoting tumor growth and immune evasion in the tumor microenvironment, the contribution of HMGB1 to the development of Kaposi's sarcoma (KS) is not well understood.

**Material and Methods**

We investigated the effect of HMGB1 on KS pathogenesis using immortalized human endothelial cells infected with Kaposi's sarcoma-associated human herpes virus (KSHV).

**Results and Discussions**

Our results showed that a higher amount of HMGB1 was detected in the supernatant of KSHV-infected cells compared to that of mock-infected cells, indicating that KSHV infection induced the secretion of HMGB1 in human endothelial cells. By generating HMGB1 knockout clones from immortalized human endothelial cells using CRISPR/Cas9, we elucidated the role of HMGB1 in KSHV-infected endothelial cells. Our findings indicate that the absence of HMGB1 did not induce lytic replication in KSHV-infected cells, but the cell viability of KSHV-infected cells was decreased in both 2D and 3D cultures. Through the antibody array for cytokines and growth factors, CXCL5, PDGF-AA, G-CSF, Emmprin, IL-17A, and VEGF were found to be suppressed in HMGB1 KO KSHV-infected cells compared to the KSHV-infected wild-type control. Mechanistically, phosphorylation of p38 would be associated with transcriptional regulation of CXCL5, PDGF-A and VEGF.

**Conclusion**

These observations suggest that HMGB1 may play a critical role in KS pathogenesis by regulating cytokine and growth factor secretion and emphasize its potential as a therapeutic target for KS by modulating the tumor microenvironment.

### EACR2024-0090
### Oxyresveratrol inhibits the tumorigenesis and metastasis of human hepatocellular carcinoma cells by targeting the CCDC19 signaling pathways

*H.L. Chiou[1], Y.H. Hsieh[2]*
*[1]School of Medical Laboratory and Biotechnology, Chung Shan Medical University, Taichung City, Taiwan*
*[2]Institute of Medicine, Chung Shan Medical University, Taichung City, Taiwan*

**Introduction**

Oxyresveratrol (OxyR), a natural polyphenolic phytoalexin commonly found in grape skins, mulberries, peanuts, and red wine, has been reported to possess multiple biological functions, including anti-cancer, antioxidant, anti-inflammatory, free radical scavenging, and neuroprotective properties. However, the role and molecular mechanism of OxyR on HCC remains unknown.

**Material and Methods**

Cell viability, colony formation, flow cytometry and in vitro migration and invasion analysis for cell growth rate, cell cycle distribution and metastatic abilities were employed to determine the effect of OxyR against HCC cells. RNA sequencing (RNA-Seq) assays was conducted to find out the differentially expressed pathways in HCC proliferation under OxyR treatment. The protein and mRNA expression levels of CCDC19 genes were verified through quantitative real-time PCR (qRT-PCR) and western blotting. However, CCDC19 expression and HCC survival analysis were conducted using data from the Cancer Genome Atlas (TCGA)-Liver Hepatocellular Cancer (LIHC) dataset and Kaplan-Meier plot. A small interfering RNA (siRNA) technique was used to silence CCDC19 expression. CCDC19 knockdown was employed to confirm the antitumor and antimetastatic role of CCDC19 in the OxyR treatment HCC cells.

**Results and Discussions**

Our results displayed that the treatment of OxyR inhibit the cell proliferation, migration and invasion of HCC cells. We also found that OxyR treatment decreased CCDC19 expression in HCC cells. We further revealed that CCDC19 exhibited a significant upregulation in HCC tissues and HCC cells, which is associated with tumor stage and poor patient survival. Silencing CCDC19 significantly inhibited OxyR-mediated inhibition of proliferation, migration and invasion.

**Conclusion**

Our study sheds light on the understanding of the anti-HCC effects of OxyR by inhibiting CCDC19 expression and could be a valuable anticancer agent for HCC.

### EACR2024-0101
### How relevant are second line histological features? Five-year survival after pancreatoduodenectomy for confirmed pancreatic cancer

*C. Jordan[1], J. Rees[1], T. Russell[2], P. Labib[2], S. Aroori[2], T. RAW Study Collaborators[3], V.K. Mavroeidis[1]*
*[1]Bristol Royal Infirmary, Department of HPB Surgery, Bristol, United Kingdom*
*[2]Derriford Hospital, Department of HPB Surgery, Plymouth, United Kingdom*
*[3]Multi-centre, Department of HPB Surgery, Global,*

Molecular Oncology 18 (Suppl. 1) (2024) 1–495
© 2024 The Authors. Molecular Oncology is published by John Wiley & Sons Ltd on behalf of Federation of European Biochemical Societies

*United Kingdom*

**Introduction**

Providing they have an appropriate performance status, patients with pancreatic ductal adenocarcinoma (PDAC) of the pancreatic head may be offered pancreatoduodenectomy (PD) with curative intent. Long-term survival is poor following this operation and advanced histological disease is known to correlate with adverse outcomes. Using a large multicentre cohort, this study aimed to investigate how peripancreatic fat invasion (PPFI), perineural invasion (PNI), microvascular invasion (MVI) and lymphatic invasion (LI) correlated with five-year survival.

**Material and Methods**

Data were extracted from the Recurrence After Whipple's (RAW) study, a multicentre retrospective cohort study of outcomes of PD performed for pancreatic head malignancy (29 centres from 8 countries, n=1484). Patients with histologically confirmed PDAC were identified and compared by their PPFI, PNI, MVI and LI statuses. Fisher's exact test was used to compare the groups.

**Results and Discussions**

A total of 885 patients (59.6%) had PDAC confirmed. Incidence rates of PPFI, PNI, MVI and LI were 72.3%, 87.0%, 58.7% and 68.0%, respectively (patients with data missing were excluded). Five-year survival was significantly shorter in patients with PPFI (20.0% vs 35.1%, p=0.0003), PNI (21.8% vs 38.5%, p=0.0009), MVI (19.8% vs 32.4%, p=0.0004) and LI (19.1% vs 37.9%, p<0.00001). Patients with PPFI (91.5% vs 63.8%), PNI (86.3% vs 60.7%), MVI (88.0% vs 71.3%) and LI (88.0% vs 71.3%) were all significantly more likely to have histological stage T3-4 (vs T1-2) disease (all p<0.00001).

**Conclusion**

In our multicentre study, the majority of PD patients with PDAC had histological evidence of PPFI, PNI, MVI and LI. All of these findings were associated with significantly reduced five-year survival. However, this is likely because these features correlated with more advanced histological disease. These findings could allow for more accurate prediction of five-year survival.

### EACR2024-0103

### Morphological characterization of colorectal cancer cell lines tumorospheres produced through different methodologies

*C. Mateos Sánchez*[1], *S. Alonso*[1], *B. González*[1]
[1]*Institute for Health Science Research Germans Trias i Pujol IGTP, Cancer Genetics and Epigenetics group, Badalona, Spain*

**Introduction**

Three-dimensional (3D) cultures of cancer cells provide a more physiologically relevant environment compared to traditional two-dimensional cultures for cancer research. They better mimic the complex microenvironment of tumors, including their morphology and spatial topography, the access to oxygen and nutrients, the dispersion of biological and chemical factors, cell–cell and cell–matrix interactions, and a more realistic extracellular matrix composition. Different methods and matrices have been developed to generate tumorspheres, resulting in distinct morphological characteristics. In this study, we have characterized the 3D morphology and proliferation of tumorspheres generated from a panel of commonly employed colorectal cancer (CRC) cell lines, using various methodologies and matrices with very different properties.

**Material and Methods**

Tumorspheres were generated from HCT116, LS174T, DLD1, SW48, SW480, SW620, HCT8, HT29, Caco2 CRC cell lines using several methods such as hanging-drop, liquid overlay, or low adherence culture plates, and different matrices: matrigel, methylcellulose, and collagen I. Morphological characteristics such as compaction, surface smoothness, and proliferation were assessed. Comparative analyses were performed to determine the influence of cell lines, methods, and matrices on the morphological features of CRC cell lines in 3D cultures.

**Results and Discussions**

Our findings reveal substantial differences in morphological characteristics of the CRC tumorspheres depending on the specific cell line, the method employed, and the matrix utilized. Some cell lines formed compact spheroids in all tested conditions, while others required particular conditions. The variations observed underscore the interplay between CRC cells, the in vitro culture methodologies, and matrix composition in shaping their 3D morphology.

**Conclusion**

The study shows significant variations in tumorosphere morphology based on the specific combination of cell line, method, and matrix. These results highlight the influence of the cell microenvironment on cellular morphology and underscore the importance of meticulous selection of experimental design in 3D studies. In summary, this research would help other researchers to optimize their selection of the most suitable combination of cell line, method, and matrix for 3D studies, facilitating more precise, reproducible and meaningful investigations in CRC research.

### EACR2024-0114

### Deciphering the dynamics of glioblastoma post-surgical microenvironment: insights into immune Responses, BBB disruption, and combinatorial therapies for recurrence inhibition

*C. Bastiancich*[1,2], *R. Stacchini*[1], *E. Snacel-Fazy*[1], *S. Robert*[3], *S. Fernandez*[4], *F. Debarbieux*[4,5], *B. Guillet*[4], *E. Tabouret*[1,6], *A. Tchoghandjian*[1,7]
[1]*Aix-Marseille University, Institute of Neuro-physiopathology - INP- UMR7051, Marseille, France*
[2]*University of Turin, Department of Drug Science and Technology, Turin, Italy*
[3]*Aix-Marseille University, Centre de Recherche Vasculaire de Marseille, Marseille, France*
[4]*Aix-Marseille University, Centre Européen de Recherche en Imagerie Médicale, Marseille, France*
[5]*Aix-Marseille University, Institute Neurosciences*

© 2024 The Authors. Molecular Oncology is published by John Wiley & Sons Ltd on behalf of Federation of European Biochemical Societies

*Timone, Marseille, France*
*[6]Assistance Publique - Hôpitaux de Marseille, Service de Neuro-Oncologie – Hôpital de la Timone, Marseille, France*
*[7]Aix-Marseille University, Réseau Préclinique et Translationnel de Recherche en Neurooncologie, PrEclinical and TRAnslational Network in neuro-oncology research- PETRA"TECH" platform, France*

**Introduction**

Glioblastoma (GBM) is an incurable primary brain tumor, necessitating standard surgical intervention followed by chemoradiation. Unfortunately, recurrences persist, leading to patient mortality [1]. Regenerative responses post-tumor debulking, while aiding healing, also induce immune reactions, fostering recurrence at resection cavity borders. Previous research demonstrated the efficacy of a nanomedicine hydrogel (GemC12-LNC) in delaying recurrence post-surgery. Combining it with an immunomodulatory drug to reverse anti-inflammatory responses is hypothesized to enhance therapeutic outcomes [2,3]. However, the post-surgical micro-environment (SMe) lacks proper characterization, hindering tailored combinatory therapy development. This study aims to examine surgery's impact on the brain and SMe, identifying time frames and therapeutic targets for combinatory approaches.

**Material and Methods**

Blood and magnetic resonance images of GBM patients pre- and post-surgery were analyzed to understand systemic immune response and blood-brain barrier (BBB) permeability changes after tumor debulking. A mouse model of tumor resection provided longitudinal SMe characterization through imaging and analytical techniques (multiparametric flow cytometry for immunophenotyping, brain clearing, and ultra-microscopy). Analysis of immune cell recruitment from the brain parenchyma or periphery was conducted from surgery to recurrence and tailored combinatory treatments were proposed.

**Results and Discussions**

Transient BBB disruption post-surgery, recovering within a week, provided a systemic treatment window. Differences in immune cell composition, morphology, and spatial localization between unresected and resected tumors were identified, emphasizing the overexpression of pro-tumoral macrophages, border-associated macrophages, and reactive microglia in resected tumors. Combining local GemC12-LNC with systemic SMAC-mimetic drug reversed this immune response, delaying post-surgical recurrence onset and increasing overall survival in GBM-bearing mice [4].

**Conclusion**

This comprehensive study delineates SMe time frames and immune cellular targets, paving the way for the design of a rational combinatory treatment to delay recurrence onset.

*[1] Hamard et al. J Neurooncol 128 (1) : 1-8 (2016)*
*[2] Bastiancich et al. J Control Release 264:45-54 (2017)*
*[3] Wang et al. Drug Del Transl Res (2023), accepted. DOI: 10.1007/s13346-023-01456-y*
*[4] Bastiancich et al. Preprint available at Research Square. DOI: https://doi.org/10.21203/rs.3.rs-3909300/v1*

### EACR2024-0159
### Arrays of 3D ECM-embedded fibroblast clusters for characterization of ECM remodeling by CAFs and for evaluation of drugs attenuating fibrotic ECM remodeling

*A. Liao[1], E. Danen[1]*
*[1]Leiden University, Leiden Academic Center for Drug Research, Leiden, The Netherlands*

**Introduction**

Chronic fibrosis can lead to organ failure and is also associated with cancer, where the tumor is interpreted as a chronic wound, and the fibrotic tumor environment drives disease progression. Fibrosis occurs in an inflammatory environment where macrophages are activated, and fibroblasts transdifferentiate into myofibroblasts. Activated macrophages stimulate the myofibroblasts to produce excessive amounts of collagen-rich ECM. Changes in proteolytic activity and high contractile forces that myofibroblasts apply onto their tissue environment, further lead to extensive remodeling of the healthy tissue matrix into a scar matrix. Similar activity is displayed by cancer-associated fibroblasts (CAFs) in tumors. Intervening with the excessive ECM remodeling by activated fibroblasts represents a candidate therapeutic strategy to normalize the architecture of fibrotic and malignant tissues.

**Material and Methods**

We developed a screening platform using an assay based on automated image guided injection of clusters of fibroblasts in wells of multi-well plates preloaded with a collagen-rich ECM network. The identical x-y-z position, spacing, and size of the ECM-embedded fibroblast clusters in each well facilitates automated real time confocal microscopy and quantitative image analysis algorithms.

**Results and Discussions**

We use this setup for automated quantitative analysis of ECM remodeling activity of CAFs obtained from early-stage colorectal cancer patients. We find that CAFs from these early-stage lesions display increased ECM remodeling capacity as compared to patient matched normal fibroblasts. We then use the same setup to screen a series of compounds for their ability to attenuate ECM remodeling triggered by TGFb-activated primary human fibroblasts. We detect known and novel pathways, and we identify small molecule inhibitors with a promising efficacy versus toxicity profile. Lastly, we detect a previously unknown side effect of ALK5 inhibitors that unexpectedly leads to activation of an alternative profibrotic pathway, which indicates that caution is warranted for the clinical application of ALK5 inhibitors.

**Conclusion**

A new screening platform using 3D ECM-embedded tumoroids provides quantitative data for toxicity versus efficacy of candidate anti-fibrotic drugs interfering with ECM remodeling capacity of fibrotic fibroblasts and CAFs. In addition, a novel profibrotic activity is revealed for ALK5 inhibitors that may limit their clinical implementation.

© 2024 The Authors. Molecular Oncology is published by John Wiley & Sons Ltd on behalf of Federation of European Biochemical Societies

### EACR2024-0188
### Interplay between miRNA and elements of innate immunity and their prognostic value in non-viral hepatocellular carcinoma

F. Ambrozkiewicz[1], M. Rajtmajerová[1], V.R. Mallela[1], E. Ali[1], A. Trailin[1], L. Červenková[2], R. Pálek[3], O. Daum[4], V. Liška[3], K. Hemminki[1]
[1]Faculty of Medicine in Pilsen- Charles University, Biomedical center- Laboratory of Translational Cancer Genomics, Plzeň, Czech Republic
[2]Faculty of Medicine in Pilsen- Charles University, Biomedical Center- Laboratory of Cancer Treatment and Tissue Regeneration, Plzeň, Czech Republic
[3]Charles University, Department of Surgery- University Hospital in Pilsen and Faculty of Medicine in Pilsen, Plzeň, Czech Republic
[4]Charles University, Sikl's Institute of Pathology- Faculty of Medicine and Teaching Hospital, Plzeň, Czech Republic

**Introduction**
Hepatocellular carcinoma (HCC) is the third deadliest cancer worldwide. Its high mortality is primarily attributed to late-stage diagnosis. The dysregulation of miRNA and their interaction with the tumor environment is poorly understood. As a novel approach, we wanted to assess the interplay between miRNA expression and the abundance of innate immune cells and their combined impact on patient outcomes.

**Material and Methods**
FFPE tissue samples were obtained from 45 non-viral HCC patients who had undergone resection and did not received neo-adjuvant treatment prior to the operation. Total RNA from paired tumor and non-tumor adjacent tissue was extracted. miRNA profiling was performed using Agilent microarrays. Differential expression analysis was performed in GeneSpring. After immuno-histochemical staining, the area fraction (AF) of CD68+ and CD163+ macrophages, CD1A+ dendritic cells, CD117+ mast cells, and NKp46+ NK cells were assessed in various regions of interest (tumor center (TC), invasive margin inner layer (IL) and outer layer (OL)). These variables were evaluated as predictors for time to recurrence (TTR), disease-free survival (DFS), and overall survival (OS) with Cox regression and Kaplan-Meier method.

**Results and Discussions**
We identified 23 differentially expressed miRNAs ($p \leq 0.05$, fold change $\geq 2$). High expression of miRNA-1972 in tumor was associated with longer TTR (HR: 0.35, p=0.039) and DFS (HR: 0.35, p=0.012). Among the analysed immune cells high AF of CD68+ macrophages in TC was associated with longer TTR (HR: 0.29, p=0.007) and DFS (HR: 0.45, p=0.027), similar associations were observed for IL (HR: 0.31, p=0.016 and HR: 0.36, p=0.009 respectively). High AF of CD117+ mast cells in TC were associated with longer TTR (HR: 0.35, p=0.048), and CD117+ mast cells in IL were associated with longer TTR (HR: 0.42, p=0.043) and DFS (HR: 0.33, p=0.009). A combination of high expression of miR-1972 and high AF of CD68+ macrophages in TC and IL were associated with longer TTR (HR: 0.09, p=0.001 and HR: 0.12, p=0.004) and DFS (HR: 0.14, p=0.001 and HR: 0.13, p=0.001). High AF of CD117+ cells in TC and IL combined with high expression of miR-1972 were associated with DFS (HR: 0.23, p=0.018 and HR: 0.20, p=0.004 respectively).

**Conclusion**
Combination of miRNA-1972 expression and AF of CD68+ and CD117+ cells refined their predictive values. The results show that factors in the tumor and its microenvironment may influence patient outcomes in HCC and their combination could be used for better therapy implementation.

### EACR2024-0190
### Chloroquine Suppresses Oral Carcinogenesis through Autophagy inhibition in 4NQO induced Oral Squamous Cell Carcinoma

M. BENBELKACEM[1], H. Chader[2], N. Moulai[3], W. Ouahioune[4], M. Bourouba[5]
[1]Laboratory of Cellular and Molecular Biology LBCM- Team Cytokines and Nitric oxide synthase. Immunity and pathogeny- Faculty of Biological Sciences, University of Sciences and Technology Houari Boumediene USTHB- Bab-Ezzouar, Algiers, Algeria
[2]Laboratory of Pharmaco-toxicology-, National agency of pharmaceutical products ANPP-, Algiers, Algeria
[3]2. Laboratory of Anatomopathology-, Frantz Fanon Hospital-, Blida, Algeria
[4]2. Laboratory of Anatomopathology, Frantz Fanon Hospital- Blida- Algeria., Algiers, Algeria
[5]Laboratory of Cellular and Molecular Biology LBCM- Team Cytokines and Nitric oxide synthase. Immunity and pathogeny- Faculty of Biological Sciences-, University of Sciences and Technology Houari Boumediene USTHB- Bab-Ezzouar, Algiers, Algeria

**Introduction**
Oral cavity is the most frequent primary site of squamous cell carcinoma of head and neck (SCCHN), and tongue is well known to be the most prevalent intraoral cancer. Tongue cancer has a significant morbidity and fatality rate. Treatment often fails for many high-risk patients, and 5-year survival is achieved in only 30-50% of cases. There is increasing evidence that autophagy plays a major role in the development of tongue cancers and the tumor resistance to therapy in established cancers. Chemotherapy- and metabolic stress-induced activation of the autophagic pathway reportedly contribute to the survival of formed tumors, thereby favoring resistance. In order to understand the importance of autophagy on Oral squamous cell carcinoma (OSCC) proliferation, we evaluated the ability of Chloroquine (CQ) to specifically induce a targeted inhibition of LC3B in presence of Arsenite (NaAsO2).

**Material and Methods**
8 weeks female BALB/c mice were chemically induced with 100 µg/ml of the 4-nitroquinoline N-oxide (4NQO) carcinogen or 16 weeks of water control, followed by up to 16 weeks of observation. Mice were euthanized and their tongues removed at 32 weeks post-treatement. The tongue tissues were cell cultured for 24h with $NaAsO_2$ in presence of CQ. The histology of the tissues was

© 2024The Authors. Molecular Oncology is published by John Wiley & Sons Ltd on behalf of Federation of European Biochemical Societies examined, and immunohistochemistry was performed for LC3B expression analysis.

**Results and Discussions**

The presence of malignancies was confirmed by histological analysis of the mice tongues induced with 4NQO. According to the immunohistochemical study of LC3B expression, exposure to $NaAsO_2$ induced autophagy, as evidenced by the 33.25% increase in LC3B tissular expression in comparison to the control. Surprisingly, adding CQ drastically reduced autophagy by 91.75% when LC3B expression was compared to control, which demonstrates chloroquine's potential to inhibit autophagy.

**Conclusion**

Taken together, our findings indicate that CQ inhibit autophagy in 4NQO induced OSCC tissues. Targeting autophagy in cancer using CQ would represent a major therapeutic target for chemosensitization and could provide new opportunities OSCC treatment.

### EACR2024-0203
### Tumor-associated glia drive the initiation of cutaneous squamous cell carcinoma

*K. Huynh[1], A. Salas-Bastos[1], S. Micheli[1], V. Cecconi[2], I. Kolm[3], M. Gwerder[1], R. Nakandalage[1], M. van den Broek[2], S. Stierli[1], L. Sommer[1]*
*[1]University of Zürich, Institute of Anatomy, Zürich, Switzerland*
*[2]University of Zürich, Institute of Experimental Immunology, Zürich, Switzerland*
*[3]University Hospital of Zürich, Department of Dermatology, Zürich, Switzerland*

**Introduction**

Cutaneous squamous cell carcinoma (cSCC), the second most common skin cancer, is a malignancy of epidermal keratinocytes and its prevalence is consistently increasing worldwide. The importance of the tumor microenvironment in cancer formation and progression is widely recognized. Correspondingly, tumor innervation is known to promote disease progression and has recently been acknowledged as a hallmark of cancer. Despite that all peripheral nerves are ensheated by glial cells, the pro-tumorigenic effects have mostly been attributed to axonal signaling, whilst the role of peripheral glia remains poorly understood.

**Material and Methods**

Histological examinations of human and murine cSCC revealed the presence of activated glial cells, termed tumor associated glia (TAG), in the cancer stroma. To investigate the contribution of TAGs to cSCC formation, we depleted them in a UV-induced cSCC mouse model. Moreover, multiplex arrays were performed to identify factors responsible for glia activation and corresponding receptors were depleted and the impact on glia activation and cSCC formation was investigated. To identify glia dependent pro-tumorigenic mechanisms, scRNAseq was performed in presence and absence of TAGs in premalignant murine lesions.

**Results and Discussions**

Here, we demonstrate that TAGs show features of embryonic Schwann cell precursors and are abundant in premalignant lesions and the stroma of established human and murine cSCC. TAGs are activated by the alarmin IL-33, and both impaired TAG activation and depletion of TAGs prevent cSCC formation in mice. scRNAseq revealed that TAGs are associated with epithelial-to-mesenchymal transition in keratinocytes, which promotes cancer cell invasiveness. Moreover, depleting TAGs decreased the number of cancer-associated fibroblasts and, consequently, the intercellular communication required for tumor initiation, identifying TAGs as key drivers of cSCC formation.

**Conclusion**

This study underscores the importance of the premalignant microenvironment for tumor initiation and might help to solve the long-standing conundrum that normal skin harbors a large number of keratinocytes with oncogenic mutations, but only rarely shows signs of malignant transformation. We propose that changes in the environment are necessary for actual tumorigenesis and that TAGs play a central role in this. Moreover, since all tissues in our body are innervated, it is very plausible that TAGs are also important for cancers of tissues other than the skin and, thus, of general relevance to the field.

### EACR2024-0217
### 3D Bioprinting of Personalised Brain Cancer Tumouroids

*P. Graber[1,2], E. Dolman[1], J. Skhinas[1], M. Tsoli[1], D. Ziegler[1,3], M. Kavallaris[1]*
*[1]Children's Cancer Institute, Lowy Cancer Research Centre- School of Clinical Medicine- Faculty of Medicine and Health- UNSW Sydney, Sydney- NSW, Australia*
*[2]Australian Centre for NanoMedicine, UNSW Sydney, Sydney- NSW, Australia*
*[3]Kids Cancer Centre, Sydney Children's Hospital, Sydney- NSW, Australia*

**Introduction**

Paediatric high-grade gliomas (pHGGs) represent a heterogeneous group of tumours that carry a bleak prognosis and account for the primary cause of death in children diagnosed with brain tumours. To effectively study and treat highly aggressive brain tumours like pHGGs, there is a need for suitable models that replicate the complex environment of a patient's tumour. Traditional brain tumour in vitro models, such as 2D or neurosphere models, fail to mimic the complex environment of a patient's tumour, while engraftment into mice is time-consuming and variable in success. Addressing this gap, we have developed 3D-bioprinted tumouroids using tunable hydrogels, to mimic the extracellular matrix of pHGGs. By incorporating biomimetic peptides and proteins, our models aim to mimic the tumour's extracellular environment, offering an advanced in vitro platform for studying pHGGs.

**Material and Methods**

We performed a matrisome gene expression analysis on 158 pHGG samples and 34 normal brain tissue samples. Based on this analysis, we developed hydrogel models containing cells derived from four pHGG patients, incorporating biomimetic peptides and proteins across nine different hydrogel conditions. The viability and growth of the cells within these hydrogels were evaluated using cell viability assays and live-cell imaging techniques.

**Results and Discussions**

Molecular Oncology 18 (Suppl. 1) (2024) 1–495
© 2024 The Authors. Molecular Oncology is published by John Wiley & Sons Ltd on behalf of Federation of European Biochemical Societies

Our bioinformatics analysis revealed significant overexpression of essential extracellular matrix components, including fibronectin, collagen IV, laminin, and hyaluronic acid, in pHGGs. We achieved high-throughput and high-viability printing of cells derived from pHGG patients in diverse hydrogel environments. The data indicate that the inclusion of specific extracellular matrix components influences the growth patterns and morphology of these tumors in vitro.

**Conclusion**

This study introduces a novel approach for expanding and investigating paediatric tumours using 3D-bioprinted models. These models mimic the tumour's extracellular matrix, enable 3D growth, and facilitate high-throughput analysis.

### EACR2024-0224

### Targeting the tumour microenvironment in pancreatic cancer

*S. Arora[1], M. Vail[1], A. Scott[1], P. Janes[1]*

*[1]Olivia Newton-John Cancer Research Institute, Tumour Targeting Program, Heidelberg, Australia*

**Introduction**

Pancreatic ductal adenocarcinoma (PDAC) is one of the most aggressive and lethal malignancies, with a five-year survival rate of less than 10%. The tumour micro-environment (TME) of PDAC is characterised by robust activation of stromal cells, resulting in extracellular matrix deposition by specialised, myofibroblast-like, cancer-associated fibroblasts (myCAFs). myCAFs are characterised by TGFβ signalling and associated with fibrotic ECM deposition, angiogenesis, increased tumour cell survival, immune suppression, and resistance to therapy [1]. We previously identified the cell guidance receptor EphA3 in the TME of diverse cancer types, and recently showed an important role in supporting tumour angiogenesis in a lung cancer mouse model [2, 3]. Since the TME is critical in PDAC, and EphA3 expression is associated with poor PDAC patient prognosis, we set out to investigate EphA3 as a potential therapeutic target.

**Material and Methods**

EphA3 expression was analysed using available single cell-RNA sequencing data from a K-Ras mutant mouse model of PDAC [4] and by immunofluorescence staining of mouse and human PDAC. shRNA knockdown of TME-expressed EphA3 was achieved using KPC (K-Ras/p53 mutant) tumour allografts in transgenic mice with doxycycline-induced expression of EphA3 shRNA {Vail, 2023 #5666}. An anti-EphA3 antibody-drug conjugate (ADC) was developed using the EphA3 antibody ifabotuzumab coupled with the DNA-binding toxin pyrrolobenzodiazepine (PBD).

**Results and Discussions**

Single-cell RNA sequencing data from K-Ras mutant mouse PDAC showed EphA3 expression in the TME, particularly in myCAFs. We confirmed its expression in CAFs in KPC mouse tumour allografts, and in human PDAC, by immunofluorescence staining of EphA3 and CAF markers. To determine if TME-expressed EphA3 supports tumour growth, we tested growth of KPC tumours in mice following induction of shRNA-mediated knockdown of EphA3 expression. Knockdown of EphA3 decreased CAFs and reduced growth of tumour allografts relative to control mice. This  indicates that EphA3 plays an important role in PDAC development, which we are further exploring. Lastly, we have also developed a novel anti-EphA3 ADC which caused significant inhibition of PDAC growth in mice.

**Conclusion**

The EphA3 receptor is expressed in CAF subtypes within the pancreatic TME and supports tumour growth. Use of our anti-EphA3 ADC has potential as a novel TME-targeted therapeutic approach.

### EACR2024-0228

### A comprehensive and scalable human liver-on-chip model for fibrosis drug discovery and gene delivery applications

*F. Bonanini[1], R. Dinkelberg[1], V. van Duinen[1], M. van Beek[2], M. Caro Torregrosa[1], D. Kurek[1], P. Vulto[3], K. Bircsak[1]*

*[1]Mimetas B.V, Model Development, oegstgeest, The Netherlands*

*[2]Mimetas B.V, Commercial, oegstgeest, The Netherlands*

*[3]Mimetas B.V, Management, oegstgeest, The Netherlands*

**Introduction**

Liver cancer, or hepatocellular carcinoma (HCC), is a formidable malignancy characterized by the uncontrolled growth of cells within the liver tissue. The silent progression of HCC complicates early detection, emphasizing the need for a deeper understanding of the liver tumor microenvironment, which plays a crucial role in disease development.

**Material and Methods**

The liver model presented here incorporates a wide array of relevant liver cell types, including hepatocytes, endothelial cells, stellate cells, and resident immune cells. This is complemented by a complete vascularization system, allowing for fluid flow across a micro vascular network that faithfully models the dimensions of liver sinusoids. All these elements are seamlessly integrated into an automated and high throughput platform known as the OrganoPlate®. To model the liver tumor microenvironment, hepatocellular carcinoma cells were integrated into healthy liver tissue model. The resulting model allowed for the observation of integrated cancer colonies, providing a more realistic platform for therapy development compared to monocultures. Simultaneously, the liver's particle clearance mechanisms were investigated using fluorescent imaging. Endothelial cell phagocytosis of E. coli particles and resident macrophages' internalization of dextran molecules were studied to explore hepatic delivery of macromolecules and nanoparticles via the systemic circulation.

**Results and Discussions**

The integration of hepatocellular carcinoma cells within healthy liver tissue revealed the formation of integrated cancer colonies, offering insights into the complex interactions within the liver tumor microenvironment. Fluorescent imaging showcased endothelial cell phagocytosis of E. coli particles and resident macrophages' internalization of fluorescent molecules. These findings elucidate the liver's particle clearance mechanisms, providing a foundation for understanding immunological processes and designing efficient drug delivery systems.

© 2024 The Authors. Molecular Oncology is published by John Wiley & Sons Ltd on behalf of Federation of European Biochemical Societies

**Conclusion**
Understanding the liver tumor microenvironment is critical for developing effective therapies for HCC. We believe this system holds the potential for a groundbreaking progress in liver modelling which, besides including functional hepatic cells, also reflects the cellular organization and interactions found within the liver lobule. In conjunction with its high throughput capability, this has the potential to revolutionize drug discovery and develop therapies for complex liver diseases.

### EACR2024-0230
### PBRM1 loss in clear cell renal cell carcinoma leads to a proangiogenic phenotype via increased CXCL5 secretion that can be selectively suppressed by CXCR2 inhibitor

*N.K. Tran[1,2,3], J. Linxweiler[4], S. Ng[5], P. Zeuschner[4], S. Rieck[6], M. Ritter[3], B.K. Fleischmann[6], K. Junker[4], M. Hölzel[1,2], N. Klümper[1,2,3]*
[1]*Institute of Experimental Oncology, University Hospital Bonn, Bonn, Germany*
[2]*Center for Integrated Oncology Cologne/Bonn, University Hospital Bonn, Bonn, Germany*
[3]*Department of Urology, University Hospital Bonn, Bonn, Germany*
[4]*Department of Urology and Pediatric Urology, Saarland University, Homburg/Saar, Germany*
[5]*Clinical Chemistry and Clinical Pharmacology, University Hospital Bonn, Bonn, Germany*
[6]*Institute of Physiology I- Life & Brain Center, Medical Faculty- University of Bonn, Bonn, Germany*

**Introduction**
Immune checkpoint blockades (ICBs) and anti-angiogenic tyrosine kinase inhibitors (TKI) have substantially contributed to improve the outcomes of metastatic clear cell renal cell carcinoma (ccRCC). However, there is still a lack of biomarkers to distinguish patients who would benefit from the aforementioned therapies. Mutations of polybromo-1 (PBRM1) occur in about one-third of ccRCC. It is well known that PBRM1-mutated tumors are highly vascularized. Nevertheless, anti-VEGF/TKIs often result in temporary tumor response, and ultimately tumor resistance. This project aims to dissect the molecular mechanism driving the proangiogenic phenotype of PBRM1-mutated ccRCC.
**Material and Methods**
We established CRISPR-Cas9 induced polyclonal PBRM1-knockouts in two ccRCC cell lines and investigated the effects of tumor-microenvironmental stimuli on PBRM1-loss versus control ccRCC cells using qPCR, bulk RNA sequencing and ELISA. Further, we queried The Cancer Genome Atlas Kidney Renal Clear Cell Carcinoma (TCGA-KIRC) dataset for angiogenic-chemokine gene signatures driven by PBRM1 mutations. Human umbilical vein endothelial cell (HUVEC) proliferation and sprouting capability as well as related-signalling pathways in the presence of ccRCC conditioned media were examined to access the association between PBRM1-deficiency and an enhanced tumor angiogenesis. Finally, in ovo xenograft assay were performed to evaluate therapeutic potential of CXCL5/CXCR2 signaling pathway disruption in ccRCC.
**Results and Discussions**
In an unbiased transcriptomic approach, we identified an elevated secretion of CXCL5 in PBRM1-loss cells. In accordance to this, our TCGA-KIRC in silico analysis revealed that PBRM1-mutant ccRCC exhibited enhanced CXCL1, CXCL2, and CXCL5 expression (all CXCR2 ligands). Exposing the cells to pro-inflammatory cytokines (IFNγ, TNFα, and IL-17A) leads to hyper-induction of CXCL5. Elevated growth and angiogenic sprouting was detected in HUVEC which were incubated with supernatant from PBRM-1 loss cells. Of note, enhanced proliferation and sprouting capacity of the supernatant of PBRM1-knockout ccRCC cell lines can be selectively suppressed by CXCR2 inhibitor and CXLC5 blocking antibody. Furthermore, the disruption of CXCL5/CXCR2 axis culminated in tumour regression in in ovo xenograft model.
**Conclusion**
CXCL5 was identified as one of the drivers of the pro-angiogenic phenotype of PBRM1-mutated tumors. Targeting CXCL5/CXCR2 axis is a promising approach for ccRCC treatment, specifically in PBRM1- defective ccRCC.

### EACR2024-0236
### Deciphering the Cancer-Associated Stromal Cell Phenotypes of Prostate Cancer

*E. Damisch[1], E. Brunner[1], G. Fotakis[2], Z. Trajanoski[2], A. Pircher[3], I. Heidegger-Pircher[1], S. Sopper[3], L. Nommensen[1], G. Schäfer[4], N. Sampson[1]*
[1]*Department of Urology, Medical University of Innsbruck, Innsbruck, Austria*
[2]*Institute of Bioinformatics, Medical University of Innsbruck, Innsbruck, Austria*
[3]*Department of Haematology and Oncology - Internal Medicine V, Medical University of Innsbruck, Innsbruck, Austria*
[4]*Department of Pathology and Neuropathology and Molecular Pathology, Medical University of Innsbruck, Innsbruck, Austria*

**Introduction**
Prostate cancer (PCa) remains a significant health concern, particularly the final and lethal castration-resistant stage. Recent research has increasingly recognized the crucial role of the tumor micro-environment (TME) in PCa progression and therapy resistance. Within the TME cancer-associated stromal cells (CASCs) exhibit remarkable functional diversity, with some promoting tumor growth and others acting as tumor suppressors. This study aimed to unravel this complexity and identify potential therapeutic avenues by comprehensively characterizing CASC heterogeneity in the PCa microenvironment.
**Material and Methods**
We used single-cell RNA sequencing (scRNAseq) on prostate cancer biopsy specimens to identify and characterize distinct stromal cell populations within the TME. Bioinformatic analysis of these scRNAseq clusters yielded prognostic gene signatures. Immuno-histochemistry (IHC) and in situ hybridization (ISH)

Molecular Oncology 18 (Suppl. 1) (2024) 1–495
© 2024 The Authors. Molecular Oncology is published by John Wiley & Sons Ltd on behalf of Federation of European Biochemical Societies techniques were then utilized to identifying these stromal cell phenotypes in patient tissues. Based on these insights, we created a stromal surface marker panel capable of distinguishing diverse CASC populations in both freshly digested biopsies and primary prostate fibroblasts via flowcytometry.

**Results and Discussions**

Our scRNAseq analysis identified distinct CASC clusters with prognostic gene signatures, validated using TCGA PCa data. IHC and ISH in patient tissues confirmed CASC phenotypes, offering spatial insights into benign versus high-grade PCa. We developed a flow cytometry panel and optimized tissue dissociation for stromal cell recovery from PCa biopsies while preserving surface markers. Coupled with extensive flow cytometry analysis, we delineated discrete CASC phenotypes reflective of patient samples' pathophysiological states. Applying this panel to primary human prostate fibroblast cultures from our CASC biobank identified three distinct phenotypes, aligning with our previous transcriptomic and functional results. Moving forward, we aim to isolate and characterize CASC phenotypes from our primary fibroblast biobank via FACS.

**Conclusion**

This comprehensive investigation not only unveils the remarkable heterogeneity of CASCs within the PCa TME, but also paves the way for their potential as therapeutic targets. The established model featuring primary CASCs offers a valuable platform to explore the complex interplay between these cells and PCa cells, ultimately contributing to the development of innovative therapeutic strategies against the lethal castration-resistant stage of PCa.

### EACR2024-0275
### Exosomal Circular RNA CircGANAB Promotes Pancreatic Ductal Adenocarcinoma Progression through Destabilizing Tumor Suppressive Long Non-coding RNAs

*C.H. Wong*[1], *U.K. Lou*[2], *J.J. Peña-Paladines*[1], *M.A. Hollingsworth*[3], *S.L. Chan*[4], *J.H.M. Tong*[5], *K.F. To*[5], *Y. Chen*[1]

*[1]The Chinese University of Hong Kong, School of Biomedical Sciences, ShaTin, Hong Kong-China*

*[2]The University of Hong Kong, School of Biomedical Sciences, Pokfulam, Hong Kong-China*

*[3]University of Nebraska, University of Nebraska Medical Center, Omaha- NE, United States*

*[4]The Chinese University of Hong Kong, Department of Clinical Oncology, Shatin, Hong Kong-China*

*[5]The Chinese University of Hong Kong, Department of Anatomical and Cellular Pathology, Shatin, Hong Kong-China*

**Introduction**

Circular RNAs (circRNAs) play important regulatory roles in many biological processes as well as disease progression. However, apart from functioning as microRNA sponges, emerging evidence suggested that circRNAs may participate in additional gene regulatory mechanisms. We previously identified the dysregulated circRNAs in pancreatic ductal adenocarcinoma (PDAC) through circRNAs-sequencing. Therefore, our current study aimed to uncover the critical roles and gene regulatory mechanisms of circRNAs in PDAC.

**Material and Methods**

CircGANAB expression was examined in PDAC cells, tumors, and serum exosomes. The oncogenic roles of circGANAB in PDAC growth, migration, and invasion were investigated using PDAC cell lines and mice xenograft models. Transcriptome profiling by RNA-sequencing after circGANAB knockdown in PDAC cells was performed to identify circGANAB-regulating genes. CircGANAB-specific pulldown assay was performed to identify circGANAB-interacting RNAs. RNA stability assay, RNA immunoprecipitation assay, and 3-dimensional structural modeling were performed to investigate the roles of circGANAB in regulating RNA stability.

**Results and Discussions**

CircGANAB was significantly upregulated in PDAC tumors with a positive correlation with tumor size and poor prognosis. Notably, circGANAB was consistently upregulated in PDAC serum exosomes, reflecting the cellular situation in PDAC primary tumors with good diagnostic potential. Gain-of-function and loss-of-function studies revealed that exosomal circGANAB created an oncogenic tumor microenvironment for promoting PDAC cell growth, migration, and invasion. Mechanistically, transcriptome profiling after circGANAB knockdown identified their important roles in many biological processes, including cell adhesion, cell-to-cell communications, and cell signaling pathways. RNA-sequencing after circRNA-pulldown identified the circGANAB-regulating genes under direct circGANAB-RNA interaction were mainly long non-coding RNAs (lncRNAs). Particularly, we found that circGANAB complexed with tumor suppressive lncRNAs GAS5, lncLDAH3, and TMEM51-AS1 for blocking the access of RNA stabilizer IGF2BP2 to lncRNAs, resulting in RNA degradation and gene downregulation.

**Conclusion**

The upregulated exosomal circGANAB may serve as a novel non-invasive diagnostic biomarker for PDAC. CircGANAB promotes PDAC progression through novel circRNA-lncRNA regulatory mechanism and may serve as a novel therapeutic target.

### EACR2024-0276
### Correlation Analysis of Serum Cytokines and Metabolites in Oral Cancer Patients with Lymph Node Metastasis

*K.C. Lai*[1,2], *C.H. Hsieh*[2]

*[1]Chang Gung University, Department of Physiology and Pharmacology, Taoyuan City, Taiwan*

*[2]New Taipei Municipal Tu-Cheng Hospital, Division of Hematology and Oncology, New Taipei City, Taiwan*

**Introduction**

Lymph node metastasis is a critical aspect of oral cancer progression, significantly impacting patient prognosis and treatment decisions. By specifically investigating its correlation with serum cytokines and metabolites, the study aims to shed light on the underlying mechanisms driving metastatic spread in oral cancer.

**Material and Methods**

© 2024 The Authors. Molecular Oncology is published by John Wiley & Sons Ltd on behalf of Federation of European Biochemical Societies

Sera from untreated oral cancer patients were collected for targeted metabolomics analysis and cytokine profiling. The correlation between cytokines and metabolites was determined using the Spearman correlation.

**Results and Discussions**

Among the evaluated cytokines, elevated serum levels of IL1A, IL-6, IL-9, IFNA2, G-CSF, and TNF were significantly associated with lymph node metastasis in oral cancer patients. Conversely, serum levels of IL-1RA and CCL5 were significantly reduced in oral cancer patients with lymph node metastasis. Additionally, serum levels of glycerol 3-phosphate, succinate, and arginine were significantly lower in oral cancer patients with lymph node metastasis. In oral patients with lymph node metastasis, glycerol 3-phosphate exhibited positive correlations with malate, glutamate, IL-25. Conversely, in oral patients with lymph node metastases, it displayed negative correlations with succinate, glutamine, IL-8, and CXCL10.

**Conclusion**

These findings underscore the potential of combined cytokine and metabolite analysis in elucidating the mechanisms underlying lymph node metastasis in oral cancer, offering novel insights for future research in oncology.

### EACR2024-0281
### Tackling Glioblastoma with melatonin-primed mesenchymal stromal cells: a synergistic anticancer strategy

*L. Olmedo Moreno[1], E. S. Norton[2], R. Bueno[1], C. Panadero Morón[1], J. M Sierra Párraga[1], H. Guerrero Cazares[2], V. Capilla Gonzalez[1]*

*[1]Andalusian Molecular Biology and Regenerative Medicine Center CABIMER-CSIC-US-UPO, Integrative Pathophysiology and Therapies, Sevilla, Spain*

*[2]Mayo Clinic, Neurosurgery, Jacksonville, FL, USA*

**Introduction**

Glioblastoma (GBM) is the most prevalent and aggressive primary brain tumor in adults without satisfactory treatment. During the last decade, mesenchymal stromal cells (MSCs) have been tested for cancer therapy, mostly as vehicle to deliver anticancer agents owing to their tumor tropism. However, the clinical efficacy of these therapies has been limited. Understanding MSC-cancer cell interaction is crucial for enhancing cancer strategies using MSCs. In this study, we explored MSCs' impact on GBM cell behavior using in vitro models. Furthermore, we assessed the synergistic anticancer efficacy of MSC therapy and melatonin (Mel) pre-treatment (Mel), a cytoprotective and oncostatic properties hormone.

**Material and Methods**

Patient-derived GBM cells, commercially available U87 cells, and primary cultured GBM1A cells were used in this study. The experimental groups were: GBM cells (GBM), GBM cells with MSCs (GBM+MSC) and GBM cells with MSCs pre-treated with 25 μM of Mel during 24 hours (GBM+$MSC^{Mel}$). Cell motility and morphology were analyzed using the 2D LiveCyte single-cell tracking system. Furthermore, GBM cell migration and invasion were evaluated in the presence of MSC or $MSC^{Mel}$ using 3D co-cultures and tumorsphere assays. Transcriptome changes in GBM cells exposed to MSC or $MSC^{Mel}$ were analyzed using RNA-sequencing (RNA-seq) and validated by immunological techniques.

**Results and Discussions**

We found that $MSC^{Mel}$ showed an improved capacity to reduce migration and invasion of GBM cells compared to untreated MSCs in 2D and 3D systems. Moreover, $MSC^{Mel}$ caused a decrease in GBM cell size. From the RNA-seq analysis, we identified 110 differentially-expressed genes (DEG) when comparing GBM+MSC vs. GBM+$MSC^{Mel}$ mostly related to cell Migration, Cytoskeletal and Extracellular matrix Remodeling which were selected to generate a gene signature designated *MCER*. *MCER* was employed to categorize The Cancer Genome Atlas program (TCGA) GBM patients using RNA-seq data, revealing two distinct clusters with variations in survival. Notably, patients in the *MCER*-like cluster demonstrated a higher survival compared to those in other clusters. Immunological techniques confirmed cytoskeleton changes in GBM co-cultured with MSC, including altered expression of vimentin and myosin markers.

**Conclusion**

This study shows that Mel enhances MSCs anticancer effects on GBM, offering valuable insights for progressing MSC-based therapies in clinical practice.

### EACR2024-0324
### Zebrafish tumor xenografts - revisiting an emerging tool in cancer research

*N. Rimmer[1], T. Zwimpfer[1], M. Núñez López[1], R. Coelho[1], F. Geissler[1], D. Zeller[1], F.C. Lombardo[1], M. Konantz[2], V. Heinzelmann-Schwarz[3], F. Jacob[1]*

*[1]University Hospital Basel and University of Basel, Department of Biomedicine- Ovarian Cancer Research, Basel, Switzerland*

*[2]University Hospital Basel and University of Basel, Department of Biomedicine- Allergy & Immunity, Basel, Switzerland*

*[3]University Hospital Basel and University of Basel, Hospital for Women- Gynecological Cancer Center, Basel, Switzerland*

**Introduction**

The zebrafish tumor xenograft model is an emerging in vivo technique in cancer research with characteristics setting it apart from mammalian models. However, there seems to be a lack of consensus regarding experimental approaches. Here, we review the current literature on zebrafish models to identify and experimentally verify methods applied to assess cancer cell growth and drug response.

**Material and Methods**

We conducted a comprehensive PubMed search on zebrafish tumor xenograft models and summarized information including cancer type, drug regimens applied, injection sites, information on readout techniques, data processing, and parameters essential for reliable performance. Apart from the literature research, we assessed toxicity of compounds and combinations. Additionally, we injected cells at 48 hpf into yolk and the duct of Cuvier and evaluated tumor engraftment using flow cytometry, live and confocal fluorescence imaging

© 2024 The Authors. Molecular Oncology is published by John Wiley & Sons Ltd on behalf of Federation of European Biochemical Societies together with image quantification systems. Besides fluorescence-based cell tracking, cancer cell proliferation and apoptosis was assessed by FUCCI and FLIP-GFP transgenic reporter systems, respectively.

**Results and Discussions**

Zebrafish tumor xenografts are broadly accepted among various cancer types (n >10) as identified in 122 original articles so far. Patient-derived xenografts were used in <10% of publications. Literature revealed cell tracker staining (42%) and transgenes (40%) for tracing cancer cells *in vivo* being equally employed. Yolk engraftment was reported in 42%, while 4% of the publications did not specify the injection site. In our experiments, we found similar tumor engraftment in fish injected with cells into the yolk sac or the duct of Cuvier (n=5 cancer cell lines). All compounds (n=5) tested revealed low toxicity to zebrafish embryos with olaparib being the most toxic at 120 μM. Nearly 60% of publications utilized only a single readout method for engrafted cancer cells. Our experiments demonstrate comparable efficacy of live cell imaging and flow cytometry visualizing cancer cells in vivo, enabling the assessment of different cell cycles and Caspase-3 mediated apoptosis with single-cell resolution (n=3).

**Conclusion**

Zebrafish tumor xenografts are consistently applied in basic cancer research, however, with variations in reporting minimal information required for reliable experimental performance and subsequent data. A combination of at least two methods to assess tumor growth in vivo is recommended.

### EACR2024-0331
### Novel insights into prostate cancer heterogeneity: the central role of PKA in osteopontin regulation

*P. Sanchis[123], A. Sabater[123], J. Bizzotto[123], P. Valacco[12], E. Vazquez[12], N. Navone[4], G. Gueron[12], N. Anselmino[4]*
*[1]Laboratorio de Inflamación y Cáncer- Departamento de Química Biológica- Facultad de Ciencias Exactas y Naturales- Universidad de Buenos Aires- Buenos Aires C1428EGA- Argentina*
*[2]CONICET-Universidad de Buenos Aires- Instituto de Química Biológica de la Facultad de Ciencias Exactas y Naturales IQUIBICEN- Buenos Aires C1428EGA- Argentina*
*[3]Universidad Argentina de la Empresa UADE- Instituto de Tecnología INTEC- Buenos Aires C1073AAO- Argentina., Department of Biological Chemistry, Buenos Aires, Argentina*
*[4]MD Anderson Cancer Center, Department of Genitourinary Medical Oncology, Houston- Texas, USA*

**Introduction**

Prostate cancer (PCa) is recognized by its heterogeneity of response to treatment, partially explained by its genomic background, but influenced by the microenvironment. In this sense, around 11% of PCa bone metastasis (BM) exhibit increased expression of Osteopontin (*SPP1*/OPN), associated with disease severity. Thus, identifying the molecular hubs driving *SPP1*/OPN induction in this subpopulation of patients is critical to improve disease management.

**Material and Methods**

We performed a comprehensive transcriptomics analysis (DEG, Ingenuity Pathway Analysis (IPA)) of publicly available patients' datasets (GSE74685, SU2C-PCF and Westbrooke et al), comparing PCa on different metastatic sites and/or treatment status. Using an indirect co-culture system (24h) to mimic the dialogue between PCa cells (PC3/C42B) and osteoblast precursors (MC3T3) in vitro, we integrated transcriptomics (RT-qPCR and RNAseq) and secretomic (ESI-MS/MS of conditioned media (CM)) data to dissect key players involved in the bi-directional crosstalk between PCa and bone cells. Protein Kinase A (PKA) pathway implication was evaluated by its induction (forskolin 1uM) or suppression (H89 10uM). Clinically relevant patient derived xenograft (PDX) models growing intrafemorally (i.f.) were used for in vivo validation.

**Results and Discussions**

Bioinformatics analysis using publicly available datasets revealed a sub-population of patients with BM displaying high *SPP1* expression levels. Consistently across different cohorts, *SPP1* induction was associated with an active PKA pathway. By functionalizing this response in vivo (BM derived PDXs models growing i.f).and in vitro (co-culture systems), we uncovered that bone released factors *Col1a1* and *Fn1* mediate the induction of *SPP1* expression in PCa cells by activating PKA, highlighting the key function of this kinase at the PCa-bone interface. Interestingly, results from experimental settings and clinical data suggest an implication of the androgen receptor (AR) as a modulator of the PKA/*SPP1* axis. Accordingly, when assessing patient longitudinal samples, we observed an induction of *SPP1* expression in a subpopulation of patients after enzalutamide exposure (AR signalling inhibitor), concomitantly with an active PKA signalling.

**Conclusion**

We uncovered PKA as a novel central hub of *SPP1*/OPN regulation in PCa in response to the bone micro-environment that could possibly be implicated in the heterogeneity of treatment response. These results underscore *SPP1*/OPN as a molecular tool to detect tumours with active PKA.

### EACR2024-0351
### Investigating the role of cancer-associated fibroblasts in castration-resistant prostate cancer progression

*F. Bonollo[1], D. Hanhart[1], P. Chouvardas[1], W. Cheng[1], N. Sampson[2], G. Thalmann[3], M. Kruithof-de Julio[1], S. Karkampouna[1]*
*[1]University of Bern, Department for BioMedical Research DBMR, Bern, Switzerland*
*[2]Medical University of Innsbruck, Department of Urology, Innsbruck, Austria*
*[3]University Hospital-Inselspital, Department of Urology, Bern, Switzerland*

**Introduction**

We aim to investigate the role of cancer-associated fibroblasts (CAFs) in the development of castration-resistant Prostate Cancer (CRPC PCa). To achieve this goal, we characterize the molecular profile of the stromal component of subcutaneous patient-derived xenograft

© 2024The Authors. Molecular Oncology is published by John Wiley & Sons Ltd on behalf of Federation of European Biochemical Societies

(PDX) models with different androgen sensitivity and we establish in vitro 3D co-cultures.

**Material and Methods**

The PNPCa (androgen-dependent soft tissue metastasis) and LAPC9 (androgen-independent bone metastasis) PDXs are used. RNA-seq is performed on the PNPCa, and LAPC9 stromal fraction at passage 0 (following tissue digestion and magnetic-associated cell sorting of tumor and stromal cells), and established 2D PDX-fibroblasts. PDX tumor and stromal cells (passage 0) are cultured in collagen drops in a growth-factor enriched medium to allow the formation of tumor organoids in direct co-culture with fibroblasts. PDX organoids are co-cultured with PDX fibroblasts (passage 2) in an indirect (transwell) setting, and their viability is assessed.

**Results and Discussions**

The stromal fraction of PNPCa and LAPC9 shows distinct gene expression profiles, with higher expression of CAF-related genes in LAPC9 compared to PNPCa stroma (*Col1a1, Col5a1, Col5a2, Tnc, Fap, Vim*). Higher expression of Tenascin C (Tnc) and Collagen Type-I (Col1a1) is confirmed through immunohistochemistry of PNPCa and LAPC9 tissues. In line with these findings, in collagen-based 3D co-cultures, LAPC9 fibroblasts show a typical myofibroblast phenotype and highly interact with tumor cells; while PNPCa fibroblasts are less elongated and localize adjacently to tumor cells. Early-passage PNPCa and LAPC9 fibroblasts cultured in 2D do not show major differences in gene expression. However, upon indirect co-culture, they promote the viability of PNPCa organoids at both dihydrotestosterone standard (1nM) and non-optimal (0.5nM, 0.25nM) concentration, compared to the monocultures.

**Conclusion**

PCa cells modify their surrounding microenvironment differently depending on the tumor stage, given that PNPCa and LAPC9 stromal cells, initially part of the mouse subcutaneous stroma, have a different gene expression profile, with the CRPC (LAPC9) stroma showing a more pro-tumorigenic phenotype. Our data suggest that collagen-based tumor-stroma 3D co-cultures, rather than fibroblast 2D cultures, are more likely to preserve these phenotypic differences. Nonetheless, 2D-cultured fibroblasts might secrete factors supporting tumor cell proliferation, at least in the initial androgen-sensitive stages (PNPCa).

### EACR2024-0355

### Semaphorin 6C-dependent regulation of viability and invasiveness of ovarian cancer cells

*C. Ruscetta[1], R. Mastrantonio[1], C. Palazzo[2], L. Tamagnone[1,2]*

*[1]Università Cattolica del Sacro Cuore, Department of Life Science and Public Health- Section of Histology and Embryology, Rome, Italy*

*[2]Fondazione Policlinico A.Gemelli IRCCS, Department of Life Science and Public Health- Section of Histology and Embryology, Rome, Italy*

**Introduction**

Ovarian cancer (OC) is the leading cause of cancer-related death in women. This is primarily due to its propensity to widely spread in the peritoneal cavity with metastatic lesions that are the major source of disease recurrence. Notably, OC metastatic dissemination implicates cancer cell adhesion and invasion of the mesothelial cell layer. Semaphorins, a large family of conserved extracellular signaling molecules, initially described as axon guidance cues, have later been implicated in the modulation of cancer cell growth, invasion and metastasis. In particular, our lab has previously demonstrated that different human cancer cells are dependent on Semaphorin 6C (Sema6C). The aim of this study was to elucidate the signaling mechanism of Sema6C in ovarian cancer cells and its potential relevance in mesothelial invasion and metastatic dissemination

**Material and Methods**

Ovarian cancer cell lines were transduced to knock-down Sema6C expression by siRNA and miRNA sequences, or conversely to force its overexpression. The invasive ability of ovarian cancer cells was assessed by an in-vitro setting that recapitulates the peritoneal lining, by establishing a monolayer of human normal mesothelial cells on extracellular matrix components.

**Results and Discussions**

MicroRNAs (miRNAs) are key regulators of oncogene and tumor suppressor gene expression in cancer. Here, we focused on the role of miR124-3p, a known tumor suppressor, putatively capable of regulating Sema6C levels. We found that miR124-3p expression in ovarian cancer cells indeed achieved Sema6C depletion, leading to decreased ERK phosphorylation, upregulation of cell cycle inhibitor proteins p21/p27, and dramatic cancer cell growth suppression, which furthermore prevented the engagement of a mesothelial layer. Similar results were achieved by the transient transfection of Sema6C-targeted siRNAs, while it was impossible to establish cell lines constitutively expressing shRNAs suppressing Sema6C transcripts. Conversely, Sema6C overexpression experiments demonstrated that this transmembrane semaphorin promotes the adhesion to extracellular matrix components and the invasive capacity of ovarian cancer cells, eliciting their migration across a mesothelial lining.

**Conclusion**

Our data are consistent with a fundamental requirement for Sema6C to support viability and growth of ovarian cancer cells, while elevated Sema6C levels were associated with higher invasiveness of the mesothelium.

### EACR2024-0367

### Targeting EDI3 in breast cancer reduces tumour growth and metastasis

*A. Glotzbach[1], S. Lüke[1], Ö. Demirci[2], B. Begher-Tibbe[1], J. Reinders[1], J.G. Hengstler[1], K. Edlund[1], R. Marchan[1]*

*[1]Leibniz Research Centre for Working Environment and Human Factors, Toxicology, Dortmund, Germany*

*[2]Dicle University, Biology, Diyarbakir, Turkiye*

**Introduction**

Metastasis remains a major problem for tumour therapy. We showed that high expression of the glycero-phosphodiesterase EDI3 (endometrial carcinoma differential 3, *GPCPD1*) was associated with metastasis in endometrial cancer. By hydrolysing glycero-phosphocholine (GPC) to choline and glycerol-3-phosphate, EDI3 is a key enzyme in choline and

Molecular Oncology 18 (Suppl. 1) (2024) 1–495

© 2024 The Authors. Molecular Oncology is published by John Wiley & Sons Ltd on behalf of Federation of European Biochemical Societies glycerophospholipid metabolism. Altered choline metabolism has been reported in several cancer types, including breast cancer, and recently, we showed that EDI3 expression is especially high in ER-HER2+ breast cancer. Furthermore, silencing EDI3 reduced viability in ER-HER2+ cell lines. In the present work, we established a doxycycline-inducible EDI3 knockdown in ER-HER2+ HCC1954 cells to investigate EDI3's role in metastasis-relevant processes in vitro and metastasis formation in vivo in this specific breast cancer subtype.

**Material and Methods**

Luciferase-expressing HCC1954 (HCC1954-luc) cells were transduced with lentiviral particles containing EDI3-targeting shRNA oligos under doxycycline control. The effect of silencing EDI3 on cell adhesion, proliferation, colony formation and anoikis was determined in vitro. Doxycycline-induced HCC1954-luc shEDI3 cells were injected into the peritoneum or tail vein of mice to generate peritoneal and lung metastases, respectively, or injected orthotopically into the mammary fat pad to generate primary breast tumours and subsequent lung metastasis. Metastasis formation was monitored by non-invasive bioluminescence imaging, and tumours analysed with MALDI imaging.

**Results and Discussions**

Inducibly silencing EDI3 led to reduced cell adhesion, proliferation, and colony formation, as well as increased susceptibility to anoikis compared to non-induced cells. Reduced luminescence, indicative of less metastases, was observed in the peritoneum and lung of mice upon injection of cells expressing less EDI3 into the peritoneum and tail vein, respectively. Furthermore, mice injected into the peritoneum with EDI3-silenced cells survived longer. These cells also produced slower growing orthotopic tumours and developed less lung metastases. Finally, MALDI imaging revealed that tumours produced from EDI3-silenced cells have altered metabolic profiles compared to tumours made from control cells, suggesting that EDI3-mediated metabolic changes may influence tumour growth and metastasis.

**Conclusion**

The reduction in metastasis observed after silencing EDI3 indicates its potential as a treatment target in metastasizing ER-HER2+ breast tumours.

### EACR2024-0372
### TERRA- Rig-I-like receptors (RLRs) mediated inflammation in a zebrafish glioblastoma model

*I. Pecchini*[1], *V. Bontempi*[1], *M.C. Mione*[1]
[1]*University of Trento, Cibio, Trento, Italy*

**Introduction**

Glioblastoma (GBM) is an aggressive brain tumor with rapid growth, infiltrative behavior, and treatment resistance. Despite being rare (15% of primary brain tumors), it is the most common and aggressive in adults, with poor survival rates (typically 12-15 months). Gaps in understanding GBM biogenesis and progression hinder effective therapeutic options. Tumor heterogeneity complicates treatment strategy development, with diverse cell populations, including abundant microglia, suggesting that inflammation plays an important role. The RNA sensing pathway, a crucial component of the innate immune system, employs cytosolic receptors like RIG-I, MDA5, and LGP2 to detect viral and endogenous RNAs. These receptors belong to the RIG-I-like RNA sensing pathway (RLR) and rely on downstream components, like MAVS, to activate interferon-mediated immune responses. Dysregulated non-coding RNAs (ncRNAs), often present in cancer, can impact RNA sensing, and immune responses. Our Zebrafish glioblastoma model employs the Alternative Lengthening of Telomere (ALT) pathway, similar to 15% of human GBMs, and is classified as ALT-positive. ALT tumors exhibit telomeric abnormalities and elevated expression of telomeric repeat-containing RNA (TERRA). In our model downregulation of TERRA with ASOs reduces microglia infiltration, suggesting TERRA's regulatory role in inflammation. Moreover, RNA-seq analysis indicates upregulation of the RLR pathway and inflammatory markers in the model.

**Material and Methods**

We studied TERRA-RLRs interaction in human cancer cell lines using an innovative Proximity Ligation Assay (PLA). To understand the role of RLRs in brain tumor development we knocked out members of the RLR pathway exploiting CRISPR-Cas9.

**Results and Discussions**

We found that in cancer cell lines TERRA interacts with MDA5, mainly in the cytoplasm, suggesting a downstream activation of the signal. Downregulation of MAVS or MDA5 in Crispant larvae reduces microglia infiltration in tumors, supporting the idea that this pathway is involved in the regulation of inflammation in glioblastoma. Besides microglia, KO of RLRs also reduced the number of proliferating cells in the brain.

**Conclusion**

Further analyses of our model are needed to clarify the role of RLRs and infiltrating microglia in brain tumor development and progression. At the same time, the ability of ncRNAs to activate the RLR pathway in cancer deserves further investigation, as it may be the missing link between cancer development and sterile inflammation.

### EACR2024-0376
### Extracellular vesicles derived from prostate cancer cells alter immune cell function and promote tumor cell progression in vivo

*A. Mazumdar*[1], *A. Mbemba-Nsungi*[1], *V. Baumgartner*[1], *S. Salemi*[1], *D. Eberli*[1]
[1]*University Hospital Zurich, Department of Urology, Zürich, Switzerland*

**Introduction**

Prostate cancer ranks as the second most prevalent malignancy among men worldwide. Notably, individuals who advance to metastatic stages face significantly poor prognoses. Therefore, comprehending the metastatic cascade and the alterations within the tumor micro-environment is crucial in combating the mortality associated with prostate cancer. In recent studies, tumor-derived extracellular vesicles (EVs), have been implicated in modulating the tumor microenvironment and promoting tumor cell progression across various

© 2024 The Authors. Molecular Oncology is published by John Wiley & Sons Ltd on behalf of Federation of European Biochemical Societies tumor models. The objective of this study is to delineate the intracellular communication between tumor cells and host cells within the prostate cancer microenvironment via EVs.

**Material and Methods**

We genetically manipulated C4-2B and PC3 cells to overexpress mEmerald tagged to the tetraspanin protein CD9 via lentiviral transduction. As CD9 is abundantly expressed on EVs, EVs secreted from these cells also exhibit fluorescence, enabling the real time visualization of EV secretion and uptake by host cells in vivo. These cells were then subcutaneously implanted in CD-1 nude mice until tumors grew to approximately 1000mm$^3$. Animals were then sacrificed and the primary tumor and distant organs were harvested for immune fluorescence staining and flow cytometry analysis.

**Results and Discussions**

Preliminary findings revealed that both C4-2B and PC3 tumor cells successfully establish subcutaneous tumors in all injected mice. Flow cytometry analysis unveiled positive mEmerald/GFP fluorescence signals in CD45+ immune cells and CD31+ endothelial cells within the tumor microenvironment, indicative of successful delivery of CD9-mEmerald-labeled EVs from tumor cells to these host cells. This finding represents a notable breakthrough, offering compelling evidence for active cross-communication between tumor cells and their microenvironment via in situ EV-mediated signaling. Furthermore, we extended our investigation to characterize the interaction between tumor-derived EVs and the monocytic cell line THP1 in vitro. Our findings illustrate that EVs induce the activation of THP1 cells and elevation of various cytokines and growth factors, which are recognized as pivotal contributors to tumor progression.

**Conclusion**

Collectively, our findings underscore the pivotal role of extracellular vesicles in orchestrating tumor cell progression. Additionally, our study contributes to improving our understanding of the mechanisms underlying cancer associated with EV-mediated communication in the tumor microenvironment.

### EACR2024-0379

### Mechanistic characterization of RRAS2 and KRAS mutations in mediastinal germ cell tumors

*U.S. Gilani[1], D. Feuersinger[1], L. Martins[1], R. Bung[1], C. Erkut[1], P. Chudasama[2], S. Sander[3], P. Horak[4], S. Fröhling[4], C. Scholl[1]*

*[1]German Cancer Research Center DKFZ and National Center for Tumor Diseases NCT Heidelberg, Applied Functional Genomics, Heidelberg, Germany*

*[2]German Cancer Research Center DKFZ, Precision Sarcoma Research, Heidelberg, Germany*

*[3]German Cancer Research Center DKFZ and National Center for Tumor Diseases NCT Heidelberg, Adaptive Immunity and Lymphoma, Heidelberg, Germany*

*[4]German Cancer Research Center DKFZ and National Center for Tumor Diseases NCT Heidelberg, Translational Medical Oncology, Heidelberg, Germany*

**Introduction**

Malignant germ cell tumors (GCTs) are commonly located in the gonads (mainly testes), but also occur at extragonadal sites with diverse histological subtypes. They originate from the embryonic precursors of germ cells, the primordial germ cells (PGCs). While patients with testicular GCTs have a high survival rate, mediastinal GCTs are associated with poor survival and their pathogenesis is poorly understood. In the precision oncology program DKFZ/NCT/DKTK MASTER, we found mutations in the small GTPase RRAS2 in four out of nine (44%) primary mediastinal GCTs co-occurring with KRAS mutations and yolk sac histology. The aim of our study is to elucidate the pathogenesis of extragonadal GCTs, with focus on the tissue-specific roles of RRAS2 and KRAS, their unusual co-occurrence and inter-dependencies, and to provide disease-representative cell and mouse models for translational studies.

**Material and Methods**

We generated immortalized epithelial and yolk sac cell lines, as well as different GCT cell lines, to express mutant RRAS2 and/or KRAS and studied their malignant characteristics in vitro and in xenotransplantations. We established embryonic stem cell lines (mESCs) from transgenic mice with inducible RRAS2/KRAS mutations, and examined the influence of these variants during differentiation of mESCs into PGCs in vitro. RRAS2 mutations were also activated in transgenic mice systemically to study their general oncogenicity.

**Results and Discussions**

Systemic expression of RRAS2 mutations in adult mice rapidly induced lethal lung and pancreatic tumors. Regarding their role in GCTs, we observed oncogenic transformation of immortalized cell lines expressing RRAS2 mutations, evidenced by MEK/ERK and PI3K/AKT pathway activation, increased proliferation and anchorage-independent growth, and a synergistic effect of RRAS2 and KRAS mutations on tumor formation with yolk sac cells but not epithelial cells. Also, invasiveness was enhanced in a GCT cell line, suggesting a role in disease progression. RRAS2 and KRAS mutations in mESCs resulted in aberrant differentiation towards primitive endoderm-like cells that were unable to differentiate into PGCs.

**Conclusion**

Our findings suggest that RRAS2 mutations are strong oncogenic drivers in vitro and in vivo, block differentiation into PGCs, the cell of origin of GCTs, and contribute to an aggressive phenotype when combined with oncogenic KRAS variants in the GCT context.

### EACR2024-0401

### Exosomes released by tumor cells mediate a new Neuropilin-dependent mechanism of endothelial cell regulation

*C. Palazzo[1], R. Mastrantonio[2], E. Testa[1], M.T. Viscomi[2], A. D'Alessio[2], C. Tamagnone[1,2]*

*[1]Fondazione Policlinico A.Gemelli IRCCS, Department of life science and public health- Section of Histology and Embryology, Rome, Italy*

*[2]Università Cattolica del Sacro Cuore, Department of life science and public health- Section of Histology and Embryology, Rome, Italy*

**Introduction**

© 2024 The Authors. Molecular Oncology is published by John Wiley & Sons Ltd on behalf of Federation of European Biochemical Societies

Extracellular vesicles (EVs) have emerged as critical regulators of cell-cell communication. Notably, endothelial cells (ECs) are prominently regulated by EVs released by neighboring cell types and especially by cancer cells, prompting the investigation of these vesicles' involvement in tumor angiogenesis and cancer progression. Our lab has previously studied the role of Neuropilin proteins (NRPs) in angiogenesis. Thus, the aim of this project was to elucidate the role of NRP1 carried by EVs for the regulation of endothelial cells.

**Material and Methods**

EVs were isolated from conditioned supernatants of diverse cancer cells using polyethylene glycol precipitation protocol. EV purity was confirmed by the presence of specific markers in western blot analysis, evaluating also the presence of NRP1. Further validation about the correct size of EVs was achieved through light scattering analysis. To asses the activity of cancer cell-derived exosomes in the regulation of ECs, wound healing and transwell assays were carried out. To understand the specific role of NRP1 carried by exosomes the protein was either silenced or overexpressed. The miRNome profile carried by exosomes was then investigated by next generation sequencing (NGS). miRNA-Scope in situ hybridization was used to assess the transfer of miRNA exosome cargo to target cells, and immunofluorescence analysis revealed expression regulation of targeted proteins.

**Results and Discussions**

We found for the first time that NRP1 is carried by tumor-derived exosomes. Moreover, NRP1 expression enables tumor exosomes to functionally regulate ECs in paracrine manner, leading to a significant increase in migration. Notably, EVs are known to carry microRNAs targeting pathways implicated in tumor angiogenesis. Indeed we found that NRP1 controls the loading of miRNA cargo in cancer cell-released exosomes, and focused our attention on miRNA 210-3p, which is primarily described as an onco-miR controlling ECs and tumor angiogenesis. The functional relevance of miRNA 210-3p transferred by exosomes was confirmed by evaluating one of its main targets, ephrin-A3.

**Conclusion**

In conclusion, our data demonstrate for the first time that NRP1-enriched exosomes can interact with and activate functional responses in ECs, correlating with NRP1-dependent regulation of miRNA exosomal cargo.

### EACR2024-0412

### Immunosuppression of key tumor infiltrating immune cell populations by fibroblast derived extracellular matrix using a 3D model, ex vivo model

*K. Baker[1], D. Hargbøl Madsen[1,2]*

*[1]National Centre for Cancer Immune Therapy, Oncology, Herlev, Denmark*

*[2]University of Copenhagen, Department of Immunology and Microbiology, Copenhagen, Denmark*

**Introduction**

Tissue architecture changes are common to solid tumors, which is exploited by cancer cells for immune evasion and survival. Emerging evidence indicates that there are inherent immunomodulatory features of the extracellular matrix (ECM) which may influence tumor micro-environment (TME) signaling. Targeting the ECM is increasingly investigated to exploit the efficacy of immune checkpoint blockade (ICB) therapies in solid tumors. To gain insight into ECM-derived immuno-modulation, cell derived matrices (CDMs) from murine derived cancer associated fibroblasts (CAFs) and normal fibroblasts (NFs), generated in either normoxic or hypoxic ex vivo conditions, were assessed for their capacities to influence key tumor infiltrating immune cell types.

**Material and Methods**

CDMs were generated from NFs and CAFs from C57BL/6J mice in normoxic and hypoxic conditions. Fibroblast RNA was isolated following 10 days of ECM deposition or alternatively ECMs were decellularized to produce CDMs and characterized for physical properties. Bone marrow derived monocytes (BMDMs) were differentiated on CDMs for 7 days followed by extraction of RNA from BMDMs and FACS analysis of cells. Transcriptomic analysis of NFs and CAFs +/- hypoxia were assessed by RNAseq. BMDMs differentiated on fibroblast CDM's were assessed by RNAseq and FACS for transcriptomic and phenotypic markers, respectively.

**Results and Discussions**

Transcriptomic analysis of fibroblasts showed very distinct transcriptomes whether cells were of CAF or NF origin, or whether these cells were cultured in normoxia or hypoxia. Many ECM-related genes were expressed differently, including Mature Collagen Synthesis genes, Proteoglycans and ECM targeting enzymes. BMDM's cultured on NF or CAF CDMs showed minor differences in transcriptome and phenotypic profiles, however, differentiation of BMDM's on hypoxic-generated CDM's showed significantly different transcriptomes and phenotypic profiles to other conditions. BMDM's differentiated on hypoxia-generated CAF CDM showed an increase in immunosuppressive genes such as PDL2, CCL22 and IL-10 when compared to the normoxic-generated CDM equivalent.

**Conclusion**

This work indicates that the ECM composition can play an influential role in the TME by directly influencing the transcriptome of key infiltrating immune populations and may contribute to tumor progression. These findings warrant studies investigating mechanisms and components of the ECM which may be targetable to reduce the immunomodulatory effects of this dynamic tumor architecture.

### EACR2024-0468

### Real-time monitoring of the tumor-infiltrating lymphocyte related to the tumor microenvironment through intravital microscopy

*H. Kim[1], J. Kim[1], H. Kwon[1], K. Akyldiz[1]*

*[1]IVIM Technology, Global Innovation Center, Seoul, South Korea*

**Introduction**

Recently, analysis of the types and functions of tumor microenvironments (TMEs) and tumor-infiltrating

© 2024The Authors. Molecular Oncology is published by John Wiley & Sons Ltd on behalf of Federation of European Biochemical Societies lymphocytes (TILs) has focused on anti-cancer drug mechanisms. These perspectives affect the development of various analysis methods for analyzing immune typing based on T cell composition and immune profiling surrounding tumor growth. According to these trends, anti-cancer remedies could be improved by precision in medical therapeutic parameters and immune therapy.

**Material and Methods**

To conduct the experiments, we used the dorsal skinfold chamber (DSC) apparatus to model the immune reaction point related to TMEs and TILs. Cancer cells were inoculated beside blood vessels as B16F10 melanoma cell lines using a DSC apparatus according to a cancer cell modeling protocol. The 4T-1 Breast cancer cell line also underwent the same parameter. Moreover, we used two cancer cell lines to examine if TIL and immune reaction points related to TME are restricted. To determine that effect on tumor growth and inhibition, we used a new precision technique called real-time visualizing and intravital microscopy to monitor TIL and immune responses related to TME.

**Results and Discussions**

Our study focused on monitoring the activation of natural killer cells, T cells, and monocyte/macrophage immune reactions by drug response within the dorsal skinfold chamber of the living mouse body. Monocyte/macrophage activation and dendritic cell activation infiltrated the tumor tissue during the initial immune reaction to tumor growth, which continued for ten days. T-cell activation upregulates the surrounding tumor blood vessels during the four-day cancer growth period. The activation of natural killer cells also increased the blood vessels and tissues surrounding the tumor during T cell activation. The TIL and TME modality changes surrounding tumor blood vessels were not restricted following the cancer cell type. Its results are also visualized through intravital microscopy imaging.

**Conclusion**

Intravital microscopy can visualize the real-time change of TIL and TME, which aligns with the findings of numerous researchers who have reported the significance of TIL and TME activations in cancer therapy. Furthermore, intravital microscopy real-time results indicate the ongoing immune response and TIL surrounding the tumor blood vessels and tissues. TIL and TME activation monitoring by intravital microscopy under real-time and living body conditions can reflect precision data for transitioning from preclinical to clinical phase experiments.

### EACR2024-0478
### Identifying Tumour Clonal Populations: CaTCH Technology for Discrimination of DTC and CTC in Breast Cancer

*M.P. Dizanzo*[1], *A. Moyano*[1], *F. Aboudou*[1], *C. Umkehrer*[2], *S.M. Cronin*[2], *A. Obenauf*[2], *M. Montagner*[1]
[1]*University of Padova, Department of Molecular Medicine, Padova, Italy*
[2]*Research Institute of Molecular Pathology IMP, Vienna BioCenter VBC, Vienna, Austria*

**Introduction**

Breast cancer is a highly heterogeneous disease and the most common malignancy in women worldwide. Conventional therapies are used, but drug resistance, relapse, and metastases remain unmet challenges. Disseminated tumour cells (DTCs) may be resistant to therapy and contribute to disease recurrence, after a period called metastatic dormancy. In breast cancer not all circulating tumour cells (CTCs) and DTCs are equally dangerous suggesting the existence of cellular subsets endowed with different metastatic ability. Identifying aggressive cells and preventing their metastatic outgrowth is a significant clinical challenge. This study aims to address dormant cells by employing a new lineage tracing technique to identify potentially harmful circulating tumour cells (CTCs) and DTC clones in breast cancer.

**Material and Methods**

We applied a technique called CRISPRa tracing of clones in heterogeneous cell populations (CaTCH) (Umkehrer C. et al. 2020), which combines precise clonal tracing of millions of cells with the ability to isolate specific clones alive at any time point from complex cellular populations. We used breast cancer cellular models with different disseminating potential: 4T07 and D2.0R cells to isolate CTC and DTC clones.

**Results and Discussions**

Barcoded breast cancer cell populations with different complexities were first sequenced to assess the representation of the barcodes. Barcoded cells were injected into wild-type and immunocompromised mice, and DTCs retrieved from lungs at different time points. Genomic DNA was purified from the isolated barcoded DTCs and is currently under analysis to identify enriched and depleted barcodes. This approach will enable us to isolate breast cancer cellular clones with different disseminating potentials using CaTCH technology.

**Conclusion**

In this study, we apply an innovative functional lineage tracing method to identify, isolate and characterise DTCs and CTCs with different metastatic potential. This will allow us to study dormant and relapsing breast cancer cells with higher resolution and identify new determinants of metastatic behaviour.

### EACR2024-0483
### Areca nut-induced M2 polarization and metabolic reprograming in macrophages promote malignant transformation in oral potentially malignant disorders

*Y.Y. Wang*[1]
[1]*Kaohsiung Medical University, School of Dentistry, Kaohsiung, Taiwan*

**Introduction**

Oral cancer is one of the leading cancers in south and southeast Asia, especially in the male population with the habits of chewing betel quid. The International Agency for Research on Cancer (IARC) concludes that betel quid and its major ingredient, areca nut, are risk factors in oral cancer development. Oral squamous cell carcinoma (OSCC), the most common malignancy of oral cavity, is always preceded by oral potentially malignant disorders (OPMD) and betel quid chewing is positively associated with the progression from OPMD to OSCC.

**Material and Methods**

© 2024 The Authors. Molecular Oncology is published by John Wiley & Sons Ltd on behalf of Federation of European Biochemical Societies

Clinical analysis for the association of macrophages with oral potentially malignant disorders and OSCC was carried out by immunohistochemical and immunofluorescent stainings. Phosphokinase array and cytokine assay were performed to determine the molecular mechanisms modulating macrophage profile in oral cancer development, followed by western blotting for confirmation. Transwell assay was performed to evaluate the effect of areca nut extract on cell migration ability. Finally, hamster model was applied to confirm the in vitro effect of areca nut extract in the buccal pouch of hamster determined by immunohistochemical and immunofluorescent staining.

**Results and Discussions**

We found that VCAM-1 secreted in the conditioned medium of ANE-treated THP-1 cells was able to promote malignant behaviors of human dysplastic oral keratinocytes (DOK), such as increase cell migration ability, altered expression of epithelial-to-mesenchymal transition markers, and morphology changes towards a mesenchymal phenotype. In clinical tissues, M2 macrophages was positively correlated with oral cancer progression. ANE induced M2 macrophage differentiation, CREB phosphorylation and VCAM-1 secretion, increased mitochondrial metabolism in THP-1 cells. Conditioned medium and VCAM-1 from ANE-treated THP-1 promoted migration and mesenchymal phenotypes in oral pre-cancer cells. In vivo study showed that ANE enhanced M2 polarization and related signaling pathway in oral buccal tissues of hamster.

**Conclusion**

Our study provides novel mechanisms for areca nut-induced oral carcinogenesis in vitro, in vivo and in clinical settings that areca nut, via promoting M2 macrophage differentiation and secretion of oncogenic cytokines to activate malignant transformation of oral premalignant cells.

### EACR2024-0489

### Cardiac cancer: mechanical cues from the heart

D. Lorizio[1], M. Chiesa[2], G. Ciucci[3], M. Pinamonti[4], R. Bussani[5], R. Vuerich[3], S. Zacchigna[1]

*[1]Centro Cardiologico Monzino, Unit of Cardio-Oncology, Milan, Italy*

*[2]Centro Cardiologico Monzino, Bioinformatics and Artificial Intelligence facility, Milan, Italy*

*[3]International Centre for Genetic Engineering and Biotechnology, Cardiovascular Biology Laboratory, Trieste, Italy*

*[4]Azienda Sanitaria Universitaria Giuliano Isontina, Pathological Anatomy and Histology, Trieste, Italy*

*[5]University of Trieste, Department of Medicine-Surgery and Health Sciences, Trieste, Italy*

**Introduction**

Cardiac cancer is a rare malignancy. Whereas the low incidence of primary tumors in the heart can be explained by the poor capacity of cardiomyocytes to proliferate, the dense vascularization and blood perfusion of the heart are not consistent with the low incidence of cardiac metastases. Our lab has demonstrated that the ectopic injection of tumor cells in the heart generates tumor masses that are smaller than those implanted in any other peripheral organ. The mechanisms that hamper tumor cell growth in the heart have not been elucidated so far. Here, we propose to study a few cases in which cancer cells can reach and form metastases in the heart to unveil the molecular mechanisms that normally prevent cardiac cancer.

**Material and Methods**

We used spatial transcriptomics to characterize the molecular profile of available cases of cardiac metastases. Tumor samples were collected from patients affected by tumors that gave rise to both cardiac and extra-cardiac metastasis. Engineered heart tissues (EHTs) were generated from primary cardiomyocytes with the inclusion of cancer cells of various origins. The system was modified by introducing two metal braces that allow the modulation of the mechanical load within the EHT.

**Results and Discussions**

Differential expression analysis between cardiac metastases and extra-cardiac tumors (primary tumors + extra-cardiac metastases) identified about 400 differentially expressed genes (DEGs). Unsupervised hierarchical clustering demonstrated a clear segregation of samples according to their anatomical location. Pathways with the highest enrichment score in cardiac metastases were related to mechanosensing and mechanotransduction and included histone demethylation, keratinization and cell-matrix interaction. This analysis suggests that mechanical forces in the heart may be responsible for controlling cancer cell proliferation. Consistent with this hypothesis, cancer cells proliferated less in overloaded EHTs, whereas increased proliferation was observed in unloaded EHTs. Silencing of Nesprin-2, a member of the LINC complex that mediates the mechanotransduction between cytoskeleton and nucleus, restored the proliferative capacity of cancer cells despite the high mechanical load in EHTs.

**Conclusion**

Collectively, these results shed light on the mechanisms that control cancer cell growth in the heart and point to mechanical stimulation as a key process that may be exploited for cancer therapy.

### EACR2024-0490

### Interaction of adipocytes and liver cancer cells in hepatocellular carcinoma

L. Richter[1,2], F. Hofheinz[1], S. Meister[1], M. Karl[3], N. Berndt[4,5,6], S. Hauser[1,2], J. Pietzsch[1,2]

*[1]Helmholtz-Zentrum Dresden-Rossendorf, Institute of Radiopharmaceutical Cancer Research, Dresden, Germany*

*[2]Technische Universität Dresden, Faculty of Chemistry and Food Chemistry, Dresden, Germany*

*[3]Technische Universität Dresden, Institute of Physiological Chemistry, Dresden, Germany*

*[4]German Institute of Human Nutrition Potsdam-Rehbruecke DIfE, Department of Molecular Toxicology, Nuthetal, Germany*

*[5]Charité – Universitätsmedizin Berlin, Institute of Biochemistry, Berlin, Germany*

*[6]Deutsches Herzzentrum der Charité, Institute of Computer-assisted Cardiovascular Medicine, Berlin, Germany*

**Introduction**

© 2024 The Authors. Molecular Oncology is published by John Wiley & Sons Ltd on behalf of Federation of European Biochemical Societies

Hepatocellular carcinoma (HCC), the most common form of liver cancer, is a heterogeneous tumour that is often associated with chronic liver disease. As a risk factor, obesity-related non-alcoholic steatohepatitis (NASH) is characterized by hepatic steatosis, inflammation and hepatocellular injury. Lack of surveillance and inadequate early diagnostic accuracy are attributed to the poor prognosis and high mortality of HCC. Therefore, an in vitro model will be developed to investigate the interaction between adipocytes and HCC cells and to identify molecular imaging biomarkers for early diagnosis.

**Material and Methods**

3T3-L1 mouse fibroblasts were differentiated into adipocyte like cells by treatment with 3-isobutyl-1-methylxanthine, dexamethasone, insulin and rosiglitazone. Adipocyte-conditioned medium (CMA) was collected, and free fatty acid concentration and adipokine levels were determined. Spheroids of HCC cell lines Hep3B and HepG2 (diameter 350 – 400 µm) were treated with CMA for 7 days. Spheroids treated with oleic and palmitic acid (2 and 200 µM), 3T3-L1 fibroblast conditioned medium (CMF) and cell culture medium served as controls. The treated spheroids were monitored for spheroid morphology and volume, and samples were collected for proteomics analysis.

**Results and Discussions**

During adipocyte maturation, lipid droplet formation was confirmed using Oil Red O staining. The adipocytes contained functional lipid droplets coated with perilipin-1. Free fatty acids were present in CMA in a concentration of about 2 µM. HCC spheroid growth was accelerated by both CMA and CMF, as reflected by a significantly larger spheroid diameter at day 7. The addition of fatty acids to the medium did not induce significant growth differences compared to the control spheroids of both cell lines, neither at the same concentration as in CMA nor at a 100-fold higher concentration. All treatments had no significant effect on the single cell diameter. Compared to CMF, CMA contained higher levels of lipocalin-2, resistin, VEGF and serpin E1. Adiponectin, TIMP-1 and MCP-1 were present in both CMF and CMA.

**Conclusion**

Our in vitro model is suitable for investigating the influence of adipocytes on HCC cells. CMA and CMF, but not free fatty acids, accelerated HCC spheroid growth. This indicates a possible role for cell-derived cytokines and growth factors, which will be analysed further. To identify potential molecular imaging biomarkers induced by adipocyte-HCC interaction, a proteomics analysis of the treated spheroids is planned.

### EACR2024-0501
### Exploring Dynamic Adaptations in Breast Cancer: ERα Loss During Bone Metastasis and Novel In Vitro Environment Emulation

*G. TURAN*[1,2], *G. Korkmaz*[2,3]
[1]*Koç University, Graduate School of Health Sciences, İstanbul, Turkiye*
[2]*KUTTAM, Research Center for Translational Medicine, İstanbul, Turkiye*
[3]*Koç University, School of Medicine, İstanbul, Turkiye*

**Introduction**

Breast cancer, the most prevalent cancer among women in 2022 at 23.8% incidence, remains lethal due to distant organ metastasis. Bone metastasis, notably frequent in ERα-positive patients, lacks comprehensive study due to sampling challenges. We aimed to investigate the influence of the bone microenvironment on ERα-positive breast cancer.

**Material and Methods**

Using RNA-seq datasets GSE152312 and GSE121677, we analyzed gene expression changes in MCF7 cells alone and co-cultured with bone marrow stromal cells. We used DESeq2 to identify genes with significant expression alterations ($p<0.05$) and GSEA to reveal enriched biological processes. MCF7 cells were cultured with hFOB and RAW264.7 conditioned medium to assess migration and invasion through transwell assays.

**Results and Discussions**

Our analysis of both datasets revealed 451 genes with increased expression and 492 genes with decreased expression. Notably, we observed a significant downregulation of ESR1 in both datasets, consistent with literature findings. Furthermore, our study showed that this downregulation of ESR1 coincided with decreased biological processes related to Estradiol, as highlighted by GSEA. In the luminal identity test conducted on hFOB and RAW264.7 cell lines, expressions of ESR1 and FOXA1 were notably low. Subsequently, MCF7s cultured in conditioned mediums from hFOB and RAW264.7 exhibited a significant reduction in ESR1 and FOXA1 expressions after 7 days. In the 2D transwell assay, RAW264.7 conditioned medium notably hindered MCF7 cell migration, while hFOB conditioned medium increased migration significantly. Results from the 3D transwell assay mirrored those of the 2D assay for RAW264.7. Regarding hFOB, there was an observable increase in cell migration of MCF7, although it was not statistically significant. RAW264.7 effectively impeded both migration/metastasis and invasion, whereas the hFOB conditioned medium promoted migration/ metastasis without significantly impacting invasion.

**Conclusion**

Altogether, our study found a notable decrease in the expression of ESR1 and FOXA1 factors in the MCF7 when exposed to the hFOB conditioned medium. Additionally, the observed increase in migration of MCF7 upon culturing hFOB conditioned medium suggests successful mimicry of the bone micro-environment. By combining transcriptome analyses of luminal cell lines grown in conditioned medium with open-source analyses, we can gain more detailed insights into biomarkers and processes related to bone metastasis.

### EACR2024-0536
### Discovering therapeutic targets for CTNNB1 mutation driven hepatocellular carcinoma using mouse organoid platform

*T.L. Wong*[1], *H. Wu*[1], *I.B. Huang*[1], *Y. Zhang*[2], *P.S. Li*[1], *L. Zhou*[3], *Y. Tu*[2], *J. Wong*[1], *A. Cheng*[2], *S. Ma*[1]

© 2024 The Authors. Molecular Oncology is published by John Wiley & Sons Ltd on behalf of Federation of European Biochemical Societies

[1]The University of Hong Kong, School of Biomedical Sciences, Hong Kong, Hong Kong-China
[2]The Chinese University of Hong Kong, School of Biomedical Sciences, Hong Kong, Hong Kong-China
[3]Sun Yet-Sen University, Precision Medicine Institute-The First Affiliated Hospital, Guangzhou, China

**Introduction**

The treatment options for hepatocellular carcinoma (HCC) have expanded in recent years, but the clinical benefits are limited, with only a short improvement in survival. This is due to the complex nature of the disease, which involves a mixture of driver and passenger mutations in cancer cells. Traditional cell lines and animal models do not provide a clear background to study the effects of individual driver mutations in detail. To address this, a mouse organoid platform was developed using HCC tissue with defined driver mutations (loss of TP53, Axin1, PTEN, and CTNNB1 activating mutations delivered via hydrodynamic tail vein injection) to study the characteristics of driver-specific HCC and their response to therapy.

**Material and Methods**

High-throughput screening was performed using driver-specific HCC mouse organoids for drug repurposing. Multi-omics analyses were used to understand the mechanism underlying the selective response to drugs.

**Results and Discussions**

High-throughput drug screening identified the ERBB family inhibitors as therapeutic targets for CTNNB1 mutation-driven HCC (Δ90CTNNB1/MYC). The sensitivity of the Δ90CTNNB1/MYC model towards ERBB family inhibitors was validated using human HCC patient-derived organoids and HCC cell lines harboring the CTNNB1 activation mutation. Transcriptome analysis revealed enrichment of the ERBB2 signaling pathway in the Δ90CTNNB1/MYC model. ATAC-seq analysis showed that increased chromatin accessibility to ERBB signaling pathway-related genes, such as *Egfr* and *Tgfa*, contributed to activation of the ERBB signaling pathway in the Δ90CTNNB1/MYC model. Phosphoproteomic analysis also predicted increased activity of ERBB family kinases in the Δ90CTNNB1/MYC model. This was confirmed by the increased levels of phospho-Egfr and phospho-Erbb2 in Δ90CTNNB1/MYC organoids and corresponding tumor tissues. The sensitivity of CTNNB1 mutation-driven HCC to ERBB family inhibitors was validated using animal models. Using publicly available data, we confirmed the activation of the ERBB signaling pathway in HCC patients with CTNNB1 activating mutation.

**Conclusion**

We identified a novel therapeutic target for CTNNB1 mutation-driven HCC. This study utilized multi-omics technologies combined with mouse driver-specific HCC organoids to understand the mechanism by which the ERBB signaling pathway is activated at the genomic and proteomic levels. Findings could provide a foundation for future repurposing of ERBB family inhibitors for the treatment of HCC patients with CTNNB1 mutations.

### EACR2024-0539
### The Investigation of Somatostatin Receptors as a Potential Target in Breast Phyllodes Tumours

H. Süer Mickler[1], M.M. Erkan[2]
[1]Acibadem Mehmet Ali Aydinlar University, Graduate School of Health Sciences, Istanbul, Turkiye
[2]Acibadem Mehmet Ali Aydınlar University, School of Medicine, Istanbul, Turkiye

**Introduction**

Somatostatin receptors (SSTRs) modulate hormonal secretion and regulate cell proliferation and apoptosis upon binding with somatostatin or its synthetic analogues. SSTRs are expressed in most neuroendocrine tumours (NET), particularly in gastroenteropancreatic NETs, serving as crucial diagnostic and therapeutic targets. The radioiodinated somatostatin analogue DOTATATE, labelled with either $^{68}$Gallium or $^{177}$Lutetium, is used for SSTR-targeting in diagnosis and treatment, respectively. SSTR expression is reported in a subset of breast adenocarcinoma and NETs; yet, remains less studied in fibroepithelial breast lesions; fibroadenoma and phyllodes tumours. while fibroadenomas are typically benign the more aggressive "cystosarcoma phyllodes" present challenges in management with potential for recurrences and metastases, highlighting the need for effective therapeutic approaches.

**Material and Methods**

Herein we investigated both gene and protein expression of SSTR in fibroepithelial lesions of the breast. Using RT-PCR and Western blotting we first confirmed the presence of SSTR in breast fibroepithelial lesions and later with immunohistochemical and immunofluorescence techniques tissue compartment that expresses SSTR was characterised.

**Results and Discussions**

Findings reveal that both fibroadenoma and phyllodes tumours express SSTRs. Immunohistochemical analyses suggested that this expression is in the stromal, not epithelial, component by demonstrating that SSTR is predominantly expressed in the areas overlapping with α-smooth muscle actin-positive myoepithelial cells around blood vessels and capillary structures. This study is the first in the literature to demonstrate SSTR positivity in mammary fibroepithelial neoplasms.

**Conclusion**

These findings shed new light on the progression of fibroepithelial neoplasms. It has long been a curiosity as to which component of these tumours is truly involved in the driver's seat. The fact that malignancy in these tumours typically arises from the stromal component and manifests as sarcoma has suggested that it may be the stromal component that is the main driver of these tumours. The preferential expression of SSTRs in the stromal component also supports this impression. In a patient undergoing a gallium scan, which is widely used in clinical practice, if a positive lesion is discovered in the breast, the possibility of a fibroadenoma or phyllodes tumour needs to be considered. Once validated, these findings may also have significant implications for the management of these tumours.

### EACR2024-0544
### Macrophage MTDH-dervied exosome contributes to pre-metastatic niche

© 2024 The Authors. Molecular Oncology is published by John Wiley & Sons Ltd on behalf of Federation of European Biochemical Societies

**formation and metastasis by transferring KLC2 in head and neck squamous cell carcinoma**

X. Liu[1], Q. Sun[1]
[1]Central Hospital Affiliated to Shandong First Medical University, Research Center of Translational Medicine, jinan, China

**Introduction**

Extracellular vesicles (EV) have been shown to support pre-metastatic niche (PMN) formation by modulating stromal cells at future metastatic sites. We have confirmed that macrophages with MTDH overexpression promoted metastasis of head and neck suqmaous cell carcinoma (HNSCC). This study aimed to explore the underlying mechanisms of how macrophage MTDH-derived exosomes modulate the lung pre-metastatic niche and metastasis in HNSCC.

**Material and Methods**

Stable THP-1 cell lines with MTDH overexpression (Mac-MTDH vs Mac-vector) was established using lentivirus transfection and then diffentiated into macrophages to collect exosomes (Mac-MTDH-EV vs Mac-vector-EV). Exosomes derived from macrophages were assessed for the ability to promote PMN formation and HNSCC metastasis by exosome education and nude mouse lung cancer metastasis model. Collagen deposition was determined by Masson staining. Mass spectrometry was performed to identify KLC2 highly expressed in Mac-MTDH-EV versus Mac-vector-EV and then dual-luciferase reporter, quantitative chromatin immunoprecipitation (qChIP) assays, and rescue experiments were performed to determine the regulatory mechanism of KLC2 in macrophages. Human umbilical vein endothelial cells (HUVEC) were treated with exosomes derived from macrophages. Endothelial/mesenchymal markers and related pathways were evaluated by real-time PCR and immunoblotting.

**Results and Discussions**

Mac-MTDH-EV promoted lung pre-metastatic niche formation and metastasis of HNSCC cell lines. It also increaed the fibronectin contents & collagen deposition. KLC2 was upregulated in Mac-MTDH-EV compared with Mac-vector-EV. The overexpression of MTDH in macrophages activated GSK-3β/β-catenin signaling pathways, which transcritionally regulated KLC2 by β-catenin directly binding to its promoter. Most interestingly, we found that Mac-MTDH-derived exosome transferred KLC2 to endothelial cells, which induced myofibroblast differentiation and endothelial-to-mesenchymal transition (EndoMT) by activating p-Smad2/3 signalling in vitro and in vivo.

**Conclusion**

Overall, our study highlights that Macrophages with MTDH overexpression increased the lung metastasis of HNSCC by exosomal KLC2 mediated EndoMT and accelarating the pro-fibrotic microenvironment.

### EACR2024-0547
### Characterization of Spontaneous Calcium Oscillations and Their Role in Mechano-Electrical Coupling (MEC)

J. Roberts[1], Y. Hayashi[2], Z. Wang[3], N. Vasudevan[1]
[1]University of Reading, School of Biological Sciences – Biomedical Sciences, Reading, United Kingdom
[2]University of Reading, School of Biological Sciences – Biomedical Engineering, Reading, United Kingdom
[3]University of Reading, Department of Mathematics and Statistics, Reading, United Kingdom

**Introduction**

Spontaneous calcium oscillations in cancer cells contribute to dysregulated cellular behaviours like proliferation and migration, further intertwining with mechanical cues within the tumour microenvironment, termed mechano-electrical coupling (MEC). This is particularly relevant as they experience mechanical shear stress during migration, highlighting the importance of understanding the impact of mechanical motion, such as stretching, on cellular microstructure and downstream electrical signalling in glioblastomas (U87) and breast cancer (MDA-MB-231) cell lines.

**Material and Methods**

Employing MCherry-labelled cells and GCaMPs as calcium indicators, we conducted confocal microscopy imaging to observe cell networks and basal oscillations. Fluorescence intensity analysis using Image J facilitated the quantification of oscillatory patterns within delineated regions of interest (ROI).

**Results and Discussions**

Our findings reveal distinct calcium wave patterns between U87 and MDA-MB-231 cells, characterized by varying kinetics, with the latter exhibiting slower oscillations. Notably, within each cell line, diversity in oscillatory patterns was observed, manifesting as either transient or periodic phenomena. Surprisingly, the percentage of oscillating cells remained consistent across different culture durations, underscoring the robustness of these cellular behaviours. Furthermore, we observed a correlation between cell morphology and calcium oscillations, particularly in the U87 cell line, where oscillating cells displayed elongated shapes. In contrast, size had no discernible impact on oscillatory dynamics in MDA-MB-231 cells, suggesting differential cellular mechanisms governing oscillations in these contexts. Hypothesizing on the underlying mechanisms, we propose that oscillations within cells are sustained across the network, possibly mediated through IP3 or RyR systems, and heightened by the activity of mitochondrial $Ca^{2+}$ uniporters. These oscillations evolve as tumour microtubules enriched in thin F-actin filaments emerge, giving rise to transient calcium spikes and perpetuating malignancy through ongoing network development.

**Conclusion**

In summary, our study sheds light on the complex interplay between mechanical and electrical cues in cancer cells, providing insights into the mechanisms driving spontaneous calcium oscillations and their implications for tumour progression. Understanding these dynamics may offer new avenues for targeted therapeutic interventions aimed at disrupting MEC-mediated cancer progression.

### EACR2024-0549
### Unveiling Innate Immune Evasion in a Zebrafish Xenograft Model

© 2024 The Authors. Molecular Oncology is published by John Wiley & Sons Ltd on behalf of Federation of European Biochemical Societies

V. Póvoa[1]
[1]Champalimaud Foundation, Cancer Development and Innate Immune Evasion Lab, Lisboa, Portugal

**Introduction**

Colorectal cancer (CRC) is the second leading cause of cancer death and immunotherapy has emerged as a treatment option for metastatic CRC patients. However, only a subset of patients responds to this treatment. A major contributor for the differences observed in therapy response as well as cancer progression is intratumor heterogeneity. A better knowledge of the complex interactions between cancer cells and the innate immune system is crucial for developing novel immunotherapy strategies.

**Material and Methods**

CRC cell lines derived from the same patient in different stages of tumor progression (SW480 primary tumor and SW620 lymph node metastasis) were injected into 2 days old zebrafish larvae. At 4 days post injection zebrafish xenografts were sacrificed and engraftment, tumor size and immune cell recruitment to the tumor micro-environment was quantified. To investigate the cellular interactions between innate immune cells and tumor cells we used specific transgenic and mutant zebrafish lines that label or ablate macrophages and neutrophil cell populations.

**Results and Discussions**

We found contrasting phenotypes, whereas SW620 cells engraft very efficiently, SW480 were rejected in 4 days. To assess if SW620 cells create a suppressive microenvironment both cells were mixed in vivo (1:1). Our results shows that in the presence of SW620, SW480 cells can engraft more efficiently, suggesting that SW620 may indeed protect SW480 from rejection. Characterization of the TME revealed that SW480 recruit neutrophils and macrophages more efficiently than SW620. Moreover, SW620 TME are enriched in M2-like macrophages, whereas SW480 are enriched in M1-like. Importantly, genetic and chemical depletion of myeloid cells increase engraftment of SW480 and tumor size, confirming the crucial role of macrophages and neutrophils in rejection/clearance. To test whether cancer innate immunoediting was occurring in this short time frame, re-transplantation experiments of SW480 escaper tumors were performed. These tumors engrafted more efficiently, generating larger tumors with reduced macrophage infiltration. Finally, to investigate the molecular alterations leading to SW480 escapers emergence, single-cell transcriptomic profiling was performed, revealing a fast subclonal selection, with clearance of regressor subclones associated with IFN/Notch signaling and escaper-expanded subclones with enrichment of IL-10 pathway.

**Conclusion**

Our work opens the possibility of using zebrafish xenografts as living biomarkers of the tumor microenvironment.

### EACR2024-0554
### Modulating NNMT in Cancer-Associated Fibroblasts Mitigates Desmoplasia in Non-Small Cell Lung Cancer through TGFβ Signalling pathway

C. Chen[1], J.C. Ho[2]
[1]The University of Hong Kong, Department of Medicne, Hong Kong, China
[2]The University of Hong Kong, Department of Medicine, Hong Kong, China

**Introduction**

Lung cancer-associated fibroblasts (CAFs) play a crucial role in tumour desmoplasia, with collagen synthesis being a hallmark of their function. Upregulation of Nicotinamide N-Methyltransferase (NNMT) expression in CAFs has been identified. Nonetheless, the underlying mechanism by which NNMT modulates collagen synthesis remains elusive. This study aims to explore the intricate regulatory network governing collagen synthesis by lung CAFs via NNMT.

**Material and Methods**

Lung CAFs were generated through co-culturing normal human lung fibroblasts (MRC-5) with non-small cell lung cancer (NSCLC) cell lines (H358 and HCC827) [termed H358-CAFs and HCC827-CAFs], or treatment of MRC-5 with TGFβ1. The expression levels of NNMT and collagen in lung CAFs were evaluated using Western blot and immunofluorescence. Either NNMTi or siRNA targeting NNMT was employed to assess the potential reduction of collagen synthesis. Subsequently, HCC827 cells were percutaneously injected into the lung of mice, with or without HCC827-CAFs, to establish OX model. The efficacy of NNMTi was then evaluated to determine its capability in mitigating desmoplasia in this model. Tumour growth was continuously monitored using micro-computed tomography (CT), and the response was evaluated according to RECIST v1.1 criteria. Collagen deposition was demonstrated by immunohistochemistry and picrosirius red staining.

**Results and Discussions**

Western blot and immunofluorescence revealed NNMT overexpression in NSCLC cells, H358-CAFs and HCC827-CAFs. Treatment with TGFβ1 induced the conversion of normal lung fibroblasts (MRC-5) into α-$SMA^+$ CAFs, resulting in elevated collagen levels and upregulation of NNMT and SMAD2/3 expression. The application of NNMTi or siRNA targeting NNMT led to a reduction in the expression levels of α-SMA, Col 1A1 and SMAD2/3 in myofibroblasts activated by TGFβ1. Administration of NNMTi in vivo led to a significant reduction in lung CAFs, effectively suppressing tumor burden (mean ± SEM: 62.51 ± 5.68 vs. 48.46 ± 1.36, $p < 0.05$), proliferation (mean ± SEM: 20.86 ± 1.24 vs. 15.35 ± 1.32, $p < 0.05$), and collagen deposition (mean ± SEM: 19.46 ± 1.31 vs. 2.23 ± 0.49, $p < 0.0001$) in the HCC827-CAFs-rich NSCLC OX model.

**Conclusion**

NNMT functions as a regulatory factor in coordinating the transformation of regular normal lung fibroblasts into myofibroblasts and controlling collagen synthesis via the TGFβ-SMAD2/3 signalling pathway. Targeting lung CAFs with NNMTi shows potential in reducing desmoplasia and inhibiting tumor growth in CAFs-rich NSCLC.

### EACR2024-0564
### Role of NOTCH pathway and related miRNAs in glioma patients and in-vitro

© 2024 The Authors. Molecular Oncology is published by John Wiley & Sons Ltd on behalf of Federation of European Biochemical Societies

**effect of miR-34a as a therapeutic approach in TMZ resistance**

B. Karacam[1,2,3], S. Punar[2,3], G. Coban[4], K. Akdur[5], I. Khan[1], S.S. Ekmekci[3], M.A. Hatiboglu[1,5]
[1]Bezmialem Vakif University- Beykoz Institute of Life Sciences and Biotechnology, Department of Molecular Biology, Istanbul, Turkiye
[2]Istanbul University- Institute of Graduate Studies in Health Sciences, Department of Genetics, Istanbul, Turkiye
[3]Istanbul University- Aziz Sancar Institute of Experimental Medicine, Department of Genetics, Istanbul, Turkiye
[4]Bezmialem Vakif University- Medical School, Department of Pathology, Istanbul, Turkiye
[5]Bezmialem Vakif University- Medical School, Department of Neurosurgery, Istanbul, Turkiye

**Introduction**

Gliomas -the most common form of central nervous system malignancies- are classified based on their molecular, genetic, and histopathologic features in grade 1 to 4. Temozolomide (TMZ) is an alkylating agent used in the treatment of high-grade gliomas in combination with radiotherapy. Even though TMZ prolongs the survival time of patients, resistance to TMZ limits the effectiveness of the treatment. Therefore, new treatment options are required to overcome TMZ resistance in high-grade gliomas.

**Material and Methods**

Glioma patients (grade 2 in 11 patients, grade 3 in 11 patients, grade 4 in 78 patients) who underwent surgical resection were included in this study. IHC and RNA isolation were done from the FFPE tissues of those patients and their adjacent normal tissues. Adjacent normal tissues (n=20) were used as a control group. The expressions of NOTCH1 and HES1 were examined with IHC. Immunostained slides were evaluated by a pathologist, scoring for staining extent and intensity using a 3-point (0-3) scale. To determine miRNAs related to NOTCH, expressions of miR-34a, miR-139b, and miR-200b were assessed with qPCR. Clinical data was correlated with the NOTCH1 staining score, HES1 staining score, and expressions of NOTCH1-related miRNAs. miR-34a mimic, which is selected from other miRNAs, was used as a therapeutic in combination with TMZ in U87 cells in-vitro. miR-34a mimic was transfected with RNAiMAX transfection agent. MTT assay, Scratch assay, Transwell assay, and Apoptosis assay were done as downstream experiments after treatments.

**Results and Discussions**

The expression of NOTCH1 increased in a grade-dependent manner. Additionally, glioma patients expressed more NOTCH1 compared to normal adjacent tissue. Grade 4 gliomas had the highest expression of HES1 and Grade 3 patients had the lowest expression among all patients. There was no expression of HES1 in normal adjacent tissue. The expression of miR-34a, miR-139b, and miR-200b was downregulated in glioma tumor tissues compared to the adjacent normal tissues. miR-34a mimic transfection was performed and validated. The expression of NOTCH1 -target gene of miR-34a- was decreased after the transfection. miR-34a enhanced the cytotoxic effect of TMZ, inhibited the migration of GBM cells, and increased the apoptotic cells.

**Conclusion**

Our results suggested that NOTCH1 and HES1 would be diagnostic markers. miR-34a, miR-139b, and miR-200b which are related to NOTCH1 were expressed less in higher grade gliomas. miR-34a has a potential role in the treatment of GBM.

### EACR2024-0566
### TIMP1 Expression Correlates with a Pro-Tumorigenic Phenotype in Glioblastoma

A. Poole[1], S. Menon[2], X. Lun[1], A. Dufour[3], D. Senger[4], S. Robbins[4]
[1]University of Calgary, Arnie Charbonneau Cancer Institute, Calgary, Canada
[2]McGill Univeristy, Lady Davis Institute for Medical Research, Montreal, Canada
[3]University of Calgary, McCaig Institute for Bone and Joint Health, Calgary, Canada
[4]McGill University, Lady Davis Institute for Medical Research, Montreal, Canada

**Introduction**

Glioblastoma Multiforme (GBM) are the most aggressive form of brain cancer due to high rates of reoccurrence and limited therapeutics. A major contributor to the aggressiveness of GBM is the unique tumor microenvironment, which contributes to the proliferation and invasion. One of the main research objectives in our lab is to further understand how the tumor microenvironment promotes glioma progression and therapeutic resistance. Previously we have demonstrated that the cytokine IL-33 promotes a pro-tumor inflammatory environment that supports tumor growth and progression. Tumors that express higher levels of IL-33 have more immune infiltration, specifically macrophage/microglia infiltration, with these cells presenting an M2-like phenotype. In addition, these tumors with higher pro-tumor immune infiltration have poorer survival. Thus we wanted to further understand what secreted factors contributed to the establishment of a pro-tumor immune environment.

**Material and Methods**

Tumor interstitial fluid (TIF) was collected from U87IL33 (IL33) and U87pcDNA(CON) tumor bearing mice at 5, 10, and 14 days. TIF was analyzed using the SomaLogic SomaScan platform and cytokine specific profiling (Luminex). Differential expressing testing between IL-33 and CON TIF at each time were independently performed using the SomaLogic DataDelve software. Differentially expressed proteins at day 14 were input into ClusterProfiler to identify enriched pathways. The top 15 enriched pathways in IL-33 TIF versus CON TIF were analyzed for common genes.

**Results and Discussions**

Day 14 showed the greatest difference in protein expression, with 267 differentially expressed proteins between IL-33 and CON, with the majority upregulated in IL33. Proteolysis and extracellular pathways were upregulated at day 14 in the IL33 pro-inflammatory model. A common protein identified in the top upregulated pathways was TIMP1. Luminex of TIMP1

© 2024 The Authors. Molecular Oncology is published by John Wiley & Sons Ltd on behalf of Federation of European Biochemical Societies further confirmed that at day 14, mouse and human TIMP1 is enriched in IL-33. TIMP1 may be expressed by glioma cells and/or macrophages in the tumor environment.

**Conclusion**

TIMP1 is known for its function as a broad matrix metalloproteinase(MMP) inhibitor that inhibits MMPs typically upregulated in glioma. TIMP1 can also act as a cytokine binding to its known receptors of CD63 and CD74, activating pathways involved in migration, proliferation and survival. Thus we believe that increased TIMP1 expression may promote a pro-tumor micro-environment, through regulating milieu surrounding the tumor and immune cell activation.

### EACR2024-0578

### Targeting tumour-stroma architecture shapes T cell dynamics and local recruitment in head and neck squamous cell carcinoma

*G. Giangreco[1], Z. Ramsden[1], A. Rullan[2], S. Hooper[1], E. Sahai[1]*

*[1]The Francis Crick Institute, Tumour Cell Biology Laboratory, London, United Kingdom*

*[2]Institute of Cancer Research, Targeted Therapy Team, London, United Kingdom*

**Introduction**

Access of CD8+ T cells inside tumour cores is often prevented by tumour microenvironment (TME) and is frequently associated with poor survival. Tumours are classified according to their immune content as inflamed, excluded or desert. However, spatial organization of different stromal non-immune TME cells is often overlooked and there is a huge unmet medical need to increase T cell accessibility and immunotherapy efficacy. Here, we aimed to study immune microenvironments and tumour-stroma architectures to identify key stromal players to target in order to increase access of anti-tumour immune cells at tumour cores.

**Material and Methods**

We established syngeneic murine models of head and neck squamous cell carcinoma – MOC1 and MOC2 – that recapitulate tumour-stroma organizations and evolution. By applying single cell RNAseq and multiplexed imaging mass cytometry, we characterized expression and localization of the main TME components. We then established an in vitro PDMS-based device to recapitulate in vivo tumour boundaries and characterize T cell dynamics. We performed a genetic screen to identify stromal targets that can alter tumour-stroma organization and increase T cell access inside tumour cores.

**Results and Discussions**

We show that MOC1 and MOC2 models have different mechanisms of immune evasion and tumour-stroma architectures. MOC1 tumours transit from a T cell infiltrated to an immune excluded/desert TME. This is correlated with higher abundance of fibroblasts and well-defined tumour-stroma interfaces (nests-like TME). MOC2 tumours are not subject to effective T-cell control, have a myeloid-rich immune TME and exhibit poorly defined tumour-stroma interfaces (mixed TME). By isolating primary CAFs and T cells, we in vitro recreated mixed and nests-like TMEs and show that T cell dynamics are influenced by their location and are enriched at the interface when nests-like TMEs are present. We then analysed human and murine head and neck transcriptomic samples to perform a genetic screening that led to identification of different fibroblast specific targets that can alter T cell accessibility inside tumour nests. Of note, we show that targeting stromal receptor tyrosine kinase signalling alters T cell dynamics.

**Conclusion**

Overall, we linked different immune evasion mechanisms to different tumour-stroma architectures and demonstrate that CD8+ T cells are enriched at tumour-stroma interphases. We identified stromal targets of interest that can have the potential to increase T cell accessibility inside tumour cores.

### EACR2024-0593

### AXL overexpression drives angiogenesis in HER2-positive breast cancer

*A. Ágreda-Roca[1], S. Torres-Ruiz[1], A. Laimeirinhas[1,2], M. Tapia[1,3], C. Hernando[1,3], M.T. Martínez[1,3], A. Lluch[1,3,4,5], B. Bermejo[1,3,5], I. Garrido-Cano[1,2,6], J.M. Cejalvo[1,3,5]*

*[1]Biomedical Research Institute INCLIVA, Breast Cancer Biology Research Group, València, Spain*

*[2]Universitat Politècnica de València, Instituto Inter-universitario de Investigación de Reconocimiento Molecular y Desarrollo Tecnológico IDM, València, Spain*

*[3]Hospital Clínico Universitario de Valencia, Clinical Oncology Department, València, Spain*

*[4]University of València, Department of Physiology, València, Spain*

*[5]CIBERONC, Centro de Investigación Biomédica en Red de Cáncer, València, Spain*

*[6]CIBER-BBN, CIBER de Bioingeniería- Biomateriales y Nanomedicina, Madrid, Spain*

**Introduction**

AXL overexpression has been related to tumor development, metastasis, and drug resistance through positive modulation of epithelial-mesenchymal transition in HER2-positive breast cancer (BC). Recent studies also support that higher AXL expression promotes an immunosuppressive microenvironment. However, its role is still being explored. Concretely, we aim to elucidate the role of AXL in angiogenesis.

**Material and Methods**

HER2-positive BC cell lines were genetically engineered to overexpress AXL byplasmid transfection, and conditioned media (CM) was collected after 48 hours. Endothelial tube formation assay was then performed by using CM on Human umbilicalvein endothelial cells (HUVECs). Moreover, gene expression of angiogenesis markerswas assessed by real-time quantitative polymerase chain reaction (RT-qPCR).

**Results and Discussions**

Overexpression of AXL significantly promoted endothelial cell tube formation in vitro compared to control. Additionally, RT-qPCR analyses demonstrated upregulation of pro-angiogenic factors in AXL-overexpressing cells.

© 2024The Authors. Molecular Oncology is published by John Wiley & Sons Ltd on behalf of Federation of European Biochemical Societies

**Conclusion**
Our findings suggest that AXL overexpression promotes angiogenesis in HER2-positive BC cell lines through upregulation of pro-angiogenic factors. In this sense, targeting AXL is a promising therapeutic strategy to improve HER2-positive BC patients' outcomes.

### EACR2024-0600
### Proteoglycans and glycosaminoglycans influence the epithelioid to spindle cell morphological transition in Biphasic Malignant Peritoneal Mesothelioma impacting its biological and clinical behaviors

*A. Nery Qualiotto[1], C. Machado Baldavira[1], T. Gutierrez Prieto[1], M. Balancin[1], A. Ab'Saber[1], T. Takagaki[2], V. Luiza Capelozzi[1]*
*[1]Faculty of Medicine of University of Sao Paulo, Pathology, Sao Paulo, Brazil*
*[2]Instituto do coração Incor- University of Sao Paulo, Pneumology Division, Sao Paulo, Brazil*

**Introduction**
Malignant mesothelioma (MM) typically exhibits both epithelioid and spindle-fibroblast-like histologies, resulting in a biphasic phenotype that impacts its biological and clinical characteristics. Prior studies have demonstrated that mesothelioma's biphasic growth potential can be replicated in derived cell sublines, which can differentiate into either epithelial or spindle-shaped fibroblast-like phenotypes, thus emulating mesothelioma's growth patterns observed in vivo. Biochemical analyses have shown that differentiated epithelial cells produce significantly higher amounts of glycosaminoglycans (GAGs), which, when linked covalently to a protein core, form proteoglycans (PGs). PGs feature a diverse array of structures, including various core proteins, classes of GAGs, and varying numbers and lengths of GAG chains. Thus, this study aimed to explore how the expression of GAGs and PGs influences the biological and clinical behavior of mesothelioma.
**Material and Methods**
To test our hypothesis, we analyzed 66 human mesothelioma samples exhibiting either epithelial or fibroblast-like morphologies, reflective of these sarcoma's biphasic growth. Quantitative immuno-histochemistry was used to examine the GAGs heparan sulfate (HS) and chondroitin sulfate (CS), as well as the PG versican, employing QuPath software for analysis.
**Results and Discussions**
Our findings indicated that cells with a fibroblast-like spindle phenotype exhibited increased expression of HS ($P<0.001$) and versican ($P<0.001$) within their matrix. Immunohistochemistry, in conjunction with histological analysis, suggests that the augmented and potentially rapid turnover of cell membrane PGs leads to a shift from polygonal to spindle-shaped morphology. Conversely, GAGs seem to play a role in cell aggregation, highlighting the distinct functions of various GAGs. A multivariate Cox regression model revealed significantly lower overall survival rates for non-operated patients [HR, 4.68 (1.58-13.86); P=0.005], which presented necrosis [$P<0.002$] and high expression of HS [P=0.01], those extra-pleural location, exposure to asbestos, epithelial phenotype and high expression of versican as co-variables.
**Conclusion**
The immunohistochemical profile of matrix GAGs and PGs in peritoneal MM influences the morphological dynamics and biological behavior of tumor cells, presenting a distinct profile from that observed in pleural MM.

### EACR2024-0603
### α5β1 integrin/fibronectin influences invasion, dissemination and macrophage phenotype in oral squamous cell carcinoma

*N.A. Rodrigues Fernandes[1], W. Wahbi[2], C. Rodrigues Nascimento[1], M. Moraes de Carvalho[1], T. Salo[2], A. Al-Samadi[2], C. Rossa Junior[1]*
*[1]Sao Paulo State University, Department of Diagnosis and Surgery- School of Dentistry at Araraquara, Araraquara, Brazil*
*[2]University of Helsinki, Department of Oral and Maxillo-facial Diseases- Faculty of Medicine, Helsinki, Finland*

**Introduction**
Desmoplastic oral squamous cell carcinomas (OSCC) are characterized by an extracellular matrix of increased stiffness and are generally more aggressive and associated with worse prognosis. Fibronectin (Fn) is considered a key component of a desmoplastic microenvironment and α5β1 integrin is its most specific receptor. We investigated the influence of α5β1 integrin signaling on OSCC in vitro and in vivo.
**Material and Methods**
In silico – we used GEPIA and TIMER to analyse Fn1 (fibronectin), Itga5 (α5) and Itgb1 (β1) gene expression in head and neck squamous carcinoma (HNSCC, n=519) from TCGA database and compare them with the non-tumoral tissue (n=44) samples, and also the correlation with infiltration of macrophages and cancer-associated fibroblasts (CAF). In vitro – OSCC cell lines (SCC9 and H314, high and low expression of α5β1 integrin respectively) were co-cultured with monocytes and CAF in the presence of Fn to form heterospheroid tridimensional cultures and assess the spatial localization of cells by confocal microscopy. α5β1 integrin signaling was inhibited in these heterospheroid cultures with the peptide ATN-161 with the subsequent assessment of in vitro invasion using Myogel, a human tumor-derived matrix. The phenotype of monocytes/macrophages (CD80 and CD163) was also assessed using flow cytometry. In vivo – co-culture of monocytes, CAF and SCC9 cells were injected in zebrafish larvae that were subsequently treated for 3 days with ATN-161 alone or as an adjunct to cisplatin and radiation. Outcomes assessed in vivo were tumor size and metastasis.
**Results and Discussions**
Fn1, Itga5 and Itgb1 are highly expressed and positively correlated with infiltration of M2 macrophages and CAFs in HNSCC. In vitro, Fn affected the spatial distribution of

© 2024 The Authors. Molecular Oncology is published by John Wiley & Sons Ltd on behalf of Federation of European Biochemical Societies tumor cells in heterospheroid cultures and increased invasion. Inhibition of α5β1 integrin signaling with ATN-161 reduced invasion and increased the ratio of CD80/CD163 expression in monocytes/macrophages. These results were observed in heterospheroids with SCC9 cells ('α5β1 high'), but not with H314 cells ('α5β1 low'), supporting a specific influence of α5β1 signaling in these outcomes. Zebrafish experiments were conducted with SCC9 cells only. In vivo, inhibition of α5β1 integrin reduced tumor size and metastasis, and when used as an adjunct to cisplatin and radiation further reduced tumor size.

**Conclusion**

α5β1 integrin/fibronectin is increased in HNSCC and is correlated with tumor invasion and dissemination and with a skewing of macrophages to the M2 phenotype.

### EACR2024-0605

### Cancer associated fibroblast-derived Wilms Tumour 1: an immunotherapeutic target that regulates T cell proliferation and differentiation within the breast tumour microenvironment

*R. Li[1], D. O' Connor[1], B. Digby[2], P. Loftus[3], N. Ge[4], M. Kerin[1], L. Barkley[1]*

*[1]University of Galway, Lambe Institute for translational research, Galway, Ireland*

*[2]University of Galway, School of Mathematics- Statistics & Applied Mathematics, Galway, Ireland*

*[3]Orbsen Therapeutics Ltd., Orbsen Therapeutics Ltd., Galway, Ireland*

*[4]University of Galway, Regenerative Medicine Institute- School of Medicine, Galway, Ireland*

**Introduction**

Cancer associated fibroblasts (CAFs) contribute to tumorigenesis and immune tolerance. Studies indicate that CAFs secrete indoleamine 2,3-dioxygenase (IDO) which activates immunosuppressive T regulatory cells (Tregs), and express programmed death ligand 1 (PD-L1) which inactivates tumour killing cytotoxic T cells (CTLs). Therefore, to discover novel strategies that specifically target CAFs within the TME is a viable therapeutic approach as it has the potential to enhance tumour immunity and reduce tumorigenesis.

**Material and Methods**

RNA sequencing was performed to identify genes upregulated in CAFs compared to normal fibroblasts from the same patient. The transcription factor, Wilms tumour-1 (WT-1), was identified as an upregulated gene. WT-1 levels in CAFs were manipulated using plasmid overexpression or siRNA downregulation strategies. CAFs were then co-cultured with stimulated T cells, and T cell proliferation and differentiation was determined using flow cytometry. To better determine the effect of CAF-derived WT-1 within the tumour microenvironment (TME) 3D patient-derived organoids (PDOs) were established.

**Results and Discussions**

Our data indicates WT-1 RNA and protein levels are upregulated in CAFs compared to normal fibroblasts. Knocking down WT-1 levels in CAFs reduces the ability of CAFs to suppress T cell proliferation and induce Treg differentiation. Conversely, increasing WT-1 levels in CAFs enhances their ability to block T cell proliferation and induce Treg differentiation. Interestingly, treatment of CAFs with paclitaxel (PTX) and doxorubicin (DOX) also increases WT-1 levels and enhances their ability to suppress T cell proliferation. Mechanistically, WT-1 regulates signal transducer and activator of transcription (STAT) 1/3 levels, which promotes PD-L1 expression and IDO release, thereby regulating the immuno-suppressive properties of CAFs. Our data also demonstrates that reducing CAF WT-1 levels results in smaller PDOs which correlates with increased levels of Granzyme B+/PD1$^{-}$ CD3$^{+}$ T cells within the TME.

**Conclusion**

Collectively, our findings indicate that therapeutic targeting of the CAF WT-1/STAT1/STAT3/PDL1/IDO axis has the potential to reduce the number of Tregs and enhance the proliferation of T cells within the TME, thereby enhancing tumour immunity and reducing tumorigenesis. Importantly, our data highlights the limitations of PTX and DOX treatment, as they cause an increase in CAF WT-1 levels which has the potential to enhance immune tolerance within the TME.

### EACR2024-0607

### Stroma-enriched ER Protein TXNDC5 Potentiates Pancreatic Adenocarcinoma Progression by Creating a Desmoplastic and Immunotolerant Microenvironment

*H.H. Wang[1], W.D. Lai[1], K.C. Yang[1,2,3,4], W.C. Liao[5]*

*[1]National Taiwan University College of Medicine, Department and Graduate Institute of Pharmacology, Taipei, Taiwan*

*[2]Academia Sinica, Institute of Biomedical Sciences, Taipei, Taiwan*

*[3]National Taiwan University, Research Center for Developmental Biology & Regenerative Medicine, Taipei, Taiwan*

*[4]National Taiwan University Hospital, Division of Cardiology- Department of Internal Medicine, Taipei, Taiwan*

*[5]Department of Internal Medicine- National Taiwan University Hospital, Division of Gastroenterology, Taipei, Taiwan*

**Introduction**

Pancreatic ductal adenocarcinoma (PDAC) features extensive desmoplasia in the tumor microenvironment (TME). Activated cancer-associated fibroblasts (CAFs) drive desmoplasia and PDAC progression by over-producing extracellular matrix (ECM) and secreting tumor-promoting factors. Recently, we have identified a ER protein thioredoxin domain containing 5 (TXNDC5), enriched in fibroblasts, as a critical mediator of organ fibrosis by driving fibroblast activation and ECM production. We hypothesized that TXNDC5 may also promote PDAC by activating CAF and remodeling ECM.

**Material and Methods**

RNA-Seq analyses and immunohistochemical (IHC) staining on human PDAC samples were conducted to determine the link between TXNDC5 and PDAC. A tamoxifen-inducible (Kras$^{LSL\text{-}G12D}$;p53$^{LoxP}$; Pdx1-CreER, KPC) spontaneous PDAC model and orthotopic KPC

© 2024 The Authors. Molecular Oncology is published by John Wiley & Sons Ltd on behalf of Federation of European Biochemical Societies allografting in *COL1A1-GFP*$^{Tg}$ mice, a transgenic line that labels CAFs with GFP, were exploited in the background of WT and *Txndc5*$^{-/-}$ mice to determine the impact of TXNDC5 on desmoplasia and PDAC progression. Orthotopic KPC allografting in control and fibroblast-specific *Txndx5* knockout (*Txndc5*$^{cKO}$) mice was conducted to clarify the role of fibroblast TXNDC5 to PDAC progression. Single-cell RNA sequencing (scRNA-Seq) on orthotopic KPC tumors from control and *Txndc5*$^{cKO}$ mice was used to determine the impact of fibroblast TXNDC5 on the cellular constituents of TME.

**Results and Discussions**

Human PDAC RNA-Seq dataset analyses revealed that increased TXNDC5 expression is associated with worse outcomes and higher ECM gene expression. IHC staining showed a marked upregulation of TXNDC5 in the stroma of human PDAC and mouse KPC tumors. Immunofluorescence staining on mouse KPC (both spontaneous and orthotopic) tumor sections showed a strong enrichment of TXNDC5 in CAFs, where increased TXNDC5 levels were accompanied by enhanced CAF activation and ECM expression. Both global and fibroblast-specific *Txndc5* deletion detered tumor growth and reduced desmoplatic changes in the orthotopic KPC tumor model. Finally, scRNA-Seq on orthotopic KPC tumors from control and *Txndc5*$^{cKO}$ mice revealed that TXNDC5 in CAFs maintains a desmoplastic, hypoxic, and immunotolerant TME supporting PDAC growth.

**Conclusion**

Fibroblast-enriched ER protein TXNDC5 plays a critical role in CAF activation, desmoplasia and tumor progression of PDAC. Targeting TXNDC5 in CAFs could be a powerful new approach to halt PDAC progression by reversing desmoplastic and immunotolerant TME.

### EACR2024-0608

### Fibroblast Growth Factor 19 (FGF19) and its analog Aldafermin synergize with MYC, potently promoting hepatocarcinogenesis

*G. Desandré[1], J. Ursic-Bedoya[1], C. Chavey[1], A. Polizzi[2], H. Guillou[2], E. Assenat[3], U. Hibner[1], D. Gregoire[1]*

*[1]Institut de Génétique Moléculaire de Montpellier, Cnrs, Montpellier, France*

*[2]Toxalim Research Center in Food Toxicology, Inrae, Toulouse, France*

*[3]Saint Eloi Hospital, Chu Montpellier, Montpellier, France*

**Introduction**

Increased expression of the fibroblast growth factor (FGF19), a hormone whose physiological function is the regulation of bile acid and glucose homeostasis, is a hallmark of a sub-group of aggressive hepatocellular carcinoma (HCC). Analogs have been developed to mimic the hepatoprotective metabolic effects of FGF19, while being theoretically devoid of its oncogenic effects. FGF19 analogs are under investigation for treatment of metabolic disorders. Our work investigates oncogenic cooperation between FGF19, or its analogs, and pathways frequently mutated in HCC.

**Material and Methods**

We performed in vivo hepatocytes transfection by hydrodynamic gene transfer (HGT) in C57BL/6J mice to combine overexpression of FGF19, FGF15, FGF19 analog (Aldafermin) with oncogenic events commonly found in HCC (p53 inactivation, MYC overexpression, Wnt/ b-catenin pathway activation). Alternatively, systemic administration of FGF19 and Aldafermin recombinant proteins were performed. Tumours were analyzed by immunohistochemistry, RT-qPCR, RNAseq.

**Results and Discussions**

Our data reveals a significant oncogenic collaboration between MYC and FGF19 (or FGF15), fostering aggressive hepatocarcinogenesis. Importantly, we demonstrate that the FGF19 analogue Aldafermin (NGM282) maintains its oncogenic potential within this framework. Tumors induced by Aldafermin+MYC and FGF19+MYC exhibit virtually identical transcriptomic profiles. Notably, the oncogenic effects of both the hormone and its analogue are evident after brief systemic administration of recombinant proteins. Therefore, we ascertain that the FGF19 analogue Aldafermin preserves its oncogenic attributes, at least in the context of MYC overexpression.

**Conclusion**

Our study identifies a robust oncogenic synergy between the FGF19/FGF15 hormone and MYC, propelling hepatocarcinogenesis. The retention of oncogenic properties by the FGF19 analogue Aldafermin raises concerns regarding its potential use in patients with compromised, mutation-prone livers.

### EACR2024-0622

### Identifying responders to high dose testosterone treatment with patient-derived models of advanced prostate cancer

*M. Lawrence[1], G. Cuffe[2], S. KeerthiKumar[3], R. Quezada-Urban[2], N. Choo[2], S. Luke[4], M. Crumbaker[5], R. Taylor[6], G. Risbridger[2], A. Joshua[5]*

*[1]Monash University, Department of Anatomy and Developmental Biology- Biomedicine Discovery Institute, Clayton, Australia*

*[2]Monash University, Department of Anatomy and Developmental Biology- Monash Biomedicine Discovery Institute, Clayton, Australia*

*[3]Peter MacCallum Cancer Centre, Cancer Research Division, Melbourne, Australia*

*[4]Flinders University, College of Medicine and Public Health, Adelaide, Australia*

*[5]Garvan Institute of Medical Research- St Vincent's Hospital, Kinghorn Cancer Centre, Sydney, Australia*

*[6]Monash University, Department of Physiology- Monash Biomedicine Discovery Institute, Clayton, Australia*

**Introduction**

The standard-of-care for advanced prostate cancer is ongoing suppression of androgen receptor (AR) activity. However, tumours eventually develop diverse mechanisms of resistance, and patients endure mounting side-effects. Bipolar Androgen Therapy (BAT) is a potential new treatment for castration resistant prostate cancer. Instead of continued AR suppression, patients receive testosterone injections to cycle between low

Molecular Oncology 18 (Suppl. 1) (2024) 1–495

© 2024 The Authors. Molecular Oncology is published by John Wiley & Sons Ltd on behalf of Federation of European Biochemical Societies

(castrate) and high (supraphysiological) levels of testosterone. Clinical trials show that ~30% of tumours respond to BAT. It is unclear why some tumours are more responsive than others. Therefore, our goal was to use patient-derived xenografts (PDXs) to compare the efficacy of BAT in different cases of metastatic advanced prostate cancer.

**Material and Methods**

We used 10 PDXs from the Melbourne Urological Research Alliance (MURAL). They spanned diverse forms of prostate cancer from patients who progressed on current treatments, including AR signalling inhibitors, chemotherapy and radioligand therapy. PDXs were treated with vehicle or BAT (testosterone cypionate injections) for up to three cycles. Serum testosterone (mg/dl) was measured on day 1, 5, 7, 14. The abundance and localisation of the full-length androgen receptor (AR) and AR-variants were examined using immuno-histochemistry. Transcriptional responses were evaluated using bulk and single cell RNAseq.

**Results and Discussions**

Changes in serum testosterone were consistent with patients in BAT clinical trials. Three of ten of PDXs (30%) were sensitive to BAT, reflecting response rates in clinical trials. BAT-sensitive PDXs included tumours with high copy number amplifications of the *AR* gene, and low levels of AR-variants. BAT-resistant PDXs included tumours with genomic structural rearrangements of the *AR* and/or expression of AR-variants. Bulk and single-cell RNA sequencing revealed that acute testosterone treatment induced widespread transcriptional changes. This occurred regardless of intratumoural heterogeneity and in both sensitive and resistant tumours, except those with genomic structural rearrangements.

**Conclusion**

Patient-derived models of prostate cancer have varying responses to BAT, similar to patients in trials. BAT responsiveness is linked to the status of *AR* alterations. The widespread transcriptional responses we observed highlight the opportunities to increase response rates to BAT by targeting convergent signalling pathways with combination therapies.

### EACR2024-0623

### The adapter protein Miro1 modulates horizontal transfer of mitochondria in cancer

*Z. Nahacka*[1], *J. Novak*[1,2], *G. Lopes Oliveira*[1,3,4,5], *P. Brisudova*[1,2], *M. Dubisova*[1,2], *S. Dvorakova*[1], *R. Zobalova*[1], *M.V. Berridge*[6], *J. Rohlena*[1], *J. Neuzil*[1,7,8]

[1]*Institute of Biotechnology- CAS, Laboratory of Molecular Therapy, Vestec, Czech Republic*
[2]*Charles University, Faculty of Science, Prague, Czech Republic*
[3]*Institute for Interdisciplinary Research- University of Coimbra, Doctoral Programme in Experimental Biology and Biomedicine PDBEB, Cantanhede, Portugal*
[4]*University of Coimbra, Centre for Innovative Biomedicine and Biotechnology, Cantanhede, Portugal*
[5]*University of Coimbra, Center for Neuroscience and Cell Biology, Cantanhede, Portugal*
[6]*Malaghan Institute of Medical Research, Berridge Laboratory, Wellington, New Zealand*
[7]*Charles University, 1st Faculty of Medicine, Prague, Czech Republic*
[8]*Griffith University, School of Pharmacy and Medical Science, Southport- Qld 4222, Australia*

**Introduction**

Recent research has revealed that cancer cells lacking mitochondrial DNA (ρ0 cells) acquire mitochondria containing their DNA payload from tumor stromal cells to restore respiration in ρ0 cells, thereby facilitating tumor formation. However, little is understood about the molecular mechanism driving this phenomenon. In this study, we investigated the involvement of Miro1, a small GTPase localized in the mitochondrial outer membrane, which is known to be engaged in the movement of mitochondria along microtubules, originally observed in neuronal cells.

**Material and Methods**

Material: mouse model of whole-body knockout of Miro1, Miro1$^{KO}$ mice decorated with mKate2 far-red fluorescent protein, cell lines with or without expression of Miro1 (MEFs, MSCs, B16, B16 ρ0). B16 ρ0 tumor derived cell lines (D10, D15, D20). Methods: fluorescent cytometry, high-end confocal microscopy, TIRF microscopy, electron microscopy, ATP (luminescent assay), mitochondrial respiration (Oxygraph-2k respirometer, Seahorse analysis), PCR, qPCR, western blotting, DNA electrophoresis.

**Results and Discussions**

Using inducible Miro1 knockout (Miro1$^{KO}$) mice, we observed a significant delay in tumor formation upon grafting B16 ρ0 cancer cells due to absence of the adaptor protein in tumor stroma. Further examination using Miro1$^{KO}$ mice transgenic for mitochondria decorated with mito::mKate2 revealed that this delay stemmed from impaired mitochondrial transfer from the stroma to grafted B16 ρ0 cells, consequently slowing the recovery of mitochondrial respiration. Depletion of Miro1 resulted in the accumulation of mitochondria in the perinuclear compartment and destabilization of the mitochondrial network. Our investigation employing electron and light microscopic techniques demonstrated the impact of Miro1 absence on the association of mitochondria with microtubules, leading to compromised mitochondrial transfer via tunneling nanotubes connecting stromal and cancer cells and acting as conduits for horizontal mitochondrial transfer.

**Conclusion**

This report marks the first identification of the role of the adaptor protein Miro1 in HMT in vivo and its involvement in TNTs.

### EACR2024-0630

### Investigating the contributions of tumour microbiome associated bacterial load in the pathobiology of head and neck squamous cell carcinoma

*K. Fenix*[1], *G. Bouras*[1], *K. Yeo*[1], *R. Valentine*[2], *S. Vreugde*[1], *A. Psaltis*[1]

[1]*The University of Adelaide, Department of ENT Surgery- Adelaide Medical School, Adelaide, Australia*
[2]*Central Adelaide Local Health Network, Department of*

© 2024 The Authors. Molecular Oncology is published by John Wiley & Sons Ltd on behalf of Federation of European Biochemical Societies

*ENT Surgery- Royal Adelaide Hospital, Adelaide, Australia*

**Introduction**

Head and neck squamous cell carcinoma (HNSCC) is the 7th most common type of cancer world-wide, leading to 450,000 deaths annually. Previous studies have shown the possibility to extract intra-tumoural microbiome signatures from public tumour WGS datasets and from 16S rRNA sequencing of cancer tissues. However, these studies commonly consider the effects of relative microbial abundances, not total bacterial load.

**Material and Methods**

Independent multiomic datasets from 118 Human Papilloma Virus negative HNSCC patients from the Cancer Genome Atlas (TCGA) and 110 patients from the Clinical Proteomic Tumor Analysis Consortium (CPTAC) were analysed by analysing all tumour WGS sequencing reads that did not map to the human genome. Likely contaminant species were removed based on sequencing centre batch effects. Bacterial load was calculated as the proportion of classified microbial reads to total library read depth. Bacterial load was integrated with matching transcriptomic, proteomic and survival datasets using non-matrix factorisation (TCGA) and variational autoencoder (CPATC) based approaches. A further validation cohort of 56 HNSCC patient tissues collected from South Australia was assessed for bacterial load by digital PCR with matched 16S rRNA sequencing and bulk RNA sequencing.

**Results and Discussions**

Low intratumoural bacterial load was associated with mesenchymal tumour phenotypes based on transcriptomic and proteomic signatures and was negatively associated with 3 and 5-year patient survival. Higher bacteria load tumours were characterised by the presence of common oral microbes *Fusobacteria*, *Treponema*, *Prevotella*, *Streptococcus*, epithelial tumour phenotypes and significantly higher survival.

**Conclusion**

These results indicate that quantitative microbiome approaches may reveal underappreciated insights to the cancer pathobiology of HNSCCs.

### EACR2024-0631

### Unveiling the Impact of Diffuse Intrinsic Pontine Glioma (DIPG) on Blood-Brain Barrier Integrity and Drug Penetration

C. Ung[1], D. Upton[1], V. Pooja[2], A. Salerno[3], R. Pandher[4], C. Mayoh[2], O. Vittorio[3], M. Tsoli[1], D. Ziegler[1,5]

[1]*Children's Cancer Institute, Brain tumour group, Kensington, Australia*

[2]*Children's Cancer Institute, Bioinformatics, Kensington, Australia*

[3]*University of New South Wales, School of Biomedical Sciences, Kensington, Australia*

[4]*Children's Cancer Institute, Experimental therapeutics and molecular oncology group, Kensington, Australia*

[5]*Sydney Children's Hospital, Kid's Cancer Centre, Randwick, Australia*

**Introduction**

DIPG, an incurable pediatric brainstem tumor, poses a formidable challenge to treatment due to the blood-brain barrier (BBB) hindering drug penetration. Our research, alongside others', have demonstrated the preservation of BBB integrity in DIPG. It is speculated that the brainstem BBB might be even less permeable in the presence of DIPG tumor cells.

**Material and Methods**

We employed in vivo orthotopic patient-derived xenograft DIPG models to investigate differences in drug efficacy with DIPG implanted in the cortex or brainstem, using survival, pharmacokinetic and immuno-histochemical analysis. We then proceeded with single-cell RNA sequencing to explore alterations in brainstem vasculature with DIPG compared to normal brainstem, and subsequently tested novel BBB modulators in vitro and in vivo to determine their effect on permeability.

**Results and Discussions**

We found greater efficacy of mTOR inhibitor temsirolimus in DIPG injected in the cortex compared to in the brainstem, with markedly prolonged survival rates, elevated drug levels, and reduced proliferating tumor cells. Additionally, we found regional variation in temsirolimus penetration in the cortex and brainstem of normal brain. Transcriptomic analysis of microvascular endothelial cells isolated from the brainstem, where DIPG cells or Matrigel were implanted, revealed significant downregulation of inflammatory pathways and antigen presentation. Apoptotic pathways were significantly upregulated, including MCL-1. Thus, MCL-1 inhibitor, S63845, was investigated as a novel brainstem BBB modulator. We found that it significantly reduced permeability in an in vitro BBB transwell model and decreased claudin-5 expression. A more potent and selective MCL-1 inhibitor, S64315, also exhibited effects on BBB permeability in vitro, with significant changes to ZO-1 and claudin-5 expression. In vivo, a single dose of S63845 increased Texas-red dextran permeability in DIPG models, indicating BBB opening. Toxicity studies have shown that repeated doses of S63845 with temsirolimus over 6 weeks is well tolerated. Therapeutic studies are currently being conducted to determine whether opening the BBB with MCL1 inhibition leads to improved treatment efficacy.

**Conclusion**

Overall, this study shows the direct impact of DIPG on endothelial cell pathways leading to a tightened BBB and diminished therapeutic effectiveness. MCL-1 inhibitors may facilitate delivery of therapeutics by opening the BBB. This discovery has the potential to enhance the efficacy of anti-DIPG therapeutics.

### EACR2024-0673

### Discovery of DN029003, a highly potent, selective, and orally bioavailable pan-KRAS inhibitor

M. Li[1], Z. Tong[1], J. Ren[1], H. Yang[1], Y. Bi[1], Y. Gao[1], Y. Ru[1], J. Wang[1], P. Wang[1], S. Yan[1]

[1]*Denovo pharmatech, Cancer biology, Shanghai, China*

**Introduction**

KRAS is one of the most commonly mutated proteins in cancer and is a carcinogenic driver of non-small cell lung, pancreatic, colorectal, and other cancers. The cycling between active (GTP-bound) and inactive (GDP-bound) states of KRAS GTPase activity is disrupted by

© 2024 The Authors. Molecular Oncology is published by John Wiley & Sons Ltd on behalf of Federation of European Biochemical Societies mutations in the KRAS allele, leading to its hyper-activation in the active GTP-bound state rather than the inactive GDP-bound state. Direct inhibition of KRAS function has been studied for decades, the next-generation KRAS inhibitors aiming to target multiple oncogenic KRAS mutations while avoiding inhibition of wild-type (WT) KRAS, potentially offering greater activity and improved safety. Herein we describe Denovo DN029003, a novel, potent and orally available small molecule pan-KRAS inhibitor. DN029003 binds non-covalently to multiple KRAS mutant alleles, including G12C, G12D and G12V, in the GTP-bound state and thereby disrupts oncogenic signaling pathways.

**Material and Methods**

Compound potency and selectivity were measured using homogeneous time-resolved fluorescence (HTRF) assays and cell-based assays measuring inhibition of p-ERK and cell growth of KRAS-mutant tumor cell lines. In vivo pharmacokinetic studies in mice and monkeys are the main approaches for evaluating and predicting the pharmacokinetics of DN029003. Tumor growth inhibition and PK/PD studies were performed in mice.

**Results and Discussions**

DN029003 treatment disrupted KRAS-G12C:RAF1, KRAS-G12D:RAF1, KRAS-G12V:RAF1 protein-protein interaction in a concentration-dependent manner. DN029003 demonstrated $IC_{50}$ values ranging from 0.25-1.1nM for KRAS-G12C, G12D, G12V in cell proliferation inhibition studies, and selectivity >2000 fold over KRAS WT in phospho-ERK cell-based assays and cell proliferation inhibition assays. DN029003 demonstrate favorable in vitro ADME properties and oral bioavailability in preclinical species. In vivo, DN029003 administered orally demonstrated dose-dependent target inhibition in G12C, G12D, G12V-mutant xenograft models.

**Conclusion**

These data indicated that our pan-KRAS inhibitor can effectively and selectively inhibit KRAS G12C, G12D, and G12V mutations. We hypothesize that this potency and selectivity, along with high oral bioavailability, will provide efficacy and tolerability for cancer patients driven by KRAS mutations.

### EACR2024-0683

### The role of Rho-Kinase in Diffuse Midline Glioma cell dissemination

*G. Pericoli[1], S. Pires Celeiro[1,2,3], M. Pellegrino[1], E. de Billy[1], G. Zanni[4], M. Vinci[1]*

*[1]Paediatric Cancer Genetics and Epigenetics Research Unit, Bambino Gesù Children's Hospital-IRCCS, Rome, Italy*

*[2]ICVS/3B's-PT, Government Associate Laboratory, Guimarães - Braga, Portugal*

*[3]Life and Health Sciences Research Institute ICVS, School of Medicine - University of Minho - Campus of Gualtar, Braga, Portugal*

*[4]Unit of Neuromuscular and Neurodegenerative Disease, Bambino Gesù Children's Hospital-IRCCS, Rome, Italy*

**Introduction**

Diffuse Midline Glioma H3K27-altered (DMG) is an aggressive tumor of the Central Nervous System (CNS) that affects children and adolescents with a very poor prognosis. DMG can arise in the pons, in the thalamus or other midline structures. Its highly infiltrative capacity causes the spreading of tumor cells through the CNS though the processes of DMG cell dissemination are still poorly understood. The Rho associated protein kinase (ROCK) plays a role in different cellular function, including cell contraction and actin organization, cell migration, invasion and neuronal architecture. However, little is known about ROCK in DMG. Our aim is to investigate the role of ROCK in DMG cell motility.

**Material and Methods**

Three different DMG primary patient-derived cell lines were used in this study. Cells were cultured in 3D as neurospheres and basal levels of Rho, Rac, ROCK, MYPT1 and P-MYPT1 were evaluated by western blot. The expression of the ROCK target, MYPT1 and P-MYPT1, was also assessed by immunofluorescence in 3D migration and invasion. Three different ROCK inhibitors (HA1077, Y27632, GSK429286) were used to evaluate their effects on DMG cell viability, migration and invasion as well as on the regulation of ROCK signaling pathway. The lysophosphatidic acid (LPA), was used as a positive control of ROCK pathway activation.

**Results and Discussions**

At basal level the three DMG cell lines express Rho, Rac, ROCK and MYPT1 but do not seem to express P-MYPT1. However, P-MYPT1 appears to be expressed in cells in 3D migration and invasion assays. Moreover, upon treatment with ROCK inhibitors, DMG cells showed a decrease of P-MYPT1 expression in 3D cell invasion, compared to untreated controls. The inhibition of ROCK did not affect DMG cell viability but interestingly increased their migration and invasion capacity. Conversely, LPA treatment appeared to decrease the motility of DMG cells.

**Conclusion**

The modulation of ROCK activity affects the migratory and invasive capacity of DMG cells in vitro. Our study provides evidence on the role of ROCK as a player in DMG cell motility and suggest that the modulation of ROCK signaling pathway should be evaluated as therapeutic option for controlling DMG disseminative growth.

### EACR2024-0688

### Leptin induces vasculogenic mimicry in MDA-MB-231 human breast cancer cells

*E.O. Lee[1,2], D.S. Han[1]*

*[1]Kyung Hee University, Department of Science in Korean Medicine- College of Korean Medicine- Graduate School, Seoul, South Korea*

*[2]Kyung Hee University, Department of Cancer Preventive Material Development, College of Korean Medicine, Graduate school, Seoul, South Korea*

**Introduction**

Leptin is a peptide hormone released from adipose tissue and plays an important role in regulating energy homeostasis. Obesity is a risk factor for breast cancer in postmenopausal women. Leptin has been implicated as a link between obesity and breast cancer. Vasculogenic mimicry (VM) is the de novo formation of matrix-rich vascular-like networks by aggressive and metastatic

© 2024 The Authors. Molecular Oncology is published by John Wiley & Sons Ltd on behalf of Federation of European Biochemical Societies cancer cells without endothelial cells. This study investigated the role of leptin in VM in MDA-MB-231 human breast cancer cells.

**Material and Methods**

Transwell invasion and three-dimensional culture VM tube formation assays were performed to determined invasive ability and vessel-like networks formation, respectively. VM-related molecules were checked by western blot.

**Results and Discussions**

Leptin dramatically increased invasive ability and formed vessel-like networks. Leptin activated STAT3 signaling and upregulated the expressions of VM-related molecules such as vascular endothelial cadherin, twist, matrix metalloproteinase-2 and laminin subunit 5 gamma-2 protein expressions. However, VM was impaired by treating leptin receptor blocking peptide or AG490, JAK/STAT3 inhibitor. Also, these inhibitors down-regulated the VM-related protein expressions.

**Conclusion**

These results demonstrated that leptin triggered VM formation of breast cancer cells via leptin receptor/STAT signaling pathway.

### EACR2024-0693
### Insulin-like Growth Factor Receptor Type-1 in Schwann Cells Regulates Pain in a mouse model of Metastatic Bone Cancer Pain

*L. Landini*[1], *M. Marini*[1], *D. Souza Monteiro de Araujo*[1], *M. Montini*[2], *M. Titiz*[1], *P. Geppetti*[1], *R. Nassini*[1], *F. De Logu*[1]

[1]*University of Florence, Department of Health Sciences, Florence, Italy*

[2]*University of Florence, Department of Experimental and Clinical Biomedical Sciences "Mario Serio", Florence, Italy*

**Introduction**

Pain is a recurrent symptom of cancer often associated to bone metastases, which is a common consequence of many primary tumors, including breast cancer. Although bone resorption associated with the invasion of cancer cells has been considered as the primary cause of Metastatic Bone Cancer Pain (MBCP), knowledge of the cellular and molecular mechanisms underlying MBCP is limited.

**Material and Methods**

C57BL6/J females mice were inoculated with E0771 breast carcinoma cells ($2 \times 10^5$ cells) that were injected into the fourth right mammary fat pad of the mouse. Control groups were injected with killed E0771 breast carcinoma cells ($2 \times 10^5$ cells). Transgenic Macrophage Fas-Induced Apoptosis (MaFIA) mice or mice with selective silencing of Insulin-like Growth Factor Receptor Type-1 (IGF1-R) or transient receptor potential ankyrin 1 (TRPA1) in Schwann Cells (Plp-CreERT$^+$ or Plp-CreERT$^-$) or in sensory neurons (Adv-Cre$^+$ or Adv-Cre$^-$) were also used. Hind paw Mechanical Allodynia (HMA) was evaluated by applying von Frey filaments of increasing stiffness applied to the plantar surface of the mouse hind paw. Some mice were treated systemically with receptor, channel, or enzyme inhibitors. Spontaneous locomotor activity, measured as the cumulative distance traveled, and time spent in the inner zone in the open field apparatus was also analyzed in some experimental sets.

**Results and Discussions**

In mice, the femur metastasis caused by intramammary inoculation of breast cancer cells resulted in IGF-1 increase in femur and sciatic nerve, and IGF-1-dependent stimulus evoked pain-like behaviors. Adeno-associated virus-based shRNA selective silencing of IGF-1R in Schwann cells or systemic administration of the IGF-1R inhibitor, picropodophyllin (PPP), attenuated pain-like behaviors. The IGF-1R activation in Schwann cells results in nitric oxide (NO) production that targets Schwann cell TRPA1. Activated TRPA1 releases macrophage-colony stimulating factor (M-CSF) that promotes endoneurial macrophage expansion and the ensuing reac

**Conclusion**

In the present MBCP model, E0771 breast carcinoma cells growing in the femur metastasis release osteoclasts to liberate IGF-1. Schwann cell IGF-1R signaling promoted an endothelial nitric oxide synthase-mediated TRPA1 activation and release of ROS that, via M-CSF-dependent endoneurial macrophage expansion, sustained pain-like behaviors.

### EACR2024-0701
### The role of genetic predispositions in the context of breast cancer tumor microenvironment

*A. Čumová*[1], *M. Buríková*[1], *J. Plavá*[1], *L. Trnková*[1], *M. Boháč*[2], *N. Novotná*[2], *L. Rojíková*[1], *M. Cihová*[1]

[1]*Cancer Research Institute, Department of Molecular Oncology, Bratislava, Slovakia*

[2]*National Cancer Institute, Department of Oncosurgery, Bratislava, Slovakia*

**Introduction**

Breast cancer (BC) is the second most commonly diagnosed cancer worldwide with more than 2.3 million new cases diagnosed in 2022. It is estimated that about 5-10% of all BC are caused by hereditary mutations in DNA damage response (DDR) genes such as *BRCA1/2*, *CHEK2* or *TP53*, and are associated with worse prognosis and more aggressive phenotype. The role of these mutations in cancer cells has been intensively studied, however, less is known about the role of these genetic predispositions in the context of tumor micro-environment. Our main aim was to elucidate the effect of DDR gene mutations in mesenchymal stromal cells (MSCs), which are present in the breast adipose tissue and interact with breast cancer cells, on cancer progression and invasion in vivo.

**Material and Methods**

Immunodeficient SCID/beige mice were orthotopically injected with BRCA1-deficient breast invasive ductal carcinoma cell line SUM149PT mixed with MSCs (ratio 2:1) from donors with different mutations undergoing prophylactic mastectomy or having previous cancer history (MSCs from a healthy donor, *BRCA1*mut MSCs from a triple-negative BC patient, *BRCA2*mut MSCs, *CHEK2*mut MSCs, *BRCA1/CHEK2*mut MSCs, MSCs from a BC patient without any mutation, and

Molecular Oncology 18 (Suppl. 1) (2024) 1–495

© 2024 The Authors. Molecular Oncology is published by John Wiley & Sons Ltd on behalf of Federation of European Biochemical Societies

MSCs from a recovered BC patient in remission) or injected with SUM149PT cells only. Mice were sacrificed after 49 days. We measured tumor volume and weight, examined tumor invasiveness and angiogenesis with immunohistochemical (IHC) analysis, and compared the gene expression in tumors using The Human Epithelial to Mesenchymal Transition (EMT) RT² Profiler PCR Array® (Qiagen).

**Results and Discussions**

Tumors in the group injected with SUM149PT cells and *CHEK2*mut MSCs or *BRCA1/CHEK2*mut MSCs displayed significantly higher weights (<0.05). IHC analysis of tumors revealed the overexpression of vimentin (a marker of EMT and invasiveness) and CD31 (a marker of angiogenesis) in the groups co-injected with SUM149PT cells and mutated MSCs compared to tumors in the groups co-injected with healthy MSCs or with SUM149PT cells only. Gene expression analysis revealed an overexpression of several genes associated with EMT, invasiveness, and aggressive metastatic phenotype.

**Conclusion**

Our findings demonstrate that stromal cells in breast tissue harboring mutations in DDR genes drive tumor progression and invasiveness and may be responsible for the aggressive phenotype of hereditary breast cancer. This underlines the crucial role of tumor micro-environment in breast cancer carcinogenesis.

### EACR2024-0704

### A reproducible and scalable ex vivo patient-derived tumor fragment platform

*S. Ernst[1,2,3], E. Blondeel[2,3], S. Gijsels[2,3], S. Vermeulen[2,3], F. De Vuyst[2,3], W. Ceelen[1,3], O. De Wever[2,3]*

*[1]Laboratory of Experimental Surgery, Department of Human Structure and Repair, Ghent, Belgium*

*[2]Laboratory of Experimental Cancer Research, Department of Human Structure and Repair, Ghent, Belgium*

*[3]Cancer Research Institute Ghent, Ghent University, Ghent, Belgium*

**Introduction**

Ample promising preclinical studies fail after translation to the clinic, forming a time and financial burden on researchers and society. This can in part be explained by biological differences between preclinical animal models and humans, a gap that ideally should be bridged to overcome unnecessary clinical trials. A way to tackle this is to study treatment responses on patient-derived material. Existing models lack characteristics of in vivo tumors. The use of ex vivo patient-derived tumor fragments (PDTFs) is gaining momentum in the field since it mimics physiology (a gradient of nutrients and oxygen), a tumor microenvironment (TME) containing extracellular matrix, immune cells, cancer-associated fibroblasts, and spatial context. However, further study is needed to investigate model fidelity, reproducibility and scalability.

**Material and Methods**

From patients providing informed consent, fresh surgical specimens were obtained to produce ex vivo PDTFs. Patients with different primary tumors were included, i.e. colorectal cancer, ovarian cancer and soft tissue sarcoma. Manual dissection versus semi-automated processing of the samples were compared in terms of reproducibility and scalability. Metabolic assays, histology, flow cytometry and bulk RNA-sequencing were performed on our PDTFs to study the optimal medium type, cryo-preservation method, culture length, TME retainment and shape.

**Results and Discussions**

Processing the tumor nodules using our semi-automated platform significantly reduces the size of our PDTFs and increases reproducibility and scalability. We consistently create PDTFs with a volume of ±0,125 $mm^3$ (0.5x0.5x 0.5mm). Using metabolic assays, we demonstrate survival for at least 14 days, and we prioritize one medium type that consistently shows the highest number of metabolically active/live PDTFs. Monitoring morphology reveals a progressively contractile phenotype positively correlating with PDTF viability. Immunohistochemistry and flow cytometry analyses demonstrate that the major TME cell types remain alive in our PDTFs for multiple days. Bulk RNA-sequencing on our PDTFs at different time points highlights minor differences. Finally, treatments were tested on our PDTFs, resulting in clinically relevant responses.

**Conclusion**

We have established a reproducible and scalable 3D ex vivo platform that recapitulates the TME of the patient tumor for multiple days. Treatment experiments demonstrate clinical relevance, meaning our PDTF platform shows potential for functional personalized medicine.

### EACR2024-0705

### Clostridium sporogenes colonizes 3D necrotic cancer spheroids; a bridge from in vitro to in vivo

*J. van de Laak[1], M. Yildiz[1], Y. Zhang[1], A. Mowday[2], L. Dubois[1], J. Theys[1]*

*[1]Maastricht University - GROW Research Institute for Oncology and Reproduction, Precision Medicine, Maastricht, The Netherlands*

*[2]The University of Auckland, Auckland Cancer Society Research Centre, Auckland, New Zealand*

**Introduction**

Most solid cancers contain areas of hypoxia and necrosis, which are associated with poor prognosis. Strikingly, this necrotic environment provides an opportunity for targeted therapy, such as the anaerobic bacteria *Clostridium sporogenes (C. sporogenes)*. Upon injection as spores, this species selectively penetrates necrotic tumor areas and germinates into vegetative bacteria eliciting anticancer effects inside the tumor. We have developed innovative genetic tools to “arm” *C. sporogenes* with various reporter and/or therapeutic genes. These novel strains can be generated effectively in a short period of time, and it would be essential to validate them in robust systems prior to in vivo studies. The aim of this study was to develop an 3D in vitro validation method that mimics tumor necrosis as a bridge from in vitro to in vivo.

**Material and Methods**

Lewis Lung Carcinoma (LLC) and CT26 cell lines were used to generate spheroids. Microscopic images were

© 2024 The Authors. Molecular Oncology is published by John Wiley & Sons Ltd on behalf of Federation of European Biochemical Societies taken daily to follow spheroid growth. Spheroids were first investigated for the development of necrotic area. Next, spores were added to the spheroid medium and intra-spheroid colonization was followed daily. Additionally, a panel of antibiotics was investigated on their ability to clear intra-spheroid colonization. Furthermore, a *C. sporogenes* strain expressing the fluorescent reporter gene unaG was generated and investigated as a live imaging tool.

**Results and Discussions**

Both spheroid models developed a necrotic core around day 5 post-seeding, with LLC spheroids reaching a volume two-fold larger compared to CT26 spheroids. When spores were added, colonization was detected for both models within 24 hours post-administration, with colonization levels up to $2.5x10^5$ and $1,75x10^6$ colony forming units (CFU)/spheroid for LLC and CT26, respectively. Colonized LLC spheroids started to disintegrate around 96 hours post-spore addition. When antibiotics were added 24h post-spore addition, CFU/spheroid decreased over time up to undetectable levels and spheroid disintegration did not occur. CT26 spheroids remained stable and intra-spheroid colonization was detected during the whole experiment. The fluorescent unaG strain was live imaged in the spheroid model, allowing live detection of intra-spheroid colonization.

**Conclusion**

Our results show the high potential of 3D in vitro models that can potentially serve as an important intermediate validation step before embarking in vivo studies. These models will be applied for testing therapeutic efficacy of our *C. sporogenes* strains.

### EACR2024-0706
### Developing an isogenic series of CRISPR edited organoids to determine transformative dependencies of KRas-mutant alleles

L. Roussel[1], X. Zhang[1], C. Mcmenemy[1], C. Cintas[1], C. Jorgensen[1]

[1]*Cancer Research UK Manchester Institute, Systems Oncology, Manchester, United Kingdom*

**Introduction**

KRas is one of the most frequently mutated oncogenes in cancers, observed in over 20% of all cancers. Mutated KRas is particularly frequent in pancreatic, colon and lung cancer, in which mutations usually affect the G12 codon. While many amino acid substitutions have been identified in tumour tissues, the G12D, G12V and G12C mutations represent over 80% of all G12 mutant. However, the distribution of these mutant is heavily variable depending on the tissue of origin. In the context of pancreatic cancer, the G12D and G12V mutant are the most common mutations followed by the G12R mutations at 13%. However in colorectal or lung adeno-carcinoma G12R occur only at 1-2%. To determine whether tissue specific signalling compensation occurs we have used CRISPR-Cas9 to engineer isogenic KRas-mutant in organoids from the pancreas of C57B/L6 mice.

**Material and Methods**

We electroporated one organoid line, derived from the pancreas of a wild-type C57B/L6 mice, with guide RNAs for both KRas and p53 complexed with CRISPR-Cas9 with different single-stranded oligodeoxynucleotides donor template to generate an isogenic series of six KRas mutants with loss of p53. We selected the line with a combination of Nutlin-3 and Erlotinib to enrich our pool population for KRas mutant and loss of p53. Following this, we orthotopically injected these lines into C57B/L6 mice and followed tumour growth in the pancreas with ultrasound. New organoid lines were generated from these mice to perform transcriptomic analysis both pre- and post-injection.

**Results and Discussions**

Following pancreatic orthotopic injection in-vivo, we found that some mutant fail to develop invasive tumours, showing only a cystic phenotype within the study length. Transcriptomic analysis revealed an enrichment of immune-related pathway in the lines which fail to show an invasive phenotype. Our transcriptomic data has helped refine a list of receptors which are differentially enriched that we are currently testing functionally.

**Conclusion**

Overall, we have generated an isogenic series comprised of 6 common KRas mutants found in patients with different propensity to develop invasive pancreatic cancer. This isogenic series include both lines which are neoplastic as well as tumour derived organoid lines. Our future work will look at employing single-cell RNA sequencing to analyse how the transcriptome of the different KRas mutant change as well as the how the microenvironment changes after in-vivo orthotopic injection.

### EACR2024-0712
### The dual role of the cellular prion protein in glioma

S. Portelli[1], P. Swainsbury[1], T. Mantamadiotis[1], V.A. Lawson[1]

[1]*The Peter Doherty Institute, Microbiology and Immunology, Melbourne, Australia*

**Introduction**

Grade 4 glioma or glioblastoma (GBM) is the most aggressive and frequent brain tumour. Current treatments are unable to overcome poor patient prognosis and GBM recurrence, necessitating an investigation of alternate biomarkers or therapeutic strategies. This study explores the novel role of the cellular prion protein ($PrP^C$) in glioma progression, following findings correlating increased $PrP^C$ expression with poor prognosis, recurrence, and therapy resistance in several cancers. Whilst the perturbation of $PrP^C$ physiological functions have been linked to cancer hallmarks, the underlying mechanisms are not well defined, including how these roles differ in low versus high grade cancers.

**Material and Methods**

To investigate the role of $PrP^C$ in glioma development, the influence of $PrP^C$ expression on patient clinical progression was assessed in a publicly available RNA sequencing low-grade glioma (LGG) and GBM dataset. In addition, knockout (KO) and wild-type (WT) expression of the $PrP^C$ gene (*Prnp*) was established in a low- and high-grade glioma (GBM) mouse model,

© 2024 The Authors. Molecular Oncology is published by John Wiley & Sons Ltd on behalf of Federation of European Biochemical Societies involving hyperactivation of the PI3K pathway, via expression of the *Pik3ca*$^{H1047R}$ oncogenic mutant and/or deletion of the tumour suppressor gene *Pten* in neural stem cells (NSCs). The effect of $PrP^C$ expression on cancer cell biology was assessed in vitro in NSCs isolated from this model.

**Results and Discussions**

Reduced expression of the $PrP^C$ gene (*Prnp*) in low-risk GBM patients was associated with improved survival. These findings were reflected in NSCs isolated from $PrP^C$ KO and WT GBM (*Pik3ca-Pten*) mice, with a significant decrease in cell proliferation and viability observed in $PrP^C$ KO derived NSCs. Whilst these findings indicate a tumour-promoting role for $PrP^C$ in GBM, the opposite was observed in the LGG model. *Prnp* expression was instead increased in low-risk LGG patients, however this did not correlate with survival. Moreover, in the LGG model with *Pten* deletion alone, $PrP^C$ ablation (KO) significantly enhanced NSC proliferation, stemness and invasiveness in vitro.

**Conclusion**

These results indicate both a suppressive and promoter role for $PrP^C$ in glioma, which may be contingent on the genetic context or grade of tumours. Understanding the role of novel factors such as $PrP^C$ in glioma may aid in the development of novel biomarkers or therapies to complement current ineffective treatments. Spatial transcriptomics analysis of tumours will be undertaken to further interrogate the dual role of $PrP^C$ in glioma.

### EACR2024-0714
### Inhibition of HDAC6 attenuates M2 polarization of macrophages by breast cancer cells in hypoxic tumor microenvironment

*U. BAGCHI[1], S. Kapoor[1], R. Dash[2]*
*[1]Amity University, Amity Institute of Molecular Medicine and Stem Cell Research, Noida, India*
*[2]Institute of Life Sciences- Nalco Square, Institute of Life Sciences, Bhubaneshwar, India*

**Introduction**

Tumor associated macrophages (TAMs) are essential for tumor progression and metastasis. Although several studies have unraveled the role of TAMs in modulating breast cancer cells, how TAMs are modulated within breast tumors is only marginally known. The hypoxic niche in solid tumors mediates its transcriptional responses, via activation of Hypoxia Inducible Factor-1(HIF-1α). Clinicopathological studies correlate that breast cancer patients with elevated HIF-1α gene signatures showed 40% greater risk of recurrence and mortality. HDAC6, a cytoplasmic deacetylase frequently upregulated in the breast tumor, modulates the chaperone activity of Heat shock protein 90 (Hsp-90) which regulates the stability of HIF-1α.

**Material and Methods**

Breast cancer cells were maintained under hypoxic conditions for 48h, followed by treatment with vehicle or ricolinostat, an oral first in class isoform-selective inhibitor of HDAC6 under clinical trials [NCT02091063]. Hypoxia induced expression of HIF-1α, stem-cell, and mesenchymal marker profile was evaluated using Western blotting, and RTPCR. The polarization of M2 TAMs in presence of conditioned medium of hypoxic MDA-MB-231 cells was analyzed by immunofluorescence and RTPCR. The data was validated, in vivo using a syngeneic murine breast cancer model.

**Results and Discussions**

Our data indicates that HDAC6 inhibition in hypoxic breast cancer cells reduced the expression of HIF1α. This led to the downregulation of hypoxia mediated cellular responses, such as invasion, migration and epithelial to mesenchymal transition. Further, it reduced the expression of stem cell markers in hypoxic TNBCs. Intriguingly, hypoxia enhanced the breast cancer cells mediated M2 polarization of macrophages. However, cells treated with ricolinostat failed to polarize macrophages to protumoral M2 TAMs, indicating that HDAC6 plays a major role in driving M2 polarization by breast cancer cells. Mechanistically, ricolinostat treatment reduced hypoxia induced phosphorylation of STAT3 (pY705), AKT (pS473) and downregulated C-myc. The tumors isolated from mice treated with ricolinostat showed reduced population of M2-TAMs and reduced tumor growth lung metastasis.

**Conclusion**

Selective inhibition of HDAC6 in hypoxic breast cancer cells abrogates hypoxia induced AKT/STAT3/Cmyc signaling, and diminishes their potential to polarize monocytes into M2 TAMs, altering the immuno-suppressive properties of the tumor microenvironment. HDAC6 inhibition stands as a promising approach to treat metastatic aggressive breast cancer.

### EACR2024-0720
### Comprehensive Characterisation of the Tumor- Immune Landscape across different Mouse Models of Glioblastoma using Spatial Analysis

*D. Klymyshyn[1], N. Jhaveri[1], A. Pratapa[1], T.H. Tam[1], R. Stad[1], S. Seidel[1], N. Nelson[2], R. Dolcetti[3], D. Moi[4], R. Mazzieri[5], T. Mantamadiotis[6]*
*[1]Akoya Biosciences, Applications, Marlborough, USA*
*[2]Abcam, Commercial, Cambridge, UK*
*[3]Peter MacCallum Cancer Centre, Clinical and Translational Immunotherapy, Victoria, Australia*
*[4]University of Melbourne, Clinical and Translational Immunotherapy, Parkville, Australia*
*[5]University of Melbourne, The Sir Peter Maccallum Department of Oncology, Parkville, Australia*
*[6]University of Melbourne, Microbiology and Immunology, Parkvile, Australia*

**Introduction**

Glioblastoma (GBM) remains one of the most challenging cancers to treat. The median survival time for GBM patients is approximately 12-15 months, and the 5-year survival rate is below 10%. There is a need for improved treatment, facilitated by relevant experimental models. Preclinical animal models play a crucial role in elucidating GBM biology and assessing the effectiveness of new therapeutic approaches. Although various experimental models are employed in GBM research, mice are predominantly utilized in preclinical

© 2024 The Authors. Molecular Oncology is published by John Wiley & Sons Ltd on behalf of Federation of European Biochemical Societies investigations. In this study, we used spatial phenotyping to comprehensively characterize and compare key proteins within the brain tumor immune micro-environment (TiME). We also conducted a detailed comparison of TiME across different immune-competent GBM mouse models.

**Material and Methods**

The Phenocycler-Fusion is a fast spatial biology solution that enables ultrahigh-plex single-cell spatial readouts. This technology is used for whole slide imaging of mouse FFPE tissues and deep immune phenotyping of >40 proteins, comprising immune cell lineages, activation states and checkpoints, as well as biomarkers for tumor, vascular and neural landscapes of various GBM mouse models.

**Results and Discussions**

We isolated spatial signatures within the mouse GBM TiME using single-cell spatial phenotyping. We focused on multiple immune biomarkers, including microglia/myeloid cells that were identified via the combinatorial expression of the key markers CD68, Iba-1, F4/80, and CD11b. The macrophages and their biomarker expression profiles revealed the significant inter- and intra-tumoral heterogeneity within different mouse GBM models, indicative of the heterogeneous and complex biology of GBM.

**Conclusion**

Our study involves the development of a customized antibody panel, an imaging protocol, and a novel bio-informatic analysis technique. Implementing this method across various mouse GBM models enabled us to investigate cell populations based on their biomarker profiles and spatial distribution. This research offers a comprehensive characterization of diverse mouse GBM models, aiming to identify the most suitable model that represents the intricate TiME of human GBM. This includes features such as invasive tumor margins, high vascularity, and the blood-brain barrier. This approach could hold significant potential for a range of applications where spatial information of biomolecules is essential, enhancing our understanding of the GBM TiME.

### EACR2024-0724

### Role of squamous-like plasticity in therapy-induced colorectal cancer relapse and metastases

*N. Doleschall[1], P. Cammareri[1], C. Billard[1], K. Myant[1]*
*[1]Institute of Genetics and Cancer, CRUK Scotland Centre, Edinburgh, United Kingdom*

**Introduction**

Colorectal Cancer (CRC) is the second leading cause of cancer related deaths in Europe, due to emerging chemoresistance, high rates of relapse and ineffective therapy options for metastatic disease. The current absence of accurate and clinically relevant models of chemotherapy and relapse contribute to our lack of knowledge for the development of efficient novel therapies targeting mechanisms of relapse as well as metastatic disease progression.

**Material and Methods**

Here we describe two novel in vivo mouse models to study chemotherapy induced CRC relapse. Treatment plans were revised to mimic clinical treatment schedules using Oxaliplatin in combination with 5-fluorouracil (5FU) in immunocompetent mice bearing either subcutaneous or orthotopic CRC tumours. A combination of bulk RNAseq and antibody-based protein detection methods were used to analyse samples from these models.

**Results and Discussions**

The models developed in this study were observed to recapitulate phenotypic response of CRC patients to adjuvant chemotherapy with high accuracy. In these models we found evidence of increased invasion and metastatic potential in chemotherapy treated colonic cancers coupled with therapy-induced immune evasion. Additionally, we identified the emergence and clonal expansion of squamous cell populations in response to chemotherapy with increasing prevalence in relapsing and metastatic primary tumours. Squamous cell populations have hitherto not been reported in mouse models of CRC and are not found in the healthy colonic epithelium. However, they have been recently reported in patients with highly aggressive metastatic CRC, opening up novel avenues for investigation.

**Conclusion**

The results of this study show that adjuvant chemo-therapy has the potential to lead to more aggressive primary CRC and translates to worse patient survival outcomes. These changes are mediated by therapy-induced cell plasticity introducing non-canonical cell lineage transformations diverging from colonic epithelial identity. Targeting mechanisms of cell plasticity has the potential to complement current standard-of-care therapy options for later stage CRC.

### EACR2024-0738

### Pulsed priming with narmafotinib enhances both gemcitabine/Abraxane & FOLFIRINOX response in pancreatic cancer

*K. Murphy[1], C. Chambers[1], D. Reed[1], J. Morton[2], Y. Wang[3], C. Burns[4], T. Cox[1], M. Pajic[5], D. Herrmann[1], P. Timpson[1]*
*[1]Garvan Institute of Medical Research & The Kinghorn Cancer Centre, Tumour Ecosystems Program, Sydney, Australia*
*[2]Cancer Research UK Scotland Institute, Precision Panc, Glasgow, United Kingdom*
*[3]University of Southern California, Alfred E. Mann Department of Biomedical Engineering, Los Angeles, United States*
*[4]Amplia Therapeutics, Amplia Therapeutics, Melbourne, Australia*
*[5]Garvan Institute of Medical Research & The Kinghorn Cancer Centre, Translational Oncology Program, Sydney, Australia*

**Introduction**

Pancreatic ductal adenocarcinoma (PDAC) is a particularly lethal malignancy with few treatment options available. Extensive remodeling of extracellular matrix (ECM) generates a highly fibrotic tumor landscape, which impairs therapeutic response. Focal Adhesion Kinase (FAK) is known to regulate ECM-feedback

Molecular Oncology 18 (Suppl. 1) (2024) 1–495
© 2024 The Authors. Molecular Oncology is published by John Wiley & Sons Ltd on behalf of Federation of European Biochemical Societies response and fibrosis, presenting a compelling drug target to reduce fibrosis and enhance treatment efficacy in PDAC.

**Material and Methods**

Here, we present pre-clinical data on combining the highly specific FAK inhibitor narmafotinib (AMP945) with gemcitabine/Abraxane and for the first time FOLFIRINOX, which are two major standard-of-care chemotherapies for PDAC patients. 3D-organotypic matrices, intravital imaging and in vivo subcutaneous and orthotopic PDAC models were used to provide rationale for an early pulsed priming regimen of narmafotinib prior to chemotherapy.

**Results and Discussions**

Narmafotinib is a new potent small molecule FAK inhibitor. Phase I safety data shows excellent safety, tolerability, and pharmacokinetics following oral administration in humans. We reveal that narmafotinib treatment during early ECM remodeling (or 'priming') reduces fibrosis, while limiting subsequent PDAC invasion. Moreover, intravital imaging demonstrates FAK inactivation and cell-cycle stalling leading to improved chemotherapeutic efficacy upon narmafotinib priming in vivo in real-time. Long-term assessment in patient-derived models demonstrates that narmafotinib priming prior to gemcitabine/Abraxane or FOLFIRINOX reduces PDAC progression and extends survival in both chemotherapy settings.

**Conclusion**

Our results using these Phase II-ready drug combinations strongly support the clinical assessment of narmafotinib in PDAC. Narmafotinib is currently in Phase Ib/IIa trials, assessing a pulsed dosing regimen prior to gemcitabine/Abraxane, and warrants future clinical assessment of narmafotinib in combination with FOLFIRINOX.

### EACR2024-0739

### Modeling cell-cell interaction within the tumor microenvironment

*M. Domagalski[1], K. Pietraszek-Gremplewicz-[1], J. Olszańska[1], D. Nowak[1]*

*[1]Faculty of Biotechnology, University of Wroclaw, Department of Cell Pathology, Wroclaw, Poland*

**Introduction**

The tumor microenvironment (TME) is a complex ecosystem modulating biology and progression characteristics of cancer cells. Modeling the cell-cell interactions of heterogeneous populations within the TME is a major experimental challenge. The selection of an appropriate research model enables the characterization of the near-physiological interactions of tumor cells with their surroundings, and here the co-culture model is a basic approach. This presentation discusses two models of co-culture, direct and indirect, constituting methods to study the interactions of colorectal cancer cells (CRC) with adipose stem/stromal cells (ASCs).

**Material and Methods**

The study of the interactions of CRC with ASCs was performed with two co-culture models, direct and indirect. The analysis mainly focused on cell proliferative and migratory potential. The first model consisted of growing one cell type on a Transwell® filter and the other on the bottom of the culture dish, in different configurations. Proliferation and migration, in this type of co-culture, were studied by videomicroscopy, XTT, and wound healing assays. The direct co-culture model incorporated the cultures of both cell types in a single culture dish. In this approach, the characteristic analysis of the individual cell type was based on the fluorescence labeling of each co-culture cell type and subsequent measurements.

**Results and Discussions**

Depending on the model, the cells showed a diverse response to indirect and direct cell-to-cell contact. Differences were evident in proliferation and migration capacity of CRC cells, although no difference was observed in ASCs. No significant morphological changes were identified in the two cell types tested after both indirect and direct co-culture. Direct cell-to-cell contact more reliably mimics physiological conditions and may allow for monitoring interactions not evident in the indirect model. Nonetheless, an appropriate application of the indirect co-culture model may enable the study of effects arising solely from the secretion of compounds into the culture medium.

**Conclusion**

Selection of an accurate model to investigate the tumor microenvironment is critical. Obtained results may differentially reflect the state of actual cell-cell interactions, but an adequate experimental approach allows for a more specific analysis of selected modes of this communication.

*Research was funded by the National Science Centre, Poland, with a grant received by dr hab. Dorota Nowak, prof. UWr (OPUS 22, No. 2021/43/B/NZ3/01458).*

### EACR2024-0743

### Deciphering the impact of ECM alterations in prostate cancer driven by PGC1A

*L. Valcarcel[1], A. Macchia[1], S. Ariane[1], J. Saez[2], Á. Gonzalo-Paulano[1], J. Bravo-Cordero[3], F. Elortza[4], A. Aransay[5], A. Carracedo[6], V. Torrano[1]*

*[1]University of the Basque Country UPV/EHU, Biochemistry and Molecular Biology, Leioa, Spain*

*[2]University of the Basque Country UPV/EHU, Microfluidics Cluster UPV/EHU, Gasteiz, Spain*

*[3]Icahn School of Medicine at Mount Sinai, Division of Hematology and Oncology, Department of Medicine, New York, United States*

*[4]CICbioGUNE, Proteomics Unit, Derio, Spain*

*[5]CICbioGUNE, Genomics Platform, Derio, Spain*

*[6]CICbioGUNE, CICbioGUNE, Derio, Spain*

**Introduction**

Prostate Cancer (PCa) is the second most common cancer in men worldwide, representing a social and economic burden. Although there are therapies for PCa treatment with favourable clinical response, there are still 10-15% of patients that relapse and develop metastatic cancer. To date, there are no effective therapeutic alternatives for these patients, remarking the need for novel strategies aimed at preventing metastatic progression. Although both pro- and anti-tumorigenic factors regulating PCa progression have been identified, these factors mainly affect cell-intrinsic phenotypes, excluding the tumour-

© 2024 The Authors. Molecular Oncology is published by John Wiley & Sons Ltd on behalf of Federation of European Biochemical Societies associated extracellular matrix (ECM) and the perturbation of cell-matrix interactions. As part of the tumour microenvironment (TME), the ECM, constituting 60% of the tumour mass, not only provides with physical scaffold to the cells, but it also promotes tumour dissemination. Still, no data is available in the context of PCa.

**Material and Methods**

In order to ascertain the role of ECM alterations in PCa, we used chick chorioallantoic membrane (CAM) assays, biocompatible PEDOT:PSS scaffolds and orthotopic prostate lobe injections as 3D models. Then, we performed second-harmonic generation (SHG) microscopy and different omic techniques to allow us characterized and study the ECM and its interactions with PCa cells.

**Results and Discussions**

We have previously described that transcriptional alteration of metabolism driven by PGC1A leads to tumour and metastasis suppression in PCa in vitro and in vivo models. Histological analysis of PGC1A negative tumours presented fibrotic features, highlighting a potential alteration in the ECM. In line with this, omics analysis of these tumours and their associated tumour interstitial liquid, showed a significant increase in genes and proteins associated with ECM organization, degradation and collagen formation. SHG multiphoton microscopy showed significant alterations in collagen direction and organization in 3D tumours. Of note, these differences were associated to tumour aggressiveness and cell proliferation.

**Conclusion**

In conclusion, using clinically relevant in vivo and in vitro aggressive prostate cancer models based on PGC1A modulation, we have shown the relevance of ECM in PCa. We will further characterize from a molecular point of view these tumours and their ECM. We believe that the study of these alterations could help developing new therapeutic strategies for patients.

### EACR2024-0744

### Matrix-mediated dormant breast cancer cells show higher resistance to chemotherapy

*U. Heras*[1,2], *L. Iturriaga*[1], *A. Abaurrea*[3], *M.I. Calafel*[4], *R. Aguirresarobe*[4], *A. Riestra*[5,6], *P. Bragado*[7,8], *M.M. Caffarel*[3,9], *A. Cipitria*[1,9]

[1]*Biogipuzkoa Health Research Institute, Group of Bioengineering in Regeneration and Cancer, Donostia/San Sebastian, Spain*

[2]*University of the Basque Country UPV/EHU, Department of Cell Biology and Histology, Faculty of Medicine and Nursing, Leioa, Spain*

[3]*Biogipuzkoa Health Research Institute, Group of Breast Cancer, Donostia/San Sebastian, Spain*

[4]*University of the Basque Country UPV/EHU, POLYMAT and Department of Polymers and Advanced Materials: Physics Chemistry and Technology, Faculty of Chemistry, Donostia/San Sebastian, Spain*

[5]*Fundación Onkologikoa Fundazioa, Department of Pharmacy, Donostia/San Sebastian, Spain*

[6]*University of Deusto, Department of Medicine, Bilbao, Spain*

[7]*Complutense University of Madrid, Department of Biochemistry and Molecular Biology, Faculty of Pharmacy, Madrid, Spain*

[8]*Hospital Clínico San Carlos, Health Research Institute, Madrid, Spain*

[9]*Basque Foundation for Science, Ikerbasque, Bilbao, Spain*

**Introduction**

In vitro 3D models have shown that a dormant state of disseminated cancer cells can be induced by physical confinement of the extracellular matrix (ECM), either by its high density and/or low adhesion, followed by a lack of angiogenic signalling. We aim to understand the mechanism underlying this ECM-mediated dormant state by comparing mechanically tuneable semi-synthetic and natural origin matrices, both avascular and vascular.

**Material and Methods**

MDA-MB-231 breast cancer cells genetically modified with the FUCCI2 cell cycle reporter were encapsulated in hydrogels, with mCherry as G0/G1 phase indicator and mVenus as S/G2/M phase marker. Norbornene-modified inert alginate gels with high and low crosslinking density were used for dormancy induction, while Matrigel was chosen as a proliferation-permissive positive control. Phosphorylated pERK:p38 dormancy signalling ratio was analysed by immunofluorescence and cyclin-dependent kinase (CDK) inhibitor regulation by qPCR. Chemoresistance was tested with five different drugs (Paclitaxel, Doxorubicin, Gemcitabine, Carboplatin and 5-Fluorouracil) using viability and metabolic activity assays. As first in ovo experiments, gels were implanted in chicken chorioallantoic membrane (CAM) to obtain vascular models.

**Results and Discussions**

We obtained stiffness values of 10 and 1 kPa for stiff and soft alginate gels, respectively. Cell cycle analysis indicated that the number of cells in the S/G2/M phase decreased significantly in stiff matrices compared to soft gels. This growth arrest was validated by a down-regulation of pERK:p38 dormancy signalling ratio. Expression of p21 and p27 CDK inhibitors were upregulated compared to cells in a proliferative state. Dormant cells also showed higher resistance to Paclitaxel compared to proliferative cells. Further drug tests are currently ongoing. Finally, in ovo assays showed that inert alginate gels were poorly vascularised with small differences in cell cycle phases while Matrigel acted as inherent ECM creating small tumours due to its high vascularisation. Future work aims to analyse dormancy signalling and chemoresistance comparing avascular and vascular models.

**Conclusion**

Stiff inert alginate models are able to induce matrix-mediated dormant state of breast cancer cells by downregulating pERK:p38 signalling ratio and upregulating p21 and p27 CDK inhibitors. This regulation endows MDA-MB-231 cells with higher resistance to drugs.

### EACR2024-0750

### Combining comprehensive tumor-immune profiling with checkpoint interaction analysis for enhanced spatial

© 2024 The Authors. Molecular Oncology is published by John Wiley & Sons Ltd on behalf of Federation of European Biochemical Societies

### understanding of human cancer tissues

*S. Bodbin[1], A. Viratham[2], R. Stad[2], V. Goubert[2], N. Ma[2], J. Monkman[3], N. Jhaveri[2], H. Elofsson[1], S. Basu[1], A. Zieba-Wicher[1], A. Kulasinghe[3]*
*[1]Navinci Diagnostics, Applications, Uppsala, Sweden*
*[2]Akoya Biosciences, Applications, Marlborough, USA*
*[3]Frazer Institute- University of Queensland, Faculty of Medicine, Brisbane, Australia*

**Introduction**

Evading the immune system is crucial for cancer progression, with the PD-1/PD-L1 checkpoint pathway playing a key role. While therapies targeting these interactions have transformed cancer treatment, their efficacy varies among patients. The poor correlation between PD-1/PD-L1 expression levels and clinical results highlights the need for more detailed protein interaction analysis. Proximity Ligation Assays, detecting ligand-receptor proximity, illuminate signaling pathway activation. Coupled with comprehensive spatial phenotyping, this approach enhances understanding of the tumor microenvironment (TME) and aids in discovering spatial biomarkers for patient stratification and targeted treatments.

**Material and Methods**

We analyzed biopsies from patients with head and neck cancer undergoing treatment with immune checkpoint inhibitors. We utilized Naveni® PD-1/PD-L1 Proximity Ligation Assays with a high-plex PhenoCode™ Signature Panel for detailed profiling of the TME using the PhenoImager HT 2.0 platform. With bioinformatic analysis, we identified distinct cellular phenotypes, spatial relationships, functional interactions and spatial neighborhoods on a whole slide level.

**Results and Discussions**

We identified notable variations in the distribution of PD-1/PD-L1 interactions across the TME in head and neck cancers, pinpointing areas where these interactions correlate with immune cells located at the tumor's edge and within tumor-infiltrating lymphocytes. Using the PhenoCode Signature Panel enabled precise mapping of PD-1/PD-L1 interactions to specific immune cell types within the TME, facilitating a comprehensive workflow for analyzing the spatial co-location of receptor/ligand interactions and cell phenotypes.

**Conclusion**

The integration of spatial immune profiling with PhenoCode Signature Panels, and protein interaction data from the Naveni® PD-1/PD-L1 assays offer profound insights into the TME, enhancing our ability to predict clinical outcomes and presents a promising approach for refining patient selection and enhancing the effectiveness of immunotherapy treatments.

### EACR2024-0754

### Breast cancer lung metastasis on a chip

*L. Fallert[1,2,3], A. Urigoitia[2,3,4], D. Jimenez de Aberasturi[3,5], A. Cipitria[1,5]*
*[1]Biogipuzkoa, Bioengineering in Regeneration and Cancer, Donostia-San Sebastian, Spain*
*[2]University of the Basque Country, Applied Chemistry, Donostia-San Sebastian, Spain*
*[3]CIC biomaGUNE, Hybrid Biofunctional Materials, Donostia-San Sebastian, Spain*
*[4]Basque Center for Macromolecular Design and Engineering, Polymat, Donostia-San Sebastian, Spain*
*[5]Basque Foundation for Science, Ikerbasque, Bilbao, Spain*

**Introduction**

Breast cancer (BC) remains the leading cause of cancer related mortality in women worldwide, primarily as a result of metastatic outgrowth in distant organs including lungs. In order to advance therapeutic strategies and patient prognosis, it is imperative to gain insight into metastatic events within the target site. This requires biomimetic materials which recapitulate lung tissue composition and mechanics, compatible with conventional as well as novel technologies such as microfluidics. The aim of this study was to develop tunable BC lung metastatic niches within microfluidic chips to investigate the role of extracellular matrix (ECM) composition and stiffness during metastatic outgrowth and drug resistance.

**Material and Methods**

Commercially available microfluidic platforms were optimized using methacrylated gelatin (GelMA) for endothelial channel formation with EA.hy926 endothelial cells, and cell viability of human mesenchymal stem cells (hMSCs) as well as MDA-MB-231 BC cells within the GelMa matrix. MDA-MB-231 cells genetically modified with FUCCI2 reporters were encapsulated in GelMA to assess cell cycle dynamics of BC cells within microfluidic chips. Lung-mimicking hydrogels were designed by synthesizing click crosslinkable Tetrazine and Norbornene modified Alginate (T-N-Alg), and decellularizing porcine lung tissue (dECM). Composite hydrogels were characterized by rheology and assessed for biocompatibility, as well as compatibility with microfluidic culture.

**Results and Discussions**

Endothelial channels established intact barriers within 5 days of seeding in microfluidic devices. GelMA encapsulated BC cells and hMSCs displayed high viability throughout culture periods of 7 days. Decellularized lung tissue retained native tissue composition and architecture, which upon further processing formed thermally crosslinkable hydrogels. T-N-Alg functionalized with dECM yielded self-crosslinkable hydrogels with tunable stiffness, falling within the range of lung tissue. The composite hydrogel was compatible with microfluidic cell culture for prolonged culture periods.

**Conclusion**

The herein developed lung-mimicking hydrogel provides a tool to establish a in vitro lung niche-on-a-chip compatible with various cell types. Future work will aim to use this platform to investigate the effect of matrix alterations on metastatic outgrowth and drug responses within lung-mimicking hydrogels.

### EACR2024-0771

### Mapping the Dynamics of Cutaneous Squamous Cell Carcinoma Progression and Immunotherapy Response in the TiME using a Spatio-temporal Approach

*N. Jhaveri[1], B. Cheikh[1], D. Klymyshyn[1],*

© 2024 The Authors. Molecular Oncology is published by John Wiley & Sons Ltd on behalf of Federation of European Biochemical Societies

*N. Ma[1], A. Viratham[1], J. Monkman[2], N. Nelson[3], A. Kulasinghe[2]*
*[1]Akoya Biosciences, Applications, Menlo Park, USA*
*[2]Frazer Institute, University of Queensland, Brisbane, Australia*
*[3]Abcam, Commercial, Cambridge, UK*
**Introduction**
Cutaneous squamous cell carcinoma (cSCC), is the second most prevalent form of non-melanoma skin cancer. The emergence of metastatic and immunotherapy-resistant cases is a growing concern. Tumor development unfolds gradually, marked by a substantial mutational burden and an immunosuppressive tumor immune microenvironment (TiME). Identifying the underlying changes across the spatial and temporal landscape of these tumors will be crucial to understand the key cellular and molecular drivers of disease progression and response.
**Material and Methods**
We used a single-cell, whole-slide method to analyze proteins associated with immune lineage, tissue structure, cellular metabolism, and stress. Our aim was to map the spatial landscape of cSCC at pre-treatment, and at multiple time points during immunotherapy to construct a temporal atlas of the tumor. We used an ultra-high plex antibody panel with over 50 major determinants in the TiME on the PhenoCycler-Fusion instrument. Deep bioinformatic analyses was performed to identify cellular phenotypes, regional heterogeneity in cellular distribution and cellular interactions based on spatial proximity determinations.
**Results and Discussions**
Earlier studies revealed distinctive phenotypes associated with both response and resistance to immunotherapies in pre-treatment cSCC biopsies. Our findings build upon these observations by providing temporal insights into the evolving dynamics of the TiME throughout immune checkpoint inhibitor therapy. In addition to differences in immune cell composition and spatial distribution patterns, we observed variations in macrophage polarization and tumor burden between responder and non-responders cohorts.
**Conclusion**
The ultrahigh-plex spatio-temporal monitoring of cSCC revealed distinct signatures of response and resistance and identified key features in the TiME that reveal deeper insights into tumour pathobiology. Our discovery-based approach identifies novel biomarkers for patient stratification and therapeutic modulation.

### EACR2024-0773
### Sorafenib and Lenvatinib induce vascular responses in patient derived HCC on-Chip models

*B. Kramer[1], O. Mocellin[1], A. Olczyk[1], T. Olivier[1], H. Lanz[1], K. Queiroz[1]*
*[1]MIMETAS, Model development, Oegstgeest, The Netherlands*
**Introduction**
Hepatocellular carcinoma (HCC) is the most common type of liver cancer. Its incidence is increasing, and is closely related to advanced liver disease. Interactions in the HCC microenvironment between tumor cells and the associated stroma actively regulate tumor initiation, progression, metastasis, and therapy response.
**Material and Methods**
In the present study, we used the OrganoPlate graft to establish a co-culture system consisting of dissociated HCC tumors (HCC 1-8) and cell lines, HCC derived fibroblasts and vasculature. Cultures were prepared and validated by assessing their response to Sorafenib and Lenvatinib (72 hours). Cultures had their viability (alamar blue assay), and chemokine/cytokine levels in the supernatant (Luminex) determined. In addition, the organization of the vasculature in the tumor compartment was studied through immunostainings, confocal imaging, and subsequent morphological analyses.
**Results and Discussions**
HCC models were characterized by a range of specific markers, tumor (albumin), endothelial (CD31 and VE-Cadherin) and stromal (aSMA) cells. CD31 immuno-stained cultures were imaged, and morphology changes quantified. Sorafenib and Lenvatinib induced changes in the tumor vasculature area and organization.
**Conclusion**
Hereby, we present vascularized patient-derived HCC models that include relevant cellular players of the HCC microenvironment. These co-cultures are highly suitable for studying specific cell types as well as patient-specific responses. We envision that this patient derived model will evolve to become a platform for understanding the interplay between angiogenesis, stroma and immune infiltrate in HCC.

### EACR2024-0775
### Cancer Combat: Unveiling Acetylsalicylic Acid's Potential in Suppressing Pancreatic Stellate Cell-Mediated Tumor Growth

*S. Sığırlı[1], D. Karakas[1], E. Ulukaya[2], I.E. Demir[3], G.O. Ceyhan[4]*
*[1]Acibadem Mehmet Ali Aydinlar University, Medical Biotechnology, Istanbul, Turkiye*
*[2]Istinye University, Medical Biochemistry, Istanbul, Turkiye*
*[3]Technical University of Munich, Surgery, Munich, Germany*
*[4]Acibadem Mehmet Ali Aydinlar University, General Surgery, Istanbul, Turkiye*
**Introduction**
Pancreatic cancer is a highly aggressive type of cancer due to its unique tumor microenvironment. Active pancreatic stellate cells (PSCs) are abundant in the PaCa microenvironment and can promote cancer aggressiveness by secreting growth factors and cytokines. The daily use of acetylsalicylic acid (ASA), the active component of aspirin, has been linked to low cancer incidence in various cancers, including pancreatic cancer however, there is currently no study indicating its role on pancreatic stellate cell-mediated cancer aggressiveness. Therefore, we aimed to investigate the effect of ASA effect on PSCs and thereby aggressiveness of pancreatic cancer.
**Material and Methods**
PSCs were evaluated for active and passive states using α-smooth muscle and Oil Red O stainings. Aspirin doses

© 2024 The Authors. Molecular Oncology is published by John Wiley & Sons Ltd on behalf of Federation of European Biochemical Societies of 1.25 and 0.625 mM that were not toxic for cells were selected for further experiments. PANC-1 and BxPC-3 PaCa cell lines were treated with the CM collected from non-treated (NT) PSCs and 24h ASA-treated PSCs. Then, evaluating changes in cell viability, migration, and invasion using SRB assay, wound healing, and matrigel invasion assays, and colony formation assays, respectively. Furthermore, the expression levels of EMT markers were compared following exposure to CM from both NT PSCs and ASA-pretreated PSCs. Moreover, the difference in CM collected from PSCs after ASA pretreatment was elucidated by ELISA assay and changes in released IL-6 levels were measured.

**Results and Discussions**

The study revealed that PaCa cells exhibited increased proliferation, migration, and invasion when exposed to CM from NT PSCs, while these aggressive characteristics decreased when incubated with CM from ASA-treated PSCs.

**Conclusion**

In summary, ASA treatment decreased the cancer-promoting abilities of PSCs by possibly changing their secretome. Further research is needed to reveal the exact mechanism of ASA on PSCs.

### EACR2024-0776

### In vitro 3D cell-derived matrix model for the exploration of immunomodulatory mechanisms of the extracellular matrix

*A. Nedenskov*[1], *D. Hargboel Madsen*[2], *K. James Baker*[3]

[1]*Herlev Hospital, National Center for Cancer Immune Therapy CCIT-DK, København, Denmark*

[2]*Univeristy of Copenhagen, Department of immunology and Microbiology, Copenhagen, Denmark*

[3]*National center of immunology CCIT-DK, Department of oncology, Herlev, Denmark*

**Introduction**

Tissue architecture changes are common to solid tumors, resulting stiffer tissue which is exploited by cancer cells to achieve immune evasion and survival. Evidence indicates inherent immunomodulatory features of the ECM and targeting the ECM has been of great interest to further exploit the efficacy of immune checkpoint blockade (ICB) therapies in solid tumors. To explore ECM-derived immunomodulation, cell derived matrices (CDMs) from fibroblast cell origins were assessed for their capacities to influence cells found in the TME.

**Material and Methods**

CDMs were produced from fibroblast/fibroblast-like cells including dermal fibroblasts, Mouse Embryonic Fibroblasts (MEFs), MCA205, fibrosarcoma cells all derived from a C57BL/6 background. Fibroblast RNA was isolated following 7 days of ascorbic acid induced ECM deposition or alternatively ECMs were de-cellularized to produce CDMs. Matrix decellularization was achieved by lysis of cells followed by washing steps to move debris and Pulmozyme treatment to digest nuclear debris. BMDMs were cultured on CDMs for 7 days followed by extraction of RNA and FACS analysis. MC38 or PAN02 cancer cell lines were also cultured on separate CDM's, with RNA extraction taking place 24 hours post seeding, and CFSE/Cell viability assays taking place after 72 hours.

**Results and Discussions**

qPCR analysis of fibroblasts mRNA expression showed the CDM composition to differ between cell types, with differences observed in key ECM genes including type I collagens and several Small Leucine Rich Proteoglycans. BMDM's cultured on CDMs showed varying phenotypes, with CDM from several cell types capable of inducing protein expression of multiple macrophage polarization markers, including MHCII and PD-L1. Gene expression analysis of BMDM's revealed an inhibition of genes associated with classical activation of macrophages, suggesting the presence of these ECM's drove cells toward a wound healing phenotype. Gene transcript analysis of BMDM's in response to mechanically disrupted CDM's reflected this, with an inhibition of M1-associated genes concurrent with induction of M2 markers. Additionally, cancer cell proliferation rates varied according to the CDM they were grown on, especially when compared to non-CDM coated wells.

**Conclusion**

This work suggests that the ECM can significantly impact the TME by directly affecting the gene expression and phenotype of key cells of the TME. Understanding these factors could lead to strategies for mitigating the immunomodulatory impact of the complex tumor structure.

### EACR2024-0790

### Pancreatic cancer patient-derived xenograft (PDX) models: establishment and thorough molecular characterization

*D. Behrens*[1], *U. Pfohl*[2], *T. Scheller*[1], *M. Becker*[1], *B. Büttner*[1], *S. Wagner*[3], *R. Lawlor*[4], *J. Hoffmann*[1], *M. Dahlmann*[1], *W. Walther*[1]

[1]*EPO GmbH, Experimental Pharmacology and Oncology, Berlin, Germany*

[2]*CELLphenomics GmbH, Cellphenomics, Berlin, Germany*

[3]*University Hospital Tübingen, Surgical Trial Unit STU, Tübingen, Germany*

[4]*University of Verona, Centre for Applied Research on Cancer, Verona, Italy*

**Introduction**

Pancreatic cancer (PC) is one of the leading causes of cancer death and also one of the most lethal malignant neoplasms worldwide. PC is associated with poor prognosis, reflected by the high mortality and incidence rate. With a 5-year survival rate of less than 5% and a median survival of less than 6 months, PC possess one of the worst prognoses. Moreover, current treatment options for PC are insufficient and the outcome for PC has not improved significantly over the past 30 years. Therefore, this study aimed at establishment and thorough molecular characterization of pancreas carcinoma (PC) PDX models and the identification of new markers and signatures defining responsiveness toward drug treatments.

**Material and Methods**

Patient tumors were collected during surgery and transplanted subcutaneously into NOD/SCID/IL2y$^-$ mice and propagated in NMRI:nu/nu mice after engraftment. Established PDX (> 3$^{rd}$ passage) were morphologically evaluated and molecularly characterized. RNAseq and mutational analyses as well as of the HLA-status were

© 2024 The Authors. Molecular Oncology is published by John Wiley & Sons Ltd on behalf of Federation of European Biochemical Societies performed. The PDX were tested for sensitivity toward standard of care (SoC) drugs (gemcitabine, abraxane, 5-FU, oxaliplatin) and some of models were examined for alterations of stroma markers during treatment.
**Results and Discussions**
Our established 45 PC-PDX models closely resemble patient tumor morphology, mutational status, gene expression profile and sensitivity toward chemotherapy. Moreover, we show replacement of human by murine stroma, and that stroma components collagen I, α-SMA, FAP and SPARC remain unchanged by implantation route (s.c. vs. orthotopic) as well as by SoC drug treatment. The mutational analyses revealed an individual mutational profile of the PDXs, showing predominantly alterations in the genes encoding KRAS, TP53, FAT1, KMT2D, MUC4, RNF213, ATR, MUC16, GNAS, RANBP2, and CDKN2A. Correlation analyses of drug sensitivity of these PDX models and their molecular profile revealed hints for signatures of response and resistance. HLA typing of 41 PDAC PDX and the comparison with the HLA profiles of 8862 GSCD indicated that our panel reflects the HLA allele distributions of a large, representative cohort.
**Conclusion**
Our PC PDX panel represents the heterogeneous pheno- and genotype of this entity. It allows to identify markers for therapy response and for new therapeutic vulnerabilities. This study strongly supports the importance and value of PDX models for improved therapies of PC.

### EACR2024-0810
### A humanized mouse model to study stromal cell biology in human colorectal cancer

*M. Frank*[1], *A. Chen*[1], *I. Neuwirth*[1], *C. Stecher*[1], *N. Braun*[1], *J. Kabiljo*[2], *M. Farlik*[3], *M. Bergmann*[2], *D. Herndler-Brandstetter*[1]
[1]*Medical University of Vienna, Center for Cancer Research, Vienna, Austria*
[2]*Medical University of Vienna, Department of Surgery, Vienna, Austria*
[3]*Medical University of Vienna, Department of Dermatology, Vienna, Austria*
**Introduction**
Currently available immunotherapeutic options fail in the majority of colorectal cancer (CRC) patients. Besides of the low mutational burden in non-responding tumors, distinct stromal cell subsets may contribute to immunotherapy resistance by recruiting immuno-suppressive myeloid and lymphoid cells to the tumor microenvironment (TME). Identifying entities to inhibit the stromal subsets that generate the immunosuppressive TME – while preserving anti-tumorigenic stromal subsets – will have significant implications for immunotherapy-resistant CRC patients. However, there is a lack of suitable preclinical in vivo models that are equipped with both, a functional human immune system as well as human stromal cells, that would allow to study the heterogeneity of stromal cells, their drivers and their impact on immune cell recruitment and immunotherapy response.
**Material and Methods**
We used scRNA-seq data of human CRC (hCRC) samples to identify surface markers of stromal subpopulations of myofibroblastic cancer-associated fibroblasts (myCAFs), immunomodulatory CAFs (iCAFs) as well as of healthy fibroblasts. To validate these markers, hCRC samples were analyzed using multicolor flow cytometry. NSG mice or human-immune-system mice were injected with hCRC cells and human colorectal fibroblasts. The heterogeneity of stromal cells in the tumors was analyzed using flow cytometry and microscopy.
**Results and Discussions**
In the in vivo models, the injected human fibroblasts become plastic and differentiate into subtypes of tumor-associated stromal cells as identified by single-cell RNA-seq studies – we can identify distinct subsets resembling iCAFs, myCAFs as well as normal stromal cells. Using either knock-out stromal cells or knock-out tumor cells, this model will allow us to study drivers of stromal cell heterogeneity and stromal-cell driven immune cell recruitment to the tumors.
**Conclusion**
This model provides a unique platform to study the immunomodulatory properties of distinct stromal subsets and will therefore provide relevant insights into stromal cell biology in the TME of hCRC. In addition, it is suitable to test potential treatment regimens on PDXs for informed personalized medicine.

### EACR2024-0826
### A multi-tissue microfluidics system to predict the metastatic potential of prostate cancer

*F. Linke*[1], *W. Teubel*[1], *L. Hoelting*[2], *T. Haefeli*[2], *M.E. van Royen*[3], *M.M. Modena*[4], *C. Lohasz*[4], *A. Hierlemann*[4], *O. Frey*[2], *W.M. van Weerden*[1]
[1]*Erasmus MC- Cancer Institute, Experimental Urology, Rotterdam, The Netherlands*
[2]*InSphero AG, Research & Development, Schlieren, Switzerland*
[3]*Erasmus MC- Cancer Institute, Pathology, Rotterdam, The Netherlands*
[4]*ETH Zurich, Biosystems Science and Engineering, Basel, Switzerland*
**Introduction**
Advanced prostate cancer (PCa) spreads towards bones, lymph nodes, and visceral organs, including liver. However, not all PCa patients will develop metastases. To identify patients, who are at a high risk for developing metastatic disease, and to conduct early drug screens in models that reflect clinically relevant metastatic sites, we are building a human-based 3D culture system that allows for studying invasion of circulating tumour cells (CTCs) into different human healthy microtissues, such as liver and bone.
**Material and Methods**
A microfluidic chip (termed iFlow), which allowed for continuous recirculation of flowing cells in co-culture with tissue-model spheroids, was developed and produced in plastic, inert material (polystyrene). As CTC benchmark, non-adherent human PCa PDX-derived cell lines were used. To model relevant metastatic sites, human liver microtissues (MCTs) consisting of isolated

Molecular Oncology 18 (Suppl. 1) (2024) 1–495
© 2024 The Authors. Molecular Oncology is published by John Wiley & Sons Ltd on behalf of Federation of European Biochemical Societies liver cells from multiple human donors and mineralized bone microtissues of MG63 osteosarcoma cells were introduced in the same chip. Over time, gene (qRT PCR) and protein (IHC) expression as well as secreted proteins (ELISA) of the tissues were measured and correlated to PCa invasive capacity (confocal imaging) under flow conditions.

**Results and Discussions**

We identified optimal experimental conditions (different medium composition, tilting parameters and seeding density) to maintain liver and bone tissue functionality and continuous recirculation of viable tumour cells to interact with the 3D microtissues in the same chip. PCa cell lines, representing different disease stages, showed specific interaction with either bone, liver, or both over the course of two weeks. In contrast, no specific interactions were observed under static conditions, which highlights the importance of including flow conditions to study metastatic potential.

**Conclusion**

Microfluidic conditions, as applied in our 3D microfluidics iFlow system, are essential to capture cancer-specific invasion of non-adherent PCa cells- mimicking CTCs- into human liver and bone tissues, which is not possible using simple, static co-cultures. The developed system will contribute to identifying tumour cell and tissue factors that determine cell invasion into healthy tissues, which may guide metastasis-targeted drug development. Importantly, the generated knowledge may also be transferred to other cancer types.

### EACR2024-0837
### Monitoring cell health at scale in 3D matrix cultures is as easy as in 2D

*M. Engel[1], C. Yee[1], L. Belfiore[1], R. Bleach[2], D. Nguyen[1]*
*[1]Inventia Life Science, Biology, Sydney, Australia*
*[2]Inventia Life Science, Biology, Dublin, Ireland*

**Introduction**

Advanced three dimensional (3D) cell culture models are aiming to address the need for adequate cell-cell and cell-environment interactions to enable biologically relevant processes. While the uptake of simple and complex 3D cultures is growing, the hurdle of collecting rich and meaningful data at scale of such cultures remains. Here, we describe a workflow for generating such advanced cell cultures with the RASTRUM™ Platform, and common 2D cell culture assays for their suitability to assess core biochemical functions in 3D cell cultures.

**Material and Methods**

To establish and validate the workflow, we treated the 3D synthetic hydrogel cultures containing glioblastoma cells (U87), hepatocytoma (HepG2, LX-2), or adenocarcoma (MCF-7) cells with either the histone deacetylase inhibitor Panobinostat or the tubulin-targeting chemo-therapy drug Paclitaxel. We show that physiologically relevant tissue functions can readily be quantified in a high-throughput manner across these different cancer types, and five assay modalities, including high content imaging, metabolic activity (RealTime Glo, Cell Titer Glo 3D) and viability (LDH-Glo, MultiTox-Glo).

**Results and Discussions**

In the study, consistent and reproducible 3D matrix cultures with U87 glioblastoma cells, HepG2 liver cancer cells, and LX-2 stellate cells were created using the RASTRUM Platform. Treatment of U87 cells with chemotherapy drugs Panobinostat or Paclitaxel resulted in significant LDH release after 48 hrs, indicating drug penetration and impact on cell viability. Real-time metabolic activity of encapsulated HepG2 cells revealed efficient gas exchange and drug penetration across a wide concentration range in the presence of Paclitaxel. Additionally, metabolic activity variations were observed in LX-2 stellate cells cultured in different RASTRUM Matrix formulations, suggesting differential environmental interactions. The assay's sensitivity allowed for early detection of matrix-dependent proliferation differences.

**Conclusion**

Through these validation experiments we demonstrate the full compatibility of the advanced 3D cell cultures with several widely-used 2D culture assays. The efficiency of the workflow, minimal culture handling, and applicability of traditional screening assays, demonstrates that advanced matrix 3D cell cultures can be used in 2D cell culture screening workflows, while providing a more holistic view on cell biology to increase the predictability to in vivo drug response.

### EACR2024-0855
### Homotypic cell fusion of M13SV1 cells contribute to chromosomal instability and the propagation of hybrid cells with atypical genomic composition

*J. Weiler[1], T. Dittmar[1]*
*[1]Witten/Herdecke University, Immunology- Center for Biomedical Education and Research ZBAF, Witten, Germany*

**Introduction**

Chromosomal abnormalities including aneuploidy and polyploidy are omnipresent traits of solid tumors and suspected to be key driver for tumor heterogeneity. Cell fusion is one possible mechanism how cells become polyploid and beyond that suspected to trigger chromosomal instability (CIN). In this study, we investigated the result of an aberrant cell fusion event among M13SV1 breast epithelial cells giving rise to genomic-instable hybrid cells in which mitotic and chromosomal aberrations may promote the expansion of progeny with putative cancer-initiating capabilities.

**Material and Methods**

Two M13SV1 cell population encoding H2B-GFP or H2B-mcherry were mixed in a 1:1 ratio to yield double-positive hybrid cells identified by the expression of both fluorescence-tagged histone variants. Long-term live imaging was performed to track the cell fate of early and late passage hybrid cells across two generations. Received data were used to count the number of mitotic aberrations as well as to analyse cell and nuclear-specific parameters, such as cell size, nuclear size, nuclear circularity and the frequency of multinucleation using various downstream analysis software. Z-stacks images and time series were acquired under a laser-scanning microscope to reconstruct a 3D projection of hybrid cells undergoing mitoses. Records were deconvoluted to create

© 2024 The Authors. Molecular Oncology is published by John Wiley & Sons Ltd on behalf of Federation of European Biochemical Societies high-resolution movies of hybrid cells that pass mitoses atypical.

**Results and Discussions**

Live cell imaging studies revealed a high number of cell fate anomalies in hybrids associated with multipolar mitoses, multi- and micronucleation or endomitosis. Real time images at high magnification confirmed the presence of mitotic aberrations like lagging chromosomes in hybrids. Interestingly, the amount of mitotic aberrations as well as of nuclear deformation remained fairly constant across the passages suggesting that CIN is a prevalent condition in hybrids that is inherited to the next generation leading to unexpected new karyotypes. Cell size and nuclear size were larger in hybrids, but decreased slightly in late passage hybrids. However, nuclear size increased simultaneously with the cell size resulting in a stable nuclear to cytoplasmic ratio.

**Conclusion**

We could demonstrate that homotypic cell fusion of normal non-cancerous cells induce CIN in fusion-derived hybrid cells that propagate to the next generation through unexpected mitotic outcomes, suggesting that at some time hybrid cells may evolve with cancer-forming abilities.

### EACR2024-0864

### Liver-specific metastasis is recapitulated in a zebrafish larval xenograft model that mimics the tumor microenvironment

*M. Basol*[1,2], *P. Korhan*[3,4,5], *E. Bagirsakci*[2,5], *N. Atabey*[3,4,5], *G. Cakan - Akdogan*[1,6]

[1]*Izmir Biomedicine and Genome Center, Cakan Zebrafish Models Lab, Izmir, Turkiye*

[2]*Izmir Biomedicine and Genome Institute, Molecular Biology and Genetics, Izmir, Turkiye*

[3]*Izmir Tinaztepe University, Department of Medical Biology, Izmir, Turkiye*

[4]*Galen Research Center, Department of Medical Biology, Izmir, Turkiye*

[5]*Izmir Biomedicine and Genome Center, Atabey Lab. on Cancer Biology and Signaling, Izmir, Turkiye*

[6]*Dokuz Eylul University, Department of Medical Biology, Izmir, Turkiye*

**Introduction**

Liver is one of the most common metastatic targets for various cancer types, and 50% of cancer patients develop hepatic metastasis. Colorectal cancer (CRC) is the most common cancer that metastasizes to the liver, and leading cause of death in CRC is liver metastasis. In hepato-cellular carcinoma (HCC), intrahepatic metastasis is the most common form of disease recurrence after liver resection. To understand and tackle liver metastasis, complex models that mimic the tumor microenvironment, including extracellular matrix, the vasculature, and the homing cells in target tissues, are required. Zebrafish larval metastasis model provides an intact organism model with added benefits of imaging, genetic and chemical perturbations. Here, we aimed to develop a liver-specific metastasis model and investigate whether organotropism of selected HCC and CRC cells are reproduced.

**Material and Methods**

A transgenic zebrafish model in which the hepatocytes express a fluorescent protein was generated. A circulating tumor cell approach was used to inject fluorescently labeled cancer cells into the vasculature of zebrafish embryos at 2 days post fertilization. Presence of metastasis in liver was quantified 4-5 days later. In order to induce fatty liver, larvae were exposed to free fatty acids, fructose, and glucose. Liver steatosis was confirmed with oil red staining, lipid droplet quantitation after BODIPY labeling and qPCR.

**Results and Discussions**

SNU449 and c-Met overexpressing SNU398 HCC cells that were previously shown to colonize to liver micro-environment in our Lab on a chip model were tested in the zebrafish liver metastasis model. Both cell types exhibited higher liver metastasis in the zebrafish compared to control SNU449 cells treated with c-Met inhibitor SU11274, or naive SNU 398 cells. Fatty liver was induced in the larvae as indicated by a significant increase in lipid droplet formation and histopathological examination. The effect of fatty liver on metastatic rate was also shown. Finally, SW480 and SW620 CRC cells with different metastatic capacities were tested in the zebrafish liver metastasis model. The findings reported here indicate that zebrafish liver microenvironment induces invasion of HCC and CRC cells that are present in the vasculature. Moreover, induction of fatty liver in the larvae affects the metastasis rate.

**Conclusion**

The zebrafish hepatic metastatic model presented here offers an intact organism model to study liver preference of metastatic cells.

### EACR2024-0877

### Spatial cell cycle phenotypic variations among clinical subgroups post-neoadjuvant chemotherapy in ovarian cancer

*A. Junquera*[1], *Z. Liang*[1], *M. Salko*[1], *S. Shah*[1], *M. Luna-Alvarez*[2,3], *E. Kahelin*[1], *M. Vääriskoski*[1,4], *U.M. Haltia*[1,4], *A. Virtanen*[1,5], *F. Anniina*[1,4,6]

[1]*University of Helsinki, Research Program in Systems Oncology, Helsinki, Finland*

[2]*Universidad Nacional Autónoma de México, Instituto de Investigaciones Biomédicas, Mexico City, Mexico*

[3]*Instituto Nacional de Pediatría, Laboratorio de Falla Medular y Cáncer, Mexico City, Mexico*

[4]*Helsinki University Hospital, Department of Obstetrics and Gynecology, Helsinki, Finland*

[5]*Helsinki University Hospital, Department of Pathology, Helsinki, Finland*

[6]*Helsinki Institute of Life Sciences, Institute for Molecular Medicine Finland, Helsinki, Finland*

**Introduction**

High-grade serous carcinoma (HGSC) is the most common and lethal subtype of ovarian cancer. Neo-adjuvant chemotherapy (NACT) is a well-established treatment modality for advanced staged HGSC. Eventual relapse and platinum resistance remain major clinical challenges. The complex interplay of multiple biological processes, like cell cycle progression, DNA repair, and immune surveillance is known to have an impact on

Molecular Oncology 18 (Suppl. 1) (2024) 1–495

© 2024 The Authors. Molecular Oncology is published by John Wiley & Sons Ltd on behalf of Federation of European Biochemical Societies disease progression. Therefore, exploring their influence on NACT-treated patients is key to improve current clinical standards.

**Material and Methods**

We prospectively recruited >300 patients to the Oncosys-OVA clinical trial (NCTXXYY). We annotated the HGSCs to distinct genomic and morphologic response groups, while collecting detailed surgical and clinical data. We employed a single-cell spatial profiling technology, tissue cyclic immunofluorescence (tCycIF), on FFPE samples from the same cohort. This multiplexed-imaging method detects up to 80 antigens from a sample, while preserving the tissue architecture. We built and validated a panel with antibodies targeting cell cycle-regulators (Cyclin A, B, D, E, pRb, p21, p27), proliferation (Ki-67, PCNA) and DNA damage (γH2AX), alongside cell-type markers for distinct cell populations. We utilized GeoMx spatial transcriptomics to capture cell-population deconvoluted gene expression profiles within regions of interest from tCycIF spatial analyses.

**Results and Discussions**

Via advanced image analysis, we reconstructed the cell cycle dynamics and assessed the expression of markers in cells in pseudotime. By conducting a multivariate assessment of the proliferation markers on tCycIF, we were able to assess the spatial distribution of cell neighborhoods with distinct proliferative characteristics. To explore the transcriptome of these regions, we developed a pipeline that integrates tCycIF and GeoMx imaging data, enabling the precise targeting of cell populations of interest in adjacent slides. These analyses were integrated with the matching clinical, pathological, and genomic profiling information, leading to the identification of recurring cellular neighborhoods with potential therapeutic implications.

**Conclusion**

By integrating multiomics and spatiotemporal analyses with clinicopathological features, we identified response-related patterns that could inform patient stratification and treatment selection. This study has the potential to drive advancements in personalized therapeutic approaches for HGSC patients.

### EACR2024-0889

### Ultra highplex spatial proteomic and transcriptomic analysis of the head and neck cancer tumour microenvironment

A. Rosenbloom[1], C. Wee Tan[2], H. Sadeghirad[3], V. Yaghoubi Naei[3], J. Monkman[3], B. Filanoski[1], L. Hamanishi[1], J. Beechem[1], A. Kulasinghe[3]

[1]*Nanostring Technologies, Research and Development, Seattle, United States*

[2]*Walter and Eliza Hall Institute, Research, Melbourne, Australia*

[3]*University of Queensland, Frazer Institute, Brisbane, Australia*

**Introduction**

Head and neck cancers are the 6th most common tumour type globally and account for ~900,000 new cases a year and 450,000 deaths. Arising from multiple anatomical sites, such as the oral cavity, pharynx, and larynx, HNSCC tumours present complex tumour micro-environments. In this study, we sought to understand the tumour and stromal properties of tongue, lip, oral cavity and pharyngeal cancers using cutting-edge, high-dimensional spatial tools.

**Material and Methods**

We profiled a tissue microarray consisting of 85 patient biopsy samples, sampled from the tongue (n=15), oral cavity (n=15), pharynx (n=28), and lip (n=27) using the Nanostring Technologies IO Proteome Atlas (570-plex) to liberate tumour- and stromal- compartment specific proteins associated with clinical endpoints, DFS (disease free survival) and OS (overall survival).

**Results and Discussions**

In this cohort study, tongue cancers had the worst overall survival when compared with lip and oral cavity cancers, which had a more favourable prognosis. Differential expression of tumour-compartments between tongue and lip cancers indicated higher expression of fibronectin, alpha smooth muscle actin, osteopontin and proteins associated with higher metastatic potential. When comparing between tongue and pharyngeal samples, proteins associated with heat shock, activation of TGF-beta and phosphorylation were increased in the tongue. In patients with a better prognosis, numerous immune cell types were co-localised to the tumour microenvironment, including CD20, CD8, CD4 and CD74, whereas within the tumour compartment, infiltration of these markers associated with a better disease free survival

**Conclusion**

Taken together, this study demonstrates that ultra-high plex spatial proteomics provides a lens of biomarker discovery across a head and neck cancer clinical cohort and that anatomy-specific tumour and TME profiles can be contrasted. Moreover, this provides an informed rationale for selecting biomarkers for deeper spatial profiling at single-cell resolution.

### EACR2024-0906

### Angiogenesis promotes regrowth of chemotherapy-induced drug tolerant persisters of colorectal cancer in vivo

M. Kanda[1,2], R. Coppo[1], K. Onuma[1], M. Inoue[1]

[1]*Graduate School of Medicine Kyoto University, Department of Clinical Bio-resource Research and Development, Kyoto, Japan*

[2]*Osaka University Graduate School of Medicine, Department of Obstetrics and Gynecology, Suita, Japan*

**Introduction**

When exposed to high concentrations of anticancer drugs, some cancer cells survive and enter a growth suppression state known as DTP (Drug Tolerant Persister). Although the characteristics of the DTP state, such as metabolic inhibition, are becoming clearer, the mechanism of the transition from DTP to re-growth has not yet been elucidated. Previously, we evaluated the growth capacity of individual cells and elucidated that colorectal cancer (CRC) organoids are composed of S- and L-cells. S-cells form small spheroids and can give rise to only S-cells when isolated (S-pattern growth). Conversely, L-cells form large spheroids and give rise to both S- and L-cells (D-pattern growth). S-cells resembled DTP features. Since Notch signal inhibition impedes the transition from S-cells to L-cells, we studied the

© 2024 The Authors. Molecular Oncology is published by John Wiley & Sons Ltd on behalf of Federation of European Biochemical Societies inhibitory effect of Notch signaling inhibition on regrowth after chemotherapy.

**Material and Methods**

Patient-derived CRC organoid lines and their xenografts were treated with gemcitabine or 5-FU. After treatment, clonogenic growth assays were performed. Residual cancer cells were cultured with supernatant of endothelial cell culture and analyzed by clonogenic growth assay and RNA sequencing. Combination treatment of an angiogenesis inhibitor and a Notch inhibitor, and gemcitabine was performed in vivo. Permission for the establishment of patient-derived organoids and xenografts was obtained from the Ethics Committee and Institute of Laboratory Animals, Graduate School of Medicine, Kyoto University.

**Results and Discussions**

Cancer cells that persisted after exposure to anticancer drugs initially exhibited S-cell like characteristics in vitro and in vivo, followed by the emergence of L-cell-like cells and transition to the regrowth phase. Inhibition of Notch signaling inhibited regrowth in vitro but was transient in vivo. When cultured with culture supernatants of endothelial cells, the JAK/STAT pathway in cancer cells was activated and the growth pattern at day 3 after treatment shifted from S-pattern to D-pattern. Therefore, it was postulated that factors secreted by vascular endothelial cells acted on cancer cells to counteract the regrowth inhibitory effect of Notch signal inhibition. Concurrent administration of an angiogenesis inhibitor, gemcitabine, and a Notch inhibitor markedly suppressed regrowth in vivo.

**Conclusion**

Combinations of vascular endothelial growth inhibitors, chemotherapeutic agents, and Notch inhibitors are promising for inhibiting DTP regrowth.

### EACR2024-0908
### Preserving the tumor microenvironment: Advancing 3D in vitro tissue models through serum-free cell culture

*A. Siebenmorgen[1], C. Wöhle[1], K. Lamfried[1], L. Martinez Carrera[1], B. Heller[2], K. Lupar[2], G. Bergamini[2], D. Poeckel[2], O. Hardt[1], B. Theek[1]*
*[1]Miltenyi Biotec B.V. & Co KG, Molecular Technologies & Stem Cell Therapy, Bergisch Gladbach, Germany*
*[2]Cellzome GmbH- a GSK company, Discovery Biology, Heidelberg, Germany*

**Introduction**

Recent advancements in cancer research, particularly in immunotherapy, have highlighted the importance of the tumor microenvironment (TME). However, replicating the complexity of the human TME in experimental models remains a challenge. As a result, advanced ex vivo cultivation techniques, such as precision-cut slices (PCS), are used to provide a faithful representation of the TME in three dimensions. Key to the success of these ex vivo models is the selection of appropriate culture conditions that maintain the integrity of the TME.

**Material and Methods**

Lung tumor tissue was sliced into PCS using the Krumdieck Tissue Slicer (TSE systems). For the cultivation of PCS, Lung TumorMACS® Medium was compared to a home-brew medium containing human serum. After the cultivation, PCS were collected and dissociated using the gentleMACS™ Octo Dissociator with Heaters and the Tumor Dissociation Kit, human. Cells were stained with the desired antibodies to determine viability, yield, and frequency, and analyzed using the MACSQuant® Analyzer 10. RNA for bulk RNA sequencing was extracted using QIAGEN's RNeasy® Kit. Libraries were prepared with QIAseq® Stranded mRNA Kit and sequenced on an Illumina NextSeq 550. Analysis was done using CLC Genomics Workbench. For single-cell RNA sequencing, cells were processed with 10x Genomics Chromium Next GEM kits and sequenced on NextSeq 550. Data preprocessing was done with Cell Ranger, followed by analysis in Python and R using Seurat. Gene signature scores were computed with AUCell, and functional enrichment analyses were performed with gProfiler2 R package.

**Results and Discussions**

PCS derived from fresh non-small cell lung carcinoma (NSCLC) tissue have been successfully maintained for 48 hours. The cellular composition was preserved and resembled the original tissue. A comparative analysis with a serum-containing medium revealed that PCS cultivated in the lung-specific medium exhibited 1.) a lower number of differentially expressed genes in comparison to the fresh sample, and 2.) a lower expression of stress genes, suggesting a more favorable environment for maintaining TME integrity.

**Conclusion**

Lung TumorMACS® Medium preserves the TME in NSCLC PCS and has a lower impact on the gene expression level, compared to a serum-containing medium.

### EACR2024-0917
### Elucidating the impact of ascites on High Grade Serous Ovarian Cancer through in vitro patient-specific multi-model characterization

*B. Barzaghi[1], P. Lo Riso[1], M.R. Sallese[1], E.C. Villa[2], A. Gatto[1], G. Picco[1], U. Cavallaro[1], G. Testa[1]*
*[1]European Institute of Oncology, Department of Experimental Oncology, Milan, Italy*
*[2]Human Technopole, Neurogenomics, Milan, Italy*

**Introduction**

High grade serous ovarian cancer (HGSOC) is the fifth cause of cancer death among women due to late diagnosis and tumor recurrence. Growing evidence relates relapse to the persistence of cancer stem cells (CSCs), a model corroborated by the prevalence of peritoneal ascites in OC patients. Indeed, ascites acts as a source of soluble factors, providing a pro-inflammatory and tumor promoting microenvironment that supports the survival of tumoral cells and mediate metastatization. Understanding the downstream effects of tumoral cells exposure to ascitic fluid (AF) would thus reveal the pathogenetic mechanisms of stemness, metastasis, and chemoresistance.

**Material and Methods**

We supplemented AF to three culturing systems of HGSOC ascites-derived cells, namely 2D tumor cells, bulk spheroids (BS) and single-cell metastatic OC

Molecular Oncology 18 (Suppl. 1) (2024) 1–495
© 2024 The Authors. Molecular Oncology is published by John Wiley & Sons Ltd on behalf of Federation of European Biochemical Societies spheroids (sMOCS) and analyzed its effect on proliferation and stemness.

**Results and Discussions**

We had already introduced sMOCS as a new model able to capture clonal features at high throughput and resolution to expose inter/intra-patient CSCs variability and that relies on supplementation with AF, pointing to soluble factors able to sustain growth from single cells. We found that AF increases proliferation for both 2D cells and spheres. 2D cells and BS cultured with AF could be propagated for higher passages, improving culture conditions. Also, we scored an increased sphere forming efficiency (SFE) in BS in AF, suggesting its ability to sustain the CSCs niche. In parallel, we performed bulk RNAseq analysis showing that AF supplementation allows to preserve the metabolic status of fresh samples, lost in standard conditions. Also, 2D cells and 3D spheroids showed a model-specific effect of ascites' supplementation, with a downregulation of genes involved in the biosynthesis of cholesterol and in inflammatory response in 2D, and an upregulation of pathways involved in proliferation and glycolysis in spheres, suggesting a phenotype specific for CSCs. Moreover, our data allowed the scoring of CSCs-related, AF-dependent genes as upregulated in spheres and de-regulated in ascites.

**Conclusion**

These results shed new light on the effect of AF on different tumor cells, highlighting endophenotypes compatible with metastatization and chemoresistance. While also affording an improvement in culture conditions for a more streamline and systematic capturing of patient-specific features, these studies offer an internally comparative experimental platform to advance the HGSOC field towards precision oncology.

### EACR2024-0927

### Major histocompatibility complex expression is associated with molecular profiles and clinical outcomes in ovarian cancer

F. Perez[1,2], L. van Wagensveld[3,4,5], A. Shabanova[1], M. A van der Aa[3], R.F. Kruitwagen[5,6], G.S. Sonke[7], K.K. Van de Vijver[8,9,10,11], P.K. Sorger[12,13], H.M. Horlings[4], A. Färkkilä[1,2,14,15]

*[1]University of Helsinki, Faculty of Medicine- Research Program for Systems Oncology, Helsinki, Finland*
*[2]iCAN digital precision cancer medicine flagship, iCAN digital precision cancer medicine flagship, Helsinki, Finland*
*[3]Netherlands Comprehensive Cancer Organization IKNL, Department of Research and Development, Utrecht, The Netherlands*
*[4]The Netherlands Cancer Institute, Departement of Molecular Pathology, Amsterdam, The Netherlands*
*[5]GROW, School for Oncology and Reproduction, Maastricht, The Netherlands*
*[6]Maastricht University Medical Centre, Department of Obstetrics and Gynaecology, Maastricht, The Netherlands*
*[7]The Netherlands Cancer Institute, Department of Medical Oncology, Amsterdam, The Netherlands*
*[8]Department of Gynecology, Center for Gynecologic Oncology Amsterdam CGOA, Amsterdam, The Netherlands*
*[9]Ghent University, Cancer Research Institute Ghent CRIG, Gent, Belgium*
*[10]Ghent University Hospital, Department of Pathology, Gent, Belgium*
*[11]The Netherlands Cancer Institute, Department of Molecular Pathology, Amsterdam, The Netherlands*
*[12]Harvard Medical School, Laboratory of Systems Pharmacology, Boston, United States*
*[13]Harvard Medical School, Department of Systems Biology, Boston, United States*
*[14]Helsinki Institute of Life Sciences, Institute for Molecular Medicine Finland, Helsinki, Finland*
*[15]Helsinki University Hospital, Department of Obstetrics and Gynecology, Helsinki, Finland*

**Introduction**

High-grade serous cancer (HGSC), is the most lethal ovarian cancer subtype. In this study, our objective was to unravel the association of the spatial tumor microenvironment with common HGSC's molecular profiles and clinical outcomes.

**Material and Methods**

We employed highplex tissue imaging (tCycIF) to characterize 250 HGSC tumors using 1000 TMA-cores. The immune cell infiltration was also assessed by pathologists using IHC for CD8, CD20, CD68, and CD103. Using DNA sequencing and methylation assay we categorized the tumor samples in four molecular profiles: BRCAloss, homologous recombination deficient (HRD) or proficient (HRP), and CCNE1-amplification. For tCycIF, we used 34 different protein markers. We categorized single cells to distinct subpopulations using the TRIBUS and SCIMAP software. We developed a machine-learning random forest pipeline to classify individual cells based on their marker intensity, morphology, and neighborhoods into molecular profiles. For validation sets, we used 1) publicly available deconvoluted cancer-cell specifc RNA-expression data of 252 samples from The Cancer Genome Atlas (TCGA) and 2) Whole-slide highplex tCyCIF imaging from 30 samples from an independent cohort.

**Results and Discussions**

Using highly multiplexed imaging, we identified a total of 4.8 million single cells, of which 1.6 million were immune cells from eight biological subpopulations. The detected immune cell proportion per sample was concordant with estimates through immuno-histochemistry (IHC) by a pathologist. Our machine learning pipeline achieved an accuracy (F1 score) of 0.77 in classification of individual BRCAloss cancer cells from HRP cancer cells in chemotherapy-naive samples. We detected that BRCAloss and HRD cancer cells exhibited enrichment in immunogenic signals (MHC-I and MHC-II) compared to HRP and CCNE1-amplified cancer cells. Furthermore, when training our model to predict high or low survival within HRD or HRP profiles, MHC-II and MHC-I were identified among the most important features for the prediction. In agreement, we observed that MHCII expression inferred in cancer cells, was also associated with survival in TCGA HGSC samples. Furthermore, the associations were corroborated

© 2024The Authors. Molecular Oncology is published by John Wiley & Sons Ltd on behalf of Federation of European Biochemical Societies in our independent validation set of 30 patients with whole-slide images.

**Conclusion**

Our study revealed distinct phenotypic cell subpopulations linked to antigen presentation which were associated with molecular profiles and clinical outcomes, paving the way for precision oncology in ovarian cancer.

### EACR2024-0936

### Characterisation of the tumor microenvironment in chronic lymphocytic leukemia throughout disease progression, with a focus on myeloid cells

*C. Castellanos Gonzalez[1], M. Coelho[1], R. Massoni-Badosa[1], N. Mack[1], S. Autenrieth[2], B. Maier[3], P. Lichter[1], M. Seiffert[1]*

*[1]German Cancer Research Centre DKFZ, Molecular Genetics, Heidelberg, Germany*

*[2]German Cancer Research Centre DKFZ, Infection-Inflammation and Cancer, Heidelberg, Germany*

*[3]CeMM- Research Centre for Molecular Medicine of the Austrian Academy of Sciences, Tumour Immunology, Vienna, Austria*

**Introduction**

Chronic lymphocytic leukemia (CLL) is characterized by the accumulation of mature B cells in peripheral blood and lymphoid organs. The tumor microenvironment (TME), comprising diverse cell types, like myeloid cells, significantly influences disease progression. Interleukin-4-induced-1 (IL4I1), mainly expressed by myeloid cells, is upregulated in various cancers, and catabolizes aromatic amino acids. Our previous research demonstrated that IL4I1 metabolites suppress T cell activity and promote CLL progression in mice. As in patients, the Eμ-TCL1 mouse model develops CLL at older ages and reflects the TME in CLL. However the temporal dynamics of the TME alterations remain unexplored. We performed an in-depth characterization of immune cells, particularly focusing on myeloid cells and IL4I1, throughout disease progression in this model.

**Material and Methods**

Spectral flow cytometry analyses were performed with spleen samples, the main organ of CLL cell accumulation, of 16 Eμ-TCL1 mice spanning 10 to over 50 weeks of age, alongside age-matched controls, to evaluate the immune cell composition. In addition, splenocytes of mice at several time points after adoptive transfer of TCL1 CLL in mice were analysed. To characterize IL4I1-expressing myeloid cells, CLL cells were transplanted into *Il4i1*$^{-/-}$ or wild-type bone marrow chimeric mice and single-cell RNA-sequencing (scRNAseq) was performed on splenocytes of CLL-bearing chimeric mice.

**Results and Discussions**

Spectral flow cytometry analyses revealed changes in the myeloid cell populations in spleen over disease progression, especially in mice older than 44 weeks with advanced disease. Additionally, disease-related changes in T cell populations along CLL development were detected, notably a decrease in CD4 and an increase in CD8 T cells. Furthermore, accumulation of patrolling macrophages rather than inflammatory, and of DC1s rather than DC2s, was observed over disease development. scRNAseq of splenocytes of CLL-bearing chimeric mice unveiled mature regulatory dendritic cells (mregDCs) as the primary Il4i1-expressing population. Longitudinal samples demonstrated mregDC accumulation alongside CLL development, accompanied by diminishing antigen-presenting capacity over time.

**Conclusion**

Our results show the dynamic changes in immune cell populations happening across CLL development. Ongoing efforts aim to elucidate the role of mregDCs and their interactions with other immune cell types in this model, providing insights into CLL pathogenesis and potential therapeutic avenues.

### EACR2024-0945

### Fully automated in situ proximity ligation assay combined with multiplex IF allows functional studies of proteins and their interactions

*H. Elofsson[1], L. Arngarden[1], O. Hahne[1], F. Oliveira[2], D. Raykova[2], A. Wicher[1]*

*[1]Navinci Diagnostics, Research and Development, Uppsala, Sweden*

*[2]Navinci Diagnostics, Sales and Marketing, Uppsala, Sweden*

**Introduction**

In situ proximity ligation assay is a well-established tool for the study of protein-protein interactions (PPI) in tissue samples. It detects proteins located within 40 nm, via antibodies conjugated to oligonucleotides that generate amplified fluorescent or chromogenic signal. Proximity ligation assay can be combined with multiplexed immunofluorescence (IF) staining of individual proteins to visualize tissue context e.g., the tumor microenvironment (TME) or immune landscape. We developed a fully automated and robust spatial interactomics assay based on the Naveni® in situ proximity ligation technology, validated on Leica Bond RX. To study of the TME, multiplex IF was performed in combination to explore immune activation and a key immune checkpoint interaction in the context of cancer and T cell markers.

**Material and Methods**

Serial FFPE samples encompassing tissues from healthy controls, Hodgkin lymphoma, ovarian, and colon cancer were run using NaveniFlex Tissue. Immune system activation was studied by detecting the Zap70/LAT interaction alongside individual T cell markers CD3 and CD8. Furthermore, immune checkpoint activation was determined by detecting the interaction between PD-1/PD-L1. Tumor tissue visualization was facilitated with pan-cytokeratin staining. All Naveni® steps, encompassing primary and Navenibody incubation, ligation, amplification, and detection, were seamlessly executed within the Bond RX system. The IF staining of the individual markers was incorporated in the Naveni® detection step. Slides were then mounted and imaged on the Olympus V200 automated slide scanner.

**Results and Discussions**

Molecular Oncology 18 (Suppl. 1) (2024) 1–495
© 2024 The Authors. Molecular Oncology is published by John Wiley & Sons Ltd on behalf of Federation of European Biochemical Societies

Congruent PD-1/PD-L1 and Zap70/LAT staining patterns were revealed in consecutive sections, demonstrating the robustness of the Naveni® method. Through multiplex IF co-staining, we visualize the coexistence of cancer and immune cells, offering valuable insights into immune checkpoint activity and T-cell activation (or lack thereof) within healthy and diseased tissues. We thus validated the robustness of our high throughput proximity ligation assay for the functional study of PPI in tissue. By automating this process on FFPE slides, we streamline the workflow, enhance efficiency, and ensure reproducibility, meeting the demand for high-throughput analysis without compromising sensitivity.

**Conclusion**

The possibility to acquire functional and spatial information about proteins and their interactions in this manner opens the door to applying the Naveni® technology both in large scale research and in the clinic.

### EACR2024-0951
### Characterization of a syngeneic cancer stem cell model of insulin-like growth factor 2 amplified glioblastoma

*K. Heemskerk[1], S. Assaf[1], X. Hao[1], M. Meode[1], S. Weiss[1], F.J. Zemp[1], H.A. Luchman[1]*
*[1]University of Calgary, Arnie Charbonneau Cancer Institute, Calgary, Canada*

**Introduction**

Glioblastoma (GBM) is plagued by extensive inter- and intra-patient heterogeneity, making it resistant to therapy. There is a small subset of GBMs that overexpress insulin-like growth factor 2 (IGF2). IGF2 is essential for fetal development, can promote cancer growth, and has a role in immunosuppression in experimental autoimmune encephalomyelitis. Using a syngeneic cancer stem cell model with IGF2 amplification, we investigated if IGF2 influences tumour and microenvironment cells to promote immunosuppression in GBM.

**Material and Methods**

Three cell lines were previously established from spontaneous brain tumours in C57Bl/6 *Trp53*$^{+/-}$/*Nf1*$^{+/-}$ mice and maintained using neural stem cell culture conditions. Whole genome sequencing was performed on the three lines and single nucleotide, copy number, and structural variation analyses were performed. All three cell lines were orthotopically allografted to assess tumorigenicity. RNA sequencing was performed on the tumorigenic line and normal neural stem cells. Growth in vitro was assessed by examining cell viability over time. Stem cell marker expression was examined by quantitative immunofluorescence. IGF2 protein levels were assessed by ELISA. Genomic and transcriptomic (single cell and bulk) data from human GBM stem cells and human GBM tissue was used to validate findings in human models.

**Results and Discussions**

Whole genome sequencing revealed loss of both copies of *Trp53* and *Nf1* in all three cell lines. However, only one of the cell lines (mBT0309) developed brain tumours. Analysis of genomic alterations and transcriptomics revealed that mBT309 exhibits amplification of the IGF2 loci and overexpression was confirmed at the RNA and protein level. Spatial and single cell transcriptomics showed that IGF2 is overexpressed in mBT309 allografted tumours and correlated with specific transcriptomic programs. In vitro growth characteristics and stem marker expression suggested high levels of IGF2 may regulate GBM stemness features. Analysis of human GBM datasets revealed that IGF2 amplified tumours have reduced CXCL11 expression. These findings suggest that high levels of IGF2 may influence immunosuppression by reducing T-cell recruitment.

**Conclusion**

Using a variety of ‘omics and molecular approaches, we have characterized a syngeneic immunocompetent GBM stem cell model harbouring IGF2 amplification; adding to the suite of models to study the GBM microenvironment.

### EACR2024-0955
### Therapy-induced senescence favors tumor spreading in non-Hodgkin lymphoma

*C. Guardia Valenzuela[1], J. Balibrea[1], M. Sydorenko[1], D. Olivares-Osuna[1], E. Castillo-Agea[1], V. Rodilla[1]*
*[1]Josep Carreras Leukaemia Research Institute, Genesis of cancer, Badalona, Spain*

**Introduction**

The outcome for non-Hodgkin lymphoma (NHL) patients varies, with many experiencing relapses within 2-3 years post-standard chemotherapy. Chemotherapeutic drugs are genotoxic agents that, while targeting proliferating cells indiscriminately, also induce senescence in healthy tissues, leading to long-term side effects resembling age-related conditions. Clinical studies involving cancer survivors treated with chemotherapy during childhood suggest that certain chemotherapies induce a variety of long-term side effects resembling age-related pathologies, including organ dysfunction, cognitive impairment, and secondary neoplasms. While cytotoxicity-related effects are common in NHL patients, the contribution of therapy-induced senescence (TIS) to disease progression remains unknown.

**Material and Methods**

To uncover the role of senescent cells in NHL progression, we combined single-cell RNA-sequencing with functional experiments in immunocompetent mice.

**Results and Discussions**

We found that the CHOP cocktail induced the expression of canonical senescence and senescence-associated secretory phenotype (SASP) factors in healthy mice tissues. This in turn facilitated lymphoma dissemination, mainly to the liver, which is frequently involved in extranodal NHL spreading. Subsequent RNA sequencing and principal component analysis (PCA) revealed major changes in liver gene expression profile, with *Cdkn1a* (p21) as a top hit among the significantly upregulated genes in CHOP-treated mice. Moreover, CHOP upregulated expression of the pro-inflammatory IL-6-JAK-STAT and NF-Kβ pathways, further supporting the role of TIS in lymphoma dissemination. Importantly, single-cell transcriptomics identified endothelial cells as a major contributor to TIS, with notable upregulation of *Cdkn1a* (p21). Interestingly, endothelial cells from CHOP-treated mice displayed upregulation of genes

© 2024The Authors. Molecular Oncology is published by John Wiley & Sons Ltd on behalf of Federation of European Biochemical Societies promoting adhesion molecule and chemokine expression, leukocyte recruitment, and vascular permeability.

**Conclusion**

Overall, our findings show that chemotherapy pre-treatment primes the host tissue microenvironment for lymphoma dissemination, being endothelial cells, a major population undergoing TIS. Endothelial senescent cells are pro-inflammatory, have an impaired vascular integrity and permeability and contribute to oxidative stress. Considering the implications of endothelial senescence in age-related vascular diseases, targeting senescent endothelial cells may offer a novel approach to mitigate chemotherapy-derived side effects and hamper NHL progression.

### EACR2024-0969

### A vascularized lung tumor-on-chip approach to decipher how endothelium and tumor microenvironment crosstalk shapes immune infiltration

*S. Ladaigue[1], M. Nurmik[1], L. Christine[1], G. Giacomo[2], M.G. Fatima[1], Z. Gérard[3], S. Fabrice[4], D. Stéphanie[2], P. Maria Carla[1]*

*[1]Institut Curie, INSERM U830- Stress and Cancer Laboratory- PSL Research University, Paris, France*

*[2]Institut Curie, CNRS UMR168- Laboratoire Physico Chimie Curie- Institut Pierre-Gilles de Gennes- PSL Research University, Paris, France*

*[3]Hospital Bichat-Claude Bernard, Université Paris Cité- CIC INSERM 1425- Thoracic Oncology Department, Paris, France*

*[4]CNRS/IIS/Centre Oscar Lambret, Lille University SMMiL-E Project- CNRS Délégation Hauts-de-France, Lille, France*

**Introduction**

The vascular barrier is an entry gate to tissues for circulating immune cells. Endothelial adhesion molecules, chemokines, and receptor-ligand interactions finely regulate this entrance. The tumor micro-environment (TME) is known to promote endothelial cell (EC) anergy, i.e. a condition characterized by lack of reactions to pro-inflammatory stimuli, thus participating to tumor immune exclusion and potentially treatment resistance. We present here an innovative 3D model of a vascularized lung TME to better understand EC immunomodulatory functions.

**Material and Methods**

We developed a vascularized lung tumor-on-chip (vTOC) using soft lithography and the guiding needle technic to create a perfusable central vessel of 200 μm diameter surrounded by a collagen gel embedding TME components. Non-small cell lung carcinoma (NSCLC) patient-derived EC were isolated, amplified and used to create the central vessel. Regarding other TME components, we used lung cancer cell lines or primary NSCLC cancer cells, primary lung cancer-associated-fibroblasts (CAF) and tumor-infiltrating immune cells. To assess the impact of TME on EC gene expression, we performed co-culture on vTOC followed by specific isolation and processing of EC RNAs for RT-qPCR and RNAseq transcriptomic analysis.

**Results and Discussions**

Using this vTOC model we found out that cancer cell lines and primary CAF decreased mRNA expression of *vcam-1* gene, which codes for leukocyte adhesion protein, in patient-derived lung EC, potentially reducing immune infiltration. Moreover, we succeeded to freshly isolate four primary autologous cell populations (tumor cells, CAF, CD8+ T-cell and EC) from NSCLC surgical resection. As a proof-of-concept experiment, we succeeded to co-culture the four cell types reconstituting an autologous vascularized lung TME on a chip. This vTOC approach is therefore suitable to recapitulate some features of TME impact on patient-derived EC and will be valuable to study the impact of TME composition and/or treatment on immune infiltration by video-microscopy for cell dynamics and by immunostaining on fixed paraffin-embedded vTOC slices for immune phenotypes.

**Conclusion**

Primary, autologous, patient-derived vTOC are useful clinically relevant models to decipher fundamental behaviors within the TME and to characterize the impact of anti-cancer drugs, providing new tools to better understand the mechanisms underlying drug sensitivity or resistance of cancer cells at tumor ecosystem level.

### EACR2024-0978

### The T-cell metabolic phenotype is dependent on risk status in Multiple Myeloma

*M. John[1], N. Afrin[1], E. Stanojkovska[1], M. Krämer[1], X. Zhou[2], M. Helal[1], H. Einsele[2], K.M. Kortüm[2], L. Rasche[1,2], A. Riedel[1]*

*[1]University Hospital Würzburg, Mildred-Scheel Nachwuchszentrum, Würzburg, Germany*

*[2]University Hospital Würzburg, Internal Medicine II, Würzburg, Germany*

**Introduction**

The prognosis of multiple myeloma (MM) has dramatically improved in recent years. Unfortunately, ~15% of patients with high-risk (HR) disease do not benefit to the same extent, due to a higher chance of getting infectious diseases leading to non-relapse mortality and a lower response rate to novel T-cell based immunotherapies. These facts indicate a link between HR disease and impaired T-cell function. To dissect this link, we analyzed the cellular and metabolic bone marrow (BM) niche as a function of risk status (RS).

**Material and Methods**

In plasma cells (CD138+) of 221 MM patients the risk status was assessed using the gene expression profiling based SKY92 micro-array platform. Flow cytometry with 20 different markers was performed on the corresponding CD138- fractions. For 20 patients single cell RNA sequencing (scRNAseq) was performed. Water soluble metabolites were analyzed in BM plasma from the same samples.

**Results and Discussions**

We find profound differences in T-cell composition between HR and standard risk (SR) patients, characterized by a lower number of T cells, but an increased $CD8a^{+}/CD4^{+}$ T cell ratio in HR disease. This increase was associated with lines of therapy and further analysis identified a therapy-induced reduction in naïve T

© 2024 The Authors. Molecular Oncology is published by John Wiley & Sons Ltd on behalf of Federation of European Biochemical Societies cells. ScRNAseq indicated an increase in CD8a$^+$ effector memory CD45RA$^+$ (EMRA) T cells, which upregulated pathways linked with apoptosis and simultaneously downregulated pathways linked with immune response in HR patients. Strikingly, one CD8a$^+$ EMRA population, enriched in treated SR patients, upregulate pathways linked with oxidative phosphorylation, indicating a better memory state of these cells. To investigate the metabolic mechanisms shaping this immune cell composition and function in the BM, we compared water-soluble metabolites from BM plasma between untreated and treated HR and SR patients. Untreated HR patients showed decreased glutamine levels compared to SR patients. We further identified changes in arginine metabolism.

**Conclusion**

Our study highlights an emerging characteristic of HR disease that is not solely related to genomic changes in tumor cells, but rather to profound differences in T cell frequencies and functions. Our study supports metabolic changes in the BM microenvironment as a driver or modifier of these pathological processes.

### EACR2024-1022

### Metformin - an oral antidiabetic agent- improves chemotherapy efficacy in colorectal cancer by inhibiting hypoxia-induced metabolic adaptation

*O. Bayrak[1], M.B. Kemahli[1], M.E. Kasap[1], Y. Basbinar[2], S. Bayrak[2]*

*[1]Dokuz Eylul University Institute of Health Sciences, Department of Translational Oncology, Izmir, Turkiye*

*[2]Dokuz Eylul University Institute of Oncology, Department of Translational Oncology, Izmir, Turkiye*

**Introduction**

Alterations in the hypoxic tumour microenvironment and the development of hypoxia-associated chemoresistance arise when cells modify their energy metabolism through the Warburg effect. New therapeutic strategies are needed in this area as this mechanism can inactivate chemotherapeutic agents commonly used in colorectal cancer. This study aims to investigate the metabolic adaptation of metformin, an oral anti-diabetic drug, in a hypoxic environment and the reversibility of hypoxia-induced drug resistance.

**Material and Methods**

In this study, we investigated the effects of oxaliplatin combined with metformin, which is commonly used in the clinic, under normoxic and hypoxic conditions in HCT-116 and HT-29 colorectal cancer cell lines with different mutations using viability assay, wound healing assays and immunofluorescence staining to measure the expression levels of HIF-1 alpha, GLUT-1 and LDH-A. The chemical hypoxia method using sodium sulfite, which has been optimised in our previous studies, was used for the generation of hypoxic conditions.

**Results and Discussions**

No difference was observed between the results of oxaliplatin monotherapy and combined treatment with metformin under normoxic conditions. Under hypoxic conditions, oxaliplatin alone was ineffective compared to the control group. However, when combined with metformin, oxaliplatin was more effective. Furthermore, immunofluorescence results showed that the expression levels of E-cadherin, LDH-A, HIF-1alpha and GLUT-1, which were observed to be upregulated under hypoxic conditions, were downregulated under hypoxic conditions after combination treatment. These results were consistent with the wound healing assay, and hypoxic combined treatment decreased wound patency.

**Conclusion**

We found that inhibition of HIF-1alpha, which is required for improved metabolic adaptation in hypoxia, had positive feedback on cell behaviour and drug effectiveness. Metformin, which inhibits HIF-1alpha, is hypothesised to increase metabolic activity in the tumour microenvironment. In this regard, metformin may be a novel therapeutic target to reverse hypoxia-associated drug resistance and metabolic adaptation in the hypoxic tumor microenvironment, especially to platinum.

*O.B. was supported by TUBITAK 2211C Domestic Priority Doctoral Scholarship Program, and Council of Higher Education 100/2000 Scholarship in Priority Field Program. The study is supported by Presidency of Health Institutes of Turkey with project number 28664.*

### EACR2024-1068

### Circular PVT1 exerts a pro-tumorigenic and immune-suppressor functions in Acute Myeloid Leukemia

*M. Ghetti[1], L. Ledda[1], F. Ruggieri[2], M.T. Bochicchio[1], E. Fonzi[3], M. Paganelli[1], G. Marconi[2], M. Gottardi[4], G. Martinelli[5], G. Simonetti[1]*

*[1]IRCCS Istituto Scientifico Romagnolo per lo Studio e la Cura dei Tumori "Dino Amadori" I.R.S.T., Biosciences laboratory, Meldola, Italy*

*[2]Università di Bologna, Dipartimento di Scienze Mediche e Chirurgiche, Bologna, Italy*

*[3]IRCCS Istituto Scientifico Romagnolo per lo Studio e la Cura dei Tumori "Dino Amadori" I.R.S.T., Unit of Biostatistics and Clinical Trials, Meldola, Italy*

*[4]Veneto Institute of Oncology IOV-IRCCS, Department of Oncology, Castelfranco Veneto, Italy*

*[5]IRCCS Istituto Scientifico Romagnolo per lo Studio e la Cura dei Tumori "Dino Amadori" I.R.S.T., Scientific Directorate, Meldola, Italy*

**Introduction**

circRNAs have been reported to play pivotal roles in cancer onset and progression. Hsa_circ_0001821, also known as circPVT1,originates from the circularization of exon 2 (410 bp length) of the PVT1 gene, which maps at the 8q24.21chromosomal band and transcribes both linear and circular RNAs. We have recently shown that circPVT1 is overexpressed in Acute Myeloid Leukemia (AML), as also reported in several cancer types. Moreover, a role of circPVT1 in the regulation of immune cell differentiation and function has been suggested. This study aims to address two faces of the same coin, namely circPVT1 roles in promoting an aggressive malignant phenotype and suppressing anti-tumor immune responses.

**Material and Methods**

We designed one antisense-oligonucleotide (ASO), spanning the junction region of circPVT1 inOCI-AML3 and KASUMI-1 cells and lentiviral shRNA targeting circPVT1 junction in OCI-AML3. The biological

© 2024The Authors. Molecular Oncology is published by John Wiley & Sons Ltd on behalf of Federation of European Biochemical Societies consequences of circPVT1 downregulation (KD), were assessed by cell viability, apoptotis, RNAseq and western blot analyses. Moreover, we combined circPVT1 silencing with ATR inhibitor(VE-821) or PARP1/2 inhibitor (talazoparib) on both cell lines. Co-culture studies of T cells from healthy subjects and circPVT1-KD OCI-AML3 cells were set up for 16h and cell killing assessed by flow cytometry analyses.

**Results and Discussions**

circPVT1 downregulation induced an impairment of leukemia cell growth and an increase of apoptosis in OCI-AML3 and KASUMI-1 AML cell lines. By transcriptome analyses, on AML cell lines and primary samples, and protein validation, we found that circPVT1 targeting regulates genes involved in DNA repair processes including the Homologous Recombination pathway and immune response-related pathways. Accordingly, circPVT1-KD cells showed a greater ability for DNA repair in response to ultraviolet radiation-induced DNA damage, as demonstrated by the reduced number of γH2AX foci. Moreover, circPVT1-KD potentiated the antiproliferative effect of ATR or PARP inhibitors on AML cell lines. Finally, circPVT1-KD silencing in AML cells enhanced T-cell cytotoxic capacity, with an increase of granzyme B, in co-culture experiments of leukemic and CD3+ T cells, but not in the presence of selected CD8+ T cells, indicating an indirect regulatory role mediated by other T cell subpopulations.

**Conclusion**

These results reported an involvement of circPVT1 in the DNA damage response, along with a role in drug response and resistance in AML and in the crosstalk between leukemic cells and the tumor microenvironment.

### EACR2024-1076
### Prognostic significance of ZEB1 and LOXL2 gene expression in relation to lymph node ratio (LNR) and TNM stage in rectal cancer

*B. Kozik[1,2], L. Todorović[3], A. Božović[2], A. Kolaković[2], T. Vasiljević[4], A. Đermanović[5], M. Đurić[6], V. Mandušić[2]*

*[1]Vinča Institute of Nuclear Sciences- National Institute of Republic of Serbia- University of Belgrade, Laboratory for Radiobiology and Molecular Genetics, Belagtade, Serbia*

*[2]Vinča Institute of Nuclear Sciences- National Institute of Republic of Serbia- University of Belgrade, Laboratory for Radiobiology and Molecular Genetics, Belgrade, Serbia*

*[3]Vinča Institute of Nuclear Sciences- National Institute of Republic of Serbia- University of Belgrade- Republic of Serbia, Laboratory for Radiobiology and Molecular Genetics, Belgrade, Serbia*

*[4]Oncology Institute of Vojvodina- Sremska Kamenica-University of Novi Sad- Faculty of Medicine, Department of Patology and Laboratory Diagnostic, Novi Sad, Serbia*

*[5]Oncology Institute of Vojvodina- Sremska Kamenica-Republic of Serbia, Department of Surgical Oncology, Sremska Kamenica, Serbia*

*[6]Oncology Institute of Vojvodina- Sremska Kamenica-University of Novi Sad- Faculty of Medicine, Department of Surgical Oncology, Novi Sad, Serbia*

**Introduction**

Disease progression after curative surgery is a major challenge in rectal cancer (RC). Genes that regulate tumor microenvironment interactions and facilitate metastatic processes are in particular focus lately as potential therapeutic targets and prognostic biomarkers. ZEB1 is a transcriptional factor that promotes tumor invasiveness via regulation of the epithelial-mesenchymal transition (EMT) process, while the LOXL2 enzyme creates premetastatic niches by remodeling the extracellular matrix. Aberrant expression of ZEB1/LOXL2 genes has been shown to enhance malignant behavior and correlate with poor clinical outcomes in colorectal cancer. This study aimed to assess the prognostic role of ZEB1/LOXL2 gene expression in rectal cancer considered as a distinct clinical entity.

**Material and Methods**

This preliminary study included 45 untreated RC patients who underwent curative resection in 2016-2018 and followed at the Oncology Institute of Vojvodina. We isolated mRNA from postoperative tumor FFPE samples. TaqMan® gene expression assays were used for quantitative real-time PCR analysis of ZEB1 and LOXL2 gene expression.

**Results and Discussions**

Within a selected cohort of patients, lymph node ratio (LNR) was found to be the strongest prognostic factor, which associated with significantly shorter overall survival ($p<0.001$) and disease-free survival ($p=0.001$). Concerning LNR, we revealed significantly increased ZEB1 expression in patients with unfavorable LNR ($p=0.043$), while elevated LOXL2 expression was also noted in the same LNR group, but without significant difference ($p=0.145$). Moreover, ZEB1/LOXL2 over-expression was associated with locally advanced stages of RC. LOXL2 expression was significantly higher in T3/4 and N1/N2 categories ($p=0.011$ and $p=0.024$, respectively), while a similar statistical trend was detected among ZEB1 expression and T3/4 categories ($p=0.064$). We also noted increased ZEB1/LOXL2 expression within tumors with lymphovascular/perineural invasion and lymphocytic infiltrate, although the observed association didn't reach statistical significance. However, obtained results support our further analyses of the impact of ZEB1/LOXL2 expression in RC progression.

**Conclusion**

Our preliminary study revealed a significant clinical correlation of ZEB1/LOXL2 expression with serious RC clinicopathologic factors, including LNR and advanced TNM stages, suggesting their potential prognostic role of disease progression, which required further analyses on a larger number of RC patients.

### EACR2024-1088
### Unravelling the role of mitochondrial release in melanoma

*N. Georges Calderón[1], C. Fuentes[1], Y. Hidalgo[1,2], L. Cereceda[1], N. Merino-Veliz[3], C. Ahumada-Marchant[3], G. Arriagada[3], F. Bustos[3], F. Alcayaga-Miranda[1,2]*

*[1]Laboratory of Nano-Regenerative Medicine- Centro de*

© 2024 The Authors. Molecular Oncology is published by John Wiley & Sons Ltd on behalf of Federation of European Biochemical Societies

*Investigación e Innovación Biomédica. CIIB- Faculty of Medicine, Universidad de los Andes, Santiago, Chile*
*[2]IMPACT, Center of Interventional Medicine for Precision and Advanced Cellular Therapy, Santiago, Chile*
*[3]Instituto de Ciencias Biomédicas- Facultad de Medicina, Universidad Andrés Bello, Santiago, Chile*

**Introduction**

Highly metastatic melanoma cells secretome promote cancer progression when compared to cells with low metastatic capacity. In recent years, secreted mitochondria from diverse cell populations have been described as a new component of the secretome involved in many physiological and pathological processes. Nevertheless, whether all cancer cells secrets mitochondria or its possible involvement in cancer progression remains unknown. In this study, we aim to search for secreted mitochondria from melanoma cancer cells using a syngeneic murine model in order to bring to scope a new possible mechanism of melanoma progression.

**Material and Methods**

The murine cell lines Melan-A, B16-F1 and B16-F10 were used as a model of healthy, cancerous, and highly metastatic cells, respectively. Transmission electron microscopy (TEM) was used to determine the presence and morphology of mitochondria within the cell lines secretome mitochondria enriched fraction (sMEF). The cell lines sMEF was characterized by measures of particle concentration by nanoparticle tracking analysis, mitochondrial DNA and Flowy Cytometry (FC) searching for differences in composition of the sMEF. Melanoma cell lines were transformed with a lentiviral particle system, enabling the stable expression of mGFP, a chimeric fluorescent protein with a mitochondrial subcellular localization peptide chain. For the in vivo analysis C57BL/6 mice were injected subcutaneously in the flank with B16-F1$^{mGFP}$ or B16F-10$^{mGFP}$. Blood samples from the tumor hosting and control animals were analyzed through FC.

**Results and Discussions**

We demonstrate for the first time the existence of mitochondria in Melan-A, B16-F1 and B16-F10 sMEF. Interestingly, the highly metastatic melanoma cell line, B16-F10 sMEF present an elevated rate of total particles, mitochondrial DNA and absolute number of granular mitochondrial probe positive events by FC in comparison to B16-F1 and Melan-A cell lines. In vivo, we identified mGFP positive particles in the blood of animals. A higher amount of mitochondrial release to circulation occurred in animals with a metastatic tumor.

**Conclusion**

Our data suggests that cancer cells differentially secret mitochondria in a malignancy-dependent manner both in vitro and in vivo. The question arises: What purpose does it serve the mitochondrial release to the melanoma? Elucidating a potential new melanoma progression holds promise for improving current therapies and diagnostic tools.

### EACR2024-1101
### MF fibroblast exosomes suppress immune cells via monocyte polarization and high PD-L1 expression

*H. Khoury[1,2], E. Hodak[1,2,3], J. Kanaeh[2], C. Arkin[2], M. Bel[1], A. Aronovich[1,2,4], B. Gorovitz[2,3,5], I. Lubin[6], L. Moyal[1,2,7]*
*[1]Felsenstein Research Center, Laboratory for Molecular Dermatology, Petach Tikva, Israel*
*[2]Tel Aviv University, Sackler Faculty of Medicine, Tel Aviv, Israel*
*[3]Rabin Medical Center, Davidoff Cancer Center, Petach Tikva, Israel*
*[4]Rabin Medical Center, Division of Dermatology, Petach Tikva, Israel*
*[5]Felsenstein Research Center, Laboratory for Molecular Dermatology, Tel Aviv University, Israel*
*[6]Tel Aviv University, Core Facility- Felsenstein Medical Research Center- Rabin Medical Center- Sackler Faculty of Medicine, Tel Aviv, Israel*
*[7]Rabin Medical Center, Davidoff Cancer, Petach Tikva, Israel*

**Introduction**

Mycosis fungoides (MF) represents most common subtype of extranodal non-Hodgkin's cutaneous T cell lymphoma (CTCL). Cancer associated fibroblasts (CAFs) are a major component in the stroma of the tumor micro-environment (TME). Exosomes are nano-size extra cellular vesicles secreted by all cells for intercellular communications. We previously demonstrated the presence of CAFs in the TME of MF and their role in MF cell migration and chemoresistance. Herein, we studied the effect of MF fibroblast (MF-F) vs normal fibroblast (N-F) exosomes on the immune cells.

**Material and Methods**

Primary fibroblasts were established from MF-patient biopsies and normal skin of healthy donors. Exosomes were isolated from the supernatant of MF-F, N-F by ultracentrifugation, and were confirmed by electron microscope Nanosight and FACS for CD81. The internalization of fibroblast exosomes into normal PBMCs from healthy donors (nPBMCs) were confirmed by microscope analysis of labeled exosomes. Immune cell characterization was done by mass flow cytometry (CyTOF). M1 ,M2 and PD-L1 analysis was done by FACS and qRT-PCR for protein markers and cytokine expression. CD4+ T helper (Th) and CD8+ T cytotoxic (Tcyt) were isolated by negative selection and their viability was tested by trypan blue staining. Detection of specific microRNAs were analyzed by qRT-PCR.

**Results and Discussions**

MF-Fs were found to secrete exosomes with a distinct protein profile compared to N-Fs. These exosomes were internalized by nPBMCs and significantly reduce their viability. Importantly, MF-F exosomes promoted polarization of macrophages towards an M2-like phenotype, upregulated PD-L1 expression and along with monocyte they reduced the viability of Th and T cyt. Exosomal microRNAs associated with M2 polarization and PD-L1 increase that were tested in MF-F vs N-F exosomes Revealed high miR-23a-3p in MF-F vs N-F exosomes. Our research illuminates, for the first time, the role of exosomes from fibroblasts in the TME of MF and their impact on immune regulation, providing insight into tumors evade immune defense with potential strategies

© 2024 The Authors. Molecular Oncology is published by John Wiley & Sons Ltd on behalf of Federation of European Biochemical Societies targeting exosomal communication and immune modulation.

**Conclusion**

Our study highlights the pivotal role of MF-F derived exosomes in shaping a tumor-supportive micro-environment in MF. By promoting M2 macrophage polarization and increasing their PD-L1 expression, for immune evasion and cancer progression. Targeting fibroblast exosomes may offer new therapeutic avenues for MF.

### EACR2024-1124
### Studying the antitumoral effect of the cell-penetrating peptide TAT-Cx43266-283-mod in different GBM preclinical models

*M. Martínez-Fernández[1], A. Álvarez-Vázquez[1], E. Jiménez-Madrona[1], R. Flores-Hernández[1], A. Tabernero[1]*

*[1]Instituto de Neurociencias de Castilla y León INCyL-Universidad de Salamanca, Instituto de Investigación Biomédica de Salamanca IBSAL, Biochemistry and Molecular Biology, Salamanca, Spain*

**Introduction**

Glioblastoma (GBM) is the most common and aggressive primary brain tumor. Part of its malignancy lies in a subpopulation of cells within the tumor that have stem-cell properties (glioma stem cells, GSCs), which are resistant to standard therapies and responsible for tumor recurrence. Since GSCs constitute an interesting therapeutic target, our laboratory has designed a cell-penetrating peptide, TAT-Cx43266-283, that has shown promising antitumor effects in preclinical models of GBM, reducing GSCs proliferation, migration and invasion and increasing the survival of GBM-bearing mice by inhibiting c-Src oncoprotein activity in these cells. Recently, several modifications have been made in this peptide in order to improve its antitumoral efficacy. Therefore, we aimed to compare the effect of this modified peptide (TAT-Cx43266-283-mod) with that of TAT-Cx43266-283 and the chemotherapeutic temozolomide (TMZ) in different GBM preclinical models. Furthermore, it has been shown that infiltrative GBM cells that evade tumor surgical resection are highly tumorigenic, so that recurrent tumors turn out to be more invasive than non-resected ones. Growth-associated protein 43 (GAP43) is a major structural protein of tumor microtubes, which have been identified as relevant cellular structures involved in intercellular communication, invasion and therapy resistance in GBM. In this sense, we also wanted to study the effect of TAT-Cx43266-283-mod treatment in GAP43 expression.

**Material and Methods**

For these purposes, we first assessed cell viability of two murine GBM stem-cell lines (GL261 and SB28) after treatment with TAT-Cx43266-283-mod. Next, as resection is the first-line treatment against GBM, we studied the effect of administering TAT-Cx43266-283-mod in a murine model of tumor resection. To this aim, the murine GBM stem-cell line GL261 was intracranially injected into the brains of C57BL/6 mice, and once the tumors had already developed, animals were subjected to GBM resection or sham operation and treated either with TAT-Cx43266-283-mod or saline until the end of the experiment. Finally, immunofluorescence analysis for GAP43 staining were performed in brain sections of these mice.

**Results and Discussions**

On one hand, we observed that TAT-Cx43266-283-mod had a higher antitumoral effect than TMZ and its counterpart TAT-Cx43266-283 in GL261 and SB28 stem-cell lines. On the other hand, although the results are preliminary and we are exploring them further, the effect of TAT-Cx43266-283-mod in combination with tumor resection on mice survival is promising, and GAP43 expression seems to be lower in the recurrent tumors of mice treated with TAT-Cx43266-283-mod compared to those treated with saline, which would explain the longer survival of these animals.

**Conclusion**

Altogether, these results bring to light the therapeutic potential of TAT-Cx43266-283-mod as a possible treatment for GBM.

*COI: TAT-Cx43266–283mod is a patent (ID: PCT1367.115) from the University of Salamanca*

### EACR2024-1127
### Targeting IL1R1+ Cancer-Associated Fibroblasts: A Novel Therapeutic Avenue in Colorectal Cancer

*V. Pozdeev[1], E. Koncina[1], M. Nurmik[2], C. Gilson[1], H. Dolznig[3], E. Letellier[1]*

*[1]University of Luxembourg, Department of Life Sciences and Medicine, Belval, Luxembourg*

*[2]Institut Curie, Cancer- Heterogeneity- Instability and Plasticity - CHIP U830, Paris, France*

*[3]Medical University of Vienna, Institute of Medical Genetics- Center for Pathobiochemistry and Genetics, Vienna, Austria*

**Introduction**

Among the various components of the tumor micro-environment (TME), cancer-associated fibroblasts (CAFs) have garnered increasing attention for their role in cancer progression and therapy resistance. Our study focuses on the functional and molecular heterogeneity of CAFs within the TME of colorectal cancer (CRC), aiming to identify targetable pro-tumorigenic fibroblast subtypes.

**Material and Methods**

By using single-cell RNA sequencing and spatial transcriptomics of patient-derived tumor samples, we identified an IL1R1$^+$ CAF subtype in CRC. To investigate the effect of this subtype, we used 3D organotypic co-culture assays of IL1R1$^+$ CAFs with tumor cells followed by gene set enrichment analysis and proliferation assays. We further investigated the immuno-modulatory function of the IL1R1$^+$ CAF subtype by analysing T cell proliferation and macrophage polarisation in direct co-culture assays. We generated a genetically engineered mouse model (GEMM) with a conditional deletion of the IL1R1 gene in the stromal compartment to study the effect of the IL1R1$^+$ CAF subtype in a CRC mouse model and employed Anakinra to clinically target the IL1R1$^+$ CAF subtype in vivo.

Molecular Oncology 18 (Suppl. 1) (2024) 1–495
© 2024 The Authors. Molecular Oncology is published by John Wiley & Sons Ltd on behalf of Federation of European Biochemical Societies

Finally, clinical relevance was assessed using publicly available datasets.
**Results and Discussions**
We showed that IL1R1$^+$ CAFs are characterised by an elevated expression of inflammatory CAF (iCAF) markers, both in bulk datasets and on immunofluorescence staining of CRC tumor tissues. IL1R1$^+$ CAFs induced proliferation of tumor cells by activating pro-tumorigenic pathways in 3D organotypic co-culture models. Importantly, the IL1R1$^+$ CAF subtype was linked to an increase of the pro-tumorigenic Th17 cell population, while simultaneously impeding the activation of CD8$^+$ T cell and macrophages. Accordingly, using a fibroblast-specific IL1R1 KO mouse or the IL-1 receptor antagonist Anakinra, we observed in vivo a reduced tumor growth accompanied by a limited infiltration of Th17 T cells into the tumor bed and an overall reduced immunosuppressive TME. This was further underlined by the presence of immune exhaustion markers in CRC patients, who present with IL1R1 expressing CAFs. Additionally, the IL1R1$^+$ CAF subtype was associated with reduced survival in hard-to-treat, stromal-enriched CRC patients.
**Conclusion**
Altogether, our study highlights the presence of a subpopulation of IL-1-signaling fibroblasts that drives tumor growth and favors immune evasion in CRC patients and suggests that targeting the IL1R1-specific CAFs might be a potential new therapeutic avenue in CRC.

### EACR2024-1128
### Impact of Endoplasmic Reticulum Stress on Immune Surveillance and Immunotherapy Efficacy in Renal and Ovarian Cancer

*L. Moráň[1,2], B. Vavrušáková[1,3], K. Lenka[1], R. Bartošová[1], K. Součková[4], K. Vašíčková[1], M. Svoboda[5], A. Poprach[6]*
*[1]Masaryk Memorial Cancer Institute MMCI, Research Center for Applied Molecular Oncology RECAMO, Brno, Czech Republic*
*[2]Masaryk University- Faculty of Medicine, Department of Histology and Embryology, Brno, Czech Republic*
*[3]Masaryk University, Faculty of Medicine, Brno, Czech Republic*
*[4]Masaryk University, Central European Institute of Technology, Brno, Czech Republic*
*[5]Masaryk Memorial Cancer Institute MMCI, KKOP – Research Center for Applied molecular Oncology RECAMO, Brno, Czech Republic*
*[6]Masaryk Memorial Cancer Institute MMCI, KKOP-Research Center for Applied molecular Oncology RECAMO, Brno, Czech Republic*

**Introduction**
In complex tumor-host dynamics, the immune system plays a dual role, with the capacity to both promote and inhibit tumor growth influenced by intricate molecular interactions in the tumor-immune microenvironment (TIME). Factors like low pH and hypoxia within TIME trigger Endoplasmic Reticulum Stress (ERS), affecting immune cells. Stressed immune cells demonstrate reduced antigen presentation, adopt suppressive behaviors, and may undergo apoptosis. Alleviating ERS is thus seen as a potential strategy to enhance immune surveillance and improve the efficacy of immunotherapy in cancer treatment. Our study explores tumor-immune interactions in ovarian (OC) and renal (RCC) cancers, focusing on ERS.
**Material and Methods**
Here we co-cultured OC and RCC cancer cells with peripheral blood mononuclear cells (PBMC), manipulating ERS using tunicamycin and tauroursodeoxycholic acid (TUDCA) in 2D and 3D. We employed cell viability assays (e.g. MTT), live cell imaging, gene and protein expression detection (e.g. qPCR, immunofluorescence (IF), Western Blot), immune cell activity detection, and electron microscopy (SEM, TEM). For clinical samples we linked ERS markers to patient outcomes using IF, qPCR and flow cytometry. The obtained results were subsequently applied on in vivo mouse models.
**Results and Discussions**
Relieving ERS increased the infiltration of PBMC into tumor spheroids and reduced the expression of BiP and PERK in PBMC. In vitro, the viability of ovarian and renal cancer cells increased when cultured alone after ERS alleviation. However, when co-cultured with PBMC, their viability decreased, indicating enhanced immune cell response. Additionally, the growth and migration of ovarian and renal cancer cells were limited in these co-cultures, along with increased expression of BiP and CHOP and an elevated CHOP/BiP ratio. We connected these findings to the molecular background of ERS in patient samples and observed a positive correlation between a favorable clinical outcome, higher lymphocyte infiltration at the tumor site, and increased levels of BiP and CHOP in cancer cells. We further applied these results to OC and RCC in vivo syngeneic mouse models, currently with very promising outcomes.
**Conclusion**
We propose ERS alleviation as a suitable approach to improve the survival and effectiveness of immune cell populations within hostile tumor environment with positive effects on anticancer immunotherapy.
*Supported by Ministry of Health of the Czech Republic, grant nr. NU21-03-00539.* *All rights reserved.*

### EACR2024-1169
### Ketogenic Diet Induces Anti-Tumorigenic Effects in Colorectal Cancer through Microbial Production of the long-chain fatty acid Stearate

*M. Tsenkova[1], M. Brauer[1], M. Kasakin[2], S. Busi[3], C. Linster[4], P. Wilmes[2], E. Letellier[1]*
*[1]University of Luxembourg, Department of Life Sciences and Medicine, Belval, Luxembourg*
*[2]University of Luxembourg, Luxembourg Centre for Systems Biomedicine, Belval, Luxembourg*
*[3]UK, Centre for Ecology and Hydrology, Wallingford, United Kingdom*
*[4]University of Luxembourg, Luxembourg Center for Systems Biomedicine, Belval, Luxembourg*

**Introduction**
Colorectal cancer (CRC) patients have been shown to possess an altered gut microbiome. Diet is a well-known

© 2024 The Authors. Molecular Oncology is published by John Wiley & Sons Ltd on behalf of Federation of European Biochemical Societies modulator of the gut microbiome. An attenuating effect of the ketogenic diet (KD) on CRC cell growth has been previously observed, however, the role of the gut microbiome in driving this effect remains unknown.

**Material and Methods**

We first generated a humanized gut microbiome mouse model, by transplanting stool samples from five healthy human donors into gnotobiotic or antibiotic-treated mice. We then combined this model with a therapeutically administered KD in an inflammation-driven CRC model (AOM/DSS) and followed tumor growth over time. Microbial transplantation experiments from KD-fed mice into recipient mice maintained under standard chow were performed to investigate the causal role of the microbiome in driving the anti-tumorigenic effect of KD. We combined metagenomics with metabolomics to mechanistically investigate the microbiome-driven effect of the KD in CRC.

**Results and Discussions**

We were able to show the cancer-suppressing properties of the KD in CRC. Most importantly, we demonstrated, for the first time, the causal role of the gut microbiome in maintaining this effect, through transplantation of the microbial community. We reported long-lasting alterations in gut microbiome function in the absence of maintained selective pressure by the KD. By combining metagenomics with metabolomics in our experiments, we identified microbial lipid metabolism to play an important role in KD-induced anti-tumorigenic effects. Specifically, fecal stearic acid levels were increased not only upon KD consumption, but also upon microbial transplantation, suggesting that the shift in the microbial community contributes to the changes in the fatty acid pool. Importantly, potential stearate-producing members were enriched in ketogenic conditions, whereas consumers were depleted. Finally, we observed reduced proliferation of tumor cells upon supplementation with stearic acid.

**Conclusion**

Our study demonstrates that the response to the ketogenic diet is mediated by the gut microbiome, and identifies the long-chain fatty acid, stearic acid, as a putative microbiome-derived anti-cancer metabolite.

### EACR2024-1205

### Molecular mechanisms underlying T cell acute leukemia dissemination

I. Pacheco-Leyva[1], T. Costa[1], M. Baessa[1], N. R. dos Santos[1]

[1]i3S - Instituto de Investigação e Inovação em Saúde, Intercellular Communication and Cancer, Porto, Portugal

**Introduction**

T-cell acute lymphoblastic leukemia/lymphoma (T-ALL/LBL) involves mainly the bone marrow, blood, and lymphoid organs, but can disseminate to other organs, aggravating disease. It was previously reported by us and others that absence of T-cell receptor (TCR) expression in T-ALL/LBL mouse models led to reduced dissemination of leukemic cells from the thymus to peripheral lymphoid organs, most notably lymph nodes. We aimed to understand which molecules could be involved in this TCR-dependent property.

**Material and Methods**

Gene mRNA expression, flow cytometry immunofluorescence analysis were performed from leukemic cells from the ETV6-JAK2 fusion transgenic mice (EJ-Tg). EJ-Tg mice were bred with Rag2, Ccr7 and Nfκb2 knockout (KO) mice; and with TCR-HY transgenic mice. Leukemic cells were infused intravenously in recipient mice.

**Results and Discussions**

Infused ETV6::JAK2 transgenic mouse leukemic T cells lacking TCR expression (i.e., EJ-Tg;Rag2$^{-/-}$) colonized much less efficiently the lymph nodes and spleens of recipient mice than EJ-Tg leukemic cells expressing endogenous or transgenic HY-TCR. Interestingly EJ-Tg;Rag2$^{-/-}$ leukemic cells expressed reduced levels of the Ccr7 chemokine receptor, a T-cell migration mediator involved in both thymic egress and peripheral lymphoid organ homing. By stimulating human T-ALL cell lines with CD3 antibody or the PMA phorbol ester, we confirmed that CCR7 expression was induced by TCR signaling. To study the role of CCR7 in vivo, we bred EJ-Tg mice with Ccr7 KO mice. Similarly to EJ-Tg mice lacking TCR (i.e. Tcrα$^{-/-}$ or Rag2$^{-/-}$), EJ-Tg;Ccr7$^{-/-}$ mice presented significantly larger thymic lymphomas and reduced splenic and lymph nodal involvement than Ccr7-sufficient littermates. By breeding EJ-Tg mice with OT-I TCR transgene and Ccr7 KO, we observed that the absence of CCR7 in OT-I;EJ-Tg mice resulted in lower lymph node and spleen dissemination of leukemic cells. To verify whether leukemic cells expressing Ccr7 was involved in homing to lymphoid organs, Ccr7-expressing EJ-Tg leukemic cells were infused in control littermates or mice KO for the Nfκb2 transcription factor, which express reduced levels of the Ccr7 ligands, in the lymph nodes. Infused leukemic cells colonized less efficiently the lymph nodes of Nfκb2-deficient mice, with no differences in the spleen and liver.

**Conclusion**

We conclude that TCR signaling is associated with expression of proteins associated with leukemic dissemination to specific niches and that CCR7 is a potential mediator of that property.

### EACR2024-1218

### Systematic Review of Tumor Microenvironment in Ovarian Cancer: Implications for Therapeutic Targeting

L.R. Rajmohan[1], P. p[2], P. d[1]

[1]ACS Medical College & Hospital, General Medicine, Chennai, India

[2]ACS Medical College & Hospital, Biochemistry, Chennai, India

**Introduction**

The tumor microenvironment (TME) plays a crucial role in ovarian cancer progression and treatment response. Understanding its influence is pivotal for devising effective therapeutic strategies. This systematic review explores the impact of TME on disease progression, treatment response, and patient outcomes, along with the therapeutic interventions targeting TME components.

**Material and Methods**

© 2024 The Authors. Molecular Oncology is published by John Wiley & Sons Ltd on behalf of Federation of European Biochemical Societies

A comprehensive literature search was conducted in PubMed, Embase, and Scopus databases to identify relevant studies focusing on the tumor microenvironment in ovarian cancer. Studies investigating the influence of TME on disease progression, treatment response, and patient outcomes, as well as therapeutic strategies targeting TME components, were included. Data synthesis and analysis were performed to evaluate the findings.

**Results and Discussions**

The TME of ovarian cancer, characterized by immune cell infiltration, angiogenesis, and extracellular matrix remodeling, significantly influences disease progression, treatment resistance, and patient survival. Therapeutic approaches targeting TME components, such as immunotherapy, anti-angiogenic therapy, and stromal modulation, have shown promising results in preclinical and clinical studies. However, challenges remain in translating these findings into clinical practice.

**Conclusion**

Understanding the dynamic interplay between ovarian cancer cells and their microenvironment is essential for developing effective therapeutic strategies. Targeting TME components holds immense potential for improving patient outcomes in ovarian cancer.

### EACR2024-1223
### DYRK1A exerts an effect on the pancreatic tumor microenvironment by regulating the properties of cancer-associated fibroblasts

*S. Pascual-Sabater[1,2], S. Varhadi[3], C. Di Vona[3,4], S. De la Luna[3,4], C. Fillat[1,2,4]*
*[1]Institut d'Investigacions Biomèdiques August Pi i Sunyer (IDIBAPS), Liver, Digestive System and Metabolism, Barcelona, Spain*
*[2]Medicine Faculty, University of Barcelona (UB), Barcelona, Spain*
*[3]Centre for Genomic Regulation (CRG), Genome Biology, Barcelona, Spain*
*[4]Centro de Investigación Biomédica en Red en Enfermedades Raras (CIBERER), Madrid, Spain*

**Introduction**

Dual-specificity tyrosine-regulated kinase 1A (DYRK1A) kinase has been associated with a broad number of cellular processes deregulated in cancer. Previous work has revealed its protumorigenic function in pancreatic ductal adenocarcinoma (PDAC), where it enhances c-MET/EGFR signaling in malignant cells. PDAC is characterized by an abundant desmoplastic stroma primarily composed of cancer-associated fibroblasts (CAFs), which act as main orchestrators of the tumour microenvironment through the secretion of a vast repertoire of factors that promote tumour growth, drug resistance and immunosuppression. In this work, we aimed at elucidating the impact of DYRK1A expression in pancreatic CAFs.

**Material and Methods**

DYRK1A expression in CAFs was confirmed by immunohistochemical analysis of pancreatic primary tumours. CAFs were generated from resected pancreatic tumours (n=3) and immortalized prior to DYRK1A knockdown using lentiviral particles. The secretome of control and shDYRK1A CAFs was compared by label-free mass spectrometry analysis of 48h supernatants. Physical properties of CAFs after DYRK1A knockdown were analyzed by performing collagen contraction assays and atomic force microscopy. Migration of CAFs and cancer cells was assessed using transwell assays and DAPI staining.

**Results and Discussions**

We have analysed the expression of DYRK1A in a set of patient-derived CAF lines and observed an over-expression of DYRK1A when compared to normal fibroblasts. Using 3 different immortalized CAF lines, we observed that shDYRK1A CAFs were less migratory, less capable of contracting a collagen matrix and presented a more rigid cytoskeleton, suggesting an impact of DYRK1A expression on cell-intrinsic mechanical properties. Moreover, we identified a DYRK1A-dependent secretome that included CXCL12, a chemokine reported to promote pancreatic cancer progression. Interestingly, PANC-1 cancer cells showed less migratory properties when exposed to shDYRK1A CAF conditioned media (CM) compared to control CAF CM. Further supporting an effect on paracrine communication, organoids cultured with shDYRK1A CAFs were fewer and smaller than those formed in the presence of shCTRL CAFs.

**Conclusion**

Taken together, these findings suggest that the pro-tumorigenic function of DYRK1A in PDAC cannot solely be attributed to its influence on malignant cells; rather, it also involves cell-intrinsic effects in CAFs and changes in their paracrine communication with other cell types within pancreatic tumours.

### EACR2024-1235
### Optimised Protocol for Scaffold-Free Spheroid Model: 3D Cell Culture of Metastatic Oral Squamous Cell Carcinoma HSC3 Line

*L. de Oliveira Siquara da Rocha[1], T. Bacelar Sacramento de Araujo[1], R. Rocha Nogueira[1], I. Nogueira Bastos[2], R. Borges Dias[1], B. Solano de Freitas Souza[3], D. Lambert[4], R. Della Coletta[5], V. Oliveira Silva[6], C. Gurgel Rocha[1]*
*[1]FIOCRUZ/UFBA, Laboratory of Pathology and Molecular Biology, Salvador, Brazil*
*[2]School of Dentistry of the Federal University of Bahia, Department of Propedeutics, Salvador, Brazil*
*[3]D'Or Institute for Research and Education, Center for Biotechnology and Cell Therapy, Salvador, Brazil*
*[4]The University of Sheffield, School of Clinical Dentistry, Sheffield, United Kingdom*
*[5]University of Campinas, Department of Oral Diagnosis, School of Dentistry, Campinas, Brazil*
*[6]Barretos Cancer Hospital, Molecular Oncology Research Center, Barretos, Brazil*

**Introduction**

3D in vitro systems offer advantages over the short-comings of bi-dimensional models by simulating the morphological and functional features of in vivo-like environments, such as cell-cell and cell-extracellular

© 2024 The Authors. Molecular Oncology is published by John Wiley & Sons Ltd on behalf of Federation of European Biochemical Societies matrix interactions, as well as the co-culture of different cell types. Nevertheless, these systems present technical challenges that limit their potential in cancer research requiring cell line- and culture-dependent standardization.

**Material and Methods**

This protocol details the use of a magnetic 3D bioprinting method and other associated techniques (cytotoxicity assay and histological analysis) using oral squamous cell carcinoma cells, HSC3, which offer advantages compared to existing widely used approaches.

**Results and Discussions**

This protocol is particularly timely, as it validates magnetic bioprinting as a method for the rapid deployment of 3D cultures as a tool for compound screening and development of heterotypic cultures such as co-culture of oral squamous cell carcinoma cells with cancer-associated fibroblasts (HSC3/CAFs).

**Conclusion**

According to this method, the present study proposes an optimized magnetic 3D bioprinting protocol to shape spheroids using metastatic HSC3 cells derived from a human tongue OSCC. The optimized method described here can support the use of heterotypic cultures and can be used to investigate tumor behavior and compounds or drugs screening, representing a relevant tool for the performance of translational oncology in Oral squamous cell carcinoma.

### EACR2024-1258
### Next Generation of Spatial Biology: High-Throughput Multiplexed Imaging Mass Cytometry™ With Whole Slide Modes

*F. Spada[1], H. Koppejan[1], Q. Raza[1], T.D. Pfister[1], L. Lim[1], D. Howell[1], N. Parsotam[1], D. King[1], C. Loh[1]*
*[1]Standard BioTools Inc., Markham, ON, Canada*

**Introduction**

Gaining spatial insights into the cellular composition of tumor tissue has tremendous potential to inform clinical and translational researchers about mechanisms behind spatial predictors of immunotherapy success, disease etiology and progression. Imaging Mass Cytometry™ (IMC™) is a high-plex spatial biology imaging technique that enables deep characterization of the diversity and complexity of the tumor microenvironment (TME). IMC supports detailed assessment of cell phenotype and function using 40-plus metal-tagged antibodies simultaneously on a single slide without issues associated with fluorescence-based spectral overlap, tissue auto-fluorescence or implementation of multiple washing and acquisition cycles.

**Material and Methods**

Currently, IMC enables user-defined regions of interest (ROI) in tissues to evaluate cellular and structural composition. To enhance the IMC user experience, we developed two new whole slide imaging (WSI) modes which enable streamlined workflows using ultrafast preview mode (PM) and high-throughput tissue mode (TM). PM samples the entire tissue at predefined spacing to rapidly capture a low-resolution image of all expressed markers in the antibody panel. PM generates an image in minutes to enable informed ROI placements while leaving the stained tissue intact for higher-resolution imaging. PM and TM are designed so acquisitions can easily occur on the same slide without additional processing steps. TM rapidly acquires images of the whole tissue at lower resolution (7 µm pixel size) at a quality that can be used for quantitative analysis of the tissue spatial biology. Specifically designed for high-throughput applications, TM in combination with a newly available 40-slide loader for the Hyperion XTi™ Imaging System permits automated and continuous imaging of more than 40 large tissue samples (400 mm2 per tissue) per week.

**Results and Discussions**

We showcase the application of WSI modes using the newly developed Maxpar® Human Immuno-Oncology IMC Panel Kit. The 31-marker panel was combined with catalog antibodies to create a 40-plus-marker panel that expands the ability to conduct comprehensive high-plex tumor and immune cell profiling. Tumor tissue micro-arrays (TMA) and whole tumor tissue sections were stained with the expanded panel. Single-cell analysis of selected ROIs, on whole tumor sections and TMAs, guided by PM data successfully provided quantitative analyses of spatial biology at single-cell resolution. In parallel, TM on whole tumor sections followed by pixel-based analysis provided a spatially resolved quantitative assessment of specific tumor and immune components of the TME.

**Conclusion**

This work demonstrates the expanded capabilities of IMC and establishes it as a reliable high-plex spatial biology imaging platform with high-throughput imaging capabilities ideally suited for translational and clinical applications.

### EACR2024-1259
### Novel Whole Slide Imaging Modes for Imaging Mass Cytometry Reveal Cellular and Structural Composition of Mouse Glioblastoma

*F. Spada[1], Koppejan[H.], Q. Raza[1], N. Zabinyakov[1], T.D. Pfister[1], N. Parsotam[1], D. Howell[1], L. Lim[1], C. Loh[1]*
*[1]Standard BioTools Canada Inc., Markham, ON, Canada*

**Introduction**

Mouse tumors have become widely used models in translational research of brain malignancies, helping to address the difficulties related to studying the human brain. Mouse brain can be regarded as a miniaturized model of the human brain, permitting visualization of the whole tissue to provide spatial cellular context. Imaging Mass Cytometry™ (IMC™) is a highly relevant tool capable of quantitative evaluation of the multiparametric protein composition in brain tumor microenvironment (TME) without the complications of autofluorescence, tissue degradation, and spectral overlap. The Hyperion XTi™ Imaging System (Standard BioTools™) utilizes IMC technology to simultaneously assess more than 40 individual structural and functional markers in tissue, providing insight into the organization and function of the TME.

**Material and Methods**

We demonstrate the whole slide imaging (WSI) application using a 40-marker panel composed of the

Molecular Oncology 18 (Suppl. 1) (2024) 1–495
© 2024 The Authors. Molecular Oncology is published by John Wiley & Sons Ltd on behalf of Federation of European Biochemical Societies

Maxpar OnDemand™ Mouse Immuno-Oncology IMC Panel Kit and the Maxpar® Neuro Phenotyping IMC Panel Kit on mouse normal and glioblastoma (GBM) brain tissue. The panel was composed of mouse-specific antibodies, which highlight tumor and immune components of the mouse TME. We performed imaging using two new features of Hyperion™ XTi. Ultrafast preview mode (PM) was applied to rapidly screen entire brain sections for marker expression signatures associated with various immuno-oncology processes. This enabled biomarker-guided selection of areas in tumor tissue that were imaged using region of interest-based IMC and analyzed using single-cell analysis (SCA). In parallel, a high-throughput tissue mode (TM) was applied to perform a detailed whole-slide scan of normal and GBM mouse brain tissues that were quantified using pixel-based analysis (PBA) to unravel the composition of the TME.

**Results and Discussions**

Using TM, we were able to successfully visualize the entire coronal and sagittal sections of normal mouse brain as well as mouse GBM tissue. PBA on TM data provided quantitative spatial expression patterns of structural and immune markers across the whole tissue. In normal tissue, we visualized the highly organized structure of the normal brain and detected neurons, oligodendrocytes, vascular-adjacent astrocytes, and axonal tracks. In GBM tissue, necrotic cores, areas with high immune infiltration, extracellular matrix deposits, and activated tumor cells were detected. SCA of GBM tissue demonstrated expansive vascularization, replicating Olig2+ cells, activation of Ras signaling, and high abundance of infiltrating immune cells.

**Conclusion**

Overall, we demonstrate the successful application of two novel WSI modes and highlight the power of IMC technology to simultaneously explore dozens of relevant biological outputs to better understand the TME of GBM and other tumors.

### EACR2024-1260
### Novel Whole Slide Imaging Modes for Imaging Mass Cytometry Unveil Extensive Cellular Heterogeneity in Human Gliomas

*F. Spada[1], H Koppejan[1], N. Zabinyakov[1], Q Raza[1], T.D. Pfister[1], N. Parsotam[1], D. Howell[1], L. Lim[1], C. Loh[1]*
*[1]Standard BioTools Canada Inc., Markham, ON, Canada*

**Introduction**

Gliomas present a complex form of cancer that is challenging to diagnose and treat, with a median survival of just over one year after diagnosis for primary glioblastoma (GBM). GBM can occur as multi-lesion, remote, or diffuse tumors, and often contains a tumor microenvironment (TME) that is devoid of peripheral immune cells. Additional hallmark features of GBM include necrosis, hemorrhage, and pseudo palisades, making it a highly heterogeneous disease requiring further investigation. Identification of the cellular and spatial level composition of the TME is vital for interpretation of GBM disease origin, progression, prediction, and treatment.

**Material and Methods**

A 40-plus-marker neuro-oncology Imaging Mass Cytometry™ (IMC™) antibody panel was used to determine the cellular and structural landscape of the brain TME. We applied the panel on a tissue microarray (TMA) containing dozens of human glioma cores and uncovered the spatial distribution of over 40 distinct molecular markers. We performed imaging using two new features of the Hyperion XTi™ Imaging System that provide whole slide scanning capabilities. Ultrafast preview mode was applied to rapidly screen tumor cores for expression signatures associated with tumor immuno-oncology processes. This enabled biomarker-guided selection of areas in tumor tissue that were imaged at higher resolution and analyzed using single-cell analysis. In parallel, a high-throughput tissue mode was applied to perform a detailed scan of the brain tumor TMA followed by pixel-based analysis to unravel the spatial composition of the TME.

**Results and Discussions**

Using tissue mode imaging, we successfully mapped the spatial location of cell populations making up human gliomas, such as neurons, astrocytes, microglia, and oligodendrocytes, across the entire TMA. Various tumor cell phenotypes, resident and infiltrating cells, and resting and activated microglia were detected across all TMA cores. Subsequent single-cell analysis of select regions of interest provided a quantitative assessment of the cellular composition of the brain TME. We classified the distinct states of neurons and quantified myeloid and lymphoid immune cell infiltration across normal, astrocytoma, and GBM tissues. Striking cellular and protein heterogeneity was observed between acquired cores, indicating the complexity of each case that is impossible to capture with low-plex visualization techniques. Therefore, IMC is a highly relevant tool capable of quantitative spatial evaluation of the high-plex protein composition in the brain TME without the complications of auto-fluorescence, tissue degradation, and spectral overlap.

**Conclusion**

Empowered by the neuro-oncology panel and new whole slide imaging modes, IMC accelerates neurological research and provides insights into the spatial complexity of gliomas and other tumors.

### Tumour Evolution and Heterogeneity

### EACR2024-0066
### Survival and inferential analysis in patients with locally advanced breast cancer treated with neoadjuvant chemotherapy and subsequent sentinel lymph node selective biopsy

*J.M. Espejo Niño[1]*
*[1]Hospital Universitario Cruces, Nuclear Medicine, Laredo, Spain*

**Introduction**

Sentinel lymph node biopsy(SLNB) allows an exactly axillar staging in patients with early disease, but not in locally advance breast cancer(LABC). Our aim was to

© 2024 The Authors. Molecular Oncology is published by John Wiley & Sons Ltd on behalf of Federation of European Biochemical Societies study, the feasibility and accuracy of the SLNB technique with and without lymphadenectomy(LDN) and with axillar clipping after neoadjuvant chemotherapy(NAC), in patients with LABC.

**Material and Methods**

Patients with LABC, scheduled for NAC, surgery and SLNB. Subsequently the patients were scheduled for adjuvant chemotherapy/hormonotherapy and radiotherapy according with the postsurgical results. Main end points were overall survival (OS) disease-free survival (DFS), mortality, SLNB identification rate (IR), sensitivity, false negative rate (FNR) of SLNB versus LDN, negative predictive value (NPV) and overall accuracy.

**Results and Discussions**

SLNB IR was between 89.8% and 100%. 55% of the not migrations were ypT1 post-NAC, 44% were ypN0 and 44,4% were minor partial response in MRI results (p-value 0,017; <0,001 and 0,020 respectively). Overall FNR was 15,7%. In patients with LDN+SLNB the FNR increased to 25%, but, with axillar clipping+SLNB+LDN and with axillar clipping+SLNB alone the FNR was reduced to 0%. 36,2% of the negative LDN were SLNB(+), which means that the sentinel node was the only positive node in these patients, and our technique effectively could change the nodal staging even if the LDN is negative. There were 15 patients (51,7%) in whom the clip was not found, that means more than a half. Concordance between the MRI and pathological results in the complete response was 65,6%. Nodal response to NAC was 51,19%(cN+ to ypN0). OS was between 89% to 97%. DFS was between 89,8% to 96,8%. Sensitivity was between 75% to 100%. NPV was between 89,6% to 100%. Mortality was 7.01%. The survival was lower, (p-value <0.05), for patients with triple negative and LuminalB/HER2 intrinsic subtype; with poor nodal response(ypN2-3); with progression or mayor partial response in MRI results and in patients with BRCA1/2 mutation.

**Conclusion**

Our study presents excellent results of SLNB alone in patients with LABC with complete nodal response with an OS and DFS >95%. The FNR is high in partial responders, so we cannot recommend the SLNB alone in LABC. We recommend, in cN+ patients, axillar clipping+SLNB+LDN because in more than 50% of the patients with axillar clipping, this was not found, and because in 36% of the patients with negative LDN, the SLNB obtained the only positive node, so these techniques together improve the node staging, OS and DFS.

### EACR2024-0639

### Impact of time-induced tissue dynamics on phenotype and drug efficacy

*E. Blondeel[1], S. Dedeyne[1], P. Rappu[2], J. Heino[2], T. Boterberg[3], J. Vandesompele[4], P. Mestdagh[4], O. De Wever[1]*

*[1]Laboratory of Experimental Cancer Research, Ghent University, Department of Human Structure and Repair, Ghent, Belgium*

*[2]MediCity Research Laboratory, University of Turku, Department of Life Technologies, Turku, Finland*

*[3]Ghent University Hospital, Department of Radiation Oncology, Ghent, Belgium*

*[4]OncoRNALab- Ghent University, Department of Biomolecular Medicine, Ghent, Belgium*

**Introduction**

Spheroids are three-dimensional (3D) cellular models which mimic tissue architecture. Spheroids show widespread application across academic and industrial research groups since they provide more physiologically relevant and predictive data to overcome the correlation mismatch between the preclinical and clinical situation. Recently, our research group has shown that four experimental parameters; cell line, culture medium, formation method, and size, have a striking impact on a diverse set of spheroid metrics (MISpheroID, Nature Methods, 2021). However, a limitation of this study was the fixed predefined timepoint to evaluate these spheroid metrics. Time-induced tissue dynamics may impact spheroid biology and consequently spheroid application.

**Material and Methods**

Differently aged spheroids of multiple established, as well as early passage patient-derived, cancer cells were evaluated by transcriptomics, proteomics, lipidomics, secretome analysis and real-time imaging combined with morphometrics. Impact of spheroid age on spheroid application was investigated by the radial migration assay and therapy evaluations.

**Results and Discussions**

Time-dependent tissue dynamics induce differently expressed RNA and proteins over time. A mitotic signature is strongly enriched at early timepoints while cellular response to hypoxia, extracellular structure organization, receptor signalling pathways, migration, induction of angiogenesis, and monocarboxylate and lipid metabolism are enriched at later timepoints. Spheroid heterogeneity at different spheroid ages was further confirmed based on quantification of the different lipid classes by lipidomics. Both triglycerides and cholesterol esters, the main components of lipid droplets, are more present in older spheroids. The impact of spheroid age on spheroid phenotype has consequences on therapy efficacy. Chemotherapeutics, radiotherapy and inhibitors of oxidative phosphorylation showed a lower effect rate in older spheroids compared to junior ones, while PI3K/mTOR, MAPK and tyrosine kinase inhibitors induced a higher effect rate at a later spheroid age. Furthermore, aged spheroids have a higher migratory capacity.

**Conclusion**

To conclude, spheroid age has an impact on spheroid biology and will have to be considered in drug efficacy evaluations.

### EACR2024-0785

### Synthetic cis-regulatory DNA for genetic tracing of cell identities and state changes in Glioblastoma

*M.J. Schmitt[1], C. Company[2], Y. Dramaretska[2], M. Serresi[2], B. Jiang[2], S. Kertalli[2], M. Grossmann[2], A. Aguzzi[3], I. Barozzi[4], G. Gargiulo[2]*

*[1]Max-Delbrueck-Center, Gargiulo Lab, Berlin, Germany*

*[2]Max-Delbrueck-Center for Molecular Medicine, Molecular Oncology, Berlin, Germany*

Molecular Oncology 18 (Suppl. 1) (2024) 1–495

© 2024 The Authors. Molecular Oncology is published by John Wiley & Sons Ltd on behalf of Federation of European Biochemical Societies

[3]University Hospital Zurich, Institute of Neuropathology, Zurich, Switzerland
[4]Medical University of Vienna, Center for Cancer Research, Vienna, Austria

**Introduction**

Glioblastoma (GBM) remains the most challenging primary solid tumor of the central nervous system despite extensive research on its molecular and cellular properties. Aggressive treatments exist, including surgical resection, radiation, and chemotherapy, but inevitable tumor recurrence and treatment resistance persist due to genetic and cellular heterogeneity, overlaid by phenotypic plasticity.

**Material and Methods**

To improve understanding of GBM heterogeneity, we developed advanced genetic tracing techniques to decipher subtype-specific transcriptional states and intrinsic/non-cell autonomous factors guiding cell fate commitment. Additionally, addressing the demand for sophisticated transcriptional reporters, enabling experimental manipulation and characterization of diseased and developmental cell states, we introduced a computational framework for out method - Logical Design of Synthetic cis-regulatory DNA (LSD). Leveraging phenotypic biomarkers and regulatory networks as input, LSD designs synthetic locus control regions (sLCRs) marking cellular states and pathways. This innovative framework yields flexible transcriptional reporters applicable to diverse biological systems.

**Results and Discussions**

We demonstrated mesenchymal GBM adaptation through partially overlapping transcriptional responses involving external signaling and ionizing radiation. Cell fate commitment to a mesenchymal state proved adaptive, reversible, and associated with increased chemotherapy resistance due to crosstalk between innate immune cells and glioma-initiating cells. In genome-scale CRISPRa screens, sLCRs unveiled both known and novel mesenchymal-GBM cell-state drivers, demonstrating broad applicability for studying complex cell states and transcriptional regulation. Our flexible reporters, functional in mouse and human tissues without minimal promoters, have short synthetic DNA cassettes that can be seamlessly integrated into AAV vectors for gene therapy. Additionally, we expanded our synthetic transcriptional reporters to trace epithelial cell responses during SARS-CoV-2 infection, revealing activation driven by interferon-α/β/γ and NF-κB pathways. Drug screens identified JAK inhibitors and DNA damage inducers as potential modulators of epithelial cell responses to SARS-CoV-2 infection.

**Conclusion**

In summary, our work advances understanding of GBM heterogeneity and introduces a versatile methodology applicable across diverse research fields, from developmental biology to infectious diseases.

### EACR2024-0832
### Impact of Phosphatidylserine Externalization on Breast Cancer Cell Fusion

M. Sieler[1], T. Dittmar[1]
[1]Witten/Herdecke University, Institute of Immunology, Department of Medicine, Center for Biomedical Education and Research ZBAF, Witten, Germany

**Introduction**

Cell fusion is a physiological process crucial for tissue development and homeostasis, but is also known to take place in the pathophysiological context of cancer. A hybrid cell resulting from a fusion of a cancer and a normal cell can exhibit new malignant properties, like a high metastatic potential or drug resistance, which arise from the high heterogeneity of those hybrid cells. In addition to fusogenic proteins, the distribution of the membrane lipid phosphatidylserine (PS) is known to play a role in cell fusion. How the fusion of cancer cells with other cells is regulated and why the fuse at all has not yet been clarified, but is important to find out because cell fusion is a potential therapeutic target in the fight against cancer.

**Material and Methods**

To detect cell fusion events, the dual split protein (DSP) assay was established and cocultures of M13SV1 breast epithelial cells overexpressing the fusogenic protein Syncytin-1 (M13_Syn1) with different breast cancer cell lines were analyzed due to their fusion rate. The quantification of externalized PS of M13SV1 wild type cells, M13_Syn1 cells and the used breast cancer cell lines was assessed with a FITC-labeled Annexin V probe. To study the impact of PS on cell fusion, further experiments are planned by knocking out the scramblase ANO6, masking PS in cell fusion assays and over-expression of the flippase ATP8A1.

**Results and Discussions**

The coculture of M13_Syn1 cells and different breast cancer cell lines showed high cell fusion rates in comparison to cocultures with M13SV1 cells. When analyzing the amount of externalized PS on the different cells, differences were detected. In order to study the impact of PS on cancer cell fusion, an ANO6 KO was induced in M13SV1 cells and two breast cancer cell lines and need to be further evaluated. To flip PS to the inside of the cells, an overexpression of the flippase ATP8A1 will be done in the cell lines and cell fusion assays will be repeated. This will show whether the PS externalization plays a role in breast cancer cell fusion.

**Conclusion**

The experiments already done show that Syncytin-1 not only is capable to induce cell fusion between breast epithelial cells and breast cancer cells, but that it may also have an impact on the externalization of PS. Whether this is just an artifact or does also play a role in cancer cell fusion and therefore would be a potential therapeutic target or marker for cancer cells ready to fuse, will be experimentally investigated in these studies.

### EACR2024-0833
### Single-cell transcriptomic analysis of laryngeal squamous cell carcinoma exhibits heterogeneous cell states and gene expression programs in the tumor ecosystem

M. Scrima[1], F. Melisi[1,2], T. Peluso[1], E. Ciervo[3,4], A. De Falco[3,4], M. Sunilrao Tathode[2], M. Bocchetti[1,2],

© 2024 The Authors. Molecular Oncology is published by John Wiley & Sons Ltd on behalf of Federation of European Biochemical Societies

*A.M. Cossu[1,2], M. Ceccarelli[3,4,5], M. Caraglia[1,2]*
*[1]Biogem scarl- Institute of Genetic Research, Laboratory of Molecular Oncology and Precision Medicine, Ariano Irpino, Italy*
*[2]University of Campania "Luigi Vanvitelli", Department of Precision Medicine, Naples, Italy*
*[3]Biogem scarl, Institute of Genetic Research, Bioinformatics Lab, Ariano Irpino, Italy*
*[4]University of Naples "Federico II", Department of Electrical Engineering and Information Technology, Naples, Italy*
*[5]University of Miami, Miller School of Medicine, Sylvester Comprehensive Cancer Center, Miami, United States*

**Introduction**
Laryngeal squamous cell carcinoma (LSCC) is a highly malignant tumor originating from the respiratory system. Despite representing only about 1% of total cancers and related deaths, it is one of the most common types of head and neck cancers. Early diagnosis and prevention of metastasis are pivotal for LSCC treatment. We aimed to profile the landscape of cellular diversity in LSCC tumors using single-cell RNA sequencing (scRNA-seq), obtaining novel information on the heterogeneity of cell types and states in the tumor ecosystem.

**Material and Methods**
Ten freshly collected, treatment-naïve LSCC biopsies were dissociated by enzymatic digestion. Cell suspension with ≥75% viable cells was then processed for single cell 3' gene expression analysis (scRNA-seq). Single CEll Variational ANeuploidy analysis (SCEVAN) algorithm was used to identify malignant cells and decipher the clonal substructure of tumors. Unsupervised clustering and marker gene expression were used to nominate non-malignant cell types. The non-negative matrix factorization (NMF) approach uncovered shared cellular programs among tumor samples. Cell-cell interactions between immune cells and cells from specific cellular programs were uncovered using multiple methods such as cell-chat and scTHI.

**Results and Discussions**
A total of 76,230 cells were collected, 27,000 (35.4 %) were malignant, and 49,230 (63.6 %) were non-malignant. LSCC samples were characterized by a high level of infiltration of stromal and immune cells, with a median purity of 28.6%, minimum purity of 17.1%, and max purity of 69.9 %. Non-malignant cell composition was almost homogeneous among patients, with 65.86 % lymphoid cells, 11.92 % myeloid cells, 11.65 % of fibroblasts, 7.04 % of epithelial cells, and 3.53 % of endothelial cells. NMF of the malignant cells revealed six consensus meta-programs. Some of them have been previously observed, and some new meta-programs, such as the immune, were uncovered with our analysis. This meta-program was characterized by activating inflammatory cytokines and specific cell-cell interactions within the tumor microenvironment. Association with clinical and pathological characteristics of the patients was performed.

**Conclusion**
Single-cell sequencing approach gave us the possibility to elucidate the heterogeneity of malignant cells in LSCC and new meta-programs that involve immune response activation. The latter evidenced new mechanisms of escape of LSCC from the immune system disclosing new potential exploitable immune-sensitizing targets.

### EACR2024-0912
### Treatment resistance in the glioblastoma ecosystem: exploring the interplay between tumor cells and the microenvironment with advanced patient avatars

*M. de Lucas Sanz[1,2], P.M. Moreno Sanchez[1,2], Y. Yabo[1,2], R. Mahsa[1,2], A. Poli[1], D. Brown[3], S.P. Niclou[1,2], A. Michelucci[1], A. Golebiewska[1]*
*[1]Luxembourg Institute of Health, Department of Cancer Research, Luxembourg, Luxembourg*
*[2]University of Luxembourg, Faculty of Science, Technology and Medicine, Belvaux, Luxembourg*
*[3]Kintara Therapeutics, Kintara Therapeutics, San Diego, United States*

**Introduction**
Glioblastoma (GBM) is the most aggressive adult brain tumor with poor prognosis despite aggressive treatment. While GBM cells present strong phenotypic plasticity and resistance mechanisms, the balance between intrinsic and adaptive resistance mechanisms remains unclear. GBM cells are further supported by immune cells, creating a highly immunosuppressive niche. Advanced models recapitulating the complex tumor ecosystem are required to assess clinically-relevant therapy responses.

**Material and Methods**
We applied a cohort of well-characterized patient-derived models, propagated as tumor organoids and orthotopic xenografts (PDOXs) to investigate GBM treatment responses to chemotherapeutic agents (temozolomide, VAL-083). Phenotypic adaptation of GBM cells and mouse-derived tumor microenvironment (TME) was assessed in vivo with MRI, single-cell RNA-seq and multiplex IHC. Functional responses to treatment in time were further studied ex-vivo in GBM stem-like cultures and organoids with cell viability assays, genetic tracing, live cell imaging and multicolor flow cytometry. To further investigate the GBM and tumor micro-environment cross-talk, we established an advanced protocol for co-culture of tumor organoids with immune cells isolated from blood and normal brain.

**Results and Discussions**
We show that advanced organoid co-cultures and PDOXs recreate clinically-relevant TME. Both models recapitulate the immunosuppressive TME allowing for the study of the GBM-immune cell crosstalk upon treatment. The chemotherapeutic treatment in PDOXs leads to an increase in the influx of microglia-derived macrophages with enhanced gene expression signatures associated with phagocytosis and antigen-presentation, suggesting a supportive role in tumor resistance mechanisms. Such changes occurred simultaneously with decreased tumor growth in vivo and activation of apoptosis and DNA repair mechanisms in GBM cells. Ex-vivo, GBM cells have heterogeneous responses in time at different doses. Treatment responses in time currently are being assessed in patient-derived models co-culture systems.

**Conclusion**

© 2024 The Authors. Molecular Oncology is published by John Wiley & Sons Ltd on behalf of Federation of European Biochemical Societies

Advanced patient-derived models, including PDOXs and organoid-based co-cultures, hold great potential to study the clinically-relevant adaptation of the GBM ecosystem upon treatment in time. Our study suggests a crucial role of phagocytosis mediated by microglia in supporting tumor resistance mechanisms. Further functional studies are needed to assess the treatment responses in time to unravel the molecular mechanisms or resistance.

### EACR2024-0920
### Investigating interactions between colorectal cancer subclones and stromal fibroblasts

Y. Zhou[1], R. Owen[2], X. Lu[2], S. Buczacki[1]
[1]*University of Oxford, Nuffield Department of Surgical Sciences, Oxford, United Kingdom*
[2]*University of Oxford, Oxford Ludwig Institute, Oxford, United Kingdom*

**Introduction**
Communication between epithelial and their neighbouring stromal cells is emerging as a key event required for the successful establishment of early colorectal cancer, but studies dissecting the precise interactions between these cellular and non-cellular components is lacking. Here, we have developed a novel in vitro primary human organoid co-culture system consisting of fluorescently labelled, gene-edited human colon organoids with primary human colonic fibroblasts that, for the first time, enables the precise dissection of these types of communication.

**Material and Methods**
Human colon organoids and paired primary human colonic fibroblasts derived from normal colon samples were fluorescently labelled. Driver genes involved in colorectal cancer were targeted in organoids using CRISPR-Cas9 system. Wild type or mutant organoids were then co-cultured with fibroblasts in basement membrane extract, phenotypes were observed, and cells subsequently extracted for downstream analyses.

**Results and Discussions**
Wild type adult human colon organoids when co-cultured with primary fibroblasts induced a profound change in organoid morphology generating larger organoids than observed in mono-culture. Compared to mono-culture controls, single-cell RNA sequencing (scRNAseq) of co-cultured WT organoids showed enrichment in a recently described regenerative colorectal stem cell (revCSC) population, which was driven by the activation of YAP-TAZ signalling pathway. Proteomic analyses of the extracellular matrix showed enrichment of several basement membrane components involved in epithelial cell-ECM interactions in co-culture, suggesting potential roles of ECM remodelling in driving the regenerative program in epithelial cells. Next, we targeted *APC* or *TP53* from WT colon organoids to model the earliest event in sporadic colorectal cancer or colitis-associated cancer. Phenotypically, both mutant organoids retained the growth advantage seen when co-cultured with fibroblasts. scRNAseq showed that, instead of the expansion of the revCSC population, a separate stem cell population, characterised by highly proliferative activity, was enriched in co-cultures for both genotypes.

**Conclusion**
Our data demonstrates that profound interactions exist between epithelial and fibroblast components that affect cell identity and behaviour including modulation of the surrounding extra-cellular matrix. Driver mutations in epithelial cells alter this two-way interaction and further insights may provide putative therapeutic targets to prevent tumour initiation.

### EACR2024-1011
### Time-Resolved Proteomics: Mapping Dynamic Cell-Cell Interactions in Colorectal Cancer

V. Mohan[1], O. Atrakchi[2], S. Piwko[2], I. Takala[2], M. Zerbib[3], R. Oren[3], T.M. Salame[4], E. Kopitman[4], B. Boursi[5], T. Geiger[6]
[1]*Weizmann Insitute of Science, Molecular Cell Biology, Rehovot, Israel*
[2]*Weizmann Institute of Science, Molecular Cell Biology, Rehovot, Israel*
[3]*Weizmann Institute of Science, Veterinary Resources, Rehovot, Israel*
[4]*Weizmann Institute of Science, Life Sciences Core Facilities, Rehovot, Israel*
[5]*Sheba Medical Center, Oncology, Tel HaShomer, Israel*
[6]*Weizmann Institute of Science, Molecular Biology, Rehovot, Israel*

**Introduction**
Colorectal cancer (CRC) is the third most deadly and fourth most commonly diagnosed cancer in the world. Metastatic colorectal cancer (mCRC) is associated with poor outcomes, with a 5-year survival rate of less than 10% and a median survival time of approximately 5 months for untreated patients. mCRC commonly spreads to the liver, facilitated by its proximity, and the lungs, often signifying advanced stages and influencing treatment decisions. Current research focuses on providing global characteristics of primary and metastatic tumors in mCRC but falls short in detailing the spatial (metastatic organ) distribution of proteins and how pathways and cell-cell communications evolve over time from early to late stages of metastasis.

**Material and Methods**
In our study we used both an injected murine metastasis model and matched human metastatic and primary tumor tissue. The MC38 colon cancer cells were injected into C57BL/6 mice tail vein for lung metastasis and portal vein for liver metastasis, alongside studying primary tumors in the colon, injected using colonoscopy. Using CyTOF, we characterized the immune and stromal landscapes within our murine tumors. We then isolated 200 cells from 14 distinct populations (both tumor and control) for in-depth proteomic analysis using Evosep-TIMSTOF SCP LC-MS setup. Additionally, we performed deep proteomic analysis on the matched human primary and metastatic tumors obtained by macrodissection of archived FFPE tissues.

**Results and Discussions**
Our CyTOF analysis revealed dynamic changes in immune and stromal cell populations within MC38 tumors, with a notable rise in pro-tumorigenic myeloid cells. We further mapped specific activation states of various immune cells, highlighting the crucial role of resident versus infiltrating macrophages. Proteomics on

© 2024 The Authors. Molecular Oncology is published by John Wiley & Sons Ltd on behalf of Federation of European Biochemical Societies sorted cell populations identified potential cell-cell interaction mechanisms between the cancer and immune cells.

**Conclusion**

Our findings suggest complex interplay between immune, stromal and tumor cells in tumor progression, with potential therapeutic targets identified through proteomics analysis and cell-cell interaction mapping. Further investigation into these interactions in human patients holds promise for improved immunotherapy based metastatic colorectal cancer treatment strategies.

### EACR2024-1100
### Developing predictive and prognostic biosensor tool based on electromagnetic signatures depending on lung cancer hallmarks; a new challenge in personalized medicine

*H. Daverat[1], N. Blasco[2], S. Robert[1], C. Dalmay[2], A. Pothier[2], F. Lalloué[1], K. Durand[1], T. Naves[1]*
*[1]OmegaHealth Institute, UMR Inserm-CHU 1308 CAPTuR, Limoges, France*
*[2]XLIM, UMR CNRS n°7252, Limoges, France*

**Introduction**

Emergence of novel cell populations propagate a heterogeneous tumour comprising thereby different subpopulations with varying degrees of cancer hallmarks. Because therapy in lung cancer remain based on traditional classification schemes, the most clinical needs remain to identify and evaluate novel biomarkers associated with the difficulty to target heterogeneous tumour and adapt appropriate-of-care therapy. We identified sortilin expression as a promising prognostic biomarker depicting a subgroup of lung adenocarcinoma with better overall survival. Likewise, in the quest of personalised medicine, tumour mutation burden (TMB) is emerging as a promising indicator of immunotherapy sensitivity remaining however limit by financial barriers. Overcoming these challenges requires the development of tools characterizing biopsy-derived cells. In face with this dilemma, we develop a biosensor tool measuring physical properties of cancer cells by providing electromagnetic signatures (EMS) linked to intracellular dielectric parameters. We aimed to determine how cancer cells EMS change according to cellular phenotype and molecular characteristics.

**Material and Methods**

To achieve these goals, several cell models were used: lung cancer cell models with inducible sortilin expression using a Tet-ON system (NIC-H1975, A549, PC9), a panel of cell lines with variable TMB levels, and synthetic cell models with increased TMB levels (NCI-H1975, U87-MG) by treatment with a mutagenic agent (N-ethyl-N-nitrosourea). Real-time cell imaging (Incucyte®) was employed to perform functional proliferation and invasion assays on Tet-ON sortilin models. Transcriptomic analyses were performed on Tet-ON models using RNA-seq to determine the specific molecular signature associated with sortilin expression. Next-generation sequencing was used to accurately assess TMB value.

**Results and Discussions**

The Tet-ON sortilin cell lines showed reduced proliferation and increased invasion. Transcriptome analyses have already identified pathways of interest involved in cancer. Using the biosensor, we have so far observed a significant correlation between the TMB of cell lines and their EMS, with EMS increasing proportionally to the TMB index.

**Conclusion**

Our comprehensive approach has so far demonstrated that TMB heterogeneity can be detected by our physical system and sortilin expression detection will be undertaken shortly. To consolidate these results, we are developing primary lung adenocarcinoma cultures to validate our findings in clinically relevant models.

### EACR2024-1197
### ONECUT2 is a driver of luminal to basal plasticity and a potential drug target in breast cancer

*I. Zamora[1], M. Gutiérrez[1], A. Pascual[1], M.J. Pajares[1], M. Barajas[1], S. You[2], B. Knudsen[3], M. Freeman[2], I. Encío[1], M. Rotinen[1]*
*[1]Public University of Navarre, Department of Health Sciences, Pamplona, Spain*
*[2]Cedars-Sinai Medical Center, Departments of Urology and Biomedical Sciences, Los Angeles, United States*
*[3]University of Utah, Department of Pathology, Salt Lake City, United States*

**Introduction**

The dynamic nature of breast cancer (BC) is a major challenge in the clinical management of patients. Cellular plasticity, characterized by the acquisition of independence from an oncogenic driver such as the estrogen receptor (ER), contributes to therapeutic failure, tumor progression, and metastatic dissemination. The transcription factor ONECUT2 (OC2) has been shown (Rotinen et al. Nature Medicine 2018) to be a master regulator of metastatic castration- resistant prostate cancer (mCRPC) tumors, where it suppresses the androgen axis and promotes a drug-resistant neuro-endocrine (NEPC) phenotype. Here, we investigate the role of OC2 in mediating the conversion between different molecular subtypes within BC.

**Material and Methods**

Clinical outcome assessments, immunohistochemistry, forced expression, shRNA, RNA-seq, western-blot, RT-qPCR, functional and computational analysis, in vivo experiments.

**Results and Discussions**

Our Kaplan-Meier analysis demonstrates that high expression of OC2 mRNA correlates with worse recurrence-free survival (RFS) and overall survival (OS). Quantitative assessment of OC2 immunostaining using a BC TMA shows that elevated OC2 levels are associated with lymph node metastasis and advanced clinical stages. Gene expression profiling of OC2-engineered luminal BC cell lines has allowed us to generate a OC2 activity signature that reveals a negative correlation with ER activation pathways and a positive correlation with basal signatures. We find that OC2 is a negative regulator of ER expression and a repressor of its transcriptional program and promotes luminal to basal plasticity in BC cells. We further demonstrate that OC2 is required for

© 2024 The Authors. Molecular Oncology is published by John Wiley & Sons Ltd on behalf of Federation of European Biochemical Societies cell survival in BC models and can be targeted with a drug-like small molecule inhibitor providing a novel therapeutic strategy for patients with OC2 active tumors. Notably, OC2 mRNA expression is significantly higher in BC specimens from patients who exhibited recurrence compared to those who remained disease-free and the combination of CSRM617 and tamoxifen displays a synergistic cytotoxic effect in luminal BC cell lines.

**Conclusion**

The transcription factor OC2 is a driver of BC heterogeneity and a potential drug target in distinct cell states within breast tumors.

### Tumour Immunology

### EACR2024-0025
### Galactose expression on monomeric and dimeric forms of IgA paraprotein from serum of patients with monoclonal gammopathy

*D. Kosanovic[1], T. Djukic[2], B. Ristic[1], I. Maslovaric[1], M. Kovacic[1], S. Mojsilovic[1], I. Drvenica[1], O. Savic[3], D. Markovic[1], V. Ilic[1]*
*[1]Institute for Medical Research, University of Belgrade, Group of Immunology, Belgrade, Serbia*
*[2]Innovation Center of the Faculty of Technology and Metallurgy, Department of Pharmaceutical Engineering, Belgrade, Serbia*
*[3]Institute for Blood Transfusion of Serbia, Department for Laboratory Diagnostic, Belgrade, Serbia*

**Introduction**

IgA monoclonal gammopathies can include various disorders, such as IgA myeloma, monoclonal gammopathy of undetermined significance (MGUS) with IgA component, and IgA-related disorders. IgA myeloma accounts only 20% myeloma cases. However, IgA myeloma is prognostically unfavourable, and often accompanied by kidney, bone, and blood diseases. Some of monoclonal IgA complications are related to their structural characteristics. Thus, monoclonal IgA might be highly polymerized causing hyperviscosity syndrome (mucosal bleeding, visual and neurological abnormalities). Monoclonal IgA express O- and N-linked glycans, but their glycans can be different from those expressed on polyclonal IgA of healthy people. In addition, it was reported that loss of Gal residue on the glycans of the IgA hinge region results in the IgA deposits and nephropathy.

**Material and Methods**

To determine the level of polymerization and expression of galactose on monoclonal IgA, these molecules were isolated by affinity chromatography on Protein M agarose from sera of patients with monoclonal gammopathies. The isolated proteins were initially analyzed using SDS PAGE. Expression of galactose on IgA was assessed by lectin blot with *Ricinus communis* lectin I (RCA I), a D-galactose-binding lectin.

**Results and Discussions**

The results of SDS PAGE revealed multiple protein fractions, with the most intensely coloured fractions presumed to represent IgA monomers and dimers. Western blot analysis confirmed the presence of IgA monomers and dimers, along with presence of incomplete IgA molecules (intermediary of IgA synthesis or degraded IgA molecules), in all samples. The results of RCA I blot demonstrated varying patterns of galactose expression, in some isolates galactose was expressed on both monomeric and dimeric IgA, while in others, galactose is expressed only on monomeric forms or in IgA dimer forms with weak expression on its monomeric form.

**Conclusion**

In summary, results showed that IgA in analyzed monoclonal gammopathies sera did not have the same structural characteristics. It will be possible to conclude if their structure influences the disease progression only after correlating structural characteristics with the patients' clinical data.

### EACR2024-0107
### The tumor-associated microbiome fuels human colorectal cancer through iNKT cell-driven immune evasion

*F. Facciotti[1], G. Lattanzi[2], A. Diaz-Basabe[3], F. Perillo[3], A. Baeri[4], F. Caprioli[2], F. Strati[5]*
*[1]IEO- European Institute of Oncology, Dept of Experimental Oncology, Milan, Italy*
*[2]Fondazione IRCCS Cà Granda- Ospedale Maggiore Policlinico, Gastroenterology and Endoscopy Unit, Milan, Italy*
*[3]IEO, Experimental oncology, Milan, Italy*
*[4]University of Milano Bicocca, Biotechnology and Biosciences, Milan, Italy*
*[5]University of Milano Bicocca, Dept of Biotechnology and Biosciences, Milan, Italy*

**Introduction**

iNKT cells account for a relevant fraction of effector T-cells in the intestine and are considered an attractive platform for cancer immunotherapy. Human iNKT cells possess cytotoxic properties against colorectal cancer (CRC) cells through the perforin–granzyme pathway, but their role in CRC progression is still controversial. Gut microbiota- iNKT cells interaction takes part in CRC pathophysiology, but the impact of CRC-associated pathogens such as Fusobacterium nucleatum and Porphyromonas gingivalis on iNKT cell functions remains unexplored. Thus, we addressed the contribution of iNKT cells to CRC pathophysiology and the effect of the tumor-associated microbiota in shaping their pro- or anti- tumorigenic functions.

**Material and Methods**

We examined the immune cell composition and iNKT cell phenotype of paired non-tumor vs cancerous tissue lesions in CRC patients (n=118,Policlinico Hospital, Milan, Jan 2017-July 2022).High-dimensional single-cell flow cytometry, metagenomics, RNAseq, ex vivo and in vitro experiments were performed to evaluate the phenotype and function of human iNKT cells. Mechanisms were dissected in chemically-induced or orthotopically implanted (subcutaneous or intracaecal) CRC murine models, either iNKT proficient or deficient. RFS analyses were performed in our cohort of patients and results were validated in the colon adenocarcinoma

© 2024 The Authors. Molecular Oncology is published by John Wiley & Sons Ltd on behalf of Federation of European Biochemical Societies cohort (COAD) of The Cancer Genome Atlas (TCGA) database.

**Results and Discussions**

The immunophenotypic profiles of human and murine iNKT cells confirmed that IFNγ-producing, cytotoxic iNKT cells limit colonic tumorigenesis whereas intra-tumor accumulation of GM-CSF+IL17+iNKT cells support colon cancer progression through recruitment of tumor-associated neutrophils (TANs) correlating with negative clinical outcomes.The tumor-associated P. gingivalis and F. nucleatum induced a protumour phenotype in iNKT cells. Moreover, P. gingivalis reduces the cytotoxic functions of iNKT cells, hampering their ilytic machinery through increased expression of chitinase 3-like-1 protein (CHI3L1) in vitro and in vivo. In vivo reactivation with αGalCer or neutralization of CHI3L1 effectively restored iNKT cell cytotoxicity.

**Conclusion**

Our results reveal that tumor-infiltrating iNKT cells can contribute to the remodeling of the TME by recruiting TANs and sculpting the CRC developmental trajectory. Our findings uncover new mechanisms of iNKT-TAN antitumor immunity suppression in CRC, supporting the targeted manipulation of iNKT cells' function to improve cancer immunotherapies.

### EACR2024-0122
### Pharmacologic inhibition of nonsense-mediated decay induces anti-tumour immunogenicity in ex vivo patient tumours

*R. Vendramin*[1], *H. Fu*[1], *Y. Zhao*[1], *S. Fernandez Patel*[1], *D. Qian*[1], *S. Quezada*[2], *J.L. Reading*[2], *Y. Samuels*[3], *C. Swanton*[4], *K. Litchfield*[1]

[1]*UCL Cancer Institute, Oncology, London, UK*

[2]*UCL Cancer Institute, Haematology, London, UK*

[3]*Weizmann Institute of Science, Molecular Cell Biology, Rehovot, Israel*

[4]*The Francis Crick Institute, Oncology, London, UK*

**Introduction**

Immunogenic neoantigens derived from somatic tumour mutations are essential to initiate and sustain anti-tumour immune responses. Frameshift insertion/deletions are a key source of neoantigens. Frameshifted transcripts often contain premature termination codons and are degraded by the nonsense-mediated mRNA decay (NMD) pathway. In cancer, NMD activity is increased in response to the heightened burden of somatic fs-indel mutations and effectively prevents the generation of immunogenic tumour-specific neoantigens.

**Material and Methods**

We pharmacologically targeted SMG1, a core NMD component, for the first-time in patient tumour and normal samples as well as in a panel of human cancer cell lines and patient-derived organoids. We analysed the effects of SMG1 inhibition (SMG1i) through RNA sequencing (RNA-seq), proteomics, HLA-peptidomics, tumour-T cell co-cultures and patient-derived tumour fragments. Ex vivo immunological responses were assessed by high-dimensional flow cytometry, cytometric bead array and single-cell RNA- and TCR-sequencing.

**Results and Discussions**

Leveraging exome, transcriptome, and checkpoint inhibition response data from over 1,000 patients we identify an association between genetic loss or lower expression of the NMD mediator SMG1 with improved responses to immunotherapy. Pharmacological targeting of SMG1 in patient-derived tumour fragments results in activation and expansion of tumour-reactive $CD8^+$ lymphocytes. Mechanistically, SMG1 inhibition increases the abundance of frameshifted transcripts and their HLA presentation, converting the neoepitope count from a low- to a high-TMB-like state without inducing mutations. Co-culture of $CD8^+$ lymphocytes with patient-derived tumour organoids or tumour cells upon SMG1 inhibition induces NMD- and antigen-dependent T cell activation and tumour cell killing.

**Conclusion**

We show a means to generate tumour neoantigens without inducing genomic DNA changes, by instead enhancing the cellular abundance of RNA transcripts encoding frameshifted mutant peptide sequences. Our findings, in a clinically relevant platform, highlight SMG1 inhibition as a novel immune-oncology approach to exploit an untapped source of highly immunogenic peptides. By targeting a pathway shared by all tumours, it offers a viable avenue for enhancing anti-tumour immunogenicity with broad applicability, particularly in scenarios with a low TMB (~ 90% of all cancers), with translational potential warranting future clinical evaluation.

### EACR2024-0128
### Expression Profile of CD47 and Impact of its Blockade on Osteosarcoma Cells

*Y. KO*[1], *J.A. Lee*[1]

[1]*National Cancer Center, Department of Pediatrics, Goyang-si, South Korea*

**Introduction**

Macrophages account for the majority of immune cells infiltrated in sarcoma tissues. CD47 is expressed on many cancer cells and acts as a macrophage immune checkpoint by interacting with SIRPα of the macrophage surface. In this study, we evaluated the feasibility of CD47 as a potential candidate for immunotherapy of osteosarcoma, exploring its expression patterns, clinicopathological correlations, and immunotherapeutic implications.

**Material and Methods**

A retrospective analysis was performed on 24 patients with osteosarcoma, and biopsy samples obtained from them were used to evaluate significant correlations between CD47 staining positivity and clinicopathologic characteristics. The expression of CD47 protein and the impact of CD47 antibodies on proliferation and apoptosis were evaluated using osteosarcoma cells. Additionally, the efficacy of CD47 antibodies on phagocytic activity in osteosarcoma cells was measured through co-culture by generating monocyte-derived macrophages obtained from healthy volunteers.

**Results and Discussions**

CD47 protein expression was identified in 20.8% of the analyzed samples, and a significant correlation was observed between positive CD47 staining and metastasis at the time of diagnosis (p=0.04). Additionally, patients

© 2024 The Authors. Molecular Oncology is published by John Wiley & Sons Ltd on behalf of Federation of European Biochemical Societies with CD47-positive tumors tended to be older than those with CD47-negative tumors (16.5±6.2 years vs. 15.7±3.0 years, p=0.07). However, no significant associations were found between CD47 protein expression and sex, tumor size, or histologic response to preoperative chemotherapy. CD47 protein expression was detected in all analyzed osteosarcoma cells, however no significant effects of CD47 antibodies on proliferation and apoptosis in these cells. In contrast, the phagocytic activity of differentiated macrophages on KHOS/NP osteosarcoma cells was significantly higher in cells pretreated with CD47 antibody, compared to the isotype control antibody.

**Conclusion**

The association of CD47 protein expression and clinical characteristics in biopsy tissues suggests the possibility of CD47 as a new immunotherapeutic target of osteosarcoma. The phagocytic activity enhanced by the CD47 antibody observed on osteosarcoma cells showed the feasibility of CD47 as a potential candidate for immunotherapy of osteosarcoma. Although future studies are needed, these findings contribute valuable groundwork for a better understanding of CD47's role in osteosarcoma and an innovative immunotherapy approach against this formidable malignancy.

### EACR2024-0183
### Melatonin Suppresses Pancreatic Adenocarcinoma by Regulation of Tumour-associated Neutrophil Infiltration and Neutrophil Extracellular Trap Formation

*Y.T. Chan*[1], *Y. Feng*[1], *N. Wang*[1]
[1]*The University of Hong Kong, LKS Faculty of Medicine, Hong Kong, Hong Kong-China*

**Introduction**

The tumor microenvironment significantly impacts the prognosis of patients with pancreatic adenocarcinoma (PAAD). Proper regulation within this environment could potentially enhance survival outcomes. In this study, we investigate the role of melatonin, an endogenous hormone with diverse bioactivities, in PAAD.

**Material and Methods**

Melatonin expression and clinical significance of induced neutrophil response were evaluated by in-house human PAAD tissues. The animal model was used to validate the anti-tumour effect of melatonin and investigate the neutrophil response. In vitro experiments and proteomics analysis were performed to look into the mechanism.

**Results and Discussions**

Our study demonstrates that pancreatic melatonin levels correlate with patient survival. In PAAD mouse models, melatonin supplementation inhibits tumor growth, while blocking the melatonin pathway exacerbates tumor progression. Interestingly, this anti-tumor effect is independent of cytotoxicity but closely tied to tumor-associated neutrophils (TANs). Depletion of TANs reverses melatonin's effects. Melatonin induces TAN infiltration and activation, leading to apoptosis of PAAD cells. Cytokine analysis reveals that melatonin primarily impacts tumor cells' secretion of Cxcl2. Knockdown of Cxcl2 in tumor cells hinders neutrophil migration and activation. Melatonin-activated neutrophils exhibit an N1-like anti-tumor phenotype, with increased neutrophil extracellular traps (NETs) causing tumor cell apoptosis. Proteomics analysis suggests that this reactive oxygen species (ROS)-mediated inhibition is fueled by fatty acid oxidation (FAO) in neutrophils. Notably, the FAO inhibitor abolishes the anti-tumor effect. Furthermore, CXCL2 expression in PAAD patient specimens correlates with neutrophil infiltration. Combining CXCL2 or TANs with NET markers provides better prognostic predictions.

**Conclusion**

In summary, our findings reveal an anti-tumor mechanism of melatonin involving the recruitment of N1-neutrophils and beneficial NET formation.

### EACR2024-0201
### Therapeutic impact and immune modulation by MYC inhibition in KRAS-driven NSCLC with diverse mutational landscapes

*Í. González-Larreategui*[1], *L. Vera*[2], *F. Giuntini*[1], *S. Martínez-Martín*[3], *H. Thabussot*[3], *I. Macaya*[2], *M.E. Beaulieu*[3], *S. Vicent*[2,4,5], *S. Casacuberta-Serra*[3], *L. Soucek*[1,3,6,7]
[1]*VHIO, Models of Cancer Therapies Group, Barcelona, Spain*
[2]*CIMA, Program in Solid Tumors and Biomarkers, Pamplona, Spain*
[3]*Peptomyc S.L., Peptomyc S.L., Barcelona, Spain*
[4]*IdiSNA, Navarra Institute for Health Research, Pamplona, Spain*
[5]*CIBERONC, Centro de Investigación Biomédica en Red de Cáncer, Madrid, Spain*
[6]*ICREA, Institució Catalana de Recerca i Estudis Avançats, Barcelona, Spain*
[7]*UAB, Universitat Autònoma de Barcelona, Barcelona, Spain*

**Introduction**

KRAS is the most frequent mutated oncogene in Non-Small-Cell Lung Cancer (NSCLC), linked to poor prognosis and tumor recurrence. Co-mutations in Tumor Suppressor Genes (TSGs), mainly TRP53, KEAP1 and STK11 (LKB1), influence treatment response of KRAS-mutated cancers. MYC, a key transcription factor downstream of KRAS, not only promotes tumor progression and treatment resistance, but also orchestrates tumor immune evasion. Although MYC was long considered undruggable, our lab pioneered the use of Omomyc as the first clinically viable MYC inhibitor. Here, we aim to determine how different TSGs mutations impact the response of KRAS-mutant tumors to MYC inhibition, as well as their effect on the modulation of the tumor immune microenvironment.

**Material and Methods**

We used mutant KRAS Lung Adenocarcinoma (KLA) isogenic cell lines CRISPR-edited to knockout TRP53, STK11 or KEAP1 genes. MYC levels were determined by western blot, and response to Omomyc was assessed by proliferation, metabolic and transcriptomic assays. For in vivo studies, cells were injected subcutaneously

© 2024 The Authors. Molecular Oncology is published by John Wiley & Sons Ltd on behalf of Federation of European Biochemical Societies into C57BL/6x129/Sv F1 mice and immune populations were characterized by flow cytometry.

**Results and Discussions**

MYC levels were increased in the TSG-edited cells compared to the parental ones, but Omomyc reduced in vitro cell growth, changed the cell cycle profile and modulated tumor immune microenvironment-related gene sets across all KLA cell lines. In mice, systemic MYC inhibition displayed different therapeutic efficacy in the different mutational profiles, and the effect of MYC inhibition on remodeling the tumor immune microenvironment varied depending on the specific TSG co-mutation. Importantly, Omomyc enhanced the therapeutic efficacy of different immunotherapies.

**Conclusion**

Prevalent co-occurring mutations in TSGs in KRAS-driven NSCLC influence the therapeutic effect of MYC inhibition by Omomyc treatment and modulate tumor immune microenvironment. Interestingly, a stronger Omomyc efficacy correlates to an enhanced anti-tumor immune response, positioning it as a potential partner for immuno-oncology treatments.

### EACR2024-0202

### An vascularized melanoma model for with unidirectional perfusion of circulating immune cells for Advancing Immune-Oncology Research

*T. Burton[1], S. de Ruiter[2], K. Queiroz[2], J. Komen[3], T. Thakkar[4], B. Trietsch[5], L. van den Broek[2]*
*[1]Mimetas B.V, Techcore, oegstgeest, The Netherlands*
*[2]Mimetas B.V, Model Development, oegstgeest, The Netherlands*
*[3]Mimetas B.V, Business Development, oegstgeest, The Netherlands*
*[4]Mimetas B.V, Commercial, oegstgeest, The Netherlands*
*[5]Mimetas B.V, Management, oegstgeest, The Netherlands*

**Introduction**

Understanding the interactions between the tumor the vascular systems and circulating immune cells is crucial for elucidating mechanisms of immune cell recruitment in cancer and in exploring potential immunotherapeutic approaches. However, current in vitro models often lack the fidelity to replicate these intricate dynamics observed in vivo. The OrganoPlate Graft has been developed for perfusion of peripheral blood mononuclear cells (PBMCs) through a vascular bed with a melanoma monolayer (A375) to study aspects such as immune cell adhesion, extravasation and interaction with the tumor.

**Material and Methods**

The OrganoPlate Graft comprises 48 chips per plate, each designed to simulate the conditions of vascular beds. The device achieves flow through passive leveling, utilizing gravity to induce a pressure drop and unidirectional flow across the vascular bed in both positive and negative inclines. This approach avoids the complications and potential artifacts introduced by external pump, reduces complexity, and increases the throughput with up to 16 plates (768 chips) served by each OrganoFlow rocker.

**Results and Discussions**

In experimental settings, formation of the vascular bed was seen to be consistent in plates and the introduction of the chemokine CXCL-12 led to the establishment of a gradient. Subsequently, PBMCs were observed to localize in clusters as a result of extravasation towards this gradient and the A375 monolayer. This behavior is consistent with known chemotactic responses in more complex in vivo environments. A notable observation was the sustained activity of PBMCs within the system. Cells continued to perfuse through the vascular bed for up to three days after seeding.

**Conclusion**

In summary, the OrganoPlate Graft provides a platform for studying immune interactions in an immuno-competent vascular bed model. Its design and functionality allow for the exploration of immune cell behaviors in proximity to tumor cells, offering potential insights for immune-oncology research. The system's ability to mimic immune cell migration and extravasation in a controlled environment, and future potential to observe tumour killing makes it a valuable tool for advancing our understanding of immune-tumor interactions and may contribute to the development of novel immune-oncology therapeutic strategies.

### EACR2024-0205

### Tumor neoantigen recognition by circulating and tumor-infiltrating T lymphocytes in esophageal adenocarcinoma

*F. Di Mauro[1,2], C. Palombo[1], M. Punta[3,4], G. Lopatriello[1,3], L. Albarello[5], C. Cattaneo[6], R. Rosati[2,7], G. Casorati[1], P. Dellabona[1], G. Arbore[1,2]*
*[1]IRCCS San Raffaele Scientific Institute, Experimental Immunology Unit, Milan, Italy*
*[2]Vita-Salute San Raffaele University, Vita-Salute San Raffaele University, Milan, Italy*
*[3]IRCCS San Raffaele Scientific Institute, Center for OMICS Sciences, Milan, Italy*
*[4]IRCCS San Raffaele Scientific Institute, Haematology and Bone Marrow Transplant Unit, Milan, Italy*
*[5]San Raffaele Hospital, Department of Pathology, Milan, Italy*
*[6]IRCCS San Raffaele Scientific Institute, Experimental Hematology Unit, Milan, Italy*
*[7]San Raffaele Hospital, Department of Gastrointestinal Surgery, Milan, Italy*

**Introduction**

Locally advanced esophageal adenocarcinoma (EAC) is a highly aggressive tumor treated with neoadjuvant chemotherapy (nCT), followed by surgery. Less than 30% of patients achieve a pathological complete response to nCT, which correlates with increased 5-year overall survival. Our recent multidimensional analysis of pre-treatment tumor biopsies highlighted a correlation between pre-existing immunity and clinical response to nCT, suggesting that T cells specific for tumor antigens, , may participate in tumor control. Because EAC is highly mutated, we sought to compare T cell responses against tumor neoantigens (TNAs) in responders versus non-responders to nCT.

**Material and Methods**

We are investigating TNA-specific T cell responses on EAC patients' blood, tumor-infiltrating lymphocytes

Molecular Oncology 18 (Suppl. 1) (2024) 1–495
© 2024 The Authors. Molecular Oncology is published by John Wiley & Sons Ltd on behalf of Federation of European Biochemical Societies

(TILs), and resected tumor-draining lymph nodes (LNs) using complementary approaches. TNAs, derived from missense mutations, in frame insertions/deletions and gene fusions, are identified by computational analysis of tumor whole exome and RNA sequencing obtained from pre-treatment tumor biopsy. Patient B lymphocytes are immortalized and either pulsed with synthetic TNA peptides or transduced with TNAs-encoding minigenes. PBMCs are pre-stimulated with synthetic peptides corresponding to identified TNAs, while TILs and LN-derived T cells are polyclonally expanded in vitro. These patient-derived T cell lines are further restimulated with autologous APCs and then assessed for TNA specificity by coculture with either peptide-pulsed or minigene-transduced APCs. T cell activation is verified by flow cytometry determination of CD137 upregulation and IFNg secretion.

**Results and Discussions**

Preliminary results with PBMCs from EAC patients showed recognition of autologous TNAs by either CD8+ or CD4+ circulating T cells suggesting a potential immunogenicity of one or more of the tested TNAs.

**Conclusion**

The enrichment of tumor-reactive T lymphocytes revealed specific recognition of tumor neoantigen, with the possibility to perform TCR sequencing to assess T cell clonality.In parallel we are also pursuing the HANSolo unsupervised screening method to comprehensively assess individual patient T cell reactivities against the entire mutanome expressed by autologous tumor in responder vs non-responder patients to nCT.

### EACR2024-0250
### Combined RASG12C(ON) and SHP2 inhibition promotes an immunogenic tumour microenvironment in lung cancer

*P. Anastasiou[1], C. Moore[1], S. Rana[1], A. de Castro[1], M. Tomaschko[1], J. Boumelha[1], E. Mugarza[1], M. Molina-Arcas[1], J. Downward[1]*
*[1]Francis Crick Institute, Downward lab, London, UK*

**Introduction**

The approval of KRASG12C-mutant specific inhibitors (G12Ci) has enabled treatment of lung cancer patients harbouring KRASG12C mutations. However, responses are short-lived highlighting the need for combination therapies. Given the crucial role of oncogenic KRAS in constraining antitumour immune responses, immune checkpoint blockade (ICB) presents an obvious option for combining with G12Ci to enhance antitumour responses and potentially extend tumour suppression. However, recent data suggests that this combination would be effective only in inflamed tumours, underlining the need for development of combinations therapies for immune excluded tumours.

**Material and Methods**

In this study we use KRASG12C-mutant transplantable models of non-small cell lung cancer (NSCLC) with varying immunogenicities to investigate antitumour responses using a novel, covalent tri-complex RASG12C(ON) inhibitor, RMC-4998, in combination with SHP2 inhibitor RMC-4550. We employ qPCR, Flow Cytometry and RNA sequencing to study the effect of this combination on the tumour microenvironment (TME).

**Results and Discussions**

KRAS inhibition was followed by MAPK reactivation, which could be suppressed through combination with SHP2 inhibitor RMC-4550 leading to elevated cancer cell apoptosis. Combined inhibition of KRAS and SHP2 led to remodelling of the TME, suppression of tumour relapse, and immune memory development which resulted in lymphocyte-dependent durable responses in an inflamed mouse model of NSCLC. Although combination of KRAS and SHP2 inhibition did not result in durable responses in an immune excluded mouse model of NSCLC, it sensitised these tumours to ICB. Of note, SHP2 inhibition induced a pan-lymphocytic infiltration independent of cancer cell intrinsic KRAS signalling, demonstrating the importance of SHP2 in remodelling the TME in a cancer cell extrinsic manner. KRAS and SHP2 combined inhibition led to exclusion of immunosuppressive innate immune cells, expression of antigen presenting and phagocytosis markers, accompanied by activation of lymphocytes expressing tumouricidal cytokines and proteases.

**Conclusion**

Our preclinical data demonstrate that combination of RASG12C and SHP2 inhibitors can remodel the TME eliciting a pan-lymphocytic anti-tumour immune response with a potential benefit in tumours that are ICB resistant.

### EACR2024-0297
### Heterotypic CD8 T cell clusters isolated from clinical samples are distinct and enriched for antitumor activity

*S. Ibanez Molero[1], J. Veldman[1], J.H.T. Joleen[2], A. George[1], H. Kelly[1], S.M. Pack[1], L. Tas[1], J. B A G Haanen[1], W. J van Houdt[3], D. Peeper[1]*
*[1]Netherlands Cancer Institute, Division of Molecular Oncology and Immunology, Amsterdam, The Netherlands*
*[2]Netherlands Cancer Institute, Division of Tumor biology and Immunology, Amsterdam, The Netherlands*
*[3]Netherlands Cancer Institute, Department of Medical Oncology, Amsterdam, The Netherlands*

**Introduction**

Functional tumor-T cell interactions are dynamic and complex, underlying both cancer progression and therapy response. Emerging evidence suggests a correlation between $CD8^+$ T cell-tumor cell proximity and immunotherapy response. Indeed, structural and functional avidity of cytotoxic $CD8^+$ T cells correlates strongly with their activity against cancer cells. This led us to investigate whether tumor-specific $CD8^+$ cells can be isolated from clinical cancer specimens as interacting cells in heterotypic clusters, and what is their therapeutic potential.

**Material and Methods**

To evaluate functional interactions between human T cells and tumor cells, we employed: (1) in vitro co-cultures; (2) patient samples and (3) ex vivo assays. Interacting (cluster) and not-interacting (singlets) T cells were isolated and characterized by single-cell RNA and TCR sequencing, as well as by functional ex vivo cytotoxicity assays with autologous tumor cells.

© 2024 The Authors. Molecular Oncology is published by John Wiley & Sons Ltd on behalf of Federation of European Biochemical Societies

**Results and Discussions**
We found that in defined co-cultures, antigen-specific T cells were commonly enriched over non-specific T cells in heterotypic clusters with tumor cells, prompting us to investigate whether such clusters could be isolated also from clinical samples. From almost all human melanoma metastases analyzed, we were able to isolate heterotypic clusters, comprising $CD8^+$ T cells interacting with tumor cells and/or antigen-presenting cells (APCs), which was validated by imaging flow cytometry. Upon rapid expansion, $CD8^+$ T cells from tumor cell and/or APC clusters exhibited enhanced cytokine production and increased melanoma-killing activity (average 7.6-fold) over T cell singlets. Using single cell sequencing, we observed that $CD8^+$ T cells from clusters were enriched for tumor-reactive and exhausted gene signatures. Integration with TCR-sequencing showed increased clonality of clustered T cells, indicative of expansion upon antigen recognition.
**Conclusion**
Together, these results demonstrate that tumor-reactive $CD8^+$ T cells are enriched in functional clusters with tumor cells and/or APCs, and that they can be isolated and expanded from clinical samples. Being often excluded in cell sorting procedures, these distinct heterotypic $CD8^+$ T cell clusters serve as a valuable source amenable to deciphering functional tumor-immune cell interactions, while they may also be therapeutically explored.

### EACR2024-0298
### Investigating the role of the immune system in MLL-AF9 infant leukaemia

*H. Vaughan*[1], *E. Doyle*[1], *S. Mariani*[1]
[1]*University of Edinburgh, Centre for Inflammation Research, Edinburgh, United Kingdom*

**Introduction**
Leukaemia is the most common cancer in infants under the age of 4, with 75% of infant leukaemia cases characterized by rearrangement of the Mixed Lineage Leukaemia (MLL) gene. Current treatment strategies, including chemotherapy and stem cell transplant, come with substantial risks due to the vulnerability of the infant host as well as limited efficacy due to chemoresistance. In adult *MLL-AF9* leukaemia, research shows a population of immune cells termed leukaemia associated macrophage's (LAMs) support chemoresistance by adopting a protective phenotype over the leukemic stem cells (LSCs), but there is an absence of research investigating this in infancy. We propose characterising these LAMs in infant *MLL-AF9* leukaemia could uncover therapeutic targets for immunotherapy.
**Material and Methods**
This research uses the double transgenic (DT) inducible *MLL-AF9* mouse model under control of the cKit promotor. Transcriptomic analysis of LAMs in DT embryos was conducted using single cell RNA sequencing (scRNA-seq). Immunofluorescent staining with cKit, CD68 and F480 was employed to visualise interactions between LAMs and cKit+ cells. Co-culture experiments used isolated and sorted DT/WT macrophages (CD45+CD11b+F480high/low) with DT/WT Lin-Sca-1+cKit+ (LSK) cells followed by colony forming unit (CFU) assays to assess progenitor potential. Flow cytometry was used to assess protein expression in the bone marrow and foetal liver of WT and DT mice.
**Results and Discussions**
Preliminary results show changes to the immune cell compartment pre and post birth in DT mice. On top of changes to the myeloid cell compartment, immunofluorescence performed on E13.5 DT foetal liver show interactions between cKit+ cells and F480+/ CD68+ macrophages. Additionally, co-culture experiments show macrophages from WT mice decrease progenitor potential of DT LSK cells while macrophages from DT mice do not have the same ability. These findings in collaboration with scRNA-seq results showing transcriptional changes to *MLL-AF9*- myeloid cells in the presence of *MLL-AF9*+ LSCs indicate macrophages undergo a level of alteration when exposed to an *MLL-AF9* leukemic environment.
**Conclusion**
Early data supports the hypothesis that embryonic macrophages transition into LAMs during *MLL-AF9*+ infant leukaemia, potentially promoting malignancy and chemoresistance. By understanding the role of LAMs and thus identifying potential therapeutic targets, this study hopes to identify novel immunotherapy strategies to enhance the treatment of human infant MLL-AF9 leukaemia.

### EACR2024-0299
### Microbial metabolic pathways guide response to immune checkpoint blockade through the activation of unconventional T cells

*I. Mimpen*[1,2], *T.W. Battaglia*[1,2,3], *B.S. Geurts*[1,2], *L.J. Zeverijn*[1,2], *L.F.A. Wessels*[2,3,4], *E.E. Voest*[1,2]
[1]*The Netherlands Cancer Institute, Molecular Oncology & Immunology, Amsterdam, The Netherlands*
[2]*The Netherlands Cancer Institute, Oncode Institute, Amsterdam, The Netherlands*
[3]*The Netherlands Cancer Institute, Molecular Carcinogenesis, Amsterdam, The Netherlands*
[4]*Delft University of Technology, Faculty of EEMCS, Delft, The Netherlands*

**Introduction**
The gut microbiome plays a crucial role in maintaining human health. Recent studies have now also identified a modulating effect of the microbiome on the efficacy of immune checkpoint blockade (ICB), showing an association between specific microbes and clinical responses. However, these microbial species lack consistency across studies and mechanisms underlying the immunomodulatory effect remain elusive. We therefore hypothesized that shared metabolic pathways across microbial species better identify a common predictive denominator to mediate response to ICB.
**Material and Methods**
To investigate the functional role of the microbiome in response to ICB, we generated a curated resource of metagenomic data, containing 816 samples, and conducted a prospective study with DNA mismatch repair-deficient (dMMR) metastatic cancers with matched genomics, transcriptomics and clinical outcome

Molecular Oncology 18 (Suppl. 1) (2024) 1–495
© 2024 The Authors. Molecular Oncology is published by John Wiley & Sons Ltd on behalf of Federation of European Biochemical Societies data. We then validated these findings using several in vitro, patient-derived tumor organoid-based, co-culture models.

**Results and Discussions**

Using our large multi-study cohort, we identified multiple microbial metabolic pathways that significantly associated to ICB response. This included the methylerythritol 4-phosphate (MEP) pathway, with intermediate (E)-4-hydroxy-3-methyl-but-2-enyl pyrophosphate (HMBPP) as a known activator of the gamma-delta (γδ) T cell subset Vγ9Vδ2, which was shown to be significantly increased in ICB responders. To further show the relevance of this pathway, we demonstrated that supernatant from bacteria contributing to this pathway as well as purified HMBPP dramatically enhanced Vδ2 T cell-mediated anti-tumor activity directed against patient-derived tumor organoids, in a BTN3A1-dependent-manner, while healthy organoids remained unaffected.

**Conclusion**

Taken together, our results suggest that focusing on certain microbial pathways across bacterial species and their metabolites may be more relevant in predicting immune modulation of ICB than selecting individual microbial species. Our findings highlight a metabolite-centered approach to identify novel modes of microbiome-immune crosstalk.

### EACR2024-0307

### LIF promotes immunosuppression through C1q+ TAMs in GBM

M. López[1], J. Seoane[1]

*[1]Vall d'Hebron Institute of Oncology VHIO, Gene Expression and Cancer, Barcelona, Spain*

**Introduction**

Glioblastoma (GBM) is the most common brain cancer in adults with unmet therapeutic needs. Leukemia-inhibitory factor (LIF), a pleiotropic cytokine expressed in GBM, has been shown to act as an oncogenic factor and to promote immunosuppression in the tumor micro-environment. Recently, a population of tumor-associated macrophages (TAMs) characterized by the expression of C1q has been described. Although poorly understood, these C1q+ TAMs correlate with poor prognosis and immunosuppression.

**Material and Methods**

We studied the effect of LIF on bone-marrow derived macrophages (BMDMs) and GBM animal models. We then analyzed the immunosuppressive effect of C1q on T-cells, both murine and human. Finally, we used a novel patient-derived platform, the patient-derived tumor tissue cultures (PDTTCs), co-cultured with T-cells, to assess the effect of LIF blockade in the tumor micro-environment on T-cell activation.

**Results and Discussions**

LIF induced the expression of C1q both by RNA and protein in BMDMs. Neutralization of LIF in GBM animal models led to a decrease in the expression of C1q in CD11b+ cells and tumor growth. Moreover, C1q repressed the activation of both murine and human CD8+ and CD4+ T-cells. Blockade of LIF in PDTTCs showed an increased activation of co-cultured T-cells.

**Conclusion**

Our results indicate that LIF promotes an immuno-suppressive C1Q+ TAMs population, that is involved in T-cell disfunction. Blockade of LIF through neutralizing antibodies may contribute to decrease this population and improve immunotherapies in GBM.

### EACR2024-0321

### Uncoupling of MYC-driven metabolic dependencies in PDAC induces immune-dependent remission of tumors

B. Krenz[1], A. Gebhardt-Wolf[1], T. Kannan[1], G. Mattavelli[2], M. John[2], W. Schmitz[1], A. Riedel[2], M. Eilers[1]

*[1]Julius-Maximilians-Universität of Würzburg, Chair of Biochemistry and Molecular Biology, Würzburg, Germany*

*[2]University Hospital Würzburg, Mildred Scheel Early Career Center, Würzburg, Germany*

**Introduction**

Deregulated expression of the MYC oncoprotein is nearly universal in human cancers and is a well described driver of immune evasion in tumors. Accordingly, genetic depletion of MYC or its co-factors in an orthotopic transplant model of PDAC has been shown to cause immune-mediated regression of tumors (Krenz et al., 2021, Gaballa et al., 2024). MYC targeting systemically, however, would also affect proliferating tissues, including distinct cytotoxic immune cell populations which are essential to facilitate regression of tumors. To obviate this problem, we aim to restore antitumor immunity by transplanting allograft immune cells that are rendered resistant against the said systemic treatment. With this approach we thereby uncouple the effects of a chemotherapy and the viability of immune cells that are essential to mediate the regression of tumors.

**Material and Methods**

As a proof-of-concept, we have used cardiac glycosides, that have been described to deplete the levels of MYC proteins in human but are ineffective in mice. To this end, we orthotopically transplanted an engineered cardiac glycoside-sensitive murine PDAC cell line into immuno-compromised NRG and into immunocompetent BL6/J mice and analyzed tumor growth, overall survival, and changes in the tumor microenvironment using flow cytometry, single-cell sequencing and mass spectrometry.

**Results and Discussions**

Administration of cardiac glycosides caused only a marginal delay in tumor growth and no regression in NRG mice. Strikingly, treating tumors in syngeneic BL6/J mice with cardiac glycosides led to complete remission of tumors within one week. Untreated tumors displayed a nutrient-poor tumor microenvironment with suboptimal conditions for the expansion and activity of anti-tumor immunity. Treatment with cardiac glycosides targets the uptake of nutrients from the micro-environment, restores amino acid availability in the interstitial fluid and at the same time impedes the translation of endogenous MYC by limiting intracellular glutamine and nucleotide levels. Consequently, PI3K-AKT-MTOR pathway is activated in cytotoxic T and NK cells, allowing for the rapid expansion of this population and immune-mediated killing of tumor cells within days.

**Conclusion**

© 2024 The Authors. Molecular Oncology is published by John Wiley & Sons Ltd on behalf of Federation of European Biochemical Societies

Collectively, we propose an approach to uncouple the targeting of the tumor cell from the immune system. This offers a solution for the impaired hematopoiesis and compromised immune system, typically presented in chemotherapy-treated patients.

### EACR2024-0357
### Targeting tumour-promoting inflammation in breast cancer by blocking oncostatin m cytokine signalling

*P. Azcoaga[1], S. Manzano[1], A. Abaurrea[1], P. Petit[1], Z. Telletxea[1], I. Garmendia[1], M.M. Caffarel[1]*
*[1]Biogipuzkoa Health Research Institute, Oncology, San Sebastian, Spain*

**Introduction**
Chronic inflammation is a canonical cancer hallmark and a major driver for metastasis. Understanding how inflammatory signals orchestrate pro-malignant effects within the tumour microenvironment is key to block tumour-promoting inflammation. Cytokines are the main regulators of inflammation. We recently characterized the cytokine Oncostatin M (OSM), member of the IL-6 family, as a as central node for multicellular interactions within the breast tumour microenvironment. We observed that myeloid-derived OSM activates an intriguing pro-tumoral signalling in cancer-associated fibroblasts (CAFs) and cancer cells, leading to increased macrophage recruitment (Araujo et al., JCI 2022). Our new unpublished data prove that OSM signalling may contribute to cancer progression by remodelling the myeloid cell compartment and promoting immune suppression.

**Material and Methods**
We generated mouse MMTV-PyMT breast tumours lacking the OSM receptor OSMR. The effect of OSM in the immune landscape and myeloid compartment of those tumours was analysed by flow cytometry and scRNAseq. Our results were complemented with in vitro co-cultures of breast cancer cells, CAFs and macrophages. The clinical relevance of the results was assessed by staining of a TMA comprising 141 human BC samples and bioinformatic analysis of human breast tumors from TCGA.

**Results and Discussions**
Our data supports that OSM activates IL1 and IL8 secretion and promotes immune suppression by remodelling the myeloid compartment. In particular, OSM activates the expression of genes involved in neutrophil activation and myeloid derived suppressor cell (MDSC) signatures, promotes protumoural M2-like macrophage recruitment, and inhibits myeloid phagocytosis through up-regulation of the “do-not-eat me” signals (CD47-SIRPA) and down-regulation of the “do-eat-me” signal SLAMF7. Depletion of OSMR signalling in a genetic breast cancer model results in increased levels of cytotoxic CD8 and CD4 T cells and B lymphocytes. High levels of OSM and its receptor OSMR correlate with IL1, IL6 and IL8 levels, and associate with increased myeloid recruitment and decreased T and B cell infiltration in human breast cancer samples. In addition, OSM activates, in cancer cells and CAFs, signatures related to hypoxia and lactate secretion, all features strongly associated with immune suppression.

**Conclusion**
Our work sheds light on the effects of OSM signalling in immune suppression and supports that the OSM pathway could be a promising candidate for therapeutic targeting in breast cancer.

### EACR2024-0358
### Identification and characterization of tumor-reactive CD8 and CD4 T cells in hepatocellular carcinoma

*L. Magré[1], Y.S. Rakké[2], R.S. van Gemerden[1], M. Doukas[3], J.N.M. IJzermans[2], J. Kwekkeboom[1], S.I. Buschow[1], D. Sprengers[1]*
*[1]Erasmus MC, Gastroenterology and Hepatology, Rotterdam, The Netherlands*
*[2]Erasmus MC, Surgery, Rotterdam, The Netherlands*
*[3]Erasmus MC, Pathology, Rotterdam, The Netherlands*

**Introduction**
Effective anti-tumor responses rely on the presence of tumor-reactive T cells in the tumor microenvironment (TME). Understanding the characteristics of these tumor-reactive T cells might reveal potential approaches for targeted immunotherapy. Most studies have been focusing on the identification of tumor-reactive CD8 T cells only, revealing CD39 as a potential marker for tumor reactivity in several tumor types. Recently, it has become clear that also a specific subset of CD4 T cells is capable of recognizing tumor antigens and may contribute to tumor clearance. In hepatocellular carcinoma (HCC) neither CD39 as a marker for tumor-reactive T cells nor the presence of tumor reactive CD4+ T cells has been studied. In this study we aimed to identify and phenotype CD39+ tumor reactive CD8 T cells in HCC and to study the presence and characteristics of tumor-reactive CD4 T cells.

**Material and Methods**
We used flow cytometry to phenotype tumor-infiltrating lymphocytes (TIL), peripheral blood mononuclear cells (PBMC) and tumor free lymphocytes (TFL) derived from HCC patients. Cancer germline antigen (CGA) and cytomegalovirus (CMV) peptide-loaded tetramers were used to identify tumor-reactive CD8 T cells and bystander T cells respectively. Using flow cytometry data Lineage tracing was performed to identify cell subsets with high phenotypic resemblance.

**Results and Discussions**
Using flow cytometry we identified a population of CD8 T cells positive for CGA peptide-loaded tetramers. Analysis revealed that these specific CD8 T cells, in contrast to CMV peptide-loaded bystander T cells, have an enhanced activation status defined by increased 4-1BB expression as well as upregulation of CD39 and CD103. This specific population of tumor-reactive T cells was unique to the TME as it was absent in PBMC and tumor adjacent tissue. Using lineage tracing we were able to identify a possible tumor-reactive CD4 subset that phenotypically resembled the tumor-reactive CD8 T cell population.

**Conclusion**
To conclude, (I) we identified a unique HCC CD8 T cell population specific for tumor antigens characterized by CD39 and CD103 expression. (II) we identified a specific HCC CD4 T cell population with phenotypic

Molecular Oncology 18 (Suppl. 1) (2024) 1–495
© 2024 The Authors. Molecular Oncology is published by John Wiley & Sons Ltd on behalf of Federation of European Biochemical Societies resemblance to tumor-reactive CD8 T cells. Future functional analysis should determine the tumor-clearing capacity of these CD4 T cells.

### EACR2024-0413
### Identification of Potential Novel Immune Checkpoints using Immunocompromised Mouse Models

*H. Linder[1], N.K. Czajkowski[1], A.Z. Johansen[1], C.Ø. Madsen[1], D.H. Madsen[1,2]*
*[1]National Center for Cancer Immune Therapy, Department of Oncology, Copenhagen University Hospital, Herlev and Gentofte, Herlev, Denmark*
*[2]Faculty of Health and Medical Sciences, University of Copenhagen, Department of Immunology and Microbiology, Copenhagen, Denmark*

**Introduction**
Cancer immunoediting explains the emergence of detectable tumors despite immunosurveillance, with malignant cells evading the immune system through diverse immunosuppressive mechanisms. Although immune checkpoint inhibitors have enhanced cancer treatment, low objective response rates persist. Identifying new immunosuppressive molecules is important to improve our understanding of immune escape and to develop novel immunotherapies.
**Material and Methods**
The immunogenic cancer cell lines (MC38 and CT26) were inoculated into C57BL/6 Ragn2-/- and BALB/c$^{nu/nu}$ mice, or their respective wildtypes (WT). Bulk tumor or FACS-sorted cancer cells underwent bulk RNA sequencing to identify differentially expressed genes (DEGs). Candidate genes were selected from DEGs using publicly available datasets. Candidates were knocked out in MC38 cells using CRISPR/Cas9. Chrome$^{51}$ release assay was used to assess CD8+ T cell-mediated killing of WT and knockout (KO) cell lines. Effects of candidate gene KO on tumor growth were evaluated in vivo by inoculating WT and KO cell lines into C57BL/6 mice. The tumor microenvironment (TME) composition of these tumors was analyzed using multicolor flow cytometry.
**Results and Discussions**
MC38 and CT26 cells formed faster-growing tumors in T cell-deficient mice. Across both models, 25 genes were upregulated in WT compared to T cell-deficient mice, including genes related to antigen-processing and presentation, and known immunosuppressive molecules. Pursuing DEGs with minimal functional annotations identified three candidate genes expressed in various human cancers. Candidate gene expression was upregulated in MC38 and CT26 upon IFN-y stimulation. MC38 cells deficient in candidate genes exhibited slower tumor growth in vivo, and the TME composition of KO tumors significantly differed from WT tumors. One candidate gene KO increased susceptibility to CD8+ T cell-mediated killing in vitro.
**Conclusion**
Three potential novel immune checkpoints upregulated in response to T cell effector mechanisms, particularly IFN-y, were identified. Deficiency in these molecules delayed tumor growth in vivo. In vitro data suggests that one candidate molecule directly inhibited CD8+ T cell cytotoxicity. Targeting the proteins encoded by these genes pharmacologically may lead to the development of innovative cancer immunotherapies.

### EACR2024-0430
### Modeling T cell-mediated tumor killing in oral cancer using organoid technology

*L. Szabó[1], N. Harnischfeger[1], L. Wilde[1], M. Krafft[1], V. Boschert[2], M. Schleyer[2], V. Buck[3], A. Kübler[2], S. Hartmann[2], K. Kretzschmar[1]*
*[1]University Hospital Würzburg, Mildred Scheel Nachwuchszentrum MSNZ, Würzburg, Germany*
*[2]University Hospital Würzburg, Klinik und Poliklinik für Mund--Kiefer- und Plastische Gesichtschirurgie, Würzburg, Germany*
*[3]University Hospital Würzburg, Pathologisches Institut, Würzburg, Germany*

**Introduction**
Oral squamous cell carcinoma (OSCC) is the most common form of head and neck cancer. Surgical interventions combined with irradiation and/or chemotherapy are current standard of care for OSCC. Still, survival rates after treatment remain low and rates of recurrence or metastasis remain high. Therapy with immune checkpoint inhibitors (ICIs) has recently been approved for treatment of recurrent or metastatic OSCC. However, only 1 out of 5 OSCC patients responds to ICI treatment. To explore mechanisms of ICI therapy resistance, we aimed to develop a novel ex vivo autologous co-culture model system of cancer organoids and T cells.
**Material and Methods**
We collected tissue and blood samples from patients with primary, recurrent, and metastatic OSCC. From the patient material, we generated a biobank of patient-derived organoids (PDOs) and isolated peripheral blood mononuclear cells (PBMCs). We then established autologous co-cultures of PDOs and PBMCs to expand tumor-reactive cytotoxic T cells. Using immunophenotyping, we assessed T-cell expansion and anti-tumor reactivity.
**Results and Discussions**
We first successfully generated PDOs from primary, recurrent, and metastatic OSCC samples. Gene-expression profiling and flow cytometry of PDOs demonstrated expression of immunomodulatory molecules such as components of the antigen presentation machinery (e.g., HLA-B, MICB) as well as immune checkpoints such as PD-L1. Although, treatment of PDOs with IFN-gamma increased the expression of these markers, we found stark patient-specific differences between the lines in IFN-gamma response. We next combined PDOs with autologous PBMCs into a suspension co-culture in the presence of interleukin-2. Over 2 weeks, we found robust immune-cell expansion in the co-culture. Comprehensive immunophenotyping confirmed that CD3+ T cells were enriched in the immune-cell fraction. While there was robust expansion of CD4+ T cells in PDO co-cultures, only 3 of 10 PDO lines showed an expansion of cytotoxic CD8a+ T cells in the presence of pembrolizumab (α-PD1), reflecting the heterogeneous response of patients to immunotherapy. To validate tumor-specificity of the expanded cytotoxic T

© 2024 The Authors. Molecular Oncology is published by John Wiley & Sons Ltd on behalf of Federation of European Biochemical Societies cells, we are currently performing organoid killing assays.

**Conclusion**

By adapting cancer organoids as immuno-oncological model, we show for the first time that tumour-reactive T cells can be expanded from OSCC patient material ex vivo. The model system can be applied ultimately to better understand why treatment fails and predict ICI treatment outcome.

### EACR2024-0444

### The natural marine product fucoidan sensitizes breast tumours to anti-PD-1 immunotherapy in vivo

*D. Hussein[1], M. Tolba[2], M. Abdollah[3]*

*[1]Faculty of Pharmacy, Future University in Egypt, Pharmacology, Cairo, Egypt*

*[2]Faculty of Pharmacy, Ain Shams University, Pharmacology and Toxicology, Cairo, Egypt*

*[3]Faculty of Pharmacy, The British University in Egypt, Pharmacology, Cairo, Egypt*

**Introduction**

Breast cancer immunotherapy has gained a lot of momentum in recent years as it aims to counteract the immunosuppressive tumour microenvironment with agents such as immune check point inhibitors (e.g., programmed cell death protein 1/PD-ligand 1 (PD-1/PDL-1) inhibitors). We investigated seaweed fucoidan, a marine drug with promising anti-cancer and immuno-modulatory effects, as an adjuvant to an anti-PD-1/PDL-1 inhibitor (BMS-202). Drugs were tested either as mono-therapies or in combination in mice bearing Ehrlich solid phase (EAC) carcinoma.

**Material and Methods**

Fucoidan was purchased as a purified extract from Buchem-BV (Holland) and BMS-202 was purchased from Abcam (USA). Female Swiss albino mice bearing EAC tumours were randomly allocated into four equal groups (n=10/group): untreated control; fucoidan; BMS-202 or their combination. Treatments continued for two weeks during which body weights and tumour volumes were recorded bi-weekly. Experimental procedures were approved by the research ethics committee of the Faculty of Pharmacy, Ain Shams University in compliance with the EU Directive 2010/EU/63.

**Results and Discussions**

Results showed a significant decrease in the tumour volume in mice treated with monotherapies. H&E staining of excised tumours of the control group demonstrated extensive sheets of pleomorphic and viable tumour cells. Conversely, BMS-202 monotherapy and the combination-treated groups revealed a significant increase in the central mass of necrotic tissue with abundant figures of fragmented and pyknotic tumour cells. The percentage tumour necrosis was significantly ($p<0.05$) higher in the combination group versus both the control and BMS-202 groups. Next, we assessed the tumour tissue levels of cleaved caspase-3 and Ki-67 (marker of proliferation Kiel 67) using immuno-histochemistry as well as phosphorylated extracellular regulated kinase (p-ERK1/2), phosphorylated protein kinase-B (p-Akt) and phosphorylated P38 mitogen-activated protein kinase (p-P38-MAPK) using Western blotting. Results revealed a significantly higher level of the pro-apoptotic caspase 3 along with significantly ($p<0.05$) lower levels of the proliferative (Ki67) and tumorigenic signals (p-ERK1/2, p-Akt and p-P38-MAPK) by 69%, 85% and 87.5%, respectively, in the tumour tissues of the combination treated group versus control.

**Conclusion**

Natural marine fucoidan augmented the anti-tumour activity of anti-PD-1 warranting further research into the underlying mechanisms.

*This project was funded by STDF-Youth grant number 43010.*

### EACR2024-0445

### Lung tumor-derived small and large extracellular vesicles modulate healthy immune cells' phosphorylation pattern

*S. Zahedi[1], M. Ejtehadifar[1], A. Luis[2], P. Borralho[2], A. Bugalho[2], H. Christian Beck[3], A.S. Carvalho[1], R. Matthiesen[1]*

*[1]Computational and Experimental Biology Group, NOVA Medical School-Research, Faculdade de Ciências Médicas- Universidade NOVA de Lisboa- Campo dos Mártires da Pátria- 130- 1169-056, Lisbon, Portugal*

*[2]Hospital CUF Descobertas, CUF Oncologia- 1998-018, Lisbon, Portugal*

*[3]Centre for Clinical Proteomics- Department of Clinical Biochemistry- Odense University Hospital- DK-5000, Department of Clinical Biochemistry- Odense University Hospital- DK-5000 Odense C- Denmark, Odense, Denmark*

**Introduction**

Emerging evidence shows the regulatory role of the extracellular vesicles (EV) in the crosstalk between the tumor cells and immune cells in the tumor micro-environment (TME) of lung cancer. Previously, by Mass spectrometry (MS)-based proteomics analysis, our research identified potential diagnostic biomarkers associated with EVs and immuno-regulatory pathways in pleural effusion samples taken from lung cancer patients (Zahedi. et al. Cancers. 2022). Consequently, we are now focused on comprehending how lung tumor-derived EVs (TDEVs) influence immune cells' activities.

**Material and Methods**

We isolated small and large EVs (sEVs and LEVs) derived from the lung cancer cell line (A549). We characterized two types of EVs by immunoblotting and nanoparticle tracking analyzer. To determine the effect of TDEVs on immune cells, we co-cultured healthy donors peripheral mononuclear cells (PBMCs) with TDEVs in in vitro conditions at different time points and EVs' concentrations. We analyzed the TDEVs' effects on PBMCs by performing a viability assay. Also, we conducted an immunoblotting assay to analyze the phosphorylation alteration in PBMCs treated with TDEVs.

**Results and Discussions**

After the characterization of EVs, first, we observed a decrease in viability and cell count of the treated PBMCs with TDEVs in a concentration and time-dependent manner. Then, we investigated the impact of TDEVs on immune cell phosphorylation. Interestingly, immunoblotting results showed increased tyrosine

© 2024 The Authors. Molecular Oncology is published by John Wiley & Sons Ltd on behalf of Federation of European Biochemical Societies phosphorylation in PBMCs after a short treatment time with TDEVs. Importantly, sEVs induce phosphorylation on PBMCs more than LEVs in a short time. TDEVs partially mimic the source cells' profiles with distinct functions so, they can regulate immune cells' activity in TME. Lung tumor cells potentially modulate healthy donor immune cells' activities by altering tyrosine phosphorylation-related pathways through TDEVs.

**Conclusion**

Together, our study highlighted that lung TDEVs decrease the viability of healthy donor PBMCs and increase overall tyrosine phosphorylation patterns in a short time of exposure. As the next step, we plan to profile the proteome of the PBMCs treated with sEVs and LEVs by an MS-based proteomics study to evaluate changes in PBMCs' proteome in more detail.

### EACR2024-0529
### Eosinophils in colorectal cancer

*S. Dulberg[1], M. Itan[2], A. Kaminitz[1], A. Munitz[2], A. Madi[1]*
*[1]Tel Aviv University, Pathology, Tel Aviv, Israel*
*[2]Tel Aviv University, Clinical Microbiology and Immunology, Tel Aviv, Israel*

**Introduction**

To further understand how tumor cell-derived factors shape the activities of eosinophils in colorectal cancer (CRC), eosinophils from multiple healthy donors were exposed to conditioned media of multiple CRC cell lines. Undertaking an unbiased global RNA sequencing approach, a shared signature of 101 transcripts was identified. Furthermore, RNA expression data was coupled with proteomics data from the tumor cells media, to discover potential ligand-receptor interactions between the different colorectal cancer-conditioned media and the eosinophils. Our analyses identified mechanisms by which eosinophils that interact with tumor cells could be potentially used as targets for future immunotherapy.

**Material and Methods**

Eosinophil isolation: Human eosinophils were isolated from the peripheral blood of healthy donors using MACS eosinophil negative selection isolation kit (Miltenyi Biotec). Preparation of tumor cell-conditioned media and stimulation of eosinophils Tumor cell-conditioned media from the following CRC cell lines was prepared: Caco-2, HCT 116, HCT 15, SW1417, SW480, SW403, SW620. RNA sequencing of human eosinophils RNA libraries were generated using Cell-seq Library protocol, and sequenced on Illumina HiSeq2500, 50 single-end run, with 58,676 genes sequenced.

**Results and Discussions**

To better characterize the potential interactions between factors secreted by CRC cells, and eosinophils, an unbiased screening-based approach was used. Human eosinophils were purified from eight different healthy donors and exposed to conditioned medium (CM) obtained from seven different CRC cell lines, and serum-free-media as control. Thereafter, the eosinophils were subjected to RNA sequencing and the CM sent for proteomic analysis. Alterations in the transcriptional profile of eosinophils, as well as potential ligand-receptor interactions, were computationally predicted and subsequently validated in functional experiments.

**Conclusion**

Eosinophils respond to secreted factors from tumor cells by increasing their ability to respond to IL-3. Increased responsiveness to IL-3 increases eosinophil adhesion, and possibly increases eosinophil survival and direct cytotoxicity. In summary, using an unbiased global screening approach, we identified a "core" transcriptional signature that arises in the context of colorectal cancer. Furthermore, by combining proteomic and genomic approaches, we provide evidence of tumor cell-eosinophil interactions in colorectal cancer that can shape eosinophil responses in the tumor microenvironment.

### EACR2024-0541
### Microtubule inhibition induces immunogenic MYC-directed synthetic lethality

*R. Liu[1], J. Aung[1], J. Klefstrom[1,2,3]*
*[1]University of Helsinki, Faculty of Medicine, Translational Cancer Medicine, Cancer Cell Circuitry lab, Helsinki, Finland*
*[2]Finnish Cancer institute, FICAN South- Helsinki University Hospital, Helsinki, Finland*
*[3]University of California, Department of Cell and Tissue Biology, California, United States*

**Introduction**

While combined chemo-immunotherapy agents are promising in breast cancer treatment, most trials picked combinations based on standard indications than for their immunostimulatory potential. Precluding activation of meaningful anticancer immune responses limits the full clinical benefits of combination therapy. This presents a need for humanized platforms evaluating therapeutic regimens based on capacity for enhancing tumor immunogenicity. Also, recognising that targeting MYC oncogenic pathways remains a persistent goal of cancer therapy, we take a synthetic lethal approach mediating pro-apoptotic functions of MYC, consequently exploiting MYC-dependent vulnerabilities to boost immunogenicity and improve treatment outcomes.

**Material and Methods**

An immunogenic cell death (ICD) evaluation platform was developed in triple-negative breast cancer (TNBC) reporter cell lines expressing calreticulin (CALR)-RFP and high mobility group box 1 (HMGB1)-GFP. Using image-based phenotypic screening, we identified 35 ICD inducers - marked by antigen presentation and dendritic cell activation among others in reporter cell lines. Subsequent in vitro dendritic cell activation assays narrowed this number down to 18 from an initial collection of 528 oncology drugs. MYC-inducible cell lines and Patient-Derived Explant Cultures (PDECs) mimicking the immune-contexture of the original tumors were used to further evaluate key ICD features such as damage-associated-molecular-patterns (DAMPs), cytokine release, cell death, and dendritic cell signatures. Along with in vivo mice vaccination models, a comprehensive approach was taken to validate the role of MYC and its ICD effects.

**Results and Discussions**

Our screens revealed an enrichment of mitosis-associated targets with microtubule modulators emerging as one of the top hits. Throughout our experiments, microtubule inhibitors consistently enhanced CALR translocation

© 2024 The Authors. Molecular Oncology is published by John Wiley & Sons Ltd on behalf of Federation of European Biochemical Societies from ER to cell membrane surface, ATP secretion, and HMGB1 release in TNBC cell lines, indicating positive ICD. Our findings also suggest sensitization by MYC to microtubule inhibitors in TNBC cells, underscoring its potential regulation of ICD-inducing pathways following prolonged mitotic arrest.

**Conclusion**

Our data strongly suggests that microtubule inhibition is a promising strategy in achieving MYC-directed synthetic lethality. Our findings raise questions on how MYC modulates pathways leading to ICD, presenting avenues for developing new biomarkers and novel combinations for enhancing efficacy of breast cancer immunotherapies.

### EACR2024-0552

### Immunotherapy for infant leukemia: Investigating and exploiting the fetal leukemia microenvironment

E. Doyle[1], A. MacCallum[1], H. Vaughan[1], S. Mariani[1]
*[1]Institute for Regeneration and Repair- University of Edinburgh, Centre for Inflammation Research, Edinburgh, United Kingdom*

**Introduction**

Infant leukemia is a tragic diagnosis with a poor prognosis. Approximately 50% of patients relapse, and chemotherapy is linked to subsequent health concerns later in life, highlighting the need for new treatment strategies. In adult leukemias, leukemia-associated macrophages (LAMs) may support the growth and chemoresistance of leukemia cells. However, there is little research on a possible role of LAMs in infant leukemia. The *MLL-AF9* translocation (t(9; 11) (p22; q23)) occurs in ~20% of infant acute leukemia cases. In *MLL-AF9*$^+$ infant leukemia, this translocation occurs in utero, meaning the leukemia microenvironment is established in the fetus. Thus, we are investigating the role of embryonic macrophages in the early progression of *MLL-AF9*$^+$ infant leukemia.

**Material and Methods**

To study the fetal leukemia microenvironment, we use a mouse with an inducible human *MLL-AF9* fusion gene under control of the cKit promoter (*iMLL-AF9:cKit-rtTA* mice). Changes in the hematopoietic compartment of *iMLL-AF9:cKit-rtTA* embryonic day (E)14.5 and E18 fetal liver (FL) and E18 fetal bone marrow (FBM) were assessed by flow cytometry, colony-forming unit (CFU) assay, and hematoxylin and eosin (H&E) staining. Single-cell RNA sequencing (scRNA-seq) of cKit$^-$ CD45$^+$ cells from E14.5 *iMLL-AF9:cKit-rtTA*$^+$ FL revealed how macrophages respond to the presence of *MLL-AF9*$^+$ leukemia cells.

**Results and Discussions**

There is no significant increase in the frequency of CD11b$^+$, cKit$^+$, CD45$^+$ or Lin$^-$Sca$^+$cKit$^+$ (LSK) cells in *iMLL-AF9:cKit-rtTA*$^+$ E14.5 FL. On E18, *iMLL-AF9:cKit-rtTA*$^+$ FL contains LSK cells with higher progenitor potential and a higher frequency of cKit$^+$ cells than the WT. On E18, *iMLL-AF9:cKit-rtTA*$^+$ FBM contains a higher frequency of cKit$^+$ cells and F4/80$^+$CD11b$^+$CD169$^+$ cells than the WT, indicating elevated numbers of hematopoietic progenitors and macrophages, respectively. H&E staining of E18 *iMLL-AF9:cKit-rtTA*$^+$ FL shows myeloid infiltration. Preliminary scRNA-seq of E14.5 FL revealed different transcriptional profiles of *MLL-AF9*$^-$, *Csf1r*-expressing cells in WT vs *iMLL-AF9:cKit-rtTA* embryos, indicating that *Csf1r*-expressing cells modulate their transcriptional program in response to the presence of *MLL-AF9*$^+$ cells.

**Conclusion**

These preliminary data indicate that *iMLL-AF9:cKit-rtTA*$^+$ embryos express a leukemia-like phenotype and that non-leukemic myeloid cells transcriptionally respond to the presence of *MLL-AF9*$^+$ leukemia cells rapidly. These results provide early support for the existence of LAMs in the *MLL-AF9*+ fetal leukemia micro-environment.

### EACR2024-0604

### Escherichia Coli infection: A disruptive factor in prostate cancer cells survival and metabolism?

L.R.S. Fonseca[1], M.G. Veiga[1], M. Feijó[1], M.I. Figueira[1], C. Gaspar[1,2], J. Rolo[1], S. Laurentino[3], A. Palmeira-de-Oliveira[1,2], S. Socorro[1]
*[1]CICS-UBI- Health Sciences Research Center, Faculty of Health Sciences- University of Beira Interior, Covilhã, Portugal*
*[2]Labfit-Health Products Research and Development Lda, UBImedical- Estrada Nacional 506- 6200-284, Covilhã, Portugal*
*[3]Centre of Reproductive Medicine and Andrology, Institute of Reproductive and Regenerative Biology, University of Münster, Münster, Germany*

**Introduction**

The microbiome is a new dimension of cancer, which goes beyond tumour-promoting inflammation. Prostate cancer (PCa) bacteriome and its relationship with cell survival and tumour growth has been characterized; however, the importance of bacteria and their toxins targeting different cancer hallmarks, such as cell survival and metabolism, remains unknown. This work investigates the effect of *Escherichia Coli* and its major component of the outer membrane, lipopolysaccharide (LPS), in PCa cells survival, migration and metabolism.

**Material and Methods**

Co-cultures *E. coli*/human androgen-sensitive (22Rv1) or castration-resistant (DU145) were established for 3 and 6h. Alternatively, 22Rv1 and DU145 cells were treated with a concentration range of LPS (0 - 1000 ug/mL) for 6 and 24h. Cell viability (MTT assay) and proliferation (Ki-67 immunocytochemistry) were evaluated. Cell migration was determined by the Scratch assay. Caspase-3-like activity was used to assess apoptosis. Spectro-photometric analysis was used to determine glucose and fatty acids consumption and lactate and nitrite content. Western blot analysis determined the expression levels of target regulators of cell proliferation and apoptosis.

**Results and Discussions**

Human PCa cell lines co-cultured with *E. coli* presented increased glucose and fatty acid consumption and altered lactate and nitrite content. *E. coli* also altered caspase-3-like activity and the expression of key regulators of cell survival, such as MAPK. LPS increased lactate production in 22Rv1 cells, though with no effect on glucose consumption. In DU145 cells, treatment with

Molecular Oncology 18 (Suppl. 1) (2024) 1–495
© 2024 The Authors. Molecular Oncology is published by John Wiley & Sons Ltd on behalf of Federation of European Biochemical Societies

LPS increased glucose and fatty acid consumption and altered lactate production in a time-dependent manner. Moreover, LPS increased cell viability, proliferation, and migration in both PCa cell lines and displayed a time-dependent effect on nitrite content and caspase-3 like activity.

**Conclusion**

Altogether, the obtained findings first indicate that *E. coli* may disrupt PCa cells metabolism and survival, highlighting the impact of the bacteriome in prostate carcinogenesis.

*Acknowledgements: Bolsa LPCC/Gillette-Cancro da Próstata (MOD.PD.NAC.015.01); FEDER (POCI-COMPETE 2020-Operational Programme Competitiveness and Internationalisation, Project 029114); FCT-Foundation for Science and Technology (Project UIDB/00709/2020 and Fonseca LR-2021.07634.BD fellowship).*

### EACR2024-0611
### Neutrophil pathways are associated with ID1 and ID3 expression levels and modulate the tumor immune microenvironment in adult patients diagnosed with B-cell precursor acute lymphoblastic leukemia

*J.N. Poveda Garavito[1], A.L. Combita[1], C.A. Orozco[2], J. Zabaleta[3], S. Quijano[4], R. Parra-Medina[5], Y. Torres-Llanos[1], N. Cruz-Rodriguez[6]*

*[1]Instituto Nacional de Cancerología, Grupo de Investigación en Biología del cáncer, Bogotá, Colombia*

*[2]Instituto Nacional de Cancerología, Grupo de Investigación Traslacional en Oncología, Bogotá, Colombia*

*[3]Louisiana State University, Health Sciences Center, New Orleans, United States*

*[4]Pontificia Universidad Javeriana, Grupo de Inmunobiología y Biología Celular, Bogotá, Colombia*

*[5]Instituto Nacional de Cancerología, Department of Pathology, Bogotá, Colombia*

*[6]Versiti Blood Research Institute, Versiti Blood Research Institute, Milwaukee, United States*

**Introduction**

The diagnosis of B-cell precursor acute lymphoblastic leukemia (BCP-ALL) in adults often carries a poor prognosis. *ID1* and *ID3* genes have been identified as predictors of poor response in Colombian adult BCP-ALL patients, playing roles in cancer development. In different cancer models, these genes have been associated with immune regulator populations within the tumor immune microenvironment (TIME). BCP-ALL development alters the immune cell composition and the bone marrow (BM) tumor microenvironment, affecting disease progression and response to therapy. This study analyzes gene expression levels of *ID1* and *ID3* in relation to TIME and immune evasion-associated genes.

**Material and Methods**

This exploratory study analyzed bone marrow (BM) samples from 6 BCP-ALL patients diagnosed at the National Cancer Institute of Colombia. First, RT-qPCR was used to assess *ID1* and *ID3* expression in BM tumor cells. Flow cytometry characterized immune populations in the TIME. RNA-seq evaluated immune genes associated with BCP-ALL immune response, while xCell and CytoSig analyzed TIME cell profiles and cytokines. Pathway analysis, gene ontology, and differential gene expression (DEGs) were examined, with functional enrichment analysis performed using KEGG ontology.

**Results and Discussions**

Patients were divided into basal and overexpression groups based on *ID1* and *ID3* expression in RT-qPCR. A total of 15,951 differentially expressed genes (p adjusted <0.05) were identified between these groups, with top genes associated with neutrophil pathways. Gene set enrichment analysis revealed increased expression of genes associated with neutrophil degranulation, immune response-related neutrophil activation, and neutrophil-mediated immunity. These findings correlated with the xCell data. Patients with high *ID1* and *ID3* levels showed significant differences in neutrophils (p=0.0008), monocytes (<0.0001) and CD4+ naive T cells (p=0.0240) compared to basal levels. Microenvironment and immune scores were also significantly different (p=0.0016 and p=0.0017, respectively), consistent with the flow cytometry results. Elevated cytokine levels associated with neutrophil activation supported these findings. Validation was performed using the TARGET and MILE databases.

**Conclusion**

Our results show important differences between the expression level of *ID1* and *ID3* in cancer cells and the populations of TIME, suggesting a role in evading the immune response of *ID1* and *ID3* in BCP-ALL, mainly related to neutrophil pathways.

### EACR2024-0615
### Multi-omics spatial analyses of immune cell state in non-small cell lung cancer reveal features associated with short relapse-free survival

*C. Marceaux[1], V. Gayevskiy[1], I. Tarasova[2], M. Christie[3], P. Antippa[4], T. Speed[2], K. Rogers[5], B. Phipson[2], M.L. Asselin-Labat[1]*

*[1]Walter and Eliza Hall Institute of Medical Research, Personalised Oncology Division, Melbourne, Australia*

*[2]Walter and Eliza Hall Institute of Medical Research, Bioinformatics, Melbourne, Australia*

*[3]The Royal Melbourne Hospital, Pathology, Melbourne, Australia*

*[4]The Royal Melbourne Hospital, Thoracic Surgery, Melbourne, Australia*

*[5]Walter and Eliza Hall Institute of Medical Research, Advanced Technology and Biology Division, Melbourne, Australia*

**Introduction**

Relapse-free survival after surgical resection is heterogeneous in lung adenocarcinoma (LUAD) and lung squamous cell carcinoma (LUSC), the two major subtypes of non-small cell lung cancer. Yet, little is known about the composition and spatial organisation of the tumour microenvironment that could explain the different prognoses for lung cancer patients.

**Material and Methods**

We performed deep profiling of the tumour micro-environment of 64 resected LUAD and LUSC using spatial proteomics with Multiplex Ion Beam Imaging and spatial transcriptomics analysis of tumour cells in

© 2024 The Authors. Molecular Oncology is published by John Wiley & Sons Ltd on behalf of Federation of European Biochemical Societies matched tumour regions using GeoMx Nanostring. These data were integrated with clinical data and survival to interrogate association between spatial molecular and cellular characteristics of the tumours patient outcomes.

**Results and Discussions**

Spatial proteomics analyses led to the stratification of 64 tumours into three subsets according to the composition of their tumour microenvironment. Myeloid-enriched tumours with infiltration of neutrophil clusters had short relapse-free survival while a lymphoid-only or mixed lymphoid/myeloid infiltrate was not associated with patient survival. Further exploration of lymphoid-infiltrated tumours revealed the identification of a subset of activated T cells (MI-T cells) with a distinct molecular profile infiltrating a subset of tumours. The presence of this discreet subset of MI-T cells in the tumour core was associated with prolonged patient survival. Transcriptomic analysis revealed TNFα signaling was activated in tumors with high infiltration of MI-T cells in the tumor core.

**Conclusion**

Overall, our comprehensive spatial analysis of the tumour microenvironment and profiling of the tumour transcriptome in matched regions led to the identification of a new subset of T cells and provided novel insights to increase the recruitment of these cells to the tumour core to promote anti-tumour immunity. These results will help with the stratification of patients who may benefit from adjuvant immunotherapy.

### EACR2024-0618

### Diffuse large B-cell lymphoma-derived-extracellular vesicles alter the level of the ubiquitinated proteins in immune cells

*M. Ejtehadifar[1], S. Zahedi[1], P. Gameiro[2], J. Cabeçadas[2], M. S. Rodriguez[3], M. Gomes da Silva[2], H.C. Beck[4], R. Matthiesen[1], A.S. Carvalho[1]*

*[1]Computational and Experimental Biology Group- NOVA Medical School-Research- Faculdade de Ciências Médicas, Universidade NOVA de Lisboa- Campo dos Mártires da Pátria, Lisbon, Portugal*

*[2]Instituto Português de Oncologia, Departament of Hematology, Lisbon, Portugal*

*[3]Laboratoire de chimie de coordination LCC -CNRS, Chemistry Laboratory, Toulouse, France*

*[4]Centre for Clinical Proteomics, Department of Clinical Biochemistry- Odense University Hospital, DK-5000 Odense, Denmark*

**Introduction**

Diffuse large B-cell lymphoma (DLBCL) is an aggressive type of non-Hodgkin lymphoma. Extracellular vesicles (EVs) released by tumors exert influence on the functions of immune cells (ICs). Many functions of the ICs could be regulated by the ubiquitin-proteasome system (UPS). In this study, we aim to investigate the impact of DLBCL-EVs on the ubiquitinated protein level within the circulating ICs.

**Material and Methods**

ICs of the 10 DLBCL patients and 10 healthy donors (HDs) were isolated from the blood samples. The proteome of the ICs was extracted and enriched by the diGLY method. Whole proteome and diGLY enriched samples were analyzed by mass spectrometry (MS). To address the effects of the EVs on ICs, we isolated small and large EVs (sEVs and LEVs) from the conditioned medium (CM) of a DLBCL cell line. Then, HDs' ICs were treated with the CM and EVs at different time points and the level of the ubiquitinated proteins was evaluated by immunoblotting.

**Results and Discussions**

Whole proteomics analysis revealed that granulation and cytoskeleton in DLBCL-ICs are the two most significant up- and down-regulated terms, respectively. The diGLY enrichment revealed that cytoskeleton-related proteins are the main ubiquitinated proteins in DLBCL-ICs. In in vitro conditions, CM and EVs increased the ubiquitination level in ICs in a time-dependent manner. By further experiments, the peak of the ubiquitinated proteins in ICs treated with LEVs was observed after 30 minutes. Meanwhile in ICs exposed with CM and sEVs the peak of the ubiquitinated proteins was detected after 24 hours. EV-depleted CM could not increase the level of ubiquitination in HD-ICs. Data here revealed that sEVs and LEVs are different in increasing the level of the ubiquitinated proteins in ICs. EVs' content and surface molecules can induce changes in recipient cells. Since sEVs and LEVs release their content at the same rate (Geng T. 2024), it seems that differences in sEVs and LEVs binding to the ICs resulted in the accumulation of the ubiquitinated proteins in different time points.

**Conclusion**

Proteomics results suggest markedly distinct ICs' protein profiles between DLBCL patients and HDs. MS and diGLY enrichment findings showed suppression of the DLBCL-ICs which have been reported before in ICs of chronic lymphocytic leukemia patients (Maffei R. 2013). In in vitro, we indicated that cancer cells-derived EVs can increase the ubiquitination level of the HD-ICs. Collectively, EVs are a way in which tumor cells can affect ubiquitination in ICs.

### EACR2024-0628

### Tunable 3D cell models recapitulating the tumour microenvironment for in vitro immuno-oncology assays

*D. Nguyen[1], R. Bleach[2], L. Belfiore[1], C. Yee[1], H. Seyedzadeh[3], J. Goyette[3], K. Gaus[3], M. Besnier[1]*

*[1]Inventia Life Science, Biology, Sydney, Australia*

*[2]Inventia Life Science, Biology, Dublin, Australia*

*[3]University of New South Wales, Medical Sciences, Sydney, Australia*

**Introduction**

In vitro assays mimicking the tumour microenvironment are essential for immuno-oncology research. While 2D cell models are common due to simplicity and cost, they lack biological relevance, hindering translation to the clinic. Current 3D models offer sophistication but have limitations. Here, we present two 3D immuno-oncology models: direct co-culture and infiltration models. These models, generated by the RASTRUM™ Platform's drop-on-demand 3D bioprinting, allow for screening immuno-modulatory compounds, assessing immune checkpoint inhibitors, and investigating combination therapies at scale.

**Material and Methods**

Molecular Oncology 18 (Suppl. 1) (2024) 1–495

© 2024 The Authors. Molecular Oncology is published by John Wiley & Sons Ltd on behalf of Federation of European Biochemical Societies

For the 3D cell direct co-culture, lung (A549) cancer cells and immune cells (peripheral blood mononuclear cells, PBMCs) were generated using the RASTRUM Platform (Inventia Life Science) in RASTRUM Px02.28P (~1 kPa) Matrices (Inventia Life Science). The printing design was prepared using RASTRUM Cloud Software (Inventia Life Science). 3D tumour cell models were then cultured for 4 days in the absence (unactivated) or presence (activated) of CD3/CD28 T cell activator. For the infiltration 3D model, lung (A549) cancer cells A549 cells were cultured in RASTRUM Matrix Px02.28P for 3 days before activated cytotoxic T lymphocytes (CTLs) were added to the media. Endpoint imaging analysis performed on infiltration and co-culture of immune cells with tumour cells in 3D cell models.

**Results and Discussions**

We used A549 cancer cells in the most physiologically relevant RASTRUM Matrix and tested their compatibility with an immuno-oncology application using PBMCs. We showed that PBMCs and tumour cells were co-cultured within a matrix that mimics the tumour microenvironment and after 4 days in co-culture, activated PBMCs inhibited the growth of tumour spheroids. We then demonstrated infiltration of CTL within the matrix over 4 days and in addition, CTL-mediated cytotoxicity, cell growth and CTL motility were modulated by matrix stiffness.

**Conclusion**

In summary, we have developed two 3D cell culture models *via* the RASTRUM Platform for studying immune cell interactions with tumour cells and their anti-tumour effects. These models allow for co-cultures replicating the tumour microenvironment, matrix tunability to influence immune cell behaviour, and immune cell infiltration into the matrix for studying immune-tumour cell associations.

### EACR2024-0638

### Peritoneal effusion in ovarian carcinoma reveals decreased B lymphocytes and increased Foxp3+ regulatory T cells (Tregs) as mediators of immunosuppressive microenvironment

*M. Sharma[1], R. Srinivasan[1], A. Raj Sharma[1], M. Sachdeva[2], P. Gupta[1], B. Rai[3], R. Bagga[4]*
*[1]Postgraduate Institute of Medical Education and Research PGIMER, Department of Cytology and Gynecological Pathology, Chandigarh, India*
*[2]Postgraduate Institute of Medical Education and Research PGIMER, Department of Haematology, Chandigarh, India*
*[3]Postgraduate Institute of Medical Education and Research PGIMER, Department of Radiotherapy and Oncology, Chandigarh, India*
*[4]Postgraduate Institute of Medical Education and Research PGIMER, Department of Obstetrics and Gynecology, Chandigarh, India*

**Introduction**

High-grade serous ovarian carcinoma (OC) frequently presents with peritoneal effusion, with cancer cells admixed with mesothelial cells, macrophages and lymphocytes constituting the 'immune micro-environment'. The aim of this study was to perform immune cell type profiling including lymphocyte subpopulations and macrophages in peritoneal effusion of patients with OC in comparison to non-ovarian cancers (NOC) and a control group with No malignant cells (NM) to discern differences.

**Material and Methods**

Multiparametric multicolour flow cytometry was performed on 79 peritoneal effusions in 3 groups: OC (41), NOC (19), and NM (19). EpCAM+ identified malignant cell proportion. CD45+ lymphocytes were gated for evaluation of T-lymphocyte subsets (CD3+ gated CD4+ T-helper-cells / CD8+ cytotoxic-T-cells), regulatory T-cells (Tregs, CD4+CD25$^{hi}$ CD127$^{low}$ / FOXP3+), Macrophages (M1,CD68+ / M2,CD163+) and B cells (CD19+/CD20+).

**Results and Discussions**

Intergroup comparison revealed significantly higher proportion of CD3+, CD8+, and CD25$^{high}$/CD127$^{low}$ FoxP3+Tregs cells, significantly decreased B-cells and M2 macrophages in ovarian carcinoma as compared to the NOC and NM groups (Table-1).

Table 1: Immune cell profiling in peritoneal effusion of ovarian carcinoma

| Parameter | Ovarian Cancer (OC) N=41 | Non-Ovarian Cancer (NOC) N=19 | Non-malignant (NM) N=19 | p-value (Kruskal-Wallis ANOVA) |
|---|---|---|---|---|
| | Median levels % (95%CI: Lower;Upper) | | | |
| EpCAM | 22.80 (22.92; 39.46) | 16.20 (12.16; 26.15) | NA | NS |
| CD3+ T cells | 66.50 (56.99; 71.80) | 13.00 (14.63; 45.10) | 19.90 (14.51;43.21) | 0.0008 (OCvsNOC) 0.0004 (OCvsNM)) |
| CD4+ T cells | 45.60 (39.02; 50.37) | 52.90 (33.11; 58.77) | 52.60 (37.52;61.83) | NS |
| CD8+ T cells | 30.30 (27.01; 35.70) | 11.40 (6.039; 20.09) | 5.200 (4.082;15.51) | 0.0001 (OCvsNOC) <0.0001 (OCvsNM) |
| CD25$^{high}$/CD127$^{low}$ FoxP3+Tregs | 27.60 (28.56; 41.95) | 37.90 (28.35; 50.23) | 15.40 (11.44;20.76) | 0.0013 (OCvsNM) 0.0012 (NOCvsNM) |
| B cells (CD19/20) | 5.300 (5.841; 16.14) | 29.90 (24.37; 44.44) | 34.20 (24.55;48.14) | <0.0001 (OCvsNOC) <0.0001 (OCvsNM) |
| CD68 M1 macrophages | 19.30 (17.66; 29.22) | 23.50 (14.93; 35.97) | 35.70 (18.36;44.46) | NS |

© 2024 The Authors. Molecular Oncology is published by John Wiley & Sons Ltd on behalf of Federation of European Biochemical Societies

| | | | | |
|---|---|---|---|---|
| CD 163 M2 macrophages | 10.70 (14.20; 23.82) | 60.40 (33.80; 66.24) | 52.30 (34.80;71.44) | 0.0047 (OCvsNOC) 0.0035 (OCvsNM) |

**Conclusion**
Immune microenvironment profiling in peritoneal effusion of ovarian carcinoma revealed high $FoxP_3^+$Tregs with lower B-cells and M2 macrophages contributing to tumour immune evasion and disease aggressiveness.

### EACR2024-0646
### Expression of immune checkpoints PD-1, PD-L1 and B7-H4 on Tumor associated macrophages in patients with Urothelial Carcinoma of Bladder: Rational therapeutic targets

*A. SINGH*[1], *D. Raja*[1], *A. Seth*[2], *S. Kaushal*[3], *A. Sharma*[1]
[1]*All India Institute of Medical Sciences AIIMS, Biochemistry, New Delhi, India*
[2]*All India Institute of Medical Sciences AIIMS, Urology, New Delhi, India*
[3]*All India Institute of Medical Sciences AIIMS, Pathology, New Delhi, India*

**Introduction**
Monoclonal antibodies targeting immune checkpoints have emerged as a potential anticancer strategy to combat Urothelial carcinoma of bladder (UCB). Studies have well established the expression of immune checkpoints on Cytotoxic T cells, however despite the benefits of these immune checkpoint blockers in UBC patients still a subset of patients doesn't benefit from it. Immune checkpoints PD-1, PD-L1, and B7-H4 have been studied on T cells but studies of their expression on macrophages are scarce in UCB. Therefore, we aim to assess the expression of immune checkpoint PD-1, PD-L1, and B7-H4 in TAMs in UBC patients.

**Material and Methods**
The frequency of PD-1, PD-L1, and B7-H4 were evaluated on macrophages in tumor tissue UBC patients by flow cytometry. Their expression on monocytes from PBMC of patient and age-matched healthy control were profiled. Tissue localization of these checkpoints was assessed by immunofluorescence on CD68+ macrophages on bladder tumor tissue. PBMC of patients were differentiated into macrophages and were supplemented with tumor-conditioned media from tumor single-cell suspension to obtain monocyte-derived TAMs in-vitro. M1 and M2 polarized macrophages were utilized to compare the expression of PD-1, PD-L1, and B7-H4. In-vitro assessment of B7-H4 and PD-L1 blockade was performed in the co-culture of PBMC and high-grade bladder cancer cell line.

**Results and Discussions**
TAMs were significantly elevated in bladder tumors ($p<0.001$). PD-1, PD-L1, and B7-H4 were observed to be elevated on bladder tumor tissue in comparison to adjacent normal tissue and showed elevated expression in high-grade tumors. Monocytes in circulation had increased expression of immune checkpoints as compared to healthy donors. We report increased infiltration of CD68+B7H4+ and CD68+PD-L1+ cells at the tumor site. Similarly, monocyte-derived TAMs expressed all three immune checkpoints (mRNA and protein) in comparison to M1 and M2-like macrophages. Upon blocking increased CD8 T cell and monocyte function.

**Conclusion**
This data provides us insight into the upregulation of B7-H4 and PD-L1 in TAMs and monocyte-derived TAMs in- vitro. In-vitro blocking of B7-H4 and PD-L1 provides a therapeutic avenue for improving the anti-tumor response against UCB. Overall, our data identifies B7-H4 and PD-L1 on macrophages as promising therapeutic targets as monotherapy or in combination in UCB patients but requires further validation.

### EACR2024-0661
### Discovery of DN027262, a Highly Potent and Selective Active TLR8-ISAC for the Treatment of Solid tumor

*W. Zhang*[1], *M. Li*[2], *J. Ren*[2], *P. Qin*[1], *W. Yang*[1], *L. Wang*[1], *Y. Ru*[2], *Y. Sun*[3]
[1]*Shanghai De Novo Pharmatech- Co.LTD., Chemistry and ADC Research, Shanghai, China*
[2]*Shanghai De Novo Pharmatech- Co.LTD., Cancer Biology, Shanghai, China*
[3]*Shanghai De Novo Pharmatech- Co.LTD., Management, Shanghai, China*

**Introduction**
Toll-like receptors (TLRs) are an important family of receptors that senses ssRNA fragments from pathogens. TLR8 activation can initiate both innate and adaptive immune responses and reverse Treg and MDSC mediated immune suppression by tumors. Immune-stimulating antibody conjugates (ISAC) combining tumor-targeting monoclonal antibodies with immunostimulatory agents allow localized delivery of immune activators into tumors. Here, we report the Denovo preclinical candidate DN027262, a novel ISAC comprising a highly selective TLR8 agonist conjugated to an anti-HER2 antibody. Data showed our TLR8-ISAC has better antitumor efficacy than SBT6050. Combination with aPD-1, we observed better CR rates and better overall survival data in preclinical models.

**Material and Methods**
In vivo efficacy studies were performed in TLR8 humanized Syngeneic models. Safety, immunogenicity, pharmacokinetics, and pharmacodynamics were assessed in single- and repeat-dose studies using cynomolgus monkeys.

**Results and Discussions**
Through structure-based drug design, we discovered a novel selective small molecule TLR8 agonist, which exhibited a more than 200-fold potency over TLR7 in EC50 values. A series of drug-linkers were synthesized and conjugated to the HER2-targeting antibody. In vitro, these ISACs were found to induce high cytokine release in a PMBC assay when co-cultured with the HER2-high expressing cancer cell lines. In vivo, tumor regression was observed with doses at 3~20 mg/kg in hTLR8 syngeneic HER2-expressing tumors models. Tumor is completely eradicated in all groups at a single dose of 7.5mg/kg via subcutaneous administration in immunocompetent murine models, with prolonged inhibition effect on tumor growth after drug withdrawal.

© 2024 The Authors. Molecular Oncology is published by John Wiley & Sons Ltd on behalf of Federation of European Biochemical Societies

Combination with αPd-1, the murine tolerance was not affected, but the mPFS of not cured mice have been doubled. DN027262 can drive tumor killing by TLR mediated activation of myeloid cells and subsequent T-cell-mediated antitumor immunity, resulting in tumor clearance and immunological memory. Consistently, in vivo cynomolgus monkey studies by consecutive treatments with DN027262 at different doses resulted in a significant increase of immune system cells including monocytes, cDCs and natural killer (NK) cells, correlated with TLR8 activation. All these changes were back to normal after a period of recovery (2-3 weeks).

**Conclusion**

DN027262 exhibited a remarkable anti-tumor activity both in vitro and in vivo and provided a favorable safety window, supporting novel insights for immunotherapies.

### EACR2024-0674

### Multiparametric mass cytometry-based profiling reveals a stimulated peripheral immunophenotype in healthy women with germline pathogenic BRCA1 variants

*K. Horti-Oravecz*[1,2], *J.Á. Balog*[3], *D. Kövesdi*[4], *I. Kelemen*[1], *A. Bozsik*[1,5], *J. Papp*[1,5], *H. Butz*[1,5,6,7], *A. Patócs*[1,5,6], *G.J. Szebeni*[3,8], *V.K. Grolmusz*[1,5,6]

[1]*National Institute of Oncology- Comprehensive Cancer Center, Department of Molecular Genetics and the National Tumorbiology Laboratory, Budapest, Hungary*

[2]*Semmelweis University, Doctoral School, Budapest, Hungary*

[3]*HUN-REN Biological Research Center, Laboratory of Functional Genomics, Szeged, Hungary*

[4]*Eötvös Loránd University and HUN-REN-ELTE Complement Research Group, Department of Immunology, Budapest, Hungary*

[5]*HUN-REN-SE, Hereditary Cancers Research Group, Budapest, Hungary*

[6]*Semmelweis University, Department of Laboratory Medicine, Budapest, Hungary*

[7]*Department of Oncology Biobank, National Institute of Oncology- Comprehensive Cancer Center, Budapest, Hungary*

[8]*University of Szeged, Department of Physiology-Anatomy and Neuroscience- Faculty of Science and Informatics-, Szeged, Hungary*

**Introduction**

Germline pathogenic variants of *BRCA1* and *BRCA2* (gpath(*BRCA1/2*)) are associated with hereditary breast and ovarian cancer syndrome (HBOC). The age-related incidence and the phenotypes of the developing breast cancer have gene-specific characteristics with gpath(*BRCA1*) correlated with immune-infiltrated triple-negative breast cancer (TNBC) at earlier age and gpath(*BRCA2*) usually predisposing to hormone-sensitive breast cancer (HRBC) at later ages. Leveraging tumor-immune interactions in TNBC led to the clinical application of immune checkpoint inhibitors in the neoadjuvant setting. Earlier observations revealed systemic immunological differences in breast cancer patients, however, no information is available on whether germline genetic predisposition alters the peripheral immune phenotype. Therefore, we aimed to apply a mass cytometry-based immunophenotyping panel to address this question.

**Material and Methods**

66 women were included in the study, among which 20 were healthy controls not carrying the familial germline predisposition to cancer, 12 and 10 healthy women were carriers of gpath(*BRCA1*) and gpath(*BRCA2*), respectively and 12-12 women were treatment-naive HRBC and TNBC patients. Peripheral blood mononuclear cells (PBMCs) were isolated by gradient centrifugation. A mass cytometry panel containing 35 cell surface markers was designed and applied with measurements performed on a Helios mass cytometer. Data-driven analysis included unsupervised clustering using the FLOWSOM algorithm and statistical analyses comparing the abundance of immune subpopulations and their phenotype. The study was approved by the Scientific and Research Ethics Committee of the Medical Research Council of Hungary.

**Results and Discussions**

We detected an elevated frequency of monocytes in cancer patients and the altered abundance of four immune subpopulations in healthy gpath(*BRCA1*) carriers, while no difference was observed in and healthy gpath(*BRCA2*) carriers compared to healthy controls. Additionally, the elevated abundance of three out of these four subpopulations (IgD-CD27+CD95+ B cells and two subpopulations of CD45RA-CCR7+CD38+ CD4+ T cells) were also elevated in TNBC patients compared to healthy controls.

**Conclusion**

Based on the phenotypes of the increased cell populations, our results reflect a TNBC-associated activated peripheral immune phenotype in healthy gpath(*BRCA1*) carriers which needs to be further analyzed to ascertain its functional relevance.

### EACR2024-0691

### PD-L1 immune checkpoint inhibitor expression on ovarian carcinoma cells in peritoneal effusions predicts for poor response to neoadjuvant chemotherapy

*M. Sharma*[1], *R. Srinivasan*[1], *A.R. Sharma*[1], *M. Sachdeva*[2], *P. Gupta*[1], *B. Rai*[3], *R. Bagga*[4]

[1]*Postgraduate Institute of Medical Education and Research PGIMER, Department of Cytology and Gynecological Pathology, Chandigarh, India*

[2]*Postgraduate Institute of Medical Education and Research PGIMER, Department of Haematology, Chandigarh, India*

[3]*Postgraduate Institute of Medical Education and Research PGIMER, Department of Radiotherapy and Oncology, Chandigarh, India*

[4]*Postgraduate Institute of Medical Education and Research, Department of Obstetrics and Gynecology, Chandigarh, India*

**Introduction**

High grade serous ovarian cancer (OC) patients frequently present with peritoneal effusion with variable numbers of malignant cells, mesothelial cells, macrophages and lymphocytes. The aim of this study was to evaluate PD-L1 / PD-1 immune checkpoint inhibitor

© 2024 The Authors. Molecular Oncology is published by John Wiley & Sons Ltd on behalf of Federation of European Biochemical Societies expression in cancer cells / lymphocytes in peritoneal effusions in patients with and without OC.

**Material and Methods**

Multiparametric flow cytometry evaluation was performed on 79 cases of ascitic fluid comprising 41 OC, 19 non-ovarian cancers (NOC) vs control 19 non-malignant (NM) effusions. PD-1 expression on CD4+ and CD8+T-cells was compared in the 3 groups and PD-L1 expression evaluated on cancer cells (CD45/ EpCAM+) and macrophages (CD45+/CD68+/CD163+) in OC and NOC groups. In 30 OC cases, response to neo-adjuvant chemotherapy (NACT) was assessed on the interval debulking specimen by chemotherapy response score (CRS) as 1- nil, 2-intermediate and 3-complete response.

**Results and Discussions**

Intergroup comparison is shown in Table-1.

Table 1: PD-1 and PD-L1 expression in immune cells and cancer cells in peritoneal effusion

| Parameter | Ovarian Cancer (OC) N=41 | Non-Ovarian Cancer (NOC) N=19 | Non-malignant (NM) N=19 | p-value (Kruskal-Wallis ANOVA) |
|---|---|---|---|---|
| | Median levels % (95%CI: Lower;Upper) | | | |
| CD4 PD-1 | 44.40 (40.02; 51.59) | 41.00 (29.55; 55.74) | 37.20 (27.40; 53.21) | Not significant |
| CD8 PD-1 | 30.10 (25.94; 34.17) | 38.20 (31.60; 45.69) | 40.30 (29.88; 53.52) | Not significant |
| CD68 M1 PD-L1 | 1.700 (3.649; 12.82) | 10.70 (7.534; 19.34) | 17.20 (13.27; 33.74) | p=0.0040 (OC vs NM) |
| CD163 M2 PD-L1 | 1.300 (1.559; 9.763) | 4.60 (2.589; 14.63) | 4.200 (1.769; 11.62) | p=0.0201 (OC vs NOC) |
| EpCAM+ PD-L1 | 5.200 (6.479; 20.30) | 8.300 (5.852; 25.12) | Not applicable | Not significant |
| Ovarian Cancer PD-L1 TPS | CRS 1 (n=9) 14.30 (2.242; 46.65) | CRS 2 (n=14) 6.250 (3.319;20.15) | CRS 3 (n=7) 1.300 (-0.753;7.096) | p=0.0353 (CRS 1 vs 3) |

Intergroup comparison revealed no difference in PD-1 levels on CD4 or CD8 T-cells. PD-L1 expression on M1/M2 macrophages in OC was significantly lower than in NOC. In OC and NOC group, cancer cells showed >1% PD-L1 tumour proportion score (TPS) in 73.1% (30/41) and 78.9% (15/19) cases;>10% TPS in 24.4% (10/41) and 42.1%(8/19) respectively. High PD-L1 levels in OC correlated with poor response to neoadjuvant chemotherapy (CRS1).

**Conclusion**

PD-1 on lymphocytes and PD-L1 on cancer cells in effusion contribute to immune evasion and tumour progression. In ovarian carcinoma, high PD-L1 expression predicted for poor chemotherapy response making it an attractive immune-oncological therapeutic target.

### EACR2024-0710
### Deciphering cancer cell autonomous STING dependent tumor-promoting functions in KrasG12/Tp53ko mouse model of lung adenocarcinoma

*C. Ursino[1], S. Du Manoir[2], L. Gros[1], N. Bonnefoy[1], J. Faget[1]*
*[1]Institut de Recherche en Cancerologie de Montpellier, Immunity and cancer team, Montpellier, France*
*[2]Institut de Recherche en Cancerologie de Montpellier, Genetic and phenotypic plasticity of cancer, Montpellier, France*

**Introduction**

Canonical STimulator of INterferon Gene (STING) signaling leads to a potent induction of type I interferon (IFN) and interferon-stimulated genes orchestrating a robust immune response in various contexts. Yet, within the intricate landscape of cancer biology, STING's behavior remains enigmatic, with its impact on tumor dynamics appearing to be context dependent.

**Material and Methods**

To elucidate STING's function in lung cancer cells and interrogate cancer cell diversity, we performed STING knockout in KrasG12D;Tp53ko (KP)-derived NSCLC cells and generated cancer clones from KP-derived NSCLC cells.

**Results and Discussions**

Strikingly, STING depletion significantly hampered tumor formation in vivo and mitigated metastasis to mediastinal lymph nodes, without affecting immune surveillance, implying an intrinsic pro-tumoral role of the STING pathway. Single-cell RNAseq analysis of available datasets from KP tumors revealed fluctuation in STING expression across oncogenic stages, surging in advanced stages, suggesting a pivotal role in tumor development. Subsequent validation through in vitro experiments with subcloned KP-derived cancer cell reinforced this notion functionally, with mesenchymal-like cells exhibiting heightened STING expression that contributes to their migration ability. Interestingly, STING expression increased post-epithelial-to-mesenchymal transition (EMT) promoting stimuli, highlighting a link to cellular plasticity. Further dissection of STING signaling in mesenchymal-like cancer clones uncovered deviation in the canonical cascade involving TBK1 phosphorylation resulting in absence of IFN expression, while the latter can be induces through STING pathway independent stimulation, as TLR3-lignad. Moreover, exploration of S6 phosphorylation and LC3 II accumulation pointed out that the STING pathway in our model is associated with mTOR inhibition and autophagy induction and fatty acid oxidization. The higher ability of STING-expressing cells to recycle cell components conferred greater metabolic fitness and flexibility, confirmed by their higher spare respiratory capacity.

**Conclusion**

Collectively, our data suggest that in lung cancer, some transformed cells evade the STING pathway from its

Molecular Oncology 18 (Suppl. 1) (2024) 1–495
© 2024 The Authors. Molecular Oncology is published by John Wiley & Sons Ltd on behalf of Federation of European Biochemical Societies antiviral function and take advantage of its ability to drive greater metabolic adaptation and energy production via autophagy to fuel tumor growth. Thus, STING may play a key role in cancer cell plasticity, allowing them to form aggressive tumors and might directly contribute to disease progression in patients.

### EACR2024-0717

### TSG6 expression stimulated by M1 macrophages is associated with HAS2-mediated hyaluronan production in melanoma cells

K. Kainulainen[1], K. Koivusalo[1], K. Mäki-Mantila[1], S. Oikari[1], J. Hartikainen[2], K. Ketola[1], S. Pasonen-Seppänen[1]
[1]*University of Eastern Finland, Institute of biomedicine, Kuopio, Finland*
[2]*University of Eastern Finland, Clinical Pathology and Forensic Medicine, Kuopio, Finland*

**Introduction**

Inflammation is one hallmark of cancer – the reactive tumor microenvironment (TME) is maintained by pro- and anti-inflammatory cytokines secreted both by tumor and stromal cells. These inflammatory mediators drive oncogenic processes and eventually suppress immune responses against tumor. Macrophages are an abundant immune cell type in the inflammatory tumor micro-environment, where they have distinct anti- and protumor functions. Tumor necrosis factor -stimulated gene 6 (TSG6) is an anti-inflammatory protein that has also a role in matrix remodeling as it is involved in hyaluronan (HA) crosslinking. Our previous studies show that secreted factors from pro-inflammatory M1 macrophages stimulate hyaluronan synthesis in melanoma cells via *HAS2* upregulation. In this study, we investigated the role of TSG6 in macrophage-melanoma cell interactions and its effects in M1 macrophage -induced protumor inflammation.

**Material and Methods**

Peripheral blood mononuclear cells (PBMC) or THP-1 monocytes were differentiated to M0 macrophages. Thereafter, cells were polarized to M1 and M2 type macrophages with LPS and IFNγ, or alternatively with IL4 and IL13/dexamethasone. The effects of secreted factors from M0, M1 and M2 macrophages on the expression of TSG6 and its relation to pro-tumor inflammation in melanoma cells were studied with RNA-seq, chorioallantoic membrane assay, siRNA transfections, qPCR, western blot and immuno-fluorescence imaging.

**Results and Discussions**

Secreted factors from M1 macrophages (M1 CM) upregulated the expression of TSG6 in MV3 and G361 melanoma cells. Inhibition of NF-κB pathway down-regulated M1 CM-induced *TGS6* expression. *TSG6* silencing suppressed melanoma cell migration but promoted their proliferation in M1 CM exposed cells. Interestingly, *TSG6* silencing further upregulated M1 CM induced *HAS2* expression and HA synthesis while it downregulated the expression of several inflammatory factors such as *IL-1β* and *TNFα.*

**Conclusion**

Secreted factors from pro-inflammatory M1 macrophages stimulate TSG6 expression via NF-κB signaling that associates with *HAS2*-mediated hyaluronan production in melanoma cells. These results suggest that TSG6 controls inflammation-induced protumor effects in melanoma cells by extracellular matrix remodeling.

### EACR2024-0728

### POSTER IN THE SPOTLIGHT

### Preclinical Characterization of an Anti–HER2-STING Immune-Stimulator Antibody Conjugate in HER2+ Solid Tumor

W. Zhang[1], M. Li[2], Z. Tong[3], J. Ren[2], P. Qin[1], Y. Ru[2], X. Li[2], Y. Sun[4]
[1]*Shanghai De Novo Pharmatech- Co.LTD., Chemistry and ADC Research, Shanghai, China*
[2]*Shanghai De Novo Pharmatech- Co.LTD., Biology, Shanghai, China*
[3]*Shanghai De Novo Pharmatech- Co.LTD., Medicinal Chemistry, Shanghai, China*
[4]*Shanghai De Novo Pharmatech- Co.LTD., Management, Shanghai, China*

**Introduction**

Stimulator of Interferon Genes (STING) is a cytosolic protein critical for induction of type 1 IFN-dependent innate immunity. Immune-stimulating antibody conjugates (ISAC) combining tumor-targeting monoclonal antibodies with STING agonist allow localized delivery of immune activators into tumors, downstream priming T cells activation and resulting as well in a higher Therapeutic Index (TI) in the clinic. Here we describe DN028073, designed to selectively deliver our currently phase 1 clinical drug DN015089 (CTR20212462) to HER2+ cells through ADC format. This approach enabled systemic delivery and enhanced prolonged drug exposure to tumor microenvironment (TME).

**Material and Methods**

DN028073 (STING agonist conjugated to an anti-HER2 mAb by TME-sensitive cleavage linker) was evaluated in preclinical in vitro and in vivo systems to characterize potency, stability, Fc-related mechanism, and antitumor activity alone and in combination with a-PD-1 treatment.

**Results and Discussions**

A series of drug-linkers were synthesized and conjugated to the HER2-targeting antibody. HER2-mediated delivery of DN028073 triggered dose dependent activation of the STING signaling pathway and STING induced gene expression, as well as robust activation of innate and adaptive immune activity when co-cultured with the HER2-high expressing cancer cell line. Following systemic administration of DN028073 in preclinical syngeneic mouse studies, divers cytokines exposure was monitored in plasma and in tumor. Compared with intratumorally administration of DN015089, the expression of chemokines was prolonged, leading to better downstream T-cell activation. In CT-26 tumor bearing immunocompetent mice, treatment with DN028073 (1mg/kg) treatment alone achieved strong antitumor activity better than treatment with Enhertu ® (10mg/kg). When combined with a-PD-1 treatment,

© 2024 The Authors. Molecular Oncology is published by John Wiley & Sons Ltd on behalf of Federation of European Biochemical Societies better anti-tumor response has been achieved with a 60% CR rate. In pharmacokinetics and pharmacodynamics, DN028073 has a preferable PK profile and can activate immune system via in intravenous (i.v.) and subcutaneous (s.c.) administration.

**Conclusion**

Preclinical strong antitumor activity and favorable drug-like properties have motivated clinical testing of STING-ISAC for novel immunotherapies. The potential of other new target delivery and new combination therapies might provide new choice for locally advanced or metastatic solid tumors.

### EACR2024-0751
### Threonyl-tRNA Synthetase Editing Domain Mutation Promotes Incorporation of Mischarged Amino Acids: Implications for Neoantigen Formation and its Synergistic Potential with Immune Checkpoint Inhibitors

*B. Lee[1], D.H. Lee[1], S.M. Lee[1], K. Um[2], S. Kang[1], M.S. Kim[2], J.W. Choi[3]*

*[1]Kyung Hee University, Department of Biomedical and Pharmaceutical Sciences, Seoul, South Korea*

*[2]Kyung Hee University, Department of Pharmacology-Institute of Regulatory Innovation Through Science, Seoul, South Korea*

*[3]Kyung Hee University, College of Pharmacy, Seoul, South Korea*

**Introduction**

Microsatellite instability (MSI) serves as a predictive biomarker for the efficacy of immune checkpoint inhibitors (ICIs), with DNA mismatch repair inhibition shown to enhance ICI therapy by boosting neoantigen production. However, interventions at the DNA level come with systemic risks, highlighting the need for safer neoantigen induction techniques. Our study introduces a novel strategy of inducing mutations at the translation stage to generate a wide array of neoantigens, aiming to improve the treatment of solid tumors significantly.

**Material and Methods**

We introduced a D259A mutation in ThrRS, impairing its correction of mischarged Thr-tRNA and possibly causing alanine misincorporation. This was cloned into pcDNA3.1-6X His and overexpressed in HCT116 and H460 cells to assess its impact on protein synthesis and neoantigen production, using GFP co-transfection, western blot, translation rate, and ER stress evaluations. Neoantigen formation was analyzed via MHC class I immunoprecipitation and mass spectrometry. The study explores the mutation's influence on immune response and cancer treatment, observing enhanced ICI synergy in a tumor-bearing mouse model using ELISPOT assay and immunofluorescence.

**Results and Discussions**

Using GFP co-transfection and western blotting, we evaluated expression and protein processing changes. Increased ER stress and altered protein folding were observed, likely due to misincorporation of alanine instead of threonine. MHC class I immunoprecipitation and MS/MS analysis showed a 10% increase in neoantigen presentation, with 5% of threonine residues replaced by alanine, enhancing immune recognition. In a mouse model with B16 cells expressing mutated ThrRS (D258A, analogous to the D259A mutation in humans), we found increased neoantigen-specific T cells via IFN-gamma ELISPOT and a significant tumor size reduction after PD-1 antibody treatment. Immunofluorescence of tumors showed more infiltrating T cells, indicating enhanced immune response.

**Conclusion**

Our study shows ThrRS mutations, turning threonine to alanine, create many tumor-specific neoantigens, boosting ICI effectiveness and suggesting a synergistic tumor-fighting strategy. This supports merging neoantigen induction with immunotherapies, offering a new cancer treatment approach to potent therapies by enhancing immune responses with neoantigens.

### EACR2024-0765
### Phenotypic Plasticity of Breast Cancer Cells Regulates Antitumoral Immunity

*A. Peura[1], R. Turpin[1], R. Liu[1], M. Heilala[2], J. Aung[1], P. Mikkonen[3], J. Klefström[1], P. Munne[1]*

*[1]University of Helsinki, Cancer Cell Circuitry Laboratory- Research Programs Unit- Faculty of Medicine, Helsinki, Finland*

*[2]Aalto University, Department of Applied Physics, Espoo, Finland*

*[3]UPM-Kymmene Corporation, UPM Biomedicals, Helsinki, Finland*

**Introduction**

Traditionally tumor tissue is has been considered as stiff, and the stiffness of the tumor microenvironment has been established to promote the progession of several cancer types. Recent research has also emphasized the significant role of the local softening of the tumor micro-environment on tumor cell phenotype. Tumor cells may encounter more soft microenvironment e.g. while metastasizing or as a result of intratumoral necrosis. Breast cancer cells embedded in the soft matrix have been shown to dedifferentiate into an undifferentiated, estrogen receptor negative phenotype, while upregulating pathways associated to treatment resistance. However, while emerging evidence suggests that the softening of the matrix has a major impact on tumor cell identity and therapy resistance, little is known how softening of the matrix impacts on the antitumor immunity.

**Material and Methods**

In this study we utilize the Patient Derived Explant Model (PDEC) that has been shown to preserve the vitality and function of both, patient derived epithelial tumor tissue and tumor infiltrating leucocytes (TILs) ex vivo. By altering the stiffness of the scaffold matrix surrounding tumor tissue in PDEC, we show how local softening of the matrix alters the phenotype of the breast tumor cells and eventually TILs. In parallel, we use fresh patient derived mononuclear cells in similar culture conditions to define direct matrix induced effects. We characterize the alterations in cellular phenotype and cytokine environment with techniques such as ELISA cytokine assay, protein analysis and single cell analysis.

**Results and Discussions**

We show that in the soft microenvironment the luminal breast cancer cells undergo a dedifferentation, resulting a

© 2024 The Authors. Molecular Oncology is published by John Wiley & Sons Ltd on behalf of Federation of European Biochemical Societies less differentiated and more progenitor like phenotype. The dedifferentiated tumor cells embedded in the soft microenvironment induce change in their local microenvironment, by upregulating a production of several immunosuppressive cytokines and polarization of tumor resident macrophages to more M2-like.

**Conclusion**

In this study we show how local stiffness induced changes in the tumor cell phenotype result in an immunosuppressive microenvironment. Furthermore, we propose a novel pathway that might be responsible for these alterations.

### EACR2024-0780

### Effect of ERAP1 modulation on the induction of protective anti-tumour immunity

*D. Guglietta[1], S. Alvarez Mourid[1], E. Reeves[1], E. James[1]*

*[1]University of Southampton, Cancer sciences, Southampton, United Kingdom*

**Introduction**

ERAP1 trimming of antigenic peptides has an important role in anti-cancer immune responses, by generating optimal peptides for MHC-I loading and presentation to CD8+ T cells. Previous studies reveal that ERAP1 overexpression in colon carcinomas results in over-trimming and destruction of strong cancer-related antigens, such as GSW11. Moreover, ERAP1 knockdown by RNAi induces strong anticancer immune reactions against CT26 colon carcinoma cells in vivo and stimulates an immunological memory. We aim to investigate if ERAP1 knock-out increases the immunogenicity of MC38 cells in vivo and determine the effects of expressing different human ERAP1 allotypes in MC38 cells.

**Material and Methods**

We established an ERAP1 knock-out MC38 colon carcinoma cell line using a CRISPR-Cas9 approach, confirming ERAP1 knock-out by PCR, Sanger Sequencing and Western Blot following IFNγ stimulation. Different ERAP1 haplotypes were introduced into ERAP1 KO cells using MSCV viral vector and confirmed by western blot. MHC I stability was analysed in vitro with BFA treatment and Flow cytometry. The MC38 cells expressing or not different ERAP1 haplotypes were then injected in the subcute of syngeneic mice (C57BL/6), tumour growth kinetic and mice survival were then measured. Tissue samples (tumour, spleen and draining lymphnode) were analyzed by flow cytometry to identify the number and phenotype of immune cells present.

**Results and Discussions**

The absence of ERAP1 reduces the stability of H-2Kb but not H-2Db complexes, with ERAP1 re-expression recovering H-2Kb stability. In vivo experiments conducted on syngeneic mice injected with wild-type or ERAP1 knock-out MC38 cells indicate that the absence of ERAP1 expression results in slower tumour growth and increased survival. The analysis of tumour immune infiltrate identifies an increase in CD4+ T cell infiltrate and an increase in both CD4+ and CD8+ activated and effector memory subsets in ERAP1 knock-out tumours compared to wild-type tumours. Mice surviving the first challenge with ERAP1 KO cells rechallenged with wild-type MC38 cells reject the tumour.

**Conclusion**

These data suggest that ERAP1 knock-out increases MC38 cells' immunogenicity in vivo and leads to the establishment of antitumour immunological memory.

### EACR2024-0812

### Characterisation of circulating and tissue-resident CD8 T cell paths towards dysfunction in lung cancer

*C. Metoikidou[1,2,3], P.E. Bonté[3], C. Goudot[3], M. van Ingen[1,2], A. Seguin-Givelet[4], N. Girard[4], D. Thommen[1,2], S. Amigorena[3]*

*[1]The Netherlands Cancer Institute, Division of Molecular Oncology and Immunology, Amsterdam, The Netherlands*

*[2]Oncode Institute, Oncode, Utrecht, The Netherlands*

*[3]Institut Curie, Inserm U932- Immunity and Cancer, Paris, France*

*[4]Institut du Thorax Curie-Montsouris - IMM, Thoracic Surgery Department, Paris, France*

**Introduction**

In the tumor microenvironment (TME), due to continuous antigen presentation, CD8 T cells become dysfunctional. T cell dysfunction contains a gradient, from early- to late-dysfunctional states. Recently, we characterised two distinct CD8 T cell subpopulations expressing memory-like gene modules in lung cancer of different origin: one being circulating and the other one being tissue-resident. In tumors, these two precursor subsets both give rise to dysfunctional cells. These results provided a model for CD8 T cell origin, ontogeny, and organization in lung cancer. Of note, the function of these states and their antitumor properties remain unclear. Therefore, protein markers that allow to isolate and functionally assess these states are important.

**Material and Methods**

To address these questions, we identified differentially expressed genes in scRNAseq data per cell state. We selected a set of surface markers allowing us to distinguish circulating, tissue-resident precursors and late-dysfunctional CD8 cells. We performed bulk RNA-seq and flow cytometry analysis on sorted CD8 T cell subsets, from lung tumor and juxta tumor tissues. In parallel, we performed ex vivo culture of patient-derived tumor fragments and cytokine profiling on collected supernatants to functionally assess these subsets.

**Results and Discussions**

Bulk RNAseq analysis validated the proposed gating strategy for sorting of circulating, tissue-resident precursor and late-dysfunctional CD8 T cells. Comparison of the precursor populations in juxta tumor unveiled that tissue-resident cells exhibit an increased activation profile compared to circulating cells, that is similar to the late-dysfunctional phenotype intra-tumorally. Flow cytometry analysis unveiled distinct functional characteristics among subsets, with late-dysfunctional CD8 T cells upregulating effector and cytotoxic as well as cell cycle markers. Cytokine profiling analysis showed that enrichment in late-dysfunctional CD8 T cells correlates with elevated immunological activity in the TME.

© 2024 The Authors. Molecular Oncology is published by John Wiley & Sons Ltd on behalf of Federation of European Biochemical Societies

**Conclusion**
Here, we developed a method for isolation of circulating, tissue-resident precursor and late-dysfunctional CD8 T cell subsets from human lung tumors. Our analysis unveils distinct expression patterns of markers associated with T cell cytotoxicity and tumor-reactivity across these CD8 states. Moreover, it sheds light into their association with the baseline immunological activity, and therefore could be useful for patient stratification strategies and biomarkers design for response prediction.

### EACR2024-0815
### Combining intra-tumor and systemic neopeptide vaccination augments anti-tumor immunity in a murine tumor model

*K. Hioki[1], M. Castro Eiro[1], K. de Vos[1], M. Wildemans[1], D. Grashof[1], Y. Lim[1], H. van de Werken[1], K. Ishii[2], P. Katsikis[1]*
*[1]Erasmus University Medical Center, Department of Immunology, Rotterdam, Netherlands Antilles*
*[2]The Institute of Medical Science- The University of Tokyo, Division of Vaccine Science- Department of Microbiology and Immunology- International Vaccine Design Center, Tokyo, Japan*

**Introduction**
Neopeptide vaccines have emerged as one of the promising strategies to treat cancers. Neopeptide vaccines are able to induce neopeptide-specific T cell responses, however there still remains a need to improve their efficacy. We have previously shown that our neopeptide vaccine containing a TLR9 and STING adjuvant combination systemically induced potent T cell responses in vivo; however, the systemic vaccines were not sufficient to control tumor growth in the AE17 mesothelioma tumor model. This may be due to the tumor microenvironment of AE17 tumors where systemic vaccine-induced T cells are not recruited or suppressed. Therefore, we aim to augment the anti-tumor T cell immunity by combining with an intra-tumor neopeptide vaccination, which might recruit and activate systemic vaccine-induced T cells in a tumor tissue.
**Material and Methods**
To examine the efficacy of the combination of systemic and intra-tumor neopeptide vaccines, AE17 tumor-bearing mice were subcutaneously immunized with the adjuvanted vaccines containing neopeptides identified from AE17 tumors. Some mice also received intra-tumor neopeptide vaccination once a week, four times. Tumor growth and survival rate were monitored, and tumor tissues and tumor-draining lymph nodes were analyzed using spectral flow cytometry and immunofluorescence staining.
**Results and Discussions**
We found that the combination of systemic plus intra-tumor neopeptide vaccines suppress tumor growth in AE17 tumor-bearing mice. Importantly, mice received intra-tumor vaccine alone or intra-tumor administration of neopeptides only or adjuvants alone combined with systemic vaccinations also failed to control tumor growth, suggesting that it is crucial to perform both systemic and intra-tumor vaccination as a combination to treat tumors. Moreover, we found that mice receiving the vaccine combination developed sterile (necrotic) ulcers on tumors, suggesting the capacity to collapse tumor tissues. Finally, we revealed that the vaccine combination increased recruitment of immune cells such as T cells into tumor tissues.
**Conclusion**
Our findings demonstrate that the combination of systemic and intra-tumor vaccination with a neopeptide vaccine containing TLR9 and STING adjuvants is able to treat cold tumors such as the AE17 mesothelioma and can serve as a novel cancer vaccine strategy.

### EACR2024-0830
### IFN-I and epigenetics behind tumor immunology

*A. Sistigu[1], M. Musella[1], N. Manduca[1], E. Maccafeo[1], P. Trono[2], R. De Maria[1]*
*[1]Università Cattolica del Sacro Cuore, Translational Medicine and Surgery, Rome, Italy*
*[2]National Research Council, Biochemistry and Cell Biology, Rome, Italy*

**Introduction**
Immunogenic- and immune-therapies have become hot spots in the treatment of cancer. Although promising, these strategies are frequently associated with innate or acquired resistance, calling for combined targeting of immune inhibitory signals. Type I interferons (IFN-I) are emerging as crucial factors able to mold the state and fate of cancer and immune cells in the tumor micro-environment through epigenetic dysregulations. In this setting, epigenetic therapy is attracting unprecedented attention as a combination partner for immunogenic- and immune-therapies to convey therapeutic benefit with significant breakthroughs on patients' quality of life.
**Material and Methods**
Human and murine cancer cell lines and tumor-specific lymphocytes were treated in vitro and in vivo with immunogenic drugs or IFN-I and the emergence of IFN-related epigenetic reprogramming was analyzed by flow cytometry, sc-omics and Tumor-on-a-Chip models. All experiments have been done in triplicate and statistical significance evaluated by two-tailed Student's t test and one-way ANOVA.
**Results and Discussions**
Suboptimal induction of cancer cell death by immunogenic chemotherapy triggers an acute IFN-I response that fails to elicit anticancer immunity and instead drives stemness, therapeutic-resistance and immune-evasion through the IFN-I-stimulated gene lysine demethylase 1B (KDM1B). KDM1B is an epigenetic regulator which erases mono- and dy-methyls on histone H3 at lysine 4 (H3K4me1 and H3K4me2) in both residual cancer cells and tumor-infiltrating immune cells.
**Conclusion**
Results from our study will shed light on detrimental nuclear reprogramming downstream of IFN-I as a novel mechanism of acquired resistance and cancer immuno-editing and will guide the development of personalized precision combined epigenetic/immune-based therapies to counteract cancer immune escape and impede tumor progression/recurrence.

Molecular Oncology 18 (Suppl. 1) (2024) 1–495
© 2024 The Authors. Molecular Oncology is published by John Wiley & Sons Ltd on behalf of Federation of European Biochemical Societies

### EACR2024-0859
### The emerging crucial role of gut-microbiota composition in pediatric brain tumors

C. Dossena[1], L. Bergamaschi[2], F. Rossignoli[1], A.G. Licata[1], M. Oriani[1], M. Marra[3], M. Carollo[3], M. Massimino[2], L. De Cecco[1]
[1]Fondazione IRCCS Istituto Nazionale dei Tumori, Integrated Biology of Rare Tumors Unit, Milan, Italy
[2]Fondazione IRCCS Istituto Nazionale dei Tumori, Pediatric Oncology Unit, Milan, Italy
[3]Istituto Superiore di Sanità, Core Facilities Technical-Scientific Service FAST, Rome, Italy

**Introduction**

Central nervous system (CNS) tumors represent the main cause of cancer-related deaths in children diagnosed at 0–14 years. It has been demonstrated that the bidirectional interaction between gut microbiota and brain, called microbiota-gut-brain axis, has a key role in patho-physiological processes, mediating proliferation of brain tumor cells. The aim of this study was to determine whether the gut microbiota composition of pediatric patients affected by CNS tumors has peculiarities.

**Material and Methods**

We included 25 pediatric patients from a mono-institutional clinical study (INT77/20) that were divided in two groups: 11 affected by SNC tumors (group 1) and 14 affected by other tumor types (group 2), non related to CNS. From their stool samples, microbial DNA was extracted, 16S DNA libraries were generated and sequenced on IONS5xl (ThermoFisher). From the resulting operational taxonomic unit (OTU) tables, the composition of gut-microbiota was analyzed.

**Results and Discussions**

To determine the richness of gut microbiota in terms of different taxa, a-diversity analysis was performed. A data matrix was obtained from the observed features and a-diversity was almost significantly lower in group 1 than in group 2 (p-value= 0.07); then, the homogeneity of taxonomic groups was determined by Pielou's evenness index, resulting higher in group 1 (p-value =0.037) than in group 2. β-diversity measured with weighted UniFrac metrics revealed that the taxonomic and phylogenetics composition of gut microbiota is different between group 1 and 2 (p-value= 0.028). Finally, linear discriminant analysis effect size (LEfSe) revealed that *Betaproteobacteria* class, *Burkholderiales* order, *Alcaligenaceae* an d *Porphyromonadaceae* families, and *Sutterella* and *Parabacteroides* genus are enriched in group 1. Conversely, the levels of *Firmicutes* phylum, *Clostridia* and *Coriobacteriia* classes, *Clostridiales* and *Coriobacteriales* orders, *Lachnospiraceae* and *Coriobacteriaceae* families, and *Balutia* genus are decreased in group 1 compared to group 2. The reduction in observed Amplicon Sequences Variants (ASVs) coupled with an elevated evenness, among pediatric patients with CNS tumors, suggests that the growth of few specific microbial taxa is favored by CNS tumor, probably because some microbial metabolites sustained tumoral growth through gut-microbiota-brain axis.

**Conclusion**

In conclusion, the particular gut microbiota composition of pediatric patients affected by CNS tumors could have a key role in sustaining pro-tumoral microenvironment.

### EACR2024-0870
### CDCP1 overexpression in urothelial carcinoma enhances Tumor-Associated Macrophage Recruitment by IL6 and CCL2 production

M. Saponaro[1], M. Eckstein[2], A. Winkler[3], D. Schmidt[3], K. Wörsdörfer[3], N. Klümper[3], M. Ritter[3], M. Hölzel[4], A. Alajati[3]
[1]Universitätsklinikum Bonn, Department of Urology and Pediatric Urology, Bonn, Germany
[2]University of Erlangen–Nuremberg, Pathology, Erlangen–Nuremberg, Germany
[3]University Hospital Bonn, Urology and Pediatric Urology, Bonn, Germany
[4]University Hospital Bonn, Institute of Experimental Oncology, Bonn, Germany

**Introduction**

CUB domain-containing protein 1 (CDCP1) is a transmembrane protein that exerts a pro-tumoral effect in various tissues. A previous study demonstrated the oncogenic role of CDCP1 in urothelial cancer (UC). Notably, CDCP1 was mostly expressed in advanced stages, where a higher immune cell infiltration is also observed. Hence, this study aims to define the interplay between tumor-associated macrophages (TAM) and tumor cells expressing CDCP1.

**Material and Methods**

First, a tumor-microarray (TMA) counting 241 muscle-invasive UC patients was analyzed for CDCP1, CD68, and CD163 expression. To perform functional experiments we induced CDCP1 knock-out (KO) in 2 UC cell lines (T24 and TCCSUP) with CRISPR/Cas9. mRNA sequencing was performed on CDCP1-expressing and -KO cells and cytokine levels were assessed with LEGENDplex™ on their conditioned media (CM) before testing its effect on the migration of peripheral blood mononuclear cell-derived macrophages. Results were confirmed with an ex-vivo bladder organoid model obtained from loxP-STOP-loxP-*CDCP1* transgenic mouse via transduction with adenoviruses encoding for CRE recombinase. Finally, cytokine production was tested upon therapeutic anti-CDCP1 antibody treatment in UC cells.

**Results and Discussions**

TMA analysis revealed that CDCP1 expression positively correlates with macrophage markers CD68 and CD163, suggesting that UC tumors overexpressing CDCP1 present higher TAM infiltration. Next, cytokines were quantified in the CM of UC cells and relative CDCP1-KOs to study whether CDCP1-expressing tumor cells are responsible for macrophage recruitment. CM collected from CDCP1-expressing cells showed higher CCL2 and IL6 levels and induced higher macrophage migration. The murine organoids provided further confirmation since the CDCP1-overexpressing ones showed higher IL6 and CCL2 production and their CM enhanced macrophage migration. mRNA sequencing was performed on CDCP1-expressing and -KO cells to investigate the

© 2024 The Authors. Molecular Oncology is published by John Wiley & Sons Ltd on behalf of Federation of European Biochemical Societies cytokine production mechanism. Of note, CDCP1-KO cells expressed higher interleukin-1 receptor-associated kinase 3 (IRAK3), which plays a suppressive role in inflammation. In line with this finding, UC cells treated with anti-CDCP1 antibodies showed impaired IL6 and CCL2 production.

**Conclusion**

This study demonstrates that TAMs benefit from the inflammatory environment accompanying CDCP1 overexpression in UC and that CDCP1 could work as a target for reducing TAM recruitment.

### EACR2024-0882
### Enhancing Anti-PD1 Immunotherapy and Prognosis in Melanoma with the PRIME Model

*S. Koskela[1,2], O. Pulkkinen[1], A. Mehmood[1], E.M. Birkman[3], S.E. Coupland[4], T. Aittokallio[2,5,6,7,8,9], M. Sundvall[5], C.R. Figueiredo[1,2]*

*[1]University of Turku, Medical Immuno-Oncology Research Group- Cancer Research Unit- Institute of Biomedicine, Turku, Finland*

*[2]University of Turku, InFlames Research Flagship, Turku, Finland*

*[3]Turku University Hospital and University of Turku, Department of Pathology- Laboratory Division, Turku, Finland*

*[4]University of Liverpool, Liverpool Ocular Oncology Research Group- Department of Clinical Cancer Medicine- Institute of Translational Medicine, Liverpool, United Kingdom*

*[5]University of Turku, Cancer Research Unit- Institute of Biomedicine- and FICAN West Cancer Centre Laboratories, Turku, Finland*

*[6]University of Oslo, Centre for Biostatistics and Epidemiology OCBE- Faculty of Medicine, Oslo, Norway*

*[7]Oslo University Hospital, Department of Cancer Genetics- Institute for Cancer Research, Oslo, Norway*

*[8]University of Helsinki and Helsinki University Hospital, iCAN Digital Precision Cancer Medicine Flagship, Helsinki, Finland*

*[9]University of Helsinki, Institute for Molecular Medicine Finland, Helsinki, Finland*

**Introduction**

Immune checkpoint therapies (ICT) have provided remarkable improvements in the treatment and clinical response of solid tumors. However, most metastatic melanoma patients develop resistance to anti-PD1 immunotherapies because of ‘cold tumor’ formation, characterized by lack of CD8+ T cells. Currently, there are no effective tools to predict the development of cold tumors and subsequent lack of response to ICT. Therefore, there is a persisting clinical need for accurate prognostic models and discovery of functional drivers of cold tumors.

**Material and Methods**

We developed PRIME, an anti-PD1 prognostic model based on a new integrative Systems Immunology approach. PRIME framework discovers targets related to efficient antigen-processing and presentation (APP) and priming of CD8+ T cells in the whole transcriptome of four integrated ICT-treated metastatic melanoma cohorts (272 patients) using bioinformatic methods. The PRIME model was developed by identifying the prognostic targets using logistic regression on the integrated ICT-treated metastatic melanoma cohorts. To identify APP-related immune-enriched potential therapeutic targets, the PRIME framework biomarkers were clustered based on their functional characteristics and analyzed for their expression patterns using new spatial transcriptomics and previously-published single-cell RNA sequencing cohorts of metastatic melanoma. The novel roles of the targets in APP were validated with RNA interference and functional experiments of antigen processing and presentation.

**Results and Discussions**

PRIME prognostic model outperforms (Area under the curve (AUC) 0.73) existing models (IMPRES AUC 0.56, TIDE AUC 0.65) in predicting RECIST responses to anti-PD1 immunotherapies in cutaneous melanoma. The spatial transcriptomics of skin and lymph node metastases and single-cell profiling identified PRIME biomarkers essential for immunogenic functions of dendritic cells (DCs) and CD8+ T cell activation. The functional studies revealed novel roles of the PRIME biomarkers in antigen processing and presentation in myeloid DCs.

**Conclusion**

These findings exposed new pathways and biomarkers that are involved in immunogenicity through antigen presentation and subsequent response to anti-PD1 immunotherapies. In summary, we present a novel method for rational biomarker and therapeutic target discovery using an integrative systems immunology approach and a powerful immunotherapy prognostication model, PRIME.

### EACR2024-0899
### Understanding the cytokine topography in immunomodulation by Tumor Extracellular Vesicles (TEVs) in the context of Pleural Mesothelioma

*E.J. Herman[1], C. Di Primio[2], J. Kopecka[3], G.P. Caviglia[4], C. Rosso[4], M. Sculco[5], I. Dianzani[5], C. Riganti[3], G. Matullo[6], E. Casalone[6]*

*[1]University of Turin, Unit of Genomic Variability and Complex Diseases- Department of Medical Sciences, Turin, India*

*[2]University of Turin, Unit of Genomic Variability and Complex Diseases- Deptartment of Medical Sciences, Turin, Italy*

*[3]University of Turin, Department of Oncology, Turin, Italy*

*[4]University of Turin, Division of Gastroenterology and Hepatology- Department of Medical Sciences, Turin, Italy*

*[5]University of Eastern Piedmont, Department of Health Sciences, Novara, Italy*

*[6]University of Turin, Unit of Genomic Variability and Complex Diseases- Department of Medical Sciences, Turin, Italy*

**Introduction**

Pleural Mesothelioma (PM) is an aggressive, rare form of cancer of the pleural compartment which is mainly caused by exposure to asbestos. Currently, there is no set

© 2024 The Authors. Molecular Oncology is published by John Wiley & Sons Ltd on behalf of Federation of European Biochemical Societies way to diagnose or treat PM and thus it is imperative to identify potential immunomodulatory factors to improve the diagnosis and prognosis. Our research aims to characterise the role of tumor derived EVs in inducing immune cell suppression.

**Material and Methods**

EVs derived from Met5a and MSTO have been co-cultured with PBMCs from healthy donors. The expression of cytokine molecules in conditioned medium has been evaluated using the Bio-Plex Pro Human Cytokine 27-Plex Immunoassay kit (Bio-Rad Laboratories, Hercules, CA, USA).

**Results and Discussions**

Most of the cytokines levels measured in the PBMCs treated with EVs from MSTO cell line revealed an increased concentration of G-CSF, GM-CSF, IFN-γ, IL-5, IL-6, IL-8, IL-9, IL-10, TNF-α, and VEGF compared to PBMCs treated with EVs from the normal mesothelium cell line, while on the other hand IL-2 concentration was seen to be diminished in PBMCs treated with EVs from MSTO compared to EVs from Met5A.

**Conclusion**

Tumor microenvironment derived immunosuppression is often induced by factors such as VEGF and IL-10 due to specific ligands expressed on the tumor cells suggesting a possible involvement of multiple cellular networks forming a convergence and subsequently leading to PM immunotolerance. Inhibition studies of GM-CSF in preclinical models of various types of cancers have been shown to increase intratumoral T cells and control tumor growth especially when combined with anti-CTLA-4 or anti-PD-1/PD-L1. Interactions between malignant cells, stromal cells, ECM components, various inflammatory cells, and a range of soluble mediators contribute to tumour development and progression. Therefore, analysing cytokine profiles coming out of these diverse interactions may uncover new therapeutic targets for mesothelioma immunotherapy and shed light on the tumor-immune axis.

### EACR2024-0902

### TYK2 inhibition causes not only apoptosis but also increased anti-tumor immune response in Anaplastic Large Cell Lymphoma

*C. Giordano[1], M. Bećirović[1], J. Leitner[2], P. Steinberger[2], L. Kenner[1,3,4], O. Merkel[1]*

*[1]Medical University of Vienna, Department of Pathology, Vienna, Austria*

*[2]Medical University of Vienna, Institute of Immunology, Vienna, Austria*

*[3]Medical University of Vienna, Christian Doppler Laboratory CDL for Applied Metabolomics, Vienna, Austria*

*[4]University of Veterinary Medicine of Vienna, Unit of Laboratory Animal Pathology, Vienna, Austria*

**Introduction**

Anaplastic Large Cell Lymphoma (ALCL) is an aggressive, CD30+ T-cell malignancy. Half of the patients carry the t(2;5) translocation, resulting in the oncogenic NPM-ALK gene product. Less is known about the driving factors in ALK negative (ALK-) patients. We demonstrated previously that the tyrosine kinase TYK2 is a dependency factor in ALCL, regardless of ALK status, suggesting TYK2 inhibitors as therapeutic option. However, TYK2 deletion has been shown to lead to reduced anti-tumor immune response in mice, questioning the benefit of systemic TYK2 inhibition. Since it has been shown that immune surveillance is active in ALCL, as demonstrated by infiltration of CD8+, Foxp3+ T-cells and CD163+ macrophages, elucidating the prevailing mechanism of TYK2 involvement in ALCL is highly warranted.

**Material and Methods**

We manipulated TYK2 activity in ALCL cell lines using the clinically approved TYK2 inhibitor Deucravacitinib. In addition, we generated a doxycycline-inducible shRNA TYK2 knock-down for in vitro use but also for murine engraftment models. Coculture systems were developed with freshly isolated and differentiated human macrophages and ALCL cell lines. This allowed us to study ALCL cell-induced macrophage polarization with or without Deucravacitinib. Also, transgenic T-cell receptor activation GFP-reporter cell lines were used to study PD-L1 dependent anti-tumor immune response.

**Results and Discussions**

Deucravacitinib led to reduction of viability and induced apoptosis in both ALK+ and ALK- ALCL cell lines and reduced phosphorylation of the downstream effectors STAT1 and STAT3. In ALK- cell lines TYK2 inhibition caused decreased PD-L1 expression, resulting in increased T-cell receptor activation as measured by a GFP-reporter cell line, while PD-L1 expression was upregulated in ALK+ ALCL. In coculture systems, Deucravacitinib completely blocked ALCL-driven polarization of macrophages to a CD163+ pro-tumorigenic phenotype mediated by interleukin-10.

**Conclusion**

We here show that Deucravacitinib induces apoptosis in ALCL, ALK- cells and concomitantly evokes an antitumor immune response by reducing PD-L1 expression. Moreover, Deucravacitinib abrogated tumor cell-induced polarization of pro-tumorigenic macrophages. This suggests Deucravacitinib as drug candidate in ALK- ALCL patients. In contrast, in the ALCL, ALK+ situation Deucravacitinib could be combined with anti-PD1 checkpoint inhibitors which will be tested by engrafting murine NPM-ALK transgenic cell lines in syngeneic C57BL/6 mice.

### EACR2024-0925

### Integrated RNA and protein profiling in the same tissue section: deciphering tumor microenvironment dynamics via automated spatial multiomics analysis

*A. Failletaz[1], A. Comberlato[1], A. Manoukian[1], P. Juričić[1], P. Bordignon[1], A. Dikshit[2], R. Delvillar[2], S. Zhou[2], L.C. Wang[2], S. Brajkovic[1]*

*[1]Lunaphore Technologies, Application Development, Tolochenaz, Switzerland*

*[2]Advanced Cell Diagnostics, Application Development, Newark, United States*

**Introduction**

© 2024 The Authors. Molecular Oncology is published by John Wiley & Sons Ltd on behalf of Federation of European Biochemical Societies

Spatial biology techniques have revolutionized our understanding of the tumor microenvironment (TME) and its intricate cellular interactions. Multiplex immunofluorescence (mIF) methods enable precise profiling of immune cells and other key players within the TME, revealing their spatial distribution and interactions (PMID: 38012408). In situ hybridization (ISH) technologies complement protein profiling by mapping cytokine- and chemokine-expressing cells, crucial for deciphering signaling networks and immune activation.

**Material and Methods**

Here, we present a novel multiomics approach that integrates RNAscope™ and sequential immuno-fluorescence (seqIF™) protocols. This innovative workflow achieves the same-section co-detection of RNAs and proteins within the TME. The process is automated using COMET™, an advanced tissue staining and imaging platform. By precisely controlling temperature and reagent distribution, COMET™ ensures maximum assay efficiency and reproducibility. Our integrated multiomics protocol allows up to three RNAscope™ detection cycles combined with twelve seqIF™ cycles, resulting in a final 12-plex RNA and 24-plex protein panel.

**Results and Discussions**

In our study, we harnessed the power of the COMET™ platform to automate the RNAscope™ protocol. By analyzing positive and negative control genes, we validated their sensitivity and specificity. To explore the intricate landscape of the tumor microenvironment (TME), we developed a panel of 12 probes targeting crucial RNA biomarkers in tumor-infiltrating lymphocytes and their activation status. Simultaneously, we employed a 24-antibody panel to detect protein biomarkers, enabling single-cell profiling within the TME. Applying this approach to human FFPE tumor tissues, we demonstrated that co-detection of RNA and protein biomarkers in the same section enhances our understanding of key cellular components involved in tumor progression and immune response. This integrated approach promises deeper insights into cancer biology.

**Conclusion**

Our findings underscore the promise of spatial multiomics technologies in advancing immune cell research and unraveling intricate cellular interactions within TME. By fully automating these technologies on platforms like COMET™, we enhance efficiency, reduce user interventions, and increase robustness. This progress has important implications for creating predictive markers, refining cancer diagnoses, and tailoring personalized therapies.

### EACR2024-0943

### The role of OSM/OSMRβ axis in shaping the immune-microenvironment favouring MASLD-Related HCC immune evasion

*B. Foglia[1], J. Nurcis[1], S. Sutti[2], C. Rosso[3], G. Caviglia[3], L. Altucci[4], U. Chianese[4], E. Bugianesi[3], M. Parola[1], S. Cannito[1]*

[1]*University of Torino, Dept. Clinical and Biological Sciences, Torino, Italy*

[2]*University Amedeo Avogadro of East Piedmont, Dept. Health Sciences and Interdisciplinary Research Center for Autoimmune Diseases, Novara, Italy*

[3]*University of Torino, Dept. Medical Sciences, Torino, Italy*

[4]*University of Campania "Luigi Vanvitelli", Dept. Precision Medicine, Napoli, Italy*

**Introduction**

Oncostatin M (OSM), a cytokine from the IL-6 family, is implicated in chronic liver diseases and hepatocellular carcinoma (HCC) progression. HCC patients with a background of metabolic dysfunction-associated steatotic liver disease (MASLD) showed elevated OSM serum levels correlating with clinical parameters and disease outcome. This study explores OSM's potential role in modulating crucial events in HCC progression including the immunesuppressive tumor microenvironment (TIME).

**Material and Methods**

To test this hypothesis, we used: a)hepatocyte-OSMRβ-/- mice and wild-type (WT) littermates that underwent the DEN/CDAA protocol of MASLD-related liver carcino-genesis to assessing how disrupting OSM/OSMRβ signaling in hepatocytes influences MASLD-related HCC development and progression; b)cohort of MASLD patients with/without HCC.

**Results and Discussions**

OSMRβ-/- mice exhibited a significant decrease in tumor volumes and weights compared to WT,with unchanged nodule numbers. In terms of inflammation,OSMRβ deletion did not alter the infiltration of F4/80+ cells suggesting OSM's potential role in qualitatively modifying the immune landscape. Analysis of HCC data from the TGCA database, revealed that OSM expression correlates positively with several immunosuppressive markers (CCL22, FOXP3, PD-L1,PTGSE2, TGFβ). Consistently, OSM transcripts levels in WT correlates with these markers and their expression is downregulated in the OSMRβ-/- mice as a consequence of the inhibition of signaling pathways involved in recruiting pro-tumoral Tumor Associated Macrophages (TAMs),T regulatory lymphocytes (T-regs) and Myeloid-derived suppressor cells (MDSCs). Multiplex Immunoassay on sera from MASLD cirrhotic and/or HCC patients revealed a significant increase of cytokines characterizing the TIME (IL1β, CCL2, IL8, CXCL13). Transcript levels of these cytokines correlated with OSM expression in MASLD patients. Accordingly, OSMRβ deletion resulted in reduced transcript levels of these genes. Of relevance, CCL15 (a chemokine downregulated in the OSMRβ-/- mice and involved in TAMs and MDSCs recruitment and activation) was found higly expressed by RNA-seq analysis in HCC patients, correlated positively with OSM mRNA levels in MASLD-related HCC patients and resulted as an independent prognostic factor associated with worse survival.

**Conclusion**

The experimental data highlight a pro-carcinogenic contribution for OSM in a MASLD background, favoring TIME, suggesting a possible role for the OSM-OSMRβ axes as therapeutic target for MASLD-related HCC.

### EACR2024-0944

### 3D Co-Cultures of Human-Derived Cancer

© 2024 The Authors. Molecular Oncology is published by John Wiley & Sons Ltd on behalf of Federation of European Biochemical Societies

### Cells and Fibroblasts for Evaluation of Cancer Therapies

*N.H. Madsen[1], B.S. Nielsen[1], I. Skandorff[1], B. Schmid[1], K. Holmstrøm[1], J. Larsen[1], M. Gad[1]*
*[1]Bioneer A/S, Cellular Engineering and Disease Models, Hørsholm, Denmark*

**Introduction**
Advancements in 3D cancer cell cultures have revolutionized preclinical drug testing, offering a more representative platform compared to animal models. Recognizing the necessity for intricate models mirroring tumor complexities, we developed 3D tumor hetero-spheroids integrating cancer cell lines, fibroblasts, and immune cells.

**Material and Methods**
We employed human-derived cancer cell lines (colon, breast and pancreas) for in vitro cancer-therapy evaluation in spheroid co-cultures with fibroblast cells. We characterized differences in the architectural organization of the spheroids and performed whole transcriptome analysis (WTA) of cancer spheroid monocultures in contrast to spheroid co-cultures of cancer cells and fibroblasts. We assessed monocyte infiltration and macrophage differentiation in our models and used it for testing therapeutic compounds and addition of CRISPR-Cas9 edited monocytes. To mimic tumor-specific T cell responses using fresh PBMCs from healthy donors we generated tumor-educated T cells and assessed killing, T cell checkpoint expression and measured cytokines.

**Results and Discussions**
Architectural studies revealed distinct patterns influenced by cancer cell lines, i.e. collagen-1 expression by fibroblasts. Transcriptome analysis showcased upregulated hypoxia (Ca-IX) and apoptosis (cleaved-Caspase-3) related markers and ECM development, simulating properties of genuine tumors. Evaluation of cytotoxicity using the NCI drug library revealed complex responses, suggesting the heterogeneity of tumor responses to therapeutic compounds. Inhibition of monocyte infiltration into spheroids was seen upon addition of a CSF1R inhibitor. The effect was replicated with CRISPR-Cas9-directed knockout of the CSF1 receptor on monocytes before addition to spheroids resulting in >90% decrease in infiltration. After co-culture between PBMCs and tumor cells, we detected increased killing of tumor spheroids (70% killing), increased expression of T cell immune checkpoints (PD-1, LAG-3 and TIGIT) and increased secretion of IFNγ in the co-culture supernatant.

**Conclusion**
We here present our 3D cancer spheroid models which are engineered to replicate the complex and dynamic tumor microenvironment more accurately compared to traditional two-dimensional cell cultures. These models aim to mimic the structural, biochemical, and cellular components of tumors, thereby providing a more physiologically relevant platform for studying cancer biology and testing therapeutic interventions.

### EACR2024-0947
### Role of Toll-like receptor 2 in tumor cell autonomous and microbiota-driven chemoresistance in breast cancer

*C. Cossu[1], A. Di Lorenzo[1], E. Bolli[1], L. Avalle[1], L. Conti[1]*
*[1]University of Turin, Department of Molecular Biotechnology and Health Sciences, Turin, Italy*

**Introduction**
Besides its importance in immune responses, Toll-Like Receptor (TLR)2 is also expressed on breast cancer (BC) cells, correlating with poor prognosis and chemo-resistance in patients. Indeed, chemotherapy induces the release of DAMPs by damaged cells, including TLR2's activatory ligand. In addition, chemotherapy may induce dysbiosis thus increasing the release of PAMPs that may activate immune receptors, including TLR2. We aimed to understand the TLR2-mediated mechanisms in BC progression and chemoresistance, while also investigating whether the breast microbiota influences chemotherapy response via TLR2 activation.

**Material and Methods**
TLR2 inhibitor CU-CPT22 was tested in combination with chemotherapy in vitro and in vivo in a 4T1 BC model. Mammary dysbiosis was induced in BALB/c mice through local administration of a non-absorbable antibiotic cocktail. Subsequently, TLR2-silenced 4T1 cells were injected into the dysbiotic mammary gland, and mice were treated with doxorubicin. Tumor growth, metastases and tumor immune infiltrate were assessed.

**Results and Discussions**
Inhibiting TLR2 increased tumor cell sensitivity to doxorubicin in vitro. Combined treatment with CU-CPT22 and doxorubicin significantly reduced tumor growth and lung metastases in a 4T1 mouse model. This combination therapy increased $CD8^+$ T cells and decreased Treg and gMDSCs in the tumor, accompanied by a shift from M2 to M1 macrophages. Thus, TLR2 inhibition enhances doxorubicin efficacy, reducing tumor progression while restoring an immunocompetent tumor microenvironment (TME). Antibiotic treatment in 4T1 tumor-bearing mice exacerbated tumor progression and chemoresistance compared to control and doxorubicin-treated mice, suggesting a potential role for dysbiosis in these mechanisms. Conversely, antibiotic-treated TLR2-silenced 4T1 tumor bearing mice showed no difference in chemotherapy response compared to doxorubicin-treated mice, indicating that TLR2 may promote dysbiosis-dependent chemoresistance.

**Conclusion**
TLR2 promotes BC progression and chemoresistance. Thus, TLR2 inhibition can restore BC sensitivity to chemotherapy. Furthermore, antibiotic-induced dysbiosis in the mammary gland promotes tumor progression and increases chemoresistance in $TLR2^{WT}$-tumor-bearing mice. However, silencing TLR2 within the tumor reverses this effect, suggesting a potential mechanism whereby increased release of PAMPs within the dysbiotic TME binds to TLR2, thereby exacerbating tumor growth and chemoresistance.

### EACR2024-0973
### Comprehensive analysis of tumoral functional markers with an automated signal amplification method for multiplex

© 2024The Authors. Molecular Oncology is published by John Wiley & Sons Ltd on behalf of Federation of European Biochemical Societies

### immunofluorescence

*F. Rivest[1], C. Sinthon[1], P. Juricic[1], P. Bordignon[1], B. Nicolai[1], S. Brajkovic[1]*
*[1]Lunaphore Technologies, Application Development, Tolochenaz, Switzerland*

**Introduction**

Multiplex immunofluorescence (IF) is a pivotal technology for extracting maximum information on the tumor microenvironment (TME). While most markers are easy to detect through standard indirect IF, some show lower and heterogeneous expression (PMID: 38424144). These could benefit from a strategy to amplify their signal; however currently available methods do not ensure the linear increase of the signal. Integrating the capacity to enhance the detection of low-expressed markers alongside a multiplex IF system could reveal tumor molecular profiles. Here, we implement a novel signal amplification method on the COMET™ platform to detect low-expressed markers in an immune-oncology panel for multiplex IF.

**Material and Methods**

COMET™ is a fully automated platform that performs sequential immunofluorescence (seqIF™) assays, consisting of repetitive staining, imaging, and elution cycles on the same sample (PMID: 37813886). Using seqIF™, our amplification method increases the number of detection antibodies linked to the primary antibodies. The performance of the amplification method was tested on low-expressed markers and diluted primary antibodies for canonical markers using human tissue microarray samples with healthy and tumoral cores. Results were compared to standard seqIF™ assays and the signal intensity was assessed using the normalized mean intensity, and signal-to-background ratio.

**Results and Discussions**

The novel amplification method was successfully integrated within a seqIF™ protocol, automated, and optimized on COMET™. Several low-expressed and heterogeneous functional markers, such as FOXP3, PD-1, and PD-L1, could be detected with a significantly amplified signal in a linear fashion, allowing for quantitative assessment. In addition, primary antibody concentration could be reduced by up to four times while keeping the same staining intensity. The efficient elution step removes the amplified detection complexes and primary antibodies, allowing subsequent staining cycles for a multiplex seqIF™ assay on the same tissue section.

**Conclusion**

Incorporating this quantitative signal amplification into the seqIF™ workflow for creating hyper-plex panels will enable the accurate detection of challenging biomarkers, including low expressed ones and markers for which the primary antibody is scarce or valuable. Combining this new amplification approach with standard seqIF™ can substantially enhance immune cells' analysis within their TME.

### EACR2024-0977
### Rasal1 impairment augments CD8+ T cell antitumoral function in murine melanoma

*M. Issa[1,2,3], A. Schcolnik-Cabrera[4]*
*[1]Massachusetts General Hospital, Neurology, Boston, United States*
*[2]Hospital Maisonneuve Rosemont, Immuno-Oncology Axis, Montreal, Canada*
*[3]University of Montreal, Microbiology and Immunology, Montreal, Canada*
*[4]University of Alberta, Medical Microbiology and Immunology, Edmonton, Canada*

**Introduction**

Immune checkpoint blockade (ICB) demonstrated effectiveness in several malignancies including colorectal, lung and skin cancer. However, a visible proportion of cancer patients are not responsive to ICB, highlighting the need for novel immunotherapy strategies that are effective in resistant patients. One such strategy could be by inhibiting intracellular immune checkpoints such as the GTPase activating protein Rasal1.

**Material and Methods**

An endonuclease-mediated mouse model of impaired Rasal1 was utilized to examine tumor growth in vivo. Parental and PDL1 overexpressing B16F10 cells were implanted in WT or mutant C57Bl/6N mice. RT-PCR was used to determine cytokine mRNA levels in activated T cells in vitro. Protein expression was determined using flow cytometry and FlowJo for tumor infiltrating leukocytes (TILs); whereas colocalization and clustering were determined using Cytobank.

**Results and Discussions**

Rasal1-mutant mice reduced melanoma growth, and prolonged survival. Rasal1-mutant mice demonstrated an augmentation in tumor infiltrating $CD8^+$ TIL activation and cytolytic function. Rasal1 impairment also resulted in $CD8^+$ TIL Wnt signaling activation, in increased intra-tumoral $CD8^+$ $TCF1^+$ T cell progenitors, and in increased $CD8^+$ stem-like memory TILs.

**Conclusion**

These results highlight the role of Rasal1 as a novel intracellular immune checkpoint in $CD8^+$ TILs, and its potential as a target for the development of cancer immunotherapies.

### EACR2024-0999
### Decoding Resistance to NK Cell Cytotoxicity in Ovarian and Breast Cancer: Pan-Tumor Insights

*J. Wischhusen[1,2], R. Weber[1,2], E. Donato[1,2], T. Hofman[3], B. Besemer[1,2], A. Trumpp[1,2]*
*[1]German Cancer Research Center DKFZ, Division of Stem Cells and Cancer, Heidelberg, Germany*
*[2]Heidelberg Institute for Stem Cell Technology and Experimental Medicine HI-STEM gGmbH, Normal and Malignant Stem Cells, Heidelberg, Germany*
*[3]Mannheim Institute for Innate Immunosciences MI3-Medical Faculty Mannheim, Department of Immunobiochemistry, Mannheim, Germany*

**Introduction**

Natural Killer (NK) cells play a crucial role in immune surveillance against tumorigenesis by identifying and eliminating tumor cells (TCs). However, the potential clinical use of NK cells in cancer immunotherapy faces challenges due to TC resistance and NK cell exhaustion. This study explores the NK cell interaction with ovarian and breast cancer TCs to unveil resistance mechanisms.

**Material and Methods**

Molecular Oncology 18 (Suppl. 1) (2024) 1–495
© 2024 The Authors. Molecular Oncology is published by John Wiley & Sons Ltd on behalf of Federation of European Biochemical Societies

Patient-derived primary ovarian and breast cancer cells were co-cultured with healthy NK cells isolated from human blood. Surviving TCs derived from overnight co-cultures, and attacked TCs sorted from co-cultures in presence of pan-protease substrate were analyzed via bulk RNA sequencing. TC survival and proliferation were assessed via flow cytometry.

**Results and Discussions**

Co-culturing TCs with primary NK cells revealed distinct sensitivities, with breast cancer lines (CTC1063, CTC1125, CTC1119) showing 40.5%, 36.5%, and 16.5%, and ovarian cancer lines (OC15, OC19, OC20) exhibiting 47.5%, 2.5%, and 18% of TC survival, respectively, at a 1:1 effector-to-target ratio. RNA sequencing of surviving TCs unveiled unique transcriptional profiles. NK-resistant breast cancer cells displayed 1209, 852, and 390 differentially expressed genes (DEGs), with 443 partially and 49 fully shared DEGs. Ovarian cancer cells presented 920, 146, and 736 unique DEGs, with 261 partially and 20 fully shared DEGs. Interestingly, 223 gene sets were enriched in the surviving TCs of all six tumor models linking NK evasion to changes in proliferation, apoptosis, and migration. In vitro analysis indeed confirmed less proliferation (20.1% in S phase) in resistant TCs compared to controls (37.8% in S phase) in the OC15 model, aligning with literature on proliferative stress triggering NK cell killing. To gain insight into TC vulnerabilities, attacked, yet still viable, TCs were identified using protease substrates and transcriptionally profiled. A total of 3043 DEGs were found in breast cancer cells, with 26% shared among CTC1063, CTC1119, and CTC1125. Enriched gene sets (81 total, 17% shared) indicated downregulated breast cancer signatures in sensitive TCs, linking tumorigenic potential to NK cell evasion.

**Conclusion**

This pan-tumor analysis highlights heterogeneous responses and shared resistance/vulnerability to NK cells across ovarian and breast cancers. The identified biological features of resistant TCs necessitate further exploration and validation, paving the way for innovative combinatorial NK-driven immunotherapies.

### EACR2024-1012
### Investigating the role of the cystine/glutamate antiporter xCT in the extracellular vesicles-mediated modulation of the pre-metastatic niche

*A. Iacoviello[1], R. Ruiu[1], M. Tapparo[2], C. Landi[3], M. Arigoni[1], F. Cavallo[1]*
*[1]University of Turin, Department of Molecular Biotechnology and Health Sciences, Turin, Italy*
*[2]University of Turin, Department of Medical Sciences, Turin, Italy*
*[3]University of Siena, Department of Life Sciences, Siena, Italy*

**Introduction**

Breast cancer is the most common malignancy among women worldwide, and metastasis is the primary cause of treatment failure and mortality. Breast cancer cells upregulate antioxidant mechanisms to maintain redox homeostasis to sustain survival and proliferation. This includes upregulation of the cystine/glutamate antiporter xCT. xCT plays an important role in breast cancer initiation and progression and it represents an attractive therapeutic target. However, the mechanisms through which xCT affects the metastatic process require further investigations. To this aim, given the reported role of xCT in the release of extracellular vesicles (EV), we decided to explore the existence of a causal link between xCT-mediated EV release and the metastatic process, with a particular focus on the formation of the pre-metastatic niche.

**Material and Methods**

We used 4T1 cells and $xCT^{KO}$ 4T1 cells, generated using CRISPR/Cas9 technology, to study the contribution of xCT to malignant properties in vitro and in vivo. EV were isolated from the cell culture media of xCT-proficient and xCT-deficient cells to explore the non-cell autonomous contribution of xCT to the metastatic process. EV were analysed through the nanoparticle tracking analysis (NTA), and LC/MS-MS was performed to characterize EV cargo.

**Results and Discussions**

The depletion of xCT in 4T1 cells reduces cell migration in vitro and lung metastasis in vivo. This is accompanied by an altered immune cell recruitment in the metastatic niche. Interestingly, these alterations are observed even at the pre-metastatic stage. To understand the mechanisms through which xCT modulate the pre-metastatic niche composition, we hypothesized that EV may play a role in this process. Consistent with this, we observed that the absence of xCT in 4T1 cells does not reduces significantly the release of EV but strongly alters the composition of their cargo. Indeed, proteins selectively expressed in EV isolated from xCT-proficient cells are pro-tumorigenic, unlike EV isolated from $xCT^{KO}$ cells. In addition, we observed that xCT is present in EV derived from $xCT^{WT}$ cells.

**Conclusion**

The absence of xCT diminishes the metastatic potential of breast cancer cells, thereby influencing the formation of a pro-metastatic niche. Moreover, xCT is involved in the regulation of EV cargo. These results provide the basis to better elucidate the mechanism through which xCT modulates the formation of the pre-metastatic niche.

### EACR2024-1025
### Gut microbiome modulation affects tumor development and immune response in a mismatch repair-deficient colorectal cancer mouse model

*P. Vieira[1,2,3], J. Pereira-Marques[1,3], M. Tavares[1,3], R. M. Ferreira[1,3], J. C. Machado[1,2,3], C. Figueiredo[1,2,3]*
*[1]Instituto de Investigação e Inovação em Saúde da Universidade do Porto, i3S, Porto, Portugal*
*[2]Faculty of Medicine of the University of Porto, Department of Pathology, Porto, Portugal*
*[3]Instituto de Patologia e Imunologia Molecular da Universidade do Porto, Ipatimup, Porto, Portugal*

**Introduction**

Colorectal cancer (CRC) is the 3rd most incident and the 2nd most lethal cancer worldwide, driven by a complex

© 2024 The Authors. Molecular Oncology is published by John Wiley & Sons Ltd on behalf of Federation of European Biochemical Societies interplay of genetic and epigenetic mutations. Among these, deficiencies in the DNA mismatch repair system due to mutations in the Msh2 gene, which can lead to microsatellite instability (MSI), are prominent. While MSI tumors hold promise for immunotherapy, response rates remain low. Recent evidence showed that the gut microbiome can influence cancer development and predict response to immunotherapeutic drugs in cancer patients. Therefore, our aim was to use CRC with MSI as a model to explore the interplay between the microbiome, the immune response, and cancer.

**Material and Methods**

We modulated the gut microbiome of an inducible genetically modified mouse model, characterized by the loss of both Msh2 alleles in Lrig1-expressing quiescent intestinal progenitor cells, resulting in CRC with a MSI phenotype. The gut microbiome was depleted with an antibiotics' cocktail followed by repopulation with *Bifidobacterium* species. After 300 days post-tumorigenesis induction, mice were euthanized. Fecal samples were collected for gut microbiome analysis by next-generation sequencing and qPCR. Intestinal tumor tissues were collected for histopathological evaluation and immune profile characterization by RNA sequencing.

**Results and Discussions**

Msh2 mice inoculated with *Bifidobacterium* species had a lower tumor incidence (39%; n=18) in comparison with control animals (60%; n=20). The number of tumors per animal and the tumor size was also smaller in *Bifidobacterium*-inoculated mice. Transcriptomics analysis showed 261 differentially expressed genes between tumors of the two experimental groups. Functional annotation clustering categorized these genes into two strong clusters which identified pathways associated with the innate and adaptive immune response. Cluster 1 contained 37 genes while Cluster 2 contained 28 genes. Cell-type enrichment analysis of these tumors revealed a decline in the adaptive immune response, characterized by decreased granulocytes and macrophages, along with an increase of the innate immune response, characterized by increased B and T cells in the *Bifidobacterium*-inoculated mice.

**Conclusion**

Further experiments are ongoing to characterize the microbiome's role in tumor development and immune modulation, potentially guiding innovative therapeutic strategies for the microbiome-immune-cancer axis.

*Acknowledgment: PV has a PhD scholarship from Fundação para a Ciência e a Tecnologia (FCT;2020.06228.BD).*

### EACR2024-1034

### Targeting therapy-resistant melanoma cell states by cytotoxic CD4 T cells

*S. Stupia[1], A. Brüggemann[1], B. Thier[1], S. Kwapik[1], A. Sucker[1], D. Schadendorf[1], F. Zhao[1], B. Izar[2], F. Rambow[3], A. Paschen[1]*

*[1]University Hospital Essen, Dermatology, Essen, Germany*

*[2]Columbia University Irving Medical Center, Division of Hematology/Oncology- Department of Medicine, New York, United States*

*[3]University Hospital Essen, Institute for Artificial Intelligence in Medicine IKIM, Essen, Germany*

**Introduction**

Melanoma cells can switch between phenotypes, ranging from melanocytic, proliferative to more de-differentiated, invasive phenotypes. By transitioning from differentiated into de-differentiated cell states, melanoma cells acquire resistance to targeted therapies with MAPK inhibitors (MAPKi), which remains a major clinical challenge. Interestingly, a subset of melanomas shows aberrant constitutive surface expression of major histo-compatibility complex class II (*const*MHC-II) molecules, presenting antigens to CD4 T cells, which recently attracted attention as direct cytotoxic anti-tumor effectors. By defining the underlying mechanisms of aberrant MHC-II expression, we asked if the *const*MHC-II-CD4-T-cell axis could be exploited to target therapy-resistant melanoma cell states.

**Material and Methods**

Patient metastases-derived melanoma cell lines were treated with MAPKi until resistance evolved and effects on tumor cell phenotype and differentiation were determined. Transcriptomes from tumor cell lines and single cells were analyzed for associations between melanoma differentiation programs and *const*MHC-II expression, driven by the transactivator CIITA (*const*CIITA). ATAC-sequencing was performed to define the transcription factors and signaling pathways involved in *const*CIITA expression. The functional relevance of *const*MHC-II expression was studied in autologous tumor-CD4-T-cell models.

**Results and Discussions**

We noted that a subset of de-differentiated melanomas with acquired MAPKi resistance gained *const*CIITA/MHC-II expression and demonstrated that CD4 T cells recognize and kill MAPKi-resistant melanoma cells in an MHC-II-dependent manner. *const*CIITA/MHC-II expression is strictly associated with the differentiation state of melanoma cells and correlates with the neural crest stem-like cell expression program. Moreover, we demonstrated that *const*CIITA expression is dependent on phenotype-specific signaling networks and permissive chromatin states and that, by exploiting these mechanisms, it is possible to induce MHC-II expression even in de-differentiated MAPKi-resistant MHC class II-negative melanoma cells, sensitizing them to CD4 T cells.

**Conclusion**

Our data suggest that tumor constitutive CIITA/MHC-II expression can be induced and exploited to target aggressive, therapy-resistant melanomas with CD4 T cells. Besides MAPKi-resistant tumors, this approach could be of particular relevance for the eradication of CD8-T-cell-resistant tumors with genetic MHC class I loss.

### EACR2024-1054

### Use of patient-derived tumoroids in a screening platform for immune cell-based killing assays

*X.. Yang[1], L. Bailey Steinitz[1], M. Mafreshi[1], J. Delgadillo[1], G. Wong[1], C. Vivek[1], C. Paul[2], J. Sharp[1], M. Dallas[2], E. Willems[1]*

*[1]Thermo Fisher Scientific, Cell Biology, Carlsbad, USA*

© 2024 The Authors. Molecular Oncology is published by John Wiley & Sons Ltd on behalf of Federation of European Biochemical Societies

[2]Thermo Fisher Scientific, Cell Biology, Frederick, USA

**Introduction**

With the ongoing expansion of cancer immunotherapy into the treatment of solid tumors, selecting suitable experimental models for developing and validating immunotherapies is a major obstacle. Patient-derived tumoroid models are increasingly attractive in this area, as they better capture patient-specific genomic, transcriptomic, and proteomic profiles, all in a 3D environment. Here, we demonstrate use of patient-derived tumoroids in a screening platform for effectively evaluating cell-based immunotherapies.

**Material and Methods**

Tumoroid models were established in vitro from fully consented patient-derived tumor tissue. Some tumoroids were engineered using a -EmGFP lentivirus to generate a stable GFP-expressing pool, which was further characterized via bulk/single-cell RNAseq and genomic sequencing. For non-GFP tumoroids, a cell-permeant mitochondrial dye (TMRM) was used to enable quantitative imaging. Chimeric antigen receptor (CAR) T cells recognizing Meso3 or CD19 were knocked-in to donor T cells using CRISPR/Cas9 and a CAR cassette adeno-associated virus (AAV). Alternatively, natural killer (NK) cells were isolated from donor blood and expanded using CTS™ NK-Xpander™ Medium. Immune effector cells (primary NK or CAR T) were co-cultured with tumoroid cells in 96-well plates at varying effector-to-target ratios and monitored via live imaging. Cytotoxicity and cell health were measured by caspase live staining and GFP/TMRM intensity.

**Results and Discussions**

A GFP-expressing patient-derived tumoroid line was successfully generated, with genomic and transcriptomic profiles highly concordant with the parental line. Immune cell killing efficiency at various effector-to-target ratios was successfully captured in a ratio-dependent manner via live imaging/analysis. Both NK and CAR T cytotoxicity was measured through caspase and GFP/TMRM intensity. CAR T killing was specific, with only Meso3-tumoroids killed by Meso3 CAR T cells.

**Conclusion**

Pooled lentiviral GFP transformation established a reporter tumoroid line without cloning, maintaining parental genomic/transcriptomic characteristics. This approach efficiently engineers patient-derived tumoroids for specific research goals. Using both reporter and non-engineered tumoroids, we developed multiplex killing assays monitoring viability/toxicity. This platform can predict tissue responses and screen solid tumor immunotherapies in real-time, cell-based assays.

### EACR2024-1077

### Characterization of a growth inhibitory microenvironment in glioblastoma

S. Menon[1,2], X. Lun[3], P. Zeng[1,4], J. Zhang[1], A. Poole[3], J. Chan[3], D. Quail[5,6], S. Robbins[1,2,3,7], D. Senger[1,2,3,7]
[1]Lady Davis Institute for Medical Research at the Jewish General Hospital, Segal Cancer Centre, Montreal, Canada
[2]McGill University, Division of Experimental Medicine, Montreal, Canada
[3]University of Calgary, Department of Oncology, Calgary, Canada
[4]McGill University, Department of Anatomy and Cell Biology, Montreal, Canada
[5]McGill University, Department of Physiology, Montreal, Canada
[6]McGill University, Rosalind and Morris Goodman Cancer Institute, Montreal, Canada
[7]McGill University, Gerald Bronfman Department of Oncology, Montreal, Canada

**Introduction**

Survival rates for glioblastoma, the most lethal form of adult brain cancer, have not improved in decades. Advances in multi-omics profiling and mouse modelling have enabled unprecedented insight into the glioblastoma tumor microenvironment. Yet, targeting of the micro-environment has been largely unsuccessful, in part due to an incomplete understanding of the interactions between glioma cells and infiltrated immune cells composed predominately of myeloid cells. By screening factors present in the inflammatory secretome of glioblastoma, we found that the dual function (nuclear and secreted) cytokine IL-33 aids glioma progression through recruitment and reprogramming of microglia and/or macrophages to a pro-tumor phenotype. Remarkably, crippling the nuclear function of IL-33 by deletion of its nuclear localization signal (ΔNLS) results in marked inhibition of tumor growth and long-term survival in vivo.

**Material and Methods**

Multiplex immunohistochemistry and spatial transcriptomics were used to characterize innate immune cell populations in tumors expressing nuclear-deficient IL-33. Human and mouse pre-clinical xenograft models of glioblastoma were used to evaluate the growth inhibitory capacity of ΔNLS IL-33. Changes in the tumor microenvironment were probed using immuno-histochemistry, flow cytometry, and high throughput proteomic profiling of tumor interstitial fluid.

**Results and Discussions**

We analyzed the progression of ΔNLS IL-33 and full-length IL-33 expressing tumors across a comprehensive time-course and found that a population of glioma-inhibitory macrophages (GIMs) was exclusive to the growth inhibitory ΔNLS IL-33 environment. Using markers informed by histological and spatial transcriptomic analysis, we confirmed the presence of GIMs in patient derived xenografts of glioblastoma of long-term survivors (>300 days) compared to short-term survivors (<100 days). Establishment of tumors using a mixture of tumor cells expressing nuclear-deficient IL-33 or full-length IL-33 resulted in prolonged survival, demonstrating that ΔNLS IL-33 enables a dominant growth inhibitory microenvironment via the recruitment of GIMs.

**Conclusion**

We establish that nuclear defective IL-33 activates a unique population of glioma-inhibitory macrophages that promote long-term survival in models of human and mouse glioblastoma. Further understanding and development of therapeutic strategies to induce GIMs could facilitate a novel treatment for patients with glioblastoma.

© 2024 The Authors. Molecular Oncology is published by John Wiley & Sons Ltd on behalf of Federation of European Biochemical Societies

### EACR2024-1167
### GALR2-overexpression in a xenograft orthotopic model of Oral Squamous Cell Carcinoma (OSCC): tumor growth and immunophenotype

*I. Brum Reis[1], M.M.D. Carvalho[1], D.P. Ferrarezi[1], C.R. Nascimento[1], Á.F. Pelegrin[1], C. Rossa Jr.[1]*
*[1]São Paulo State University, Department of Diagnosis and Surgery, Araraquara- SP, Brazil*

**Introduction**

Controversial studies indicates that Galanin receptor 2 (GALR2) may be a tumor suppressor or an oncogene in Oral Squamous Cell Carcinoma (OSCC). We investigate the influence of GALR2 in a murine xenograft orthotopic model of OSCC, assessing tumor growth and the associated immune response in the microenvironment.

**Material and Methods**

A xenograft orthotopic model of OSCC was induced in 10 athymic nude Balb/c mice by injecting $5x10^5$ SCC9 cells (pcDNA: empty-vector-transfected, n=5 or GR2: GALR2-overexpressing, n=5) directly into the floor of the mouth. Two weeks after tumor induction animals were euthanized and tumors were processed for histopathology, immunofluorescence (GALR2, CD80); RT-qPCR (GALR2, Galanin, N-Cadherin, Vimentin, CD47, PTGS2, IL-6, TNF, IL-10, TGFb) and flow cytometry (CD45, CD11b, F4/80, CD80).

**Results and Discussions**

Histopathology indicated that pcDNA tumors were also better-differentiated tumors in comparison with GR2 tumors. Notably, GR2 tumors had increased expression of Vimentin and N-cadherin in comparison with pcDNA tumors. There were no significant differences in GALR2 and Galanin gene expression between tumors; however GALR2 immunofluorescence indicated higher expression of GALR2 in GR2 tumors. Macrophages (CD80+ cells) were detected near the tumor invasive front, and the average of FIR was higher in pcDNA tumors. F4/80 expression was not significantly different, suggesting that overall macrophage infiltration was not affected but macrophage phenotype skewed to the M1-end of the spectrum in pcDNA tumors. Flow cytometry analysis showed reduced infiltration of CD45+CD11b+ myeloid cells in GR2 tumors, with no difference in the overall leukocyte infiltration (CD45+ cells). Flow cytometry verified the IHC data indicating that infiltration of macrophages (F4/80+ cells) was similar in both tumors. GR2 tended to have a higher expression of CD47 suggesting that efferocytosis activity was reduced in this microenvironment. Expression of PTGS2 was higher in GR2 tumors compared to pcDNA. To further characterize the immune response in the TME, we determined expression of IL-6, TNF, IL-10 and TGFb. Expression of all these candidate genes associated with M2-like TAM phenotype were increased in GR2 tumors

**Conclusion**

The results suggest that in this particular xenograft orthotopic model of OSCC, overexpression of GALR2 promotes tumorigenesis and more aggressive tumors, stimulates expression of cytokines associated with M2-like TAMs and is associated with a reduced infiltration of OSCC by myeloid cells.

### EACR2024-1172
### Crosstalk Between Gene Expression Profiles, Microbial Composition, and Hypoxia in Oral Potentially Malignant Disorders: Insights from a Molecular Analysis

*A. Licata[1], C. Gurizzan[2], D. Lenoci[1], M. Lucchetta[1], C. Resteghini[3], L. Lorini[2], L. De Cecco[1], P. Bossi[3]*
*[1]Fondazione IRCCS Istituto Nazionale dei Tumori, Integrated Biology of Rare Tumors- Department of Research, Milan, Italy*
*[2]IRCCS Humanitas Research Hospital, Department of Biomedical Sciences, Milan, Italy*
*[3]Humanitas University IRCCS Humanitas Research Hospital, Department of Biomedical Sciences, Milan, Italy*

**Introduction**

Oral Potentially Malignant Disorders (OPMD) encompass various conditions in the oral cavity posing a risk of malignant transformation, with an annual rate of 1 to 7%. Despite extensive research, current clinical factors inadequately predict malignant progression. Our study aims to utilize gene-expression (GE) data to independently stratify OPMDs and explore their correlation with the oral microbiome. Additionally, we declare no financial interests in this research.

**Material and Methods**

Archival FFPE OPMD tissue specimens from 66 patients were analyzed. The study, supported by Fondazione AIRC IG_2018 nr_21740 to P. Bossi, included patients with OPMDs who underwent complete excision at the University of Brescia, Italy, from March 1996 to November 2019. Ethical approval was obtained, and patients provided written informed consent. Sequencing libraries were prepared using QuantSeq 3' mRNA-Seq Library Prep. Multiple gene signatures were employed to predict both the immune microenvironment and prognosis. To examine the microbial composition and functions within the gene expression (GE) data, we utilized a metagenomic approach to decontaminate, extract, and analyze microbial RNA reads from the GE data.

**Results and Discussions**

FFPE OPMD samples showed a hypoxic gene profile signature in 10 out of 66 eligible patients, of which 7 subsequently developed oral malignant transformations. Taxonomic composition differences were observed, with *Fusobacterium* and *Leptotrichia* genus dominating hypoxic patients. These patients exhibited lower oral microbiome diversity for both Shannon and observed species indexes. *Fusobacterium nucleatum* and *Pasteurella multocida* showed consistent upregulation in hypoxic patients, suggesting their potential role in hypoxia. Metaproteomic analysis identified 2003 microbial proteins, with Q8RIH3 from *Fusobacterium nucleatum* significantly upregulated, indicating specific microbial activity in hypoxia. These findings underscore the interplay between GE profiles, microbial composition, and hypoxia in OPMDs, with implications for predictive biomarkers and therapeutic targets.

**Conclusion**

© 2024 The Authors. Molecular Oncology is published by John Wiley & Sons Ltd on behalf of Federation of European Biochemical Societies

The oral microbiota of OPMD patients may contribute to the formation of a pro-tumoral hypoxic environment. The significance of *Fusobacterium nucleatum* in this context emphasizes the necessity for additional research into predictive biomarkers and potential therapeutic targets.

### EACR2024-1188
### Evaluation of checkpoint inhibitor efficacy and/or engraftment in a humanized immune system mouse with and without transgenic human cytokine expression in a model lacking murine Fc gamma receptors

*M. Buczek[1], N. Smith[1], M. Macbride[1], E. Andino[1], E. Sack-Flynn[1], I. Gladwyn-Ng[1], D. Freer[1], M. Vedder[1], K. Bott[1], L. Baskin[1], J. Richardson[1]*
*[1]Taconic Biosciences- Inc., Research and Development, Rensselaer- NY, United States*

**Introduction**
Traditional humanized immune system (HIS) mice retain murine immune cells such as neutrophils and macrophages, and Fc gamma receptors (FcγRs) on these residual murine immune cells can interact with human IgG-based therapeutics to confound preclinical results. To determine whether knockout of murine Fc gamma receptors (FcγRs) in a super-immunodeficient mouse model would alter aPD1 efficacy compared to the parent strain, we studied tumor growth kinetics, human reconstitution, and tumor infiltrating leukocytes (TILs) in each strain engrafted with HCC827 lung adenocarcinoma tumor cells treated with pembrolizumab or vehicle. Additionally, we also evaluated the human engraftment and immune profile in a myeloid-expressing model.
**Material and Methods**
Two studies with either HIS NOG (huNOG) or HIS FcγR knockout NOG mice (FcResolv™ huNOG) huNOG-EXL and FcResolv huNOG-EXL were made using identical protocols with CD34+ cells (3 shared donors) but not studies. Baseline reconstitution was evaluated in all naïve animals. HCC827 cells were inoculated in remaining NOG. Following randomization on D7, mice were dosed twice weekly for 4 wks then euthanized for blood, spleen, and tumor analysis. Bodyweight, clinical observations, and tumor growth were measured.
**Results and Discussions**
For a given donor, in both studies, no significant differences were seen between the FcResolv and parent strain in overall human chimerism. Pembro treatment showed significant tumor growth inhibition in 1 donor in FcResolv huNOG, but not huNOG mice. Human TILs in pembro-treated mice were significantly different between the strains for all donors, with more CD8+ T cells and fewer TAMs in FcResolv huNOG compared to vehicle-treated mice, and no significant differences in huNOG. Evaluation of murine TILs revealed differences in murine macrophage populations, regardless of treatment, with Ly6Clo dominant in FcResolv huNOG and Ly6Chi dominant in huNOG.
**Conclusion**
Our study demonstrates when treated with anti-PD1, FcResolv huNOG show expected pharmacodynamic changes and donor-dependent efficacy whereas pembro-treated huNOG showed neither. Additionally, the FcResolv NOG-EXL demonstrated human engraftment and myeloid lineage providing the opportunity to extend these differences where human myeloid cells are concerned. These differences are due to the presence or absence of murine FcγRs and their impact on antibody IgG-based therapeutics.

### EACR2024-1194
### Targeting ERAP1 to enhance antitumor immunity in melanoma

*P. Gragera Álvarez[1], M. Nikopaschou[2,3], D. Verena[1], S. Dolci[3], M. Samiotaki[5], Y. Kaminskiy[6], R. Brance[6], E. Stratikos[2,3], D. Fruci[1]*
*[1]Ospedale Pediatrico Bambino Gesù, Oncohaematology, Roma, Italy*
*[2]National and Kapodistrian University of Athens, Department of Chemistry, Athens, Greece*
*[3]University of Rome "Tor Vergata", Department of Biomedicine and Prevention, Roma, Italy*
*[3]National Centre for Scientific Research Demokritos, Agia Paraskevi 15341, Athens, Greece*
*[4]University of Rome "Tor Vergata", Department of Biomedicine and Prevention, Roma, Italy*
*[5]Biomedical Sciences Research Center "Alexander Fleming", Vari, Greece*
*[6]SciLifeLab, Department of Oncology-Pathology, Karolinska Institutet, Solna, Sweden*

**Introduction**
Endoplasmic reticulum (ER) aminopeptidase (ERAP1) is a metallopeptidase implicated in various biological functions, including blood pressure control, angiogenesis, and immunity. In the ER, ERAP1 is responsible for trimming precursor peptides to generate peptides of optimal length for binding to major histocompatibility complex class I (MHC-I) molecules. While loss of this enzyme activity is frequently correlated with reduction in MHC-I surface expression, which could contribute to tumor immune evasion, impairment of ERAP1 expression in tumor cells can lead to the generation of a new immunopeptidome. This altered repertoire of peptides can stimulate potent $CD8^{+}$ T-cell and NK-cell responses. Therefore, ERAP1 represents an intriguing target for enhancing antitumor immune responses. Dissecting the role of ERAP1 in tumor progression is essential and may lead to the discovery of new synergies in cancer immunotherapy.
**Material and Methods**
A murine melanoma cell line expressing ERAP1 was infected with an all-in-one lentiviral vector carrying all the required elements for the CRISPR/Cas9 machinery and a specific single guide RNA targeting the *ERAP1* gene to knock-out this enzyme. Loss of ERAP1 at the protein level was confirmed by western blot. In vivo experiments in NSG mouse were performed to collect material for immunopeptidome analysis on tumor samples. Splenocytes from tumor-bearing mice were used to perform the co-culture ex vivo assays.
**Results and Discussions**
Here, we investigate the extent to which ERAP1 shapes the antigen repertoire and enhances anti-tumor immunity in a murine melanoma model. We observed that the inhibition of ERAP1 reduces the stability of the MHC-I/

© 2024 The Authors. Molecular Oncology is published by John Wiley & Sons Ltd on behalf of Federation of European Biochemical Societies peptide complex, suggesting a change in the antigen repertoire presented in the MHC-I molecules. The cell lines were subcutaneously injected in NSG mice to obtain tumors used to perform an immunopeptidome analysis. In ex vivo assays involving genetic or pharmacological inhibition of ERAP1, we observed increased susceptibility of tumor cells to immune cell-mediated lysis, significantly elevating both apoptosis and tumor cell death. In vivo experiments in syngeneic C57BL/6 mice to monitor growth in mice and study the effect of ERAP1 silencing on the tumor microenvironment are in progress.

**Conclusion**

Our data suggests that inhibition of ERAP1 could be used to strengthen the antitumor immune response, opening a new window of opportunity for the potential use of ERAP1 inhibitors as a promising therapeutic target for the treatment of melanoma.

### EACR2024-1209
### Deciphering Bladder Tumor Microenvironment key players: BMP6, CXCL12 and TGF-β1 epigenetic regulation and impact in epithelial-mesenchymal transition

*C. Lima[1,2], S. P. Nunes[1,3,4], Â. Rodrigues[5], U. Chianese[2], V. Constâncio[1], M. Cantante[1,5], R. Benedetti[2], L. Altucci[2,6,7], C. Jerónimo[1,8], M. P. Correia[1,8]*
*[1]Research Center of IPO Porto CI-IPOP/RISE@ CI-IPOP Health Research Network- Portuguese Oncology Institute of Porto IPO Porto and Porto Comprehensive Cancer Center Porto.CCC Raquel Seruca- Porto- Portugal,Cancer Biology and Epigenetics Group, Porto, Portugal*
*[2]University of Campania "Luigi Vanvitelli"- Naples- Italy, Department of Precision Medicine, Naples, Italy*
*[3]University Hospital "12 de Octubre"- Madrid- Spain, Biomedical Research Institute I+12, Madrid, Spain*
*[4]CIEMAT Centro de Investigaciones Energéticas- Medioambientales y Tecnológicas- Madrid- Spain, Molecular Oncology Unit, Madrid, Spain*
*[5]Portuguese Oncology Institute of Porto IPO Porto- Porto- Portugal, Department of Pathology, Porto, Portugal*
*[6]Institute of Experimental Endocrinology and Oncology "Gaetano Salvatore" IEOS-National Research Council CNR- Naples- Italy, ieos, Naples, Italy*
*[7]Molecular Biology and Genetics Research Institute- Avellino- Italy, Biogem, Avellino, Italy*
*[8]School of Medicine and Biomedical Sciences- University of Porto ICBAS-UP- Porto- Portugal, Department of Pathology and Molecular Immunology, Porto, Portugal*

**Introduction**

Non-muscle invasive bladder cancer (NMIBC) progression to muscle-invasive bladder cancer (MIBC) represents a significant clinical concern. Tumor micro-environment (TME) and epithelial-mesenchymal transition (EMT) play a major role in bladder cancer (BlCa). Fibroblasts are predominant stromal BlCa TME cells, able to secrete cytokines that modulate tumor cells. Epigenetic reprogramming has been associated with TME modulation, including cytokine regulation. Here, we aimed to understand the interplay between TME cytokines and EMT in BlCa and to uncover epigenetic mechanisms behind cytokine regulation.

**Material and Methods**

A Cytokine/Chemokine PCR Array was performed on 3 NMIBCs, 5 MIBCs and 5 normal tissues. *TGFB1*, *CXCL12* and *BMP6* expression were evaluated in The Cancer Genome Atlas database and in an IPO-Porto cohort, comprising 68 primary BlCa tissues and 11 metastases. A single-cell BlCa database (Chen et al. 2020) was consulted to evaluate cytokine expression in BlCa TME. Primary bladder fibroblasts were treated with Panobinostat, to assess cytokine levels and histone marks. BlCa cell lines were treated with exogenous TGF-β1, BMP6 and CXCL12, and EMT-related genes were evaluated.

**Results and Discussions**

Normal tissues exhibited a significantly higher expression of *BMP6* and *CXCL12*, compared to BlCa TME. However, *CXCL12* was increased in MIBCs, compared to NMIBCs, and *BMP6* was slightly higher in MIBCs than in NMIBC high-grade. Moreover, *CXCL12* and *TGFB1* levels were upregulated in metastases, compared to MIBCs. In silico single-cell data showed fibroblasts as the most abundant stromal TME cells in BlCa, expressing high *TGFB1* and *CXCL12* levels. *BMP6* was mainly expressed by fibroblasts and endothelial cells, although in a much lower amount. Treatment of fibroblasts with Panobinostat unraveled that *CXCL12* and *BMP6* were epigenetically regulated by histone acetylation, as transcript expression was significantly increased. Upon TGF-β1 and BMP6 treatment, BlCa cells presented upregulation of EMT-related molecules, including *CDH2*, *SNAI1* and *SNAI2*, while CXCL12 treatment increased *CTNNB1* expression levels.

**Conclusion**

TGF-β1, BMP6 and CXCL12 were disclosed as important cytokines in BlCa. Remarkably, these cytokines were able to drive modulation of EMT-related molecules in BlCa cells. Moreover, in fibroblasts, *BMP6* and *CXCL12* were found to be epigenetically regulated by histone acetylation.

### EACR2024-1211
### Characterisation of the tumour immune microenvironment in homologous recombination-deficient prostate cancer

*J. Kwon[1], S. Phyu[1], K. Chan[1], R. Bryant[2], E. Parkes[1]*
*[1]University of Oxford, Oncology/Centre for Immuno-Oncology, Oxford, United Kingdom*
*[2]University of Oxford, Nuffield Department of Surgical Sciences, Oxford, United Kingdom*

**Introduction**

5-13% of prostate cancer (PCa) tumours harbour alterations in homologous recombination (HR) genes, which are associated with earlier onset of disease and a poor prognosis. Aberrations in HR drive genomic instability and subsequent inflammation, yet there are currently no immunocompetent in vivo models of PCa exploring how deficiencies in homology-directed repair impact the tumour immune microenvironment (TIME).

Molecular Oncology 18 (Suppl. 1) (2024) 1–495
© 2024 The Authors. Molecular Oncology is published by John Wiley & Sons Ltd on behalf of Federation of European Biochemical Societies

Here, we developed two syngeneic mouse models of HR-deficient PCa to investigate the effects of defective dsDNA break repair on the TIME.

**Material and Methods**

*Palb2* was targeted in MyC-CaP (Hi-*Myc*) and DVL3 (*Pten*$^{-/-}$*Trp53*$^{-/-}$) cells using a pooled crRNA approach and abrogated *Palb2* expression was validated via sequencing, immunoblotting, clonogenics, immunofluorescence imaging and flow cytometry. MyC-CaP and DVL3 cr*Palb2* cells were injected subcutaneously into the flank of immunocompetent male FVB and C57BL/6 mice respectively, to assess tumorigenicity, tumour growth delay and immune composition in response to immune-modulating treatments.

**Results and Discussions**

Upon attenuating *Palb2* levels in MyC-CaP and DVL3 cells, functional validation of cr*Palb2* demonstrated a significant increase in γH2AX foci and concomitant reduction in RAD51 recruitment to dsDNA breaks in both cell lines. Loss of *Palb2* expression also significantly enhanced sensitivity to PARP inhibition (olaparib) and elicited constitutive activation of the innate immune cGAS-STING and inflammasome pathways. Following engraftment of MyC-CaP and DVL3 cells into immunocompetent mice, cr*Palb2* tumours consistently propagated more slowly than their wildtype counterparts. Moreover, treatment with 3 x 5 Gy fractionated radiotherapy induced a significant growth delay in MyC-CaP cr*Palb2* tumours, confirming retention of the HR-deficient phenotype in vivo. Bulk RNA-seq of MyC-CaP cr*Palb2* tumours exhibited enrichment towards cytokine secretion and a heightened immune response. Furthermore, spectral flow cytometry analysis of MyC-CaP cr*Palb2* tumours revealed global changes in immune subsets across the TIME, namely reduced $CD8^+$ T cell infiltration and increased influx of PMN-MDSCs, M2 macrophages and dendritic cells.

**Conclusion**

Our novel immunocompetent model of HR-deficient PCa indicates a dichotomous role for HR deficiency in the prostate TIME, whereby it may promote anti-tumour immunity via recruitment of antigen-presenting cells, whilst simultaneously endorsing chronic inflammation and mediating therapy resistance through immuno-suppression.

### EACR2024-1214
### Systematic Review of Immune Checkpoint Inhibitor Therapy in Triple-Negative Breast Cancer: Current Status and Future Directions

L.R. Rajmohan[1], P. p[2], P. d[1]
*[1]ACS Medical College & Hospital, General Medicine, Chennai, India*
*[2]ACS Medical College & Hospital, Biochemistry, Chennai, India*

**Introduction**

Triple-negative breast cancer (TNBC) represents a subtype with limited treatment options and poor prognosis. Immune checkpoint inhibitors (ICIs) have emerged as a promising therapeutic avenue, yet their efficacy and mechanisms of action in TNBC remain unclear. This systematic review aims to evaluate the efficacy of ICI therapy compared to standard treatments and elucidate potential biomarkers and resistance mechanisms.

**Material and Methods**

A systematic literature search was conducted in PubMed, Embase, and Scopus databases to identify studies evaluating ICI therapy in TNBC. Articles reporting on clinical trials, biomarkers, and mechanisms of resistance were included. Data extraction and synthesis were performed to assess treatment efficacy and identify relevant biomarkers and resistance mechanisms.

**Results and Discussions**

ICIs demonstrate promising efficacy in TNBC, with durable responses observed in a subset of patients. However, response rates vary, and ICIs show modest benefits compared to standard chemotherapy. Potential biomarkers, including PD-L1 expression and tumor mutational burden, correlate with ICI response, yet their predictive value remains uncertain. Mechanisms of resistance involve tumor microenvironment alterations, upregulation of alternative immune checkpoints, and tumor cell intrinsic factors. Combination therapies targeting multiple pathways show potential to overcome resistance and enhance treatment efficacy.

**Conclusion**

ICI therapy holds promise as a treatment option for TNBC, but challenges remain in optimizing patient selection and overcoming resistance. Further research is needed to validate biomarkers, elucidate resistance mechanisms, and develop effective combination strategies to improve outcomes in TNBC patients.

### EACR2024-1233
### Can NK92 cells effectively kill cultured patient derived glioblastoma cells?

S. Graham[1], J. Reinecke[2], M. Cleveland[2], H.J. Kim[2], J. Yu[2], J. Hucklesby[3], A. Anchan[2], K. Angel[3]
*[1]University of Auckland, Molecular Medicine and Pathology, Auckland, New Zealand*
*[2]University of Auckland, Department of Molecular Medicine and Pathology- School of Medical Sciences-, Auckland, New Zealand*
*[3]University of Auckland, School Of Biological Sciences, Auckland, New Zealand*

**Introduction**

In this research we asked the simple question of whether NK92 cells could effectively kill patient-derived cultures of glioblastoma cells.  The rationale here is that NK92 cells have been shown to effectively kill other forms of cancer cells and are reportedly safe following infusion into more than 100 patients as a cell therapy. We postulate that at least some glioblastoma cells should have a suitable activation signature to allow recognition by NK92 cells.  However, it is our hypothesis, that the majority of the glioblastoma cells will not be sensitive to NK92-mediated killing at realistic effector: target (E:T) ratios.

**Material and Methods**

The glioblastoma cells used were originally derived from patient tumour resections by Auckland Cancer Society Research Centre and glioblastoma cultures were derived

© 2024 The Authors. Molecular Oncology is published by John Wiley & Sons Ltd on behalf of Federation of European Biochemical Societies by Professor Bruce Baguley's team. We have established cultures with a stem-like phenotype (serum-free conditions) and also a more differentiated and mixed GBM phenotype (serum-grown). The cells used in this study were all grown in 5% serum (FBS). NK92 cells were obtain from ATCC. A high-content imaging platform and analysis pipeline was developed to enable measurement of GBM cell loss.

**Results and Discussions**

We have currently assessed the effect of the NK92 cells on more than 6 patient-derived glioblastoma cultures. The major observation being that most of the GBM cells were insensitive to NK92 cells and were not killed even at E:T ratios of 1:1. However, at a high ratio of 5:1 certain glioblastoma cultures were more sensitive to the NK92 cells. Realistically, this ratio would be incredible challenging to achieve in an actual tumour micro-environment. We are currently comparing the sensitivity with the expression of activation/inhibition signatures by the GBM cells. This should also identify targets to enhance this sensitivity.

**Conclusion**

The observed insensitivity of most of the glioblastoma cultures to the NK92 cells is likely a combination of a lack of sufficient activation ligands and an overwhelming expression of suppressive ligands. We are currently conducting an in-depth analysis of the activation-inhibition ligand signatures to identify those interactions controlling evasion and to identify interactions we can manipulate. The favourable outcome being to lower the threshold to make the GBM cells more sensitive to NK92 attack.

### EACR2024-1253

### Unravelling the role of the circadian clock in tumour immunity using breast cancer models

*B. Hu[1,2], N. Covens[1,2], D. Laoui[1,2], H. Acloque[4], E. Hadadi[1,2,3]*

*[1]Brussels Center for Immunology, Vrije Universiteit Brussel (VUB), Brussels, Belgium*

*[2]Dendritic Cell Biology and Cancer Immunotherapy Lab, VIB Center for Inflammation Research, Brussels, Belgium*

*[3]Myeloid Cell Immunology Lab, VIB Center for Inflammation Research, Brussels, Belgium*

*[4]INRA GABI laboratory, Team Mammary Gland and Lactation (GaLac), Jouy en Josas, France*

**Introduction**

Circadian rhythms modulate of all our cellular processes and physiology to adapt to the environment. Mismatch between the timing of our external environment and intrinsic circadian clocks leads to circadian rhythm disruption (CRD). CRD and tumorigenesis are mutually interconnected: CRD is associated with increased risk of cancer while most tumours, including breast cancer, exhibit disrupted circadian rhythms. By now, CRD is known to be associated with an immunosuppressive shift within the tumour microenvironment and accelerated metastasis. Yet, the underlying mechanisms and potential therapeutic effects are poorly understood. Objective: Evaluate the impact of tumour specific clock deficiency (Bmal1 KO) on tumour characteristics and tumour immune microenvironment. Furthermore, to investigate whether tumour progression itself rewires the circadian clock in myeloid cells and how this affects their phenotype.

**Material and Methods**

We used the conditional Bmal1 KO spontaneous mammary tumour (MMTV:PyMT-Cre*Bmal1fl/fl ) and E0771 syngeneic tumour models. WT or KO mice were housed in normal light/dark (12h:12h) conditions. Sample collection were performed at matched circadian timing. At sacrifice, tumours, lung, blood, and bone marrow were collected for further analysis using flow cytometry, mRNA-seq, RT-qPCR, blood analyzer and immunostaining.

**Results and Discussions**

Data analysis is still ongoing, however, our preliminary data shows thatin Bmal1-deficient enhances the stem cell-like compartment in the tumours. As expected, similarly to CRD we observed an immunosuppressive shift with decreased levels of MHC-IIhi TAMs, along with an increase in FoxP3+CD4+ Tregs within the immune compartment. Interestingly, we found systemic dysregulation in circadian behaviour of myeloid cells in clock proficient E0771 tumour bearing mice.

**Conclusion**

The data indicate that the circadian clock has significant impact on anti-tumour immunity and tumour cells may utilize circadian dysregulation to evade immune responses.

### EACR2024-1265

### Bacteria-induced translation dysregulation in solid cancer as a source for targetable neoantigens

*S. Sagie1[1,2], T. Babu[1,], A.C. McPherson[3], D. Malko[1], M. Meisel[3], Y. Samuels[1]*

*[1]Department of Molecular Cell Biology, Weizmann Institute of Science, Rehovot Israel*

*[2]Institute of Oncology, Sheba Medical Center, Ramat Gan, Israel*

*[3]Department of Immunology, University of Pittsburgh School of Medicine, Pittsburgh, PA, USA*

**Introduction**

Traditional cancer immunotherapy targets neoantigens that are derived from tumor-specific mutations. However, many tumors have low tumor mutational burdens (TMB), limiting target availability and necessitating an expansion of the neoantigen pool.. We recently demonstrated that IFNg signaling-mediated tryptophan depletion evokes adaptive, non-canonical protein synthesis events that lead to the presentation of aberrantly translated peptides that can trigger immune responses. In addition, in the last few years, there has been an increasing appreciation of the role of the microbiome in tumor progression and response to therapy, with hundreds of bacteria species identified in many tumor types. We demonstrated that intra-tumor bacteria can enter patient-derived melanoma cells, their peptides can be presented by the tumor cells, and elicit immune reactivity which offers an additional pool of immunogenic cancer-specific peptides. This research introduces an innovative strategy using bacteria-induced tryptophan depletion to generate novel

© 2024 The Authors. Molecular Oncology is published by John Wiley & Sons Ltd on behalf of Federation of European Biochemical Societies neoantigens through non-canonical translation frameshift events in cancer cells.

**Material and Methods**

For this project, we combined, bacterial engineering, bacteria and cancer co-culture experiments, metabolomics, and immunopeptidomics with an innovative analytic pipeline for the identification of aberrant peptides.

**Results and Discussions**

We have found that bacteria such as F. Nucleatum and L.Reuteri can deplete tryptophan when cultured with cancer cells both in vitro and in vivo. This depletion is dependent on the Indole pathway and does not produce Kynurenine, an immunosuppressive metabolite.We have further established that mouse cancer cells frameshift on tryptophan codons in the absence of tryptophan, thus presenting a valid model to test this approach. Importantly, bacteria are found in murine tumors after in vivo intra-tumor injection and induce tumor rejection only with bacteria that deplete Tryptophan.

**Conclusion**

Future work will involve establishing this model and examining the production and MHC presentation of aberrant peptides after bacteria-induced tryptophan depletion in vivo.

### LATE-BREAKING ABSTRACTS

### EACR2024-0260

### Development and functional validation of distinct models of anti-KRAS extracellular vesicles for the treatment of PDAC

*D. Baranda Martínez-Abascal[1,2], I. Abasolo[1,2,3,4], H. Friedrichsen[5], M. Conceição[5], M. Fidel[1,2], D. Rafael[1,2,4], F. Andrade[1,2,6], D. Tomlinson[7], M. Wood[5], J. Seras-Franzoso[1,2,8]*

*[1]Vall d'Hebron Institute of Research, Advanced Therapies and Interventions- Nanomedicine, Transplantation and Donation - Clinical Biochemistry-Drug Delivery and Therapy Group CB-DDT, Barcelona, Spain*

*[2]Health Institute Carlos III, Web Center of Biomedical Research of Bioengineering- Biomaterials and Nanomedicine CIBER-BBN, Madrid, Spain*

*[3]Vall d'Hebron Campus Hospital, Clinical Biochemistry Service, Barcelona, Spain*

*[4]Vall d'Hebron Institute of Research- Autonomous University of Barcelona, Functional Validation & Preclinical Research FVPR/U20 ICTS Nanbiosis, Barcelona, Spain*

*[5]Institute of Developmental and Regenerative Medicine-University of Oxford, Department of Paediatrics, Oxford, United Kingdom*

*[6]Faculty of Pharmacy- University of Barcelona, Department of Pharmacy and Pharmaceutical Technology and Physicochemistry, Barcelona, Spain*

*[7]Astbury Centre for Structural and Molecular Biology-University of Leeds, School of Molecular and Cellular Biology, Leeds, United Kingdom*

*[8]Autonomous University of Barcelona, Department of Genetics and Microbiology, Barcelona, Spain*

**Introduction**

Extracellular vesicles (EVs) are increasingly being studied as drug delivery systems due to their high stability and low immunogenicity. Here we explored the loading of therapeutic affimers, small proteins mimicking antibody activity, into EVs in three distinct nanoformats according to the affimer situation within the EV architecture: extravesicular, luminal membrane-bound and luminal. Affimers were validated to inhibit difficult-to-drug oncogene KRAS. Vehiculization on EVs is expected to guarantee protection while in the blood-stream and to enhance intracellular delivery upon vesicles internalization into cancer cells. EV-affimer nanoparticles were produced following fully scalable methods and are being tested aiming to develop an innovative nanomedical treatment for pancreatic ductal adenocarcinoma.

**Material and Methods**

Endogenous loading DNA constructs were transfected into EV producing HEK293F cells. Recombinant proteins were expressed on vesicles from the outset. Through fusion to tetraspanin CD63 C-terminus affimer was located on the lumen of the vesicle, while fusion with 2$^{nd}$ loop of CD63 granted external location. Luminal, no membrane-bound, loading was reached by fusion to a peptide derived from EV soluble protein N-Syntenin. EVs were isolated by size exclusion chromatography. Constructs included mClover3 as a reporter. Cargo loading was quantified by flow cytometry as well as western blot (WB). Functional validation is done by treating model pancreatic cancer cell line MiaPaca-2 with EVs and checking KRAS signalling cascade with WB along with cytotoxicity MTT and cell cycle inhibition assays.

**Results and Discussions**

Single particle analysis by flow citometry showed an 88% of fluorescent vesicles from CD63 C-terminus construct production, while N-Syntenin construct generated 25% of fluorescent particles and CD63 2$^{nd}$ loop corresponded to a 16%. WB showed affimer concentration in CD63 C-terminal EVs was around 20 times higher than in N-Syntenin EVs and 100 times higher than in CD63 2$^{nd}$ loop EVs. In first assays, C-terminal EVs seem to reduce pERK expression and to decrease 4-5% cell cycle in MiaPaca-2.

**Conclusion**

Affimer was found into EV cargo with all tested strategies, being CD63 C-terminus loading the most effective construct. Nevertheless, other constructs are also effective and can be considered depending on the particulars of the therapeutic protein to be delivered. More therapeutic efficacy assays are needed to confirm antitumoral efficacy of C-terminal EVs and to evaluate other EV conformations.

### EACR2024-0264

### IGFBP6 as a potential regulatory node in nuclear XIAP-associated breast cancer drug resistance

*B.D.S. Mendonça[1], R. Binato[2], E. Abdelhay[2], N.S. Lima[1],*

© 2024The Authors. Molecular Oncology is published by John Wiley & Sons Ltd on behalf of Federation of European Biochemical Societies

R.C. Maia[3], R.S. Carvalho[4], G. Nestal de Moraes[5]
[1]UFRJ, IBqM, Rio de Janeiro, Brazil
[2]INCA, Centro de Transplante de Medula Óssea CEMO, Rio de Janeiro, Brazil
[3]INCA, Programa de Hemato-Oncologia Molecular, Rio de Janeiro, Brazil
[4]UFRJ, Faculdade de Farmácia, Rio de Janeiro, Brazil
[5]Universidade Federal do Rio de Janeiro, Instituto de Bioquímica Médica Leopoldo de Meis IBqM, Rio de Janeiro, Brazil

**Introduction**

X-linked inhibitor of apoptosis protein (XIAP) is an antiapoptotic protein that performs its canonical functions at the cytoplasm. However, our group has demonstrated that nuclear XIAP is associated with unfavourable clinical outcome in breast cancer as well as increased cell growth and drug resistance in vitro. Therefore, this work aimed to investigate the molecular mechanisms associated with nuclear function and localization of XIAP in breast cancer.

**Material and Methods**

To address this, we have performed both transcriptomic and proteomic large-scale approaches with breast cancer cell lines overexpressing ectopic XIAP in distinct subcellular locations. Protein and mRNA levels were examined by immunoblotting and RT-qPCR. Protein localization by immunofluorescence. Drug response was assessed by clonogenic and MTT assays. siRNA-based knockdown and gene overexpression were performed with liposomal transfections.

**Results and Discussions**

RNA microarray analysis with nuclear XIAP overexpressing cells ($XIAP^{NLS}$ – nuclear localization signal insertion) has revealed the most representative differentially expressed genes (DEGs) implicated in cellular processes relevant in cancer, such as response to stimuli, localization, communication, proliferation, transport, locomotion, migration, cell motility and protein phosphorylation. Interestingly, the validation analysis has shown differential expression of genes associated with the WNT/β-catenin pathway, as well as the *IGFBP6* tumor suppressor gene. Through in silico analysis of patients' samples, we observed *IGFBP6* expression reduced in invasive breast cancer compared to normal breast tissues. In $XIAP^{NLS}$-overexpressing cells, we observed a significant reduction in levels of *IGFBP6* transcripts compared to wildtype XIAP exhibiting cytoplasmic subcellular localization. Notably, IGFBP6 knockdown modulated long-term cell proliferation and doxorubicin sensitivity in cells overexpressing nuclear XIAP, further suggesting that nuclear XIAP might target IGFBP6 pathway to promote chemoresistance.

**Conclusion**

Collectively, our data establish a role for XIAP in the regulation of pathways related to cell proliferation in the nucleus, with IGFBP6 emerging as a potential regulatory node mediating nuclear XIAP effects in breast cancer.

### EACR2024-0364
### Repurposing Ivermectin to Enhance Chemotherapy's Effectiveness in High-Grade Serous Carcinoma

M. Nunes[1], S. Ricardo[2]
[1]Institute for Research and Innovation in Health i3S of the University of Porto, Differentiation and Cancer Group, Porto, Portugal
[2]Toxicologic Pathology Research Laboratory- University Institute of Health Sciences 1H-TOXRUN- IUCS-CESPU, Applied Molecular Biosciences Unit UCIBIO, Gandra, Portugal

**Introduction**

Standard treatment for high-grade serous carcinoma is carboplatin and paclitaxel, but it can cause resistance and harmful side effects which can be detrimental to the patient's health. A promising approach is to combine repurposed drugs with chemotherapy to improve efficacy, reduce toxicity, and overcome resistance. Ivermectin, an antiparasitic agent, may have anti-cancer properties. studies have shown that it may also have potential anti-cancer properties. In some cases, it has been found to enhance the effectiveness of chemotherapy drugs and even reverse resistance to them. Ivermectin can acts as a chemosensitizer by blocking drug efflux, increasing drug accumulation, and improving antineoplastic drugs' efficacy. In a previous study, we showed that when paclitaxel and ivermectin were combined, they produced the highest cytotoxic effect and the strongest synergism among all tested combinations for two chemoresistant cell lines. This combination had a chemotherapeutic impact superior to both drugs alone, as demonstrated in a 2D cell culture model. However, it's important to note that 2D cell culture models don't always accurately represent in vivo cell environments. Therefore, 3D cell culture technologies have been developed to conduct drug studies more precisely.

**Material and Methods**

We aimed to investigate whether combining paclitaxel with ivermectin led to a therapeutic benefit in a matrigel-based 3D cell model. To achieve this, we measured the cytotoxic effects of paclitaxel alone and in combination with ivermectin on three cell lines: two tumor chemoresistant (OVCAR8 and OVCAR8 PTX R P) and a non-tumoral (HOSE6.3) cell lines. We used CellTiter-Glo® Luminescent assays to measure cellular viability and evaluated the synergistic interactions using Zero Interaction Potency, Loewe, Bliss Independence, and Highest Single Agent reference models using SynergyFinder Plus Software.

**Results and Discussions**

Our research has discovered that combining Paclitaxel and Ivermectin has significant cytotoxic effects and robust synergism when using a matrigel-based 3D cell culture model. This effect was observed in both chemoresistant cell lines, demonstrating the potential of this two-drug combination for managing high-grade serous carcinoma.

**Conclusion**

By combining paclitaxel and ivermectin, we can potentially revolutionize the treatment of high-grade serous carcinoma. This approach offers patients a safer and more effective therapeutic option.

### EACR2024-0507
### Delving into the potential of microRNA-371-373 cluster derived from extracellular

© 2024 The Authors. Molecular Oncology is published by John Wiley & Sons Ltd on behalf of Federation of European Biochemical Societies

### vesicles as potential biomarkers in testicular germ cell tumors

N.T. Tavares[1,2], C. Lourenço[1,2,3], V. Constâncio[1,2], M. Cantante[4], I. Braga[1,5], R. Henrique[1,4,6], A. Bagrodia[7], C. Jerónimo[1,6], J. Lobo[1,4,6]
*[1]Research Center of IPO Porto CI-IPOP/RISE@ CI-IPOP Health Research Network- Portuguese Oncology Institute of Porto IPO Porto/Porto Comprehensive Cancer Center Raquel Seruca Porto. CCC Raquel Seruca- 4200-072 Porto- Portugal, Cancer Biology and Epigenetics Group, Porto, Portugal*
*[2]School of Medicine and Biomedical Sciences- University of Porto ICBAS-UP- 4050-313 Porto- Portugal, Doctoral Programme in Biomedical Sciences, Porto, Portugal*
*[3]i3S-Instituto de Investigação e Inovação em Saúde- Universidade do Porto- 4200-135 Porto- Portugal, Instituto Nacional de Engenharia Biomédica, Portugal, Portugal*
*[4]Portuguese Oncology Institute of Porto IPOPorto- 4200-072 Porto- Portugal, Department of Pathology, Porto, Portugal*
*[5]Portuguese Oncology Institute of Porto IPOPorto- 4200-072 Porto- Portugal, Department of Urology, Porto, Portugal*
*[6]School of Medicine & Biomedical Sciences- University of Porto ICBAS-UP- 4050-313 Porto- Portugal, Department of Pathology and Molecular Immunology, Porto, Portugal*
*[7]University of California San Diego- La Jolla- San Diego CA- USA, Department of Urology, San Diego, United States*

**Introduction**

Testicular germ cell tumors (TGCT) are the most common tumors in young-adult men. These tumors hold a unique molecular background and their incidence is rising. Extracellular vesicles (EVs) are membrane-derived particles with vital roles in cell communication, and EV-derived microRNAs (miRNAs), being highly protected from degradation, are considered as potential cancer biomarkers. For TGCT there is already a well-identified miRNA biomarker, miR-371a-3p, part of a germ-cell specific cluster, miR-371-373 cluster. This miRNA has been tested in different clinical settings with well-established cohorts, showing high specificity and sensitivity. Despite its remarkable clinical relevance, cell secretion dynamics from the cluster are still widely unknown, it was never tested in EV-derived setting and there are clinical questions for which this biomarker is unfit. Thus, we aim to isolate EVs from different TGCT sample types (cell lines, tissue and plasma), test the presence and levels of these 3 miRNAs in EVs and check if there is an increment in sensitivity for the plasma-derived EV-miRNA fraction.

**Material and Methods**

EVs were isolated by a differential ultracentrifugation protocol, divided into two populations (large EVs (lEV) and small EVs (sEV)) and characterized according to MISEV guidelines. A panel of 9 TGCT cell lines, patient-derived tissue explants conditioned media and matched plasmas representative of different histological types were used. Conditioned media were retrieved after cells were cultured in medium supplemented with EV-depleted fetal bovine serum, while tissue explants were cultured overnight. Plasma samples were processed routinely in our Biobank. EV-RNA was extracted and miR-371-373 cluster miRNAs were tested by RT-qPCR after preamplification for clinical samples.

**Results and Discussions**

The cell lines results revealed a pattern of EV-miRNA levels similar to the cell lysates' original RNA, corroborating our hypothesis that these miRNAs are secreted into EVs, both into lEVs and sEVs. Additionally, we were able to isolate EVs from TGCT fresh tissue (tumor and normal) conditioned media. TGCT tumor tissues released a significantly higher number of particles into the medium when compared with normal tissues, both lEV and sEV.

**Conclusion**

We were able to validate the release of miR-371a-3p in EVs from cell lines, and we are optimizing the measurement of miR-371-373 levels in EVs by RT-qPCR for clinical samples, in order to validate our findings in relevant patient samples.

### EACR2024-0556
### Differences in starvation-induced autophagy response in rat breast cancer cells and normal mammary epithelial cells

M. Gotowiec[1], A. Smoliński[1], M. Suchecki[1], W. Paskal[1], P. Włodarski[1]
*[1]Medical University of Warsaw, Department of Methodology, Warsaw, Poland*

**Introduction**

Breast cancer (BC) is one of the most heterogeneous cancers with varied molecular patterns. Autophagy is a context-dependent, conserved degradation mechanism that enables cells to withstand stressful conditions. Previous studies have shown specific molecular differences in starvation-induced autophagy response in different human BC cells, yet such were uncharacterized in rat cells commonly used as allogeneic models. The regulatory mechanism may involve dysregulation of microRNAs (miRNAs) responsible for post-transcriptional regulation of gene expression. Elucidating the miRNA expression profile of starvation-induced autophagy response in normal mammary epithelial and BC cells may shed light on new molecular targets.

**Material and Methods**

Rat mammary epithelial cells (normal) and BC cells modelling different subtypes (RBA, HH-16.cl.4, SHZ-88) were exposed to 24-hour EBSS starvation or cell medium (CM) treatment. The differences in cell proliferation (CCK-8), migration (Scratch Assay) and autophagic flux (CAT dye) between EBSS-treated and CM-treated cells were measured. STRING database was used to determine proteins involved in autophagy and starvation response and those selected were screened against known miRNA interactions using miRTargetLink 2.0 tool. Total RNA isolated from EBSS- and CM-treated cells was used to measure autophagy-associated miRNA expression changes using qPCR.

**Results and Discussions**

© 2024 The Authors. Molecular Oncology is published by John Wiley & Sons Ltd on behalf of Federation of European Biochemical Societies

EBSS-induced starvation reduced the proliferation of all cells with noticeable BC subtype-dependent differences, yet promoted BC cells migration when applied for a shorter time (6h). Autophagosome accumulation was prominent in RBA, HH-16.cl.4 and normal cells but not SHZ-88 cells exposed to chloroquine, but starvation increased autophagosome formation only in RBA and HH-16 cells. We determined a starvation-associated miRNA signature consisting of twenty-seven miRNAs, many associated with tumorigenesis and decreased survival outcomes in BC. We observed increased basal expression of several starvation-associated miRNAs in BC cells compared to normal cells and starvation-associated expression changes specific to BC cells.

**Conclusion**

Starvation-induced autophagy response in rat BC cells is not universal and potentially depends on the subtype**.** Starvation is associated with BC-specific miRNA dysregulation, showing therapeutic potential associated with autophagy modulation.

*This project was funded by the Polish Ministry of Science and Higher Education (SKN/SP/569610/2023) and the Medical University of Warsaw (69/M/MG/N/23).*

### EACR2024-0763

### Mechanosensitive polycystin proteins are implicated in glioma progression

A. Gargalionis[1], A.I. Giannopoulou[1], P. Sarantis[1], P. Korkolopoulou[2], E.J. Boviatsis[3], C. Piperi[1], A.G. Papavassiliou[1]

[1]*National and Kapodistrian University of Athens- Medical School, Department of Biological Chemistry, Athens, Greece*

[2]*National and Kapodistrian University of Athens- Medical School, First Department of Pathology, Athens, Greece*

[3]*National and Kapodistrian University of Athens- Medical School, Second Department of Neurosurgery, Attikon University General Hospital, Athens, Greece*

**Introduction**

Accumulating data highlight the important role played by mechanical forces in solid tumors development, invasion, and metastasis. Neoplastic cells bear mechanosensing proteins, such as the polycystin family members, that recognize changes in mechanical tension/stiffness in their microenvironment and are involved in signaling pathways regulating carcinogenesis. Our aim was to study cellular localization, expression, and potential correlations of polycystins with clinical and pathological parameters in a patient population with gliomas, and to investigate underpinning molecular mechanisms.

**Material and Methods**

Immunohistochemical analysis of the expression of polycystin-1 (PC1) and polycystin-2 (PC2) was performed in a patient cohort of 87 patients for PC1 and 82 patients for PC2 operated for brain glioma. The *IDH* mutation status of the tumors was also evaluated. PC1 expression was detected in neoplastic glioma cell lines T98G and GOS-3, as well as in normal epithelial tissue.

**Results and Discussions**

PC1 displayed cytoplasmic and, rarely, nuclear localization in gliomas. PC2 demonstrated cytoplasmic and frequent nuclear localization. Both proteins exhibit increased expression in the peri-necrotic and peri-vascular areas, suggesting a correlation of their over-expression with the endothelial hyperplasia that characterizes the hypoxic environment of the tumor. Statistical analysis of PC1 and PC2 expression revealed associations with pathologic parameters. PC1 and PC2 share a strong positive correlation of their expression in gliomas ($p$=<0.001). PC2 shows increased expression in grade 3 and 4 tumors compared to grade 2 tumors ($p$= 0.007). PC1 shows reduced expression in glioma cells compared to normal epithelial cells.

**Conclusion**

Mechanosensitive PC1 and PC2 share a strong positive correlation in glioma tissues and this suggests their potential synergy in this tumor type. Furthermore, polycystins' expression in peri-necrotic areas implies their potential association with hypoxic factors affecting angiogenesis. Increased expression of PC2 in high-grade tumors suggests its potential involvement in aggressive oncogenic features. Conclusively, polycystins are implicated in glioma progression.

*The research project was supported by the Hellenic Foundation for Research and Innovation (H.F.R.I.) under the "2nd Call for H.F.R.I. Research Projects to support Faculty Members & Researchers" (Project Number: 3226).*

### EACR2024-0782

### Cytotoxic and cytostatic effect of a flavone formulation derived from olive leaf in breast cancer cellular models

M. Losada-Echeberría[1], P. de Juan-Macià[1], M. Pavlíková[2], G. Luengo-Gil[2], P. Conesa-Zamora[2], V. Micol[1], E. Barrajón-Catalán[1]

[1]*Instituto de Investigación- Desarrollo e Innovación en Biotecnología Sanitaria de Elche IDiBE, Design and development of bioactive molecules, Elche, Spain*

[2]*Instituto Murciano de Investigación Biosanitaria, Patología Molecular y Farmacogenética, Cartagena, Spain*

**Introduction**

Breast cancer (BC) is the most common tumor in women and the last year 2 million new cases were diagnosed worldwide. The new therapies decreased the mortality but its necessary a continuous searching for new therapies for cases with the worst prognosis, like triple negative BC (TNBC). Polyphenols have emerged as one of the main families of compounds with potential therapeutic activity in many diseases. In previous studies, it was determined in vitro antiproliferative activity of an olive leaf extract and the main compounds responsible of this activity were diosmetin, apigenin and luteolin.

In this work, the antiproliferative activity of the mixture of flavones, diosmetin, apigenin and luteolin (DAL511) was tested in BC cell models of the three main molecular subtypes.

**Material and Methods**

Cell lines: MCF10A (no tumoral mammary epithelium), MCF7 (Luminal), JIMT-1 (HER2+) and MDAMB231 (TNBC). Clonogenic capacity: The assay was performed with a low-density seed, crystal violet dye. Migration and invasion capacity: The wound healing assays were performed with Hoechst-33342 dye and matrigel GFR (Corning) in the invasion assay. Cell-cycle analysis: The

© 2024 The Authors. Molecular Oncology is published by John Wiley & Sons Ltd on behalf of Federation of European Biochemical Societies assay was performed using the Cell Cycle kit (Millipore). Mitochondrial membrane potential: The assay was performed using the MitoPotential kit (Millipore). Apoptosis analysis: The assay was performed by utilizing the Annexin V and Dead Cell Kit (Millipore). Reactive Oxygen Species (ROS) production: The assay was performed using the dichloro-fluorescein diacetate dye.

**Results and Discussions**

The results in the three cell lines for the DAL511 treatment show a decrease in clonogenic capacity as well as in migration and invasion. The results in MCF7 and MDAMB231 cell lines show an increase in the percentage of cells in the subG0, which corresponds to a cytotoxic effect and this correlates with the increase in the early and late apoptosis and the loss of the mito-chondrial membrane potential. In addition, the ROS production show an increase in both cell lines. For the JIMT1 cell lines the results show mainly an arrest in the G2/M phase of the cell cycle which corresponds to a cytostatic effect and secondarily an increase in the percentage of cells in the subG0 corresponding with the apoptotic cells and the cells with mitochondrial depolarization observed.

**Conclusion**

The combination DAL511 has cytotoxic and cytostatic effect in BC cellular models. The cytotoxic mechanism of the DAL511 mixture is the apoptotic effect with mitochondrial involvement and an increase in ROS production.

### EACR2024-0857
### Exploring the Therapeutic Potential of Marine Compounds in the Tumor Microenvironment of HCT-116 Cells and Their Effects on Epithelial-Mesenchymal Transition

*P. de Juan Maciá*[1], *M. Losada-Echeberría*[1], *E. Barrajón-Catalán*[1], *V. Micol*[1], *M. Herranz-López*[1]

[1]*Instituto de investigación- desarrollo e innovación en biotecnología sanitaria- de elche IDIBE, Universidad Miguel Hernández, Elche, Spain*

**Introduction**

This study focuses into the investigation of the tumor microenvironment (TME) of colon cancer at the in vitro level, focusing on the interplay between adipose tissue surrounding the tumor, adipocytes, and colon epithelial cells. The research aims to elucidate cell communication dynamics and their impact on metabolic changes, including the Warburg effect, the reverse Warburg effect, and the epithelial-mesenchymal transition (EMT). Furthermore, the study analyzes the therapeutic potential of two marine compounds (referred to as Compound 1 and Compound 2) against these conditions. Recognizing the pivotal role of adipose tissue in shaping the TME, the research also investigates the influence of obesity on these interactions.

**Material and Methods**

The study conducted in vitro assays to explore the dynamics of the tumor microenvironment (TME) in colon cancer. HCT-116 cells were co-cultured with adipocytes to mimic TME conditions, resulting in the formation of colonospheres. These colonospheres were then treated with Compound 1 and Compound 2, followed by the assessment of various TME and epithelial-mesenchymal transition (EMT) markers. Molecular analyses, including RT-qPCR, Western blot, and Luminex assays, were employed to evaluate the effects of the compounds.

**Results and Discussions**

The findings reveal a notable alteration in the morphology of tumor cells, resulting in the formation of colonospheres, indicative of tumor growth in the tumor microenvironment (TME). Concurrently, an upregulation in the expression of the molecular marker N-cadherin, associated with epithelial-mesenchymal transition (EMT), was observed, underscoring the phenotypic changes occurring within the TME. A change in the metabolism of the tumour line due to the micro-environment generated by the adipocytes has also been observed, showing a possible Warburg effect. Importantly, the administration of the compounds resulted in a reduction in the number of colonospheres and cancer cell viability. Furthermore, the compounds exhibited the ability to attenuate the expression of certain markers within the microenvironment, highlighting their therapeutic potential in combating colon cancer progression

**Conclusion**

The study has observed an assay that replicates the communication between adipocytes and tumor cells, and it has observed the therapeutic potential of two marine compounds.

### EACR2024-0904
### Unveiling Chemotherapy-induced Metabolic Rearrangement in Drug-resistant Medulloblastoma

*L. Manfreda*[1], *B. Marzullo*[2], *E. Rampazzo*[3], *C. Marchioro*[3], *M. Canton*[3], *E. Mariotto*[3], *L. Persano*[3], *G. Viola*[3], *D. Tennant*[2], *R. Bortolozzi*[4]

[1]*University of Padova, Department of Women's and Children's Health, Padova, Italy*

[2]*University of Birmingham, Institute of Metabolism and Systems Research, Birmingham, United Kingdom*

[3]*University of Padova, Department of Women's and Children's Health, Padova, Italy*

[4]*University of Padova, Department of Pharmaceutical and Pharmacological Sciences, Padova, Italy*

**Introduction**

Medulloblastoma (MB) is the most common brain tumor in childhood, notorious for its high recurrence rates post-treatment and the debilitating neurocognitive and endocrine complications linked to aggressive chemo-therapy. The acquisition of drug resistance by MB cells remains a primary factor contributing to treatment failure. To dissect the intricate interplay among tumor treatment, emergence of drug resistance, and tumor relapse initiation, we established an in vitro model of MB resistance by subjecting medulloblastoma cells to a combination of chemotherapeutic agents: Vincristine, Etoposide, Cisplatin, and Cyclophosphamide (VECC).

**Material and Methods**

We employed LC-MS metabolomic approach to assess metabolite levels in both cells and media. Additionally,

© 2024 The Authors. Molecular Oncology is published by John Wiley & Sons Ltd on behalf of Federation of European Biochemical Societies mitochondrial morphology and cellular lipid content were evaluated through flow cytometry and confocal microscopy using MitotrackerGreen/DeepRed and Bodipy493-503, respectively. All these analyses were underpinned by protein expression data, and metabolic profiling using the Seahorse XFe96 Analyzer.

**Results and Discussions**

Multi-omics data analysis underscored metabolism as a highly deregulated hallmark in MB-resistant cells. Notable enrichments were observed in fatty acid metabolism, amino acid metabolism, pyruvate metabolism, the tricarboxylic acid (TCA) cycle, mitochondrial biogenesis, and oxidative phosphorylation. Metabolomic analysis and isotope tracing with [U]13C-glutamine and [U]13C-glucose validated a significant metabolic rewiring between naïve and resistant cells. Specifically, we observed modulation in nucleotide metabolism with a reduction of nucleotide catabolism and increased nucleotide synthesis but also a strong increase in amino acid uptake and utilization. These findings are consistent with the high-throughput drug screening we previously published, which pointed out increased sensitivity of resistant cells to antimetabolites

**Conclusion**

Recent studies emphasize the importance of metabolic plasticity in cancer cells for adapting to chemotherapy. In this context, our data supports the idea that chemotolerant MB cells, through the deregulation of several metabolic pathways, can counteract chemotherapy-induced stress by maintaining high antioxidant capacity and macromolecule biosynthesis. The specific identification of how and why sensitive cells can modulate their metabolism to adapt to chemotherapy resistance at first will allow us to identify a metabolic vulnerability to be used to make this metabolic advantage into their Achilles hell.

### EACR2024-0923
### Machine Learning Analysis and Risk Score Determination of Kallikrein, Osteopontin and VEGF Proteins in the Early Diagnosis of Ovarian Cancer

*E. Gumusoglu-Acar[1], K. Kara[1], S. Cevik[1], H. Aytar[1], S. Topuz[2], T. Gunel[3]*
*[1]Istanbul University, Molecular Biology and Genetics, Istanbul, Turkiye*
*[2]Istanbul University, Faculty of Medicine / Gynocologic Oncology, Istanbul, Turkiye*
*[3]Istanbul University, Molecular biology and Genetics, İstanbul, Turkiye*

**Introduction**

Ovarian cancer (OC) is uncontrolled cell division and proliferation that takes place in the epithelium or embryonic cells in the ovaries of women. As is known, in the early stages, ovarian cancer doesn't show any symptoms which makes it difficult to diagnose until the advanced stages. Although many methods are used in the diagnosis of ovarian cancer, studies have focused on biomarkers because of their accurate diagnostic potential. A few protein biomarkers used in the diagnosis of ovarian cancer; are CA-125, osteopontin (OPN), HE4, VEGF (Vascular Endothelial Growth Factor), and Kallikreins (KLK). In this study, it is aimed to analyze the circulation levels of these proteins in OC patients and healthy controls. Afterward, a risk score for each individual will be determined using machine-learning methods to give an idea about disease diagnosis and progression.

**Material and Methods**

In this project, clinical data such as age, pathological status, cancer stage, and blood samples were obtained from the patients. Blood samples were drawn from 13 patients with serous papillary adenocarcinoma ovarian cancer and 30 healthy individuals. Protein isolation and quantification were performed on the obtained samples. Antigen-antibody binding was tested using the ELISA (Enzyme Immunoassay and Enzyme-Linked Immunosorbent Assay) method on isolated VEGF, Osteopontin, and KLK proteins. Clinical data and protein analysis results were analyzed with the Decision Tree method, which is a bioinformatics analysis and artificial intelligence method. As a result of all these steps, preliminary information and risk score data were planned to be provided to gynecologist oncologists.

**Results and Discussions**

Spectrophotometric readings of KLK and OPN proteins indicated insignificant results. We found lower serum VEGF levels in healthy women compared with those in women with ovarian cancer. The overall median VEGF concentration (range) was 515.2 pg/ml in ovarian cancer patients.

**Conclusion**

In light of these findings, it can be concluded that there are difficulties in detecting the proteins examined in the blood.  Between these proteins only VEGF protein is suitable for the early diagnosis of ovarian cancer. The evaluation of clinical data using bioinformatic analyses from patients diagnosed with serous papillary adenocarcinoma ovarian cancer will illuminate the way for screening and early diagnosis of ovarian cancer with user-friendly tools for potential patients.

### EACR2024-1013
### Advanced spatial biology techniques elucidate mechanisms of uza-cel's TCR-directed anti-tumor activity

*R. Broad[1], A. Liddle[1], R. Naidoo[1], M. Isabelle[1], A. Tipping[1], K. Kaye[2], J. Campos[3], K. Hardikar[3], C. Evans[4]*
*[1]Adaptimmune, Translational Sciences, Abingdon, United Kingdom*
*[2]Adaptimmune, Translational Sciences, Cambridge, United States*
*[3]Propath UK, Molecular Pathology, Hereford, United Kingdom*
*[4]Adaptimmune, Translational Sciences, Philadelphia, United States*

**Introduction**

Uzatresgene autoleucel ("uza-cel", formerly ADP-A2M4CD8), a next-generation T-cell receptor (TCR) T-cell therapy modified with a CD8α co-receptor alongside a MAGE-A4–specific TCR, is being investigated for the treatment of HLA*02–eligible subjects with advanced solid tumors. Advanced spatial biology assays were employed to characterize the tumor microenvironment

Molecular Oncology 18 (Suppl. 1) (2024) 1–495
© 2024 The Authors. Molecular Oncology is published by John Wiley & Sons Ltd on behalf of Federation of European Biochemical Societies

(TME) and elucidate mechanisms of anti-tumor activity and drivers of response.

**Material and Methods**

Multiplex immunofluorescence (mIF) and spatial transcriptomics approaches were employed to assess TME characteristics from a preliminary dataset of baseline and post-infusion biopsies collected from urothelial, ovarian and esophageal patients from the SURPASS clinical trial (NCT04044859). These spatial biology approaches offer the advantage of preserving the architectural features of the TME, whilst revealing the spatial relationships between tumor and immune cells and capturing the positional context of transcriptional activity.

**Results and Discussions**

The data presented demonstrates that uza-cel initiates a broad intra-tumoral adaptive and innate immune response with evidence of activation of both engineered and endogenous T-cells. Spatial transcriptomics employed across these samples reveals an enrichment of HLA-DRA in responding baseline biopsies. Further enrichment of HLA-DRB4, as well as B2M, TAP1 and TAP2, was also observed in responders post-infusion biopsies. Furthermore, CD45+ and PanCK/CD45+ mixed segments at baseline show higher expression of HLA-DRB4 in responders with further increases post-infusion. No differences were observed in PanCK+ only segments. This suggests HLA-DRB4 enrichment is driven by CD45+ cells and is a marker of response. Within these HLA-DR enriched regions associated with response, there is clear evidence of uza-cel infiltration and increased granzyme B activity with higher densities of HLA-DR+ T-cells noted in responders from mIF analysis. This highlights activation of both endogenous T-cells and uza-cel engineered T-cells.

**Conclusion**

Through advanced spatial biology analysis, we observed infiltration and broad activation of both uza-cel and endogenous T-cells into solid tumors, corresponding upregulated Antigen Processing machinery and prominent tumor-directed killing by these T-cells. Additionally, HLA-DR enrichment at baseline and post-infusion highlights a potential marker of response to uza-cel. Our data provides further translational insight into the TME and anti-tumor activity observed in uza-cel patient biopsies.

### EACR2024-1024
### Investigating the effects of beta-adrenergic blockade on sarcoma patient-derived tumor-infiltrating lymphocytes

*M. Schattefor[1], D. Hargbøl Madsen[1], K. Yixin Fjæstad[1], M. Nielsen[1]*

*[1]National Center for Cancer Immune Therapy CCIT-DK, Department of Oncology, Herlev, Denmark*

**Introduction**

Research at the intersection of cancer immunotherapy and neurobiology has illuminated the critical role of beta-adrenergic signaling in the tumor microenvironment. Tumor-infiltrating lymphocyte (TIL) therapy, a promising approach harnessing the immune system to combat cancer, faces challenges in overcoming the immunosuppressive tumor milieu. Beta-adrenergic signaling mediated by norepinephrine and epinephrine, influences immune cells within tumors, exacerbating immunosuppression. Norepinephrine and epinephrine, released under stress conditions, stimulate beta-adrenergic receptors on immune cells, which can dampen anti-tumor responses and foster a tolerogenic environment. The non-selective beta-blocker propranolol offers a strategic approach to counteract these effects, potentially enhancing the efficacy of TIL therapy. Understanding the complex interplay between beta-adrenergic signaling and T cell function is pivotal for advancing TIL therapy. We aim to investigate whether intratumoral epinephrine and norepinephrine act as a limiting factor for TIL therapy efficacy. By elucidating these mechanisms, we aim to devise strategies to mitigate the immunosuppressive effects of beta-adrenergic signaling and enhance the success of TIL therapy in combating cancer.

**Material and Methods**

Sarcoma patient samples were retrieved from the Center for Cancer Immune Therapy (CCIT-DK) biobank, with all patients providing written informed consent. These tissue fragments were cultured in RPMI-1640 media, 1% penicillin-streptomycin, 10% human serum, 1.25 µg/mL Fungizone and subsequently stimulated with 3000 IU/mL Interleukin-2. Cells were continuously treated with 10 µM propranolol, whereas the control group remained untreated. Following a minimum incubation period of three weeks, the cells were harvested and stained for flow cytometric analysis. This encompassed markers to discern various T cell populations, along with markers indicative of exhaustion and activation states.

**Results and Discussions**

Following a three-week period, we successfully expanded 8-9 million TILs per condition, for one patient thus far. Subsequent flow cytometric analysis unveiled a notable augmentation in the percentage of CD3+ CD4+ T cells, accompanied by a decrease in the CD3+ CD4- CD8- population. Additionally, we observed an increase in TCRαβ and CD27 expression. This experiment will be repeated for additional patients, to allow for statistical analysis.

**Conclusion**

Results remain inconclusive due to a lack of sufficient data.

### EACR2024-1039
### Unveiling ALDH-Mediated Stemness: Novel CSC-Targeted Photodynamic Therapy for Endometrial Cancer

*B. Serambeque[1,2], B. Costa[1,2,3], R. Teixo[1,2], S. M. Lopes[3], C. Almeida-Ferreira[1,2,], N.A.M. Pereira[3], M. Pineiro[3], M. F. Botelho[1,2,4], T. M. V. D. Pinho e Melo[3], M. J. Carvalho[1,2,5,6], M. Laranjo[1,2,4]*

*[1]Univ Coimbra, Coimbra Institute for Clinical and Biomedical Research iCBR Area of Environment-Genetics and Oncobiology CIMAGO- Institute of Biophysics- Faculty of Medicine, Coimbra, Portugal*

*[2]Univ Coimbra, Center for Innovative Biomedicine and Biotechnology CIBB, Coimbra, Portugal*

*[3]Univ Coimbra, Coimbra Chemistry Centre-Institute of Molecular Sciences and Department of Chemistry, Coimbra, Portugal*

© 2024 The Authors. Molecular Oncology is published by John Wiley & Sons Ltd on behalf of Federation of European Biochemical Societies

[4]Clinical Academic Center, Coimbra, Coimbra, Portugal
[5]Univ Coimbra, Universitary Clinic of Gynecology- Faculty of Medicine, Coimbra, Portugal
[6]Gynecology Service, Department of Gynecology- Obstetrics- Reproduction and Neonatology- Unidade Local de Saúde de Coimbra, Coimbra, Portugal

**Introduction**

In endometrial cancer (EC), the increased expression and activity of aldehyde dehydrogenase (ALDH) mediate stemness, suggesting that this enzyme holds potential as a biomarker and could be a desirable therapeutic target. Thus, to investigate a novel conservative and less invasive therapy for EC, we developed a cancer stem cells (CSC)-targeted photodynamic therapy (PDT) approach (A-PX-based PDT), through the structural modulation of our 4,5,6,7-tetrahydropyrazolo[1,5-a]pyridine-fused chlorins (PX) with aldehyde moieties (A-PX), and its therapeutic potential will be explored.

**Material and Methods**

Endometrial CSC were obtained from ECC-1 and RL95-2 cell lines through a 5-days sphere-forming protocol. To determine the drug-light interval (DLI), endometrial CSC were incubated with 5 μM of three derivatives of PX, a dialdehyde (A-PX1) and two mono-aldehydes (A-PX2/3), for 2-24 hours. The fluorescence intensity was measured by fluorimetry. In parallel, confocal microscopy was performed to verify the PX/A-PX internalization into endometrial CSC. To evaluate the efficacy of A-PX on endometrial CSC, spheres were incubated with A-PX (1-10 μM), followed by a light activation, and cell viability and metabolic activity was evaluated through a luminescent viability assay and a resazurin assay, respectively.

**Results and Discussions**

The preliminary findings of endometrial CSC uptake showed internalizations of up to approximately 100 nM, indicating a DLI of two hours. Confocal studies suggest that PX1 (a dihydroxymethyl derivative) and A-PX exhibit the ability to internalize consistently into CSC. Early data revealed that PX seems to decrease the metabolic activity of endometrial CSC. A-PX seem to induce a reduction in metabolic activity and cell viability of endometrial CSC, showing a higher effect on RL95-2 CSC, and displaying a concentration-dependent outcome.

**Conclusion**

The preliminary results suggest that A-PX-based PDT could be a promising therapeutic modality for EC, encouraging further exploration of its potential to eliminate endometrial CSC.

*Funding: CIBB strategic projects UIDB/04539/2020 (DOI: 10.54499/UIDB/04539/2020), UIDP/04539/2020 (DOI: 10.54499/UIDP/04539/2020) and Associated Laboratory funding LA/P/0058/2020 (DOI: 10.54499/LA/P/0058/2020) from FCT. CQC Strategic Projects UIDB/00313/2020 and UIDP/00313/2020 from FCT, PTDC/QUI-QOR/0103/2021 (FCT, I.P./MCTES, PIDDAC). PhD Scholarship from FCT/European Social Fund to BS (2020.07672.BD), BC (2022.12013.BD), and RT (SFRH/BD/116794/2016).*

### EACR2024-1058
### The role of SPARC in the induction of CAFs via endothelial-to-mesenchymal transition and its prognostic value for metastatic melanoma response to treatment

*D. Lechevalier[1], E. Romone[1], A. Vrobelova[1], L. Martin[2], Y. Le Corre[2], N. Clere[1]*
[1]MINT, Umr INSERM 1066 - CNRS 6021, Angers, France
[2]University Hospital of Angers, Dermatology, Angers, France

**Introduction**

Cancer-Associated-Fibroblasts (CAFs) are known to be a part of therapeutic resistance in metastatic melanoma. CAFs can originate from endothelial cells (ECs) through endothelial-to-mesenchymal transition (EndMT). During this process, ECs lose their endothelial markers and acquire mesenchymal traits. SPARC protein is described as an inducer of different tumorigenesis processes including epithelial-to-mesenchymal transition in melanoma. Our aim was to investigate its potential role in EndMT thus in the formation of CAFs and therapeutic resistance in melanoma.

**Material and Methods**

Conditioned medium (CM) from SKMEL28 melanoma cell line was used to treat HUVECs for 48 hours and Western-Blot (WB) was used to confirm the expression of mesenchymal (α-SMA, SM22α) and endothelial proteins (vWF, VEGFR2). Flow cytometry (FC) was used to visualize double-stained cells α-SMA$^+$/vWF$^+$. The role of SPARC was adressed using SPARC-knocked-out SKMEL28 cells, that was confirmed with WB and ELISA analysis. Patients were included from the Dermatology unit of University Hospital. This study was authorized by legal french institution and all patients gave written consent. Inclusion criteria were patients treated for stage III/IV melanoma. Blood samples for SPARC measures by ELISA were retrieved at the beginning of the treatment and every 3 months, so was therapeutic response.

**Results and Discussions**

WB confirmed the expression of SPARC in SKMEL28 SPARC $^{+/+}$ cells and its lack in KO SKMEL28 cells. Secretoma analysis showed a concentration of SPARC around 72 ng/mL in SKMEL28 Sparc$^{+/+}$ and around 4 ng/mL for SKMEL28 Sparc $^{-/-}$. An increase in α-SMA and SM22α expression was found in HUVECs treated with CM from SKMEL28 SPARC $^{+/+}$ in comparison with control condition. A slight decrease in vWF and VEGFR2 expression was observed. FC showed an increase in α-SMA$^+$/vWF$^+$ HUVECs after treatment. No difference in the expression of endothelial or mesenchymal markers was described in HUVECs treated with CM from SKMEL28$^{-/-}$. 50 patients were included. We found no correlation between SPARC concentrations and response to treatment.

**Conclusion**

CM from SKMEL28 induced a partial EndMT of HUVECS. While high SPARC level was found in CM, it appears that the inhibition of this protein in SKMEL28 did not influence EndMT. These data suggest that other signaling pathways need to be investigated. SPARC does not seem to be a predictive marker for treatment response. A high concentration could reflect an agressive disease, that could benefit from BRAF/MEK inhibitors.

### EACR2024-1074
### Role of BUD23, a key ribosome

© 2024 The Authors. Molecular Oncology is published by John Wiley & Sons Ltd on behalf of Federation of European Biochemical Societies

### biogenesis factor, in cellular senescence and tumor initiation

*K. ZHU*[1], *V. Juric*[2], *J.M. Flaman*[1], *L. Baillon*[3], *J. Czarnecka-Herok*[1], *J.J. Diaz*[3], *F. Catez*[3], *J. Ablain*[2], *D. Bernard*[1], *N. Martin*[1]
[1]*Cancer Research Center of Lyon- Inserm U1052- CNRS UMR 5286- Université de Lyon- Centre Léon Bérard- Equipe Labellisée La Ligue Contre Le Cancer, «Cellular senescence- cancer and aging» team, Lyon, France*
[2]*Cancer Research Center of Lyon- Inserm U1052- CNRS UMR 5286- Université de Lyon- Centre Léon Bérard, «Adhesion and signaling in metastatic melanoma» team, Lyon, France*
[3]*Cancer Research Center of Lyon- Inserm U1052- CNRS UMR 5286- Université de Lyon- Centre Léon Bérard, «Ribosome- translation and cancer» team, Lyon, France*

**Introduction**

Cellular senescence is a stable cell proliferation arrest induced by many stresses and playing a crucial role in many physio-pathological contexts. Senescent cells present a secretome named senescence-associated secretory phenotype (SASP) and also display a senescence-associated β-galactosidase (SA-β-gal) activity. In response to pro-tumoral signals, cellular senescence is induced and acts as a critical anti-tumoral barrier that needs to be bypassed for tumor development. It is therefore crucial to understand how senescence is controlled. By a targeted loss-of-function screening we identified a new regulator of senescence, BUD23 (rRNA methyltransferase and ribosome maturation factor).

**Material and Methods**

siRNA silencing and viral transduction technologies were used for gene expression level modulation. Transcriptome analysis and real-time quantitative PCR were used to analyze mRNA levels and Western blot was used for protein detection. Cellular senescence was characterized by SA-β-gal staining, crystal violet staining and cell count. The TCGA database was used to study BUD23 expression in cancer.

**Results and Discussions**

We observed that BUD23 knockdown triggers premature senescence in MRC5 human fibroblasts. Loss of BUD23 induces ribosome imbalance and the impaired ribosome biogenesis checkpoint, which activates p53. We further observed that BUD23 protein level is downregulated in RAF oncogene-induced senescence and that BUD23 overexpression promotes a bypass of this senescence. According to TCGA database, BUD23 expression is higher in skin cutaneous melanoma with mutated BRAF than wild type BRAF. We further showed that BUD23 knockdown induces senescence in the BRAF-mutated melanoma cell line COLO679, suggesting that BUD23 could play a role in melanomagenesis.

**Conclusion**

We identified BUD23, a key ribosome biogenesis factor, as a novel senescence regulator. This work will help to understand the molecular and cellular basis of cellular senescence and tumor initiation, especially in melanoma.

### EACR2024-1084
### Mitochondrial Transfer from Cancer cells to Immune cells: A new cancer immune evasion mechanism?

*C. Fuentes*[1,2], *N. Georges*[1,2], *F. Flores-Santibañez*[2], *N. Merino-Veliz*[3], *C. Ahumada-Marchant*[3], *G. Arriagada*[3], *F.J. Bustos*[3], *F. Alcayaga-Miranda*[1,2]
[1]*Universidad de los Andes, Laboratory of Nano-Regenerative Medicine- Biomedical Innovation and Research Center- Faculty of Medicine, Santiago, Chile*
[2]*IMPACT, Center of Interventional Medicine for Precision and Advanced Cellular therapy, Santiago, Chile*
[3]*Universidad Andres Bello, Biomedical Science Institute- Faculty of Medicine, Santiago, Chile*

**Introduction**

The tumor microenvironment is a hostile site, where cancer cells have developed several mechanisms to thrive. Among these, nutrient deprivation, hypoxia, and the presence of immunoregulatory populations cooperate in cancer immune evasion allowing the establishment of tumor cells and cancer progression. Recent data has shown that tumor cells hijack mitochondria (MT) from surrounding immune populations increasing their survival, their proliferation rate and inducing metabolic changes promoting cancer development. Although this MT transfer is suggested to be unidirectional, from immune cells towards cancer cells, recent data from our group has shown that melanoma cells do release extracellular MT. Hence, we aimed to study the effect of tumor derived extracellular mitochondria (t-MT) on tumor infiltrating immune cells.

**Material and Methods**

B16-F10 cells were transformed to express vexGFP fluorescent protein on their MT (B16 mitoGFP), allowing the tracking of mitochondrial transfer. For in vivo assays, C57BL/6 mice were inoculated with B16 mitoGFP cells SC. After 14-18 days, mice were sacrificed, and tumors were processed and labeled for flow cytometry (FACS) analysis. Additionally, confocal microscopy was performed to validate the internalization of t-MT by immune cells. For in vitro assays, T cells from C57BL/6 mice, polyclonally activated, were co-cultured with B16 mitoGFP cells. t-MT transfer and immune cell phenotype was analyzed using FACS.

**Results and Discussions**

Interestingly, tumor associated macrophages, tumor infiltrating myeloid derived cells and tumor infiltrating T cells, showed the presence of $GFP^+$ events in tumors, indicating t-MT transfer to diverse immune populations. Since, $CD8^+$ T cells have a pivotal role on the antitumoral response, we focus our research on this key immune cell population. Stinkingly, $CD8^+$ T cell phenotype analysis after B16mitoGFP coculture, suggests a higher frequency of inhibitory receptors Lag-3 and PD-1 expression on the population that has internalized t-MT. Therefore, this process may be involved in T cell exhaustion, a key process on tumor immune evasion.

**Conclusion**

Even at low frequencies, to our current knowledge, these results are the first data showing that immune cells, do internalize t-MT in vivo. Future studies will allow us to describe the effect this transfer has on tumor infiltrating immune cells.

*ACKNOWLEDGEMENTS: ANID-BDN#2022-21220451; FONDECYT#1230875; ANID-BASAL#FB210024. There are no financial conflicts of interest to disclose.*

© 2024The Authors. Molecular Oncology is published by John Wiley & Sons Ltd on behalf of Federation of European Biochemical Societies

### EACR2024-1174
### Contribution of Rb1 and p53 pathway signaling alterations to the development of the Primitive Neuronal Component in Glioblastoma

*E. Somenza*[1], *F. Pagani*[2], *M. Gryzik*[1], *R. Ronca*[1], *P.L. Poliani*[3]
[1]*University of Brescia, Department of Molecular and Translational Medicine, Brescia, Italy*
[2]*Universitätsklinikum Hamburg-Eppendorf, Laboratory for Translational Research, Hamburg, Germany*
[3]*IRCCS San Raffaele, Pathology Unit, Milan, Italy*

**Introduction**

GBM-PNC is a rare histological variant of glioblastoma with nodules of poorly differentiated primitive cells, expressing less GFAP glial marker, elevated Ki-67 proliferation index, and primitive neuronal features, such as increased propensity for cerebrospinal fluid dissemination. In our cohort, 18/24 GBM-PNCs harbor cell cycle control impairment with disrupted Retinoblastoma-associated protein 1 (RB1) tumour suppressor function and either TP53 mutation or Mdm2-4 amplification. We established in vitro models to test our hypothesis that both p53 and Rb1 pathway signaling impairments are required to commit a conventional GBM toward the gain of a GBM-PNC phenotype.

**Material and Methods**

We selected two RB1 wildtype conventional GBM stem cell populations (GBM GSCs), one harboring a non-synonymous TP53 mutation, and designed a CRISPR/Cas9 lentiviral vector to target *RB1* exon 2. Mocks and knockout neurospheres were tested for proliferation, invasion, apoptosis, stemness and cell cycle assay. In vitro tumorigenesis was modelled with 3D organoids, cultured in Matrigel for 15 days, processed (formalin-fixed and paraffin-embedded) and stained for the phenotypic expression of the main glial and neuronal markers.

**Results and Discussions**

Interestingly, all TP53mut RB1 -/- organoids significantly downregulated GFAP glial marker expression and maintained high β-III-tubulin neuronal marker expression as compared to the mock clones. As expected, no markers modulation was observed in the TP53wt RB1-/- controls. Moreover, the cell cycle assay showed an increasing polyploidy in TP53mut RB1 -/- clones, reminiscent of the mitotic aberrations typical of the GBM-PNC phenotype, characterized by multinucleated cells and apoptotic processes.

**Conclusion**

Our results confirm that RB1 loss alone is not enough to trigger the switching of a conventional GBM into a GBM-PNC biphasic phenotype. Rather, we demonstrate that the concomitant alterations of p53 pathway control and RB1 function, disrupting cell cycle control, could represent the predisposing feature for a conventional GBM to become a GBM-PNC. As a matter of fact, the observed reduced glial phenotype and the increased polyploidy in TP53mut RB1 -/- GSCs clones could reflect the development of a primitive neuronal component and the typical chromosomal aberrations. Given our data, we aim to confirm that our molecular models are suitable for the study of the GBM-PNC origin, investigating the axis RB1-Early B-cell Factor 3 that we identified (paper in draft) as a novel booster of the PNC component.

### EACR2024-1178
### Understanding EV cancer communication through proteomic signature: from tissue to liquid biopsies

*C. Lourenço*[1,2,3], *N.T. Tavares*[1,2], *V. Constâncio*[1,2], *S. Monteiro-Reis*[1,4], *S. Paulino*[1,5], *F. Lobo*[6], *Â. Rodrigues*[5], *Â. Carvalho*[7], *C. Jerónimo*[1,8]
[1]*Research Center of IPO Porto CI-IPOP/RISE@CI-IPOP Health Research Network- Portuguese Oncology Institute of Porto IPO Porto/Porto Comprehensive Cancer Center Raquel Seruca Porto. CCC Raquel Seruca, Cancer Biology and Epigenetics Group, Porto, Portugal*
[2]*School Medicine and Biomedical Sciences- University of Porto ICBAS-UP, Doctoral Programme in Biomedical Sciences, Porto, Portugal*
[3]*i3S-Instituto de Investigação e Inovação em Saúde-Universidade do Porto, INEB-Instituto de Engenharia Biomédica- Universidade do Porto, Porto, Portugal*
[4]*Faculty of Engineering University of Porto- Campus FEUP, INEGI-Laeta, Porto, Portugal*
[5]*Portuguese Oncology Institute of Porto IPO Porto, Department of Pathology, Porto, Portugal*
[6]*Portuguese Oncology Institute of Porto IPO Porto, Department of Urology, Porto, Portugal*
[7]*UPTEC, BEAT Therapeutics, Porto, Portugal*
[8]*School of Medicine & Biomedical Sciences- University of Porto ICBAS-UP, Department of Pathology and Molecular Immunology, Porto, Portugal*

**Introduction**

Bladder cancer (BlCa) is the 9th most frequently diagnosed cancer worldwide. Diagnosis and management rely on invasive procedures, highlighting the need for minimally invasive alternatives. Tumor cells secrete extracellular vesicles (EVs), lipidic nanostructures that, to an extent, mimic their cell of origin and carry tumor information to other tissues via biofluids. These show a crucial role in cancer origin and progression, showing potential as biomarkers for diagnosis and prognosis of BlCa. However, the identification of tumor-specific EVs in complex body fluids containing vesicles from various cell types remains a challenge. Hence, this study aims to identify unique protein patterns in BlCa-EVs, using tumor and normal adjacent explants to matched liquid biopsies (plasma and urine) and cell lines.

**Material and Methods**

Cells were grown for 72 h in multiple 150 mm petri dishes with 20 mL of medium containing EV-depleted fetal bovine serum until reaching 80-90% confluency. Tumor and normal adjacent tissues from transurethral removal of bladder tumor were cultured for 20h with approximately 6 ml of media. EVs from plasma, urine and conditioned media from cells and tissues were isolated by ultracentrifugation (100,000g, 1h10min). Size distribution and concentration were assessed by Nanoparticle tracking analysis (NTA), shape by transmission electron microscopy (TEM), and protein content by micro bicinchoninic acid (microBCA). The

© 2024 The Authors. Molecular Oncology is published by John Wiley & Sons Ltd on behalf of Federation of European Biochemical Societies proteomic profile of BlCa cell lines was analysed by liquid chromatography–mass spectrometry (LC-MS/MS). Currently, we are analysing the proteomic profile of BlCa tissues' conditioned media and matched liquid biopsies.

**Results and Discussions**

A full characterization of BlCa-EVs was performed, using a full spectrum of samples: cell lines, tissue explants and liquid biopsies. BlCa-derived EVs present unique secretion dynamics between cell lines. Both SW780 and 253J secreted the lowest amount of particles to the medium, whereas J82 secreted the highest number of EVs. We found 7 proteins commonly expressed in all 6 BlCa cell lines and derived EVs. We now aim to identify unique protein patterns by comparing EVs from healthy donors and patients and investigate any differences in the protein makeup of BlCa-EVs found in urine and plasma from the same patients.

**Conclusion**

This approach will determine if circulating EVs accurately reflect the protein signature of bladder tumors and identify BlCa biomarkers and therapeutic targets, while uncovering the communication network mediated by BlCa-EVs.

### EACR2024-1179
### Unveiling NDUFA4L2, a novel marker for pseudohypoxic paraganglioma, as a key regulator of mitochondrial dynamics and energy metabolism

*J. Wang*[1,2], *S. Richter*[3], *M. Peitzsch*[3], *S. Nölting*[4,5], *H. Remde*[6], *M. Robledo*[7,8], *M. Parasiliti-Caprino*[9], *S. Herzig*[1,2], *N.S. Pellegata*[1,2,10], *H. Mohr*[1]

[1]*Institute for Diabetes and Cancer, Helmholtz Centre Munich, Neuherberg, Germany*
[2]*Joint Heidelberg-IDC Translational Diabetes Program, Inner Medicine 1, Heidelberg University Hospital, Heidelberg, Germany*
[3]*Institute of Clinical Chemistry and Laboratory Medicine University Hospital Carl Gustav Carus- Technische Universität Dresden, Dresden, Germany*
[4]*Department of Endocrinology- Diabetology and Clinical Nutrition, University Hospital Zurich USZ and University of Zurich UZH, Zurich, Switzerland*
[5]*Department of Medicine IV, University Hospital- LMU Munich, Munich, Germany*
[6]*Division of Endocrinology and Diabetes- Department of Internal Medicine I, University Hospital of Wuerzburg, Wuerzburg, Germany*
[7]*Heriditary Endocrine Cancer Group, Spanish National Cancer Research Centre CNIO, Madrid, Spain*
[8]*Centro de Investigación Biomédica en Red de Enfermedades Raras CIBERER, Institute of Health Carlos III ISCIII, Madrid, Spain*
[9]*Endocrinology- Diabetes and Metabolism- Department of Medical Sciences, City of Health and Science University Hospital- University of Turin, Turin, Italy*
[10]*Department of Biology and Biotechnology, University of Pavia, Pavia, Italy*

**Introduction**

Pheochromocytomas and Paragangliomas (PPGL) are rare neuroendocrine tumors that can be subdivided into at least 3 main sub-clusters based on their transcriptional signature. Among these sub-clusters, pseudohypoxic PPGL (p-PPGL) associate with the highest risk of aggressive and metastatic disease. Defects in the TCA-Cycle or in hypoxia-sensing pathways induce the stabilization of HIF2α andpredispose to p-PPGL formation. We found that the mitochondrial protein NDUFA4L2, a downstream target of HIF2α, heavily affects mitochondrial dynamics and energy metabolism in p-PPGL.

**Material and Methods**

Expression of the *NDUFA4L2* gene was assessed in rat p-PPGL (MENX model) at various stages of progression, as well as in human patient samples derived from The Cancer Genome Atlas (TCGA). Protein levels of NDUFA4L2 were validated in proteomics analysis and immunohistochemistry (IHC) using 109 human PPGL FFPE samples. PPGL cell lines overexpressing NDUFA4L2 were generated and analyzed by multiple omics approaches. Immunofluorescence, Seahorse assays and metabolomics were used to assess mitochondrial function. Finally, the influence of NDUFA4L2 overexpression on the sensitivity to PPGL therapies was assessed in viability assays.

**Results and Discussions**

We identified NDUA4L2 to be extremely upregulated during PPGL development specifically in the pseudo-hypoxic subcluster, where its levels correlate with tumor progression. NDUFA4L2 overexpression (NDUFA4L2-OE) in non-pseudohypoxic PPGL cell lines resulted in a switch from oxidative phosphorylation to aerobic glycolysis, through disruption of the TCA-cycle. Furthermore, NDUFA4L2-OE increased mitochondrial fission, a feature associated with higher metastatic rates and chemotherapy resistance. Indeed, we found that NDUFA4L2-OE can reduce the responsivity of PPGL cells to CVD (cyclophosphamide, vincristine and dacarbazine) therapy, one of the standard treatments for metastatic PPGL.

**Conclusion**

Our findings suggest that upregulation of NDUFA4L2 drives the metabolic reprogramming observed in p-PPGLs. NDUFA4L2 is a suitable IHC marker for the pseudohypoxic sub-cluster and represents an interesting novel therapeutic target.

### EACR2024-1192
### Effects of cancer-derived extracellular vesicles on the gene expression profile of different cell types constituting the tumour microenvironment

*A. Brokane*[1], *L. Sadovska*[1], *E. Endzeliņš*[1], *P. Zayakin*[1], *S. Folkmane*[1], *K. Spunde*[1], *D. Lapiņa*[1], *J. Valpētere*[1], *A. Zajakina*[1], *A. Linē*[1]

[1]*Latvian Biomedical Research and Study centre, Molecular Medicine, Riga, Latvia*

**Introduction**

The tumor microenvironment (TME) is a complex system of cancer cells, stromal cells, immune cells, the extracellular matrix and other constituents, which can significantly influence cancer progression. Extracellular vesicles (EVs) are nanosized membranous particles which have been found to play a pivotal role in mediating

© 2024 The Authors. Molecular Oncology is published by John Wiley & Sons Ltd on behalf of Federation of European Biochemical Societies intercellular crosstalk within the TME, altering recipient cell functions and potentially promoting tumour growth and metastasis. This study investigates the effects of cancer cell-derived EVs on the gene expression profiles of various TME cell types, aiming to identify cancer-associated changes in gene expression and their implications for non-invasive cancer diagnostics.

**Material and Methods**

SW620 colorectal cancer cells were cultured in a hollow fibre bioreactor, followed by EV isolation and characterization. Recipient cells, including fibroblasts (HS68, WI38), monocytes (U937, THP1), and colorectal cancer cells (SW480), were treated with cancer-derived EVs. EV uptake efficiency was assessed by fluorescence microscopy and flow cytometry. Subsequently, RNA sequencing and qRT-PCR were used to identify and validate genes whose expression was modulated by cancer-derived EVs.

**Results and Discussions**

In the HS68 cell line, 8 differentially expressed genes were unique to EVs and 45 were shared with the protein co-isolates between experimental groups. From these, a total of 11 altered genes were selected for validation, where MMP10 ($P_{adj}$ = 1.73E-89, Log2FC =3.04), MMP11 ($P_{adj}$ = 1.95E-50, Log2FC =2.11), RGS9 ($P_{adj}$ = 8.54E-47, Log2FC =1.97), and STC1 ($P_{adj}$ = 3.18E-18, Log2FC = 1.35) emerged as top candidates, exhibiting significant upregulation in cells treated with cancer-derived EVs. Furthermore, high expression of these genes in colorectal cancer tissues is associated with poor overall patient survival. qRT-PCR validation on a repeated experiment in HS68 fibroblasts not only corroborated these findings but also revealed a direct correlation between gene expression levels and EV concentration, highlighting the dose-dependent effect of cancer-derived EVs on cells constituting the TME.

**Conclusion**

These results highlight the role of cancer-derived EVs in modulating gene expression within TME cells, affecting key processes like metabolism, proliferation, differentiation, and extracellular matrix remodelling. Ongoing research explores if analogous alterations are detectable in EVs from cancer patient plasma, underscoring a potential for developing new non-invasive cancer diagnostic and/or prognostic tools.

### EACR2024-1216

### Investigating the Role of Cell-in-Cell Structures in Oral Squamous Cell Carcinoma Using 3D Culture Models and Spatial Transcriptomics

*L. de Oliveira Siquara da Rocha[1], A.Z. Abdullah[2], R. Della Coletta[3], A. Khurram[2], D. Lambert[2], C. Gurgel Rocha[4]*

*[1]FIOCRUZ-BA/UFBA, Laboratory of Pathology and Molecular Biology, Salvador, Brazil*

*[2]University of Sheffield, School of Clinical Dentistry, Sheffield, United Kingdom*

*[3]School of Dentistry- University of Campinas, Department of Oral Diagnosis, Campinas, Brazil*

*[4]FIOCRUZ/UFBA, Laboratory of Pathology and Molecular Biology, Salvador, Brazil*

**Introduction**

Cell-in-cell (CIC) structures found in oral squamous cell carcinoma (OSCC) have been associated with aggressive behavior and poor prognosis. However, little is known about the role of cancer-associated fibroblasts (CAF) in their formation and their implications in tumor progression and cellular senescence, due to limitations of two-dimensional (2D) cell culture. Our study aims to investigate the influence of CAFs on CIC occurrence in OSCC using three-dimensional spheroid culture methods and spatial transcriptomics technology.

**Material and Methods**

Spatial transcriptomics analysis was performed using the Visium pipeline on oral squamous cell carcinoma cases. Investigation was performed on the expression of CIC-related markers, cellular phenotype markers, and senescence-associated markers. Furthermore, homotypic and heterotypic OSCC spheroids were obtained in vitro using metastatic (SCC04) and non-metastatic (H357) cell lines and oral fibroblasts with different profiles: normal, myofibroblasts, and senescence-induced fibroblasts. Spheroids were obtained using the Nanoshuttle bio-printing system (Greiner Bio-One), and histological analysis was performed to evaluate the presence of CIC structures.

**Results and Discussions**

Preliminary findings from spatial transcriptomics analysis of OSCC cases show expression of markers associated with CIC structures, such as E-cadherin, N-cadherin, and Caveolin-1, alongside senescence markers p21 and p16. Meanwhile, histolomorphological analysis of OSCC spheroids show dimensional and structural changes in spheroids obtained from different fibroblast profiles, specifically normal and senescent fibroblasts. The three-dimensional spheroid model was successful in reproducing CIC structures, which can be found in all obtained samples.

**Conclusion**

Our work represents a step towards studying the implications of CAFs and CIC structures in OSCC prognosis. Spatial transcriptomics findings in OSCC cases indicate a possible associated between CIC and CAF presence, and cellular senescence. By applying the Xenium pipeline, we aim to investigate these structures and their implications at a cellular and subcellular level in spatial transcriptomics analysis.

*This project was funded by the British Council and the Newton Advanced Fellowship Grant.*

### EACR2024-1217

### Effect of combined treatments with Chlorogenic Acid and 5-Fluorouracil on Wnt/B-catenin pathway modulation in colorectal cancer

*A.M. Sepúlveda[1], A.M. Castañeda[1], G.A. Santa-González[1], D.F. Uribe Yunda[1], J. Pedroza-Díaz[1]*

*[1]Instituto Tecnológico Metropolitano-ITM, Grupo de Investigación e Innovación Biomédica-GI2B- Facultad de Ciencias Exactas y Aplicadas, Medellín, Colombia*

**Introduction**

Colorectal cancer is the fourth in incidence and the third in mortality of cancer around the world. Unfortunately,

© 2024 The Authors. Molecular Oncology is published by John Wiley & Sons Ltd on behalf of Federation of European Biochemical Societies chemotherapy agents used for its treatment, like 5-Fluorouracil (5-FU), shows low specificity, high toxicity, and hypersensitivity reactions. Natural compounds have shown positive effects as a possible adjuvant to chemotherapy in different types of cancer. Chlorogenic acid (CGA) is a polyphenol present in high proportions in a wide variety of plants species, which has been shown anticancer effect by suppressing proliferation, inducing apoptosis, and modulating signaling pathways involved in the progression of colorectal cancer, such as Wnt/B-catenin. The aim of the present work is to evaluate the effect of combined treatments with 5-FU and CGA in In Vitro models of colorectal cancer.

**Material and Methods**

Human colorectal adenocarcinoma cell lines SW480 and HT-29 were culture and maintained in standard conditions. Pretreatment was carried out for 24h with CGA (18μM) alone, then, addition of CGA (18μM) and 5-FU (20 μM) for the next 24h was performed and the cytotoxicity was assessed using MTT. To evaluate the expression of target and components members of Wnt/B-catenin pathway, gene expression levels were evaluated by qRT-PCR. Finally, DNA content was analyzed to determine the cell distribution in cell cycle phases by flow cytometry. All experiments were performed with cells in exponential phase and by triplicate.

**Results and Discussions**

The MTT results shown that the combined treatments induced a reduction of cell viability ≈40% in both cell lines. Likewise, downregulation of gene expression levels of *CTNNB1* (B-catenin), *TCF-4* (T-cell factor 4), *c-JUN* (Jun proto-oncogene) and no changes in *CCND1* (Cyclin D1) expression, were observed in treated vs untreated cells. Finally, flow cytometry shown that both cell lines had a typical cell cycle pattern, however, in treated cells changes in the distribution of the cycle phases were note. These results shown the capability of CGA in combination with 5-FU for the reduction of cell viability in both SW480 and HT-29 cells, apparently by modulating the Wnt/B-catenin pathway.

**Conclusion**

Our results suggest an antitumoral effect of CGA in combination with 5-FU through the modulation of the Wnt/B-catenin pathway, indicating a potential synergistic/additive effect for the intervention of colorectal cancer.

*Acknowledgment: Programa Jóvenes Investigadores e Innovadores ITM-2023 and Minciencias grant (project code: 115080763215 CT 811-2018).*

### EACR2024-1219

### Utilizing bead-based multiplex serology for the detection of nasopharyngeal carcinoma in Malaysian population

*G.W. Tan*[1], *A. Babi*[2], *B. Michels*[2], *K.E. Tan*[1], *C.T. Low*[1], *W.R. Kim*[1], *S.H. Shahid*[1], *N.A. Adam*[1], *T. Waterboer*[2], *L.P. Tan*[1]

[1]*Institute for Medical Research, Cancer Research Centre, Shah Alam, Malaysia*

[2]*German Cancer Research Center, Infections and Cancer Epidemiology, Heidelberg, Germany*

**Introduction**

Nasopharyngeal carcinoma (NPC) is an Epstein-Barr Virus (EBV)-associated epithelial cancer which is rare in most parts of the world, but has a very high age-standardized rate in Southeast Asia and Southern China. In Malaysia, it disproportionately affects the lowest socioeconomical class as early as around the age of 40, and late-stage presentation at diagnosis often leads to financial catastrophe and a 5-year survival rate as low as 27%. Cost-effective tests are needed for early detection and intervention, reducing the disease burden.

**Material and Methods**

We evaluated the utility of a bead-based multiplex EBV serology platform in combination with EBV DNA test to detect NPC in Malaysian population. Plasma samples were obtained from 100 NPC patients and 300 controls including community controls (n=219), hospital controls (n=72) and non-NPC patients (n=9). IgA and IgG antibodies against 23 EBV antigens were analyzed using multiplex serology. Plasma EBV DNA was detected using real-time PCR, targeting the BamHI-W region.

**Results and Discussions**

Receiver operating characteristic analysis identified EBV DNA and 19 serology markers with area under the curve (AUC) above 0.9, indicating their potential diagnostic utility. Among these markers, EBV DNA exhibited the highest AUC of 0.960 (95% confidence interval [CI] 0.930 - 0.990) Logistic regression analysis on dichotomous data using the forward selection method revealed a final model consisting of two serology markers (EAD IgA, and BGLF2 IgG) and EBV DNA. This model demonstrated an improved AUC of 0.978 (95% CI 0.955-1.000), highlighting the enhanced predictive ability when combining these markers for identifying individuals with NPC. Evaluation of the four serology markers combination (EAD IgA, BGLF2 IgG, LF2 IgA, LF2 IgG) and two serology markers (BGLF2 IgG and LF2 IgG, or EAD IgA and LF2 IgA) combination reported by Simon et al showed AUC of 0.965 (95% CI 0.937 - 0.994), 0.958 (95% CI 0.927 - 0.989) and 0.944 (95% CI 0.910 - 0.978), respectively. Incorporating EBV DNA into these models resulted in slight improvement of performance, with AUC of 0.979 (95% CI 0.956 - 1.001), 0.974 (95% CI 0.949 - 0.998) and 0.976 (95% CI 0.954 - 0.998), respectively.

**Conclusion**

In conclusion, this study highlights the potential of EBV DNA and specific serological markers for NPC detection. The identified two serology markers (EAD IgA and BGLF2 IgG) and EBV DNA combination demonstrates similar or improved accuracy compared to previously reported combinations.

### EACR2024-1226

### Regular physical exercise improves response to neoadjuvant chemotherapy by modulating gene expression profile and reprogramming immune tumour microenvironment in breast cancer patients

*D. Skorinkina*[1], *K. Eglītis*[2], *P. Zayakin*[1], *L. Sadovska*[1], *A. Mlynska*[3], *A. Brokāne*[1], *A. Kļaviņa*[4], *J. Eglītis*[2],

© 2024 The Authors. Molecular Oncology is published by John Wiley & Sons Ltd on behalf of Federation of European Biochemical Societies

*K. Silina[5], A. Linē[1]*
*[1]Latvian Biomedical Research and Study Centre, Cancer Biomarker group, Riga, Latvia*
*[2]Riga Eastern Clinical University Hospital, Latvian Oncology Center, Riga, Latvia*
*[3]National Cancer Institute, Laboratory of Immunology, Vilnius, Lithuania*
*[4]Latvian Academy of Sport Education, n/a, Riga, Latvia*
*[5]ETH Zurich, Institute of Pharmaceutical Sciences-Department of Chemistry and Applied Biosciences, Zurich, Switzerland*

**Introduction**

A growing number of studies show that physical exercise is feasible and safe for cancer patients and enhances various physical and cognitive capabilities thus improving the quality of life and disease outcomes, however the molecular mechanisms behind these effects are poorly understood. Gaining insight into the mechanisms would facilitate the incorporation of exercise into treatment plans and help to develop novel therapeutic strategies and preventive measures for cancer. We reasoned that enrolling breast cancer (BC) patients receiving neoadjuvant chemotherapy (NAC) into a high-intensity interval training (HIIT) program provides a unique opportunity to investigate the effects of exercise on tumour biology and response to NAC.

**Material and Methods**

50 BC patients were randomised into HIIT and control groups. Patients assigned to the HIIT group performed personalised home-based HIIT programs during NAC. Response to NAC was assessed using the standard Miller-Payne grading system. BC and normal breast tissues from 20 patients from the HIIT and retrospective control groups were subjected to RNA sequencing analysis. To infer the composition of tumour-infiltrating immune cells from the bulk RNA-seq data the CIBERSORTx was used. To validate the findings, 7-plex immunofluorescence assay for immunophenotyping of tumour-infiltrating NK cells was developed and applied for the quantification of NK cell populations in the tumour sections.

**Results and Discussions**

In the HIIT group, the Miller-Payne grades were significantly higher than in retrospective control group ($p$=0.012) and the number of responders was significantly higher in the HIIT as compared to prospective (80% vs 47%, $p$=0.036) and retrospective control groups (80% vs 50%, $p$=0.047). RNA-seq analysis revealed exercise-induced alterations in the tumour-intrinsic gene expression profile that are associated with favourable outcomes of BC patients and changes in the composition of tumour-infiltrating immune cells. The fraction of activated NK cells was increased in patients exercising during the NAC, whereas tumour tissues from the control group were infiltrated with resting NK cells.

**Conclusion**

These findings support the incorporation of physical activities in the treatment plans of BC patients and prompt the initiation of a clinical trial investigating the effects of HIIT on the efficacy of immunotherapy.

### EACR2024-1249
### Patients derived organoids (PDOs)

### Immune Cells co-culture in ovarian cancer: the role of TUmoRmicroEnvironment (PICTURE)

*C. Nero[1], A. Ciucci[2], F. Camarda[3], C. Di Mario[4], S. Mastrogiovanni[3], E. Cesari[5], E. Gremese[6], A. Battaglia[7], C. Sette[8], G. Scambia[3]*
*[1]Fondazione Policlinico Universitario A.Gemelli IRCCS, Dipartimento della Salute della donna- del bambino e di sanità pubblica - UOC Ginecologia Oncologica, Rome, Italy*
*[2]Università Cattolica del Sacro Cuore, Dipartimento per la tutela della salute della donna e della vita nascente- sezione di Ginecologia ed Ostetricia, Rome, Italy*
*[3]Fondazione Policlinico Universitario A.Gemelli IRCCS, Dipartimento della Salute della donna- del bambino e di sanità pubblica UOC Ginecologia Oncologica, Rome, Italy*
*[4]Fondazione Policlinico Universitario A.Gemelli IRCCS, Dipartimento Scienze dell'invecchiamento- ortopediche e reumatologiche - UOSD Immunologia Clinica, Rome, Italy*
*[5]Fondazione Policlinico Universitario A.Gemelli IRCCS, Dipartimento della Salute della donna- del bambino e di sanità pubblica, Rome, Italy*
*[6]Fondazione Policlinico Universitario A.Gemelli IRCCS, Dipartimento Scienze dell'invecchiamento- ortopediche e reumatologiche - UOC Immunologia Clinica, Rome, Italy*
*[7]Università Cattolica del Sacro Cuore, Dipartimento Universitario Scienze della Vita e Sanità Pubblica Sezione di Ginecologica e Ostetricia, Roma RM, Italy*
*[8]Università Cattolica del Sacro Cuore, Dipartimento di Neuroscienze - Sezione di Anatomia Umana, Rome, Italy*

**Introduction**

High-grade serous ovarian cancer (HGSOC) is estimated to account for 50–60% of all OC and typically presents at advanced stage (III-IV). New treatments for advanced stage HGSOC are needed since most HGSOC recur, with 5-year survival reaching about 30%. Immune cell infiltration and mutational burden phenotype are known to contribute to HGSOC progression. Nevertheless, clinical trials with immunotherapeutic drugs yielded unsatisfactory results for most HGSOC cases. Thus, identification of an ex vivo system to be used as a proxy for patients'immunotherapy response prediction and/or of drugs that may improve release of HGSOC-associated neoantigens to be recognized by immune cells is of extreme interest. In our study, we developed a preclinical co-culture model of HGSOC organoids and autologous T cells to assess the ability of tumor-educated T cells to cooperate with chemo- and immunotherapeutic treatments in favoring tumor cell death.

**Material and Methods**

We set up a co-culture model of autologous mononuclear cells (MC) and patient-derived organoids (PDO) from HGSOC patients undergoing primary cytoreductive surgery (IRB approval 4531). PDOs were treated with IFNγ and/or Carboplatin (Cp) and co-cultured with MC with or w/o Nivolumab (anti-PD1 mAb),in the presence of IL-2 in CD28-coated plates. T cell activation ($CD137^+$ cells) and tumor cell (TC) apoptosis (Caspase 3/7 expression) were analyzed by flow cytometry.

**Results and Discussions**

© 2024 The Authors. Molecular Oncology is published by John Wiley & Sons Ltd on behalf of Federation of European Biochemical Societies

We generated PDOs from three HGSOC patients. Spontaneous apoptosis (10-11%TC) was observed in all PDO cultures. In vitro Cp treatment increased spontaneous TC apoptosis up to 31%. Co-culturing PDO with educated autologous T cells induced TC apoptosis up to 18% in the absence of treatment. Furthermore, co-culture of PDOs with educated autologous T cells in the presence of Cp increased TC apoptosis up to 56%. T cell re-challenging with autologous PDO cultures favored T cell activation (up to 18%), irrespective of previous PDO treatment. Only one PDO/T cell co-culture was performed with and w/o Nivolumab; in this experiment Nivolumab did not modify T cell-mediated apoptosis.

**Conclusion**

In this pilot study we confirm the feasibility of establishing an OC-PDO/T cell co-culture. Although these data need to be confirmed in a larger cohort of HGSOC patients, we provide preliminary evidence for the capacity of tumor-educated autologous T cells to recognize and kill OC cells and to synergize with chemotherapy. The unexpected poor effect exerted by Nivolumab deserves further investigation.

© 2024 The Authors. Molecular Oncology is published by John Wiley & Sons Ltd on behalf of Federation of European Biochemical Societies
